# Genome sequencing of *Rigidoporus microporus* provides insights on genes important for wood decay, latex tolerance and interspecific fungal interactions

**DOI:** 10.1038/s41598-020-62150-4

**Published:** 2020-03-23

**Authors:** Abbot O. Oghenekaro, Andriy Kovalchuk, Tommaso Raffaello, Susana Camarero, Markus Gressler, Bernard Henrissat, Juna Lee, Mengxia Liu, Angel T. Martínez, Otto Miettinen, Sirma Mihaltcheva, Jasmyn Pangilinan, Fei Ren, Robert Riley, Francisco Javier Ruiz-Dueñas, Ana Serrano, Michael R. Thon, Zilan Wen, Zhen Zeng, Kerrie Barry, Igor V. Grigoriev, Francis Martin, Fred O. Asiegbu

**Affiliations:** 1 Faculty of Life Sciences, Department of Plant Biology and Biotechnology, University of Benin, P.M.B 1154 Benin City, Nigeria; 2 Faculty of Agriculture and Forestry, Department of Forest Sciences, University of Helsinki, P.O. Box 27, FIN-00014 Helsinki, Finland; 3 Centro de Investigaciones Biológicas, Consejo Superior de Investigaciones Científicas, Ramiro de Maeztu 9, E28040 Madrid, Spain; 4 Department of Pharmaceutical Microbiology at the Hans Knöll Institute, Friedrich Schiller University, Jena, Germany; 5 Aix-Marseille Université, Architecture et Fonction des Macromolécules Biologiques, CNRS, UMR 7257, 13288 Marseille, cedex 9 France; 6 USC1408 Architecture et Fonction des Macromolécules Biologiques, Institut National de la Recherche Agronomique, F-13288 Marseille, France; 7 Department of Biological Sciences, King Abdulaziz University, 23218 Jeddah, Saudi Arabia; 8 US Department of Energy Joint Genome Institute, Lawrence Berkeley National Laboratory, 1 Cyclotron Road, Berkeley, CA 94720 USA; 9 Department of Plant and Microbial Biology, University of California Berkeley, Berkeley, CA 94720 USA; 10 Mycology Unit, Botanical Museum, Finnish Museum of Natural History, University of Helsinki, P.O. Box 7, Helsinki, Finland; 11 Forestry experiment center of north China, Chinese Academy of Forestry, 102300 Beijing, China; 12 Universidad de Salamanca, Instituto Hispano-Luso de Investigaciones Agrarias (CIALE), Villamayor, Spain; 13 Institut National de la Recherche Agronomique (INRA), Laboratory of Excellence Advanced Research on the Biology of Tree and Forest Ecosystems (ARBRE), UMR 1136 Champenoux, France; 14 University of Lorraine, Laboratory of Excellence ARBRE, UMR 1136 Champenoux, France; 15 Present Address: Department of Plant Science, University of Manitoba, MB R3T 2N2 Winnipeg, Canada

**Keywords:** Comparative genomics, Ecological genetics

## Abstract

Fungal plant pathogens remain a serious threat to the sustainable agriculture and forestry, despite the extensive efforts undertaken to control their spread. White root rot disease is threatening rubber tree (*Hevea brasiliensis*) plantations throughout South and Southeast Asia and Western Africa, causing tree mortality and severe yield losses. Here, we report the complete genome sequence of the basidiomycete fungus *Rigidoporus microporus*, a causative agent of the disease. Our phylogenetic analysis confirmed the position of *R. microporus* among the members of *Hymenochaetales*, an understudied group of basidiomycetes. Our analysis further identified pathogen’s genes with a predicted role in the decay of plant cell wall polymers, in the utilization of latex components and in interspecific interactions between the pathogen and other fungi. We also detected putative horizontal gene transfer events in the genome of *R. microporus*. The reported first genome sequence of a tropical rubber tree pathogen *R. microporus* should contribute to the better understanding of how the fungus is able to facilitate wood decay and nutrient cycling as well as tolerate latex and utilize resinous extractives.

## Introduction

The white rot fungus known as *Rigidoporus microporus* (Sw.) Overeem (*Basidiomycota*, *Agaricomycotina*) is the most economically important pathogen of the tropical tree *Hevea brasiliensis* (Willd. ex A. Juss.) Müll.Arg., also known as Para rubber. Para rubber is principally valued for its latex content; the latex or natural rubber is a very significant industrial commodity used in a variety of industries from car manufacturing to healthcare. The market value of global annual production of natural rubber is over US$ 50 billion. Elastomers derived from natural rubber are indispensable in space, water, and ship technologies^1^. A major part of the plantation forestry in Africa and Asia is the growing of Para rubber.

**Figure 1:**
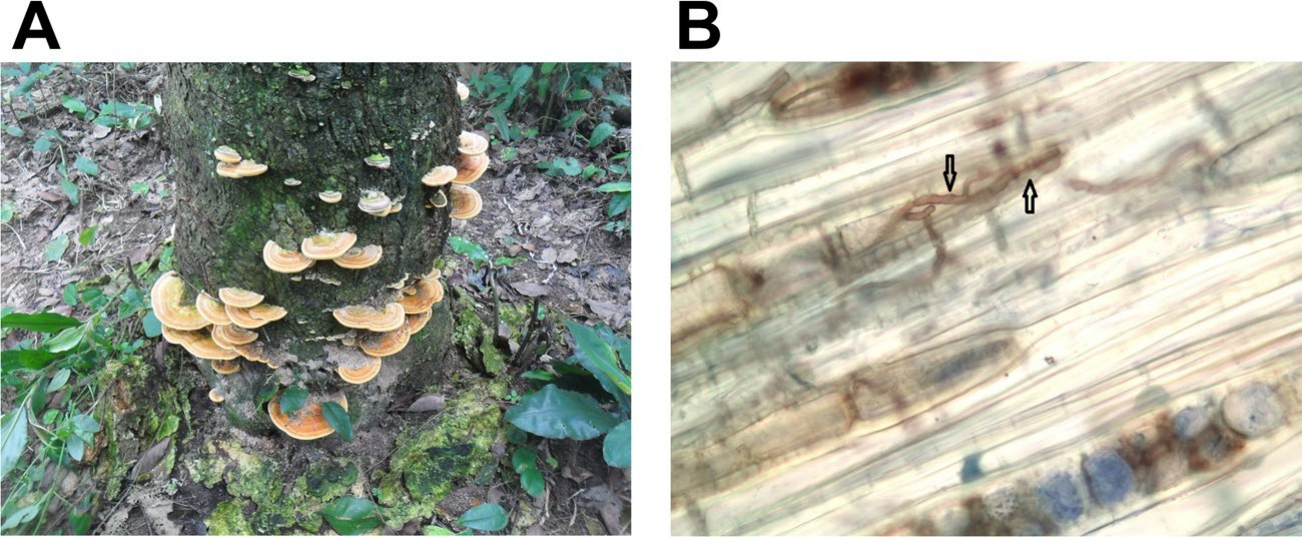
(**A**) Fruiting bodies of *Rigidoporus microporus* developing in the lower part of *Hevea brasiliensis* trunk heavily colonized by the pathogen (plantation of the Rubber Research Institute Nigeria, July 2017). (**B**) Longitudinal section of *H. brasiliensis* wood colonized by *R. microporus* (stained by aniline blue).

*Rigidoporus microporus* is therefore a fungal species of significant economic importance. As a necrotrophic pathogen, it has an extensive host range and affects many tropical and subtropical trees, food and cash crops^2,3^. However, it is best known as a causative agent of the white root rot disease of the rubber tree and for the devastating impact it has on commercial rubber tree plantations (Fig. 1)^4^. The fungus produces rhizomorphs (thread-like mycelial aggregation of fungal hypha), which can grow several meters in the soil before they reach and attack roots of a suitable host, and it continues to rot wood long after the tree has fallen^5^. Population genetic studies suggested that a host jump and the lack of coevolution between the host and the pathogen might be the possible reasons for the aggressive behavior of the pathogen on rubber trees. This is further supported by the fact that the disease is devastating in Asia and West Africa, but not a serious problem in South America, the centre of origin of the rubber tree host^3^. Presence of high density of fungal rhizomorphs of *R. microporus* in the soil indicates a capacity to switch from a necrotrophic to a saprotrophic lifestyle^2,6^. As white rot fungi, members of the genus *Rigidoporus* are known to play an ecological role in nutrient and carbon cycling in tropical forests^7^.

The genus *Rigidoporus* was traditionally assigned to the order *Polyporales*, however, as demonstrated recently, the genus is polyphyletic^8,9^. Our previous results provided evidence that *R. microporus* might in fact belong to the order *Hymenochaetales* rather than *Polyporales*^3^. Sequence homology analysis from *de novo* transcriptome studies of *R. microporus* showed the highest similarity to *Fomitiporia mediterranea*, a member of *Hymenochaetales*^10^. The order *Hymenochaetales* in its current circumscription includes several hundred species, with the majority of them being wood-decaying fungi^11^. Only a few members of *Hymenochaetales* have been hitherto encompassed by genome sequencing projects. The sequenced species include *Fomitiporia mediterranea*^12^, *Schizopora paradoxa*^13^, *Phellinus noxius*, *P. lamaensis*, *P. sulphurascens* and *Porodaedalea pini*^14^. Availability of the genome of *R. microporus* provides opportunities for the comparative genome analysis of the species of *Hymenochaetales* with members of other orders of *Agaricomycotina*.

Genome and transcriptome studies of white rot fungi have intensified in the past decade due to their lignocellulose-degrading abilities and potential applications in the bioenergy processing and utilization. The 1000 Fungal Genomes project made significant strides in elucidating the genes responsible for the lignocellulose degradation and succeeded in sequencing dozens of white rot fungi genomes^15^. The analysis of the *de novo* transcriptome assembly of *R. microporus* revealed potential lignocellulose-degrading machinery typical for white-rot basidiomycetes^10^. It agrees with the pattern of wood decay observed previously in decay tests^2^. Additionally, and more importantly, the transcriptome study revealed rubber tree latex (*cis*-1,4-polyisoprene) degradation potential in a white rot fungus^10^. The ability to degrade natural rubber latex may play a role in the survival of the pathogen in a latex-rich environment of the host tree^10^.

Little is known about the molecular determinants controlling wood decay, virulence and pathogenicity of *R. microporus* on rubber tree. Traditionally, plant cell wall-degrading enzymes (PCWDEs) secreted by necrotrophic fungal pathogens were considered as the main factors triggering host cell death by affecting cell wall integrity^16^. However, necrotrophic fungi are also able to produce specific proteins, which induce host cell death by interfering with the components of plant defense system^17,18^. These necrotrophic effectors are functional counterparts of effectors employed by biotrophic and hemibiotrophic fungi during host colonization^19^.

In natural forest environment, different species of wood-degrading fungi commonly compete with each other for available resources^20^. The interspecific fungal interactions are often accompanied by the secretion of an array of enzymes and metabolites with antagonistic properties. The role of small secreted proteins (SSPs) or fungal effectors in this type of interactions remains elusive, however, it was hypothesized that they might be involved not only for pathogenic interactions but also in interspecific competition^21^.

Horizontal gene transfer (HGT) is an integral part of genome evolution in fungi. The lateral transfer of genes, gene clusters or entire chromosomes can have significant effects on disease breakout and metabolic activity^22,23^. Importantly, HGT was demonstrated as an important driving force shaping the adaptation of fungi to the phytopathogenic lifestyle^24^.

We report the results of our analysis of the first genome sequence of a tropical rubber tree pathogen *R. microporus*. The availability of the fungus genome further complements the sequenced genome of its rubber tree host^25^. The main objectives of this study were (i) to perform the analysis of the plant cell wall-degrading machinery of the fungus, (ii) to elucidate the mechanisms of latex degradation and utilization by *R. microporus*, (iii) to identify putative effectors used by *R. microporus* during host tree colonization, (iv) to identify putative horizontal gene transfer (HGT) events and their role in the adaptation of *R. microporus* to its pathogenic lifestyle, and (v) to clarify the systematic position of *R. microporus* within the order *Hymenochaetales*. Whole genome and transcriptomic analysis of *R. microporus* grown on natural rubber latex revealed a variety of genes potentially related to pathogenicity and virulence, effector-like proteins, lignocellulose and latex degradation. This study is expected to accelerate further research on this economically important tropical wood rotting fungus.

## Methods

### Fungal isolates and culture conditions

*R. microporus* isolate ED310 used for the genome sequencing was isolated from a diseased *H. brasiliensis* tree from a rubber tree plantation at the Rubber Research Institute, Nigeria in 2012^3^. The isolate was deposited in HAMBI culture collection center with ID number HAMBI/FBCC 2356. Genomic DNA was isolated from 7 days-old *R. microporus* cultures harvested from 2% w/v malt extract agar (MEA) plates overlaid with cellophane according to the protocol provided in the Supplementary Note 1.

### Genome sequencing and annotation

Sequencing of the *Rigidoporus microporus* ED310 genome was done using Pacific Biosciences RS II. Unamplified libraries were generated using the Pacific Biosciences standard template protocol to create >10 kb libraries. Five micrograms of gDNA was used to create each library, followed by shearing of the DNA using Covaris g-Tubes™ to create sheared fragments of >10 kb. The sheared DNA fragments were then further processed using Pacific Biosciences SMRTbell template preparation kit. The DNA fragments were treated with DNA damage repair and the ends were repaired to create blunt-ended fragments which were 5´ phosphorylated. Adapters were then attached to the fragments to create the SMRTbell template for sequencing. The SMRTbell templates were then treated with exonuclease to purify them and size-selected using AMPure PB beads. PacBio primer was annealed to the SMRTbell template library and sequencing polymerase (V. P6) was bound to them. Sequencing of the SMRTbell template libraries was carried out on a Pacific Biosciences RSII sequencer using Version C4 chemistry and 1 × 240 min movie run times.

Filtered subread data was assembled with Falcon version 0.7.3 (https://github.com/PacificBiosciences/FALCON) to generate the initial assembly. Assembly of the mitochondrial genome was done separately from the Falcon pre-assembled reads (preads) using an in-house tool (assemblemito.sh), used to filter the preads, and finally cleaned with Quiver version smrtanalysis_2.3.0.140936.p5 (https://github.com/PacificBiosciences/GenomicConsensus). A secondary Falcon assembly was created using the filtered preads and improved with finisherSC version 2.0^26^ and cleaned with Quiver. Contigs less than1000 bp were excluded.

The annotation of the *R. microporus* genome was executed with the JGI genome Annotation pipeline, which uses tools for gene prediction, annotation and analysis^27,28^. Different gene predictors were utilized to generate multiple sets of gene models. The predicted gene models were automatically filtered based on results of similarity to proteins from other species coupled with the support from the gene expression data to produce a final non-redundant catalog of genes which represents the best gene model located at each locus. Multi-gene families prediction was carried out using the Markov clustering algorithm (MCL)^29^, a useful part of the JGI annotation pipeline, and annotation was done using PFAM domains present in cluster member sequences. Identification of secreted proteins were done using SignalP v.4.1 (sensitive mode)^30^, TargetP v.1.1^31^ and TMHMM v.2.0^32^ to predict the presence of signal peptide, targeted cellular localization and transmembrane domain (TM), respectively. Proteins having more than two TMs and/or a single TM not overlapping with the signal peptide were excluded from the analysis. The predicted secretome was blastp against PHI-base v.4.4, the pathogen-host interaction database^33^ with the cut off E-value set to 10^–5^ to identify potential virulence-related secreted proteins.

### Annotation of class II peroxidases, multicopper oxidases and GMC oxidoreductases

A screening of the automatically-annotated genome of *R. microporus* was performed by BLASTing the amino acid sequences of five selected class II peroxidases (generic peroxidase, GP; short manganese and long manganese peroxidases, MnP-short and MnP-long; versatile peroxidase, VP; and lignin peroxidase, LiP) against the filtered model protein database of this fungus available at MycoCosm. Six class II gene models were identified and manually annotated based on: (i) the highest sequence identities for each protein sequence derived from the predicted gene; (ii) multiple alignment with 145 heme peroxidase protein sequences from twelve fungal species (ten species of *Polyporales* and two species of *Hymenochaetales*); and (iii) examination of theoretical molecular structures obtained by homology modeling using crystal structures of related peroxidases as templates and programs implemented by the automated protein homology modeling server “SWISS-MODEL”^34^.

The multicopper oxidase genes present in *R. microporus* genome were analyzed following two different strategies: (i) BLASTing with *R. lignosus* laccase (1V10.A) as a probe; and (ii) SEARCHing by keyword using “multicopper oxidase”.

After multiple alignment with already known MCO proteins to search for conserved motifs and residues, the sequences were manually curated. Molecular models of distinctive proteins were built up to better determine the different types of MCOs.

The screening for each of the GMC oxidoreductase families, glucose oxidase (GOX), pyranose-2-oxidase (POX), pyranose dehydrogenase (PDH), aryl alcohol oxidase (AAO), methanol oxidase (MOX) and cellobiose dehydrogenase (CDH), was performed by querying in the filtered model protein database of *R. microporus* using previously characterized sequences of GMC oxidoreductases from 10 fungal species^35^ as template, and sequences with E-values ≤ 1e-100 were selected.

### Identification of putative horizontal gene transfer events

The predicted proteins from the *R. microporus* genome were scanned with the program DarkHorse^36^ to identify candidate horizontally transferred genes. We considered as HGT candidates proteins with a normalized LPI score of less than 0.8 and that had at least 10 significant BLAST matches. For each of the 59 candidates, a BLAST search of the nr database was performed and the 50 best matches were used for phylogenetic tree construction. The protein sequences were aligned with MAFFT v.7.388^37^ and phylogenetic trees were constructed with FastTree v.2^38^. BLAST searches, alignments and phylogenetic analyses were performed with Geneious v.11^39^.

### RNA-seq analysis of *R. microporus* transcriptional responses to natural latex

Growth rates of *R. microporus* in the presence of the rubber tree latex were estimated *in vitro*. Natural rubber tree latex (CAS no. 9006-04-06), Weber & Schaer GmbH & Co. KG (Hamburg, Germany) as low ammonia latex milk (Neotex LA) was utilized for the experiment. Before media preparation of the latex milk, the stabilizing ammonia was removed by centrifugation (5 min at 10,000 × *g*). The ammonia-free top layer was collected and utilized for further experiments. The latex was added to the modified Pachlewski P5 agar medium^40^ (without carbon source) at the concentration of 0.8% (v/v). This medium was used to overlay P5 agar plates supplemented with either 0.1%, 0.5%, and 1% glucose or 1% rubber wood sawdust as a source of carbon (Supplementary Fig. 1). Non-overlaid plates were used as a control.

Fungal hyphal growth was measured six days post inoculation (Supplementary Fig. 2). The set-up containing 1% rubber wood sawdust had the highest hyphal growth and was thus selected for RNA extraction (Supplementary Fig. 2). Total RNA was extracted from three biological replicates of both the treatment and control according to the protocol of^41^.

Library construction and sequencing were performed at the Beijing Genome Institute, Hong Kong. Messenger RNA extracted from total RNA using oligo (dT) beads was fragmented in buffer to generate short fragments of 200 bp. Random hexamers were then used to synthesize first strand cDNA, followed by addition of dNTPs, RNase and DNA polymerase I to synthesize second strand cDNA. Sequencing adaptors were attached to fragments which were then amplified by Polymerase Chain Reaction. Six cDNA libraries (3 biological replicates for latex-growing cultures and 3 for control) were created. The six cDNA libraries were sequenced separately using the Illumina HiSeqTM 2000 sequencing platform.

### Analysis of RNA-seq data

The quality of RNA-seq reads was assessed using FASTQC (v0.11.2). Afterwards, the *R. microporus* genome was indexed and mapped with STAR v.2.5.2b^42^. Raw read count table was generated by htseq-count script within HTSeq v.0.6.1p1^43^ using the obtained uniquely mapped reads. The count table was then used to identify differentially expressed genes with DESeq v 1.38.0^44^. Gene Ontology (GO) (biological process and molecular function) enrichment analysis among differentially expressed genes were performed with topGO v.2.30.2^45^ using Fisher’s exact test (p < 0.05). The GO annotations of all predicted genes were retrieved from the JGI portal.

### Interactions of *R. microporus* with other fungal species

*R. microporus* was co-cultured with three representative species of basidiomycetes which were selected partly based on their lifestyle as saprotrophs and/or as biocontrol agents in other pathosystems: saprotrophic white-rot fungus *Phanaerochate chrysosporium*, saprotrophic white-rot and saprotroph/biocontrol agent *Phlebiopsis gigantea* and the saprotroph *Mycena* sp. Dual cultures were prepared on MEA plates and cultivated at 25 °C. Three time points were selected and used for RNA extraction. For time point 1 (no hyphal contact), *R. microporus* was co-cultured with either the saprotrophic fungi *P. chrysosporium*, *P. gigantea* or *Mycena* sp. for 2 days, 4 days and 6 days, respectively. At time point 2 (initial hyphal contact), *R. microporus* was co-cultured with *P. chrysosporium*, *P. gigantea* and *Mycena* sp. for 4 days, 6 days, and 10 days, respectively. For time point 3 (a few days after hyphal contact), *R. microporus* was co-cultured with *P. chrysosporium, P. gigantea* and *Mycena* sp. for 8 days, 8 days, and 13 days, respectively. Expression level of the analyzed genes was compared with their expression in the pure culture of *R. microporus* (normalized as 1.0). RNA was extracted using TRI Reagent (Sigma-Aldrich. Inc., USA) according to manufacturer’s instructions. cDNA was synthesized with Thermo Scientific reagents (DNase I, EDTA, oligo-dT, RevertAid Reverse Transcriptase) according to the protocol supplied by the manufacturer. qPCR was performed on LightCycler® 480 Instrument II (Roche, Switzerland). Seven target genes (protein models 933844, 207751, 252098, 692493, 170617, and 238476) were selected from the predicted set of *R. microporus* SSPs based on their deduced amino acid sequence and transcriptomic expression profile. Additionally, two reference genes (actin and TFIIIC)^46^ were used. The statistical analysis of qPCR results was performed in EasyqpcR^47^.

### Phylogenetic analysis

Forty single-copy genes were initially screened, and ten of them were selected for the phylogenetic analysis (Supplementary Table 1) based on several criteria explained below. The single copy orthologs were obtained from the database OrthoDB v9.1^48^. The orthologs were selected based on their low evolutionary rates and presence across the class *Agaricomycotina*. Single copy ortholog of *Fomitiporia mediterranea* (*Hymenachaetales*) for each gene was obtained and used to perform a blastP on the filtered model sets of proteins of the respective genomes in the Joint Genome Institute (JGI) genome portal (Supplementary Table 2) using default parameters. Sequences obtained from the blast results for each gene were then analyzed further. Alignments of sequences for each of the individual single copy genes were obtained using MAFFT v. 7^37^. Quality control was manually carried out to detect and remove potential paralogs. The aligned proteins were then treated with Gblocks^49^ with the following settings: maximum number of contiguous non-conserved positions = 4; minimum length of blocks allowed = 10. Proteins detected as paralogs after Gblocks treatment were removed before phylogenetic analyses. The final individual 10 protein alignments after Gblocks treatment were concatenated to produce a single alignment using Geneious R6.0.6^39^. The final ten gene concatenated dataset has a length of 12,074 aa. Individual alignments for each gene and the concatenated datasets are provided as Supplementary Files 1–11. Phylogenetic trees were constructed using two different phylogenetic methods, maximum likelihood (ML) and neighbor joining (NJ). ML was performed in the online version of PhyML 3.0^50–52^, using automatic model selection by SMS^53^ and Akaike information criterion (AIC)^52^. NJ analyses was conducted using MEGA 7^53^.

## Results

### Genome features

**Figure 2:**
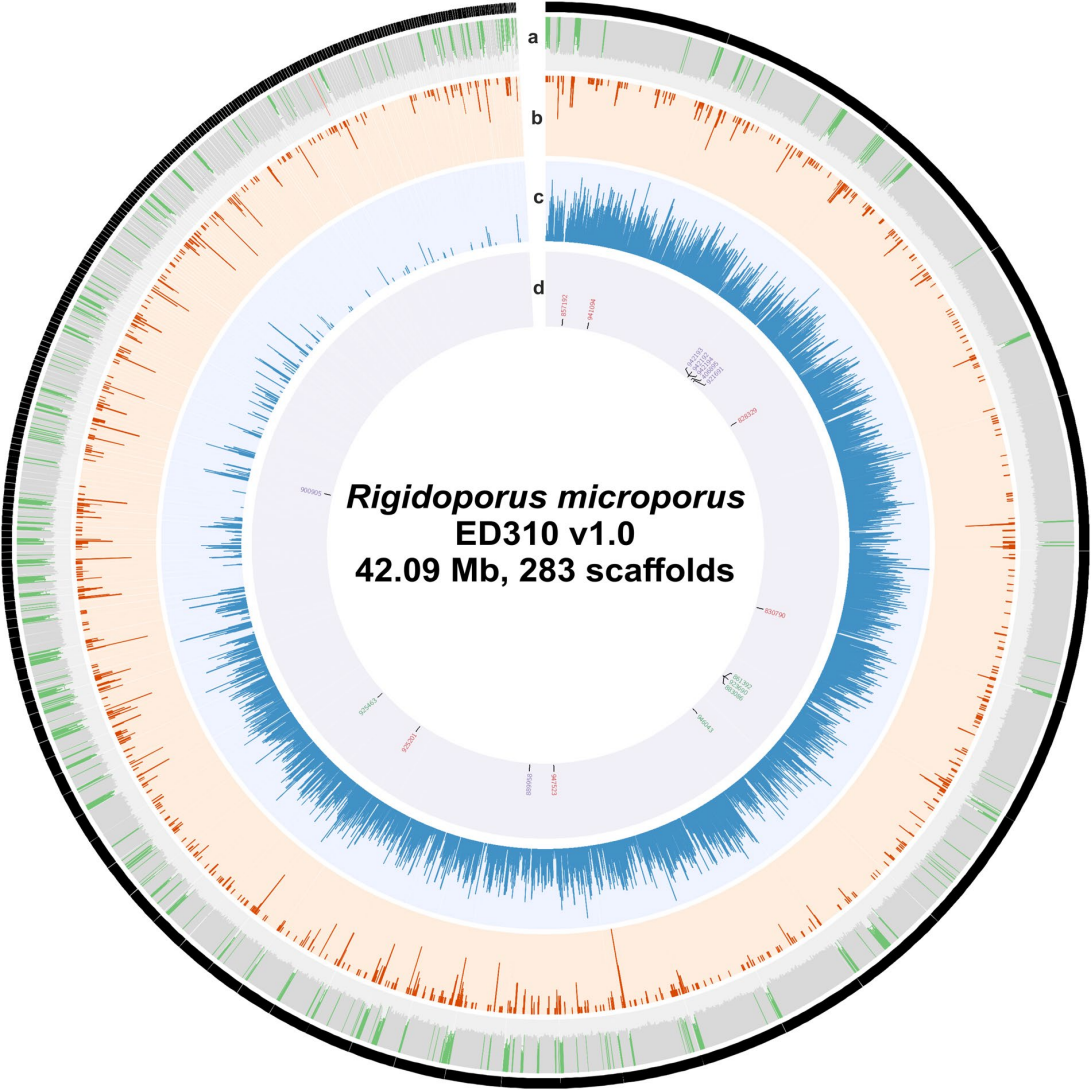
The *Rigidoporus microporus* ED310 v1.0 genome. The 283 scaffolds were arranged clockwise. Each circle from the outside to the inside depicts: (**a**) GC content (red > 0.55, green <0.45); (**b**) Transposable elements density; (**c**) Gene density; (**d**) Groups of protein id for multicopper oxidases (purple), manganese peroxidases (red) and GMC oxidoreductase (green).

The genome of *Rigidoporus microporus* was assembled into 283 contigs (281 contigs >2 kb) with an average read coverage depth of 80.32× and a genome assembly size of 42.09 Mbp (Fig. 2). The genome size of *R. microporus* is within the range reported for other members of *Hymenochaetales*. Using diverse gene prediction methods, which included the use of previously generated RNA-seq data^10^, we predicted 10,917 protein-coding genes. The mitochondrial genome of *R. microporus* contains 27 predicted protein-coding genes (Supplementary Table 3) and 24 tRNA genes (Supplementary Table 4). General properties of the *R. microporus* genome can be found in Table 1.

**Table 1:** Main features of the *Rigidoporus microporus* ED310 v1.0 genome assembly.

Genome Assembly	
Genome Assembly size (Mbp)	42.09
Sequencing read coverage depth	80.32×
# of contigs	283
Contig N50	12
Contig L50 (Mbp)	1.08
CEGMA	99.1%
Three largest contigs (Mbp)	2.49, 2.42, 2.19
Number of ESTs	34441
% mapped to genome	96.2%
Average gene length (bp)	1868
Average transcript length (bp)	1467
Average exon length (bp)	222
Average intron length (bp)	74
Average protein length (aa)	436
Average number of exons per gene	6.60
# of gene models	10917

### Phylogenetic analysis

**Figure 3:**
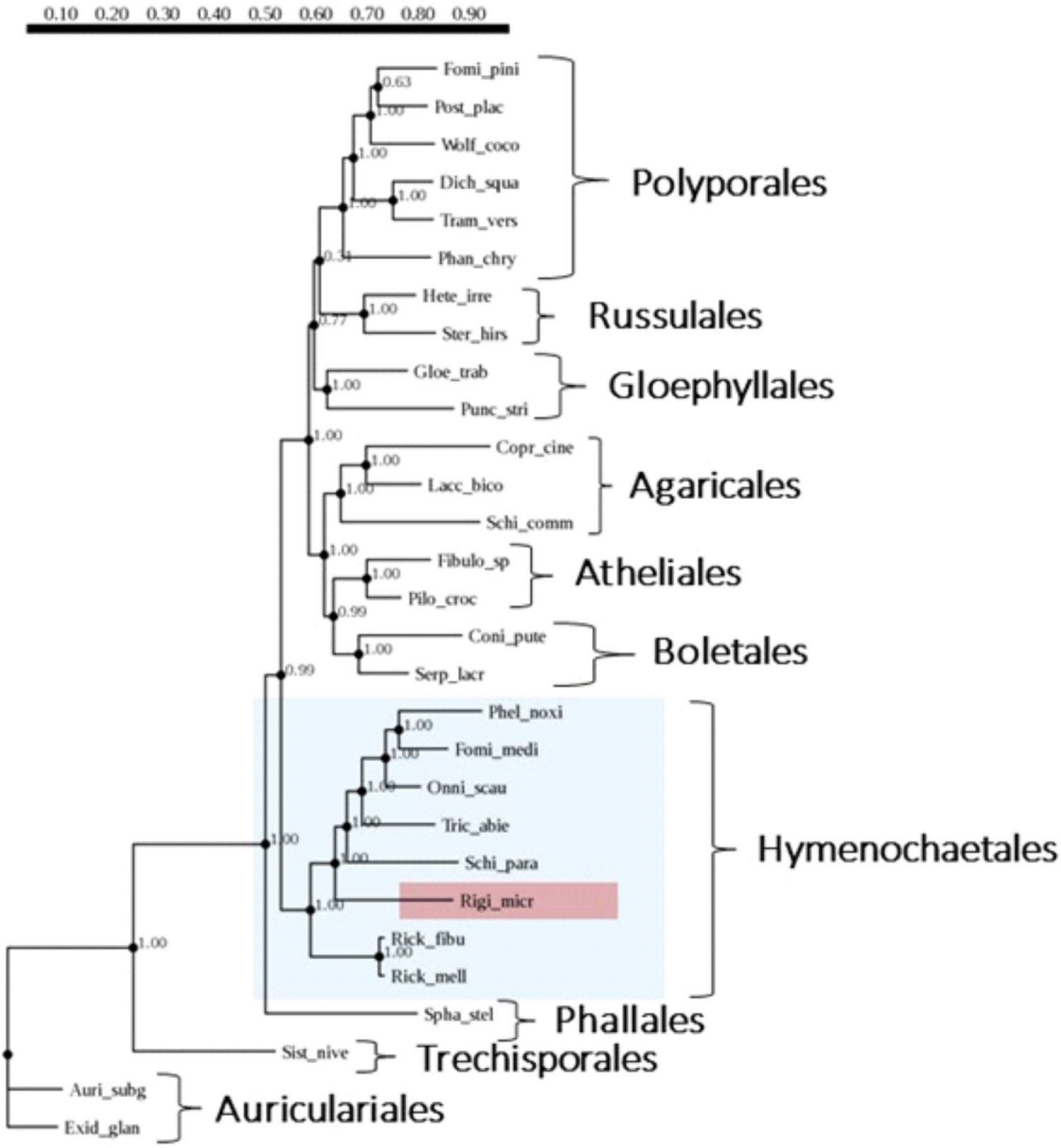
Maximum likelihood (ML) phylogenetic tree of 29 selected fungal species based on the analysis of 10 single copy gene data set using PhyML 3.0. *Auricularia subglabra* and *Exidia glandulosa* (both *Auriculariales*) were used as an outgroup.

Our phylogenetic analysis based on 10 single-copy conserved genes unambiguously places *R. microporus* within the order *Hymenochaetales*. Corresponding clade received 100% bootstrap support in both ML (Fig. 3) and NJ (Supplementary Fig. 9) analyses. *R. microporus* occupies a basal position within that group, being a second most basal branch after the two species of *Rickenella* (Fig. 3).

### Wood-degrading enzyme machinery

*R. microporus* possesses a repertoire of carbohydrate- and lignin-degrading enzymes characteristic for white-rot fungi. A comparison of the number of predicted *R. microporus* CAZy genes with that of other basidiomycetes is provided in Supplementary Table 5.

**Figure 4:**
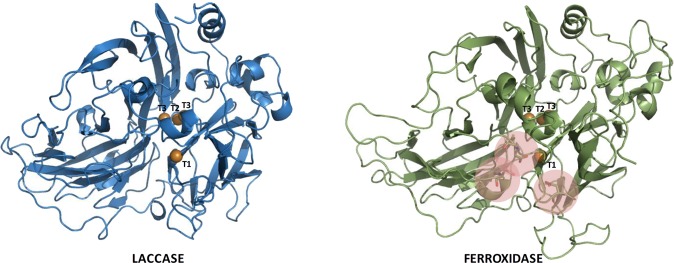
Structure models for *R. microporus* laccase and ferroxidase showing the overall folding in three cupredoxin-type domains, the four catalytic coppers are depicted as orange spheres and the Fe-binding site is highlighted in the ferroxidase model.

Predicted lignin-degrading enzymes are represented by six class II peroxidases (PODs), five laccases and eight GMC oxidoreductases (Supplementary Note 2). Analysis of the multicopper oxidase genes present in *R. microporus* genome gave a total of seven multicopper oxidases (MCO). Further molecular models of the distinctive proteins were built up to better determine the different types of MCOs (Fig. 4). It was concluded that two out of the seven MCO sequences corresponded to ferroxidases (889958, 900905) similar to the fungal Fet3 proteins, while the other five sequences were laccases (921691, 942192, 942193, 942194, 406895). The identified two ferroxidases, four laccases, four manganese peroxidases and three aryl alcohol oxidases also appeared in the mentioned reduced virulence gene list from PHI-base database, further supporting their roles of promoting fungal colonization and virulence.

Our analysis showed that *R. microporus* has the second lowest (after *Onnia scaura*) number of CAZy genes among the sequenced species of the order *Hymenochaetales*. Within the CAZys, *R. microporus* possess the lowest number of glycosyltransferases (GT) and the second lowest numbers of glycoside hydrolases (GH) and enzymes with axillary activities (AA) among the *Hymenochaetales*. At the same time, it has the highest number of LPMO (AA9) genes and the second highest number of CBM genes among the analyzed species of this order (https://genome.jgi.doe.gov/mycocosm/annotations/browser/cazy/summary;QzCg6t?p=Rigmic1). The number of *R. microporus* CAZymes tentatively involved in the degradation of hemicellulose and pectin is within the range observed in other *Hymenochaetales*, but lower than the average values for the sequenced species of *Agaricomycotina*. At the same time, *R. microporus* has the highest total number (39) of cellulose-degrading enzymes (members of the families GH6, GH7, GH45, GH74 and AA9) among the sequenced members of *Hymenochaetales*. This number is also significantly higher than the average for the class *Agaricomycotina*.

**Figure 5:**
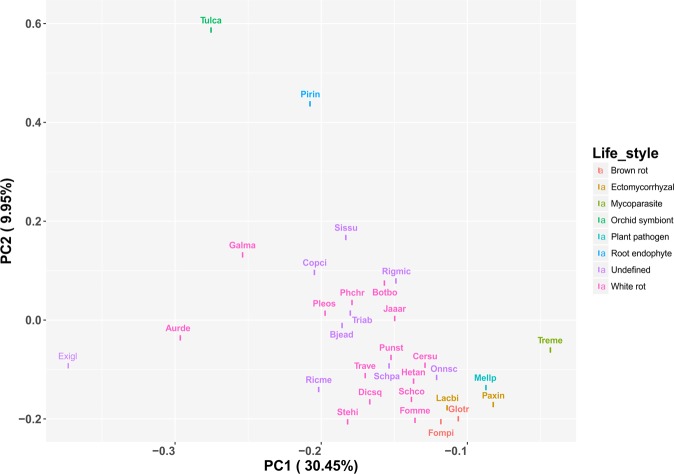
PCoA plot based on the number of CAZYme-encoding genes in the set of Agaricomycetes species of different lifestyles.

Principal coordinates analysis based on the copy numbers of different classes of CAZymes placed *R. microporus* close to *Botryobasidium botryosum* (*Cantharellales*) (Fig. 5), a species with a wood decay mode that might be plesiomorphic for the Agaricomycetes as a whole^54,55^. However, unlike *B. botryosum*, *R. microporus* possesses ligninolytic class II peroxidases (PODs) and laccases, characteristic for the white-rotting fungi.

### Secondary metabolism

The genome of *R. microporus* encodes a diverse set of genes implicated with a predicted role in secondary metabolism (Supplementary Table 6). The sole NRPS gene *nrp1* in *R. microporus* encodes a protein with the domain structure A-T-C-T-C, which is a common architecture for production of hydroxamate siderophores involved in iron homeostasis^56^. Indeed, the protein shows 27% identity to the well-characterized SidC protein from *Aspergillus fumigatus*^57^. However, the domain pattern of Nrp1 suggests that it is more likely a SidD homolog (20% identity). SidD condenses anhydromevanolyl hydroxyornithine to form the siderophore fusarinine C, which is further acetylated to triacetylfusarinine C (TAFC), the major siderophore in *Aspergillus nidulans* and *A. fumigatus* to acquire iron from the environment^58^. The formation of hydroxamate-based siderophores requires a specific monooxygenase for hydroxylation of L-ornithine or L-lysine to form precursor molecules for the siderophore biosynthesis^55^. A SMO1-like flavin-dependent monooxygenase (*mon1*) is encoded next to the *nrp1* indicating that *mon1* and *nrp1* form a SM gene cluster. It is therefore very likely that *R. microporus* produces hydroxamate-derived siderophores, as it has already been shown for other basidiomycetes such as *Laccaria* sp. and *Ceriporiopsis subvermispora B*^59,60^.

The *R. microporus* NPRS-like protein 1 Nlr1 is 65% identical to the L-α-aminoadipate reductase Lys2 from *C. subvermispora, Dichomitus squalens* and *Heterobasidion irregulare*. L-α-aminoadipate reductases catalyse the ATP- and NADH-dependent reduction of L-α-aminoadipate to its 6-semialdehyde, the 6^th^ step in fungal L-lysin biosynthesis starting from α-ketoglutaric acid. Like most Lys2-like enzymes, Nrl1 has an N-terminally extended domain (ADA domain), that is shown to be essential for its catalytic activity^61^. Therefore, we assume that *R. microporus* synthesizes L-lysine *de novo* as shown for many other fungi^62^.

In total, 16 ArmP-like terpene cyclases (TCs) encoded in 11 gene clusters were identified in the *R. microporus* genome (Supplementary Fig. 6). All TCs contain the aspartate (D) rich domain of the active site that coordinates a trinuclear Mg^2+^cluster that binds the diphosphate of the isoprenoid substrate^63^. However, 15 TCs have the classical consensus DDX motif where X is a hydrophobic amino acid such as Phe, Tyr, Trp, Leu or Val. In Ter12 one Asp residue is replaced by Glu at the first position (EDX) and the protein is probably inactive. The position of the active sites in the TCs is highly variable in *R. microporus*: The catalytic sites are positioned either at the N-terminus (9 TCs), at the C-terminus (4 TCs) or in the centre of the polypeptide chain (3 TCs). Active sites with a DDY/FX_2–3_CD consensus motif are exclusively found in N-terminal active sites. Consensus motifs of LIX_6–9_DX_2–3_DD are located in N-terminal and central active sites, whereas MDD consensus motifs are only observed in C-terminal active sites. Ter1 and Ter10 are highly identical (99.1%) and differ only in 3 amino acids (I286L, Y306H, M341V) suggesting that both TCs are redundant in their function as frequently observed in SM enzymes from basidiomycetes^64^.

Interestingly, two possible large SM gene cluster encode three TCs each (Ter3A-C and Ter6A-C, respectively). The close localization of the genes indicates a clustered co-expression, suggesting that *R. microporus* follows the biosynthesis gene cluster paradigm as shown for numerous species of ascomycetes and basidiomycetes^65^. Many reading frames were identified that may encode tailoring enzymes, often located in close vicinity to the key enzymes. The FAD, NAD or metal ion-dependent oxidoreductases/monooxygenases represent the largest group of possible backbone-modifying enzymes in *R. microporus*. Interestingly, within the cluster of *ter3A-C*, a possible major facilitator superfamily (MFS) transporter was found. MFS transporters are known to transport small molecules rather non-specifically into the extracellular space, which in turn causes resistance to the producer of anti-fungal metabolites^66^.

Three out of 11 terpene gene clusters encode C6 zinc finger proteins. C6-binuclear Zn (II)_2_Cys_6_ transcription factors have been shown to be involved in the regulation of sexual life cycle and secondary metabolism in asco- and basidiomycetes^67^. Both identified large gene clusters encode zinc finger proteins, suggesting that the set of genes are co-expressed and need special triggers to be activated such as stress conditions. Very often, these gene clusters remain silent under standard laboratory conditions^68^.

### Horizontal gene transfer events

Analysis of the predicted proteins with the program DarkHorse identified 59 candidate horizontally transferred genes with normalized LPI (Lineage Probability Index) scores of less than 0.8 and at least 10 BLAST hits. Of the 59 candidates, 5 had best hits to proteins from bacteria, and 47 had best hits to proteins from the Ascomycota (Supplementary Table 7). A phylogenetic tree was constructed for each candidate. Nine final HGT candidates were selected based on manual inspection of the phylogenetic trees. Among the nine final candidates, 2 indicate putative HGT events from bacteria and seven indicate HGT events from the Ascomycetes (Supplementary Fig. 6). The identified genes had similarities to pectate lyase (gene models 941964 and 975179), L-amino acid oxidase (983304), Na^+^/H^+^ exchanger family proteins (871220), fucose-specific lectin (1012254), peptidylprolyl isomerase (978311), aldehyde-activating protein (897619) and *Metarhizium anisopliae* and *Pseudogymnoascus* sp. hypothetical proteins (882912 and 870991, respectively).

### Expression of a sub-set of SSP or putative effector-like genes in interspecific fungal interactions

**Figure 6:**
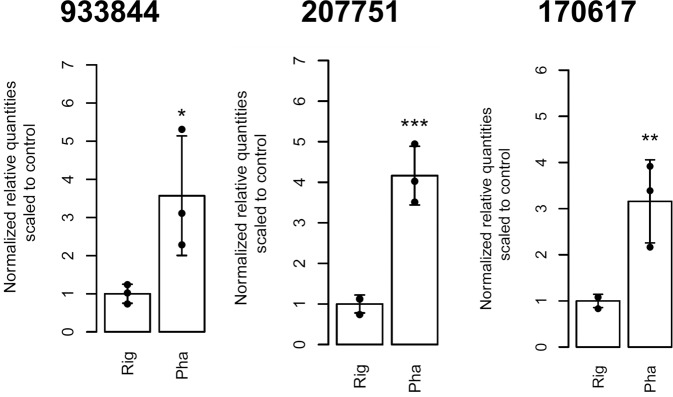
Expression of *R. microporus* SSP-encoding genes upon the interaction with the white-rot fungus *Phanerochaete chrysosporium*. Data for the time point 3 (a few days after initial hyphal contact) are presented. Genes showing statistically significant differences in the gene expression level are shown. Asterisks indicate the *p* value (*p < 0.05, **p < 0.01, ***p < 0.001). Rig – control experiment (*R. microporus* grown in pure culture), Pha – interaction of *R. microporus* with *P. chrysosporium*.

The development of fungal co-cultures is illustrated in Supplementary Fig. 6. No significant differences in the expression level of the analyzed genes could be observed at the time point 1 (Supplementary Fig. 6). At the time point 2, all selected genes except for the gene 933844 had lower expression level than in the control culture (Supplementary Fig. 7). At the time point 3, three genes encoding predicted small secreted proteins (933844, 207751, and 170617) were found to be upregulated in *R. microporus* co-cultured with saprotroph *P. chrysosporium* (Fig. 6). The gene 941140 was induced in the co-culture with *Mycena* sp., whereas the gene 692493 was downregulated in the co-cultures with *P. gigantea* and *Mycena* sp. (Supplementary Fig. 8).

### Transcriptomic analysis of *R. microporus* response to natural latex

Our RNA-seq analysis identified 51 genes with the significantly higher transcript abundance (FC ≥ 4, FDR ≤ 0.05) (Supplementary Table 8) and 199 genes with the significantly lower transcript abundance (FC ≤ 0.25, FDR ≤ 0.05) (Supplementary Table 9) during the growth on latex-containing medium. A list of the most highly up-regulated and down-regulated transcripts that have functional annotation are shown in Tables 2 and 3. The set of the genes with the higher expression level included three genes encoding predicted tripeptidyl peptidases, two aspartic protease genes, two genes for cytochrome P450 as well as genes for a putative GH16 glycosyl hydrolase, an ammonium transporter, a MFS transporter, a manganese peroxidase, a NADPH oxidase, a catalase and four hydrophobin-encoding genes. Among the genes with the lower transcript abundance, we identified two LPMO-encoding genes, genes for GH5 and GH13 glycosyl hydrolases, CE16 carbohydrate esterase and two genes encoding putative carbohydrate-binding module proteins CBM1 and CBM13. Additionally, Gene Ontology (GO) terms enrichment with respect to lower and higher transcript abundance is shown in Supplementary Table 10.

**Table 2:** Twenty most up-regulated genes with functional annotation during growth of *R. microporus* on latex.

Gene ID^a^	log2FC^b^	InterPro Hit ID^c^	InterPro Hit description	P value	FDR
fgenesh1_pm.14_#_20	5.033	IPR000209	Peptidase S8	8.46E-12	2.04E-08
gm1.2981_g	3.760	IPR001461	Aspartic peptidase	2.64E-13	8.46E-10
gm1.6302_g	3.728	IPR008972	Cupredoxin	1.02E-09	1.15E-06
fgenesh1_kg.26_#_192	3.589	IPR001128	Cytochrome P450	2.65E-21	2.55E-17
gm1.7580_g	3.370	IPR018487	Hemopexin-like repeats	4.18E-08	1.75E-05
fgenesh1_pg.13_#_190	3.301	IPR015366	Peptidase S53	3.04E-09	2.25E-06
fgenesh1_kg.9_#_415	3.058	IPR001338	Hydrophobin	8.53E-07	0.000158
gm1.8776_g	3.010	IPR001128	Cytochrome P450	1.85E-09	1.62E-06
gw1.23.264.1	2.907	IPR001338	Hydrophobin	4.55E-06	0.000534
fgenesh1_kg.28_#_197	2.900	IPR011701	Major facilitator superfamily	2.68E-05	0.001675
e_gw1.4.738.1	2.853	IPR001138	Zn(2)-C6 fungal-type DNA-binding domain	2.18E-13	8.46E-10
e_gw1.9.587.1	2.639	IPR001338	Hydrophobin	1.16E-09	1.15E-06
fgenesh1_kg.32_#_144	2.568	IPR001138	Zn(2)-C6 fungal-type DNA-binding domain	4.50E-06	0.000534
fgenesh1_pm.28_#_38	2.560	IPR011614	Catalase core domain	1.81E-08	9.16E-06
fgenesh1_kg.24_#_265	2.520	IPR013130	Ferric reductase transmembrane component	1.59E-07	4.50E-05
CE673895_2159	2.399	IPR001138	Zn(2)-C6 fungal-type DNA-binding domain	4.76E-10	7.64E-07
fgenesh1_pg.9_#_56	2.369	IPR000757	Glycoside hydrolase, family 16	5.73E-07	0.000117
gm1.4102_g	2.258	IPR000209	Peptidase S8	4.50E-08	1.80E-05
fgenesh1_pm.16_#_51	2.236	IPR000782	FAS1 domain	8.30E-10	1.14E-06
gm1.7636_g	2.216	IPR000120	Amidase	8.24E-06	0.000818

^a^corresponds to assembled and annotated genes from the transcriptome. ^b^Binary logarithm of fold change calculated from the fragments per kilobase per million reads (FPKM). ^c^is the best hit of InterPro database.

**Table 3:** Twenty most down-regulated genes with functional annotation during growth of *R. microporus* on latex.

Gene ID^a^	log2FC^b^	InterPro Hit ID^c^	InterPro Hit description	P value	FDR
fgenesh1_pg.19_#_67	−6.907	IPR001138	Zn(2)-C6 fungal-type DNA-binding domain	8.567E-06	8.189E-04
estExt_fgenesh1_pg.C_3_t10061	−6.691	IPR001810	F-box domain	6.455E-05	2.889E-03
fgenesh1_kg.3_#_116	−6.408	IPR009071	High mobility group box domain	1.294E-04	4.529E-03
gm1.9245_g	−6.378	IPR002523	Mg2+ transporter protein	1.737E-04	5.375E-03
estExt_Genemark1.C_210131	−6.250	IPR008030	NmrA-like domain	2.662E-04	7.038E-03
gm1.11294_g	−6.060	IPR002575	Aminoglycoside phosphotransferase	1.371E-03	2.045E-02
estExt_Genemark1.C_120167	−4.715	IPR023378	YheA	4.676E-05	2.381E-03
e_gw1.25.73.1	−4.325	IPR006094	FAD linked oxidase	2.987E-04	7.544E-03
estExt_Genemark1.C_240010	−4.318	IPR002921	Fungal lipase-like domain	2.672E-04	7.043E-03
gm1.1019_g	−4.262	IPR002048	EF-hand domain	2.963E-05	1.767E-03
CE350168_11990	−3.731	IPR003819	Taurine catabolism dioxygenase TauD	3.026E-05	1.767E-03
estExt_fgenesh1_pg.C_3_t10054	−3.697	IPR001128	Cytochrome P450	4.287E-04	9.684E-03
fgenesh1_pg.13_#_14	−3.663	IPR001938	Thaumatin	6.251E-06	6.915E-04
gm1.5464_g	−3.605	IPR000772	Ricin B lectin domain	2.218E-06	3.222E-04
gm1.9351_g	−3.544	IPR009078	Ferritin-like superfamily	2.931E-03	3.187E-02
CE623133_924	−3.475	IPR001128	Cytochrome P450	1.158E-05	1.014E-03
fgenesh1_kg.11_#_723	−3.245	IPR001138	Zn(2)-C6 fungal-type DNA-binding domain	3.337E-05	1.911E-03
e_gw1.5.1225.1	−3.230	IPR000719	Protein kinase domain	3.028E-03	3.261E-02
e_gw1.15.148.1	−3.188	IPR001128	Cytochrome P450	8.441E-05	3.377E-03
gm1.5005_g	−3.125	IPR007111	NACHT nucleoside triphosphatase	7.097E-09	4.553E-06

^a^corresponds to assembled and annotated genes from the transcriptome. ^b^Binary logarithm of fold change calculated from the fragments per kilobase per million reads (FPKM). ^c^is the best hit of InterPro database.

### Genes putatively involved in pathogen/host interactions

The predicted set of *R. microporus* proteins included 813 putative secreted proteins. Among the secreted proteins that have hits in the database PHI-base^33^, 229 proteins have hit annotations of “reduced virulence”, “loss of pathogenicity” or “effector (plant avirulence determinant)”. The hits of 38 proteins were annotated as “effectors” based on experimental evidence collected in this database. A total of 70 and 201 proteins were assigned to the annotation of “reduced virulence” and “loss of pathogenicity”, respectively (Supplementary Table 11). Potential function of effectors based on PHI blast is listed in Supplementary Table 12.

## Discussion

The availability of the genome sequence of *R. microporus* provides excellent opportunities for the genome mining and comparative analysis of this important tree pathogen. Our multigene phylogenetic analysis clearly showed that *R. microporus* is nested within the representatives of the order *Hymenochaetales*. This observation agrees with the results of the previous phylogenetic reconstructions^3,69^. However, the placement of *R. microporus* within the order of *Hymenochaetales* might require some nomenclatural rearrangements, as the type species of the genus *Rigidoporus* belongs to the order *Polyporales*^8^. The nomenclatural issues concerning *Rigidoporus* and related species will be fully addressed in a separate forthcoming publication (Miettinen *et al*., *in prep*.).

The size of the *R. microporus* genome is similar to the genome size of such members of *Hymenochaetales* as *Trichaptum abietinum*, *Schizopora paradoxa* and *Onnia scaura*. However, the number of predicted protein-coding genes in *R. microporus* is somewhat lower than in the mentioned species.

*R. microporus* is an efficient wood decomposer, capable of simultaneous degradation of lignin and cellulose, characteristic for white-rot fungi^2^. Our analysis of PCW-degrading capabilities of *R. microporus* revealed that this species has relatively low numbers of GT- and GH-encoding genes. At the same time, the repertoire of CAZys encoded by *R. microporus* reflects its specialization towards an efficient cellulose degradation. In particular, a high number of LPMO-encoding genes in *R. microporus* genome might indicate the important role of this class of enzymes in cellulose degradation. The number of genes encoding lignin-degrading enzymes in *R. microporus* genome is lower than in other representatives of *Hymenochaetales*, nevertheless, their repertoire is sufficient for lignin degradation, as indicated by the *in vitro* experiments on wood decomposition^2^. The presence of lignin-degrading peroxidases clearly separates *R. microporus* from *Botryobasidium botryosum*, even if these two species grouped together in our PCoA analysis based on the copy number of CAZyme-encoding genes.

A role of *R. microporus* secondary metabolites (SM) in the infection process or in the metabolic switch from the necrotrophic to saprotrophic lifestyle is not established. The majority of encoded key enzymes involved in SM production are terpene cyclases (TC) (Supplementary Table 6). Hence, soluble and volatile terpenes seem to be the major metabolites produced by *R. microporus*, as already demonstrated for other higher fungi^70^. In addition, there are a few characteristic multidomain enzymes such as one non-ribosomal peptide synthetase (NRPS) protein and a NRPS-like protein encoded within the genome. However, putative polyketide synthases (PKS) or PKS-NRPS hybrid genes seems to be absent in the genome. In contrast, a wide variety of SM tailoring enzymes, such as monooxygenases and methyltransferases are encoded to modify the terpenoid backbones. There are no Arm1-like flavin-dependent halogenases encoded in the genome, suggesting that no halogenated terpenes are formed. Furthermore, many of the SM genes are clustered and contain regulatory elements (transcription factors) and/or putative transporters for efficient metabolite export. An in-depth analysis of the fungus´ metabolome and volatome may not only contribute to the discovery of novel biosynthetic routes and compounds but would also unravel the ecological impact of the produced metabolites on the fungus itself, its host tree, forest ecosystem or its microbial competitors.

The majority of the candidate horizontally transferred genes appear to have originated from ancestral members of the Ascomycota. This was unexpected since, in the case of *Colletotrichum* spp., bacteria appeared to be the most frequent donors of horizontally transferred genes^71^. In fact, most horizontally transferred genes in fungi are of bacterial origin^22^ and the presence of a relatively large number of ascomycete to *R. microporus* transfers suggests that there may be a unique aspect to the *R. microporus* ecology or biology that enables such transfers. An alternative explanation to HGT is that the genes were vertically inherited from a common ascomycete/basidiomycete ancestor. This would require massive numbers of gene losses in many basidiomycete lineages, an explanation that is less parsimonious as it requires a larger number of evolutionary changes. Also, if HGT candidates were the product of vertical inheritance followed by gene loss, then one would expect to find basidiomycete/ascomycete HGT candidates in the ascomycetes, which is not the case.

The use of artificial agar media to assess fungal growth and interactions have always been a major concern particularly on whether such results could be extrapolated to natural conditions^72,73^. Previous studies have used artificial agar media to assess the dynamics of fungal growth and interactions^74,75^. Other authors^76^, noted that growth in artificial medium provides best possible ways of analyzing fungal interactions and growth. Other studies^77^ reported that interactions of some fungi on woody substrate and artificial (agar) media did not significantly vary. The analysis of interspecific interactions between *R. microporus* and other fungi indicated a potential role of SSP in these interactions. The changes in the expression of SSP-encoding genes were particularly pronounced in the case of interaction of *R. microporus* with the saprotroph *P. chrysosporium*. The latter species was characterized by faster growth rate, and the higher expression of SSP by *R. microporus* could be a defense reaction against a strong competitor. Previous studies have shown saprotrophic basidiomycetes SSPs as putative effectors^78^. The interactions of *R. microporus* with saprotrophic biocontrol agent *P. gigantea* and saprotrophic *Mycena* sp. had less pronounced effect on the expression of SSP-encoding genes.

*R. microporus* is a successful pathogen of rubber trees, able to efficiently colonize and kill living trees. During the host colonization process, the fungus likely interacts with the latex, abundantly produced by the host tree. However, the role of latex in the antimicrobial defense reactions of rubber tree is not well understood. Due to practical and technical limitations, conducting *in-vivo* study of the growth of the fungus on latex on natural wood substrate was not feasible. In this study, the alternate choice of using natural latex in artificial media for the bioassay was reinforced based on results from earlier published papers. We could not observe any fungistatic or fungicidic effect of latex in our *in vitro* experiments. On the contrary, fungal cultures developed faster on media supplemented with latex, indicating that some of its components might be metabolized by the fungus. The main component of natural latex is poly (*cis*-1,4-isoprene), a polymer highly resistant to microbial degradation. However, latex also contains a fraction of proteins, preventing latex from coagulation *in planta*. Cis-1,4-polyisoprene, a main constituent of latex rubber, is indeed hydrophobic^79^. In planta latex particles are surrounded by hydrophilic layer, which prevents them from the aggregation within latex-carrying vessel elements. It is however possible that the ability to degrade latex might be the most relevant at the initial stages of pathogen establishment, when it has to cross host tissues rich in latex during post-penetration into sapwood. It is also possible that Rubber trees might use latex to seal wounds in a similar way as conifer trees use oleoresin. However, once the fungus reaches the sapwood, the ability to degrade latex might become less relevant, as there is very little latex (if any) in the sapwood. Furthermore, in our transcriptome data, several gene transcripts encoding hydrophobin proteins were differentially expressed during growth on rubber wood and latex. It is possible as it has been documented in other studies that the hydrophobin facilitates the growth of fungal hyphae into the air from moist environments thereby facilitating host interactions as well as infectivity and invasive growth of pathogenic fungi^80^. We are however unable to speculate on the precise impact of the hydrophobin genes on the *Rigidoporus* cell wall and the consequent interaction, further studies will be required to prove it. We were not able to identify homologs of bacterial proteins involved in latex degradation (rubber oxygenase roxA^81^ and latex-clearing protein lcp^82^) in the genome of *R. microporus*. The analysis of RNA-seq data suggests that *R. microporus* might utilize proteinaceous components of the latex, as higher transcript abundance was found for several genes encoding proteases and transporter proteins. A number of genes encoding oxidative enzymes also were up-regulated, however, it remains unclear whether any of those can participate in rubber degradation, as available data indicate that dioxygenase activity is required for the cleavage of polyisoprene backbone^83^.

*R. microporus* is a fungal pathogen of considerable economic importance, and the availability of its genome sequence provides better opportunities for the understanding of its pathogenicity determinants and for the development of advanced control strategies. Our analysis contributed to the establishment of phylogenetic relationships of this species and confirmed its position within the order of *Hymenochaetales*. The analysis of the repertoire of PCW-degrading enzymes encoded in the genome of *R. microporus* indicated that, despite the lower numbers of lignin-degrading peroxidases, GT and GH-encoding genes compared with other white-rot fungi, *R. microporus* has the capacity for efficient wood degradation. It possesses a diverse set of SM genes, and the biological role of their corresponding products deserves further investigations. Being a white-rot fungus, members of the genus *Rigidoporus* are known to play major roles in nutrient and carbon cycling in tropical forest. It is therefore not surprising that *R. microporus* harbor a repertoire of a wide range of useful enzymes important for lignocellulose degradation with potential applications in bioenergy processing and utilization.

The availability of both the pathogen and host genome would help to facilitate the long-needed detailed studies on resistance research and host-related interactions as well as on the ecology and physiology of the pathogen. The genome has also provided much needed insight and clarification on systematics of the *Hymenochaetales*. Furthermore, the control and management of white rot disease of rubber in most tropical countries have been done without knowledge of the population genetics of the different isolates obtainable from each country and continent. Most management practices have been based on clearing and burning of infected roots and stumps. Such measures can reduce the disease incidence. Knowledge of population genetics of plant pathogens can facilitate breeding efforts to control plant diseases. There are only a few population genetic studies reported for *R. microporus* pathogen on rubber tree. These include studies in Asia on genetic variability and characterization^84,85^ and somatic incompatibility^86^ of the pathogen in different geographic areas in Thailand and Indonesia respectively. They are limited in scope, and there is therefore a need for a detailed study of the genomics, population biology, population genetics and host pathogen interaction of this economical important tropical tree pathogen. This can now be greatly facilitated by availability of the first genome sequence of this rubber tree pathogen. We expect that the availability of the genomic data will further stimulate studies on the biology of this fungal species and its role in wood decay and carbon cycling in nature.

## Supplementary information

Supplementary Information.

Supplementary Table 5.

Supplementary Table 7.

Supplementary Table 8.

Supplementary Table 9.

Supplementary Table 10.

Supplementary Table 11.

Supplementary Table 12.

## Data Availability

Genome assembly and annotations used in this study are available at the JGI fungal genome portal MycoCosm (http://jgi.doe.gov/fungi). The genome of *R. microporus* can be accessed at https://genome.jgi.doe.gov/Rigmic1/Rigmic1.home.html. The genome assembly has been deposited at DDBJ/EMBL/GenBank under the following accession number PRJNA345706. The raw data from the transcriptome have been submitted to the National Center for Biotechnology Information under the following accession number PRJNA497786.
