# Author Correction: Genome sequencing of *Rigidoporus microporus* provides insights on genes important for wood decay, latex tolerance and interspecific fungal interactions

**DOI:** 10.1038/s41598-020-74783-6

**Published:** 2020-10-20

**Authors:** Abbot O. Oghenekaro, Andriy Kovalchuk, Tommaso Raffaello, Susana Camarero, Markus Gressler, Bernard Henrissat, Juna Lee, Mengxia Liu, Angel T. Martínez, Otto Miettinen, Sirma Mihaltcheva, Jasmyn Pangilinan, Fei Ren, Robert Riley, Francisco Javier Ruiz-Dueñas, Ana Serrano, Michael R. Thon, Zilan Wen, Zhen Zeng, Kerrie Barry, Igor V. Grigoriev, Francis Martin, Fred O. Asiegbu

**Affiliations:** 1 Faculty of Life Sciences, Department of Plant Biology and Biotechnology, University of Benin, P.M.B 1154, Benin City, Nigeria; 2 Faculty of Agriculture and Forestry, Department of Forest Sciences, University of Helsinki, P.O. Box 27, N00014 Helsinki, Finland; 3 Centro de Investigaciones Biológicas, Consejo Superior de Investigaciones Científicas, Ramiro de Maeztu 9, E28040, Madrid, Spain; 4 Department of Pharmaceutical Microbiology at the Hans Knöll Institute, Friedrich Schiller University, Jena, Germany; 5 Aix-Marseille Université, Architecture et Fonction des Macromolécules Biologiques, CNRS, UMR 7257, 13288 Marseille, cedex 9, France; 6 USC1408 Architecture et Fonction des Macromolécules Biologiques, Institut National de la Recherche Agronomique, 13288 Marseille, France; 7 Department of Biological Sciences, King Abdulaziz University, Jeddah, 23218 Saudi Arabia; 8 US Department of Energy Joint Genome Institute, Lawrence Berkeley National Laboratory, 1 Cyclotron Road, Berkeley, CA 94720 USA; 9 Department of Plant and Microbial Biology, University of California Berkeley, Berkeley, CA 94720 USA; 10 Mycology Unit, Botanical Museum, Finnish Museum of Natural History, University of Helsinki, P.O. Box 7, Helsinki, Finland; 11 Forestry experiment center of north China, Chinese Academy of Forestry, Beijing, 102300 China; 12 Universidad de Salamanca, Instituto Hispano-Luso de Investigaciones Agrarias (CIALE), Villamayor, Spain; 13 Institut National de la Recherche Agronomique (INRA), Laboratory of Excellence Advanced Research on the Biology of Tree and Forest Ecosystems (ARBRE), UMR 1136, Champenoux, France; 14 University of Lorraine, Laboratory of Excellence ARBRE, UMR 1136, Champenoux, France; 15 Present Address: Department of Plant Science, University of Manitoba, Winnipeg, MB R3T 2N2 Canada

Correction to: *Scientific Reports* https://doi.org/10.1038/s41598-020-62150-4, published online 23 March 2020.

The original version of this Article contained errors in the author affiliations.

Otto Miettinen was incorrectly affiliated with ‘Department of Plant and Microbial Biology, University of California Berkeley, Berkeley, CA, 94,720, USA.’ The correct affiliation is listed below.

Mycology Unit, Botanical Museum, Finnish Museum of Natural History, University of Helsinki, P.O. Box 7, Helsinki, Finland.

Fei Ren was incorrectly affiliated with ‘Mycology Unit, Botanical Museum, Finnish Museum of Natural History, University of Helsinki, P.O. Box 7, Helsinki, Finland’. The correct affiliations are listed below.

Faculty of Agriculture and Forestry, Department of Forest Sciences, University of Helsinki, P.O. Box 27, FIN-00014, Helsinki, Finland.

Forestry experiment center of north China, Chinese Academy of Forestry, 102300, Beijing, China.

Michael R. Thon was incorrectly affiliated with ‘Forestry experiment center of north China, Chinese Academy of Forestry, 102300, Beijing, China’. The correct affiliation is listed below.

Universidad de Salamanca, Instituto Hispano-Luso de Investigaciones Agrarias (CIALE), Villamayor, Spain.

Igor V. Grigoriev was incorrectly affiliated with ‘Universidad de Salamanca, Instituto Hispano-Luso de Investigaciones Agrarias (CIALE), Villamayor, Spain’. The correct affiliations are listed below.

US Department of Energy Joint Genome Institute, Lawrence Berkeley National Laboratory, 1 Cyclotron Road, Berkeley, CA, 94720, USA.

Department of Plant and Microbial Biology, University of California Berkeley, Berkeley, CA, 94720, USA.

These errors have now been corrected in the PDF and HTML versions of the Article.

