# Supplementary material for: Genome sequencing of Rigidoporus microporus provides insights on genes important for wood decay, latex tolerance and interspecific fungal interactions
Source: Sci Rep. 2020 Mar 23;10:5250. doi: 10.1038/s41598-020-62150-4 (PMC7089950; doi:10.1038/s41598-020-62150-4)
Supplement: Supplementary file 1 — Supplementary Information. [file 41598_2020_62150_MOESM1_ESM.pdf]

Genome sequencing of *Rigidoporus microporus* provides insights on genes important for wood decay, latex tolerance and interspecific fungal interactions

Abbot O. Oghenekaro, Andriy Kovalchuk, Tommaso Raffaello, Susana Camarero, Markus Gressler, Bernard Henrissat, Juna Lee, Mengxia Liu, Angel T. Martínez, Otto Miettinen, Sirma Mihaltcheva, Jasmyn Pangilinan, Fei Ren, Robert Riley, Francisco Javier Ruiz-Dueñas, Ana Serrano, Michael R. Thon, Zilan Wen, Zhen Zeng, Kerrie Barry, Igor V. Grigoriev, Francis Martin, Fred O. Asiegbu

Supplementary Notes

Supplementary Note 1. Protocol for the isolation of *R. microporus* genomic *DNA*

BUFFER A: 0.35 M sorbitol
0.1 M Tris-HCl, pH 9
5 mM EDTA, pH 8

BUFFER B: 0.2 M Tris-HCl, pH 9
50 mM EDTA, pH 8
2 M NaCl
2% CTAB

BUFFER C: 5% Sarkosyl (N-lauroylsarcosine sodium salt, SIGMA L5125)

Potassium Acetate 5M (pH 7.5)
RNAse A (10 mg/ml)
Proteinase K (20 mg/ml)
PVP 1 %Chloroform:Isoamylalcohol (24:1) or phenol/chloroform/isoamyl alcohol (25:24:1)
Sodium Acetate (NaAc) 3M
Isopropanol 100%
Ethanol 70%

Lysis Buffer for 17.5 ml:

2.5 volume of Buffer A:	6.5 ml
2.5 volume of Buffer B:	6.5 ml
1.0 volume of Buffer C:	2.6 ml
PVP 0.1 %	1.75 ml
Proteinase K (20 mg/ml)	125 µl

NB: phenol/chloroform/isoamyl alcohol (25:24:1) can be replaced by chloroform/Isoamyl alcohol (24:1)

1. Pre-warm the lysis buffer at 65°C.
2. Harvest mycelia in a cold Falcon 50ml tube. Up to 500mg can be used in a single extraction.
3. Grind the mycelia with liquid nitrogen and mortar/pestle.
4. Add the 17.5ml of LYSIS BUFFER and mix by inverting the tube several times until the powder mycelium is dissolved.
5. Incubate 30 min at 65°C and mix by gentle inverting frequently (every 2-3 min).
6. Add 5.75ml KAc (5 M). Mix by inverting the tube, and incubate 30 min on ice.
7. Centrifuge 20 min at 5000 g at 4°C.
8. Transfer the water phase in a new Falcon tube and add 1 volume of phenol/chloroform/isoamyl alcohol (25:24:1) and mix by inverting.
9. Centrifuge for 10 min at 4000 g at 4°C.
10. Transfer the supernatant in a new Falcon tube.

11. Add 1/10 volume of NaAc (3M) and 1 volume of isopropanol (at room temp) and incubate 5 min at room temperature.
12. Centrifuge 30 min at 10000 g at 4°C.
13. Discard supernatant and wash pellet with 2 ml 70% ethanol.
14. Centrifuge 10 min at 10000 g at 4°C.
15. Discard the supernatant and dry the pellet (around 10 min in laminar flow).
16. Resuspend the pellet in 500 µl (or less) TE buffer.
17. Transfer to a 1.5ml Eppendorf tube.
18. Add RNase A at 100 µg/ml final concentration and incubate at 37°C for 1 to 2 hr.
19. Add 1 volume of phenol/chloroform/isoamyl alcohol (25:24:1) and mix by inverting.
20. Centrifuge for 10 min at 4000 g at 4°C.
21. Transfer the supernatant in a new Eppendorf tube.
22. Add 1/10 volume of NaAc (3M) and 1 volume of isopropanol (at room temp) and incubate 5 min at room temperature.
23. Centrifuge 30 min at 10000 g at 4°C.
24. Discard supernatant and wash pellet with 1 ml 70% ethanol.
25. Centrifuge 10 min at 10000 g at 4°C.
26. Discard the supernatant and dry the pellet (around 10 min in laminar flow).
27. Resuspend the pellet in 100 µl (or less) TE buffer.
28. Store at -80°C.

Supplementary Note 2. Class-II peroxidases, multicopper oxidases and GMC oxidoreductases in the genome of *Rigidoporus microporus* ED310 v1.0

Class-II peroxidases

Manual annotation of the different peroxidase gene models

Six Class-II gene models were identified and manually annotated based on: i) the highest sequence identities for each protein sequence derived from the predicted gene; ii) multiple alignment with 145 heme peroxidase protein sequences from ten species of *Polyporales* and two species of *Hymenochaetales*; and iii) examination of theoretical molecular structures obtained by homology modeling using crystal structures of related peroxidases as templates and programs implemented by the automated protein homology modeling server "SWISS-MODEL" [1].

The six heme peroxidases could be annotated as manganese peroxidases on the basis of the presence of a Mn(II)-oxidation site near the internal heme propionate formed by three acidic residues homologous to *Phanerochaete chrysosporium* MnP1 Glu35, Glu39 and Asp179. They were subsequently classified as members of two MnP subfamilies differing in both catalytic properties and stability [2]: i) MnP-short (3 models with IDs 828329, 941094 and 947523) with Mn-mediated and Mn-independent activity on low redox potential substrates; and ii) MnP-long (3 models with IDs 830790, 857192 and 925201) only able to oxidize Mn^{2+}, that include a C-terminal tail extension associated with their high acidic stability and catalytic properties.

A representation of the homology models obtained for two of the six MnPs identified in *R. microporus* ED310 v1.0, including key amino acid residues putatively involved in catalysis, is

presented in Fig. A. These models were obtained using different crystal structures of ligninolytic peroxidases deposited at PDB (https://www.rcsb.org/pdb/home/home.do) as templates.

Comparison with other Class-II peroxidases from Polyporales

MnPs derived from ancestral GPs were the first ligninolytic peroxidases to appear at the end of Carboniferous period by progressive incorporation of three acidic residues forming the Mn-oxidation site [3]. A well-supported explanation for the subsequent evolution of ligninolytic peroxidases from an ancestral MnP in the order *Polyporales* has been recently described by reconstruction and resurrection of ancestral peroxidases [4]. As a result of this evolutionary process, VPs arose by incorporation of a catalytic tryptophan in an ancestral MnP. Then the Mn(II)-oxidation site was lost in ancestral VPs giving rise to LiP enzymes several times independently in the evolution of *Polyporales* [5]. Unlike what is observed in white-rot *Polyporales,* MnPs are the only ligninolytic enzymes found in the *Hymenochaetales* genomes sequenced to date (including *R. microporus*) [3, 6] (**Table A1**), suggesting a different evolutionary history of these enzymes in both fungal orders. Concerning *Hymenochaetales,* the number of MnP isoenzymes identified in *R. microporus* (6 representatives) is significantly lower compared with that found in the other two species analyzed belonging to this order (12 and 16 representatives in *Schizopora paradoxa* and *Fomitiporia mediterranea*, respectively). However, the diversity of MnP subfamilies encoded in its genome (short and long MnPs) is intermediate between *F. mediterranea* (encoding short, long and atypical MnPs) and *S. paradoxa* (only containing long MnP genes).

We have prepared a phylogenetic tree by maximum likelihood analysis of 118 ligninolytic peroxidases identified in eleven *Polyporales* species (including those from *R. microporus*) (Fig. B). As observed in this tree, MnPs appear grouped in different clusters. Long MnPs from *R. microporus* are clustered together with the classical long MnPs from *P. chrysosporium*[7] and the well characterized enzymes from *Ceriporiopsis subvermispora*[8], although forming a separate group inside this cluster suggesting that some differences could exist between them (in catalytic activity, stability, etc.). Regarding the short MnPs, 947523 appears isolated, arising from the same ancestral node from which a large cluster comes out. On one hand, this large cluster follows the evolutionary path to LiPs (on the top). On the other hand, a small miscellaneous group emerges containing a few ligninolytic peroxidases, including the two other short MnPs from *R. microporus* (828329 and 941094) together with four additional short MnPs from *C. subvermispora*, *Bjerkandera adusta*, *Trametes versicolor* and *Phlebia brevispora*, two VP-LiP from *C. subvermispora* occupying an intermediate position between typical LiPs and VPs according to their structural and catalytic properties[9] and one GP from *B. adusta*.

A phylogenetic tree was constructed by maximum likelihood analysis based on amino acid sequences of 143 ligninolytic peroxidases and eight generic non-ligninolytic peroxidases identified in ten *Polyporales* and three *Hymenochaetales* species (including *R. microporus*) (**Fig. B**). In the produced tree, MnPs from *R. microporus* appear grouped in three different regions of the tree. The long MnPs of this fungus are clustered with long MnPs identified in other *Hymenochaetales* species (i.e. *F. mediterranea* and *S. paradoxa*). Long MnPs from *Hymenochaetales*, in turn, form a well-defined cluster with that of long MnPs from *Polyporales*, including the classical MnPs from *P. chrysosporium* and the well-characterized enzymes from *Ceriporiopsis subvermispora* [8]. The

appearance of long MnPs from *Polyporales* and *Hymenochaetales* in two well-defined groups within the same cluster suggests that both groups come from a common ancestor that arose long before the appearance of the first fungi belonging to these two orders, and that their properties (e.g. catalytic activity, stability, etc.) most probably will be different, although this still has to be demonstrated.

Concerning short MnPs, 947523 from *R. microporus* appears grouped with a short MnP (ID 91941) from *F. mediterranea*, both *Hymenochaetales* enzymes arising from the same ancestral node from which a large cluster comes out. On one hand, this large cluster follows the evolutionary path to the *Polyporales* LiPs (on the top). On the other hand a miscellaneous group emerges containing ligninolytic peroxidases from different families, including: i) the two other short MnPs from *R. microporus* (828329 and 941094), which are closely related to the short MnP 123070 from *F. mediterranea*; ii) twenty two additional short MnPs from *Ceriporiopsis subvermispora*, *Bjerkandera adusta*, *Trametes versicolor*, *Ganoderma* sp., *Dichomitus squalens* and *Phlebia brevispora*; iii) two VP-LiP from *C. subvermispora* occupying an intermediate position between typical LiPs and VPs according to their structural and catalytic properties [9]; and iv) one GP from *B. adusta*.

Fig. A. Homology models for the molecular structures of two Class-II heme peroxidases from the *R. microporus* ED310 v1.0 genome. A) MnP-short (947523) and B) MnP-long (857192) harboring a putative Mn2+ oxidation site (formed by two glutamates and one aspartate).

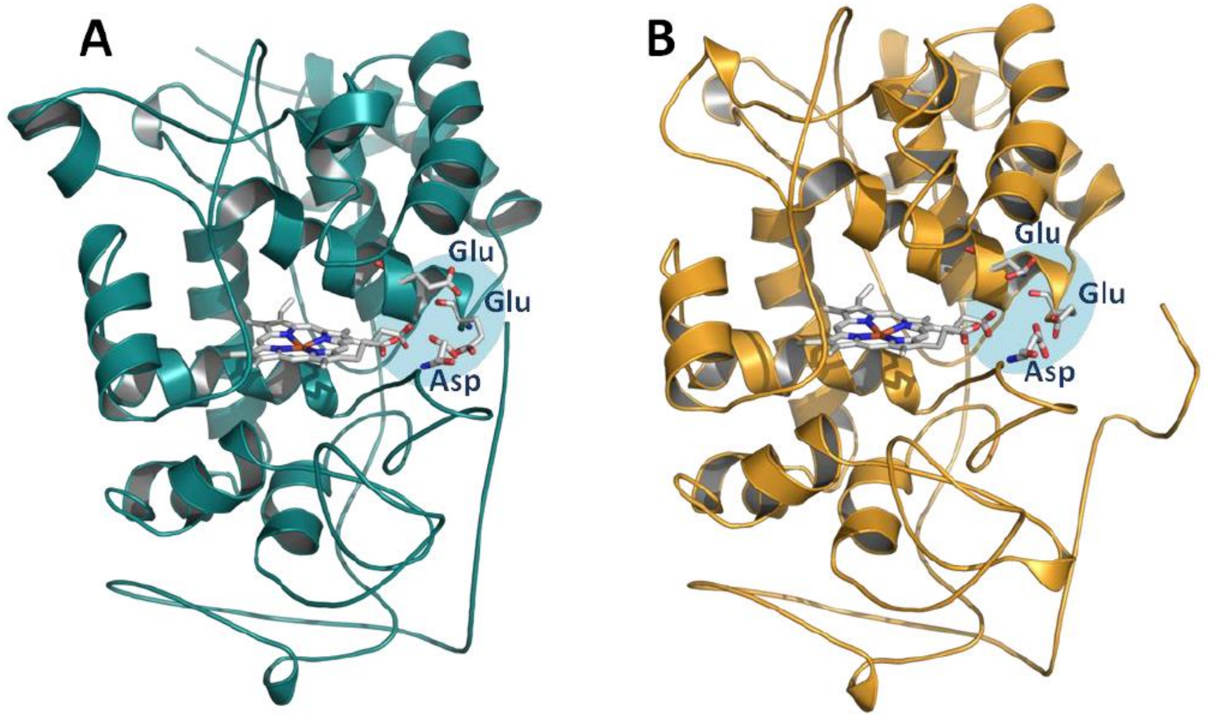

Table A1. Inventory of genes (151) of Class-II peroxidases (from the lignin peroxidase, LiP; versatile peroxidase, VP; generic peroxidase, GP families; and the short and long MnP subfamilies) in the genomes of ten species of *Polyporales* (Bjead, *Bjerkandera adusta*; Phlbr, *Phlebia brevispora*; Phach, *Phanerochaete chrysosporium*; Dicsq, *Dichomitus squalens*; Gansp, *Ganoderma* sp.; Trave, *Trametes versicolor*; Cersu, *Ceriporiopsis subvermispora*; Fompi, *Fomitopsis pinicola*; Pospl, *Postia placenta*; and Wolco, *Wolfiporia cocos*) and three species of *Hymenochaetales* (Fomme, *Fomitiporia mediterranea*; Schpa, *Schizopora paradoxa*; and Rigmi, *Rigidoporus microporus*) able to produce white-rot and brown-rot wood decay. Atypical MnPs and atypical VPs are characterized by lacking one of the three acidic residues (two glutamates and one aspartate) forming the Mn-oxidation site typical of MnPs and VPs.

	Polyporales										Hymenochaetales		
Decay mode	White-rot							Brown-rot			White-rot		
Fungal species	Bje ad	Phl br	Pha ch	Dic sq	Gan sp	Tra ve	Cer su	Fom pi	Pos pl	Wol co	Fom me	Sch pa	Rig mi
MnP-short	6	3	0	5	6	12	1	0	0	0	3	0	3
MnP-long	0	3	5	4	0	0	12	0	0	0	11	12	3
MnP-atypical	0	0	0	0	0	0	0	0	0	0	2	0	0
all MnP	6	6	5	9	6	12	13	0	0	0	16	12	6
LiP	12	5	10	0	0	10	1	0	0	0	0	0	0
VP	1	0	0	3	1	2	1	0	0	0	0	0	0
VP-atypical	0	0	0	0	1	1	0	0	0	0	0	0	0
Ligninolytic peroxidases	19	11	15	12	8	25	15	0	0	0	16	12	6
GP	1	0	1	0	0	0	1	2	1	1	1	4	0
All class II peroxidases	20	11	16	12	8	25	16	2	1	1	17	16	6

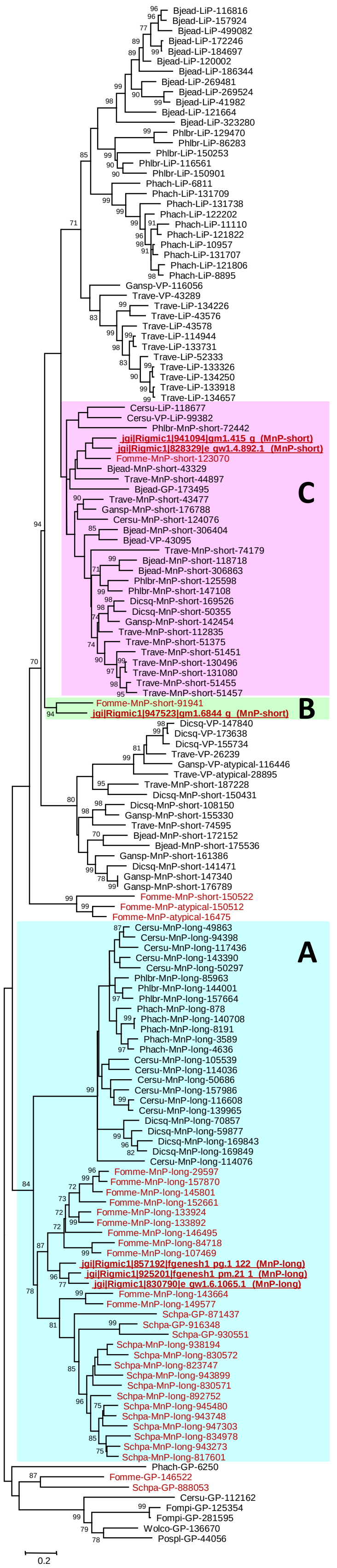

Fig. B. Maximum likelihood phylogenetic tree showing evolutionary relationships among 151 Class-II heme peroxidases from three species of *Hymenochaetales* (shown in red font) and ten species of *Polyporales* (shown in black font) genomes available through Mycocosm, including structural-functional classification based on [10]. MnPs from *R. microporus* are shown in bold, underlined, red font. Numbers on branches represent bootstrap values supporting that branch; only the values ≥70% are presented. Fungal abbreviations are as shown in **Table A1**. Peroxidase abbreviations: i) GP, generic peroxidase; ii) MnP-short and MnP-long, two different manganese peroxidase (MnP) subfamilies including a typical Mn(II)-oxidation site, formed by two glutamates and one aspartate residues, and differing in the length of their C-terminal tails; iii) LiP, lignin peroxidase harboring an exposed tryptophan residue located at the same position described for the catalytic Trp171 of P. chrysosporium LiP; iv) VP, versatile peroxidase including a Mn(II)-oxidation site like in MnP, and a catalytic tryptophan like in LiP; v) MnP-atypical and VP-atypical, MnP and VP lacking one of the three acidic residues forming the Mn(II)-oxidation site described above; and vi) VP-LiP intermediate states, two *Ceriporiopsis subvermispora* peroxidases occupying an intermediate position between typical LiPs and VPs according to their structural and catalytic properties [9]. All enzymes are shown with their JGI identification number.

Multicopper oxidases

A total of seven multicopper oxidases (MCO) were identified found in *R. microporus* ED310 v1.0 genome. After multiple alignment with already know MCO proteins to search for conserved motifs and residues, the sequences were manually curated. In particular, 921691 sequence was curated to correct the intron/exon positions in two key regions of the protein. Then, a phylogenetic analysis of the seven *R. microporus* MCO protein sequences was performed with several basidiomycete laccase sequences with known crystal structure and several fungal ferroxidases (most of them from the order *Polyporales*). The tree was constructed by using the maximum likelihood method (Fig. C).

It was concluded that two out of the seven MCO sequences corresponded to ferroxidases (889958, 900905) similar to other fungal Fet3 proteins, while the other five sequences were laccases (921691, 942192, 942193, 942194, 406895).

Fig. C. Maximum likelihood tree for MCO proteins from *R. microporus* showing two separate clusters, one corresponding to laccases and the other to ferroxidases, respectively.

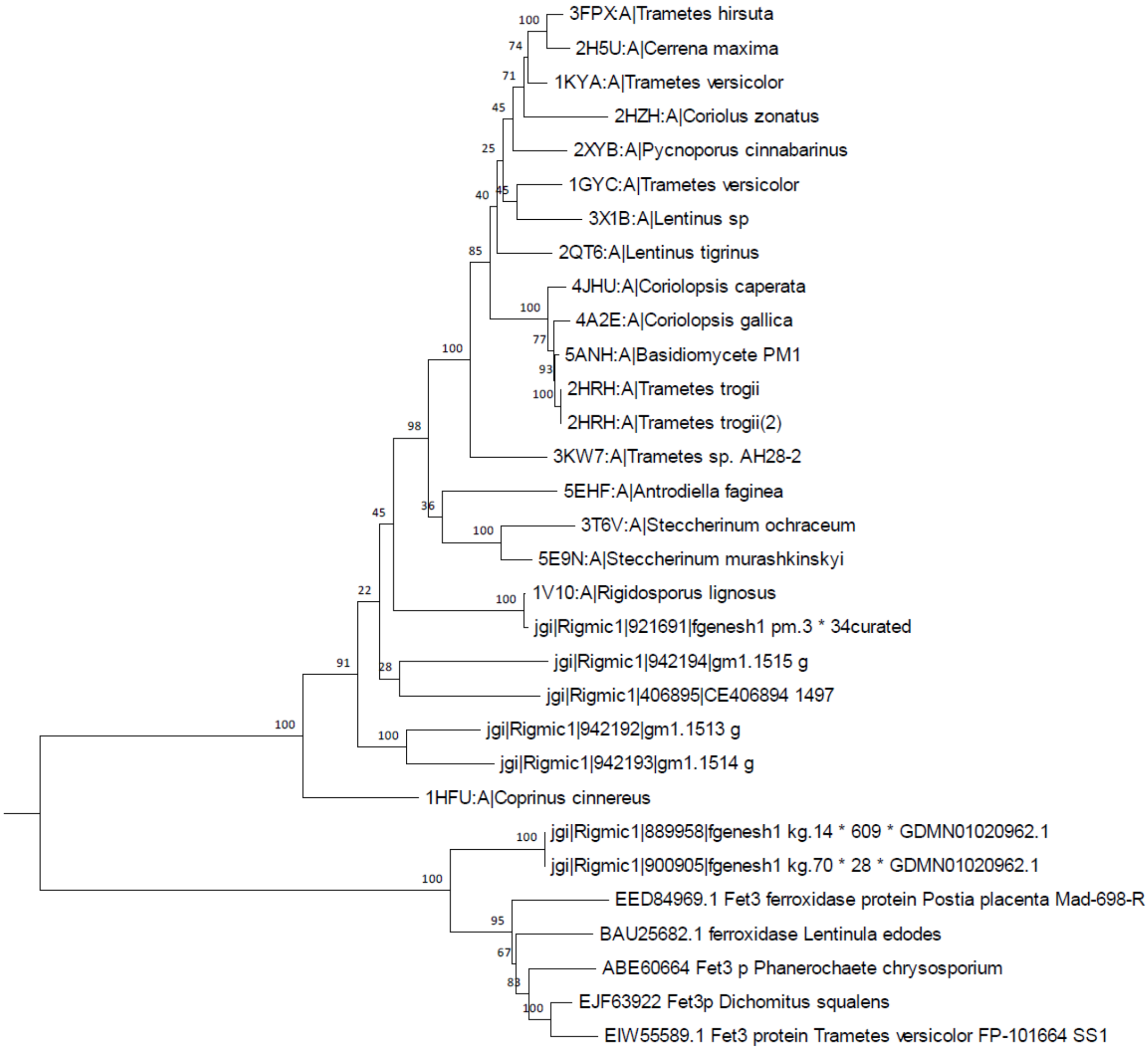

GMC oxidoreductase

The GMC oxidoreductase genes identified in *R. microporus* ED310 genome are summarized in Table A2. A phylogenetic tree was also prepared for the five GMC oxidoreductases from *R. microporus* and 94 enzymes identified in other 10 additional fungal genomes (Fig. D).

Table A2. GMC oxidoreductases in *Rigidoporus microporus* ED310 genome.

		Alignment values		
**Query (GeneBank sequence)**	**Protein ID**	**Score**	**E value**	**% Identity**
**Glucose oxidase**				
ABN79922 *Penicillium expansum*		No hits found		
AAD01493 *Penicillium amagasakiense*		No hits found		
CAD88590 *Botryotinia fuckeliana*		No hits found		
AAF59929 *Aspergillus niger*		No hits found		
EAS27606 *Coccidioides immitis*		No hits found		
JGI131961 *Phanerochaete chrysosporium*[1]	**889492**	1319	$2.14e^{-143}$	50.7
	**889470**	1164	$1.39e^{-122}$	57.4
	**922063**	1052	$2.18e^{-107}$	51.9
**Pyranose-2-oxidase**				
ACJ54278 *Gloeophyllum trabeum*		No hits found		
BAD12079 *Lyophyllum shimeji*				
AAS93628 *Phanerochaete chrysosporium*				
AAO13382 *Peniophora* sp.				
**Pyranose dehydrogenase**				
AAW92124 *Agaricus bisporus*		No significant hits found		
AAW92123 *Agaricus xanthodermus*				
AAW82997 *Leucoagaricus meleagris*				
**Aryl-alcohol oxidase**				
AAC72747 *Pleurotus eryngii*	**883086**	1182	$1.64e^{-120}$	45.9
	**923690**	1164	$8.46e^{-114}$	47.6
	**861392**	1193	$5.98e^{-103}$	46.5
**Methanol oxidase**				
ABI14440 *Gloephyllum trabeum*	**946043**	2833	0.0	82.8
AAF141329 *Pichia methanolica*	946043	1801	0.0	55.3
Q00922 *Candida boidinii*	946043	1745	0.0	52.8
AAV66467 *Candida boidinii*	946043	1646	0.0	51.2
**Cellobiose dehydrogenase**				
AAC50004 *Trametes versicolor*	**925463**	2014	0.0	56.8
CAA61359 *Phanerochaete chrysosporium*	925463	2011	0.0	53.8
ACF60617 *Gelatoporia subvermispora*	925463	1965	0.0	55.0
BAD32781 *Coniophora puteana*	925463	1903	0.0	57.2
AAC32197 *Pycnoporus cinnabarinus*	925463	1867	0.0	56.2

[1]JGI ID protein 131961 corresponds to a previous version of *Phanerochaete chrysosporium* genome. In the actual version of *Phanerochaete chrysosporium genome* (v2.2) this protein has JGI ID protein 2910568.

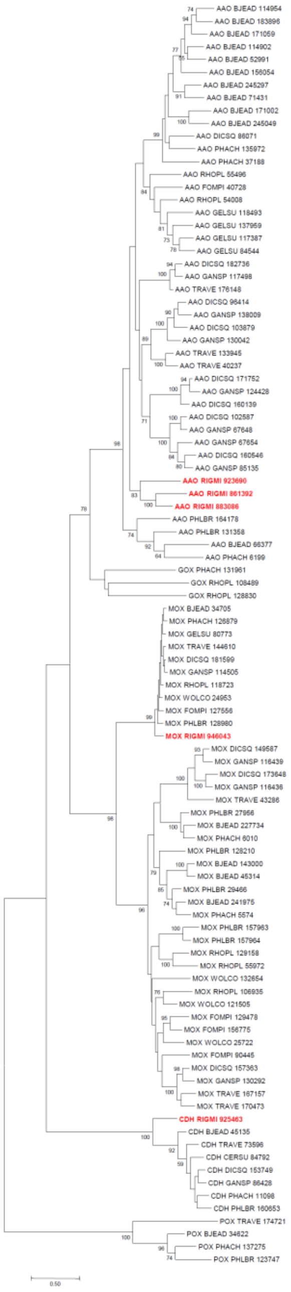

Fig. D. Phylogenetic tree showing evolutionary relationships among 99 GMC oxidoreductases identified in 11 fungal genomes (GMCs from *R. microporus* are shown in red letters). Numbers on branches represent bootstrap values supporting that branch; only the values ≥70% are presented. Fungal abbreviations are the same as in Fig. B. All enzymes are shown with their JGI identification number.

Supplementary Figure 1. Scheme illustrating the preparation of plates overlaid with latex-containing medium and control plates (without latex overlay).

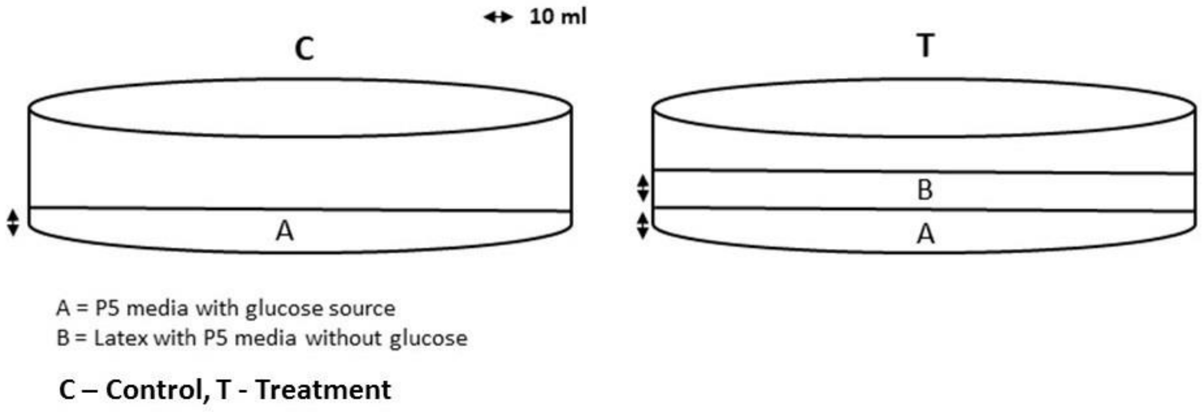

Supplementary Figure 2. Growth of *R. microporus* on latex-overlaid and control plates. (A) Mycelial growth after 6 days post inoculation (B) *R. microporus* grown on rubber wood dust and on latex (these samples were selected for RNA extraction and sequencing) (C) Colony size of *R. microporus* in mm after 6 days post inoculation.

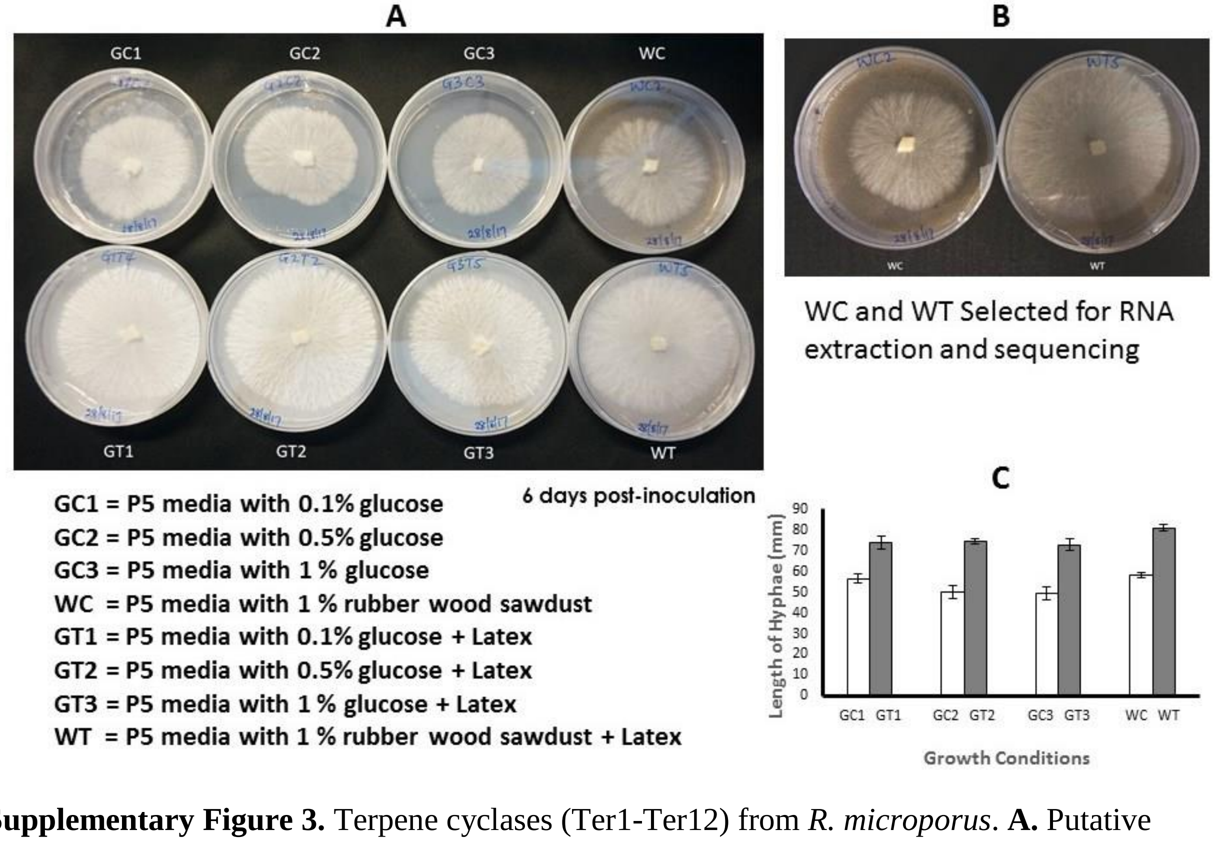

Supplementary Figure 3. Terpene cyclases (Ter1-Ter12) from *R. microporus*. **A.** Putative terpene cyclases with their active domains (aspartate rich domains). Two large gene clusters span the TC genes *ter3A*, *ter3B* and *ter3C* or *ter6A*, *ter6B* and *ter6C*, respectively. **B.** Active sites from TCs shown in Supplementary Fig. 3A. Amino acids with a majority of 25% are highlighted

in colors. The position of the active sites in the polypeptide chain is indicated on the left site of the panel. Note that the consensus motif DDX$_{3\text{-}4}$CD indicates N-terminal active sites in *R. microporus*. In Ter12, the DD motif is replaced by ED.

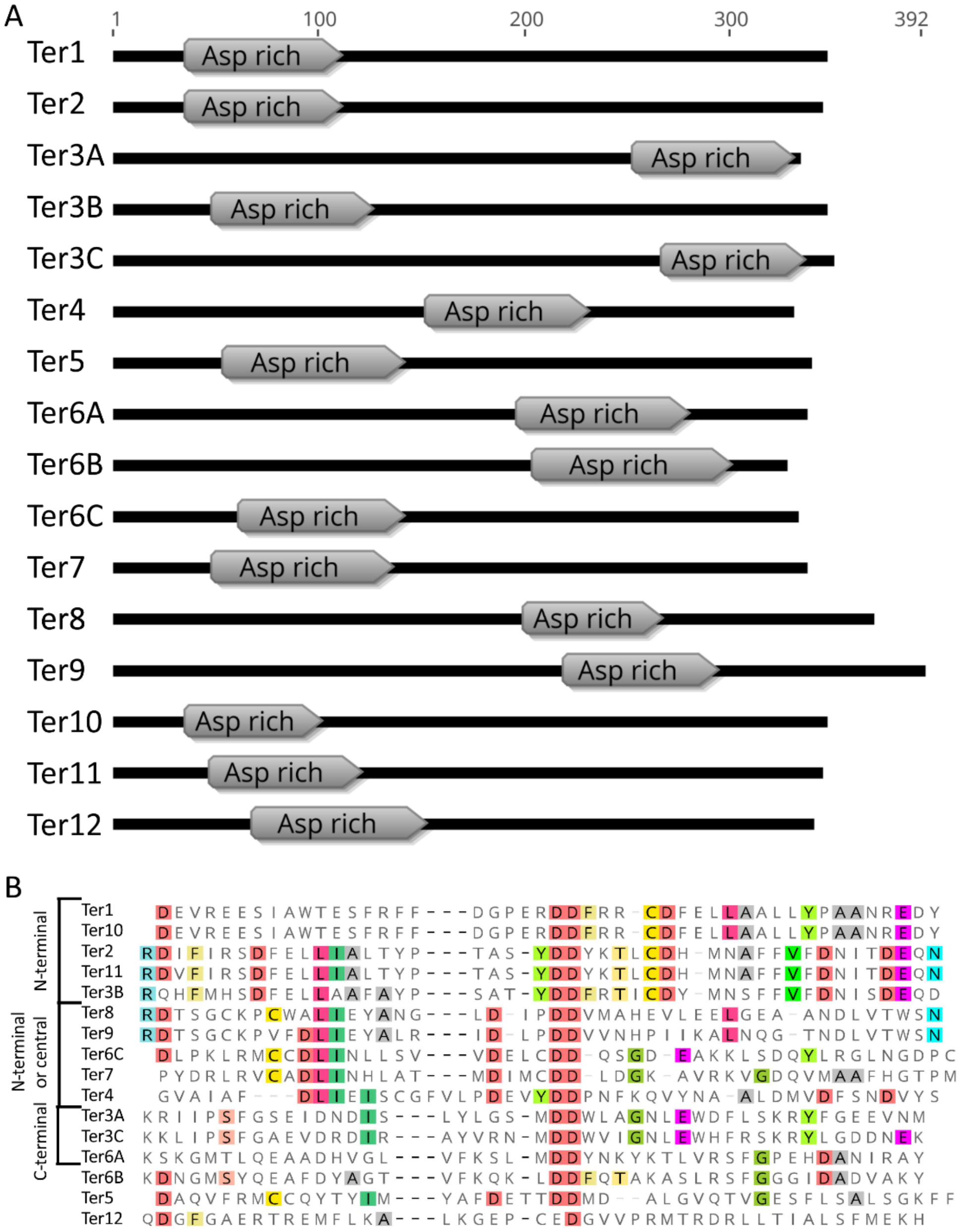

Supplementary Figure 4. Phylogenetic reconstructions illustrating the origin of *R. microporus* genes predicted to originate from HGT events. (A) locus 983304; (B) locus 975179; (C) locus 941964; (D) locus 882912; (E) locus 871220; (F) locus 870991; (G) locus 1012245; (H) locus 948311; (I) locus 897619

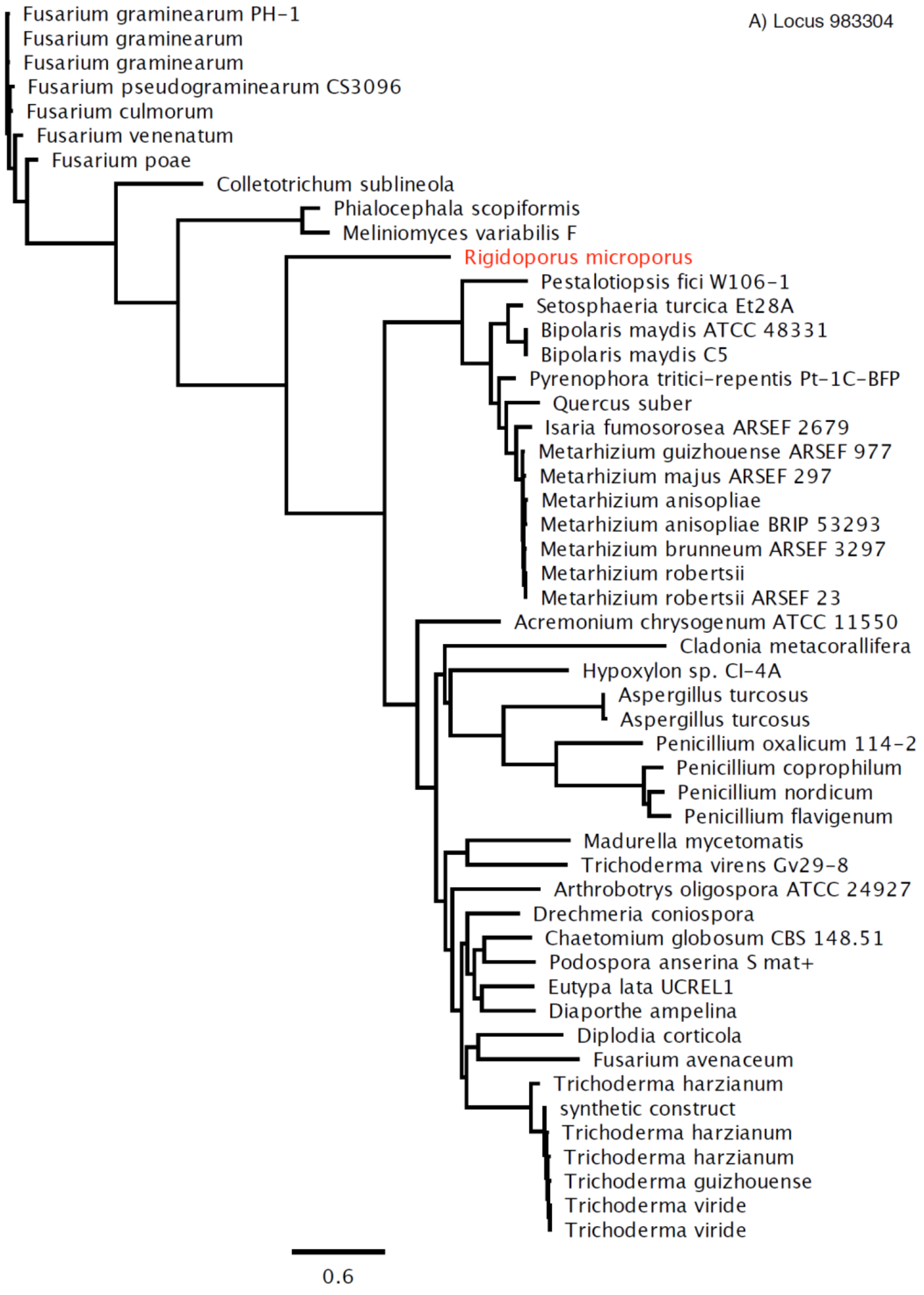
A) Locus 983304
Fusarium graminearum PH-1
Fusarium graminearum
Fusarium graminearum
Fusarium pseudograminearum CS3096
Fusarium culmorum
Fusarium venenatum
Fusarium poae
Colletotrichum sublineola
Phialocephala scopiformis
Meliniomyces variabilis F
Rigidoporus microporus
Pestalotiopsis fici W106-1
Setosphaeria turcica Et28A
Bipolaris maydis ATCC 48331
Bipolaris maydis C5
Pyrenophora tritici-repentis Pt-1C-BFP
Quercus suber
Isaria fumosorosea ARSEF 2679
Metarhizium guizhouense ARSEF 977
Metarhizium majus ARSEF 297
Metarhizium anisopliae
Metarhizium anisopliae BRIP 53293
Metarhizium brunneum ARSEF 3297
Metarhizium robertsii
Metarhizium robertsii ARSEF 23
Acremonium chrysogenum ATCC 11550
Cladonia metacorallifera
Hypoxylon sp. CI-4A
Aspergillus turcosus
Aspergillus turcosus
Penicillium oxalicum 114-2
Penicillium coprophilum
Penicillium nordicum
Penicillium flavigenum
Madurella mycetomatis
Trichoderma virens Gv29-8
Arthrobotrys oligospora ATCC 24927
Drechmeria coniospora
Chaetomium globosum CBS 148.51
Podospora anserina S mat+
Eutypa lata UCREL1
Diaporthe ampelina
Diplodia corticola
Fusarium avenaceum
Trichoderma harzianum
synthetic construct
Trichoderma harzianum
Trichoderma harzianum
Trichoderma guizhouense
Trichoderma viride
Trichoderma viride
0.6

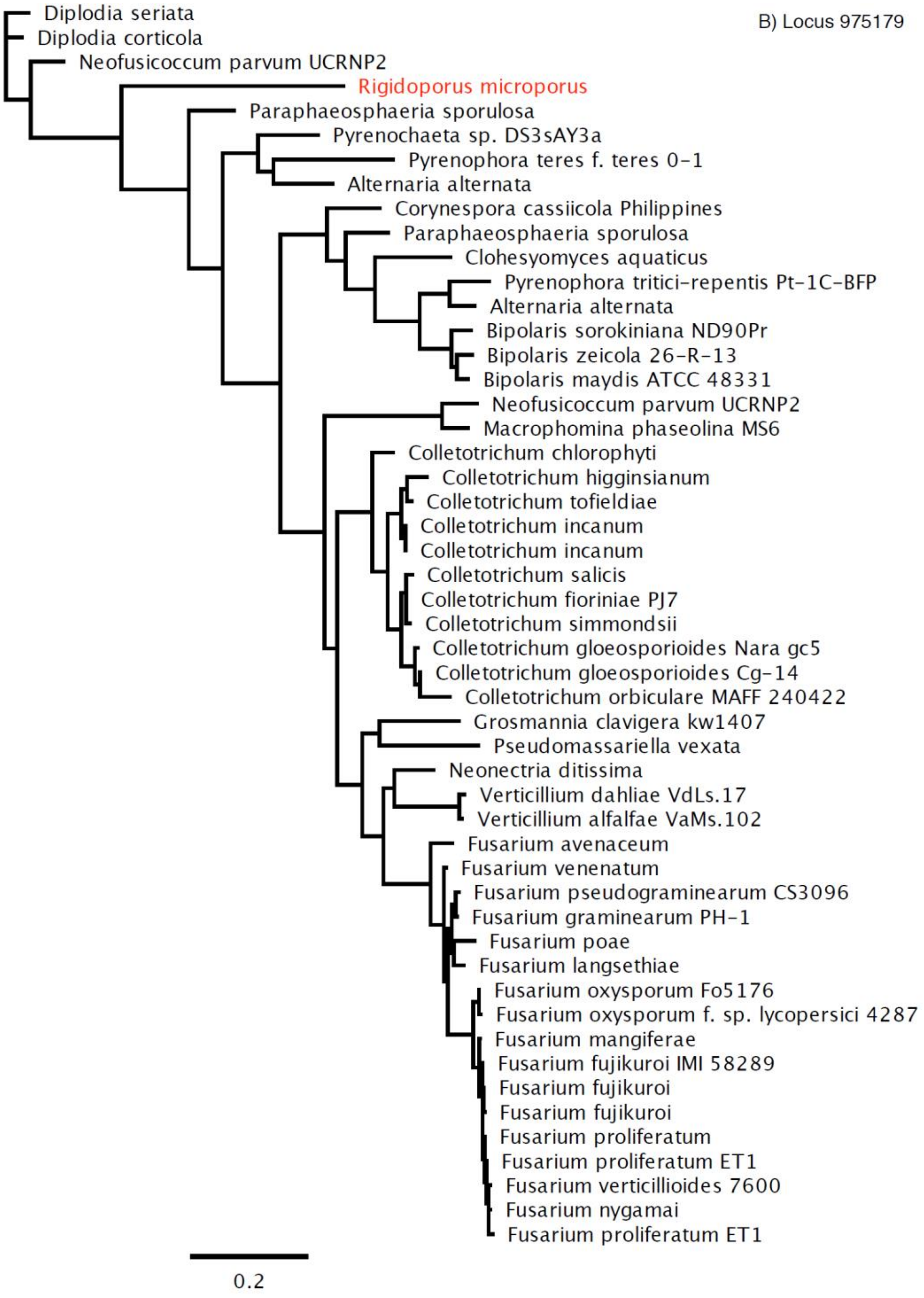
B) Locus 975179
Diplodia seriata
Diplodia corticola
Neofusicoccum parvum UCRNP2
Rigidoporus microporus
Paraphaeosphaeria sporulosa
Pyrenochaeta sp. DS3sAY3a
Pyrenophora teres f. teres 0-1
Alternaria alternata
Corynespora cassiicola Philippines
Paraphaeosphaeria sporulosa
Clohesyomyces aquaticus
Pyrenophora tritici-repentis Pt-1C-BFP
Alternaria alternata
Bipolaris sorokiniana ND90Pr
Bipolaris zeicola 26-R-13
Bipolaris maydis ATCC 48331
Neofusicoccum parvum UCRNP2
Macrophomina phaseolina MS6
Colletotrichum chlorophyti
Colletotrichum higginsianum
Colletotrichum tofieldiae
Colletotrichum incanum
Colletotrichum incanum
Colletotrichum salicis
Colletotrichum fioriniae PJ7
Colletotrichum simmondsii
Colletotrichum gloeosporioides Nara gc5
Colletotrichum gloeosporioides Cg-14
Colletotrichum orbiculare MAFF 240422
Grosmannia clavigera kw1407
Pseudomassariella vexata
Neonectria ditissima
Verticillium dahliae VdLs.17
Verticillium alfalfae VaMs.102
Fusarium avenaceum
Fusarium venenatum
Fusarium pseudograminearum CS3096
Fusarium graminearum PH-1
Fusarium poae
Fusarium langsethiae
Fusarium oxysporum Fo5176
Fusarium oxysporum f. sp. lycopersici 4287
Fusarium mangiferae
Fusarium fujikuroi IMI 58289
Fusarium fujikuroi
Fusarium fujikuroi
Fusarium proliferatum
Fusarium proliferatum ET1
Fusarium verticillioides 7600
Fusarium nygamai
Fusarium proliferatum ET1
0.2

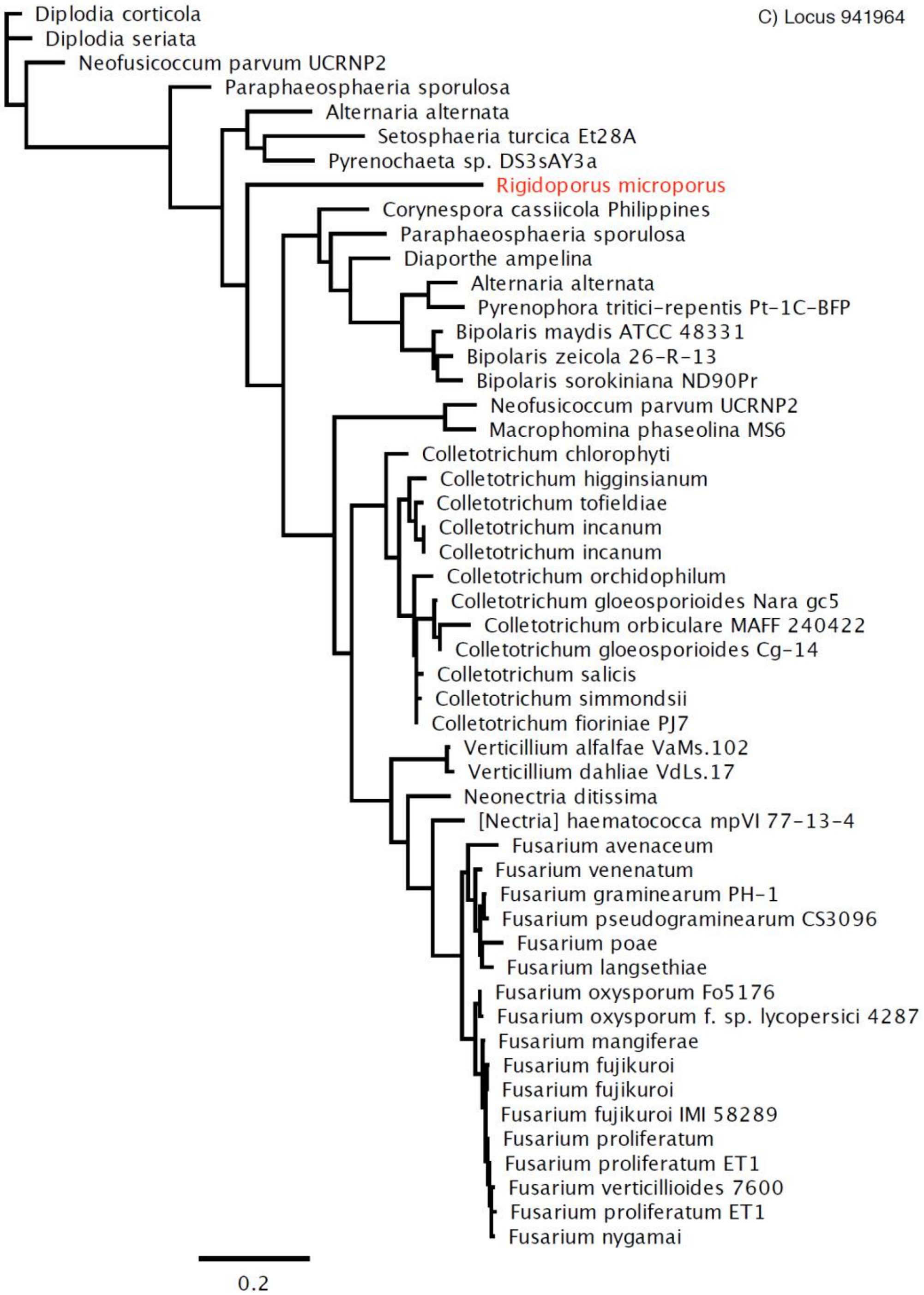
C) Locus 941964
Diplodia corticola
Diplodia seriata
Neofusicoccum parvum UCRNP2
Paraphaeosphaeria sporulosa
Alternaria alternata
Setosphaeria turcica Et28A
Pyrenochaeta sp. DS3sAY3a
Rigidoporus microporus
Corynespora cassiicola Philippines
Paraphaeosphaeria sporulosa
Diaporthe ampelina
Alternaria alternata
Pyrenophora tritici-repentis Pt-1C-BFP
Bipolaris maydis ATCC 48331
Bipolaris zeicola 26-R-13
Bipolaris sorokiniana ND90Pr
Neofusicoccum parvum UCRNP2
Macrophomina phaseolina MS6
Colletotrichum chlorophyti
Colletotrichum higginsianum
Colletotrichum tofieldiae
Colletotrichum incanum
Colletotrichum incanum
Colletotrichum orchidophilum
Colletotrichum gloeosporioides Nara gc5
Colletotrichum orbiculare MAFF 240422
Colletotrichum gloeosporioides Cg-14
Colletotrichum salicis
Colletotrichum simmondsii
Colletotrichum fioriniae PJ7
Verticillium alfalfae VaMs.102
Verticillium dahliae VdLs.17
Neonectria ditissima
[Nectria] haematococca mpVI 77-13-4
Fusarium avenaceum
Fusarium venenatum
Fusarium graminearum PH-1
Fusarium pseudograminearum CS3096
Fusarium poae
Fusarium langsethiae
Fusarium oxysporum Fo5176
Fusarium oxysporum f. sp. lycopersici 4287
Fusarium mangiferae
Fusarium fujikuroi
Fusarium fujikuroi
Fusarium fujikuroi IMI 58289
Fusarium proliferatum
Fusarium proliferatum ET1
Fusarium verticillioides 7600
Fusarium proliferatum ET1
Fusarium nygamai
0.2

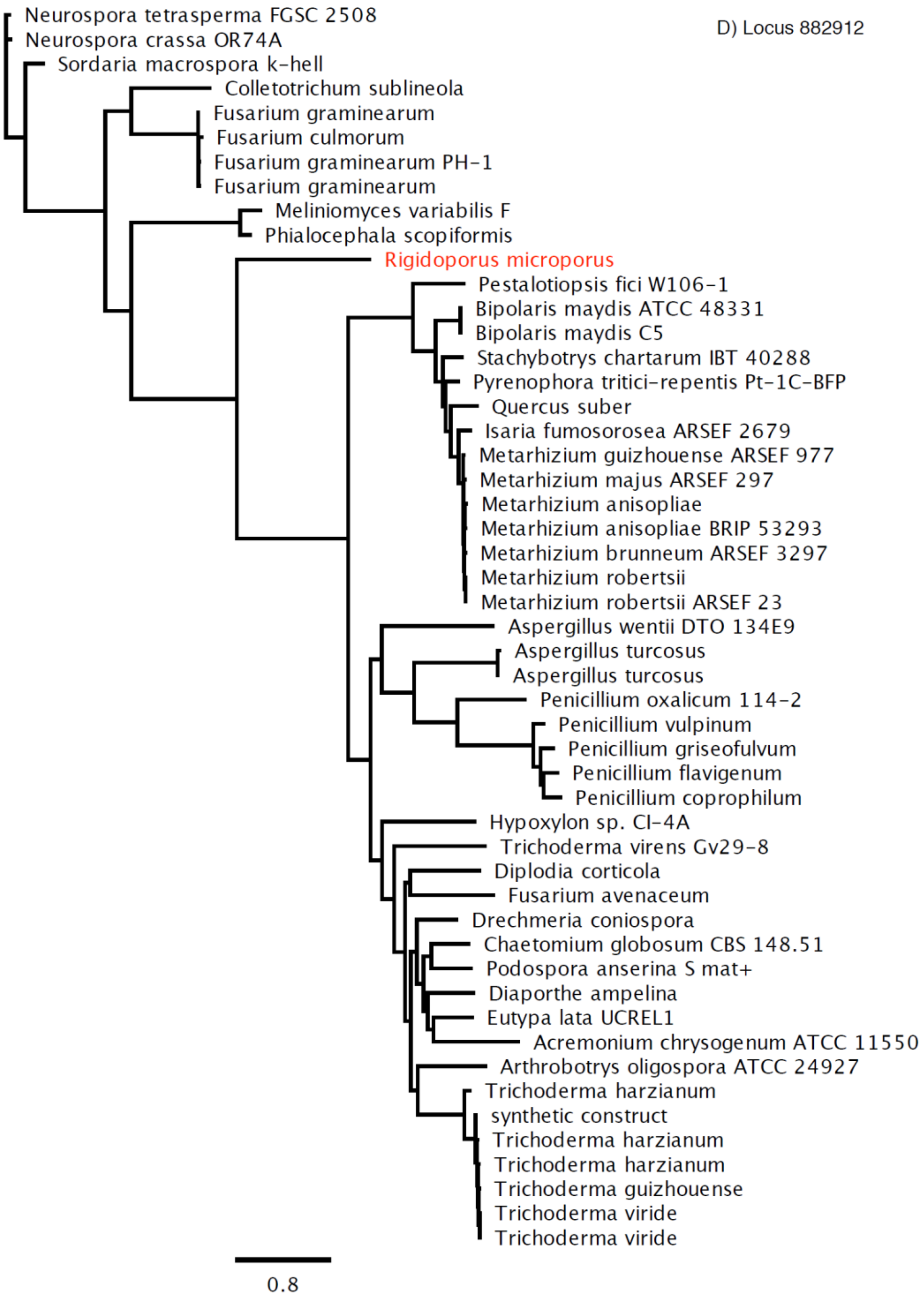
D) Locus 882912
Neurospora tetrasperma FGSC 2508
Neurospora crassa OR74A
Sordaria macrospora k-hell
Colletotrichum sublineola
Fusarium graminearum
Fusarium culmorum
Fusarium graminearum PH-1
Fusarium graminearum
Meliniomyces variabilis F
Phialocephala scopiformis
Rigidoporus microporus
Pestalotiopsis fici W106-1
Bipolaris maydis ATCC 48331
Bipolaris maydis C5
Stachybotrys chartarum IBT 40288
Pyrenophora tritici-repentis Pt-1C-BFP
Quercus suber
Isaria fumosorosea ARSEF 2679
Metarhizium guizhouense ARSEF 977
Metarhizium majus ARSEF 297
Metarhizium anisopliae
Metarhizium anisopliae BRIP 53293
Metarhizium brunneum ARSEF 3297
Metarhizium robertsii
Metarhizium robertsii ARSEF 23
Aspergillus wentii DTO 134E9
Aspergillus turcosus
Aspergillus turcosus
Penicillium oxalicum 114-2
Penicillium vulpinum
Penicillium griseofulvum
Penicillium flavigenum
Penicillium coprophilum
Hypoxylon sp. CI-4A
Trichoderma virens Gv29-8
Diplodia corticola
Fusarium avenaceum
Drechmeria coniospora
Chaetomium globosum CBS 148.51
Podospora anserina S mat+
Diaporthe ampelina
Eutypa lata UCREL1
Acremonium chrysogenum ATCC 11550
Arthrobotrys oligospora ATCC 24927
Trichoderma harzianum
synthetic construct
Trichoderma harzianum
Trichoderma harzianum
Trichoderma guizhouense
Trichoderma viride
Trichoderma viride
0.8

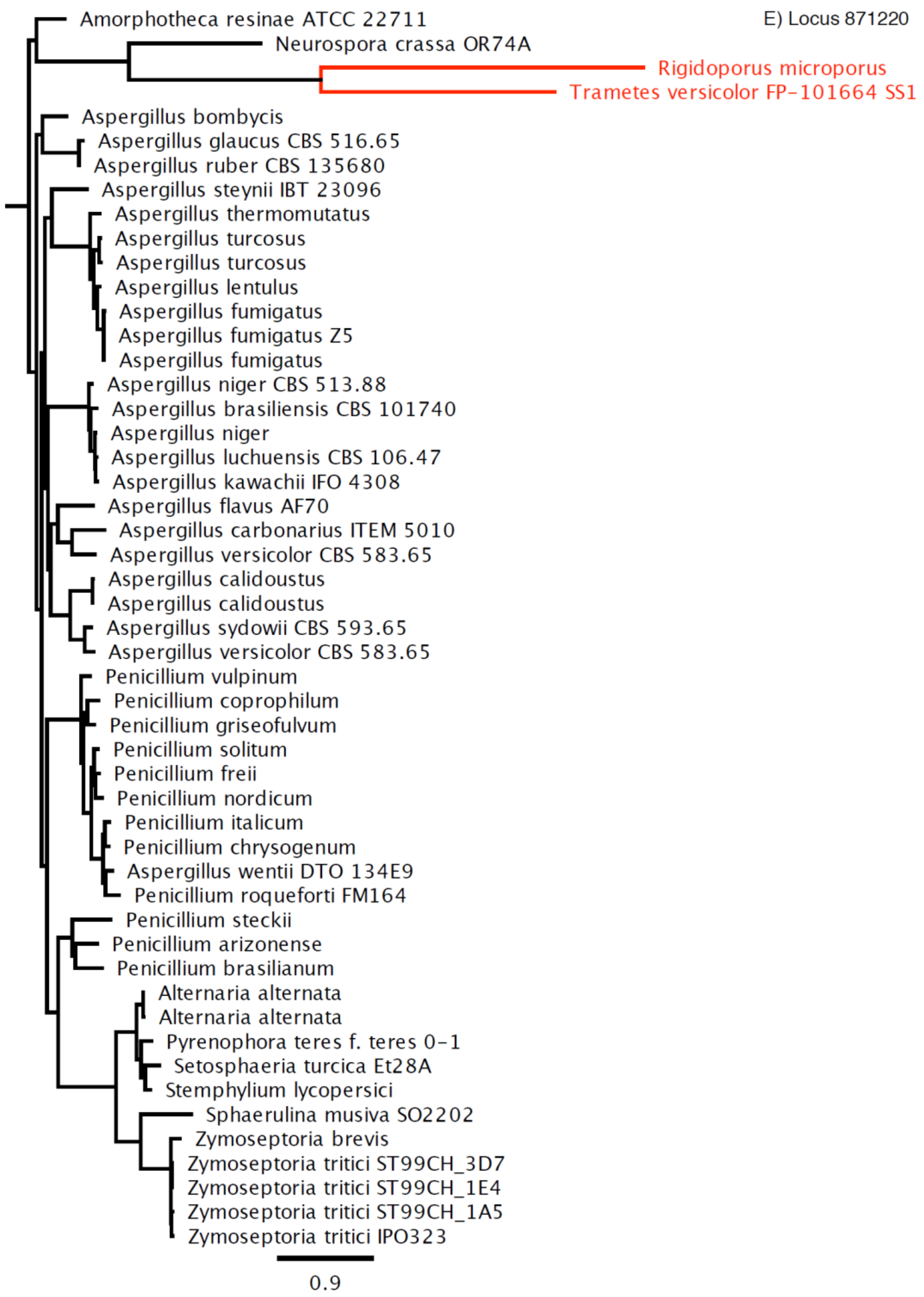
E) Locus 871220
Amorphotheca resinae ATCC 22711
Neurospora crassa OR74A
Rigidoporus microporus
Trametes versicolor FP-101664 SS1
Aspergillus bombycis
Aspergillus glaucus CBS 516.65
Aspergillus ruber CBS 135680
Aspergillus steynii IBT 23096
Aspergillus thermomutatus
Aspergillus turcosus
Aspergillus turcosus
Aspergillus lentulus
Aspergillus fumigatus
Aspergillus fumigatus Z5
Aspergillus fumigatus
Aspergillus niger CBS 513.88
Aspergillus brasiliensis CBS 101740
Aspergillus niger
Aspergillus luchuensis CBS 106.47
Aspergillus kawachii IFO 4308
Aspergillus flavus AF70
Aspergillus carbonarius ITEM 5010
Aspergillus versicolor CBS 583.65
Aspergillus calidoustus
Aspergillus calidoustus
Aspergillus sydowii CBS 593.65
Aspergillus versicolor CBS 583.65
Penicillium vulpinum
Penicillium coprophilum
Penicillium griseofulvum
Penicillium solitum
Penicillium freii
Penicillium nordicum
Penicillium italicum
Penicillium chrysogenum
Aspergillus wentii DTO 134E9
Penicillium roqueforti FM164
Penicillium steckii
Penicillium arizonense
Penicillium brasilianum
Alternaria alternata
Alternaria alternata
Pyrenophora teres f. teres 0-1
Setosphaeria turcica Et28A
Stemphylium lycopersici
Sphaerulina musiva SO2202
Zymoseptoria brevis
Zymoseptoria tritici ST99CH_3D7
Zymoseptoria tritici ST99CH_1E4
Zymoseptoria tritici ST99CH_1A5
Zymoseptoria tritici IPO323
0.9

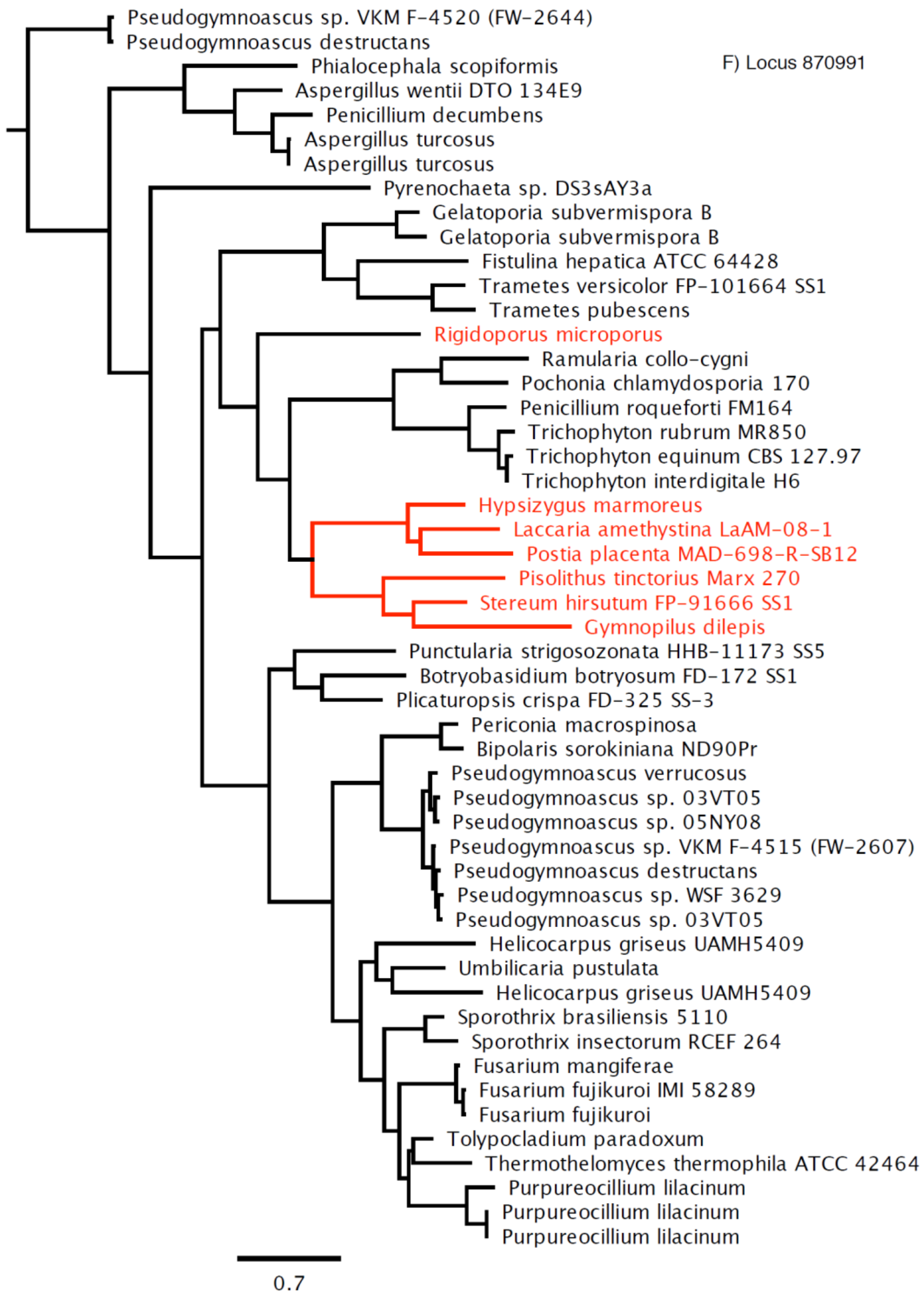
F) Locus 870991
Pseudogymnoascus sp. VKM F-4520 (FW-2644)
Pseudogymnoascus destructans
Phialocephala scopiformis
Aspergillus wentii DTO 134E9
Penicillium decumbens
Aspergillus turcosus
Aspergillus turcosus
Pyrenochaeta sp. DS3sAY3a
Gelatoporia subvermispora B
Gelatoporia subvermispora B
Fistulina hepatica ATCC 64428
Trametes versicolor FP-101664 SS1
Trametes pubescens
Rigidoporus microporus
Ramularia collo-cygni
Pochonia chlamydosporia 170
Penicillium roqueforti FM164
Trichophyton rubrum MR850
Trichophyton equinum CBS 127.97
Trichophyton interdigitale H6
Hypsizygus marmoreus
Laccaria amethystina LaAM-08-1
Postia placenta MAD-698-R-SB12
Pisolithus tinctorius Marx 270
Stereum hirsutum FP-91666 SS1
Gymnopilus dilepis
Punctularia strigosozonata HHB-11173 SS5
Botryobasidium botryosum FD-172 SS1
Plicaturopsis crispa FD-325 SS-3
Periconia macrospinosa
Bipolaris sorokiniana ND90Pr
Pseudogymnoascus verrucosus
Pseudogymnoascus sp. 03VT05
Pseudogymnoascus sp. 05NY08
Pseudogymnoascus sp. VKM F-4515 (FW-2607)
Pseudogymnoascus destructans
Pseudogymnoascus sp. WSF 3629
Pseudogymnoascus sp. 03VT05
Helicocarpus griseus UAMH5409
Umbilicaria pustulata
Helicocarpus griseus UAMH5409
Sporothrix brasiliensis 5110
Sporothrix insectorum RCEF 264
Fusarium mangiferae
Fusarium fujikuroi IMI 58289
Fusarium fujikuroi
Tolypocladium paradoxum
Thermothelomyces thermophila ATCC 42464
Purpureocillium lilacinum
Purpureocillium lilacinum
Purpureocillium lilacinum
0.7

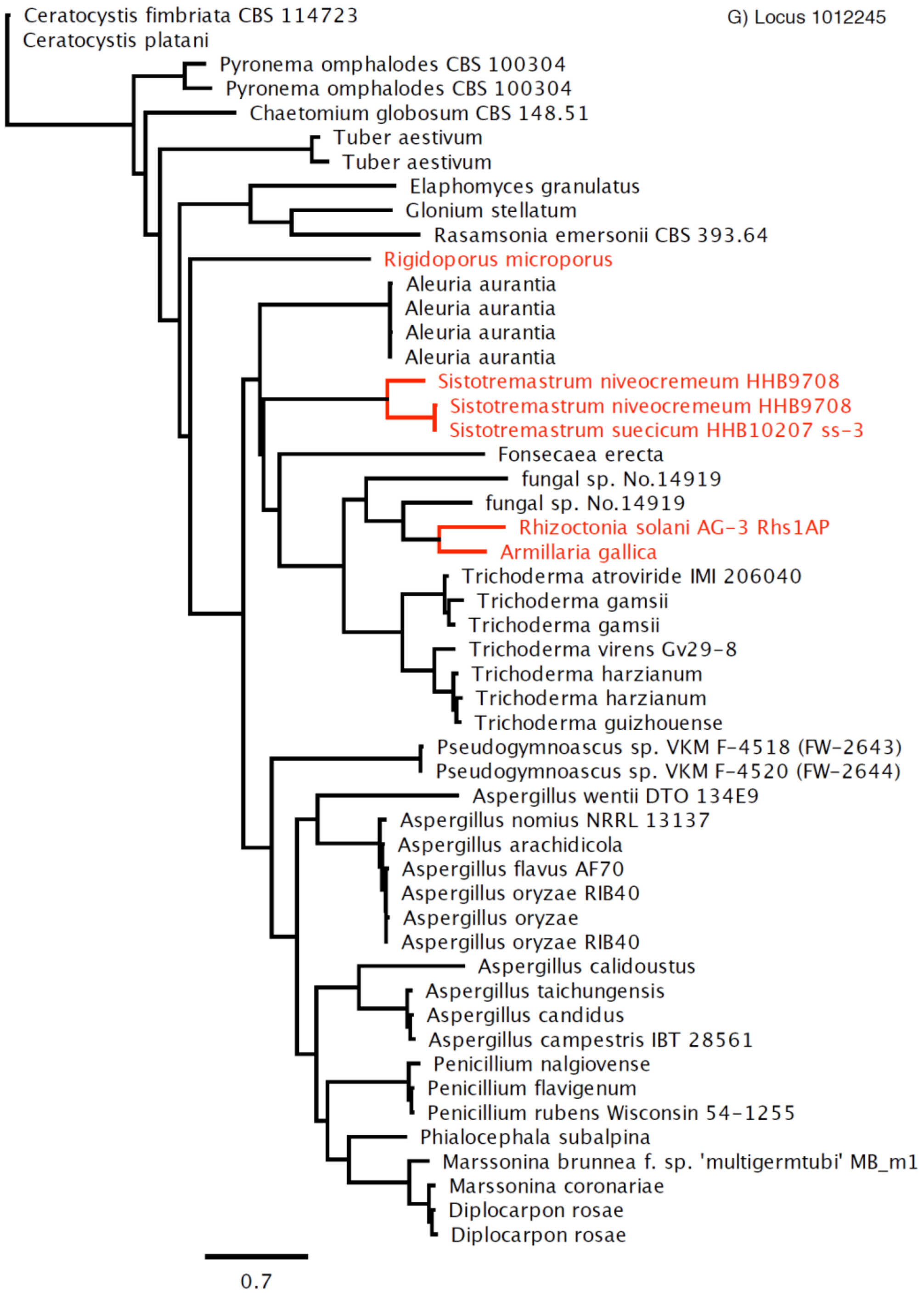
G) Locus 1012245
Ceratocystis fimbriata CBS 114723
Ceratocystis platani
Pyronema omphalodes CBS 100304
Pyronema omphalodes CBS 100304
Chaetomium globosum CBS 148.51
Tuber aestivum
Tuber aestivum
Elaphomyces granulatus
Glonium stellatum
Rasamsonia emersonii CBS 393.64
Rigidoporus microporus
Aleuria aurantia
Aleuria aurantia
Aleuria aurantia
Aleuria aurantia
Sistotremastrum niveocremeum HHB9708
Sistotremastrum niveocremeum HHB9708
Sistotremastrum suecicum HHB10207 ss-3
Fonsecaea erecta
fungal sp. No.14919
fungal sp. No.14919
Rhizoctonia solani AG-3 Rhs1AP
Armillaria gallica
Trichoderma atroviride IMI 206040
Trichoderma gamsii
Trichoderma gamsii
Trichoderma virens Gv29-8
Trichoderma harzianum
Trichoderma harzianum
Trichoderma guizhouense
Pseudogymnoascus sp. VKM F-4518 (FW-2643)
Pseudogymnoascus sp. VKM F-4520 (FW-2644)
Aspergillus wentii DTO 134E9
Aspergillus nomius NRRL 13137
Aspergillus arachidicola
Aspergillus flavus AF70
Aspergillus oryzae RIB40
Aspergillus oryzae
Aspergillus oryzae RIB40
Aspergillus calidoustus
Aspergillus taichungensis
Aspergillus candidus
Aspergillus campestris IBT 28561
Penicillium nalgiovense
Penicillium flavigenum
Penicillium rubens Wisconsin 54-1255
Phialocephala subalpina
Marssonina brunnea f. sp. 'multigermtubi' MB_m1
Marssonina coronariae
Diplocarpon rosae
Diplocarpon rosae
0.7

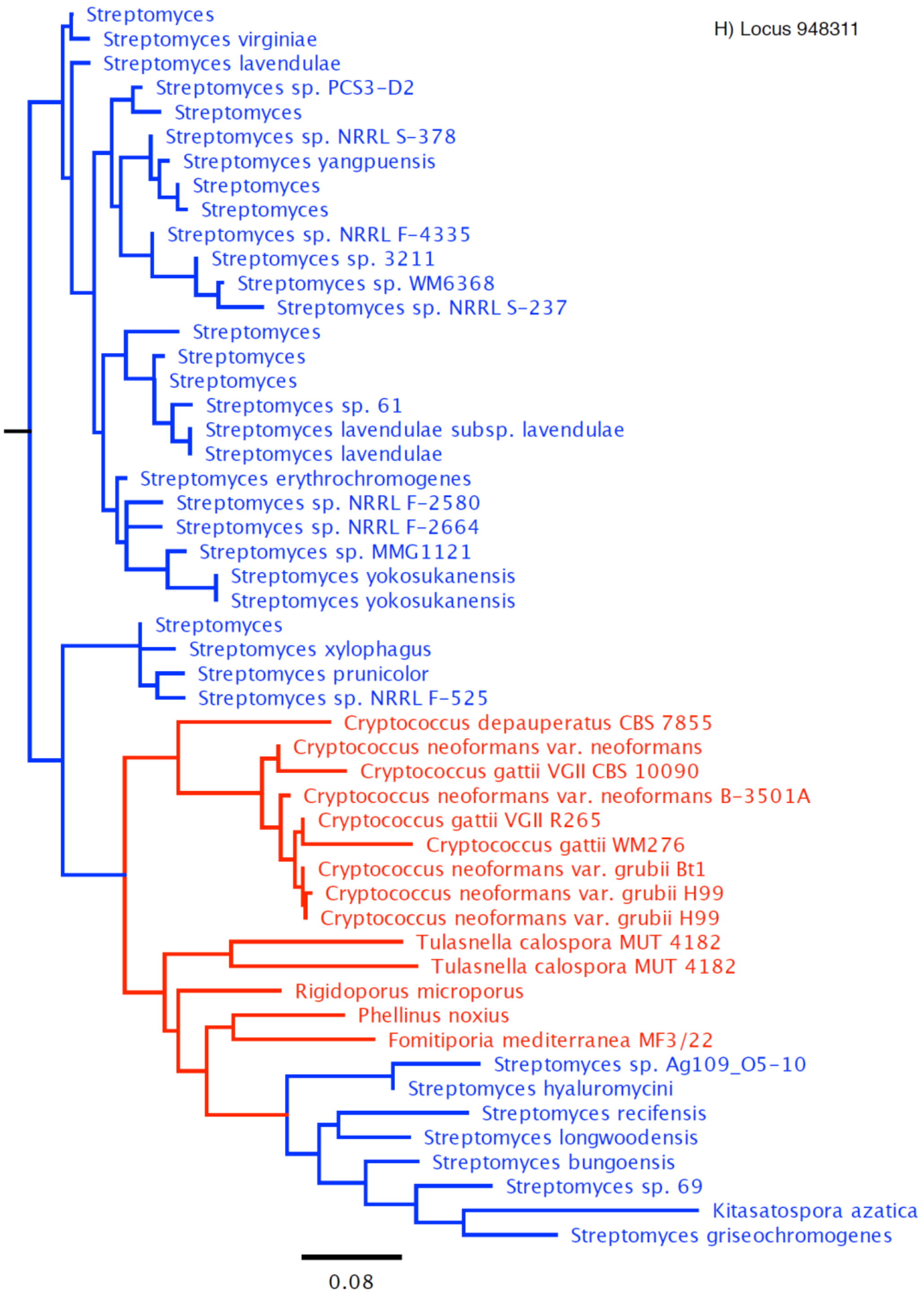

H) Locus 948311
Streptomyces
Streptomyces virginiae
Streptomyces lavendulae
Streptomyces sp. PCS3-D2
Streptomyces
Streptomyces sp. NRRL S-378
Streptomyces yangpuensis
Streptomyces
Streptomyces
Streptomyces sp. NRRL F-4335
Streptomyces sp. 3211
Streptomyces sp. WM6368
Streptomyces sp. NRRL S-237
Streptomyces
Streptomyces
Streptomyces
Streptomyces sp. 61
Streptomyces lavendulae subsp. lavendulae
Streptomyces lavendulae
Streptomyces erythrochromogenes
Streptomyces sp. NRRL F-2580
Streptomyces sp. NRRL F-2664
Streptomyces sp. MMG1121
Streptomyces yokosukanensis
Streptomyces yokosukanensis
Streptomyces
Streptomyces xylophagus
Streptomyces prunicolor
Streptomyces sp. NRRL F-525
Cryptococcus depauperatus CBS 7855
Cryptococcus neoformans var. neoformans
Cryptococcus gattii VGII CBS 10090
Cryptococcus neoformans var. neoformans B-3501A
Cryptococcus gattii VGII R265
Cryptococcus gattii WM276
Cryptococcus neoformans var. grubii Bt1
Cryptococcus neoformans var. grubii H99
Cryptococcus neoformans var. grubii H99
Tulasnella calospora MUT 4182
Tulasnella calospora MUT 4182
Rigidoporus microporus
Phellinus noxius
Fomitiporia mediterranea MF3/22
Streptomyces sp. Ag109_O5-10
Streptomyces hyaluromycini
Streptomyces recifensis
Streptomyces longwoodensis
Streptomyces bungoensis
Streptomyces sp. 69
Kitasatospora azatica
Streptomyces griseochromogenes
0.08

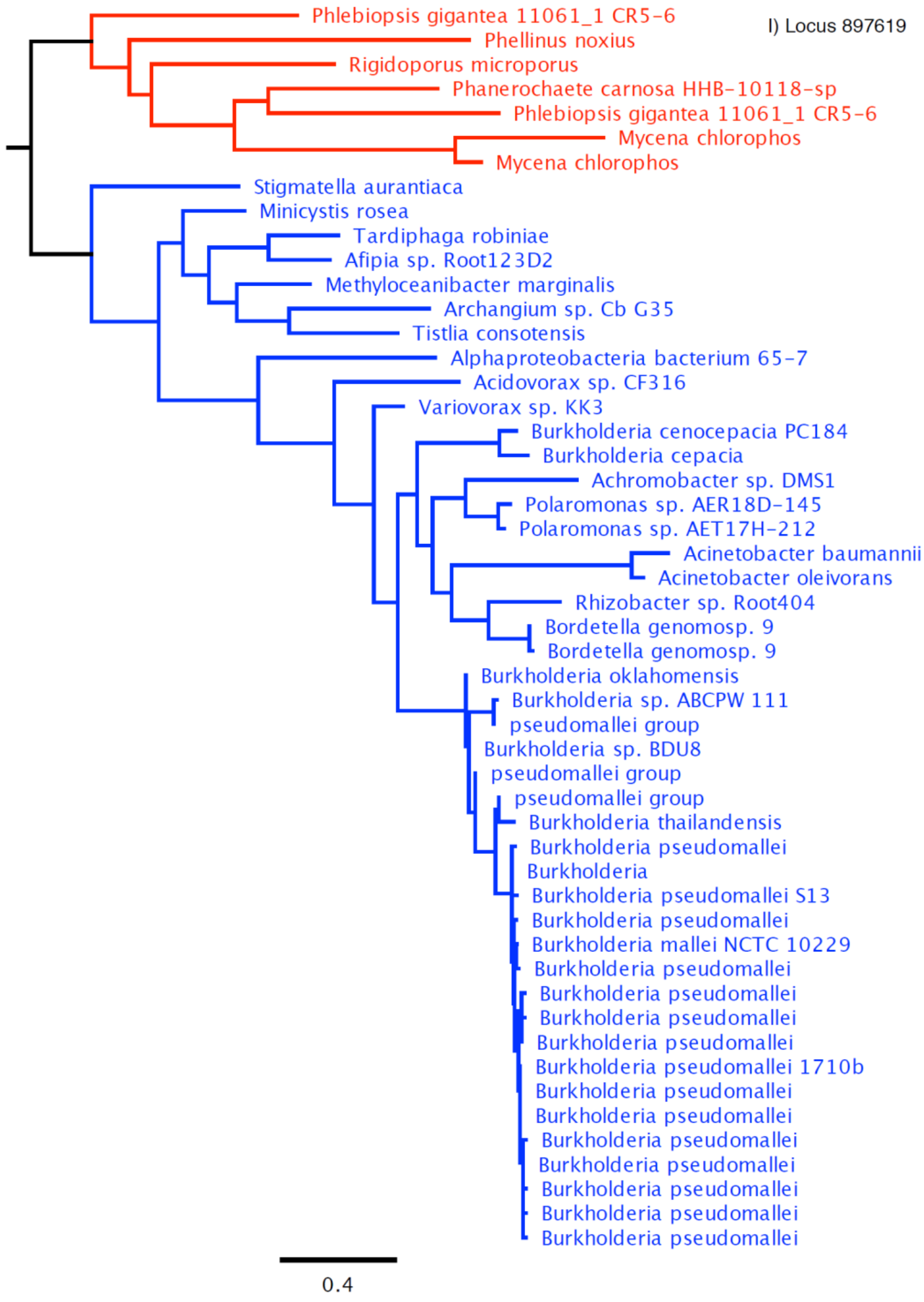

l) Locus 897619
Phlebiopsis gigantea 11061_1 CR5-6
Phellinus noxius
Rigidoporus microporus
Phanerochaete carnosa HHB-10118-sp
Phlebiopsis gigantea 11061_1 CR5-6
Mycena chlorophos
Mycena chlorophos
Stigmatella aurantiaca
Minicystis rosea
Tardiphaga robiniae
Afipia sp. Root123D2
Methyloceanibacter marginalis
Archangium sp. Cb G35
Tistlia consotensis
Alphaproteobacteria bacterium 65-7
Acidovorax sp. CF316
Variovorax sp. KK3
Burkholderia cenocepacia PC184
Burkholderia cepacia
Achromobacter sp. DMS1
Polaromonas sp. AER18D-145
Polaromonas sp. AET17H-212
Acinetobacter baumannii
Acinetobacter oleivorans
Rhizobacter sp. Root404
Bordetella genomosp. 9
Bordetella genomosp. 9
Burkholderia oklahomensis
Burkholderia sp. ABCPW 111
pseudomallei group
Burkholderia sp. BDU8
pseudomallei group
pseudomallei group
Burkholderia thailandensis
Burkholderia pseudomallei
Burkholderia
Burkholderia pseudomallei S13
Burkholderia pseudomallei
Burkholderia mallei NCTC 10229
Burkholderia pseudomallei
Burkholderia pseudomallei
Burkholderia pseudomallei
Burkholderia pseudomallei
Burkholderia pseudomallei 1710b
Burkholderia pseudomallei
Burkholderia pseudomallei
Burkholderia pseudomallei
Burkholderia pseudomallei
Burkholderia pseudomallei
Burkholderia pseudomallei
Burkholderia pseudomallei
0.4

Supplementary Figure 5. Development of fungal co-cultures of *R. microporus* with *Phanerochaete chrysoporium*, *Phlebiopsis gigantea* and *Mycena* sp. Time points of sample harvesting fro qRT-PCR analysis are illustrated.

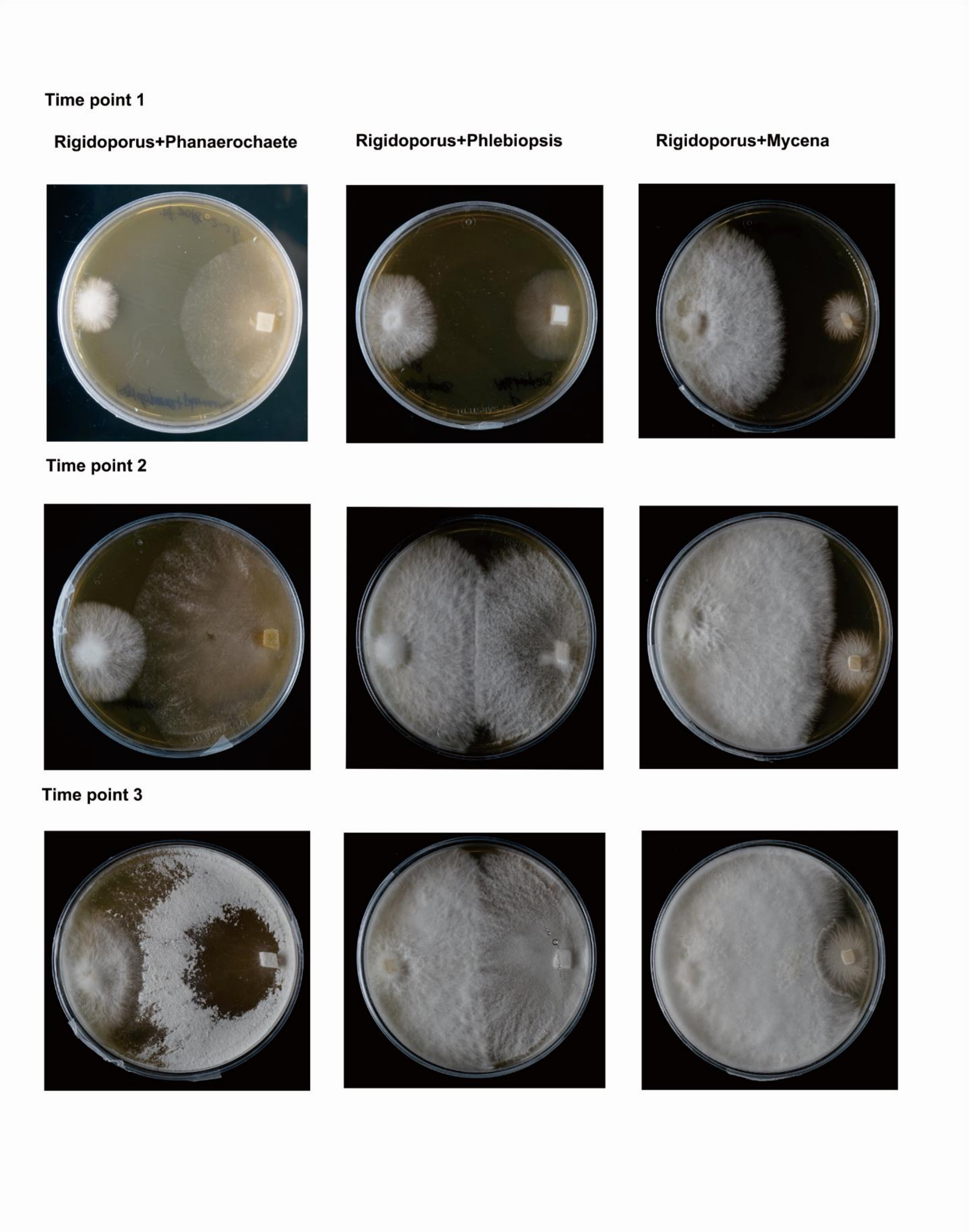

Supplementary Figure 6. Results of qRT-PCR analysis of the expression level of predicted effector-encoding genes in dual co-cultures of *R. microporus* with the indicated fungal species at the time point 1.

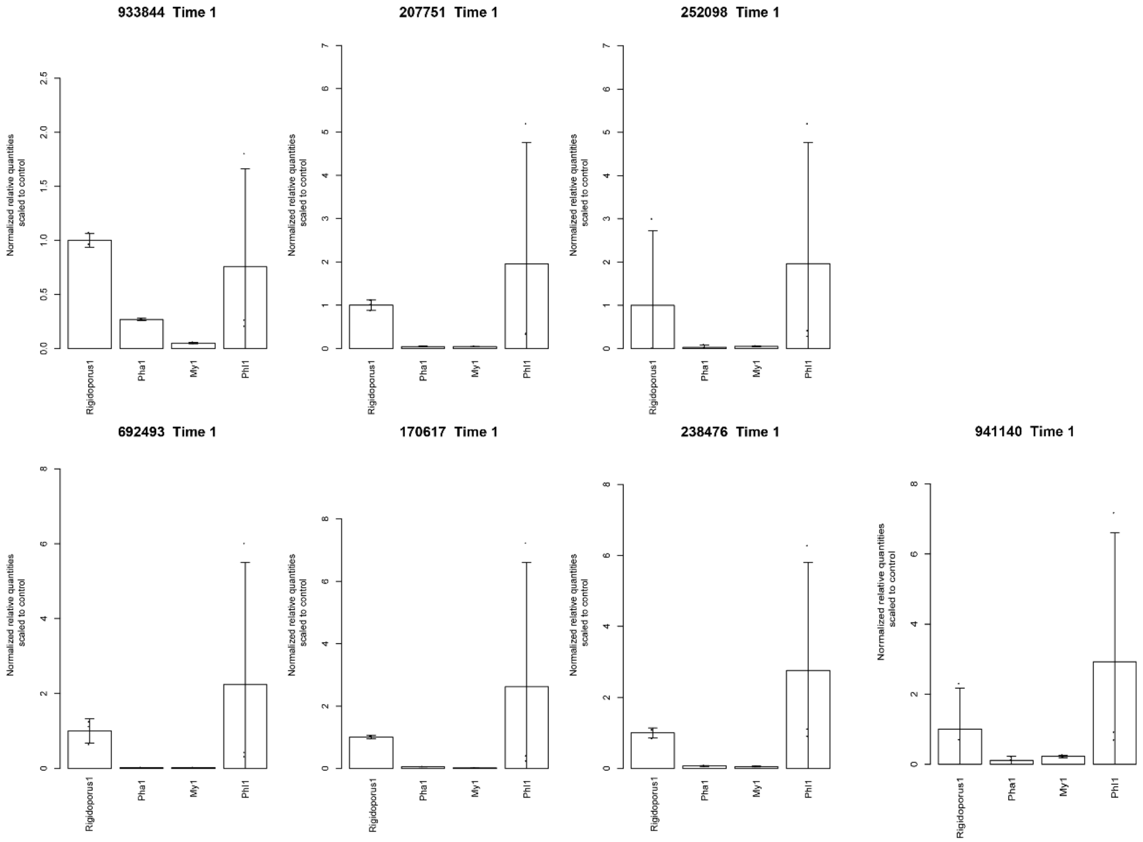

Supplementary Figure 7. Results of qRT-PCR analysis of the expression level of predicted effector-encoding genes in dual co-cultures of *R. microporus* with the indicated fungal species at the time point 2.

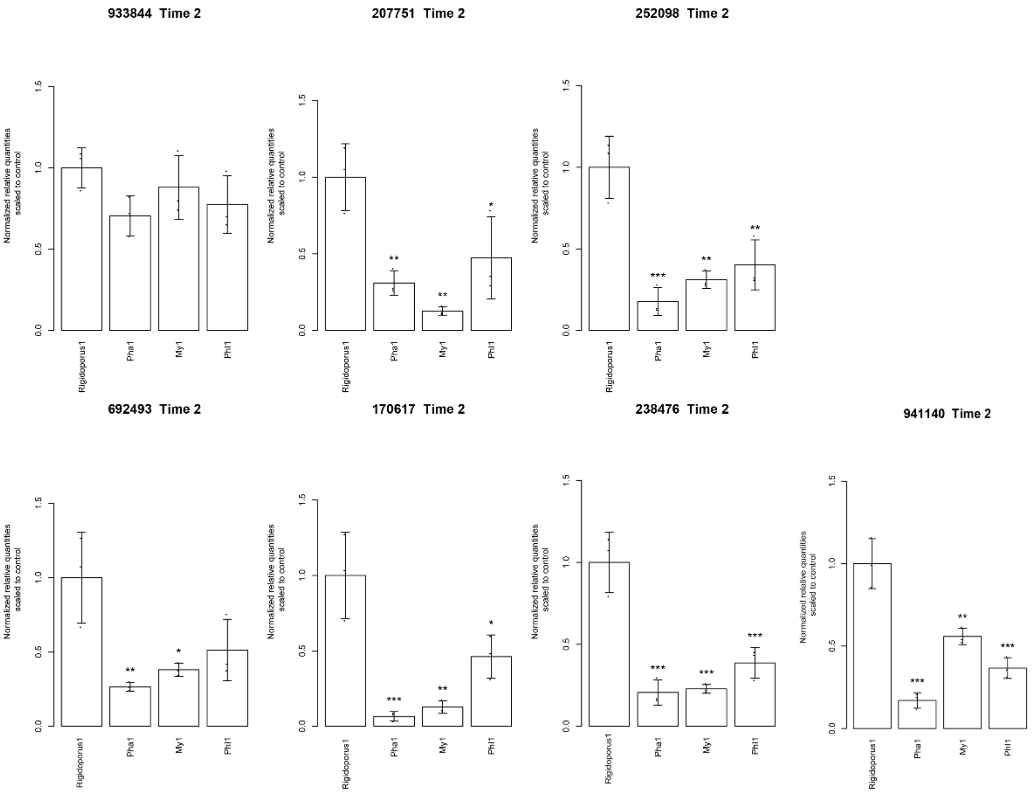

Supplementary Figure 8. Results of qRT-PCR analysis of the expression level of predicted effector-encoding genes in dual co-cultures of *R. microporus* with the indicated fungal species at the time point 3.

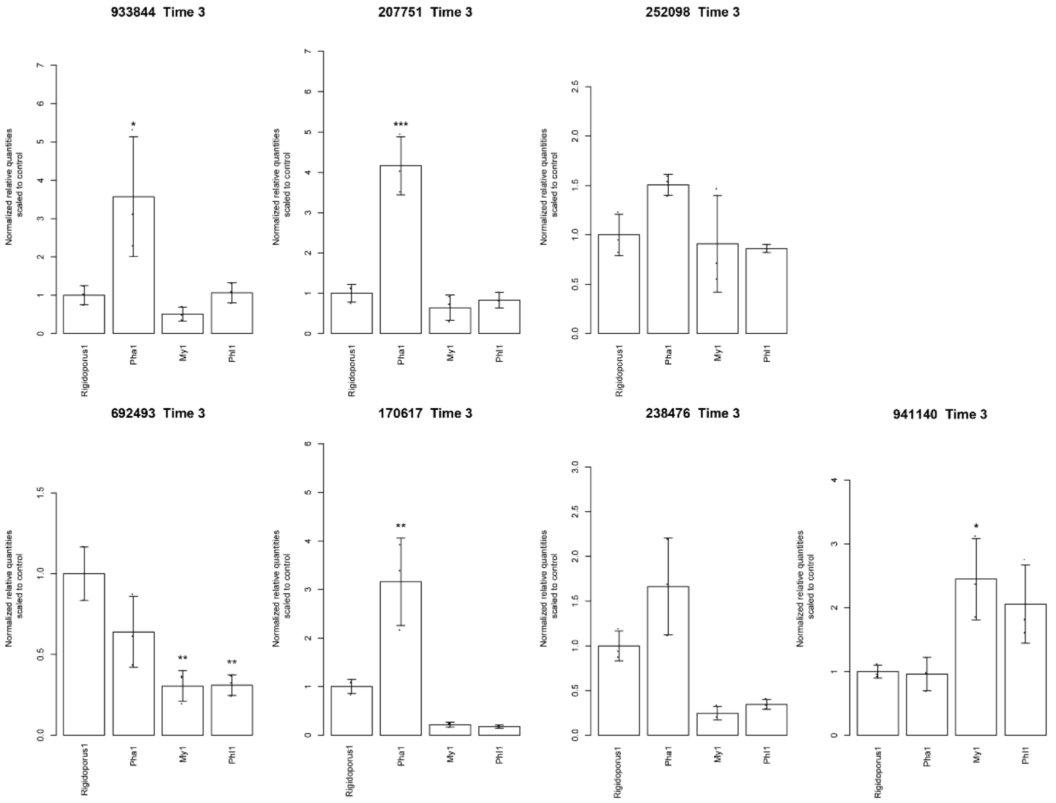

Supplementary Figure 9. Neighbor-Joining (NJ) phylogenetic tree based on 10 single copy gene datasets mined from the genomes of 29 fungal species using MEGA 7. *Auricularia subglabra* and *Exidia glandulosa* were used as an outgroup.

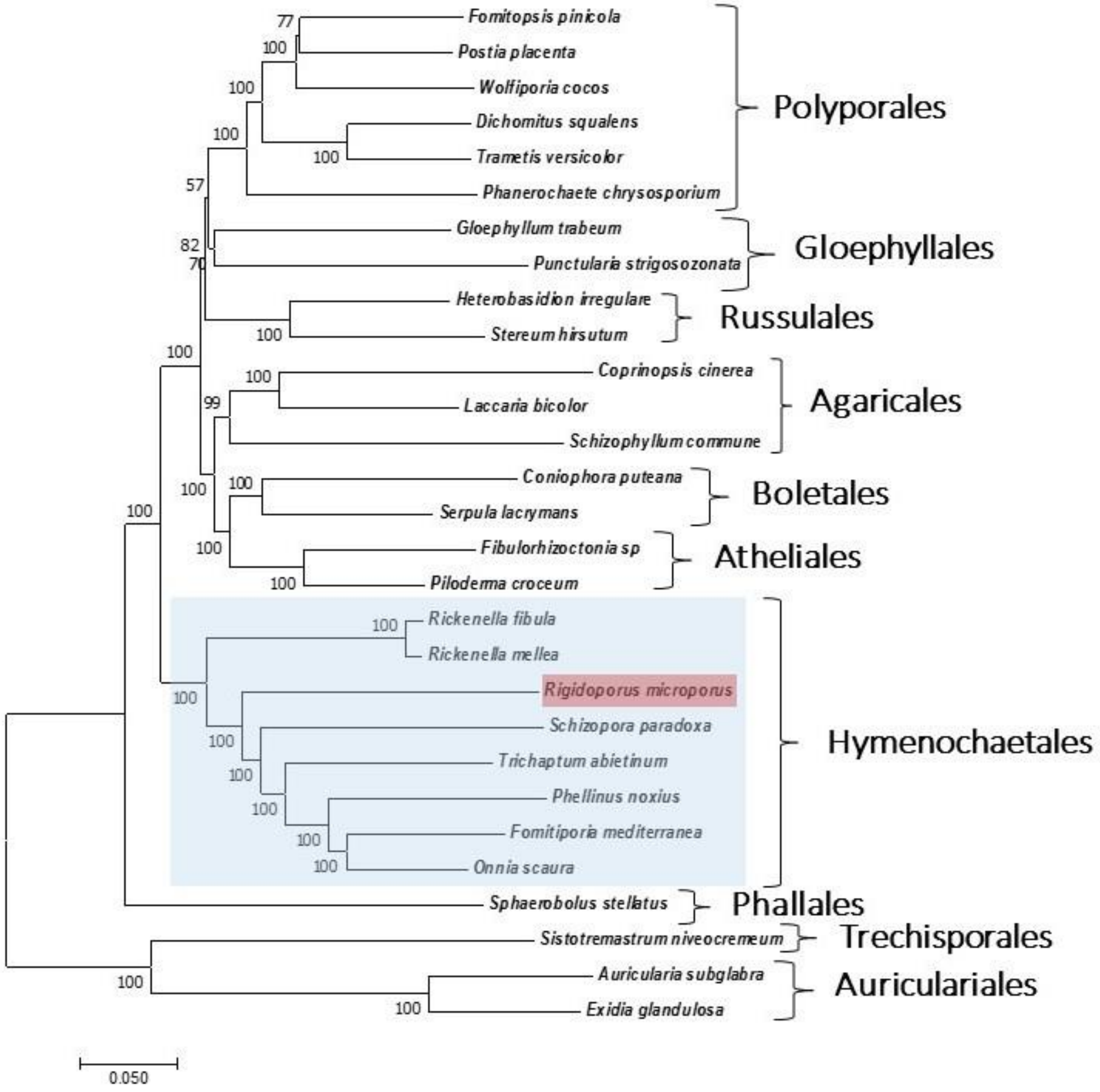

Supplementary Table 1. Alignment details of single copy orthologous genes used for the multigene phylogenetic analysis. Total of 12, 074 amino acid sites from concatenation of Gblocks-treated alignments of all 10 orthologs were used for phylogenetic tree construction.

**S/N**	**Orthodb ID**	**Gene**	**Full length**	**Length post Alignment/Gblocks**
1	EOG091T0023	Myosin motor domain	2250	856 (38%)
2	EOG091T0066	GRAM domain	1873	373 (19%)
3	EOG091T002F	SEC7 domain	2337	1265 (54%)
4	EOG091T003Y	CCR4-NOT	2868	1604 (55%)
5	EOG091T000T	DNA-directed polymerase	2441	1687 (69%)
6	EOG091T002P	ARID domain	3412	1187 (34%)
7	EOG091T0004	Dyein heavy chain	3959	1327 (33%)
8	EOG091T0069	DNA polymerase type-A	2928	791 (27%)
9	EOG091T0012	HECT	4139	2264 (54%)
10	EOG091T003N	Zinc Finger, PHD-type	2788	720 (25%)

Supplementary Table 2. Genomes of selected representatives of Agaricomycetes used for the phylogenetic analysis. Species belonging to the *Hymenochaetales* order are highlighted.

S/N	Symbol	Genome	Reference
1	AS	*Auricularia subglabra v 2.0*	[3]
2	CP	*Coniophora puteana v 1.0*	[3]
3	CC	*Coprinopsis cinerea v 1.0*	[11]
4	DS	*Dichomitus squalens v 1.0*	[3]
5	EG	*Exidia glandulosa v 1.0*	[12]
6	FS	*Fibulorhizoctonia* sp *v 1.0*	[12]
7	FM	*Fomitiporia mediterranea v 3.0*	[3]
8	FP	*Fomitopsis pinicola v 3.0*	[3]
9	GT	*Gloephyllum trabeum v 1.0*	[3]
10	HI	*Heterobasidion irregulare v 2.0*	[13]
11	LB	*Laccaria bicolor v 2.0*	[14]
12	OS	*Onnia scaura v 1.0*	Unpublished, approval to use genome data obtained from PI
13	PC	*Phanerochaete chrysosporium v 2.2*	[15]
14	PN	*Phellinus noxius v 1.0*	[16]
15	PI	*Piloderma croceum v 1.0*	[17]
16	PP	*Postia placenta v 1.0*	[18]
17	PS	*Punctularia strigosozonata v 1.0*	[3]
18	RF	*Rickenella fibula v 1.0*	Unpublished, approval to use genome data obtained from PI
19	RI	*Rickenella mellea v 1.0*	Unpublished, approval to use genome data obtained from PI
**20**	**RM**	***Rigidoporus microporus v 1.0***	**This Work**
21	SC	*Schizophyllum commune v 3.0*	[19]
22	SP	*Schizopora paradoxa v 1.0*	[6]
23	SL	*Serpula lacrymans v 2.0*	[20]
24	SN	*Sistotremastrum niveocremeum v 1.0*	[12]
25	SS	*Sphaerobolus stellatus v 1.0*	[17]

26	SH	*Stereum hirsutum v 1.0*	3
27	TV	*Trametis versicolor v 1.0*	3
28	TA	*Trichaptum abietinum v 1.0*	Unpublished, approval to use genome data obtained from PI
29	WC	*Wolfiporia cocos v 1.0*	3

Supplementary Table 3. List of ORFs detected in the mitochondrial genome of *R. microporus*.

Gene	Feature	Strand	Start	Stop	Note / E-value, score, Ident	Length (nt | aa)
ORF1	CDS	+	526	1695	intron-encoded LAGLIDADG endonuclease [*Ustilago bromivora*] / 2e-155, 453, 58%	1170 | 389
ORF2	CDS	+	15586	16161	DNA polymerase family B [*Parasitella parasitica*] / 0.03, 48.5, 30%	576 | 191
ORF3	CDS	+	25726	27576	NADH dehydrogenase subunit 2 [*Fomitopsis palustris*] /0.0, 584, 55%	1851 | 616
ORF4	CDS	+	29065	29832	cytochrome oxidse subunit 2 [*Trametes cingulata*] /5e-155, 440, 81%	768 | 255
ORF5	CDS	+	30736	31086	unnamed protein product [*Oikopleura dioica*] / 0.005, 45.4, 36%	351 | 116
ORF6	CDS	+	31372	32181	translation initiation factor IF-2 [*Chryseobacterium piscicola*] / 4.8, 40.0, 38%	801 | 269
ORF7	CDS	+	36514	37689	homing endonuclease [*Thelephora ganbajun*] / 9e-138, 405, 70%	1176 | 391
ORF8	CDS	+	39571	40713	orf360 (mitochondrion) [*Rhizopus oryzae*] / 0.0, 520, 71%	1143 | 380
ORF9	CDS	+	43654	45621	NADH-ubiquinone oxidoreductase chain 5 [*Heterobasidion irregulare*] /0.0, 1033, 77%	1968 | 655
ORF10	CDS	+	46579	47202	hypothetical protein [*Heterobasidion irregulare*] / 1e-05, 55.1, 28%	624 | 207
ORF11	CDS	+	47800	48618	cytochrome c oxidase subunit 3 [*Hericium coralloides*] /2e-134, 389, 70%	819 | 272

ORF12	CDS	+	50560	51576	NADH dehydrogenase subunit 1 [*Thelephora ganbajun*] / 0.0, 602, 88%	1017 | 338
ORF13	CDS	+	2351	2803	hypothetical protein SCLCIDRAFT_146523 [*Scleroderma citrinum* Foug A] /2e-78, 245, 81%	453 | 150
ORF14	CDS	+	3239	4690	NADH dehydrogenase subunit 4 [*Hericium coralloides*] / 0.0, 754,76%	1452 | 483
ORF15	CDS	+	5339	6700	40S ribosomal protein S3 [*Thelephora ganbajun*] / 4e-24, 113, 31%	1362 | 453
ORF16	CDS	+	14288	14926	hypothetical protein POSPLADRAFT_1161041 [*Postia placenta* MAD-698-R-SB12] /2e-99, 295, 74%	639 | 212
ORF17	CDS	+	11262	11768	LAGLIDADG type homing endonuclease [*Ganoderma meredithae*] / 1e-65, 209, 66%	507 | 168
ORF18	CDS	+	16524	17399	hypothetical protein GALMADRAFT_82379 [*Galerina marginata* CBS 339.88] / 2e-40, 153, 38%	876 | 291
ORF19	CDS	+	24231	25295	MerR family transcriptional regulator [*Tetragenococcus solitarius*] / 1.2, 40.4, 29	1065 | 354
ORF20	CDS	+	27576	27974	NADH dehydrogenase subunit 3 [*Hericium coralloides*] / 5e-56, 179, 76%	399 | 132
ORF21	CDS	+	37971	39332	LAGLIDADG homing endonuclease [*Rhizoctonia solani* AG-1 IB] /0.0, 752, 85%	1362 | 453
ORF22	CDS	+	40848	42176	endonuclease [*Trichosporon asahii var. asahii* CBS 8904] / 1e-165, 484, 57%	1329 | 442
ORF23	CDS	+	42258	42974	hypothetical protein PHLGIDRAFT_412339 [*Phlebiopsis gigantea* 11061_1 CR5-6] / 7e-149, 426, 87%	717 | 238
ORF24	CDS	+	49170	49931	ATPase subunit 6 [*Hyphodontia nespori*] /2e-134, 387,89%	762 | 253
ORF25	CDS	-	19207	19512	hypothetical protein PAXRUDRAFT_19721 [*Paxillus rubicundulus* Ve08.2h10] / 1.5, 37.7, 37%	306 | 101

ORF2 6	CDS	-	1959 6	20174	GGDEF domain-containing protein [*Ferrimonas kyonanensis*] / 9.6, 37.7, 31%	579 | 192
ORF2 7	CDS	-	1814 7	18740	von Willebrand factor type A domain containing protein [*Trichomonas vaginalis* G3] / 0.010, 25.8, 57%	594 | 197

Supplementary Table 4. List of tRNA genes detected in the mitochondrial genome of *R. microporus*.

tRNA #	Begin	End	Type	Codon	Score	Pseudo
1	117	187	Ile	GAT	34.5	No
2	7072	7154	Leu	TAA	44.2	No
3	12767	12837	Ala	TGC	38.4	No
4	12955	13026	Phe	GAA	56.4	No
5	13299	13370	Thr	TGT	48.8	No
6	13511	13583	Gln	TTG	39.3	No
7	22291	22361	Val	TAC	29.2	No
8	22651	22725	Leu	TAG	32.3	No
9	22898	22972	Arg	TCG	45.2	No
10	23750	23822	Trp	CCA	37.5	No
11	33243	33318	His	GTG	33.6	No
12	33628	33701	Met	CAT	49.1	No
13	33959	34031	Lys	TTT	41.6	No
14	34306	34389	Tyr	GTA	31.30	No
15	34392	34465	Met	CAT	42.0	No
16	34468	34542	Pro	TGG	22.90	No
17	34698	34774	Asn	GTT	52.9	No
18	35082	35167	Ser	TGA	53.0	No
19	35475	35548	Asp	GTC	53.6	No
20	35564	35635	Gly	TCC	49.1	No
21	35803	35877	Arg	TCT	45.9	No
22	35912	35983	Met	CAT	37.2	No
23	35986	36056	Glu	TTC	35.3	No
24	36320	36400	Ser	GCT	19.40	No

Supplementary Table 6. Putative natural product genes in the *R. microporus* genome based on sequence homology.

Predicted protein function	Name	Protein ID	Scaffold	Nucleotide range	Protein length (aa)	Domain architecture	Notes	Clusters with
**Non-ribosomal peptide synthase**	Nrp1	944992	7	351562-359604	2322	A-T-C-T-C	Hydroxamate siderophore biosynthesis SidD homolog (*Aspergillus fumigatus*)	Mon1
**Non-ribosomal peptide synthase-like protein**	Nrl1	924741	14	333882-338533	1414	ADA-A-T-R	L-alpha-aminoadipiate semialdehyde reductase, lys2 homolog	
**Polyketide synthase**		none						
**Terpene cyclases**	Ter1	839171	56	6927-8081	344			
	Ter2	854007	56	62349-63618	342			
	Ter3A	986842	34	118174-119565	331			TerB, TerC, Znf1, Oxi1,Oxi2
	Ter3B	838119	34	120482-121634	344			TerA, TerC, Znf1, Oxi1, Oxi2
	Ter3C	950024	34	123309-124443	348			TerA, TerB, Znf1, Oxi1, Oxi2
	Ter4	908344	2	778087-779644	328			
	Ter5	827067	2	1035754-1036879	337			
	Ter6A	826611	2	1317761-1318884	335			Ter6B, Ter6C, Znf2, Tra3
	Ter6B	941833	2	1319987-1321105	325			Ter6A, Ter6C, Znf2, Tra3

	Ter6C	1009122	2	1323298-1324404	330			Ter6A, Ter6B, Znf2, Tra3
	Ter7	827315	2	1379789-1380916	335			Tra4
	Ter8	923615	8	241176-243312	367			Met1, Oxi3
	Ter9	961004	11	1022511-1023965	392			
	Ter10	840896	113	21080-22234	344			Tra5, Oxi4, Oxi5
	Ter11	918081	69	52544-53935	342			Oxi6
	Ter12	921347	2	765545-766639	338			
**Oxidoreductases**	Smo1	649158	7	348288-350743	610		Hydroxamate siderophore biosynthesis SMO1 homolog (*Ceriporiopsis subvermispora B*)	Nrp1
	Oxi1	838076	34	110606-113318	641		flavin-dependent monooxygenase	Ter3A, Ter3B, Ter3C, Znf1, Oxi2
	Oxi2	917151	34	107336-109469	445		FAD-dependent monooxygenase	Ter3A, Ter3B, Ter3C, Znf1, Oxi1
	Oxi3	930040	11	1029997-1033267	574		FAD-dependent monooxygenase	Ter9, Met1
	Oxi4	903147	113	27612-29356	451		NAD-binding monooxygenase	Ter10, Tra5, Oxi5
	Oxi5	1016622	113	36489-38304	445		FAD/NAD-binding monooxygenase	Ter10, Tra5, Oxi4
	Oxi6	900847	69	45092-47959	563		FAD/FMN-containing dehydrogenase	Ter11
**Methyl transferases**	Met1	887163	11	1028487-1029997	309			Ter9, Oxi3

**Halogenase**		none						
**Phenylalanine ammonia lyase**	Pal1	948695	20	346668-350530	943			
**Transporters**	Tra1	939108	56	4628-5296	115			Ter1
	Tra2	925935	56	2063-4383	505		MFS transporter	Ter1
	Tra3	921485	2	1305217-1307637	518		MFS transporter	Ter6A, Ter6B, Ter6C, Znf2
	Tra4	921505	2	1382623-1384887	549		MFS transporter	Ter7
	Tra5	840884	113	24779-27057	554		MFS transporter	Ter10, Oxi4, Oxi5
**Histone Acetyl Transferase (HAT)**	GNAT	840891	113	14063-14836	187			Ter10?
**Transcription factor**	Znf1	457826	34	113730-115864	538		C6 Zn finger	Ter3A, Ter3B, Ter3C, Oxi1, Oxi2
	Znf2	826554	2	1315176-1315774	164		C6 Zn finger – potentially only co-activator	Ter6A, Ter6B, Ter6C, Tra3
	Znf3	950902	69	50600-51801	358		C6 Zn finger	Ter11

Abbreviations in domains of NRPS and NRPS-like proteins are:
A: adenylation domain
T: thiolation domain/peptidyl carrier protein
C: condensation domain
ADA: adenylation activating domain
R: reductase domain

Supplementary files 1-11 – Aligned sequences of single copy orthologous genes (1-10) and concatenated sequences of all 10 genes (11) used for the multigene phylogenetic analysis. Total of 12, 074 amino acid sites from concatenation of Gblocks-treated alignments of all 10 orthologs were used for phylogenetic tree construction.

01-Myosin motor domain

```
>CC
MRRTGQDQCI IFTGETASGK SEARRLAIKS LITLSVPPAG KKGSKLAQQI PASEFVLESF
GNARTLFNPN ASRFGKYTEL QFTRGRLCGV KTLDYYLERG RVAAVPSGER NFHIFYYLVR
GVNDDERGHL NLGDGRNFRY LGGQDALKFD QLKIALKTIG FSKRSVAQCM QLVAAILHLG
NLDFTIDKSR NEDAAVVRNL DTLNLVADFL GVDPQGLEGA LSYKTKMVKK ELCTVFLDVE
GAEANRDELA KTLYSLLFAW VNEYVNQRLA VGDFDAFIGL LDLPGPQNMS RPNSLDQFIV
NYANERVQGF VRNKIFNGNQ QEFQFEGLTH LLPSLSTPDN SETLRLLHNY PGGLIHIMDD
QARRQPKKTD GTMVEAFQKR WGNHSSFKTS GVVDGASFTV SHYNGAVTYA VEGFLERNLD
SVNPDFVSLL RGGSINPFVK SLFSSKAIAT QAHPRHEETI VAAQQAVKPM RAPSMRRKGT
PCVAGEFRAA LDTLFATLDE TQPWFVFCIN PNDSQLPNQL EGRSVKGQVR SCGMGGVARR
NLVYWEVNMT FGEFEERYAG VLEEVGVTPS FKDVVLGKTK VWLSQRAFRA LEDRLRIKEG
GERVGRELGM AGASNQRLPL VSNASPFEEY EPPSRIRGDE ESYAPSRNMF AGGDGVAAPG
ETTEVLKESS ARRRWVAFTW LLTWWIPSPF LVWFGRMKRM DVRQAWREKL ALNLIIWFIC
GCTVFVIAVL GPVICPTEHV YSTTELSDHS NKVYTAIRGE YGGREINDLF PVQVSALCDG
IDGNVSPYVM LSSRNTDTNA VYHDFRASTN DPRPDWYFQQ MVTMRYTARV GFRGYTPKEI
RNMANRGQSV AIYNGL
>FP
MRRTTQDQSI LLTGETGSGK SENRRLAIKT LLELSVSQPG KKGAKLVNQV PAAEFVLETF
GNARTLFNPN ASRFGKYTEL QFTRGRLCGM KTLDYYLERN RVAGAPSGER NFHVFYYLVA
GASPEERQYL HLQDKTTYRY IGLRDAVRFD QLKMALKNVG FSKRHVAQTC QLVAAILHLG
NLEFTIDRFR NEEAAVVRNT DVLDIVADFL GVQPSALEAS LSYRTKLVKK EVCTVFLDPD
GASDNRDELA KSLYALLFAW LNEHINQRLC RDDFNTFIGL FDLPGPQNLS RPNSLDQFCV
NFANERLQNF IQKKLFESHV VEYNSEGISR LVPQVAYFDN SECLRLLQNK PGGLIHIMDD
QARRMPKKTS HTMVEAFTKR WGNHSSFKAG SIDRFPTFTV NHFTGPVVYS SENFLERNLE
ALNPDFVSLL RGGSSNPFIR SLYSNKAITA QAHPRNEETI VAAQQPVKPM RAPSTRRKGT
PCVAGEFRAA LDTLFETLEE TQSWYVFCIN SNDSQLPNQL EGRSVKGQVR SLGLAEVAKR
NVTVFKANMT PEEFVQRYSG YLTTLNVHEG DKDIVLGMSQ VYLSQRAFHA LEDDLRSKDT
EEQKRNRMRD AEASNQQLPL VQNAQPMDDY DGRSAYTSHR ESYAPSRNMF QDADGEIQEG
ETTEVLKEST LRRRWVMICW LLTFWCPTPF LRWFGRMKRP DVQQAWREKL ALNMLIWFIC
GCAIFVIAVF GNLICPTEHV FTTSELQEHS NNVYTSIHGE YGGVSSDDIF PLQVSALCNG
VSGSVNPYVQ YSSANTDVNA QYHDFRAWTG DSRPDWYFEQ MVQMRWNNRV GWMGYTTQDL
NNMAGKGSSV GVYEGI
>GT
MRRTGQDQSI LLSGETGSGK SENRRLSIKT LLELSVSSPG KKGSKLAHQV PAAEFILETF
GNSRTLFNAN ASRYGKYTEL QFTRGRLCGV KTLDYYLERN RVAQVPSGER NFHIFYYLVA
GASPEERQHM HLDERANYRY LGQRDGARFE QLKMAMKNVG LSKRHVAQTC QLVAAILHLG
NLEFTIDRHR NEDAAVVRNT DTLGIVADFL GVQPAALEAA LSYKTKLVRK ELCTVFLDPD
GASDNRDDLA KTLYSLLFAW LNEHINQKLC KEDFATFIAL FDLPGPQNMS RPNSLDQFCV
NFANERLHNF IHKRIFERDT AQYASEGLSN YVPQVPYFDN SECLRLLQNK PGGLIHIMDD
QALRMPKKTD HSMVEAFGKR WGNHSSFKMG SIDRYPTFTV HHYNGPVTYS SEGFLERNLD
AVNPDFVSLL RGGSANPFVK GLFSAKAIAT QAHPRNEDTI VAAQQPQKPM RAPSTRRKNT
PCVAGEFCSA LDTLFETLEE SQSWYVFCIT PNDSQLPNQL EGRSVKGQVR AMGLPEIARR
FINVFEVNMT PDEFVERYKE SLASLNVHEG DQDIVIGQHK VFLSQAAFHK LEDHLRSKDV
EEQKRNRLRD AEASSQALPL VSHASPFDDF DGRSRLTSNR ESYAPSRNMF QNADADVQEG
ETTEVLKESS ARRRWVALCW MLTWWVPTPC IRWVGRMKRP DVQQAWREKL ALNMIIWFVC
ACAIFVIAVL GNVICPTEHV FSTSELASHN NNVYTSIRGE YGGTSSDNIF PVQVSALCNG
VSGSVSPYVV LDSTNTDPNA QYHDFRAFTN DSRPDWYFEV MTQMRWKNRV GFMGYTPKEI
```

RNMASNKKAV GIYNGL
>PP
MRRTNQDQSI LLSGETGSGK SENRRLAIKT LLELSVSQPG KKGSKLAHQV PAAEFVLEAF
GNSRTLFNPN ASRFGKYTEL QFTRGRLSGM KTLDYYLERN RVSGAPSGER NFHIFYYLVS
GASAEERQHL HLQEKTTYRY LGQRDAVRFD QLKVALKNVG FSKRHVAQTC QLLAAILHLG
NLEFTIDRAR NEDAAVVRNT DILEIVADFL GVQPAALEAA LSYRTKLVKK ELCTVFLDPD
GASDNRDELA KSLYALLFAW LNEHINQRLC RDDFNTFIGL FDLPGPQNLS RPNSLDQFCI
NFANERLQNF IQKRLFESHA SEYANEGISR FVPQIPYFDN SECLRLLQNK PGGLIHIMDD
QARRIPRKND HTMVESFTKR WGNHSSFKSG GMDRFPTFTV NHFGGPVTYS AESFLERNLD
ALNPDFVSLL RGGSTNPFVR GLYSAKAIAT QAHPRDEETI VAAQQPVKPM RAPSTRRKGT
PCVGGEFRAA LDTLFETLEE TQSWYVFCIN PNDSQLPNQL EGRSVKGQVR SIGLAEIARR
NINVFEANMT QDEFVDRYKA QFTSLNVVEG EKDVVLGTNK VFLSQAAFHA FEDDLRSKDT
EEQKRNRLRD AEASNQALPL VNNAAPFDDF DGRSAYTSHR ESYAPSRNMF QDADGEIQDG
ETAEILKETP LRRRWVMLCW VLTFWVPTPL LAWAGRIKRP DVQQAWREKL AINMLIWFVC
GCVIFIIAIF GPLICPTQHV FNTNELETHS NNAYVAIHGE YAGTTADALF PLQVSALCNG
VTGSVNPYVV FDSGNTDVNA QYHDFRAWTN DPRPDWYFES MVQMRWNNRV GWIGYTMKDL
SSMASSGKSV GVYDGI
>WC
MRRTTQDQSI LLSGETGSGK SENRRLAIKT LLELSVSQPG KKGAKLAYQV PAAEFVLETF
GNARTLFNPN ASRYGKYTEL QFTRGRLCGM KTLDYYLERN RVAGAPSGER NFHIFYYLVA
GASVEERQHL HLQEKTTYRY LGQRDAVRFD QLKMALKNVG FSKRHVAQTC QLIAAILHLG
NLEFTIDRSR NEDAAVVRNT DVLELVSEFL GVQPAALEAT LSYRTKLVKR ELCTVFLDPD
GASDNRDELA KSLYALLFAW LNEHINQRLC RDDFNTFIGL FDLPGSQNLS RPNSLDQFCV
NFANERLQNF VQKRLFESHV SEYNSEGIAR YVPQVPFFDN SECIRLLQHK PGGLIHIMDD
QARRMPRKTN QTMVEAFSKR WGNHSSFKAG AIDRCPTFTV NHFSGPVTYS SENFLERNLD
ALNPDFVSLL RGGSTNPFIR ELYSAKAIAT QAHPRDEETI VSAQQPVKPM RAPSTRRKNT
PCIAGEFRAA LDTLFETLEE TQSWYVFCIN PNDSQLPNQL EGRSVKGQVR SLGLSEVSRR
CVNVFEVNMT SDEFVQRYRA HLTALNVTEG EKDIVLGVRQ VFLSQAAFHA FEDDLRSKDT
DEQKRNRLRD AEASSQALPL VSNASPFDDY DGRSAYTSHV ESYAPSRNMF QDADGEIQEG
ETAEVIKETP LRRRWVILCW ILTWWVPNPL LAWLGRMKRP DIQQAWREKL AINMLIWFVC
GCAVFVIVFF GPIICPTEHV FTTSELQSHS NNVYTSIRGE YGGTSSDAIF PVQVSALCNG
VTGSVSPYVV LSTSNTDANA QYHDFRAWTN DPRPDWYFEV MVQMRWNNRV GWMGYTSKDL
QNMASEGKSV GVIDSI
>PC
MRRTAQDQCI LLSGETGSGK SENRRLAIKT LLDLSVSNPG KKGSKLAHQL PAAEFVLESF
GSARTLFNAN ASRFGKYTEL QFTRGRLCGV KTIDYYLERA RVASVPSGER NFHIFYYLMA
GASPEERQHM HLNEKATYRY LAQRDAIRFD QLKMALKNVG FSKRHVAQTC QLIAAILHLG
NLEFTIDRHR NEDAAVVRNM DVLEIVAEFL GVQPAALETA LTYRTRLMKK ELCTVFLDPD
GAADNRDELA VTLYSLLFTW LNEHINQRLH KDDFTSFIAL LDFPGPQNLS RPNGLDQFCV
NFANERLQNF IQKRLFESHV SEYNAEGIAR FVPQVPYFDN SECIRLLQHR PGGLIHIMDD
QARRMPRKTN HTMVEAFSKR WGNHSSFKVG SADRFPTFTV NHFTGPVTYS AEGFLEKNQD
TLSPDFVQLL RGGSINPFVR GLFTSKAVAT QVHPKNEETI IAAQQPVKPM RAPSTRRKNT
PCIAGQFRAA LDMLFETLEE AQAWYVFCIN PNDSQLPNQL EGRSVKGQVR SLGLAEVARR
CVNVFEVAMT PQEFVERYRD TLHQVGIQEG EKDIVLGMTM VFLSHAAFHR LEDDLRAKDT
EEQKRNRIRE AEASSQQLPL VSNASPFDEY DVRSALTSHR ESYAPSRNMF QNADGEIQEG
ETVEILKESS ARRRWVMLCW ILTWWIPTPV LVHIGRMKRP DIQQAWREKL ALNMLIWFMC
ACAVFVIAVI GPLICPTEHV FSTSELQSHS NNVYTSIRGE YGGVAADNLF PVQVSALCNG
VTGSVSPYVV LDSSNTDPNA QYHDFRAWTS DPRPDWYFES MTLMRWNNRV GFVGIDSKGL
KNMANQRRIV AVYRGL
>DS
MRRTQQDQSV IFTGETGSGK SENRRLAIKT LLELSVSQPG KKGSKLGNQV PAAEFVLETF
GNSRTLFNPN ASHFGKYTEL QFTRGRICGV KTLDYYLERS RVSGAPSGER NFHIFYYLIA
GASAEERTHM HLSDRTQYRY LGARDAVRFE QLKVALKNVG FSKRHVAQTC QLIAAILHLG
NLEFTIDRSR NEDAAVVRNL DVLEIVSEFL GVQPSALEAA LSYKTKLVKK ELCTVFLDPD

GASDNRDELA RMLYSLLFAW LNEHINQRLC RDDFTTFIGL FDLPGPQNMS RPNSLDQFCI
NFANERLINF MQKRLFESHV QEYNNEGISR FVPHVPYFDN SECIRLLQNR PGGLIHIMDD
QARRMPRKTD HTMVEAITKR WANHSSFKSG GMDRFPTFTI NHYSGPVTYS AQNFLEKNLD
AINPDFVSLL RGGSMNPFVR GLFSGKAIAT QAHPRNEDTI VSAQQPVKPM RAPSTRRKNT
PCVAGEFRQA LDVLFETLED TQSWYIFCIN PNDSQLPNQL EGRSVKAQVR SVGLSEIARR
NVNVFEAMMT PDEYVQRYQS LLTSLGVTEG ERDVVLGMSM VFLSQAAFRR LEDDLRSKDT
EEQKRNRIRD AEASNQQLPL VANASPFDDF DGRSAYTSHR ESYAPSRNMF QNADGEVQEG
ETAEVLKESS ARRRWVALCW MLTFWCPTFL LRYVGRMKRL DVQQAWREKL ALNMIIWFVC
ACAVFVIVFI SPIICPTEHV FNTAELNGHS NNVYVAIRGE YGGTQADDLF PVQVSALCNG
ISGSVNPYVV LTTKNTDQNA QYHDFRSWTN DPRPDWYFEQ MTMLRWNYRV GFVGYSPKQI
KSMASTGSSV AIYKGL
>TV
MRRTAQDQSM LFTGETGSGK SENRRLAIKT LLELSVSQPG KKGSKLGNQV PAAEFVLEAF
GNSRTLFNPN ASHFGKYTEL QFTRGRISGV KTLDYYLERN RVAGAPSGER NFHIFYYLVA
GASPEERQHM HLSDKTQYRY LGARDAVRFD QLKVALKNVG FSKRHVAQTC QLIAAILHLG
NLEFTVDRHR NEDAAVVRNL DVLEIVSEFL GVQAAALEAT LSYKTKLVKK ELCTVFLDPD
GASDNRDELA KTLYSLLFAW LNEHINQRLC RDDFATFIGL FDLPGPQNMS RPNSLDQFCV
NFANERLLNF IQKRLFEAHV QEYNTEGISR FVPHVPYFDN SECLRLLQHR PGGLIHIMDD
QGRRMPRKTD HTMVEAFTKR WGNHSSFKAG GMDRFPTFTV NHYSGPVTYS ASGFLEKNLD
AINPDFVSLL RGGSMNPFVR GLFSAKAIAT QAHPRDEDTI VAAQQPVKPM RAPSTRRKNT
PCVAGEFRQA LDVLFETLED TQSWYVFCIN PNDSQLPNQM EGRSVKAQVR SVGLSEIARR
SVNVFEAMMT PDEYTQRYGG LLQAVGVSEG ERDVVLGLSM VFLSQAAFHR LEDDLRSKDT
EEQKRNRMRD AEASNQALPL VQHASPFDDF DGRSAYTSQR ESYAPSRNMF QNTDGEIQEG
ETTEVVKESS ARRRWVLLCW MLTWFIPNFA LTKIGRMKRD DVRQAWREKL AINMIIWFVC
GCAIFVIAII SPLICPREHI FNSQELAEHS NNVYTSIRGE YGGASADNVF PVQVSALCNG
ISGTVSPYVV LSSSNTDVNA QYHDFRSWTT DPRPDWYFES MTQMRWQARV GYVAYSSKQL
KSMASSGNSV GIYRGF
>SC
MRRTNQDQSI LLSGETASGK SENRRLAIKS IIELSVSSPG KKGSKLASQI PAAEFVLETF
GNARTLFNPN ASRFGKYTEL QFSRGRLCGI KTLDYYLERS RVSAVPNGER NFHIFYYLVA
GAAPEERQHM HLTDKATYRY LGASDANRFD QLKVALKTIG LSKRHVAQTC QLIAAILHLG
NLEFTIDRHR NEDAAVVRNV DELEIVAEFL GVTPATLESA LSYKTKLVKK ELCTVFLDPD
GASDNRDDLA KTLYSLLFAW LNEHINQRLC KDDFATFIGL FDLPGPQNMS RSNSLDQFVV
NFANERLHNF IQKRLFENHV NEYAAEGIAH YVPQVPYFDN SECVRLLQNK PGGLIHIMDD
QSRRLPKKTD QTMVEAFQKR WGNHTSFRAG GLDRFPTFTV SHFNGPVTYS AESFLERNLD
ALNPDFVSLL RGGSDNPFVK GLFSAKAIAT QAHPRNEETI VAAQQVVKPM RAPSTRRKNT
PCVVGEFRSA LDTLFETMED TQSWYVFCIN PNDSQLPNQL EGRSVKGQVR SAGLTEVARR
NTVVFEVGMT PEEFCERYKD DLESLGVTEG DTDIVLGKHK VFLSHGAFHR FEDQLRSRDT
EEQKRNRMRD AEASNQRLPL VANAAGFDDF DGRSRFTGRD ESYAPSRNMF GNADGEIQDG
EVTEIIKESS ARRRWVMLCW ILTFWVPTPC LAWVGRMKRP DVQQAWREKL ALNMLIWFIC
GCAVFVIAIL GNIICPTQHV YSTGDVAEHN DSVYTYIRGE YGGGSSDDIF PVQVSAVCNG
KDGSVSPFVS MEVSNTDEYA KYHDFRAWTN DSRPDWYYES MTEMRWTSRV GFIGWTKKEL
KNKASSGSSV AIIDNM
>PS
MRRTNQDQSI LLTGETGSGK SENRRLAIKS LLELSVSNPG KKGSKLAAQV PAAEFVLETF
GNARTLFNPN ASRFGKYTEL QFNRGRLCGV KTLDYYLEKG RVAGAPSGER NFHIFYYLVA
GASPEERQHM HLIDKATYRY LGHHDGIRFD QLKMALKNVG FSKRHVAQTC QLIAAILHLG
NLEFTIDRGR NEDAAVVRNL DILNVVAEFL GVQPAALEAA LSYRTKMVRK ELCTVFLDPD
GASDNRDDLA KTLYSLLFAW LNEHINQKLC KDDYATFVAL FDLPGSQNMS RPNNLDQFCI
NYANERLQNF ILNQIFENHV SEYESEGIAA WVPRVPYFEN SECLRMIQNQ PGGLIHIMDD
QARRAPKKTD HTMVEAFSKR WGNHSSFKAG GVDRYPTFTV NHFNGPVTYS SENFLERNLD
AVNPDFVSLL RGGSNNPFVK GLFSSKAIAT QAHPRNEETI VAAQQPVKPM RAPSTRRKNT
PCVAGEFRSA LDTLFETLQD TQSWYVFCIN PNDSQLPNQL EGRSVKGQVR SAGLSAIAQR
NRCVFEVGMT PQEFVDRYQE PLTAIGVTEG EGDIVLGNHK AFLSQAAFHK LEDQLRSQDA

EEQKRNRMRD AEASSQQLPL VQNASSLDDY DQQSRFTSAR ESYAPSRNMF QGADGEIQEG
ETTEVVKETS ARRRWVALCW ILTFWMPNPF LRWFGRMKRM DVRQAWREKL ALNLIIWFVC
ACAVFVIAVL GNLICPTEHV FNTNELASHS NNVYTSIRGE YGGTSADNIF PVQVSALCNG
VDGTVSPYVI LDSSNTDVNA QYHDFRVFTN DSRPDWYFEQ MTVMRWNNRV GFMGYTPRAL
KNLANNKKNV AIYDGM
>LB
MRRTTQDQCI LFSGETGSGK SENRRLAIKS LLELSVSNPG KKGSKLATQL PAAEFVLETF
GNARTLFNPN ASRFGKYTEL QFSRGRLSGV KTLDYYLERN RVAAVPSGER NFHIFYYLVA
GASPEERQHL HLLDKSTYRY LGQRDAQRFD QLKIALKTIG FSKRHVAQTC QLIAAILHLG
NLEFTVDRHR NEDAAVVRNT DILEIVADFL GIQPAALEAA LSYKTKLVKK ELCTVFLDPD
GASDNRDDLA KTLYSLLFAW LNEHINQRLC KDDFTSFIGL FDLPGPQNMS RPNSLDQFCI
NFANERLQNW VQKRLFESHV NEYNLEGIAR LVPQVPYFDN SECIRVLQNS PGGLVHIMDD
QARRQPKKTD HTMVEAFQKR WGNHSSFKTG AVERFPSFTV NHFNGPVTYS SEGFLDRNLD
AVNPDYVSLL RGGSINPFVK GLFSTKAIAT QAHPRNEDTI VAAQQAVKPM RAPSTRRKNT
PCVAGEFKAA LDTLFETLDE TQPWYIFCVN PNDSQLPNQL EGRSVKGQVK STGLVEIAKR
CTSMFEVNMT PEEFCERYSE GLEGGGISEG DRDLVLGQHK VFLSQVAFHK FEDQLRSHDV
EEQKRNRIRD QEASNQALPL VSNASPFEDF DGRSRFTSNR ESYAPSRNMF QNTDGEIQEG
ETSEVLKESS ARRRWVALCW MLTFWVPTPL LTYVGRMKRM DVRQAWREKL ALNLLIWFIC
ACAVFVIAVL GVVICPTEHV FSTSELASHS NNVYTSVRGE YGGQSADNIF PVQVSALCDG
TSGSVSPYVT LDSSNTDPNS VYHDFRAFTN DSRPDWYFES MVVMRYTARV GFLGYTPKEI
RNMASAGRSA AIYNGL
>HI
MRRTTQDQCI ILNGETGSGK SENRRLAIKT LLELSVSNPG KKGSKLAHQV PAAEFVLESF
GNARTLFNAN ASRFGKYTEL QFSRGRLSGV KTLDYYLERN RVAGAPSGER NFHIFYYLVA
GASPEERQHM HLTDKMTYRY LGQRDAQRFD HLKMALKNVG FSKRHVAQTC QLIAAILHLG
NLEFTTDRSR NEDAAVVRNT DILALVAEFL GVTASALETA LSYKTKMVKK ELCTVFLDPD
GASDNRDDLA KTLYSLLFAW LNEHINQRLC RDDFVTFIGL FELPGPQNMS RPNSLDQFCI
NFANERLQNF IQKRLFESHV AEYNSEGISR FAPQVPYFDN TECIRLLQNK PGGLIHIMDD
QARRLGKKTD HTMVEAFGKR WGNHSSFKVG SMDRFPTFTV NHFNGPVTYS CEGFLERNLD
ALNPDFVSLL RGGSINPFVR GLFSGKAIAT QMHPRNEETI VAAQQPIKPM RAPSTRRKNT
PCVAGEFRAA LDTLFETLEE TQAWYVFCVN PNDAQLPNQL EGRSVKGQIR SMGLPEIARR
CETMFEVGMT PREFCDRYRE QIAGAGVSEG EKDLVLGQYK VFLSQVAFHK LENYLRSKDG
EEIKRNRMRE AEASNQALPL VSNASPFEDF DARSRLTSNH ESYAPSRNMF QNADGEIQEG
ETTEVVKETS ARRRWVALCW LLTWWVPTPF LKWFGRMKRF DVQQAWREKL ALNLIIWFIC
ACAVFVIAVL GNLICPTEHV FSTNELSAHS NNVYTSIRGE YGGKSADDLF PVQVSALCNG
VTGSVSPYVT LKFSNTDVNS KYHDFRVFTN DSRPDWYFES MTVMRWNNRV GFVGYTPKEI
KNMASQKRSL AVYNGL
>SH
MRRTTQDQCI VLSGETGSGK SENRRLAIKT LLELSVSNPG KKGSKLASQV PAAEFVLESF
GNARTLFNAN ASRFGKYTEL QFTRGRLCGI KTLDYYLERS RVAGAPSGER NFHIFYYLVA
GASPEERQHM HLLDKTTYRY LGQRDALRFD QLKLALKNVG FSKRHVAQTC QLIAAILHLG
NLEFTIDRAR NEDAAVVRNT DILALVAEFL GVTTSALETT LSYKTKMVKK ELCTVFLDPD
GASDNRDDLA KNLYSLLFQW LNEHMNQRLF KDDYVTFIGL FDLPGPQNMS RPNSLDQFCI
NFANERLHNF IQKRMFEAHR AEYQSEGIAQ YVPEVPYFDN SECLRLLQNK PGGLIHIMDD
QARRSAKKTD HTMVEAFSKR WGQHSSFKVG SVDRFPTFTV NHFNGPVTYS SDGFLEKNAH
SLNPDFVSLL RGGSVNPFIR GLYNSSAIAT QNHPRNEDTI VAAAQPVKPM RAPSTRRKGG
TCVAGTFRSA LDTLFETLEE TQTWYVFCIN PNDSQLPNQI EGRAVKGQTR SMGLTEISRR
CVNVFEVGMT PREFCDRYRE QIAAVGISEG GNDLVLGQYK VFLSQRAFHK LEDYLRAKDV
DEQKRNRMRE AEISTGALPL VSNASPFDDF DANSRLTSHR ESYAPSRNMF QNADGEVQEG
ETTEVIKETS ARRRWVALCW MLTFWVPTPF LRWFGRMKRL DVQQAWREKL ALNILIWFIC
GCAVFVIAVL GNLICPTEHV FNTSELSSHS DSVYTSIRGE YGGTSSDDIF PVQVSALCNG
IDGSVNPYVA LQFSNTDTNA QYHDFRVSSN DSRPDWYFES MTVMRWNNRV GFVGYTPKEL
KSKAAAGSSL GIYNGL
>CP

MRRTTQDQSL LISGETGSGK SENRRLAIKT LLELSVSNPG KKGSKLASQV PAAEFVIESF
GNARTLFNPN ASRYGKYTEL QFTRGRLCGV KTLDYYLERN RVAAVPSGER NFHIFYYLVA
GATTEERQHL RLVDKAAYRY LGQRDANRFE QLKGALKSIG FSKRSVAQTC QLLAAILHLG
NLEFTIDRGR DVDAAVVRNT DTLAIVAEFL GVQPAALEAT LSYKTKMVKK ELCTVFLDTD
GASDNRDDLA KTLYSLLFAW LNEHINSRLC RDDFDTFIGL VDLPGPQNMS RPNSLDQFCI
NFANERLQNF IQKQIFEYHV TEYTSEGVAE YVPTVPYFDN SECLRLLQNQ PGGLIHIMDD
QARRSQKKTD QSMVEAFGRR WGNHSSFKVG SMDRFPTFTV NHFNGPVTYS AEGFLERNLD
ALNPDFVSLL RGGSVNPFVK GLFTGKAIAT QAHPKDEDTI VAAQQTIKPM RKPSTRRKGT
PCVAGEFRSA LDTLFETLGD TQNWFVFCVN PNDSQLPNQI EGRSVKGQVR SACIAEVARR
NVTVFEAGMT PEEFVDRYRE ALTAIGIGAA ERDLVTGTYK VFLTHAAFHA LENHLRSLDT
EEQKRNRMRD AEASQQNLPL VQHAAGGDDY DARSRLTSNR ESYAPSRNMF QNADGEVAEG
ETTEVVKETS ARRRWVMLTW MLTFWVPTPF LTWFGRMKRP DVRQAWREKL AINMLIWFIC
GCAVFVIAIM GLIICPTEHV YSTSELQSHS NNVYTSIRGE YGGTDASDIF PVQVSALCSG
EGQGVSPWVQ LNSGNSDPNA QYHDFRAATN DSRPDWYFES MTVMRWNNRV GYMGYTPKEI
SSLANSGSSV GIIDGL
>FS
MRRTTQDQSI LLSGETGSGK SENRRLAIKA FLELSVSNPG KKGSKLAAQV PASEFVLESF
GSARTLFNPN ASRFGKYTEL QFSRGRLTGI KTLDYYLERN RVCGAPNGER NFHIFYYLVA
GASPEERQHL HLGDKTTYRY LGARDANRFD QLKVALKAIG LSKRHVAQTC QLVAAILHLG
NLEFMIDRGK NEDAAVVRNT DILEIVAEFL GIQAHDLESA LSYKTKLVKK ELCTVFLDPD
GASDNRDDLA KTLYSLLFAW LNEHINQRLC RDDFATFIGL FDLPGPQNMS RPNSLDNFCM
NFANERLQNW IQQRVFDSQT EEYAAEGISR FVPTVPYFDN AECIRLLQNK PGGLIHIMDD
QTRRAPKKTD QTMVEAFGKR WGNHSSFKVG SADRFPTFTV SHFNGPVTYA SDGFLARNAD
ALNPDFVSLL RGGSMNPFVK GLFSGKAIAT QAHPRNEDTI VSAQQPVKPM RAPSTRRKGT
PCVAGEFRSA LDTLFETLNE TQAWLVFCLN PNDSQMPNQI EGRSIKGQVR SAGLPEVAKR
SVCVFEVGME PEEFCDRYRL ALNDLGIIEG DEDVVMGAHK VFLSQMAFHA LEDRLRGTDV
EEQKRNRMRD AEASSQALPL VANASPFDDF DNRSRLTSQH ESYAPSRNMF QNTDGEIHEG
EVTEIVKETA NRRKWVALCW ALTWYVPNFA LTHIGRMKRL DVRQAWREKL AINIIIWFVC
GCAIFVIAFL GDVICPTEHV FSAAEFASHN TAPYTSIRGE YAGVPSDNIF PVQVSALCNG
VTGTVSPYVT LSSKNTDVNQ VYHDFRAYTL DPRPDWYFEQ MTQMRWNNRV GFLGITTKLM
KSMASAGSSI GIYNGL
>PI
MRRTSQDQSV LLSGETGSGK SESRRLAIKA LLELSVSSPG KKGSKLAAQV PASEFVLESF
GNARTLFNPN ASRFGKYTEL QFSRGRLSGI KTLDYYLERN RVAGAPSGER NFHIFYYLVA
GASPEERQHL HLLDKSVYRY LGSQDANKFD QLKVALKTIG LSKRHVAQTC QLVAAILHLG
NLEFMVDRSR NEDAAVVRNI DILEIVADFL GVQPYALEAA LSYKTKLLKK ELCTVFLDPD
GASDNRDDLA KTLYSLLFAW LNEHINQRLC RDDFATFIGL FDLPGPQNMS RPNSLDHFCI
NFANERLHNW IQRRLFESHV DEYAAEGISR FVPTVPYFDN AECIRLLQNK PGGLIHIMDD
QARRAPKKTD QTMVEAFAKR WGNHSSFKVG TLDRSSTFTI NHFNGPVTYS SEGFLERNLD
ALNPDFVSLL RGGSINPFVK SLFSGKAIAT QAHPRNEDTI VAAQQPVKPM RAPSTRRKGT
PCVTGEFRSA LDTLFETLNE TQAWFVFCIN PNDSQLPNQL EGRSVKGQVR SAGLPEIARR
CVNTFEVNMT PEEFCERYRA PLVELGVIEG ARDVVLGQHK VFLSQAAFHG LEDHLRSSDT
EEQKRNRMRD AEASNQVLPL VANASPFEDY DGRSRYTTHR ESYAPSRNMF QNADGEIQEG
ETTEVMKETS AHRKWVALCW MLTFWVPSFL LKWWGRMKRP DVRQAWREKL ALNMLIWFIC
ACAVFVIAVL GDVICPTQHV FSTSELASHS NNVYTSIRGE YGGTSADNIF PVQVSALCNG
VTGSVSPYVT LDSSNTDVNA QYHDFRAFTA DSRPDWYFES MTEMRWNARV GYVGYTPKEL
KNMANTGSSV GIIDSL
>SL
MRRTSQDQSI LLSGETGSGK SENRRLAIKT LLELSVSNPG KKGSKLAGQV PAAEFVIESF
GNARTLFNPN ASRFGKYTEL QFTKGRLCGI KTLDYYLERN RVAGAPSGER NFHIFYYLVA
GATAEERQHL HLLDKTNYRY LGSRDANRFD QLKVALKSIG LSKRHVAQTC QLVAAILHLG
NLEFTVDRSR NEDAAVVRNV DILDVVSEFL GVQPSALEAA LSYKTKMVKK ELCTVFLDPD
GASDNRDDLA KTLYSLLFAW LNEHINQRLC RDDFDTFIGL FDLPGPQNMS RPNSLDQFCI
NFANERLQSF TQKRLFESHV DEYTNEGISR FVPTVPYFDN SECVRLLQNK PGGLIHIMDD

QARRAHKKTD HSMVEAFGKR WGNHSSFKMG SLDRFPTFTV NHFNGPITYS AEGFLERNLD
ALNPDFVSLL RGGSINPFVK GIFSGKAIAT QAHPKNEDTI VSAQQPIKPM RAPSMRRKGT
PCVAGEFRAA LDTLFDTVAE TQAWYVFCVN PNDSQLPNQL EGRSVKGQVR SAGLSEIARR
NVNVFEVGMT PEEFCDRYKE PMGAMGIMSS ERDVVLGNFK VFLSGVAFHK LEDQLRSRDV
EEQKRNRLRD AEASKEGLPL VANASPFEDF DGRSRLTSNR ESYAPSRNMF QNLDGEIQEG
ETAEVVKESS ARRKWVMLCW MLTWWVPSPF LKWFGRMKRE DVRQAWREKL ALNIIIWFVC
GCAVFVIAVL GLIICPREYV FSASELESHS NNVYTSIRGE YGGVAADNIF PVQVSALCNG
VSGTVSPWVI LDSSNTDPNS QYHDFRAWTN DSRPDWYFES MTMMRWKNRV GYSGYTPQEI
GNLASSGKSI GIYNNL
>RM
MRRTGQDQSI LFSGETGSGK SENRRLAIKS ILELSVSNPG KKGAKLSTQV PSAEFVLECF
GNARTLFNSN ASRFGKYTEL QFTRGRLCGI KTLDYYLERS RVAGAPSGER NFHVFYYLVA
GASAEEKQHL GLSDKANFRY LGARDAVRFD QLKVALKNIG MSKRHVAQTC QLLAAILHLG
NLEFVIDRRR NEDSAVVKNI DALELVADFL GVQPTALETA LSCKVKLVKK ELCTVFLDPD
GASDNRDDLA KMLYSLLFAW LNETINQKFC RDDFTTFIGL FDLPGPQNMS RSNSLDQFCV
NFANERLHNW IQRRMFEMHV AEYNSEGIAR FVPEVPYFDN SECVRLLSNM PGGLIHIMDD
QARRAPKKTD HSMVEAFGKR WGNHSSFKQG GMDRFPTFTV NHFNGPVTYS SEGFLERNLD
ALNPDFVSLL RGGSVNPFVR SLFSAKAIAT QAHPRNEDTI VAAQQPQKPM RQPSMRRKGT
PCIAGEFRAA LDTLFQTLDE TQAWLVFCVN PNDAQMPNQL EGRAVKGQVR SSGLSEVARR
CVNVFEVNMS PAEFCDRYRD FLAVLNVHEG ERDVVVGQHK VFLSQLAFHK LEDYLRSTDT
DEQKRNRMRD AEGSSQQLPL VANASPFDDI DGKSRLTSHR ESYAPSRNMF QNADGEVMEG
ETTEDIKETS TRRRWVAFVW ILTWWVPNPF LRWFGRMKRL DVRQAWREKL AINIIIWFLC
LSVAFVIAVL GNLICPTEHV FTVAELNSHS NNVYTAIRGE YGGTKADNIF PVQVSALCNG
IDGQVNPYVV LDSVNTDINA QYHDFRVFTN DSRPDWYFQA MTIMRWGYRV GFMGYTPKEL
RNMANSGSTV GIYNGL
>SP
MRRTGQDQAI VLSGETGSGK SENRRLAIRS LIELSVSNPG KKGSKLSSQI PSAEFVLESF
GNARTLFNPN ASRFGKYTEL QYTRGRLCGV KSLDYYLERS RVAGAPSGER NFHIFYYLTA
GATAEERQHM RLTDKTHFRY LGPRDSVRFE QLKVALKSVG LSKRQVAQSC QLVAAILHLG
NLEFITDRQR NEDAAVVRNT DTLDTVAEFL GVQPAALETA LSCKMKLVKK ELCTVFLDPD
GASDNRDDLA KILYSLLFSW LNESINQKLC RDDFTTFIGL FDLPGPQNMS RANSLDQLCV
NYANERMHHW IQKRLFESHI EEYKVEGISR YVPSVPYFDN AECVRLMSNM PGGLIHIMDD
QARRAPKKTD QTMVEAFGKR WGNHSSFKVG GMDRFPTFTV NHFNGPVTYS SEGFLEKNLD
SLNPDFVSLL RGGSANPFIR SLFSNKAIAT QAHPRNEETI VSAQQSMKPM RAPSTRRKGT
GCIAGEFRSA LDTLFSTLDE AQAWFVFCIN PNDSQLPGQL EGRAVKGQVR SAGLPEIARR
CVNVFEVNML PTEFCDRYKD QLSALNIHEG ERDVVQGQRK VFLSQRAFHK LENHLRVNDA
DEQKRNRTKD TEGSSQNLPL VNHASPFDDI EGKSRLTSRP ESYAPSRNMF QNEDGEVLEG
ETAEDIKESS NRRRWVAFVW MLTWWIPNIA LVWCGRMKRL DVRQAWREKL ALNMLIWFFC
GCAVFVIAVL GNLICPTEHV FSVTELASHS NNIYTAVRGE YGGLDATNIF PVQVSALCNG
VTGSVNPYVT LDFTNTDPNA QYHDFRVSTS DSRPDWYFES MTLMRYNYRV GFVGYTPKEL
KNMANSGRSV GVYNGL
>RF
MRRTSQDQSI LLSGESGSGK SENRRLAIKS LLELSVSNPG KKGSKLSSQI PSAEFVLESF
GNARTLFNPN ASRFGKYTEL QFTRGRLCGI KTLDYYLERS RVAGAPSGER NFHIFYYLVA
GATLEERQHL HLTDKMTYRY LGQRDALRFD QLKLALKSIG LSKRHVAQSM QLLAAILHLG
NLEFTIDRHR NEDAAVVRNT DILDIVSEFL GVQPAALETA LSCKMKLVKK ELCTVFLDAE
GASDNRDDLA KMLYSLLFAW LNESINQRLC RDDFATFIGL FDLPGPQNMS RSNSLDQFCI
NFANERLQNW IQKRMFEFHV EEYASEGISR FVPQVPYFDN SECVRLLSNM PGGLIHIMDD
QARRMPKKTD HTMIEAFGKR WGNHSSFKVG AMDRFPTFTV NHFNGPVTYS AEGFLEKNLD
ALNPDFVSLL RGGSINPFVR SLFSAKAIAT QAHPRDEDTI VAAQQPVKPM RAPSTRRKNT
PCVAGEFRKA LSTLFTTLDE TQPWFVFCVN PNDSQLPNQL EGRAVKGQVR SSGLSEIARR
SVNVFEANMM FAEFCERYGD QLAALNIVEG ERDVVIGQHK VFLSQAAFHK FEDHLRAGDV
EEQKRNRLRD AEASSYTLPL VQHAASFDFD DGKSRLTSHR ESYAPSRNMF QTTDGEVLEG
ETTEVIKESS GRRRWVALCW MLTWWIPSPL LTWVGRMKRL DVRQAWREKL ALNMIIWFVC

LSTAFVIAVL GDLICPTEHI FNTSELAGHS NNVFTSIRGE YGGTSADNIF PVQVSALCNG
VSGSVNPYVI LDSSNTDPNS QYHDFRVSST DPRPDWYFES MVLMRWNYRV GFVGYTSKEL
KSMANTGRSV AVYNGL
>RI
MRRTSQDQSI LLSGESGSGK SENRRLAIKS LLELSVSNPG KKGSKLSSQI PSAEFVLESF
GNARTLFNPN ASRFGKYTEL QFTRGRLCGI KTLDYYLERS RVAGAPSGER NFHIFYYLVA
GATLEERQHL HLTDKMTYRY LGQRDALRFD QLKLALKSIG LSKRHVAQSM QLLAAILHLG
NLEFTIDRHR NEDAAVVRNT DILDIVSEFL GVQPAALETA LSCKMKLVKK ELCTVFLDAE
GASDNRDDLA KMLYSLLFAW LNESINQRLC RDDFATFIGL FDLPGPQNMS RSNSLDQFCI
NFANERLQNW IQKRMFEFHV EEYASEGISR FVPQVPYFDN SECVRLLSNM PGGLIHIMDD
QARRMPKKTD HTMIEAFGKR WGNHSSFKVG AMDRFPTFTV NHFNGPVTYS AEGFLEKNLD
ALNPDFVSLL RGGSINPFVR SLFSAKAIAT QAHPRDEDTI VAAQQPVKPM RAPSTRRKNT
PCVAGEFRKA LSTLFTTLDE TQPWFVFCVN PNDSQLPNQL EGRAVKGQVR SSGLSEIARR
SVNVFEANMM FEEFCERYGD QLAALNISEG ERDVVIGQHK VFLSQAAFHK FEDHLRAGDV
EEQKRNRLRD AEASSYTLPL VQHAASFDFD DGKSRLTSHR ESYAPSRNMF QTTDGEVMEG
ETTEVIKESS GRRRWVALCW LLTWWIPNPI LRWVGRMKRL DVRQAWREKL ALNMIIWFVC
LSTAFVIAVL GDLICPTEHI FNTSELAGHS NNVFTSIRGE YGGTSADNIF PVQVSALCNG
VSGSVNPYVI LDSSNTDPNT QYHDFRISST DPRPDWYFES MVLMRWNYRV GFVGYTSKEL
KTMANAGRSV AVYNGL
>FM
MRRTQQDQCI LLSGETCSGK SENRRLAIKS IIELSVSNPG KKGSKLSTQI PSAEFVLESF
GNARTLFNPN ASRFGKYTEL QFTRGRLSGV KTLDYYLERG RVAGAASGER NFHIFYYLVA
GASAEERQHL KLTDKSTFRY LGPRDAMRFD QLKVALKNVG LSKRVVAQTC QLLAAILHLG
NLDFIIDRQR NEDAAVVKNT DILETVAEFL GVQPQALENA LSCKMKLMKK ELCTVFLDPD
GASDNRDDLA KILYSLLFSW LNEQINQKLC RDDFSTFIGL FDLPGPQNMS RSNSLDQFIV
NFANEKLHSW IQKRMFESHV DEYNQEGIAH YVPPIPYFDN AECVRLLSHM PGGLIHIMDD
QARRMPKKSD HTMVEAFGKR WGNHSSFKVG GIDRFPTFTV NHYNGPVTYS SEGLLEKNLD
ALNPDFVSLL RGGSVNSFIR NLFCGKAIAT QAHPRNEETI VAAQQSVKPM RAPSTRRKGT
PCTAGEFRQA LVTLFSTLDE TQAWYVFCIN PNDSQIPLQL EGRAVKGQIR SAGLTEIAKR
NANVFEVSMT PSEFCDRYGA HLLALNIHEG ERDLVIGSFK VFLSHRAFHK LEDRLRAEDV
EEQKRNRLRD AEASSQQLPL VSHASPFDDL DAKSRLTSNR ESYAPSRNMF QNDDGEVMEG
ETTEDIKESS SRRRWVAFVW LLTWWCPNIF LIWCGRMKRL DVRQAWREKL ALNMLIWFCC
LVAAFIIAVL GNLICPTEHV FSSSELQAHN NSMLTAIRGE YAGTTADDLF PVQVSAVCNG
VSGSVNPFVT LNSKNTDPNA QYHDFRAWKN DSRPDWYFES MTLMRWNYRV GFVGLTGKQV
KNLATSGRAV AIYNGL
>PN
MRRTQQDQCI LLSGETCSGK SENRRLAIKS IIELSVSNPG KKGSKLSTQI PAAEFVLETF
GNARTLFNPN ASRFGKYTEL QFTRGRLSGV KTLDYYLERS RVAGAPSGER NFHIFYYLVA
GATAEERQHL RLTDKASFRY LGPRDGVRFD QLKVALKSVG LSKRSVAQTC QLLAAILHLG
NLEFIIDRQR NEDAAVVKNT DVLETVAEFL GVQPQALENA LSCKMKLVKK ELCTVFLDPD
GASDNRDDLA KILYSLLFSW LNENINQKLC RDDFTTFIGL FDLPGPQNMS RSNSLDQFCV
NFANERMHNW IQRRMFEIHV DEYNQEGITR YVPSVPYFDN SECVRLLTNM PGGLIHIMDD
QARRMPKKTD HTMVEAFSKR WGNHSSFKVG GMDRFPTFTV NHFNGPVTYS SEGLLEKNLD
AVNPDFVSLL RGGSVNPFIR GLFTGKAIAT QAHPRNEDTI VAAQQTVKPM RAPSTRRKGT
PCTAGIFRTA LETLFSTLDE AQAWFVFCIN PNDSQLPLQL EGRAVKGQIR SAGLTEIAKR
CANVYEVNMT TSEFCDRYKA QIATLNVFEG ESDIVLGNYK VFLSQRAFHK LEDRLRAEDV
EEQKRNRIRD AEASSQQLPL VANASPFDDM DAKSRLTSNR ESYAPSRNMF QNDDGEVLEG
ETTEDIKESS ARRRWVAFVW LLTWWCPNIF LIWCGRMKRL DVRQAWREKL ALNLIIWFIC
LAAAFVIAVL GNLICPTEHV FSQSELASHD GNMYTSIRGE YAGTSSDDLF PIQVSAVCNG
IDGSVDPLVT LNSKNTDTYT QYHDFRAWQN DSRPDWYFES MTYMRWNYRV GFLGLTSKEV
SSQAKNGKAV AIYNGL
>OS
MRRTQQDQCM LLSGETCSGK SESRRLAIKS IIELSVSNPG KKGSKLSTQI PSAEFVLESF
GNARTLFNPN ASRFGKYTEL QFTRGRLSGI KTLDYYLERS RVAGAPSGER NFHIFYYLVA

GATAEERQHL KLTDKSTFRY LGPRDAVRFN QLKVALKSIG LSKRVVAQTC QLLAAILHLG
NLEFIVDRQR NEDAAVVRNI DILETVAEFL GVQPQALENA LSCKMKLVKK ELCTVFLDPD
GASDNRDDLA KILYSLLFAW LNESINEKLC RDDFSTFIGL FDLPGPQNMS RSNSLDQFCV
NFANERLHHW VQKRMFDSHV DEYNQEGLSR FVPSVPYFDN AECVRLLSNM PGGLMHIMDD
QARRMPKKTE HTMVEAFGKR WGNHSSFKVG GIDRFPTFTV NHYNGPVTYS SEDILEKNLD
ALNPDFVSLL RGGSVNPFIR SLFSGKAIAT QAHPRNEDTI VAAQQPVKPM RAPSTRRKGT
PCVAGEFRAA LDTLFSTLDE SQAWFVFCIN PNDAQMPNQL EGRAAKGQIR SAGLTEVARR
NVNVFEANMA PSEFCNRYHA QLSALNIHEG ERDIVLGSTK VFLSQRAFHK LEDRLRAEDV
EEQKRNRLRD AEASNQQLPL VANASPFDDM DGKSRLTSNR ESYAPSRNMF QNGDGEILDG
ETTEDIKESS TRRRWVAFVW LLTWWCPNIF LIWCGRMKRL DVRQAWREKL AINIIIWFIC
LCAAFVIAVL GNLICPTEHV FSTSELASHN NSAYTSVRGE YAGTTADDIF PVQVSALCNG
VSGSVNPYVS LNSKNTDPNS VYHDFRAWKN DSRPDWYFES MTLMRWNYRV GFLGLTGKEV
KNKANDGNSI AIYDGL

>TA

MRRTTQDQSI VLSGETGSGK SENRRLAIKS LIELSVSNPG KKGSKLSSQI PSAEFVLESF
GNARTLFNPN ASRFGKYTEL QFTRGRLCGV KTLDYYLERS RVAGAPSGER NFHIFYYLAA
GATAEERQHL RLTDKAHFRY LGPRDAMRFD QLKVALKSVG LSKRMVAQTC QLVAAILHLG
NLEFIVDRHR NEDAAVVKNT DVLDIVAEFL GVQPQALETA LSCKMKLVKK ELCTVFLDPD
GASDNRDDLA KILYSLLFSW LNESINQKLC RDDFSNFIGL FDLPGPQNMS RSNSLDQFCV
NFANERLHGW IQKQMFEAHV DEYNQEGISR FVPTVPYFDN AECVRLLSNM PGGLIHIMDD
QARRAPKKTD HSMVDAFGKR WGNHSSFKVG GMDRFPTFTI NHFNGPVTYS SEGFLEKNID
ALNPDFVSLL RGGSVNPFIR SLFSNKAIAT QAHPRNEDTI VAAQQPVKPM RAPSTRRKGT
PCVAGEFRSA LNTLFTTLDE TQAWNVFCIN PNDSQLPNQL EGRAVKGQVR SAGLSEIARR
CVNIFEVNMT LEEFCERYRD QLSALNIHEG EKEIVIGQHK VYLSQRAFHK LEDRLRGEDM
EEQKRNRLRD AEASSQQLPL VANASPFDDI DGKSRLTSNR ESYAPSRNMF GNTDGEVLEG
ETTEDIKESS ARRRWVAFVW LLTWWCPNIF LIWCGRMKRL DVRQAWREKL ALNMIIWFMC
LCTAFVIAVL GALICPTEHV FSTNELSSHS NNVYTAVRGE YGGTTADSIF PVQVSALCNG
VSGSVNPFVT LDSSNTDPNA QYHDFRAFTS DSRPDWYFES MTLMRWNFRV GFVGYTMKEV
KQMANSGRSA AIYNGL

>SS

MRRTGQDQSI ILTGETCSGK SENRRLAIKA ILELSVSNPG KKGSKLSQQI PSAEFVLETF
GNARTLHNPN ASRFGKYTEL QFTRGRLCGV KTLDYYLERN RIAGALSGER NFHIFYYLVA
GASAEERTHL KLAEKTAYRY LGHGDSQRFD QLKQALKSVG LSKRHVAQTC QLLAAIIHLG
QLDFTIDRYR NQDAAVVRNL DVLQIVAEFL GVQPSALESV LSYKTTMVKK ELCTVFLDAE
GASDNRDNLA KTLYSLLFAW LNESINQKLC REDYNTFIGL FDLPGPQNLS RSNSLDQFCI
NYANERLQNW IQRELFENYT EEYNIEGIAR FVPKIPYFDN SECIRLITNK PGGLIHIMDD
QARRQPKKTD HTMVEAFSKR WGNHSSFKVG TMDRFPTFTV NHFNGPVTYS AESFLERNCD
AVNPDFVSLL RGGSINPFVR GLFSAQAIAT QAHPKDEDTI VAAQQPVKPM RAPSTRRKKS
PGVAGEFRAA LDTLFETIDE TQSWFVFCIN PNDAQLPNQL EGRGVKIQIR NLGLPEIAKR
SAVVFEANMT PAEFCDRYRE QIAAVGIIEG EEDIVVGQFK VFLSHVAFHK LEDYLRANDA
EETKRNRIRE AEASNQQLPL VAHASPFSDY DGRSQLTSAR ESYAPSRNMF QNAEGEILEG
EVTEEFRESS ARRRWLFIVW TLTFWCPNFL LSYVGRMKRL DIRQAWREKL AINMIIWFLC
ACAAFVIAVL GVVICPTSHV FSMSELQDHS NNAFTSIRGE YSGLTADNIF PVQVSALCNG
VSGSVNPYVV LNSDNTDPNA QYHDFRVSTN DPRPDWYFEQ MVQMRFLYRM GFLGFQPNEV
SNMADQGKAV GIYNGL

>SN

MRRTGQDQGI LISGESGSGK SESRRLIIKS LLELSVSNPG KKGSKLSNQI PSAEFVLEAF
GNARTLSNPN ASRFGKYTEL QFTRGRVSGV KTLEYYLERS RVTGAPSGER NFHIFYYLIA
GASTEERQHL HLHDKQAFRY LGQRDGLRFE QLKMAMKSVG LSKRSVAQMC QLIAAILHLG
NLEFTIDRHR NLDAAVVKNV DVLDIVADFL GVQPSALEAV FSYKTKLVKK ELCTVFLDPE
GAAENRDALA QTLYSLLFSW VNENINQRLH RDDFSSMIGL LDLPGPQNIS RSNSLDQFCV
NYANEKLQNW IQKSLFESHV EEYSAEGISN YVPQVPYFDN SECVRLLENR PGGLIHIMDD
QARKMPKKTE HTMVEAFGKR WGNHSSFKIG NMDRFPSFTV NHYTGGVTYS AEDFLEKNLA
ALNPDFVSLL RGGSINPFVR GLFSSKAIAT QAHPRNEDTI VAAQQPVKPM RAPSTRRKGT

ACKAGEFRAA LEILFQTFDE TQPWFVFCLN PNDSQLPNQL EGRGVKAQIR SIGLSEIARR
NRTVFEVSMT PSEFCERYRD QLALLGVEDG EWDLVQGQYK VFLSHLAFHK LEDHLRATDV
EEQKRNRLRD AEASSQQLPL VANAAPFEDF DGVSRYTSHR ESYAPSRNMF QNADGEIQEG
EVTEEIRDTS TRRRWLVLVW FLTFWLPNPF LSWFGRMKRL DVRQAWREKL AINMVIWFIC
GCAVFVIAVL GNVICPREFV FGTDELASHS NHIYTAIRGE YGGLDATDIF PVQVSALCDG
TDGNLSPYIV LDATNTDVNA QYHDFRVSTT DPRPDWYWEN MVILRYHYRV GFMGYTPSTL
RDMASAGLAV GVYDGL
>AS
MRRTGSDQSI ILSGDTASGK SENRRLAIKS ILELSVSNPG KKGSKLAVQL PAAEFVLESF
GNARTLFNAN ASRFGKYTEL QFTRGRLCGV KTLEYYFERN RAAGAPSGER NFHIFYYLLA
GASPEERQHL KLSEKTTFRY LGQRDALRFE QLKGALKNVG MSKRHVAQAC QLVAAILHLG
NLDFTIDRHR NEDAAVVRNT DVLEIVADFL GVDSAALEAV LSYKTKLVKK EVCTVFLDPD
GASDNRDDLA KMLYSLLFSW LNESINQRFC RDDFSTFIGI FDLPGTQNLS RSNSLDQFCV
NFANEKLHQF IQRSIFEKHT DEYANEGISR FVPSVPFFDN SECVRLLCNK PGGLIHIMDD
QARRMQKKTD HTMVEAFGKR WGNHSSFKVG AMDRFPTFTI NHYNGPVTYS SESFLERNLD
ALNPDFVSLL RGGSINPFVR GLFSSKAIST VAHPRNEDTV VAAQQPQKPM RAPSTRRKGT
PCAAGQFQNA METLIQTLDD TQPWYVFCIN PNDSQLPNQF EGRSVKAQVR CAGLPEIARR
AAIVFEVSMT PAEFCVRYKD PLVALNLSEG EHDVVLGQFK VFLSHAAFHK FEDRLRATDV
EEQKRNRLRE MEASSQALPL VAHAQPFSDD DGQSRYTSHR ESYAPSRNMF QNFEGEVMEG
EVAEEIHDSS ARRRWLALVW LLTWWLPTPF LSWFGRMKRM DVRQAWREKL AINLIIWFLC
GCTVFVIAVL GNLICPREYV FNADELSSHA KHVYTAIRGE YGGQDIARLF PVQVSALCNG
INGRVSPWLT LDPVNIATDS QYHDFRAWSS DYRPDWYFEQ MTVMRYKYRV GFVGFTNTVL
RDMAGAGRIV AVYNNI
>EG
MRRTGQDQSI LFSGDTASGK SENRRLAIKS ILELSVSNPG KKGSKLATQL PAAEFVLESF
GNARTLFNSN ASRFGKYTEL QFTRGRLCGV KTLEYYFERN RAAGAPSGER NFHIFYYLLA
GASPEERQHL KLSDKATFRY LGPRDALRFD QLKGALKNVG MSKRHVAQTC QLVAAILHLG
NLEFTIDRHR NEDAAVVRNT DVLEIVADFL GVDPAALEAV LSYKTKLVKK EVCTVFLDPD
GASDNRDDLA KMLYSLLFSW LNESINQRFC RDDFVTFIGI FDLPGTQNLS RSNSLDQFCV
NFANEKLHHW IQRSIFEKHT DEYAKEGISR FVPTVPFFDN SECIRLLSNK PGGLIHIMDD
QARRMPKKTD HTMVEAFGKR WGNHSSFKVG PMDRFPTFTV NHYNGPVTYS SESFLERNLD
ALNPDFVSLL RGGSINPFVR DLFSNKAIST VMHPRNEDTV VAAQQPQKPM RAPSTRRKQT
PCTSGQFQNA METLIQTFDD TQPWYVFCIN PNDSQLPNQF EGRSVKAQVR CSGLPEIARR
SGIVFEVSMT PAEFCARYAE CMTAWGVTGG DHDLVVGQFK VFLSHAAFQK FEDRLRATDV
EEQKRNRMRE MEASSQQLPL VAHAAPFSDD DGHSRYTSNR ESYAPSRNMF QNFDGEVMEG
EVTEEIHDTS ARRRWLAVVW LLTWWLPTPF LTWFGRMKRL DVRQAWREKL AINIIIWFMC
GCTIFVIAVL GNLICPREYV FNADELSSHS NHVYTAIRGE YGGQDISRLF PVQVSALCNG
ITGSVSPWLT LDTINIAVDA QYHDFRAWST DYRPDWYFEQ MTVMRYKYRV GFVGYTTQLL
RDMAAAGRTV AIYNNI

02-GRMA domain

>SS
SLPAVMFTSS SSTFLTFKPK ESLHFTFLTI GSRGDVQPYI ALGKRLLQEG HNIEYAYVGG
DPAELMRICV ENGMFTVSFL KEGATKFRGW IDDLLKTAWE ACQGTDVLVE SPSAMGGIHI
AEALKIPYFR AFTMPWTRTR AYPHAFAVPE HKTYVMFDQV FWRGTSGQIN RWRRKVLGLP
STNLDKLEQH KVPFLYNFSP AVVPPSLDWY EWIRVTGYWF LDDADVGARK WTPPDGLVEF
IDSAHAAGKK VVYSDPGAMT KCVVEAILKS GVYAILSKGW SDRLQPKESY PPQIYPISSI
PHDWLFPGIP TIIKPFFGDQ YFWADRMEAL GIGSCVRKLT IDHLTAALIT ATTDERQISR
ARAIGQTIRA EDG
>CC
SLPAVMFTSA SSTFLTFKPP KPLHFTFLTI GSRGDVQPYI ALAKGLIADG HGIEYGCVGG
DPAELMRICV ENGMFTVSFL KETLQKFRGW LDDLLQTSWE ACQGTDILIE SPSAMGGYHI
AEALGIPYFR AFTMTWSRTR AYPHAFAVPE RKSYVMFDQV FWRATAGQIN RWRRHTLGLG
ATSLDRMEPH KIPFLYNFSP TVVPPPLDWP EWIRITGYWF LDDADVGSKK WTPPDSLVEF

IDNAHQSGKK VVYSDPRSMT KCVIDAVVQS GVHAILSKGW SDRLEPEEPL PPQIYPITSA
PHDWLFAGVP TIIHPFFGDQ FFWADRVEAL GVGTGVRKLT VSALKDALVS ATTDQKQIDR
AKLVGEQIRR VHF
>SL
SLPAVMFTSA SSTFLTFKPQ KPLHFTFLTI GSRGDVQPYI SLARGLMADG HGIEFGYVGG
DPAELMRICV DNGMFTVSFL KEGVQKFRGW LDDLLKTSWD ACQGTDVLIE SPSAMGGIHI
AEALQIPYFR AFTMTWTRTR AYPHAFAVPE HKSYVMFDQV FWRATSGQIN RWRRNVLHLG
STSLDKMEPH KIPFLYNFSP HVVPPPLDWP EWIRVTGYWF LDDAEVGAKK WVPPPDLIPF
IDSAHQAGKK VVYSNPQAMT RCIIEAIVQS GVYAILSKGW SDRLEPEEPL PKQIYAISSI
PHDWLFAGIP TIIRPFFGDQ FFWADRVEAL GIGSGVRKLT VESLTEALRS ATTDVKQIDR
AKLVGEHIRA PLL
>DS
SAPAYMFTSQ SSTFLSFKPE KSLHFTFLTI GSRGDVQPYI ALAKGLKADG HGIEFGYVGG
DPAELMRICV ENGTFTVAFL KEGVQSFRGW IDDLLKTSWE ACQGTDVLIE SPSAMAGYHI
AEALKIPYFR AFTMTWSRTR AYPHAFAVPE HKTYVLFDQV FWRGTAGQIN RWRKHTLGLP
GTSLDKMEPH RIPFLYNFSP TIVPPPLDWP EWIRITGYWF LDAADVGSKK WEPPQDLLDF
IDAARKANKK IVYPDPKAMT RCVIDAIVQS GVHAILSKGW SDRLEPEEPL PKQIYPISSI
PHDWLFAGIP TIIKPFFGDQ FFWGDRVEAL GIGAAVRKLT VESLSQALRE ATTNQKVIDR
AKLVGEQIRA ENG
>TV
AAPAHMFTSQ SSTFLSFKPD KSLHFTMLTI GSRGDVQPYI ALAKGLMADG HGIEFGYVGG
DPAELMRICV ENGTFTVAFL KEGVAKFRGW IDDLLKTSWE ACQGTDVLIE SPSAMGGYHI
AEALKIPYFR AFTMTWTRTR AYPHAFAVPE HKTYVLFDQV FWRGTAGQIN RWRRNTLGLP
GTSLDKMDPH KIPFLYNFSP IIVPQPLDWP EWIRVTGYWF LDDADVGSKK WEPPQSLLDF
MAEARKAKKK IVYPDPKTMT RCVIDAIVES GVYAIMSKGW SDRLEPEEPL PKQIYPISSI
PHDWLFAGIP TIIKPFFGDQ FFWADRVEAL GVGAAVRKLT VDVLAQALRD ATTNQKQIDR
AKAVGEQIRA ENG
>FP
SLPAVMFNST SSTFLTFKPD KSLHFTFLTI GSRGDVQPYI ALAKGLMKDG HGIEFGYVGG
DPAELMRICV ENGTFTVAFL KEGMLKFRGW IDDLLKTSWD ACQGTDVLVE SPSAMSGYHI
AEALGIPYFR AFTMTWTRTR AYPHAFAVPE HKSYVLFDQV FWRATAGQIN RWRRNTLGLG
GTSLDKMEPH KIPFLYNFSP VIVPPPLDWP EWIHVTGYWF LDDADVSSKK WSPPQDLIDF
LDSARKAQKK VVYSDPKSMT RCVIEAIVRS GVYAILSKGW SDRLDPEEPL PKQIYPIASV
PHDWLFGGIP TIIKPFFGDQ FFWADRVEAL GIGTAVRKLT VESLTQALIT ATTDQKQITR
ARYVGEKIRA EDG
>PP
SLPAVMFNST SSTFLTFRPT QSLRFTFLTI GSRGDVQPYI SLAKGLMKDG HGIEFGYVGG
DPAELMRICV ENGTFTVAFL REGMLKFRGW VDDLLQTSWE ACQDTDVLIE SPSAMSGFHI
AEALKIPYFR AFTMTWSRTR AYPHAFAVPD HKSYVLFDQV FWRATAGQIN RWRRNTLGLP
STSLDKMEPH KIPFLYNFST KVVPRPIDWP EWIHVTGYWF LDDAEVSSKK WAAPPDLIEF
LENARKESKK IVYSDPKAMT RCVIDAVVRS GVYAILSKGW SDRLEPEEPL PKQIYPVASI
PHDWLFAGIP TIIRPFFGDQ FFWGDRVEAL GVGTALRKLT VESLTQALIA ATTDQKQIER
ARELGEKIRA EDG
>WC
SLPAVMFNSA SSTFLTFKPK QSLHFTFLTI GSRGDVQPYI ALAKGLMKDG HGIEFGYVGG
DPAELMRICV ENGTFTVSFL KEGMMKFRGW IDDLLKTSWE ACQGTDVLVE SPSAMAGYHI
AEALKVPYFK AFTMTWSRTR AYPHAFAVPE HKSYVLFDQV FWRATAGQIN RWRRETLGLP
STSLDKMEPH KAPFLYNFSP TIVPPPLDWP EWIHVTGYWF LDDADVSSKK WTPPPDLVEF
IDSAHKAKKK IVYSDPKAMT RCVIDAIVQS GVHAILSKGW SDRLDPEEPL PKQIYPITSI
PHDWLFAGIP TIIKPFFGDQ FFWADRVEAL GIGTAVRKLT VESLTQALVN AATDQKQIDR
ARVVGERIRS EDG
>SN
STPAVMFTST SSTFITFKPE ESLQFTLLTI GSRGDVQPYI ALAKGLIAEG HDIEFTYVGG
DPAELMRICV ENGTFTVAFL REGLSKFRGW IDDLLQTSWD ACQNTDVLIE SPSAMSGIHI

AEALQIPYFR AFTMPWTRTR AYPHAFAVPD RKTYVMFDQV FWRGTAGQIN RWRKNVLGLA
PTNLDKLEQH KVPFLYNFSP TVVPQPLDWY EWIRVTGYWF LDDADVSAKK WTPPTDLIEF
IDEARQKNKK IVYSDPDAMT RCVVEAIVES GVYAILSKGW SDRLGPEAPL PPQIYKISSV
PHDWLFAGIP TIIKPFFGDQ FFWADRVEAL GIGSSVRKLT VASLTAALLS ATTDRKQIDR
AKIIGQAIRS ENG
>GT
SVPAVMFTSS SSTFLTFKPK ESLHFTFLTI GSRGDVQPYI ALAKGLMADG HGIEFSCVGG
DPAELMRICV ENGTFTVAFL KEGLLKFRGW LDDLLKTSWE ACQGTDVLVE SPSAMAGYHI
AEALGIPYYR AFTMTWTRTR AYPHAFAVPE HRSYVLFDQV FWKAISGQIN RWRRNVLRLG
GTNLDKMEPH KVPFLYNFSP TVVPPPLDWP EWIHVTGYWF LDDAEVGAKK WSPPPDLLEF
IDSAHNAGKK VVYSNPKAMT RCVIDAIVKS GVCAILSKGW SDRLEVEEPL PKQIYSIASL
PHDWLFAGLP TIIHPFFGDQ FFWADRVEAL GIGTGVRHLT VEALTAALLA ATTDEKQIAR
ARQVGEKIRS ENG
>FS
SMPAIMFNST SSTFLTFKPK ESLQFTFLTI GSRGDVQPYI ALAKPLMADG HGIEYGYVGG
DPAELMRICI ENGTFTLSFV KEGLLKFRGW VDDLLATSWE ACKGSDVLIE SPSAMGGYHI
AEALGIPYFR AFTMTWSRTR AYPHAFAVPE HKSYVMFDQV FWRATSGQIN RWRRNVLHIG
GTSLDKMEPH KIPFLYNFSP MIVPPPLDWP EWIRVTGYWF LDDAEVGSKK WSPPPELEAF
IDNAHALGKK VVYSDPKAMT RSVIEAVVRS GVHAILAKGW SDRLEAEDPL PKQIFPVNSV
PHDWLFAGIP TIIRPFFGDQ FFWADRVEAL GIGCGVRKLT VETLTEALRA ATTDIKQIDR
ARSVGEHIRA ENG
>SC
SLPAVMFTSA SSTFLTFKPN KSMHFTCLTI GSRGDVQPYI ALAKGLMADG HGIEYGYVGG
DPAELMRICV ENGMFTVSFL KEGLQKFRGW LDDLLKTSWD ACQGTDVLIE SPSAMGGLHI
AEALRIPYFR AFTMTWTRTR AYPHAFAVPE RKSYVMFDQV FWRAIAGQVN RWRRKSLNLD
STNLDRMEPH KIPFLYNFSP TVVPPPLDWP EWIRITGYWF LDDADASGKK WSPPDSLNKF
IHKARKEHKK IVYSDPKAMT RCVIEAVVNS GVRAILSKGW SDRLDIEEPL PSSIYPIASI
PHDWLFAGIP TIIHPFFGDQ FFWADRVEAL GVGSGVRRLT VESLTDALRA ATTDVKQIEK
AKAVGEKIRS EDG
>PC
SLPAVMFAST SSTFLTFKPK KPLHFTFLTI GSRGDVQPYI ALAKGLMADG HGIEFGYVGG
DPAELMRICV ENGTFTVAFL KEGVQKFRGW IDDLLKTSWE ACQGTDVLIE SPSAMSGIHI
AEALRIPYFR AFTMTWSRTR AYPHAFAVPE RKTYVLFDQV FWRGTAGQIN RWRRNTLGLP
GTSLDKMEPH KVPFLYNFSP VVVPPPLDWP EWIRITGYWF LDDANVSSQK WLPPPDLLEF
IAAAHKENKK IVYSDPKAMT RCVVDAVVQS GVYAILSKGW SDRLDAPEPL PKQIYPVQSI
PHDWLFAGIP TIIKPFFGDQ FFWADRVEAL GVGSAVRKLS VQSLTDALIA ATTDQKQIQR
AKEIGEQIRA ENG
>CP
SVPAVMFAST SSTFLTFKPK ESLHFTFLTI GSRGDVQPYI SLAKGLMQDG HGIEFGFVGG
DPAELMRICV ENGTFTVAFL KEGLLKFRGW LDDLLRTSWE ACQGTDVLVE SPSAMGGIHI
AEALQIPYFR AFTMTWTRTR AYPHAFAVPD RKTYVMFDQV FWRAISGQVN RWRRNVLHLP
NTSLDRLEPH KVPFLYNFSP TLVPPPLDWP EWIHVTGNWF LDDADVSATK WTPPPDLLPF
IDSAHAQGKK VVYSDPQAMT RCVIEAVVRS GVYAILSKGW SDRLEPKEPL PPQIYSISSI
PHDWLFAGKP TIIRPFFGDQ FFWADRVEAL GIGTGVRKLT VEALTDALTS ATTDIKQIDR
ARIIGEQIRS ENG
>RF
SLPAVMFTSK SSTFLTFKPQ KSLRFTFLTI GTRGDVQPYI ALAKGLIADG HGIEFGYVGG
DPAELMRLCV ENGMFTVSFL KEGVQKFRSW IDDLLKTAWE ACQGTDVLIE SPVAMGGIHI
AEALKIPYFR AFTMTWTRTR AYPHAFAVPE RKTYVMFDQV FWRGTAGQIN RWRKHMLGLS
STSLDKLEPH KVPFLYNFSP TVVPPPLDWP EWIRVTGYWF LDDADGGSKT WSPPDGIVEF
IDKAHNAGKK VVYSDPDAMT RCVVEAIVKS GVHAILSKGW SDRLEPEIPL PSQIYPIQSI
PHDWLFAGIP TIIKPFFGDQ FFWADRVEAL GIGSGVRKLT VEHLTDALVY ATSDARQIAR
AKLVGEQIRS ENG
>RI

```
SLPAVMFTSK SSTFLTFKPQ KSLRFTFLTI GTRGDVQPYI ALAKGLIADG HGIEFGYVGG
DPAELMRLCV ENGMFTVSFL KEGVQKFRSW IDDLLKTAWE ACQGTDVLIE SPVAMGGIHI
AEALKIPYFR AFTMTWTRTR AYPHAFAVPE RKTYVMFDQV FWRGTAGQIN RWRKHMLGLS
STSLDKLEPH KVPFLYNFSP TVVPPPLDWP EWIRVTGYWF LDDADGGSKT WSPPDGIVEF
IDKAHNAGRK VVYSDPDAMT RCVVEAIVKS GVHAILSKGW SDRLEPEIPL PPQIFPIQSI
PHDWLFAGIP TIIKPFFGDQ FFWADRVEAL GIGSGVRKLT VDNLTEALAY ATTDAKQIAR
AKLVGEQIRS ENG
>LB
SLPALMFTSA SSTFLNFKPH KSLRFTFLTI GSRGDVQPYI ALAKGLIADG HGIEYGYVGG
DPAELMRICV ENGTFTVSFL KEGLQKFRGW LDDLLKTSWE ACQGADVLVE SPSAMGGYHI
AEALAIPYFR AFTMTWTRTR AYPHAFAVPE RKTYVMFDQV FWRATAGQIN RWRRDLLHLG
PTSLDKMEPH KIPFLYNFSP HVVPPPLDWP EWIRVTGYWF LDDADVSSKK WTPPQDLIDF
IDNAHQSRQK VVYSDPKTMT RCVVEAVLQS GVRAILSKGW SDRLESEEAL PAEIYPISSV
PHDWLFAGIP TIIRPFFGDQ MFWADRVEAL GVGTGVRKLT VQSLTEALVL ATTDQKQIDR
AKAIGEQIRG EKG
>PS
SLPAHMFTSA SSTFLTFKPK ESLHFTFLTI GSRGDVQPYI ALAKGLMADG HGIEFGYVGG
DPAELMRICV ENGTFTVSFL KEGLLKFRGW LDDLLKTSWE ACQGTDVLIE SPSAMAGFHI
AEALRIPYFR AFTMTWTRTR AYPHAFAVPE RKTFVMFDQV FWRAISGQIN RWRRKTLGLP
STSLDKMEPH KVPFLYNFSP TIVPPPLDWP EWIRVTGYWF LDSAEVSAQK WTPPAELVQF
IDSAHQAGKK VVYSDPKGMT RSVIEAIVRS GVYAILSKGW SDRLEPEEPL PKQIYQINSI
PHDWLFAGIP TIIRPFFGDQ FFWADRVEAL GVGAGVRHLT SESLAQALIA ATTDEKQIQR
AKVVGERIRA ENG
>PI
SLPAIMFTSA SSTFLTFKPK ESLHFTFLTI GSRGDVQPYI ALAKALLADG HGIEYGYVGG
DPAELMRICV ENGTFTLAFM KEGLLKFRGW LDDLLATSWE ACQGTDVLIE SPSAMGGYHI
AEALGIPYFR AFTMTWSRTR AYPHAFAVPE RKSYVMFDQV FWRATSGQIN RWRRNVLHLA
STSLDKMEPH KIPFLYNFSP IVVPPPLDWP EWIRITGYWF LDDADVGSKK WTPPSDLEIF
IDSAHKVGKK VVYSDPKAMT RTIVEAIVHS GVHAVLSKGW SDRLEPEEPL PPQIFRISSV
PHDWLFAGIP TIIKPFFGDQ FFWADRVEAL GVGSGVRKLT VESLTDALRA ATTDIKQIDR
AKLIGEQIRA ENG
>HI
SLPAVMFTST SSTFLTFKPK NPLRFTFLTI GSRGDVQPYI ALAKGLMADG HGIEFGYVGG
DPTELMRICV ENGTFTVSFL REGMQKFRGW IDDLLKTAWD ACQDTDVLVE SPSAMAGYHI
AEALRIPYFK AFTMTWSRTR AYPHAFAVPE RKTYVMFDQV FWRGTAGQIN RWRRNTLGLP
STSLDKMEPH KIPFLYNFSP IVVPPPLDWP EWIRVTGYWF LDDASVGAKK WTPPPDLVEF
IDSAHSQGKK VVYSDPKAMT RCIVDSIVQG GVHAILSKGW SDRLEAEEPL PKSIYPLASV
PHDWLFAGIP TIIRPFFGDQ FFWADRVEAL GVGTGVRKLT VDSMAEALIA ATTDHKQIDR
AKLVGEQIRS ENG
>SH
SLPAVMFTST SSTFLTFKPK ESLRFTFLTI GSRGDVQPYI SLAKGLMRDG HGIEFGYVGG
DPAELMRICV ENGTFTVSFL REGVAKFRGW IDDLLKTAWD ACQDSDVLIE SPSAMAGYHI
AEALRIPYFR AFTMTWSRTR AYPHAFAVPE RKTYVMFDQV FWRGTASQIN RWRRNILGLP
STSLDKMEPH KIPFLYNFSP TVVPPPLDWP EWIRVTGYWF LEDASASASK WTPPPDLVEF
IDNAHALGKK VVYSDPKAMT RTVIEAIVQS GVHAILSKGW SDRLEPEEPL PKQIYPLASV
PHDWLFAGIP TIIKPFFGDQ FFWADRVEAL GVGSGVRKLT VESLAEALGT ATTDERQITR
AKVIGEAIRS ENG
>AS
TVPAVMFTST SSTFLTFKPQ ESLRITLLTI GSRGDVQPYI ALGKGLIADG HGMEFGYVGG
DPAELMRICV ENGTFTVGFI REGVQMFRGW IDDLLKTSYE ACKGSDLLIE SPSAMAGIHI
AEALKIPYYR AFTMPWTRTR AYPHAFAVPE HKTYVMFDQV LWRGTASQIN RWRRRLLGLP
PTNLDKMEQH KVPFLYNFSP AIVPPPLDWY EWIRVTGYWY LDDADVSAKK WQAPQDLLDF
IASARDAGKK IVYPDPTSLT RTVVDAIQQS GVHAILSKGW SDRLEPEIQL PSSIFPIASV
PHDWLFAGIP TIIKPFFGDQ FFWADRVEAL GIGSSVRKLT VESLAQALHA ATTDEKQIAK
```

AAIVGQQLRA ENG
>EG
SVPAVMFTST NSTFLTFKPQ ESLRFTFLTI GSRGDVQPYI ALGKGLVADG HGLEFGYVGG
DPAELMRICV DNGTFTVGFL REGVTMFRGW IDDLLNTSWE ACQNTDVLVE SPSAMAGIHI
AEALRIPYFR AFTMPWSRTR AYPHAFAVPE HKTYVMFDQV FWRGTASQIN RWRRRTLGLP
STTLDKIEQH KVPFLYNFSP AVVPQPLDWY EWIRVTGYWF LDDADVSAKK WEAPPDLVEF
IDNAHNAGKK VVYPDPTALT RTVVEAIEKS GVHAILSKGW SDRLEPEVRL PPQIFPIASV
PHDWLFAGIP TIIKPFFGDQ FFWADRVEAL GIGSAVRKMT VDSLATALHA ATTDEKQIGR
AALVGQQIRA ENG
>RM
SLPAVMFTSQ SSTFLSFKPD RSMHFTCLTI GSRGDVQPYI ALAKGLIADG HGIEFGYVGG
DPAELMRICV ENGMFTVSFF KEGIQKFRGW IDDLLKTSWE ACQGTDVLIE SPSAMGGIHI
AEALAIPYFR AFTMTWTRTR AYPHAFAVPE HKSYVMFDQV FWRAISGQIN RFRRNALRLP
STNLDRMEPH KVPFLYNFSP SVVPTPLDWP EWIRITGYWF LDDAEVSAQR WHPPKPLEDF
LTSARNAGKK HCRFRPRSMT RCVIDAIKAS GVYAVLSKGW SDRLEPEAPL PPQIFPINSL
PHDWLFAGIP TIIKPFFGDQ FFWGDRVEAL GVGSCVRKLT VESLAEALIQ ATTNEKQIDR
ARILGEQIRS ENG
>TA
SLPAVMFTST SSTFLTFKPQ RPLHFTCLTI GSRGDVQPYI ALAKGLMADG HGIEFGYVGG
DPAELMRICV ENGMFTFSFM KESLQKFRGW IDDLLKTSWE ACQGTDVLIE SPSAMAGIHV
AEALRIPYYR AFTMTWTRTR AYPHAFAVPE HKSYVAFDQV FWRFTAGQIN RWRRNTLKLG
STNLDKLEPH KVPFLYNFSP SVVPPPLDWP EWIRVTGYWF LEDAEVSAQK WSPPQDLVDF
IEGAHKIGKR VVYSDPEAMT KCVVDAIKRG GVYAILSKGW SDRLEPEVPL PPEIYPIKSI
PHDWLFAGIP TIIKPFFGDQ FFWGDRVEAL GVGTSVRKLT VENLADAFIA ATTDEKQIAK
AKLIGQRIRA VSK
>SP
SLPAVMFTST SSTFLTFKPQ QPLHFTCLTI GTRGDVQPYI ALAKGLMADG HGIEFGYVGG
DPSELMRICV ENGMFTVSFL REGVQKFRGW IDDLLKTSWE ACQGTDVLIE SPSAMGGIHI
AEAMRIPYYR AFTMTWTRTR AYPHAFAVPE HKSYVMFDQV FWRATAGQIN RWRRNTLKLQ
STNLDKMEPH KVPFLYNFSP TVVPPPLDWP EWIRVTGYWF LDDADVTAKK WEPPQDLIEF
IDKAHSSGKK IVYSDPDAMT RCVVEAIEKS GVHAILSKGW SDRLQPEIPL PPSIYPLKSV
PHDWLFAGIP TIIKPFFGDQ YFWGDRVEAL GIGSCVRKLT VDGLADAFIA ATTDEKQIEK
ARHIGEQIRS ENG
>PN
DLPAVMFTST SSTFLTFKPR QPMHFTCLTI GSRGDVQPYI ALAKGLIADG HGIEFGYVGG
DPAELMRICV ENGMFTVSFL KEGIQKFRGW IDDLLKTSWE ACQGTDVLIE SPSAMAGIHI
AEALGIPYYR AFTMTWTRTR AYPHAFAVPD HKSYVMFDQV FWRATAGQIN RWRRNTLNLP
STTLDKLEPH KVPFLYNFSP TVVPPPLDWP EWIRITGYWF LDDAEVSASK WTPPQDLADF
IDNAHKAGKK VVYSDPDVMT RCVIEAIMKA GVCAILSKGW SDRLEPEIPL PPEIYPVKSV
PHDWLFAGIP TIIKPFFGDQ FFWGDRVEAL GVGSCVRKLT VDGLAEALIA ATTDERQIAK
AKLVGERIRS ENG
>FM
DLPAVMFTST SSTFLTFKPR HPLHFTCLTI GSRGDVQPYI ALAKGLMADG HGIEFGYVGG
DPAELMRICV ENGMFTVSFL KEGIQKFRGW IDDLLKTSWE ACKGTDVLIE SPSAMAGIHI
AEALRIPYYR AFTMTWTRTR AYPHAFAVPE HKSYVMFDQV FWRATAGQIN RWRRETLGMS
STNLDKLEPH KVPFLYNFSP TVVPQPLDWP EWIRVTGYWF LDDADVSAEK WSAPKDLVDF
IDSAHQAGKK VVYSDPEAMT RCVVEAIIRS GVYAILSKGW SDRLEPEVPL PSQIYTIKSI
PHDWLFAGIP TVIKPFFGDQ YFWGDRVEAL GVGSCVRKLS VEALSEALTL ATTDEKQIAK
ARLVGERIRS ENG
>OS
SLPAVMFTST SSTFLTFKPR QPLHFTFLTI GTRGDVQPYI ALAKGLLADG HGIEFGYVGG
DPAELMRICV ENGMFTVSFL KEGIQKFRGW IDDLLKTSWE ACQGTDVLVE SPSAMAGIHI
AEALRIPYYR AFTMTWTRTR AYPHAFAVPE HKSYVMFDQV FWRATAGQIN RWRRNTLKLS
GTNLDKLEPH KVPFLYNFSP TVVPPPLDWP EWIRVTGYWF LDDADVSAMT WSPPKDLVDF

IEAAHQAGTK VVYSDPDVMT RCVVEAIVRS GVHAILSKGW SDRLEPEIPL PPQIYSIKSI
PHDWLFAGIP TIIKPFFGDQ FFWGDRIEAL GIGTCVRKLT VESLANALTI ATTDEKQIAK
AKLVGDKIRS ENG

03-SEC7 domain

>RM
DKPREIFEPL RLACESGNEK LMIASLDCIS KLISYSFFVD LVDLVTHTIT ACHTETTSDA
VSLQIVKALL ALVLSPTILV HQSSLLKAVR TVYNIFLLSP DPINQTVAQG GLTQMVHHVF
DMFTKDAFLV FRALCKLTMK TLNTESERDL KSHAMRSKLL SLHLVLTILN SHMQVFVDHN
SIIYSSSTNE STSFIQATKQ YLCLAISRNA LSPVPQVFEI SVEIFWRVLS GMRTKLKKEI
EVFFHEIFTP ILEMKTSTLK QKSVILGMLA RLCQEPQALV EIYLNYDCDR EAVDNIYEHL
MNTLSKIASP ALSTSALAVP GANLGLSEQQ LKRQGLECLI SVLRSLLAWG TDDPGRFESE
KQRKNALQEG IRRFNYKPKG IEYLIENRFI PSKAPVDIAK FLLSTDGLNK ATIGEGDEAN
IATMHAFVDL MDFSDQPFVN ALRMFLQSFR LPGEAQKIDR FMLKFAERYI AGNDAAYVLA
YSTILLNTDA HNPQIKNRMT KTDFIKNNKG INDLPEELLL AIFDEITNNE IRMKDEVANV
GRDLQKEAFV IQSLGTLQDQ FFSASHFVHV KPMFEVAWIP FLAGISGPLT DTDNLEIVEL
CLEGFKAFVT TLAKFTFLNN LGEMKTKNME AIKTLLDIAV TEGNHLRGSW QEVLTCVSQL
ERMQLISSGK SRKIPNEELA NESRSTHITV AADMVFSLSH YLSGTAIVEF VRALSHVSWE
EIQSSGLSEH PRLFSLQKLV EISYYNMNRI RLEWSNLWEI LGEHFNKVCC HHNPHVGFFA
LDALRQLAMR FLEKAELPHF KFQKDFLKPF EYTMIHNSNP EIRDLVLQCL QQMVQAKAHN
LVSGWRAMFG VFSAASKVPT ERIVNSSFEI VSRINKDHFS DVVKHGAFAD LTVCITDFCK
VSKDEAMIKF WFPVLFSFYD IIMNGEDLEV RRLALDSLFN TLKEYGTDFT IDFWDSICQE
LLFPIFAVLK SSQDITRWST QEDMSVWLST TMIQALRDLI DLYTYFFETL ERFLDGLLEL
LCVENDTLAR IGTSCLQQLL EKNVKKLSPA RWERVVTAFV RLFKTTTPHQ LFDESLRMDR
RRIFKQIIVK CVLQLLLIET TNELLRNSDV YNTIPPEHLL RLMGVLDHSY QFARVFNEDR
ELRTALWKET SSASTLVTVL VQMYNDPRPE HQAIRVQVAE RLMPLSLGVI QDFNKLRPES
QSKNITAWIP VIGQIAQTFC RLDEKTFARY LPAIYPLTTD LLSKEQEVRG YLREYYIRVG
QSQGI
>SP
DKPREIFEPL RLACETRSEK LMVASLDCIQ KLVSYSFFVE LVDIVAHTIT SCHTESTPDA
VSLQIVKALL SLVLSSTMLI HQSSLLKAVR TVYNIFLMSP DPVNQTIAQG GLTQMVHHVF
DLFVKDAFLV FRALCKLTMK NLNTESERDL RSHAMRSKLV SLHLVLTILN SHMPVFVDPS
SIIYSSSSNE ATTFINATKQ YLCLSLSRNA LSPVPQVFEI SVEIFWRVLA GMRTKLKKEI
EVFLHEIFIP ILEMKTATLK QKSVILGMLQ RLCEDPRALV EIYLNYDCDR EAVDNIYEHL
MNTISKIATP ALSTAALAVP GANLGLSEQQ LKRQGLDSLV AVLRSLVAWG TDDPGRFENA
KQRKTVLQEG LRKFAQKPKG IDFMLEQGFL SSKSPHDIAA FLLSTEGLNK AAIGEADTEN
VEIMHAFVDQ LNFAGLSFIE AMRTFLQAFR LPGEAQKIDR FMLKFAERYI AGNDAAYVLS
YSTILLNTDA HNPQVKNRMT KAEFIKNNRG INDLPEETLN AIFDDITSNE IRMKDEIANV
GRDLQREAYV MQSLGMANDQ FFSASHFVHV RPMFEVAWMA FLAGISGPLT QADDMEIVEL
CLEGFKAFVT TLAKFTFLNN LGEMKPKNME AIKALLDIAV SDGNNLRGSW QEVLSCVSQL
ERMQLISSGK TRKLPAEELA NESRSTHITV AADMVFSLSH YLNGTAIVEF VRALSSVSWE
EIQSSGLSEH PRLFSLQKLV EISYYNMNRI RLEWSNLWEI IGEHFNQVCC HHNPHVGFFA
LDALRQLAMR FLEKEELSHF KFQKDFLRPF EYTMIHNANP EIRDMVLTCL QQMIQARAHN
LRSGWRTMFG VFSAASKVLT ERIVNSSFEI VTRLNKEHFT EIVKYGSFAD LTVCITDFCK
VNKDDANIKY WFPVLFSFYD IIMNGEDLEV RRLALDSMFS TLKTHGSTFT TEFWDSICQE
LLFPIFAVLK SSSDVSRWTT QEDMSVWLST TMIQALRDLI DLFTYFFETL EHLLDGLLDL
LCVENDTLAR IGTSCLQQLL ENNVKKLSAD RWERVVTTFV KLFRTTTPHQ LFDENLRVDR
RRIFKQIIVK CVLQLLLIET TNELLQNKDV YDTIPPEHLL RLMGVLDHSY QFARMFNDDK
DLRTALWKET SSAETLVNVL LQMYYDSRPK HQELRSQVAE RFLPLGLGVI QDFNKLRPDT
QAKNIAAWMP VIAKILDGFN RLDEKAFVRY LPGIYPLATD LLARDTASRT SLREYFVRVG
KVQGI
>TA
DRPREIFEPL RLACETGNEK LQVVSLDCIS KLISYSFFVE LVDVVVHTIT ACHAETTPDT
VSLQIVKALL SIVLSATLLV HQSSLLKAVR TVYNIFLLSP DAVNQTVAQG GLTQMVHHVF

DLFIKDAFLV FRALCKLTMK NLNTESERDL KSHAMRSKLV SLHLVLTILN SHMNVFVDPS
SIIFSTSSNE ATSFINATKQ YLCLSLSRNA LSPVPQVFEI SVEIFWRMLS GMRTKLKKEI
EVFLHEIFIP ILEMKTATLK QKSVILGMLQ RLCQEPQALV ELYLNYDCDR EAADNIYEHL
MNTISKIATP ALTTNALAVP GHNLGMSEQQ LKRQGLESLV AVLRSLVTWG IDDPERFESA
KQRKTILQDG IRRFTSSPKG IAFLLENGFI PARTPADIAR FLLHTDGLNK SAIGEGDEEN
IAIMHSFVDQ MDFANTPFVD ALRMFLQAFR LPGEAQKIDR FMEKFAQRYI EGNEAAYVLS
YSIILLNTDA HNPQVKKHMT KEDFFKNNRK INDFPEEFMS IIYDDITMNE IRMKDEIANV
GRDLQKEAYV MQSLGIANNQ FFSASHFVHV KPMFEVAWIP FLAGISGPLT DTDDLEVVEL
CLEGFKAFVT TLAKFTFLNN LGEMKAKNME AIKTLLDIAV SEGNHLRGSW YEVLTCVSQL
ERMQLISSKS SRRMPAEELA NESRSTHITV AADMVFSLSH FLSGTAIVEF VRALSSVSWE
EIQSSGLSEH PRLFSLQKLV EISYYNMGRI RLEWTNLWEI IGEHFNQVCC HHNMHVGFFA
LDALRQLAMR FLEKEELPHF KFQKDFLKPF EYTMIHNTNP DIRDMVLQCL QQMIQARVHN
LVSGWRTMFS VFSAASKVLT ERIVNSAFEI VTRLNKEHFS EIVRHGAFAD LTVCITDFCK
VSKDDAMIKF WFPVLFGFYD IIMNGEDLEV RRLALDSLFT TLKTYGSSFT VEFWDTVCQE
ILFPIFAILK SSSDLSRWST QEDMSVWLST TMIQALRDLI DLYTFYFETL ERFLDGLLDL
LCVENDTLAR IGTSCLQQLL ESNVKKLSVA KWERVVTTFV KLFKTTTPHQ LFDENLRMER
RRIFKQIIVK CVLQLLLIET TYELLQNKDV YNTIPPEHLL RLMGVLDHSY QFARMFNEDK
ELRTALWKET NSAETLVNIL VQMYYDPRPE HVALRPQVAD KLLPLGLGVI SDFNKLRIDT
QAKNIAAWMP VISIILQGFV RLDEKAFGRY LPAIYPLTSE LLAKDSEVRA GLRDYFIRVG
QVQGI
>PN
DKPREIFEPL RLACETGNEK LQIASLDCTS KLISYSFFLE LVDLVTHTIT ACHTETTPDT
VSLQIVKALL ALVLSPTLLV HQSSLLKAVR TVYNIFLLSP DPINQTVAQG GLTQMVHHVF
DLFFKDAFLV FRALCKLTMK NLNTESERDL KSHAMRSKLV SLHLVLTILN SHMQVFVDPS
SIIYSSSSNE ATSFINATKQ YLCLSLSRNA LSPVPQVFEI SVEIFWRVLT GMRTKLKKEI
EVFFHEIFIP ILEMKTSTLK QKSVILGMLQ RLCQEPQALV EIYLNYDCDR EATDNIYEHL
MNTISKISTP NLTTAALAVP GHNLGLSEQQ LKRQGLESLV AVLKSLVTWG TDDPGRFESA
KQRKTILQEG IKRFNYKPKG IEFLLDNGFI PSREPVEIAK FLLSTDGLSK ATIGEGDEEN
IAIMHAFVDL LDFSNLPFVD ALRLFLQSFR LPGESQKIDR YMLKFAERYI AGNDTAYVLS
YSTIMLNTDA HNPQVKNRMT KEEFIKNNRG INDLPDEFLL SIFDEIQTNE IRMKDEIASV
GRDFQKEAYV MQSLGMANDQ FFSASHFVHV RPMFEVAWIP FLAGISGPLT DTDDLEVVEL
CLEGFKAFVT TLAKFTFLNN LGEMKTKNME AIKALLDIAV SDGNHLRGSW HEVLTCVSQL
ERMQLISSGR ARKMPAEELA NESRSTHITV ATDMVFSLSH YLSGTAIVEF VRALSAVSWE
EIQSSGLSEH PRLFSLQKLV EISYYNMGRI RLEWSNLWEI IGEHFNQVCC HHNPHVGFFA
LDALRQLAMR FLEKEELPHF KFQKDFLKPF EYTMIHNNNP DIRDMVLQCL QQMIQARVHN
FVSGWRTLFS VFSAASKVLT ERVVSSAFEI VTRINKDHFS EIARNGAFAD LTVCITDFCK
QCLDDAMIKF WFPVLFSFYD IIMNGEDLEV RRLALDSLFT TLKEYGSTFT VEFWDTVCQE
LLFPIFAVLK NSSDLSRWST QEDMSVWLST TMIQALRDLI DLYTYYFETL ERFLDGLLDL
LCVENDTLAR IGTACLQQLL EKNVKKLSAA RWERVVTTFV KLFKTTTPHQ LFDENLRTDR
RRIFKQIIVK CVLQLLLIET TNELLQNDEV YDTIPPEHLL RLMGVLDHSY QFARMFNEDK
ELRYALWKET SSADTLVTVL CRMYHDTRPQ HLELRPQIAD KFLPLGLGVV NDFNKLRMET
SQKNIAAFMP VVSKIIKGFC SLSDKAFGRF LPAIYPSTAE LCARDPEVRS HLRDYFVRVG
QFQGI
>FM
DKPREIFEPL RLACETGNEK LQIASLDCIS KLISYSFFLE LVDIVTHTIT ACHTETAPDA
VSLQIVKALL SLVLSPTLLV HQSSLLKAVR TVYNIFLLSS DPVNQTVAQG GLTQMVHHVF
DLFFKDAFLV FRALCKLTMK NLNTESERDL RSHAMRSKLV SLHLVLTILN SHMQVFVDPS
SIIYSASTNE ATSFINATKQ YLCLSLSRNA VSPVPQVFEI SVEIFWRLLT GMRTKLKKEI
EVFFHEIFVP ILEMKTATLK QKSVILGMLQ RLCQEPQALV EIYLNYDCDR EAADNIYEHL
MNTLSKISSP ALSTSALAVP GQNLGLSEQQ LKRQGLESLV AVLRSLVTWG TDDPGKFESA
KQRKTILQDG IRRFNYKPKG VEFLIQNGFI PSREPVEVAK FLLNTDGLSK AVIGEGDDEN
IATMHAFVDQ LDFSGMAFVD ALRTFLQTFR LPGEAQKIDR FMLKFSERYI AGNDTAYVLS
YSTIMLNTDA HNPVVKQRMT KADFIKNNRG INDLPEEFLS EIFDDIQTNE IRMKDEMANV
GRDLQKEAYV MQSLGMANDQ FFSASHFVHV RPMFEVAWIP FLAGISGPLT DTDDLEVVEL

CLEGFKAFVT TLAKFTFLNN LGEMKAKNME AIKALLDIAV SDGNHLRSSW HEVLSCVSQL
ERMQLVSNGR ARKMPAEELA NESRSTHITV AADMVFSLSH YLSGTAIVEF VRALSAVSWE
EIQSSGLSEH PRLFSLQKLV EISYYNMNRI RLEWSNLWEI IGEHFNQVCC HHNPHVGFFA
LDALRQLAMR FLEKEELPHF KFQKDFLRPF EYTMIHNNNP DVRDMVLQCL HQMIQARVHN
FVSGWRTLFS VFSAASKVLT ERVVNSAFEL VTRLNKEHFA EIIRHGAFAD LTVCITDFCK
VSKDDAMIKF WYPVLFSFYD IIMNGEDLEV RRLALNSLFT TLKTHGSTFS VEFWDTVCQE
LLFPIFAVLK SSSDLSRWST QEDMSVWLST TMIQALRDLI DLYTFYFETL ERFLDGLLDL
LCVENDTLAR IGTACLQQLL ENNVKKLSAG RWERVVTTFI KLFRTTTPHQ LFDENLRGER
KRIFKQIIVK CVLQLLLIET TSELLQNNEV YDTIPPEHLL RLMGVLDHSY QFARMFNEDK
ELRTALWKET SSADTLVTVL SRMYYDPRPQ HLALRAQIAD KFLPLGLGVI TDFNKLRMES
SAKNISAWMP VVARIVQGFC GLSDKAFGRF LPATYPAVSE LVARDSEVRS HLRDYFVRVG
QFQGI
>OS
DKPREIFEPL RLACETGNEK LQIASLDCIS KLISYSFFLE LVDIVTHTIT ACHTEMTPDA
VSLQIVKALL SLVLSPVILV HQSSLLKAVR TMYNIFLLSP DPVNQTVAQG GLTQMVNHVF
DLFFKDAFLV FRALCKLTMK NLNTESERDL KSHAMRSKLV SLHLVLTLLN SHMQVFVDPS
SIIYSASSNE ATSFINATKQ YLCLSLSRNA LSPVPQVFEI SVEIFWRVLS GTRTKLKKEI
EVFFHEIFIP ILEMKTSTLK QKSVILGMLQ RLCQEPQALV EIFLNYDCDR EAADNIYEHL
MNTISKIASP ALSTTALAVP GQNLGLSEQQ LKRQGLESLV AVLRSLLAWG TDDPGRFESA
KQRKTILQDG IKRFNYRPKG IEFLLENGFI ASREPAEIAK FLLNTDGLSK ATIGEGDDKN
IAIMHAFVDQ LDFSNLPFVN ALRLFLQSFR LPGESQKIDR YMLKFAERYI AGNDTAYVLS
YSTIMLNTDA HNPQVKNRMT RDDFIKNNRG INDLPEELLS AIYDEIVSNE IRMKDEVANV
GRDLQKEAYV LQSLGMANDQ FFSASHFVHV RPMFEVAWIP FLAGISGPLT DTNDLEVVEL
CLEGFKAFVT TLAKFTFLNN LGEMKAKNME AIKALLDIAI TEGNHLRGSW HEVLTCVSQL
ERMQLISSGK PRKMPTEELA NESRSTHITV ATDMVFSLSH YLSGTAIVEF VRALSAVSWE
EIQSSGLSEH PRLFSLQKLV EISYYNMNRI RLEWSNLWEI IGEHFNQVCC HHNPHVGIFA
LDALRQLAMR FLEKEELPHF KFQKDFLKPF EYTMTHNNNP DIRDMVLQCL QQMIQARVHN
FVSGWRTLFS VFSAASKVLT ERIVNSAFEI VTRLNRDHFP EVIRHGAFAD LTICITDFCK
VSKDDAMIKY WFPVLFSFYD IIMNGEDLEV RRLALDSLFT TLKSYGSTFT VDFWDTVCQE
LLFPIFAVLK SSSDLSRWTT QEDMSVWLST TMIQALRDLI DLYTFYFETL ERFLDGLLDL
LCVENDTLAR IGTSCLQQLL EKNVKKLSAA RWERVVTTFV KLFKTTTPHQ LFDENLRTER
KRIFKQIIVK CVLQLLLIET TNELLQNDEV YDTIPPEHLL RLMGVLDHSY QFARMFNEDK
ELRTALWKET SSADTLVTVL RRMYYDPRPH HQALRPKIAD KFLPLSLGVI TDFNKLRMES
SAKNISAFMP VVARIIEGFC HLDDKAFGRY LPAIYPLTAE LLARDSEVRS HLRDYFIRVG
QFQGI
>CC
DHAREILEPL RLACETQNEK LVIASLDCIS KLVSYSFFAE LVDLVAHTIA SCHNESTSDT
VSVQIVKALL GLVLSQTTIV HHHSLLQSVR TVYNVYITST SPQIQMLAQG SLTQIVDHVF
DLFLKDAFLI FRALCKLSLK PIPPENEQDP KAHTYRSKVM SLQLILHVLN QHMALLVDPA
SIIYSSSTQD TVTFDKQISP HLLQSLTRNA PSPVPAVFEL SVEIFWRALY SMRAQLKTEI
GVLLHEIYIP ILEMKTSTLR QKAVILGMLA RLCQEPQALV EIYLNYDCDE RAVDNIYERL
MNIISKFGTN SLNGGVLAAS GSTLGLSETQ IKRQALECLV AVLRSLVAWG TDDPSRFESA
KQRKTALLEG IKKFNFKPKG IQFLIENGFI PDNNPKCIAT FLHETDGLNK TMLGEGDEEH
VAIMHAFVDM MDFKDTLFVD SLRHFLQAFR LPGEAQKIDR FLLKFAERYI NGNDAAYVLG
YSVIMLNTDA HNPQVKKRMT KTDFIKNNRG INDLPEDLLS SIFDDIVSNE IVMNDEIASV
GRDLQKEAYV LQTSGMSNDQ FYSASQSIHI RPMFEVAWMP FLAGLSNPLQ ETDDLQVVEL
CLEGFRAFVT TLAKFTFLNN LGEMKIKNME AIKALLDVAV HDGNNLKASW REVLKCVSQL
EHMQLIGTGR SKRVPNEELA SQSRSTHITV AADMVFSLSH YLSGTAIVDF VRALCDVSWE
EIQSSGNSTH PRLFSLQKLV DISYYNMNRI RLEWSNLWEI LGEHFNQVCT HPNAVVASFA
LDALRQLAVR FLEKEELPHF KFQKDFLKPF EYTMIHNRST DIRDMVLQCL HQMIQAKVHN
LRSGWRTMFG VFSAASKVME EGIVISAFDI VKNVNATHFP SIVKHGAFAD LTVCITDFCK
ISKDDGMIKY WYPVLFGFYD IIMNAHDLEV QKLALESLFS ALKVYGTTFP AEFWDTVCQE
LLFPIFAILK NKHDLSRFHS QEDMSVWLQS TMFQALRALI ELFTFHFSIL ERLLDGLLEL
LSVENKAFSQ IGTSCLQQLL ETNVTRLSPE RWEKVSATFV RLFRTTTPHQ LFDENLRAER

QQIFGQIIVK CILQLLLIEM TSDLLKNEEF YSAIPPDQLL KIMGILDHSY QFARSFNDDK
QLRTELWKES TSAATLVNVL LRMYYDNRPD YRPYRHQVAE RLLPLALGVL GDYNKLRPDT
QAKNIYAWNP VVAEILDGFG RFDDNAFNTF LHAIYPLAVE LLSRDPEIRQ PLKVYFLRVG
RAQGI
>SN
FDPRIVFEAL KLACETRSEK LMVASLDCIS KLISYSFFSD LAELITHTVT SAYTETTPDA
VSLQIVKALL ALVLSPDILI HHSSLLKAVR SVYNVFLLSQ DPVTQMVAQG GLTQIVNHVF
ELFVKDAFLV FRALCKLTMK PLATESERDI KSHAMRSKLL SLHLILNVLN SHMSVFVDPT
SIIYSGSSGE ATQFLQATKQ YLCLSLSRNA VSSVPQIFEI SVEMLWTVIS GMRTKLKKEI
EVLLTEIFIP ILELRMSTLR QKASILSLLS RLSQDPQALV DIYINYDCDS EALENIYERV
INIISKMASP SLSTSALSMP GNGAVLSESL LKRQALECLV SVLRSLAAWG TDDPDRFQNA
KQRKTTLLAG IKKFNFKPKG VAFFIDAGFI SAREPEPIAK FLMSTDGLSK TMIGEGEEAN
KAIMHAFVDL LDFSDLSFTD ALRMFLQAFR LPGEAQKIDR FMLKFAEKYI EGNDAAYVLA
YSTIMLNTDA HNPQVKNRMT KLDFVKNNRG INDLPEEFLG TIFDEIQSNE IRMKDEVANV
GRDFQKEAYI MQSTGMLSHQ FFSASHYHHV KPMFEVAWMS YLAGLSGPLQ DTNDLEIVEL
CLDGFKSFVT TLAKFTFLNN LGEMKTKNME AIKTLLDVAV NEGNQLKGCW RDILTCVSQL
ERMQLIGGRR QRKPPAEELA AESRSTHITV AADMVFSLSH YLSGSAIVDF VQALSDISWE
EIQSSGMSET PRLFSLQKLV EISYYNMGRI RIEWSNMWAI LGEHFNMICC HNNIHVVFFA
LDALRQLAMR FLEKEELSHF KFQKDFLKPF EYTMIHNTNN DARDMVLQCL QQMIQARMQN
MRSGWRTMFG VFSAAARANN ERVTTTGFEI VTRLNEDHFE TVVQNGAFAD LTVCITDFCK
ASRDEPTIKF WYPVLFGFYD IIMNGEDLEV RQMALDSLFS TLKKYGTNFP ADFWDTICKE
LLFPIFAVLK STADVSRFST QEDMSVWFST TMVEALRNVI DLYTHYFDLL ERTLDGLLEL
LSVENDTIAR IGTSCFQKLL ESNVKKLSKS HWERVVSTFV KLYKMTTPHQ LFDENLRVER
RRVFQQIVVK CVLQLLLIET THELLQNREI YETIPPEQLL RLMGVLDHSY QFARSFNDDK
ELRNGLWKES ASASTLVNVL FRMYFDTRPD HQASKQLVAD RLLPLGLGVI QDFNKLKPES
NMKSIAAWSP VVAVILHGFC GFEDQGFKRY LPAIYPLVTD ILSREPDIRL AARTFFVRVG
KLQNL
>AS
DKPREIFEPL RLACETRNEK LMIASLDCIS KLISYSFLVE LVDLVTHTIT SCYTESTPDA
VSLQIVKALL ALVLSPVTLV HHSSLLKAVR TVYNVFLLSQ DPVNQVVAQG GLTQIVNHVF
DLFLKDAFLV FRALCKLTMK PLNTESERDL KSHAMRSKLL SLHLVQTILS THMNIFVSPS
SYIYSSSSRE STPFIQATKQ YLCLALSRNA VSPVPQVFEI SVEIFWRVLS GMRKQLKREI
EVLLNEIFIP ILEMRNATAK QKGVLLNMLS GLCQDPQALV EIYLNYDCDK DAIDNIYERL
MNVISKIGTP SLSTTALGHQ PDAAHQNEVN LRRLSLECLV FVLRSLVAWG TDDPGKFESA
KQMKTTLNEG IKKFNFKPKG IEFFLDTGFI PSNTPQDIAR FLLETEGLSK AAIGEGDDLN
VAVMHAFIDM LDFTELNFLD SLRLLLQSFR LPGEAQKIDR YVLKFAARFM ECKDAAYVLS
YSVILLNTDA HNPQVKKRMT KTDFLKNNRG INDLPEEFLN EIYDDIVHNE IRMKDEVLSL
GRDLQKEAYA LQSSGMANDQ YFSASHFVHV KPMFEVAWMP VLAGISGPLQ DTDDLEIVEL
CLDGFKAFVT TLAKFTFLNN LGEMKSKNME AIKTLLDIAV TDGNQLKGSW HEVLTCVSQL
ERMQLISSAL SRKALKEELA NESRSTHITV AADMVFSLSH YLSGTAIVDF VQALSDVSWE
EIQSSGMSQH PRMFSLQKLV EISYYNMNRI RLEWSNMWVI LGEHFNQVCT HSNVHVAFFA
LDALRQLAMR FLEKEELPHF KFQKDFLKPF EYTMINNANP DVRDMVLQCL HQMIQARVHN
MRSGWRTMFG VFSASARVVT ERIPTTAFEI VTRLYHEHFP DVVRHGSFAD LTVCMGEFCK
VSKDDPMVKF WYPILFAFYD IIMNGEDIEV RRLALDSLFN TLKEHGPTFP VEFWDTICRE
ILFPIFAVLK SKKDFSRFST EGDMSVWLST TMIQALRDLI DLYTFFFDTM ERFLDGLLDL
LSTENDTLAR IGTSCLQQLL EKNAAKLSND KWERVIKTLI GLFKLTTPHQ LYDEKLRADR
KRIFKQIIVK CVLQLLLIET VRDLLQNHDV YRNIPPQLLL RLLSVLEHSY QFARAFNDDK
ELRTGLWKES SSAATLVTIY LRMYNDPRPD YMSLRQPVAD RLLPLGQGVI QDFNKLKIDS
QGKNIAAWSP VVAELLRGFN DFDDQTFTRY LPAIYPLATD LMARDKDIRE SLKSVFTRVG
VAKGI
>EG
DKPREIFEPL RLACETRNEK LMIASLDCIS KLISYSFLVE LVDLVTHTIT SCYTESTPDA
VSLQIVKALL SLVLSPVTLV HHSSLLKAVR TVYNVFLLSQ DPVNQVVAQG GLTQIVNHVF
DLFLKDAFLV FRALCKLTMK PLATESERDL KSHAMRSKLL SLHLVQTILS THMNIFVSPS

SYIYSSSSRE STPFIQATKQ YLCLALSRNA VSPVPQVFEI SVEIFWRVLS GMRKQLKREI
EVLLNEIFIP ILEMRNATPK QKAVLLTMLS RLCQEPQALV EIYLNYDCDK DAIDNIYERL
MNVISKIGTP SLSTTALAGH GKDAVPNEIN LRRLSLECLV FVLRSLVAWG TDDPTRFESA
KQLKTTMMEG IRKFNSKPKG IEFFIDVGFI PTNSAQDIAK FLLETDGLSK AAIGEGDEFN
VSVMHAFTDM LDFGGHDFIG ALRLFLQSFR LPGEAQKIDR YMLKFAAAFM ASQDAAYVLA
YSTIMLNTDQ HNPQVKKRMT PLDFIKNNRG INDLPDEFLN SIFDDIVKNE IRMKDEILSL
GRDLQKEAYV LQSNGMASDQ YFSASHFVHV KPMFEVAWMP VLAGISGPLQ DTDDLEIVEL
CLDGFKAFVT TLGKFTFLNN LGEMKTKNME AIKTLLDIAV TDGNQLKGSW HEVLTCVSQL
ERMQLISSGV SRKQLKDELA NESRSTHITV AADMVFSLSH YLSGTAIVDF VQALSDVSWE
EIQSSGMSQQ PRMFSLQKLV EISYYNMNRI RLEWSNMWVI LGEHFNQVCT HSNPHVAFFA
LDALRQLAMR FLEKEELPHF KFQKDFLKPF EYTMTHNTNP DVRDMVLQCL HQMVQARVHN
MRSGWRTMFG AFSAASKVVT ERIPTTAFDI VTRLYKEHFP SIVRHGSFAD LTVCISDFCK
ASKDDQMIKF WFPILFAFYD IIMNGEDLEV RRLALDSLFS TLKEYGSTFP VEFWDSICQE
TLFPIFAVLK SRQDLSRFHT QEDMSVWLST TMIQALRDLI DLYTFYFDTL ERFLDGLLEL
LGTENDTLAR IGTSCLQQLL ENNAAKLSVD KWERVIKSLI GLFKLTTPHQ LYDEKLRVER
KRIFKQIIVK CVLQLLLIET VRDLLQNQDV YRNIPPQLLL RLLSVLEHSY QFARAFNDDK
ELRMGLWKES SSAATLVTVL LRMYNDPRPD YSPLRQPVAD RLLPLAQGVI QDFNKLKVDS
QGKNITAWSP VVAELLRGFN DFDEQTFTRY LPAIYPLATD LMARDKDIRE ALRAVFTRVG
QAKGI
>SS
DRPRDIFEPL RLACETRNEK LMIASLDCIS KLISHSFFVE LPDLVAHTIT SAYTETTPDS
VSLQIVKALL ALVLSPTVLV HHSSLLKAVR TVYNVFLLSQ DPVNQVVAQG GLQQMINHVF
DLFIKDAFLV FRALCKLTMK ALNTESERDV KSHAMRSKLL SLHLVLTILD SHMNLFVDPR
SNIYSSSSNE VTPFIQATKQ YLCLSISRNA VSLSPQVFEL SVEIFWRVLS GLRTKLKPEI
EVLLNQILIP ILEIRSSTLK QKVVLLDMLH RLCQDPQALV EIYLNYDCDE ESTENIYERL
MNIISKITTP TLSTNALSHT PSHQTLTETQ LKRQSLECLV AVLHSLKAWG TDDPERFESA
KQKKTTLLEG IRKFNIKPKG IQFLLETGLI PSRTPQEIAK FLLYTDGLNK ALIGEGDEEN
IATMHAFVDQ LEFTDVPFTQ ALRLFLQTFR LPGEAQKIDR FMLKFAERYI AGNDTAYVLS
YSVILLNTDA FNPQVKRRMS KQDFIKNNTG IDDLADEYLG VIYDDITSNE IRMKDEVANV
GRDLQKEAYI LQSSNMANEQ FFSASRAVHV RPMFEIAWMS FLAGLSNPLQ DTDDLEIVEI
CLDGFKAFVT TLAKFTFLNN LGEMKTKNME AIKTLLDVAV DEGNYLKGSW HEVLTCVSQL
ERMQLISSGR HKKLPNEDLA NESRSTHITV AADMVFSFSP NLSGAAIVDF VQALSDVSWE
EIQSSGLSDH PRLFSLQKLV EISYYNMKRI RLEWSNLWAI LGEHFNQVCC HSNPNVGFFA
LDALRQLAMR FLEIEELSHF KFQKDFLKPF EYTMIHNPNP SIREMVLTCL HQMIQAKVHN
MRSGWRTMFG VFSAASKVLT EDVVNSAFEL VTRLNRNHFS DIVRYGAFAD LTVCITDFSK
CSKDDPMIKY WFPILFSFYD IIMNGEDLEV RRLALDALFS TLKTYGTSYP VPFWDTVFQE
LLFPIFAVLK SSQDLSRFST QEDMSVWLST TMIQALRDLI DLYTYYFDIL ERVLDGLLDL
LCVENDTLAR IGTSCFQQLL ENNARKLTPE KWERIVTTFV RLFKTTTPHQ LFDESLRVER
RRIFKQIIVK CVLQLLLIET TNELLQNPEV YQTIPPEHLL RLMGELDHSY QFARIFNEDK
ELRTGLWKES SSAATLVNVL TRMYKDTRLD AQQSKQQVAD RLIPLGLGVL EDFNALKADT
QAKTIATWTP VVAEVLQGFN SLDDQTFSRY LPAFYPLTVD ILSRDPGLRE SLRSFFLRVG
FSCGI
>SH
DRPREIFEPL RLACETRNEK LIIASLDCIS KLISYSFFVE LIDLVVHTIT QCHTETTADA
ISLQVVKALL ALVLSSTILV HQSSLLKAVR TVYNVFLMST DPVNQTVAQG GLTQMVHHIF
ELFLKDAFLI FRSMCKLTMK SLVTESEREM KSHGMRSKLL SLHMVLIILN SHMDIFVNPY
SMVYSSSSRD PVPFIQMANQ YLCLTLSRNA VSPVPQVFEV SVEIFWRVLS GLRTKLKKEI
GVLFHEIFIP VLELKTSTLK QKSVILGMLS RLCQDPQALV EIFLNYDCDS KAIDNIYEHL
MNILTKIAAP SLSTSALSVP GTTLGQSEQQ LRRQGLESLV AVLRSLVAWG TDDPSRFESA
KQKKTTLLEG IKKFNFKPKG ISFLIETGFI PSKEPQDIAR FLLNTDGLSK TMIGEGDDEH
IATMHAFVDL IDLRNMPFVD ALRAFLQAFR LPGEAQKIDR FLLKFAERYI EGNDTAYILS
FSVIMLNTDA HNPQVKNRMT KADFVKNNRG INDLPEELLH AIYDDIVNNE IRMKDEVATV
GRDLQREQYM LQSSGMINDQ FYSASHFVHV RPMFEVAWIP FLAGISGPLQ ETDDMETVEL
CLDGFKAFVT TLAKFTFLNN LGEMKAKNME AIKTLLDIAV TEGNNLKGSW HEVLSCVSQL

EHMQLISSGK PRKLPNEELA NESRSTHITV AADMVFSLSG YLSGTAIIDF VQALCDISWE
EIQSSGLSQH PRLFSLQKLV EISYYNMSRI RLEWSNIWDI LGEHFNQCCC HSNPHVGFFA
LDSLRQLAMR FLEKEELTHF KFQKDFLKPF EYTMTHNQNP DIRELVLQCL QQMIQARVQN
MRSGWRTMFG VFSAASKVLT ERITSSAFEI VTRLNKEHFA SIVRYGSFAD LTVCITDFCK
VSKDDPMIKY WFPVLFSFYD VIMNGEDLEV RRLALDSLFS TLKTYGAAFP VDFWDTVCQE
LLFPIFAVLK SSQDLSRFST QEDMSVWLST TMIQALRDLI DLYTYYFEIL ERFLDGLLDL
LCVENDTLAR IGTSCLQQLL ENNVKKLSAA RWERVATTFV KLFKTTTPHQ LFDESLRVER
RRIFKQIIVK CVLQLLLIET TNDLLRNSEV YSTIPPEHLL RLMGVLDHSY QFARMFNEDK
ELRTGLWRES SSASTLVHVL LRMYYDPRPE HQAARPQVAE RLLPLGLGVL QDYTKLRSDT
QAKNIAAWTP VVGEILHGFC RFDDKAFMRY LSAIYPLAAE LIAREPQIRE GLRDYFVRAG
RLQGI
>SC
DRPREIFEPL RLACETRSEK LMIASLDCIS KLISYSFFEE LVDLVVNTIT QCHNESTPES
VSLQIVKALL ALVLSQTILV HHSSLLKAVR TVYNIFLLSN DPVNQTVAQG GLTQMVNHVF
DLFIKDAFLV FRALCKLTMK PLNTESERDL KSHAMRSKLL SLHLVLSILN SHMPLLVDPS
AIIYSSSSHD ATTFVQAINQ YLCLSLSRNA VSPVLQVFEL SVEIFWRVLS GMRTKLKKEI
EVLFREIFMP ILEMKTSTLK QKAIILGMFS RLCQDPQALV EIYLNYDCDR ESADNIYEHL
MNIISKIGSP ALSTQAMSIA GSNMGHSEAQ LKRQGLECLV TVLRSLVAWG TDDPSKFETA
KQRKTTLLEG IKKFNFKPKG IQFLIENGFI PSNSPQDIAA FLLHTDGLSK AMIGEGDEAN
VTIMHAFVDM MDFRGLGFVD ALRTFLQSFR LPGEAQKIDR YMLKFADKYM ANNNAAYVFA
YSVILLNTDA HNPQVKKRMT KADFIKNNRG INALPEDFLT TVYDEIVSNE IRMKDEVANV
GRDLQKEAYV MQSSGMASEQ FFSATQFVHV RPMFEVAWIP FLAGLSGPLQ ETDDIEIVEL
CLDGFKAFVT TLAKFTFLNN LGEMKAKNME AIKTLLDIAL HEGNHLKGSW HEVLTCVSQL
EHMQLLSSGR SKKLPAEALA NESRSTHITV ASDMVFSLSH YLSGTAIVDF VQALCDVSWE
EIQGSGLSQN PRLFSLQKLV EISYYNMNRI RVEWTNIWAI LGEHFNQVCC HNNPHVGFFA
LDALRQLAMR FLEKEELPQF KFQKDFLKPF EYTMVHNQNP EIRDLVLVCL QQMIQARVEN
MRSGWRTMFH VFSAASRVLT ERIVNSAFEI VTRLNKEHFG AIVRHGAFAD LTNCITQFCK
VSKSQPSDHF WHPVLFGFYD IIMTGEDLEV RRLALDSMFS TLKTYGAGFP LEYWDAICSE
LLFPIFSVLK SSQDLSRFST QEDMSVWLST TMIQALRNLI DLYTYYFEIL ERSLDGLLDL
LCVENDTLSR IGTSCLQQLL ENNVKKLSPA RWERVVTTFI KLFKTTTPHQ LFDESLRIER
RRIFKQIIVK CVLQLLLIET TSDLLRNDEV YSTIPPEHLL RLMGVLDQSY RFARDFNEDK
ELRTGLWKES SSAATLIHIL TQMYFDPRPE HRKARPQISE RLLPLGLGVI EDFNKLRQES
QAKNILAWTP VVSEILDCFS RLDDKSFKMY LPAIYPLATH LLDRDPEIRA GLKAYYLRVG
FAQGI
>CP
DRPRAIFEPL RLACETRTEK LMIASLDCIS KLISYSFFAE LVDLVVHTIT SCHTEATPET
VSLQVVKALL ALVLSPTILV HHSSLLKAVR TVYNVFLLSS DPVNQMVAQG GLTQMVHHVF
DFFVKDAFLV FRALCKLTMK PLNTESERDL KSHAMRSKLL SLHLVLTILH SHMIMFTHPQ
AIIYSTSSNE ATSLVQAINQ YLCLSLSRNA VSPVPQVFEV SVEIFWRVLL GMRTKLKKEI
EVLLHEIFIP VIEMRTSTLK QKAVILAMFA RLCQEPQALV EIYLNYDCDS GATDNIYEHL
MNILSKIATT PHSSAMLQVP GSLIGLSEGQ LRRQGLECLV SVLRSLVTWG GDDPSKFESA
KQKKTTLLEG IKRFNYKPKG IQMFIETGWI PSNAPKDIAK FLLTTDGLSK AMIGEADEEN
VAVMHALVDY LDFRNLPFLD ALRMFLQSFR LPGEAQKIDR FMLKFADRYI AGNDAAYILA
YSVILLNTDA HSPQVKNRMT KLDFRKNNRG INDLPEEFLD TIYDQIQSNE IRMKDEVANV
GRDLQKEAYL TQSNGMANAE FFSASHFVHV RPMLEVTWIA FLAGLSGPLQ NTDDLETVEL
CLEGFRAFVT TLGKFTFLNN LGEMKTKNME AIKTLLDIAV NEGNYLKGSW HEVLSCVSQL
EQMQLISSGK GRKLPAEELA NESRSTHITV AADMVFSLSH YLTGTAIVDF VRALCDVSYE
EIKSSGLSQH PRMFSLQKLV EISYYNMNRI RLEWSNLWEI LGEHFNQVCT HDNPSVSFFA
LDSLRQLSMR FLEKEELAHF KFQKDFLKPF EYTMTKNPNP DVRDMVLQCI QQMIQARVQN
MRSGWRTMFG VFSAASKVLT ERIAASAFEM VNSLNNEHFA SIVRHGAFAD LTVCITEFCK
VSKDDVMIRF WFPVLFSFYD IIMNGEDIEV RRLALDSLFS TLKTYGATFP VDFWDTVCQE
LLFPIFAVLK SSQDVSRFST QEDMSVWLST TMIQALRDLI DLYTHYYEIL ERFLDGLLDL
LCVENDTLAR IGTSCLQQFL ENNVSKLSSA RWERVASTFV KLFKTTTPHQ LFDDSLRVER
RRVFKQIIVK CVLQLLLIET TSDLLRNDTI YTTIPPEQLL RLMGVLDHSY QFARMFNDDK

ELRTGLWKES SSAATLVHVL LRMYFDERPE HQAARPQIAE RLLPLGLSVL QDYIKLRADT
QARNIAAWTP VVAEILHGFC RFDNKAFLRY LPAIYPLTTG LLARDPEIRL GLKMYFERVG
YSQGI
>FS
DRPREVFEPL RLACETRSEK LMIASLDCIS KLISYSFFAE LVDLVAHTIT ACHTEVTPET
VSLQIVKALL SLVLSPVVLV HQSSLLKAVR TVYNVFLLST DPVNQMVAQG GLTQMVHHIF
DLFVKDAFLV FRALCKLTMK ALNTESERDL KSHAMRSKLL SLHLVLTVLN SHMALFVDPT
AIIYSSSSNE ATTFVQAINQ YLCLSLSRNA VSPVPQVFEI SVEIFWRVLA GMRTKLKKEI
EVLLHEIFIP ILEMRTSTLK QKAVILGMLS RLCQDPQALV EIYINYDCDS EAVDNIYEHL
MNIISKIGTP ALSTYTLSVS GSTMGLSESQ LKRQGLECLV GVLRSLVAWG IDAASRFESA
KQKKTILVEG VKKFNFNPKG MQFFIESGFI PSNDPPVIAE FLLTTDGLSK AMIGESDEHN
VAVMHAFIDQ LDFKDMTFLQ ALRALLAAFR LPGESQKIDR IVLKFSERYI ANNDCAYVLS
YSTIMLNTDQ HSPQVKKRMD KDEFVRNNRG INDLPEELLH EIYDSIRTNE IRMKDEVANV
GRDLQKEAYV TQSNGMANDQ FFSASDFVHV KTMFEVAWMS FLAGLSGPLQ ETDDLEVVEL
CLDGFKAFVT TLAKFTFLNN LGEMKTKNME AIKALLDIAV TEGNNLKGSW QEVLMCVSQL
EHMQIITSTK GRKLPTEELA NESRSTHITV AADMVFSLSH YLSGTAIVDF VQALTDVSWK
EIQSSGMSQH PRLFSLQKLV EISYYNMSRI RLEWSNLWDI LGEHFNRVCC HNNPHVGFFA
LDSLRQLAMR FLEKEELSHF KFQKDFLKPF EYTMTHNQNP DVRDMVLQCL QQMIQARVQN
MRSGWRTMFG VFSAASKVLT ERIANSAFEI VTRLNKDHFA TIVRYGSFAD LTVCITEFCK
CSKDDPMIRY WFPVLFGFYD IIMNGEDLEV RRLALDSLFS TLKTYGKTYT VEFWDTVCQE
LLFPIFAVLK SSQDLSRFST QEDMSVWLST TMIQALRDLI DLYTFHFDIL ERFLDGLLDL
LLAENDTLAR IGTSCLQQLL EKNVTKLSPA RWERVATTFV KLFRTTTPHQ LFDETLRVER
RRTFKQIIVK CVLQLLLIET TNDLLRNDEV YNTIPPDHLL RLMSVLDHSY QFAREFNEDK
DLRTGLWKES SSAATLVHVL LRMYYDERPE HEAVRPQIAT QLMPLGLGVL QDYTKLKADT
QAKNITAWTP VVAEILEGFC KFDEKSFARY LPAVYPLVTE LLSRDPEIRQ GVRLYFVRVG
HAQGI
>GT
DRPREIFEPL RLACETRNEK LMVTSLDCIS KLISYSFFVE LVDLVVHTIT SCHTETTPET
VSLQIVKALL ALVLSPTVLV HQSSLLKTVR TVYNVFLLST DPVNQMVAQG GLTQMVHHVF
DLFIKDAFLV FRALCKLTMK PLNTESERDL KSHAMRSKLL SLHLVLTVLN SHMALFVAPT
SIIYSSSSHE ATPFVQAVNQ YLCLSLSRNA VSSVPQVFEL SVEIFWRVIS GMRTKLKKEI
EVLLHEIFIP ILEMKTSTLK QKAAILGMLQ RLAQDPQALV EIYLNYDCDS QAADNIYEHL
MNIISKIGTP SLSTTALSVP GSTLGLSDFQ LRKQGLECLV TVLRSLVAWG TDDPSKFESA
RQKKTTLLEG IKKFNSKPKG IEFFIENGFI PSRSPQDIAR FLLHTDGLNK TMIGEGNEEN
IAIMHAFVDM LEFRNLGFVD ALRTFLQTFR LPGEAQKVER FMEKFAERYF TTNGAAFVLA
FSTIMLNTDA HSPQVKSRMS KADFLKNNRG INDLPEEYLS ALYDDIVANE IRMKDEIANV
GRDLQREAYL MQSSGMANDQ FFSASHAVHV RPMFEAAWMP FLAGLSGPLQ DTDDPEVIEL
CLDGFKAFVT TLAKFTFLNN LGEMKSKNME AIKTLLDVAV SEGNYLKSSW HEVLTCVSQL
ERMQLISSGR PRMLPTEELA NESRSTHITV AADMIFSLSH YLNGTAIVDF VQALCDVSWD
EIQSSGLSPR PRLFSLQKLV EISYYNMNRM RLEWLSLWDI LGQHFNQVCC HSNPNVGFFA
LDSLRQLAMR FLEKEELPHF TFQKDFLKPF EYTMVHNANP DVRDMVLQCL SQMIQARVHN
LRSGWRTLFG VLSAASKVLT ERIVSSAFEI VTRVNRDHFS AIARHGAFAD LTVCITDFCK
VSKDDPMIRF WFPVLFGFYD IIMNAEDLEV RRLALDSLFT TLKTYGPDYP IDFWDTVCQE
LLFPIFAVLK SSQDLSRFNS QEDMSVWLQS TMIQALRDLI DLYTYLFDIL ERFMDGLLEL
LCVENDTLAR IGTSCLQQLL EHNVKKMSPA RWDRITATFV KLFRTTTPHQ LFDESLRVDR
RRIFKQIIVK CVLQLLLIET LNDLLRNDLV YSTIPPEHLL RMMGVLDHSY QFARSFNEDK
DLRMALWKES SSASTLVHVL LRMYYDPRPE HQAARPQIAE RLLPLELGVL QDYNRLRLDT
QAKNIAAWTP VVAEILQGFC RFDDKAFLRY MPAVYPLATE LLSRGDEIRE GLKDYYMRVG
YAQRI
>PS
DKPREIFEPL RLACETRNEK LMIASLDCIS KLISYSFFVD LVDLVVHTIT ACHTETTPDP
VSLQIVKALL AIVLSSTTLV HQSSLLKAVR TVYNVFLLST DPITQTVAQG GLTQMVHHVF
DLFVKDAFLV FRALCKLTMK PLNSESERDL KSHSMRSKLL SLHLVLTVLN SYMPLFVSPS
ALIYSSSSHE ATPFVQAVNQ YLCLSLSRNA VSPVPQVFEL SVEIFWRVLS GMRTKLKKEI

EVLLHEIFIP IIEMKSSTLK QKGVILGMFY RLCQDPQALV EIYLNYDCDS EASDNIYEHI
ANLISKIATP SYTTTSLAVS GSTVGLSERQ LKRQGLECLV AILKSLVVWG TDDPNRFESA
RQKKTTLLEG VKKFNMKPKG VEFFLETGFI PNRNPQDIAK FLLYTDGLSK AMIGEGDEQN
IAIMHAFVDL LDFKDLSFVD ALRLFLQSFR LPGEAQKIDR YMLKFAERYI AGNDAAYVLA
YSTVMLNTDQ HNPQVKKRMT KADFIKNNRG INDLPEEYLS LVFDEIASNE IRMKDEVANV
GRDLQREAYI MKSHGMANDQ FFSASHFVHV RPMFEVAWIP FLAGLSNPLQ DTDDLEIVEL
CLDGFKAFVT TLAKFTFLNN LGEMKAKNME AIKALLDIAV TDGNNLKGSW REVLQCVSQL
EHMQLITSGR LRKLPAEELA NESRSTHITV AADMVFSLSH YLSGTAIVDF VRALCDVSWE
EIQSSGLSQH PRLFSLQKLV EISYYNMNRI RLEWSNLWDI LGEHFNQVCC HNNPHVGFFA
LDSLRQLAMR FLEKEELPHF KFQKDFLKPF EYTMVHNANP EIRDMVLQCL QQMIQARVQN
LRSGWRTMFG VFTAAAKVLT ERIVNSAFEI VTRLNKEHFS AIVRHGSFAD LTVCITDFCK
ASKDDPMIKF WFPVLFGFYD IIMNGEDLEV RRLALDSLFT TLKTYGSSYP VAFWDTVCQE
LLFPIFAVLK SSQDLSRFST QEDMSVWLST TMIQALRDLV DLYTFHFDIL ERFLDGLLDL
LCVENDTLAR IGTSCLQQLL ENNVRKLSPA RWERVVTTFI RLYKTTTPHQ LFDESLRTER
RRVFKQIIVK CVLQLLLIET TNDLLRSKQV YDTIPPEQLL RLMAVLDHSY QFARMFNEDK
ELRTGLWKES SSASTLVHIL LQMYYDPRAD HRSARPQIAD KLLPLGMGVL QDYSKLRPDT
QAKNIAAWTP VVAEILHGFS RFDEKAFSRY LPAVYPIAVD ILARDPEIRE GLRSYFSRVG
YVQGI
>LB
DRPREIFEPL RLACETKNEK LTVASLDCIS KLISYSFFAE LVDLVAHTIT SCHTETTPDP
VSLQIVKALL SLVLSPTIIV HHSSLLKAVR TVYNVFLLSS DPVNQMVAQG GLTQMVHHVF
DLFIKDAFLV FRALCKLTMK PLNTESERDP KSHPMRSKLL SLHLVLTVLN SHMSLFVDPT
AIIYSSSTNE ATNFVQAINQ YLCLSLSRNA VSPVPQVFEI SVEIFWRVLA GMRTKLKKEI
EVLLHEIFIP ILEMRTSTLK QKAVIIGMLS RLCQDPQALV EIYLNYDCDS EAADNIYEHL
MNIISKFGTI SWSNSGLAIS GSTMGLSDAQ LRRQGLECLV AVLRSLVVWG TDDPSKFESA
KQKKTTLMEG IKKFNFKPKG IQFLLEAGFI ASKDPRDIAT FLLTTDGLSK SMIGEGDEEN
ISIMHAFVDQ LDFKDHPFID ALRIFLQSFR LPGEAQKIDR YMLKFADRYI AGNDTAYVLA
YSVIMLNTDA YNPQVKKRMT KTDFIKNNRG INDLPEELLS EIFEDIANNE IRMKDEVASV
GRDLQKEAYV MQSNGMANDQ FFSASHFVHV RPMFEVAWIP FLAGLSGPLQ ETDDLEVVEL
CLDGFKAFVT TLAKFTFLNN LGEMKTKNME AIKALLDVAV TEGNNLKGSW HEVLTCVSQL
EHMQLISSGR THKLPTEELA NESRSTHITV AADMVFSLSH FLSGTAIVDF VQALCDVSWE
EIQSSGLSQH PRLFSLQKLV EISYYNMTRI RLEWSNLWDI LGEHFNQVCC HNNPHVGFFA
LDALRQLAMR FLEKEELAHF KFQKDFLKPF EYTMVHNQNP EIRDMVLQCL QQMIQARAQN
MRSGWRTMFG VFSAASRVLT EHIASSAFEI VTRLNKDHFP AIVRYGAFAD LTVCITEFSK
VSKDDEMIRY WFPVLFGFYD IIMNGEDLEV RRLALDSLFT TLKTYGSTYP LEFWDTVCQE
LLFPMFAVLK SSQDLSRFNT QEDMSVWLST TMIQALRDLI DLYTFYFDIL ERFLDGLLDL
LCVENDTLAR IGTSCLQQLL ENNMTKLSST RWERVTTTFV RLFRTTTPHQ LFDDNLRVER
RRTFKQIIVK CVLQLLLIET TNDLLRNENV YNTIPPEQLL RLMGVLDHSY QFARMFNDDK
ELRTGLWKES SSAATLVHVL LRMYYDSRPE HQAARPQIAE RLLPLGLGVL QDYNKLRPDT
QSKNIIAWTP VVAEILDGFC RFDDKAFSRY LPAIYPLAID LLSREAEVRL ALKTYFVRVG
YAHGI
>PI
DRPREIFEPL RLACETRNEK LMIASLDCIS KLISYSFFAE LVDLVAHTIT ACHTETTPET
VSLQIVKALL SLVLSPVILI HQSSLLKAVR TVYNVFLLST DPVNQMVAQG GLTQMVHHVF
DLFIKDAFLV FRALCKLTMK ALNNESERDL KSHAMRSKLL SLHLVLTVLN SHMPLFVDPS
AIIYSSSSNE ATTFVQAINQ YLCLSLSRNA VSPVPQVFEI SVEIFWRVLS GMRTKLKKEI
EVLLHEIFIP ILEMRTSTLK QKAVILGMLS RLCQDPQALV EIYLNYDCDS EAVDNIYEHL
MNIISKIGTP SLSTYSLSVS GSTMGLSEHQ LKRQGLECLV AVLRSLVAWG TDDPSRFESA
KQKKTTLLEG IKKFNYKPKG VQFLIETGFI PSKDPSTIAY FLLTTDGLSK AMIGEAEDEN
IATMHAFVDQ LDFKDLSFLD ALRIFLQAFR LPGEAQKIDR FMLKFAERYI AGNDTAYVLA
YSTIMLNTDA HSPQVKKRMT KADFVKNNRS INDLPEELLH SIFDDIVSNE IRMKDEVANV
GRDLQKEAYV MQSNGMANDQ FFSASHFVHV RPMFEVAWMS FLAGLSGPLQ ETDDLEVVDL
CLEGFKAFVT TLGKFTFLNN LGEMKTKNME AIKTLLDIAV TEGNSLKGSW QEVLTCVSQL
EHMQIITSGK TRRLPTEELA NESRSTHITV AADMVFSLSH YLSGTAIVDF VQALTDVSWK

EIQSSGLSQN PRLFSLQKLV EISYYNMNRI RLEWSNLWDI LGEHFHKVCC HNNPHVGIFA
LDSLRQLAMR FLEKEELPHF KFQKDFLKPF EYTMIHNPNP DIRDMVLQCL QQMIQTRVQN
MRSGWRTMFG VFSAASKVLT ERIANSAFEI VTRLNKDHFA AIVHYGSFAD LTVCITEFCK
VSKEDNMIRY WFPVLFGFYD IIMNGEDLEV RRLALDSLFS TLKTYGSTYT VEFWDSVCQE
LLFPIFAVLK SSQDLSRFST QEDMSVWLST TMIQALRDLI DLYTFHFAIL ERFLDGLLDL
LCVENDTLAR IGTSCLQQLL ENNVAKLSPA RWDRVATTFV KLFRTTTPHQ LFDETLRVER
RRIFKQIIVK CVLQLLLIET TNDLLRNDEV YNTIPPEHLL RLMSVLDHSY QFAREFNEDK
DLRTGLWKES SSAATLVHVL LRMYYDNRPE HEAARPQIAK QLMPLGLGVL RDYSKLRADT
QVKNIAAWTP VVAEILEGFC RFDDKAFARY LPAIYPLATD LLSREPEIRQ GLKMYFVRVG
YVHSI
>HI
DRPREIFEPL RLACETRNEK LMVASLDCIS KLISYSFFVE LVDLVVHTIT TCHTETTADS
VSLQVVKALL ALVLSSTILV HQSSLLKAVR TVYNVFLMST DPVNQTVAQG GLTQMVHHIF
DLFLKDAFLV FRSMCKLTMK PLNTESERDL KSHAMRSKLL SLHMVLVILN AHMDVFVSPS
SLIHSSSSHE ATPFVQMANQ YLCLSLSRNA VSPVPQVFEI SVEIFWRVIT GLRTKLKKEI
EVLFHEIFFP ILEMKTSTLK QKAVILGMLS RLCHDPQALV EIYLNYDCDS QAADNIYEHL
MNIITKIGTP SLATSAVSVS GSALGHSEQQ LRRQSLECLA AVLRSLVAWG TDDPGRFESA
KQKKTTLLEG VKKFNFKPKG IQFLLETGFI PSKAPQDVAR FLLQTDGLSK SMIGEGDEEN
IATMHAFVDM LDLRNMPFVD ALRVYLQAFR LPGEAQKIDR FMLKFAERYI EGNDTAYVLS
YSVILLNTDA HNPQVKKRMT RADFVKNNRG INELPEELLS VIFDDIVNNE IRMKDEVATV
GRDLQREAYM LQSNGMANEQ FFSASHFVHV RPMFEVAWIP FLAGISGPLQ ETDDLEVVEL
CLDGFRAFVT TLGKFTFLNN LGEMKTKNME AIKTLLDVAV TEGNNLKGSW REVLSCVSQL
EHMQLISSGR TRKLPNEELA NESRSTHITV AADMVFSLSH YLSGTAIVDF VQALCDVSWE
EIQSSGLSQH PRLFSLQKLV EISYYNMSRI RLEWSNLWDI LGEHFNQVCC HSNPHVGFFA
LDALRQLAMR FLEKEELPHF KFQKDFLKPF EYTMIHNANP DIRDMVLQCL QQMIQARVQN
MRSGWRTMFG VFSAASKVLT ERITSSAFEI VTRLNKEHFP AIVRYGSFAD LTVCITDFCK
VSKDDPMIKY WFPVLFSFYD VIMNGEDLEV RRLALDSLFS TLKKYGATFP VDFWDTVCQE
LLFPIFAVLK SSQDLSRFST QEDMSVWLST TMIQALRDLI DLYTFYFEIL ERFLDGLLDL
LCVENDTLAR IGTSCLQQLL ESNVRKLSPA RWERVATTFV KLFRTTTPHQ LFDESLRVER
RRIFKQIIVK CVLQLLLIET TNDLLRNDEV YNTIPPEHLL RLMGVLDHSY QFARLFNEDK
ELRTGLWKES SSASTLVHVL LRMYYDPRAE HQAARPQVAE RLLPLGLGVL QDYSKLRSDT
QAKNIAAWTP VIGEILRGFC RFDDKAFTRY LPAIYPLAAD LIARDPEIRE GLRDYFVRVG
YAQGI
>RF
DRPREIFEPL RLACETRNEK LMIASLDCIS KLISHSFFID LVDLVTSTIT ACHMETTPDT
VSLQIVKALL SLVLSSTLLV HQSSLLKAVR TVYNIFIMSH DPVNQTVAQG GLTQMVNHVF
DMFIKDAFLV FRALCKLTMK PLNTESERDF TSEGMRSKLL TLHLVLTVLN SHMAVFAEPW
SIIYSTSSNE TTTFINATKQ YLCLSLSRNA LSPVPQVFEI SVELFWRVLS GLRTKLKKEI
EVFFHEIFVP ILEMKTSTLK QKGVILGMLL RLCQEPQAVV EIYLNYDCDR EAADNIYEHL
MNTISKIATP SLSTAALAVP GAGLGFSESQ LKRQGLECLV AVLRSLVAWG TDDPGKFVSA
KQRKTTLLEG IKKFNFKPKG IEFLVDTGFI PTRSPYEIAK FLLQTDGMNK TMIGEGDDEN
IAIMHAFVDQ LDFTNLPFVD ALRVFLQSFR LPGEAQKIDR FMLKFAERYI AGNDCAYVLS
YSTIMLNTDA HNPQVKNRMT KLDFIRNNRG INDLPEDFLS AIFDDILSNE IRMKDEVANV
GRDLQKEAYV MQSHGMANDQ FFSASHFIHV RPMFEVAWIP FLAGISGPLQ DTDDMEVVEL
CLDGAECFVT TLAKFTFLNN LGEMKTKNME AIKTLLDIAV TEGNYLKGSW HEVLTCVSQL
ERMQLISSGR SRKLPAEELA NESRSTHITV AADMVFSLSH YLSGDAIVDF VKALSSVSWE
EIQSSVASEH PRLFSLQKLV EISYYNMNRI RLEWSHLWDI LGEHFNQVCC HHNPHVGFFA
LDALRQLAMR FLEKEELPHF KFQKDFLKPF EYTMTHNANP DIRDMVLQCI QQMIQARVQN
MRSGWRTMFG VFSAASKVLT ERVVNSAFEI VSRLNEDHFS AVVRYGAFAD LTVCITDFCK
VSKDDPMIKF WFPVLFSFYD IIMNGEDLEV RRLALDSLFS TLKTYGSSFP VEFWDTVCQE
LLFPIFAVLK STSDLTRWTT QEDMSVWLST TLIQALRDLI DLYTYFFDTL ERFLDGLLEL
LCVENDTLAR IGTSCLQQLL ESNVKKLSPT RWERVTSTFV KLFKTTTPHQ LFDDSLRAER
RRIFKQIIVK CVLQLLLIET TNELLQNDEV YNTIPPEHLL RLMGVLDHSY QFARVFNEDK
DLRTALWKET SSAATLVNIL ARMYYDTRPE YQVLRPQVVD KLLPLGLGVM QDFNKLRPET

HAKNIASWTP VIAEILNGFC RFDDKAFSRY MPVIYPPAAE LLTKDAEVRL GLRDFFTRVG
RAQGI
>RI
DRPREIFEPL RLACETRNEK LMIASLDCIS KLISHSFFID LVDLVTSTIT ACHTETTPDT
VSLQIVKALL SLVLSSTLLV HQSSLLKAVR TVYNIFIMSH DPVNQTVAQG GLTQMVNHVF
DMFIKDAFLV FRALCKLTMK PLNTESERDF TSEGMRSKLL TLHLVLTVLN AHMAVFAEPW
SIIYSTSSNE TTTFINATKQ YLCLSLSRNA LSPVPQVFEI SVELFWRVLS GLRTKLKKEI
EVFFHEIFVP ILEMKTSTLK QKGVILGMLL RLCQEPQALV EIYLNYDCDR EAADNIYEHL
MNTISKIATP SLSTAALAVP GAGLGFSESQ LKRQGLECLV AVLRSLVAWG TDDPGKFVSA
KQRKTTLLEG IKKFNFKPKG IEFLVDSGFI PTRSPYEIAK FLLQTDGMNK TMIGEGDDEN
IAIMHAFVDQ LDFTNLPFVD ALRVFLQSFR LPGEAQKIDR FMLKFAERYI AGNDCAYVLS
YSTIMLNTDA HNPQVKNRMT KLDFIRNNRG INDLPEDFLS AIFDDILSNE IRMKDEVANV
GRDLQKEAYV MQSHGMANDQ FFSASHFIHV RPMFEVAWIP FLAGISGPLQ DTDDMEVVEL
CLDGFKAFVT TLAKFTFLNN LGEMKTKNME AIKALLDIAV TEGNYLKGSW HEVLTCVSQL
ERMQLISSGR SRKLPAEELA NESRSTHITV AADMVFSLSH YLSGDAIVDF VKALSSVSWE
EIQSSVASEH PRLFSLQKLV EISYYNMNRI RLEWSHLWDI LGEHFNQVCC HHNPHVGFFA
LDALRQLAMR FLEKEELPHF KFQKDFLKPF EYTMTHNANP DIRDMVLQCI QQMIQARVQN
MRSGWRTMFG VFSAASKVLT ERVVNSAFEI VSRLNEDHFS AVVRYGAFAD LTVCITDFCK
VSKDDPMIKF WFPVLFSFYD IIMNGEDLEV RRLALDSLFS TLKTYGSSFP VEFWDTVCQE
LLFPIFAVLK STSDLTRWTT QEDMSVWLST TLIQALRDLI DLYTYFFDTL ERFLDGLLEL
LCVENDTLAR IGTSCLQQLL ESNVKKLSPT RWERVTTTFV KLFKTTTPHQ LFDDSLRAER
RRIFKQIIVK CVLQLLLIET TNELLQNDEV YNTIPPEHLL RLMGVLDHSY QFARVFNEDK
DLRTALWKET SSAATLVNIL ARMYYDTRPE YQALRPQIVD KLLPLGLGVM QDFNKLRPET
HAKNIASWTP VIAEILNGFC RFDDKAFSRY MPVIYPPAAE LLTKDVEVRL GLRDFFTRVG
RAQGI
>DS
DKPREIFEPL RLACETRNEK LMIASLDCIS KLISYSFFVE LVDLVVHTIT SCHTESTPET
VSLQIVKALL SLVLSSTVLV HQSSLLKAVR TVYNVFLLSA DPVNQMVAQG GLTQMVNHVF
DLFIKDAFLV FRALCKLTMK PLNSESERDL KSHSMRSKLL SLHLVLMILN SHMHIFASPS
AIIYSSSSNE ATPFIQAASQ YLCLCLSRNA VSPVPQVFEI SVEIFWRVVS GLRTKLKKEI
EVLLHEIFIP ILEMKTSTLK QKAMIVSMLQ RLCQDPEALV EIYLNYDCDS EAADNIYEHF
MNIISKIGTP SFSTAALSVP GSTIGNSEAQ LRRQGLECLV AALKSLVAWG TDDPSKFESA
KQKKTTLLEG IKKFNFKPKG VDFFLETGFI PSREPKDIAR FLLETDGLSK VAIGEGDAEN
IAIMHAFVDM LDLSNMPFVD ALRQFLQAFR LPGEAQKIDR FMLKFAERYM SGNDTAYVLA
FSTIMLNTDA HSRQVKNRMT KQGFIANNRG INDLPEEFLS AIYDDITTNE IRMKDEIATV
GRDLQKEQYM MQSNNMANEQ FFSASHFIHV RPMFEVAWIP FLAGLSGPLQ DTDELEIVEL
CLEGFKAFVS TLTKFTFLNN LGEMKTKNME AIKTLLDVAV TEGNQLKASW RDVLTCVSQL
EHMQLISSGR SRKPPTEELA NESRSTHITV AADMVFSLSH YLSGTAIVDF VRALCDVSWE
EIQSSGMSQH PRLFSLQKLV EISYYNMNRI RLEWSNMWEI LGEHFNQVCC HKNPHVGFFA
LDALRQLAMR FLEKEELPHF KFQKDFLRPF EYTMIHNSNP DIRDMVLQCL QQMIQARVHN
LRSGWRTMFA VFSAASKAAT ERIASSAFEI VTRLNKEHFP SIVRHGSFAD LTVCITDFCK
VSKDDPMIKY WFPVLFGFYD VIMNGEDLEV RRLALDSLFN TLKTYGSTYP VEFWDTVCQE
LLFPIFAVLK SSQDLSRFST QEDMSVWLST TMIQALRNLI DLYTFYFETL ERFLDGLLDL
LCVENDTLAR IGTSCLQQLL ENNVKKLSAA RWERIALTFV KLFRTTTPHQ LFDESLRVER
RRIFRQIIVK CVLQLLLIET TNDLLRNDEV YNTIPPEHLL RLMGVLDHSY QFARMFNDDK
ELRTGLWKES SSASTLVHVL LRMYYDPRPE HQSARPQVAD RLLPLGLGVL QDFNKLRLDS
QAKNIAAWTP VVAEILQGFV KFDDKAFTRY LPAIYPLATD LLSREPEIRE GLREYFLRVG
YIQGI
>TV
DRPREIFEPL RLACETRNEK LMIASLDCIS KLISYSFFAE LVDLVVHTIT SCHTENTPET
VSLQIVKALL ALVLSPTILV HQSSLLKAVR TVYNIFLLSL DAVNQMVAQG GLTQMVNHVF
DMFIKDAFLV FRALCKLTMK PLNSESERDL KSHAMRSKLL SLHLVLMILN SHMPIFVSPS
AIIYSSSSHE ATPFIQAASQ YLCLSLSRNA VSPVPQVFEI SVEIFWRVVA GLRTKLKKEI
EVLLHEIFIP ILEMKTSTLK QKAVILSMLQ RLCQEPQALV EIYLNYDCDG EAVDNIYEHL

MNIISKIGTP SFSTASLSVP GSTIGNSEAQ LRRQGLECLV AVLKSLVSWG TDDPTKFESA
KQKKTTLLEG IKKFNFKPKG IEFLIETGFI ASREPKDIAR FLLETDGLNK AAIGEGDEEN
ITIMHAFVDT MDLGNMPFVT ALRTFLQAFR LPGEAQKIDR YMLKFAERYI ATNDTAYVLA
YSTILLNTDA HNPQVKNRMT KQGFIANNRG INDLPEDLLN AIYDEIVSNE IRMKDEVANV
GRDFQKEAYV MQSNNMASEQ FFSASHFVHV RPMFEVAWIP FLAGISGPLQ DTDDIEVVEL
CLEGFKAFVS TLAKFTFLNN LGEMKTKNME AIKTLLDVAV TEGNHLKASW REVLTCVSQL
EHMQLLSSGR VRKPPTEELA NESRSTHITV AADMVFSLSH YLSGTAIVDF VRALCDVSWE
EIQSSGLSQH PRLFSLQKLV EISYYNMNRI RLEWSNMWDI LGEHFNQVCC HKNPHVGFFA
LDALRQLAMR FLEKEELAHF KFQKDFLRPF EYTMIHNSNP DVRDMVLQCL QQMIQARVHN
LRSGWRTMFA VFSAASKAAT ERIANSAFEI VVRLNKEHFS SIVRHGSFAD LTVCITDFCK
VSKDDPMIKY WFPVLFGFYD VIMNGEDLEV RRLALDSLFS TLKKYGSTYP LEFWDTVCQE
LLFPMFAVLK SSQDLSRFST QEDMSVWLST TMIQALRNLI DLYTFYFETL ERFLDGLLDL
LCVENDTLAR IGTSCLQQLL ESNVKKLSPA RWERVATTFV KLFRTTTPHQ LFDESLRVER
RRIFRQIIVK CVLQLLLIET TNDLLRNDEV YNTIPPEHLL RLMGILDHSY QFARMFNEDK
ELRTGLWKES SSASTLVHVL LRMYYDPRPD HQAARPQVAD RLMPLGLGVL QDFNKLRLDT
QAKNIAAWTP VVAEILQGFV RFDDKAFTRY LPAVYPLATD LLSREPEIRE GLREYFLRVG
YIQGI
>PC
DRPREIFEPL RLACETRNEK LMIASLDCIS KLISYSFFVE LVDLVVHTIT SCHSESTPET
VSLQIVKALL ALVLSPTILV HQSSLLKAVR TVYNVFLLSV DPINQTVAQG GLTQMVNHVF
DLFIKDAFLV FRALCKLTMK PLNAESERDL KSHAMRSKLL SLHLVLTILN NHMALIVSPN
SIIYSSSSND STTFVQAITQ YLCLCLSRNA VSPVPQVFEA SVEIFWRVIS GMRTKLKKEI
EVLLHEIFIP ILEMKTSTLK QKAVILNMLQ RLSQDPQALV EIYLNYDCDS EAVDNIYEHL
INIVSKLATP ALSTNALSGP GSTLGTSESQ LRRQSLECLV STLKSLVTWG TDDPTKFESA
KQKKTTLLEG IKKFNFKPKG IQFFLETGFI PSPAPQDVAR FLLETDGLSK AMIGEADEEN
VATMHAFVDL MDFRGLEFVD ALRVFLQAFR LPGESQKIDR YMLKFAERYI AGNDTAYVLA
YSTIMLNTDA HNPQVKSRMT KADFIKNNRG INDLPEELLS SIYDDIVNNE IRMKDEVANV
GRDLQKEAYM MQSNNMANEQ YFNASHFVHV KPMFEVAWIP FLAGLSGPLQ GTDDLEIVEL
CLDGFKAFVT TLAKFTFLNN LGEMKTKNME AIKALLDVAV TEGNNLKSSW REVLTCVSQL
EHMQLITSGR PRKLPTEELA NESRSTHITV AADMVFSLSH YLSGTAIVDF VRALCDVSWE
EIQSSGMSQH PRLFSLQKLV EISYYNMNRI RLEWSNLWDI LGEHFNQVCC HNNPHVAFFA
LDALRQLAMR FLEKEELPHF KFQKDFLRPF EYTMVHNSNP EVRDMVLQCL QQMIQARVAN
LRSGWRTMFG VFSSAAKVPT ERIVSSAFEI VTRLNKDHFQ AIVRHGAFAD LTVCITDFCK
VTKDDPMIKY WFPTLFGFYD VIMNGEDLEV RRLALDSLFS TLKTYGSTFP VEFWDTVCQE
LLFPIFAVLK SSQDLSRFST QEDMSVWLSS TMIQALRNLI DLYTFHFEIL ERFLDGLLDL
LCVENDTLAR IGTSCLQQLL ESNVKKLSPA RWERVATTFV KLFRTTTPHQ LFDESLRVER
RRIFKQIIVK CVLQLLLIET TNDLLRNEEV YNTIPPEHLL RLMGVLDHSY QFARAFNEDK
ELRTGLWKES SSASTLVHIL LRMYYDPRPE HQASRPQVAD RLLPLSLGVL QDFTKLRLDS
QAKNIAAWTP VIAEILHGFA KFDDKAFATY LPAIYPLATE LLAREPEIRQ NLRDYFLRVG
YIQGI
>SL
DRPREIFEPL RLACETRNEK LMIASLDCIS KLISYSFFAE LVDLVVHTIT ACHSENTPET
VSLQVVKALL SLVLSPTIFV HHSSLLKAVR TVYNVFLLST DPVNQMVAQG GLTQMVHHLT
DLFVKDAFLV FRALCKLTMK PLNSESERDI KSHAMRSKLL SLHLVLTVLN SHMPLFNDPS
AIIYSSSSND DTMFIQAINQ YLCLSLSRNA VSPVPQVFEV SVEIFWRVLS GMRTKLKKEI
EVLLHEIFIP ILEMKTSTLK QKAVILGMLS RLCQDPQALV EIYLNYDCDS EAVDNIYEHL
MNIISKICTP SLNSTSLSVS GSMMGLSEAQ LRRQGLECMV AVLRSLVSWG TDDPTKFESA
KQKKTTLLEG IKKFNFKPKG VQFLIETGFI PSRAPRDVAQ FLLTTDGLAK AMIGEGEEEN
IATMHAFVDL LDFRNLPFID ALRIFLQAFR LPGEAQKIDR FMLKFAERYI AGNDTAYVLA
YSTILLNTDA HSPQVKNRMT KSDFYKNNRG INDLPEEFLS TIYDDIVKNE IRMKDEIANV
GRDLQKEAYM LQSNGMANDQ FFSASHFVHG RPMFEVAWIP FLAGLSGPLQ NTDDLEIVEL
CLDGFKAFVT TLGKFTFLNN LGEMKAKNME AIKTLLDVAV TEGNSLKGSW REVLTCVSQL
EHMQLISSGR SRKLPAEELA NESRSTHITV AADMVFSLSH YLSGTAIVDF VQALCDVSWE
EIQSSGLSQH PRLFSLQKLV EISYYNMNRI RLEWSNLWDI LGEHFNQVCC HNNPHVGFFA

LDSLRQLSTR FLEKEELPHF KFQKDFLKPF EYTMTHNANP DIRDMVLQCL QQMVQARVQN
MRSGWRTMFG VFSAASRVLT ERIASSAFEI VTRLNEDHFA AIVRHGAFAD LTVCITDFCK
VSKDDTMIRF WFPVLFGFYD IIMNGEDLEV RRLALDSLFT TLKTYGSTYP VEFWDTVCQE
LLFPIFAVLK SSQDVSRFST QEDMSVWLST TMIQALRDLI DLYTFYFDIL ERFLDGLLDL
LCVENDTLAR IGTSCLQQFL ENNVTKLNPS RWERVATTFV RLFRTTTPHQ LFDDNLRVER
RRIFKQIIVK CVLQLLLIET TNDLLRNDAV YNNIPPEQLL RLMGVLDHSY QFARMFNDDK
ELRTGLWKES SSAATLVHVL LRMYFDERPE HQAARPQIAE RLLPLGLSVL QDYTKLRSDT
QAKNITAWTP VVAEILEGFC RFDNKAFVRY LPAIFPLTTG LLARDPEIRL GLKMYFERVG
YSQGI
>FP
DRPREIFEPL RLACETRNEK LMVASLDCIS KLISYSFFVE LVDVVVHTIT SCHSETTPDP
VSLQIVKALL ALVLSSTILV HGSSLLKAVR TVYNVFLLSS DPVNQTVAQG GLTQMVNHVF
DLFIKDAFLV FRALCKLTMK PLNSESERDL KSHAMRSKLL SLHLVLTILN THMAIFVSPS
AIIYSSSSRE ATSFVQAVNQ YLCLCLSRNA VSPVPQVFEI SVEIFWRVIS GLRTKLKKEI
EVLLHEIFIP ILEMKTSTLR QKVVILNMIY RLCQDPQVLV EIYLNYDCDS EAVDNIYEHL
MNIISKIGTP SLSTTALAGP GSAAGLTEQQ LKRQGLECLV AVLKSLVVWG TDDPTKFESA
KQKKTTLLEG IKKFNFKPKG IQFFLETGFL PSKSPKDIAK FLLETDGLSK AMIGEGEEEN
IAIMHAFVDM MEFKDLAFVD ALRTFLQAFR LPGEAQKIDR FMLKFAERYI GGNDAAYVLA
YSVILLNTDA HNPQVKRRMT LADFIKNNRG INDLPEELLS TIYDDIVSNE IRMKDEVANV
GRDLQKEAYM MQSNSMTNEQ FFSASHFTHV RPMFEVAWIP FLAGISGPLQ DTDDLEIVEL
CLDGFKAFVT TLSKFTFLNN LGEMKTKNME AIKALLDVAV TDGNNLKGSW REVLTCVSQL
EHMQLISSSR SRKPPTEELA NESRSTHITV AADMVFSLSH YLSGTAIVDF VRALCDVSWE
EIQSSGLSQH PRLFSLQKLV EIAYYNMNRI RLEWSNLWDI LGEHFNQVCC HNNPHVGFFA
LDALRQLAMR FLEKEELPHF KFQKDFLRPF EYTMIHNSNP DIRDMVLQCL QQMIQARVGN
MRSGWRTMFG VFSAASKVPT ERVANSAFEL VTRVNKEHFS AIVRNGAFAD LTVCITDFCK
VNKDDPMIKF WFPVLFGFYD VIMNGEDLEV RRLALDSLFS TLKTYGKTFP VDFWDTVCQE
LLFPIFAVLK SSQDMTRFNT QEDMSVWLST TMIQALRNLI DLYTFYFETL ERFLDGLLDL
LCVENDTLAR IGTSCLQQLL ENNVKKLSPA RWERVATTFV KLFRTTTPHQ LFDENLRVER
RRIFRQIIVK CVLQLLLIEM TNDLLRNDEV YNTMPPEHLL RLMGVLDHSY QFARMFNEDK
DLRTGLWKES SSASTLVHVL LRMYYDTRSE HQAARPQVMD RLMPLGLGVL GDFNKLRVDT
QLKNIVAWTP VVAEIMQGFV RLDDKAFGRY MPAIYPLATD LLSRDPEVRD RLREYFKRVG
YIQGI
>PP
DRPREIFEPL RLACETRNEK LMIASLDCIS KLISYSFFVE LVDVVVHTIT SCHSENAPET
VSLQIVKALL ALVLSSTILV HQSSLLKAVR TVYNVFLLST DPVNQTVAQG GLTQMVHHVF
DLFIKDAFLV FRALCKLTMK PLNSESERDL KSHGMRSKLL SLHLVLTILN SHMALFVSPS
AVIYSSSTHE ATSFVQAVNQ YLCLCLSRNA VSPVLQVFEI SVEVFWRVIS GLRTKLKKEV
EVLMHEIFIP ILEMKTSTLK QKAIILGMLQ RLCQDPQVLV EIYLNYDCDS EAVDNIYEHL
MNIISKIGTP SLSTTALTGP GSQPVLSEQQ LRRQGLECLV AVLKSLVAWG TDDPSKFESA
KQKKNTLLEG VKRFNTKPKG IQFFIETGFI PSNSSQDIAR FLHETDGLNK AMIGEGDEEN
IVIMHAFVDQ MDFRNLPFVD ALRTFLQGFR LPGEAQKIDR FMLKFAERYI AGNDAAYILA
YSTILLNTDA HNPQVKRRMS LQDFIKNNRG INDLPEDFLT SIYQSIVTNE IRMKDEVANV
GRDLQKEAYV MQSNNMTNEH FFSASHFVHV RPMFEVAWIP FLAGLSGPLQ DTDDLDIVEI
CLDGFKAFVT TLAKFTFLNN LGEMKTKNMD AIKTLLDVAV TEGNNLKGSW REVLTCVSQL
EHMQLISSSR VRKPPTEELA NESRSTHITV AADMVFSLSH YLSGTAIVEF VRALCDVSWE
EIQSSGLSQH PRLFSLQKLV EIAYYNMNRI RLEWSNIWEI LGEHFNQVCC HNNPHVGFFA
LDALRQLAMR FLEKEELPHF KFQKDFLRPF EYTMIHNSNP DIRDMVLQCL QQMIQARVGN
MRSGWRTMFG VFSAASKVPT ERIVSSAFEL VTRLNKEHFT AIVRHGAFAD LTVCITDFCK
VNKDDPMIKF WFPVLFGFYD VIMNGEDLEV RRLALDSLFT TLKSYGKTFP VDFWDTVCQE
LLFPIFAVLK SSQDLSRFST QEDMSVWLST TMIQALRNLI DLYTFYFETL ERFLDGLLDL
LCVENDTLAR IGTSCLQQLL ESNVKKLSPA RWERVATTFV KLFRTTTPHQ LFDESLRVER
RRIFRQIIVK CVLQLLLIET TNDLLRNDEV YNTIPPEHLL RLMGVLDHSY QFARMFNEDK
ELRTGLWKES SSASTLVHVL LRMYYDLRPE HQAARPQVAE RLLPLGLGVL GDFNKLRIDT
QLKNIAAWTP VVAEILQGFV RFDDKAFARY LPAIYPLATD LLSRDPEIRE GLRDYFMRVG

YIQGI
>WC
DRPREIFEPL RLACETRNEK LMIASLDCIS KLISYSFFVE LVDVVVHTIT SCHSETTPEA
VSLQIVKALL ALVLSPTILV HQSSLLKAVR TVYNIFLLST DPVNQTVAQG GLTQMVNHVF
DLFIKDAFLV FRALCKLTMK SLNTESERDM KSHAMRSKLL SLHLVLTILS SHMPIFVSPS
AIIYSSSSHE ATSFVQAVNQ YLCLCLSRNA VSPVPQVFEI SVEIFWRVIS GLRTKLKKEI
EVLLHEIFIP ILEMKTSTLK QKAVILNMLQ RLCQDPQVLV EIYLNYDCDS EAVDNIYEHL
MNIITKIGTP SLSTTALVGP GAPVALSEQQ LRRQGLECLV AVLKSLVAWG TDDPSKFESA
KQKKTTLLEG IKKFNFKPKG IQFFIETGFI SSNSPKDIAK FLLESDGLSK AMIGEGDEEN
IAIMHAFVDM MDFRNLAFVN ALRTFLQAFR LPGEAQKIDR FMLKFAERYI AGNDTAYVLA
YSIILLNTDA HNPQVKRRMT LADFVKNNRG INDLPGEFLS SIYDDIVSNE IRMKDEVANV
GRDLQKEAYM MQSNNMTNDQ FFSASHFVHV RPMFEVAWIP FLAGISGPLQ ETDDLEIVEI
CLDGFKAFVT TLAKFTFLNN LGEMKTKNME AIKALLDVAV TEGNHLRTSW REVLTCVSQL
EHMQLISSNR VRKPPTEELA NESRSTHITV AADMVFSLSH YLSGTAIVEF VRALCDVSWE
EIQSSGLSQH PRLFSLQKLV EISYYNMSRI RLEWSNLWEI LGDHFNQVCC HNNPHVGFFA
LDALRQLAMR FLEKEELPHF KFQKDFLRPF EYTMIHNSNP DIRDMVLQCL QQMVQARVGN
MRSGWRTMFG VFSAASKVPT ERIVNSAFEI VTRLNKEHFS AIVRHGAFAD LTVCITDFCK
VSKDDPMIRF WFPVLFGFYD VIMNGEDLEV RRLALDSLFT TLKTYGSTFP VDFWDTVCQE
LLFPIFAVLK SSQDLSRFST QEDMSVWLST TMIQALRNLI DLYTFYFETL ERFLDGLLDL
LCVENDTLAR IGTSCLQQLL ESNVKKLSSP RWERVATTFV RLFRTTTPHQ LFDESLRVER
RRIFRQIIVK CVLQLLLIET MNDLLRNKEV YDTIPPEHLL RLMGVLDHSY QFARMFNEDK
ELRTGLWKES SSAATLVHVL LHMYYDPRPA HQAARPQVAE RLMPLGLGVL GDFIKLRVDT
QLKNIAAWTP VVAEIMQGFA QLDDKAFARY LPAIYPLATE LLSREAELRE ALRDYYRQVG
YIQGI

04-CCR4-NOT
>AS
QRQAIILAAQ AKFGPEAVAP ILQAIFPTLS LQVGTSLVTA LIQLGPEITS DSQTVRAVLA
RFGLTAAIPP TDAQVVEIVQ TLARRVADTH PLCDVGALIR ALSSFGVSIN WANVVRAFDW
PDRGVDTATL KLVIAVLVHS PRAEHPAVAG FWQTWSNPLS QLRLLDALLS LPSDTFNFVT
LPGRRVVKVD DVAGASPTIK ALAANVQGHT WNSLDLFETL VRVGVSDSPE VRACVHDMLD
KAVKISAELV HMGLLQVPPW NELQVEYSTK LLNMFLAGHP NHQLVFMRIW QIEPTYLTTA
FRDFYTENPL NITRILDILD ALLDVRPFIF ALDEYLNLDK WLADNITQHG SAFLRAVIDF
LDVKTTSEKQ ARVTENPDRT MALNAQTIAI FLRVLRNSSS MLAQADIDYC LEIRDACLQV
YPRLMDQEPG FSVVSYSQEI ESEVDGIYKQ MYEENISIEQ VIQMLQRMKE STTARDHEIF
SCMLHFLFDE YKFFQYPARE LNMTANLFGS LVQHKLIDYI PLGIAIRYVL DALQCPSDSN
LFSFGVQALS RFEGRLREWQ PLCQALLRIP HFAEDRPDLA DAARRAELSD KILFIINNLA
PSNFDSKLQE MKERFSTEPN NHALYLRFLD GMEKQPLMKL IVHETIVKSA NLLNSEKTMN
SPSERTVLKN LASWLNQPIK HKNIAFKELL LEGYDQHRLI LAVPFVCKIL EGSAKSKIFQ
PPNPWLMAVI ALLAEVYHYA DIKLNLRFEI EVLWKKLDID GANIEPTSLI RNRPRNMTMH
IETILGSLPS VVVINPQLAL QATQAFKQAV TMAVDRSVRE IILPVVERSV TIAGISTREM
ITKDFATEGD EGRLRTSAHA MARRLAGSLA LVTCREPLRS NLTAHIRSFL LEHGFTEQML
ISLLVNDNID IACNAIERAA MDRAAADVDE SFVQAFDARR RHREQRPGSN VTSNLPDLLR
IKPGGLTNQQ LHVYEEFFTH LMAEVDNVLG QSPQSLTMLP QNHPLKVLGR QIEGLADNED
TLLNFSQRIV HALFKVSTQL GRDFYTAMLE RLCRTSEKVA HEALSWLLYS EDERKFSVPV
IATLMRAGLI PLVEHDAHLA KTNNPTIIDF AVGLIRQLTS AESTLAKFHN SIDWFMKWVQ
IFQRSASAEK SFVTFIQQLT KEGVLNGDET FAFFRVSAEA CIDNYRKQTS TGNLTNIFQP
IDALSRLIAL LVKYHGESSQ DSFKIKLLSK ILTIIVLVLA HAHETQGFQQ KPFFRFFSSF
LNDLHSMEAN LGSTYFQILL SLANNFQTLQ PTYFPGFAFS WITLISHRLF MPKLLLSENR
EGWACFHTLV ICLFKFLANF LRPVQLSDAV RDLYRGAMRL LVVLLHDFPE FLSEYYFTIC
DVIPPRCIQL RNVVLSAYPA SLVLPDPHLR NIKMGPIPPI LSEFTVPLKI GDLRSFLDQF
LLNRASTASL PFLKEFLEKY NLSAINALVM YVGVSSVAQA KARSGSSIFV PSDPGVVLLQ
YLVTNLDAEG QYHVLSAAIM HLRYPNAHTH WFSSLLLFLF AEINDEGFRE IMTRALLERF
IVHRPHPWGA MVTFIELLRN PRYEFWTHDF TRVAPEIQLL LDGV

>EG
QRQAIILAAH AKFGPEAVAP ILQTIFPSIS LQPGTSLVQA LIQLGPEITS DTVTVRALLA
RFGITPPTPP TDAQVVEIVQ TLARRVADTH PLCDVGALVR ALSSYGVPLN WAAVIRAFDW
PDRGVDTATL KLVIAILVHS PRAEHPAVAG FWMTWANPLS QLRLLDALLS LPSDTFNFVT
LPGRRVVKVD DVAGASPTIK ALAANVQGHT WNSLDLFETM VRLGVHESPE VRACVHDMLD
KAVKISAELV HMGLLQVPPW NELQVEYATK LLNMFLAGHP NHQLVFMRIW QIDPTYLTTA
FRDFYAENPL NITRILDILD ALLDVRPFIF ALDEYLNLDK WLADNIAQHG ATFLRAVIDF
LDVKTTSEKN ARVTESPERT MALNAQTIAI FLRVLRNSSS MLAQADIDYC LEIRNACLQV
YPRLMDQEPG FSVVSYSQEV ETEVDSIYKK MYEDDISIDQ VIVKLQAYKE STNTREHEIF
SCMLHFLFDE YKFFQYPHRE LGMTAYLFGS LIQHRLIDYI PLGIAIRYVL DALQCPPESN
LFQFGAQALE RFVGRLREWQ PLCQALLRIP HFVDSRPDLA EAARRAEVSD RILFIINNLA
PSNFESKLQE MKERFSSEPN NHALYLRFLD GLEKQGLMKL VIHETIMKSA MLLNAEKTMS
SPSERSVLKN LASWLNQPIK HKNIAFKELL LEGYDNGRLI LAIPFVCKIL EACAKSKVFQ
PPNPWLMAVI ALLSELYHFA DIKLNLRFEI EVLWKKLDID GAKIEPTLLV RNRPRAMNLH
IEELLASLSN TIVINPQLAL QATQAFKGAV TLAVDRSVRE IILPVVERSV TIAGISTREL
VTKDFAMEGD EQRMRIAAQV TATKLAGSLA LVTCKEPLRS NLTTHIRNYL LEHGFTEQML
ISLLVNDNIE QACKAIEQAA MDRAKADVDD SFMQAFETRR RHREHRPGST ATSNLPDALR
IRPGGLSQQQ MHVYEEFFTH LMVEVDAVLN QAPQTFSMLP QNHPLRTYGR QIESLAKSEE
TLLNFSQRVV HALFKVQTQL GRDFYAAMLE RLCRTSEKVA QEALLWLLYA EDERKFSVPV
IATLLRAGLI PALDQDIQLA KSFNPTIIDF TVGLLRQLFP GDASQKQFQY SFEWFLKWVQ
IFQRSATTEK SFVTFIQQLQ KEGILSGDEQ FSFFRVCAEA SVESYRKQAM TGNLTNIFQP
IDALSRLIAL LIKYHGDTTQ DHVKVKYLSK ILTIIVLVLA HAHEKQGFQQ KPFFRFFSSF
LNDLHSMESN LGTAYFQILI TLSDNFSTLQ PTFFPGFAFS WITLISHRLF MPKLLLSENR
EGWSAFHKLV ICLFKFLASF LRPVHLSVAV RDLYRGAMRL LVVLLHDFPE FLGEYYFTIC
DVIPPRCIQL RNVVLSAYPA TLVLPDPHLR NVKMGPIPPI LSDFTSTLKT GDLRNYLDQF
LLNRANSSTL PFVKECLEKY NLSAINALVM YVGVSSVAQA KARSGSSVFV PSDPGVVILQ
YLVTNLDAEG QYHVLSAAIM HLRYPNAHTH WYSSLLLFLF AEINDERFRE IMTRALLERF
IVHRPHPWGA MVTFIELLRN PRYDFWNHEF VRVAPEIQML LEGV
>SS
QRHDIIMTVQ SKYPHDIISP VVQQVFANLS VPSGSNVVEI LCDLGTDITS DPSYVRAVLQ
RFGFTEAVPP TDVQVTEMMS TLSKRTFEST QLCDARTLLR VLNSFDVNIN WPSLIKAFDW
PDRGVDTPTL KLLIVILLNS PHVEPHAVAG FWTIWTNSLY QLRLLDALLS LPADTFNFVS
LPGRRIVTVD DVANASPTIK ALAANVQGHT WNSLELFEVL ITLGDSDVPE IRTCVREMLD
KAVKISAELV HMGLLQVSPW NGTQKEYAAK LLSMFLAGHP NHQLVFMRIW QIDPTYLTTS
FRDYYNESPL NITRILDILD TLLDVRPFTF ALDEYLNLDK WLLDNINTHG AEFLHSVIEF
LDFKTASEKT ARLADPPEKT MALNPQTITI FLRILRNNAS MLSDEDTSYY FEVRNACLQI
HPRLMDQEPG FAVVTYSTEV ENEVDSIYKQ MYDESITIDQ VIQRLKQAKD STNPRDQEIF
SCMLHFLFDE YKFFQYPPRE LAMTGYLFGS LIQHRLVDYI PLGIAIRYVL DALQCPPETN
LFKFGIQALS RFESRLSEWQ PLCEKLLQIP HLLEARPDLA ESARRAEVSD KILFLVNNLA
PSNFDAKVRE MKERFCTEPN NHHLYIRFLE ALDRQLLFKF VLHETFVKSA NLLNSEKTLT
SSSERAILKN LGSWLNKPIL HKNLSFKDLL LEGYDSNRLI TAIPFVCKIL EGCSKSLVFK
PPNPWLMGVI SLLVELYHFA ELKLNLKFEI EFVCKSLDLD LDQVPATTIL RNRPRGITPQ
IESILVNLSN MIQISSDLAL HTNIAFKRAV QLAVDRAVRE IIVPVVERSV TIAGISTREL
VSKDFASEPN EEKLRSAAHL MARKLAGSLA LVTCKEPLKT NLSNHIRHFL TEHGFAEHPI
ITMIANDNID LCCAAIEKAA MDRAVADVNE NFTPAFDLRR RHREHRFNPN YMAHLPDLLR
LKQGGVMPLQ GRVYEDFFNR MMNDLDRVLT QLGQSLSSFP PNHELRQFGR TVAVLASPDE
LALLFSQKVV SHLYKTPLTI GREMYVDILD RLCQEFPKVA KEAVDWLLTS EDERKYNVPV
TIALIRGSLV TIWQEDEQLS KDPRPSLLNF TASFIRECLT TDATRQELAL SIEWFHHWSL
LYRESASSEK AFLSFIPQFT SEGYLAEDTT TLFFRICMEA SVAAYHQRIA TGDDGRAFQD
IDAFSRLIVM LIKYNGDVNA DTKKLRYLTK ILSIVVLVLV HQHEERGFQQ KPFFRFFSSL
LHDLHSVESS LHTAYLPLLQ TICDTFNTLK PAHFPGFAFS WMSLISHRLL MPKLLASDAQ
EGWHNLHQVL LALLHFLAPF LRAGELPRSI RDVYQGTLRI FVVLLHDFPD FLTEYHFSLC
DAIPPRCVQM RNLITSAFPA TLTLPDPYLR NVRMGPIPTT RFDIAAALKT YELAPTLEAL
MLKRGPPSFL QSLKDRLSTY NLSAMNAVVF YIGLSSVAQA KSKSGSPTFV PSDPGVGIVT

YLATNFDPEG QYHLLGAILL HLRYPNAHTH WFSSLMLHLF IDMKDIRFKE IMTRILLEHI
AVHRPHPWGV MVTFIELLRN PKYEFWNHEF VRSSPEITLI MENI
>SP
QRQALLSATQ AKFGVETIAP VFQQILSHIS LPSGTTLVQL MVQLGPDLTS NANVLRGILG
RFGISEHHAP TDALNVELFS TLGRLAAEGT PMGDVGLLVQ MMGAFAPNLD WSKVIESFDR
PERGVDMATL KLLIAILGNS SHTNKPSISG FWSTWKNSLY QLKLLDALLS LPSDTFNFVN
LPGRRVVMVD DVSSASPTIK ALAINVQGHT WNSLDLFEVL VRLGGSEIIE VRNCVRDILD
KAKRISAEIV HMGLLQVPPW NAIQEEYLQQ LLEMFLNGHP NHQLVFMRIW QIEPPYLTNA
LRDFYEKNPM NITRILDILE PLLEVRPFIF ALDEYLNLDK WLADNINQHG AEFLHAVIEF
LELKAQNEKI TRTSDPSART MALSPQTIAI FLRVLRNNSS DMDERDVDYC IEVRSVCLQI
HPRLMDQDPG FSVVSYSADI EAEVDSIYKQ MYDEQITIDG VIALLQKTKE STNPRDHEIF
SCMLHFLFDE YKFFQYPARE LAMTGYLFGS LIQHQLVDYI PLGIAIRYVL DALQCPPDSN
LFKFGLQALS RFEARLPEWR LVCQALMDIP HLAEARPDIA ETVRRAEVSD KILFIINNLA
PSNFDAKVEE MNERFSIEPN NHQLYLRFLD GLKSSALMKL ILHETFVKSA ALLNSEKTKA
SSSERSVLKN LGSWLDKPIK FKNMSFKDFL IEGSESDRLI VAIPFVCKIL EACSKSKAFK
PPNPWLMGII SLLAELYHFA ELKLNLKFEI EVLCKALGLD MDDIEVANIL RSRPRNVGAH
IEGILANLAN SVIVNSQLAY MRDPNFKRSV QLGIDRAVRE IILPVVERSV TIASLTTREL
VVKDFATESS EDRVRKAGHL AGQKLAGSLA LVTCKEPLKT TLPGHIRAYL TENGFSEAQI
VLLLAQENLD IACEAIEKAA MDRAIGEIDE AFVPHLEARR RHHEQRPGSS FASGLPDPLR
VKANGPQEAQ LRVYEEFFSL CMSKLETILQ ETSASLAVLP QTHDIRTIVH RILTIADRFK
LPLLISQKIV QYLYKTSTQL GRELYATLLE QLCQSYDEVA KEAITWLIYA EDDRKFNIPV
TVTLLRCRLV SVAEEDQQLA RSPRPVLQDF AVGLMRECLT CDATRQQFTL TLDCFQQWVA
VFQRSSSPEK SFVPYITNLT KQNILKAEDS SFFFRVCAET SVGHYSNCLA SGDFDNAFLA
LDAMSKLIVF IIKYHGDANN LNAKVHYMTK ILSIVVLVVA NRHEEQGFEQ KPFFRFFSSL
LSDLHGIESH LGDAYLPLLV ALSDTFSSLQ PVYFPGFAFS WMTLVSHRLF MPKLLSSSNR
DGWQAFHKLL LALFKFMAPF LRNAKLQLAS RNLYRGTLRL LLVLLHDFPD FLSEYYFSLC
DVVPPRCIQL RNIIMSAFPA SATLPDPHLP GMKMSPIPSI FSDFTATLKT GDLRTYLDQY
LFNRIAPNSL APLKERLESY NISVVNAVVM YIGVSSVAQA KARTGSPLFV STDPGAVTLS
YLAANLDPEG QHHLLSAMVM HLRYPNPHTY WFSSLLLYLF SEVNDDRFRE IMTKVLLERF
IVHRPHPWGA LMTFIELLRN PKYEFWARDF VRMVPEVALL LESV
>PN
QRQALVAAAQ TKFGVEMMSP ILSQLFQNLS LPPQTTVVQV LNQFGPEITG DVDVVRGLLT
RFGMNDQTPP SDEQIVDIFS TLSRLTVENA QLCDVAALVR ALSSFRVKHD WSKVIHVFDW
PDRGVDTNTL KLLIAILVNS PRVEKPAVSG FWSQWNNSLY QLRLLDALLS LPSDTFNFVS
LPGRRVVTVE DVAAASPTIK TLAANVQSHT WNSLDLFEVL VRLGDSENEN VRNCVREMLD
KAVRISADIV HMGLLQAPPW NALQVEYSRQ LLGMFLSGHP NHQLVFMRIW QIEPSYLTSA
LREFYEENPM NITRILDILD SLLEVRPFVF ALDEYLNLDK WLEDNIIKHG AEFLHGIIVF
LETKMQNEKI ARTIDPQART MALSPTTIAI FLRALRNFSS VMDEADMDFC IETRNACLQI
HPRLMNQEPG FSVVNYPPNI EAEVDGIFKQ MYDEQITIDQ VIVMLQRTKE STNPRDHEIF
SCMLHFLFDE YKFFQYPPRE LAMTGYLFGS IIQQQLVDYI PLGIAIRYVI DSLQCPPETN
LFKFGVQALS RFESRLPEWK VLCEALLSIP HLAEQRPDII EVVRRAETSD KILFIINNLA
PSNFDAKLAE MKSSFSSEPN NHQLYLRFLD GLDCKKLAKF ILHETYIKST AMLNSDKTKA
SASERTLLKN LASWLDKPIK HKNIAFKELL IEGAESDRLL VAIPFVCKVL EGASKSKAFR
PPNPWLMAVI SVLAELYHFA ELKLQIKFEI EFLCKALGID LETVEVSNIF RSRPRNVGAH
IEELLAQLAS AVTINSQLGL SNNQLFKQAI QDAIDRSVRE IILPVVERSV TIAGITSREL
CVKDFASEAS EEKLRKAGHL TCQKLASSLA LITCKEPLRT NIPGHIRTYL AEHGFTEQQV
IMLITQDNLD IACETIEKAA MDRATREVDT SLALSYDARR RHREMRSNIP FIANLPDSLR
IKPMGLQPHQ LRVYEDFFNV LIQKLEAILP DVTPSFSSVP LSHEIRNILH RLVSLSDRVR
TPLLMSQKIV QHLYKTPLQL GREVYVTALE QLCRTYDDVA KEAINWLICA EDDRKFNIPV
TVTLIRSRLI TVTDEDLQLA KNIRPNLQDF ASGLIRECLS TEATQISFQY TLDCFQQWVS
VFQRSSAPEK MFVPYVTQLS KQGILKAEDS SFFFRVCTES SIAHYSKCMA SGEYDHSFLA
LDAMSRLIVY IIKYHGDANN LQAKVHYFSK ILSIVVLVIA NRHEEQGFQQ KPFFRFFSSL
LSDLHSLETQ LGSVYFALLL ALSDTLSSLQ PTYFPGFSFS WMTLISHRLY MPKLLSSENR
EGWSAFHKLL TALFRFLAPF LSSANFTPAS RSLYRGTLRL LLVLLHDFPD FLSEYYFSLC

DIIPHRCIQL SNIILSAFPA SVNLPDPHLR NINMGPIPPI VPDFAILQKS GDLKAHLDQC
LANRVSQVSL STLKERLENY NISLMNTMVM YIGVTTVAQA KARSGNPIFN PSDPAVVVLS
YLAYNLDPEG QHHLVSAMVL HLRYPNAHTH WFSSLVLHLF EQANDETFRE ILTRVLLERF
IVHRPHPWGA LLTFIELLRN PKYDFWNKDF VRAVPEVTLM LDNV
>TA
QRQALIAAAH TKYGHEAVSP ILQQLLPKMS LPPGTTLVQC LIQLGADITS EGDVTRALLA
RFGITEQNPP KDAQVVEIFS TLGRHAAEGT TLCDVGALVR AISSLRAKLD WSKVIEAFDW
SDRGVDTATL KLLIAILVNS PRADKPAVAG FWTNWKNSLY QLRLLDALLS LPSDTFNFVT
LPGRRVVAVE DVASASPTIK SLAANVQSHT WNSLDLFEVL VRLGDSENAD VRNCVRDMLD
KAMRISADIV HMGLLQVPPW NSLQIEYSRQ LLGMFLAGHP NHQLVFMRIW QIEPTYLTTA
LREFYDENPL NITRILDILD SLLEVRPFTF ALDEYLNLDK WLADNVSAHG AEFLRAVITF
LDLKAQNEKI ARTLDPNAKT MALSAPTITI FLRTLRMHSS LLDDRDAEYC LEVRNACLQI
HPRLMDQEPG FSVVSYTPDI EAEVDGIYKQ MYDEQITIDQ VIQLLQRKKD STNPRDHEIF
SCMLHFLFDE YKFFQYPARE LGMTGYLFGS LIQHQLVDYI PLGIAIRYVL DALQCPPDTN
LFKFGVGALL RFESRLPEWK PLCQALLEIP HLAEQRPDIV ENVRRAEVSD KVLFIINNLA
PSNFDDKLAE MKERFSTEPN NHQLYLRFLD GLDSKVLARF ILHETYVKSA VMLNAEKTKT
SSSERAILKN LASWLDKPIK HKNLSFKDFL IEGADNDRLI IAIPFTCKVL EWAGKSKAFK
PPNPWLMAVI GLLAELYYYA ELKLNLKFEI EVLCKSLGLD LDKVEVANIL RSRPRNVGAH
IEEILTALSN SVVVNPQLAL HGNPVFKRAV QEAVDRSVRE IILPVVERSV TIATITTREL
GMKDFASEAS EEKMRKATHL AGQKLAGSLA LVTCKDPLRQ NMPGHMRAYL NDHGFSEQQV
VLLLVSDNLD IACEAIEKAA MERAIREVDT ALAGSFDSRR RHRDARSGSP FISNLPDPLR
IKLTGLQPHQ LRVYDEFFNL LIEKVEASLA NCPPSLSTVP SNHELHSLIP FLRTILDRLR
APLLMSQKIV QLLYKTPTQV GRDVYVTLLK ELCKEYAEVK KEATNWLIFA EDERKFNVAV
TVTLLRNGLV TITDEDMQLA KNARPSLQDF AAGFLRECLT TDATRQSIPY ALDCFQQWVN
VFQRSSTPEK SFVPYITQLT KQGILKAEDS SFFFRVCAES SAAHYTACMA QGDYENQFLA
LDAISRLIVY IIKYHGDADN VHAKVHYFKK ILSIVVLVVA NMHEEQGFQQ KPFFRFFSSL
LSDLHSIETY LGNAYFQLLI ALCDTFSSLQ PVYFPGFAFS WMTLISHRLF MPKLLTSENR
EGWSAFHKLL LALFKFLAPF LRSAKMSGAS RNLYRGTLRL LLVLLHDFPD FLSEYYFTLC
DIIPPRCVQL RNVILSAFPT SITLPDPHLR GLSMGPIPPI LSDFTLILKN GDLRTYLDQC
LLNRATPGSL AALKDRLETY NLSLMNAVVM YIGVSSVAQA KARSGSALFV PADPGVVALM
YLAVNLDPEG QHHLISAMIL HLRYPNAHTH WFSSLLLYLF SEVSNERFKE VMTRVLLERF
IVHRPHPWGA LVTFIELLRN PKYDFWKKEF IHVAPEVTLM LEGV
>FM
QRQALVFSAQ KKFGAELVSP ILAQLFQKLS LPPQTTLVQA LNQLGPDISN DVEVVRGLLV
RFGISDQNLP TNEQITDTFM TLSRLTAEGA QLCDVGTLVR VLSSFRAQLD WSKAIEVFDW
PERGVDTNTL KLLIAILVNS PRADKPAVAG FWSNWKNSLY QLRLLDALLS LPSDTFNFVS
LPGRRVVTVD DVAAASPTIK TLAANVQSHT WNSLDLFEIL VRLGDSEIEN VRSYVREMLD
KAVRISADIV HMGLVQVPPW NALQVEYSRQ LLGMFLGGHP NHQLVFMRIW QIEPTYLLTA
LREFYDENPM NITRILDILD SLLEVRPFVF ALDEYLNLDK WLSDNIEKYG ADFLHSIILF
LDHKMQNEKI ARTVDPQART MALSPTTIAI FLRALRTFSN VMDERDAEYC VETRNACLQI
HPRLMNQEPG FNVVSYSTDI EAEVDGIFKQ MYDEQITIDQ VIIMLQRTKE STNTRDHEIF
SCMLHFLFDE YKFFQYPPRE LAMTGYLFGS IIQQQLVDYI PLGIAIRYVI DSLQCPPDTN
LFKFGVQALT RFESRLAEWK LLCEALLNIP HLAEQRPDII EAVRRAETSD KILFIINNLA
PSNFEAKLTE MKERFSTEPN NHQLYLRFLD GLDSKPIAKF ILHETFVKSA SMLNSDKTKS
STSERTILKN LASWLDKPIK HKNLSFKEFL IEGADSDRLV VAIPFVCKVL EGAAKSKAFR
PPNPWLMAVI SLLAELYHFA ELKLNLKFEI EVLCKALSID LDTVEVANIL RSRPRTVGAH
IEEILANLAT SVIINHQLAL HTNHAFKQAV QEGIDRAVRE IILPVVERSV TIASITSREL
CVKDFASEPN EEKLRKAGHM ACQKLAGSLA LVTCKDPLRT NMAAHIRSYL LDHGFTEQQV
IMLIVQDNID VACEAIEKAA IDRAIKEVDA ALAQSYDARR RYRDVRSGSA FIAGLPDPLR
IRPNGLQPLQ QRVYEEFFNI LVQKLESILS EIPTSLALVP SNHELRNVVN RVLSLADRVR
TPLFMSQKIV QHLYKTPSQI GRDIYVALLD QLCQSFEEVA KEAINWLICA EDERKFNIPA
TVTLLRSHLI TPTDEDMQLA KNSRPSLQDF AAGLIRECLT SETTQHQFQY TLDRFQQWVG
IFQSSSNPEK MFVQYVTALS KQGILKVEDS SFFFRVCAES SISHYTKSVA AGDFGHSFLA
LDAMSRLIVY IIKYHGDANN LQAKVHYFTK ILSILVLVVA NRHEEQGFQQ KPFFRFFSSL

LSDLSSLGDQ LGNVYFHLLV ALSDTFSSLQ PVYFPGFAFS WMTLISHRLF MPKLLLSENR
DGWSAFHKLL LALFKFLAPM LRSANFTLAS RNLYRGGLRL LLVLLHDFPD FLSAYYFSLC
DVIPPRCTQL RNIVLSAFPA SITLPDPSLR NTKMGPIPLV LSDFSAILKN GDLKGYLDQC
LLSRMPQTSL ATLKERMETY NVPLINAMVM YIGVSSVAQV KARSGSSVFI PTDPGVVALT
YLAYNLDPEG QYHLANAMIL QLRYPNAHTH WFCCLMLYLF EHAKDDRFRE IMTRVLMERF
FVHRPHPWGA LLTFIELVRN PKYDFWNKDF LRVAPEVTAI LDNV
>OS
QRQALVAASQ TKFGAEMMSP ILQQLFQSLS LPPQTTLVQV LNQFGPDITS DADVVRGLLS
RFGMTDQCPP TDEQIVEIFS MLSRLAADGI ALCDVGALVR ALSSFRVKLD WSKVIEAFDW
PDRGVDTATL KLLIAILVNS PRADKPAVAG FWTNWNNSLY QLRLLDALLS LPSDTFNFVS
LPGRRVVTVD DVSAASPTIK TLAANVQSHT WNSLDLFEVL VRLGNSEDGD VRIFVRDMLD
KAVRISADIV HMGLLQVPPW NALQVEYTRQ LLGMFLGGHP NHQLVFMRIW QIEPKYLATA
LREFYEENPM NITRILDILD SLLEVRPFIF ALDEYLNLDK WLQDNVDAHG AEFLHGVISF
LELKMQNEKI ARTIDPQART MALSPSTIAI FLRCLRNYSS VMDERDVDYC IETRNACLQI
HPRLMDQDTG FAVVTYPADI ESEVDGIFKQ MYDEQITIDQ VIAMLQRTKE SSNPRDHQIF
SCMLHFLFDE YKFFQYPPRE LAMTGYLFGS LIQQHLVDYI PLGIAIRYVL DSLQCPPDTN
LFKFGVQALT RFESRLPEWK LLCEALLNIP HLAEQRPEII EMVRRAETSD KILFIINNLA
PSNFDAKLAE MKERFSTEPN NHQLYLRFLD GLDNKTLAKF ILHETFFKSA TMLNSEKTKS
SSSERTVLKN LGSWLDKPIK HKNLAFKEFL VEGADSDRLI VAIPFVCKIL EAAAKSKAFR
PPNPWLMAVI SLLAELYHFA ELKLNMKFEI EVLCKTLGVD LDNVEVANIL RTRPRNVGTH
IEEILANLAN SVTINHQLAL HTNQSFKQAV QEAVDRSVRE IILPVVERSV TIASITTREL
CIKDFASDPN EEKMRKAGHM ACQKLAGSLA LVTCKDPLRT NMPAHVRSYL ADHGFTEQQV
ILLIVQDNVD VACEAIEKAA MDRAIREVDN ALAPSYEARR RHREVRPGTA FIAGLPDPLR
IKPNGLQPYQ LRVYEDFFGL WVAKLEAILA EVPTSLTLVP PNHEIRTCVH RILALCDPVR
TPLITSQKVV QYLFKTPSQL GREIYVTLLD QLCRSFDDVA KEAINWLISA EDERKFNIPV
TVTLLRGHLI NVTDEDLQLA KNARSNLQDF SAGLIRECLT SDATQQQYQY TLDCFQQWVG
IFQRSSAPEK MFVPYVTQLS KQGILKVEDS SFFFRVCAES SITHYTKSVT AGDFEHAFLA
LDAMSRLIVY IIKYHGDANN LQAKVHYFTK ILSIVVLVVA NRHEEQGFQQ KPFFRFFSSL
LSDLHALETQ LGSVYFHLLI ALSDTFSSLQ PVYFPGFAFS WMTLISHRLF MPKLLLSENR
EGWSAFHKLL LSLFKFLAPF LRTASLTVAS RNLYRGSLRL LLVLLHDFPD FLSEYYFTLC
DIIPSRCIQL RNIILSAFPA SLNLPDPHMR NVKMGPIPPI LSDFTLTLKN GDLRGYLDQC
LLNRVTQTSL AALKDRLETY DLSLMNAVVM YIGVSSVAQA KARSGSSLFV PSDPGVVALT
YLAFNLDPEG QHHLIGSMIL HLRYPNAHTN WFSSLLLYLF AESKDDHFKE ILTRVLLERF
IVHRPHPWGA IITFIELLRN QKYDFWGKDF IRVAPEVTLM LENV
>SC
QRQALIVASQ SKYGREVVAP ILYNILPQLS LPPGATLVQT LMQLGPDVTN DPDVVRAIFA
RFGITDAAPP SDNQVVELIQ TLGRLAAEGS TMCDVGAVVR ALSSYPVQLD WPAVIRSFDW
PDRGVDTATL KLLIAVLLNS PRAHPHAVTG FWTVWNNSLY QLRLLDALLS LPADTFNFVS
LPGHRIVTVD DVSIASPTIK SLAANVQGHT WNSLDLFQVL VRLADSDSPD IKGCIRDMLD
KAIKISAELV HMGLLQVPNW NEIRLEYSRK LLAMFLGGHP NHQLVFMRIW QIQPSYLTDA
FRDFYEENPQ NITRILDILE ALLEVRPFTF ALDEYLNLDK WLGDKVAAHG TEFLRSIADF
LKEKMESEKL TRITDPPVRT MPINPSTVAI ILRVIRGSSH LMEQADVDYA LDVQNMCFQV
YPRLMDVESG LAVIQYPQDI ENEVDLIYKR MYDENISIDE VITMLQQLRT SSNPRDQEVF
SCMIHFLFDE YRFFQYPDRE LAMTGYLFGS IIQHDLLDYM PLGIAVRYVI DALNCPPDKN
LFRFGVQALS RFESRLPEWQ PLCAELLKNQ HLLEARPDLA ITLQRAELSD KILFIVNNLA
PSNFDTKLVD MKQSFSTEPN NHSLYLRFLD ALNRSSLNKY ILHETFVKAA ALLNSEKTVQ
SSTERSILKN VGSWLDRPIK HKNLSFKDLL MEGYDSGRLI VAIPFVCKTL EPAAQSKVFR
PPNPWLMAVI SLLAELYHFA ELKLNLKFEI EVLCKSLDID LDTVEAAVIM RNRPRQVGTQ
IEQILTTLAQ HVQISSTLSM LGNPAFKRAV QLAVDRAVRE IILPVVDRSV TIAGISTREL
VAKDFATEAN EEKMRKAAHS MAAKLAGSLA MVTCKEPLRT NLAQHLRQYL AEHGFSDINH
IMDLALDNLD NACSAIERAA MERAISDVDE GFAPAYEMRI HHRETRSGHS LTTNLPDILR
LHKNGVLPVQ LNVYEDFFGV LIRDLDNVLV QLPQSLALLP PNHDVRHLVR QIMLNADRRQ
APLQMSQKIV QLLYKAPTQL GREVYVALLD QLCRHFEETG KEAINWLIYA EDERKYNIPV
TVTLLRSRLF DLAVYEQQLA KDPRPSLINF VIGLIRECLT ADATQSQFPY CIEWFQSWVQ

IYHRSPNPEK SFVGFITQVL KSGVLNIDES QLFFRVCAET SVNHYAKAVA VGNYASAYSY
VDAMSKLVVF IIKYHGDPNA DQAKVHYFKK VLSILILVLA YFHEEQEFQQ KPFFRLFSSL
LNDLNSMEAS LGTVYFPLLV VFCETLSGLQ PIYFPGFAFS WMGLISHRLL MPKLLLSENR
EGWAIMQKLL IALFKFLAPF LKDSELSVPA RDMYRGALRI LLVLLHDFPD FLSEYYFSLC
DVIPYRCIQL RNIVLSGFPQ TMVLPDPHLR GMKMGPIPPI LSDFAAGLKG GDLKAYLDQY
LLGRGSTSFL PSLKERLETY NLSLLNALVM YIGVSSVAQA KARNGTALFA PSDPGVVALQ
YLANSLDAEG QFHLITSMTL HLRYPNAHTY WFCSLLLHLF TEVEDEKFQE IMTRVLLERF
MVHRPHPWGA VMTFVELLRN PKYNFWSKEF INVAPEVSML LESV
>SN
QRQALIVAAR TKYGAEIVNP ILHQIFPNLS LRPGTDLVQV LNELGPEITS DVDTVRALFR
RYGLTDSSPP NDAQVAEIIS SMSRHASEGG QLCDVNALVR AIASFHTSLN WPALVHVFDR
PDRGVDTATL KLLISILLNA PREEPHAVTG FWMEWRNSLH QLRLLDALLS LPGDTFNFVL
VPGRRVVTVD DVAGASPTIK ALAANVQGQT WNSLSLFETL IHFGNSDQAE IQACVRDMLD
KAVKISAELV HMGLLQVPPW NALQAEYSGK MLAMFLAGHP NHQLVFMRIW QIDPTYLTTA
FTDFYHDNQL NITRILDILD TLLAVRPFTF ALDEYLNLDK WLADNIADHG AEFLRAVIEF
LDAKTASEKE TRVTEPAART MALSPQTIAI FLRVLRNNSS VLAPGDVDYC LEIRNACLQI
HPRLMEQEPG FSVVSYSAEI EKEVDSIYKQ MYDENISIDQ VIALLQRSKT SKNPRDHEIF
SCMLHFLFDE FKFFNYPDRE LHMTGYLFGS LIQYNLVDYI PLGIAIRYVL DALQCPPDTN
LFKFGIQALS RFETRLPEWQ PLCQALLRIP HLLEARPDLA TNLRRAEVSD KILFIVNNLA
PSNFESKVDE MKERFSTEPN NHQLYLRFLD SLSKPDLHKL IVHETFVKSA ALLNSEMTQK
STTERTILKN LGAWLNKPIK HKNMAFKEFL LEGYDTNRLI VAIPFVCKIL EACAKSTIFK
PPNPWLMAVI ALLVELYHFA ELKLNLKFEI EVLCKTLDID LTRIDASTVL RNRPRVVGSH
IEGILGNLSN LVVINAQLAL HTNHSFRRAV TVAIDRGVRE IIVPVVERSV TIAGISSREL
VSKDFAMEGS EDKMRKAALL MAQKLAGSLA LVTCKEPLKS NLGAHIRHAL TEQGFNEQQV
IGMLVLDNIE AACAAIERAA MDRAVMEIDD ALAPAYEMRR RHREVRPGLE FLAHLPDPLR
IKPNGLQLPQ WRVYEDFFVV MTHELEMLVT QIPQPLSALP VAHEVKQLIR QILFCADRDK
VALQMSQRIV QHLYKTASQL GREAYVTILD QLCRSFPEVQ KEATEWLIYA EDERKYNVPV
TVTLLRSGLI PVAQQDAQLA REARPSLIDF TANLIRECLA GDATQAQFQD SLSWFQQWVM
IFQRSPSPEK AFIPFVSQLQ KEGILKQDDS FLFFRICTEA SIESYTGAVT GEDPEAAFEA
IDAMSKLIAL IIRYHGEANN DQLKAHYLTK ILSIVVVVLA HAHEEQGFQQ KPYFRFFSSL
FNDLHAIEPH VGSAYFPLMI ALSDTFSSLQ PIYFPGFAFS WMCLISHRLF MPKLLSSESR
EGWSAFHTLL VSLFKFLGFF LNALDLKDAA RDLYRGAVRI LLVLLHDFPE FLAEYYFSIC
EAIPPKCIQL RNVVLSAFPM HVVLPDPYLR NLQIGPIPPI LSDFTSALQP GELRTYLDHF
LLNRGTTSFL PSLKERLEKY NLSLINSLVM YIGVSSVAQA KARSGSAVFV ASDPGVVILQ
YLAMNLNVEG QHHLLSSMVL HLRYPNAHTH WFSSLLLHLF VEVKDEVFCE LMTKVLLERF
IGHRPHPWGA LVTFIELLRN PKYEFWSREF TRIAPEITML LEGV
>CC
QRLALIMTFQ TKYGKEALAP ILPALFANLS LSSSATLVQT FVQLGSEITS DRDAMHGLLQ
RFDISDANPP HDAQLVEIVS ALGRLIAEGT NVPDASVLIH TLASYRANLN WPAAIKTFDR
PDRTVDTPTL KLLIAILLNA PRQEPHAVAG FWEPWSNPLY QLRLLDALLS LPGDTFNFVS
LPGKRIVTVD DVSGATPTIR SLALNVQGYT WNSLELFQIL VKLADSESQE IRNCVREMLD
KAIKISAEIV QMGLLQVPQW NEIRLEYSRK LLAMFLAGHP NHQLVFMRIW QIKPNYLTNA
FREFYEENNL NITRILDILD ALLEVKPFTF ALDEYLNLDK WLADNVANHG EDFLRSVILF
LQQKMESEKV SRLSDPAVRT LTLSPNTITI ILRVLRNNSS AMTEADVEAS IEVRNACLQI
HPRLMEVEPG LTVVNYSNEI ETEVDSIYKQ MYDDVITVGQ VIQMLKQYKE SSNTHDHEVF
SCMIHFLFDE YKFFQYPARE LQMTADLFGS LIQHKLIDYI PLGIAIRYII DALNCPPETN
LFRFGRQALG RFEFRLVEWR PLCEALLRIP ALAETSPELI QTIQRAEISD KILFIVNNLA
PSNFEVKLKE MREQFSTEPN NHPLYLRFLD SLDKKPLNKF ILQETFVKAA ALLNSEKAMQ
SGSERNTLKN IGSWLDKPIK HKNLSFKELL MEGFDSGRLV VAIPFVCKTL EPCAKSRVFK
PPNPWLMAVL SLLVELYHFA DLKLNLKFEI EVLCKGLEID LDAIEATTIL RNRPGVNQLA
IESLLAALSN NAQINPQLSL NVNPTFNRAV RLAIDRAVRE IIVPVVERSV TIAGISTTEL
VVKDFASEPN EQKLRRAGHV MAQKLAGSLA LVTCKEPLKT NLAAHFRQAL NEHGFTEQAV
VQILVQDNLD IACNTIEKAA MDRVAVDIDE SFAQSYEIRR RHRETRPAST FTNTLPDPLR
IKLSGLQPQQ LSVYEDFFAS HTRDLEAVVV QIPQSLAALP ANHDIRVLVS RVLMLADRER

SPLMISQKIV QLLYKTPSQL GREVYIAILE QLCQSFEEVA KEAINWLLYA EDERKYNIPV
TITLLRSGLI NPTLHDQQLA KNARPNLVAY AVGLIREALT GDLPQSNFQF SIEWFHQWVA
IFQRTPSPEK AFVPFITSLT KQGILKVEDS SFFFRVCTEA SVAVYIKCIA TGEFDYAFQA
LDAFSRLIVY IIKYHGDANN EQAKVHYFTK ILSIFVLVLA NMHEEQGFQP KPFFRFFSTL
VNDLHSVEGS LGPVYFQLLL ALSDTYSSLQ PTYFPGFAFS WMCLISHRLF MPRLLLSENR
EGWSAFHKLL LSLFKFLAPF LKEGDLRQAE RDLYRGVLRL LLVILHDFPD FLSEYYFTLC
DIIPPRCVQL RNIILSAFPA AVVLPDPHLR NVSMGPIPPI LSDFTSALRT GDLRNHLDQY
LLSRSSVAFL SQLKDKLESY NLSLINAIVM YIGVSSVAQA KARSGSSLFV ASDPGAVALQ
YLATNLDNEG QHHLLSSIIL HLRYPNAHTH WFSSLLLHLF LEVKDDRFRE IMTKVLLERF
IVHRPHPWGA LVTFIELLRN PKYDFWSKPF VRIAPEVTVL LESV
>RM
QRLALITAAQ ARFGTEIMSP MLQQLFTNLS LPPGTTLVSA LVQLGGEITS DAEVVRGLLT
RFGIAESNPP SDVQVTEVFS TLSRLATEGA SLFDVGALVR ALSGFRVQPD WSKVIESFDR
PERGVDAPTL KLLIAVLMNS PRAVPHAVTG FWSNWSNQIY QLKLLDALLS LPNDTFNFVA
LPGRRVVTVD DVAGASPTIK SLAANVQSHT WNSLDLFEVL VRLGVSENTD VRGIVREMLD
KAVRISADIV HMGLLQVPPW NAIQLEYSSQ LLGMFLAGHP NHQLVFMRIW QIEPTYLTTA
LREHYEENPV NITRILDILD ALLEVRPFVF ALDEYLNLDK WLADNVNAHG AEFLHAVINF
LELKAQNEKI ARTSDPSART MSLSAQTIAI FLRVLRNSST LMDEKDVDYC LEVRNACLQI
YPRLMDQEPG FSVISYSTEV EAEVDGIYKQ MYDEQITIDG VIALLQRTKV STAPHDHEIF
SCMLHFLFEE YKFFQYPPRE LAMTGYLFGS LIQHQLVDYI PLGIAIRYVL DALHCPPETN
LFKFGLQALS RFESRLQEWR PLCQALIEIP HLIEARPDLA DNIRRIGISD KILFIVNNLA
PNNFDSKLDE MKEWFGTEPN NHQLYLRLLD GMDCTVLVKF ILHETITKSA TMLNSEKTKQ
SGIDRTVLKN LGSWLDKPIK QRNISFKELL LEGADSDSLL LAIPFVCKVL EAASRSRVFK
PPNPWLIGIM GLLAELYNHA DLKLHLKFEI EVLCGALGIQ LDQVEVSSIY RGRPRVVGSH
IENILSTLTA VVKINPQLAL HTNQSFKRAV QLAIDRSVRE IILPVVERSV TIATITAREL
GVKDFASEVN EDRLRKAGQI ISQQLALSLA LVTCKEPLKS NMPTHIRHYL NEQGFNEQPV
VQMIVQDNLD VACATIERAA TDRAVVELDE ALASACEVRR RHLRERPDTQ FIANLPDILR
IKPGGLIARQ LRVYEDFFNG IVAQLESVLA QTPISLSALP PNHDIRNLLR RILQIADRSQ
TPLLLSQKLV QHLYKTTSQL GRDVYAALLE QLCRAFDEVA KQAIPWLLFA DDDRKFNIPV
MITLLRCRLI NVAEQDQHLA KDPRHALQNF SAGLIRECLT SSASQADYVY TIPCFQQWVS
VFQRSSTPEK AFIPYITQLT KQGILKAEDS SLFFRVCAES SVNAYTSAVL AGEYENAFLA
LDAMSRLIVY IIKYHGDANN DQAKTHYFSK IMSILVLVIA NFHEELGFQQ KPFFRFFSTL
LNDLHDMENY LGNVYLHLLV TLSDTFSSLQ PVYFPGFAFS WTTLISHRLF MPKLLLTDNR
EGWAAFHKLV LALLRFMAPF LRTADMQVTS RNLYRGLLRL LLVLLHDFPE FLSEYYFTLC
DAIPSRCIQL RNIVLSAYPP SCILPDPHLV HTRMGPIPPI LSDFTLVLKN GDLRTLLDQC
LLNRVTTSPL SVLADRLEMY SLTLVNATVM YIGVSSVAQA KARSGSALFA PGDPGVVAMT
YLAVNLDTEG QHHLVSAIVM HLRFPNAHTH WFGSLLLHLF AEIRDECFRE VVTKVLLERF
VVHRPHPWGA LMTFIELLRN GKYDFWSGSY IRAVPEVALL LDSV
>PS
QRQELLRAAR TKYGDEIIEP ILQRILPNLS LAPGTDLVDT FIDLGPENTG DVQSMRALLF
RFGITDANPP TDSQLLEVVQ ALARFAVEGV GVGDVSALVA AFGSFHTKLD WPAVIRSFDW
PDRSVDTATL KLLIAILLNS PEAEPHAVTG FWDIWDNALA QLRLLDALLS LPGDTFNFVT
LPGRRIVTVD DVSVASPTIK SLAANVQGHT WNSLDLFEVL VRFSEIGTPE IVSCVHEMLD
KAVKISAELV HMGLLQVEDW SGIRREYGDK LLHMFLNGHP NHQLVFMRIW QIRPTYLTDA
FRDFYNESEV NITRILDILD SLLDVQPFTF ALDEYLNLDK WLQDNINTHG KEFLMAILDF
LQAKMESEKA ARTSEVAVRT MPLNPQTITI FLRTLRNSSS MMEKKDVDYC LEVRNSCLQV
HPRLMEVEPG FTVVSYSPEI EAEVDSIYKQ MYDESITIDE VIVMLQRFKN STNPRENEIF
SCMLHFLFDE YKFFQYPARE LAMTGYLFGS LIQYELVDYI PLGIAIRCVM DALKCPPQTN
LFKFGLEALA RFEGRLAEWR PLCELLLEIP NLLQQRPELG PIIHRAESSD KMLFIVNNLA
PSNFESKIDE MREQFSTEPN NHQLYLRFLD AIGSKTLFRF ILHETFVKSA ALLNSDKTMQ
SSSERAILKN VGSWLDQPIK HKNLAYKELL LEAFDNTRLI VAIPFVCKSL EPCASSKVFR
PPNPWLMAVI GLLAELYHFA ELKLNLKFEI EVLCKALNIN LDTVEATSLI RNRPRAVGSH
IEGILSSLLS LVTISPQLAL HTNIAFKRAI QLGVDRAVRE IILPVVERSV TIAGISTREL
VVKDFATDTN VDRLQKSAHM MAQKLAGSLA LVTCKEPLRS NLATHMRQSL AEHGFGEIQI

IMLLVQDNLE FACQAIEQAA KDRAVDEIDD ALMPSYEGRR RHNQQRPNNI YPVPLPDPLR
IKASGVQPVQ LRVYEDFFLI IVKELDAILE QLPDSLTALP ANHEVRQLVR QVLFLTEPSH
TALALSQKIV QCLYRTQSQL GREIYVALLQ QLCDMFRDVR QEALPWLAEA EDDRKFNVPV
TVLLFKSGLL KVSQQDVHLA KNPRPILQTY VAGLIRECVT ANASHHQFVT CVPHFQRWVG
IYQRSPKLDV HFEYFVRELE KTRVLSTDDS LLFFRVCGEA SISHYVRSVA TGQFDYAFQA
VDAFARLVTM LVRFQGDKGF DQAKVYYLKK ILSTVTLILA HLHEEQGFQQ KPFFRFFSSL
VNDFHAIKSS LGSVYFRFLT TISETFSSLQ PRYFPGFAFS WTCLVSHRHF MPNLLMSEHR
EGWSSFHELL LSLFKFLAPF LKNADLQPAM KDLYRGTLRL LLVLLHDFPE FLSEYYFTLC
DAIPPRCVQM RNIILSAYPA GLILPDPNLR DLKMGPIPAV LSDFAAGLRD PELRSQLDQY
LVNRGTPLFL PSLKSRLEGY DLSFINSLVM YIGMSSVAQA KVRSGATLFV PTDPGVVALQ
YLATNLDMEG QYHLLSSMVL HLRYPNAHTQ WFSSLVLHLF LEIQEGRFGE VVTRVLLERF
VVHRPHPWGA MVTFIELMRN PKYEFASKEF VRIASEVTLL LESV
>RF
QRQALLAAIQ AKYGFEALGS RVHQILTMMS LSSGTSLVQT LIELGPDITS DSDIVRALLN
RFGITDQKPP RDAQIVEIFS NLGRLAAEGA TLCDVGALVR ALNSFNVQLN WAKVIESFDW
SDRGVDTATL KLLIAVLHNA PRAEPHAVAG FWTKWSNSLY QLRVLDALLS LPSDTFNFVS
LPGRRVVTVD EVASASPTIK SLAANVQGHT WNSLDLFEVL SRLGDAESPE VRGCIHDMLD
KAVKISADLV HMGLLQVPPW NAIHMEYTQK LLSMFLGGHP NHQLVFMRIW QIEPSYLTTA
LREFYEENPM NITRILDILE SLLEVRPFIF ALDEYLNLDK WLADNVNQHG AEFLHAVIAF
LELKAQNEKT SWLSDPSVRT MALNPQTIAI FLRVLRNSSS VMDEADVDYC LEVRNACLQI
HPRLMDQEPG FSVVSYMPEV EAECDQIYKK MYDEQISIDD VIALLQRSKE STNPRDHEVF
SCMLHFLFDE YKFFQYRARE LHMTGYLFGS LIQYQLVDYI PLGIAIRFVL DALQCPPDTN
LFKFGVQALS RFESRLPEWK PLCQALAEIP HLAEARPDVA EAVERAEVSD KILFLVNNLA
PSNFDAKTTE MKERFSHEPN NHSLYLRFLD ALDRPPLGKF ILNETFVKSA SVLNSEKTLQ
SASERALLKN LASWLDKPIK HKNVSFKDLL IEGADSNRLI VAIPFVCKVL ESCAKSKAFK
PPNPWLMAVI SLLAELYTFA DLKLNLKFEI EVLCKALDLD LDKIEPTSIL RTRPRAVGAH
IESILMNLAN LVVINPQLAY HTNQSFKRAV QSAVDRSVRE IIMPVVERSV TIAGISTREL
VAKDFCTEQN EEKMRKAGHL MAQKLAGSLA LVTCKEPLRS NMASHVRHYL SDHGFNEQQV
ILLIVQDNID VACAAIEKAA MDRAVADVDD GFGQAYETRR RHREQRPGAP FIATLPEPLR
IKASGLQMQQ TRVYEDFFNL LVSELEGMLP HVPNSLTVLP AGHEVRGLVR RILFLADRNR
TPLAMSQKIV QLLYKTSSQL GRELYVALLE QLCQSFEDVA KEAITWLIYA EDERKFNIQV
TVTLLRSRLI NVAQQDQQLA KDPRTTLQDF TAGLIRECLT SDAAQSQFSF AIECFQQWVN
VFQRSSVPEK SFVPYITQLT KQGILKAEDS SFFFRVCTES SIDQYMKAVA TGDYEHAFQA
LDAMSRLIVY IIKYHGDANN DQAKVHYLTK ILSIVVLVLA SRHEEEGFQQ KPFFRFFSSL
LSDLHSVEAQ LGTAYFQLLL AIGDTLSSLQ PTYFPGFAFS WMTLISHRLF MPNLLLSDNR
EGWSAFHKLL LSLFKFLGPF LRTAHLELPS RDLYRGTLRL LLVLLHDFPE FLSEYYFTLC
DIIPPRCIQL RNIILSAFPP SITLPDPHLR NIKMGPIPPI LSDFTFILKT GDLRGYLDQF
LLNRGTQASL SALKDRLEVY NLSVINAIVM YIGVSSVAQA KARSGSSVFV ASDPGVVALT
YLAVNLDPEG QHHLLSSIVL HLRYPNAHTH WFSSLLLYLF SEVKDERFKE IMTKVLLERF
IVHRPHPWGA LVTFIELLRN PKYEFWSRDF IRVAPEVTLL LESV
>RI
QRQALLAAMQ SKYGFEALGS RVHQILTMMS LSSGTSLVQT LIELGPDITS DSDIVRALLN
RFGITDQKPP RDAQIVEIFS NLGRLAAEGA TLCDVGALVR ALNSFNVQLN WAKVIESFDW
PDRGVDTATL KLLISVLHNA PRAEPHAVAG FWTKWSNSLY QLRVLDALLS LPSDTFNFVS
LPGRRVVTVD EVASASPTIK SLAANVQSHT WNSLDLFEVL SRLGDAESPE VRGCIHDMLD
KAVKISADLV HMGLLQVPPW NAIHMEYTQK LLSMFLGGHP NHQLVFMRIW QIEPSYLTTA
LREFYEESPM NITRILDILE SLLEVRPFIF ALDEYLNLDK WLADNVNQHG AEFLHAVIAF
LELKAQNEKT SWLSDPSART MALNPQTIAI FLRVLRNSSS VMDDADVDYC LEVRNACLQI
HPRLMDQEPG FSVVSYMPEV EAECDQIYKK MYDEQISIDD VIALLQRSKE STNPRDHEVF
SCMLHFLFDE YKFFQYRARE LHMTGYLFGS LIQYQLVDYI PLGIAIRFVL DALQCPPDTN
LFKFGVQALS RFESRLPEWK PLCQALAEIP HLAEARPDVA EAVERAEVSD KILFLVNNLA
PSNFDAKTTE MKERFSHEPN NHSLYLRFLD ALDRPPLGRF ILNETFVKSA SVLNSEKTLQ
SASERALLKN LASWLDKPIK HKNVSFKDLL IEGADSNRLI VAIPFVCKVL ESCAKSKAFK
PPNPWLMAVI SLLAELYTFA DLKLNLKFEI EVLCKALDLD LDKIEPTSIL RTRPRAVGAH

IESILMNLAN LVVINPQLAY HTNQSFKRAV QSAVDRSVRE IIMPVVERSV TIAGISTREL
VAKDFCTEQN EEKMRKAGHL MAQKLAGSLA LVTCKEPLRS NMASHVRHYL SDHGFNEQQV
ILLIVQDNID VACAAIEKAA MDRAVADVDD GFGQAYETRR RHREQRPGAP FIATLPEPLR
IKASGLQMQQ TRVYEDFFNL LVSELEGMLP HVPNSLTVLP TGHEVRGLVR RILFLADRNR
TPLAMSQKIV QLLYKTSSQL GRELYVALLE QLCQSFEDVA KEAITWLIYA EDERKFNIQV
TVTLLRSRLI SVAQQDQQLA KDPRTTLQDF TAGLIRECLT SDAAQSQFSF AIECFQQWVN
VFQRSSVPEK SFVPYITQLT KQGILKAEDS SFFFRVCTES SVDQYMKSVA TGDYEHAFQA
LDAMSRLIVY IIKYHGDANN DQAKVHYLTK ILSIVVLVLA SRHEEQGFQQ KPFFRFFSSL
LSDLHSVEAQ LGTAYFQLLL AIGDVLSSLQ PTYFPGFAFS WMTLISHRLF MPKLLLSENR
EGWSAFHKLL LSLFKFLGPF LRTAHLELPS RDLYRGTLRL LLVLLHDFPE FLSEYYFTLC
DIIPPRCIQL RNIILSAFPP SITLPDPHLR NIKMGPIPPI LSDFTFILKT GDLRGYLDQF
LLNRGTQASL SALKDRLEVY NLSVINAVVM YIGVSSVAQA KARSGSSVFV SSDPGVVALT
YLAVNLDPEG QHHLLSSIVL HLRYPNAHTH WFSSLLLYLF SEVKDERFKE IMTKVLLERF
IVHRPHPWGA LVTFIELLRN PKYEFWSRDF IRVAPEVTLL LESV
>GT
QRQALIMAAQ TKYGLDIVMP IVKRILPNLT LPPGTTVVQA LQQLGPEITE DPEIVLALFH
RFGISKTNPP RDEQVIEIIS TFARLATEGA VLCDVSSVIR AINSFNLELD WPAVIKSFDR
PERAVDTATL KLLITILLSA PRFEPHAVAG FWSAWENPMY QLRLLDALLS LPADTFNFVS
LPGRRIVTVD DVANASPTIK SLAANVQGQT WNSLDLFEVL VTHAGSESLE VHNYVREMLD
KAVKISAELV HMGLLQVSSW NDIRLEYTQK LLAMFLAGHP NHQLVFMRIW QIDPPYLTNA
FRDFYEESPL NITRILDILD SLLEVRPFTF ALDEYLNLDK WLADNVSAHG AEFLHSVIQF
LDIKMESEKA TRITDPAVRT MPLNPQTIAI FLRVLRNSSS MLHESDVDYC LEVRNACLQI
HPRLMDAEPG FTVVSYSPEI EAEVDAIYKQ MYDEQITIDE VIAMLQRNKA SSNPRDHEIF
SCMLHFLFDE YKFFQYPPRE LAMTGYLFGS IIQYELVDYI PLGIAIRYVL DALNCPPETN
LFKFGIQALM RFESRLSEWQ PLCQALLKIP HLLEARPELV PIIQRAETSD KILFIVNNLA
PSNFDSKVTE MKEYFSTEPN NHPLYLRFLD MLDGPILPKF ILHETYIKSA ALLNSEKTAQ
SSSERVILKN VGVWLDKPIK HKNLSFKDLL IEGYDNNRLI VVIPFVCRTL EPAAKSTVFK
PPNPWLMAII SLLAELYHFA ELKLNLKFEI EMLCKSLDID LDGIEATSVL RNRPRVVGAH
IESLLSQLSQ RVMISPQLPF AHNHVFKRAV QLAVDRAVRE IIMPVVERSV TIAGISTREL
VAKDFVTEPN EEKLRKAGHY MAQKLAGSLA LVTCKEPLKS NLTTHIRQFL LEQGFSDQSV
VMLLVQDNLD EACHAIEQAA MDRAVADVDE GFATAYELRR RHREQRTGSS FVASLPDPLR
IRAAGVQANQ MLVYEDFFAL LNQELEAFIP QMPQSLAALP PNHDLRQIIR QIVLVTEAER
TALLVSQKIV QYLYRTQWQL AREIYVALLD QLCQNFADVA KEAITWLIYA KDDRKFNVPA
TLTLLRSGLV QVQTQDEQLA KDPKIQLQNF AARLIRECLS IDGSQTQWSF TIDWFQQWVS
IYQRSPSPEK AFVPFITQLT KQGILKVEDS SFFFRVCAES SVQMYMKCVA TGDFTHAFQA
LDAMARLIVY IIKYHGDANN DQAKVHYLTK ILSIFVLVLA NMHEEQGFQQ KPFFRFFSSL
INDLHTIEAQ LGTAYFQLLI AISDTFSSLQ PTYFPGFAFS WMSLISHRLF MPKLMLHENR
EGWAAFHKLL LSLFKFLSPF LKTADFRLPS RDFYRGTLRL LLVLLHDFPE FLSEYYFSLC
DVIPPRCIQL RNIILSAFPP SLVLPDPHLH DVKMRTIPPI LSDFTASLKN GDLRDILDQH
LLGRGTPSTL PSLKERLEAY NLSLINSVVM YIGVSSVAQS KAKNGSSLFL PSDPGVVLLQ
YLVENLDVEG QHHILGAVVL HLRYPNAHTH WFSQLLLHLF SEVKDSRFRE VMTKVLLERF
IVHRPHPWGA LATFIELLSN PKYGFWNEDF VRVTPEVTLL LESV
>LB
QRQALILAAQ TKYGRETMGP IIRRIFANLS VTPGTTSVQA LVQFGPDITS DVDAIRALLE
RFGISDANPP RDAEVMEMMT TLGRLAAEGS VLCDVGALVR ALGTYHVKIS WPSVIKSFDW
PDRGVDTATL KLLIAILLNS PRVEPHAVTG FWEAWSNSLY QLRLLDALLS LPADTFNFVQ
LPGRRIVTVD DVAVASPTIK SLAANVQGHT WNSLELFQVL VRLAGSDSSE VRNCVREMLD
KAIKISAELV HMGLLQVPPW NDIRLEYSRK LLAMFLAGHP NHQLVFMRIW QIEPSYLTDA
FRDFYDENPL NITRILDILE SLLDVRPFTF ALDEYLNLDK WLADNVANHG GEFLHSIIRF
LEQKMESEKT CRLSDPAVRT MSLNPNTITI ILRVLRNNSA AMLEGDIEAC REVRNACLQV
HPRLMDIEPG LTVVTYSAEI EAEVDGIYQQ MYDENTSIDD VIAMLQQHKE SANPRDHEVF
SCMIHFLFDE YKFFQYPARE LAMTGYLFGS LIRHQLIDYI PLGIAIRYIL DALNCPPETN
LFKFGIQALG RFEFRLTEWR PLCEALLQIP HLAEVRPDLM AIIQRGELSD RILFIVNNLA
PSNFDVKLDE MREQFSSEPN NHSLYLRFLD ALNRQKLSKF VLQETFIKAA ALLNSERSMQ

SGSDRNTLKN VGAWLDQPIK FKNLSFKELL IEGYESSRLI VAIPFVCKTL EPCVKSKVFK
PPNPWLMAVM SLLAELYHFA ELKLNLKFEI EVLCKSLDIN LDAVEATTIL RNRPRAVGAH
IEAILLNLSH QVQINAQLSL NVNHAFKRAV QLAVDRAVRE IIIPVVERSV TIAGISTREL
VTKDLATDPN EDKVRRAGHL MAQKLAGSLA LVTCKEPLKS NLATHLRQYL NEHGFNEQQV
VLLLVSDNLD FACTAIEKAA MDRAVSDVDE GFATSYESRR RHREQRNGSN FSSTLPDPLR
IKSTGLQQHQ FRLYEDFFGV LTRDLEAVMV QIPQSLAALP PNHDIRHLVQ QVVILADRER
TPLMMSQKIV QLLYKTPSQL GREVYVALLD QLCRTFEDVA KEAITWLLYA EDERKYNVPV
TLALLKSGLI NTTLQDQQLA KDPRPTLLAY TAALIRECLS SDASQNQFQY SIEWFQQWVN
IFQRSHSPEK NFVPFITQLT KQGILKVEDS SFFFRVCAES SVNSYIKSIS VGDYDYAFQA
LDAVSRLIVY IIKYHGDANN DQAKVHYMTK ILSIFVLVLA NLHEEQGFQQ KPFFRFFSSL
VNDLQSVESH LGPAYFQLLI AISDTYSSLQ PTYFPGFAFS WMCLISHRLF MPKLLLSENR
EGWSAFHKLL LSLFKFLSPF LKDADLQPAA RDLYRGALRL LLVLLHDFPE FLSEYYFTLC
DAIPPRCIQL RNIILSAFPP AVILPDPHLR NVNMGPIPPI LSDFTSGLKS GDLRGYLDQY
LLNRGTPSFL HSLKDRLESY NLSLINSLVM YIGVSSVAQA KARSGSSLFI ASDPGAVALQ
YLTTNLDVEG QHHLMSSIVL HLRYPNAHTH WFSSLLLHLF VEVKDDRLRE VMTKVLLERF
IVHRPHPWGA LVTFIELLRN PKYDFWSKDF IRIAPEVTLL LESV
>CP
QRQSLIAAAQ AKYGPDIVAP ILQKIFPTMS LPPNASLVQT LVQLGPEITS DADVVKALLM
RFNITESSPP REAQVTEIVT SLARFVTEGV PLCDVGALIR ALSSLQPTMN WVNVIKSFDR
PDRGVDTATL KLIISVLMNS PLVTPHAVSG FWEPWSNSLY QLKLLDALLS LPGDTFNFVS
LPGRRVVTVD DVASAGPTIK ALASNVQVHT WNSLELFEVL VSLADSESAD TRAFIREMLD
KAIKISAELV HMGLLQVQNW NEIRTEYSRK LLGLFLAGHP NHQLVFMRLW QIDPTYLLDA
FRDFYNENPL NITRILDILE NLLEAQPFTF ALDEYLNLDK WLADHVAEHG ASFLHAMVSF
LEIKMESEKT ARLSDPAVRT IPLSPQSVTV FLRTLRSNTS VMSREDIDYC LEVRSACLQV
YPRLMEVEPG FTVVNYSPDI EAEVDAIYKQ MYDEQTTIDE VIAMLVRCKG SSDQRDSEIF
SCMLHFLFDE YRFFQYPARE LAMTGYLFGS IIQHQLVDYV PLGVAIRYVV DALSCPADTN
LFKFGLQALG RFESRLPEWQ PLCQALLRIP TLMEARPDLS SVIHRAELSD KILFIVNNLS
PNNLEAKLAE MKEYFSTEPN NHQLYLRFLD GLDRKVLSKF ILHETFVKSA SVLNHEKTMQ
QTSERHILKN IGAWLDRPIK HKNLSFKDLL IEGYDNGRLM VAIPFVCKTL EPCAKSKVFK
PPNPWLMAVI SLLAELYHFA DLKLNLKFEI EVLCKGLDID LDTVEATTML RTRPLDVGGH
IESILQGLSQ RVVINPQLAL HINQAFKRAV QLAVDRSVRE IIVPVVERSV TIAGISTREL
ATKDFATEPS EDKLRKAGHL MAQKLAGSLA LVTCKEPLKS NLATHLRSFL VDHGFNEQQV
FAILVQDNLD VACSAIEKAA MERVISDVDE GFAASYDLRR RHREVRGGSN FTINLPDPLR
IKANGLQANQ FAVYEDFFTA ILRDLEALMT QLPQSLASLP PNHDVRHLVR QILYLADRHR
TPLMMSQKIV QLLYKTSSQL GREIYVALLD QLCRSFEDVA KEAITWLVYA EDERKLNIPV
TVTLLRSGLI SFSLQDQQLA KDPRPSLLNF AAGLIRECLS GDASVSQFTF SLEWFQQWVQ
IYQRSHTPEK AFVPYITQLT KQGVLKAEDS SFFFRVCAES GVNSYLKCVA AGDYEHAFQA
LDALSRLIVY IIKYHGDANN DQAKVHYFTK ILSIFVLVLA NMHETQGFQQ KPFFRFFSSL
INDLHAVESH LRTAYFQLLL SISDTLSSLQ PTYFPGFAFS WLCLISHRLF MPKLLLSENR
EGWSAFHRLL LSLFKFLAPF LKEADLQIAS RDLYRGSLRL LLVLLHDFPE FLSEYYFTLC
DSIPSRCIQL RNIILSAFPS TITLPDPHLL NYKMGPIPPI LSDFTSNLKN GDLRTHLDQY
LLNRGSPTFL PSLKDCLDSY NLPLINSLVM YVGVSSVAQA KARSGSSIFV SGDPGVVALH
YLATNLDVEG QHHLLSSMVM HLRYPNAHTH WFSSLLLYLF VEVQDDHFRE VMTRVLLERF
IVHRPHPWGA LVTFIELLRN QKYEFWSKEF TRVAPEVHML LDSV
>SL
QRQALIAAAQ AKYGPEIVAP ILQRIFPTLS LPPGASLVQT LIQLGPDITS DADTIRALLL
RFGISDATPP RDSQVIELIT SLARLAAEGT TLCDVGALVR ALSSFPVNLN WANVIKAFDW
PDRGVDTATL KLLIAILVNC PRADPHAVTG FWGTWSNSLY QLKLLDALLS LPADTFSFVS
LPGRRIVTVD DMANASPTIK SLAANVQGHT WNSVELFEVL VRQSCSESID IKNCVQEMLD
KALKISAELV HMGLLEVKRW SEIRIECSQK LLNMFLAGHP NHQLVFMRLW QIEPSYLTDA
FRDFYEESPL NITRILDILE NLLEVRPFTF SLDEYLNLDK WLLDNVNNHG AEFLHAAILF
LEIKMDAEKA ARVSDPATRT MSLNPQIIAV FLRLLRHNSA KMSREDIDYC LDVRNTCLQV
YPRLMDAEPG LTVVNYSPEI EAEVDAIYKQ MYDEQTTIDE VVSLLQRSKA STDSRDHEIF
SCMLHFLFDE YKFFQYPPRE LAMTGYLFGS LIQHQLVDYL PLGIAIRYVV DALNCPPETN

LFKFGLQALS RFESRLSEWQ PLCQALLRIP HLMEARPDLT AVIHRAELSD KILFIVNNLA
PSNFDSKLAE MQEHFSTEPN NHQLYLRFLD ALDKQPLAKL ILQETFIKSA ALLNSEKTAQ
NSSERATLKN VGAWLDKPIM HKNLSFKDLL VEGYDNGRLI VSIPFVCKTL EPCARSKVFK
PPNPWLMAVI SLLAELYHYA DLKLNLKFEI EVLCKGLDID LDAVEATTVL RNRPRAISAH
IEAILATLVH HVVINPQFPL QSNHSFKRAI QLAVHHAVRE IIMPVVERSV TIAGISTREL
VAKDFATEAS EEKLRKAAHL MAQKLAGSLA LVTCKEPLKS NLGTHIRSFL AEHGFNEQHV
IVILVQDNLD IACSAIEKAA MERAISDVDE GFAASYDVRR RHHETRNPSS FSGNLPEPLR
IKSTGLQPHQ AAVYEDFFSV LLRDMEAVVM QVPQSLASLP PNHDIRHMVR QILYLADRQR
TPLLMSQKIV QLLYKSSSQL GREIYVTLLD QLCHSFEDVA KEAITWLLYA EDDRKLNVPV
TVALLRSGLV NMSLQDQQLA TEPRPTLLTF AANLIRECLS SEASQSQFAY SLEWFQQWVA
IFQRSHSPEK AFVPFITQLT KQGILKVEDS SFFFRVCAES SVNSYIKCAS TGEYEYAFQA
LDAMSRLIVY IIKYHGDANN DQAKVHYLTK ILSIFVLVLA NMHEEQGFQQ KPFFRFFSSL
INDLHSIEAH LGTAYFQLLI AISDTFSSLQ PTYFPGFSFS WMCLISHRLF MPKLLLSENR
EGWSAFHKLL LSLFKFLSPF LKEADLQVPS RDLYRGSLRL LLVLLHDFPE FLSEYYFTLC
DVIPPRCIQL RNIILSAFPP AIILPDPHLR NIKMGPIPPI LSDFASGLKN GDLRNYLDQY
LLNRGTPSFL PSLKERLESY NLSLINSLVM YIGVSSVAQA KARSGSSLFV ASDPGVIALQ
YLVTNLDVEG QHHILSSMVL HLRYPNAHTH WFSSLLLHLF VEVKDERFKE VMTRVLLERF
IVHRPHPWGA LVTFIELLRN PKYEFWHKEF IRVAPEVTLL LESV
>FS
QRQAIVAAAQ AKYGNEIVAP ILQRIFPTLS LPPNTSLVQT LIQLGPDITS DPDTVRALLL
RFGISEVNPP RDVQVVEIIS ALARLAADGT AMCDVGALVR ALSSFPVNLD WAAVIKSFDW
PDRGVDTATL KLLIAILVNC PRAEPHAVTG FWLTWTNPMY QLRLLDALLS LPADTFNFVT
LPGRRVVTVD DVAGASPTIK SLAANVQGHT WNSLDLFEVL VKLGDSDVME IRNTVREMLD
KAVKISAELV HMGILQVPHW GEVRLEYSRK LLAMFLGGHP NHQLVFMRIW QIEPTYLTDA
FRDFYEESPL NITRILDILE SLLQVQPFIF ALDEYLNLDK WLADNVNNHS GDFLHAVIQF
LDIKMESEKV ARISDPAVRT MPLSPQTITI FLRMLRSNSA KMHNDDVDYC LEVRNACLQI
HPRLMDVEPG FTEVRYSADI ENEVDSIYKQ MYEEQITIDE VIALLQRTKN SHDPHDHEIF
SCMLHFLFDE YKFFQYPPAE LALTGYLFGS LIQYQLVDYI PLGITIRYII DALTCPPETS
LFKFGIQALK RFKDRLSEWQ PLCQALLNIP HLMEAHPELA STIHRADVSD RILFIVNNLA
PSNFDSKLKE MKEQFSTEPN NHSLYLRFLD ALDRQALSKY VTQETLVKSA TMLNSERTLQ
NSSERATLKN VGAWLDVPIK HKNLSFKDLL IEGYDNGRLI VAIPFICKTL EPCAKSKVFQ
SPNPWLMAVV SLLAELYHYA DLKLNLKFEI EVLCKGLDID LDTVDATTVL RNRPRAVGAH
IESILASLAH HVIVSQQLAL HGNHSFKRAV QLAVDRAVRE IILPVVERSV TIAGISTREL
VAKDFATEPS DDKLRKAGHL MAQKLAGSLA LVTCKEPLRS NLGTHLRQHL AEHGFAEVQV
LAILAADNLD VACAAIEKAA MERAVSDVDE GFAASYEARR RHRELRNGSN FALNLPDPLQ
IKANGLQPHQ AGVYEDFFSA TIRDLEAVMI QLPQSLAALP PNHDIRHLVR QILFLADRHR
TPLLMSQKIV QLLYKTSSQL GREVYVALLD QLCHSFEDVA KEAITWLLYA EDERKFNVPV
TVTLLQSGLV NISLQDQQLA KEPRPSLLNF AAALIRECLS TDASQSQFAY SIEWFQQWVA
IFQRSHSPEK SFVPFINQLT KQGILKVEDS SFFFRVCAES SVNSYVKHIA AGDFEYAFQA
LDAMSRLIVY IIKYHGDANN DQAKVHYLTK ILSIFVLVLA NLHEEQGFQQ KPFFRFFSSL
INDLHSIEAQ LGSAYFQLLI AISDTFSSLQ PTYFPGFAFS WMCLISHRLF MPKLLLSENR
EGWSAFHKLL LSLFKFLSPF LKEADLQHAS RDLYRGGLRL LLVLLHDFPE FLSEYYFTLC
DVIPPRCIQL RNIVLSAFPP SITLPDPHLR SVKMGPIPPV LSDFTSGLKS GDLRSYLDQY
LLNRGTPSFL PSLKDRLESY NLPLINSLVM YIGVSSVAQA KARSGSALFV PSDDGVVALQ
YLATNLDVEG QHHLLSSVVL HLRYPNAHTH WFSSLLLHLF LEVKDDRFRE VMTRVLLERF
IVHRPHPWGA LVTFIELLRN PKYDFWSKEF IRVAPEVTIL LESV
>PI
QRQALIAAAQ AKYGNEIVAP ILQRIFPTLS LPPNTSLVQT LIQLGQDITS DPDTVRALLL
RFGISESNPP RDAQVVDIMS TLARLAAEGT TMCDVGALVR ALSSFHVSLD WASVIKAFDW
PDRGVDTATL KLLIAILVNC PRPEPHAVTG FWSTWANPMY QLRLLDALLS LPADTFNFVS
LPGRRVVTVD DVAGASPTIK SLAANVQGHT WNSLDLFEVL VRLADSESVD IRNCVREMLD
KAVKISAELV HMGLLQVPNW NEIRLEYSRK LLAMFLGGHP NHQLVFMRIW QIEPSYLTDA
FRDFYEESPL NITRILDILE SLLQVQPFVF ALDEYLNLDK WLADNVTNHG GDFLHAVIQF
LDVKMESEKA TRVSDPAVRT MQLSPQTITI FLRMLRNNSV KMNPDDVDYC LEVRNACLQI

HPRLMDVEPG FTEVRYAQDI ESEVDSIYKQ MYDEQITIDE VIALLQRTKS SHDPRDHEIF
SCMLHFLFDE YKFFQYPARE LAMTGYLFGS LIQHQLVDYI PLGIAIRYIL DALTCAPDTN
LFKFGLQALS RFENRLSEWQ PLCQALLRLP HLMEARPELA GSIHRADVTD RILFIVNNLA
PSNFDSKLTE MREQFSTEPN NHSLYLRFLD ALDRQVLFKL ITEETLVKSA SMLNSEKTLQ
NSSERATLKN VGAWLDIPIR HKHLSFKDLL IEGYDSGRLI VAIPFVCKTL EPCGKSTVFK
PPNPWLMAVV SLLAELYHYA DLKLNLKFEI EVLCKSLDID LDSVEATTVL RNRPRVVGAH
IESILSGLAH HVTVSQQLTL HVNPSFKRAV QLAVDRAVRE IILPVVDRSV TIGGISTREL
VAKDFATEPS DEKLRKAGHA MAQKLAGSLA LVTCKEPLRS NLGTHLRQHL AEHGFAEVQV
LAILAADNLD VACSAIEKAA MERAVSDVDE GFAASYEARL RHRELRNGSN NTISLPDPLQ
IKANGLQPHQ ASVYEDFFTV LIQDLEAVMV QLPQSLAALP PNHDIRHLVR QILFLADRHR
TPLLMSQKIV QLLYKTPSQL GREVYVALLD QLCRSFEDVA KEAITWLLYA EDERKFNVPV
TVTLLQSGLV DIPLQDQQLA KDPRPSLLNF ASALIRECLS TDASQSQFAY SIEWFQQWVN
IFQRSHSPEK SFVPFINQLT KQGILKVEDS SFFFRVCAES SVNSYVKHVA TGDYDYAFQA
LDAMSRLIVY IIKYHGDANN DQAKVHYLTK ILSIFVLVLA NLHEEQGFQQ KPFFRFFSSL
INDLHAIETQ LGTAYFQLLI AISDTFSSLQ PTYFPGFAFS WMCLISHRLF MPKLLLSENR
EGWSAFHKLL LSLFKFLSPF LKEADLQHAS RDLYRGALRL LLVLLHDFPE FLSEYYFTLC
DAIPPRCIQL RNIILSAFPP TIALPDPHLR NVKMGPIPPI LSDFTSGLKS GDLRSYLDQY
LLNRGTPSFL PSLKDRLENY NLPLINSLVM YIGVSSVAQA KARSGSSLFV ASDDGVVALQ
YLATNLDVEG QHHLLSSVVL HLRYPNAHTH WFSSLLLHLF LEVKDDRFRE VMTKVLLERF
IVHRPHPWGA LVTFIELLHN TKYDFWSKDF IRVAPEVTLL LDSV
>HI
QRQALIAAAQ AKYGSETVAP MLHQIFPRLS LPPGTTLVQA FNQFGPEITN DVDTVRALLL
RFGVSDSNPP RDAQVIEYMS TLARQAAEGS TLGDVNAFVR ALSSYSVPLN WANAIKAFDI
PDRGVDTATL KLLIAILLNS PRAEVHAVTG FWSMWSNSLY QLRLLDALLS LPADTFNFVT
LPGRRIVTMD DVTNASPTIK ALAANVQGHT WNSLDLFEVL VRLADSDSAD IRNFVREMLD
KAVRISAELV HMGLLEAPSW NEIRLEYSQK LLNMFLAGHP NHQLVFMRIW QIQPTYLTNA
FRDFYEDSPL NITRILDILD ALLDVRPFAF ALDEYLNLDK WLADNVTAHG ADFLHDVIAF
LDTKMESEKA TRISEAAVRT MTLNPLTITI FLRVLRNSAS IMHPNDVDYC LEVRNACLQI
HPRLMDAEPG FSVVTYASDI EVEVDGIFKQ MYDEHITIDE VIAMLQRNKN SSNSRDHEIF
SCMLHFLFDE YKFFQYPPRE LAMTGYLFGS IIQYELVDYI PLGIAIRYVI DALNCSPETN
LFKFGIQALS RFESRLSEWQ PLCQALLSIP HLLEARPDLA ASIQRAEVSD KILFIVNNLA
PTNFDSKLED MKGSFSTEPN NHQLYLRFLD ALDKGTLLKF VLQETIAKSA HLLNAEKTMQ
SSSERAILKN MGSWLDKPIK HKNLGFKELL VEGYDNGRLI VAIPFVCKTL EPAAKSKVFR
PPNPWLMAVV SLLAELYHYA ELKLNMKFEI EVLCKSLDID LDTVEATAVL RSRPRVIGSQ
IEVILSDLIQ HVTVNAQLAF STNHTFKRAV QLAVDRSVRE IILPVVERSV TIAGISTREL
VAKDFATEGN EETLRQAAHS MAQKLAGSLA LVTCKEPLRS NLSSHMRQFL IEHGFSEQPV
VVMLVQENLD LACQAIEKAA MERAIADVDE SFAPAYEARR RHRQTNRGSN FSQTLPDPLR
IKPSGVQPNQ IGVYEDFFGA LVKELEAVLI QLPQSLAALA SNHEVRHLVR QILYLADRHR
TPLLISQKIV QLLYKTPSQL ARDIYVALLD QLCHSFEDVA KEAITWLIYA DDERKLNIPV
TVTLLRSGLI TIAQQDQQLA KDQRPSLQNF AAGLIRECLT CDASQSQFSF TIEWFQQWVT
VFQRSHSPEK SFVAYITQLT KQGILKAEDS SFFFRVCAES SVNSYLKCMA SGDYAYAFHA
LDAMSRLIVY IIKYHGDANN DQAKVHYLTK ILSIFVLVLA NMHEEQGFQQ KPFLRFFSSL
INDLHAIEGH LGSVYFQLLL AISDTFSSLQ PTYFPGFAFS WMSLISHRLF MPKLLLSENR
EGWSAFYKLL LSLFKFLSPF LKSAELQQSS RDLYRGSLRL LLVLLHDFPE FLSEYYFSLC
DMIPPRCIQL RNIVLSAFPP TLTLPDPHLR NVKMGPIPPI LSDFTSGLKT GDLRTHLDQY
LLNRGSPSFL PSLKDRLETY NLSLINSLVM YIGVSSVAQA KARSGSSLFN AGDPGVVALR
YLATSMDTEG QHHLLSSIVL HLRYPNAHTH WFSSLVLYLF VEVKDDMFSE VVTKVLLERF
IVHRPHPWGA LVTFIELLRN PKYDFWSKDF IRAAPEVTLL LESV
>SH
QRQALIAAAQ AKYGSETVAP MLHQILPRLS LPPGTTLVQT FNQLGPDITN DVDTIRALMQ
RFGMTEANPP TDIQIVEYMS TLARQAAEGT TLGDVNAFVR ALSNSSTTLN WANVIKAFDI
PDRGVDTATL KLLIAILLNS PRTEPHAVTG FWMPWTNSIY QLRLLDALLS LPGDTFNFVS
LPGKRIVTMD DVTNASPTIK ALAANVQGHT WNSLDLFEVL VRAADSDSTD LRNLVREMLD
KAVRISAELV HMGLLEAPTW NEIRLEYSNK LLGMFLGGHP NHQLVFMRIW QIQPTYLTNA

FRDFYEESPL NITRILDILD SLLDVRPFGF ALDEYLNLDK WLSDQVSKHG ADFLHDVIAF
LDAKMESEKT TRVSDPQVRT MTLNPLTITI FLRFLRNNAT SMRPNDVDYC LEIRNACLQI
HPRLMDAEPG FTVINYSPEI EAEVDGIFKQ MYDEQITIDE VIAMLERNKS STNPRENEIF
SCMLHFLFDE YKFFQYPARE LAMTGYLFGS IIQFQLVDYI PLGIAIRYVI DALNCPPETN
LFKFGIQALS RFESRLSEWQ PLCQALLNIP HLLEARPDLG ATIQRAEVSD KILFIVNNLA
PTNFEAKLED MKGSFSTEPN NHNLYLRFLD ALDRKVLSKF VLQETIVKSA SMLNSEKTMQ
SSSERSVLKN VGSWLDKPIK HRNLSFKDLL IEGYESGRLL VAIPFICKTL EPAAKSTVFR
PPNPWLMAVM ALLAELYHFA ELKLNQKFEI EVLCTSLSVA LDSIEPTAIL RHRPRAVGAQ
IEVLLGELVG RVTISGQLAL PSNPAFKRAV QLAVDRSVRE IILPVVERSV TIAGISTREL
VAKDFATEPN EETLRGAAHS MAQKLAGSLA LVTCKEPLRS NLSNHLRQFL NDHGFSDQAV
IMLLVQDNID LASGTIEKAA MDRAVAEVDE GFAGAYDARR RHRQTAPRSA FSASLPDPLR
IKVNGVQPNQ IGVYEDFFNA IIKELDAVLP QLPSALSTPS STPDLRIPVR QILFIADRVR
TPLLISQKIV QLLYKTNVQL ARDIYVMLLD QLCHAFDEVA KEAITWLIYA DDERKLNVPV
TVTLLRSGLI TIAQQDQQLA KDQRPSLQNF AAGLIRACLT SDASQSQFTY TIEWFQQWIV
IFQRSPSPEK NFVPFITQLA KQNILKADDS SFFFRVCAES SVESYMKSMA RGDFTYAFQS
LDAVARLIVY IIKYHGDANN DQAKVHYLTK ILSIFVLVLA NMHEEQGFQQ KPFLRFFSSL
INDLHSIEKD LGAVYFQLLI AISDTFSSLQ PTYFPGFAFS WMSLISHRLF MPKLLLSQNR
EGWSAFYKLL LSLFKFMSPF LKSAEFQTSS RDLYRGSLRL LLVLLHDFPE FLSEYYFTLC
DVIPSHCIQL RNIILSAFPP TLVLPDPHLR NVKMGPIPPI LSDFTSGLKT GELRNYLDQY
LLSRGSPAFL SSLKDRLEEY NLSLINSLVM YIGVSSVAQA KARSGSSLFN PADPGVVALQ
YLATNLDTEG QHHLLSAMVL HLRYPNAHTH WFSSLLLHLF VEVKDDMFCE VTAKVLLERF
IVHRPHPWGA LVTFIELLRN SKYDFWTKDF IRAAPEVTLL LESV
>PC
QRQALIMAAQ TKYGPEIIMP ILQRILPNIH LQQNTTLVQL MIQLGPEMTC DPDITSAVLA
RFGMTENNPP QDEQVVEIVS QLARLAAEGP VSVDVGTLVH VLARLNPSLN WAMAIQAFDR
PDRGVETSTL KLLIAILLSC PLSEHHAVSG FWQMWKNSLY QLRLLDALLS LPSDTFNFVT
LPGRRIVSTE DIPATSPSIK ALAANVQVHT WNSLDLFEVL VRLADSESLE VRNFVREMLD
KAVKISAEIV QMGLLQVTPW GEIRLEYSQR LLAMFLAGHP NHQLVFMRIW QIEPKYLLNS
LREFYEENPL NITRILDILD HLLDCKPFAF ALDEYLNLDK WLADNVTAHG ADFLHGVIGF
LDSKMESEKL TRISDPAVRT MPLSPQTITI FLRVLRANSN LMHESDVDYC LEVRNACLQI
HPRLMDVEPG FNVITYSPEI EAEVDSIYKQ MYDEHITIDD VIALLQRNKT SNNPRDHEIF
SCMLHFLFDE YKFFQYPPRE LAMTGYLFGS LIQYQLVDYI PLGVAIRYVL DALNCPPETN
LFKFGLQALV RFESRLPEWQ PLCQALLKIP HLLDARPELV NILSRAEVSD KILFIVNNLA
PSNFDAKLTE MKEQFSTEPN NHQLYLRFLD ALNIKTLFNF VLHETFVKSA QLLNSEKTLQ
VTSERTILKN VGAWLDRPIK HKNLSFKELL IEGFDNGRLI VAIPFVCKTL EPCARSKVFR
PPNPWLMAVI SLLAELYHFA ELKLNLKFEI EVLCKSLEID LETVQATTIL RNRPRALDAH
IENILASVLP LVVINPALAL HTNQTFKRAM QMAVERSIRE IIMPVVERSV TIAGISTREL
VLKDYVTEQS EEKLRHAGRL MSQKLAGSLA QVTCKEPLRS NLASHLRLVL TEFGFAEVEV
IALLVNDNVD IACQAIEKAA MDKAALEVDE AFVTHFEARR RHREQRPGSQ FANSLPDPLR
IRPIGVQPIQ AAVYEDFFTA LIKDLESVLP QLPQSLAALP PNHEIRQLLR EILFLTDRQR
TPLLISQKIV QLLYKTTLQL AREIYVALLD QLCHSFEEVA KEAINWLIYA EDERKFNVPV
TVTLLRSGLV NIGQEDQQLA KDPRPSLLNF AAGLIRECLS SDASQAQFAY TLEWFQQWVH
IYQRSPSPEK SFVPYISQLT KQGILKAEDS SFFFRVCMES SVNSYVKCVN SGEFDYAFQA
LDAFSRLIVY MIKYHGDANN EQAKVHYLTK ILSIVVLVLA NMHEEQGFPQ KPFFRFFSSL
LNDLHAIEAS LGAVYFPLLL AVSDTFSSLQ PTFFPGFAFS WMSLISHRLF MPKLLLSESR
EGWPTFYRLL LALFKFLAPF LKAADLQPAT RDLYRGSLRL LLVLLHDFPE FLSEYYFTLC
DIIPPHCIQL RNVILSAFPP TIILPDPHLR NVKMGPIPPV LSDFSSVLKA GDLRTYLDQY
LLNRGSPSFL PSLKERVDKY NLSLMNALVM YIGVSSVAQA KARSGSSLFV ASDPGVVALQ
YLANNLDIEG QHHLLSAMVL HLRYPNAHTH WFSSLMLHLF NEIKDDKFRE IMTKVLLERF
LVHRPHPWGA LVTFIELLRN PKYDFWNHDF IRIAPEVTLL LETV
>WC
QRQALIAAAH AKYGSEIVVP ILQRILPHMS LPPETTLVQA LVQMGADVTS DPEVIRALLV
RYGISDKDPP TDKQVVEIIE SLARLASEGA ALPDVGSLVR ALSSFGGKIQ WANAIKVFDM
PERGVDTATL KLLIAILLNA PRADPHAVTG FWQIWNNSMY QLRLLDALLS LPADTFNFVS

LPGHRIVTVD DVNTASPTVK SLAANVQGHT WNSLDLFEVL VRLADADSPG LRNFVRDMLD
KAVKISAELV HLGLLQAPNW GEIRLAYTQR LLSMFLAGHP NHQLVFTRIW QIEPTYLTNA
LREYYDESPQ NITRILDILD TLLEVRPFTF ALDEYLNLDK WLADNVATHG AEFLHAVIEF
LEIKMEDEKA TRITDPAVRT LQLSPQTITI FLRVLRNNSN IMHESDIDYC LQIRNACLQI
HPRLMDAEPG FTVITYPPDI EAEVDAIYRQ MYEDQISIDD VIVLLQRNKA SNNPRDHEIF
SCMLHFLFDE YKFFQYPPRE LAMTAYLLGS IIQCELVDYI PLGIAIRYVL DALKCPPETN
LFKFGLQALS RFEGRLHQWR PLCNALLDIP ALLEVRPELG IIIRRAEVSD KILFIVNNLA
PSNFDSKLTE MREHFSTEPN NHQLYLNFLD ALDVQALFRF VLHETYIKSA ALLNSEKTMQ
STSERTVLKN VASWLDRPIR HKNLSLKDLL IEGYDNGRLI VAVPFVCKTL EPCAKSKVFK
PPNPWLMAVI SLLAELYRYA EMKSLLKFEI ELLCKALDIN LDAIQPTTIL RNRPRVLGTH
IENILQSLLP HVIIHPQLAL HTNQTFKRAV QMAIDRSVRE IIVPVVERSV TIAGISTREL
VAKDFAMEPS EEKMRKAGHL MAQKLAGSLA LVTCRDPLKT NLVGHIRTFL NECGFNEQPV
VQLLVQDNLE LACQVIEKAA MDRAIVDVDD GFAAAYEVRR RHRETHPGHP LATQFPEPLR
IKPAGIQQIQ AVVYEDFFNA MVKDLEALLL QLPQSLAALP PTHEIRLLVR QILYLADHQR
TPLLMSQKIV QLLYKTSSPL GREIYVALLD QLCHIFEDVA KEAITWLIYA EDERKFNVPV
TVTLLRSRLI SIEQEDQQLA KNPRPTLLTF AAELIRECLS IDATQSQFAY TLEWFQQWVS
IFRGSHTPEK SFIPFITQLT KQGILKVEDS SFFFRVCAES SVNSYIKCVN AGEYGLAFQA
LDAMSRLIVY IIKYHGDANN DQAKVHYLTK ILSIFVLVLA NMHEEQGFQQ KPFFRFFCSL
LHDLHANASS LETAYFPLLL ALSDTFSSLQ PTYFPGFAFS WMSLISHRLF MPKLLLSENR
ERWSAFYKLL ISLFKFLSPF LKAADLQQAS RDLYRGSLRI LLVLLHDFPE FLSEYYFSLC
DVIPARCIQL RNVILSAFPP GVMLPDPHLR SIDTGPIPAI LSDITSGFKP GDLQGYLDQY
LLNRGSPSVL SSLKDRLESY NLSLINSLVM YIGVSSVAQA KTRSGSALFV STDPGVVALQ
YLATNVDAEG QHHLLSAMLL HLRYPNAHTH WFSSVMLHLF LEIKSEQFRE VMTKVLLERF
LVHRPHPWGA LVTFIELLRN PKYDFWSQEC IRSVPEVTIL FEQV
>DS
QRQALILAAQ AKYGSEIVSP ILQHIMPNLS LPPGTSLVQA LVQLGPDITS DADVVRSLMA
RFGISEVNPP TDAQVVDLVT SLARLASEGT SLPDVGAVVR ALSSFNSSLN WAAAIQAFDI
PDRGVDTATL KLLIAILMNT PREQHHAVTG FWSLWSNTQY QLRLLDALLS LPADTFNFVN
LPGRKIVTVE DVAGASPTIK SLAANVQGHT WNSLDLFEVL VQAADFNSTE ITNLVREMLD
KAVKISAELV HMGLLQVPSW NDIRLEYTQR LLAMFLAGHP NHQLVFMRIW QIEPAYLTNA
FRDFYDESNL NITRILDILD ALLEVRPFKF ALDEYLNLDK WLADNVTTHG ADFLHAVIAF
LELKMDSEKT VRVSDPPVRT MQLSPQTIAI FLRVLRNSSS IMHENDVDYC LEVRNACLQI
HPRLMDIEPG FSVVTYSTEI ETEVDGIYKQ MYDEQITIDD VIKLLQRNKA SSNPRDHEIF
SCMLHFLFDE YKFFQYPHRE LAMTGYLFGS LIQYQLVDFI PLGIAIRYVL DALNCPPETN
LFKFGIQALS RFESRLSEWQ PLCQALLKIP HLLEARPDLA VSIQRAEVSD KILFIVNNLA
PSNFESKLAE MKGHFSIEPN NHQLYLRFLD ALDVQSLFRF VLHETLVKSA VLLNSEKTQQ
LSSERAVLKN VGSWLDRPIK HKNLSFKDLL IEGYDNNRLV VAIPFVCKTL EPAARSKVFR
PPNPWLMAVI SLLTELYHFA ELKLNLKFEI EMLCKALDID LDVVQATTIL RNRPRVLGAH
IENILSSILP HVTINPQLAL NTNPSFKRAI QMAIDRAVRE IILPVVERSV TIAGISTREL
VAKDFVTEPN EDKLRKAGHL MAQKLAGSLA LVTCKEPLKG NLGSHIRQFL SEFGFTDQQV
IFLLVQENIE LACQAIEKAA MDRAVIDVDD GFAAAYELRR RHRESRPGSN VFGSLPDPLR
IKPTGVQQIQ AAVYEDFFNA LVRDLEAVLI QLPASLAALP PNHEVRHLVR QILFIADRSR
TPLLMSQKIV QLLYKTPSQL GREIYVALLD QLCHSFEEVA REAITWLIYA EDERKFNVPV
TVTLLRSGLV TISQEDQQLA KDPRPSLQNF AAGLIRECLA ADATQAQFSY SLEWFQQWIS
IYQRSHSPEK SFVPYITQLT RQGILKAEDS SFFFRVCAES SVNSYIKHVN AGEFGFAFQA
LDAMSRLIVY IIKYHGDANN DQAKVHYLTK ILSIFVLVLA NMHEEQGFQQ KPFFRFFSSL
LNDLHSVESS LGSAYFQLLI AISDTFSSLQ PTYFPGFAFS WMSLISHRLF MPKLLLSDNR
EGWSAFYKLL LSLFKFLAPF LKTADLQPAG RDLYRGTLRL LLVLLHDFPE FLSEYYFSLC
DVIPPRCIQF RNIVLSAYPP NVVLPDPHLR DIDMGPIPPI LSDFAASLRA GDLRMYLDQF
LLNRGPQTFL SSLKDRLETY NLPLINSLVM YIGVSSVAQA RARSGGPLFV PTDPGVVALH
YLATNLDVEG QHHLLSAMVL HLRYPNAHTH WFSSLMLYLF HDIQVDQFRE IVTRVLLERF
LVHRPHPWGA LVTFIELLRN PKYNFWNQEF IHIAPEVTLL LENV
>TV
QRQALILAAQ AKYGSEIVSP ILQRILPNLS LPNGTSLVQA LVQLGPEATA DPDVVRALFV

RFGLTENNPP TDAQLVEVIT SLARLASEGT HLPDVGSVVR ALNSLNGELN WAAAIKAFDM
PDRGVDTATL KLLIAILMNA PRDNQHAVTG FWQLWSNTLY QLRLLDALLS LPADTFNFVN
LPGRKIVTVD DVAGASPTIK SLAANVQGHT WNSLDLFEVL VQAADLNNPD VENTVREMLD
KAVKISAELV HMGLLQVPPW NDIRLDYTQR LLAMFLAGHP NHQLVFMRIW QIEPVYLTNA
FRDFYEESPL NITRILDILD SLLEVRPFTF ALDEYLNLDK WLADNVSAHG ADFLHAVIAF
LDLKMESEKA QRVSDPPVRT MQLSPQTITI FLRVLRNSSA IMHESDIDYC LEVRNACLQI
HPRLMDTEPG FAVVSYSAEI ETEVDGIYKQ MYDEQITIDD VIKLLQRNKT SSSPRDHEIF
SCMLHFLFDE YKFFQYPPRE LAMTGYLFGS LIQFQLVDFI PLGIAIRYVL DALGCPPETN
LFKFGLQALS RFESRLAEWQ PLCQALLKIP HLLEARPELA VIIQRAEVSD KILFIVNNLA
PSNFDAKLLE MKGQFSTEPN NHQLYLRFLE ALNLQPLSHF ILHETLVKSA ILLNSEKTMQ
LGSERAILKN VASWLDRPIK HKQLSFKDLL IEGYDNGRLI VAIPFVCKTL EPAARSKVFR
PPNPWLMAVV SLLTELYHFA ELKLNLKFEI EMLCKALDVD LDIMQATTIL RNRPRVLGAH
IENILSSVLP QVTFSPQLAL NTNPSFKRAV HMAIDRAVRE IILPVVERSV TIAGISTREL
VAKDFVTESS EDKMRRAGHL MAQKLAGSLA LVTCKEPLKG NLGSHMRHFL TEFGFTEQQV
IFLLVQDNIE LACSAIEKAA MDRAVIDVDE GFAAAYELRR RHREQRPGSP IVTSLPDPLR
IKASGVQPSQ MRVYEEFFNA LVRDLEAVLL QLPTSLAALP PNHEVRHLVR QILFLADRTR
TPLLMSQKIV QLLYKTPSQL GREIYVALLD QLCHSFEDVA KEAITWLVYA EDERKFNVPV
TVTLLRSGLV TIVQEDQQLA KDPRPSLQTF AAGLIREYLA ADSTQGQFSY SMEWFQQWVS
IYQRSHSPEK SFVPYITQLT RQGILKAEDS SFFFRVCAES SVNSYIKHVN AGEFGFAFQA
LDAMSRLIVY IIKYHGDANN DQAKVHYLTK ILSIFVLVLA NMHEEQGFQQ KPFFRFFSSL
LNDLHSIESS LGTAYFQLLL AIGDTFSSLQ PTYFPGFAFS WMSLISHRLF MPKLLLSDGR
EGWSAFYKLL LSLFKFLGPF LKSADLQAAG RDLYRGTLRL LLVLLHDFPE FLAEYYFSLC
DVIPPRCIQL RNIILSAYPP NIILPDPHLP DIDMGPIPPI LSDFAAGLRA GDLRLYLDQF
LLNRNSQTFL SSLKDRLEPY NLSLINSLVM YIGVSSVAQA RARSAGPLFV PTDPGAMALH
YLATNLDVEG QYHLLNAMVL HLRYPNAHTH WFTLLMLHLF HEIEADSFRE VMTRVLLERF
LVHRPHPWGA LVTFIELLRN PKYQFRQQDF VNVAPEVTLL LENV
>FP
ERQALITAAM AKYGPDTVVP MLRRIFPELS LPAGTTFVQA LVQLGPEITA EPAAVHALLT
RFNFTASNPP QNAQVVEWVQ SLARLASEGT VLPDVGSLVK ALDSFAVIFD WAGVIKAFDM
PDRGVDTATL KLLIAILQNT PRAKPHAVTG FWQTWLNPLY QLRLLDALLS LPADTFNFVT
LPGARIVTVD DVANASPTIK SLAANVQGHT WNSLDLFEVL VRLSDCDSPD VRNFVREMLD
KAVKISAELV HMGLLQAPNW GEIRVDYTQR LLALFLAGHP NHQLVFMRMW QIEPSYLTNA
FRDFYEESPL NITRILDILD ALLEVRPFTF ALDEYLNLDK WLADNVTAHG ADFLHAVIAF
LDIKMESEKA TRISDPAVRT MPLNPQTITI FLRVLRNSSS IMRESDVDYC LDIRNACLQI
HPRLMDAEPG ISVVSYSAEI EAEVDGIYKQ MYDEQISIDD VITLLQRHKE STNPRDHEIF
SCMLHFLFDE YKFFQYPPRE LAMTGYLFGS LIQYQLVDYI PLGIAIRYVV DALNCAPETN
LFKFGIQALT RFESRLAEWQ PLCQALLKIP HLLEARPDLA AAIHRAEVSD KILFIVNNLA
PSNFESKLSE MREHFSTEPN NHQLYLRLLD ALDMPLLLRF VLNETFVKSA SILNSEKTLN
SSSERTILKN IGSWLDRPIK HKNLSFKDLL IEGYEHGRLT VAIPFVCKTL EPCANSKVFR
PPNPWLMAVI SLLAELYHFA ELKSILKFEI ELLCKALDID LDGVQATTIL RNRPRALGAH
IENILSSLLP LVQISPQYQL HTNQTFKRAV QMAVDRAVRE IIMPVVERSV TIAGISTREL
VSKDFATEPN EERMRKAGHL MAQKLAGSLA LVTCKEPLKT NMTAHLRNFS NEFGFNESSS
VNGIVADNLE LACQAIERAA MDRAVIDVDD GFAAAYEARR RHREQRPGSA LASTLPEPLR
IKPTGVQPNQ AGVYEDFFSL VVKDLEAILL QIPPSLSSLP PNHEIRGLVR QILNLAERQR
TPLLMSQKIV QYLYKTPTQL GREIYAALLE NLCTSFEEVA KEAITWLIYA DDERKYNVPV
MATLLRTGLV PILQQDQQLA KDPRPNLLNF AAELIRECLS TDASQSQFVY TLEWFQQWVS
IFQRSHSPEK AFVPFITQLT KQNILKAEDS SFFFRVCAES SVNSYIKCIQ AGEFTYAFQA
LDAMSRLIVY IIKYHGDANN DQAKVHYLTK ILSIFVLVLA DMHEEQGFEQ KPFFRFFCSL
LNDLHSIEGS LGTAYFQLLV AISDTFSSLQ PTYFPGFSFS WMSLISHRLF MPKLLLSENR
AGWSAFYKLL VSLFKFLSPF LKAADLQPAS RDLYRGSLRL LLVLLHDFPD FLSEYYFSLC
DVIPPRCIQL RNIILSAFPP TIMLPDPHLR SFEMGPIPPI LSDFASVLKA GDLRLYLDQY
LLNRGVQSFL PSLKDRLETY NLSLINALVM YIGVSSVAQA KARSGSSLFV ASDPGVVALQ
YLATELDAEG QHHLLGAMVL HLRYPNAHTH WFSSLMLHLF VEVKSNQFRE ILTKVLLERF
LVHRPHPWGA LVTFIELLRT SKYDFWNQEF IRVAPEVTLL LENV

>PP
QRQALIAAAQ NKYGSEIVAP IVQRLVPSLS LLPDATIGQS LADLGLDMSN DPEVVLAMLA
RFGVSATSPP TNEQVVDVVQ GLALMAAEGR MLPDVGTLIR VLASFDSNIQ WASIIKAFDM
PDRGVDTATL KLLIAILVNA PRDEDHAVTG FWQLWNNPLY QLRLLDALLS LPADTFNFVS
LPGHRIVTVD DVANASPTIK SLAANVQGHT WNSLDLFEVL VRLSESDSID VRNFVREMLD
KAVKISAELV HMGLLEVPPW SEIRLDYTQR LLSMFLAGHP NHQLVFMRIW QIEPSYLTNA
FRGFYEESPL NITRILDILD ALLEVRPFTF ALDEYLNLDK WLADNVTAHG ADFLHSVIAF
LDLKMESEKA TRISDPVVRT MPLNPQTITI FLRVLRNSSS MMHESDVDYC LEVRNACLQI
HPRLMDVEPG FTVVSYSAEI EAEVDGIYKQ MYDEHISIED VIALLQRHKT STNPKDHEVF
SCMLHFLFDE YKFFQYPPRE LAMTGFLFGS LIQHQLVDYI PLGIAIRYVL DALNCAPETN
LFKFGIQALS RFESRLSEWQ PLCQALLKIP HLLEARHDLA ASIQRAEVTD KILFIVNNLA
PSNFEAKLAD MREQFSTEPN NHQLYLRFLD ALDVQPLNRY VLHETLVKAA ALLNADKTMQ
LTSERVILKN MASWLDRPVK HKNLSFKDLL LEGYENGRLL VAVPFVCKTL EPCAQSKVFR
PPNPWLMAVI SLLAEMYHFA DMKSILKFEV ELLCKALDID LDAVQLTATL RNRPRVLGAH
IENILSSILP RVTINPQLSL HTNQTFKRAV QMAVDRAVRE IIVPVVERSV TIAGISTREL
VAKDFATEPN EEKMRQAGHL MAQKLAGSLA LVTCKDPLKS NLGGHIRALL VDCGFSEVGG
IMVLANDNLD LACQAIEKAA MDRAVIDVDE GFAAAYETRR RHREQRPTTA ITSTFPDPLR
IKASGVQPIQ AAVYEDFFNI MVKDLEALLL QLPQSLAALP PNHDVRQLVR QVLFLADRHR
TPLLMSQKIV QLLYKTPSQL GREIYVALLD QLCHSFEDVA KEAITWLIYA EDDRKLNVPV
TVTLLRSGLI NVVQEDQQLA KDPRPSLLNF AAELIRECLT ADASQNQFTH TLAWFQQWVT
IFQRSHSPEK AFVPFITQLT KQGILKVEDS SFFFRVCAEA SVESYITCIN AGEYGYAFQA
LDAMSRLIVY IIKYHGDANN DQAKVHYLTK ILSIFVLVLA NLHEEQGFQQ KPFFRFFCSL
LNDLHTIEAN LGTAYFQLLL AIGDTFSSLQ PTYFPGFAFS WMSLISHRLF MPKLLLSENR
EGWSAFYKLL VSLFKFLSPF LKAADLEPAS RDLYRGSLRL LLVLLHDFPD FLSEYYFTLC
DVIPPGCIQL RNIILSAFPP TTLLPDPHLR NVDMGPIPPI LSDFTSGLKA GDLRAYLDQY
LLNRGSPSFL PSLKERLEAY NLPLVNSLVM YVGVSSVAQA KARSGSSLFT PTDPGVIVLQ
YLATNLDMEG QHHLLGSMVL HLRYPNAHTH WFSSLMLYLF QEVKNDQFRE VMTKVLLERF
LVHRPHPWGA LVTFIELLRN PKYDFWSQEF IRVAPEVTLL LETV

<u>05-DNA-directed polymerase</u>
>RM
GQRKLYLQIC FRNVSDLLAV RKELVPLALE NGAKLSAVDA YAEVVDARDS IIDAREFDVP
YYLRVAIDNE IRVGLWYSIS FVNGQPQIDR LDRADPVVMA YDIETTKAPL KFPDQAIDQV
MMISYMIDGQ GYLITNREIV SEDIEDFEYT PKEGIEGPFT IFNEADEAAT ITRFFQHIQA
VKPTVMATFN GDFFDFPFLC ARAKVHGIDM FLETGFAKDS EDEFKSRSCV HMDCFRWVKR
DSYLPQGSQG LKAVTTAKLG YNPIELDPEL MTPYAMEQPQ VLAQYSVSDA VATYYLYMKY
VHPFIFSLCN IIPLCPDEVL RKGSGTLCET LLMVEAYRAQ VIMPNRHEDP LGNMFEGHLL
ASETYVGGHV EALEAGVFRS DINTNFKIVP AAAQLIDQLD AALTFYITNY DEVKAEIQAK
LETMRDNPIR FDKPLIYHLD VAAMYPNIML SNRLQPDSVV EEAMCAVCDF NRPGKTCDRR
MTWAWRGEYF PAQRDEFNME WFPPKKPSGP KRRYVDLSPA EQTALLHKRL GDYSRKVYKK
TKETKVVNRE SIICQRENPF YVDTVRTFRD RRYEYKGLHK TWKKKLDGIA EVDEAKKMIL
AHKCILNSFY GYVMRKGARW HSMEMAGITC LTGATIIQMA RQLVEQIGRP LELDTDGIWC
MLPGIFPENF KFKMKNGKTL ALSYPCTMLN QMVHARFTNH QYHDLDKETG HFQVHSENSI
FFELDGPYKA MILPSSKEED KLLKKRYAVF NDDGSLAELK GFEVKRRGEL QLIKIFQSQI
FEKFLLGTTT EECYAAVAEI ADRWLDVLFS KAESLSDEEL VDLIAENRSM SRTLAEYGGQ
KSTSISTAKR LAEFLGDQMV KDKGLACKFI ISQKPIGAPV TERAVPVAIF SAEESVKRTY
LRRWLKDNGL ANFDLRSILD WDYYIERLGS VIQKLITIPA AMQKVANPVS RIRHPDWLHR
RVRAMDDKFK QHKMTDFFRG RFTMWLAVNN DIVPVTIRIP REFYLNYKTP EHYTRERVVR
MLPHNRACGD ILMNKINQPN VDGAYELQTL NRARDNGVDL GQMSRKIVFL HHAYTPSAPV
HCFSIVLPDG IKLHVVDPAT RRQPTDYHSN EATALRAISR ELGLLENKAY TLVLSSTRDT
HYFTSMVPKL AKFPILRMPS SKPGHILDFP WQSALAKKLL SRYLSLSAWL HRTIAQASHY
PLFFSDIDFA RRLVAQDMVL WWSPGEHPDL GGYEDDALPS DELVNPEFTS PGVYSRVCLS
IQVRNLAVNS VLQSSIVNEL EGCGGTTGFD TAQATATLSD SNLPPQTFMV LKHMVRSWLT
KNAGPAGLTI DHFWRWISSS SSHMFDSCIQ RFVHGLMRKT FIQLLAEFKR LGSNLVYADF

TRLVLVTSKP PGTANAYATY LLTAVTSHEL FKHVYLRTDR FYDFLSFMDP ANEGGVLCED
PLAIEPPKQL SVFLTWNIKK FLPPAVQDHF KAVIKYFIVE MHKISRARVK EIDAIKGFIS
QRLTRKMLQT VFPILPGSYL NLTDPALEFV KFTCAIFGLA HDYQIEIGLL KRNLLELVGV
KEFSEVASFH NPCDPLKLSM VTCQYCDYLR DFDFCRDDDL FPWTCPECLC EYDRTAIEFS
LIQMLHRLER GFSQQDLKCS KCKQIRSDNV SKHCCSGSYE LTIGKVEVRR KLRTIVNVAI
VHKLNRL
>SN
GHRRLYLQLC FRNVSDLLTV RRDLLPLAQA NAAKIDAVDA YAEVVDPRDA IIDIREYDVP
YYLRVAIDQD IRAGLWYAVT FVDGKPLFDR VKRAEPVVMA YDIETTKAPL KFPDQAIDQV
MMISYMIDGQ GFLITNRDIV SEDIEDFEYT PKDGYEGPFT IFNEADEEAV LRRWFEHIRT
ARPTVMATFN GDFFDFPFVA ARAKVHGLDL YLETGFAIDS EEEFRSRTCV HMDCFRWVKR
DSYLPQGSQG LKAVTVAKLG YNPIELDPEL MTPYAVEQPQ VLAQYSVSDA VATYYLYMKY
VNPFIFSLCN IIPLNPDEVL RKGSGTLCET LLMVEAYRGN IIMPNRHEEE HGNMFEGHLI
ASETYVGGHV EALEAGVFRS DIPTHFKIDK TAAQLIDQLD AALKFCVANY DEVKQQITEA
LEEMRDNNIR SDKPLIYHLD VAAMYPNIML SNRLQPDSVV DESVCAVCDY NRPGKQCDRR
MKWAWRGEYF PAHRDEFNME QFPPKRPNDP KRRFHDLSPA EQSALLHKRL GDYSRKVYKK
TKETKVIERE SIICQKENPF YIDTVRRFRD RRYEYKGLLK TWKKNLEPIA EIEEAKKLTL
AHKCILNSFY GYVMRKGARW HSMEMAGITC LTGATIIQMA RQLVERIGRP LELDTDGIWC
MLPSIFPENF KFKLKSGKSI AFSYPCTMLN HLVYDGFTNH QYHDLDPDTG EYKIHSENSI
FFELDGPYKA MILPSSKEED KLLKKRYAVF NDDGSLAELK GFEVKRRGEL QLIKIFQSQL
FERFLLGTTT EECYAAVAEV ADRWLDVLFT KGESLEDDEL VELIAENRSM SKTLAEYGAQ
KSTSISTAKR LAEFLGNQMV KDKGLACKFI ISARPMGAPV TERAIPVAIF SAEESVKRTY
LRKWLKDNSL ANFELRSILD WNYYIERLGS VVQKLITIPA AMQKVSNPVP RIHHPDWLQR
RVAAMDDKFQ QHKMTDFFHG KFIMWLSLAN ELVAIPLRIQ REFYINFKTS PDYTHEAVTR
TLPQNRPCLF LFMHLTSNSD IDGIYERQTL NRARNVGFDL QQLETEPVFL FHASSSDSRI
HVFALFDPVG ARIHIIDPSS QRQRTVYHGS LSTAFKGLTR ELNNLENQSH VLVISSHKDV
DYYEANISNL IKFPVFMMAG PKKAHSLDLL WQKDVSKKMF SRYIFLGRWL YELTEKAKYF
SLSCQDLDFA RRLVKQDMLL WWSPLPKPDL GGVEDLNTVV EEPKNPEYCT PGLYTNVSIA
VNMYNLAINS VIQSSLVNEM EGSGGTTAFD APQTALTLGE SATSVQVFQT LKTMVKTWVI
DAHGPAELSV EHFWRWLCSS ASAMHDPSLQ RFVHGLMKKT FYQLLAEFKR LGSNVIYADF
NTIWLVTSKP PGAAYAYSTY ITTAATSHDL FRHILLRTET YYNFLLFLDN ANYGAIVCEN
PEEDKLPEGL PLAMSWNIAA FLPPAFQPHF RQAVAFFIVE MAMIKTRKIK ELEVSQAFIA
ERLTRKLLSM VFPILPGSHI NMEDPAFEFV KFVCAAFSPS KDYGNELGIL RRNLLEIVGV
KEFNDAAIFK NPCEPFKLPM VICQFCDHVR DFDFCRDQEL MTWLCPMCDG EYDRLAIEFM
LVSCVDKIIE LFGAQDLRCH RCKQSRAENV SRSCCSGHYE LTVNKVELRR RLRTIVNISI
VHGLDKL
>DS
GHRRLFLQLH FRNVSDLLTV RCDIMPLALA NSAKLDAVDA YAEVVDPREG IVDIREYDVP
YYLRVAMDNE IRVGLWYAIS FVQGQPAFAR VKRADPVVMA FDIETTKAPL KFPDQAIDQV
MMISYMVDGQ GYLITNREIV SEDIEDFEYT PKEGYEGPFI VFNEPDEAAT IRRWFSHIQE
VKPTVMATFN GDFFDFPFLD ARSKVHGIDM FLESGFAKDS EDEYKSRGCV HMDCFRWVKR
DSYLPQGSQG LKAVTTAKLG YDPIELDPEL MTPYAMEQPQ VLAQYSVSDA VATYYLYMKY
VHPFIFSLCN IIPLCPDEVL RKGTGTLCET LLMVEAIRGQ IIMPNRHEES HGTMYEGHLV
ASETYVGGHV EALEAGVFRS DIVTHFKIEP SAVQLIDQLD AALTFCVTNY DEVKAEIQAK
LEEMRDNPQR MDSPLIYHLD VAAMYPNIML SNRLQPDSVV DEATCAVCDF NRPGKTCDRR
MTWAWRGEYF PARRDEYNME TFPPKKPGGP QRKFVDLSEA EQTALLHKRL GDYSRRVYKK
IKDTKIENRE AIICQRENPF YIDTVRRFRD RRYEYKGLHK TWKKNLDSIA EVDEAKKMIL
AHKCILNSFY GYVMRKGARW HSMEMAGITC LTGATIIQMA RQLVEQIGRP LELDTDGIWC
MLPGVFPENF KFKLANGKAI AFSYPCTMLN HLVHAKFTNH QYHELDPETG EYKVHSENSI
FFELDGPYKA MILPSSKEED KLLKKRYAVF NDDGSLAELK GFEVKRRGEL QLIKIFQSQI
FEKFLLGTTL EECYAAVAEV ADRWLNVLVS HAEDLSDDEL VELIAENRSM SKTLSEYGGQ
KSTSISTARR LAEFLGDQMV KDKGLACKFI ISAKPMGAPV TERAVPVAIF SAEESVKRTY
LRKWLKDNSL TSFDLRSILD WDYYIERLGS VIQKLITIPA AMQKVANPVP RIHHPDWLHR
RVVAMEDKFR QHKVTDFFPG RFTLWLSING SLTAVPLRIP REFYIHLRTP ELYQCQKVAR

SMPRDRPCVN LFIDITNDPN VDGVYELQNL TRAEAIGFDL QQLGRKYIFL YHACAANAPV
HVFALFIPGE AKLHIVDPAT RRQPTTYHGN DATALKAISR ELGLVENRSY IVVISSSKEQ
SYFDLHTPKL AKFPVLSMSK AKSPHSLDFP WQTSVAQKLI NRYLSLGPWL DRTIAMADYY
PLLLADIEFA RRLTSQDVIL WWSPGDRPDL GGIEEDNRPQ EELAKTEFKS PGCYSNVCLE
IAVRNLAVDA VLHSVIVNEL EGSGGATAFD ASMQDLTLGE SNVSPHIFSI LKGLVKAWMV
DKISPATIAI DHFWRWISST ASNMYDTGIH RFVHGLMRKT FIQMLAEFKR LGSHVVYADL
SRILLVTSKP PGTAHAYATY ITTAVTSHEL FQHVYLHTER FYDFLIFMDE ANYGAVVCEN
PLAIEPPEEL AIEMRWNIQS FLPPAIQADF RAVVRYFLVE FFRTAQKKVE ELSACREFVS
RKLTRRMLRV LFPILPGSHL PLSNPGLELV KFTCAVFALA KEYTLEVGLL KRNLLDLVGV
REFASEAAFV NPCEPLKLSA VPCRHCDSMK DFDFCRDPVL LPWLCTKCGG EYDRAGIEFA
LVRLVHELER RFAQQDLRCA RCSQVRADNV SRHCCSGAFV LTMNKADVRR RLRTVVNVAL
AHGMGRL
>TV
GHTRLYLQLH FRNVSDLLTV RRDIMPLALA NSAKLDAVDA YAEVVDPREG IIDIREYDVP
YYLRVAMDND IRVGLWYAVS FTAGQPGFER VKRADPVVMA YDIETTKAPL KFPDQAIDQV
MMISYMIDGQ GFLITNREIV SEDIDDFEYT PKEGYEGPFT VFNEPDEAAV IRRWFSHIQD
VKPTVMATFN GDFFDFPFVA ARAKVHGIDM FLEIGFAIDS EDEYKCRSCV HMDCFRWVKR
DSYLPQGSQG LKAVTTAKLG YNPIELDPEL MTPYAIEHPQ ILAQYSVSDA VATYYLYMKY
LHPFIFSLCN IIPLCPDEVL RKGTGTLCET LLMVEAFRGQ IIMPNRHEES HGNMYDGHLL
ASETYVGGHV EALEAGVFRS DIETHFKIEP SAVQLIDDLD AALTFCVTNY DEVKAAVQAK
LEVMRDNPQR MDQPLIYHLD VAAMYPNIML SNRLQPDSVV NEATCAVCDF NRPGKECDRK
LQWAWRGEFF PARRDEFNME SFPPKKPGGP QRKFNDLPQA EQTALLHKRL GDYSRRVYKK
IKDTKIETRE AIICQRENPF YIDTVRRFRD RRYEYKGLHK TWKKNLDSIA EVDEAKKMIL
AHKCILNSFY GYVMRKGARW HSMEMAGITC LTGAKIIQMA RQLVEQIGRP LELDTDGIWC
MLPGVFPENF KFKLANGKAL AFSYPCTMLN HLVHAEFTNH QYHELDPEAG EYMVHSENSI
FFELDGPYKA MILPSSKEED KLLKKRYAVF NDDGSLAELK GFEVKRRGEL QLIKIFQSQI
FEKFLLGSTL DECYAAVAQV ADRWLDVLFS HADDLSDEEL VELIAENRSM SKTLAEYGGQ
KSTSISTARR LAEFLGDQMV KDKGLACKFI ISSKPMGAPV TERAVPVAIF SAEESVKRTY
LRKWLKDNSL TNFELRSILD WDYYIERLGS VIQKLITIPA AMQKVTNPVP RIRHPDWLFR
RVAALDDKFH QHKVTDFFPG RFTLWLSVDG TLSAVPLRIP REFYMHLRTP EMYQCEKVVR
SLPRDRQCVN LFIDITNDPN VDGVYEFQTL NRAETTGFDL QQLSRKYIFL YHACSNNAPV
HVFALFIPGE VKLHIVDPAT RRQPSTYHGN DVTALKAISR ELGLVENRSF IVVVSSSKEQ
SYFDLHTPKL SKFPVLSMNK TKSAHSLDFP WQTSVAQKLV SRYLSLGKWL DTAMAQADYY
PLLLADVEFA RRLMSQDVVL WWSPGDRPDL GGIEEDNRPQ EELAKTEFTS PGCYSNVCLE
ITVRNLAVDA VLHSVIVNEL EGSGGATAFD AAMQDLTLGE SNVSPLAFSI LKSLVKTWML
DKISPATTVL DHFWRWISST ASHMYDTSIH RFIHGLMRKT FIQMLAEFKR LGSHVVYADL
SRILLVTSKP PGTAHAYATY ITTAVTSHEL FQHVYLHTER FYDFLIFMDE ANYGAVVCEN
PLAIEPPEEL AIEMRWNIQS FLPPAIQSDF RALVRYFLVE FFRTSQKKAN EISACREFVQ
RKLIRRTLRM LFPVLPGSHL HLTNPGLEFA KFACAVFALA KEHTLEVGLL KRSLLELVGV
REFAPEASFQ NPCEPLKLAS VPCQHCDAMK DFDFCRDPAL LPWLCARCDC EYDRTAIEFA
LVRMVGDAER RFAQQDLRCS RCAQLRSDNV AKHCCSGNYQ LTMNKAEVKR RLRTIVNVAL
AHNMGRL
>GT
GHRRLFLQLH FRNVSDLLSV RRDIVPLALA NGAKRDAVDA YAEVVDPREA IIDVREYDVP
YYLRVAIDNE IRVGLWYAVT FTAGQPSFER VKRADPVVMA YDIETTKAPL KFPDQAIDQV
MMISYMVDGQ GYLITNREIV SEDIEDFEYT PKEGYEGPFT IFNEPDEAAA ITRFFQHIQE
VKPTVMATFN GDFFDFPFLC ARSKVHGIDM FLETGFAIDS EDEFKSKTCV HMDCFRWVKR
DSYLPQGSQG LKAVTTAKLG YDPIELDPEL MTPYAMEQPQ VLAQYSVSDA VATYYLYMKY
VHPFIFSLCN IIPLKPDEVL RKGSGTLCET LLMVEAYRGH IIMPNRHEEA QGNMYEGHLL
ASETYVGGHV EALEAGVFRS DIATHFKIEP SAAQLIDELD AALMFCVTNY DEVKQQIQSA
LEEMRDNPLR HDKPLIYHLD VAAMYPNIML SNRLQPDSVV DESVCAVCDY NRPGKQCDRR
MTWAWRGEFF PAHRDEYNME TFLSKRPGGP HRRFTDLSES EQTALIHKRL GDYSRKVYKK
MKDTKVENRE SIICQRENPF YIDTVRTFRD RRYEYKGLHK TWKKNLDTIA EVEEAKKMIL
AHKCILNSFY GYVMRKGARW HSMEMAGITC LTGATIIQMA RQLVEQLGRP LELDTDGIWC

MLPGIFPENF KFKLKNGKAI AFSYPCTMLN HLVHDKFTNH QYHDLDPVTG QYTIHSENSI
FFELDGPYKA MILPSSKEED KLLKKRYAVF NDDGSLAELK GFEVKRRGEL QLIKIFQSQI
FEKFLLGSTT EECYAAVAEV ADRWLDVLFS RAENMPDNEL FDLIAENRSM SKTLAEYGGQ
KSTSISTAKR LAEFLGDQMV KDKGLACKFI ISARPMGAPV TERAIPVAIF SAEESVKRTY
LRKWLKDNSL ANFDIRSILD WEYYIERLGS VIQKLITIPA AMQKVANPVP RIHHPDWLHR
RVVAMDDKFR QHKVTDFFPG RYTLWLSIDS ELVAIPLRVP REFYIHFRSP DQYFCEQVVK
HLPRGQPCVN LFVDLTIDPN VDSVFELQTL NRAESAGVDL HQLTHKYIFV YHACSNNAPV
HVFAVFHPDG VKLHIVDPAT RRQGTTYHSS DVTALKAVSR ELGLLEGRSY TVVISSMKEQ
IYFDSHVPRL AKFPVLSMSK AKGPHSLDFP WQANVAQKML SRYLSLGPWL DRTISLADYY
PLYVADLEFA RRLLKEDMVL WWSPGDRPDL GGIEEDKRQT QEIASTEFLS PGCYVNVCLE
IAVRNLAVNS VLHSVVVNEL EGSGGATAFD SAPRDLTLGE SNVSPKTLNV LKSMIKTWLL
DKISPATLAI DHFWRWISSN ASNMYDGSIH RFVHSLMRKT FIQLLAEFKR LGSHVVYADF
SRILLVTSKP PGTAHAYATY ITTAVTSHEL FQHVYLNTER FYDFLVFMDQ ANLGGVVCED
PLAIEPPEEL AIEMRWNIET FLPKAIQEDF RSIVRFFVIN LYRTQQQKVK EMEATREFIA
RRLTRRCLKT VFPVLPGAHL AMTNPPLEFV KFVCAVFHLA KDYQVEMGLL RRNLLELVGV
REFADDAVFR NPCEPLRLTN VPCRHCDALR DFDFCRDTEL LPWACGECGG DFDRTAIELA
LIDIVYTLER TFTQQDLRCS KCKQIQSDNV SRHCCSGAYQ LTISKADVRR KLKTIVNVAM
VHNLGRL

>PS

NPGRLFLQLC FRNVSDLLTV RREIVPLALA NGAKRDAVDA YAEVVDPREG IIDAREYDVP
YYLRVAIDND LRVGLWYAVT LTAGQPSFER VKRADPVVMA YDIETTKAPL KFPDQATDQV
MMISYMIDGQ GYLITNREIV SEDIEDFEYT PREGYEGPFT VFNEPDEPST IMRWFRHVQE
VKPTVMATFN GDFFDFPFLC ARAKVHGIDM FQEIGFAIDN EEEFKSRSCV HMDCFRWVKR
DSYLPQGSQG LKAVTTAKLG YNPIELDPEL MTPYALEQPQ VLAQYSVSDA VATYYLYMKY
VHPFIFSLCN IIPLNPDEVL RKGSGTLCET LLMVEAYRGH IIMPNRHEEE HGNMYEGHLL
ASETYVGGHV EALEAGVFRS DIPTHFKIDP TAAQLIDQLD AALEFCVTNY DEVKGQIQAK
LEEMRDNPLR MDKPLIYHLD VAAMYPNIML SNRLQPDSIV DEATCAVCDY NRPGKTCDRK
LTWAWRGEFF PAHRDEFNME SFPPKKPGWP QRRFTDLSEA EQTALMHKRL GDYSRKVYKK
TKDTKVEERE SIICQRENPF YVDTVRTFRD RRYEYKVLHK TWKKNLDGIA EVDEAKKMIL
AHKCILNSFY GYVMRKGARW HSMEMAGITC LTGAKIIQMA RQLVERIGRP LELDTDGIWC
MLPGTFPEDF KFKLKSGKPI GFSYPCTMLN HLVHAQFTNH QYHDLDPETG EYKVHSENSI
FFELDGPYKA MILPSSKEED KLLKKRYAVF NDDGSLAELK GFEVKRRGEL QLIKIFQSQI
FEKFLLGTTT QECYAAVAQI ADRWLDVLYS RADDLSDEEL VELIAENRSM SKTLAEYAGQ
KSTSISTAKR LAEFLGDQMV KDKGLACKFI ISQKPMGAPV TERAVPVAIF SAEESVKRTY
LRKWLKDNSL ANFELRTILD WDYYIERLGS VIQKLITIPA AMQKVPNPVP RIRHPDWLHR
RVMAIDDKFH QRKVTDFFPG RFMLWLNVDE RLVPIPLRIP REFYIHTRTP ELWTAEKVVR
GLPRGAPCVN LFIDLTNDPN VDGVYELQTL NRAQGTGLDL YQLERKYVFL YHAMAATAPV
HVFALFFPDG VRLHVVDPAT RRQPTTYHGN DVTALKAISR ELGLMENRSF TVVISSLKDQ
AYFDANVPRL ANFPVWTMPQ AKSAHSLDFP WQSSVAQKMV IRYLTVASWI DASVALATYY
PLLLADIDFA RRLTKEDLVL WWSPGDLPDL GGIQDDKRPS EPLPGTEFLS PGCYFNVCLE
IEVRNLAVNA ILHSVIVNEL EGSGGATAFD SAQRDLTLGE SNVSPKTFGI LKAMVKTWLL
DKISPSSLTI DHFWRWISSS VSHLYDPSIH RFVHGLMRKT FIQLLAEYKR LGSHVVYADL
SRILLVTSKP PGTADAYATY INTAVQSNEL FQHIWLKTER FYDFLLFMDP ANLGGVVCEN
PKAVEPPEEI VIEMQWNIQN FLPPAIQKDF ASIVRYFMIE LYRSRQKKVK EMELTREFIA
RRLTRKLLRM VFPVLPGSHL RLYDPVLEFV KFVCAVLALA KEYQIEVGLL RRSVLELTGV
REFSNDAVFR QPCEPLKLSN VPCKHCDSLR DFDFCRDPEL LPWLCGNCGG EYDRTAIEFA
LIDLLFGLER TFAQQDLRCT KCKQIQSDNV SRHCCSGSYQ LTINKADVRR KLRTMVNVAI
LHNLHRL

>PC

GHRRLYLQLH FRNVSDLLTV RRDVMPLALA NSAKLDAVDA YAEVVDPREG IIDIREFDVP
YYLRVAMDNE IRVGLWYAIT FETGQPVFER VKRADPVVMA YDIETTKAPL KFPDQAIDQV
MMISYMVDGQ GFLITNREIV SEDIEDFEYT PKEGYEGPFT VFNEADEPSA IRRFFSHIQE
VKPTVMATFN GDFFDFPFLC ARAKIHGIDM FLETGFAIDP EEEFKSRTCV HMDCFRWVKR
DSYLPQGSQG LKAVTTAKLG YNPIELDPEL MTPYAMEQPQ VLAQYSVSDA VATYYLYMKY

VHPFIFSLCN IIPLNADEVL RKGSGTLCET LLMVEAFRGN IIMPNKHEEA HGKMYEGHLV
ASETYVGGHV EALEAGVFRS DIATQFKIEP TAVQLIDQLD AALTFCVTNY DEVKAQIQAK
LEEMRDNPLR MDNPLIYHLD VAAMYPNIML SNRLQPDSVV DESVCAVCDF NRPGKCCDRR
MTWAWRGEFF PARRDEFNME TFPPKKPGQP QRKFADLSPA EQTALLHKRL GDYSRKVYKK
VKDTRVENRE AIICQRENPF YIDTVRRFRD RRYEYKGLHK TWKKNLDSIA EVDEAKKMIL
AHKCILNSFY GYVMRKGARW HSMEMAGITC LTGATIIQMA RQLVEQIGRP LELDTDGIWC
MLPGIFPENF KFKLANGKAI AFSYPCTMLN HLVHAQFTNH QYHDLDPETG EYKIHSENSI
FFELDGPYKA MILPSSKEED KLLKKRYAVF NDDGSLAELK GFEVKRRGEL QLIKIFQSQI
FEKFLLGTTT EECYAAVAQV ADRWLDVLFS KADSLSDEEL VELIAENRSM SKTLAEYAGQ
KSTSISTAKR LAEFLGDQMV KDKGLACKFI ISAKPLGAPV TERAVPVAIF SAEESVKRTY
LRKWLKDNSL TNFDLRSILD WDYYIERLGS VIQKLITIPA AMQKVANPVP RIRHPDWLHR
RVVAQGDKFR QHKMTEYFPG KFTLWLSTDT GLVSVPLRIP REFYVHLRLT DMYSVEKVVR
TLPRDQPCVN LFIHLTNDPS VDGVYEQQTL NRAEVTGFDL NQLDRKYALL FHAMSSSANV
HVFALFLPSG VRLHIVDPAT RRQATTYHAN DLTALKALSR ELGLLENQGL TMVISSLKEQ
TYFDSYVPKL AKFPVLSMSK AKALHSLDLP WQPAAAQKMI RRYLTLGAWL DRTIALADYY
PLLLADIEFA RRLTAQDILL WWSPTDRPDL GGIEDDRRPT EELPKTEFMS PGCYSNVCLE
VTVRNLAVDA VLHSMIVNEL EGSGGATAFD ATQKDLTLGE SSVSPVTFSI LRSMVKTWLL
DKISPASLAI DHFWRWISSN ASNLYEPSLH RFVHGLMRKT FIQMLAEFKR LGSHVVYADL
SRILLVTSKP PGTAHAYATY ITTAVTSHEL FQHVYLHTER FYDFLIFMDP ANLAGIVCED
PLAVDPPEEL CLEMRWNIQS FLPPAIQEDF KKAIRNFLVE FFKVKQKKTK EQEDSKEFIQ
RKLLRRMLKM VFPVLPGSHL HMTNPVLEFV KFTCAVFALS KEYQIEVGLL KRNLLELVGV
REFSPEAAFQ NPCEPLKLSN VPCRHCDSLR DFDFCRDPEL LEWTCANCYG EYDRTMIEFA
LIEMVWELER RFAQQDLRCT KCKQIQSDNV SRFCCSGNYQ LTISKADVRR RLRTMVNVAL
VHKFPRL
>FP
GHRRLYLQLH FRNVSDLLTV RRDLMPLALA NSAKLDAVDA YAEVVDPREG IIDIREYDVP
YYLRAAMDNE IRVGLWYAVT FTAGQPAFER VKRADPVVMA YDIETTKAPL KFPDQQIDQV
MMISYMIDGQ GFLITNREIV SEDIEDFEYT PKEGYEGPFT VFNEADEAAT IQRFFSHIQD
VKPTVMATFN GDFFDFPFLC SRAKIHGIDM FLETGFTKDS EDEFKSRGCV HMDCFRWVKR
DSYLPQGSQG LKAVTTAKLG YDPIELDPEL MTPYAMEQPQ TLAQYSVSDA VATYYLYMKY
VHPFIFSLCN IIPLCPDEVL RKGTGTLCET LLMVEAFRGK IIMPNRHEDE HGNMYEGHLL
ASETYVGGHV EALEAGVFRS DIPTHFKIEP SAAQLIDELD AALTFYVTNY DEIKGQIQAA
LEEMRDNPLR MDKPLIYHLD VAAMYPNIML SNRLQPDSVV DEAVCAVCDF NRPGKACDRR
MTWAWRGEFF PARRDEFNME TFPPKKPGGP QRKFIDLAQA EQTALLHKRL GDYSRKVYKK
IKDTRVENRE AIICQRENPF YIDTVRRFRD RRYEYKGLHK TWKKNLDSIA EVDEAKKMIL
AHKCILNSFY GYVMRKGARW HSMEMAGITC LTGATIIQMA RQLVEQIGRP LELDTDGIWC
MLPGIFPENF KFQLANGKAL SISYPCTMLN HLVHAQFTNH QYHDLEAETG DYKVHSENSI
FFELDGPYKA MILPSSKEED KLLKKRYAVF NDDGSLAELK GFEVKRRGEL QLIKIFQSQI
FERFLLGETT EECYSAVAQI ADRWLDVLFS KAENLPTQEL VELIAENRSM SKTLAEYAGQ
KSTSISTAKR LAEFLGDQMV KDKGLACKFI ISARPQGAPV TERAVPIAIF SAEDSVKQTY
LRRWLKDNSL TTFDIRSILD WEYYIERLGS VIQKLITIPA AMQKVSNPVP RIKHPDWLHR
RVVALDDKFH QHKVTDFFPG HFAMWLSVGG DLFSVPLRIP REFYIHVRSP ELYTYEKVMR
GLPRDRPCVN LFIDLTNDPS VDGVFEMQTL NRAEAIGFEL AELDRKYIFL YHACSSSAPV
HVFAIFSPGT VRLHIVDPAT RRQPSTYHGT DVAALKAISR ELGTMENQSY TVVISSSKEQ
PYFDAHVPKL GKFPVLSMPK TKVAHTLDFP WQTNVAQKMV NRYLAMGTWL DRTIALASYY
PLLLADVDFA RRLMEHDFVL WWSPGNRPDL GGIEEDARPT EELPKTDFMS PGCYSNVCLE
ITVRNLAVNS VLHSLMVNEL EGAGGATAFD STQRDLTLGD SNVSPQMFSV LKNMVKTWLL
DKISPATVAI DHFWRWISSK AACMYDPSLH RFIHGLMRKT FIQMLAEFKR LGSHVVYADL
SRVLLVTSKP PGTAHAYATY ITTAVTSHEL FQHVYLRTER FYDFLVFMDQ ANLGGVVCED
PLAIDPPEEI CIEMRWNIET FLPPAIQRDF RNVVRFFLVE LFRTCQKKTQ EVEASREFIS
RKLTRRLLKA VFPVLPGSHL TMTNPILEFV KFSCTVFTLA KDYQIEVDLL KRNLLELVGV
REFASEATFH NPCEPLILSN VPCRHCDALR DFDFCRDPEL LPWLCGNCGG EYDRTMVEFA
LMDMVWDLER RFAQQDLRCA KCKRIRSDNL SRHCCSGSYQ LTVNKADVRR KLRTIVNVSV
AHNLNRL

>PP
GHRRLYLQLH FRNVSDLLAV RRDIMPLALA NSAKLDAVDA YAEVVDPREG IIDIREFDVP
YYLRVAMDND IRVGLWYAVT FTAGQPSFER VKRADPVVMA YDIETTKAPL KFPDQAIDQV
MMISYMVDGQ GFLITNRDIV SEDIEDFEYT PKEGYEGPFT IFNEPDEAAT IRRFFSHVQD
VKPTVMATFN GDFFDFPFLC ARAKVHGIDM FLEIGFAKDS EDEFKSRTCV HMDCFRWVKR
DSYLPQGSQG LKAVTTAKLG YNPIELDPEL MTPYAMEQPQ TLAQYSVSDA VATYYLYMKY
VHPFIFSLCN IIPLNPDEVL RKGSGTLCET LLMVEAYRGK IIMPNRHEDE YGNMYEGHLL
ASETYVGGHV EALEAGVFRS DIPTHFKIEP AAVQLIDELD AALTFCVTNY DEVKAQIQVA
LEEMRDNPLR MDEPLIYHLD VAAMYPNIML SNRLQPDSVV DESVCAVCDY NRPGKACDRR
MTWAWRGEFF PARRDEFNME TFPPKRPGQP QRKFVDLSDA EQTALLHKRL GDYSRKVYKK
IKDTKVENRE AIICQRENPF YIDTVRRFRD RRYEYKGLHK TWKKNLDSIA EVDEAKKMIL
AHKCILNSFY GYVMRKGARW HSMEMAGITC LTGATIIQMA RQLVEQIGRP LELDTDGIWC
MLPGIFPENF KFKLANGKAI SMSYPCTMLN HLVHAQFTNH QYHDLDPETG EYKVHSENSI
FFELDGPYKA MILPSSKEED KLLKKRYAVF NDDGSLAELK GFEVKRRGEL QLIKIFQSQI
FERFLLGNTT EECYSAVAQV ADRWLDVLFS KAENLGDDEL VELIAENRSM SKTLAEYAGQ
KSTSISTAKR LSEFLGAQMV KDKGLACKFI ISERPMGAPV TERAVPVAIF SAEENVKRTY
LRKWLKDNSL TAFDIRSILD WNYYIERLGS VIQKLITIPA AMQKVANPVP RIRHPDWLHR
RVAALEDKFR QHKVTDFFAG RFVLWLAVGG DLISIPLRIP REFYLHLRTP DMYSSEKVTR
GLPRERPCVN LFIDLTNDPN VDGVYELQSL NRAETIGFDL DQLGRKYIFL YHACASNAPV
HVFALFIPGN VKLHIVDPAT RRQPSNYHGN DVAALKAISR ELGLVETQSY MIVISSMKEQ
TYFDAYVPRL SKFPVISMSK AKAPHTLDFP WHTSVAQKMV NRYLSMGTWL DRTIALADYY
PLLLSDIEFA RRLTEHDLVL WWSPGDKPDL GGAEDDCRPS EELPKTEFMS SGCYSNVCLE
ITVRNLAVNA VLHSVIVNEL EGSGGATAFD SSQRDLTLGD SSVSPQTFSI LKSMVKTWLL
DKISPATIAI DHFWRWISSN ASHMYDPSIH RFVHGLMRKT FIQMLAEFKR LGSHVVYADL
TRVLLVTSKP PGTAHAYATY ITTAVTSHEL FQHVYLHTER FYDFLLFMDQ ANLSGVVCED
PLAVNPPEEL TIEMRWNIET FLPPAIQRDF RTVIRFFLVE LFRTLEKKMR ELEASREFIT
RRLTRKMLKA VFPLLPGSYL TLTNPILEFV KFTSAVFSLA KDYQIEVGLL KRNLLELIGV
REFASEAAFR NPCEPLRLTN VPCRHCDGLR DFDFCRDPEL LPWLCVACGG EYDRTMVEFA
LLGMVWDLER RWAQQDLRCG KCKQLQSDNV SRHCCSGSYQ LSMNKADVRR KLRTIVNVSI
AHNLTRL
>WC
GHRRLYLQLH FRNVSDLLAV RRELMPLALA NSAKLDAVDA YAEVVDPREG IIDIREFDVP
YYLRVAMDNE IRVGLWYAVT HVAGQPQFER VKRADPVVMA YDIETTKAPL KFPDQAIDQV
MMISYMVDGQ GYLITNRDIV SEDIEDFEYT PKEGYEGPFT VFNEPDEASV IRRFFSHVQD
VKPTVMATYN GDFFDFPFLC ARAKVHGIDM FVEIGFAKDS EDEFKSRTCV HMDCFRWVKR
DSYLPQGSQG LKAVTTAKLG YNPIELDPEL MTPYAVEQPQ TLAQYSVSDA VATYYLYMKY
VHPFVFSLCN IIPLCPDEVL RKGSGTLCET LLMVEAYRGK IIMPNRHEEE HGKMYEGHLL
ASETYVGGHV EALEAGVFRS DISTHFKIEP SAVQLIDELD AALTFCVTNY DEVKQQIQSA
LEEMRDNPHR MDKPLIYHLD VAAMYPNIML SNRLQPDSVV DEAVCAVCDY NRPGKTCDRR
MTWAWRGEFF PARRDEFNME TFPPKRPGGE QRKFVDLTDA EQTALLHKRL GDYSRKVYKK
IKDTKVENRE AVICQRENPF YIDTVRRFRD RRYEYKGLHK TWKKNLDPIT EVDEAKKMIL
AHKCILNSFY GYVMRKGARW HSMEMAGITC LTGATIIQMA RQLVEQIGRP LELDTDGIWC
MLPGIFPENF KFKLSNGKAI GFSYPCTMLN HLVHAKFTNH QYHDLNPETG EYGVHSENSI
FFELDGPYKA MILPSSKEED KLLKKRYAVF NDDGSLAELK GFEVKRRGEL QLIKIFQSQI
FEKFLLGTTT EECYAAVAQI ADRWLDVLFS KADSLEDEEL IELIAENRSM SKTLAEYGGQ
KSTSISTAKR LAEFLGDQMV KDKGLACKFI ISASPQGAPV TERAVPVAIF SAEESVKQAY
LRKWLKNNSL ADFNIRSILD WGYYIERLGS VIQKLITIPA AMQKVANPVS RIQHPDWLHR
RVVALDDKFH QHKMTDFFPG RFAMWLNVGG DLVSIPLRIP REFYVHLRAP DMYLCEKVVR
SLPRDRPCAN LFIDLTNDPN IDGVFELQTL NRAETVGFEL DQLGRKYIFL YHACSNSAPV
HVFALFIPGS VRLHIVDPAT RRQPSTYHGN DATALKAISR ELGLIENQSF MVVISSSKEQ
TYFDAYVPRL AKFPVLSMSK TKIPHTLDFP WQTNVAQKMI SRYLCLGTWL DRTIALADYY
PLLLADIDFA RRLSEQDHVL WWSPGSRPDL GGIEDDARPT EELPKTEFMS PGCYSNVCLE
ITVRNLAVNA VLHSVIVNEL EGSGGSTAFD ASQRDLTLGD SNVSPQMFSI LKGMVKTWLL
DKISPASIAI DHFWRWISSN ASCMYNPSLH RFIHGLMRKT FIQMLAEFKR LGSHVVYADL

SRVLLVTSKP PGTAHAYATY ITTAVTSHEL FQHVYLRTER FYDFLLFMDQ ANLGGVVCED
PLAVEPPEEL SIEMRWNIET FLPLAIQRDF RNTIRFFLVE FFRTCQKKTQ EMGACREFIA
RRLTRKTLKA VFPLLPGSHL HLSNPILEFV KFCCAVFGLA KEYQIDVGLL KRNLLELVGV
REFASEATFH NPCEPLRLAN VPCRHCDNLR DFDFCRDPEL MPWLCSNCGG EYDRTMIEFA
LMDMVFDLER RFAQQDLRCS KCQRLQSDNV SRHCCSGNYQ LSIIKADVRR RLRTIVNVSI
AHSLSRL
>SC
GHQRLYIQLC FRNVTDLLTV RRDIMPIALA NGAKRDAVDA YAEVVDPQDS IIDIREYDVP
YYLRVAIDID IRVGLWYGVT FEAGQPHFER VKRADPVVMA YDIETTKAPL KFPDQALDQV
MMISYMVDGQ GYLITNREIV SEDIGDFEYT PKEGYEGPFI VFNEADEAAT ISRFFQHIQE
IKPTVMATFN GDFFDFPFLD ARAKANGIDM FLEIGFTKDN EDEYKSRCCV HMDCFRWVKR
DSYLPQGSQG LKAVTTAKLG YNPIELDPEL MTPYAQEQPQ VLAQYSVSDA VATYYLYMKY
VHPFIFSLCN IIPLCPDEVL RKGTGTLCET LLMVEAYRGH IIMPNRHEEA HGNMYEGHLL
ASETYVGGHV EALEAGVFRS DIPTDFKIDP TAIQLIDQLD AALTFCVTNY EEVKGEIQRM
LEVMRDDPKR TDKPLIYHLD VAAMYPNIML SNRLQPDSMV DESVCAVCDY NRPGKTCDRR
LEWAWRGEFF PAHRDEYNME HFPPKRPGNP ERRFVDLSPA EQTALLHKRL GDYSRKVYKK
VKDTKVETRT SIVCQRENPF YVDTVRRFRD RRYEYKGLHK TWKKNLDSMA EVDEAKKMIL
AHKCILNSFY GYVMRKGARW HSMEMAGITC LTGAKIIQMA RALVERIGRP LELDTDGIWC
MLPGVFPENF KFKLDNGKNI GFSYPCTMLN HLVHDQYTNH QYHDLDPETG EYKIQSENSI
FFELDGPYKA MILPASKEED KLLKKRYAVF NDDGSLAELK GFEVKRRGEL QLIKIFQSQI
FEKFLLGTTT QECYAAVAEV ADQWLDVLFS HADSLPDEEL VELIAENRSM SKTLAEYAGQ
KSTSISTARR LAEFLGDQMV KDKGLACKFI ISQRPIGAPV TERAVPIAIF SAEEDVKRTY
LRKWLKDNSL VNFDLRSILD WDYYIERLGS VIQKLITIPA GMQKIPNPVP RIRHPDWLHR
RVANAVDKFK QNKVTDFFPG RYMLWLSVEA DLVSVAVRVP RQFYINLRTP DLYQWEKVTR
TLPRGLPATN LFIDLMNDPN VDGVFEQQTL NKAETVGFNL QQLETKFILL YHACSATAAL
HVFALFYPGG VKMHVVDPAT RRQPTTFHGN DATALKAVSR ELGLIENRGY TVVISSSSKDQ
SYFDRSVQKL TKFPILSMTK IRQAHTLDFP WQLHVAHKMN ARYLAMGPWL DRLIATAQYF
PLVVSDISFA RRLVQQDMVL WWSPGENPDL GGIECDRRPT DDFPRTEFMS PGAYPNVCLE
ITIRNLAVNS VLHSVLVNEL EGSGGATAFD ANQRDVTFGE SNVSPHMFSI LRNMLKGWLL
DRIGPASMGI DHFWRWISSS NATMYDPSLH RFVHGLMRKT FIQMLAEFRR LGSQIVCADF
GRVLLATSKP PGTAYAYATY LTSAVTSHEL FEHVKLTTDR FYDFLLFMDP ANVGGVVCED
PLAVEPPTEL ALEMRWNIAQ FLPKAIRPDF EAVVQYFIVE LFKARQKKVQ EADLIQEFIS
RKLTRKMLRA VFPVLPGSHL TLESPPLEFV KFACAVFTLA KDYTVEVGLL KRLLLELIGV
REFASEAAFR NPCEPLVLHG VPCRHCDALR DFDLCRDPDL LPWACATCGG EYDRLAIEFR
LVGMVQGMER AFAQQDLRCS RCQQVQSDNV SRTCCSGAYQ HTMNKADIRR RLRTAVNVAI
VHNLPRL
>HI
GHRRLYLQLC FRNVSDLLAV RRDIVPLALA NSAKRSAVDA YAEVVDPREG IIDVREYDVP
YYLRVAMDNE IRVGMWYAIT FNAGHPALDR VKRPDPVVMA YDIETTKAPL KFPDQAIDQV
MMISYMVDGQ GYLITNREIV SQDIEDFEYT PKEGYEGPFI VFNEADEAAT ITRFFEHIQE
VKPTVMATFN GDFFDFPFLN ARAQVHGIDM FLETGFAKDT EDEYKSRTCV HMDCFRWVKR
DSYLPQGSQG LKAVTVAKLG YNPIELDPEL MTPYALEQPQ TLAQYSVSDA VATYYLYMKY
VHPFIFSLCN IIPLCPDEVL RKGTGTLCET LLMVEAFRGH IIMPNRHEEE HGNMYEGHLL
ASETYVGGHV EALEAGVFRS DISTHFKIEP SAAQLIDELD AALSFCVANY DEVKGQIQAA
LEEMRDNPLR MDKPLIYHLD VAAMYPNIML SNRLQPDSVV DESVCAVCDY NRPGKTCDRR
LTWAWRGEFF PARRDEYNME TFPPKKNGGP PRKFVDLSET EQTALIHKRL GDYSRKVYKK
IKDTKVENRE AILCQRENPF YVDTVRRFRD RRYEYKGLHK TWKKNLDSLA EVDEAKKLIL
AHKCILNSFY GYVMRKGARW HSMEMAGVTC LTGATIIQMA RQLVEQVGRP LELDTDGIWC
MLPGVFPENF KFKLRNGKAI GFSYPCTMLN HLVHDQFTNH QYHDLDPETG EHKVHSENSI
FFELDGPYRA MILPSSKEED KLLKKRYAVF NDDGSLAELK GFEVKRRGEL QLIKIFQSQI
FEKFLLGSTT EECYAAVAQV ADQWLDVLFT KAEDLSDEEL VELIAENRSM SKTLAEYGTQ
KSTSISTAKR LAEFLGSQMV KDKGLACKFI ISERPFGAPV TERAIPVAIF SADESVKRTY
LRKWLKDNGL TQFDLRSILD WEYYIERLGS VIQKLITIPA AMQKVANPVP RIRHPDWLHR
RVVALDDRFQ QHKVTDFFPG RYNLWLSVDS KLVSAPLRIP REFYLHLRNP DLYSCDKVVK

NLPRNLPCVN LFVDLTNDPN VDGIFELQTL NRAQITGLDL DALQPKYVFL YHACSINRPT
QIFALFLPNG VRLHLVDPAT RRQPADYHAN DITALKAVSR DLGMLEDQSL TVVISSVKEQ
SYFDAFLPKL SKFPVLSMPK ARAPHSLDFP WQTHFAQKML IRYFSLGPWL DRMVSLAEYY
PLFLSDLDFA RRLTNQDTLL WWSPSGRPDL GGMEDDRRFT EELPQTDFVS PGCYSNVSLE
ITVRNLAVNA VLHSVVVNEL EGSGGTTAFD SAKRDLTLGE SNISPQTIGV MKSMVKTWLL
DKISPASLAI DHFWRWISSR ASHMYDPSMH RFVHGLMRKT FIQLLAEFKR LGSHVVYADF
SRILLVTSKP PGTAHAYATY ITTAVTSHEL FQHIYLFTEK FYDFLIFMDE ANLGGIVCED
PLAVEPPDEL IIEMNWNIQK HLPVAIQRDF IDLVRYFIIH LYKTKQKRTK EMDSVKDFLA
RRLMRKLFMF IFPLLPGSYL NMSDPTLEFI KFVCAVFELA KDYQSEVGLL KRNALELIGV
KYFGDEVIFR NPCDPLKLPS VPCRHCDALR DFDFCRDPDL LPWSCSDCGG EYDRVAIELE
LMEFVHGLER TFAQQDLKCG KCKQLRSNNV AKHCCSGSFQ LTINKADVRR KLKTVFNVAI
VHKLVRL
>SH
GHRRLYLQLC FRNVSDLLAV RRDIVPLALA NSAKRSAVDA YAEVVDPREG IIDVREYDVP
YYLRTAMDNE LRVGMWYGVT FNAGQPEFER VKRPDPVVMA YDIETTKAPL KFPDQAIDQV
MMISYMVDGQ GYLITNREIV SEDIDDFEYT PKEGYEGPFI IFNEADEAAT IRRFFQHIQE
VKPTVMATFN GDFFDFPFLC ARAQTHDIDM FLEIGFAKDS EDEFKSRTCV HMDCFRWVKR
DSYLPQGSQG LKAVTVAKLG YNPIELDPEL MTPYAMEQPQ VLAQYSVSDA VATYYLYMKY
VHPFIFSLCN VIPLCPDEVL RKGTGTLCET LLMVEAFRGH IIMPNRHEEE HGNMYQGHLL
ASETYVGGHV EALEAGVFRS DIETDFKIVP SAAQLIDELD AALTFCVTNY DEVKQQIQTA
LEFMRDNTSL QAKPLIYHLD VAAMYPNIML SNRLQPDSVV DEAACAVCDY NRPGKTCDRR
LTWAWRGEFF PARRDEFNME TFPPKRVGLP PRRFPDLSPS EQTALLHKRL GDYSRKVYKK
IKDTKVENRE TIICQRENPF YVDTVRRFRD RRYEYKGLHK TWKKNLDSLA EVDEAKKLIL
AHKCILNSFY GYVMRKGARW HSMEMAGVTC LTGATIIQMA RQLVEQVGRP LELDTDGIWC
MLPGCFPENF KFKLTNGKSI GFSYPCTMLN HLVHDQFTNH QYHDLDLESG EYKVHSENSI
FFELDGPYRA MILPSSKEED KLLKKRYAVF NDDGTLAELK GFEVKRRGEL QLIKIFQSQI
FEKFLLGTTT QQCYAAVAEV ADQWLDVLFS KAADLTDEEL VELIAENRSM SKTLAEYGGQ
KSTSISTAKR LAEFLGDQMV KDKGLACKFI ISARPLGMPV TDRAVPVAIF SAEESVKRMY
LRKWLKDNGL TNFDLRSILD WDYYIERLGS VIQKLITIPA AMQKVPNPVP RVRHPDWLHR
RVAALGDTFR QHKMTDFFPG RFNLWLQVNT ELVALPVRIP REFYVHMRSA ELYSCDKVVK
NLPRNTPCLN LFVDLTNNPN VDGVFELQTL NRAQTTGLDL EQLERKYIFL FHACSLNAPV
HVFAIFMPTG VRLHIVDPAT RRQPSTYHSS DLTALQAVSR ELRLYESQSY TVIISSMKEQ
SYFDEHCPKL SRFPVLSMSK SKGPHSLDFP WQSNVAHKML SRYISLGSWM DRMVALAEYY
PLFLSDLEFA RRLISQDCVL WWSPSGSPDL GGMEGDQRYT EELPHTEFKS PGCYSNVSLE
ITVRNLAVNS VLHSVVVNEL EGSGGTTAFD SAQRDLTLGE ANISPQTFGI MKSIVKGWLL
DKISPASLTI DHFWRWVTSK SSHLYDPSIH RFIYGLMRKT FIQLLAEFKR LGSHVVYADF
SRILLVTSKP PGTAHAYATY LTTAVSSNEL FQHIFLQTER FYDFLLFMDQ ANLGGVVCED
PLAVVPSRDV SINMQWNIQK YLPPAIQRDF ADFVRYFLVE LYKARQKRSK EVDSVKEFIA
RKLTRKLFKY IFPLLPGSYL RLVDPTLEFI KFACAVLELA KDFQSEIGLL KRNALELIGV
KSFNERAVFR NPCEPLKLSN VPCRHCDALR DFDFCRDMDL LPWLCAVCGG EYDRTAIELE
LMEMVHALEQ TFAQQDLKCG KCKQVRSDNV SRICCSGTYQ PTLSKADVRR KLRTIVNVAI
VHNLGRL
>CC
GHRRLFLQIC FRNISDLLAV RREIMPLALA NGAKRDAVDA YAEVVDPREC IIDIREYDVP
YHLRVAIDND IRVGLWYAIT FTAGQPTFER VKRADPVVMA YDIETTKAPL KFPDQAIDQV
MMISYMVDGQ GYLIVNREIV SEDIEDFEYT PKEGYEGPFI VYNEPNEEAT IKRWFSHIQE
VKPTIMATFN GDFFDMPFID ARAKVNGLDM FLETGFAIDA EEEYKSRTCV HMDCFRWVKR
DSYLPQGSQG LKAVTTAKLG YNPIELDPEL MTPYAVEQPQ VLAQYSVSDA VATYYLYMKY
VHPFIFSLCN IIALNPDEVL RKGSGTLCET LLMVEAYRSH IIMPNKHEET HGNMYEGHLL
SSETYVGGHV EALEAGVFRS DIATDFKVVP EALTLIDDLD AALTFCVTNY EEVKREIQDK
LELMRDNPKR VDNPLIYHLD VAAMYPNIML SNRLQPDSMV QESDCAVCDF NRPGKTCDRR
LQWAWRGEYF PAHRDEFNME SFPGKRAHDP PRRFTDLSPA EQTALLHKRL GDYSRKVYSK
TKETKVETRT SIVCQRENPF YVDTVRRFRD RRYEYKGLHK KWKNNLDSIA EVDEAKKMIL
AHKCILNSFY GYVMRKGARW HSMEMAGITC LTGATIIQMA RALVEQIGRP LELDTDGIWC

MLPGVFPENF KFNLKNGKSI KFSYPCTMLN HLVHDKFTNH QYHDLNPETG EYEVHSENSI
FFELDGPYKA MILPSSKEED KLLKKRYAVF NDDGSLAELK GFEVKRRGEL QLIKIFQSQI
FEKFLLGTTL QECYAAVAEV ADQWLDILYT RGETLSDEEL VDLIAENRSM SKTLAEYGGQ
KSTSISTARR LAEFLGDQMV KDKGLACKFI ISAKPIGSPV TDRAVPVAIF SAEDSVKRTY
LRKWLKDSSL NNFDLRAILD WDYYIERLGS VIQKLITIPA AMQKVANPVP RIRHPDWLFR
RVAAQGDKFK QNKMTDFFPG RFLLWVYVDA KLVSIPLRIP REFYVHLKKP EFYSVEKVTK
NLPHDLPCTN LFIDLTNDPN VDGVFELQSL NRAQQTGLDL QQLDTKHIFL YHATTPNGKV
QVFAVFYPNG TKLHIIDPAV RRQPTVHHSN DVTALKAISR ELGLLTDSSL MVVISSNKDV
SYFDRLVPRL SKFPVVAMSS ARNAHLLNLP WQSTAGVKIL KRYLALGPWL DRMIALADYY
PLLLSDITYA RRLIHQDTVL WWSPSDLPDL GGIEADIQAL EDWPRTDFLS PGVYSNVCLE
VTVRNLAVNS VLQSVVINEL EGSGGTTAFD SSQRDITLGG SRVSTHAFSI LKNMLKGWLL
DKISPACLAL DHFWRWVTSK GSQLYDPSLH RFIHGLMRKT FIQLLAEFKR LGSHVVYADF
STILLATSKP PGTAHAYATY ITTAVTSHEL FQHIYLHTEK FYDFLICMDR ANMGGVVCED
PLAVEPPAEL AVEMNWNIQH FLPPAIQHDF GSVVQYYIVE LYRIRQKNDS EKSMIVDFIA
RKFTRKLLKV IFPTLPGSHL HMTHPTLELI KFLCAVFSLA KDYTVEIGLL KRNLLELINV
REFAQEAIYR NPCEPLKLSN VPCRHCDLLR DFDFCRDLSL LPWLCTNCNG EYDKTVIEFS
LIDMTKSIEQ KFARQDLRCS KCQQIQSDNV SRYCCSGTYQ YTLNKAEAKK TLKTMVNIAR
EYKLARL

>LB

GHRRLFLQLC FHNISDLLTV RRDILPLAQA NSAKRDAVDA YAEVIDPREG IIDIREYDIP
YYLRVAIDND IRVGLWYAVT FTAGHPSFER VKRADPVVMA YDIETTKAPL KFPDQAIDQV
MMISYMVDGQ GYLITNREIV SEDIEDFEYT PREGYEGPFI IFNEKDEAAT IKRFFSHIQD
VKPTVMATFN GDFFDFPFLD ARSKVNGIDM FLETGFAKDA EDEYKSRTCV HMDCFRWVKR
DSYLPQGSQG LKAVTTYKLG YNPIELDPEL MTPYALEQPQ ALAQYSVSDA VATYYLYMKY
VHPFIFSLCN IIPLNPDEVL RKGSGTLCET LLMVEAYRGH IIMPNRHEDA HGSTYEGHLL
ASETYVGGHV EALEAGVFRS DIATDFKIVP AAVQLMDDLD AALTFCVTNY EEVKAEIKAA
LEVMRDNPKR VDNPLIYHLD VAAMYPNIML SNRLQPDSMV DESVCAVCDY NRPGKTCDRR
LEWAWRGEFF PAHRDEFNME SFPPKRPGGP QRRFPDLNPT EQTALLHKRL GDYSRKVYKK
TKDTKVENRE AIVCQRENPF YVDTVRRFRD RRYEYKGLHK TWKKNLDSIT EVDEAKKMIL
AHKCILNSFY GYVMRKGARW HSMEMAGITC LTGATIIQMA RALVEQIGRP LELDTDGIWC
MLPGVFPENF KFKLTNGKNI GFSYPCTMLN HLVHAKFTNH QYHDLDPDTG EYVIHSENSI
FFELDGPYKA MILPSSKEED KLLKKRYAVF NDDGSLAELK GFEVKRRGEL QLIKIFQSQI
FEKFLLGTTT QECYAAVAQV ADQWLDVLYS QAESLGDDEL VELIAENRSM SKTLAEYGSQ
KSTSISTAKR LAEFLGDQMV KDKGLACKFI ISAKPMGAPV TERAIPVAIF SAEESVKRTY
LRRWLKDNGL VTFDLRSILD WNYYIERLGS VIQKLITIPA AMQKVSNPVP RVRHPDWLFR
RVAGAVDKFK QNKLTDFFPG RFMLWLQVDS ELLAVPLRVP REFYIHLKKP EYYSCEKVTR
NLPHDLPCIN LFVDLTNDPN VDGVFEQQTL NRARQVGFDL NQLDRKYLFL YHACSANAPL
HVFAVFMPGG VRLHIVDPAT NRQPTAYHSN DTTALKAVSR ELGLFEDKSF IVVISSSKDQ
SYFDRLVPKL AKFPVLCMSQ AKGPHTLDFP WHSHLAQKML NRYLSLGLWL DRMIDLAEYY
PLMLSDISFA RRLVQQDIVL WWSPGDQPDL GGIEHDRRPT EDLPKTDFLS AGVYSNVCLE
ITVRNLAVNS ILQSVMVNEL EGSGGATAFD STQRDLTLGE SQVSAQTFGI MKNMLKSWLV
DKINPANLAL DHFWRWITSS ASHLYDQSIH RFVHGLMRKT FIQLLAEFKR LGSVVVYADF
STILLATSKP PGTAHAYATY ITTAVTSHEL FQHIYLNTER FYDFLVCMDR ANLGGIVCED
PLALEPPEEL AMEMRWNIEQ FLPPAIQGDF SMIIQYFIIE LYRIKQKKAN EIELISEFIA
RRLTRKMLKV VFPVLPGSYL HMSNPTLEFT KFACAVFALA KEYRNEVGIL KRNLLELVGV
REFAGEATFR NPCEPLKLAN VPCRHCDVLR DFDFCRDPEL LPWLCNHCGG EYDRVAIEFM
LIEMTRALER NFAQQDLRCG KCQQIQSDNV SRYCCSGSYQ FTISKADMRR KLRTIVNVAR
EYNLPRL

>CP

GHRRLYLQLC FRNVSDLLAV RRDVAPLALA NGAKRDAVDA YAEVVDPGEC IIDVREYDVP
YYLRVAMDNA MRVGLWYAVT FTAGQPGFER VKRADPVVMA YDIETTKAPL KFPDHALDQV
MMISYMIDGE GYLITNREIV GEDIEDFEYT PKEGYEGPFT VFNEPDEAAT IMRFFHHIQS
AKPTVMATFN GDFFDFPFLC ARSKANGIDM FLETGFAIDQ EDEFKSRCCV HMDCFRWVKR
DSYLPQGSQG LKAVTTAKLG YNPIELDPEL MTPYAMEQPQ VLAQYSVSDA VATYYLYMKY

VHPFIFSLCN IIPLCPDEVL RKGSGTLCET LLMVEAYENH IIMPNRHEDA HFNMHEGHLL
ASETYVGGHV EALEAGVFRS DIPTDFKIVP EAAQLIDQLD DALKFCVTNY DEVKSEIQAA
LEEMRDNPKR LDKPLIYHLD VAAMYPNIML SNRLQPDSIV DESVCAVCDY NRPGKTCDRR
LEWAWRGEFF PAHRDEYNME TFPSKRPGGP QRRFADLTDA EQTALLHKRL GDYSRKVYKK
IKDTKVEMRT SIVCQRENPF YVDTVRRFRD RRYEYKGLHK TWKKNLDSIA EVDEAKKMIL
AHKCILNSFY GYVMRKGARW HSMEMAGITC LTGATIIQMA RALVEQLGRP LELDTDGIWC
MLPGVFPENF KFKLNNGKAL AFSYPCTMLN HLVHAQFTNH AYHDLNPETG DYDIHSENSI
FFELDGPYRA MILPSSKEED KLLKKRYAVF NDDGSMAELK GFEVKRRGEL QLIKIFQSQL
FERFLLGTTT EECYRAVAHA ADQWLDILFS KAETLSDEEL VDLIAENRSM SKTLAEYAGQ
KSTSISTAKR LAEFLGDQMV KDKGLACKFI ISAQPHGAPV TERAVPVAIF SAEESVKRTY
LRKWLKNNGL TSFELRAILD WDYYIERLGS VIQKLITIPA AMQKVANPVP RIRHPDWLHR
RVAGQVDKFK QNKMTDFFLG RFNLWLSVDN SVVSIPLRIP REFYLHFKTP EYYSCAKVVR
HLPRNFQCNN LFTDLTNDPN VDGVFEMQTL NRAQSVGVDL TQLDRKYVFL YHACSVSAPI
HVFALFLPAG VKLHIVDPAT RRQPTTYHNK DTTALKAISR ELGTLEDRSY IVVLSSMKDQ
SYFDMWTPKL AKFPVLSMPK TRKPHSLDFP WQTHVAKNML SRYLNVGSWL DRSIALADYY
PILLSDISFA RRLAEQDIVL WWSPSDRPDL GGVEQDNRPT EELQTTEFNS PGCYSNVCLE
ITVRNLAVNS VLHSLVVNEL EGAGGATSFD SQQRDLTLGE SQVSSITFGI LKSLIKGWLI
DKISPATLTI DHFWRWVSSS ASHMYDPNIH RFIHGLMRKT FIQMMAEFKR LGSHVVYADF
SRILLATSKP PGTAHAYATY ITTAVNSNEL FQHIFLRTER FYDYLLFMDQ ANLGGVVCED
PLALEPPEDI SIELRWNIQT FLPAAIQQDF ANIMQYCIVK LFKIRQKKEN EIELTRDFIA
RQLQRKLLST VFPVRPGSYL ALSNPVLEFV KFACAVFGLA RDYSVEVGLL KRSLLDLAGV
REFAAEAVFR NPCEPLKLSN VPCRHCDQLR DFDFCRDEEL APWTCGTCGG EFDRMAIELD
LMKMVANLER AYTQQDMVCV KCKQLRSDNV SRWCCSGAYQ LTIGKAEMRR RLRTIVNVAL
VHGLPRL
>SL
GHRRLYLQLC FRNVSDLLTV RRDIAPLALA NGAKRDAVDA YAEVVDPGEC IIDIREYDVP
YYLRVAIDNE LRVGLWYAVT FTAGQPSFER VKRADPVVMA YDIETTKAPL KFPDQAIDQV
MMISYMVDGQ GYLITNREIV SEDIEDFEYT PKEGYEGPFI VFNEPDEAAT MMRFFQHIQE
VKPTVMATFN GDFFDFPFLC ARAKANGIDM FLETGFAIDS EDEFKCRTCV HMDCFRWVKR
DSYLPQGSQG LKAVTTAKLG YNPIELDPEL MTPYAMEQPQ VLAQYSVSDA VATYYLYMKY
VHPFIFSLCN IIPLCPDEVL RKGSGTLCET LLMVEAYRGR IIMPNRHEDA HGNMHQGHLL
ASETYVGGHV EALEAGVFRS DIPSDFKVVP SAVQLIDDLD AALMFCVTNY DEVKNEIQAA
LEVMRDNPKR TDKPLIYHLD VAAMYPNIML SNRLQPDSMV DESVCAVCDY NRPGKTCDRR
LEWAWRGEFF PAHRDEFNME SFPSKRPGGP QRKFADLTET EQTALLHKRL GDYSRKVYKK
IKDTKIETRE SIVCQRENPF YVDTVRRFRD RRYEYKGLHK TWKKNLDSIA EVDEAKKMIL
AHKCILNSFY GYVMRKGARW HSMEMAGITC LTGATIIQMA RALVEQIGRP LELDTDGIWC
MLPGVFPENF KFKLNNGKSI AFSYPCTMLN HLVHAQFTNH QYHDLDPETG EYVVHSENSI
FFELDGPYRA MILPSSKEED KLLKKRYAVF NDDGSLAELK GFEVKRRGEL QLIKIFQSQI
FEKFLLGSTT EECYAAVAEV ADQWLDILFS KAETLGDDEL VDLIAENRSM SKTLAEYGGQ
KSTSISTAKR LAEFLGDQMV KDKGLACKFI ISARPMGAPV TERAIPIAIF SAEESVKRTY
LRKWLKDNSL TNFDLRSILD WDYYIERLGS VIQKLITIPA AMQKVTNPVP RVRHPDWLHR
RVAGAIDKFK QNKVTDFFQG RFTLWLSIDG ELTPVALRIP RVFYLHLKSL GHYSCDKVIR
NLPRGLHCSY LFTDLINDPN VDGVFEQQTL NRAQSSGVDL VQLDRKYIFL YHACSNSAPI
HVFVLFLPSG VKLHVVDPAT RRQPTTYHNN DITALKAISR ELGLLENMSY SIVVSSTKEQ
TYFDTWVPKM SKFPVLSMSK TKAAHTLDFP WQTHVAQKLL YRYLSVGGWL DRLVVLADYY
PLLLSDITFA RRLIQQDTVL WWSPSERPDL GGLEDDFRPV EELQNTEFMS PGCYSNVCLD
VAVRNLAVNS VLHSVVVNEL EGSGGTTAFD SAQRDITLGE SSLSPQTFGI LRTMVKGWLL
DRISPATLAI DHFWRWVSTS ASNMHDPSIH RFVHGLMRKT FIQMLAEFKR LGAHVVYADF
SHILLATSKP PGTAHAYATY VATAVTSHEL FQHIYLKAER FYDFLLFMDP ANMGGVVCED
PLAVDPPEEL SIEMRWNIQT FLPPAIQDDF GKVLQYFIVE LFQIRQKKVN EMDATREFIA
RRLTRRLLKV VFPLLPGSHL HLINPILEFI KFTCAVLGLA QEYRIEVGLL KRNLLDLAGV
REFATEAIFR NPCEPLRLAN VPCRHCDTLR EFDFCRDPEL MPWLCGGCGV EYDRIAIELE
LMRMVCSLER SFAQQDLRCA KCKQVRSDNV SRYCCSGAYQ LTMNKAEVRR RLRTVVNVAI
VHGMAKL

>FS
GHRRIYLQLC FRNVADLLNV RRDIVPLALA NSAKRDAVDA YAEVVDPREG IIDVREYDVP
YYLRVAIDNE IRVGVWYSVT FNAGQPSFER VKRADPVVMA YDIETTKAPL KFPDQAIDQV
MMISYMVDGQ GYLITNRDIV SEDIEDFEYT PKEGYEGPFI VFNEADEAAT IMRFFEHIRE
VKPTVMASFN GDFFDFPFLD ARSKANGIDM FLETGFAKDS EDEYKSRACA HMDCFRWVKR
DSYLPQGSQG LKAVTTSKLG YNPLELDPEL MTPYAVEQPQ ILAQYSVSDA VATYYLYMKY
VHPFTFSLCN IIPLNPDEVL RKGSGTLCET LLMVEAYRGQ IIMPNRHEEA HGSMYDGHLL
ASETYVGGHV EALEAGVFRS DIPTDFNVVP SAVQLIDDLD AALKFCVTNY DEVKAEITAA
LELMRDNPKR TDKPLIYHLD VAAMYPNIML SNRLQPDSVV DEAVCAVCDY NRPGKTCDRR
LDWAWRGEFF PAHRDEFNME TFPPKFPGKP QRRFPELTEA EQTALLHKRL GDYSRKVYKK
TKDTKVETRE AIVCQRENPF YVDTVRAFRD RRYEYKGLLK TWKKNLDSIT EIDEAKKLVL
AHKCILNSFY GYVMRKGARW HSMEMAGITC LTGATIIQMA RALVEQLGRP LELDTDGIWC
MLPGVFPENF KFKLKNGKSI GFSYPCTMLN HLVHAQFTNH VYHDLDPETG EYIVHSENSI
FFELDGPYRA MILPSSKEED KLLKKRYAVF NDDGSLAELK GFEVKRRGEL QLIKIFQTSI
FEKFLLGSTT EECYAAVAQV ADQWLDVLFS KADTLGDDEL VDLIAENRSM SKTLAEYGGQ
KSTSISTAKR LAEFLGDQMV KDKGLACKFI ISAKPMGAPV TERAVPVAIF SAEESVKRVY
LRKWLKDNSL ANFELRSILD WNYYIERLGS VIQKIITIPA AMQKVSNPVP RIRHPDWLHR
RVAGAVDKFK QNKVTDFFAG RFTLWLSVDA KLVSVSLRIP RTFYLNLKSP EFYSYEKVVR
NLPHDIRGAN LFMDLTNDPN VDGVFEQQTL NRAENIGFDL DQLDRKFIFI YHASSSTGNL
HVFAVFVPTG VKLHVVDPAV HRQNTTYHSK DITALKAINR ELSFMEDMSY TLVISSTKEQ
AYFEAHIPRL TKFPVLSMPK TKMAHALDFN WQTHVGPKML NRYLRLGQWL GRQISWADYY
PLFLADINFA RRLNEQDMII WWSTSDRPDL GGIENDRRPI EDLPNTEFVS PGCYPNVCLE
VTVRNLAINS VLHSVMVNEL EGSGGATAFD SAQRDLTLGE SSVSVQTFGI LKTMIKTWLL
DKISPAAVAV DHFWRWISSS ASHMFDPSLH RFIHGLMRKT FIQMLAEFKR LGSHIVYADF
SHILLATTKP PGTAHAYATY IMTAVTSNEL FQHIYLNTER FYDFLLFMDQ SNMGGMVCED
PLAITPPEEL SIEMRWNIAS FLPLVIQSEF NSIIQFFIVE LFRIRQKKTK EMDAMREFIA
RRLTRKVLRA VFPTLAGSHL RLENPVLEFC KFACAVFGLA KEYQVEIGLL KRSLLELINV
REFSSEATFR NPCEPLKLSN VPCRHCDALR DFDFCRDPDL SAWLCDTCGG DYDRTTIELM
LMEVVYSLER SFAQQDLKCA KCKQIQSDNV SRYCCSGSYQ HTVSKVDVRR KLKTVVNVAI
MHNLGRL
>PI
GHRRIYLQLC FRNVSDLLTV RRDIMPLALA NSAKRDAVDA YAEVVDPREG IIDIREFDVP
YYLRVAIDND IRVGLWYAVT FTAGQPSFQR VKRADPVVMA YDIETTKAPL KFPDQAIDQV
MMISYMVDGQ GYLITNRDIV SEDIEDFEYT PKEGYEGPFI IFNEADEAAT ITRFFEHIRD
VKPTVIASFN GDFFDFPFLD ARARINGIDM FLETGFAKDA EDEYKSRTCV HMDCFRWVKR
DSYLPQGSQG LKAVTTAKLG YNPIELDPEL MTPYAMEQPQ ILAQYSVSDA VATYYLYMKY
VHPFIFSLCN IIPLNPDEVL RKGSGTLCET LLMVEAYRGQ IIMPNRHEEA HGSMYDGHLL
ASETYVGGHV EALEAGVFRS DIPIDFKIEP SAVHLIDDLD AALTFCVTNY DEVKREITAA
LELMRDNPKR TDKPLIYHLD VAAMYPNIML SNRLQPDSMV DESICAVCDY NRPGKTCDRR
LDWAWRGEYF PAHRDEFNME SFPPKRPGGG QRRFPDLTET EQTALLHKRL GDYSRKVYKK
TKETKIETRQ AIVCQRENPF YVDTVRRFRD RRYEYKGLLK TWKKNLDSIA EIDEAKKLIL
AHKCILNSFY GYVMRKGARW HSMEMAGITC LTGATIIQMA RALVEQIGRP LELDTDGIWC
MLPGVFPENF KFKLDNSKSI AFSYPCTMLN HLVHAQFTNH EYHELNPETG EYVVHSENSI
FFELDGPYRA MILPSSKEED KLLKKRYAVF NDDGSLAELK GFEVKRRGEL QLIKIFQTSI
FEKFLLGATT EECYAAVAEV ADQWLDVLFS KADTLGDDEL VELIAENRSM SRTLAEYGGQ
KSTSISTAKR LAEFLGDQMV KDKGLACKFI ISARPMGAPV TERAVPVAIF SAEESVKRVY
LRKWLKDNSL TNFDLRSILD WDYYIERLGS VIQKLITIPA AMQKVSNPVP RIRHPDWLHR
RVAGAVDKFK QNKVTDFFSG RFTLWLSVDS ELVSVTLRVP RIFYLNLKTP DFYSCEKVIR
NLPRDLNGAN LFVDLINDPN VDGVFEQQTL NRAERTGFDL SQLDRKFVFF YHACSGNGAV
HVFALFLPAG VTLHIVDPAT RRQPTTYHSK DATALRAISR ELGLLENMSH TVVISSSKDQ
TYFDSSMPKL SNFPVLAMSK AKGHHSLDFP WHSHVAQKML NRYLNFGPWL DRMIQLADYY
PLLLADISFA RRLSQQDMVL WWSPGDRPDL GGVENDKRPT EELPNTEFVS PGCYSNVSLE
VTVRNLAVNS VLHSVMVNEL EGSGGATAFD STQRDLTLGE SSVSAQTFAI LKSMIKTWLL
DKISPATLAV DHFWRWISSS ASHMFDPSIH RFIHSLMRKT FIQMLAEFKR LGSHIVYADF

SRILLATSKP PGTAHAYANY ITSAVTSNEL FQHIYLNTER FYDFLLFMDQ ANMGGVVCEN
PLAVAPPEEL AIEMRWNIES FLPPAIQGDF SAVVQYFLVE LFRIRQKKAN EMDDSREFIA
RRLTRKLLKV VFPTHPGAHL QLVNPVMELV KFMCAIFGLV KDYSVEIGLL KRNLLELIGI
KEFATEAIFR NPCEPLKLSN VPCRHCDALR DFDFCRDIEL IPWFCQHCGG EYDRIAIELM
LMEVVQTLER TFAQQDLKCS KCKQIQSDNV SRYCCSGTYQ FTISKADVRR KLRTIVNVAL
VHNLSRL
>AS
GHRKLYLQLC FKNVTDLLAV RRDIMPLALA NSAKMNAVDA YAEVVDPRES IVDIREFDVP
YFLRVAIDNE IRVGLWYTVT FSAGKPTISR VQRAEPVVMA YDIETTKAPL KFPDQQIDQV
MMISYMIDGQ GFLITNRDII SEDIDDFEYT PKEGYEGPFT VFNEPDEAAT IKRFFQHIQT
AKPTVMATFN GDFFDFPFLC ARAKVHDIDM LLETGFAKDS EDEFKSNTCV HMDCFRWVKR
DSYLPQGSQG LKAVTTAKLG YNPIELDPEL MTPYALEQPQ TLAQYSVSDA VATYYLYMKY
VHPFIFSLCT IIPLRPDEVL RKGSGTLCET LLMVEAYRAG VIFPNKHEDP HGNTFEGHLL
ASETYVGGHV EALEAGVFRA DIPTHFKIVP SACQLIDGLD AALKFCVTNY EDIKSQIQSA
LEEMRDNPMR MDKPRIYHLD VAAMYPNIML SNRLQPDSVV DEATCAVCDY NRPGKTCDRR
MTWAWRGEYY PAQRDEYNME MFPPRRPGLP KRRFVELGEA EQAALLHKRL GDYSRKVYKK
TKETRVVERE AIICQRENPF YVETVRTFRD RRYEYKGLHK TWKKNLDSVA DIEEAKKMIL
AHKCILNSFY GYVNRKGARW QSMEMAGITC LTGAHIIQMA RQLVEQIGRP LELDTDGIWC
MLPDVFPEDF TFQVSKGKPL KFSYPCTMLN HLVHDKFTNH QYHDLEKETG EFKVHSENSI
FFELDGPYLA MILPSSKEED KLLKKRYAVF NYDKSLAELK GFEVKRRGEL QLIKIFQSEI
FDKFLLGSTT EECYAAVAEI ANQWLDILFS RANSMSDDEL FELIAENRSM SRTLAEYAGQ
KSTSISTAKR LAEFLGEQMV KDKGLACKFI ISAKPIGAPV TERAVPVAIF SADEAVKRTY
LRRWLKDNSL STFDLRSILD WDYYIERLGS VIQKLITIPA AMQKVSNPVP RIRHPDWLFK
RVAALDDKFH QHKVTDFFPG RFALWLSTGS DLVSITLRIP REFYINLKRA PGYLSEQVAR
TLPRNRPSGS LFAELTNNTN VDGVYELETL NKARDDGVDL TGLEHKYLFL YHASSGSSSV
NVFALFTPRG ARLHVVDPAT RRQPVSYHKT ESAAQKAVSR DLGLLEKEQY VLVISSAKGM
SWHQAAIPKV ERFPVVMMTS SLRSTHSLLN WHVEVVKRMC TRYLAAGPWL HSLVGQATYY
ALFFADVDFA RRLLKEDMIL WWSSGSRPDL GGIEDDVRTT EEVINPQLVV PGCYDNVCLS
VQVRNLAINS VLQSSLVNEM EGAGGSTAFD SAQSSTTLGD AAVSPLTFNV IKSMVRSWLL
DKAGPADLVI SHFWRWMSSI AAQMHDPGLM RFVHGLMHKT FMQMLAEFKR LGSNVVAADF
GSILLVTSKP PGTAYAYGTY LKGAVTSHEL FKHMYLEIDR YYDYLVYMDN ANQAAVVCQN
PQEVEPPKSL AVTMAWNIEN FLPPAVQRIF HESVNFFMIS MYRIKRERNK EMEAVRSFIA
TRLTRKMLRS VFPVLPGSHL TLHNPALEFV KAVCAVFALA KDFKTEIGIL KRNMLDLVGV
REFAEEAVFR NPCEPFTLTM VICPCCADMR DMDFCRDVDL LPWQCAQCGY DFDKQEIDFA
LVSIIERLEA SFTTQDLRCS KCKQVRSDNV SKHCCSGEWR YILSKAEFRR RLRTAVNVAT
VHNLPLL
>EG
GHRRMYLQIL FKNVTDLLAV RRDIMPLALA NSAKMNAVDA YAEVVDPREC IIDIREYDVP
YYLRVAMDNE IRVGLWYTIT FSAGKPTVSR VARAEPVVMA YDIETTKAPL KFPDQEIDQV
MMISYMIDGQ GFLITNRDIV SEDIEDFEYT PKEGYEGPFT VFNEPDEEAT IKRFFQHIQT
AKPTVMATFN GDFFDFPFLY ARSKVHGIDM LLETGFAKDN EDEFKSNTCA HMDCFRWVKR
DSYLPQGSQG LKAVTTAKLG YNPLELDPEL MTPYAMEQPQ TLAQYSVSDA VATYYLYMKY
VHPFIFSLCN IIPLNPDEVL RKGTGTLCET LLMVEAFRGG VIMPNRHEDP YGNLFEGHLL
ASETYVGGHV EALEAGVFRS DIPTHFKIKP EACQLIDDLD RALKFCVTNY DDVKGQIQAA
LEEMRDNPMR MDKPRIYHLD VAAMYPNIML SNRLQPDSII DEATCAVCDY NRPGKTCDRR
LPWAWRGEFF PAQRDEYNME WFPPKRVGLP KRRFIELSES EQSALLHKRL GDYSRKVYKK
TKETRIVERE AIVCQRENPF YVDTVRRFRD RRYEYKGLHK TWKKNMDALA DIEEAKKMIL
AHKCILNSFY GYVMRKGARW HSMEMAGITC LTGAHIIQMA RALVDQIGRP LELDTDGIWC
MLPDVFPEDF TFEVSKGKPL KFSYPCTMLN HLVHDKFTND QYHDLDKETG EYAIHSENSI
FFELDGPYRA MILPSSKEED KLLKKRYAVF NFDGSLAELK GFEVKRRGEL QLIKIFQSQI
FEKFLLGSTT EECYAAVAEI ANQWLDVLFS HADTLNDEEL VDLIAENRSM SRTLAEYAGQ
KSTSISTAKR LAEFLGEQMV KDKGLACKFI ISAKPIGAPV TERAVPVAIF SADEAVKRTY
LRRWLKDNSM TTFDLRSILD WSYYIERLGS VIQKLITIPA AMQKVSNPVP RIRHPDWLHK
RVAALDDKFQ QHKVTDFFPG RFQLWLSTGS ELVSIALRIP REFYLNLKTA PGYLSEPAVR

TLPRNRPCHS LFAQLTNNPA VDGVYELETL NKARAEGVDL STLEHKYIFL FHASSSSASV
NVFALFMPTG VRLHVVDPAT RRQPVSYHKT EGAAQKAVSR DLGLVEKESY ILVISSSKSM
SWHQSAIPKI ERFPVVMMTS SLRSTHALLN WQVEVVKRMS TRYLAAGPWL HDSVEHATYY
ALYFADLDFA RRLVKEDMVL WWSSGSRPDL GGIEDDIRTT EELANSQLTY PGCYDNVCLS
VQIRNLAINS VLQSSLVNEM EGAGGSTAFD SAQAATSLGD AAVSPLTFAV VKSMVKSWLL
DKAGPADLVV NHFWRWMTSM SSQMFDPGLM HFVHGLMHKT FMQLLAEFKR LGSNVVSADF
GSILLVTSKP PGTAYAYATY ITSAVTSNEL FKHIHLETDR FYDYLVFMDN ANQAAVVCQN
PREVEPPQSL AVTMAWNIET FLPTALQRHF HDSVNYFMVS MYRIKREKNK EADAVRTFIA
QRLTRRMLRA VFPVLPGSHL TMTNPALEFV KFVCAIFALA KDFKTEIGIL KRNMLDLVGV
REFADDAVFR NPCEPFVLSM VICPYCADVR DMDFCRDADL LPWQCAQCDY DFDRQEIDFA
LVSIVERLET AFTTQDLRCA KCKQVRSDNV SKHCCSGEWR YSLSKAELRR RLRIAVNVAT
VHNLVLL
>SS
GHRRLYLQLC FRNVSDLLTV RREILPLALA NSKKMDAVDA YAEVVDPRES IIDIREYDVP
YYLRVAIDND IRVGLWYAVT FIAGQPSFER VKRADPVVMA YDIETTKAPL KFPDQAVDQV
MMISYMIDGQ GYLITNREIV SEDIEDFEYT PKEGYEGPFT VFNEKDEVAT LRRFFEHVKA
AKPTVMATFN GDFFDFPFLK ARSEIHGIDM FLEIGFAIDN EEEFKSRTCA HLDCFRWVKR
DSYLPQGSQG LKAVTTAKLG YNPIELDPEL MTPYAIEQPQ VLAQYSVSDA VATYYLYMKY
VHPFIFSLCN IIPLNPDEVL RKGSGTLCET LLMVEAYRGE IIMPNRHEDP HGNMFEGHLL
ASETYVGGHV EALEAGVFRS DIPTHFKVVP EAVQLIDQLD AALTFCVTNY DEVKGKIQSA
LEEMRDNPMR FDNPLIYHLD VAAMYPNIML SNRLQPDSVV DESVCAVCDY NRPDKTCDRR
MTWAWRGEYF PAQRDEFNME AFPPKRPGDP KRKFVDLPAA EQTALLHKRL GDYSRKVYKK
TKDTKVVYRE SIICQRENPF YVDTVRRFRD RRYEYKGLLK TAKKNLDSMA QQDEGKKLIL
AHKCILNSFY GYVMRKGARW HSMEMAGITC LTGATIIQMA RQLVEQIGRP LELDTDGIWC
MLPGIFPENF TFQVNKGKSV AFSYPCTMLN HLVHAQFTNH QYHDYNKDSG RYDIHSENSI
FFELDGPYKA MILPSSKEED KLLKKRYAVF NEDGSLAELK GFEVKRRGEL QLIKIFQTQI
FERFLLGTTT EECYAAVAEV ADRWLDVLFT KADSLSDEEL VELIAENRSM SKTLAEYGAQ
KSTSISTARR LAEFLGDQMV KDKGLACKFI ISQKPIGAPV TERAVPVAIF SAEESVKRTY
LRKWLKDNSL ANFELRSILD WNYYIERLGS VIQKLITIPA AMQKVSNPVP RIRHPDWLHR
RVAALDDKFQ QHKMTDFFPG HFTMWLSMDN DLIPVSLRIP REFYLNLKQM ESYSHEKLVR
TLPRSQPCLN LFINLVNNPN VDGVYELKNL NRARDIGFDL KQLERKYIFL FHSSAATAPV
HVFGLFYPTG VHLHIVDPAT RRQPTAYHGN DTTALKAISR ELGLIENRSF ALIISSGKEL
SYYETQISKL SRFPVFAMPS NRASHSLDFP WQADVAKKML SRYLVAGGWL LRTWDQAVYF
PLFMADIEFA RRLQKQDMVL WWSPNEQPDL GGSEDDRRAG EELVSPEFHA PGCYSNVCLS
ISVRNLAVDA VLQSSIVNEL EGSGGSTAFD SAQANITLGD SSVSPSVFAI LKNMVKTWLL
DKASPADVAV GHFWRWICST AAQMFEPNLQ RFIHGLMRKT FIQLLAEFKR LGSNVVFADF
SRILLVTSKP PGTAYAYATY ITSAVMSHEL FKHVDLQSEQ YYNYLVYMDH VNCGAIVCKD
PLATESAKGL VVSMDWNIER FLPPAVQQHF TEVVKTFIVD MYKIKQMKQK ELEACRTYIT
QRLTRKMFRA VFPILPGSHL NLTNPPLEFV KSACAVFGLA KEFSIEVGIL KRNLLDLVGV
KEFADEAIFR NPCEPLKLPM VICQHCNHIR DFDFCRDADL FPWKCPECDC EYDRLAIEFA
LIDLIHRLEA NFAPQDLKCG KCKQIRADNV SKHCCSGTYQ FTVSPAEFRR KLRTIVNVAT
VHNMQKL
>SP
GHRRLYLQIC FKNVSDLLAV RRDIVPLAVE NGAKLNAVDA YAEVVDPREC ILDAREFDVP
YYLRVAIDNE MRVGLWYTVT FVAGQPQFDR VKRADPVVMA YDIETTKAPL KFPDSAIDQV
MMISYMIDGQ GYLITNREIV SEDISDFEYT PKEGYEGPFT IFNEPDEAAT IMKFFHHIQT
AKPTVMATFN GDFFDFPFLC ARAKAHGIDM FLETGFAIDP EDEFKSRTCV HMDCFRWVKR
DSYLPQGSQG LKAVTTAKLG YDPIELDPEL MTPYALEQPQ TLAQYSVSDA VATYYLYMKY
VHPFIFSLCN IIPLNPDEVL RKGSGTLCET LLMVEAYRGN IIMPNRHEDA HGNMFEGHLL
ASETYVGGHV EALEAGVFRS DIPTHFKIVP EAAQLIDQLD DALTFCVLNY DEVKAEIQAA
LEQMRDNPML FEKPLIYHLD VAAMYPNIML SNRLQPDSVV DESVCAVCDY NRPGKQCDRR
MTWAWRGEFF PAQRDEYNME MFPPKKPGLP QRRYPDLSAS EQTAILHKRL GDYSRKVYKK
TKETKVVNRE SIICQRENPF YVDTVRRFRD RRYEYKGLLK TWKKKLDSIA EVDEAKKLIL
AHKCILNSFY GYVMRKGARW HSMEMAGITC LTGATIIQMA RQLVEQIGRP LELDTDGIWC

MLPGTFPENF KFKLSNGKPL QVSYPCTMLN HLVYAQFTNH QYHDLDPETG SYKVHSENSI
FFELDGPYRA MILPSSKEED KLLKKRYAVF NDDGSLAELK GFEVKRRGEL QLIKIFQSQI
FEKFLLGNTT QECYGAVAEV ADRWLDVLFT HADTLSDEEL VDLIAENRSM SKTLAEYAGQ
KSTSISTAKR LAEFLGDQMV KDKGLACKFI ISAKPIGAPV TDRAVPVAIF SAEESVKRTY
LRKWLKDSSL ANFELRSILD WEYYIERLGS VIQKLITIPA AMQKVANPVP RIRHPDWLHR
RVVASDDKFK QHKMTDFFRG KFTMWLSVNS ELIPITLRIP REFYLNFKTP VPYLRERVIR
TLPRERPCLH LFMAEINNPN VDGVYELQTL NKARDNGIDL TQVDHKYLFL YHAYSPSAPL
HVYALFLPNG VKLHIVDPAT RRQPTDYHGS EAAALKAISR ELGTLENRSF VLLLSSAKDF
TYFESMVPKL VKFPVLRLPG GKAAHALDFP WQSGIAKKMF ARYFSISPWL RRSIQQASYF
PLFLCDIDLA RRFAAQDMLL WWSAVDRPDL GGLEDDLQLS EETTNPEFLT PGMYSNVCLN
IQVRHLAIDA VLQSAVVNEL EGSGGTTAFD SAQPNVTLGE ANLSPQTFAT LKQMVRAWLL
DKACPSSLTI DHFWRWISTT SSRMYEPSVQ KFVHGLMRKT FIQLLAEFRR LGSNIVCADF
SRIVVVTSKP PGTAYAYATY ITTAVTSQEL FKHIHLRTDQ FYDFLLFMDP ANQGGILCED
PLAVEPTDQL AVFSSWNIKK FLPAAVQDKF RSVIKYFIVE MFKIRSSKQK ELDASKNFIM
QRLTRKMLQT VFPTLPGSHL NLTDVTLEFV KFTCAVFGLA KEFQIEVGLL KRNLLELVGV
REFADAAAFK SPCDPLKLSM VTCKYCDHIR DFDFCRDEDL FPWYCSECGC EYDKGVIEYS
LLQVLWRLER SFTEQDLRCG KCKQIQSDNV SKYCCSGSYQ LTVTKADARR KLRTMINVAI
VHNLSRV
>RF
GHRRLYLQLC FRNVSDLLTV RREVVPLALA NGAKLDAVDA YAEVVDPRDA IVDVREFDVP
YYLRVAIDND IRVGLWYTVS FVAGQPQFER VKRADPVVMA YDIETTKAPL KFPDQAIDQV
MMISYMVDGQ GFLITNREIV SEDIDDFEYT PKEGYEGPFT VFNEADEAAT ITRFFQHIQT
AKPTVMATFN GDFFDFPFLC ARAKVHGIDM FVETGFAIDS EDEFKSRTCA HMDCFRWVKR
DSYLPQGSQG LKAVTTAKLG YHPIELDPEL MTPYAMEQPQ ILAQYSVSDA VATYYLYMKY
VHPFIFSLCN IIPLNPDDVL RKGSGTLCET LLMVEAYRGE IIMPNRHEEE HGNMFEGHLL
ASETYVGGHV EALEAGVFRS DISTHFKIEP SAAQLIDELD AALSFCVTNY DDVKSQIQSA
LEQMRDSPLR HDKPLIYHLD VAAMYPNIML SNRLQPDSVV DESVCAVCDY NRPGKTCDRR
MTWAWRGEYF PAQRDEYNME WFPPKKPDGP KRRYTDLAPA EQTALLHKRL GDYSRKVYKK
TKDTKVVNRE SIICQRENPF YIDTVRTFRD RRYEYKGLLK TWKKNLDAVA EVDEAKKMVL
AHKCILNSFY GYVMRKGARW HSMEMAGITC LTGATIIQMA RQLVEQIGRP LELDTDGIWC
MLPGIFPENF KFQLKNGKSV AFSYPCTMLN HLVHAKFTNH QYHDLDRETG TYAVHSENSI
FFELDGPYRA MILPSSKEED KLLKKRYAVF NDDGSLAELK GFEVKRRGEL QLIKIFQSQI
FEKFLLGTTT EECYAAVAEV ADRWLDVLFS KADTLGDDEL VELIAENRSM SKTLAEYGGQ
KSTSISTAKR LAEFLGDQMV KDKGLACKFI ISAKPIGATV TERAVPVAIF SADESVKRTY
LRKWLKDNSL ANFDLRSILD WDYYIERLGS VIQKLITIPA AMQKVSNPVP RIRHPDWLHR
RVAAIDDKFT QHKLTDFFRG RFTLWLSVNN DVVPVKLRIP REFYLNFKSE QYYSRERVIR
TLPRDRPCLH LFVNEMNNPN VDGVYELQTL NRARDHGVEL EQLIRKYVFL YHAFSPSSPI
NVFALFMPNG VKLHIVDPAT RRQTTDYHGN EATALKAVSR DLGVMENKGY TLVLSSVKEL
GHFERSVPKL SKFPVLCMPS NRAGHALDFP WQSNVGKKVL TRYFHLAPWL QRVVAQAAYY
PLFLCDMEFS RRLLKQDMVL WWSAGEKPDL GGLEDDMHAT EELVNPEFVS PGCYSNVCLS
VQVRNLAINS ILQAAIVNEL EGSGGTTAFD STQAAVTLGD SSMSPQTFAT LKAMVKTWLL
DKASPSHLTI EHFWRWISSS GAHMFDPSIQ RFVHGLMRKT FIQLLAEFKR LGSNVVYADF
SRIVLVTSKP PGTAHAYATY ITSAVTSHEL FKHVLLRTDQ FYDFLLFMDP ANQGGVVCED
PLALEPPEQL AVFMSWNINK FLPPAVQDRF RRIVRYFLVE MYKIRRSKVK EMDATRSFIA
QRLTRKMLQT VFPLLPGSHL HLTDPALEFV KFTCAAFALA RDYQIEVGLL KRNVLDLVGV
KEFSDHAVYH NPCDALKLSM VTCKYCDNLR DFDFCRDEDL FPWFCSECDC EYDRVAIEFA
LIQIVHRLER SFAQQDLRCS RCKQIQSDNI SKHCCSGSYQ LTIPKADVRR KLRTIVNVAI
AHNLSRL
>RI
GHRRLYLQLC FRNVSDLLTV RREVVPLALA NGAKLDAVDA YAEVVDPRDA IVDVREFDVP
YYLRVAIDND IRVGLWYTVS FVAGHPQFER VKRADPVVMA YDIETTKAPL KFPDQAIDQV
MMISYMVDGQ GFLITNREIV SEDIDDFEYT PKEGYEGPFT VFNEADEAAT IMRFFQHIQT
AKPTVMATFN GDFFDFPFLC ARAKVHGIDM FVETGFAIDS EDEFKSRTCA HMDCFRWVKR
DSYLPQGSQG LKAVTTAKLG YHPIELDPEL MTPYAMEQPQ ILAQYSVSDA VATYYLYMKY

VHPFIFSLCN IIPLNPDDVL RKGSGTLCET LLMVEAYRGE IIMPNRHEEE HGNMFEGHLL
ASETYVGGHV EALEAGVFRS DISTHFKIEP SAAQLIDELD AALSFCVTNY DDVKSQIQAA
LEEMRDSPLR HDKPLIYHLD VAAMYPNIML SNRLQPDSVV DEAVCAVCDY NRPGKTCDRR
MTWAWRGEYF PAQRDEYNME WFPPKKPDGP KRRYTELAPA EQTALLHKRL GDYSRKVYKK
TKDTKVVNRE SIICQRENPF YIDTVRTFRD RRYEYKGLLK TWKKNLDAVA EVDEAKKMVL
AHKCILNSFY GYVMRKGARW HSMEMAGITC LTGATIIQMA RQLVEQIGRP LELDTDGIWC
MLPGIFPENF KFQLTNGKSV AFSYPCTMLN HLVHAKFTNH QYHDLDKETG TYAVHSENSI
FFELDGPYRA MILPSSKEED KLLKKRYAVF NDDGSLAELK GFEVKRRGEL QLIKIFQSQI
FEKFLLGTTT EECYAAVAEV ADRWLDVLFS RADTLGDDEL VELIAENRSM SKTLAEYGGQ
KSTSISTAKR LAEFLGDQMV KDKGLACKFI ISAKPIGATV TERAVPVAIF SADESVKRTY
LRKWLKDNSL ANFDLRSILD WDYYIERLGS VIQKLITIPA AMQKVSNPVP RIRHPDWLHR
RVAAIDDKFT QHKLTDFFRG RFTLWLSVNN DVVPVKLRIP REFYLNFKSE HLYTRERVIR
TLPRDRPCLH LFVNEMNNPN VDGVYELQTL NRARDHGVEL DQLIRKYVFL YHAFSPSSPI
NVFALFMPNG VKLHIVDPAT RRQSTDYHGN EATALKAVSR DLGVMENKGY TLVLSSVKEL
SHFERSVSKL SKFPVLCMPS NRAGHALDFP WQSNVGKKVL TRYFHLAPWL QRVVSQAAYY
PLFLCDVEFS RRLLKQDMVL WWSAGEKPDL GGLEDDMPAT EELINPEFVS PGCYSNVCLS
VQVRNLAINS ILQAAIVNEL EGSGGTTAFD STQAAVTLGD SSMSPQTFAT LKAMVKTWLL
DKASPSHSTI EHFWRWISSS GAHMFDPSIQ RFIHGLMRKT FIQLLAEFKR LGSNVVYADF
SRIVLVTSKP PGTAHAYATY ITSAVTSHEL FKHVLLRTDQ FYDFLLFMDP ANQGGVVCED
PLALEPPEQL AVFMSWNINK FLPPAVQDRF RRIVRYFVVE MYKIRRSKMK EMDATRSFIA
QRLTRRMLQT VFPLLPGSHL HLTDPALEFV KFTCAAFALA KDYQIEVGLL KRNVLDLVGV
KEFSDQAVYH NPCDALKLSM VTCKYCDNLR DFDFCRDEDL FPWFCSECDC EYDRVAIEFA
LIQIVHRLER SFAQQDLRCS RCKQIQSDNI SKHCCSGSYQ LTIPKADVRR KLRTIVNVAI
AHNLSRL
>PN
GHRRLYLQLR FRNVSDLLAV RRDIVPLAQA NGAKLNAVDA FAEVVDPRDA IIDVREYDVP
YYLRVAIDNE IRVGLWYSVT FTAGQPQMDR VKRADPVVLA YDIETTKAPL KFPDSATDQV
MMISYMIDGQ GFLITNREIV SEDIEDFEYT PKEGLDGPFT VFNEADEAAT IMRFFRHIQE
VRPTVLATFN GDFFDFPFLC ARAKTHGIDM FLEIGFAKDS EDEFKSRTCV HMDCFRWVKR
DSYLPQGSQG LKAVTTAKLG YDPIELDPEL MTPYAIEQPQ TLAQYSVSDA VATYYLYMKY
VHPFIFSLCN IIPLCPDEVL RKGTGTLCET LLMVEAYRGH IIMPNRHEDP HGNMFEGHLL
SSETYVGGHV EALEAGVFRS DIATHFKIVP AAAQLIDELD AALSFCVTNY NEVKSQIQSA
LELMRDNPLR FDKPLIYHLD VAAMYPNIML SNRLQPDSVV DESVCAVCDY NRPGKTCDRR
MTWAWRGEYF PAQRDEYNME GFPPKRTGDP QRRFTDLSPS EQSALLHKRL GDYSRKVYKK
TKETRVVQRE TIICQRENPF YVDTVRRFRD RRYEYKGLHK TWKKKLDAVA EVEEAKKMIL
AHKCILNSFY GYVMRKGARW HSMEMAGITC LTGATIIQLA RQLVEQIGRP LELDTDGIWC
MLPGVFPENF KFELKNGKTI GFSYPCTMLN HLVHAKFTNH QYHDLDPETG EYKVHSENSI
FFELDGPYKA MILPSSKEED KLLKKRYAVF NDDGSLAELK GFEVKRRGEL QLIKIFQSQI
FEKFLLGSTT QECYSAVAEI ADRWLDVLFS KAECLSDEEL VELIAENRSM SKTLAEYAGQ
KSTSISTARR LAEFLGDQMV KDKGLACKFI ISAKPMGAPV TERAVPIAIF SAEESVKRTY
LRKWLKDNSL ANFDLRSILD WEYYIERLGS VIQKLITIPA AMQKVPNPVP RIRHPDWLHR
RVIALDDKFK QHKVTDFFRG RFTMWLCVNA ELIPVTLRIP REFYLNFKTP EYYTRERVIR
TLPRDRQCLH LFTNEINHPN VDGAYELQTL NRLRDDGINL VQLERKYLFL YHAYAPTSPV
HVYALFLPNG LKLHIVDPAP RRQPTDYHSN EIAALKALSR ELGTLESKSY TIVLSSAKDF
IYFSGMVPKL SKFPVLRMPA TKASHALDFP WQSVVAKKMF VRYLAFGSWL HRAISQAAYY
PLFICDIEMA RRLVAQDIVL WWSANEKPDL GGSEDDSPPT EELNNPEFLA PGLYSNVCLS
VQVRNLAIDA VLQSALVNEL EGSSGATAFD LAQPNVTLGD SNLSPHTFAV LKQMVRTWLL
DKACAASITL DHFWRWVSSS SSRMYEPSIQ RFVHGLMRKT FIQLLAEFKR LGSNVVYADF
SRIILVTSKP PGTAHAYATY INSAVTSNEL FKHIYLRTDR FYDFLLYMDP ANNGAVVCED
PLALVPPKQL SISSNWNIKK FLPPAVQDYF KNVVRYFVVQ MAKIKREKVK EMDQTRAFVA
QKLTRKMLHY VFPVLPGSYL HMTDPALEFI KFSCAVFGLA QEYQIELGIL KRNLLELVGV
KEFSDLAIFR NPCDPLKLSM VTCRFCDHIR DFDFCRDEEL LLWYCPECDG EYDRTAIEFS
LIQVLYRLER NFTQQDLKCS RCKQIQSDNM SKHCCSGNYQ LMVSKAEMKR KLRTIINVAI
THGLSRL

>TA
GHKRLYLQLC FRNVSDLLSV RREIVPLATE NGSKLNAVDA YAEVVDPRDC IIDAREFDVP
YYLRVAIDNE IRVGLWYAIT FVAGHPQFDR VKRADPVVMA YDIETTKAPL KFPDQAIDQV
MMISYMIDGQ GFLITNREIV SEDIEDFEYT PKEGLEGPFT IFNEADEAAT IMRFFQHIQE
VKPTVMATFN GDFFDFPFLY ARSKVHGIDM FLETGFTKDS EEEYKSRTCV HMDCFRWVKR
DSYLPQGSQG LKAVTTAKLG YNPIELDPEL MTPYAMEQPQ TLAQYSVSDA VATYYLYMKY
VHPFIFSLCN IIPLCPDEVL RKGTGTLCET LLMVEAFRGH IIMPNRHEDE HGNMFDGHLL
ASETYVGGHV EALEAGVFRS DIATHFKIVP EAAQLIDELD AALTFYVTNY DEVKAQIQSA
LEEMRDNPLR FDKPLIYHLD VAAMYPNIML SNRLQPDSVV DESVCAVCDY NRPGKKCDRR
MTWAWRGEYF PAQRDEYNME MFPPRKPGGP QRRYVDLTQT EQTALLHKRL GDYSRKVYKK
TKETKVVNRE SIICQRENPF YVDTVRRFRD RRYEYKGLHK TWKKTLDAVA EVDDAKKMIL
AHKCILNSFY GYVMRKGARW HSMEMAGITC LTGATIIQLA RQLVEQIGRP LELDTDGIWC
MLPGVFPENF KFKMSNGKTL PISYPCTMLN HLVHAKFTNH QYHDLDPETG EYMVHSENSI
FFELDGPYKA MILPSSKEED KLLKKRYAVF NDDGSLAELK GFEVKRRGEL QLIKIFQSQI
FERFLLGTTT QECYAAVSEI ADRWLDVLFS KADNLSDEEL VELIAENRSM SKTLAEYGGQ
KSTSISTAKR LAEFLGNQMV KDKGLACKFI ISAKPIGAPV TERAVPIAIF SAEESVKRTF
LRKWLKDNSL ANFELRSILD WDYYIERLGS VIQKLITIPA AMQKVPNPVP RIRHPDWLHR
RVAALDDKFK QHKVTDFFRS RYTMWLSVNS DLVPVTVRIP REFYLNFKTA EHYSRERVVR
TLPRDKPCLH LFMNEINNPN VDGAYELQTL NKARDDGVDL SQVDRKYIFV YHAFSPSAPV
NVFAIFLPDG VRLHVVDPAS RRQPTDYHSN EATALKAISR ELGTLENKSF TVVISSAKDL
TYFTSAVPKL GKFPVLRMPS SRAGHALDFP WQANVAKKMF SRYLSFAPWL KRAITQAAYY
PLFLCDIELA RRVVAHDMVL WWSNGERPDL GGLEYDLPPT EELISPEFLT PGLYSNVCLL
IQVRNLAIDA VLQSAVVNEL EGTGGTTAFD SSQPNVSLGE SNLSPQTFSV LKQMLKTWLL
DKASPSSITL DHFWRWVSSS ASRMYEPSIQ RFVHGLMRKT FIQLLAEFKR LGSNVVYADF
SRIILVTSKP PGTAHAYATY ITTAVTSHEL FKHIYLRTDR YYDLLLYMDP ANFGGVVCED
PLALEPPVKL SILSCWNIKN FLPPAMQDHF RSIIRLFISQ MAKIKRDKLR EIDGGKAFIA
QKLTRKLLQI VFPVLPGSYL HLTHPALEFI KFTCAVFGLA QDLQIEIGIL KKNLLELIGV
RQFADDAIFR NPCDPLKLSM VTCKYCDHIR DFDFCRDDEL LPWYCPECEG EYDRAAIEFS
LIQVLHRLER NFAQQDLRCA KCKQIQSDNV SRHCCSGSYQ LTIAKTDVRR KLRTIVNVAK
VHNLGRL
>FM
GHRRLYLQLC FRNVSDLLAV RREIVPLAME NGAKLSAVDA YAEVVDPRDA IIDVREFDVP
YYLRVAIDNE LRVGLWYNVS FTAGQPQFDR VKRPDPVVLA YDIETTKAPL KFPDSATDQV
MMISYMIDGQ GFLITNREIV SEDIDDFEYT PKEGLEGPFT IFNEPDEAAT ITRFFQHFQE
VKPTVVATFN GDFFDFPFLC ARAMVHGIDM FLEIGFSKDS EDEYKSRTCV HMDCFRWVKR
DSYLPQGSQG LKAVTTAKLG YDPIELDPEL MTPYAMEQPQ TLAQYSVSDA VATYYLYMKY
VHPFIFSLCN IIPLNPDEVL RKGTGTLCET LLMVEAYRGH IIMPNRHEDP HGNMFEGHLL
SSETYVGGHV EALEAGVFRS DIPTNFKIVP EAAQLIDELD AALTFCVVNY EQVKGQIQAA
LELMRDKPLR FDKPLIYHLD VAAMYPNIML SNRLQPDSVV DESVCAVCDY NRPGKTCDRR
MTWAWRGEYF PAQRDEYNME QFPAKRPGGP QRRFNELSPA EQSALLHKRL GDYSRKVYKK
TKETRIVQRE TIICQRENPF YVDTVRRFRD RRYEYKGLHK TWKKNLDAVA EVEEAKKMIL
AHKCILNSFY GYVMRKGARW HSMEMAGITC LTGATIIQMA RQLVEQIGRP LELDTDGIWC
MLPEVFPENF KFQLANGKTI GFSYPCTMLN HLVHAKFTNH QYHDLDPETS QYKVHSENSI
FFELDGPYKA MILPSSKEED KLLKKRYAVF NDDGSLAELK GFEVKRRGEL QLIKIFQSQI
FEKFLLGSTT QECYAAVAEI ADRWLDVLYS RAESLSDEEL VELIAENRSM SRTLAEYAGQ
KSTSISTARR LAEFLGDQMV KDKGLACKFV ISAKPIGAPV TERAVPIAIF SAEESVKRTY
LRKWLKDNSL ASFDLRAILD WDYYIERLGS VIQKLITIPA AMQKVPNPVP RIRHPDWLHR
RVVALDDKFK QHKVTDFFRG RFTMWLCVNA ELIPVTLRIP REFYLNFKAP EHYIRDRVIR
TLPRDRPCFH LFTNEINNPN VDGAYELQTL NRARDNGVDL WQMDRKYLFL YHAYTASAPV
HVYALFLPNG VKLHVVDSAT RRQLTDYHSN ETTALKAISR ELGALEKKSF TIVLSSAKDF
TYFSSSVPKL NRFPVLRMPS TKASHILDFP WQSAIAKKMF LRYFSLAPWL QRTIAQAAYY
PLFFCDIELA RRLSAQDMVL WWSAGEKPDL GGFENDMPPA EELTNPEFLS PGLYSKVCLS
IQVGNLAIDS VLQSALVNEL EGSGGTTAFD SAQPNVTLGD SNLSPQMFAI IKQMVRAWLL
DKASPVSVTL DHFWRWVSSS SAMMYEPSVQ RFIHGLMRKT FIQLLAEFKR LGSNVVYADF

SRILLVTSKP PGTAHAYATY ITTAVTSHDL FKHIYLRTER FYDFLLYMDP ANYGAVVCED
PLAIEPPKQI AISSTWNINK FLPPAVQGHF RTVVRYFIAQ MAKIRREKIK EIDESKVFIA
QKLTRRMLQV VFPVLPGSYL HMTEPTLEFI KFSCAVFSLA QDYQIEIGLM KRNLLELVGV
REFSDQALFR NPCDPLKLSM VTCCYCDHIR DFDFCRDDDL LPWYCPECDG EYDRTAIEFA
LIQHLHRLER NFVQQDLRCA RCKQIQSDNV SRHCCSGNYQ LTTSKADVRR KLRTIINVAI
THNLTRL
>OS
GHRRLYLQLC FRNVSDLLSV RREIVPLAVE NGAKLNAVDA YAEVVDPREA IIDAREFDVP
YYLRVAIDNE IRVGLWYTVS FVAGQPQFDR VKRADPVVMA YDIETTKAPL KFPDQAIDQV
MMISYMIDGQ GFLITNREIV SEDIDDFEYT PKEGLEGPFT IFNESDEAAT IMRFFQHIQE
VKPTVIATFN GDFFDFPFLC ARAKVHGIDM FLETGFAKDS EDEFKSRTCV HMDCFRWVKR
DSYLPQGSQG LKAVTTAKLG YDPIELDPEL MTPYAMEQPQ VLAQYSVSDA VATYYLYMKY
VHPFIFSLCN IIPLNPDEVL RKGTGTLCET LLMVEAFRGH IIMPNRHEDP HGNMFEGHLL
SSETYVGGHV EALEAGVFRS DIATHFKIVP EAAQLIKELD AALNFCVVNY EQVKNQIQSA
LELMRDNPMR FDKPLIYHLD VAAMYPNIML SNRLQPDSVV DESVCAVCDY NRPGKKCDRR
MTWAWRGEYF PAQRDEYNME WFPPKRPDGP QRRFMDLSQA EQTALLHKRI GDYSRKVYKK
TRETKVVNRE TIICQRENPF YVDTVRRFRD RRYEYKGLHK MWKKKLDVVT EVEEAKKMIL
AHKCILNSFY GYVMRKGARW HSMEMAGITC LTGATIIQMA RQLVEQIGRP LELDTDGIWC
MLPGIFPENF KFQLANGKSV GFSYPCTMLN HLVHAQFTNN QYHDLNPETG DYKIHSENSI
FFELDGPYKA MILPSSKEED KLLKKRYAVF NDDGSLAELK GFEVKRRGEL QLIKIFQSQI
FEKFLLGTTT QECYAAVAEV ADRWLDVLFS KADSLSDEEL VELIAEKRSM SKTLAEYAGQ
KSTSISTARR LAEFLGDQMV KDKGLACKFI ISAKPIGAPV TERAVPVAIF SAEESIKRTY
LRKWLKDNSL ANFELRSILD WEYYIERLGS VIQKLITIPA AMQKVANPVP RIRHPDWLHR
RVVALDDKFK QHKVTDFFRG RFTMWLCVNS DLIPVTLRIP REFYLNFKVP EHYTRDRVIR
TLPRDRQCLY LFTNEINNPN VDGAYELQTL NRARDDGVDL WQIDRNFIFL YHAFTPSAHV
HVYALFLPSG VKLHIVDPAT RRQPSDYHSN EATALKAISR ELGMLENKSF TILLSSAKDF
VYFSSMVPKI NKFPVLRMPS TKVSHALDFP WQDVVAKKML LRYFSLAPWL RRTITQAAYY
SLFFCDIELA RRLVAQDMVL WWSAGERPDL GGFEDDMPPT EELVNPEFLT TGLYSNVCLS
VQVRNLAVDA VLQSALVNEL EGSGGTTAFD SAQPNVTLGD SSISPQTFTT LKQMVRTWLL
DKASSSSLTL DHFWRWVSSS AAQMYEPSIQ RFVHGLMRKT FIQLLAEFKR LGSNVVYADF
SRIVLVTSKP PGTAHAYATY IITAVTSHEL FKHVYLRTDQ FYDFLIYMDP ANHGAVVCED
PLALEPPKQL AVLSNWNIKK FLPPAVQDHF QNMMRYFVTH MVKIRRQKLQ EMDESRTFIA
QKLTRKMLHV VFPVLPGSYL HMSEPALEFI KFTCAVFELA QDYQIEIGLL RRNLLELVGV
REFSDQAIFR NPCDPLKLSM VTCRYCDYIR DFDFCRDNEL LPWYCPECQG EYDKTAIEFA
LIQDLHRLER NFAQQDIRCS RCKQIQSDNV SRHCCSGRYQ LVIGKADVRR KLRTMINVAI
THNLSRL

06-ARID domain

>AS
APAAGKLDFT AIKTSSPRPF ALEDCPVFYP SLEEFKDPMK YMQVVGPKAR DYGICKIVPP
VGWKMPFVTD TEFRFTTRLQ RLNSIEASSR AKLTFLEQLY RFHSSQGNTN IAVPTVNYRR
LDLWLLRKEV QKLGGYDAVK NKKWGELAQI MGYNAQGVAA QLKASYSKVI LPFENYSDHV
RSALCDGCDA GYHTFCLDPP LSAIPRGQWF CQKCLFGTGD YGFDEGEEHT LQSFMMRDLT
FRRLWFASHP PYSESDVERE FWRLVQTPFE TVEVEYGADV HSTTHGGMPT PETHPRNPYS
RDPWNVNNVP ILPESLLRYI KSDISGMTVP WTYVGMIFST FCWHNEDHYT YSMNYMHWGE
TKTWYGIPGA DALKFEAAIR KEAPDLFDAQ PDLLYQLVTL MNPARLRDAG VRVYACNQRA
GEFVVTFPRA YHAGFNHGLN FNEAVNFALP DWLPFGLECV KRYQEHRKLP VFSHDELLIT
VTQHSHSIKT AVWVLDSLRE MIDRETAQRR VVRPGLQETL EEYDPENQYQ CHVCKAFCYL
AQITCGCVAC LEHAQLLCGC RVLRKRFSDE QLEDIYSKIM ERASIPTDWQ AKLQRTLQDS
ARPNLRVLRA LLAEGERVSF HLPELLALRK CVQRANEWVE VATSFTTRKQ ANKRDRPERG
LKDVYALLEE VELLGFDCPE IETLRRITQS AEEFKKKARL TILETQITIG AGLNMQLEEL
DEMRRIFMRC KLVRDLLDDI RSLLVRAKAC GLTEESKLMK TLLEKQRIGE DWVQKVTALL
NLEQKPLPEI PIDPTVLDRL KAVRAKARDL ERQARIMLAP ELPRPSDALK LVARAEKDFM
IPVIDELKRS AEFANDLEEK CDAVLSKRFR YGTPFGLFRK WVAYGHTHLS FQLHNFEKLD

RQLIAHSQWI ERLPWYCHSD AILRDVRDCT NPDEDHPPSD EFISCICDRQ VRPPPPGEAS
DAVQCDHCFA RFHGACAANG GSCPFCDHHH WNGSIHKERN WHFCFLPTML VTAPDITKFY
STAWKELEYI ISRVDRLCVS IGSFLSFASN QRPEFLPQVR HYMRKLFRIQ FAISPNPDVS
YGLDLAGLHR ILAGKMHKKR RRVKIVFQQD VGPEPADGTQ CLCKGTVCSR WHHETCVFFC
PACSIKRNRA YPYGEVRVRQ APAEQLRRVQ LPPNKNALVL DLIRYSF
>EG
PAPAAKLDFS SIKTESPRPF GLEDCPAFYP SMEEFKDPMK YLQTVAPKAQ EFGICKIVPP
VGWKMPFVTD TEFRFTTRLQ RLNSIEASSR AKLTFLEQLY RFHSSQGNSS IAVPTVNHKR
LDLWLLRKEV QKLGGYEAVK NKKWAELGQL MGYQSPGLAS QLKNSYMKII LPFENYSDHV
RSALCDGCDA GYHIFCLDPP LSAIPRGQWF CSKCLFGTGD FGFDEGEEHT LQSFMTRDRT
FRRHWFQAHP PYSEGDVESE FWRLVETPFE TVEIEYGADV HSTTHGGMPT LETHPRDPYA
RDPWNVNNIP ILQDSLLRYI KSDISGMTVP WTYVGMIFST FCWHNEDHYT YSINYMHWGE
TKTWYGIPGA DALKFEAAIK KEAPDLFEAQ PDLLYQLVTL MNPSRLREEG VRVYACNQRA
GEFVVTFPRA YHAGFNHGFN FNEAVNFALP NWLPFGLECV KRYQEHSKLP VFSHDELLIT
ITQHSHSIKT AVWVLDSLRE MIDRETAQRR AVLPGIQETL EEYDPENQYQ CHVCKAFCYL
AQITCGCVAC LEHAKLLCNC RVLRKRFSDE QLEDVFSKVV ERAAIPSDWQ AKLQRTLQET
SRPNLRVLRA LLAEGERVSF HLPELLALRK CVQRANEWVE VATSFTTRKQ ANKRERPERG
LKDVYALLDE VELLGFDCPE IEQLRRIAQT AEEFRKKARL TILETQITLG QGLNMHLDEL
DEMKRIFMRC RLVRDLLDDV RQLLNRAKHC GLADDSKVVL GLLEKQRAGD EWCKKVTDLL
ALPKKPLVEI PIEPTLLSNL TASRNSARGY ERQAKFMLAP ELPRPKDALD LVHSAEKAFD
IPIIEDLRRS AEFAQDLEEK CEAILLKRFK HGSPFTVFRK WVAYAHAHLS FRLANFEKLD
RQLIAHSQWI ERLPWYCHSD AILRDVRDCT NPEDETPPQD EFISCICERQ VRPPPPGEVS
DAVQCDHCYA RFHGACATNG GSCPFCDHNH WNGSIHKDRN WHFCFLPTML VTAPDITKFY
SVSWKELEYI VARVDRLCVS IGSFLSFASN QRLELIPQVR QYMRKLFRIQ FAVSPNPEVS
YGLDLAGLHR MLASKPPKKK RRVRLVFQPE VAPDPADGTR CVCNGAGCSQ WYHETCVWFC
PICAVKKTRV YPYAEIRVRQ TPSEQLRRVS LAPNKNAIIL ELHRYSV
>RM
IHVSPKLDMS SVKTASPRPF GLEDCPVYYP TADEFKNPMV YIRSISEHAR KYGICKIVPP
EGWKMPFATD TEFRFKTRLQ RLNSIEATSR AKINFLEALY QFHKQQGNPR VTVPTINHTP
LDLWLLRKEV QKLGGFETVK EKKWVDIGRQ LGYTGPGLST QIKNSYTRVI LPFEHYQSEG
ARNLCDGCDC GFHTFCLTPA LSTIPKGQWF CHTCLFGTGD FGFDEGEDHS LSTFQARDLA
FRKMWFQSHP PVTEDDVEEE FWRLVEAQDD TVEIEYGADV HSTTHGGMPT LETYPLDPYS
KDPWNLNNLP ILSDSLLRYI KSEISGMTVP WTYVGMVFST FCWHNEDHYT YSANYMHWGE
TKTWYGIPGE DAEKFETAIR QEAPDLFELQ PDLLFQLVTL MKPDRLTSAG VRVYACNQRA
GEFVLTFPKA YHAGFNHGLN FNEAVNFALP DWLPFGRDCV RRYQEHKKLP VFSHDELLIT
ISQQSNTIHT ATWLNESFKE MTDRELSGRR RVRLGVKEVL EERDPEEQYQ CNYCKAFCYL
SQIYCTCVVC LEHIDYLCEC RTLRLRFSDE ELSNTQQTIE SSAAIPDIWG DKFARLLEES
PRPSLRALRA LVAEADRVNY PFQHLGSLRK CVEAANEWVR ETNVYTTRRI SRRGDGTRPT
LEGVRDLLRR VEDLGFDTPE IALLQNIATQ AEAAKAKAKL LLDIRELLSG YTLSLQLDEL
VELEEFCNKM DITIEILMAY LDLPVRARAC NYDMTSPVFE KMQKLSKVVD RNNDAVREVL
SRPIKLLEEL LMDPQLEMKL DQVYNQAVAY QKQAEAWLNP RMPEAQEAMK LVQKAEKEFQ
IDNIAELKQL AEHAYDLEER CERVLRNRYL PQPMLQVVTK WREIADKQLR FSLPNCDALL
KQIQLHQQWL TKLPWWCHGD EILEDVVDYT KPEDDSPPHD EFFTCICFEP VRKPPPNVSS
DAVQCDHCFA RFHGRCAKNG GSCPFCDPNH WNGNIHRERS WHSCYLPTVL NNAPDVTRYH
SSEWKQLKVI VEHIDRFCTV TGHFLKQAQN QRPDLIPQVR HFMRKLYKIG FAVSPNPEVS
FGLDLAGLHR ILASRRIKKR RRPRFVFGQD LDKDWIDGTR CICRGRSCNR RYHTGCVFLC
PLCCLRKGKA YRYADVRVKS FSDKPIYVRL NPPTTSTIFV DMIHFMP
>SC
IPISPTLDLN SVKTAGQRLF GLQDCPEYHP TAEQFQDPMA YIQSIAEEAK QFGICKVVPP
PDWKMPFVTD TEFRFKTRLQ RLNSIEASAR AKINFLEKLY RFHKQQGHPR VSVPTINNKA
LDLWTLRKEV DKLGGYEAVK AKQWADLGRV LGYRGPGLST QIKNSYARII LPYEHYMARV
KNSLCDGCDC GFHTFCLDPP LEAIPKEQWF CFACLSGTGD YGFDEGEEHC LSSFQTRDNE
FRRMWFEGHP PVPEYYLEEE FWRLVQSTQE TVEVEYGADV HSATHGAMPT LETHPLDPYS
KDQWNLNNIP IVADSLLRYI KSDISGMTVP WTYVGMTFST FCWHNEDHYT YSINFMHWGE

TKTWYGIPGD DAERFEAAMK REAPDLFEAQ PDLLFQLVTL MNPKHVRDAG VRVYACNQRA
GEFVLTFPKS YHAGFNHGLN FNEAVNFALP DWLSYDRDCV ERYRRHRKMP VFSHDELLVT
ITQQAQTVKA ATWLLDSLKE MTDREMADRQ SVRRGIKERV EAEDPEEQYQ CAVCKVFCYL
SQVVCPCVVC AEHVDALCQK LTLRLRFSDH DLYSTLATVQ ERSSVPAQWR QKYRSLIAET
ARPPLKTLKS ILAEGDKMGC AVPELLTLRK CVIRAGEWLD EATHFLQRKQ NRKREKLEKG
LDDLLQLLKE VEDMGFDTPE IAALRVLAER AATLQQRALD ILAKALSADA SSVNVALEEV
QAVERIVERE ELEKELMEQV RGLLSRARAC GLTNENKYVE ILEAKDKEGG DWEEKAQAIL
HAEVKTVADL PVVPNVLARL RTLYDRAKDY EKQVGIWLGA DKPTLADVHR FIDKVEKEFR
IDMVEDLKRA VKIADDLESR CKQVINHKYQ PDDMFETISQ WTTYARNHLS FQLPFFQKLD
SQLEQHRAWL REIPWYCHGS DLLKDVMDAT RPEDDFAPAD EYFTCICDLP VRPPAEGEVN
TAVQCDHCYA RFHAECANNG GSCPFCDHHH WNGQIPRART WHFCYLPSIL LKAPALTKQY
SREWRELEVI VHRVDRLSAL IGQFLAYCSN QRPEHIPQVR HYMRKLWRIQ FVVSPNPDVS
FGLDLAGLHR LLAGRKTKRK RRPKFLFGQD QDRDWVDGTR CICRALACRR KYHGPCVFLC
PLCCVRKNRR YAWGDVRVKT HAKEQIYVKL AEPRMSTLIL ELIKYIP
>SN
VTVPPDLDMK SLKMESPRPF GLEDCPTFRP TPEQFKDPMA YINSISATAK DFGICKVVPP
AGWKMPFVTD TKFRFKTRVQ RLNQIEAAAR AKVNFLEQLY RYHKTQGHSR MSIPTINHKP
VDLWLLRKEV TKLGGYEAVR NKKWGDLGRL LGYTGPGLSA QLKNAYTRVI LPFEQFSEQV
RNSLCDGCDC GFHIFCLDPP LTAIPRGQWF CTTCLVGTND FGFDEGEEHT LASFHARDLA
FRKLWFERHP PVSESDVERE FWRLVQTPNE TVEIEYGADV HSTTHGAMPS LETHPTEPYS
RDGWNLNNMP INPDSLLRYI KSDISGMTVP WTYVGMIFST FCWHNEDHYT YSINFMHWGE
TKTWYGIPGD DAEKFEAAIR QEAPDLFEAQ PDLLFQLVTL MNPGRLREAG VRVYGCNQRA
GEFVITLPKA YHCGFNHGFN FNEAVNFALP DWLPHGRACV QRYQEHRKLP VFSHDELLIT
ITQYSQSIKT AVWLYDSLKE MVDREFRRRE EIRPGITEVH DDSDTEEQSQ CSVCNVFCYL
SQISCQCGVC LDHSDELCSC RTLHLRFMDD ELTDILSKVE ERAMLPKAWQ AKLQKTLGES
SRPLLRLLRA LLAEGERINY RLPELPSLRK FVQKANEWVE SVNNMTVRKR KRPREKPERR
LSDVYQLLDE VDGLGFDCPE IALLRGLASQ AEDLKTRSQR LLCDTVISEA SSMNIYLEEV
QDLERIVMRH KLLQDMLDEI RNLLARARAC ELPADNQYMK VLLAKQNAGD DWDQKAARVL
AQPLKTIEEL PVDPAVLDKL RAIRAKAKDF ERQALQWLHP DLPKVQEAIR LVTRAEKEFS
IPAIRELRRT ADFAADLEQR SEAVLKNRYQ NSQIFDTMRK WGEYAREHLQ FQLPNFTKLE
AQLESHELWM ERLPWYCHGQ PVLDDVLAST KPDDDTPPQD DFQTCICFEP VRPPPPGVTS
DAVQCDHCYA RFHGPCVLKG GSCPFCDHHH WNGTLHKERT YHYCFLPTIL LSAPEITKNY
SPAWRQLETI VSRVDRLSQL ISAFLAFAAN HRPEFIPQVR HYMRKLFRIG FAVSPNPEVS
YGLDLAGLHR ILASQRPKKR RRPKFVFGSD VDKDWEDGTR CVCGGKKCHR IYHEPCVLQC
PLCSLKKLKP YPTAEIRVKK FSKELLRVQL PPPTAPTISI ELIRFIP
>SS
VSAPKVLDMD TVKQRRIRQF GLEDCPTFYP TEEEFSDPLS YIRSISDRAQ QYGICKIVPP
EDWNMPFVTD TEFRFKTRVQ RLNSIEAASR AKLNFLEQLY RYHKQQGNSR VSIPTINHKP
LDLWLLRKEV QNLGGYDVVK SKRWGELGRA LGYSGPGLSA QLKNAYTRII LPYENFYNHV
RNSLCDGCDC GFHIYCLDPP LQSIPKGQWY CHTCLFGTQD FGFDEGEEHS LASFQARDLE
FRRRWFATHP PISENNVEEE FWRLVQSPFE TVEIEYGADV HSTTHGAMPT LENQPLDPYS
RDGWNLNNIP ILSDSLLRYI KSDISGMTVP WTYVGMVFST FCWHNEDHYT SSINYMHWGE
TKTWYGIPGD DAEKFEAAIK KEAPDLFEAQ PDLLFQLVTL MNPARVREAG VRVYACNQRA
GEFVITFPKA YHAGFNHGFN FNEAVNFALP EWLPYGRSCV QRYQEHRKLP VFSHDELLIT
ITQQSTSIKT AMWLYPNLQE MHQREMQRRA ILRPNMEEVL VNHDSEEQYQ CAICKVFCYL
SQVTCSCVVC LDHADNLCND RVLRKRFSDE YLTDILQKVA ERAAVPIAWN NKLHKLLEES
PKPQLRSLRA LLAEGERINF GLAELPMLRK CVLKGNEWVD SANVFLARKP NRKRDRPERG
LDDLYTVLEE VTHLGFDTPE ITALRTMAGQ AEDIKAKART LLCDTLLAQG SSLNIYLEEM
AKVERISMRS KLLKELLEEV RALVSRARAC DLQEDNQSMK SLEDKLRVGD EWESKATAIL
ARPQKTIEEL PVDATTHDHL TNVLARAKEF EEQAKAWARP GLAKVTDALR LIARSEKEFS
IPAVDELKET ANTAYELEQR CENALKNNYQ HETPFDAMRK WQSYAQDKLA YSLPNVEKMA
RQLELHDQWL KRLPWYCHGQ QVMEDVLECT KPEDDNPPND EYFTCICTVP VRPPPPGQAS
DAVQCDHCYA RFHGACAANG GSCPFCDHHH WNGTIHKERS YHSCFLPTIL LGAPEITKYY
SLAWKHLNII VDRVERLTKV VGNFLQFAAN QRPEFIPRVR HYMRKLFKIQ FAVSPNPEVS

YGLDLAGLHR ILASQRLKKR RRPKFLLAPD ICKEGKDGTR CVCRGLKCKN TYHVTCVYLC
PLCCIRKGRN YPYADLRVKT FSRDPIKFRL PPSTGLVIPV TLLRFFP
>SH
IPVNSRLDFS KIRTQSPRPF GLEDCPVFEP TEEEFRDPMG YVKKIENQGR RYGMVKIIPP
KGWKMPFVTD TEFRFTTRLQ RLNSIEASSR AKINFLEQLY RFHQQQGNTR VSVPTINNKP
MDLWVLRKEV QDMGGYEVVK QKKWADIGRL LGYTGPGLST QLKNSYTRVI LPYEHFCERV
KNSLCDGCDC GFHMFCLDPP LLSIPKGQWF CHTCLFGTGD FGFDEGEEHS LSSFQARDLA
FRKLWFLGHP PVTEDDVERE FWRLVKSSNE TVEIEYGADV HSTTHGAMPT LETHPLDPYS
KDPWNLNNMP ILQESLLRYI KSDISGMTVP WTYVGMIFST FCWHNEDHYT YSINFMHWGE
TKTWYGIPGE DAEKFEAAIR REAPDLFETQ PDLLFQLVTL MNPTSLREAG VRVYACNQRA
GEFVITYPKA YHAGFNHGLN FNEAVNFALP DWLPYGRQCV QRYREHRKLP VFSHDELLIT
ITQQSQSIAT AIWLNPSLME MTDRELANRR KARLRLRESL EREDTEEQYQ CNICKSFCYL
SAVTCTCVVC VDHVSSLCKC RSLRLRFSDN ELMETQEKIQ ERAQIPDQWK AKLSRLLLEN
ARPHLKALKA LLAEGDKIQY DMKGLEDLRR CVVKAGEWVQ MANEILVRKP NRKRDRPDKS
LDDLYELLRQ VDDLGFDCPE IGMLRTLAGQ AEEVRGKAKA LLCEKLLLDA SSLNVSLDEL
IDVEKMVARE QLIKELLEEV SGYLTRARIC GLSSENRHIK DLEARQRAGQ TWDERARHVL
SQPFKTIEEL PIDPAVLSKI ANAREKAKDY ERQATTWLNP EKPKVQDVMR LVTRAEKDFN
IPAVKELKRM ADLAGDCENK CDELLRDRFE HDDVFEIIDK FVNYAKQHLT FAMPKFETID
EQLTQHYRWL ETMPWKKDVD LLMKDIMDNT RPEEDLPPED EFFTCICLDA VRPPEQGQVS
DAVQCDHCSA RFHAACAKSG GSCPFCDHSH WNGTIRKERS FQFAVLPVIL NEAPVITRFY
SPEWKQLEIM VHRISRLSTV IAQFLSFSSN QKRDYIGQVR HYMRKLYKLQ FNISPREDLT
FGLDLAGLHR IIASQRTKKR RRPKFMFGQD VDADWLDKTR CICRGRTCSK MYHGGCVFIC
PLCCLRKNRR YEYAELRVKL FSKEMIYKKL PPPYTQTLFV ELIRFTP
>PN
DLPPNTLDMS NLKKMKTRPF GLEECPTFFP TTSEFKDPLT YIRSISAKGQ EHGIIKIVPP
EGWEMPFVID SEYRFKTRAM RLNSIEAASR AKINFLEALY RFHRQEGNPR VTVPTINHRP
VDLWLLRKEV QKLGGFEAVK GKKWSELGRL LGYSGPGLST QLRNSYIRVI LPYEHYSKNV
RGSLCDGCDC GFHIFCLTPP LSSIPKGQWF CHTCLTGTGD YGFDEGDEHS LASFQARDLA
FRKMWFEKHP PVTEMDVENE FWRLVQSSSE TVEIEYGADV HSTTHGGMPT LETHPLDPYS
KDPWNLNNIP ILQDSLLRYI KSEISGMTVP WTYVGMIFST FCWHNEDHYT HSINYMHWGE
TKTWYGIPGE DAEKFEAAIK KEAPDLFETQ PDLLFQLVTL MSPAKLKESG VRVYACNQRA
GEFVITFPKA YHAGFNHGFN FNEAVNFALP DWLPLGRECV KRYREHKKLP VFSHDELLMT
ISQQSQAIKT AIWLNESFKE MTDDELKGRQ DLRLGVREVL DQQDPEDTSQ CIFCKCFCYL
SEVVCSCVSC WDHSKYLCNC KELRLRYSDE EISNTQTAIA SRAMVPVNWR NKFNKLLADS
PKPQLRALRA LMAEGERINF PLEELSVLKA CVNRANKWVT DANSFTTRNN SRKRDRQEKS
LSDLYKVLDE VDTLGFDCNE VEALRSLTVQ AEDLRVKAKA LLCEALLAHG GSTNVYLEEL
VQIDNLITQH KLTAEILQEI RQFLSRAKAC DLPSDNKFTV LLENQLKNGT DLDGKMAAIL
SREKKTIAEL PFDPAILAQI RAFKERAERL EKQANEWLAP ELPNVEDVLK VVEAAEKEFD
LPSISNLKRT AEFASDLEKR CSAVLSKRYM HEPCFDAMKK WRTYAREHLT FRLPNFDKLC
VEIDREDDWA RGLPWFSEEK KIFEDVIRYT LPMDDQPPKD EFFTCICFSP VRPPPPDQVS
DAVQCDHCFA RFHGKCASNG GSCPFCDPNH WNGTLNKTRN FHFCYLPTIL KNAPEISRKY
SVHYDRLKFI VENVERLCSV IGTYLSHLSN QRPHELPQIR HYLRKLFVLK FAVSPNPEVS
FGLDLSGLHR ILGGKKLKKR RRPRFVFGQD IDQDWVDGTR CICRGQTCQR KYHAACVFTC
PLCCLRKLKS YRWADVRVRT YSRDIIRIKL PAPTQATIFV DLVNFTP
>SP
ITVTSALDLN TVKTSSPRPF GLSDCPTFYP TEEQFKDPMA FIRSIGDQGR SSGICKIVPP
EGWKMPFVTD TEFRFKTRLQ RLNSIEASSR AKINFLEALY RYHKQQGNPR VTVPTINHKP
LDLWLLRKEV NKLGGFEVVK GKKWVDIGRL LGYGGPGLST QLRNSYIRVI LPFEHFSDGI
RKSLCDGCDS GFHMFCLVPP LSAIPKGQWF CHTCLFGTGD FGFDEGEEHR LSSFQARDLA
FRKMWFQAHP PVSETDVENE FWRLVQSPYE TVEIEYGADV HSTTHGAMPT LETHPLDPYS
KDPWNLNNIP ILSESLLRYI KSEISGMTVP WTYVGMIFST FCWHNEDHYT YSINYMHWGE
TKTWYGIPGD DAGKFEAAIR KEAPDLFETQ PDLLFQLVTL MNPARLTSAG VRVFGCNQRA
GEFVVTFPKA YHAGFNHGFN FNEAVNFALP DWLPYGLDCV RRYQEHKKHP VFSHDELLIT
ITQQSNTIQT ALWIVDSLNE MVNRELKGRD AARRGVKEEA DNASRDEQFQ CSFCKAYCYL

SRVVCTCAVC LEHINYLCEC AVIRTRFTDP ELHNIQSTIA TRAAMPENWR NKLGKLLMES
ATPQLRTMRA LVAEADRINY PLKELPVLKR CVAKANEWVD SANTFITRKQ SRKRERPEKS
LEDLYALLKE VERLGFESPE IGQLRVLGSQ VEECKRNAAE LLCETLLAHG SSLNVYVKEL
VDVENIVLQE QLLKEMLDEV RQFLMRARAL NLPGDNKHLK VLEAKLQAGN DWDDRAARVL
SQPVKTIEDL PVDPGVLNRL SAARTKALEY ERQAKAWLNP ELPKVSEALR FAQKAEHEFK
IPAIEDLQRT ADFALDLEER CEQVLKGRYQ HGPVFDAIEK WQKYAQEHLS FALPNFDKLN
KQLESHAAWM TKLPWYNQGS EVLTDVLEYT KPEDDGPPDD EFFTCICFLP VRPPPPGQTS
DAVMCDHCFA RFHGRCAANG GSCPFCDPNH WNGNIHKERS WHFYYLPLIM EAAPDISKHY
SEGYRDLQVI THHVERLSAV IKHFLAFASN HRSEYVPQIR HYMRKLYKLQ FAIGSNPENS
FGLDLAGLHR MLSVMRVRKR RRPKFVFGQD VDKDWRDGTR CICRGQTCSR RYHAACVFMC
PLCCLRKCKP FPTADMRVKT FSRELIRVRL DAPKTSTIFI DLVQFTP
>TA
IPISPTLNLD SVKTSSPRPF DLEDCPTYYP SPEEFKDPLG YIRSITPQAK NYGIAKIVPP
IGWNMPFVTD TEFRFKTRIQ RLNSIEASSR SKINFLESLY RFHKQRGRPN VAVPTINHQP
IDLWSLRNEV DNLGGLEAVQ TKKWTDVGRL LGYRGPGLST QLRNSYVRVI RPYEEFLAEV
RKTLCDGCDC GFHIFCLNPP LSMIPKGQWF CDTCVIGTGD FGFDEGEEHS LSSFQARDKA
FRQMWFQSHP PMSEYDVEKE FWRLVQSPYE TVEIEYGADV HSTTHGAMPI LEMHPLNEYS
RDPWNLNNIP ILPDSLLRYI KSEINGMTVP WTYVGMVFST FCWHNEDHFT YSINYMHWGE
TKTWYGIPGE DAEKFEAAIK KEAPELFESQ PDLLFQLVTL MSPARLTQSG VRVFACNQRA
GEFVVTFPKA YHAGFNHGFN FNEAVNFALP DWLPYGRECV RRYQEHRKLP VFSHEELLIT
ISQQSHSIKT AIWLTESFGE MVDKELANRK AITLGISEVL EERDPEDQYQ CIYCKAFCYL
SQIICPCPVC LQHNEFLCDC RTLRLRFSNE DLRNMKMTIA NRAQIPTAWR SKLMKLLKES
PKPQLRAMRA LVAEADRINY PLPELVWLRK CVARANQWVD SANSFITRKQ SRKRDRPEKT
LEELFAHLHE VEDLGFDCPE IGQLRTLASS AKDFKVNAGV LLCETLLAHG SSLNVHLEEL
YEVENIVLQE QLMKELLEET RQFLSRAKAC DLPDDNKYMK LLEDKLRAGN DWDERAANIL
KQPIRTIEEL PVDPAVLSRI AATRARALEL DRQAKVWLSP ELPRVQDALR LVQRAEKEFS
IPSIDDLKRT ADFASDLEER CEAVLKCRYQ HGSIFDGIEK WVAYAQEHLT FYLPNFDKLK
KQLELHRLWI KKLPWYCHGH EILNDVLEYT KPEDDNPPED EFFTCICFDP VRPPPQGQIS
DAVQCDHCFA RFHGKCAANG GSCPFCDPNH WNGNIHQERS WHFCYLPTVL QCAPDITKNY
SEAWKELENI VQHIERLCGV IGHFLSFASN QRQEYIPQVR HYMRKLYKIQ FAVSPNPEVS
FGLDLAGLHR ILASRRLKKR RRPRFVFGQD VDKDWIDGTR CICRGQTCNR KYHAACVYAC
PLCSLRKVKI YRWADIRVRN FSRELIRIKL NPPMTPTIFI DLIQFTP
>FM
ISVSSTLDMS TVKTTSPRLF ELEDCPTFYP SPEEFKDPMS YIRSISPRGQ EYGIIKIVPP
IGWKMPFVTD TEYRFKTRAM RLNSIEASSR AKINFLEALY RFHRQQGNPR VTVPTINYKP
LDLWLLRKEV QKLGGFEKVK EKKWSEVGRL LGYTGPGLST QLRNSYIRVI LPYEVYSDGI
RSSLCDGCDC GFHMFCLVPP LTSVPKGQWF CHTCLFGTGD YGFDEGEEHS LSSFQARDQA
FRKMWFETHP PISEPDVENE FWRLVQSPYE TVEIEYGADV HSTTHGAMPT LETHPLDPYS
KDPWNLNNIP ILQDSLLRYI KSEISGMTVP WTYVGMVFST FCWHNEDHYT HSINYMHWGE
TKTWYGIPAE DAEKFEAAIK KEAPDLFETQ PDLLFQLVTL MSPARLKESG VHVYACDQRA
GEFVITFPKA YHAGFNHGLN FNEAVNFALP DWLPLGRECV KRYQSHKKLP VFSQDELLIT
VTQQSHSIRT AIWLNDSFKE MTETEIKNRK AVRLGVPETL IEHDPEDQYQ CAYCKAFCYL
SQVMCPCVVC LEDVKYLCDC QLLRLRFSDE ELLNIQSTVS SRAAIPENWH KKLMKLLNDS
PKPQLRALRA LVAEADRINY PMKEVASLRR CVTRANEWVE AANSFITRKQ SRKRDRPEKT
LEELYSVLRD VENLGFDCPE IGLLRNLASQ AEEFKTKAKA LLCETLLAHG TSLNVYLEEF
YKIENYVLQD RLVKELLDEI REFLNRAKAC ELPSGNKYMI LLEERLKAGT DWDERAAGVL
NQPIKTIEEL PVDPSVLKRI QTTRSRALEY EKQAKEWLSP QLPTVQEALR LVQKAEKEFN
IQAIQDLKRT VDFAYDLEER CEAVLKNRYE HGSCFDAMNR WRTYAREHLT FRLPSFDKLN
VEIEKHEQWQ KKLPWYCHAD EILRDVVDYT KPEDDEPPHD EFFTCICFEP VRPPPPGVVS
DAVQCDHCYA RFHGRCAVNG GSCPFCDPNH WNGTIHSDRS YHFCYLPTVL HNAPEISKNY
SEHWQELKTI VEHIERLCNV VGNFLSIASY QRAEYIPQVR HYLRKLYKIK FAVSPNPEVS
FGLDLAGLHR ILANRRMKKR RRPKFVFGQD VDRDWVDGTR CICRGQTCLR KYHNACVFYC
PLCCLRKGKM YRWADIRVRT FSRNLIKIKL APPVTPTIFI DLVQFYP
>OS

ISVSSTLDMS TVKTSSPRPF ELEDCPTFYP SPEEFKDPMS YIRSISPRAQ NYGIVKVVPP
IGWKMPFVTD TEYRFKTRAM RLNSIEASSR AKINFLEALY RFHRQQGNPR VTVPTINHKP
LDLWSLRKEV QKLGGFEAVK GKKWADLGRL LGYGGPGLST QLRNSYIRVI LPYEHYSERV
RGSLCDGCDC GFHTFCLVPP LSTIPKGQWF CHTCLFGTGD FGFDEGEEHS LSSFQARDQA
FRKMWFQTHP PVTELDVENE FWRLVQSPFE TVEIEYGADV HSTTHGGMPT LETHPLDPYS
RDAWNLNNIP ILPDSLLRYI KSEISGMTVP WTYVGMIFST FCWHNEDHYT HSINYMHWGE
TKTWYGIPGE DAAKFEAAIK KEAPELFETQ PDLLFQLVTL MSPARLKESG VRIYACDQRA
GEFVVTFPKA YHAGFNHGLN FNEAVNLALP EWLPLGRECV KRYQEHKKLP VFSQDELLIT
ITQQSHSIRT AIWLNDSFKE MTDQEMEGRR AVRLGVREIL EERDAEEQYQ CVYCKAFCYL
SQVICPCVVC LEHVKYLCDC RVLRLRFSDE ELLNIQSTIA ARAAIPENWH SKLTKLLNDS
AKPQLRALRA LVAEADRINY PLKELSALRK CVTRANDWVD VANSFTTRKQ SRKRERPEKT
LDELYAVLKE VDNLGFDSNE IALLRNLATQ AEDFKARASA LLCDALLAHG SSLNVNLEEL
YKVENTVLQD HLIKELLDEI RQYLARAKAC DLPPDNKYMK VLEAKLQAGN DWDERAASVL
TQPVKTIDEL PVDPAVLNRI KATRTRALEF ERQAKGWLSP ELPSVQDALR LVQRAEKEFS
IPAIHDLKRT ADFAYDLEER CEAVLKDRYQ HGSVFDAMDK WRTYAREHLT FRLPSFDKLN
AELELHEQWV KKLPWYCHGR EILNDVLDYT KAEDDTPPDD EFFTCICFDP VRPPPPGKTS
DAVQCDHCFA RFHGKCAVNG GSCPFCDPNH WNGNIHNERN YHFCYLPTVL HNASEITKSY
SEYWEQLKLI VQHVERLCAV IGHFLSFASN QRAEYIPQVR HYMRKLYKIQ FAVSPNPEVS
FGLDLAGLHR ILASRRMKKR RRPRFLFGQD IDRQWVDGTK CICRGQTCQR KYHTACVFTC
PLCCLRKAKP YRWADIRVRQ FSRDIIRIKL TPPVTPTIFI DLIQFTP
>RF
IPVSSKLDMS SVKTSSPRPF DLEDCPTFYP THEEFRDPMA YIKSISDRAR NSGICKVVPP
MGWKMPFVTD TEFRFKTRVQ RLNSIEASSR AKLNFLEALY RFHKQQGNPR VTVPTINHKP
LDLWLLRKEV HKLGGYEAVK GKKWADLGRL LGYGGPGLSM QLRNSYVRVI LPYEHFSDRV
RNSLCDGCDC GFHTFCLDPP LSVIPKGQWF CDTCLSVTVD FGFDEGEEHS LSRFQARDLA
FRKMWFESHP PVSEFDVENE FWRLVQSPHE TVEIEYGADV HSTTHGAMPT LETHPLDPYS
KDGWNLNNIP IVSDSLLRFI KSDISGMTVP WTYVGMVFST FCWHNEDHYT YSINFMHWGE
TKTWYGIPGE DAEKFEAAIR REAPDLFESQ PDLLFQLVTL MNPKRLTSAG VRVFACNQRA
GEFVVTFPKA YHAGFNHGLN FNEAVNFALP DWLPFGRDCV QRYQEHRKHP VFSHDELLIT
ITQQSQSIKT AIWLNDSLQE MTDREMAGRR LLRFKIKEVV EARDREDQYQ CAICKAFCYL
SQVICVCVAC LDHAQLLCDC RTLRMRIPDD ELLNTQCMIT SRAAVPGNWQ AKLRRVLSES
ARPPLRSLRA LLAEGDRINY PLPELPTLRK CVTRANEWVD TANSFTTRKQ SRKRDRPERG
LADLYALFTE VGRLGFDTPE IGLLKNLAAQ AEEVKARAAV LLCEALIAHG SSLNVHLDEL
YQVEDIVLQE QLIKDLLDEV RHLLARARAC HLSGDNKYMK ILEARLQAGN DWDQRAADVL
AQPIKTIQDL PVDPAVMIRI MTARAKALEF EKMAKAWLTP ELPRVSDALR LVHRAEREFS
IPSVHDLKRT AEFANDLEER CDRVLKNRYE HGPMFETMQK WRSYAREHLT FILPNFDKLN
KQLDLHQQWI QKLPWFCHGK DVLDDVIETT DPKDDIPPND EFFTCICFVP VRPPSNGEQS
DAVQCDHCSA RFHGLCAKNG GSCPFCDPHH WNGSIHKNRS WHFCYLPTVL HSAPEVTKSY
SQDWKDLETI VRRVDRLSGV IGQFLSSVSS RRLEMIAQVR HYMRKLYQMQ FAVSPNPEVS
FGLDLAGLHR ILAGPARKKK RRSKFIFGQD VDKDWIDGTR CICRGRTCKR QYHKACVYVC
PLCSLRKGRS YRYADVRVKT HSRELIKIRL PKPVTQTLFV ELVEYVP
>RI
IPVSSKLDMS SVKTSSPRPF DLEDCPTFYP THEEFRDPMA YIKSISDRAR NSGICKVVPP
MGWKMPFVTD TEFRFKTRVQ RLNSIEASSR AKLNFLEALY RFHKQQGNPR VTVPTINHKP
LDLWLLRKEV HKLGGYEAVK GKKWADLGRL LGYGGPGLSM QLRNSYVRVI LPYEHFSDRV
RNSLCDGCDC GFHTFCLDPP LSVIPKGQWF CDTCLSVTVD FGFDEGEEHS LSRFQARDLA
FRKMWFESHP PVSEFDVENE FWRLVQSPHE TVEIEYGADV HSTTHGAMPT LETHPLDPYS
NDGWNLNNIP IVSDSLLRFI KSDISGMTVP WTYVGMVFST FCWHNEDHYT YSINFMHWGE
TKTWYGIPGE DAEKFEAAIR REAPDLFESQ PDLLFQLVTL MNPKRLTSAG VRVFACNQRA
GEFVVTFPKA YHAGFNHGLN FNEAVNFALP DWLPFGRDCV QRYQEHRKHP VFSHDELLIT
ITQQSQSIKT AIWLNDSLQE MTDREMAGRR LLRFKIKEIV EARDREDQYQ CAICKSFCYL
SQVVCVCVAC LDHAQLLCDC RTLRLRISDD ELLNTQCMIT SRAAVPGNWQ AKLRRVLSES
ARPPLRSLRA LLAEGDRINY PLPELPTLRK CVTRANEWVD TANSFTTRKQ SRKRDRPERG
LADLYALFTE VGRLGFDTPE IGLLKNLAAQ AEEVKARAAV LLCEALIAHG SSLNVHLDEL

YQVEDIVLQE QLIKDLLDEV RHLLTRARAC HLSGDNKYMK ILEARLQAGN DWDQRAADVL
AQPIKTIQDL PVDPAVMIRI MTARAKALEF EKMAKSWLTP ELPRVSDALR LVHRAEREFS
IPSVHDLKRT AEFANDLEER CDRVLKNRYE HGPMFETMQK WRSYAREHLT FILPNFDRLN
KQLDLHQQWI QKLPWYCHGK DVLDDVIETT DPKDDIPPSD EFFTCICFVP VRPPSNGEQS
DAVQCDHCSA RFHGLCAKNG GSCPFCDPHH WNGSIHKNRS WHFCYLPTVL HSAPEVTKSY
SQDWKDLETI VRRVDRLSGV IGQFLSSVSS RRLEMIAQVR HYMRKLYQMQ FAVSPNPEVS
FGLDLAGLHR ILAGPARKKK RRSKFIFGQD VDKDWIDGTR CICRGRTCKR QYHKACVYVC
PLCALRKGRS YRYADVRVKT HSKELIKIRL PKPVTQTLFV ELVEFVP
>CC
IQVSPHLDLS TVKTPSSRPF GLQNCPEFHP TTEEFKDPMA YIRSISDRAK DYGICKIIPP
EGWRMPFVTD TKFRFKTRLQ RLNSIEASSR AKLNFLEQLY RFHKQQGNPR VVVPTINHKP
LDLWLLRKEV HKMGGYEAVK GKKWSDLGRT LGYRGPGLST QLKNSYTRVI LPFEHYTERA
RNSLCDGCDC GFHIFCLDPP LTFIPKEQWF CFTCLSGTGD FGFDEGAEHS LSTFQARDLE
FRKLWFETHP PVSEYDVEEE FWRLVASPHE TVEIEYGADV HSTTHGAMPT METHPLDPAS
KDPWNLNNIP ILPDSLLRFI KSDISGMTVP WTYVGMAFST FCWHNEDHYT YSINYMHWGE
TKTWYGIPGD DAEKFEAAIM SEAPDLFENQ PDLLFQLVTL MNPQRLTEAG VRVFACNQRA
GEFVITFPKA YHAGFNHGLN FNEAVNFALP DWLPLGRDCV QRYREHRKLP VFSHDELLIT
ITQQSQSIKT AIWLVDCLRE MTEREFEDRK KVRLGLAEIL KEEDPEEQYQ CHVCKAFCYL
SQVVCQCVVC ADHVELLCEA MTLRKRFSDE ELTETLARVS ERASQPTAWR SKLARLLTEN
ARPPLRSLRA LLAEGDRINY PLPEMPNLRK CVTRANEWVD FANSFIIRKQ SRKRDRPDRG
LDELYAHLRE VENLGFDCPE INTLKTLAQQ AEDTKAKAIA LLCKRLLLEG SSLNVLLDEL
NEVEKIVDRE QLVNELLEEV RQLLTRARSC GLPSDNKHMQ FLEARLREGN TWEERARAVL
EQPIKTIAEL PIDPTIIDRL MAARAKALDF KKQAKAWLAC AKSRISEALR LAARSEKDFS
IPEVTELKKA AEWALDLESK SESVLRNRYQ LEDLFATIKK WQSHAVNHLR YSLPTFEKLT
EQVRLHELWL RDLPWFCHSE ELLADVLDCT RPEDDAPPTD EYYTCICNHP VRPPPPGVVS
DAVQCDHCFA RFHGECAKNG GSCPFCDHHH WNGTIHKQRS WHFCYLPMLA RKAPEISKHY
SRDFRELELI VHRVDRLSAS IGQFLSYTSH QRPEFIPQVR HYMRKLFRIQ FAVSPNPDIS
YGLDLAGLHR ILATRRPKKR RRPRFTFGQD VDKNWSDGTR CICRGVTCQR LYHVGCVFQC
PICCLRRGKR YEYSDVRVRT FSRELIFKKL SQPVGRTLFV ELVNYQP
>LB
IQAPSIFDLS SVKTSSPRPF GLQDCPEFFP TAEEFKDPMS YIRSISERAE PYGICKIIPP
ENWKMPFVTD TKFRFKTRLQ RLNSIEASSR AKLNFLEQLY RFHKQQGNPR VLVPTINHKP
LDLWLLRKEV QKMGGYDAVK GKKWSDLGRI LGYRGPGLST QIKNSYTRVI LPFEHFCDRA
RNSLCDGCDC GFHTFCLDPP LSSIPKEQWF CFTCLSGTGD FGFDEGEEHS LSTFQARDLE
FRRMWFESHP PVSEYDVEEE FWRLVQSPNE TVEIEYGADV HSTTHGAMPT METHPLNPYS
KDPWNLNNIP ILPESLLRFI KSDISGMTVP WTYVGMAFST FCWHNEDHYT YSINFMHWGE
TKTWYGIPGD DAEKFEAAIK CEAPDLFEAQ PDLLFQLVTL MNPQRVTEAG VRVFACNQRA
GEFVVTFPKA YHAGFNHGLN FNEAVNFALP DWLPYARACV QRYREHRKLP VFSHDELLIT
ITQQSQSIKT AMWLIGSLEE MTQREMNDRR KARLGLAEIL EEEDPEDQYQ CNICKAFCYL
SQVTCQCVVC VDHVSLLCEN QTLRKRFSDE ELLDIQAKVA ERAAVPSTWR GKLSKLLLEN
ATPQFRLLRA LLTEGDRINY PLPELASLRK CVTRATEWMD SANAFLIRKQ SRKRDRPDQG
LDDLYALIRE VKNLGFQCNE IGSLDKLVQD CEKMKSEASA LLCRRLLIQG SSLNVLLDEL
IEIEKIVDRE QLVTELLEEV RHLLTRARLC NLPQDNKHVQ LLEVRQREGD DWEGRARNVL
EQPIKTIAEM PIDPTVLDRL MTARTKALDF DKQARAWLSC EKPRLTDVLR LASRAEKDFS
ISSIQLLKQT ADIAADLETR CEQVLKNHYH SEDIFETIGQ WKDYAKDHLK FSLPCFEKLD
AQLKLHEQWR RELPWYCHIQ GLLEDVLECT RPDDDLPPTD EYFTCICNAP VRPPPPGIVS
DAVQCDHCYA RFHGECAKNG GSCPFCDHHH WNGTIHKQRS WHFCYLPNIL NNAPEITRHY
SEDYRQLEII VHGVDRLSAV IGQFLSYTSN QRPEYIPQVR HYMRKLYKIQ FAVSPNPDVS
FGLDLAGLHR ILAGRKPKKR RRPRFTFGQD IDQDWADGTR CICRGRTCGK RYHAGCVFSC
PLCCVRKNKT YEYSDVRVKT FSKDLMFKRL LLPYKPTLFV ELVSFQP
>PC
IPVSPVLDLS TVKTPNPRPF GLTDCPTFRP TPEQFKDPMA YIKSIAENAK SYGMCKIVPP
LGWSMPFVTD TEFRFKTRLQ RLNSIEASSR AKVNFLEQLY RFHKQQGNPR VSVPTINHKP
LDLWLLRKEV HRMGGYEQVR NKKWADLGRL LGYGGPGLAT QLKNSYTRVI LPYEHFCDHV

RNSLCDGCDC GFHTFCLDPP LQTIPRGQWF CHNCLFGTGD FGFDEGEEHS LSSFQARDLE
FRKLWFTSHP PVSETDVERE FWRLVQSPNE TVEVEYGADV HSTTHGAMPT SETHPLNTYS
KDPWNLNNIP ILPESLLRYI KSDISGMTVP WTYVGMVFST FCWHNEDHYT YSINYMHWGE
TKTWYSIPGD DAEKFEAAIR KEAPDLFEAQ PDLLFQLVTL MNPQRLREAG VEVYACNQRA
GEFVVTFPKA YHAGFNHGFN FNEAVNFALP DWLSLGLDCV KRYQEHRKHP VFSHDELIIS
ITQRSQAIKT AIWLNDSLQE MVERELSARQ RARLDMGEVL EEVDTEDQYQ CKICNCFCYL
SQITCSCVTC IDHADELCKC QVLRKRFSDS YLQDTQYAVA ERAGIPGAWE SKFEKLLNES
ARPPLRAMRA LLAEGDRISY PLKQLHHLRK CVTRANEWVD AANSFLVRKP NRKHDKPDRS
LSDLYALLRE VQDLGFDSPE IGQLQQLAKD AEDTKAKARE LLCEELMNKG GALNVQVDEL
TEIEKIVLRE QLLKELLEDV RRYINRARAC ALPADNRHMK SLENKLRLGT AWEDRVKAVL
DKPHRALQEL PIDPNLLDML KQTSLRGRDI ERQITAWLSG DKPRVQEVVK MVTRAEKEFD
IPVVRDMRRT VDFAVDLETR CDAVLKNRYQ HNDLFQTMRQ WKSYAKEYLS FTLPNFERLD
KQLTLHFRWL EGLPWFCHGK AILDDVVEST RPEDDQPPQD EYFTCICTNP VRPPAPGQTS
DAVQCDHCFA RFHGVCAANG GSCPFCDHQH WNGTIRKERS WHFCYLPTMM MHAPEITKNY
SEDWKQLEIV VHRVERLVGV IGQFLAYVGN QRAEYIPQVR HFMRKLYKIQ FAVSPNPETS
YGLDLAGLHR VLAGQRMKKR RRPKFTFGQD IDKDWVDGTR CICRGRTCSK TYHGGCVFMC
PLCCLRKNRT YPYSEVRVKT FSKEIIYMKL PPPYTQTLFV ELVRFTP
>DS
LPVAPTLDLS SVKTPNTRPF GLTDCPTFHP TPEQFKDPMA YIASISDTGK KYGMCKIVPP
MGWNMPFVTD TEFRFKTRLQ RLNSIEASSR AKVNFLEQLY RFHKQQGNPR VSVPTINHKP
LDLWLLRKEV HKLGGFDAVR DKKWADLGRL LGYTGPGLAT QIRNSYNRVI LPYEQFCERV
RNSICDGCDC GFHMFCLDPP LANIPRGQWF CHSCLFGTGD FGFDEGQEHS LSSFQARDRE
FRRLWFLSHG PVTEDDVELE FWRLVQTPTE TVEVEYGADV HSTTHGGMPT LETHPLDPYS
KDPWNLNNIP ILPQSLLRYI KSDISGMTVP WTYVGMIFST FCWHNEDHYT YSINYMHWGE
TKTWYSIPGS SAEKFEAAIK KEAPDLFEAQ PDLLFQLVTL MNPQRLKEAG VEVHACNQRA
GEFVVTFPKA YHAGFNHGLN FNEAVNFALP EWLPLGLDCV KRYQEHRKMP VFSHDELLIT
ITQQSHSIQT AMWLNDSLQE MTDREMDART RARLQMGEVL EETDGDDQYQ CATCKVFCYL
SQITCPCIVC IDHVDQLCKC HVLRKRFSDT ELQDIQAKVS ERAAIPGMWR NKLKKLLDES
PCPPLKSLKA IFTEGERIQH PLAELNSLRK CVNKANEWLE AANAIIIRKP TRKRDKPERS
LDDLYALLSE VANLGFDAPE IAQLRNIAQE AEETRKKARL LLCRRLILQS ATLNVTVDEI
VEVDKIVLRE QLLKDLLEDV RQLVGRADAC SLPMEHSQMQ RLQALYQAGT TWEDRAQRLL
EKSDRTLEEL PVDPELLERI IDLRKKGREY EKQAKIWLLA DKPKVQDVVK LVADAQRDFA
IPAVQDLQRT VAWAQDLETR SEAVLNATYS GKDIFETMQT WRDYAKKHLT FSLTNFSKLE
SQLESHNRWI QSLPWYCHGQ PILDDVVEAT RPEDDLPPSD EYFTCICTTP VRPPALGQVS
DAVQCDHCFA RFHGVCAANG GSCPFCDHHH WNGTIHKERN WHFCYLPTIL MHAPDITKHY
SEEWKQLEII VHRVDRLCGV IGQFVSFLGN QRAEYIPQVR HYMRKLYKIQ FAVSPNPEVS
FGLDLASLHR VLAGQRMKKR RRPKFVFGQD QDKDWLDGTR CICRGRTCGK LYHGGCVFMC
PLCCVRKNRV YPYSEVRVKT FSKDIIYMRL PPPYTQTLFV DLLRFTP
>TV
LSVPQMLDLS SVKTADARPF GLTDCPTFHP TPEQWKDPMA YIASIADAGK RYGMCKIVPP
AGWNMPFVTD TEFRFKTRLQ RLNSIEASSR AKVNFLEQLY RFHKQQGNPR VSVPTINHKP
LDLWLLRKEV HKLGGYDAVK DKKWADLGRL LGYTGPGLAT QLRNSFSRVI LPYEQFCDRV
RSSICDGCDC GFHMFCLDPP LVNIPRGQWF CHTCLFGTGD FGFDEGEEHS LSSFQARDLE
FRRLWFLSHP PVTEDDVEKE FWRLVQSQNE TVEVEYGADV HSTTHGGMPT LETHPLDPYS
KDPWNLNNIP ILPQSLLRYI KSDISGMTVP WTYVGMIFST FCWHNEDHYT YSINYMHWGE
TKTWYSIPGC DAGKFEAAIR KEAPDLFEAQ PDLLFQLVTL MNPQRLKEAG VDVYACNQRA
GEFTVTFPKA YHAGFNHGLN FNEAVNFALP DWLPLGLDCV KRYQEHRKMP VFSHDELLIT
VTQQSQSIQT ALWLNDPLQE MTDREMDART RARRQMNEVL EETDGDDQYQ CSVCKVFCYL
SQITCTCIAC IDHVDQLCKC HVLRKRFSDT ELQDIQARVS ERAAVPTVWR GKLKRLLDDS
PRPPLKGLRN LFTEGERIQF PLAELNSLRK CVNKANEWLE AANGILIRKP TRKRDKPDRT
LEDLYALLSE VENLGFDAPE IAQLRTIAAE AEDTRRKARA LLCEHLILHA QTLNVLVDEI
VEVEKIVLRE QLVKELLEDV RQLMARAHAC NLPAENMYMQ RLETLLRAGS NWEEKAKALL
DKQQRTLEDL PVDPDLLERI LDLRKKGKDF EKQAKIWLLS EKPKVQEVVR LVTEHDKDFE
IPAVEDLKRT VDFAQDLEAR CEAVMRGIYN PPDVFHTMLQ WRKYAKEHLT FSLPNFETLD

KQLTTHFRWL EGLPWWCHGQ PILDDVVEST RPEDDLPPND EYFTCICTTP VRPPAHGTVS
DAVQCDHCFA RFHGICAANG GSCPFCDHHH WNGTIHKERN WHFFFLPQLL LQAPDVTKNY
SEEWKQLEII VHRVDRLCAV IGQFLSFAAN HRVDYIPQVR HYMRKLYKIQ FNVASNPDAN
FGLDLAGLHR ILAGQRMKKR RRPKFVFGQD VDKDWNDGTR CICRGRTCNK FYHGGCVFMC
PLCCVRKNRV YPYSEVRVKT FSKDIVYMRL PPPYTQTLFV ELIRFTP
>FP
IRVTSFLDLS TVKTPNRRPF GLTDCPVFHP TLEQWKDPLA YVKSISDNAR KYGMCKIVPP
AGWSMPFVTD TEFRFKTRLQ RLNSIEASSR AKVNFLEQLY RYHKQQGNPR VTVPTINHKP
LDLWLLRKEV QKQGGYEAVR NKKWADLGRL LGYSGPGLST QMKNSYSRVI LPYENYRERV
RNSLCDGCDC GFHMFCLDPP LGAIPKGQWF CHTCLMGTGD FGFDEGEEHT LSTFQARDLE
FRRLWFKSHP PVTETDVENE FWRLVQSQQE TVEVEYGADV HSTTHGGMPT LETHPLDPYS
KDPWNLNNVP ILPDSLLRYI KSDISGMTVP WTYVGMVFST FCWHNEDHYT YSINYMHWGE
TKTWYSIPGE DAEKFEAAIR REAPDLFEVQ PDLLFQLVTL MNPNRIKEAG VDVYSCNQRA
GELVITFPKA YHAGFNHGLN FNEAVNFALP DWLPFGLDCV KRYQEHRKLP VFSHDELLIT
ITQQNHSIQT ALWLNDSLQE MMVREMRIRD KARLQMSEVT EGMDAEDQCQ CTKCKVFCYL
SQITCSCAVC IDHIDDLCKC RVLRKRFDDA ELQEIQMKVS ERAAVPGAWK AKLTKLLSEH
SRPPLRSLRA LLAEGERINH PLPELASLRK CVARANEWVD AANAFLVRKP SRKRDRPEHG
LTDLYAVLNE VDRLGFDCQE IGFLKNLAKE AEETREKARL LLCDRLLLHG LSLNVLVEEL
LEVEKIVLRE QLLKELLEDV RNWVARARQC DLPPENSFMK RIERLLRLGD EWERCVKDVL
MKPQRTIEQL PIYPDLLDRL LDARSRAKDY EKQAKLWISP EKPKVQDAVK LVARADKEFN
IPAVSDLKRT VDFAMDLETR CDAVLKHRYQ HSDIFQTMLQ WRKYAKEHLT FTLPNFERLD
KQLSAHFRWL ESLPWFCHAQ NLMEDVIEST RPEDDLPPND EYFTCICMTP VRPPAQGTTS
DAVQCDHCFA RFHGVCAANG GSCPFCDHHH WNGTIHKERN WHFCYLPTIL LHAPEVTKNY
SDQWKQLEII VHRVDRLAGV IGQFLSFASN QRAEYIPQVR HYMRKLYKIQ FAVSPNPEVS
FGLDLAGLHR ILAGQRVKKR RRPKFIFGQD VDPDWRDGTR CICRGRTCDK LYHAGCVFMC
PLCCIRKNRA YPYSEVRVKN FSKEIIYMKL PPPYTQTLFV ELIRFTP
>PP
IPVKPALDMS TVKTPNRRPF GLTDCPVFRP TLEQFKDPLA YIKSISEKAK AYGMCKIVPP
LGWEMPFVTD TEFRFKTRLQ RLNSIEASSR AKVNYLEQLY RFHKQQGNPR VVVPTINHKP
LDLWLLRKEV QKQGGYEMVR NKKWADLGRL LGYSGPGLST QMKNSYTRVI LPYEHYRERV
SKSSPHDQSA CFHMFCLDPP LTTIPKGQWF CHTCLCGTAD FGFDEGEEHS LSSFQARDLE
FRRQWFKRHP PVTETDVENE FWRLVQSQHE TVEVEYGADV HSTTHGGMPT LETHPLESSS
KDPWNLNNIP ILPDSLLRYI KSDISGMTVP WTYVGMVFST FCWHNEDHYT YSINYMHWGE
TKTWYSIPGA DADKFEAAIR REAPDLFEVQ PDLLFQLVTL MNPNRIRDAG VDVYACNQRA
GEFVITFPKA YHAGFNHGLN FNEAVNFALP DWLPFDLDCV RRYQEHRKLP VFSHDELLIT
ITQQNQSIQT ALWLNDNLQE MMVRERRIRD KARLGLKDRP EKTDPEDQYQ CSFCKVFCYL
SQITCDCVVC VDHIDELCKC RYLRLRFDDT EIQDIQMKVS DRAAIPSTWR AKLDRLLMES
ARPSLRSLRA LMAEGERINY PLSELHALRK CVVRANEWVD TANSFLIRKV SRKRDKPERT
LDELYTALSE VEDLGFDCQE INALKALANE AQKTREKARA LLCERLLIEG STLNVLVDEL
VEIEKIVSRE QLLKELLEDI RQFAARASQC ELSTDNPQMV VLSRMLHAGE EWEARVKDVL
GRPLKTVEEL PVDVELVDRL VATLVRAKDY EKQAKAWLMP EKPKVQEAAK LVTRAEKDFN
IPAVQDLKRT VDFALDLEAR CDAVLKNRYE HGDIFQTMLQ WRKYAKEHLT FTLLNFDKLD
KQLVQHARWL EGLPWYCHGQ AILEDVIEST RPEDDLPPND EYFTCICTTP VRPPAAGTVS
DAVQCDHCFA RFHGVCAANG GSCPFCDHHH WNGTIHKERS WHWCYLPTIL MHAPDVTKNY
SEDWKQLEII VHRVDRLSQV IGQFLSFASN QRADYIPQVR HYMRKLYRIQ FAVSPNPEVS
FGLDLAGLHR ILAGQRVKKR RRPKFTFGQD VDKDWTDGTR CICRGRTCSK LYHAGCVFMC
PLCCIRKNRA YPYSEVRVRT FSKDMIYMRL PPPYTQTLFI ELIRFVP
>WC
IPVSRTLDPL SVKTPNPRPF GLTDCPIYAP TMEQWKDPLA FIRSISDEAR TYGMCKIVPP
MGWEMPFVTD TEFRFKTRLQ RLNSIEASSR AKVNFLEQLY KFHKQQGNPR VVVPTINHKP
LDLWLLRKEV QKLGGYEAVR GKKWADLGRM LGYGGPGLST QMKSSYARVI LPYEEYRDIV
RNSLCDGCDC GFHIFCLDPP LNSIPKGQWF CHTCLFGTGD FGFDEGEEHS LSSFQARDLE
FRRLWFKSHP PVSETDVENE FWRLVQSQQE TVEVEYGADV HSTTHGGMPT LETHPLNPYS
KDPWNLNNIP ILPDSLLRYI KSDISGMTVP WTYVGMVFST FCWHNEDHYT YSINYMHWGE

TKTWYSIPGS DAEKFEAAIR REAPDLFEVQ PDLLFQLVTL MNPNHLREAG VDVYSCNQRA
GEFVITFPKA YHAGFNHGFN FNEAVNFALP DWLPFGLDCV KRYQEHRKLP VFSHDELLIT
ITQQNQSIQT AIWLNDNLQE MMAREMRLRD QARFQMREAL EELDPEDQYQ CTICKVFCYL
SQITCHCVVC IDHVDQLCKC RILRKRFDDM ELKEILTKVS ERAAIPSTWR NKLNKLLTES
ARPQLRNLRG VLAEGERINY PLPELSSLRK CVLRANEWVE TANALLVRKP GRTRDKPERS
LDELYAILRD VDNLGFDCQE IGHLKTLAKD AEETKEKALT LLCERLLLHG FSHNVLVDEL
VEVEKIVLRE QLLKDLLEDI RQFVARATQC DLPADNQQMK HLKSLLRVGE DWESRALEVL
NKPQKTIDEL PVDPEVMDRL VTACVRAKEY EKQAKAWLIP GKPKVQEAMK LVSRAEKDLN
IKAIHDLKRT VDFALDLESR CEAVLRHRYQ HGDIFHTMLQ WRKYAKEHLT FILPNFEKLD
KQLTLHYRWL EGLPWFCHGQ SILEDVVEST RPEDDLPPND EYFTCICTTP VRPPAAGTVS
DAVQCDHCFA RFHGVCAANG GSCPFCDHHH WNGTIHKERS WHFCYLPTIL LHAPDVTKNY
SEDWKQLEII VHRVDRLSAV IGQFLSFASN QRPEYIPQVR HYMRKLYRIQ FAVSPNPEVS
FGLDLAGLHR ILAGQRIKKR RRPKFVFGQD IDKDWLDGTR CICRGRTCNK RYHAGCVFMC
PLCCIRKNRG YPYSEVRVKT FSKDLIYMKL PPPYTQTLFV ELIRFTP
>CP
IEVAPVLNLA SVKTISPRPF GLEDCPAFYP TPEEFKDPMA YIRKISDKAT EYGICKVVPP
VGWKMPFVTD TEFRFKTRLQ RLNSIEASSR AKVNFLEQLY RFHKQQGNPR VSVPTINHRP
LDLWLLRKEV HKLGGYEAVK MKKWSDLGAL LGYRGPGLST QIRNSYTRVI LPYEHYCERV
RNSLCDGCDC GFHMFCLDPP LSSIPKGQWF CHTCLLETGD FGFDEGEEHS LSTLQVRDAE
FRRMWWSKHA PVSEEEVEQE FWRLVQSPDE TVEVEYGADV HSTTHGAMPT METHPLDPMA
KDPWNLNNMP IVSDSLLRYI KSDISGMTVP WTYVGMVFST FCWHNEDHYT YSINFMHWGE
TKTWYGIPGD DAEKFEAAIK SEAPDLFEAQ PDLLFQLVTL MNPARLTEAG VRVYACNQRA
GEFVITFPKA YHAGFNHGFN FNEAVNFALP DWLRLGRDCV ERYREHRKLP VFSHDELLIT
ITQQSQSIKT AIWLADSLRE MVVRELGERA RVRLGMKEVL EEADPEDQYQ CAICKMFCYL
SQVTCQCVVC ADHVDLLCEH LTLRLRFSDG ELQDTLSKVV ERSEVPGAWK KKLQKVLEES
ALPPLRSLRA LLAEGERINY PLPELPTLRK CVQRANEWVD SANGFIIRKQ SRKRDRPDRT
LSDLYALLRE AENLGFDAPE LGVLEALARQ AEDISANAQV LCSERLLLDA SSLNVYLDPV
LEIEKVVSRE RLLDELLDEV RRLLTRARAC DLAPDNKYVQ RLEARHRAGA DWEERARMML
EQPGKTIEEL PTDPTIHDRL KSALAKAMDF DKQAKGWATA DKPTPSQLSG FIARCEKDFH
IPSVKNLSHI AQYGREVEDK CDAVLRNRYP HKDVFKEIEE WGRYARQHLS LSLPFFQQLD
KQLNLHYRWL EDLPWYCHGS EILDDVIEAT RPEDDLPPTD EYFTCICQDP VRPPPPGGQS
DAVQCDHCFA RFHGKCAANG GSCPFCDHNH WNGKIHKERN WHFCFLPLIL TRAPDLTHNY
SDTWKQLEII VHRVDRLSSV IGHFLTFASN QKTSLLPQVR HYMRKLYKIQ FAVSPSREIS
FGLDLAGLHR ILAGLPHKKR RKPKLVFGPD ADEDWYDGTR CVCRRNACKR RYHAPCVWTC
PLCCVRKNKS YPFAEVRVKM HGQELIVERM PLPRPDAIVL DLVSFTP
>PS
ATVSPPMDMS TVKTASPRPF GLEECPTFYP TMEEFKEPMA YVQSISEEGM KYGLCKIVPP
SGWKMPFVTD TEFRFKTRVM TLNSIEASSR AKLNFLEQLY RYHKQQGNPR VSVPTINHKA
LDLWRLRREV HDRGGYEEVR HKLWAEIGRA LGYTGPGLSA QLKNSYTRVI LPYEHFQHHV
RNSLCDGCDC GFHIFCLDPP LASIPRGQWF CHTCLFGTGD YGFDEGEEHC LSSFQKRDRI
FRQMWWTRHP PVSEDDVERE FWRLVQSPRD TVEIEYGADV HSTTHGGMPT LETHPLDPYA
KDPWNLSNIP ILADSLLRYI KSDISGMTVP WTYVGMVFST FCWHNEDHYT YSINYMHWGE
TKTWYGIPGK DAELFEAAIK KEAPELFEAQ PDLLFQLVTL MNPQTLRDAG VPVYACNQRP
GEFVITFPKA YHAGFNHGFN FNEAVNFALP HWLPFGLDCV QRYREHKKPP VFSHDELLIT
ITQHSQTIKT ALWLQDSLRE MTNREMRMRE QARDGLGEVL EEEDPEDQYQ CTTCKTFCYL
SQITCQCVSC IEHRQQLCAC RILRKRFTDA TLRETLAKVE ERAAIPKQWR SKLHKLLMDS
ARPQLRSLRA LLAEADRINY PLPEVSALRK CVMRANEWVE SANAFLVRKQ TRKREAKERT
LDDLRKVLDE VEDLGFDAPE IASLQALEKQ ATDAKAQAQA LLCERLILDC STLNVQVEEL
AELDKIVASE QLVQDLSGEV QQLLGRIRTC GLPPDNKYVM MLHARRHAAS SFEEKVRELL
ADPHKTIEQM PVDPEVMGDL QLVIVRAKEY QAKAKAWLVP DKPRVEEAME LVTLAENEFN
ILAVQDLKRT TEFAQDLEAR CEAVHEHRYR HGDLFEMFDK WKAYGRDHLT FYMPNFEKLD
VQLKLHYRWV ESLPWYCHGK QILEDVIEST RPEDDLPPND EYFTCICTQA VRPPPAGTVS
DAVQCDHCYA RFHGVCAANG GSCPFCDHHH WNGTIHKDRS WHFCYLPTIL LSAPDITRNY
SEDWKQLEII VHRVDRLSTV IGQFLSFSSN QRPEYIPQVR HFMRKLYKIQ FAVSPSPEVS

FGLDLAGLHR ILAGQRTKKR RRPKFTFGQD IDKNWVDHTR CICRGRTCNR LYHAGCVFMC
PLCCLRKNRP YPYAEVRVKN FAKEIIYRKM PPPYTKTLFV ELTANAA
>GT
IPVSPTLDMS SVKTSAPRPF GLEDCPAFYP TSEEFQDPMA YIRSISDIAK DYGICKVVPP
EGWKMPFVTD TEFRFKTRLM RLNSIEASSR AKVNYLEQLY RFHKQQGNPR VCVPTINHKP
LDLWLLRKEV KRLGGYEAVR AKKWADLGRI LGYGGPGLST QIKNSYTRVI LPYEHFQERV
RSSLCDGCDC GFHMFCLDPP LTSIPKGQWF CHTCLFGTGD FGFDEGQEHS LSSFQARDAA
FRKMWFKTHP PVSEHDVEEE FWRLVQSPTE TVEVEYGADV HSTTHGAMPT LETHPLDPYS
KDPWNLNNIP IVHDSLLRYI KSDISGMTVP WTYVGMVFST FCWHNEDHYT YSVNFMHWGE
TKTWYGIPGE DAEKFEAAIK REAPDLFEAQ PDLLFQLVTL MSPKRLTEAG VRVYACNQRA
GEFVITYPKA YHAGFNHGLN FNEAVNFALP DWLPYGLSCV QRYREHRKLP VFSHDELLVT
ITQQSQSIQT AIWLNDSLKE MTEREKDVRQ RARLGLTEVL EEEDAEDQYQ CTVCKAFCYL
SQITCPCVAC IDHVAFLCEC RVLRLRFSDD TLLETQAKVE ERAAIPDNWR AKLHKTLAET
ARPQLRTLRA LLAEGERINH HLPDLATLKK CVYRANEWTD AANSFIIRKQ SRKRDRQDGG
LEEVYELLRQ VDNLGFDSQE ITQLRNLAKE AEDTKAKALE LLCERLLLHE SSLNVHLDEL
DQVKNLVLRE QLIKELLDEV RHLLSRARSI NIPEDDKYMM LLRSRERAGS EWEESVKQLL
SKPCKDINKL PVDPTVFDRL MNARAKAKEY EKQARLYLFP EKPRPQDAMK LVTRVEKDYA
IPSVHDLKRT AEFALDLETR CDTVLKNRYS HGDMFDIMHQ WQGYAREHLT FALPNFEKLD
AQLKSHYRWL EGLPWYCHGK QILEDVIEST RPEDDLPPSD EYFTCICTKA VRPPPPGSVS
DAVQCDHCYA RFHGVCAANG GSCPFCDHHH WNGAIHKDRS WHFCYLPTIL LSAPEITKNY
SQDWKDLEII VHRVDRLSMV IGQFLAFASN QRPEYIPQVR HYMRKLYKIQ FAVSPNPEVS
FGLDLAGLHR ILAGQRMKKR RRPRFTFGQD VDKDWQDGTR CICRGRTCNK WYHAGCVFMC
PLCCLRKNRT YPYAEVRVKT FSKDIVYMKL PPPYTNTLFV ELIRFTP
>HI
IPVAPALDFA GVRTSSPRPF GLEDCPTYYP TQEDFRDPMA YVRKISEQAK EYGMCKVVPP
PGWKMPFVTD TEFRFTTRLQ RLNNIEASSR AKMNYLEQLY RFHQQQGNTR VAVPTINNKP
LDVWRLRKEV HALGGFNEVK ARKWADLGRL LGYGGPGLST QMRWSYIRVI HPYEEFCERV
RASLCDGCDC GFHMFCLDPP LASVPKGQWF CHTCLFGTGD FGFDEGEEHS LSSFQARDRE
FRKRWFEDHP PVSEHDVENE FWRLVESPTD TVEIEYGADV HSTTHGAMPT LETHPLDPYS
KDPWNLNNIS ILSDSLLRYI KSDISGMTVP WTYVGMVFST FCWHNEDHYT YSINYMHWGE
TKTWYGIPGE DAEKFEAAIR REAPDLFEAQ PDLLFQLVTL MNPKRLTDAG VRVYACNQRA
GEFVITYPKA YHAGFNHGLN FNEAVNFALP DWLPYGRQCV QRYREHRKLP VFSHDELLVT
ITQQSQSIAT AIWLNDSLKE MTDREMEDRR KARLNMGESL IEADIEEQYQ CKVCNVFCYL
SQVTCACIVC VEHAGMLCSC RVLRTRFSDT QLLETQAAVA ERAAIPGNWQ AKFDKVLKES
ARPPLRALRA LLAEGDRIGY SLSELHDMRK CVNRANEWLD AANGFIVRKQ SRKRERPDRG
LDELYEMLKE VEDLGFDCPE IGVLKERAAK AEEMKERART LLCQRLLMEG SSNNVYLDEL
TDLDKLVARE QLLDELLEEA SQYLVRARAC DLPLDNKHLK KLEARQRAGQ TWDERARHVL
SQPYKTIEEL PIDPTILDRI GTARLKAKEF EKQAQTWLVP EKPRVQDVLR LVTRAEKDFS
IPAVRELKRM TDFALDLESR CDQVLKNTYE HEDIFVIMLK WVDYAKQHLD FSLPKFEKLD
QQLTQHYRWI ESLPWYCHGK QLFDDVMEST KPEDDHPPDD EFFTCICNDA VRPPAPGTVS
DAVQCDHCYA RFHGQCARSG GSCPFCDHHH WNGSIHKERS WHFYYMPTIL MYAPEITKNY
SEDWKQLEVI VHRVDRLAAV IGQFLSFASN QRREYISQVR HFMRKLFKIQ FAVSPNPEVS
FGLDLAGLHR IVAGQRTKKR RRPKFQFGQD IDQDWLDGTR CICRGRTCSK VYHGGCVFIC
PLCCLRKNRR YEYAELRVKM FSKDIIYKTL RPPYTQTLFV ELIRFTA
>SL
IPVSPLLDLS SVKTSGPRPF GLEDCPVFYP TLDEFNDPMT YVRSISDSAK DYGICKIVPP
NGWKMPFVTD TEFRFKTRLQ RLNSIEASSR AKINFLEQLY RFHKQQGNSR VSVPTINHKP
LDLWLLRKEV HKLGGYDAVK NKKWSDLGRL LGYGGPGLST QIKNSYTRVI LPYEDFCERV
RTSLCDGCDC GFHMFCLDPP LATIPKGQWF CHTCLFGTGD FGFDEGEEHS LSSFQARDAE
FRRLWWSAHP PVSEYDVENE FWRLVQTPQE TVEIEYGADV HSTTHGAMPT METHPLDPYA
KDPWNLNNMP IVSDSLLRFI KSDISGMTVP WTYVGMVFST FCWHNEDHYT YSINYMHWGE
TKTWYGIPGD DAEKFETAIK SEAPDLFEAQ PDLLFQLVTL MNPKRLIDAG VRVHACNQRA
GEFVITFPKA YHAGFNHGFN FNEAVNFALP DWLPYGRDCV QRYREHRKLP VFSHDELLIT
ITQQSQSIKT ALWLADSLKE MTEREIAARE KARFGMTEVI DEVDPEEQYQ CIICKVFCYL

SQVNCQCVVC VEHADLLCDH LTLRLRFTDA DLLDTQTKVV ERAGIPSSWK GKLSKVLIDS
ARPQLRSLRA LLAEGERVNY HLPELPALRK CVNRAGEWVD SANTFLIRKQ SRKRDRPDRG
LEDLYSLLRE VENLGFDTPE ISVLQGLARQ AEEIKLKALD LLCERLLLQG SSLNVHLEEI
VEIEKIVMRE HLLRELLEDI RGLLTRARSC NLPQDNKYMK TLESRQRAGD NWEERARHVL
EQPYKTIDEL PIEVTVYDRL MSARNKAKDF EKQAKAWLSP DKPKVQEVMR FVARAEKDFS
IPAIQDLKRT ADIAFDLETR CEAVIKNRYQ HGDIFDMMRR WKTYTREHLP FSLPIFEKLD
VQLSQHQRWL EDLPWYCHGQ AILDDVVEST RPEDDLPPSD EYFTCICTTP VRPPPPGVVS
DAVQCDHCFA RFHGVCAANG GSCPFCDHHH WNGAIHKERS WHFCFLPTIL LSAPDITRNY
SDAWKQLEII VHRIDRLSSV IGHFLSYASN RRAEYIPQVR HYMRKLYKIQ FAVSPSREVS
FGLDLAGLHR ILAGQRIKKR RRPRFTFGQD IDKDWTDGTR CICRGRTCNK LYHAACVFMC
PLCCLRKNRS YPYSEVRVKT FSKDIIYVKM LPPYTQTLFV ELTRFVP
>FS
IPVSPVLDMS SVKTDAPRPF GLEDCPSFYP TEEEFKDPMA YVRSISDTAQ NYGICKVVPP
VGWKMPFVTD TEFRFKTRLQ RLNSIEASSR AKLNFLEALY RFHQQQGNPR VSLPTINHKP
LDLWLLRKEV HKMGGYDEVK GKKWSDLGRI LGYGGPGLST QIKTSYTRVI LPFEQFSERV
KNLLCDGCDC GFHMFCLDPP LTAIPKGQWF CHTCLFGTDD FGFDEGEEHS LSSFQARDLE
FRKLWFESHP PVSEYDVESE FWRLVQSPAE TVEIEYGADV HSTTHGGMPT LESHPLDQYA
KDPWNLNNIP IVSDSLLRFI KSDISGMTVP WTYVGMIFST FCWHNEDHYT FSVNFMHWGE
TKTWYGVPGD DAEKFEAAIK KEAPDLFEAQ PDLLFQLVTL MNPKRLVDAG VRVYGCNQRA
GEFVVTFPKA YHAGFNHGFN FNEAVNFALP EWLSYGRDCV QRYRDHKKLP VFSHDELLIT
ITQQSQTIKT ASWLLGSLKE MTDREMARRV YARLGLAEIL EEEDPEDHYQ CTVCKMFCYL
SQITCQCVVC IDHAEHLCDH LIMRKRFSDA HLLDTLNKVA ERAAAPSTWR GKLGKLLTES
ARPQFKSMRA LLAEGDRIGY HIPELAPLRK CVARGNEWMD ASNTYLIRKQ SRKRDRPDGG
LEELYALLRE VENLGFDCPE IASLQALAQH AEEAKAQARR LLCERLLLQG TSINVHLDEL
LEVQKIVTRE QLIKELLEDI RHLLTRARAC NLPPDNEHMK LLADRQRAGD NWEERAKSIL
AQPFKTIDEL PIDHAVLDRL LSALSKAQEL DKQAKAWLVP EKPKVADVMR LVIKAEKEYS
IPSISDLKRT AEIAADLESR CDDVLKNRYQ HGDLFDSMRK WKAYAVDHLT FALPMFDKLD
KQLIVHYKWL ESLPWYCEAQ KLIADVLDAT RPEDDNPPND EFYTCICTAA VRPPPPGILS
DAVQCDHCFA RFHGECAKSG GSCPFCDHNH WNGNIHKERS WHFHLLPEVL HAAPEITKNY
SEHWKQLEII VHRVDRLTAH IGQFCMFAQN HLAHFIPHVR HYMRKLYKLQ FQIGATREES
YGLDLASLHR VLAGQKMKKR RRPKFTFGQD LDKDWQDGTR CICRGRTCNK TYHTGCVFIC
PLCCLRKNIT YPYSDVRVKN FSKDIVYVKM LPPRTQTLFV ELVRFSP
>PI
IPVSPILDLS SVKTRGPRPF GLEDCPAFYP TVDEFKDPMA YVRSISTMAK NYGICKIVPP
AGWKMPFVTD TEFRFKTRLQ RLNSIEASSR AKLNFLEALY RFHQQQGNPR VSLPTINHKP
LDLWLLRKEV HKMGGYDAVK GKKWSDLGRT LGYGGPGLST QLKNSYTRVV LPFEQFSERV
KNLLCDGCDC GFHMFCLDPP LTVIPKGQWF CHTCLFGTDD FGFDEGEEHC LSSFQARDLE
FRKLWFESHP PVSEYDLENE FWRLVQSPHE TVEIEYGADV HSTTHGAMPT METHPLNQYS
KDPWNLNNIP IVSESLLRFI KSDISGMTVP WTYVGMVFST FCWHNEDHYT FSINFMHWGE
TKTWYGIPGD DAEKFEAAIK SEAPDLFEAQ PDLLFQLVTL MNPQRLTDAG VRVYGCNQRA
GEFVITFPKA YHAGFNHGFN FNEAVNFALP EWLPYGRDCV QRYREHRKLP VFSHDELLIT
ITQQSQSIKT ATWLIDSLKE MVDREMGGRA YARLGLGELL EEEDPEDQYQ CTICKMFCYL
SQITCQCVVC IDHAEFLCDH LIMRKRFSDA DLLETLNKVS ERASLPAVWR GKLNKLLMES
ARPQFRSLRA LLAEGERINF FIPELAPLRK CVTRGNEWMD AANTFLIRKQ SRKRDRPDGG
LDELYALLRE VENLGFDCPE IGNLQTLGQH AEEAKHKARH LLCERLLLQG TSINVHLDEI
TEVQKIVTRE QLIRELLEDI RHLLTRARAC NLPADHRLMK LLEDKQRAGD NWEERAQSIL
AQPFKTIDEL PIDPAVLDRL LAALAKAQDL DKQAKVWLVP ERPKVQDVMR LVQKAEKEYS
IPSISDLKRT AEIAVDLENR CEDVIKNRYQ HGDLFDMMRK WKIYAADHLA FALPMFDKLD
KQLVIHYKWL ESLPWYCEAQ KLISDVLEAT RPEEDNPPND EYYTCICTLA VRPPPPGILS
DAVQCDHCFA RFHGDCAKSG GSCPFCDHNH WNGTIHKERA WHFHLLPEIL HAAPDITKNY
SEHWKQLEII VHRVDRLTAH IGQFCMFAQN HLAHFIPHVR HYMRKLFKLQ FQIGATLEES
YGLDLAGLHR ILAGQKVKKR RRPRFTFGQD IDKDWTDGTR CICRGRTCNK VYHTGCVFVC
PLCCLRKNRT YPYSEVRVKN FSKEIVYMKM LPPYTQTLFV ELIRFTP

07-Dyein heavy chain

>DS
FEVEHLKYAT LATSPHLATQ KQIATIKTAR NVIEYNEQYV TKRLLVNIIW AFSGDARGEW
SGWQSRVPVI EILYSWLSEH KPLMLCGPPG SGKTMTLFSA LRKLPDMEVV GLNFSSYRKT
PNGVILAPTQ IGRWLVVFCD EINLPATDKY GTQRVISLVE CGGYWRTSDM AWVKLERIQF
VGACGRVPLS HRFLRLVMVD YPGELSLKQI YGTYNRALLK VVPNLRAYAE PLTDAMVTLY
LASQKRFTTD IQAHYVYSPR ELTRWVRGIY EAIRPLEILS VEGLVRVWAH EALRLFQDRL
VTEEEKRWTD EAIDNAAMEH FPTINILFSN WTSKNKARLR VFYEEELDVP LVLFNDVLDH
VLRIDRVFRQ TTLSRFVAWM NGLSIFQIKV SNKYTGEDFD LLANAEVPGL FEGFTMNPPE
NGLASRAATS PALFNRCVLD WFGDWLQTYN PPSMFPIAYR VVNALVHVHE SMHHINQRLS
RRQGRYPRHY LDFINHYVRL HNEKREELER HLHVGLDKLV EQVEELRKSL AIKRKLKQMV
ADQQEAEQKK AASIEIQAAL KEQDKNIKQR REVVMADLAD AEQMTKQLRD LMKRDFLSRP
SYNFETVQRA SKACMIAELE KSIERYKEEY AGLIRVESKV DRSMRLLESL SRTFDTEMGT
IVGDVLLSAA FLAYAEWSSH LAEAGIKFKA ELSLPRLSWQ SKSLPSDNLC TVTSFLDEAF
LKVLESALRF GNTLLIQDVE HLDPILNEIR RTGGRVLIRL GNQDIDFSPS FPSVEFSPDI
CSRVTSQSLD QVLKVETDLM KMQGEFRLRL RTLEKLLLQA LNESSGNILD DDKVINTLET
LKREAAETDV VMKEVEEVTA EYLPLAQACS AVFFILEQLN LVNHFYQFSF LDIFDYILHH
NPNLQGVLMN DLFVIVYKRT SRALDELEFF LESGVVTFAK QPMFKPVLTH LTEHEDEWIP
FLIIIKCLRP DRLLQSVPGY DASYRVENQE GFTLADQAVA LAARQGSWVL LKNVHLAPSW
LGEKKLQTLN PHRNFRLFLT MEANPSIPVN ILQSRILMNE PPPGIFLLAW FHAVVQEDMA
AAFTTIDTWL NSVAKGRANV DPAAIPWDAI RTLVKQSVYG GRVDSDFDQR IVDAFVDGLF
TPHAYNVDFP DGTKLEHFMS WPSWLSLPPT AERRKMRMLA DDDHERCREW LQQLPANFQT
LQKQSAEHSD PLYRLFFREG SIGRLLSQVR KDLADVLMSS LTKGTIPTHW RRYKVHKISN
FARRLAQLDQ IAQLDNGGLF FPEAYITATR AVAHRKKWSL ETLRLELDIE KVNDPGLVLE
GAAWATD

>TV
FEVEHLKYAT LATSPHLVTQ KEIALIKTVR NVIEYNEQYA TKRLLVSLIW AFSGDARGEW
TGWQGRVPVI DILYSWLSEH KPLMLCGPPG SGKTMTLFSA LRKLPDMEVV GLNFSSYRKT
PNGVILAPVQ IGRWLVVFCD EINLPATDKY GTQRVISLVE CGGYWRTTDM AWVKLERIQF
VGACGRVPLS HRFLRLVMVD YPGELSLKQI YGTYNRALLK VVPNLRAYSE PLTDAMVALY
LSSQKRFTTD VQAHYVYSPR ELTRWVRGIY EAIRPLEILS VEGLVRVWAH EALRLFQDRL
VTEEEKRWTD EAIDNAAMEH FPTINILFSN WTSKDKARLR VFYEEELDVP LVLFNDVLDH
VLRIDRVFRQ TTLSRFVAWM NGLSIFQIKV HNKYTGDDFD LLANAEVPGL FEGFTMNPPE
NGLASRAATS PALFNRCVLD WFGDWLPSYN PPALFPISYR VVNALVHVHE SMHQINQRLS
RRQGRYPRHY LDFINHYVRL HNEKREELER HLHVGLDKLV EQVEELRKSL AIKRKLKRMV
ADQQEAEQKK AASIEIQAAL VEQDKHIKER REVVMADLAD AEQMTKQLRD VMKRDFLSRP
SFNFETVQRA SKACMIAELE ASIERYKEEY AGLIRVESKV DRSMKLLESL SRTFDTEMGT
IVGDVLLSAA FLAYAEWSSH LAEAGVQFKA ELSLPRLSWQ SKSLPSDNLC TVTSFLDEAF
LKVLESALRF GNTLLIQDVE HLDPILNEIR RTGGRVLIRL GSQDIDFSPS FPSVEFSPDI
CSRVTSQSLD QVLKVETDLM KMQGEFRLRL RTLEKLLLQA LNESSGNILD DDKVIDTLET
LKREAAETDL VMKEVEEVTA EYLPIAQACS AVFFILEQLN LVNHFYQFSF LDIFDYILHH
NPNLRSVLMN DLFLVVYKRT SRALDELEFF LDSGLVTFAK HPLFKPVLTH LTEHEDEWVG
FLTVVKCLRP DRVLQSVPGY DASYRVENQE GFTLADQAVA MAARQGSWVL LKNVHLAPSW
LGEKKLQTLN PHRNFRLFLT MEANPSIPVN ILQSRILMNE PPPGIFLLAW FHAVVQEDMA
AAFNTIDTWL NSVAKGRANV DPASIPWDAI RTLIKQSVYG GRVDSDFDQK IIDAFVDGLF
TSAAYNVDFP EGTKLDHFLS WPSWLSLPPT AERRKMRMLA DDDNERCREW LEQLPANFQT
LQKQSAEHSD PLYRLFFREG SIGKLLTQVR RDLADVLMSS LTKGTIPDHW RRYKVHKISN
FARRLAQLDQ IAGLDNGGLF FPEAYITATR AVAHRKRWSL ETLRLQLDIE KVNDPGLVLE
GAAWASD

>PC
FEVEHLKYAT LATSANLATQ KQIAAIKTVR NIIEYNENYV TKRLLINIIW AFSGDAKGEW
VAWQTRVPQI EILYSWLSEH KPLMLCGPPG SGKTMTLFSA LRKLPDMEVV GLNFSSYRKT
PNGVILAPAQ IGRWLVVFCD EINLPATDKY GTQRVISLVE SGGYWRASDM AWVKLERIQF
VGACGRVPLS HRFLRLVMVD YPGEVSLKQI YGTYNRALLK VVPNLRAYAS PLTDAMVDLY

LASQKRFTTD IQAHYVYSPR ELTRWVRGIY EAIRPLEMLS VEGLVRVWAH EALRLFQDRL
VTEEEKQWTD EHIDSAAMEH FPTINILFSN WTSKNKARLK VFYEEELDVP LVLFNDVLDH
VLRIDRVFRQ TTLSRFVAWM NGLSIFQIKV SNKYTGDDFD LLANAEVPGL FEGFTMNPPE
NGLASRAATS PALFNRCVLD WFGDWLPSYN PPVHFPIAYR VVNALVYVHQ SLHQINQRLS
RRQGRYPRHY LDFINHYVRL YNEKRDELER HLHVGLDKLV TQVEELRKSL AIKRKLKRMV
ADQQEAEQKK AASIEIQAAL VEQDRHIEQR RAVVMADLAD AEQMTRQLRE LMKKEFLSRP
SYNFETVNRA SKACMISELE ASIERYKEEY AALIRVQSKV DRSMKLLESL SRTFETEMST
IVGDVLLSAA FLAYAEWSTH LADANIKFKT ELSLPRLSWQ SKSLPSDNLC IVTSFLDEAF
LKVLESALRF GNTLLIQDVE HLDPILNEIR RTGGRVLIRL GNQDIDFSPS FPSVEFSPDI
CSRVTSQSLD QVLKVETDLM KMQGEFRLRL RTLEKLLLQA LNESTGNILD DDKVIDTLET
LKREAAETDV VMKEVEQVTA EYLPIAQACS SVFFILEQLN LVNHFYQFSF LDIFDYVLHH
NPNLKSVLLK DLFLTVYKRT SRALEDLELL LESGLENFAK HPMFKPVIQH IVENEDQWAA
FLAIVKCLRP DRLLQSVTGY DASYRVENQE GFTLADQAIA TAARQGSWVL LKNVHLAPSW
LGEKKLQTLN PNRNFRLFLT METNPSIPVN ILQSRIFMNE PPPGIFLLAW FHAVVQEDLA
SAFNTIDAWL NAAAKGRANV DPASIPWDAV RTLIKQSVYG GRVDSDFDQK ILDAFVDGLF
TPYAYNVDFP DGTKLEHFLS WPAWLSLPPT AERRKMRMIA DDDFDRCKEW LGQLPTEFHG
LDKQSGDNQD PLYRLFYREG SIGKLLGQVR RDLADVLMSS LTKGTIPNHW RRYKVHKVSD
FARRLAQLTN IAGLGNGGLF FPEAYITATR AVAHRKKWSL ETLHLQLDIE RVNDPGLVLE
GASWTTD
>FP
FEVEHLRYAT LATSPHLVTQ KQIAGVKTVR NVIEYNEQYL EKRLLVSIIW AFSGDARGEW
FPWTSRVPVI DILYSWLSEH KPLMLCGPPG SGKTMTLFSA LRKLPDMEVV GLNFSSYRKT
PNGVMLAPAQ IGRWLVVFCD EINLPATDKY GTQRVISLVE ANGYWRTTDM AWVKLERIQF
VGACGRVPLS HRFLRLVMVD YPGELSLKQI YGTYNRAALK VLPNLRTYAE PLTDAMVALY
LASQKRFTTD SQAHYVYSPR ELTRWVRGIY EAIKPLETLT VEGLVRVWAH EALRLFSDRL
VTEEEKSWTD ESIDNVAMEN FPTINILFSN WTSKNKARLR IFYEEELDVP LVLFNDVLDH
VLRIDRVFRQ TTLSRFVAWM NGLNIFQIKV SNKYTGDDFD LLANAEVPGL FEGFTMNPPE
NGLASRAATS PALFNRCVLD WFGDWLPSYD PPAMFPTAYR VVNALVFVHQ SLHAINQRLS
RRQGRYPRHY LDFINHYVRL HNEKREELER HLHVGLDKLV TQVEELRKSL AIKRKLKRMV
ADQQEAEQKK AASIEIQAAL AEQDKHIEQR RAVVMADLAD AEQMTKHLRE LMKKDFVSRP
SFNFETVNRA SKACMVAELE ASIEQYKQEY AALIRVESKV NRSMTLLDSL SRTFDTEMST
IVGDVLLSAA FLAYGEWSAH LVEANVKFKT ELSMPRLSWQ SKGLPSDNLC TVTSFLDEAF
LKVLESALRF GNTLLIQDVE HLDPILNEIR RTGGRVLIRL GSQDIDFSPS FPSVEFSPDI
CSRVTSQSLD QVLKVETDLM KMQGEFRLRL RTLEKLLLQA LNESSGNILD DDKVINTLET
LKREAAETDL VMREVEQVTA EYLPLAQACS AVFFILEQLN LVNHFYQFSF LDIFDYVLHQ
NPNLKGVLLN DLFLVAYKRT SRALDELEFL LESGLESYSK HAIFKPVLEH VVQHENEWIT
LVLIIKCLRP DRLLQSVTGY DASYRVENQE GFTLADQAIA LAARQGSWVL LKNVHLAPSW
LGEKKLQTLN PHRNFRLFLT MEANPSIPVN ILQSRILMNE PPPGIFLLAW FHAVVQEDMA
SAFTTIDIWI NSVAKGRANV DPAAIPWDAL RTLVKQCVYG GRVDSDFDQK IIDAFVDGLF
TPAAYNVDFP DGTKLDHFLT WPAWLSLPPT AERRKMRMLS DDDHERCKEW LEQLPSTFHT
LEKQTSDNQD PLYRLFYREG TIGKLLSQVR RDLGDVLMSS LTKGTIPDHW RRYKVHRISD
FARRLAQLDQ IAGLDNGGLF FPEAYITATR AVAHRKKWSL ETLNLKLDID KVNDPGLVLE
GAKWDTD
>PP
FEVEHLRYAT LATSANLITQ KQVATIKTVR NVIEYNEQYV TKRLLVNIIW AFSGDARGEW
FGWQSRVPVI EILYSWLSEH KPLMLCGPPG SGKTMTLFSA LRKLPDMEVV GLNFSSYRKT
PNGVILAPVQ IGRWLVVFCD EINLPATDKY GTQRVISLVE AGGYWRASDM AWVKLERIQF
VGACGRVPLS HRFLRLVMVD YPGELSLKQI YGTYNRALLK VVPNLRTYAE PLTDAMVTLY
LASQKRFTTD IQAHYVYSPR ELTRWVRGIY EAIRPLEVLS VEGLVRVWAH EALRLFQDRL
VTEEEKQWTD ENIDTAAMEH FPTINILFSN WTSKNKARLR VFYEEELDVP LVLFNDVLDH
VLRIDRVFRQ TTLSRFVAWM NGLNIFQIKV SNKYTGEDFD LLANAEVPGL FEGFTMNPPA
NGLASRAATS PALFNRCVLD WFGDWLPSYN PPTMFPIAYR VVNALVFVHQ SLHQINQRLS
RRQGRYPRHY LDFINHYVRL HNEKRDELER HLHVGLDKLV TQVEELRKSL AIKRKLKRMV
ADQQEAEQKK AASIEIQAAL VEQDKHIAQR RAVVMTDLAD AEQMTKQLRD VMKRDFVSRP

SYTFETVQRA SKACMISELE ASIEQYKEEY AALIRVESKV NRSMKLLESL SRTFDTEMST
IVGDVLLSAA FLAYGDWSTH LAEANIKFKT ELSLPRLSWQ SKGLPSDNLC TVTSFLDEAF
LKVLESALRF GNTLLIQDVE HLDPILNEIR RTGGRVLIRL GSQDIDFSPS FPSVEFSPDI
CSRVTSQSLD QVLKVETDLM KMQGEFRLRL RTLEKLLLQA LNESTGNILD DDKVIDTLET
LKREAAETDL VMKEVEQVTA EYLPLAQACS AVFFILEQLN LVDHFYQFSF LDIFDYVLLH
NPNLKNVLTN DLFLMVYKRT SRALDEMEFL MESGLESYGK QPLFQPVVTH VLQHEDEWLQ
FLIIVKCLRP DRLLQSVPGY DASYRVENQE GFTLADQAIA FAARQGSWVL LKNVHLAPSW
LGEKKLQTLN PHRNFRLFLT MEANPSIPVN ILQSRILMNE PPPGIFLLAW FHAVVQEDMA
SAFNTIDAWI NAVAKGRANV DPAIIPWDAI RTLVKQCVYG GRVDSDFDQK ILDAFVDGLF
TPSAYNVDFP DGTKLDHFLS WPAWLSLPPT AERRKMRMLS DDDLERCREW LEQLPSNFNT
LQKQGGDNQD PLHRLFSREG SIGRLLTQVR RDLADVLMSA LTKGTIPDHW RRYKVHKISD
FSRRLAQLNN IAGLDNGGLF FPEAYVTATR AVAHRKKWSL ETLHLKLDID KVNDPGLVLE
GAAWESD
>WC
FEVEHLRYAT LATSANLATQ KQIAAIKTVR NVVEYNEQYV TKRLLVNIIW AFSGDARGEW
FAWQSRVPVI EILYSWLSEH KPLMLCGPPG SGKTMTLFSA LRKLPDMEVV GLNFSSYRKT
PNGVILAPVQ IGRWLVVFCD EINLPATDKY GTQRVISLVE SGGYWRTSDM AWVKLERIQF
VGACGRVPLS HRFLRLVMVD YPGELSLKQI YGTYNRALLK VVPSLRTYAE PLTDAMVTLY
LASQKRFTTD IQAHYVYSPR ELTRWVRGIY EAIRPLETLS VEGLVRVWAH EALRLFQDRL
VTEEEKRWTD ESIDSAAMEH FPTINILFSN WTSKHKARLR VFYEEELDVP LVLFNDVLDH
VLRIDRVFRQ TTLSRFVAWM NGLNIFQIKV SNKYTGDDFD LLANAEVPGL FEGFTMNPPE
NGLASRAATS PALFNRCVLD WFGDWLPSYN PPAMFPIAYR VVNALVYVHQ SLHQINQRLS
RRQGRYPRHY LDFINHYVRL HNEKREELER HLHVGLDKLV TQVEELRKSL AIKRKLKRMV
ADQQEAEQKK SASIEIQAAL VEQDKHIEQR RAIVMNDLAD AEQMTKHLRD TMKREFLSRP
SFNFETVQRA SKACMIAELE ASIERYKEEY AALIRVESKV NRSMKLLESL SRTFDTEMGT
IVGDVLLSAA FLAYGEWSSH LNDANIKLKA ELSLPRLSWQ SKGLPSDNLC TVTSFLDEAF
LKVLESALRF GNTLLIQDVE HLDPILNEIR KTGGRVLIRL GSQDIDFSPS FPSVEFSPDI
CSRVTSQSLD QVLKVETDLM KMQGEFRLRL RTLEKLLLQA LNESTGNILD DDKVIDTLET
LKREAAETDV VMREVEQVTA EYLSLAQACS AVFFILEQLN LVNHFYQFSF LDIFDYVLHH
NPRLANVLLN DLFLMVYKRT SRALEELEFL LESGLDSYGK HPLFSSVVTH VLQNEDQWIP
FLLIIKCLRP DRLLQSVTGY DASYRVENQE GFTLADQAIA FAARQGSWVL LKNVHLAPSW
LGEKKLQTLS PHRNFRLFLT MEANPSIPIN ILQSRILMNE PPPGIFLLAW FHAVVQEDMA
SAFTTIDTWV NTVAKGRANV DPAIIPWDAV RTLVKQCVYG GRVDSDFDQK ILDAFVDGLF
TSAAYNVDFP DGTKLEHFLS WPSWLSLPPT AERRKMRMLA DDDHERCKEW LGQLPSSFNT
LQRASGDNQD PLYRLFSREG SIGKLLSQVR RDLADVLMSS LTKGTIPNHW RRYKVHKISD
FARRLAQLDQ IARLDNGGLF FPEAYITATR AVAHRKKWSL ETLQLQLDID RVNDPGLVLE
GATWETD
>HI
FEVEHLRYAT LATSANIITQ KQVAGIKTAR NILEYNEQYV SKRLLISIIW AFSGDAKGEW
SAWAARVPQI EILYSWLSEH KPLMLCGPPG SGKTMTLFSA LRKLPDMEVV GLNFSSYRKT
PNGVILAPVQ LGRWLVVFCD EINLPAADKY GTQRVISLVE SGGYWRTSDM SWVKMERIQF
VGACGRVPLS HRFLRLVMVD YPGEVSLKQI YGTYNRGILK VVPNLRTYAE PLTDAMVAFY
LASQKRFTTD IQAHYVYSPR ELTRWVRGIY EAIRPLEVLS VEGLVRVWAH EALRLFQDRL
VAEDERQWTG DNIDNTALEH FPTINILFSN WTSKNKARLR VFYEEELDVP LVLFNDVLDH
VLRIDRVFRQ TTLSRFVAWM NGLSIFQIKV SNKYTGDDFD LLANAEVPGL FEGFTMNPPE
NGLASRAATS PALFNRCVLD WFGDWLSSYQ PPAMFPIAYR VVNALVHVHM SLYQINLRLS
RRQGRYPRHY LDFINHYVRL YNEKRDELER HLHVGLDKLV TQVEELRKSL AIKRKLKQMV
SDQQEAEQKK AASIEIQAAL VKQDKHIEQR RSIVMADLAD AEQMTKQLRE VMKKDFLSRP
SFNFETVQHA SKACMISELE ASITKYKEEY AFLIRVQGKV DRSMKLLESL SRTFDAEMST
IVGDVLLSAA FLAYGEWMNH LVEANIKFKP ELSFTRLSWQ SKGLPSDNLC TVTSFLDEAF
LKVLESALRF GNPLLIQDVE HLDPILNEIR RTGGRVLIRL GSQDIDFSPS FPSVEFSPDI
CSRVTSQSLD QVLKVETDLM KVQGEFRVRL RTLEKLLLQA LNESSGNILD DDKVIDTLET
LKREAAETDL VMREVEQVTA EYLPLAQACS SVFFILEQLN LVNHFYQFSF LDIFDYVLHH
NPNLHGILLK DLFLVVYRRT SRALDELEFL LESGLDTYAK QSLFKPVQAH ILEHESDWIP

FLLIIKCLRP DRLLQSVAGY DASYRIDNQE GFALADQAIG VASRQGTWVL LKNVHLAPQW
LGEKKLQTLN AHRNFRLFLT MEANPSIPVN ILQSRVIMNE PPPGIFLLAW FHAVVQEDMA
AAFTTIDAWL HSIAKGRANV DPEQIPWDAL RTLIKQSVYG GRVDSDFDQK IIDAFVDGLF
TSAAYNVDFP EGTKLDHFLS WPSWLSLPPT AERRKMRTLT DDDLERCREW LTQLPSTFNV
LQKQSGDNSD PLYRLFSREG SIGKLLNQVR KDLIDVLMSS LTKGTIPDHW RRYKVKKIPD
LGLRLAQLDN LATLDNGGLF FPEAYITATR AVAHRKKWSL ETLHMRLDIE RVNDPGLALE
GASWIEE
>SH
FEVEHLRYAT LATSANLMTQ RQVAGLKTVR NILEYNEQYV SKRLLVSIIW AFSGDSKGEW
SAWAAKVPTI EILYSWLSEH KPLMLCGPPG SGKTMTLFSA LRKLPDMEVV GLNFSSYRKT
PNGVILAPIQ LGRWLVVFCD EINLPAADKY GTQRVISLVE CGGYWRTTDM AWVKLERIQF
VGACGRVPLS HRFLRLVMVD YPGEVSLKQI YGTYTRALLK VVPNLRGHAE PLTDAMVSFY
LASQKRFTTD VQAHYVYSPR ELTRWVRGIY EAIRPLELLS VEGLVRVWAH EALRLFQDRL
VSEDEKQWTD DNIDMTAMEH FPTIDILFSN WTSKHKARLR VFYEEELDVP LVLFNDVLDH
VLRIDRVFRQ TTLSRFVAWM NGLSIFQIKV SNKYTGDDFD LLANAEVPGL FEGFTMNPPE
NGLASRAATS PALFNRCVLD WFGDWLSSYS PPAIFPIAYR VVNALVHVHM SLYNINQRLS
RRQGRYPRHY LDFINHYVRL YSEKRDELER HLHVGLDKLV TQVEDLRKSL AIKRKLKQMV
SDQQEAEQKK AASIEIQAAL VEQDKHIAQR RNVVMADLAD AEQMTKQLRD LMKKDFLSRP
SFNFETVNRA SKACMIAELE ASITRYKDEY ALLIRVEGKV DRSMKLLGSL SRTFDAEMST
IVGDVLLSAA FLAYGEWMSH LVDANIKFKP ELSFTRLSWQ SKGLPSDNLQ TVTSFLDEAF
LKVLESALRF GNPLLIQDVE HLDPILNEIR RTGGRVLIRL GSQDIDFSPS FPSVEFSPDI
CSRVTSQSLD QVLKVETDLM KVQGEFRVRL RTLEKLLLQA LNESTGNILD DDKVIDTLET
LKREAAETDI IMREVEQVTA EYLPLAQACS AVFFILEQLN LVNHFYQFSF LDIFEFILHH
NPNLQGVLMK DLFLTVFKRT SRALDELEFL LESGLEVYAK QPLFRPVQNH IAEHEEEWAT
FLLVIKCLRP DRLLQSVTGY DASYRVESQE GFSLADQAIA TASRQGTWVL LKNVHLAPSW
LGEKKLQTLN AHRNFRLFLT MEANPSIPVN ILQSRIIMNE PPPGIFLLAW FHAVTQEDMA
SAFTTIDAWC RSVAKGKANI DPALIPWDAL RTLIKQSVYG GRVDSDFDQR ILDAFVDNLF
TPAAYNVDFP EGTKIDQFLA WPSWLSLPPT AERRKMRTLA DDDLDKCREW LAQLPSSFNV
LTKQTGDNQD PLYRLFAREG SIGNLLGQVR KDLADVLMSS LTKATIPVHW RRYKVKRIPD
LARRLAQLDN LASLDTGGLF FPEAYITATR AVAHRKKWSL ETLHLRLDIE RMNDPGLVLE
GASWAED
>PS
FEVEHLRYAT LATSANLTTQ QQVAALKTVR NIVEYNEQYA TKRLLLNIIW AFSGDAKGEW
TPWQGKVPVI EILYSWLSEH KPLLLCGPPG SGKTMTLFSA LRKLPDMEVV GLNFSSYRKT
PNGVVLAPVQ IGRWLVVFCD EINLPAADKY GTQRVISLVE CNGYWRASDK AWVKLERIQF
VGACGRVPLS HRFLRLVMVD YPGELSLKQI YGTYIRGALK VVPNLRAYSE PLTNAMVDFY
LASQSRFTTD AQAHYVYSPR ELTRWVRGVY EAIRPLEILS VEGLVRVWAH EALRLFQDRL
VTEEERRWTD EQIDNIAMEH FPTINILFSN WTSKYKARLR VFYEEELDVP LVLFNDVLDH
VLRIDRVFRQ TTLSRFVAWM NGLSIFQIKV SNKYTGDDFD LLANAEVPGL FEGFTMNPPE
NGLASRAATS PALFNRCVLD WFGDWLASYT PPIHFPIAYR VVNALVFVHQ SMHQINQRLS
RRQGRYPRHY LDFIHHYVRL YNEKRNELER HLHVGLDKLV EQVEELRKSL AIKRKLKQMV
TDQQEAESKK KASIEIQAAL VEQDKHIAQR REIVMADLAD AEQMTKAHRD LMKKDFLSRP
SFNFETVNRA SKACMISELE ASIQKYKEEY ALLIRVQGKV DRSMKLLESL SRTFDAEMST
IVGDVLLSAA FLAYGEWSNH LTEANIKFKP ELSFARLSWQ SKSLPSDSLC TVTSFLDEAF
LKVLESALRF GNPLLIQDVE HLDPILNEIR RTGGRVLIRL GNQDIDFSPA FPSVEFSPDI
CSRVTSQSLD QVLKVETDLM KIQGEFRLRL RTLEKLLLQA LNESSGNILD DDKVIDTLET
LKREAAETDV VMKEVEQVTA EYLPLAQACS SVYFILEQLN LVNHLYQFSF LDIFDYVLHQ
NPHLKGILLN DLFLVVYQRT SRALDELEFL LESGLQTFAK DEIFKPVKKH IQENESDWIP
FLLVIKCLRP DRLLQSVPGY DASYRVENQE GFTLADQAIS LAARQGSWVL LKNVHLAPAW
LGEKKLQTLN PHRNFRLFLT MEANPSIPVN ILQSRLIMNE PPPGIFLLAW FHAIVQEDMR
SACNTIDIWL NAAARGRANI DPAAIPWDAV RALIKQAVYG GRVDSDFDQR VLDAFVDGLF
TPSAYNVDFP DGTKIEHFLS WPSWLSLPPT AERRKMRTLE DDDHERCKEW LALLPANFNT
LSKPTTENQD PLYRLFHREG MIGKLLSQVR RDLTDVLMSE LTKGAIPTHW KRYKVQKIPN
LARRLGQLDH IAGLENGGLF FPEAYITATR AVAHRKKWSL ETLRLYLDIE QINDPGLVLE

GANWSTD
>GT
FEVEHLRYAT LATSANLIAQ KQIAAVKTVR NVIEYNEQYV TKRLLVNIIW AFSGDAKAEW
TAWASKVPVI DILYSWLSEH KPLLLCGPPG SGKTMTLFSA LRKLPDLEVV GLNFSSYRKT
PNGVVLAPTQ IGRWLVVFCD EINLPATDKY GTQRVISLVE CNGFWRTSDM SWVKLERIQF
VGACGRVPLS HRFLRLVMVD YPGELSLKQI YGTYTRAALK VVPNLRGHAE PLTDAMVEFY
LASQKRFTTD VQAHYVYSPR ELTRWVRGIY EAIRPLEMLT VEGLVRVWAH EALRLFQDRL
VTEEEKQWTD DNIDLVAMQH FPTINILFSN WTSKNKARLR VFYEEELDVP LVLFNDVLDH
VLRIDRVFRQ TTLSRFVAWM NGLSIFQIKV SNKYTGDDFD LLANAEVPGL FEGFTMNPPQ
NGLASRAATS PALFNRCVLD WFGDWLPAYN PPAHFPIAYR VVNALVFVHM SMYAINQRIS
RRQGRYPRHY LDFINHYVRL FNEKRNELER HLHVGLDKLV TQVEELRKSL AIKRKLKRMV
TDQQEAEQKK AASIEIQAAL VEQEKHIEQR RAVVMADLAD AEQMTKQIRE LMKKDFLSRP
SFNFETVNRA SKACMIAELE AKIQTYKEEY ALLIRVQSKV DRSMKLLDSL SRTFDVEMST
IVGDVLLSAA FLAYGEWASH LGEAGVKFKQ ELSFPRLSWQ SKSLPSDNLC TVTSFLDEAF
LKVLESALRF GNPLLIQDVE HLDPILNEIR RTGGRVLIRL GNQDIDFSPS FPSVEFSPDI
CSRVTSQSLD QVLKVETDLM KVQGEFRLRL RTLEKLLLQA LNESTGNILD DDKVIDTLET
LKREAAETDL VMREVEQVTA EYLPLAQACS SVFFILEQMN LVNHFYQFSF LDIFDYVLHQ
NPNLKNVLLN DLFLMVYKRT SRALDELEFL LESGLQSFSK HSIFKPVQSH ISEHENEWIP
FLLIIKCLRP DRLLQSVPGY DASYRVDNQE GFSLADQAIA GAARQGSWVL LKNVHLAPSW
LGEKKLQTLN PNRNFRLFLT MEANPSIPVN ILQSRIIMNE PPPGIFLLAW FHAIIQEDMI
SAFNTIDAWL GAAARGRANI DPAAIPWDAL RTLIKQSVYG GRVDSDFDQR TLDAFVDGLF
TPAAYNVDFP DGTKLEHFLS WPSWLSLPPT AERRKMRQLA DDEHDRCQEW LSYLPSSFNT
LSKPSTENQD PLYRLFFREG TIGKLLEQVR RDLEDVLMSS LTKGTIPDHW RRYKVHKIPN
LARRLGQLDK IAGLDSGGLF FPEAYITATR AVAHRKKWSL ETLNLRLDIE RIDDPGLVLE
GAAWAQD
>SC
FEVEHLKYAT LATTANLATQ QQMADMKTAR NVIEYNEQYV TKRLLLSIIW AFSGDAKGEW
IQWQSKVPVI EILYSWLSEH KPLMLCGPPG SGKTMTLFSA LRKLPDMEVV GLNFSSYRKT
PKGIILAPVQ IGRWLVVFCD EINLPAADKY GTQRVISLVE HGGYWRTSDM AWVKLERIQF
VGACGRVPLS HRFLRLVMVD YPGEVSLKQI YGTYNRAALK VVPNLRTYAE PLTDAMVAFY
LASQKRFTTD IQAHYVYSPR ELTRWVRGIY EAIRPMEVLS VEGLVRVWAH EALRLFQDRL
VSEDERQWTD EHIDNAAMEY FPTINILFSN WTSKNKARLR VFYEEELDVP LVLFNDVLDH
VLRIDRVFRQ TTLSRFVAWM NGLSIFQIKV SNKYTGDDFD LLANAEVPGL FEGFTMNPPE
NGLASRAATS PALFNRCVLD WFGDWLPSYS PPAIFPIAYR VVNALVHVHL SLHAINQRLS
RRQGRYPRHY LDFINHYVRL YSEKRDELER HLHVGLDKLV TQVEELRKSL AIKRKLKRMV
SDQQEAEQKK AASIELQAAL VEQDKHIEQR RAVVMADLAD AEQMTKHLRD IMKRDFLSRP
SYNFETVQRA SRACLIAELE GKIAKYKEEY ALLIRVQSKV DRSMKLLESL SRTFDAEMST
IVGDVLLSAA FLAYGGWSNH LSEANIKFKP ELSFTRLSWQ EKSLPSDTLT TVTSFLDEAF
LKVLESALRF GNPLLIQDVE HLDPILNELR RTGGRVLIRL GSQDIDFSPA FPSVEFSPDI
CSRVTSQSLD QVLKVETDLM KVQGEFRLRL RTLEKLLLQA LNESTGNILD DDKVIDTLET
LKREAAETDV VMKEVEEVTV EYLPLAQACS AVFFVLEQLN LVNHFYQFSF LDIFDYILHH
NPHLKGVLMN DLFLIVYKRT SRALDEAEFL LESGLEAYAK SSIFKPVQNH FIENENQWAA
FLLIIKCFRP DRLLQSVPGY DASYRVENQE GFSLADQAIA AASRQGTWVL LKNVHLAPSW
LGEKKLQTLN PHRNFRLFLT MEANPSIPTN ILQSRLIMNE PPPGIFLLAW FHAIVQEDMA
AAFGTIDAWL VAASKGKANV DPATIPWDAL RTLVKESVYG GRVDSDFDQR IVNSFVDGLF
TPAAYNIDFP EGTKMEHFLT WPSWLSLPPT AERRKMRMLA DDDLDRCREW LGHLPEKFNV
LERPTGDNQD PLHRLFSREG NIGNLLTQVR RDLSDVLMSA LTKGTIPDHW RRYKVHHIAD
FSRRLAQLDH IAGLDNGGLF FPEAYITATR AVAHRKKWSL ETLNLRLDIE RVNDPGLVLE
GASWSTD
>CC
FEVEHLKYAT LATAAHLQTQ KEIAGIKTVR NVIEYNEQYV SKRLLVSLIW AFSGDAKGEW
IAWQSRVPDI EILYSWLSEH KPLMLCGPPG SGKTMTLFSA LRKLPDMEVV GLNFSSFRKT
PNGVVLAPIQ IGRWLVVFCD EINLPASDKY GTQRVISLVE SGGFWRTSDM AWVRLERIQF
VGACGRVPLS HRFLRLVMVD YPGEVSLKQI YGTYNRALLK VVPNLRTYAG PLTDAMVAFY

LASQKRFTTD AQAHYVYSPR ELTRWIRGIY EAIKPLEILS VEGLVRVWAH EALRLFQDRL
VTEEERNWTD EHIDGVAMEH FPTINILFSN WTSKNKARLR VFYEEELDVP LVLFNDVLDH
VLRIDRVFRQ TTLSRFVAWI NGLSIFQIKV SNKYTGDDFD LLANAEVPGL FEGFTMNPPE
NGLASRAATS PALFNRCVLD WFGDWLPSYN PPANFPIAYR VVNALVYVHS SLHQINQRLS
RRQGRYPRHY LDFINHYVRL YTEKRDELER HLHVGLDKLV IQVEELRKSL AVKRKLKRMV
SDQQEAEQKK AASIEIQAAL VEQDRHIEQR RAVVMADLAD AEQMTKQLRE LMKKDFLSRP
SYNFETVNRA SKACMIAELE SKIAQYKDEY ALLIRVQSKV DRSMKLLESL SRTFDAEMST
IVGDVLLSAA FLAYGEWSNH LTEANIKFKP ELSFTRLSWQ SKSLPSDNLT TVTSFLDEAF
LKVLESALRF GNPLLIQDVE HLDPILNEIR RTGGRVLIRL GSQDIDFSPS FPSVEFSPDI
CSRVTSQSLD QVLKVETDLM KVQGEFRLRL RTLEKLLLQA LNESTGNILD DDKVIDTLET
LKREAAETDL IMKEVEQVTA EYLPLAQACS SVFFVLEQLN IINHFYQFSF LDIFDYVLLH
NPHLKSILLN DLFLVVYKRT SRALDELEFL LESGLELFSK HSIFKPVQNH VLNNEDTWIN
FLIVMKCFRP DRLLQSVPGY DASYRVENQE GFTLADQAIA AASRQGTWVL LKNVHLAPSW
LGEKKLQTLN PHRSFRLFLT MEMNPSIPVN ILQSRLIMNE PPPGIFLLAW FHAIVQEDMA
SASRTIDAWL DAASRGKANI DPAAIPWEAL RTLIKESVYG GRVDSDFDQR ILDSFVNSLF
TPAAYNVDFP DGTKLEHFLS WPSWLSLPPT AERRKMRMLA DDDLERCREW LALLPSKFNV
LPPQSADSQD PLYRLFAREG SIGRLLDQVS RDLADVLISA LTKGTIPEHW KRYKVPKIAN
FARRLQQLDT VAGLDNGGLF FPEAFITATR AVAHRKKWSL ETLSLCLDIE RVNDPGLALE
GAAWASD
>CP
FEVEHLRYAT LATSAHLETQ KQIASNKTAR NIIEYNEQYV TKRLLVSIIW AFSGDAKGEW
VAWQSKVPVI EILYSWLSEH KPLMLCGPPG SGKTMTLFSA LRKLPDMEVV GLNFSSYRKT
PNGVILAPVQ IGRWLVVFCD EINLPAQDKY GTQRVISLVE CGGYWRSSDM AWVTLERIQF
VGACGRVPLS HRFLRLVMVD YPGELSLKQI YGTYSRALLK VVPTLRAYAE PLTDAMVAFY
LDSQKRFTAD IQAHYVYSPR ELTRWVRGIY EAIRPLEILS VEGLVRVWAH EALRLFQDRL
VTEEERQWTD EHINNAAMEN FPTINILFSN WTSKNKARLK VFYEEELDVP LVLFNDVLDH
VLRIDRVFRQ TTLSRFVAWL NGLSIFQIKV SNKYTGDDFD LLANAEVPGL FEGFTMNPPE
NGLASRAATS PALFNRCVLD WFGDWLPSYS PPDHFPIAYR VVNALVYVHS SLHAINQRLS
RRQGRYPRHY LDFINHYVKL YNEKREELER HLHVGLDKLV TQVEELRKSL AIKRKLKRMV
SDQQEAEQKK AASIEIQAAL VEQDKHIAQR KEVVMADLAD AEQMTKPLRD LMKREFLSRP
SYNFEMVQRA SKACMISELE SKIATYKDEY ALLIRVQNKV DRSMKLLESL SRTFDAEMST
IVGDVLLSAA FLAYGDWSGH LSEANVKFKS ELSLTRLSWQ SKSLPADNLT TVTSFLDEAF
LKVLESALRF GNPLLIQDVE NLDPILNEIR RTGGRVLIRL GNQDIDFSPS FPSVEFSPDV
CSRVTSQSLD QVLKVETDLM KAQGEFRLRL RTLEKLLLQA LNESTGNILD DDKVISTLET
LKREAAETDV VMREVEEVTA EYLPLAQACS AVFFILEQLN LVNHFYQFSF LDIFDYVLHH
NPNLAGVLLS DLFVVVFKRT SRALDELEYL LEAGLEHYAK NSIFKPVLNH VMERPEEWAP
FILIIKCLRP DRLLQSVTGY DASYRVENQE GFALADQAIA AAARQGTWVL LKNVHLAPTW
LGEKKLQTLN PHRNFRLFLT MEANPSIPVN ILQSRLIMNE PPPGIFLLAW FHAVVQEDMA
SAFGTIDTWL NSVAKGRANV DPATIPWDAL RTLIKQSVYG GRVDSEFDQR ILDSFVDKLF
TPMAYNVDFP DGTKVEHFLD WPSWLSLPPT AERRKMRMLS DDDFERSREW LAQLPSKLNV
LPKQASEKSD PLYRFFSREG TIGKLLGQVR KDLESVLMSS LTKGTIPNHW RKYKVPKIPD
LSRRLAQLEE IVTMQQGGLF FPEAYITATR AVAHRKRWSL ETLHLRLEIG EVKNPGLSLE
GASWNVE
>SL
FEVEHLRYAT LATSANLITQ TQVASIKTVR NIIEYNEQYV TKRLLVSIIW AFSGDAKGEW
TAWQSKVPVI EILYSWLSEH KPLMLCGPPG SGKTMTLFSA LRKLPDMEVV GLNFSSYRKT
PNGVILAPVQ IGRWLVVFCD EINLPAQDKY GTQRVISLVE CGGYWRASDM AWVKLERIQF
VGACGRVPLS HRFLRLVMVD YPGEISLKQI YGTYNRALLK VVPTLRAYSE PLTDAMVAFY
LASQKRFTTD IQAHYVYSPR ELTRWVRGIY EAIRPLEMLS VEGLVRVWAH EALRLFQDRL
VTEEEKHWTD EHIDNAALEH FPTINILFSN WTSKNKARLK IFYEEELDVP LVLFNDVLDH
VLRIDRVFRQ TTLSRFVAWL NGLSIFQIKV SNKYTGDDFD LLANAEVPGL FEGFTMNPPE
NGLASRAATS PALFNRCVLD WFGDWLPSYS PPAHFPIAYR VVNGLVYVHS SLHQINQRLS
RRQGRYPRHY LDFINHYVRL YNEKRDELER HLHVGLDKLV TQVEELRKSL AIKRKLKRMV
ADQQEAEQKK AASIEIQAAL VEQDNHIAQR RAVVMADLAD AEQMTKPLRD LMKRDFVSRP

SYNFETVQRA SKACMISELE AKIGKYKEEY ALLIRVQSKV DRSMKLLESL SRTFDAEMST
IVGDVLLSAA FLAYGEWSNH LSEANITFKP ELSLVRLSWQ SKSLPSDNLT TVTSFLDEAF
LKVLESALRF GNPLLIQDVE HLDPILNEIR RTGGRVLIRL GSQDIDFSPS FPSVEFSPDI
CSRVTSQSLD QVLKVETDLM KVQGEFRLRL RTLEKLLLQA LNESTGNILD DDKVIGTLET
LKREAAETDV IMKEVEQVTS EYLPLAQACS SIFFILEQLN LVNHFYQFSF LDIFDYVLHH
NPNLKNVLLS DLFLIVYKRT SRALDELEFL LESGLENYAK QALFRPVLTH ITERENDWVP
FLLIIKCLRP DRLLQSVPGY DASYRVELQE GFTLADQAIA AASRQGSWVL LKNVHLAPTW
LGEKKLQTLN PHRNFRLFLT MEANPSIPVN ILQSRIIMNE PPPGIFLLAW FHAVVQEDMA
SAFGTIDTWL NAIAKGRANV DPAIIPWDAL RTLIKQSVYG GRVDSDFDQR ILDTFVDGLF
TPTAYNVDFP DGTKLEHFMT WPSWLSLPPT AERRKMRMLA DDDHDRCREW SSQLPATFNT
LPKQSNDNPD PLYRLFFREG SIGKLLDQVR KDLADVLMSS LTKGTIPSHW RRYKVPKIPD
LARRLAQLDG ITSMDNGGLF FPEAYVTATR AVAHRKRWSL ETLNLRLDIG RVNDPGLVLE
GAAWATD

>LB

FEVEHLKYAT LATSANLETQ KQIASIKTVR NVIEYNEQYV TKRLLISIIW AFSGDAKGDW
IAWQSRVPVI EILYSWLSEH KPLMLCGPPG SGKTMTLFSA LRKLPDLEVV GLNFSSYRKT
PNGVILAPIQ IGRWLVVFCD EINLPAADKY GTQRVISLVE CGGYWRASDM AWVKLERIQF
VGACGRVPLS HRFLRLVMVD YPGEISLKQI YGTYNRALLK VVPNLRAYAE PLTDAMVSFY
LASQKRFTTD VQAHYVYSPR ELTRWVRGIY EAIRPLEILS VEGLVRIWAH EALRLFQDRL
VSEEERLWTD ENIDAAALEH FPTINILFSN WTSKNKARLR VFYEEELDVP LVLFNDVLDH
VLRIDRVFRQ TTLSRFVAWM NGLSIFQIKV SNKYTGDDFD LLANAEVPGL FEGFTMNPPE
NGLASRAATS PALFNRCVLD WFGDWLPSYG PPAHFPVAYR VVNALVHVHS SLHPINQRLS
RRQGRYPRHY LDFINHYVRL YTEKRDELER HLHVGLDKLV TQVEELRKSL AIKRKLKRMV
ADQQEAEQKK AASIEIQAAL VEQDRNIEQR RAVVMADLAD AEQMTRQLRD TMKKDFLSRP
SYNFETVQRA SKACMIAELE SKIARYKDEY ALLIRVQSKV DRSMKLLESL SRTFEAEMST
IVGDVLLSAA FLAYGEWSNH LAEANIKFKP ELSFTRLSWQ SKSLPSDNLT TVTSFLDEAF
LKVLESALRF GNPLLIQDVE HLDPILNEIR RTGGRVLIRL GSQDIDFSPS FPSVEFSPDI
CSRVTSQSLD QVLKVETDLM KVQGEFRLRL RTLEKLLLQA LNESSGNILD DDKVIDTLET
LKREAAETDV VMREVEQVTA EYLPLAQACS SVFFVLEQLN LANHFYQFSF LDIFDYILHH
NPNLKNVLLK DLFLVVYKRT SRALDELEFL LESGLNNFAK QTLFKPVQAH ITEHEDHWIP
FLLIMKCFRP DRLLQSVPGY DASYRVENQE GFTLADQAIA AAARQGTWVL LKNVHLAPSW
LGEKKLQTLN PHRNFRLFLT MEANLSIPVN ILQSRLIMNE PPPGIFLLAW FHAVVQEDMA
SAFSTIDAWL NSVSKGRANI DPASIPWDAL RTLVKQSVYG GRVDSDFDQR ILNSFVDGLF
TAQAYNVDFP DGTKMEHFLT WPSWLSLPPT AERRKMRMLA DDDLDRCREW LSQLPSEFNT
LTPQSAEHQD PLYRLFSREG SIGKLLDQVR KDLTDVLMSA LTKGQIPEHW RRYKVNKIAN
FSRRLSQLDS IAKLDKGGLF FPEAYVTATR SVAHKKKWSL ETLTLRLDLE RVNDPGLALE
GASWSTN

>FS

FEVEHLRYAT LATSANLTTQ KEIVTIKTVR NVTEYNEQYV EKRLLVSIIW AFSGDAKGEW
IAWQTRVPVI EILYSWLSEH KPLLLCGPPG SGKTMTLFSA LRKLPDMEVV GLNFSSYRKT
PNGVILAPVQ IGRWLVVFCD EINLPAADKY GTQKVISLVE CGGFWRASDM SWVKLERIQF
VGACGRVPLT HRFLRLVMVD YPGEVSLKQI YGTYNRAVLK VVPSLRAYAE PLTDAMVSFY
LASQKRFTTD IQAHYVYSPR ELTRWIRGIY EAIRPLEVLS VEGLVRVWAH EALRLFQDRL
VNDEERLWTD EHIDAAALEH FPTINILFSN WTSRNKARLR VFYEEELDVP LVLFNDVLDH
VLRIDRVFRQ TTLSRFVAWM NGLSIFQIKV SNKYTGEDFD LLANAEVPGL FEGFTMNPPQ
NGLASRAATS PALFNRCVLD WFGDWLASYN PPTNFPIAYR VVNALVYVHS SLHQINQRLS
RRQGRYPRHY LDFINQYVRL YNEKRDELER HLHVGLDKLV TQVEELRKSL AIKRKLQRMV
ADQQEAEQKK AASIEIKAAL REQDMHIEQR RAVVMADLAD AEQMTKQLRE TMKKDFLSRP
SYNFEAVQRA SKACMIAELE AKITQYKDEY ALLIRVQGKV ERSMKLLESL SRTFDAEMST
IVGDVLLSAA FLAYGEWSNH LAEASITFKP ELSLTRLSWQ SKSLPADNLT TVTSFLDEAF
LKVLESALRF GNPLLIQDVE HLDPILNEIR RTGGRVLIRL GNQDIDFSPS FPSVEFSPDI
CSRVTSQSLD QVLKVETDLM KVQGEFMLRL RTLEKLLLQA LNESTGNILD DDKVIDTLET
LKREAAETDV VMKEVEEVTA EYLPLAQACS SVFFILEQLN LVNHFYQFSF LDIFDYILLH
NPNLKNILLK DLFLVVYKRT SRALDELEFL LESGLEGYAK QSLFKPVQNH ITEHESEWVP

FLLIVKCLRP DRLLQSVPGY DASYRVENQE GFALADQAIA AASRQGSWVL LKNVHLAPSW
LGEKKLQTLN PHRNFRLFLT MEANPSIPVN ILQSRIIMNE PPPGIFLLAW FHAIVQEDMA
AAFGTIDTWL NAVAKGRANI DPAAIPWDAL RTLVKQSVYG GRVDSDFDQR ILDAFVDTLF
TPSAYNVDFP DGTKIEHFLS WPAWLSLPPT AERRKMRMLQ DDDLERCREW LSQLPSVFNI
LSKQSGDNQD PMYRLFSREG SVGKLLGQVQ KDLADVLMSS LTKGTIPTHW RRYKVNKIPN
LARRLVQLNE IAGLDNGGLF FPEAYITATR AVAHRKRWSL ETLTMRLDIE KVNDPGLALE
GAAWSTD
>PI
FEVEHLRYAT LATSANLLTQ KEIAAIKTVR NIIEYNEQYV TKRLLVSIIW AFSGDAKGEW
VAWQTRVPVI EILYSWLSEH KPLMLCGPPG SGKTMTLFSA LRKLPDMEVV GLNFSSYRKT
PNGVILAPVQ IGRWLVVFCD EINLPAADKY GTQKVISLVE CGGYWRASDM SWVKLERIQF
VGACGRVPLS HRFLRLVMVD YPGEVSLKQI YGTYNRAVLK VVPSLRAYAE PLTDAMVAFY
LASQKRFTTD VQAHYVYSPR ELTRWARGIY EAIRPLEVLP VEGLVRVWAH EALRLFQDRL
VNEEERLWTD ENIDAAALEH FPTINILFSN WTSKNKARLR VFYEEELDVP LVLFNDVLDH
VLRIDRVFRQ TTLSRFVAWM NGLSIFQIKV SNKYTGEDFD LLANAEVPGL FEGFTMNPPE
NGLASRAATS PALFNRCVLD WFGDWLPSYN PPAHFPIAYR VVNALVYVHS SLHQINQRLS
RRQGRYPRHY LDFINHYVRL YNEKRDELER HLHVGLDKLV TQVEELRKSL AIKRKLKRMV
ADQQEAEQKK AASIEIQAAL VKQDKHIEQR RAIVMADLAD AEQMTKLIRE TMKKDFLSRP
SYNFETVQRA SKACMISELE AKIDKYKEEY ALLIRVQSKV DRSMKLLESL SRTFDAEMST
IVGDVLLSAG FLAYGEWSNH LAEASIKFKP ELSLTRLSWQ SKSLPADNLT TVTSFLDEAF
LKVLESALRF GNPLLIQDVE HLDPILNEIR RTGGRVLIRL GSQDIDFSPS FPSVEFSPDI
CSRVTSQSLD QVLKVETDLM KVQGEFRLRL RTLEKLLLQA LNESSGNILD DDKVIDTLET
LKREAAETDV VMKEVEQVTA EYLPLAQACS SVFFILEQLN LVNHFYQFSF LDIFDYVLHH
NPNLKNVLVN DLFLVVYKRT SRALDELEFL LESGLESYSK QPIFKPVQNH IMEHESDWIP
FLLIIKCLRP DRLLQSVPGY DASYRVENQE GFTLADQAIA TASRQGSWVL LKNVHLAPSW
LGEKKLQTLN PHRNFRLFLT MEAQPSIPVN ILQSRILMNE PPPGIFLLAW FHAVVQEDMA
SAFGTIDTWL NSVAKGRANV NPATIPWDAL RTLIKQSVYG GRIDSDFDQR ILDAFVDNLF
TPSAYNVDFP DGTKMEHFLS WPSWLSLPPT AERRKMRMLA DDDLDRCREW LSQLPSTFNT
LVKQSGENQD PMYRLFFREG SVGKLLGQVR KDLADVLMSS LTKGTIPTHW RRYKVTKIPN
LARRLAQLND IADLENGGLF FPEAYITATR AVAHRKRWSL ETLNMRLDIE KVNDPGLALE
GAAWSTD
>SS
FEVEHLKYAT LATSPNLEVQ RSIATIKTVR NVIEYNEQYV TKRLLISIIW AFSGDAKGEW
LPWQSKVPVI EILYSWLSEH KPLLLCGPPG SGKTMTLFSA LRKLPDMEVV GLNFSSYRKT
PNGVILAPIQ IGRWLVVFCD EINLPAPDKY GTQRVISLVE SGGYYRTLDM AWVKLERIQF
VGACGRVPLS HRFLRLVMVD YPGEVSLKQI YGTYNRAMLK VVPNLRAYAE PLTDAMVAFY
LASQKRFTTD VQAHYVYSPR ELTRWVRGIY EAIKPLDILS VEGLVRVWAH EALRLFQDRL
VTEEEKQWTD ENIDAVAMEN FPTINILFSN WTSKNKARLR VFYEEELDVP LVLFNDVLDH
VLRIDRVFRQ TTLSRFVAWM NGLSIFQIKV SNKYTGDDFD LLANAEVPGL FEGFTMNPPE
NGLASRAATS PALFNRCVLD WFGDWLPSYT PPINFPIAYR VVNALVHVHQ SMHHFNTRLS
RRQGRYPRHY LDFLHHYVRL FTEKRDELER HLHVGLDKLV TQVEELRKSL AIKRKLRRMV
ADQQEAEQKK AASIEIQAAL AEQERSIEQR RAVVLADLAN AEQLTKPLRE FMKKEYLSRP
SYNYEVVNRA SKACMVAELE TSIKRYKEEY AFLIRVQSKV DRSMTLLESL SKTFETEMST
IVGDCLLSAA FLAYGEWCAH LTDANTKFKP ELSLTRLSWQ SKSLPSDTLC TVTSFLDESF
LKVLESALRF GNALLIQDVE NLDPILNEIR RTGGRVLIRL GNQDIDFSPS FPSVEFSPDI
CSRVTSQSLD QVLKVETDLM KVQGEFKLRL RTLEKLLLQA LNESTGNILD DDKVIDTLEK
LKREAAETDI IMKEVEEVTA EYIPLAQACS SIFFILEQMN LVNHFYQFSF LDIFDYVLHQ
NPNLKNILMD DLFLVVYQRT SRALDEMELL LESGLQAYAR SSMFKGIVPH LAEHQADWMK
FLLLIKCFRP DRLLQSVTGY DASYRVENQE GFTQADQAIA GAARQGYWVL LKNVHLAPSW
LGEKNLQTLN PHRNFRVFLT METNPSIPVN LLQSRIFMNE PPPGIFLLAW FHAIVQEDMA
AAFNTIDIWL NSVAKGKANV DPASIPWDAV RTLVKQSVYG GRIDSDFDQK VLDAFVDRLF
TPFSYNVDFP EGTKMEHFLA WPSWLSLPPT AERRKMRTLV DDDLQHCREW LSSLPETLNA
LQKRSGDNSD PLFRFFSREA SVGKLLVQVR RDLSDVLLSH LTKGTIPDHW HRYKVTKIPN
LGRRLAQLET ISQLNSGGLF FPEAYVTATR AVAHRKKWSL ETLDLRLDLE QIQDPGLVLE

GASYSGG
>RM
FEVEHLRYAT LATSPNLSTQ GQIVSIKTVR NVVEYNEQYV TKRLLLNIVW AFSGDAKGDW
IAWSSKVPVI EILYSWLSEH KPLMLCGPPG SGKTMTLFSA LRKLPDMEVV GLNFSSFRKT
PSGVILAPMQ IGRWLVVFCD EINLPATDKY GTQRVISLVE YGGYYRTSDM AWVKLERIQF
VGACGRVPLS HRFLRLVMVD YPGEASLKQI YGTYNRAMLK VVPNLRAYAE PLTDAMVSFY
LASQKHFTTD MQAHYIYSPR ELTRWVRGIY EAIKPLELLS VEGLVRVWAH EALRLFQDRL
VSEDERVWTD DNIDATAMEH FPTINILFSN WTSKNKARLK VFHEEELDVQ LVLFNDVLDH
VLRIDRVFRQ TTLSRFVAWM NGLSIFQIKV SNKYTGEDFD LLANAEVPGL FEGFTMNPPE
NGLASRAATS PALFNRCVLD WFGDWLPSYA PPTYFPIAYR VVNALVYVHQ SLYQINRRLS
RRQGRYPRHY LDFIQQYVRL YNEKRDELER HLHVGLDKLV TQVEELRASL AIKRKLKRMV
TDQQEAEQKK AASIEIQAAL VEQDRHIEQR RAIVMADLAD AEQMTKPLRE VMKKDYLSRP
SFNFETVNHA SKACMIAELE ASILKYKEEY ALLIRVQSKV DRSMKLLDSL SRTFDTEMST
IVGDVLLSAA FLAYGEWSHH LSDAGIKFKA ELSFPRLSWQ SKTLPSDNLC TVTSFLDDAF
LKVLESALRF GNPLLIQDVE HLDPILNEIR RTGGRVLIRL GSQDIDFSPS FPSVEFSPDI
CSRVTSQSLD QVLKVETDLM KVQGEFKLRL RTLEKLLLQA LNESTGNILD DDKVIDTLET
LKKEAAETDV VMREVEQVTA EYLPIAQACS SVFFILEQLS LINHFYQFSF LDIFDYVLHH
NPNLHGILFD DLFLVVFKRT SRALEEVEFL LESQLNSYSK HTLFKDVKTH MTDHEENWKS
FLLLIRCFRP DRLVPSVTGY DASYRVDNQE GFALADQAIA LAARQGTWVL LKNVHLAPSW
LGEKKLQTLN PHRNFRLFLT MEANPVIPVN ILQSRVVMNE PPPGIFLLAW FHAVVQEDMS
AAFGTIDAWL GAVAKGRANI DPAAIPWDAI KTLIKQSVYG GRIDSDFDQK ILDTFVDSLF
TPSAYNLDFP EGTKLEHFLS WTSWLYLPPT AEQRKMRTLA DDELEKCKEW LAILPTTFAT
FAKQSVENQD PLYRLFFREG QVGRLLTQVR RDLADVLMSH LTKGTIPAHW LRYKVKKVPG
LARRLQQLDR IASLDGGGLF YPEAYITATR AVAHRKKWSL ETLDLRLDIE DVSDPGLVLE
GAAWSSS
>SP
FEVEHLKYAT LATSSNLLTQ KQITAIKTVR NVIEYNEQYV TKRLLVNIIW AFSGDSRGDW
IAWSSRVPVI EILYSWLSEH KPLMLCGPPG SGKTMTLFSA LRKLPDMEVV GLNFSSYRKT
PNGVILAPVQ IGRWLVVFCD EINLPATDKY GTQKVISLVE SGGYYRTSDM AWVKLEHIQF
VGACGRVPLS HRFLRLVMVD YPGEISLNQI YGTYNRAMLK VVPNLRAYAE PLTESMVSFY
LESQRRFTAD IQAHYIYSPR ELTRWVRGIY EAIKPLEVLS VEGLVRVWAH EGLRLFQDRL
VNEEEKTWTD ETIDATAMQH FPTINILFSN WTSKNKARLK VFHEEELDVQ LVLFNDVLDH
VLRIDRVFRQ TTLSRFVAWM NGLSIFQIKV SNKYTGEDFD LLANAEVPGL FEGFTMNPPE
NGLASRAATS PALFNRCVLD WFGDWLSTYK VPDYFPIAYR VVNALVHVHQ TLYQINRRLS
RRQGRYPRHY LDFIQQYVRL YNEKRDELER HLHVGLDKLV DQVEDLRKSL AIKRKLKRMV
ADQQEAEQKK ATSIEIQAAL IEQDKNIAQR KEAVMEELAD AETISKQLRE FMKKEYLSRP
NFNFETVNRA SKACMISELE ASIGRYKDEY AVLIRVQGKV DRSMKLLESL SKTFDTEMST
IVGDVLLSAA FLAYGEWSSH LTEANIKFKT ELSFPRLSWQ SKSLPTDSLC TVTSFLDDAF
LKVLESALRF GNPLLIQDVE HLDPILNEIR RTGGRVLIRL GNQDIDFSPS FPSVEFSPDI
CSRVTSQTLD QVLKVETDLM KVQGEFRLRL RTLEKLLLQA LNESTGNILD DDKVIDTLET
LKKEAAETDV VMKEVEQVTA EYLPIAQACS SVFFILEQLN LINHFYQFSF LDMFDYVLNH
NPKLERILFD DLFLIVFRRT SRALDVLEFL LESGLNSYAK HSLFQPVQSH MQSNEQEWIS
FLLLIRCFRP DRLVQSVIGY DASYRVDNQE GFSLADQAIA VAARQGSWVL LKNVHLAPSW
LGEKKLQTLN PNRNFRLFLT MEANPVIPVN ILQSRVLMNE PPPGVFLLAW FHAVVQEDMA
SAFGTIDIWL NSVAKGRTNI DPMSIPWDAL RTLVKQSVYG GRVDSDFDQK LLDSFVDSLF
TPAAYNLDFP EGTKLEHFLT WPSWLSLPPS AERRKMRSLA DDDLERSKEW LAGVPETFNT
ITKQTLENQD PLYRLFFREG EVGKLLAQVR RDLADVLMSH LTKGTIPTHW LRYKVKKIPN
FARRLQQLDR IAGLDSGGLF FPEAYITATR AVAHRKRWSL ETLDLCLDIE KMNDPGLVLE
GASWSSE
>RF
FEVEHLKYAT LATSPNLVTQ KQVASIKTVR NVIEYNEQYV TKRLLVNIIW AFSGDAKGEW
IAWQSRVPAI EILYSWLSEH KPLMLCGPPG SGKTMTLFSA LRKLPDMEVV GLNFSSYRKT
PNGVILAPVQ IGRWLVVFCD EINLPATDKY GTQRVISLVE CGGYYRPSDM AWVKLERIQF
VGACGRVPLS HRFLRLVMVD YPGELSLKQI YGTYNRAMLK VVPSLRAYSE PLTDAMVEFY

LSSQKRFTTD VQAHYIYSPR ELTRWVRGIY EAIKPLEVLS VEGLVRVWAH EALRLFQDRL
VTEEEKTWTD ENIDSTAMQH FPTISILFSN WTSKYKARLR VFHEEELDVQ LVLFNDVLDH
VLRIDRVFRQ TTLSRFVAWM NGLSIFQIKV SNKYTGDDFD LLANAEVPGL FEGFTMNPPE
NGLASRAATS PALFNRCVLD WFGDWLPSYN PPQYFPLAYR VVNALVHVHI SLHQINLRLS
RRQGRYPRHY LDFIHHYVHL YNEKRDELER HLHVGLDKLV TQVEELRKSL AIKRKLKRMV
ADQQEAEHKK AASIKIQAAL VEQDRNIEER RAVVMADLAD AEQMTKPLRD LMKRDFLSRP
SYNFETVNRA SKACMISELE SSIRTYKDEY ALLIRVQSKV DRSMTLLGSL SRTFDTEMST
IVGDVLLSAA FLAYGEWSHH LTEANVKFKL ELSFPRLSWQ SKSLPSDSLC TVTSFLDEAF
LKVLESALRF GNPLLIQDVE HLDPILNEIR RTGGRVLIRL GNQDIDFSPS FPSVDFSPDI
CSRVTSQSLD QVLKVETDLM KVQGEFRLRL RTLEKLLLQA LNESTGNILD DDKVIDTLET
LKREAAETDV VMREVEQVTA EYLPLAQACS SVFFILEQLN LVNHFYQFSF LDIFDYVLHH
NPNLKGILFN DLFLVVYKRT SRALDELEFL LESGLMSYSK NALFKPVREH IQGHESEWVS
FLLLIKCFRP DRLLQSVTGY DASYRVENQE GFTLADQAIA TAARQGTWVL LKNVHLAPSW
LGEKKLQTLT PNRNFRLFLT MEANPVIPVN ILQSRIIMNE PPPGIFLLAW FHAVVQEDMA
AAFGTIDTWL NTVAKGRTNV DPAVIPWEAV RTLVKQSVYG GRIDSDFDQK LLDTFVDALF
TPAAYNLDFP EGTKVEHFKT WPSWLSLPPS AERRKMRTLE DDDLDRCNEW VSALPATFNS
IQKQSTDNQD PLYRLFSREG HIGKLLSQVR KDLGDVLMSQ LIKGTIPTHW RRYKVTKVVD
LARRLEQLDK IAGLDNGGLF FPEAYVTATR AVAHRKRWSL ETLDLRLDIG NINDPGLVLE
GASWASD
>RI
FEVEHLKYAT LATFPNLLTQ KQVATIKTVR NVIEYNEQYV TKRLLVNIIW AFSGDAKGEW
IAWQSRVPAI EILYSWLSEH KPLMLCGPPG SGKTMTLFSA LRKLPDMEVV GLNFSSYRKT
PNGVILAPVQ IGRWLVVFCD EINLPATDKY GTQRVISLVE CGGYYRPSDM AWVKLERIQF
VGACGRVPLS HRFLRLVMVD YPGELSLKQI YGTYNRAMLK VVPSLRAYSE PLTDAMVEFY
LSSQKRFTTD IQAHYIYSPR ELTRWVRGIY EAIKPLEVLS VEGLVRVWAH EALRLFQDRL
VTEEEKTWTD DNIDSTAMQH FPTISILFSN WTSKYKARLR VFHEEELDVQ LVLFNDVLDH
VLRIDRVFRQ TTLSRFVAWM NGLSIFQIKV SNKYTGDDFD LLANAEVPGL FEGFTMNPPE
NGLASRAATS PALFNRCVLD WFGDWLPSYN PPQYFPLAYR VVNALVHVHI SLHQINLRLS
RRQGRYPRHY LDFIHHYVHL YNEKRDELER HLHVGLDKLV TQVEELRKSL AIKRKLKRMV
ADQQEAEHKK AASIKIQAAL VEQDRNIEER RAVVMADLAD AEQMTKPLRD LMKRDFLSRP
SYNFETVNRA SKACMISELE SSIRTYKDEY ALLIRVQSKV DRSMTLLGSL SRTFDTEMST
IVGDVLLSAA FLAYGEWSHH LTEANVKFKP ELSFPRLSWQ SKSLPSDSLC TVTSFLDEAF
LKVLESALRF GNPLLIQDVE HLDPILNEIR RTGGRVLIRL GSQDIDFSPS FPSVDFSPDI
CSRVTSQSLD QVLKVETDLM KVQGEFRLRL RTLEKLLLQA LNESTGNILD DDKVIDTLET
LKREAAETDV VMREVEQVTA EYLPLAQACS SVFFILEQLN LVNHFYQFSF LDIFDYVLHH
NPNLKGVLFN DLFLVVYKRT SRALDELEFL LESGLMSYSK NALFKPVREH IQEHETDWVS
FLLLIKCFRP DRLIQSVTGY DASYRVENQE GFTLADQAIA TAARQGTWVL LKNVHLAPSW
LGEKKLQTLT PNRNFRLFLT MEANPVIPVN ILQSRIIMNE PPPGIFLLAW FHAVVQEDMA
AAFGTIDTWL NTVAKGRTNV DPAVIPWEAV RTLVKQSVYG GRIDSDFDQK LLDTFVDALF
TPAAYNLDFP EGTKVEHFKT WPSWLSLPPS AERRKMRTLE DDDLDRCKEW LSALPATFNS
IQKQSTDNQD PLYRLFSREG HIGKLLGQVR KDLGDVLMSQ LIKGTIPTHW RRYKVTKVVD
LARRLEQLDK IASLDNGGLF FPEAYVTATR AVAHRKRWSL ETLDLRLDIE NINDPGLVLE
GASWASD
>TA
FEVEHLKYAT LATSPHLETQ KQVASIKTAR NIIEYNEQYV TKRLLVNLIW AFSGDARGEW
ISWSSRVPKI EILYSWLSEH KPLMLCGPPG SGKTMTLFSA LRKLPDMEVV GLNFSSFRKT
PNGVILAPVQ IGRWLVVFCD EINLPATDKY GTQRVISLVE SGGYYRTSDM AWVKLERIQF
VGACGRVPLS HRFLRLVMVD YPGEISLKQI YGTYSRAMLK VVPNLVNYAE PLTDAMVLFY
LASQKRFTSD TQAHYVYSPR ELTRWVRGIY EAIKPLEILS VEGLVRVWAH EALRLFQDRL
VTEDEKVWTD ENIDMTAMQC FPTINILFSN WTSKNKARLR VFHEEELDVQ LVLFNDVLDH
VLRIDRVFRQ TTLSRFVAWM NGLSIFQIKV SNKYTGEDFD LLANAEVPGL FEGFTMNPPE
NGLASRAATS PALFNRCVLD WFGDWVPKYN PPQYFPIAYR VCNALVYVHQ TLYQFNKKLS
RRQGRYPRHY LDFLQHYVKL YNEKRDELER HLHVGLDKLV TQVEELRKSL AIKNKLKRMV
SDQQEAEQKK AASIEIQAAL VEQDRNIDQR RNVVMADLAD AQQMTKQLRE LMKRDFLSRP

SFNFETVNRA SKACMITELE TSIKRYKEEY ALLIRVQSKV DRSMRLLESL SRTFDAEMST
IVGDVLLSAA FLAYGEWSNH LTEAGIMFKP ELSFPRLSWL SKSLPSDNLC TVTSFLDDAF
LKVLESALRF GNPLLIQDVE HLDPILNEIR RTGGRVLIRL GNQDIDFSPS FPSVEFSPDI
CSRVTSQTLD QVLKVETDLM KVQGEFRLRL RTLEKLLLQA LNESTGNILD DDKVIDTLET
LKTEAAETDI VMSEVEQVTA EYLPIAQACS SVFFILEQLN LINHFYQFSF MNIFDYVLHH
NPNLQGVLYD DLFLIVFRRT SRALDEYEFL LEGGLQSHSR HIIFKPVQVH LRDHEDEWSA
FLILVKCFRP DRLVQSVKGY DASFRVENQE GFSLADQAIA QASRQGTWVL LKNVHLSPSW
LGEKKLQTLN PHRNFRLFLT MEANPVIPVN ILQSRILMNE PPPGIFLLAW FHAVVQEDMN
AAFGTIDTWL HSVAKGRANI DPMQIPWDAI RTLVKQIVYG GRVDSDFDLK LLDTFVDSLF
TPASYNLDFP EGTKLDHFMS WPSWLSLPPS AERRKMRSLT DDDLERCKEW LGALPESFNT
LQKQSADNQD PLYRLFFREG EVGKLLRQVR RDLADVLMSH LTKATIPTHW LRYKVKKIPN
LALRLQQLDK IASLDTGGLF FPEAYITATR AVAHRKKWSL ETLDLRLDVE RVNDPGLILE
GAAWVGN
>PN
FEVEHLKYAT LATSPNLSSQ KQIASIKTAR NVIEYNEQYV TKRLLVNIIW AFSGDARGEW
IAWSSRVPII EILYSWLSEH KPLMLCGPPG SGKTMTLFSA LRKLPDMEVV GLNFSSYRKT
PNGVILAPVQ IGRWLVVFCD EINLPATDKY GTQRVISLVE LGGYYRTSDM AWVRLERIQF
VGACGRVPLS HRFLRLVMVD YPGEISLTQI YGTYNRAMLK VVPNLRAYAE PLTDSMVSFY
LASQKRFTSD IQAHYIYSPR ELTRWVRGIY EAIKPLESLS VEGLVRVWAH EGLRLFQDRL
VTEDEKNWTD ENINSTAMTH FPTLNILFSN WTSKNKARLK VFHEEELDVQ LVLFNDVLDH
VLRIDRVFRQ TTLSRFVAWM NGLSIFQIKV SNKYTGEDFD LLANAEVPGL FEGIYHESPE
NGLASRAATS PALFNRCVLD WFGDWRPSYK PPEFFPTAYR VVNALVYIHQ SLYQINKKLS
RRQGRYPRHY LDFIQQYVRL YNEKRDELER HLHVGLDKLV TQVEELRKSL AIKRKLQRMV
SDQQEAEQKK AASIEIQAAL VEQDKHIEQR RNIVMADLAD AEQMTKQLRD LMKREFLSRP
TFKFETVNHA SKACMIAELE SSIHKYKEEY ALLIRVENKV ERSMRMLESL SQTFDEEMST
IVGDVLLSAA FLAYGEWSTH LGDASIKYKA ELSFPRLSWQ SKSLPSDSLC IVTSFLDDAF
LKVLESALRF GNPLLIQDVE HLDPILNEIR RTGGRVLIRL GNQDIDFSPS FPSVEFSPDI
CSRVTSQTLD QVLKVETDLM KIQGEFRLRL RTLEKLLLQA LNESTGNILD DDKVINTLET
LKREAAETDV VIREVEQVTA EYLPIAQACS SVFFILEQLN LINHFYQFSF LDIFDYVLHH
NPNLKNVLFD DLFLLVFKRT SRALEEYEFL LESGLESHAK HELFKPVREH MKNNGEEWIT
FLLLTKCFRP DRLAQSVTGY DASYRVENQE GFALADQAIA LAARQGTWVL LKNVHLAPSW
LGEKKLQTLN PNRNFRLFLT MEANPVIPVN ILQSRILMNE PPPGIFLLAW FHAVVQEDMS
AAFGTIDAWI NSVAKGRANI DPVTIPWDAV RTLVKQSVYG GRIDSDFDQK LLDTFVDSLF
TPSAYNLNFP EGTKIDHFLS WPSWLSLPPT AERRKMRSLA DDDLERCKEW LGALPESFST
LARQSSDSQD PLSRLFQREE EVGKLLHQVR RDLADVLLSH LTKGTIPGHW MRYKVKKIPN
LSLRLSQLDR IAKLDDGGLF FPEAYITATR AVAHRKRWSL ETLDLRLDIE RVNDPGLVLE
GAAWSVD
>FM
FEVEHLKYAT LATSPNLRTQ REIAGIKTAR NVLEYNEQYV TKRLLVNIIW AFSGDARGEW
IAWSSRVPVI EILYSWLSEH KPLMLCGPPG SGKTMTLFSA LRKLPDMEVV GLNFSSYRKT
PNGVILSPVQ IGRWLVVFCD EINLPATDKY GTQRVISLVE CGGYYRTADM AWVRLERIQF
VGACGRVPLS HRFLRLVMVD YPGEISLKQI YGTYNRAMLK VVPTLRAYAE PLTDAMATFY
LESQKRFTTD MQAHYIYSPR ELTRWVRGIY EAIKPLEMLS VEGLVRVWAH EALRLFQDRL
VSEDEKVWTD ENINSTAMQY FPTINILFSN WTSKHKARLR VFHEEELDVQ LVLFNDVLDH
VLRIDRVFRQ TTLSRFVAWM NGLSIFQIKV SNKYTGEDFD LLANAEVPGL FEGFTMNPPE
NGLASRAATS PALFNRCVLD WFGDWLPAYK PPQFFPIAYR VVNALVYIHQ SLYQINKKLS
RRQGRYPRHY LDFISQYVRL YNEKRDELER HLHVGLDKLV TQVEELRKSL AIKRKLQRMV
SDQQEAEQKK AASIEIQAAL VEQDKHIEQR RSIVMADLAD AEQMTKPMRE LMKREYLARP
TFNFETVNRA SRACMIAELE ASIRTYKEEY ALLIRVENKV ERSMRMLDSL SQTFDEEMST
IVGDVLLSAA FLAYGEWSHH LHEAHIKYKA ELAFPRLSWQ SKSLPSDSLC IVTSFLDDAF
LKVLESALRF GNPLLIQDVE HLDPILNEIR RTGGRVLIRL GSQDIDFSPS FPSVEFSPDI
CSRVTSQTLD QVLKVETDLM KLQGEFRLRL RTLEKLLLQA LNESTGNILD DDKVINTLET
LKKEAAETDV VMREVEQVTS EYLPIAQACS SVFFILEQLN LINHFYQFSF LDIFDFILHH
NPNLQNVLFD DLFLVVFKRT SRALDEYEFL LESGLDNYAK HPMFKPVLDS IRSNESDWVP

FLLLIKCFRP DRLVSSVTGY DASYRVEGQE GFALADQAIA VAARQGTWVL LKNVHLAPSW
LGEKKLQTLN PNRSFRLFLT MEANPVIPVN ILQSRVIMNE PPPGIFLLAW FHAVVQEDMS
AAFGTIDVWL NSIAKGRANV DPVSVPWDAV RTLIKQSVYG GRIDSDFDQK LLDTFVDSLF
TPAAYNLDFP EGTKIDHFLS WPSWLSLPPT AERRKMKSLA DDDSEHCREW LALLPENFNT
LAKQAVENQD PLNRLFLREG EIGKLLNQVQ RDLGDVLLSH LTKGTIPTHW LRYKVKKIPN
LARRLAQLDR IAGLDTGDLF FPEAYVTATR AVAHRKKWSL ETLDLRLDIE RVNDPGLVLE
GASWSSD
>OS
FEVEHLKYAT LATAPNLLTQ KEIASIKTVR NVIEYNEQYV TKRLLVNIIW AFSGDSRGEW
VDWSSRVPVI EILYSWLSEH KPLMLCGPPG SGKTMTLFSA LRKLPDMEVV GLNFSSYRKT
PNGVILAPVQ IGRWLVVFCD EINLPATDKY GTQRVISLVE LGGYYRTSDM AWVRLERIQF
VGACGRVPLS HRFLRLVMVD YPGEISLKQI YGTYNRAMLK VVPNLRAYAE PLTDSMVAFY
LASQKRFTSD VQAHYIYSPR ELTRWVRGVY EAIKPLEMLS VEGLVRVWAH EALRLFQDRL
VNEDEKVWTD DNIDTTAMVH FPTINILFSN WTSKNKARLK VFHEEELDVQ LVLFNDVLDH
VLRIDRVFRQ TTLSRFVAWM NGLSIFQIKV SNKYTGEDFD LLANAEVPGL FEGFTMNPPE
NGLASRAATS PALFNRCVLD WFGDWVSSYK PPQYFPVAYR VVNGVVYVHQ SLYQINKKLS
RRQGRYPRHY LDFIQQYVRL YNEKRDELER HLHVGLDKLV TQVEELRKSL AIKRKLQRMV
SDQQEAEQKK AASIKIQAAL VEQDRHIEQR RNVVMADLAD AEQMTKQLRD LMRRDFLSRP
SFTFEMVNRA SKACMIAELE ASIRTYKEEY AFLIRVENKV DRSMRMLESL SQTFDDEMST
IVGDVLLSAA FLAYGEWSSH LSEANIKFKS EISFPRLSWQ SKSLPSDSLC IVTSFLDDAF
LKVLESALRF GNPLLIQDVE HLDPILNEIR RTGGRVLIRL GSQDIDFSPS FPSVEFSPDI
CSRVTSQTLD QVLKVETDLM KVQGEFRLRL RTLEKLLLQA LNDSTGNILD DDNVINTLET
LKKEAAETDV VMREVEQVTA EYLPIAQACS SVFFILEQLN LVNHFYQFSF LNIFDYILHH
NPNLQNVLFD DLFIVVFKRT SRALDEYEFL LESGLNSYAK HAIFKPVQLH VRDNEDDWVP
FLLLIKCFRP DRLVQSVTGY DASYRVENQE GFSLADQAIA LAARQGTWVL LKNVHLAPSW
LGEKKLQTLN PNLNFRLFLT MEANPVIPVN ILQSRIIMNE PPPGVFLLAW FHAVVQEDMN
AAFGTIDIWL NSVAKGRANV DPVSIPWDAV RTLVKQSVYG GRVDSDFDQK LLDTFVDSLF
TPAAYNLDFP EGTKLDHFLS WPSWLSLPPT AERRKMRSLA DDDLERCREW LAALPESFNT
LSKQSSVSQD PLGRLFFREG EVGKLLSQVR RDLADVLLSH LTKGTIPFHW LRYKVKKIPN
LARRLAQLDH IAGLDNGGLF FPEAYITATR AVAHRKRWSL ETLDLRLDIE RVNDPGLVLE
GATWASD
>SN
MSVDHKRVAH FIGTKVLIAN NGIAAVKEIR SIRQWSDTFG TDRAIEFTVM ATPEDLKAVW
AGWGHASENP RLPESLAASK HKVVFIGPPG SAMRSTIVAQ SADVPCMPWS GTGISDTVQS
ENGFLTVPDA AYAAACVTSA EQGMERAAKI GYPIMIKASE GGGGKGIRKV DAPEAFKNAY
IAVCGEVPGS PVFVMLLADQ YGSAISLFGR DCSVQRRHQK IIPVTIAKRE KFEEERAAVR
LAKLVGYVSA GTVEYLYSPA EDVFYFLELN NMVVALKELS IRGDFRTTVE YLIKLLETQV
FEENTTGWLD TLISNRLTAE RPEATVICGA VTKAHSEECW TEYKRVLDKG QVPARDTLKT
VFGVDFIYEG DGKSHSVYWR EEVGSVRLMV DSKTCLIEQE AYAEIEVMKM YMPFEGQLPK
MGLPSVVGSK PHQQLNFGID ILNNILDGYD NQAIMASTLK VVEVLRNPEL PFSEASAVLS
ALSGRIPGKL EDSIRATIEN AKTKTNPVET MFRASLAPLV DVVDRYKSGL KVHEVIANLL
SRYEATEKLF GGSIEAVVAL VLSHTKAQSK NKLVLSLLDM IKQMEQVLRA SVTATYYGEP
GGSHQLPSAD ILREVLAAFE VYVRRAYRAY ELLSRRQGSV SDLTYLLGQN IASFPDFKSL
TNGFAKVAAS LPLFNQAPNV LNLALRIFDK EDDMPEEEWR QKFLTLLNDS KAGVWQEEEA
IRNIEPALAF QLELSRLSNY NLTPCFVMDS RFFVRALIRP GRIRGNMRMS EVSAQYRHSD
CNHIATVTFD EVVEAISGFI ERHGKRLWRL QVTGAEIRIA LEDSEGNVTP IRCVIDNVSG
FIVNFHKGST ILKSIGEKPL HLQPVNQPYP TLQPKRYQAH LVGTTYVYDF PDLFGKALHN
HWLARRVLDP SVKIPQTLLE SKELDENEEL QEVDNDITYK IGSFGPQEDH FFYAASQYAR
SRLGVECLKG SGLIATSRAY DDIFTITLQR AVQVEGQPII LTKVLGREVY TSNLQLGGTQ
IMGSDLEGVA HIIQWLSYVP ASKGTSLPVA ISWDRDITYV PPKGPYDPRW FIEGKVDDKG
SFQETLSGWA QTVVVGRARL GGIPMGLIAV ETRTIERVAD PANPTSFEQH IMEAGQNSAY
KTAQAIFDFR EGLPLIIFAN WGAWVVLDPS INSAQMEMYA DVDRDKVLGL MERLDPSYAS
YKKDSKDAAQ ASEKLVARET ELQKQIALLY ADLHDRGRME AKGCAKPTVW KDARRRFYWN
LRARLAQESV LKQLQSAASE RLESLVTSSS SDARAYAEEL EQLDLKPTIV DLQADKAALH

SLVHVFG
>AS
AAVDHAKVAH FIGTKVLIAN NGIAAVKEIR SIRQWSETFG TERAVEFTVM ATPEDLKAVW
AGWGHASENP RLPESLAASK HKIVFIGPPG TAMRSTIVAQ SAGVPTMAWS GTGITDTELS
PQGWVTVPDK AYKDACVTTV EEGLEKADQI GYPVMIKASE GGGGKGIRKV ESPDAFKNAF
GAVSGEVPGS PIFIMVLADQ YGNAISLFGR DCSVQRRHQK IIPVTIAKPE LFEEERAAVR
LSKLVGYVSA GTVEYLYSHQ EDSFYFLELN NMVVALKELS IRGDFRTTVE YLINLLQTQA
FEENTTAWLD TLISNKLTPE RPDWAVICGA VTKAHSEGCW AEYKRILDKG QVPAKDVLKT
VFAVEFIYEG DGKSHSVYWR EEVGATRLMI DSKTCLIEQE AYAEIEVMKM YMPFEGLLPP
MGMPHVSGNK PHQHLEYCVD VLNNILDGYD NAAMMGATLK LIEVLHNPEL PFYAVGSILS
TLSQRMPAKL EDAIRHAMDM AKAKGAGQEA MVRAQLAPLV EAVERFRGGL KAHETITALL
ARYQETEHLF GGSIEAVAAL VLSHTKAQSK SKLVLALLDI VKQMEQVLRA SVSSSYYGES
GIGHRMPSAE VLREVLAALE VYVRRSYRAY TINYRRQGSV SDLSYVINKH IAAFTNLAAL
ERGFEKVVSA LPAFDQPPNV LTIAVQIFDK EDDLVDSVWQ EKFIALVNDR TAGVWQEEAA
LRNIEPALAY QLELGRLSNY NLAPCFAFDT RFFIRALVRP GRLRGGMTTA EVGQQYRNSD
CNHIYNVTYD EVLQAMAGFI ERHGKRLWRL HVTGAEIRII LEDNEGNVTP IRAVIENVSG
FIVNYHKGTK ILKSIGEMPL HLQPVNQPYP TLQPKRYQAH LIGTTYVYDF PDLFSKALQN
AWLEARAARP GLAIPKKVLE SRELDEHDRL QEVDNDITYK IGSFGPLEDQ FFNLASQYAR
SYLGVESLRG SGLIATSRAY DDIFTITLQR AVQVEGQPII LTKVLGREVY TSNLQLGGTQ
IMASDLEGAT HIVRWLSYVP ERRGAPLPVT IPWDREIGYT PPKGAYDPRW FIEGKEEDDK
SFQETLGGWA QTVVTGRARL GGIPMGVIAV ETRTIERVAD PANPLSFEQR IMEAGQNSSY
KTAQAIFDFR EGLPLMIFAN WGAWVVLDPS INSEQMQMYA DVDREKLLGL MDRLDPAYAE
FKRASTDKTE ATEKLAAREQ ELQKSLALLY ADLHDRGRME AKGCAKRSVW KESRRYFYWA
LRARLARSSA LAEIAKQRIQ LLDSLLPSVA ATMRETAEML EALDLTRLLT QVRGDQAVVE
GLERLRD
>EG
TTVDHSKVSH FIGTKVLIAN NGIAAVKEIR SIRQWSETFG TERAVEFTVM ATPEDLKAVW
AGWGHASENP RLPESLAASK HKIVFIGPPG TAMRSTIVAQ SANVPCMAWS GTGITDTELT
PQGWVTVPEE AYKKACVTTV DEGLQKAEEI GYPVMIKASE GGGGKGIRKV ESPDAFKNAF
GAVLGEVPGS PVFIMVLADQ YGNAISLFGR DCSVQRRHQK IIPVTIARPE RFEEERAAVR
LAKLVGYVSA GTVEYLYSPS DDLFYFLELN NMVVALKELS IRGDFRTTVE YLINLLQTQA
FEENTTAWLD GLITNKLTPE RPDQTVICGA VTKAHSDACW AEYKRILDKG QVPAKDVLKT
VFAIDFIYEG DGKSHSVYWR EEVGATRLII DSKTCLIEQE AYAEIEVMKM YMPFEGQLPS
MGLPGVAGNK PHQRLEFYVD VLNNILDGYD NSALMTSTVK LLDVLRNPEL PFFTTSAILA
ALSGRMPAKL EDQVRNALEV AKGKSGSPEA MVRAQLAPLT EAVERFRGGL KAHETITALL
SRYEQTESLF GGSIEAVAAL VLSHTKTQSK SKLVLALLDI VKQMEQVLRA SVSSSYYGES
GLGHRMPSAE VLREALAALE TYVRRSYRAY TVNYRRQGSV SDLSYVISKH IASFPNIAAL
EHGFKKVVAT LPPFDQPPNV LTIALRVFDK EDSLEDSIWQ EKFASLVNGN VDGTWTEEAA
IRNIEPALAY QLELSRLSNY KLTPCFVFDT RFFIRALVRP GRLRGGMTMA EVSQQHRNAD
CNHISNVTYD EVLQAMAGFI DRHGKRLWRL HVTGSEIRIA LEDDEGNVTP IRAVIENVSG
FIVNYNRGSK ILKSIGEMPL HLQPVNQPYA TLQPKRYQAH LIGTTYVYDF PDLFSKALHN
VWLEARNARP DLVLPKKVLE SRELDEHDCL QEVDNDITYK IGSFGPQEDQ FFYIASQYAR
RFLGVESLRG SGLIATSRAY DDIFTITLQR SVQVEGQPII LTKVLGREVY TSNLQLGGTQ
IMASDLEGAT HILRWLSYVP MRRGGPLPIT VPWDRDIGYT PPKGPYDPRW FIEGKEEDDK
SFQETLSGWA QTVVVGRARL GGIPMGVIAV ETRTIERVAD PANPTSFEQR IMEAGQNSAY
KTAQAIFDFR EGLPLIIFAN WGAWVVLDPS INSEQMQMYA DVEREKLLGL MDRLDATYAG
HKRASLDKTQ AAELLAKREQ ELQKSLALLY ADLHDRGRME AKGCAQPTVW KESRRHFYWA
LRARLARSSA LAQFAESRVQ LLDSLLPSDR TSIRETADML EALDLSSTLT QVRGDQAVME
GLERLRD

08-DNA polymerase type-A

>AS
KLPDITFDLP PLQGDSIDEH FHNIGRNVAE PYLSMASEFA EQLLCFDVET MPHISPFPIM
ATARSTTHWY SWLSPWLLGR IVVGHNVSYD RARIREEYNL ERTATRFIDT MSLHVAVKGI

SSHQRPAWMK WKKEKRRWEE LTSANSLADV AMLHCGITVR KAVRDDFLVA SREEIAGNLD
KYLGYCGTDV KTTYAVFRAV FPKFVAACPS PVSFAGVMSM GSAFLTVNQE WENYLQRAEA
KYRELERAVK VSLLKLAKDA WAQDVWLSQL DWTPKIPAWY RELLPRLEDE LDLTTRSAVA
PLLLKLGWRG HPLFRSREHG WMYRVQETAR EFKDAHDVEA FFKLPHKDGE EANVGNPLSK
TFMKYAEDGT MTSPSATTRL AIDLNAQSSY WVSARDRVLQ QLVVWDKWGM IIPQVITMGT
VTRRAIEKTW LTASNAKSNR IGSELKAMVR APPGYAIVGA DVDSEELWIS SVMGDAQFGL
HGATAIGWMT LEGTKSAGTD LHSKTASILG ISRNDAKVFN YSRIYGAGMK HASLLLLQSN
PGMSLEKAQE LATELYVKTK GRNTHRDIFD RKFWFGGSES YVFNKLEEIA MSNRPQTPAL
GCGVTDALAK EYLPQTFGSD YLPSRINWVV QSSGVDYLHL LIVSMEYLLK TYDIKARYLI
SVHDELRYLV VEEDKYRCAL ALQIANLWTR CQFAWRLGMD DLPQGVAFFS AVDVDWLLRK
EVDMPCVTPS QLTPLAPGES LDMRGILEKT HSGSLRRESY TAPNHMSHRA TTSHFLKAQA
TQEFGEVKLM A
>EG
NLPDLAFTLP QLHGASIDEH FHNIGSDVAK PYLDLATQFA EQLLCFDVET LPHISPYAVM
AVAVSPTNWY SWLSPWLLGR IVVGHNVSYD RQRIREEYSL ERSASRFLDT MSLHVAIKGI
SSHQRPAWNK WKKQKRSWEE LTSANSLLDV AQLHCGITLK KAVRDDFLTA TREEILEDVD
RYLTYCAGDV HATHSVYRVV FPKFREACPS PVSFAGVLSM GSACLPVNQE WEKYLERAET
KYRELEDGVK YELYKLAKDA WQDDEWLSQL DWTPVVPAWY RELLPVLDDV LDLTVRTRAA
PLLLKLCWRG YPLFHSRAYG WMYRVQEKER KFVVEHDVEK FFKLPHKDGE EANVGNPLSK
TFLKYALDGT LTSPSAVTRK ALDMNAQSSY WISARDRVLN QVVVWQKWGL ILPQVITMGT
VTRRAIEKTW LTASNAKANR IGSELKAMVR APPGYAIVGA DVDSEELWIS SVMGDAQFGL
HGATAIGWMT LEGTKAAGTD LHSKTASILG ISRNDAKVFN YSRIYGAGMK HASLLLLQSN
PGMSIEKAQQ LALELYARTK GRNTHRDYFG RKFWFGGSES YVFNKLEEIA MSDRPQTPAL
GCGVTDALAK KNLPQTFGSD FLPSRINWVV QSSGVDYLHL LIVSMEHLLK AYDIKARYLI
SVHDELRYLV VEEDKYRLAL ALQIANLWTR CQFAYRLGMD DLPLGVAFFS AVDIDWLLRK
EVDMPCITPS QPEPLAPGES LDMEGVLAKT NGGSLWRDGY VPPDCMTHRA QTSHFLKAQA
TQELGEVKAR A
>CP
VLPDTGFQLP PLQGTSIDEH FFAIGARAAQ PWMDMAHDLA ETALVFDVET MPKYHPYAVM
ACAASPHAWY AWISPWLLAR VIVGHNVSYD RARMREEYSL DGTETRFLDT MSLHVATSGI
SSHQRPAWMK YRKDKKRWED ITSANSLADV AKLHCGIAVD KELRSDFMTH APAQIRADVR
RYLGYCANDV GVTHAVYARV LPTFRQRCPS PVSFAGVLTM GSSFLTVNES WEAYIADAER
AYRELERGIK KRLVELAEEA WREDPWLAQL DWTPKWPKWY WDLAKPKKGT LDITVRNRLA
PLLLRLSWLG FPLFHSREHG WMFRVRLTPL EFFDPADEHL FYKLPHKDGE KANVGSPLAK
TFGKYARDGT LASPWEAAKD ALDMNARCSY WISARDRIIN QMVVWDKWGM ILPQVVAMGT
VTRRAIEKTW LTASNAKKDR VGSELKAMVR APPGYSIVGA DVDSEELWIS SCMGDAQFGL
HGATALGWMT LEGTKAAGTD LHSKTASILG ISRDQAKVFN YSRIYGAGMR HAVLSLLQAN
AGMSQEEAQR LAENLYASTK GKNTHRDLFG RKFWFGGTES FVFNKLEEIA LSERPTTPAL
GCGITHALSR EYLPETFGGD YMTSRINWVV QSSGVDYLHL LIVAMDYLVR AYGIKARYLL
SVHDELRYLV ADRDRYRAAL ALQIANVWTR SLFAYKLGMD DLPQGVAFFS SVDVDRVLRK
EVDMPCVTPS QLSPIPPGES LNIIQTLEKT HGGSLWPVDY VPPDCMKHRA ESAGFLRAQA
TNDFSELKHH A
>PN
VLPETEFVLP LLQGENIDEH FHCIGIDAAE PYLSVAKKFA EDMLVFDVET LPNYHPYAVM
ACAASDKYWY SWISPWLLGK LIVGHNVSYD RARVLEEYNV EDSSIRYLDT MSLHVAVKGI
SSHQRPAWLK YRKNKKRWED VTSANSLADV AKLYCNIRIE KATRNDFMTL SREEILGNIQ
KYLNYCSSDV EATHAVFTKV FPDFLLTCPS PVSFAGVLRM GSSFLTVDEN WEEYLKSAEG
IYLGLKEKIK KRLEALADEA WKDDMWLSQL DWTPKWPKWY WDLTKPKKGT LDVTIRSRVA
PLLLQLSWKG WPLFHSREHG WTFRVRQNKL EFHDAADAIL FYKLPHKDGD KANVGNPLGK
TFIKYAQDGT LTSPGEDTKA ALEMNAQCSY WISSRDRILN QMVVWKKVGM IIPQVITMGA
VTRRAIERTW LTASNAKKDR VGSELKAMVR APSGYKIVGA DVDSEELWIA SIMGDAQFGL
HGATALGWMT LEGTKSAGTD LHSKTASILG INRNQAKVFN YSRIYGAGMK HAILLLLLQSN
PSMPQETAQG QAEKLYRSTK GQKTHRNFFE RKFWFGGTES YVFNKLEEIA MSDKPLTPAL
GCGVTYALSK EYLQTGFGND YMTSRINWVV QSSGVDYLHL LIVSMEYLCK KYEIRARYLI

SVHDELRYLV KDEDRYRAAL ALQIANLWTR CLFAYKLGME DLPQGVAFFS AVDIDHVLRK
EVDLPCVTPS QPSEIPPGES LNIQQVLEKT DEGSSLLRSY TKPDCLKHRT SSTYFLKAQA
MSKRRDVEGL E
>SS
VHPEISFTLP PLQGDTIDAH FHAIGAKIAQ PYLSLSKEMA EDMLVFDVET LPNYSPFAVM
ACAASPTAWY TWISPWLLGR IVIGHNVAYD RCRTLEEYRL ERPGTRWLDT MALHVAVNGI
SSHQRPAWGK YRKQKKRWEE LTSANSLADV AYLHCGIEVD KSTRDDFMTS TPEAIRSDIN
SYLTYCANDV AVTHRVFQMT LPAFLQKCPS PVSFAGICSM GSSFLPVDDN WERYIQSADS
KYQVLESKVV EALRKLAEEA WKGDVWLEQL DWTPKIPAWY FKLGKSRVES LEVTLRSRMA
PLLFKLKWLG WPLVHSREHG WTFRVRLLEL SFYHEEDETL YFKLPHKDGD TANVGNPLAK
TFIKFAADGT LSSPNESAVE ALNINAQCSY WISARDRIMK QLVIWDKWGM ILPQVITMGT
VTRRAIERTW LTASNAKANR IGSELKAMVR APEGYAIVGA DVDSEELWIS SCMGDAQFGF
HGATAIGWMT LEGTKKAGTD LHSKTASILG ISRDQAKVFN YSRIYGAGMK HAMLLLLQGN
ASMLPEEAQR LAGDLYASTK GKNTHRDLFG NKFWYGGTES YLFNKLEQIA MSDEPKTPAL
GCGVTYALSK QYLPEKFGSD YLPSRINWVV QSSGVDYLHL LIVAMEHLLQ RYDIDARYLI
SVHDELRYLV KEEDKYRAAL ALQVANLWTR SMFAYRLGMD DLPQGIAFFS AVDVDKVLRK
EVDMSCVTPS QPVPISPGET LDIAKTLEKT NGGSLNKDGY VSPDCLAHRA KSEYWLDAQS
TDVFERIKFL A
>FS
VLPDTAFTLP PLHGRTLDEH FHAIGAAAAE PYRALAAAFA EGAVCFDVET MPNEHAYAVL
ATAATADAWY AWISPWLLGR VVVGHNVSYD RARIRDEYAL AGSATRFVDT MALHIAVKGI
SSHQRPAWMK HRKGKSRWED ITSVNSLAAV AKLHCGIALD KTARSDFMSA TPAQVRGDVR
AYLDYCAGDV HATHRVLAAV LPQFLARCPS PVSFAGMLSM GSSFLCVDEG WEGYLADAER
TYRDLDRGVK ARLLELAEAA SRGDPWLSQL DWTPKVPKWY WDLSKPKKGA VDITVRTRVA
PLLLRLSWLG WPLVHSREHG WAFRVRLAPL AFYDPADDAL FYKLPHKDGA AANVGNPLAK
PFMKFAQDGT LTSPEAAAAE ALDMNAQCSY WISSRDRIMN QNVVWQKRGV IIPQVIAMGT
VTRRAIERTW LTASNAKANR VGSELKAMVR APAGYAIVGA DVDSEELWIA SCMGDAQFGL
HGATAIGWMT LEGTKAAGTD LHSKTAGILG ISRDQAKVFN YSRIYGAGMK HAMQLLVQSN
AGMLPEVAQQ LAQNLYASTK GKTTHRDVFG RKFWFGGTES YLFNKLEEIA LSDKPETPAL
GCGVTDALSK KFLPEEFGSN YMTSRINWVV QSSGVDYLHL LIVSMDHLIA KYNINARYLI
SVHDELRYLV TESDKYRLAL ALQIANIWTR SLFAYKLGMD DLPQGVAFFS AVDVDTVLRK
EVDMPCVTPS QPHPIPAGES INIAQTLQRT HGGSLHADGY QKPDCMKHRS QGAAFLQAQA
TTDFAEIKHL A
>RM
RASGGKIHFT PLQGQTIDEH FYNIGAKAAE PWLSLAKDFA EDLLVFDVET MPGIHPFAVI
ACAMSPTNWY SWISPWLLGR VVVGHNVSYD RARILEEYHV SGTQTRFLDT MALHVAVKGI
SSHQRPAWMK YRKVKKRWED ITSANSLEDV AKLHCGIRLR KAARNDFLTH TREEILSGIQ
EYLDYCWRDV DVTHAVYAKV FPEYLKACPN PVSFAGILTM GSSFLTVNHS WRDYLENSER
VYNELNERVK QRLLELAEEA WKNDVWLSQL DWTPKWPKWY WELAKPRKGS LNLTVRTRFA
PLLLRLSWLG WPLFYSREHG WTFRVRLKPL KFYHHHDEPL FYKLPHKDGE VANVGSPLGK
TFIKYAQDGT LTSPGNGAES ALDMNAQCSY WISSRDRILN QMVIWDSWGM ILPQVITMGT
VTRRAIEKTW LTASNAKANR VGSELKAMVR APPGYSIVGA DVDSEELWIS SAMGDAQFGL
HGATALGWMT LEGTKAAGTD LHSRTASILG ISRDQAKVFN YSRIYGAGVR HAVLLLLQGN
PSLSPDVAQK QAEKLYASTK GKNTHRDLFN RKFWFGGSES YVFNKLEEIA LSDKPKTPAL
ECGITHALSK EFLPPGFGTD YMTSRINWVV QSSGVDYLHL LIVSMDHLIS KYDIKARYLI
SVHDEVRYLV KQEDRYRAAL ALQIANLWTR CQFAYRLGMD DLPQGVAFFS AVDVDKVLRK
EVDLPCVTPS QTVPIPPGES LDIFKALEKT NGGSLHCDGY TTPNCIVHRS MSPAFIRAQA
TTEPGEVKRL A
>RF
VLPDTGFALP PMQGRTIDEH FYRIGNAAAQ PWLAFAKDFA EDMISFDVET MPNYHNYAVM
ACAMSSTHWY SWISPWLLGR IVVGHNVSYD RARVLEEYSI KGTKTRFLDT MALHVAVKGI
SSHQRPAWMK YRKTKKRWED ITSANSLADV AKLYCKVEMD KEIRNDFMTH TPAEILANVH
EYFDYCANDV HVTHSVFAAV LPQFLTACPN PVSFAGILTM GSSFLTVDQE WEAYLENAER
KYRELEEDIK KRLLALAERA WRTDEWLSQL DWTPKWPKWY WELAKPKKGT VDITVRNRIS

PLLLRLSWQG WPLFHSREYG WTFRVRQKPL TFFDKADISL FYKLPHKDGE SANVGSPLSK
TFIKYAQDGT LTSPGDEAKD ALDMNAQCSY WISSRDRILK QMVVWQKWGM ILPMLITMGT
VTRRAIEKTW LTASNAKKNR IGSELKALVR APPGYALVGA DVDSEELWIS SVMGDAQFGL
HGATAIGWMT LEGTKAAGTD LHSKTANILG ITRDQAKIFN YSRIYGAGMR HAVLLLLQSN
ASMKPEDAQK QSTKLYSSTK GKNTHRDLFG RKFWFGGTES FVFNKLEEIA LSDRPQTPAL
GCGITYALSK EYLSPGFGTD YMTSRINWVV QSSGVDYLHL LIVAMDHLIA KYNIDARYLI
SVHDELRYLV AEHDKYRAAL ALQVANLWTR SLFAYRLGMD DLPQGVAFFS AVDVDTVLRK
EVDLPCVTPS QPIPMPPGES LNIIQVLEKT NHGSLHEDGY IMPDRLAHRS PSAAFLRAQA
TTELGELRRL A
>RI
ILPDTGFTLP PMQGRTIDEH FYRIGNAAAQ PWLAFAKDFA EDMISFDVET MPNYHNYAVM
ACAMSSTHWY SWISPWLLGR IVVGHNVSYD RARVLEEYSI KGTKTRFLDT MALHVAVKGI
SSHQRPAWMK YRKAQKRWED ITSANSLADV AKLHCNIEMD KEIRNDFMTH TPAEILANVH
DYLDYCANDV HVTHSVFAAV LPQFLAACPN PVSFAGILTM GSSFLTVDQE WEAYLEKAEG
KYRELEEDIK KRLLALAEKA WRNDEWLSQL DWTPKWPKWY WELAKPKKGT VDITVRNRIS
PLLLRLSWQG WPLFHSREYG WTFRVRQKPV TFFDKADVSL FYKLPHKDGE SANVGSPLSK
TFIKYAQDGT LTSPGDEAKD ALDMNAQCSY WISSRDRILK QMVLWQKWGM ILPALITMGT
VTRRAIEKTW LTASNAKKNR VGSELKALVH APPGYALVGA DVDSEELWIS SVMGDAQLGL
HGATAIGWMT LEGTKAAGTD LHSKTANILG ITRDQAKIFN YSRIYGAGMR HAVLLLLQSN
ASMKPEDAQK QAQKLYSSTK GKNTHRDLFG RKFWFGGTES FVFNKLEEIA LSDRPQTPAL
GCGITYALSK EYLSPGFGTD YMTSRINWVV QSSGVDYLHL LIVAMDHLIT KYNIDARYLI
SVHDELRYLV AEHDKYRAAL ALQIANLWTR SLFAYRLGMD DLPQGVAFFS AVDIDTVLRK
EVDLPCVTPS QPIPIPSGES LNIVQVLQKT NQGSLREDGY IKPDRLAHRS PSAAFLRAQA
TTELGELRRL A
>FM
ILPNTGFTLP PLQGSSIDEH FHRIGHAASE PWLGLAKQFA EEMLSFDVET MPNHHQYAIM
ACAMSPTHWY SWISPWLLGR IIVGHNVSYD RARVLEEYHV KGTNSRFIDT MSLHVAVKGI
SSHQRPAWMK YRKNKKRWED ITSANSLADV AKLHCGIDIG KEIRNDFMTR SREEIFEGIN
DYLDYCATDV EITHAVYTKV FPDFLQACPS PVSFAGVMTM GSSFLTVNEQ WENYLRNAEG
IYRELNEKIK KRLISIAEDV WKDDVWLSQL DWTPKWPKWY WDLAKPKKGT LDLTIRSRLA
PLLLRLSWSG WPLFHSREHG WIFRVRSKKL DFYDPADAHL FYKLPHKDGE SANVGSPFGK
TFMKYSQDGT LTSREGDTKD ALDMNAQCSY WISSRDRILN QMVVWNKVGM IIPQVITMGA
VTRRAIEKTW LTASNAKKNR VGSELKAMVR APKGYAIVGA DVDSEELWIS SAMGDAQFGL
HGATALGWMT LEGTKAAGTD LHSKTASILG ISRDQAKVFN YSRIYGAGMR HAILLLLQSN
PNMLPDIAQQ QAEKLYASTK GKNTHRDIFG RKFWFGGSES YVFNKLEEIA MSDKPRTPAL
GCGVTYALSK EYLPAGFGTD YMTSRINWVV QSSGVDYLHL LIVSMEHLIA KYDIKARYLM
SVHDELRYLV REEDKYRAAL ALQIANLWTR CLFAYKLGMD DLPQGVAFFS AVDVDHVLRK
EVDLPCVTPS QPTPIPPGES LNIIKALEKT NGGSLWRDGY VKPDCLIHRA SSANFLRAQA
TTEFGEIKQL A
>OS
VLPDTGFTLP PLQGGSIDEH FYRIGVEAAQ PWLNLAKQFA EDMLSFDVET MPNYHPYAVV
ACAMSPNHWY SWISPWLLGR LVVGHNVSYD RARILEEYRV EGSNTRYIDT MSLHVAVKGI
SSHQRPAWMK YRKSKKRWED ITSANSLADV AKLHCDIDIG KETRNDFMTH AREDILENLQ
DYLDYCSTDV EVTHAVYAKV FPDFLRACPN PVSFAGVMTM GSSFLTVNEQ WEEYLKNAEG
IYRELDDKIK KRLVALAEQV WKEDVWLSQL DWTPKWPKWY WELAKPKKGT LDVTVRSRVA
PLLLQLSWAG WPLFHSREHG WVFRVRLKKL NFYDSGDAHL FFKLPHKDGE SANVGSPFGK
TFVKYAQDGT LTSPGDEAKD ALDMNAQCSY WISSRDRISN QMVVWDKVGM ILPQVITMGA
VTRRAIEKTW LTASNAKKNR VGSELKAMVR APAGYAIVGA DVDSEELWIS SAMGDAQFGL
HGATALGWMT LEGTKAAGTD LHSKTASILG ISRDQAKVFN YSRIYGAGMR HAILLLLQSN
PNMLPDVAQK QAEKLYASTK GKNTHRDTFR RKFWFGGSES YIFNKLEEIA MSDKPLTPAL
GCGVTYALSK EYLSPGFGTD YMTSRINWVV QSSGVDYLHL LIVSMEHLLA KYDIKARYLL
SVHDELRYLV KEEDKYRAAL ALQIANLWTR CLFAYKLGID DLPQGVAFFS AVDVDHVLRK
EVDLPCVTPS QPTPIPPGES LNIVKVLEKT NGGSLWRDGY VKPDCLTHRA SSANFLRAQA
TSEFGEIRRL A

>SP
ILPDTGFNIP DLQGKNIDEH FFRIGADAAQ PWLDLAKTFA EEMLTFDVET MPNYHNYAVM
ACAMSKSNWY SWVSPWLLDR IVVGHNVSYD RARILEEYHI DGTKTRFLDT MSLHVAVKGI
SSPQRPAWMK YKKLKKRWED ITCANSLADV ARLHCGIDMS KEIRNDFMTH TRSQIFDGIQ
DYFEYCSKDV AVTHAVYSKV FPDFLKACPS PVSFAGVMAM GSSFLTVNEE WEKYLAKAEG
FYLEAEEKIK RRLIEIAEET WKEDVWLSQM DWTPKWPKWY WDLTKPKKGT VDITIRSRLA
PLLLRLSWLN WPLFHSREHG WIFRVRQKKL TFYDPADAHL FYKLPHKDGD SANVGNPFGK
TFVKYAQDGT LTSPGDEAKS ALDMNAQCSY WISSRDRIMK QMVVWQKWGM IIPQVVTMGT
VTRRAIEKTW LTASNAKKNR VGSELKAMVR APPGYAIVGA DVDSEELWIS SAMGDAQFGL
HGATALGWMT LEGTKAAGTD LHSKTASILG ITRDQAKVFN YSRIYGAGMR HAILLLLQSN
PNMLPEIAQK QAEKLYASTK GKNTHRDIFG RKFWFGGTES FVFNKLEEIA LSDRPQTPAL
GCGITHALSK EYLPPGFGTD YMTSRINWVV QSSGVDYLHL LIVSMEYLIK RYNIKARYLI
SVHDELRYLV KEEDKYRAAL ALQIANLWTR CLFSYRLGMD DLPQGVAFFS AVDVDWVLRK
EVDLPCVTPS QPDPIDPGES LNITQILEKT NGGSLWSDGY VSPNCLAHRA SSAEFLRAQA
TSEFGEVKRL A
>TA
ILPETNITLP PLQGNSIDEH FYHIGAASAQ PWLDLAQRFA EEMLSFDVET LPNYHPYAVM
ACAVSATHWY SWISPWLVGR VVVGHNVSYD RARILEEYHV GGTNTRFLDT MSLHVAVKGI
SSHQRPAWMK YRKSKKRWED ITSANSLADV ARLHCGIDIS KEIRNDFMTH SREEILENIQ
DYLDYCSSDV SVTHSVFSKV FPDFLKACPS PVSFAGILTM GSSFLTVNEQ WEEYLKRAEG
TYQELEEKVR RRLIELAEKA WKNDVWLSQL DWTPKWPKWF WDLTAPKKGT VDITVRNRFA
PLLLRLSWSG WPLFHSRQHG WIFRVRLKKL NFHDPNDTLL FYKLPHKDGE LANVGSPFGK
SFLKYAQDGI LTSPGDEAKD ALDLQAQCSY WISSRDRILK QMVVWDKWGM IIPQMITMGT
VTRRAIEKTW LTASNAKKNR VGSELKAMVR APKGYAIVGA DVDSEELWIS SAMGDAQFGL
HGATALGWMT LEGTKAAGTD LHSKTASILG ISRDQAKVFN YSRIYGAGKK HAILLLLQSN
PNMLPEKAEA QAEKLYASTK GKNTHRDLFG RKFWFGGTES FVFNKLEEIA LSDNPQTPAL
GCGITHALSK EYLSPGFGTD YMTSRINWVV QSSGVDYLHL LIVSMEHLIK KYNIQARYLI
SVHDELRYLV KEEDKYRTAL ALQIANLWTR CLFAYKLGMD DLPQGVAFFS AVDVDTVLRK
EVDLPCVTPS QPVPIPPGES LNITQVLERT NGGSLWSDGY TRPNCLVHRA SSAYFLRAQA
TAEFGEVKRL A
>WC
VLPDISFALP PLQGRTIDQH FYAIGMDAAQ PWLGLAKDLA EEMFVFDVET LPKCSPYPIM
ACAVSKNAWY SWVSPWLIGR VVVGHNVSYD RARIKEEYNI NTTGTRFLDT MALHVAVKGI
SSHQRPAWTK YRKEKKRWEE FTSANSLADV ASLHCGITID KEIRNDFLIC TREEIHEKLQ
DYLNYCATDT AVTHAVFTKT LPAFLKTCPS PVSFAGVLTM GSSLLTVNEQ WEKYLKSAER
TYNDLSNAVK QRLIKLAEQA WEDDPWLSQL DWTPKWPKWY WDLTAPNKGT PDITVRSRIA
PILLRLEWRE WPLFHSREHG WTFRVRATAL EFKHEADASL FYKIPHKDGE KANVGNPLAK
SFMQHARDGI LTSPEPVANA ALDMNAQCSY WISARDRVMN QMVVWQKWGI ILPQVITMGA
VTRRAIEKTW LTASNAKKNR VGSELKAMVR SPPGYSIVGA DVDSEELWIS SVMGDAQFGL
HGATALGWMT LEGTKAAGTD LHSKTAKILG ISRDQAKVFN YSRIYGAGMR HAVLLLLQAN
ASLSPDEAQK AAERLYASTK GKSIRHHCFG RKFWHGGMES LVFNKLEEIA MSSKPQTPAL
GCGVTDALSK EYLPETFGGD YMTSRINWVV QSSGVDYLHL LIVAMDHLIR KYKIHARYLI
SVHDELRYLV KEEDQYRAAL ALQIANLWTR SLFAFKLGMS DLPQGVAFFS AVDIDKYLRK
EVNMDCVTPS QPTPLPPGES LDIKEILEKT GGGSLSPDGY KEPECLAHRA QSVHWLKAQV
TDDFNEVKTL D
>SN
RLPDISFDLP PLQGRTLDEH FWNVGAESAQ PWLGKAKNFA DDMLVFDVET LPNYSPFAIM
ATAASTSAWY AWISPWLLGR IVVGHNVSYD RARIREEYHI QRTTTRYIDT MALHVAVKGI
SSGQRPSWIK YNKAKKRWED LTSANSLADV AKLHCGIEID KTVRNDLMTH SPSQILDDIR
TYLSYCATDV STTHAVFTRV LPAFLTACPN PVSFAGVLTM GSSFLSVNGT WKEYLKAAEN
KFRELEESVK DALKELAEVA WKDDVWLSQL DWSEKWPKWY WELAKPRKGS IDVTVRNRIA
PLLLQLKWRG YPLFHSREYG WIFRVRQREL SFYEDADLHL FYKVPHKDGE DANVGSPLAK
PFMKYLQDGT LTSPSSVAKE ALDMNAQCSY WISARDRVLN QMVVWDSWGM IVPQLITMGT
VTRRAIEKTW LTASNAKSNR VGSELKAMVR APPGYAIVGA DVDSEELWIS SVMGDAQFGL

HGATAIGWMT LEGTKAAKTD LHSKTADILG ITRDAAKVFN YSRIYGAGMR HAQLLLLQNS
ASMVPEKAKK LAEDLYAATK GKNTHRDLFG RKFWFGGSES FVFNKLEEIA LSDRPQTPAL
GCGVTYALSK EYLPNEFGSD YMPSRINWVV QSSGVDYLHL LIVAMEHLIL RYGIQARYLI
SVHDELRFLV KEEDKYRAAL ALQIANLWTR SQFAYRLGMD DLPQGVAFFS AVDVDTILRK
EVFMTCETPS HQHKLEPGES LDIQQILEKT DGGKLGDVVY IEPDCLVHRA PGPEFLQAQA
TDQFNEIKAL A
>SC
VLPNTSFTLP PLLGSTLDQH FHAMGVEASE PYLSFAKRFA EDMLAFDVET LPKVHQYPIM
ACAASPTAFY AWLSPWLLGR VVVGHNVSYD RARIKEEYEL AQSRTRFLDT MSLHVAVKGM
SSHQRPAWMK YKKEKKSWQD VTSANSLRDV AKLHCGIEVS KEIRDDFMTS TRESILADVT
DYLSYCAKDV VTTHAVYCKV LPGFLEACPH PVSFAGILTM GSAFLTVDDG WKRYIQSAEG
IFRKMEDGVK RKLHDMAENA WKDDVWLSQL DWEPKWPKWY WEITKPKKGS MDLTVRNRLA
PLLFRLSWLG HPLFHSREHG WVYRVRRKMV SFYDPADDTL FFKLPHKDGD SANVGSPLSK
TFMSHASNGT LSSPFNEAKE ALELNAMCSY WISSRDRIMN QMVVWQQWGV IVPQVITMGT
VTRRAIEKTW LTASNAKKNR VGSELKAMVR APPGYAIVGA DVDSEELWIS SCMGDAQFGI
HGATALGWMT LEGTKSAGTD LHSKTASILG ISRDQAKVFN YSRIYGAGMR HAVLLLMQSN
ASMKHEEAQK LAENLYASTK GKNTHRDLFE RKFWFGGSES YVFNKLEAIA LSDKPETPAL
GCGVTHALVK EYLPTGFGSD YMPSRINWVV QSSGVDYLHM LIVSMDHLIQ KYDIKARYLI
SVHDELRYLV EEKDRYRAAL ALQIANCWTR CMFAYKLGMD DLPQGVAFFS AVDVDHVLRK
EVDMPCVTPS QPHPIPPGES LNITKVLEKT HGGSLWPDGY KEPNCLTHRA QNAAFLQAQA
TTEFNEIKRL A
>PS
VLPDVSFTLP PLWGDNIDEH FHFIGSTIYQ PWITIAEDFA ERMLVFDVET MPEYSPYPVM
ACAATQNGWY AWISPWLLGR IIVGHNVSYD RARIAEEYRL EGTKTRFVDT MSLHVAVKGI
SSHQRPAWNK HRKAKKRWED LTSANSLADV ASLYCGIKVE KEIRSDFMTH SREEILEGVQ
DYLNYCANDV DVTHAVYSKV LPAFRERCPH PVSFAGILTM GSSFLPVNQE WERYLENAER
TYKELDDGVK SRLVELAEQA WKESPWLSQL DWTPKWPKWY WDLTRPRKGS LDLTVSKRIS
PLLLQLSWQG WPLFHSREHG WAFRVTSTPL YFAHQADAKL FYKMPHKDGE EANVGNPLGK
TFMKYAQDGT MTSPSDAAKD ALDMNGQCSY WISARDRILN QIVVWQKWGV ILPQMVTMGT
VTRRAMERTW LTASNAKKNR VGSELKAMVR APPGYAIVGA DVDSEELWIA SVLGDAQFGM
HGATALGWMT LEGTKAAGTD LHSKTASILG ISRDQAKVFN YSRIYGAGMR HAVQLLLQNS
AGMSVEKAQE LATELYARTK GKNTHRDLFG RKFWYGGTES VVFNKLEAVA LADVPQTPAL
GCGVTDALTK AYLTEGFGTD YMTSRINWVV QSSGVDYLHM LIVAMEHLMR DFNIRARYLI
SVHDELRYLV KEEDKYRAAL ALQIAHLWTR TMFAYRLGLD DLPQGVAFFS AVDVDKVLRK
EVDMPCVTPS QPNPIPPGES LSIVDVLKKT DGGSLYPGGY VPPNTMQHRA ESAYFLRAQT
TSELAEVRML A
>PI
VLPDISFTLP HLQGDNLDEH FYRIGTSAAE PWLSFSKEFA ERMLCFDVET MPEYHPFAVM
ATAASKNAWY AWISPWLLGR IIVGHNVSYD RSRILEEYNL NGTQNRFIDT MALHIAAKGI
SSHQRPAWMK HHKNKKRWED LTSINSLADV AKLHCNIDMD KEIRNDFMTS IPSAIRDSIH
TYLDYCANDV FVTHSVFAKV LPSFLDRCPN PVTFAGVLTM GSSFLTVNES WDAYLRDAER
TYKNLELSIK KRLVELAEQA WKDDPWLSQL DWTPKWPKWY WNLAKPKKGT IDITARNRVA
PLLLRLSWLG WPLFHSREHG WTFRVRLAPL EFHDPADDKL FYKVPHKDGE HANVGNPLAK
PFMKFAQDGT MTSPGDEAKE ALDMNAQCSY WISARDRILN QNVVWQKWGV IIPQVITMGT
VTRRAIEKTW LTASNAKKNR VGSELKAMVR APDGYAIVGA DVDSEELWIS SCMGDAQFGL
HGATAIGWMT LEGTKAAGTD LHSKTAGILG ISRDQAKVFN YSRIYGAGMR HAVLLLLQSN
AGMLPDEAQK LAKNLYASTK GQSTQRDMFG RKFWFGGTES YLFNKLEEIA LSDRPQTPAL
GCGVTDALSK EFLPPGFGTD YMTSRINWVV QSSGVDYLHL LIVAMNYLVA KYDIKARYLI
SVHDELRYLV KEEDKYRAAL ALQIANLWTR SLFAYKLGMD DLPQGVAFFS AVDVDKVLRK
EVDMPCITPS QPEPIPSGES LNITQILEQT NGGSLWVDGY QNPDCLAHRA KGAAFLQAQA
TTEFAEVKHL A
>CC
VLPDIGFQLP PLQGSNLLEH FHRIGLHAAE PWLSLSKEHA ESMLTFDVET MPKHHPYAVM
ACAASENAWY AWISPWLLGK VVIGHNISYD RARIGEEYRL EGTQTRFIDT MSLHAAVYGI

SSHQRPAWMK HRKARKRWED ITAVNSLAEV AKLHCGIEVN KDARDDLFEG NIEEVRNNLH
DYLDYCCNDV HVTHEVYKKI LPSFLQSCPH PVSFAGILTM GSSFLPVNQE WEAYIERAEK
TYRDMQAKVE KKLVSLAEDA WKSDPWLSQL DWTPKWPKWY WEATKPRKGT LDITTRGRIA
PLLLRLSWKD WPLTHSREHG WIYRVRSKPL DFYDNADAHL FYKLPHKDGE KANVGSPLGK
TFIKYAQDGT LKSPGDEAKE AFDLNAICSY WISARDRIMN QMVVWEKWGI IVPQVVTMGT
VTRRAIEKTW LTASNAKKNR VGSELKAMVR APEGYAIVGA DVDSEELWIS SCMGDAQFGL
HGATAIGWMT LEGTKAAGTD LHSKTAKILG ISRDQAKVFN YSRIYGAGMR HAILLLLQGS
SAMLPEAAQK LAENLYASTK GKNTHRDIFE RKFWFGGSES YLFNKLEEIA LSDKPQTPAL
GCGVTYALSK EYLPTGFGSD YLPSRINWVV QSSGVDYLHL LIVSMQYLIQ RYNINARYLI
SVHDELRYLV TDQDKYRLAL ALQIANLWTR CMFAYKLGMD DLPQGVAFFS AVDVDKVLRK
EVFMPCTTPS QPDPIPSGES LDIAGVLEKT NGGSLFADGF QTPDCLVHRA PNAAWLRAQA
TTDFAEIKAL A
>SH
VLPDTSFTLP PLTGPDLSSH FHRIGSACSH PYLSLARSLA EEALVFDVET LPEYGPYAVM
ACAASKNGWY AWISPWLMGR VVVGHNVSYD RARVKEEYHV EGSKNRFLDT MALHVAVKGI
SSHQRPAWMQ YRKSKKRWED ITSANSLADV AKLHCGIEVD KEIRNDFMTH TPEEIMANVQ
DYLTYCASDV NVTHSVYAKV LPDFLDACPN PVSFAGILTM GSSFLTVNQE WERYIENAER
TYRELDTKVK ERLLELAVEA WRDDVWLSQL DWTPKWPKWY WELTKPKKGT IDITTRSRVT
PILLKLSWKD CPLFHSREHG WTYRIRQTPL SFHDPSDETL FHKVPHKDGE SANVGSPLGK
TFIKFSQDGT LKSPGDLANS ALDMNAQCSY WISARDRVLK QMVVWEKWGI IIPQVITMGT
VTRRAIEKTW LTASNSKKNR VGSELKAMVR APDGYAIVGA DVDSEELWIS SVMGDAQFGL
HGATAIGWMT LEGTKAAGTD LHSKTASILG ISRDQAKVFN YSRIYGAGMR HAVLLLLQAN
SGMLPEEAQK LAENLYASTK GKNTHRDIFG RKFWFGGTES FVFNKLEEIA LSDRPQTPAL
GCGVTYALSK EYLPAEFGSD YMPSRINWVV QSSGVDYLHL LIVSMDHLIQ KYDIQARYLI
SVHDELRYLV KKEDKYRLAL ALQVANLWTR SMFAYRLGMD DLPQGVAFFS AVDVDFVLRK
ETDMTCVTPS QPVPIPPGES LNVEQVLAKT KGGSLWADGY VEQDCLQHRA HSAEFLRAQA
TQEFEEIKHL A
>LB
VLPDTGFQLP PLQGSNLLEH FHRIGSHAAQ PWLNLGKSFA EDMLTFDVET MPNYHPYPVL
ACAASLNSWY VWISPWLLGR VIVGHNVSYD RGRILEEYNV VSTKNRFIDT MALHVAVKGI
SSHQRPAWMK YRKSKKRWED LTSANSLADV AKLHCGIDMD KQTRSDFMTS TPKEILNNIA
DYLDYCSTDV SVTHQVYATA LPDFLTACPH PVSFGGILTM GSSFLTVNEE WDAYLARAEK
VYRDLEEGVQ NKLKKLAEDA WKDDVWLSQL DWTPKWPKWY WDATKPKKGT LEITVRNRIA
PILLRLSWFG WPLFHSREHG WTFRVRLSPL SFYDPTDDKL FYKLPHKDGE KANVGSPLGK
SFIKFAQDGT MKSPGDEAKE ALDMNALCSY WISARDRILN QMVVWQKWGI ILPQVITMGT
VTRRAIEKTW LTASNAKKNR VGSELKAMVR APEGYAIVGA DVDSEELWIS SCMGDAQFGL
HGATAIGWMT LEGTKAAGTD LHSKTASILG ISRDQAKVFN YSRIYGAGMR HAILLLLQSN
ASMLPEIAQK LAENLYASTK GKNTHRDIFE RKFWFGGTES YLFNKLEEIA LSDKPQTPAL
GCGVTYALSK EYLPAEFGSD YMPSRVNWVV QSSGVDYLHL LIVSMDHLIS KYNINARYLI
SVHDELRYLV AEEDKYRTAL ALQIANLWTR SLFAYKLGMD DLPQGVAFFS AVDIDKVLRK
EVDMPCVTPS QPIPIPPGES LDIFGVLAKT NGGSLWKDGY AEPDCLKHRS ASAAFLSAQA
TTDFGEVKGL A
>PC
VLPDIGFTLP SLQGRTLDEH FYSIGSVAAQ PWLTLAKDLG EQMLVFDVET MPEYSPYPVM
ACAATKNAWY AWISPWLLGR VVVGHNVSYD RARILEEYST DGTQTRFLDT MALHVAVKGI
SSHQRPAWMK YRKDKKRWED ITSANSLADV AKLHCNIDVD KSVRNDFCTH SREQISSDIT
SYLDYCSSDV YVTHAVYRQV LPAFLTSCPS PVSFAGILTM GSALLTVNHE WERYIENAER
TYKELEDKVK NRLIDLARQA WKEDVWLSQL DWTPKWPKWY WDLAKPKKGS LDITVRNRIA
PILLRLSWQG WPLFYSREYG WTFRVRATPL TFYDAADEAL FYKLPHKDGE KANVGSPLSK
TFIKYAQDGT LTSPGDEAKD ALDMNAQCSY WISARDRILK QMVVWQKWGI IIPQVITMGT
VTRRAIEKTW LTASNAKKNR VGSELKAMVR APNGYAIVGA DVDSEELWIS SVMGDAQFGL
HGATAIGWMT LEGTKAAGTD LHSKTASILG ISRDQAKVFN YSRIYGAGMR HAVLLLLQAN
PGMLPEEAQK LAENLYASTK GKNTHRDFFG RKFWFGGTES FVFNKLEEIA LSDKPQTPAL
GCGITYALSK EYLPAEFGSD YMTSRINWVV QSSGVDYLHL LIVSMEHLIK KYDIKARYLI

SVHDELRYLV KEEDKYRAAL ALQIANLWTR CMFALKLGLD DLPQGVGFFS AVDIDEVLRK
EVDMPCVTPS QPEPIPPGES LDIYKVLEKT NGGSLHPDGY QEPDYLAHRA NDAAWLRAQA
TSELAEIKIL A
>HI
VLPDTNFDLP PLQGSTIDEH FHRIGDRAAH PWLELAVTLA EDMIVFDVET MPNYHPYAIM
ACAASRNGWY AWISPWLLGR VIVGHNVSYD RARIKDEYHI QGSQNRFLDT MALHVAVKGI
SSHQRPAWMK YRKSKKRWED ITSANSLADV AKLHCDIEMD KEIRNDFMTQ NPETIRQGIQ
DYLHYCAEDV FVTHSVYSHV LPEFLTVCPS PVSFAGILTM GSSFLTVNES WEKYLRDAER
TYRDLEDKVK TRLIDLATEA WKDDVWLSQL DWTPKWPKWY WDLTKPKKGT IDITSRSRIS
PILLKLSWQG WPLFHSREHG WTFRVRSASV DFHDAADDVL FYKLPHKDGE SANVGNPLAK
AFIKFSQDGT LTSPGTEAKD ALDMNAQCSY WISARDRVLN QMVVWHKWGI IVPQVITMGA
VTRRAIEKTW LTASNAKKNR VGSELKAMVR APDGYAIVGA DVDSEELWIS SVMGDAQFGL
HGATAIGWMT LEGTKAAGTD LHSKTAGILG ISRDQAKVFN YSRIYGAGMR HAVLLLLQAN
ASMLPDEAQK LAEQLYASTK GKNTHRDIFG RKFWFGGTES FVFNKLEEIA LSDRPQTPAL
GCGVTYALSK EYLPAEFGSD YMPSRINWVV QSSGVDYLHL LIVAMEHLIA TYDIEARYLI
SVHDELRYLV KEEDRYRAAL ALQIANLWTR SQFAYKLGMD DLPQGVAFFS AVDVDHVLRK
ETDMPCVTPS QPDPIPPGEC LDIQKTLDLT NGGSLLRGGY PQPDCLQYRA ESAAFLQAQA
TNEFAEVKHL A
>SL
ILPDTGFTLP PMQGRNIDEH FHRIGSNSAQ PWLSLAKGFS EQMLVFDVET MPPYHPYAVM
ACAASGNAWY SWISPWLLGR IVVGHNVSYD RGRILEEYDL RGTQTRFLDT MSLHVAVKGI
SSHQRPAWMK HRKSKKRWED LTSANSLADV AKLHCDITVD KGIRNDFMSH TPSEILANIH
DYLNYCSQDV FVTHAVFSQV LPAFLARCPS PVSFAGILTM GSSFLTVNES WEAYLENAER
TYRELDEKVK TRLVDLAEEA WKDSSWLAQL DWTPKWPKWY WDLAKPKKGS LDITVRNRIA
PILLRLSWLG WPLFHSREHG WMFRVRLTPL QFYDPADGVL FYKLPHKDGE KANVGSPLGK
SFMKYAQDGT MTSPGGEANE ALDMNAQCSY WISARDRIIK QMVVWQKWGI ILPQVITMGT
VTRRAIEKTW LTASNAKKNR VGSELKAMVR APEGYAIVGA DVDSEELWIS SCMGDAQFGL
HGSTAIGWMT LEGTKAAGTD LHSKTASILG ISRDQAKVFN YSRIYGAGMR HAVLLLLQSN
ASMLPDQAQK LAENLYASTK GKNTHRDIFG RKFWFGGTES FLFNKLEEIA LSDCPRTPAL
GCGITYALSK EYLPAEFGSD YMTSRINWVV QSSGVDYLHL LIVSMDYLIK TYDIKARYLI
SVHDELRYLV ADDDRYRAAL ALQIANLWTR SLFAYKLGMD DLPQGVAFFS AVDVDTVLRK
EVDMPCVTPS HPTPIPPGES LNIVRTLEKT NGGSLWADGY RSPDCLTHRA KSAAFLRAQA
TSEFSEVKHL A
>FP
VLPNTAFTLP PLQGRNVDEH FHAIGASAAQ PWLSLAKDFA EDILVFDVET LPNYSPYAVM
ACAASKSAWY SWVSPWLLGR AVVGHNVSYD RGRILEEYSV EGTRTRFLDT MALHIAVKGI
SSNQRPAWTK HRKAKKRWED LTSANSLADV AKLHCSIEMD KEIRNDFMTS TREDILQGIQ
EYLNYCAGDV AVTHAVFCKT LPAFLTACPS PVSFAGVLTM GSSFLTVNEQ WEEYLANAER
TYKELEAKVQ KQLHDLAYQA WKSDVWLSQL EWTPRWPKWY WELTKPRKGS LDLTVRSRVA
PLLLRLSWQG WPLFHSREHG WTFRVRATPL AFKLDADASM FYKLPHKDGE QANVGSPLGK
TFVKFSQDGT LTSPGDEAKE ALDMNAQCSY WISARDRVLN QMVVWQKWGI ILPQVITMGT
VTRRAIEKTW LTASNAKKNR VGSELKAMVR APEGYAIVGA DVDSEELWIS SIMGDAQFGL
HGATAIGWMT LEGTKAAGTD LHSKTASILG ISRDQAKVFN YSRIYGAGMR HAMLLLLQSN
AAMLPEQAQQ LAQNLYASTK GKNTHRDLFG RKFWYGGTES FVFNKLEEIA LSDHPQTPAL
GCGVTYALSK EYLPATFGDD YMPSRINWVV QSSGVDYLHL LIVSMEHLIK KYNIQARYLI
SVHDELRYLV KDEDRYRAAL ALQIANLWTR SLFAFKLGMS DLPQGVAFFS SVDVDRVLRK
EVDMPCVTPS QSSAIPPGES LDITTVLEKT NGGSLWADGY RQPDCLVHRA DSAAWLRAQA
SAEIEEIRQL A
>PP
VLPDISFTLP QLQGHTLDEH FYAIGSAAAQ PWLGLAKDLA ENMLVFDVET MPAYSPYPVM
AVAASKNCWY SWISPWLLGR VVVGHNVSYD RGRILEEYNI QPTQTRFLDT MALHIAVKGI
SSHQRPAWIK YRKSKKRWEE LTSANSLADV AKLHCDITMD KEVRNDFMKC TPAEILDGVQ
DYLNYCSTDV GVTHAVFSKT LPGFLSACPS PVSFAGILTM GSSLLTVNEE WDAYLENAER
TYKELEDKVK TRLHELAHKA WKSDVWLSQL DWTPKWPKWY WDLTRPKKGS MDLTVRNRFS

PLLLRLSWQG WPLFHSREHG WTFRVRAKPL AFNDSADATL FYKLPHKDGD KANVGSPLGK
TFIKYAQDGT LTSPGDEAKE ALDMNAQCSY WISARDRVLK QMVVWQRWGI ILPQVITMGT
VTRRAIEKTW LTASNAKANR VGSELKAMVR APKGYAIVGA DVDSEELWIS SVMGDAQFGL
HGATAIGWMT LEGTKAAGTD LHSKTASILG ISRDQAKVFN YSRIYGAGMR HAVLLLLQSN
AGMLPEQAQK LAQDLYASTK GKNTHRDLFG RKFWYGGTES FVFNKLEEIA LSDRPQTPAL
GCGVTYALSK EYLPATFGAD YMPSRINWVV QSSGVDYLHL LIVAMDHLIK KYNIHARYLI
SVHDELRYLV KEEDRYRAAL ALQIANLWTR SLFAFKLGMD DLPQGVAFFS AVDIDKVLRK
EVDMTCVTPS QPVPIPPGES VDIGTVLAKT NGGSLWPDSY RQPHCLTHRA SSTAWLRAQA
TSSIAEINGL A
>DS
VLPNVGFTLP PLQGRTLDEH FYRIGTSAAQ PWLTLVQDLA EEMLVFDIET MPRYHDYAVM
ACAATPNAWY AWISPWLLGR VVAGHNVSFD RKRVLEEYNI QPTQTRFLDT MSLHVAVKGI
SSHQRPAWQK YRKSKERWED KTSLSSLADV AKLHCGIEVD KGARDDFLSE TREAILEDIH
TYLDYNATDV EVTHAVFAKT LTAFLAACPS PVSFAGILTM GSSLLTVNEE WEAYLANAER
AYKELEDRIK KRLIELAQQA WKEDVWLAQL DWSPKWPRWY WELAKPKKGT LDLTVRNRYA
PLLLRLSWQG WPLFYSRGHG WTFRVRASPL AFDDNVDPQL FYKLPHKDGE DANVGSPLGK
TFMKYAQDGT LASPFEEAKD ALDMNAQCSY WISSRDRVTK QMVVWQKWGI ILPQVITMGT
VTRRAIEKTW LTASNAKQNR IGSELKAMVR APPGYAIVGA DVDSEELWIS SVMGDAQFGL
HGATALGWMT LEGTKAAGTD LHSKTASILG ISRDQAKVFN YSRIYGAGMR HAVLLLLQGN
AGMLPEKAEE LARNLYASTK GQKSYGRYFD RKFWYGGTES FVFNKLEEIA LSDRPRTPAL
GCGITHALSK SLLPDEFGSD FMTSRINWVV QSSGVDYLHL LIVSMEHLIA KYNIQARYLI
SVHDELRYLV KEEDRYRAAL ALQVANLWTR SLFAFKLGLD DLPQGVAFFS AVDIDHVLRK
EVDMPCVTPS QPNPIPPGES VDILKTLEKT RGGSLWQGGY SSPDCLAHRA NSASWLRAQS
TSNFDEVKSL A
>GT
VLPGTAFPLP PLLGANLDQH FHAVGRRAGQ PYLALARALA EEMLVFDVET MPRYGPHPVL
ACAASTNAWY AWISPWLLGR VVVGHNVAYD RARVREEYAL GGTGNRWLDT MALHVAVTGI
SSHQRPAWMK YRKSQKRWED LTSANALADV AKLHCGIEMD KEVRNDFMTR TPEEILENVH
DYLTYCANDV AVTHAVFSKV LPEFLARCPH PVSFAGILTM GSSFLTVNES WEEYIENAER
KYKELEDKVK SRLVQLAQQA WKDDVWLSQL DWTPKWPQWY WELTRPKKGT IDLSVRTRIA
PLLLRLSWLG WPLFHSREHG WTFRVRASEL DFYHPDDETL FYKLPHKDGE KANVGSPLGK
TFMKYAQDGT LKSPGDEAKD ALDMNAQCSY WISARDRVMN QMVVWQKWGM ILPQVVTMGT
VTRRAMEKTW LTASNAKKNR VGSELKAMVR APEGYSIVGA DVDSEELWIS SVMGDAQFGL
HGATAIGWMT LEGTKAAGTD LHSKTASILG ISRDQAKVFN YSRIYGAGMR HAVLLLMQAN
AGMLPEQAQA LAEQLYAQTK GKNTHRDLFG RKFWYGGTES FVFNKLEEIA LSDSPRTPAL
GCGITHALSK EYLPPGFGTD YMTSRINWVV QSSGVDYLHL LIVSMAHLID KYKINARYLI
SVHDELRYLV ADEDKYRAAL ALQIANLWTR CLFAYRLGMG DLPQGVGFFS SVDVDKVLRK
EVDMPCVTPS QPHPIPPGES LNIQALIEKM DGASLWKDGY QEPDCLVHRA SSAAWLRAQA
TTELSEIKLL S
>TV
VLPNIEFTLP PLQGRTLDEH FYRIGSSAAQ PWLTLAQDLA EEMLTFDVET LPAYTPFAVM
ACAASKHAWY SWISPWLLGR VVVGHNVSYD RGRVLEEYNI NGTQTRFIDT MALHIAVKGI
SSHQRPAWIK HRKSKKRWED LTSANSLADV AQLHCDITMD KEVRNDFMTS TPEEIRDGIH
TYLDYCSTDV AVTHAVFAKT LPAFLTACPN PVSFAGVLTM GSSLLTVNEE WDKYIANAER
TYRELEEKVK MRLVDLAHEA WKDDAWLSQL DWTPKWPKWY WDLTKPKKGT LDLTVRNRIS
PILLRLSWLG WPLFHSREHG WTFRVRATPL VLADDADADL FYKLPHKDGE KANVGSPLAK
TFMKYSQDGT LASPFEEAKG ALDMNAQCSY WISARDRVMN QVVVWQKWGV ILPQVITMGT
VTRRAIEKTW LTASNAKKNR VGSELKAMVR APPGYALVGA DVDSEELWIS SVMGDAQFGL
HGATAIGWMT LEGTKAAGTD LHSKTASILG ISRDQAKVFN YSRIYGAGMR HAVLLLLQSN
AGMLPEQAQK LAENLYASTK GKNTHGRLFG RKFWYGGTES FVFNKLEEIA LSDRPQTPAL
GCGITYALSK EYLPVGFGSD YMPSRINWVV QSSGVDYLHL LIVSMEHLIA KYNIQARYLI
SVHDELRYLV KDEDRYRAAL ALQIANLWTR SYFAYKLGMD DLPQGVAFFS SVDVDRVLRK
EVDMPCVTPS QPVPIPAGES LDILQTLEKT HGGSLWADGY SPPDCLAHRA HSAPWLRAQA
TSDLDEIKRL A

09-HECT

>SN

ASLISTLLTT PEDGIADALD PIATWNWPRS DLHSWIKVLN RFDGILENLV NIHKVDQLQY
DTFTAAEKKT LLGILKFEKL LLENSTNRKL FNSYDRLNCL LYTSDLDILL SVLQLILRPA
QQYSAQPTVS AALKISTSRL QALAKRWPGL REQGIELADL VSAKIAEVEA LASQINEVNF
TFYRNLRPFL HLSKDASSIA AEISSTANVP EERFELLCRI RTSQALAPAR VKLVIIRLLA
LSIFAHTHNE SEAAATLFLY EPDLVPHISD LLQLESQIRH TVSEIAKADA VVPQSFVEAL
LSFLSFLATH TSGGNLVVGA GLVPLLIQVI ENRVAGRIGI VSKTLTLLDN VLYGYSNAFQ
IFVNSRGVET MVQRIEYEVD EHLATSRSAL LKHLLRSIHR MMQSSGTSEG LRGLIDSSLP
KSIKKIMDNR GLFGVAVLPL AINAMATFVH NEPTYLPVIQ EAGLPTSFYD AVEGGLEPSI
EVIQATTNAI GALCLNQAGQ DQLTARPSII PSVISVFTSE KHLKVLQDKE NASLIGSAID
ELIRHHPSLK TQVFDAIGAM LDRIESLGNT WSEKEGKEHL YRLENIIVSY LDIVGKFLES
LFQHTDGLER IGRLIALPCL PYDFATSLLV QVIRTMVEVS PSSTLSSLAN QVKTSLNETR
EFWSVPGGQS NLQFRKLVTL HIRIALLSDI YQGYAHGRSV NSMLLGTLHR SFIWENLVLK
DGLNARALKH LATHIPLAPF FQAIVKRRNP DEAHKKQIHA TSSLVAGIMV EHLYGTVVIS
LVTMLLFDEL LLAFNRAGGK KLLLALSGLK VALHLLHSLV SARPLFDSGQ TQFLVTRKKD
TDPEYFEPHD FLVKMRLAVL PLVSELWRAD WLPSIALGVA KEVVQTLLVI IGGEREPRSA
AERALIRSHN NINAATEYLL AHPELDLLRK PLKADLGRVA LTLVDEQPGI RAFSPTADDL
HEHSLTVRFR LLALILPTLP KWLASHLLVA QSVLMLGEGL RDVSTPLFVG PSFEEPRSIL
LDVGIRLLKI STLSSDELLA DLRLLVQLTR DHHSALVFVE REGLSLLALQ VHLIIILLRHI
IEDKTTVISL MRHEIKRRFA HPQVLEATNF VRHTSALVLR DPEAFLEASK ATLKLAQIES
VTQFLITELY RVGKYACFLM QCLTELLFSY NSCKIAFVAY SKKKHRPTLL NFLLVDMISH
QPFSTQKRGI LCNWAKSVIL ALCVDVGKDS SGEITSVRKF VLDGLSKAIK ESSTSELLEA
HYGKLIALSD LCHRLLTVRT ETPLHMAKLM LEKGFVSTLT SALNDVDLNY PNVRTLVTTI
LKPLEQLTRV AIKMGRDDTP DLYRNSSLGM YTDPTSHPLL VDSGDRPDFQ PLPTVLRWSE
ESKITHGQYA TDRPTRFINH LINALLPAAR ERVTVMIGGN PVDITDTGID PTFLEALPDD
MREEVLNQHF RERRTERATQ AAESHISPEF LNALPPEIRA ELLDQEAEQP TEMDPASFLA
TLDPQLRQVV LLEQDDGFLQ TLPAIPSHRE AVQLLDKAGV AVLVRLLFYP HKDTLQKILV
NICENAKTRA EVLNLLLGVL HDSSGDVASV DKSFAQLTVR PHLVAQRSIS ALTYIVSSND
LSSVFFLTEH ELPPGMRKSR KGKTKERQSQ THFPIALLLG LLDKKVLLKT STMIESVAAL
LATVTRPLAT LKKSLLQHPP QISEQILRLV VNILTVGECS SKTFQHCLSL IQNLAYIPNA
KDTVASELRA KAQDLGRSIL RDLEDLVILL STKFSPASAD QAKLLRVLKT IDYIFTIYDS
FNFAPLWKEL SACLSAVDDS GNVETLATVL LPLIESLMVV CKNVDSKDTA HDLFLSFTDA
HRKALNLMVR NNPSLMSGSF ALLVNNPRVL DFDNKRNYFS QQLRRRREHH TTLQLNVRRA
RVFEDSFHYL QRKTGDQIKY GKLSIRFYEE EGVDAGGLTR EWFTILARQM FNPDYALFQP
CAADKLTYQP NRASAINPEH LSFFKFIGRI IGKAIYDGRL LDAHFARSLY RQILGKPVDY
RDVEWVDPEY YNSLIWILEN DPSHLELTFS FGENKVVDLK ENGSTIPVSQ ENKREFVQLS
AQYRLISSIK EQIDALLGGI YEIIPKDLIS IFNEQEVELL ISGTPDIDVD EWRAATDYNG
YTSSDPVIVW WWRALKSFNR EERAKVLGFA TGTARVPLSG FTDLQGVQGN QRFSIHKAYG
DPDRLPQAHT CFNQIDLPQY TSYEKLRAQL LLAITEGSTG FGFA

>CP

AELISKLLNT PNDDLADALA EIDVWKWPRS DLNAWTKVLD KFDEILEEVI RDWDLQKLQV
NVFTPLTKSL ICEILKFERL LLENSTNRKT FNSYDRLNDL LFTSDLDVLI LALNLLLRPS
QQYSAQPAVS HALNISTPRL QSLAKRWSGL REYGVTLFDL ARNNTAQLDV LPADAREVNF
VYYKHVDERI IREKEVTEIV ADVLEAHTLS SDKFELICRT RCAQALFPER EKLIIVRLLA
IAIYGHTHNE SQASSALFLY EPDLPMHVAE LLQLDQNIRN TIVETSNPES KLPHSFVDAL
LSFVTFLASH GAGGNMVVGA GLIPLLVQVI ENRLVQRLPM VSKAMQLVDN VLYGFANAFQ
LFANARGVDV LVGRIQHEVD GELPVARAAV LKHTLRSLHR MMQSSGTSEG LRGLIDSSVL
ASVKKVVQYR GLFGPSVLPI AINIMATFVH NEPTCLPTIQ EAGLPLVFYK AIEDGIEPVI
EVVQSIPNAI GALCLNQAGQ DQLAARPSII PGILSIYTSE RHLKMLKDKE NAAMVGTAID
ELIRHHPSLK TAVFESIKAT MTKIETLGEA YEVPENMKEW YGLDNIIISF FDVFCRFLEG
FFQHADGLER IGRMTALPCL PYDFANSILI QVLRLMVEVG PNEALFYFSK LLATSLAETQ
PFWGSLGPDS NYQFRSLVIL HIRTTLVADI FLAYTHGRGA MGLLLGALHR ACVWENILLK

DTLNAKALKH LTHGLPLSPF FQSIAKRRNP DPVQKKQILN ASSVVANIMV EHLYYSVMLG
LVTILLVDEL LFSFHKIGGQ ELTRAQGGLK VALHLIHPLI SAQPLFESGQ TLLVMTRKKE
TDADYFESHH FLVQLRLACA PFLQRVWEAP WILSCPLPLV KTTVQAILEL TSAENEPRSA
AERALVRTHN NVNAATELLL AHPELQAARA PILDGLCQRS LQLVDNFPSD IGFHTTSHDG
KEEALAVRCR VLALVLPNVP KWLAAHLLVT EMLLSVGQQP SNVAVPILAG PLYPEARPTI
FSYCLRLLAV PDLPRDELLS TLRILVVVTQ DHDTASQLVE QDVISSLGSQ SYVAIILRHI
AEDPGTVRSI MTQEIKRFFS QPHVIDVHGF LRHCSSLALR DPTIFVQATR SLCQLQQPES
LVHFVINELI KSLKYSCFLM QCLTELLFSY DACKAAFLSF SPKKPRTAAL QFLLSELITF
GAVDNKGKAT LCGWAMSVIV ALCIDTSKEL SNELVSIRKF VLDSINRAIK ELPSSESLDA
RYGRLLALSD LCSRLLTVRF ENSTHVAKIM LEKNFVATLT NALAEVDLNY PHVRSLVAAI
LKPLENLTRI AIKMSREEMP DLYRNSALGM YSENTVHPLL MDKSESGGFD PLLTVQRWAE
ESKIIHGNHV AERAGKLANH IIIALLPAAL QRVTVMVHGS SVDITDMGID PTFLEALPDD
IREEVINQHV RDQRAAAVER PSESQISPEF LDALPPDIRA ELIQQERMEG EEMDPASFIA
SLEPPLRQVV LLDSDDGFIQ TLPKPPPPRD AIQLLDKSGV ATLVRLLFFP QKNILFKVLV
NICENSKTRA ELFNLLLSVL QSGPGDLAAV DRSFAQMTTR PDLVAQRSVE ALTYIVNANE
LSSLFFLTEH ELPYALRKTK KGKGKEKQPQ THYPIVLLLG LLDRQSLLKT TTTMDSVVTL
LSTVTKPLVG LKKVVLQNPP QIPHSVLRLI VNILTIGECS SRTFQQSLTL ISNLSYIPDG
RDVIAQELKV KAQECGHNIF LDLNELMASL TSKFSSPSSD QAKLLRVLKT IDYMYTIYES
FNFTTLWRRL GDCLAAIEER PEIEHIATVL LPLIESLMVV CKHVGSKETM EDLFVSFTDA
HRKILNSMVR TTPSLMSGSF SLLVQNPRIL DFDNKRNYFT QQVHRRREHH STLQLNVRRA
RVFEDSFQQF HSKDGERIKH AKLNVRFYDE EGVDAGGVTR EWFQILARQM FDPNNALFQP
CAADRLTYQP NKNSWVNPEH LSFFKFVGRV IGKAIYDGRL LDAYFAKSIY RQLLGKPVDY
RDVEWVDPEY YNSLCWILEN DPTPLELTFS FGRNRIFPLK EGGEQISVTN ENKREFVQLS
ASFRLYSSIK EQIEHLVSGF HDIIPKDLVN IFNEKELELL ISGTPDIDVD EWRAATEYNG
YTSSDPVIVW WWRALKSFNR EERAKVLSFA TGTSRVPLGG FVDLQGVQGV QRFSIHRAYG
DPDRLPQAHT CFNQIDLPQY SSYEMLRQQL MLAISEGGEG FGFA
>SL
VEVIEKLANT SNDDLAEVLS EVDSWKWPRS DLNAWIKVLN KFDVVLEEAI RDYDVDKIQV
NIFTPATKKV VCEILRFERL LLDNSTNRKM FSSYDRLNSL LFTSDLDILI LALNLLLRPS
QQYSAQPAVS HALNISTPRL QSLCKRWPSL QESGISLLDL ASNTASHVGA IPTDVREVNF
TYYRHIDEEI LSKKEPMDVL ADIVEVHALS DDKFELLCRI RSAMALAPGR EKLIIVRLLA
IAIFGHTHSE SQATSSLFLH EPDLMTHIAE LLQLDHGIRK TVADVANPEC QLPHSFVDAL
LSFVTYLASH AAGGNMVVGA GLVPLLVQVI ENRLTQRLAM VSKIMQLVDN VLYGFTNAFQ
LFCNGRGVET LVGRIQHEID GELPVARAAV LKHTLRSMHR MMQSSGTAEG LRGLIDSSIL
QSIKKIIQYR GLFGPSILPM AMNIMATFVH NEPTSLPTIQ EAGLPEMFYQ AIEAGLEPVI
EVIQAIPNAV GALCLNQVGQ DQLSARPSII PGILSIFTSE RHLKVLQEKE NAVLIGTGID
ELIRHHPSLK APVFDAIKST LGKIEELGQS FVVPEELEQW YGLDNILVSY IDVVCRFLEG
LFQHEDGLDR LGRLTALPCL PYDFANSVLV QVMRTMAEVG PNEALFYLMT LVGSSLAETQ
TFWQSLDSQS NQHFRSLITL HVRITIMADI FSTYAHGRAA ISLLLGALHR ACIWENILLK
AGLNAKALKH LTHGLPLAPF FQAVVKRRNP DPTQKKQILT SSAVIAEIML GHLYHSIMLG
LITILLVDEL LLAFHRIGGQ ELIHAHGGLK VALHLLHPLV SSKPLFESGQ TLLVMSRKKD
DDPDYFEPHN FLVRLRLAIT PLLHNIWDAF WLVSAPLPLL KSVIQTVLEL TGGENEPRSA
AERALIRSHN NVNAATELLI SHPLLNIARE PLKSNLVRQT LRFVDEHPSI IAFSPAAYDL
QEQPLAVRCR LLALVLLSIP KWLAPHLLVI ESLFTIADQP PVATIPMECG TSYHDARPIV
FEFCLKLFAN PNLPRDELLS ALRLLVLLTR DYDVARELMK RNGVALIASL SYVAIILRHV
AEDKSALRGI VLQEVNKLFS QPRVLDVASY MRSCGTMALR DPHVFLQVTQ SVCQLQEPES
LVHYLIGELI KSIKFSCFLM QCLTELLFSY DACKTAFLSY VPKKPKTAAL HFLLSELVSF
GTINARARIT LCSWAMSVIV ALCVDCSKEV SPELVSVRKF VLEAVSRAIK DLSPAENPDA
RYGRLLALAD LCNRLLTVRF ENSTHIAKIM LEKNFVSTLT TALAEVDLNY PNVRGLVASI
LRPLENLTRV AIKMSREETP DLYRNSSLGM YPEVTVHPLL LDPPAGHAFD PLLTMQRWTE
EAKILNGKFV SERGSTLANH VTLALLPAAI ERVTIMIHGN SVDITDMGID PTFLEALPDD
IREEVINQHV RDQRAARIER PPDSQISPEF LNALPPEIRA ELIQQERIEP GDIDPASFIA
SLDPQLRQVV LLDSDEGFIQ TLPKPPSPRD AIQLLDKSGV ATLVRLLFFP QKNLLFKVLV
NICENAKTRA ELFNLLLSIL QSGPGDLSAV DKSFAQMTTR PDLVAQRCLE ALTYIVSANE

LSSLFFLTEH ELPVGLRKSK KGKGKEKQPQ THYPIVLLLS LLDRHSLLKT PSNMESIVAL
LSTVTKPLTS LKRVILGNPP QIPHSVLRLI VNILTIGECS GRTFQQSLML IQNLSHISDA
RDVIAQELKV KAQEFGQNIY IDLNELAMAL ASRFSLPSSD QAKLLRVLKT IDYMYSIYES
FRFTPLWRRL GDCLATIEEK PDTEHIATVL LPLIEALMVV CKYVGSKEAM EDLFVNFTDT
HRKVLNLMVR NNPSLMSGSF SLLVHNARVL DFDNKRNYFG QQLHRRREHH VTLQLNVRRA
RVFEDSFQYL QRKTGDQIKY GKLSVRFYDE EGVDAGGVTR EWFQILARQM FDPNNALFQP
CAADRLTYQP NKNSWVNPEH LSFFKFVGRV IGKAIYDGRL LDAYFAKSLY RQILGKQVDY
KDVEWVDPEY YNSLCWILEN DPTPLDLTFS FGRSRIFPLK EGGESIPVTQ ENKREFVQLS
ANFRLYSSIS EQIENLLAGF YEIIPKDLIT IFNEQELELL ISGTPDIDVD EWRAATEYNG
YTSSDPVIVW WWRALKSFNR EERAKVLSFA TGTSRVPLGG FVDLQGVQGV QRFSIHRAYG
DSDRLPQAHT CFNQIDLPQY SSYEMLRQQL LLAINEGGEG FGFA
>SS
AILIATILRT PTESLPDVLS PIETWKWPRS DLHWWIKVLN KFDIVLEEII RDYDVDKLQV
NEFTPLTKKI LLSVLAFEKL LLENSTNRKL FNSYDRLSSL LFSADLDVVV ADLQLLLRPA
QQYSAQASVS HALNISTSRL QALAKRWPNI RENDLEFSAL ASKKQAEVEG LPNEASEVNY
TFYRHLGPLA HSSKSAINIL ADTVESYSVP NEKFELLAKI RVARSLGKGR VKLVTIRLLA
IAVYCLTQNE NTAQTSLFLY EPDLIAQVAD FLQLDRGIRK TVTDIANPQS TLPNSFVEAL
IAFVTFIASH ASGGNMVVGA GLIPLLIQII ENKNALRLPI VSKTMQLVDN ISYGFPNAFQ
LFCNARGVDV VVKRIEEEVN GHLSIPRSSV LKHTLRTLHR MMQTSGTSEG LRGLIDSSLT
KSIKKIIEHR KLFGSSVLPI AIHVMSTFVH NEPTSLGVLQ EAKLPETFYS SVEDHIEPSI
EVIQAIINAI GALCLNQTGQ EQFTARPNII PSIFSIFTSD RHLKVLSEKE NAVVFGTAVD
ELIRHHPSLK DKVFAAISAI FEQMEQKGKD LPEDTENESS YKLDNIIVAF VDVVSRFLEG
MFQHTDCVDR LSRIIVLPCI PYNYACSVMV QLLRVMTEVA PTQTVNKILE FVKVALDDTK
EFWSTMDGKS NDEFRKLVSL YTRVTLLADV FSTYTHGRSA LSLVLGSLHR ACIWENIVLK
GEFNAKALKH LVAEIPLTPF FQCVVKRRNP DTTAKRQVAG IIDQVAKIVL SHLYSTVMMG
LITLMIFEEL LWSFTKFGGE ELVHVSGGLK VALHLLHILV SSKPLLDSPQ TVLITTKKKE
TDPDYFEPHN FIVMLRAIVL PFMQELWDAP WLVNAPVSVC KSVVQTMLEL MAGENEPRGA
ATRALTRMRN NVAAAAEYLL AQPELNDARE ALKETLGRRS LQLLDEHHSI KDFSPSAYEV
HEQPLAIRCR LLALVLPVVP KWLAPHLLVT EALLVAGDEP KPATLPVSDE PCYPAAGDKI
FHICLKFLNY PDLPQDELIA VMRLLVYLTR EPSKARELVK LGGVASLGCQ VYVAIIFRHI
IEDKTTLDNI MRQEIKRFFT NPRITDVTSF IRNSGGMALR DPKTFVHTVE SMCQLDQSEA
MLHYLISELM RVGKYACFLM QCLTELLFSY NSCKMAFVNY TKKKHRPTML NFLISELTSF
GTIDSKRRVM LCNWAMSVIV ALCLHIPKDV PADLITVRRL VLDSINRALR DPLPVESIDE
RYGRLLALSD LCHRLLTVKP DGALHMAKLM LEKNYVATLT TALAEVDLNY PNVRTVVTSI
LRPLEQLSKI AIKMGREETP DLYRNSALGI YPESAVHPLL VDRSDIQDFG PLPTQQRWAD
EAKMANGKFI GDRVNRLSNH VILALLPAAL ERVTVLINGN PVDITDTGID PTFLEALPDD
MREEVLNQHF RERRMARQEP TADSQISPEF LDALPPELRA EILQQERAEP VDIDPASFLA
SLDPHLRQVV LMDQEDGFLQ TLPKVNVPRD AIQLLDKTGL ATLVRLLFFP QKSLLLKIFV
NLCENSKTRG ELLNLLLNIL QDGTGDVAAV DKTFAQLSTR PNLVVQRSLE ALTFIVSANE
FASRFFLTEH EIATGLKRSR KGKGKEKQPQ VQYPIVPLLA LLDRQTLLKT PSMMDSLAAL
LATITRPLAT LKKIPPLQPP TIPQASLRLI VNILTAGECS ARTFQSTLAL IQHLAFLADV
RDTIAMELRA RAQDLGHNIY KDLDDLVQVL AAKFSPASSD QAKLLRVLKT IDYMYSIYES
FRFTPLWSRL GDCLDIVEEK PDVEHMATVL LPLIESLMVV CKHVGVQEAI EDLFTSFTDA
HRKVLNLMVR NNPSLMSGSF ALLVQNPRVL DFDNKRNYFY QQLRRRREHH GTLQLNVRRQ
RVFEDSFQYL QRKTGEQIKY GKLSVRFYDE EGVDAGGVTR EWFQILARQM FNPDYALFQP
CAADKLTYQP NRASWVNPEH LTFFKFVGRI IGKAIFDNRL LEAYFARSVY RQLLGKLVDY
RDVEWVDPEY YNSLVWILEN DPTPLDLTFS FGVTSVVELK EGGATIAVTN ENKREFVQLS
AQYRLTNSIK EQLNALLGGF YDVIPKDLIS IFNEKEVELL ISGTPDIDID EWRAATEYNG
YTSSDPVIVW WWRALKSFNR DERAKVLSFA TGTSRVPLGG FGELQGVQGV QRFSIHKAYG
DSDRLPQAHT CFNQIDLPQY SSYEKLRQQL LLAVNEGGEG FGFA
>HI
AELINKLSNT PDEDLHEVLG KVDAWTWPRS DLNAWIKVLN KFDAILEDVI RDYDVDKLQV
NVFTPSTKKT VCEILRFERL LLENSTNRKT FSSYDRINGL LSSSDLDVVV LALNLLLRPA
QQYSAQPAVS RALSISTPRL QSLAKRWPNL REYGVSLVDL VAKGKESVEA LPAEAREVHF

TFYKHVDVHT LESKPTMDVL ADLTDAFNVP EERFELMCRI RSAQVLLPGR EKLVIIRLLA
NAIFVHTHSE HQALSSLFLY EPDLISHISE LLALDSGVRK TVADVASSES TLPQSFVDAL
LSFVTFLASH AAGGNMVVGA GLVPLLIQIL ENRLANRLPV VSKAMQLVDN VLYSFANAFQ
LFCNNRGVDV LVDRIEYEVD GQLPVVRAAA LKHILRSTHR MMQSSGTSEG LRGLIDSSLL
KSIKKIIEHR SLFGPSVLPL AINIMACFVH NEPTSLSIIQ EANLPEAFYK AVEAGLEPVI
EVIQSIPNAL GALCLNQAGQ DQLEARPTII PGLFSIFTSE RHQRVLQDKE NAVLIGTAVD
ELIRHHPFLK AAVFEAIKST LSKIEELGNA YLVPDGIKSW YSLENIIISY IDVHCRFLEG
LFQHSDGLER LGRLTELPCV PYDFANSVLV QVIRTMVDAD RHGTLTFLAK LVKVSLGETK
EFWNGVKQES NSTFRKLVTL HIRVTLLSDI YATYSHARSS TGLLLGSLHR VCIWENVLLK
SAFNGKALRH IANQIPLSPF FQAVVKRRTP DPPQRHQIVE SAAVVAEVML KHVYYTFMLG
LVTTLLVDGL LDAFFRKGGQ ELVHAFGGIK VALHLLLPLV SPKPIFDSSQ TIILVTRKKD
GDPDYFEVHN FLVQLRLVVF PVARDLWEAS WLVSAPQGLI RSVVQVTLEI VNGESEPRAS
VERALMRTHN NVNAATELLL ANPELDVLRE EVKPNLGALA LRLVDEHHAI KRFAPDAAGS
REQHLAVRCR LLALVLSTVH KWLAALLLVA EALLVLGEEP RSITLPLLEG PPYTEARPVL
FDVALGILRL TNLPRDELLA SLRLLVLLTR DHEISLEFIK RDGVPLLGSQ SYITIIILRHI
VEDPAVVRFI MRQELQRLFS LPRVTDVQNY VRHCSSMALR DPETFVEVTQ SLCELDRPET
VVHFLLAELM KAVKYCGFLM QCLTELLFSY DICKVAFLSY SPKRHRMVAL HFLLYDLITY
GTISAHKRVI LCNWAMSLIV ALCVETSKDV SPELISVRKF VLEAVNRAIK DLAPSDTMDA
RYGRLLALSD LCNRLLTVRF EGPTHIAKIM LEKNFVSTLT NALADVDLNF PNVRGVVAAI
LKPLEVLSKI AIKMSREETP DLYRNSSLGM YADIVTHPLL LERPESRQFD PQITLQRWLE
EGKILHGKFS ADRFLKVPNH IILTMLPAAI ERVTVMIHGS PVDITDTGID PTFLEALPDD
MREEVLNQHV RDQRAARVER PADSQISSEF LDALPPEIRA EILQQERIEA AEIDPASFIA
SLDPQLRQDV LMEQDEGFIQ TLPKPPPPRD AIQLLDKGGI AVLIRLLFFP QKSSLFKVLV
NLCENSKTRT ELFNLLLNIL QDGTGDLASV DRSFAQMTFR PDLIAQRCLE ALTFIVSSNE
LSSLFFLTEH EVPASFRKSR KGKGKEKQPQ TQYPIVLLLS LLDRESLLKT PSILESVVSL
LDTVTRPLTS LKKILLAHPP QISHSVLRLI VNILTVGECG AKPFHHSLSL IQHLSYIPDA
REVIAQELRA KTQDFGHSLY QDLDELANAL ASKFSSPSSD QAKLLRVLKT IDYMYSIYES
FRFTSLWKRL GDCLSIIEQR PEIEHIATVL LPLIESLMVV CKYVGSSESM EDLFVTFTDA
HRKVLNVMVR NNPSLMSGSF SLLVHNPRVL DFDNKRNYFN QQLRRRREHH GTLQLNVRRQ
RLFEDSFQYF HRRTGEQIKY GKLSVRFYDE EGVDAGGVTR EWFQILARQM FDPNNALFEP
CAADRLTYQP NKASWVNPEH LSFFKFVGRV IGKAIYDGRL LDAYFARSLY RQILGKPVDY
KDVEWIDPEY YKSLCWILEN DPSLLDLTFS FGVTKIVDLK ENGASIPVTM ANRREFVQLS
AQYRLYSSIK EQLEHLLSGF YEIIPKDLVA IFNEQELELL ISGTPDIDVD EWRAATDYNG
YTSSDPVIVW WWRALKSFNR DERAKVLSFA TGTSRVPLSG FGDLQGVQGV QRFSIHRAYG
DSDRLPQAHT CFNQIDLPQY SSYEMLRQQL LLAINEGGEG FGFA
>SH
AELINKLHNT PNDELHEVLS EIDVWKWPRS DLNAWIKVLN KFDAILEEYI RDYEVDKVQV
NVFTPITKKT ICEILRFERL LLENSTNRKT FSSYDRINSF LSSSDLDVVV LALNLLLRPA
QQYSAQPAVT RALSISTPRL QSLAKRWSNL REYGVSLVDL VTSGKHIVEE LPSEAREVHF
SFYKHIDAPT LESKPTMDIL ADVIEKYDVP EERFELMCRI RTAQVLFPGR EKVVIVRLLA
NAIFVHTHPE SQAMSSLFLY EPDLIPHIAE LLGLDRGVRK TVNDVANPDS TLPQSFVDSL
LSFVTFLASH AAGGNMVVGA GLVPLLIQII ENRLPQRLSV VSRAMQLVDN VLYSFANAFQ
LFCNNKGVDV LVDRIQYEID GQLPVVRAAV LKHILRSMHR MMQSSGTAEG LRGLIDSSLL
KSIQKVMEHR SLFGPSVLPI AINIMATFVH NEPTSLAIIQ EAGLPETFYK TVDAGLEPVI
EVIQSIPNAI GALCLNQAGQ DQLTARPTII PGLFSIFTSE RHQRVLQDKE NAVLIGTTVD
ELIRHHPMLK TSVFEAIKST LSKIEDLGNA YVVQDNVKQW YKLENTIISY IDVLCRFLEG
LFQHSDALER LGRLTALPCL PYDFGNSVLV QVIRTMVEAA PAETMTYLAK LVRTSLDETK
EFWASQNEDS NQSFRKLVTL HIRVTLLSDI YSTYAPSRGA NSLLLGTLHR VCVWENVVLK
SKLNGKALKH LANQIPLSPF FQAVVKRRNP EPSQKQQILS SAGVVADILI KHLYHTFMLG
LVTTLIADEL LDAFYRKSGQ ELIHAYGGIK VALHLLHPLV SPKPIFESSQ TIILLSRKKE
TDPDYFEPHN FLVKLRIAVL PLTRELWEAE WLAMAPHSLI RSVVQVILEI VNGDGEPRPA
AESALTRTHN NVNAATEFLL ANPELNTARE SLKADIGRLS LRLVDEHPAI KEFSPQARGE
KEQALAVRFR LLALVLAGVA KWLAAMLLVS EAMLTLGEEP RTISLPLATG PLYLDARPAL
FDLCIKLLSV PKLTRDDLLA SLRLLVLFTR DHYQALAFVE RGGIPLLGSQ SYITIIILRHI

VEDDDELAVI MRNEIARFLL HPRVIDVPNY IRHLNSMALR FPIAFIKITS EMCKLDHPET
LIHFLLGELI RVVKYSGFLM QCLTELLFSY DFCKTAFLSY SPKKHRTAAL HFLLNDLVSY
GTVGEYKRVT LCNWAMSVIV ALCVDANRDL TPEIVSTRKF VLEAVSRAIK DPAPSDSVDA
RYGRMLALAD LCQRLLTVRF ENPTHIAKIM LEKNFVATLT TALAEVDLNF PGAKSLVACI
LKPLELLSKI AIKMSREETP DLYRNSSLGM FPEVVTHPLL LEPPHAREFD PFSTLQRWGE
EAKVLHGKFS AERFMKLANH LILSMLPDAI ERVTVMIHGS AVDITDTGID PTFLEALPDD
MREEVVNQHI RDQRAARVER PPDSQISSEF LDALPPEIRA EILQQERVEA AEIDPASFIA
SLDPQLRQDV LMDQDDGFIS SLPKPPAQRD AIQLLDKSGI AVLVRLLFFP QKNTLFKVLV
NLCENSKTRT ELFNLLLNIL QDGATDLAAI DRSFSQMTVR PDLIAQRCLE ALTYIVTSNE
LSSLFFLTEH ELSIGLKRSR KGKGKEKQPQ TQYPIVLLLG LLDRQSLLKT PSMLESVVSL
LDTVTRPLTS LKKILLANPP QIPHSVLRLI VNILTVGECG AKPFHHSLSL IQHLSYIPDA
REVIAQELRA KTQDFGTSLH QDLEELSNAL ASKFSSPSSD QAKLLRVLKT IDYMYSIYES
FSFTPLWRRL GDCLSVIEQR PEIEHIATVL LPLIESLMVV CKYVGATESM EDLFVTFTDD
HRKVLNVMVR NNPSLMSGSF SLLVHNPRVL DFDNKRNYFN QQLRRRREHH GTLQLNVRRQ
RLFEDSFQYL QRKTGDQIKY GKLSVRFYDE EGVDAGGVTR EWFQILARQM FDPNNALFEP
CAADKLTYQP NKASWVNPEH LSFFKFVGRV IGKAIYDGRL LDAYFARSLY RQILGKPVDY
RDVEWIDPDY YKSLCWILEN DPTALDMTFS FGVMKIVPLK EGGETLPVTL ENRREFVQLA
AQYRLYSSIK DQIENLLSGF YDIIPKDLVS IFNEQELELL ISGTPEIDVD EWRAATDYNG
YNSSDPVIVW WWRALKSFNR DERAKVLSFA TGTSRVPLSG FGDLQGVQGT QRFSIHRAYG
DPDRLPQAHT CFNQIDLPQY SSYEKLRQQL LLAINEGGEG FGFA
>PS
ADLIAKISDT PNELLGTALS QIETWAWPRS DLNAWIKVLN RFDEILEDCI RDYEIDKIQT
RPFDNDIKYL VSGILKFERL LLENTTNRKM FASYDRIKSL MSTSDLDILL LALNLLLRPA
QQYSAQPHVS QALSISTQQL LCLAKRWPHA RDNNLSLVDL ASQGVEQVNG LPPEAREVNF
TFYKHIDHQT IESQSAMDVL KEAVNKYEIP EEKYELLCRI RAAGVLVPSR QKYLTVRLLA
ISIFCHTHGE TQAQSSLFLY EPDLVAHIAE LLQSDKGVRR TVASVSDPLS SMPQSYVEGV
LSFVTYLASH TTGGNMLVGA GLVPLLIQLL DNRLPNRLAI VSKTVQLTDN VLYGFPNAFQ
LFVNNRGVDT LVGRIQSEVT GQLSVSRAAV LKHTLRSMHR MMQSSGTAEG LRGLIDSSLL
KSIHQILENR TLFGPSIVSL AINIMATFIH NEPTCLTVIQ EAKLPEVFYK SLDAGIEPSI
EVIQAIPNAL GALCLNQTGQ DQLAARPTVI PGLLSIFTSE KHLKVLQDKE NAVLVGTAVD
ELVRHHPSLK APVFEAIKSS LSKIEDMGSE FEVPAKLKPW YTLDNIVVSF IDVFGRFLEG
LFQHVDGLTR LGRITALPCL PYEFANSVLV QVIRTLAEVA TNETLAFLSG IVKESLTETK
DFWFKVGGDS NHEFHKLTVL HTRITLLSEV YATFGHPRTS VALLLGALHR TCVWENIVFK
VDPNGKALRH LVTQIPLGPF FQAIVKRRQL ERTQRQQFLH SGSVIADVLV QHLYHTVMLG
HAAILLVEEL VFAFYRAGGQ ELVHSYGGLK VALRLLEPLV TAKSLLEATQ TQLVATTKPE
TDSAYFEARN FLVKLRTAIL PVVRTMWESS WLPQVPVGVS RSIASVVLEL AKGEGEPRSA
AERALIRMHN NVAAAAELLL AQPELAKARE PLRNNMGKLA LRLVDEQPSI KEFQTTAYDV
HEQPMALRFR LLALVMPVLP KWLATHMLVA ESLLTAGEDI RSITLPLMTG PTHPEARSFM
FDLCLRLLAV PQLPRDEYLS SLRLLVFLTR DHSTASEFIA RGGLPLLGAK SYISLIVRHI
VEEPRVLQHT MQREIKRLLA HSNFPDPSHY VRTCSAAALR DPAAFIQATE AVCQLHRPDE
VVYCLISELM QTVKYPCFLM QCLTELLFSY DACKLAFLSY SPRKHRTAAL NFFLYDLISF
GSIHARKRVT LCNWAMSVLV ALCVNGSKDV SDDLVSVRKF VVDAISRAIK EVIPSDTIDA
RYGRLLALAE LSHRLLTVRF DSPTHIAKVM LEKNFVSHLT NALGEIDLNY PNVRGLVTAL
LKPEQHLTKI AIKMSREETP DLYRNSSLGM FPEATTHPLL LDRPENRGFD PLHTGQRWAE
ETKMLYRTSE SERLAKLADH VVLALLPAAI ERITVMIHGS PVDITDLGID PTFLEALPDD
MREEVLNQHV RDQRAARVER PADSQLDAEF LDALPPELRA EIIQQETLEP ADIDPASFIA
SLDPQLRQVV LMDQDDGFIQ TLPKPNAARE SIQLLDKGQI TVLVRLLFFP EKNLLFKVLV
NVCENGKTRA ELLNILINIL QDGTGDLAAI DKSFSQMSVR PELIAQRCLD ALTYIVNNNE
TSSLFFLSEH EIPAGLRRSK KGKGKEKQSS THYPVVLLLG LLDRPTLMRT PSILESVVGL
LATVTRPLAT LKRPLLTDPP QIPQHALQLI VNILTMGECS ARTFQQCLAL IQHLSYMADA
REVIAQELRS RAQELGQNIQ ADLDVLAKAL PSKFSSPSSD QAKLLRVLKT IDYMYSIYES
FRFTPLWKRL GDCLAIIEQR PEVEQVATVL LPLIEALMVV CKYVVLDESM EDLFVTFTDT
HRKVLNLMVR NNPSLMSGSF SLLVHNPRVL DFDNKRNYFT QQLHRRREQH STLQLNVRRA
RVFEDSFQYL QRKTGDQIKY GKLSIRFYDE EGVDAGGLTR EWFQILARQM FNPDYALFQP

CVADKLTYQP NRASWVNPEH LSFFKFVGRI IGKAIYDGRL LDAYFARSFY RQLLGKPVDY
RDVEWVDPEY YNSLCWILEN DPTGLDLTFD FGVTKIVPLK ENGTQIHVTN ENKKEFVQLS
AQYRLYTSIK DQIDAILAGF YDIIPKDLIS IFNEQEVELL ISGTPDIDVD EWRAATEYHG
YTSSDPTIVW WWRALKSFNR EERAKVLSFA TGTARVPLGG FGDLQGVQGV QKFSIHKAYG
EQDRLPQAHT CFNQIDLPEF SSYEMLRQQL LLAINEGGEG FGFA
>SC
AELINKLLAT PNDDLADVLS QIESWKWPRS DLNAWIKVLN KFDAVLEEVI RDYDVDKLQI
NPFTPATKRT VSEILRFERM LLENSTNRKM FSSYDRLNSL LFTSDLDILI LAENLLLRPS
QQYSAQPSVS HALSIQTPRL LSLAQRWPRA REYGLSLYDL ATKGKAKVEA LPPDAREVDF
TFYRHIDEQT LQSKPAIEVL ADAVETYSVP EEKLELLCRI RTAQCLAPGR QKLVISRLLA
IAIFAHTHPE SQATSSIFLY EPDIITSIAE LLQVDRGVRA IVADIARPES TITNSYVEAI
LGFLSYIASH ASGGNMIVGA GIVPLLIQLI DNRLPKRLTM VSKTMQLVDN VLYSFTNAFN
LFCSSRGVTT LVDRIGYEVD TEMPVARSAV LKHLLRSLHR MMQASGTSEG LRGLIDTSIL
KSIKKIIEHR STFGSNVYPI AINVMATFVH NEPTALPIIQ ETGLPEAFYK AVESGVEPAI
ETLQAIPNAI GALCLNEAGQ AQLAARPSII PSIFSIFTSE SHIKVLLDKE NAVLLGSAID
ELIRHHPTLK TSVFQAITAT LGKIETLGNE FVVPNNIRHW YQLENIIVSF VDIFNRFLEG
LFQHTDGLDR LAKLTALPCL PYDFASSVIV QVIRTMAEVS TTETMALLTK SIKESLEAAG
FFWMLLDKQS NRKFRLLTTL HVRLTLLSDV FATYTPTRVS NSLLLGNLHR ATVWENILLK
AGLNAAALKH LTHVFPLAPF FQALVKRRNT DQAFKKQISD TASIISKIAI DHFYYTAMLG
FTAVLLVDEQ LWNWYRAGGQ ELAHALGVLK VALHLLLPLI SSKPLFESPQ TALVMTRKPE
TDPDYFAPHD FLVRLRLAIL PLLQSMWQAP WIKNVPPTIR QPVIHAVLEL IGAENEPRSA
VERALRRTHN NISAATELLL AHPELNAIRE PLRASVSRIA LTIVDEDPSV KTFSPYAYDT
QEQPLANRCR LLALVLPPVP KWLAAHLLVV EGLLTLAASP KAITLPVSVG PALVEARKVV
FDFCLRLMAI PDLPDDPVLS SLRIFVICTR DHSMACEFVK KDGLSRMGSS SYVAIILRHV
AEDLPTVESI MKQNIKRFLN QPRNVDVLTY TRNCSAMALR DPKAFLRATE ALCQLASPDA
MVHYLIAELM RVVKYACFLM QCLAELLFSY DSCKMAFLTY SPKKYKSSTL HFLLSELVTY
GAINSKSRLA LCNWAMSVLV ALCVDSSKEV PAELASVRKF VLEALSRSIK DAQNVEGQEA
RYGRLLALSD ACYRLLTVKV DTPTHIARVM LEKGFVATLT NALSDVDLNY PHVRQLVASM
LRPLEHLTKV AIRMSKEETP DLYRNSALGM FPEATTHPLL LDGSSSRELE PLLTIQRWAE
ELKIVNGDFA NERATKLANH VTLTLLPSAI ERVTVMINGN PVDITDTGID PTFLEALPDD
MREEVLNQHI RDQRAARIER PADSQISDEF LDALPPEIRA EIIQQEAIEP AEIDNASFIA
SLDPTLRQAV LLDQDDGFIQ SLPKPPAHHD AIQLLDKTGI AVLVRLLFFP QKSLLHKVLV
NLCENSKTRT ELFTLLLNIL NDGTVDVATV DTRFSQLSMK PDLVAQRCLE ALSYIVNENE
ASSLFFLTEH ELSAGLRRSK KGKGKERQPQ LQYPVVLLLS LLDRPTLLKT PSIMEQVATL
LATVTRPLTS LKRVTLSHPP TILHNVLRLI VNFLTIGECT GRTFQQSLAL IQHLSYIPDA
RDVIAEELKT KAQEFGQALY QDLDELANAM VSKFSASTSV QAKFLRVLKT IDYMFTIYES
FRFTPLWKRL GDCLAIIEQK PETEIVATVL LPLIESLMVV CKYVGTKDSM EELFVSFTDA
HRKILNLMVR NNPSLMSGSF SLLVNNPRVL DFDNKRNYFT QQLHRRREHY GTIQLNVRRA
RVFEDSFQHL QRKTGDQIKY GKLSVRFYDE EGVDAGGVTR EWFQILARQM FDPNNALFQP
CAADRQTYQP NKNSWVNPEH LSFFKFVGRV IGKAIYDGRL LDAYFAKSLY RQLLGKPVDY
RDVEWVDPEY YNSLCWILEN DPTPLDLTFS FGVQRIVPLK EGGETLPVTN ENKREFVQLS
AQYRLYSSIK SQIEALSEGF YEIIPKDMIT IFNEQELELL ISGTPDIDVD EWRAATDYVG
YTSSDPNIVW WWRALKSFDR DERAKVLSFA TGTSRVPLGG FTELQGVQGT QKFSIHRAYG
DEDRLPSAHT CFNQIDLPQY SSYEKLRQQL LLAISEGATG FAFA
>PC
AELIDKLADT PHEQLPDVLA EIDHWRWPRS DLNAWIKVLN KFDAIMEDII RDYEIEKVQL
KPFSAFDKRL LCEILRFERL LLENSTNRKT YNSYDRLNSF MTTSDLDVLI YSLNLLLRPA
QQYSAQPAVS HALSLNTNRL TSLSKRWPSL HDFDINLVAL AGQGRAQVDA LPNEAREVSF
TFYRHIDSQT LETKPTMEIW AEAIETYSVP DERFELLCRI RSARALTRAR EKLVIVRLLA
IGLFCHTHPE QTTFNNLFLY EPDLVHHIAE LLQLDRGIRK TITELANAES NIPQAFVEAI
LAFVTYIAAH AAGGNMVVSA GLIPLLVQVI ENRLPNRLYA LSKTMQLLDS ILYGYTNAFQ
LFCNARGIEV LVGRIEYEVD GKISVGRATV LKHIMRSMHR MMQSSGTSEG LRGLLDSSLV
QSVKKVMANR DVFGANVLAI AINIMATFIH NEPTCLPVIQ EAGLPEAFYG VVESGLEPVI
EVVQSIPNAM GALCLNQVGQ DQLASRPGII PGFFSIFTSE KHQRMLQEKE NAVIIGTAVE

ELVRHHPSLK NQVFEAIKQT MARIEELGNA YQVSDDNKHW YVLDNVIVSF IDVLGKFLEG
YFQHTDGLDR LARLTALNCL PYDFANSVLV QVVRTMVEAS TTESLAFIVK IVNESLQATT
NLRDDNAGDS NVAFRNLVTL HVRIMLLSDI FATFSQGRAL TTLLLGSLHR SSIWENVMLK
EKMNMKALKH LASQLPLAPF FQSVVRRRNP DPAQRQRILD AADVVADVAV KHLYYTMMLG
LVTILLIDEL LQAFLRKGGQ TLIHAHGGLK VALHLLHPLI SFKPIVDSPQ SSFLLTTKKD
TEPGWFDPRN LVVQLRIAIL PLLKDIWQSS WLISAPLGVS KSVIHAVLEL LNTDQEPRSA
AERALIRARN NVSHATELLL AQPELNELRE PLKEGLGSRI LGLVDEHPAV KKFSGAALDH
HEQPLSVRFR ILALVLVTIP KWLASHLLVT EALLMMGEEP RSVGLPLETG PKFNEKRPVI
LDFCLRLLHV PQLPRDELLS SLRLFMLLTR DHTLALEFVK RDGISALGTH PYIASILRHI
VEDPATLKQI MQQEVKAFLS NPRLLEVTGF VRSCGAMALR DPQAFVQATA EVCRLSSPDS
LVHFLISELI KSVKYSCFVM QCLTELLFSY DSCKVAFLSY SPKKHRTHAL QFLINDLLSF
GTINAKQQIM LCNWAMSVIV ALCVDTTKDV PPERASVRKF VLDAINRAIK DLPGHETGEA
RYSRLLALAD LCYRLLTVRF DAPTHIAKVM LEKNFVATLT NALAEVDPNF PDIRGVVTGI
LRPLEYLTKI AIKMSREEAP DLYRNSSLGM YPEGTTHPLL LDRSDGRGFE PLLTRDRWAE
EVKSLHGRFE QSRITKIAGH VVLALLPEAI ERVTVLIHGN EVDITDTGID PTFLEALPDD
MREEVLNQHI RDQRAARVER PVDSQISPEF LDALPPELRA EIIQQENLEP AEMDPADFLA
SLDPALRQTV LMDSDDMFIQ ALPKPSTPRE AIQLLDKHAI AVLIRLLFFP QKNLLSKVLV
NLSENGKTRT DIFNLLLGIL QDGTGDLSSI DRSFAQMSFR PELVAQRCLD ALTYITATNE
ASSVFFLTEQ ELPAGLRRSK KGKGKEKQSQ TYYPVVLLLG QLDRQTLLRT PSLMESVAGL
LSLVTKPLTS LKKVLLSHPP NIPQQVLRHV VNILTAGECS SRAFSHTLAL IQHLSFVPGA
RDVIAQELCT RAQEFGQRLY SSLDELATAL ASKFSPASSD QAKLLRLLKT IDYMYSIYEG
FRFTALWRRL GDCLSIIEER TDLEHIATVL LPLIESLMVV CKYVGPKESM EDLFVSFTDA
HRKVLNLMVR NNPSLMSGSF SLLVNNPRVL DFDNKRNYFN QQLHRRREHH GTLQLNVRRQ
RVFEDSFQYL QRKSGEQIKY GKLSVRFYDE EGVDAGGVTR EWFQILARQM FDPNYCLFQP
CAADRLTYQP NKASSINPEH LSFFKFVGRI IGKAIYDGRL LDAYFARSLY RQILGKPVDY
RDVEWVDPEY YKSLCWILEN DPTPLDLTFS FGVTKLIELK ENGAQISVTN ENKREFVQLS
ANYRLYSSIK EQIEALLSGF YEIIPKDLIQ IFDEKELELL ISGTPDIDVD EWRAATEYNG
YTSSDPVIVW FWRALKSFNR EERAKVLSFA TGTSRVPLGG FVELQGVQGV QRFSIHKAYG
EVDRLPQAHT CFNQIDLPQY SSYEMLRQQL LLAIHEGGEG FGFA
>DS
AELIDRLINT PNDDLHEVLS QIDSWKWPRS DLNAWMKVLN KFDAILEEAI RDYDIDNLQV
NVFTPLTKKT VCEILRFERL LLENSTNRKT FNSYDRLNSL MFSSDLDVLI LALNLLLRPA
QQYSSQPAVS HALSISTPRL TSLAKRWPNL RDYDLNLVDL VTKGRAQVEA LPTEAREVNF
VFYRHIDSKT IESKPAMDIL ADAIEAYHVP DEKFELLMRI RGAQAMTHAR EKLVVVRLLA
TAIFGHTHSD SQAQSSLFLY EPDLITHIAE LLQLDRGVRK TVADVAQSTS TLPQAFVEAL
FSFITFLATH ASGGSMIVGA GLIPVLIQAI ENRLPNRLYV VSKTMQLLDN VLYGYTNAFT
LFCNARGVDI LVDRIEYEVD GKLSVARTAV LKHTLRSIHR MMQSSGTAEG LRGLLDSSLL
KSVKKIMQHR AIFGPSALAL AINIMAIFVH NEPTCLPVIQ EAGLPEVFYS VIEKGLEPVI
EVIQSVPNAL GALCLNQAGQ DQLTARPNTI PSLFSIFTSE DHQRVLQEKE NAVLIGTSVE
ELIRHHPTLK EKVFVAIKST MARIEELGTS YSVPDDIKHW YRLDNLIISY IDVFGKFLEG
FFQHTDGLNG LAKLTTLPCL PYDFANSVFV QVVRTMAEAA TNETLAFLVR LVQESLAECK
DFWGSLDEQP NTQFRNLITL HTRVSLLSDI YATYSHGRAT QTLLLGALHR ACVWENIILK
SILNAKGVKH LVGQIPLAPF FQSIVRRRGS DLAQKQKIKE AAGIIADVLV KHLYYTSMLN
AATILLVDEL LVAFVRVGGQ ELSHAFSGLK VALQLIQPTI SAKPLFDATQ TALALTVKKD
TDADYFEPHN FLVRMRAAAL PVVRSIWEAE WLPSAPISVS KLVVHIVMEL LNAENEPRSA
AERALTRTRN NVNAATELLL SHPELTTARE PLKEGIGRRV LSLVDEHPSI QAFLPSAYDV
QEQPLSVRCH LLALVLPTLP KWLSAQLLVI ESLLVLGDSP RSVTLPIAQG PRYAEARNTL
FDFSMRLLGV PTLPKDELIS VLRLIVLLTR DHNIAEAFME RNGVAALGIQ SYIALIVRHI
VENQITLQHV MRQEVKRYFS HPRNVDIGSF VSGCSSTALR DPEAFVKVAE EMCTLSQPET
LIHYLIGELM KTVKYPCFIM QVLTELLFSY ESCKIAFLSY TPKKHRTAAI QFLLSDLMTF
GTINARKQIT LCNWAMSVIV ALCVDTVKDV PTDLVSVRKF VLEAISRALK DLPSSDSPEL
RYSRLLALSD LCHRLLTVRF EVPTHIAKVM LEKNFVATLT NVLSEVDLNY PNIRNVVSSV
LRPLEYLTKI AMKMSREEAS QLYRNSSLGL FAENVTHPLL LDRTEGRSLE PLLTIQRWSE
EVKMLHGKFE QARLSRLHNH VVLALLPAAA ERVTVMIHGA PVDITDTGID PTFLEALPDE

MREEVLNQHV RDQRAAQLER PADSAISAEF LDALPPDIRA EIIQQEAAEP LDIDPADFIA
SLDPQLRQVV LMDSDDVFIQ SLPKAPQSRE AIQLLDKSAL AALVRLLFYP HKNLLYKVLV
NLCENAKSRT DLFNLLLSIL QDGSGDLASI DKSFAQMSVR PELIVQRCLE ALTYIVSSNE
LSSLFFLTEH ELPIGLRRSK KGKGKEKQPQ THYPIVLLLG LLDRPSILRT PSIVESVVGL
LATVTRPLAS LKKVLLANSP HIPHHVLRLI VNVLTAGECS GRTFMQSLAL IQHLSYIPDA
REVIASELRS RAQEFGQSLF AALDELAVAL AAMFSSASSD QAKLLRVLKT IDYMYSIYET
FRFSPLWKRL GDCLSIIEEK PEMEHIATVL LPLIESLMVV CKYVGPKESM EDLFVTFTDA
HRKVLNLMVR NNPSLMSGSF SLLVHNPRVL DFDNKRNYFN QQLHRRREHH STLQLNVRRQ
RVFEDSFQYL QRKTGEQIKY GKLSVRFYDE EGVDAGGVTR EWFQILARQM FDPNYALFQP
CAADKLTYQP NRASWVNPEH LSFFKFVGRV IGKAIYDGRL LDAYFARSLY RQLLGKQVDY
KDVEWVDPEY YNSLCWILEN DPSPLDLTFS FGVTKLVELK EGGASIPVTQ ENRKEFVQLS
ANYRLYSSIK DQIESLLAGF YEIIPKDLVS IFNEQELELL ISGTPDIDVD EWRSATEYNG
YTSSDPVIVW FWRALKSFNR EERAKVLSFA TGTSRVPLGG FVELQGVQGT QRFSIHKAYG
DTDRLPQAHT CFNQIDLPQY SSYEMLRQQL LLAINEGGEG FGFA
>TV
VEFINKLADT PNEDLPEVLS QIDSWKWPRS DLNAWIKVLN KFDEILEEAI RDYDIDNLQV
NVFTPLTKKT ICEILRFERL LLENSTNRKT FNSYDRLNNL MFSSDLDVLI LALNLLLRPA
QQYSSQPAVS HALSISTPRL TSLAKRWPNL RDYDINLVDL VSKGKAQIEA LPTEAREVNF
VFYRHIDSTT IQSKPAMDIL ADAIEAHNVP DEKFELLMRI RGAQVMTNER EKLVIVRLLA
TAIFGHTHSD SQAQSSLFLY EPDLIAHIAE LLQLDRGVRK TVSDVAQPTS TLPQAFVEAL
FSFITFLATH SSGGNMIVGA GLVPVLIQAI ENRLPNRLYL VSKTMQLLDN VLYGYNNAFQ
LFCNAHGVDI LVDRIQYEVD GKLSVARTAV LKHTMRSMHR MMQSSGTSEG LRGLLDSSLL
KSVKKVLQNR AIFGPSVLGL AINIMSIFVH NEPTCLPVIQ ETGLPEAFYG IVEKGLEPVI
EIIQSVPNAI GALCLNQAGQ DQLAARPNII PSIFYIFTSE EHQRVLQEKE NAVLIGTSLE
ELIRHHPSLK DKVFIALKAT MAKIEELGNS YTPPNEIKHL YRLDNIIISY IDVFGKFLEG
FFQHAEGLDH LARLTALPCL PYDFANSVLV QVIRTMAEAA TTETLAFLLK LVQDSLEECK
DFWESMEERP NERFRKLVTL HVRTSLLSDI YATYSHGRAS ATLLLGSLHR SCIWENIVLK
ATLNAAGLRH LVSQMPLSPF FQSVVRRRSS DVAQKQKIRE AAGILADVLV QHLYYTTMLS
TTTVLLVDEL LAAFARVGGQ ELTHAYGGLK VSLVLLQPLV SSRPLFDSTQ TPMAITTKKD
TDAEYFEPHN FLVRMRLAAL PLIRTIWESS WLTAAPISVS KTVVQVVMEI LNAENEPRSA
AERALVRTRN NVAAATELLL AHPELNAARE PFKEELGRRV LSLVDEHPSI HAFSPKAYDV
QEQPLFVRCH LLALVLPVIP KWLPAQLLVI ECLLVIGEAP RSITLPIAQG TRHAEARNAL
FEFCMRLLAV PTLPKDELIS ALRILVLLTR DSSVAEGFVK RGGVAMLGIQ SYIAIIMRHI
VENPTILQHV MRQQLRQFFS HPRVVDVGTF VSGCNSAALR DPEAFVKVTE DLCQISNPET
LIHYLIGELT RTVKYPCFIM QVITELLFSY DACKIAFLSY SPKKHRTAAI QFLLSDLLTF
GTINARKQIT LCNWAMSVIV ALCVDTTKDV PTELVSIRKF VLEAISRALK DLPTSENTES
RYSRLLALSD LCHRLLTVRF EVPTHIAKVM LEKNFVSTLT NVLAEVDLNY PNIRNVVSSV
LRPLEYLTKV AMKMSRDAAT DLYRNSSLGL FAENVTHPLL LDRTEGRSLE PLLTTQRWGE
EAKMLHGRFE QTRLSKLQNH VIIALLPAAI ERIMVTIHGN PVDITDTGID PTFLEALPDD
MREEVLNQHV RDRRAAQVER PADSQISAEF LEALPPDIRA EIIQQEAVEG VDIDPATFIA
SLDPQLRQVV LMDSDDVFIQ SLPKALPSRD AIQLLDKSAL AALLRLLFYP HKNLLYKVLV
NLCENAKSRT DLFNLLLSIL QDGSGDLASI DKSFAQMSVR PELIVQRCLE ALTYVVSSNE
LSSLFFLTEH ELPFGLRRSK KGKGKEKPPQ THFPIVLLLS LLDRPSILRT PSIVESVVAL
LATVTRPLAG LKKVLLANPP HIPHHVLRLI VNVLTAGECS GRTFAQSLSL IQHLSFIPDA
REVIASELRA RAQEFGQSLH SALAELSVAL ASKFSTASSD QAKLLRVLKT IDYMYSIYES
FRFSSLWQRL GDCLSIIEEK PEMEHIATVL LPLIESLMVV CKYVGPKESM EDLFVSFTDA
HRKVLNLMVR NNPSLMSGSF SLLVHNPRVL DFDNKRNYFN QQLHRRREHH GTLQLNVRRQ
RVFEDSFQYL QRKTGDQIKY GKLSVRFYDE EGVDAGGVTR EWFQILARQM FDPNYALFQP
CAVDKLTYQP NRASWVNPEH LSFFKFVGRV IGKAIYDGRL LDAYFARSIY KQLLGKPVDY
KDVEWVDPEY YNSLVWILEN DPSPLDLTFS FGVTKLVELK EGGATIPVTQ ENKKEFVQLS
ANYRLYSSIK EQIEALLTGF YEIIPKDLGS IFNERELELL ISGTPDIDVD EWRSATEYNG
YTGSDPVIVW WWRALKSFTR EERAKVLSFA TGTSRVPLGG FVDLQGVQGT QRFSIHKAYG
DTDRLPQAHT CFNQIDLPQY SSYEMLRQQV LLAINEGGEG FGFA
>GT

AELINKIHDT PDEDLPEVLG EIDSWKWPRS DLNAWVKVLN KFDGVLEEVI KDYEIDKVQI
NMFTPRTKKT TSEILRFERL LLENSTNRKI FSSYDRLNSL LSTADLDILI LTLNLLLRPA
QQYSAQPSVA QALHISTPRL QSLAKRWPNL REYDVGLVDL VSNGKARVEE LPSEAREVNF
TFYRHLDNET LDAKPAMEIL ADATETYAMP DDKFELLCRI RSAQVLAAGR EKLVIARLLA
IAIFCHTHSE SQASAALFLY ELDIITHIAE LLQLDRGVRK TVNDVANPDS TLPHAFVDAL
LSFVTFIASH AAGGNMVVGA GLIPLLIQII ENKLPQRLPV VSKTMQLVDN VLYGFSNAFQ
LFCNNRGVEV LVDRIEHEID GQLPVARGAV LKHLLRSLHR MMQSSGTAEG LRGLIDSSIL
KSIKKVIENR ILFGPSVLPI AINIMSTFVH NEPTSLAVIQ EAGLPETFYK TIEAGIEPVI
EVIQAIPNAI GALCLNQAGQ DQLSARPTII PGLFSVFTSE RHLKVLQDKE NSVLIGSAID
ELIRHHPSLK PPVFEAIKST LGKIEDLGNA YVVPEDLRPW YGLDNIIVHY IDVVGRFLEG
LFQHTDGMER LLRLTALPCL PYDFANSVLV QVIRTMGETA PNEALVYLAK QVKESLADTK
GFWESLEEDS NRRFRNLVTL HTRITLLSEV YATYAHSRAT AGFLLGALHR ACVWENIIFK
TGLNAKALHH LVKQIPLSPL FQAVVKRRNP DPVQRKHISD AASIVANVML KHLYYTVMLG
LVTILLVDDL LVAFYRAGGR ETLPAYGGLK VALHLLLPLV SSKPLFDSGQ TAFATTRKED
TDPDYFEPHN FLVRVRLAAL PLMRELWEAP WLVSAPLSLS KSVIQIILQI VSGEHEPRSA
AERALMRTHN NVNAATEMLL AHPELDEARA PLKAQMGRQA LRLVDEHPSI KRFSPQAYDM
QEQPLSVRCR LLALVLPPIP KWLASHLLVV EALLVLAQEP TTITLPLRIG PDYAEARSTL
FNFCMRLLAL PNLPRDELLS ALRLLVLLTC DHQMAVQFAK QGGVSLVGMK SHITLLLRHI
VEDSTVLHNI MRQEIKRFLS HPRIVDVGSY LKQCSSLALR DPAVFIKVTA SICQLSQPDP
MVHFLITELH QAVKYPAFLI QCLTELLFSY DTCKSAFLSY SFKKHKAAAL HFVLSDLISF
GTIQSRRRIM LCNWAMSIIV ALCVDTFRDI PADVVAARKI VIEAIGRAIR DLPSSEPVDS
RYGRLLALAD LCNRLLTVRF ESPTHIGKIM LEKNFVATLT NALGEVDLNY PNVRGVVTAL
LRPLEHLTKI AIRMSREETP DLYRNSSLGM YPEPTTHPLL LDRSRTREFE PFLTLQRWAE
QARIFHGKFV SDRLNKLSNH VVIALLPAAI ERVTVMIHGS PVDITDTGID PTFLEALPDD
MREEVLNQHV REQRTSRVER PSDSQISPEF LDALPPDIRA EIIQQENLEP SEMDAASIIA
SLDPHLRQAV LLDQEDGFIQ SLPKPPTPRD AIQLLDKAGI VILARLLFFP HKNLLFQVLV
NLCENAKTRT ELFNLLLNIL QDGSGDLSAV DKSFSQMSFR PDVIAQRCLD ALTFIVNSSD
HFPLFFLTEH ELPVGLRRSK KGKGKEKQAQ TYYPIVLLLG LLDRQSLLKA PTIMESVVSL
LATITRPLSS LPKMLLANPP QIPHAVLRFV VNILTAGECS GRTFQQSLAL IQHLSAIPDA
REVIAQELKS RAQELGQSLY ADLDALAEVL ASKFASASSD QAKLLRVLKT IDYMYSIYES
FRFTPLWRRL GDCLSVIEER PELEHIATVL LPLIESLMVV CKYVGPKESM EDLFISFTDA
HRKVLNLMVR NNPSLMSGSF SLLVNNPRVL DFDNKRNYFN QQLHRRRDAH TTLQLNVRRS
RVFEDSVQHL QRKTGDQIKY GKLSVRFYDE EGVDAGGVTR EWFQILARQM FDPNNALFQP
CAADKLTYQP NSRSWINPEH LIFFKFVGRV IGKAIYDGRL LDAYFARSLY RQILGKPVDY
RDVEWVDPEY YNSLCWILEN DPSPLDLTFS FGTTKVIPLK EGGTSIPVTQ ENKKEFVQLS
AQYRLYLSTK DQIEALLAGF YEIIPKDLIA IFNEQELELL ISGTPDIDVD EWRAATEYHG
YSSSDPVIVW WWRALKSFNR DERAKVLSFA TGTARVPLGG FTELQGVQGT QRFSIHKAYG
DPDRLPQAHT CFNQIDLPQY TSYEMLRQQL LLAINEGGEG FGFA
>PP
TELIEKLLET PDEDLAEVLG QIDSWKWPRS DLNAWIKMLN KFDAILEEVI RDYDIDKLQV
NALTPTTKKT VCEILRFERL LLENSTNRKT FNSYDRLNSL MFTSDLDVLI LALNLLLRPA
QQYSSQPAVL HALSISTPRL TSLAKRWPNL RDYDVNLADL VSKGKAQVEA LPNEAREVNF
IFYRHIDSKI IESREPMDIL ADTIEAYSVP DEKFELLTRI RAARALTSTR EKLVTVRLLA
TAIFGHTHQE SQAQSLLLLY EPDLITHVAE LLQLDRGVRK TVAEVAQPSS TLPQSFVEAL
LSFVTFIATH ASGGNMVVGA GLLPLLIQVT ENRLSNRLYV VSKTMQLLDN VLYGFNNAFQ
LFCNARGVEI LVERIKYEVD GKLPVARTAV LKHTLRSIHR MMQSSGTAEG LRGLLDSSLL
KSVKKIMENR SVFGPSILPI AINIMATFVH NEPTCLPVLQ EAGLSEVFYT LVESGLEPVI
EVIQAVPNAI GALCLNQAGQ DQLTARPNII PGLFSIFTSE RHQRVLQEKE NSVLIGTAIE
ELIRHHPALK QSVFDAIKST MDKIIELGNN YIPPSDIKHW YMLDNVIVSY IDVLGKFLEG
FFQCQEALYR LGRLTALPCL PYDFANSVLV QVIRTMAEAS TLETIAFLVS LVHQSLEDTK
QFWAEMEERS NAQVRQLVIL HIRTSLLSDI YATYSHGRAT TSLLLGALHR ACVWENIVLK
AGLNVKILKH LASQIPLAPF FQAVVRRRNA DPGHKQKIRE TSSIVADVMV KHLYYTVMLG
LTTILLVDEL LVAFNQAKGQ ELLHAYGGLK VALHLLHPLI SSKPLFESAQ TALITTSKKE
TEPDYFEPHN FFVKMRVAVI PLLRDLWEAS WLVTAPLGLI KSIVLAAMEI LNVEGEPRSA

AERALARTRN NINAATELLL AHPELNTSRE GLKVGLGKQA LRLVDEHPSI KAFSPSAYDV
QEQPMGVRCR LLALVLPNIP KWLAAHLLVA EALLVLGNDP RSISLPLTTG RRYPEGRAIL
FDFCLRLLAL PSLPKDELLS SLRLFVLLTE DHQMAQDFLR REGVTLLGIQ SHIAIILRHV
VEDGPTIRHI MHQEIRRFFA HPRAVDVGSY VAGCNALALR DPAAFVETTR EICQLSQPES
LIHFLIGELV KSVKYACFLM QCLTELLFSY DSCKLAFLSY SPKKHRTAAI NFFLSDVMSF
GTINAKRRIS LCNWAMSIIV ALCVDTAKDV APELVSVRKF VLEAVSRALK DLPTSEDPEA
RYSRLLALAD LCHRLLTVRF DMPTHIAKVM LEKNFVATLT NALADVDLNY PHIRGVVSAI
LRPLEYLSKI AIKMSRDDTP DLYRNSSLGL CGEAAIHPLL IDRTDVRGLD PLLTVQRWLE
EVKMLHGRFE QGRLTKLSNH VILALLPAAV ERVTVMIHGS QVDITDTGID PTFLEALPDD
MREEVLNQHV RDQRAARVER PADSHISPEF LDALPPEIRA EIIQQESLEP AEIDPASFIA
SLDPQLRQVV LMDSDDVFIQ SLPKPPTQRD AIQLLDKSSM AILVRLLFFP QKTLLYKVLV
NLCENSKTRT DLFNLLLNIL QDGSGDLASI DKSFAQMSVR PELIVQRCLE ALTYIVSSNE
LSSLFFLTEH ELPAGLRRSK KGKGKEKQPQ THYPVVLLLS LLDRPSILKT PSIVESVVTL
LATVTRPLAS LKKVLLTRPP VVPHTVMRFI VNVLTAGECS GRTFSQSLSL IQHLSFVPDA
RDIIANELRS RAQDFGHALY TALDELAAAL AAKFSSPSSD QAKLLRVLKT IDYMYSIYES
FKFAPLWQRL GDCLSIIEEK PELEHVATVL LPMIESLMVV CKYVGAKETM EDLFVTFTDA
HRKVLNLMVR NNPSLMSGSF ALLVHNARVL DFDNKRNYFS QQLHRRREHH TTLQLNVRRP
RVFEDSFQYL QRKTGEQIKY GKLSVRFYDE EGVDAGGVTR EWFQILARQM FDPNYALFQP
CAADRLTYQP NKASWVNPEH LSFFKFVGRI IGKAIYDGRL LDAYFARSLY RLILGKPVDY
RDVEWVDPEY YNSLCWILDN DPSALELTFN FGVTKIVDLK ENGRSIPVTQ ESKREFVQLS
AQYRLYSSIK DQIEALLAGF YEIIPKDLIS IFNEQELELL ISGTPDIDVD EWRAATEYNG
YTSSDPVIVW FWRALKSFNR EERAKVLSFA TGTSRVPLGG FTELQGVQGV QRFSIHRAYG
DTDRLPQAHT CFNQIDLPQY SSYEMLRQQL LLAINEGGEG FGFA
>FP
AELINKLLDT PEDDLHEVLS QIDTWRWPRS DLNAWIKVLN KFDAVLEDVI RDYDIDKLQT
NVFTPATKKT VCEILRFERL LLENSTNRKT YNSYDRLNSL MFTSDLDVLI LALNLLLRPA
QQYTAQPAVL HALSISTPRL TSLAKRWPNL REYDISLVDL VTKGRPQLEA LQNEAREVNF
TFYRHVDSKT IESKEPMDVL ADTIDAYSVP DEKFELLTRI RAARAVNVSR EKLVIARLLA
TAIFGHTHSE AQAQSSLLLY EPDLVAHVAE LLQLDRGVRK TVADVAQPTS TLPQSFVEAL
FSFVTFIATH ASGGNMVVGA GLIPLLVQVI ENRLPNRLYV VSKAMQLVDN VLYGFNNAFQ
LFCNSRGVDV LVDRIEYEVV GKLSVARAAV LKHTLRSMHR MMQSSGTSEG LRGLLDSSLL
KSVKKIMENR SVFGPTVLPI AINIMSTFVH NEPTCLPVVQ EAGLPEAFYS VVESGLEPVI
EVIQAVPNAI GALCLNQTGQ NQLAERPNII PGLFSIFTSE RHQRVLQDKE NAVLIGTAVE
ELIRHHPSLK QSVFASIKAT MEKIYSLGKN YTPTDDIKQW YILDNTIVSF VDVLCKFLEG
FFQHQEALDR FGKLTALPCL PYDFTSSLFV QVIRTMAEGA TNETVAFLIN IVREALEDTK
ELWETLQEES NDRLRRFITL HIRTSLLSDI YATYAHGRAA TALLLGALHR ACVWENIILK
SKLNAQILKH LASQIPLAPF FQAVVRRRTA DSAHKQKVRD TAAIVAQVMR QHLFYTVMLG
LTTILVVDEL LVAFNVAGGQ ELVHAFGGLK VALHLLHPII TAKPLLEASQ TAMLQSSRKE
ADPDYFEPHN FLVRMRVAII SLLTRLWDAS WLVSAPLGVT KSAVQIVMEV LNTEGEPRSA
AERALTRTRN NVTAATELLL AHPELNDSRD PFKNSMGAHV LKLVDEHPSI KSFSTRAYDV
QEQPMAVRCR LLALVLPTLP KWLAAHLLVT EAILLAGDEP KSITLPLTAG STYPEAKGIS
FDFCMRLLAI PMLPKDELIS ALRLLVLLTR ERRFADDFVR REGLNFLGLQ SHVAIILRHV
VEDMPTLQHV MRQEIKRFFS HPRMVDVSGY VSGCNALALR DPSVFVQITR ELCQLSQPEA
LVHFLISELV RSVKYSCFLM QCLTELLFSY ESCKVAFLSY SPKKYRTAAI QFLLSDMMSF
GTISARKRIL LCNWAMSVIV ALCVDTSKEV PAELVSVRKF VLEAISRALK DLPASEKAET
RYSRLLALAD LCHRLLTVRF EVPTHIAKVM LEKNFVATLT NALAEVDLNY PNIRGVVTSI
LRPLEFLSKI AIKMSREEAP DLYRNSSLGL FPDVAVHPLL VDPSQGRSLD PLLTVQRWLE
EVKMLHGKFE QARLNKLVGH VVLSLLPAAV ERVIVTIHGN PVDITDTGID PTFLEALPDD
MREEVLNQHV RDQRAARVER PADSQISPEF LDALPPELRA EILQQESIEV ADLDPATFIA
SLDPQLRQVV LMDSDDMLIQ SLPKPPAPRD AIQLLEKPAI AVLIRLLFYP HKNLLYKVLV
NLCENSKTRT DLFNLLLNIL QDGSGDLAAI DRSFAQMSFR PELIVQRCLE ALTYIVSFNE
LSTIFFLTDH ELPAGLRRSK KGKGKEKQPQ THYPVVLLLS LLDRPSVLKT PSIVESVVTL
LATVTRPLAS LKKTLLAHPP VIPHTVMRSI VNILTAGECS GRTFSQSLSL IQHLSYVPDA
REVIANELRS RAQDFGHSLL TALDELVVAL ASKFSPASSD QAKLLRVLKT IDYMYSIYES

FRFTPLWKRL GDCLAIIEEK PETEHIATVL LPLIESLMVV CKYVGSKESM EELFVSFTDS
HRKVLNLMVR NNPSLMSGSF SLLVHNPRVL DFDNKRNYFN QQLHRRREHH GTLQLNVRRP
RVFEDSFQYL QRKTGEQIKY GKLSVRFYDE EGVDAGGVTR EWFQILARQM FDPNYALFQP
CAADRLTYQP NKNSWVNPEH LSFFKFVGRI IGKAIYDGRL LDAYFARSLY RQLLAKPVDY
KDVEWVDPEY YNSLCWILDN DPSALELTFS FGVTKIIDLK ENGRSIPVTQ ENKREFVQLS
AQYRLYSSIK DQIEALLGGF YEIIPKDLVS IFNEQEVELL ISGTPDIDVD EWRAATEYNG
YTSSDPVIVW WWRALKSFNR EERAKVLSFA TGTSRVPLGG FTELQGVQGV QRFSIHRAYG
DADRLPQAHT CFNQIDLPQY SSYEMLRQQL LLAINEGGEG FGFA
>WC
VEFVDKLYNT PDDELHQALG QIDTWRWPRS DLNAWTKVLN KFDAILEEVI RDYDIDKLQV
NVLTPVTKKT VCEILRFERL LLENSTNRKI FNSYDRLNSL MFTSDLDVLL LTLNLLLRPA
QQYSAQPAVI HALSLSTPRL TSLAKRWPNL REYDLNLAEL VSKGKAQVEA LPNEAREVHF
TFYKRIDSKT IESKEPMDIL ADTIEAYNVP EEKFELLTRI RTARALVPAR ERLVTIRLLA
TAVYGHTHSE SQAQSSIFLY EPDVIVHIAE LLQLDRGVRK TVADVAQPSS TLPQPFVEAL
LSFVTYIATH ASGGNMVVGA GLVPLLIQVI ENRLPNRLYV VSKTMQLLDN VLYGFNNAFQ
LFCNGRGVDI LVDRIEYEVD GKLSVARAAV LKHTLRSMLR MMQSSGATEG LRGLLDSSLL
KSVKKIMETR SIFGPSVLPI AINIMATFVH NEPTCLAVIQ EAGLPEAFYR VVEAGLEPVI
EVIQAVPNAI GALCLNQAGQ DQLTARPNII PGLFSIFTSE RHQRVLQDKE NAVLIGTAVE
ELIRHHPSLK QSVLDSIKST MDTICQIASE YVPAESDRHW YLLDNVIVSY IDVVGKFLEG
FFQHQGALER LGKLTALPCL PYDFATSVLV QVFRTMSDTS PGETVTFMTN LIHEALADTR
EFWASMQEQS NAHLRKLITL HIRTSLLSDI FSAYSHGRSS VSLLLGALHR ACVWENIVLK
AGLNARIFKH LASQIPLAPF FQSVVRRRST DIVQRQKVRE VASVFADVMG KHLYYTVMLG
LSTILLVDEL LTTFNRVGGQ ELVHAYGGLK VALHLLHPIV SVKPLFESPQ TALMMTSRRD
SDSDFFDPHN FMVRMRVAVV PLLRDIWQAP WLVSAPLGVC KSVVQIAMEI LNVEGEPRSA
AERALVRTRN NVSAATELLL AHPELNAARD PIKDGLGAQA LRIVDEHPSV KAFSPAAYDV
QEQPLAVRCR LLALVLPTIP KWLAAHLLVT EALLVADQDC PTIELPFTIT RHYQEARTIV
FDFCLRLLAV PSLPKDELLS ALRLFVFLTE DHEMAKEFVR RDGVGLVGIQ SHVAIILRHI
AEDRHTLQHV MRQEIRRYLA HPRLVDAGSF VVGCNALALR HPESFIQVTQ DICQLSNPET
LVHFILTELI QSIRYPCFLM QCLTELLFSY ESCKVALLSF SPKRHRTAAL QLLLSDVMSF
GTINARKRIM LCNWAMSVVV ALCVDTSKDV STELVSVRKF VLEAISRQLK DLPSSENPET
RYSRLLALAD LTYRLLTVRF EAPTHIAKVM LEKNFVATLT NALAEVDLNY PNIRGVVTAV
LRPLDFLSKI AIKMSRDEST DLYRNSSLGL FPEAVTHPLL VDRSDARILE PLLTVQRWLE
EVKMLHGRHE QGRLSKLNNH VILALMPAAV ERVTVMIHGS PVDITDTGID PTFLEALPDD
MREEVLNQHV RDQRAARVER PPDSQISAEF LDALPPELRA EILQQESIEP AELDPASFLA
SLDPGLRQVV LMDSDDVFIQ SLPKPSTPRD AIQLLDKPSI TILVRLLFFP HKNLLFKVLV
NLCENSKTRS DLFNLLLNIL QDGSGDLASI DRSFAQMSFR PELIVQRCLE ALTYIVSSNE
LSSLFFLTEH ELPLGLRRSK KGKGKEKHPQ THYPVVLLLS LLDRPSILKT PSIVESVVTL
LATVTRPLAS LKKVLLTKPP VIPHTVMRLI VNILTAGECS GRTFSQSLAL IQHLSFVPEA
RDVIANELRS RAQDFGHGLH AALDELATAL ASKFSPASSD QAKLLRVLKT IDYMFSIYES
FRFAPLWRRL GDCLAIIEER QEMEHIATVL LPLIESLMVV CKYVGTKESM EDLFVTFTDA
HRKVLNLMVR NNPSLMSGSF SLLVHNPRVL DFDNKRNYFN QQLHRRREHH GTLQLNVRRQ
RVFEDSFQYL QRKTGDQIKY GKLSIRFYDE EGVDAGGVTR EWFQILARQM FDPNYALFQP
CAADRLTYQP NKASWVNPEH LSFFKFVGRV IGKAIYDGRL LDAYFARSLY RQLLGKPVDY
KDVEWVDPEY YNSLCWILEN DPSALELTFS FGVTKIVDLK ENGRSTPVTQ ENKREFVQLS
AQYRLYSSIK DQIEALLNGF YEIIPKDLIT IFNEQELELL ISGTPDIDVD EWRAATDYNG
YSSSDPVIVW FWRALKSFNR EERAKVLSFA TGTSRVPLGG FTELQGVQGV QKFSIHRAYG
DTDRLPQAHT CFNQIDLPQY SSYEMLRQQL LLAINEGGEG FGFA
>CC
AEFIKRTLKA SNEELTAILS EVDVWKWPRS DLNAWIKVLN KFDAVLEEII AEYDLDKLQL
KTFSPSTKRL VSEILRFKRL LMENSTNRKT YNSYDRLNSL LFTSDLDILL LTLNLLLRPA
QQYSAQPAVS HALSLSSGRL QSLAKKWPHV REYGAGLVDL VSKGDAEVES LPVEAREVNM
TFYRHIDERE VLAKPAMEVL ADAVKTYKVP DEKFEILCRI RTAAALDKGR EKLLTARLLA
IAIYCHTHAE SRASSTLFVY EPDLIIHIAE LLQVDNGVRK TVNDLPHADS TIPQSFVDAL
LGFLTYIVSH QSGSSMVVGA GLVPLLIQLI ENRSPTRLTT TSKTMQLLDN VLYSAPTGFH

LFTTARGLDT LVERIEYEVD VDIPIARIAV LKHMLRSMHR MMQSAGTAEG LRNLINMSLL
KTVKKIIEYR GLFGPTILPF AINIMTTFVH NEPTSLTIIQ EAGLPEAFYN TIEAGIEPAI
EVIQAIPNAI GALCLNEVGQ AQLAKRPSII PAVFSLFTSE RHLKVLNDKE NAVLIGTAID
ELIRHHPSLK APVFSALKSA LSRIEELGLE YVVPSNLTQW YQLDSIIVSY LDVIGRFLEG
LFQHTDGLNR LGRMTSLPCL PYDFANSVMV QVMRTLAEVA TNDTIQHLSK LVKESLDETA
DFWKTPSHES NAKFRSLVTL HIRITLLSDV FSTYAHGRAA IGLLLGTLHR ASIWENIVFN
AGLNAAAIKH ITHGLPLAPF FQAMVKRRNP DSAQRKQIQE SSKIVADIML KHLYYTVVLG
LFSLLLIEEQ LYAFYRIDGK ELVQAYGGLK VVLHLLQPTI TAKYVVESGQ SHLIVTRKKD
TDPDYFEPHH FLVKLRVAAL PLLTSLWQSG WLLQAPVAVT RAVVRALLEV MKGEGEPIID
AERALIRAGN NVNAATELLI SHPILNQGRD ALGSSLSRHG LTLIDEHQQV KDFSPHAYDV
QEQPMANRCR LLALILENPP KWLAGHLLVI ESLFILSDEP RAISIPIAVG PCRQAAKQVV
FKLCLRLLTL EELQSDELLS VLRLLVLFTR NREMADSLLQ ADALAQMGGS SYIATILRHL
VEDSSTVKNI MQQNIKRYFS HPRVVEAGTY VKNCSSMALR DPELFIEATK SLCQLNQPES
TVHLLITELI SCVKYVFFVM QCLSELLLSY DSCKLAFLSY SPKRFRSGTL HFILNELLTF
GTINLRNKIM LCSWATNMMI ALCADTAKDV SADLASVRKY VLEALGRAIK DLPSDDNLNS
KYGRLLALSD LCNRLLTVRF ETLTHIAKVM LEKNFVATLT ASLSDVDLNY PHVRSLVTAI
LKPLQYLTKV AIKMSHEETP DLYRNSSLGM YPEAIVHPLL LNHRHGREFE PLVTIQRWTE
EAKILHGEFV AERVAKLVNH VILTLLPEAI ERVTVMIHGN PVDITDTGID PDFLEALPDD
MREEVLNQHV RDQRAAQVER PADSQISNEF LDALPPEIRA EILQQEAIEP TEIDPASFIA
SLDPTLRQAV LMEQDDGFLA TLPKPKAQHD AIQLLDKGGV AVLVRLLFFP KKSFLFRIFV
NLCQNSKTRT ELFNFLLSIL QDGTGDLAAV DKSFAQMSVR PDLVAKKCLD ALTYIVTANP
HASLFFLTEH ELPAGLRKSK KGKGKEKQPQ SHYPIVLLLG LLDRKALLRT PAIMDSVVNL
LASVTKPLTE LKKVLLANPP QIPHAVLRLI VNILTVGECP AKTFQQSMNL IQHLSYISDA
RDVIAQELRT KAQEFGRVLI ADLEQLMQTL STKFSPASST QAKLLRVLKT IEHMYSIYES
FNFSGLWRKL GDCLGLVGAK PETEHIATVL LPLIESLMVV CQHVGTKEAM EELFISFTDN
HRKLLNLMVR NNPSLMSGSF SLLVNNPRVL DFDNKRNYFN QQLHKRREHR HTLQLNVRRA
RVFEDSFQHL QRKTGDQIKY GKLNVRFYDE EGVDAGGVTR EWFQILARQM FDPNNALFQP
CAADKQTYQP NKNSWVNPEH LSFFKFVGRV IGKAIFDGRL LDAYFARSLY RQLLGKPVDY
KDVEWVDPEY YKSLCWILEN DPTVLDLTFS FGVNRVIPLK EGGDQIPVTQ ENKREFVQLS
AQYRLYSSIK EQIENLSAGF YEIVPKDLIT IFNEQELELL ISGTPDIDVD EWRAATDYVG
YTSSDPNIVW WWRALKSFNR DERAKVLSFA TGTSRVPLNG FTDLQGVQGV QRFSIHRAYG
ENDRLPQAHT CFNQIDLPQY SSYEMLRQQL LLAINEGGEG FAFS
>LB
AELITKLINT STSELLDVLS QIDSWKWPRS DLNAWIKVLN KFDGILEEII NEYDLDKLQL
NPFTPTSKRL IAEILRFERL LLENSTNRKT FSSYDRLNSL LFTSDLDILV LALNLLLRPS
QQYSAQPSVS HALSISTTRL QSLAKKWPHL REYGIGLVDL SCEGNAELEA LPAEAREVNF
TFYRHIGEAT VLAQPVMNVL ADAIDAYSIP DEKFEVLCRI RTAASLVKDR EKLVIIRLLA
IAIYGHTHPE SQATSNLFLY EPDLIVHIAE LLQIDHGIRK TVGDVANEES RLPHTFIEAL
LSFVTFIASH ASGGNMVIGA GLIPLLIQMI DNKSPLRLQA VSKTMQLVDN VLYSFTNAFH
LFCGARGVDV LVERIEHEVD GELPIARVAV LKHILRSMHR MMQSSGTTEG LRGLIDMSLL
RSVKKIIEYR GLFGSSILPF AINIMATFVH NEPTSLTIIQ EAGLPKTFYE AIEAGIEPSI
EVIQAIPNAI GALCLNESGQ AQLANRPSII PAIFTIFTSQ RHLKVLIEKE NAVLIGTAID
ELIRHHPTLK SAVFEALKST MSNIENLGLS YRIPKDILQW YQLDNMVVSF IDILGRFLEG
LFQHTDGLSH LGRFTALPCL PFDFANSVMV QVMRTMTEVA TSETLLHLAT LVKDSLAETH
GFWENVTEPS NERFRCLVTL HIRITLLSDV FATYAHGRGA IGLLLGSLHR ASIWENIALN
AGLNASALKH ITHGLPLAPF FQAMVKRRNP DAAQKKQMAE SSKIVADIML KHLYYSVILG
LLALLLYDDQ LAAFYRADGK DLLHAYGGLK VALSLLHPII SSRPLLDSGQ TFLLVSRKKD
TDPDYFEPHN FLVRLRTAAL PTVRRLWESS WLVQAPLGVI RAVVRTVLEL ANGENEPRSA
AERALTRTHN NVNAATEYLL SHPSLNEARE PLVASISRQS LLLIDEHTQV KAFSLSPDNA
HEQPLANRCR LLALVLKHPP RWLASHLLVT EALFTLAEEP RTITLPIPVG PKHLEARGVI
FDFCLRLLEV DDLASDELLS ALRLLVLFTR DKDMASQFVN REGLDLLGGS SYIATILRHV
VEDSTTVQHI MKQAIRRYFA QPRVVEIATY VRNCSAMALR DTALFIETTS SLCQLGQPEA
LIHLLINELM ATLKYLCFLM QCLTELLFSY DTCKLAFLSF SPKRFRTVTL HFLLNELITF
GTINARNRIT LCNWAMSVVV ALCVDTSKDV SSDLVSVRKF VLETLSRAIK ELSPSESMEA

RYGRLLALAD LCHRLLTVRF EVPTHLAKVM LEKNFVATLT TALSEVDLNY PNVRNLVTSI
LRPLEHLTKI AIKMSREETP DLYRNSALGM YPESTTHPLL LDQRQGREFD PLLTLQRWAE
EMKILHGDFV TERIGKLINH VTLALLPAAV ERVTVIIHGS AVDITDTGID PTFLEALPDD
MREEVLNQHV RDQRAARVER PPDSQISSEF LEALPPEIRA EIIQQEAIEP ADIDPASFIA
SLDPTLRQAV LMDQDDGFIQ TLPKFSPLHD AIQLLDKAGI AVLVRLLFFP QKTLLFKALV
HLCENAKTRT ELFNLLLNIL QDGTGDLAAV DKSFSQLSVK PDLIAQRCLE ALSYIVSAND
LSSLFFLTEH ELPFGLRKTK KGKGREKQPQ THYPIVLLLG LLDRQSLLRT PAIMEAVVSL
LATVTRPLTS LKKILLANPP QVPHAVLRLI VNILTIGECS GRTFQQSLSL IQHLSYIPDA
RDVIAHELKS KAQEFGQTLD TDLEELAGAL AAKFSPASST QAKLLRVLKT IDYMYTIYES
FRFTPLWRRL GDCLSVIEKK PDTENIATVL LPLIEALMVV CKYVGSKESM EDLFISFTDS
HRKVLNLMVR NNPSLMSGSF SLLVNNPRVL DFDNKRNYFN QQLHRRREHH GTLQLNVRRA
RVFEDSFQYL QRKTGDQIKH GKLSVRFYDE EGVDAGGVTR EWFQILARQM FDPNNALFQP
CAADKLTYQP NKNSWVNPEH LSFFKFVGRV IGKAIYDGRL LDAYFARSLY RQLLGKPVDY
KDVEWVDPEY YKSLCWILEN DPTVLDLNFS FGVNQIIPLK EGGESISVTQ ENKREFVQHS
AQYRLYSSIK DQIESLSTGF YEIIPKDLIT IFNEQELELL ISGTPDIDVD EWRAATEYNG
YTSSDPNIVW WWRALKSFNR DERAKVLSFA TGTSRVPLSG FVDLQGVQGV QRFSIHRAYG
ESDRLPQAHT CFNQIDLPQY SSYEMLRQQL LMAINEGGEG FAFS
>FS
TEIIIKLLKT SSEDLPDVLA QINSWKWPRS DLNAWIKVLN KFDAIFEHVI QKHDIDKLQV
NAFDAVTKKT VCEILRFERL LLENSTNRKL FSSYDRINSL LLTSDLDVLV LSLHLLLRPS
QQYSAQPAVT SALNISTPRL KSLAKRWPNL REYDVSLVDL VSNGRSVVDA LPSEAREVNF
TFYRYIDQPA IESQNEMKIL ADVIEKHSVP DEKFELLCRI RAAAVLANGR EKLVIVRLLA
IAIYGHTHSE SSAMSSLFLY EPDLIPHLAE LLQLDRGVRK TVADIADPDC KLPHSFVEAL
LTFVTYTASH QAGGNMVVGA GLVPLLVQVI GNTIPERLAV VSKTMQLVDN VLFSFANAFQ
LFCNGRGVEA LVDRIEFEVD GELPVARAAV LKHTLRSMHR MMQSSGTAEG LRGLIDSSIL
KSLKKIIENR GLFGPSVVPI AINIMATFVH NEPTSLPVIQ EAGLPEAFYK AIEAGLEPVI
EVIQAIPNAI GALCLNQAGQ DQLAGRPSII PGIFAIFTSE RHLKVLQEKE NAVLIGTAID
ELIRHHPSLK VAVFESIKST MGKIEDLANT QVVGKDIEHW YKLDNIVVSF IDIVGRFLEG
LFQTTDGLER IGRFTALPCI PYDYANSLLV QIMRNMTDVA PNETLAFLQN LVKSSLAETQ
EFWGTMDDHS NRQFRNLITL HIRVALLSDV FSTYAHGRAA ISILLGTLHR ACIWESILLK
AGLNAKALQH LTHGLPLAPF FQAIVKRRNP DPVQRRQIMG SASNIAQVML KHIYFSIMLG
TITVLLVDEL LSEFYRAGGQ ELAHAYGGLK VALRLIHTLI SPKSLQESPQ TLLMASRKKD
TDPGYFEAHN FLVRVRVAFL PLLKEMWEAP WLAPAPLGVV KSVVQAVMEM MAGESEPRSA
AERALIRMHN NVNAATELLL SQPELNDARE PLIGSIPKQA LQLVDAHPTI KSFSPHAHGV
KEEPLSMRCR LLALVLPGVP KWLAAHLLVT EALLAMGEQA RTITLPLSAG PSYPEARNIV
FDYCLRVLAI ADLPRDEFLA VLRLLVILTQ DHRVACQFVQ RDGVALIGSQ SYVAIVLRHV
IEDSSVLQQI MKQEIKHFFA QPRVLDVGNY VRHCSAMALR NPLIFVQLTK SLCKLQSAEI
LVHYLIGELM RVSKYACFIM QCLTELLFSY DSCKVALLSY APKKYRMTVL QFLLSDLVSF
GGINSQHRHL LCNWAMSIVV ALCVDTSKDV SHDLISVRKF VLEAVSRAIK DLSQSGTIDS
RYGRLLALSD LCHRLLTVRF ETPTHIAKVM LEKNFVATLT NALAEVDLNY PNVRSLVAGI
LRPLEHLTKI AIKMSREETP DLYRNSALGM YAEATTHPLL LDKPDGPEFD PQLTLQRWGD
EVKTLHGKFV SERVGKLANH VVLALLPAAI ERVTVMIHGS AVDITDTGID PTFLEALPDE
MREEVLNQHV RDQRAARVER PADSTISDEF LDALPPEIRA EIIQQERLEP TDMDPASFIA
SLDPQLRQTV LLDSDDGFIQ SLPKLPVPRD AIQLMDKTGV AVLVRLLFFP QKNHLFKVLL
NLCENTKTRM ELFNLLLNIL QDGTGDLAAV DKSFAQMSVR PELVAQRCLE ALTYIVGANE
MSSLFFLTEH EIPPGLRKSK KGKGKEKQPQ IHYPVVLLLG LLERQSLLKT PSTMESVVGL
LAIVTRPLTS LKRTLLTHPP QIPHAALRLI VNILTVGECS GRTFQQSLAL IQNLSHIPDA
RDVIAQELKA KAQEFGQSLY VDLDELATAL ASKFSPASSI QAKLLRVLKT IDYMYSIYES
FRFTPLWRRL GDCLAVIEEK PDTEHIATVL LPLIEALMVV CKYVGSKESM EDLFVSFTDA
HRKVLNLMVR NNPSLMSGSF SLLVNNPRVL DFDNKRNYFT QQLHRRREHH NTLQLNVRRA
RVFEDSFQYL QRKTGDQIKH GKLSIRFYDE EGVDAGGVTR EWFQILARQM FDPNNALFQP
CAADRLTYQP NKNSWVNPEH LSFFKFVGRV IGKAIYDGRL LDAYFARSLY RQLLGKQVDY
KDVEWVDPEY YNSLCWILEN DPTALDLTFS FGVSRIAPLK EGGDTLPVTQ ENKREFVQLS
AQYRLYASIK EQIESLLTGF YEIIPKDLVT IFNEQELELL ISGTPDIDVD EWRAATEYNG

YNSSDPVIVW WWRALKSFNR DERAKVLSFA TGTSRVPLSG FVDLQGVQGV QRFSIHRAYG
DSDRLPQAHT CFNQIDLPQY SSYEMLRQQL LLAINEGGEG FGFA
>PI
ADIITKLLTT PNDNLPDVLG QINSWKWPRS DLNAWIKVLN KFDTILEDVI RDYDIDKLQS
KAFTPPTKKT LCEMLRFERL LLENSTNRKM FSSYDRLNSL LFTSDLDVLV LTLHLLLRPS
QQYSAQPAVT SALNISTPRL RSLAKRWPNL REYGVGLVDL ASKGRPEVEA LPSEAREVNY
SFYRHIDQQN LESKEAMSLL ADTVERYSVP EEKFELLCRI RSAAVLTKGR EKLVIIRLLS
IAIFGHTHIE AQAMSSLFLY EPDLIVHIAE LLQLDRGVRK TIADVGRLDC KLPHSFVEAL
LSFVTYIASH SAGGNMVVGA GLVPLLVQII GNKIPERLAV VSKTMQLVDN VLYSFTNAFQ
LFCGAHGVEA LVDRIEHEVD GELPVARAAV LKHTLRSMHR MMQSSGTAEG LRGLIDSSIL
QSLKKIIEHR ALFGPSVVPI AINIMATFVH NEPTSLPIIQ EAGLPEAFYK AIEAGLEPVI
EVIQAIPNAI GALCLNQAGQ DQLSGRPSII PGIFAIFTSE RHLKVLQDKE NAVLIGTAID
ELIRHHPSLK TPVFEAIKST LSKIEDLGNA YVVPEDIQQW YKLDNMVVSF IDIVGRFLEG
LFQTTDGLDR IGRLTALHCI PYDYANSLLV QVMRTMTDVA SNETLLYLQK LVIASLEETK
EFWGSVDDES NRRFRSLITL HIRIALLSDV FATYTHGRAA IGILLGTLHR ACILENILLK
AGLNAKALRH LMHGLPLTPF FQAIVKRRNP DTAQRKQIMS SSIAIADIML KHIYLSVMLG
TVTILLLDEL LAAFYRAGGQ ELVHAYGGLK VALHLIYPLI SSKPLHESPQ TLLIASRKKD
TDPDYFEGHN FLVRLRLAAL PLLKDLWEAT WLVPAPLPVV KSVVQAVMEL TGAENEPRSA
AERALARTHN NVSAATELLL SQPDLDEARR QLKAGMSKKA LRLVDQHPSI KAFSPFAHDV
REEPLAMRCR LLALVLPSVP KWLASHLLVT ESLLTMGEQP RTIILPIAAG PPFLEARAIM
FDFCLRLLAI PDLPRDDLLA VLRLFVLLTK DHNVACQFVQ RDGISLLGSQ SYIALILRHV
IESPSVLQNI MKQEIKRFFA QPRIIDVGSY VRHCNPMALR DPEIFIHVTE SLCQLQHPET
VVHFLVSELM RTSKYSCFLM QCLTELLFSY DSCKIAFLSY SPKKHRTATL QFLLSELVSY
GTLNSRNRHS LCNWAMSVIV ALCVDSSKDV TPDLVSVRKF VLEAVSRATK ELSPSESLDS
RYGRLFALSD LCHRLLIVRF ETPTHIAKVM LEKNFVSTLT NALAEVDLNY PNVRSLVAAI
LRPLEHLTKI AIKMSREETP DLYRNSSLGM YAEATTHPLL LDRFEGREFE PMSTLQRWTE
EVKILHGKFV SERVGKLGNH VILSLLPAAV ERVTVMIHGS PVDITDTGID PTFLEALPDE
MREEVLNQHV RDQRAARVER PPDSQISDEF LNALPPEIRA EIIQQERQEP AEIDPASFIA
SLDPQLRQTV LLDSDDGFIQ TLPKLPPARD AIQLLDKGGV AVLVRLLFFP QKNHLFKVLV
NLCENAKTRT ELFNLLLNIL QDGTGDLAAV DKSFAQMSFR PELVAQRCLE ALTFIVSSNE
LSSLFFLTEH EVPPGLRRSK KGKGKEKQPQ IHYPVVLLLG LLDRQSLLKT PSTMESVVGL
LATVTRPLTS IKKILLTNPP QIPHAVLRLI VNILTVGECS GRTFQQSLAL IQHLSYIPDA
RDVIAQELKS KAQEFGQSLY ADLDELATAL AAKFSPASSI QAKLLRVLKT IDYMYSIYES
FRFTPLWRRL GDCLGVIETK PDTEHIATVL LPLIEALMVV CKYVGSKESM EDLFVSFTDA
HRKVLNLMVR NNPSLMSGSF SLLVNNPRVL DFDNKRNYFT QQLHRRREHH GTLQLNVRRA
RVFEDSFQYL QRKTGDQIKH GKLSIRFYDE EGVDAGGVTR EWFQILARQM FDPNNALFQP
CAADRLTYQP NKHSGVNPEH LSFFKFVGRV IGKAIYDGRL LDAYFSRSLY RQLLGKQVDY
KDVEWVDPEY YNSLCWILEN DPTALDLTFS FGVRRIAALK EGGETLPVTQ DNKREFVQLS
AQYRLFSSIK DQIENLLAGF YEIIPKDLVT IFNEQELELL ISGTPDIDVD EWRSATEYNG
YTSSDPVIVW WWRALKSFDR DERAKVLSFA TGTSRVPLSG FVDLQGVQGV QRFSIHRAYG
ESDRLPQAHT CFNQIDLPQY SSYEMLRQQL LLAINEGGEG FGFA
>AS
AALVQTIAST PQDELAGLLA SYTAWVWPRS DLHGWINVLN NFDGMFEKII TAYAVDKLQT
APFSQPDKHL LLEILRFERM MFENSTNRKL FSSYDRLNAL LASSDLDVVV SVLQLLLRPA
QQYSAQPAVA HVLHIASGRL ESLSRRWTGL RDHGLEVADL VSKKSEEVDE LPASASDVRF
TYYPQISSVA TSNKEPMAIL RETIEAHHVP EEQFELMCKI RTAWALGKSR EQLVIIRMLA
IAVYAHTQSE SHAQAALFLY EPDLVSHLAE ILQLDRDARK VVADAANSES TISSAFIETV
LSFVSYIASH AAGGNMIIGA GLVPILIQIM DNTLPSRLSV VSKTMSLLDS VLYGFSNAFS
ILCNAHGVES LVARIEHEVD PRLSVARTNV LKHLLRSVHR MMQSPGTTEG LRGLIDSSLP
KTIKKIFQNK ALFGPVVLPL AINIMSTFVH NEPTSLAIMQ ESGLPETFYD TIEGGLEPSI
EVLQAIPNAI GALCLNQAGQ DQLAARQNVI PMLFATFTSE KHVKVLHDKE NAMVMGSAID
ELIRHHPNLK TAVFDAVITT LTKIEELGNA YVDPKGGETP FKLDNIIVTF VDVIGRFLEG
LFQHTDGLER LGRLFTLPSM PADFGGTEFL QVIRTIAEVE PTQTLQKLAQ HVKSSLEETR
DFWKDMGATA NIKFRQLITL NTRISMLAEV YSAIAHGRSA SASLLGDVHR AFIWENILLK

SAVNITALKH LAGQIPVTLF LQALIKRRNV DSSHKKHATQ MAKYVADILV KHLYHTVMIG
FASVLLTGDL LITIWQAGAQ SLIHAFGGLK VTLHLLHQMT SSKALFESTQ APLLITRLKD
TDKDYFEPHD LLVKLRASVL PLVARMWTPT WVTGPPLSIT KSIVQSLLEI MRADKEPRSA
AERALVRTGN NVNAATEYLL SHPELQALRA KLQDGLAAHA FKLVDEHPNI KTFSPSAYDV
HEQPLAVRLR LLGVILPPPP KWVGALLLVA ELLLALSEEA PAVTLPLAVG PDYADARTKL
FGFALKLLQS AELIRDDMLA VLRILVLLTR DATFASEFVR RDGLARLGLE SYVAILMRHA
VEDKSILRSI ITQEIKRFFN QSRLVDISSF MRGGISLVLR DPKAFIDAAA ANCELTGTVA
VVHFLTSELM RVIQYACFLM QCLTEMLFSY EPCKTAFLSY SRTKSRPTTL HFLLHEIISY
GDYHARRRML LCNWASSVVV ALSVDVSKDV SADLVAVRKT VVDAVNKAIK ESSTTEPVAA
RYGRLLAMAE LCHRLLSVKV DVAIHTAKIM LEKGFVATLT NALADVDLNY PHVKTLISAV
LRPLEYLTRI AIKMGREETP DLYRNSALGM YPDVVTHPLL MDRHDHADLQ PQPTSTRWAE
EVQISHGKFA PGRMEKLTNH VVLALLPAAR ERVIVRVNGA DIDITDTGID PEFLEALPDD
MREEVLNQHF RERAIAQQQI PADSHISAEF LEALPPDIRA ELVQQESAEP SDIDPASFLA
SLHPHLRQAV LLEQDGTFLQ ILPRPAVQRD AIQLLDKNGI ATLVRLLFFP QKTLLHKVLV
NLCENSKSRI ELLNLLLSIL HEGTGDLLAV DKSFSQLSVR PNLVAQRSLE ALAYIVSNNE
LSSLFFLSEQ EVAAGLRRSR KGKGKERQPA VHYPVVALLN LLDRQLVLKT VSIMDSVASL
LAIVTKPLTT LKSSLLSRPP VIQAGALRLI VNILTGGECS SRTFQHSLTL IQNLACLPES
RDTIASELRS RAQDLGSSIY MDLDELVKQL AVKFSPASSD QAKLLRVLKT IDYMYSVYET
FKFSSLWRRL GDVLSIVQEK SDVEHTATVL LPLIESLMVV CKHVGTKESM EELFVGFTDA
HRKVLNLMVR NNPSLMSGSF SLLVHNPRVL DFDNKRNYFN QQLHRRREHY GTLQLNVRRA
RVFEDSFQYL QRRTGDQIKY GKLSVRFYDE EGVDAGGVTR EWFQILARQM FNPDYCLFQP
CAADKLTYQP NRASAVNPEH LSFFKFVGRV IGKALYDGRL LDAYFARSLY RQLLGKPVDY
RDVEWVDPSY YSSLCWLLEN DPAPLDMTFS FGVTKVVPLK ENGASIPVTI ENRREFVQLA
AEYRLYSSIK DQIESLLSGF YEIIPKDLIS IFNEQEVELL ISGTPDIDVD EWRAATEYNG
YTASDPVIVW WWRALKSFSR DERAKVLSFA TGTSRVPLGG FVELQGVQGV QRFSIHKAYG
GTDRLPQAHT CFNQVDLPQY SSYEMLRTQL LLAINEGGEG FGFA
>EG
AALVANIAAT PQDELADLLA SFPAWVWPRS DLHAWIGVLN LFDGILEGII SSYAVDKLQV
NAFAPPAKNQ LQEILRFERM LLENSTNRKL FSSYDRLNAL LASSDLDVVV SVLQLLLRPA
QQYSSQPAVT QVLNISSGRL ESLSRRWSNL RDYGLDVSDL VSKKREEVDQ LPPVASDLKF
TFYRYLPAVA TSTQDPMLIL AEIIEAHDIP EERFELMCKI RTAWALGKGR EKLVVIRILA
IALYAHTQSE SHAQSALFLY EPDLVTHLAE LLQLDRDVRT VVADIAKADS TTPSSFIEAI
LSFVSYIASH ASGGNMIVGA GLVPLLIQVI DIELPSRLSV VSKTMSLLDS VLYGFTNSFS
IFCNARGVET LVTRIEHEVD SGLSVARSNV LKHLLRSVHR MMQSPGTTEG LRGLIDSSLP
KSLKKIFVHK ALFGPVVLPL ATNIMSTFVH NEPTSLAIMQ EQGLPEAFYD TIDDGLEASI
EVLQAIPNAI GALCLNQAGQ DQLAARPTVI PKLFETFTSE KHIKVLHEKE NAMVMGSAVD
ELIRHHPNLK TTVFASVIAT LAKIEELGNA YVEPTGAETP FKLDNIIVSY VDVIGRFLEG
LFQHTDGLER LGRLFTLPAM PFDFGSTEFL QVIRTIAEVT PTQTLQKLTQ HVKMSLEETK
DIWQSLGAKA NVRFRQLVTL NTRVTMLAEV YSAIAHGRSA STSLLGDLHR SFIWENILLK
SALNIAAIKQ LAGQIPVTSF LQALIKRRNV DSTHKKQASQ IAALLADVLV KHLYHTVMLG
FFSVLLCGEL LVTLWRVGAQ ALVHSFGGLK VALHLLHTMT SSKLLFESTQ TPVLITRVKD
TEPNYFEPHD LLIKLRATTL PLIKRLWTDA WVSGPPLSIT RSIVQNMLEI MRADREPRSA
AERALIRMGN NVSAATEYLL SHPELDAARA EIREYLVQHA FKLVDEHPNV KNFSPSAYDV
HEQPLAVRLR LLGLVLSSPP KWLGALLLVA ELLLGLADEP SAVTLPLSSG PDYSDARTKL
FDFAFSLLQT PELSREDLLA SLRLLVLLTR DSELASEFVK RDGLPRLGLE VYVAILMRHA
VEDSKVLRTV MTQEIRRFFG QTRVVDMTTF MRGASHLVLR DPKAFMDATS AMCVLTGSET
VVHFLVSELM KVSRYACFLM QCLTELLFSY EVCKSAFLSY SRSKSKPTVL HFLLHDMISY
GELTSRRRML LCNWAASVVV ALSVDVSKEI STDLVAVRKN VIDAVNKAIK ESSPTETVAA
RYGRLMAMAE LCYRLLTVKV DVAIHTSKIM LEKGFVATLT NALAEVDLNY PNVKNLISAI
LRPLEYLTRI AIKMGKEETP DLYRNSALGM YADVTTHPLL VDKVEHIELQ PQPTSARWAE
EIQITHGKSV PERVERLSNH VALRLLPAAR QRVVVTINGA EVDITDTGID PEFLEALPDD
MREEVISQHF RERGMAQQPI PADSQISAEF LEALPPEIRA ELLHQESVEP SDIDPASFLA
SLDPHLRQTV LLEQDDVFLQ TLPRPAVQRD AIQLLDRSGI ATLVRLLYFP QKSLLHKVLV
NICENSKSRT ELLNLLLGIL HEGTGELLAV DKSFAQLSVR PNLVAQRSLE ALSFIVSNNE

LASLFFLSEQ DQPAGLRRSR KGKGKEKQST VHYPVVALLG LLDRQLVLKT VTIMDSVASL
LAIVTKPLTG LKSALLSRPP TIPHNVLRLI VNILTGGECS SRTFQHSLAL IQNLACLPDS
RDTIASELRS RAQDLGTSIY MDLDELVKQL AVKFSPASSD QAKLLRVLKT IDYMYSVYET
FKFSSLWRRL GDVLAIVQEN PDVEHTSTVL LPLIESLMVV CKNVGATESM DELFVSFTDA
HRKVLNLMVR NNPSLMSGSF SLLVHNPRVL DFDNKRNYFN QQLHRRREHH GTLQLNVRRA
RVFEDSFQYL QRRTGEQIKY GKLSVRFYDE EGVDAGGVTR EWFQILARQM FNPDYCLFQP
CAADKLTYQP NRASAVNPEH LSFFKFVGRV IGKAIYDGRL LDAYFARSLY RQLLAKPVDY
RDVEWVDPSY YNSLCWLLEN DPAPLDMTFS FGVTKVIPLK ENGASIPVTI ENRREFVQLA
AEYRLYSSIK DQIESLLAGF YEIIPKDLVS IFNEQELELL ISGTPDIDVD EWRAATEYNG
YSASDPVIVW WWRALKSFSR DERAKVLSFA TGTSRVPLGG FVDLQGVQGV QRFSIHKAYG
GTDRLPQAHT CFNQVDLPQY SSYEMLRTQV LLAINEGGEG FGFA
>SP
ATLIAEILAK PTEDLTDFLA SIDSWKWPRS DLNAWIKVLD KFDTVLEDVI RDYDIDSLQV
KDFTPEVKNL VIGILKFERL LLENSTNRKL YQSYDRLNSL LSSSDLDILV ASLLLLLRPS
QQYTGQHGLS QTLQVSSDRL ESLAKSWPSL REHGIEMLSL IDNSDERLER LPQETSEVHF
TFYRHLGSLA QSSRSAQEVY VDAVKHHDVP EHKFDLLCRI RTAHALGSGR QNLTMARLLS
IAIYAHTHTE TQTQSNMYLF EPDLSNRIAE VLQLERGVRK SVAEISNPTT ALPNLFVDAL
LSFVGLIAQY TSGGSLIVGA GLIPQLIQII SITAPERVSF VSKSMSIVDH VLYAYPTAFT
MFCNGRGVDV LTERIKQEID GLLQHGRSGL LKHILRSMHK MMQNSGTAEG MRGLIDSSLL
KSVEKVIIYR GVFGPSIFPI AINVVATFLH NEPTSFSAIQ EAKMPDRVYD AIESGVELSM
EVIQAFTNVI GALMLNQAGQ EQLSSRPSII PSLFTIFTSE RHLKVLQEKD NAVLIGTYFD
ELVRHHPTLK EAVFSSIMST LGKIHALGMV YTPPESEKFW YGLDNPVVDF INAMCQFLNG
LFHQLGGLSR LTDLLELPSL PYNFGGAQLI QLTRIISDVS PRKILDHLSE RVKRTLDITK
PFWETLGENS NYMFRNLVSL HVQVALLSDI YAAFAHNRSA IGLLLGALHR AFIWESILFK
NALNAKALAF LASQIPLSPV LLAIAKRRGV DVTQRKDFIS VAETLATVMH SHLYNTLMLT
FISMMLFDEL LIQFRKQGGQ ELVHATGGFK VALHLLHSLI SYKPMTDVHQ IGNLGAVRSE
SDPDFYEPFD FLVKMRLSIA PIILETWQSS WLVTAPPALS RYVLLAVKDI LAAENERRGA
ALHALERARN NVTLATEYLL SVPELDELRE QIRVSFGTHA LRLADAHPTI EKFSPGALDV
QEIPLSVRCR LLALLLQPLP KWLAPLFLAA ESLLVAAEDI KAISLPLLCG PSYKDVQLVL
LDICIRLLHL PSLPRDEGLA VTSMLVQLTR NRASAKAFVE RGGLVRLSFH SHAIIIFRHL
MEDPATIEAI MRFEIKRYFS SSRAFDVSNF ARSNGDMLVR DPQTFVSVTK SLCQLEHPEA
IIHGILDELT KIGKYACLLM TYLAELLISY DQCKASFLSY PKKRSKSAAL SFILSEFVTY
GNFIRKQRMV MSDCAIQLIV AICVDNSSTT TTDFSNVRKL VLECIGKALR ESSSVESIGD
RYGRLQALGE LCYRLLTVRF EACMQIAKIM LEKSFVAILT SVVSDIDLKF PNVKNLVSGL
LRPLEHLTKV AIKMGRRETP DLYRHSALGM YADSMMHPLL VDPQNIVPFV PLLTLQRWSE
EASATLRKQV QERAGRTSNH ITCALLPEAR ERITVLIHGN PVDITDTGID PTFLEALPDD
MREEVINQHF REQRTVQAEQ AVDSSISADF LDALPPEIRA ELLQQERLEP ADIDPASFIA
SLDPQLRQSV LMESDEGFLQ TLPKPPQIRD AVQLLDKTGV ASIVRLLFFP QRNLLFKVFV
NLCENSKSRT DLFNLLLSIL QDGTGDLAIV DKNFAQLSFR PDLVAQRCLD ALSYIVETND
SSSLFFLTEH ELPAGLRRSR KGKGKEKQPQ SHYPVVLLLG LLDRQTLLKT PSIVDAVAGL
LNTITRPLSS LKKILLANPP QIPHHALRLI VNILTVGDCS GKTFQHTLAL INHLSFLPDA
REVIAQELKS RAQDFGHNIS SDLDRLITAL ASKFSPASSD QAKLLRILKT IDYMYTIYDS
FRFTPLWNKL GDCLAVVENK PDIEHISTIL LPLIESLMVV CKNVGLKESI DNLFVSFTDD
HRKVLNLMVR NNPSLMSGSF SLLVQNSRVL DFDNKRNYFT QQLHRRRDHY GTIQLNIRRS
RMFEDSYHAF LNKSGDQIKY GKLSVRFYQE EGVDAGGVTR EWFQVLARQM FNPNYALFEP
CAADRQTYQP NKASKINPDH LFYFKFVGRV IGKAIYDGRL MDAHFARSLY RQLLGKPVDY
RDVEWVDPEY YNSLCWILEN DPTPLELTFI FGQRRTISLK DNGASIPVTI ENRKEYVQLS
ARYRLYSSIQ EQIEHLLSGF YEIIPKELIS IFNEQEVELL ISGTPDIDVD EWRAATEYTG
YSSADPVIVW WWRALKSFNR DEKAKVLSFA TGTSRVPLGG FVDLQGVQGV QRFSIHRAYG
DPDRLPQAHT CFNQIDLPQY SSYEKLRQQL LLAINEGGEG FGFA
>RM
TALIADILAK DKEELPDFLA SIDSWKWPRS DLNIWIKVLN KFDQLLEDCI RDYEVDKLQT
REFDQHTKQL LCEILKFERF LLENSTNRKM FNSYDRLNGL LFTSDLDVLI ATLFLLLRPS
QQYSSQPAVS HSLHISTTRL ECLARFWPTI RDHGVDMLDF VSKTQEKVEN IPPEASEVNL

TFYRHLGPLS LSSRSAMEIL NDAVKSYQVP EERYELMCRI RMARSLGVGR QKLMIARLLA
IAIYAHTHNE TQAQSSLFLY ETDLLARLSD LLQLDRGVRK TVAEISNPES TLPHMFVDAL
LTFVIYLASH TSGGNMVVSA GLVPLLIQII GITLPQRLAV VSKTMQLVDN VLYGFVNAFQ
MFCNSHGVEA LTERIRHEVQ GLLPVSRASV LKHILRSMLR MMQSSGTAEG MRTLIDSSLL
KSVETIIEYR GVFGPTILPI AINIIATFVH NEPTSLATIQ EAKVPETIYN ALEAGLEPSI
EVIQSIPNAI GAMCLNQAGQ DQLASRPTVI PALFSIFTSE RHLRVLQDKE SAAIIGAAID
ELIRHHPSLK TLVFNAVVSV IGKIETLGNA YVPPKDIEHW YKIENVIVDF IYILGRFLEG
LFQHKDGLNT FTRLLSLPCL PYDLSSSQLV QVIHTMAEVA PTETHNHLVQ QIKQSVRATE
GFWQKSGATS NVTFRKLITL HNCLTLLADV CTSYTHGQSP ISLLLGSVHR NCLFENITLK
NELNAHALRY IAIQIPLVPL FQALGRRRGH DTSMRQQAIS MAETIATVMC GHLYNTFMLR
LVAVLLSDEL LYQFRQRGGQ ELVHVYGGLK VALPLLQALV SYKPTSESPS MVTGSSARSE
THRDYFEPHD FLVKLRLAIA PLIADIWQSD WLVSSPPGVS KHVIQSMFAI LAGENEPRSA
AVRALTRLHN NISAATEYLL SQTELSTLRT EWRSNVGALA LRLLDVHSTI EKFMPAAYDI
NEEPLSLRFR LLALILQPLP KWLASLLLVM ESLLVLSEEP QAISVPLFTG PLYTEVRAHL
LELSLRLLGL PVLQRDDYLA TLRIVVFLTR PEEGARQFKE RNGLSLLACQ SYTAIILRHL
TEDTVTLDHF MRQEVKRVLT SRDGDNTSNY IRTCVPIASR DPRLFVSVTK SLCQLSSPEM
VVHFLLNELL RFGKYACFLM QCLTELLFSY DQCKSAFLSF GKKRSKPVAL HFLLSDLVSV
GAFNTKKRVM LCNWAMSVIV ALCVDSTNNM SVDLSAVRKA VLDAIAKAIK EAPPGETIDS
RYGRLLALAD LCHRLLTVRF ETPLHLAKMM LEKNFVAILT NALSSVDLNY PNVRSLIAAK
LRPLEHLTKV AIKMGREETP DLYRNSSLGM YPDTTTHPLL VERTDSTDFG PLPTIQRLTE
EAKIMHGKHL YDRITRLSNH IILSLLPDAR ERVTVLIHGN PVDITNTGID PTFLEALPDD
MREEVLNQHF REQRSARVEQ PAESQISPEF LEALPPELRA EILQQERLEP VDMDAATFIA
SLDPNLRQVV LLEQDDGFLQ TLPKLAPPRD AIQLLDRSGV ASLVRLLFYP KRNVLHKIML
NLCENTKTRT ELFNLLLSVL HDGTGDVAMV DRSFSQLSFK PDLVAQRCLD ALASIVVNND
VSSLFFLTEH ELPAGLKRSK KGKGKEKQPQ SYYPIVLLLS LLDRQALLKT PSIMDSVAGL
LDAITRPLAN LRKLFLANPP QIPHPSLRLI VNILTVGECS GRTFQHTLAL IQHLSYLPDA
RDVIAQELKS KAQEFGQNLY ADLEALILAL ASKFSPASSE QAKLLRVLKT IDYMYSIYES
FNFAPLWQKL GDCLTVVEQK PNVEHVATIL LPLIESLMVV CKHVGLKESM EDLFVTFTDN
HRKVLNLMVR NNPSLMSGSF SLLVQNPRVL DFDNKRNYFN QQLRRRRDHY GALQLNVRRA
QVFQDSYHIF IHKSGDQIKY GKLSVRFYNE EGVDAGGVTR EWFQILARQM FDVNYALFEP
CAADTQTYQP NRASAVNPDH LSYFKFVGRV IGKAIFDGRL MDAHFARSLY RQLLGKKVDY
RDVEWVDPEY YKSLCWILDN DPTVLDLTFI FGRRDIIPLK ENGTSIPVTL ENRKEYVQLS
AQYRLTDSIK DQIEKLLEGF YEIIPKDLIS IFNEQEVELL IAGTPDIDVD EWRAATEYNG
YSSSDPVIVW WWRALKSFSR DERAKVLSFA TGTSRVPLGG FTDLQGVQGV QRFSIHKAYG
QPDRLPQAHT CFNQIDLPQY TSYEMLRQQL LLAINEGGEG FGFA
>RF
ASLIADILAV SNEDLSQFLS SIDNWKWPRS DLNSWIKVLN RFDTILEEVI RDYDVDRLQT
TIFTPRTKAT VCEILKFERL LLENSTNRKM FNSYDRLNSL LFSSDLDVLV ASLLLLLRPS
QQYSAQPALS HALHISTSRL ASLAGRPPIL REYGVDMLDL VSAGKEKIRN LPQEASEVSL
SFYRHLGPMA QSSRESMDVL ADAIQTYDVP DEKYELLCRI RNAQALGETR EKLVVIRLLS
IAVYAHTHTE AVAQSSLFLY EPDLVTRIAE LLQLDRAVRK TVAEIANPHS TLSHLFVDAL
LSFVIYIASH AAGGNMVVGA GLVPLLIQTI ENKLPERLQV VSKTMQLVDN VLYGFTNAFQ
IFCNGRGVEV LAERIQYEVD GLLPFARTGV IKHTLRSMHR MMQSSGTTEG LRGLIDSSLL
ASVKKIIGHR GLFGPTVLPI AINIMATFVH NEPTSLVVIQ EAGLPEAFYE VIENGLEASI
EVIQSIPNAI GALCLNQAGQ DQLASRPSII PGLLTIFTSE RHLKVMQDKE NSALIGSSID
ELIRHHPSLK AVVFDALKST LSKIEDMGST YTPPSDIEQW YKLDNLIVSF IDILSKFLEG
LFQHTDCLTR LTRMLALPCM PYDFANSVLV QVIRTMTEVA PTETLGHLAK EVKISLEETR
DFWQSLDGKS NNIFRRLLSL HNQTTMLSDV YTSYTHGRGA VGLLLGALHR ACLWENILLK
TGLNARALKH VASQIPLAPL FQSVARRRHP DPTQKKQAAA TCTAVATVML QHLYYTVMLG
LITVLLFDEL LVKFRQIGGQ ELVHLYGGLK VALHLLHSLV SFKTSSDSNQ NTLFTSQVPE
TDPEYYESHD LLVKMRLAAL PFIRDLWRCD WLVSAPPGVS KYVIQSVLDI VGGERDPRTA
AVRALTRSHN NVSVATEYLL AHPELNLARE TLTADLGTLA LRLIDSHPSI RKFSPSAFDV
HEEPLAVRCR LLALILPTIP KWLACHLLVT ESLLVLADEP RSITLPILTG PSYSEARPIL
FDFAFRLLGV LSLPRDELLA TLRLLVQLTR DHALASEFVR RDGVALLGCQ SYIAIIFRHV

VENKSTLESI MRQEVRRWFT QPRVVDVTSF VRTCAPMAAR DPQTFVKVTQ SLCQLLHSES
VVHFMIGELL RVGKYACFLM QCLTELLFSY DTCKHAFLSF STKRHRTHAL TFLLTDLVSF
GAFNAQKRMM LCNWAMSVIV ALCIDCSKDV STDLSSIRKT VLEAVSRSIK DAPSLESDDT
RYGRLLALAE LCHRLLTVRF ETPIQIAKIM LEKNFVATLT GILSDIDLNY PNMRSLVVAI
LRPLEHLTKI AIKMGREETP DLYRNSSLGM YPEPMPHPLL IDRSESHDFS PLPTLQRWTE
EAKISHGKFL NERFGKLCNH ITLALLPDAR ERVTVMIHGN AVDITDTGID PTFLEALPDD
MREEVLNQHF RERRSARVEQ PPESTINPEF LEALPPEIRA EILQQERLEP AEIDPASFIA
SLDPQLRQVV LLDQDDVFLQ SLPKPTSSRD AIQLLDRSSI ATLVRLLFFP QKNILHKVLL
NLCENSKSRT DLFNLLLSIL QDGSGDLVMV DRSFAQMSVR PELIAQRCLD ALSFIVSSNE
LSSLFFLTEH ELPVGLRRSK KGKGKEKQAQ SHYPIVLLLG LLDRQTLLTA PSIMDSVAGL
LASVTRPLMS LKKMLLANPP QIPHPALRLI VNILTVGECS GRTFQQTLAL IQHLTCIPDA
RDVIAQELKA KAQEFGQILY TNLDDLVKEL ISKFSPASSD QAKLLRVLKT IDYMYSIYES
FRFAPLWRRL GDCLAIVEQK PDVEHIATIL LPLIESLMVV CKYVGSKESM EDLFVSFTDA
HRKVLNLMVR NNPSLMSGSF SLLIHNPRVL DFDNKRNYFN QQVHRRREHY GTLQLNVRRA
RVFEDSFQYL QRKTGDQIKY GKLSVRFYDE EGVDAGGVTR EWFQILARQM FNPNYALFEP
CAADKQTYQP NRASAVNSEH LSFFKFVGRV IGKAIYDGRL LDAYFARSLY RQLLGKPVDY
RDVEWVDPEY YKSLCWILEN DPTALDLTFI FGKRDIIPLK EGGASVPVTQ DNKREYVQLS
AQYRLYSSIK EQIESLLGGF YEIVPKELIS IFNEQEVELL ISGTPDIDVD EWRAATEYNG
YSSSDPVIVW WWRALKSFNR DERAKVLSFA TGTARVPLGG FVELQGVQGV QRFSIHRAYG
DSDRLPQAHT CFNQIDLPQY SSYEMLRQQL LLAINEGGEG FGFA
>RI
ASLIADILAV SNEDLSQYLA SIDNWKWPRS DLNSWIKVLN RFDTILEEVI RDYDVDRLQT
TIFTPRTKAT VCEILKFERL LLENSTNRKM FNSYDRLNSL LFSSDLDVLV ASLLLLLRPS
QQYSAQPALS HALHISTSRL ASLAGRPPIL REYGVDMLDL VSAGKEKIRN LPQEASEVSL
SFYRHLGPMA QSSRESMDVL ADAIQTYDVP DEKYELLCRI RNAQALGETR EKLVVIRLLS
IAVYAHTHTE AVAQSSLFLY EPDLVTRIAE LLQLDRAVRK TVAEIASPHS TLSHLFVDAL
LSFVIYIASH AAGGNMVVGA GLVPLLIQII ENKLPERLQV VSKTMQLVDN VLYGFTNAFQ
IFCNGRGVEV LAERIQYEVD GLLPFARTGV IKHTLRSMHR MMQSSGTTEG LRGLIDSSLL
ASVKKIIGHR GLFGPTVLPI AINIMATFVH NEPTSLVVIQ EAGLPEAFYE VIESGLEASI
EVIQSIPNAI GALCLNQAGQ DQLASRPSII PGLLTIFTSE RHLKVMQDKE NSALIGSAID
ELIRHHPSLK AIVFDALKST LSKIEDMGST YTPPSDIEQW YKLDNLVVSF IDILSKFLEG
LFQHTDCLAR LTRMLALPCM PYDFANSVLV QVIRTMTEVA PTETLGHLAK EVKVSLEETR
DFWQSLDGKS NNIFRRLLSL HNQTTMLSDV YTSYTHGRGA VGLLLGALHR ACLWENILLK
TGLNARALKH VASQIPLAPL FQSVARRRHP DPTQKKQAAA TCTAVATVML KHLYYTVMLG
LITVLLFDEL LVKFRQIGGQ ELVHLYGGLK VALHLLHSLV SFKTSSDSNQ NTLFTSQIPE
TDPEYYESHD LLVKMRLAAL PFIRDLWRCD WLVSAPPGVS KYVIQSVLDI VGGEREPRTA
AVRALTRSHN NVSVATEFLL AHPELNLARE TLTADLGTLA LRLIDSHPSI RKFSPSAFDV
HEEPLAVRCR LLALILPTIP KWLACHLLVT ESLLVLADEP RSITLPILAG PSYSEARPIL
FDFAFRLLGV LSLPRDELLA TLRLLVQLTR DHALAGEFVR RDGVALLGCQ SYIAIIFRHV
VENQSTLESI MRQEVRRWFT QPRVVDVTSF VRTCAPMAAR DPQTFVKVTQ SLCQLLHPES
VVHFMVGELL RVGKYACFLM QCLTELLFSY DTCKHAFLSF STKRHKTQAL TFLLTDLVSF
GAFNAQKRMM LCNWAMSVIV ALCIDCSKDV STDLLSIRKT VLEAVSRSIK DAPSLESDDT
RYGRLLALAE LCHRLLTVRF ETPIQIAKVM LEKNFVATLT SILSDIDLNY PNMRSLVVAI
LRPLEHLTKI AIKMGREETP DLYRNSSLGM YPEPMPHPLL IDRSESHDFS PLPTLQRWTE
EAKISHGKFL NERFGKLCNH ITLALLPDAR ERVTVMIHGN AVDITDTGID PTFLEALPDD
MREEVLNQHF RERRSARVEQ PPESTINPEF LEALPPEIRA EILQQERLEP AEIDPASFIA
SLDPQLRQVV LLDQDDVFLQ SLPKPTSSRD AIQLLDRSSI ATLVRLLFFP QKNILHKVLL
NLCENSKSRT DLFNLLLSIL QDGSGDLVMV DRSFAQMSVR PELIAQRCLD ALSFIVSSNE
LSSLFFLTEH ELPAGLRRSK KGKGKEKQAQ SHYPIVLLLG LLDRQTLLTA PSIMDSVAGL
LASVTRPLMT LKKMLLANPP QIPHPALRLI VNILTVGECS GRTFQQTLAL IQHLTCIPDA
RDVIAQELKA KAQEFGQILY TNLDELVKDL ISKFSPASSD QAKLLRVLKT IDYMYSIYES
FRFAPLWRRL GDCLAIVEQK PDVEHIATIL LPLIESLMVV CKYVGSKESM EDLFVSFTDA
HRKVLNLMVR NNPSLMSGSF SLLIHNPRVL DFDNKRNYFN QQVHRRREHY GTLQLNVRRA
RVFEDSFQYL QRKTGDQIKY GKLSVRFYDE EGVDAGGVTR EWFQILARQM FNPNYALFEP

CAADKQTYQP NRASAVNSEH LSFFKFVGRV IGKAIYDGRL LDAYFARSLY RQLLGKPVDY
RDVEWVDPEY YKSLCWILEN DPTALDLTFI FGKRDIIPLK EGGASVPVTQ DNKREYVQLS
AQYRLYSSIK DQIESLLGGF YEVVPKELIS IFNEQEVELL ISGTPDIDVD EWRAATEYNG
YTSSDPVIVW WWRALKSFNR DERAKVLSFA TGTARVPLGG FVELQGVQGV QRFSIHRAYG
DSDRLPQAHT CFNQIDLPQY SSYEMLRQQL LLAINEGGEG FGFA
>PN
SALIQEILAT PVEGLAKLLR SIETWKWPRT DLNAFLKVLN KFDAILEEVI QEYEVDTLQV
KGYAPDKKEL LLEILRFERL LLEHSMNRKV FNSYDRLNSL MFTSDLDVLI ATLLLLLRPS
QQYSSQPALS HSLHISTSRL ESLAKSPPTL REHGVEMLDL VSKGEKSIEK LPMEASEVHF
QFYRHLGPLS QSSRSAMDIF ADTVKSHQVP DEKFELLCRI RFARALGTGR KKLVIARLLA
IAIYVHTHAE NQAQSSLFLY DTDLVSRIGE LLQQDREIRE NFGDISNSSS TVPNLFVDAL
ISFVTFISAH QTGGNVLVSA GLIPLLIQAI SIENVERLSV IPKILQLLDN VLYGYSNAFT
LFCNSRGVEA LVTRIKNEVD GLIQITRSSL LKQLLKSLHR MMQSSGTSEG MRGLIDTSVL
KSVQTIIEYR GIFGPPVLPI AINIVATFVH NEPTSLTAIQ EAKLPETIFK AFETGIEPSF
EVIQSIPNVL GALCLNQAGQ EQLAAHPSII PALFSILTSE THLKVLLEKE NAANMGSSID
ELVRHHPALK NIVFNSIIST LKKIEELGKD YVPSDDVQQF YRLDNIIINY IDAVGRFLEG
LFQHTEGLDC IARLLALPCL PYDYANHIVV QVIRIIVEVC PVDALVHLIK QVKESLRETQ
EFWETPREES NDFFHRIITL HVRVSLLADA YSTYIQGRQT VGPLLGSLHR SCMWENIVLK
NAFNAHALRH IVSQIPLGSF FQSVAKRRAP DEQQKQQAEI MSATLADVLL KHLYHTVMIS
LVTVLMFDEL LVQFKKVGGQ ELVHVYGGLR MVLNLLILLI SHTPNTDMGQ IAQYICRKPE
THPDFYQQND FLVRMRLAVA PVIKELWEAE WLLNAPPAVS KNVIKCIQAI ISGDNEPRES
AIRALARTQN NVSYATDFLL NNPELDIIRQ SLVSNIGPLA LRLADVQPVI QKFSWAAYDV
QEEPLAVRCR LLALILQPLP KWLPALLLAM ESLLVTSEEP RPIPLVLVTG PRYAEARSTL
FELCIRLLLI PSLPRDELLA TLRMLVQLTR DHTMAAQFVH RDGVSLLGFQ SHIAIILRHL
VEDPKVLSSV MTQEVKKLFN QFKTTEVLTY VRNSMAVAAR DYQMFLDVTK ELCSLVRPES
VVHFMIGELI IVGKYACFLM QCLSELLFSY EQCKIAFLAY PKKRPKSVAL SFLLKELITF
GAFHARKRII LCNWAMSVLV ALCVDNSHTH QHEIGSIRKL VLESISKAIK DAPSHESIDA
RYGRTLALVD LCYRLLTVRF EAPMHLAKIM LEKNFVATLT GVLNDIDLNY PNMRSLVNTI
LRPLEYLTKV AIKIGREETP DLYRNSSLGM IPDVTSHPLL VDRGDGREFG PLQTVTRWSE
EAKITHGKHL QDRTQKLCNH IILALLPVAR ERVTVMIHGN SVDITDTGID PTFLEALPDD
MREEVLNQHF REQRSARAEV TVESQISTEF LDALPPEIRA EILREERLEP TEMEAADFIA
SLDPQLRQVV LLDSDDGSHR FPRKTPQARD AIQLLDKSGI AALVKLLFFP QKSVLNKVLL
NLCENSKSRT ELFNLLLSIL QDGTGDLAVV DKSFSQLSVK PDLVAQRCLD ALSYIVETNE
LSSLFFLTEH ELPVGLKKSK KGKGKDRQPQ SHYPIVLLLG LLDRHALIKT PSIMDAVAQL
LDAVTRPLTS LKKILFSNPP QIPHPALRSI VNILTVGECS GRTFQHTLAL IQHLSFLPDA
RDVIAQELRT RAHEFGQSLS LDLDSLISAL VAKFSPASSD QAKLLRVLKT IDYMYSIYET
FRFTSLWKKL SECLTVVESK SNVEHIATIL LPLIESLMVV CKNVGVKQSV DDLFVEFTDE
HRKILNLMVR NNPSLMSGSF SLLVQNPRVL DFDNKRNYFN QQLRKRRESY PSLHVPVRRA
RVFEDSFQVF QNKTGEQIKY GKLSVRFHHE EGVDAGGVTR EWFQILARQM FNPDYALFEP
CAADKQTYQP NRASDVNPEH LSYFKFVGRV IGKAIYDGRL MDAHFARSLY RQLLGKRVDY
RDVEWVDPEY YKSLCWILEN DPTVLDLTFI FGKHEVIPLK ENGTTLPVTM ENRKEYVQLS
AQYRLHTSIA KQIDSLLSGF YEIIPKDLIS IFNEQEVELL ISGTPDIDVD EWRAATEYNG
LTSSDPVIVW WWRALKSFTR DERAKVLSFA TGTSRVPLGG FTELQGVQGV QRFSIHRAYG
EPDRLPQAHT CFNQIDLPEY SSYEKLRQQL LLAINEGGEG FGFA
>TA
AALIADIIGT STEDIPKFLG AIISWRWPRS DLNAWIKVLN KFDTVLEDVI REYDVDGLQV
RPFSPQTKET ICAILNFERL LLENSTNRKM YNSYDRLNSL LSTSDLDVLV ATLMLLLRPS
QQYSSQPALS HSLHISTQRL ESLAKVPPSF REQGIEMLDL VSKWDKSIGE LPQEISEVNF
SFYRHLGPLA QTSRSTMEIL ADAVKSHQVP EEKFELLCRI RNAWALGQDR KKLVISRLLS
IAVYAHTHPE NQAQSSLFVF DSDLVSRIAD LLQLDRGVRK TVTDISRPES ELSNSFVDAL
LSFVILLASQ PMGGNMVVGA GLVPLLIQVI GINQPQRLPI VSKTMQLVDN VLYTFVNAFS
IFCNCRGVDA LTERIRHEVD GLLQLQRAGV LKHILRSMHR MMQSSGTTEG MRGLIDSSLL
KSVDKIIQYR GVFGPTALPT AINIVATFVH NEPTSLTAIQ EAKLPETIYS ALEAGIEPSF
EVIQAIPNVI GALCLNQTGQ DQLASHPSVI PALFSILTSE RHLKVLLDKE NAVSIGSAMD

ELVRHHPSLK TIVFSALVAT LKQIEDLGNN YVPPEEIRQW YSLDNLVVNF IDALGRFLEG
LFQHTEGMDC FGRLLALPCL PYDYANSVIV QVVRTMAEIA PTETLGHLSK QVNESLDETN
KFWQAPQSRS NDFFRRMVTL HIRLTLLSDV YATYTHGRNA VALLLGALHR VCIWENIALK
SSLNALALKH LASQIPLSPF FQAIAKRRNP DESQKQQAAA VSAVLAETMF KHIYHTVMLG
LITVLLFDDL LVHFVRLGGQ ELVHVFGGLK MALHLLYSLI SHKPSADAAQ IALFISRKPE
SHPEYYEPHD FLVKMRLAVA PLIKEIWQSS WLTSAPPAVN KYVIQSVQEI IVGANEPRAS
AIRALQRSHN NVSFATEYLL THPELSTLRE TFTTDLGPIM LRLVDAHPSI EKFSPMAFDV
QEEPLSVRCR LLALVVQPLP KWLAALLLAM EIFLTCAEEP RPVPLVLLTG PPYTEARATL
FDLSLRLLRI PSLPRDELLA ILRVLVVLTR ERDYADKLIG QGGVPLLGIQ SHVAIIFRHL
VEDRILLESV IGQEIKRIMT GSRTIDVLSY VRSSQALAAR DPRIFIDVTQ KCFTLVRPDI
VIHHLLGELL RVGKYACFLM QCLSELLFSY DQCKLAFLSY PKTRSKSTAL NFILSELVSF
GAFNARKRII LCNWAMSVIV ALCVDGGSNG SVDLPSVRKL VLESISKSLK ESPPHETIDA
RYGRTLALAD LCHRLLTVRF ETPMQLAKIM LEKNFVATLT NVLTDIDLNY PNMRSLVAAI
LRPLDFLTKI AIKMGREETP DLYRNSSLGM FTESMAHPLL IDRSETTEFG PQPTSQRWLE
EAKILHGKHL QERMQRLCNH LVLALLTEAR ERITITIHGN EVDITDTGID PTFLEALPDE
MREEVLNQHF REQRAARADQ RADSQISPEF LDALPAEIRA EILQQERVEP AEINAADFIA
SLDPQLRHVV LLDSDDAILQ SLPKATQIRD AIQLLDRTGI AALLRLLFFP QKNTLHKVLL
NLCENSKSRT DVFNLLLGIL QDGSGDVALV DKNFAQLSFR GDVVVQRCLD ALSFIVETNE
LSSLFFLTEH ELPASLKRSK KGKGKEKAPQ SHYPIVLLLG LLDRQTLVKT PSILDSIAAL
LDAVTRPLTS LKKILLLNPP QIPHPALRLI VNILTVGECS SRTFQRTLAL IQHLAYLPDA
REVIAQELKV RAQEFGQNLL VDLDALISAL ASKFSPASSD QAKLLRVLKT IDYMYSIYES
FRFTPLWRKL GDCLSVVESK ANVEHVSTIL LPLIETLMVV CKNVGLKPSV DDLFVSFTND
HRKILNLMVR NNPSLMSGSF SLLVQNSRVL DFDNKRNYFN QQLHKRREHY PTLQINVRRA
RVFEDSYHTL QGRSGDQIKY GKLSVRFYGE EGVDAGGVTR EWFQILARQM FDPNYALFEP
CAADKQTYQP NQASSINPDH LLYFKFVGRI IGKAIYDGRL MDAHFARSLY RQLLGKPVDY
RDVEWVDPEY YKSLCWILEN DPSILDLTFI FGQHKIIPLK ENGASIPVTL ENRKEYVQLS
AQYRLHSSIK DQIESLLSGF YEIIPKELIS IFNEQEVELL ISGTPDIDID EWRAATEYNG
YTSSDPVIVW WWRAMKSFNR DERAKVLSFA TGTSRVPLGG FVELQGVQGV QRFSIHKAYG
DPDRLPQAHT CFNQIDLPQY SSYEKLRQQL LMAINEGGEG FGFA
>FM
AALIADILAT PTNGLSELLA PIDAWKWPRS DLNAWIKVLN KFDAVLEDVI REYDIDGLQV
KPFTRETKEI ICEVLKFERL LLENSTNRKM FNSYDRLNSL LFSSDLDVLV ATLLLLLRPS
QQYSSQPALS HSLHISTSRL ESLAKTSPML REHAIEMIDL VSKGGKRLRD LPQEASEVNF
TFYSHLGPLA QSSRSAIEIF ADAVKSHQVP DERYELLCRV RFAQALGAGR QKLVIARLLA
IAVYAHTHSE TQAQSSLFLY DTDLVNRIAE LLQQDHEVRK TVADISNIES TLPNLFVDAL
VSFITFIASH AGGGNLVVGA GLVPILIQII GITHEQRLPI VSKSMQLVDN VLYGVMNAFT
LFCNSRGVEV LTERIKYEVD GLMHTTRAGV LKHLLRSIHR MMQASGTTEG MRGLIDSSLL
KSVQNIIEYR GVFGPTVLPI AINVVATFVH NEPTSLTAIQ EAKLPETILK AFEAGIEPSF
EVVQSIPNAL GALCLNQAGQ DHLALHPSII PALFSIFTSE AHLKVLLEKE NAVSIGSSID
ELVRHHPSLR QIVFDSLIST LKKIEVMGTN YVPPCDIQQF YSLDNVIINY IDVIGRFLEG
LFQHTEGLAC IARLLALPCL PYDYANAVLV QVIRTMVEVA PADTLTHLAQ QVSESLNETK
GFWQSTNGES NAFFHKLITL HVRITLLSDV YSTYTHGRHA VGLLLGALHR SCIWENIVLK
NGLNAVALKH IASQIPLGPF FQAVAKRRNP DEAQKKQAAS ISATLADVMY KHLYHTVMIG
LITFLLFDEL LLQFRKVGGQ ELVHVFGGLK MALHLLYLMI SFKPAIDPGQ IAQYISKKPE
NHPDFYEPHD FLVKMRLQIA PLIRDIWQST WLVSAPPAVS KYVIQCVQEI TSGENEPRAA
AVRALSRTNN NVNFATEYLL THPDLDEIRK SLISDIGPQA LRLADAHPSI EKFSPAAYDV
QEEPLAVRCR LLALILQALP KWLPALLLAM ESLLVTAEEP RAVPMVLVVG PPYVEARSML
FELCIRLLHI PSLPRDELLA TLRMLVQLTR DRNMADQFVR RNGVSLLGFQ SHIAIILRHL
VEDRLVLETV MKQEIKRWLS TSKTVEVLTY VRNSTSMAAR DPQVFLDITK ELCTLVRPES
VVHFMLGELM RVGKYACFLM QSLSELLFSY EQCKFAFLTY PKKRSKSSAL SFLLTELLSF
GPFNSKKRVI LCNWAMSVIV ALCVDSSQEN APDVTSIRKL VLESISRSFK DILATEPMDV
RYGRILAMAD LCHRLLSVRF ETPMHLAKIM LEKNFVSTLT TVLAEVDLNY PNMRSLVAAI
LRPLEYLTKV AIKMGREETP DLYRNSSLGM YAEAMSHPLL VDRTDTPEFG PLQTIQRWSE
EAKITHGKHL QERVQRLCNH IVLALLPDAR ERVTITINGN EVDITDTGID PTFLEALPDD

MREEVLNQHL REQRPTQVAP PVESQISADF LDALPPEIRA EILREERLEP TDMGAADFIA
SLDPQLRQVV LLDSDDGILQ TLPKVPTPRD AIQLLERPGI AALVRLLFFP QKSTLHKVLL
NLCENSKSRT ELFNVLLSIL QDGTGDLALV DKSFSQLTFR PDLIAQRCLD ALNYIVGTNE
LSSLFFLTEH ELSAGLKRSK KGKGKEKQAQ SHYPIVLLLG LLDRQTLLKT PSIMDSVAGL
LDSVTRPLTS LKKILLANPP QIPHPALRSI VNILTVGECS GRTFQHTLAL IQHLSFLPDA
RDIIAQELRL KANDFGANLS KDLDELITAL VAKFSPASSD QAKFLRVLKT IDYMYSIYET
FRFTLLWEKL SECLSVVESR DNVEHIATIL LPLIESLMVV CKHVGVKQSV DDLFVSFTDD
HRKILNMMVR NNPSLMSGSF SLLVQNPRVL DFDNKRNYFN QQLHKRREHY PSLQVNVRRS
RVFEDSFHAF QHKTGDQIKY GKLSVRFYAE EGVDAGGVTR EWFQILARQM FNPNYALFEP
CAADRQTYQP NRASEINPDH LSYFKFVGRV IGKAIYDGRL MDAHFARSLY RMLLGKRVDY
RDVEWVDPDY YKSLCWILEN DPSMLDLNFI FGRHAVIPLK ENGASIPVTM ENRKEYVQLA
AQYRLHSSIA KQIENLLAGF YEIVPKELIS IFNEQEVELL ISGTPDIDVD EWRAATEYHG
YSSSDPVIVW WWRALKSFNR DERAKVLSFA TGTTRVPLGG FGELQGVQGV QRFSIHRAYG
EPDRLPQAHT CFNQIDLPEY SSYERLRHQL LLAINEGGEG FGFA
>OS
AALIADILAT STDDLSKLLA PIDSWRWPRS DLNAWIKVLN KFDAVLEDAI REYDVDGLQV
SDFSPQRKQS VCEILKFERL LLENSTNRKM FNSYDRLNSL LFTSDLDVLV STLLLLLRPS
QQYSSQPALS HSLHISTSRL ESLAKGAPAL REHGVEMLDL VSKGEKPVES LPQEASEVNF
TFYRHLGPLA QSSRSAMEIF ADAVKSHQVP DDKYELLCRV RTARALGAGR QKIVVARLLA
IAVYAHTHSE TQTHSSLFLY DTDLVNRIAE LLQHDRGVRK TVSDISNAES TLPNLFVDAL
VSFITYIASH ASGGNMVVGA GLVPLLVQVI GITHPQRLPV VSKTMQLVDN VLYGFMNAFT
MFCNGRGVEA LTERIRYEVD GLLQFTRAGV LKHLLRSMHR MMQSSGTTEG MRGLIDSSLL
KSVDKIIEYR GVFGPTVLPI AVNIVATFVH NEPTSLTAIQ EAKLPETIYK ALEAGIEASF
EVVQSIPNAL GALCLNQTGQ DQLAAHPSII PALFSIFTSE KHIRVLTDKE NAASIGSSID
ELVRHHPSLR TIVFSSLIST LRKIEELGNA YVPPSDIRQY YLLDNIIINY IDAIGRFLEG
LFQHTDGLDC FARLLALPCL PYDYANAVLV QVIRTMVEVA PTETLTHLAK QVKESLDETT
EFWQLTGGAS NSIFHKLVTL HIRITLLSDV YATYTHGRQA VGLLLGALHR VCIWENIVLK
EGLNALALKH VASQIPLGPF FQAIAKRRNP DEASKQQAVA ISATLANVMY KHLYYTVMLG
LITVLLFDEL LVQFRKVGGQ EMVHVYGGLK MALHLLYSLI SFKPAADPGQ VTQYISRKPE
THPDYYEAHD FLVKMRLAVS PLIRDIWQSS WLVSAPPAVS KYVIQSVQEI VTGDNEARAP
ASRALTRTNN NVNFATEYLL SHPELDIMRK SFTADIGPLA LRLADAHPSI EKFSPAAYDV
QEEPLAVRCR LLALILQPLP KWLPALLLAM ESLLVSAEEP RAVPLVLLTG PAYSEARTTL
FELCIRLLHI STLPRDELLA TLRMFVELTR DRNMASQLVH RDGVSLLGFQ SHIAIILRHL
VEDRNVIESI MKEEVKRWFS NPRAVEVLTY VRNSTSMAAR DPLVFVDVTK DMCTLLRPEA
VVHTLLSELI RVGKYACFLM QSLSELLFSY EQCKVAFLTY PRKRSKSVAL NFLLSELVSF
GAFNARKRII LCNWAMSVIV ALCVDSSPNH TLDLTSIRKL VLESISRSIK DTPSSEPVDT
RYGRTLALAD LCHRLLTVRF ETPMHLAKIM LEKNFVATLT NVLSDVDLNY PNMRSLVAAI
LRPLEYLTKV AIKMGRQETP DLYRNSSLGM YAEAMTHPLL VDRSDNPEFG PLPTIQRWSE
EAKITHGKHL QDRVQRLCNH IVLALLPDAR ERITVTIHGN TVDITDTGID PTFLEALPDD
MREEVLNQHF RDQRSSRVEQ SVESQISPEF LNALPPEIRA EILREERLEP ADIDAADFIA
SLDPQLRQVV LLDSDDGIIQ TLPKPSQARD AIQLLDRLGI AALVRLLFFP QKTILHKVLL
NLCENSKSRT ELFNVLLSIL QDGTGDLAMV DKSFSQLSFR PDLVAQRCLD ALTFIVATNE
LSSLFFLTEH ELLAGLKKSK KGKGKEKQAQ SHYPIVLLLG LLDRHNLLKT PSIMDSVAGL
LDAVTRPLTS LKKILLANPP QIPHPALRSI VNILTVGDCS GRTFQHTLAL IQHLSFLPDA
RDVVAQELRS KALDFGQNLS ADLDALIAAL VAKFSPASSE QAKLLRVLKT IDYMYSIYET
FRFTPLWRKL GDCLAVVESK ANVENIATIL LPLIESLMVV CKNVGVKESV DDLFVSFTDD
HRKILNLMVR NNPSLMSGSF SLLVQNPRVL DFDNKRNYFN QQLHKRREHF GTLQLNLRRA
RVFEDSYHAF QHKTGDQIKY GRLSVRFYAE EGVDAGGVTR EWFQILARQM FDPNYALFEP
CAADKQTYQP NRASEINPDH LSYFKFVGRV IGKAIYDGRL MDAHFARSLY RQLLGKRVDY
RDVEWVDPEY YKSLCWILEN DPTVLDLTFI FGRHAVIPLK ENGASIPVTM ENRKEYVQLS
AQYRLHSSIE KQIDSLLSGF YEIIPKDLIS IFNEQEVELL ISGTPDIDVD EWRAATEYNG
YTSSDPVIVW WWRTLKSFNR DERAKVLSFA TGTSRVPLGG FGDLQGVQGV QRFSIHRAYG
DPDRLPQAHT CFNQIDLPQY SSYEKLRQQL LLAINEGGEG FGFA

10-Zinc Finger

>AS
KSSASSATRK KHARKLRRLG KKDAVTKRRA LDELAHFPAL ALHAERRVRA LTASTHAVLL
GPWCMLAHDV DRAIAAAARP AWFVARAAID PASVHAELNP EEASPDRNAR LRVGALGALA
WGDAQPAVRR ASWALVGSLL SVAALRSAWV EADAGVRLAM WEPLLVFLTK YPQAWAYAEF
LQFLTLGSPA RGYPTVLVAL STIPPDIFTA LWGAVDGRAL AFLAALVECA LFFASRPKAF
LIARLVPPQP AVLDHMRAEA AGEPFPEGWA VVEPLDTHGK TAYERVLGAL LERALARDNL
WVLAHLALLA THREAVGVAW LLRRLLSGAE VADAEAWIKA ARGMERSAPL AAHAILRTVS
STSLEPPLLA RYRTELAATL AGVPGLRLLR TLNAAAPDVS GDAALVPQQR AVFLAKALEK
WLASDLDEDV ECELAELFVH LAPVLQTVIG KHWEVILDII ENNLENVDLN EDGDLLLLSR
TLRLLAAVIE LAASTKMLRV WASRERAVMG LVLRLSKPRS ICKHLLVNLE FIVIESAAST
ASAKVKTGFT EQIRGLDLIA TYFAPNILGM LGVTKPFKLD CWAVDEFYLA LYDPLSARLL
AAHLYFRALQ CVPSLVRAWF VKSSDRQLHN AVSTFTSSYF SPPLIAHLLA PLRSLIPENK
WRAWVLGAQI VGEAGVLGGL LHFRKNVAGH FEGQVECAIC YCVIKPCRTC KNRFHASCLY

>EG
KSSASSATRK KHARKLRRLG KKDAVTKRRA LEELTHFPSL ALHPARRVRA LTASTHAALL
GPWCMLAHDM DRAVAMAARP TWFASRAAMD PAALHAELNP EEAATDRAAR LRVGALGALG
WGQDQPGVRR AAWSLVGLLV ASAVLRSAWV EPDAVVRAAM WEPLLVFLTK YPTAWAYAEF
LQFLALGSPA RGFPTVLVIL STVPPDIFTA LWGAVDGRAL AFLSAFVECV LFFASRPKAF
VLARLVPPPA SELERMRVEA ARVPYPPSYA VVEPLDSHGK TAYTRVLEAL VERSLARENP
WIIAHVALLA TPREAVGLGK LLKRLLNGAE VNDAEAWIRA ARSLERSTPG ASHAILRTIS
STALEPPLLA RYRTELAATL AGVPGLRLLR ALNAAAPDAS GDAALVPQQR AVFLMKTIER
WIEDAIDEEV ESGMTELLVS LAPVLQSVPG KHWETVLDLI ENGLESTDMG EEDELLSLAR
TLRLLASVIE LAASTKMLRQ WAPREKKIMA LVLRLSAARA ACKELLISLE YLVIESAAST
ASAKVKAGFV EQLRDLDLVE LYFAPNILGL LGVMKSFKLD VWAIEEFFLS LYDPLSPRLL
SAHLFFRALE STPTLIRGWF LKSKDRQLHT AVSAYISSYF SPPLIATALQ PLRALIPENR
WRAWTFGLAQ NGDGGVLEGL RNFRKNVAGH FDGQVECAIC YWCVVGCGPV ASPRRRTQFF

>CP
KSSASSGTRK KVARKLRALG KKDPVTKAKA LDELNRLPVL FTHPARRIRL LAASVHAALL
GTWLLLAHDL DRVVVTQAER SWFTERVLLD PLSVHAALNP EEQDADKSAR LRYAALGALR
WGLGQPQVRV AAWTLVLCLL GPAVLRSAWV ETDPGVRSAI TRPVLIFLKE YPNAWAYTDF
LSYLALGSPI EGYPSVLIVL STIPSSIFAA FWAAIDGRAL AFLGAVLECL VFMVQRPAVF
ICAYLLPPSR SELEGMLASL PAHAADPSIA LLEPADNRGY GPYARVVSAL LRRRIANEET
WVLPHALRLD EVRESRVLYA LLQHLFADAD TSDAEHWLLL ARRLEKTAPQ TALTIVAALT
THAPEPARLD RYRNELAADA LGIKGLLLLR RLVATAPDPE SDVVFLPQQR AVNLMRVCQA
WIAGAGLEGV ESVMTLLFYH LAPIVQNVAG AHWDLVWDVV ENNLENCSFD DDATLTTLGR
SLRLIILIED LVKTNKSLKA WAERRSAILS LVKDLSAPRS LCRELAVSIE HLVVEAAVDT
ASLKVRTGYV QHLRSLGVVG KNFIPHIFEV LNLFKPFKLS IWEVDGYHLD YYDPLSIRLF
AAHLYHRALL TIPVLVRSWI SDCTDKQLLS RVLDFTSSFF SPGIIRAELA LVRQAVSEER
WRAWVLGVQQ TQNGHIVDGL SMFTKNVKLH FAGLVECAIC YSIIKPCRTC KNRFHAACLY

>CC
KSSATAATRK KHAKKLRSFN KKAQVTKIRA LEELNHVPAL LIHPSRRVRF LAASIHLSFL
GTWSMSAHDV DKTVATTSTK SWFIQQTVLD PEGIYTSLHP EESDQDKKAR LRIGALGALG
WGYAQPPVRK AMWSLVHVLL SRAMLRSAWV ESDLSVQSVL WHPLLTFLKD FPSAWAYEEF
LEFLRKGSPI QGYPVVVLIM STIPASMFAA FWEALDSRAL AFLSSVLEST GFLIRRTQVI
LYGRLLPPTE KELEEMLEGL SSTPIDPSMA VVDPLDQNGF SSYARGVFAV LNRVLAKKNL
WALKHFVVLE GILEARVLRV VLDHVLEELE SADAGKWVVY ARKIERSAPE TSLSIIYSVA
QARLETQKLD RYRNEVAASL LGIPGLLALR KLAASAPDPD SDVVFLPQLR AVNVVKACQK
WVEEGVDEEV ENAMLPVFCA LAPILQNDHG KHWEFVFDLV ETVLENSSVE DDDTLVGLSR
ALKMLLVIED LCMTNKGLRA WEPRRTNVLT LVRDLSLPRS ICRELLLSIE YLVIEVGVGN
ASFKVKSMYI EQLRNFDLVG SKLLPNIIAA LRLDKAFKIG LWGVDEFYVQ YFEPISIPVL

AAHLYYRSLL CVPSLVHTWV LDCKDRQLSN AITTITAQYF SPLIIQAELA HVRSPVDENR
WRAWVLAVQQ THNGRIVDGL SLFKKNVTLH FEGQVECAIC YSRYGRSRTS PPLFLLIVFV

>SS
KSSASSATRK KHAKKLRKLA KKDTVTKGRA LEELERFPAL CVHPSRRLRQ LSAALQTLLL
GAWCLSAWDV DRSVSHRASR SWFITTTIMD PSAAYTALVP PEPLEDRNAR LRMSALGALR
WGTGQPIVRR AAWALVNALM ATAVLRSAFV ESDITVRGSM WEALLLFLTK VKNAWAYTEF
LAFLALGSPV QGYPTVLVIL STLPRKIFDA LWSAIDGQAL AFLAAWSECL VWVAGRPNLF
VLGFVLPPTP EEFRALLNAT STNPLDPTLA VIEPLDSTGY SPYARAATGL LDRQLARANL
WALQYVLTLD SVRDSLVLHT VLQHVLPNTS KEEADGWIEL AREIEGSAHQ TSLAVALAVA
EFAPEPPRLD RYRNELASKI SGVSGYRFLR SLNAIAPDPE SDVIFMPQQR AVFLVQALQG
WMGSDIDEGV EVEVTKVLAD IAPILLGLQG AHWEFIMDLV ESNIENCSFK DLDTLVLLSR
TLRLVSVIMD LAKTNKYLRE WGDRSKNVLT LVRDLSIPRS ICREQALSIE HLVIEAGVDT
ASLKVKTAYV EQLRNLDLIG TYFLPNVFDI LGVGGPFKLD AWGIDEYWLP MYDDQSLSLL
AAHVYYRALL TVPSLVRTWW EGLKDRQLST AISTFTSSYF SPVLIASEFS QIKPTVTEKV
WRRWLFAVQQ VHNGRIVDGL TLFKKTVSLH FEGQVECAIC YSILKPCKTC KNRFHAGCLF

>SH
KSSASSGTRK KHARKLRRLS KKDAVTKGKA LEELMHLPSL FLHPSRRIRL LATSLHVSFL
GAWSMASRDV DRQVASYARR SWFVQRAVMD PAGLYLYLNP EESEVDRRAR LRIGGLGVLQ
YGHGQPVVRT ATWNVLAALL SVAVLRSAFV EPDPLVRTAM WKPLLLFLKD HPTAWAYSEF
LQFLELGSPT QGYPTIVIIL STIPSSIFTS FWAAIDGRAL AFLTSLLECL SFIIRRVEAF
IVGYLLPPTV HQLDIKLSSL RSTPAHPILA ITQSLDSIGF SSYARLVSAL LDLYKARSNI
WALKHFIALD TYRTSRVLLE VLKHVLVDAG KADTEQWMML ARRLERGAPQ TSLAIVHAIA
RQGSEPPLLA RYRNELAAGI LGISGLTLLR RLTASAPDPE SDVVFLPQPR AVNFMKTCQK
WITGNIDDDV QSEMTGVFMD MVPLLQNVPG SHWDLIFDII ENNLENASFE DDETLVVLWR
TVRLIQMIQD LVVYNKALKA WQERQMTILG LLRDLSTPRS LCREAALSIE YLVIEVGVDT
TSMKVRSGYT EQIRNLDLVA SNFIPLVLNI LNLWKAFKLD LWSINEHYLS SYDPLSLKLL
TAHLYYRALL TIPSLIRSWL HDCKDRNLSA SVTNYTSQHF SPVIINTELV QLRIRVTESR
WRGWLLGVQQ IQNGHIIDAL SLFKKNVSLH FEGQGECAIC YSIIKPCKTC KNRFHSSCLY

>RM
KSSASSGTRK KHARKKERLG KKDSVTKRKA LEDWFSLPTL FLHTSRHIRQ LSASIHASLL
GSWCMAMHDP DRQAAMYARR SWFVRRSLLD PQRVYLDLNP EESENDRKAR LRIGGLGALK
WGDGQPLVRK SAWGLLSTLL SSAVLRSAWV EPDGQVRSIM WEPLLLFLLH FPESWAYQEF
LQFLQLGSPT QGYPAIILVL SSIPSTILTS FWSALDGRAL AFLSSLLECL ILLIRRPDFF
TFGYLYPPSR EVLDEMLEEL PSNPIDPTLS AIEPLDTSGL CEYARAVDAL LERHLAKENN
WLLRHCMCLE SIRDARILYA TIQRILNGAT TGDAEHWMLL ARKFETKARL ASEAIILAVS
ESGLEPPRLD RLRNELAAES LGVKGLRLLH RLALTVPDPD SDVVFLPQNR AVNFMKACQS
WITSDIEEDV ESEMLPVFLH LLPILQSIPG AHWDLVFDVI ENNLENSSFD DNSSLVTLTR
TLQLLTAVQE LTRTNKALRA WQEPGKYPLQ VCFMVSTPRS MCWEMAFSIA IPVIEAGIDT
DSLRVKMGYG NQLRDLGLIE NNLLPTVLDM LDLYKPFPLE PWAVSEFYID YYDPLSPKLL
SAHLFYRALL SVPSLVSTWW SALKDKQLQG TISTFTTRHF SPVLISAELA HVRNPAEENR
WRGWLLAVQQ VQNGRIVDGL SMFKKNVTHH FENQTECAIC YSIIKRCKTC KNRFHASCLY

>SC
KSSASSGTRK KNARKLRNLN KKAWPTKIKA LEDLRHFPGL LLHSSRRIRM LAVSLHAALL
GAWCMAAHDV DRSVAIAGSR TWLPRRAIFD PDGAFSAEQA SEAVEDMRAR LRVGALGCLR
WGQDQPAVRR AGWTLLQATV LRLILRSSLT EVDNNVQNVL WGPLISFLQE YPQTWPFQLL
LTFLGLGSPV QGYPTVMLML STIPSTVFTA FWSALPSLSS NLLSSVVECL VFCIRRRNLW
LAAHLIPPPS DTLDRRLDAL PPQPIDPSLA VLVDTDAHGY SSYARIVALL PLRALARQQP
WVLRHLIAWD TIRQARIVTA LLDHVFEEVP KEEADGWVAL VRTSQKTAPE TSLAILAAIA
RTAPDAPRLD RYRNEVAANL QGTSALPMVR QLVAMAPNAD SGVPILPGPR AVNVMKACQA
WIAGDAAEDL EALLPLLFTH LAPVLQTVPG AHWGLIFDVM ENNMETLSLE DDAALPALAR

TLRLIIAVRD LATTTKSLRA WQEREKGILA LVRDMSAPRS ACRELLLTVE HLVIESAVDT
ASMKVRMGYI DQLRSLSVVD TYLMPNIIHM LRLDRAFKLD QWAIDEFYVS LYESFALPAF
AAHIYFRALS TIPSLIASWI QDCKDRSLSA TVGTLTATHF SPVIIARELA HVRTAVDDRR
WKSWMLAVQQ VQSGHILDGL ALFKKNVTLH FEGQVECAIC YSIIKPCKTC KNRFHAGCLY

>FS
KSSATSTTRK KHARKLRRLG KKDAVTKRRA LEELKHAPLL LLHPSKRIRL LAATLHAAIL
GAWRMSAHDP DKHVASVAST SSFLCRALLD SNGLWAALNP GEGETDRNAR LRIGALGALA
RGFTQPPVRR AAWAVVATLL SRVALRSAWI ESDVGVWRAL RDPLLVFLRA YPEAWAYASF
LAFLPHPAPG ATYPALVVLL STLPPSLFDA LWAGLGGGFG MLVKAILECL VFVVRGPSVF
GLAFLLQPTR FELDNMLVAL PSEPIDSSLG VLDPLDARGF SAYARVVYAV LDRQLARKNL
WALRHLLAFE GARGCRVLFR VLHHFFANAS KEDADAWFTL ARKVEKTAPR TSLTIIAAIT
QFAPEPSRLD RYRNELAADL FGVPGLHTLR KLAAVAPDPE SDVVFLPQQR TINVMKTCQQ
WITSDIDEEV ESEMTLIFLH FAPLLQNVPG AHWDLVFDVI ENNLESCSLS DNTALVTLAR
TLRLIIAIED LAATNKALRA WHERRISALT LVRNLSTPRS TCRELVLYIE YLVIESGVDT
ASMKVRSDYV NQLRNLDLIA MRFIPSIFTL LNLYKAFKLD PWAVDEYYIE HYEPLSLQLL
ASHLFYRALL TVPSLVRKWL HDCTDRQLSS AVVAYTSLNF SPVIIRTELL EVKSPVLEDR
WRAWVLGVQQ IQNGRIVDGL RHFKKNVALH FEGQVECAIC YSIIKPCRTC KNRFHAACLY

>LB
KSSATSATKK KHAKKLRSFN KKAQPTKQRA LEELQHISAL FIHPSRRIRA LTASLHTSFL
GTWCIAAHDV DRVVSVSALK AWFVQKTALY PSEMYVHLNP EEDEQDRKSR LRVGALGVAR
WGWGQPNVRK AAWALVQTLL GPAILRSAWV EPDLAVQTTM WQPLLTFLKE FPNSWAYREF
LQFLELGSPM QGYPTVIIIL SSIPSSLFKS FWAAIDSRAL AFLASLLECM VFLLKRPSIF
FFAYLLPPSA AELNEMLAAI PADAIDPSMG VIEPLDKRGF SSYARIVDAL LDRIHAKQNL
WALRHFIVLD GVRDTRVLSV VLDRILGDID VEEADMWIQL ARKLERLAPQ TAMTIAAAIA
ATGTEPQKLD RYRNELAASL LGIRGLLTLR KLAATAPDPD SEVVFLPQVR AVNVVKVFQQ
WVASDVGEDV ESAMIHVYIH LTPILQNLAG NHWEFIFDVL ESVLENSQIT DDEALVPLAN
ALRLIIVLQD LTVTNKLLKA WDERSSVLLT MVRDISLPRS TCRELTLSIE YLVIEAAAAA
ASFKIKSGYV EQLRNSNLIV TQFIPTLLGL LRLDKVFKCD SWFVDEFYIE LYETITLRVL
AAHLFYRALL CVPSLIHTWV LDCKDRQLTY CVTTYTSTYF SPVLIRAELA HVRSPVDEDR
WRAWVLAVQQ THNGRITDGL GLFKKNVTLH FEGQVECAIC YSIIKPCKTC KNRFHAGCLY

>HI
QSSASSATRK KHARKLRKFS KKDAVTKTKA LEELQYIPSL FTHPSRRIRL LTAGLHSTLL
GSWCMASRDV DRQVSIYARR SWFVQRAILN PTELYLYLNP EENDSDRRAR LRVGGLGVVQ
WGLAQPVVRT SAWSLLQVLL SVAVLRSAFV EPDTQVRSVM WRPLLTFLKE FPRAWAYHEF
LQFLELGSPL QGYPTVLIIL STIPSSIFTS FWAAIDGRAL EFLSALLECM TFIIRRPSLF
LFAYLLPPSQ ARLYDELKAS RSDPAHPSLA INHPLDIYGF TSYARAVSTL LDRHAAKTSI
WALRHLLVLD SYRDSAVLLA ALQYIVGDAD KGEADLWMGL ARKIEKSAPQ TALAIITAVV
QFGSEPPRLE RYRNELAAGI LGIPGVLLLR RLAATAPDFD SDVVFLPQPR AVNFMKQCQQ
WIASDIDEDV ESEMTAVFYH LVPILQNVSG SHWDFIFDVI ENNLENSSFD DDMTLTILWR
TIRLIQVIED QVTYNKALRA WQRRQIAILS LLRDLSTPRS TCREAALSIE YLVIESGVNT
ASMKVRSGYT EHLRSLDLIA KHFIPLVLDA LGLYQFFKLG IWAVDEFYLE SFDPLSLPLF
AAHLYYRGLV TAPSLFRMWL LDCKDYTLST SVGNYTSQHF SPVIIGHELG HVKSPVTENR
WRGWLLGTQQ IQNGHIADAL SLFKKNVSLH FEGLIECAIC YSIIKPCKTC KNLFHSSCLF

>PI
KSSASAGTRK KHARKLRLFG KKDAVTKRRA LEELQHVPAL FLHPSKRIRL LTSSLHMSLL
GTWCMASHDI DRQVSTHAHR SWFVQRALLD PGGVYLYLNP DENEQDRKAR LRVSAFGAIT
WGWNQPVVRK SAWTTLQALL STAVLRSAWV EPDAAVQGVM WQPLLTFLKE FSSAWAYREF
LQFLELGSPS QGYPTIVIIL STIPSPIFTS FWAAIDGRAL AFLSSLLECT IFLIRRPCVF
IFAYLFPPSP GEVDQMLRAM PSDPIHSSLA VLDPLDNRGY SAYARLVCAL LDRQAARRNF
WALQHFLALD NVMDSRILRR VLQPVLSDVS MDEADQWMIL ARRLEKTAPQ ASMAIVSSIT

```
QFAPEPPRLD RYRNELAAGL LGIPGLLSLR KLSATAPDPA SDVAFLSQPR AVNVVKAFQQ
WITSDVDEEV ESEMTLIFIH LAPLLQNVSG AHWEFMFDII ENNLENCSFA DDITLVALAR
TLRLIVAIQD LALTTKTLRA WEKRHISILT LVRDLSAPRS VCRELVLSIE YLVIELGVDT
ASLRVRSGYI NHIRNLDIIA THFVPSILGL LELYKAFKLD FWAVDEYYLD FYESISLQLL
ASHLFYRALL TVPSLIRAWL LDCTDRQLSS SVVSYTSQYF SPVIIRTELA HVKSPVLEDR
WRAWVFGIQQ IQNGRIVDGL SLFKKNVALH FEGQVECAIC YSIIKPCRTC KNRFHAGCLY

>SL
KSSASSGTRK KNARKLRALG KKDPVTKAKA IEELQRVPVL FTHPARRIRL LAASLHASLL
GTWCMSSYDI DRQVASFGQK SWFIQKALLD PNGIYLYLNP EESEMDRNAR LRVSAFGAFR
WGFGQPLVRQ NAWVLLSLLL STAVLRSAWI DTDVTVRNTM SRPLLLFLKE FPKSWAYNEF
LQFLELGSPT EGYPIVIIIL STIPSSIFSS FWAALDGRAL AFLSSLLESM VFLLRRPSVF
LAAYLFPPSS EELDSMLSDL PSEPAEPSLA LFESLDRKGY SPYVRVVSTL LSRQLSRDNL
WALRHILALD TIRDSRILSS VLQHIFANAT KGDADSWMVL ARKFEKTAPQ TSLSIMSSIT
RFAPEPSRLD RYRNELAANM LGISGLLTLR KLVAVAPDPD SEVVFLPQQR AVNVMKACQE
WITSDVNEEV ESMMTLLFYH LAPILQNVPG SHWELVFDVI ENNLEHCSIS DNTTLVTLGR
SLQLIIIIQD LVTTNKALRA WEERQLSILT LVKNISAARS LCRELALSVE YYVIEAAVDT
ASMKVRSSYF NHMRSLDIIS THFIPNIFDI LDIFKAFKLD VWTTDEYYLD MYEPLSARLL
AAHLYYRALV TVPALIRSWV SDCTDKQLLA RVIAYTSSYF SPVIIKAELA QVRDSVSEDR
WRGWVLGVQQ IQNGRVVDGL TLFTKNVTLH FAGQVECAIC YSIIKPCKTC KNRFHAGCLY

>SN
KSSATSSTRK KHAKKLRRLA KKDNVTKRRA LEDLQHLPIL LLHQSRRLRL LASGLHTSLL
GAWCMAVHDI DRQVAIQARK SWFLRESFFD PEVVYNRLNP EEEEADRHAR LRFGALGSLR
WGIAAVPVRK AAWSLLQSLV GPAVVRSAWI ESDAIVRSGM WEPLVVFLSK FPQSWAYREF
LEFLELGSPL QGYPTIMIVI STLPSRIFTS FWAAIDGRAL AFLSALLECV LFIVAKPDVY
LLGYLMPPAE KDLNSLLNAG SPVVVDSTAS VLDPFDSLGF SEYSRIVMAI LDRQLIKENI
WSVRHVMILN NARDALVLLQ IMQLVLSDVS KASADLWVQL ARSIEAKSSE TSLAITYALT
RAGPEPPRLD RLRNELASTL AGVPGRRLLR RLAASAPNPD GDVVFLPQQR AIFLVQTFQK
WIASDLDIGV ENLMTLMFFY LAPILQNLPG SHWELIFDIM ENNLENASFE DDSTLFALFW
TLRLIISLED LTASNKALKA WVERRSTMLR FVRDLSEPRE KCRELLLNAE SLVVEAGVDT
TSLKVKMAYL DGLREAEVIG NNLLPVIFDL LGVSKPLNLS PWAVDEYFID LYEPLSNQLL
ASHIYYRVLM TVPSLVRSWW LECTDRQLTS SVSSFTSGSF SPVIIAQQLS HIRQPVKEDR
WRAWLFAVQQ VQNGRIFDAL SLFKKNVSLH FEGQVECAIC YSIIKPCKTC KNRFHASCLY

>SP
KSSATSGTRK KHARKLRRLG KKDTVTKRKA LEDLQHLPPL LLHYSRQIRL HAAELLIAYL
GSWCLATQDM DRQVSLTARK SWFVKRSVYD PQGVYLELNP EESEDDRNAR IRTGGLGVIR
WGSDQPVVRK AAWGIVNSLL SSSILRSAWV EPQTSVRYVM WEPLLLFLTQ FPNAWAYREF
LQFLELGSPI QGYPVIVVIL STIPETIFAS FWAALDGRAL AFLNSLLECL VFLVKRPDIF
AFAYLSEPSQ VELDGMLQAL PSDPSDYSLA TVDRLDKTGL SEYARIVNTL LDRKLARDNM
WLLTHFVQLD SVRDSRILHF VVRHLLSGSS ASDADQWMLF ARQLEKKAPL SAVGIVLAIT
ESGLEPPRLD RYRNELASGI LSIPGLLLLH RLSATTPSLE SDVIFLPQPR AVNFMKACQQ
WVTSDIDEDV ECEMLVIFQY LVPILQNMPG SHWDLMFDMI ENNLETSSFS DQSSLTLLTR
TLRLLQDIQV LTQSNKSLKA WKEREKAILG LVRDLSVPRA ICWELALTIE YVVIEAGVDT
ASLRVKMGYA SQLRDMNIIS SKFLPTIFDL LGLYKPVQLE MWTLQEYFVQ LYDEISANLL
AAHLYYRALL HIPSLVASWW GDCKDRQLSM AVANMTARHF SPVLISAELK HVKDPVEEKR
WRGWLLAVQQ IQNGRIVDGL GIFKRNVTNH FENQTECAIC YSIVKRCRTC KNRFHAGCLY

>RF
KSSASSATRK KHARKLRKLA KKDTITKGKA LEDLKHLPSL FLSSSHRIRL LTAALHFSIL
GSWFLATHDI DRQVSHQSRK SWFLQRALLD PFGLYTDLNP DEGEEDRRGR LRCGALGGVK
WGHGQVAVRK STWSVLYSLL SSAVLRSAWV EPEQTVRAGM WEPLLTFLTV MGIRYSYREF
LQFLQLGSPI QGYPTIVVIL STIPHTIFDS FWASLDGRAL AFLGSLLECL VFLVKRPDIF
```

IFGYLFLPSK DELDDMLDCS SSDPVDPSLA IIQPLDASGY STYARILNAL LDRQLVRTNV
WALRHFMVLD SYRESFVLYS VLQHVLSGAT KDDADQWMIL AKSLEKHAPQ TSIAITLAVT
EYAPEPPRLD RYRNELAAGI LGIPGLLLLR KLAATAPDPD SDVVYLPQLR AVNFVKAYQQ
WVTSDIDEAV ESEMTVILTH LAPILQNVAG SHWDLIFDMV ENNLENCSFN DGTTLVMLAR
TLKLLLSIRD LAETNKTLRT WQEREIPILT LVRDLSVPRS LCWESAISIE YLVVEAGVDT
ASLKVKMGYA SQIRDLGIIA TNFLPTIFAS LDLYRAFQLD IWAIEEYYIQ LHEPLSLKLL
AAHLFYRALL SVPSLIASWW NDCKDRTLST AISSMTTRSF SPVLIAAELA HVKDPVDEKR
WRAWLLAVQQ VQNGRIVDGL SIFKKNVTSH FEDQTECAIC YSIIKRCKTC KNRFHASCLY

>RI
KSSASSATRK KHARKLRKLA KKDTITKGKA LEDLKHLPSL FLSSSHRIRL LTAGLHFSIL
GSWFLATHDV DRQVSHQSKK SWFLQRALLD PSSLYTNLNP DESEEDRRGR LRCGALGGMK
WGHGQVAVRK STWAVLYSLL SSAILRSAWV EPDQTVRAGM WEPLLTFLTG NRQAWSYREF
LQFLQLGSPI QGYPTILVIL STIPYTIFDS FWASLDGRAL AFLGSLLECL VFLVKRPDIF
IFVYLFPPSK DELDDMLDYS SSDPVDPSLA VIQPLDASGY STYARILNAL LDRHLVRTNI
WALRHFLVLD SYRESFVLYS VLQHVLSGTT KADADQWMIL ARSLEKHAPQ TSMAITLAVT
EYAPEPPRLD RYRNELAAEI LGIPGLLLLR KLAATAPDPD SDVVYLPQLR AVNFVKAYQQ
WVTSDIDEAV ESEMTVILTH LAPILQNVAG SHWDLIFDMV ENNLENCSFN DGTTLVMLAR
TLKLLLSIRE LAETNKALRT WQEREMPILI LIRDLSVPRS SCWGSALSIE HLVVEAGVDT
ASLKVKMGYA SQIRDLGIIA TNFLPTIFAS LDLYRAFQLD IWAIDEYYIQ LHEPLSLKLL
AAHLFYRALL NIPSLIASWW NDCKDRTLST AISSVTTRSF SPVLIAAELA HVKDPVDEKR
WRGWLLAVQQ VQNGRIVDGL SIFKKNVTSH FEDQTECAIC YSIIKRCKTC KNRFHASCLY

>TA
KSSASSATRK KHARKLRRLS KKDAVTKRKA IEDLRHLPTL LLHASRQIRA TSASLHYSLL
GSWALSAHDV DRQASSSARR VWFAHNTVLD PLALYHELNP EESEDDRKAR LRIGALGIFK
WGANQPAVRR SAWSMLLSLL SSAVLRSAWV EPDPLVRGVM WEPLLVFLTQ KSRAWAFAEF
LRFLQLGSPV QGYPTVVVIL STVPSSILTS FWAALDGRAL AFLSALLECT VFLVKRPDIF
IFAFVLAPSE DSLADMLQEL PSYPLHPAIA VVDPLDSDGY SEYARVVSAL LDRHLARNSI
WLLRHFLVLD TAQTARILYT TMKHVLSGAD KADAEQWMTL GRRLERQAPQ ASLAIVLSIT
ESGLEPPSLD RYRNEQASGI LGIPGLALLR RLAVSAPDPE SDVIFLPQTR AVNFMKTCQA
WLSSDIAEDV ESEMTGVFVH LAPILQNVPG SHWELIFDLI ENNLENISSL ENSNLPVLSR
TLKLLLTIQD LSKTNKSLRT WKEHELAILK LVRDLSVPRQ VCWEMAFSIE YLVVEAGVDN
ASLRVKMGYA SQLRDMGLIS TSFLPNVLGL LNVYKSFSLE YWKVDEYYVQ LYDVLSPKLL
AAHLFYRALL NVPSLIASWW GECKDRQLSN AVATFTTKYF SPVLIAAELG HVKDPVDEKK
WRGWLLAVQQ VQNGRIVDGL SMFKKNVTHH FENQTECAIC YSIIKRCKTC KNRFHAGCLF

>PN
KSSASSATRK KHARKLKRLG KKDSVTKRKA LESLQHLPSL LLNASRRIRL LTAVLQASFL
GSWCMATEDI DRQVAIQAKR AWFIRRALFD PASIYAELNP EESESDRKGR IRTNALGVLR
WGDGQPVVRR TAWDVLKIFL SSAILRSAWV EPDAIVRASM WEPLLTFLKQ FPKSWAYREF
LQFLALGSPT QGYPAIVVII STIPPSIFTS FWAAFDGQAL AFLRALLESL VFLIKRPNLF
VFGFLLPPPK DTIESMLQSF PSNPLDPTLA IVDPLDCGGL SKYSRLIYAL LNRDLMRENI
WVLRHFLVLD KIGDSRVLYE VMRHILKGAT RTDAEEWVTL SRHIEKRAPN TFSAIMLAVN
ESGLEPPKLD RYRNEFAAGM MGVGGLWLLH KLALIAPDPE SETVFLPQNR AVNFMKTCQN
WISSDISEDV ESEMTLIFQY LSPILQNVPG AHWEFIFDVI ENNLENSSFE GDSSLVVLSR
TIKLIIIIQD LSSTNKTLRS WKEREISILT LLRDLSTPRF NCWELAFSVE YLVVEAGVDT
TSLRVKMGYA AQLQGQDLIG SSFLPSIFDL LEVHRPFQLE PWLVEEYYVQ YYDPQSPKLF
SAHLLYRALL NIPSLISSWW GNIKDRQLLG AISSFITKHY SPVLISEELR IIRDPVEEKR
WRGWLLAVQQ TQNGRIVDGL SMFKKNVMHH FENQTECAIC YSIIKRCKTC RNRFHASCLY

>FM
KSSATSATRK KHALKLRRLG KKDSITKRKA LEELQHLPSL LLNVSRRIRL LAAAIHTSLL
GSWCMAAEDV DRMVSIQAKQ AWFIRRALFD PMAVYSDLNP EESDDDRKGR FRVGALGVLR

WGDEQPAARK AGWSVLRILL SSAVLRSAWV EPDSGVQSSM WEPLLKFLTS FPQAWAFREF
LLFLELGSPA QGYPTIVIIL STIPPPILTS FWAALDGRAL AFLSALLECL ILLIKRPDVF
VFGYILPPSQ DQLEALLYDL PSNPVDPILA IVDPLDRSGL CEFARVVNSL LSRDFARQNI
WLLRHFLILD DLQESRAFCT VVQYVLNGAV RKDAEQWMTL AKRLENRSPN TALAIAHAVN
KSGLEPPSLD RYRNELAASA LGVGGVALLH RLNLVAPDPN SDVVFLPQNR AINFMKACQS
WIASDISEDV ESEMTLVFQH LAPILQTVSG AHWDFVFDVV ENNLENCSFV DTSSLVALTR
TLKLILIIQD LCGSNKSLRA WQEREMQILS LVRNISIPRS ICWEMALKIE YLVVEAAVET
ASLRVKIGYA TQLRNLDLIG SNFLPFIFDL LEVYKPFPLE QWSVEEYFVQ FYDTLSPRLL
AAHLFYRALL NVPSLIASWW TGCKDRQLVI AVSNLTTKYY SPVLIAAELQ HVKDPVEENR
WRGWLLAVQQ IQNGRIVDGL SIFKKNVTHH FENQTECAIC YSIIKRCKTC KNRFHAGCLF

>OS
KSSASSATRK KHARKLRRLG KKDSVTKRKA LEDLQHLPSL LLSSSRRIRL LAAGIHASLL
GSWCMATEDV DRQVAVQAKK SWFIKRALFD PVAIYTDLNP EESEGDRKGR IRVGALGVLK
WGDSQPTVRK AAWGVLLSLL SEAVLRSAWL EPDAGVRASM WEPLLTFLTQ NPKAWAYREF
LQFLQLGSPV QGYPTVIIIV STIPPSILIS FWAALDGRAL AFLSSLLECL VLLVKRPDFF
VFGFLLPPSQ EGLDDILRDF PSNPLDSVLA IVDPLDHAGF SEYTRVVNAL LNRPLARSNI
WLLRHFLTLD NVRETRVLYA VMQHVLNGAA SLDAEQWMTL ARRLEKQSPN TALAIILAVT
ESALEPPSLN RYRNELAAGI LGIRGLALLR RLAITAPDPD SDVVFLPQNR AVNFMKVCQT
WVTSDIDENV ESEMTLIFLH LAPILQNVPG AHWEFVFDVI ENNLENCSFA DSSTLPALAR
TLKLILAVQD LAITNKSLRS WHERELPILT LVRDLSVPRS VCWEMAFSIE YLVVEAGVDT
ASLRVKMGYA TQLRDLDMIG SSFLPIVLDL LDVYKPFQLE VWSVEEYFVQ LYDQFSPKLL
AAHLFYRALL NVPSLISSWW GSCKDRQLST AVASLTTKHY SPVLIAAELG HVKDPVEEKK
WRGWLLAVQQ IQNGRIVDGL SMFKKNVTHH FENQTECAIC YSIIKRCRTC KNRFHAGCLY

>PS
KSSATSGTRK KHARKLRNLR KKDAVTKARA LEDLQHLPSL LTSASRRVRL LAAGLHDSLL
GTWCAATHDV DRQVALQASR SWWTTKVVLD PGGAHAYLNP EEKGQDRDAR LRIGGLGALG
WGFEQPGVRR AAWGLLSVLL SPALLRAAWT EPDALVRAAM WEPLLVFLQG FPQAWTWIEF
LQFLALGSPL QGYPVVVLVL STIPPSLFTA FWAALDGGAL AFIGALLECI LFLSRRRDVF
VVGHVLSPSR AELDAMLGRL PATPLHVSLG VVDPLDVDGF SSYARVVSAL LDRQLARTNI
WALRHLLALD NPRTSRIVYP VVHHVLSGSD KPHAEQWVQI LRKMEKQAPE SSLAILFAVA
QSGLEPTRLD RYRNELAAEL TGVPGLKYLR RLHMTIPNPD SDVAFLPQPR AVNLVKSFQK
WVADAVNEEV ESVMTDIFVA LAPILQNVSG SHWDFMFDVV ENNLENCSLA EEETLTLLAR
SLRFVSTILE LVTSNKQLKA WNERQSAVLV LIRNISVPLS TCRELALSIE FLVVEAGVDT
ASLRVKAGYI EQLRDLDIVA SRLLPAIFEL LQVYKAFKLG MWAVENYYVD LYDPISLQVL
AAHVYYRALL VVPSLIRTWV NDCKDRTLSR TITTYTSTHF SPVLLEAELT HIKESVLEDR
WRSWVLGVFA LQNGSIIEGL RLFMKNVAGH FEGQVECAIC YSVIKPCRTC KNKFHAGCLY

>WC
KSSASSATRK KHARKLRRLA KKDGVTKRRA LEELVHLPFL LLHPTRGIRQ LATSLHSSLL
GTWLLATYDV DRQTAARARE TWFLLRALRD PGGLYAALNP EESEGDRRAR LRVGACGAVG
WGHDQPGVRS AAWSLLHALL SRVVLRSAWV ELDVGVRGVL WVPVLIFLRD FPQCWAWREF
MDFLALGAPV QGYPAVLVVL STVPDSILVN FWAALDGRAL AFLEALCECV VLIVRRPEIF
LYAHLLPPTA QEMASMLETL PSCPADASIA VLDPLSSDGF SSYARISYGL LDRHTAKTNA
WTLRHFLALD TVRESRILYS ILQHALDEAT TAEAEQWILL ARKTERTARQ MSLAILYAVT
QYAPEPPILD RYRNELAAGM MGVPGLWLLR RLAASAPDPE SDVIFLPQQR AVNVLKACQQ
WITADLEEEV DSAMTLVFLP LAPILQNVPG GHWDLMFDVL ENNLEDVSLT EPDTLVTLSR
TLRLFMATED LASTNKALRE WQQRRLTCLT LIRDLSKPLS VCRELALQAE HMVIEAAVDT
ASFGVKSQFI SQLRDLALVG THLLPCLFSI LGLYTPFKLG IWEIDQYDLD LYTALCLSLL
SAHIYYRALL TVPTLVRSWL SDCRDRQLAL SVSSYTARNF SPALIHAELV HVRDPVPEEK
WRAWILGLQQ IRSGSIVDGF GFFKRNIISL FEGLTECAIC YSIVKPCKTC KNRFHAGCLY

>GT

KSSASSGTRK KHARKLRSLR KKDAVTKAKA LEELLHMPAL FLHPSRRIRL LAASVHSSLL
GAWCMAGSDA DRLVARHARA SWMLQRTALD PGAVYLSLNP EESEDDRNAR LRVGALGGLK
WGCGQPVVRR AAWALVQALL STVVLRSAWV EPDPTVRTVM WQPLMMFLKE FPQAWAYDEF
LQFLQLGSPL QGYPTVVIVL STLPASFFTS FWAAVDARAL AFLSSLLECT AFLVRRPDLC
LFAYVLPPDS GTMLRMLEGL PTYPEDPSAA VVNNLDSEGY SQYGRVTTAF ADRQAAKEHI
WALQHLLAAD NPREARVLYA ALQTVMSGAT KEEADEWIML ARKLEKKAPL TSIALVSCVT
AFGPEPPRLD RYRNELAAGI FGVPGLLSLL RLAATAPDPE SDVVFLPQPR AINLMKACQQ
WVESDISEDV ESAMTLIFYH LVPILQDVPG GHWDFIFDVI ENNLENGSFN ENSTLVTLAR
TFRLIIAIQD IATTNKSLRA WDERRQAVLT LVRDLSTPQL MSLELAMSIE NLVIEAGVDT
ASFKVKSGYI QHLRDLDLIS TRFLPYIFNA LGLYSTFKLD VWAVDEYHVS LYDPVSAALQ
AAHLYYRGLL IIPSLIRAWL QDCKDRTLST TVTTFTAQHY SPVIIATEFA RVKSPVEENR
WRAWVLGVQQ IQNGSIVDGL SLFKRNVSLH FEGQVECAIC YSIIKPCKTC KNRFHSTCLY

>PC
KSSASSGTRK KHARKLRKLA KKDSVTKRRA LEDFQHVPAL FLHPSRRVRQ LSVNLHVSLL
GSWLLASYDI DRQVSVAARG AWFIQRVLLD PSGIYLQINP EESEADRKAR MRMSGFGALE
WGWNQPGVRR ACWAAIQALL SVAILRSAWA EPDAAVRNFM WQPLLTFLRE YPNAWAYEDF
RQFLELGSPV QAYPAVIIVL STIPPSIFTS FWAAIDGHAL AFLSALLECV VFMVRRPELF
LFSTLVPPPQ EELDAMLEDL PTYASHVSLA VVDPLDAFGY SVYARVVLSL LDRDAAKQSL
WALRHFQALD NTRESRILHS LLRHILPNVS KEEADLFIGL ARTIEKKAPR ASLAIIYAIT
QYAPEPPRLD RLRNELAAGL LGVPGLWLLR RLAATAPDPE SDIVYLPTNR AVNLMKPLQQ
WITSDLDEEV DYQMTSIFIH LAPILQSVPG AHWDLVFDVM ENNLENSSLS EASSLPLLHR
TLQLVIAIED LTSTNKALRA WLERRNQILT LVRDLDVPLS LCRELALTVE HLVLEASVES
ASLKVKVEYV NHLRQEGLIT DYFLPLIFNL LGLYHAFKLD IWSIDEYYLD LTSSLRLPLL
AAHLYYRALR VVPGLVRSWL ADCRDRQLNG TVTAYTSTHF SPAIIRAELA QAKDPVTEDR
WRSWVLGVQQ IRGGSIVDGL AFFKKNVTSH FEGQSECAIC YSMIKPCRTC KNKFHSGCLY

>DS
KSSATSATRK KHARKLRLLA KKDATTKRRA LEDMQHIPAL FLHPSRRIRL QAIALHSSFL
GSWCLAVHDV DRQVSSFARE SWFVQRTLLD PSGVYLYINP EENEQDRKAR LRVGAFGATE
WGFEQPVVRR SAWSLLQTLL SSAILRSAWV EPDPNVRAAM WQPLLVFLKE HPNAWAYTEF
LQFLATGSPA QGYPTVLIIL STIPSSIFVS FWAAVDARVL AFFASVLESL VFVVRRPYAF
LLAFLLPPSR AELDSMLEAL PSTPLDPSLA VVDPLDTQGL AKYARTVHGL LERQTAKESM
WALHHILALD TVRESRVLHM ILRHALSTAT KADSEQWMLV ARKIEKLAPH TSLAIIFSIT
RYAPEPTRLE RFRNELAAGT LGIPGLWLLR NLAASAPDPD SDVVFLPQQR AINLIKACQQ
WITSDLEEDV QSEMTLVFAS LVPILSNVPG GHWDLVFDVV ENNLENSTLD DPSTYVTLSR
TLQLFMIIED FASSNKALKA WDERRSAILT LIRDLSTPLS MCRELALQIE ELVIEAAVDT
ASMKVKSGYI DQLKDLDLVG SRLLPVVFGL LDLYKAFKLE IWDVDEFYLD YYTTISLRLL
TAHLYYRALL LVPSLIRAWL NDCRDRQLHT TVTAYTSRHF SPAIIRTELE RVKDPVTEDR
WRAWVLGVRQ IRSGSIVDGL SFFLKNVTSH FAGIAECAIC YSIVKPCKTC KNRFHAACLY

>TV
KSSASSGTRK KHARRLRLLS KKDATTKRRA LEELQHLPTL FLHPSRRLRL LAIGLHTSIL
GSWCLAAHDV DRQVSSHARE SWFVQRTLLD PGGVYLYLNP EEDELDRKAR LRVGAFGATK
WGFGQPVVRR SAWSLLQTLL SSAVLRSAWV EPDTIVRAAM WQPLLMFLKD YPNAWAYTEF
LQFLATGSPA QGYPTVLVIL STIPPSIFTS FWAAVDARVL AFLSSVSESL TFFVRRPYLF
LYAFLLPPSQ EELLVMLDDL PSTPLDPSLG AVDPLDSAGF NKYARVVNGL LDRQMSKENV
WALRHLFALD NVRTSRILHM ILQHAFRNAT KADAELWMGA VRQTEKLAPH TALAIVFSVT
RYAPEPPRLD RYRNERAAGA LGIPGLWLLR NLAAAAPDPE SDVIFLPQLR AVNLIKACQQ
WITSDLDEEV QSEMTLLFAH LAPILQGVPG AHWDLIFDVV ENNLEDASLD DPSTLVTLSR
TLRLFIIIED LVSTNKALRA WEERRAANLT LIRDLSTPLS VCRELALQIE TLVIEAAVDT
ASLKVKSGYI DQIRDLDLVA AKLLPTLFSL LDLYKAFKLD IWDIDEFYLD YYTSISLRLL
AAHVYYRTLL LLPSLIRTWL TDCRDRQLST AVASYTSKHF SPAIIRTELA RVKDPVTDDR
WRAWELGVQQ IRSGSIVDGL SFFLKNVTSH FEGVAECAIC YSVVKPCKTC KNRFHAACLY

>FP
KSSASSATRK KHARKLRRLA KKDSITKRRA LEDFQHMPAL FLHPSRRIRL LSVGLHSSLL
GSWLLAAHDV DRQVASLARD CWFVHRTLLD PGGVYLYVNP EENEADRRAR LRMGACGSAE
WGWNQPGVRR AAWSLLQTVL SSAILRSAWV EPDPNVRTAM WQPLLTFLRE HPIAWAYREF
LQFLELGSPL QGYPTIIVII STIPPSIFAS FWAAVDGRAL AFFSALLECL VFLVRRAEVF
LFANLLPPSR SSLDDMLERF PSVPGDSSLA VLDPLDASGM SEYARAVGAL LDRHLAKTNG
WALRHLLALD TVRESCILHV VLEHTLGNAS KEDAQQWVQL GRRLERQAPH TSIAIVYSVT
QHAPEPPLLD RYRNELAAGI YGVPGLWLLR RLVATAPDPE SDIVFLPQLR AVNLMKTCQQ
WITSDIDEDV ESEMTLIFLH LAPILQNVPG AHWDFIFDVM ENNLENSSLV EQGSLVVLSR
TLRLFIAIQD LASTNKALRD WHDRENTSLT FVRDISAPLS ACRELALQIE HMVIEAAVES
ASLKVKSAYI EQLLDLGLVS DRLLPNVITT LGLQKPFKLD IWSIEEMYLD SYSGLSVGLL
AAHLYYRALL IVPSLIRSWL SECRDRQLLN RVSTYTATHF SPAIIRTELT EVKDPVPEDR
WRAWMLGIQQ IRSGSITDGL SFFKKNVSSH FEGLAECAIC YSIIKPCRTC KNRFHAGCLY

>PP
KSSASSGTRK KHARKLKRLA KKDSITKRRA LEDLQHVPAL FMHPSRRIRL LAVGLHSSLL
GAWCTAAHDV DRQVSLLARQ SWYTHRTLLD PSGVYAYVNP EENELDRNAR LRVGACGAAE
WGFAQPGVRR AAWSLLQTML SGAVLRSAWV EPDVNVRATM WQPLLTFLKD HPSAWAYREF
LQFLELGVPL QGYPTVLIIL ATIPPTVFTS FWAALDGRAL AFLSSLLECL VLMVRRPDVF
LFSHLLPPSR ADLDDMLIRL PYTPADPSLA VVDPLDQAGF SVYARAVYAL LDRHAAKINV
WALRHLLALD TPRESRIIHV ILQHVLSFVT KPEAEQWLQL ARRLEKQAPH MSLAIIYSIT
QYAPEPLMLD RYRNELAAGM LGVPGLWLLR RLAAVAPDPE SDIIFLPQLR AVNLMKVCQQ
WIASDLDEEV ESEMTLVFFH LAPILQNVQG VHWDLIFDVM ENNLENASLK EASTLVNLSR
TLRLLIAVED LTSTNRSLRA WQERETTCLS LVRDLFTPVS ICRELALQIE HIVIEAAVES
ASLKVKSGYI DQLRELGLVY DNLLPSIFTI LGLYKAFKLD IWAIDEYYLD FCSPLSLRLL
AAHLYYRALL VVPSLIRGWL SDCRDRQLLS AITAYTAAHF SPAIVRSVLA TLRDPVAEER
WRAWVLGVQQ IRSGSIVDGL AFFKKNITSY FEGLAECAIC YSFIKPCKTC KNRFHAGCLY

11-Concatenated alignments 10genes

>Coprinopsis cinerea
MRRTGQDQCIIFTGETASGKSEARRLAIKSLITLSVPPAGKKGSKLAQQIPASEFVLESFGNARTLFNPNASRF
GKYTELQFTRGRLCGVKTLDYYLERGRVAAVPSGERNFHIFYYLVRGVNDDERGHLNLGDGRNFRYLGGQDALK
FDQLKIALKTIGFSKRSVAQCMQLVAAILHLGNLDFTIDKSRNEDAAVVRNLDTLNLVADFLGVDPQGLEGALS
YKTKMVKKELCTVFLDVEGAEANRDELAKTLYSLLFAWVNEYVNQRLAVGDFDAFIGLLDLPGPQNMSRPNSLD
QFIVNYANERVQGFVRNKIFNGNQQEFQFEGLTHLLPSLSTPDNSETLRLLHNYPGGLIHIMDDQARRQPKKTD
GTMVEAFQKRWGNHSSFKTSGVVDGASFTVSHYNGAVTYAVEGFLERNLDSVNPDFVSLLRGGSINPFVKSLFS
SKAIATQAHPRHEETIVAAQQAVKPMRAPSMRRKGTPCVAGEFRAALDTLFATLDETQPWFVFCINPNDSQLPN
QLEGRSVKGQVRSCGMGGVARRNLVYWEVNMTFGEFEERYAGVLEEVGVTPSFKDVVLGKTKVWLSQRAFRALE
DRLRIKEGGERVGRELGMAGASNQRLPLVSNASPFEEYEPPSRIRGDEESYAPSRNMFAGGDGVAAPGETTEVL
KESSARRRWVAFTWLLTWWIPSPFLVWFGRMKRMDVRQAWREKLALNLIIWFICGCTVFVIAVLGPVICPTEHV
YSTTELSDHSNKVYTAIRGEYGGREINDLFPVQVSALCDGIDGNVSPYVMLSSRNTDTNAVYHDFRASTNDPRP
DWYFQQMVTMRYTARVGFRGYTPKEIRNMANRGQSVAIYNGLSLPAVMFTSASSTFLTFKPPKPLHFTFLTIGS
RGDVQPYIALAKGLIADGHGIEYGCVGGDPAELMRICVENGMFTVSFLKETLQKFRGWLDDLLQTSWEACQGTD
ILIESPSAMGGYHIAEALGIPYFRAFTMTWSRTRAYPHAFAVPERKSYVMFDQVFWRATAGQINRWRRHTLGLG
ATSLDRMEPHKIPFLYNFSPTVVPPPLDWPEWIRITGYWFLDDADVGSKKWTPPDSLVEFIDNAHQSGKKVVYS
DPRSMTKCVIDAVVQSGVHAILSKGWSDRLEPEEPLPPQIYPITSAPHDWLFAGVPTIIHPFFGDQFFWADRVE
ALGVGTGVRKLTVSALKDALVSATTDQKQIDRAKLVGEQIRRVHFDHAREILEPLRLACETQNEKLVIASLDCI
SKLVSYSFFAELVDLVAHTIASCHNESTSDTVSVQIVKALLGLVLSQTTIVHHHSLLQSVRTVYNVYITSTSPQ
IQMLAQGSLTQIVDHVFDLFLKDAFLIFRALCKLSLKPIPPENEQDPKAHTYRSKVMSLQLILHVLNQHMALLV
DPASIIYSSSTQDTVTFDKQISPHLLQSLTRNAPSPVPAVFELSVEIFWRALYSMRAQLKTEIGVLLHEIYIPI
LEMKTSTLRQKAVILGMLARLCQEPQALVEIYLNYDCDERAVDNIYERLMNIISKFGTNSLNGGVLAASGSTLG
LSETQIKRQALECLVAVLRSLVAWGTDDPSRFESAKQRKTALLEGIKKFNFKPKGIQFLIENGFIPDNNPKCIA
TFLHETDGLNKTMLGEGDEEHVAIMHAFVDMMDFKDTLFVDSLRHFLQAFRLPGEAQKIDRFLLKFAERYINGN

DAAYVLGYSVIMLNTDAHNPQVKKRMTKTDFIKNNRGINDLPEDLLSSIFDDIVSNEIVMNDEIASVGRDLQKE
AYVLQTSGMSNDQFYSASQSIHIRPMFEVAWMPFLAGLSNPLQETDDLQVVELCLEGFRAFVTTLAKFTFLNNL
GEMKIKNMEAIKALLDVAVHDGNNLKASWREVLKCVSQLEHMQLIGTGRSKRVPNEELASQSRSTHITVAADMV
FSLSHYLSGTAIVDFVRALCDVSWEEIQSSGNSTHPRLFSLQKLVDISYYNMNRIRLEWSNLWEILGEHFNQVC
THPNAVVASFALDALRQLAVRFLEKEELPHFKFQKDFLKPFEYTMIHNRSTDIRDMVLQCLHQMIQAKVHNLRS
GWRTMFGVFSAASKVMEEGIVISAFDIVKNVNATHFPSIVKHGAFADLTVCITDFCKISKDDGMIKYWYPVLFG
FYDIIMNAHDLEVQKLALESLFSALKVYGTTFPAEFWDTVCQELLFPIFAILKNKHDLSRFHSQEDMSVWLQST
MFQALRALIELFTFHFSILERLLDGLLELLSVENKAFSQIGTSCLQQLLETNVTRLSPERWEKVSATFVRLFRT
TTPHQLFDENLRAERQQIFGQIIVKCILQLLLIEMTSDLLKNEEFYSAIPPDQLLKIMGILDHSYQFARSFNDD
KQLRTELWKESTSAATLVNVLLRMYYDNRPDYRPYRHQVAERLLPLALGVLGDYNKLRPDTQAKNIYAWNPVVA
EILDGFGRFDDNAFNTFLHAIYPLAVELLSRDPEIRQPLKVYFLRVGRAQGIQRLALIMTFQTKYGKEALAPIL
PALFANLSLSSSATLVQTFVQLGSEITSDRDAMHGLLQRFDISDANPPHDAQLVEIVSALGRLIAEGTNVPDAS
VLIHTLASYRANLNWPAAIKTFDRPDRTVDTPTLKLLIAILLNAPRQEPHAVAGFWEPWSNPLYQLRLLDALLS
LPGDTFNFVSLPGKRIVTVDDVSGATPTIRSLALNVQGYTWNSLELFQILVKLADSESQEIRNCVREMLDKAIK
ISAEIVQMGLLQVPQWNEIRLEYSRKLLAMFLAGHPNHQLVFMRIWQIKPNYLTNAFREFYEENNLNITRILDI
LDALLEVKPFTFALDEYLNLDKWLADNVANHGEDFLRSVILFLQQKMESEKVSRLSDPAVRTLTLSPNTITIIL
RVLRNNSSAMTEADVEASIEVRNACLQIHPRLMEVEPGLTVVNYSNEIETEVDSIYKQMYDDVITVGQVIQMLK
QYKESSNTHDHEVFSCMIHFLFDEYKFFQYPARELQMTADLFGSLIQHKLIDYIPLGIAIRYIIDALNCPPETN
LFRFGRQALGRFEFRLVEWRPLCEALLRIPALAETSPELIQTIQRAEISDKILFIVNNLAPSNFEVKLKEMREQ
FSTEPNNHPLYLRFLDSLDKKPLNKFILQETFVKAAALLNSEKAMQSGSERNTLKNIGSWLDKPIKHKNLSFKE
LLMEGFDSGRLVVAIPFVCKTLEPCAKSRVFKPPNPWLMAVLSLLVELYHFADLKLNLKFEIEVLCKGLEIDLD
AIEATTILRNRPGVNQLAIESLLAALSNNAQINPQLSLNVNPTFNRAVRLAIDRAVREIIVPVVERSVTIAGIS
TTELVVKDFASEPNEQKLRRAGHVMAQKLAGSLALVTCKEPLKTNLAAHFRQALNEHGFTEQAVVQILVQDNLD
IACNTIEKAAMDRVAVDIDESFAQSYEIRRRHRETRPASTFTNTLPDPLRIKLSGLQPQQLSVYEDFFASHTRD
LEAVVVQIPQSLAALPANHDIRVLVSRVLMLADRERSPLMISQKIVQLLYKTPSQLGREVYIAILEQLCQSFEE
VAKEAINWLLYAEDERKYNIPVTITLLRSGLINPTLHDQQLAKNARPNLVAYAVGLIREALTGDLPQSNFQFSI
EWFHQWVAIFQRTPSPEKAFVPFITSLTKQGILKVEDSSFFFRVCTEASVAVYIKCIATGEFDYAFQALDAFSR
LIVYIIKYHGDANNEQAKVHYFTKILSIFVLVLANMHEEQGFQPKPFFRFFSTLVNDLHSVEGSLGPVYFQLLL
ALSDTYSSLQPTYFPGFAFSWMCLISHRLFMPRLLLSENREGWSAFHKLLLSLFKFLAPFLKEGDLRQAERDLY
RGVLRLLLVILHDFPDFLSEYYFTLCDIIPPRCVQLRNIILSAFPAAVVLPDPHLRNVSMGPIPPILSDFTSAL
RTGDLRNHLDQYLLSRSSVAFLSQLKDKLESYNLSLINAIVMYIGVSSVAQAKARSGSSLFVASDPGAVALQYL
ATNLDNEGQHHLLSSIILHLRYPNAHTHWFSSLLLHLFLEVKDDRFREIMTKVLLERFIVHRPHPWGALVTFIE
LLRNPKYDFWSKPFVRIAPEVTVLLESVGHRRLFLQICFRNISDLLAVRREIMPLALANGAKRDAVDAYAEVVD
PRECIIDIREYDVPYHLRVAIDNDIRVGLWYAITFTAGQPTFERVKRADPVVMAYDIETTKAPLKFPDQAIDQV
MMISYMVDGQGYLIVNREIVSEDIEDFEYTPKEGYEGPFIVYNEPNEEATIKRWFSHIQEVKPTIMATFNGDFF
DMPFIDARAKVNGLDMFLETGFAIDAEEEYKSRTCVHMDCFRWVKRDSYLPQGSQGLKAVTTAKLGYNPIELDP
ELMTPYAVEQPQVLAQYSVSDAVATYYLYMKYVHPFIFSLCNIIALNPDEVLRKGSGTLCETLLMVEAYRSHII
MPNKHEETHGNMYEGHLLSSETYVGGHVEALEAGVFRSDIATDFKVVPEALTLIDDLDAALTFCVTNYEEVKRE
IQDKLELMRDNPKRVDNPLIYHLDVAAMYPNIMLSNRLQPDSMVQESDCAVCDFNRPGKTCDRRLQWAWRGEYF
PAHRDEFNMESFPGKRAHDPPRRFTDLSPAEQTALLHKRLGDYSRKVYSKTKETKVETRTSIVCQRENPFYVDT
VRRFRDRRYEYKGLHKKWKNNLDSIAEVDEAKKMILAHKCILNSFYGYVMRKGARWHSMEMAGITCLTGATIIQ
MARALVEQIGRPLELDTDGIWCMLPGVFPENFKFNLKNGKSIKFSYPCTMLNHLVHDKFTNHQYHDLNPETGEY
EVHSENSIFFELDGPYKAMILPSSKEEDKLLKKRYAVFNDDGSLAELKGFEVKRRGELQLIKIFQSQIFEKFLL
GTTLQECYAAVAEVADQWLDILYTRGETLSDEELVDLIAENRSMSKTLAEYGGQKSTSISTARRLAEFLGDQMV
KDKGLACKFIISAKPIGSPVTDRAVPVAIFSAEDSVKRTYLRKWLKDSSLNNFDLRAILDWDYYIERLGSVIQK
LITIPAAMQKVANPVPRIRHPDWLFRRVAAQGDKFKQNKMTDFFPGRFLLWVYVDAKLVSIPLRIPREFYVHLK
KPEFYSVEKVTKNLPHDLPCTNLFIDLTNDPNVDGVFELQSLNRAQQTGLDLQQLDTKHIFLYHATTPNGKVQV
FAVFYPNGTKLHIIDPAVRRQPTVHHSNDVTALKAISRELGLLTDSSLMVVISSNKDVSYFDRLVPRLSKFPVV
AMSSARNAHLLNLPWQSTAGVKILKRYLALGPWLDRMIALADYYPLLLSDITYARRLIHQDTVLWWSPSDLPDL
GGIEADIQALEDWPRTDFLSPGVYSNVCLEVTVRNLAVNSVLQSVVINELEGSGGTTAFDSSQRDITLGGSRVS
THAFSILKNMLKGWLLDKISPACLALDHFWRWVTSKGSQLYDPSLHRFIHGLMRKTFIQLLAEFKRLGSHVVYA
DFSTILLATSKPPGTAHAYATYITTAVTSHELFQHIYLHTEKFYDFLICMDRANMGGVVCEDPLAVEPPAELAV
EMNWNIQHFLPPAIQHDFGSVVQYYIVELYRIRQKNDSEKSMIVDFIARKFTRKLLKVIFPTLPGSHLHMTHPT
LELIKFLCAVFSLAKDYTVEIGLLKRNLLELINVREFAQEAIYRNPCEPLKLSNVPCRHCDLLRDFDFCRDLSL

LPWLCTNCNGEYDKTVIEFSLIDMTKSIEQKFARQDLRCSKCQQIQSDNVSRYCCSGTYQYTLNKAEAKKTLKT
MVNIAREYKLARLFEVEHLKYATLATAAHLQTQKEIAGIKTVRNVIEYNEQYVSKRLLVSLIWAFSGDAKGEWI
AWQSRVPDIEILYSWLSEHKPLMLCGPPGSGKTMTLFSALRKLPDMEVVGLNFSSFRKTPNGVVLAPIQIGRWL
VVFCDEINLPASDKYGTQRVISLVESGGFWRTSDMAWVRLERIQFVGACGRVPLSHRFLRLVMVDYPGEVSLKQ
IYGTYNRALLKVVPNLRTYAGPLTDAMVAFYLASQKRFTTDAQAHYVYSPRELTRWIRGIYEAIKPLEILSVEG
LVRVWAHEALRLFQDRLVTEEERNWTDEHIDGVAMEHFPTINILFSNWTSKNKARLRVFYEEELDVPLVLFNDV
LDHVLRIDRVFRQTTLSRFVAWINGLSIFQIKVSNKYTGDDFDLLANAEVPGLFEGFTMNPPENGLASRAATSP
ALFNRCVLDWFGDWLPSYNPPANFPIAYRVVNALVYVHSSLHQINQRLSRRQGRYPRHYLDFINHYVRLYTEKR
DELERHLHVGLDKLVIQVEELRKSLAVKRKLKRMVSDQQEAEQKKAASIEIQAALVEQDRHIEQRRAVVMADLA
DAEQMTKQLRELMKKDFLSRPSYNFETVNRASKACMIAELESKIAQYKDEYALLIRVQSKVDRSMKLLESLSRT
FDAEMSTIVGDVLLSAAFLAYGEWSNHLTEANIKFKPELSFTRLSWQSKSLPSDNLTTVTSFLDEAFLKVLESA
LRFGNPLLIQDVEHLDPILNEIRRTGGRVLIRLGSQDIDFSPSFPSVEFSPDICSRVTSQSLDQVLKVETDLMK
VQGEFRLRLRTLEKLLLQALNESTGNILDDDKVIDTLETLKREAAETDLIMKEVEQVTAEYLPLAQACSSVFFV
LEQLNIINHFYQFSFLDIFDYVLLHNPHLKSILLNDLFLVVYKRTSRALDELEFLLESGLELFSKHSIFKPVQN
HVLNNEDTWINFLIVMKCFRPDRLLQSVPGYDASYRVENQEGFTLADQAIAAASRQGTWVLLKNVHLAPSWLGE
KKLQTLNPHRSFRLFLTMEMNPSIPVNILQSRLIMNEPPPGIFLLAWFHAIVQEDMASASRTIDAWLDAASRGK
ANIDPAAIPWEALRTLIKESVYGGRVDSDFDQRILDSFVNSLFTPAAYNVDFPDGTKLEHFLSWPSWLSLPPTA
ERRKMRMLADDDLERCREWLALLPSKFNVLPPQSADSQDPLYRLFAREGSIGRLLDQVSRDLADVLISALTKGT
IPEHWKRYKVPKIANFARRLQQLDTVAGLDNGGLFFPEAFITATRAVAHRKKWSLETLSLCLDIERVNDPGLAL
EGAAWASDVLPDIGFQLPPLQGSNLLEHFHRIGLHAAEPWLSLSKEHAESMLTFDVETMPKHHPYAVMACAASE
NAWYAWISPWLLGKVVIGHNISYDRARIGEEYRLEGTQTRFIDTMSLHAAVYGISSHQRPAWMKHRKARKRWED
ITAVNSLAEVAKLHCGIEVNKDARDDLFEGNIEEVRNNLHDYLDYCCNDVHVTHEVYKKILPSFLQSCPHPVSF
AGILTMGSSFLPVNQEWEAYIERAEKTYRDMQAKVEKKLVSLAEDAWKSDPWLSQLDWTPKWPKWYWEATKPRK
GTLDITTRGRIAPLLLRLSWKDWPLTHSREHGWIYRVRSKPLDFYDNADAHLFYKLPHKDGEKANVGSPLGKTF
IKYAQDGTLKSPGDEAKEAFDLNAICSYWISARDRIMNQMVVWEKWGIIVPQVVTMGTVTRRAIEKTWLTASNA
KKNRVGSELKAMVRAPEGYAIVGADVDSEELWISSCMGDAQFGLHGATAIGWMTLEGTKAAGTDLHSKTAKILG
ISRDQAKVFNYSRIYGAGMRHAILLLLQGSSAMLPEAAQKLAENLYASTKGKNTHRDIFERKFWFGGSESYLFN
KLEEIALSDKPQTPALGCGVTYALSKEYLPTGFGSDYLPSRINWVVQSSGVDYLHLLIVSMQYLIQRYNINARY
LISVHDELRYLVTDQDKYRLALALQIANLWTRCMFAYKLGMDDLPQGVAFFSAVDVDKVLRKEVFMPCTTPSQP
DPIPSGESLDIAGVLEKTNGGSLFADGFQTPDCLVHRAPNAAWLRAQATTDFAEIKALAAEFIKRTLKASNEEL
TAILSEVDVWKWPRSDLNAWIKVLNKFDAVLEEIIAEYDLDKLQLKTFSPSTKRLVSEILRFKRLLMENSTNRK
TYNSYDRLNSLLFTSDLDILLLTLNLLLRPAQQYSAQPAVSHALSLSSGRLQSLAKKWPHVREYGAGLVDLVSK
GDAEVESLPVEAREVNMTFYRHIDEREVLAKPAMEVLADAVKTYKVPDEKFEILCRIRTAAALDKGREKLLTAR
LLAIAIYCHTHAESRASSTLFVYEPDLIIHIAELLQVDNGVRKTVNDLPHADSTIPQSFVDALLGFLTYIVSHQ
SGSSMVVGAGLVPLLIQLIENRSPTRLTTTSKTMQLLDNVLYSAPTGFHLFTTARGLDTLVERIEYEVDVDIPI
ARIAVLKHMLRSMHRMMQSAGTAEGLRNLINMSLLKTVKKIIEYRGLFGPTILPFAINIMTTFVHNEPTSLTII
QEAGLPEAFYNTIEAGIEPAIEVIQAIPNAIGALCLNEVGQAQLAKRPSIIPAVFSLFTSERHLKVLNDKENAV
LIGTAIDELIRHHPSLKAPVFSALKSALSRIEELGLEYVVPSNLTQWYQLDSIIVSYLDVIGRFLEGLFQHTDG
LNRLGRMTSLPCLPYDFANSVMVQVMRTLAEVATNDTIQHLSKLVKESLDETADFWKTPSHESNAKFRSLVTLH
IRITLLSDVFSTYAHGRAAIGLLLGTLHRASIWENIVFNAGLNAAAIKHITHGLPLAPFFQAMVKRRNPDSAQR
KQIQESSKIVADIMLKHLYYTVVLGLFSLLLIEEQLYAFYRIDGKELVQAYGGLKVVLHLLQPTITAKYVVESG
QSHLIVTRKKDTDPDYFEPHHFLVKLRVAALPLLTSLWQSGWLLQAPVAVTRAVVRALLEVMKGEGEPIIDAER
ALIRAGNNVNAATELLISHPILNQGRDALGSSLSRHGLTLIDEHQQVKDFSPHAYDVQEQPMANRCRLLALILE
NPPKWLAGHLLVIESLFILSDEPRAISIPIAVGPCRQAAKQVVFKLCLRLLTLEELQSDELLSVLRLLVLFTRN
REMADSLLQADALAQMGGSSYIATILRHLVEDSSTVKNIMQQNIKRYFSHPRVVEAGTYVKNCSSMALRDPELF
IEATKSLCQLNQPESTVHLLITELISCVKYVFFVMQCLSELLLSYDSCKLAFLSYSPKRFRSGTLHFILNELLT
FGTINLRNKIMLCSWATNMMIALCADTAKDVSADLASVRKYVLEALGRAIKDLPSDDNLNSKYGRLLALSDLCN
RLLTVRFETLTHIAKVMLEKNFVATLTASLSDVDLNYPHVRSLVTAILKPLQYLTKVAIKMSHEETPDLYRNSS
LGMYPEAIVHPLLLNHRHGREFEPLVTIQRWTEEAKILHGEFVAERVAKLVNHVILTLLPEAIERVTVMIHGNP
VDITDTGIDPDFLEALPDDMREEVLNQHVRDQRAAQVERPADSQISNEFLDALPPEIRAEILQQEAIEPTEIDP
ASFIASLDPTLRQAVLMEQDDGFLATLPKPKAQHDAIQLLDKGGVAVLVRLLFFPKKSFLFRIFVNLCQNSKTR
TELFNFLLSILQDGTGDLAAVDKSFAQMSVRPDLVAKKCLDALTYIVTANPHASLFFLTEHELPAGLRKSKKGK
GKEKQPQSHYPIVLLLGLLDRKALLRTPAIMDSVVNLLASVTKPLTELKKVLLANPPQIPHAVLRLIVNILTVG
ECPAKTFQQSMNLIQHLSYISDARDVIAQELRTKAQEFGRVLIADLEQLMQTLSTKFSPASSTQAKLLRVLKTI

EHMYSIYESFNFSGLWRKLGDCLGLVGAKPETEHIATVLLPLIESLMVVCQHVGTKEAMEELFISFTDNHRKLL
NLMVRNNPSLMSGSFSLLVNNPRVLDFDNKRNYFNQQLHKRREHRHTLQLNVRRARVFEDSFQHLQRKTGDQIK
YGKLNVRFYDEEGVDAGGVTREWFQILARQMFDPNNALFQPCAADKQTYQPNKNSWVNPEHLSFFKFVGRVIGK
AIFDGRLLDAYFARSLYRQLLGKPVDYKDVEWVDPEYYKSLCWILENDPTVLDLTFSFGVNRVIPLKEGGDQIP
VTQENKREFVQLSAQYRLYSSIKEQIENLSAGFYEIVPKDLITIFNEQELELLISGTPDIDVDEWRAATDYVGY
TSSDPNIVWWWRALKSFNRDERAKVLSFATGTSRVPLNGFTDLQGVQGVQRFSIHRAYGENDRLPQAHTCFNQI
DLPQYSSYEMLRQQLLLAINEGGEGFAFSKSSATAATRKKHAKKLRSFNKKAQVTKIRALEELNHVPALLIHPS
RRVRFLAASIHLSFLGTWSMSAHDVDKTVATTSTKSWFIQQTVLDPEGIYTSLHPEESDQDKKARLRIGALGAL
GWGYAQPPVRKAMWSLVHVLLSRAMLRSAWVESDLSVQSVLWHPLLTFLKDFPSAWAYEEFLEFLRKGSPIQGY
PVVVLIMSTIPASMFAAFWEALDSRALAFLSSVLESTGFLIRRTQVILYGRLLPPTEKELEEMLEGLSSTPIDP
SMAVVDPLDQNGFSSYARGVFAVLNRVLAKKNLWALKHFVVLEGILEARVLRVVLDHVLEELESADAGKWVVYA
RKIERSAPETSLSIIYSVAQARLETQKLDRYRNEVAASLLGIPGLLALRKLAASAPDPDSDVVFLPQLRAVNVV
KACQKWVEEGVDEEVENAMLPVFCALAPILQNDHGKHWEFVFDLVETVLENSSVEDDDTLVGLSRALKMLLVIE
DLCMTNKGLRAWEPRRTNVLTLVRDLSLPRSICRELLLSIEYLVIEVGVGNASFKVKSMYIEQLRNFDLVGSKL
LPNIIAALRLDKAFKIGLWGVDEFYVQYFEPISIPVLAAHLYYRSLLCVPSLVHTWVLDCKDRQLSNAITTITA
QYFSPLIIQAELAHVRSPVDENRWRAWVLAVQQTHNGRIVDGLSLFKKNVTLHFEGQVECAICYSRYGRSRTSP
PLFLLIVFVIQVSPHLDLS TVKTPSSRPF GLQNCPEFHP TTEEFKDPMA YIRSISDRAK DYGICKIIPP
EGWRMPFVTD TKFRFKTRLQ RLNSIEASSR AKLNFLEQLY RFHKQQGNPR VVVPTINHKP
LDLWLLRKEV HKMGGYEAVK GKKWSDLGRT LGYRGPGLST QLKNSYTRVI LPFEHYTERA
RNSLCDGCDC GFHIFCLDPP LTFIPKEQWF CFTCLSGTGD FGFDEGAEHS LSTFQARDLE
FRKLWFETHP PVSEYDVEEE FWRLVASPHE TVEIEYGADV HSTTHGAMPT METHPLDPAS
KDPWNLNNIP ILPDSLLRFI KSDISGMTVP WTYVGMAFST FCWHNEDHYT YSINYMHWGE
TKTWYGIPGD DAEKFEAAIM SEAPDLFENQ PDLLFQLVTL MNPQRLTEAG VRVFACNQRA
GEFVITFPKA YHAGFNHGLN FNEAVNFALP DWLPLGRDCV QRYREHRKLP VFSHDELLIT
ITQQSQSIKT AIWLVDCLRE MTEREFEDRK KVRLGLAEIL KEEDPEEQYQ CHVCKAFCYL
SQVVCQCVVC ADHVELLCEA MTLRKRFSDE ELTETLARVS ERASQPTAWR SKLARLLTEN
ARPPLRSLRA LLAEGDRINY PLPEMPNLRK CVTRANEWVD FANSFIIRKQ SRKRDRPDRG
LDELYAHLRE VENLGFDCPE INTLKTLAQQ AEDTKAKAIA LLCKRLLLEG SSLNVLLDEL
NEVEKIVDRE QLVNELLEEV RQLLTRARSC GLPSDNKHMQ FLEARLREGN TWEERARAVL
EQPIKTIAEL PIDPTIIDRL MAARAKALDF KKQAKAWLAC AKSRISEALR LAARSEKDFS
IPEVTELKKA AEWALDLESK SESVLRNRYQ LEDLFATIKK WQSHAVNHLR YSLPTFEKLT
EQVRLHELWL RDLPWFCHSE ELLADVLDCT RPEDDAPPTD EYYTCICNHP VRPPPPGVVS
DAVQCDHCFA RFHGECAKNG GSCPFCDHHH WNGTIHKQRS WHFCYLPMLA RKAPEISKHY
SRDFRELELI VHRVDRLSAS IGQFLSYTSH QRPEFIPQVR HYMRKLFRIQ FAVSPNPDIS
YGLDLAGLHR ILATRRPKKR RRPRFTFGQD VDKNWSDGTR CICRGVTCQR LYHVGCVFQC
PICCLRRGKR YEYSDVRVRT FSRELIFKKL SQPVGRTLFV ELVNYQP
>Fomitopsis pinicola
MRRTTQDQSILLTGETGSGKSENRRLAIKTLLELSVSQPGKKGAKLVNQVPAAEFVLETFGNARTLFNPNASRF
GKYTELQFTRGRLCGMKTLDYYLERNRVAGAPSGERNFHVFYYLVAGASPEERQYLHLQDKTTYRYIGLRDAVR
FDQLKMALKNVGFSKRHVAQTCQLVAAILHLGNLEFTIDRFRNEEAAVVRNTDVLDIVADFLGVQPSALEASLS
YRTKLVKKEVCTVFLDPDGASDNRDELAKSLYALLFAWLNEHINQRLCRDDFNTFIGLFDLPGPQNLSRPNSLD
QFCVNFANERLQNFIQKKLFESHVVEYNSEGISRLVPQVAYFDNSECLRLLQNKPGGLIHIMDDQARRMPKKTS
HTMVEAFTKRWGNHSSFKAGSIDRFPTFTVNHFTGPVVYSSENFLERNLEALNPDFVSLLRGGSSNPFIRSLYS
NKAITAQAHPRNEETIVAAQQPVKPMRAPSTRRKGTPCVAGEFRAALDTLFETLEETQSWYVFCINSNDSQLPN
QLEGRSVKGQVRSLGLAEVAKRNVTVFKANMTPEEFVQRYSGYLTTLNVHEGDKDIVLGMSQVYLSQRAFHALE
DDLRSKDTEEQKRNRMRDAEASNQQLPLVQNAQPMDDYDGRSAYTSHRESYAPSRNMFQDADGEIQEGETTEVL
KESTLRRRWVMICWLLTFWCPTPFLRWFGRMKRPDVQQAWREKLALNMLIWFICGCAIFVIAVFGNLICPTEHV
FTTSELQEHSNNVYTSIHGEYGGVSSDDIFPLQVSALCNGVSGSVNPYVQYSSANTDVNAQYHDFRAWTGDSRP
DWYFEQMVQMRWNNRVGWMGYTTQDLNNMAGKGSSVGVYEGISLPAVMFNSTSSTFLTFKPDKSLHFTFLTIGS
RGDVQPYIALAKGLMKDGHGIEFGYVGGDPAELMRICVENGTFTVAFLKEGMLKFRGWIDDLLKTSWDACQGTD
VLVESPSAMSGYHIAEALGIPYFRAFTMTWTRTRAYPHAFAVPEHKSYVLFDQVFWRATAGQINRWRRNTLGLG
GTSLDKMEPHKIPFLYNFSPVIVPPPLDWPEWIHVTGYWFLDDADVSSKKWSPPQDLIDFLDSARKAQKKVVYS
DPKSMTRCVIEAIVRSGVYAILSKGWSDRLDPEEPLPKQIYPIASVPHDWLFGGIPTIIKPFFGDQFFWADRVE
ALGIGTAVRKLTVESLTQALITATTDQKQITRARYVGEKIRAEDGDRPREIFEPLRLACETRNEKLMVASLDCI

SKLISYSFFVELVDVVVHTITSCHSETTPDPVSLQIVKALLALVLSSTILVHGSSLLKAVRTVYNVFLLSSDPV
NQTVAQGGLTQMVNHVFDLFIKDAFLVFRALCKLTMKPLNSESERDLKSHAMRSKLLSLHLVLTILNTHMAIFV
SPSAIIYSSSSREATSFVQAVNQYLCLCLSRNAVSPVPQVFEISVEIFWRVISGLRTKLKKEIEVLLHEIFIPI
LEMKTSTLRQKVVILNMIYRLCQDPQVLVEIYLNYDCDSEAVDNIYEHLMNIISKIGTPSLSTTALAGPGSAAG
LTEQQLKRQGLECLVAVLKSLVVWGTDDPTKFESAKQKKTTLLEGIKKFNFKPKGIQFFLETGFLPSKSPKDIA
KFLLETDGLSKAMIGEGEEENIAIMHAFVDMMEFKDLAFVDALRTFLQAFRLPGEAQKIDRFMLKFAERYIGGN
DAAYVLAYSVILLNTDAHNPQVKRRMTLADFIKNNRGINDLPEELLSTIYDDIVSNEIRMKDEVANVGRDLQKE
AYMMQSNSMTNEQFFSASHFTHVRPMFEVAWIPFLAGISGPLQDTDDLEIVELCLDGFKAFVTTLSKFTFLNNL
GEMKTKNMEAIKALLDVAVTDGNNLKGSWREVLTCVSQLEHMQLISSSRSRKPPTEELANESRSTHITVAADMV
FSLSHYLSGTAIVDFVRALCDVSWEEIQSSGLSQHPRLFSLQKLVEIAYYNMNRIRLEWSNLWDILGEHFNQVC
CHNNPHVGFFALDALRQLAMRFLEKEELPHFKFQKDFLRPFEYTMIHNSNPDIRDMVLQCLQQMIQARVGNMRS
GWRTMFGVFSAASKVPTERVANSAFELVTRVNKEHFSAIVRNGAFADLTVCITDFCKVNKDDPMIKFWFPVLFG
FYDVIMNGEDLEVRRLALDSLFSTLKTYGKTFPVDFWDTVCQELLFPIFAVLKSSQDMTRFNTQEDMSVWLSTT
MIQALRNLIDLYTFYFETLERFLDGLLDLLCVENDTLARIGTSCLQQLLENNVKKLSPARWERVATTFVKLFRT
TTPHQLFDENLRVERRRIFRQIIVKCVLQLLLIEMTNDLLRNDEVYNTMPPEHLLRLMGVLDHSYQFARMFNED
KDLRTGLWKESSSASTLVHVLLRMYYDTRSEHQAARPQVMDRLMPLGLGVLGDFNKLRVDTQLKNIVAWTPVVA
EIMQGFVRLDDKAFGRYMPAIYPLATDLLSRDPEVRDRLREYFKRVGYIQGIERQALITAAMAKYGPDTVVPML
RRIFPELSLPAGTTFVQALVQLGPEITAEPAAVHALLTRFNFTASNPPQNAQVVEWVQSLARLASEGTVLPDVG
SLVKALDSFAVIFDWAGVIKAFDMPDRGVDTATLKLLIAILQNTPRAKPHAVTGFWQTWLNPLYQLRLLDALLS
LPADTFNFVTLPGARIVTVDDVANASPTIKSLAANVQGHTWNSLDLFEVLVRLSDCDSPDVRNFVREMLDKAVK
ISAELVHMGLLQAPNWGEIRVDYTQRLLALFLAGHPNHQLVFMRMWQIEPSYLTNAFRDFYEESPLNITRILDI
LDALLEVRPFTFALDEYLNLDKWLADNVTAHGADFLHAVIAFLDIKMESEKATRISDPAVRTMPLNPQTITIFL
RVLRNSSSIMRESDVDYCLDIRNACLQIHPRLMDAEPGISVVSYSAEIEAEVDGIYKQMYDEQISIDDVITLLQ
RHKESTNPRDHEIFSCMLHFLFDEYKFFQYPPRELAMTGYLFGSLIQYQLVDYIPLGIAIRYVVDALNCAPETN
LFKFGIQALTRFESRLAEWQPLCQALLKIPHLLEARPDLAAAIHRAEVSDKILFIVNNLAPSNFESKLSEMREH
FSTEPNNHQLYLRLLDALDMPLLLRFVLNETFVKSASILNSEKTLNSSSERTILKNIGSWLDRPIKHKNLSFKD
LLIEGYEHGRLTVAIPFVCKTLEPCANSKVFRPPNPWLMAVISLLAELYHFAELKSILKFEIELLCKALDIDLD
GVQATTILRNRPRALGAHIENILSSLLPLVQISPQYQLHTNQTFKRAVQMAVDRAVREIIMPVVERSVTIAGIS
TRELVSKDFATEPNEERMRKAGHLMAQKLAGSLALVTCKEPLKTNMTAHLRNFSNEFGFNESSSVNGIVADNLE
LACQAIERAAMDRAVIDVDDGFAAAYEARRRHREQRPGSALASTLPEPLRIKPTGVQPNQAGVYEDFFSLVVKD
LEAILLQIPPSLSSLPPNHEIRGLVRQILNLAERQRTPLLMSQKIVQYLYKTPTQLGREIYAALLENLCTSFEE
VAKEAITWLIYADDERKYNVPVMATLLRTGLVPILQQDQQLAKDPRPNLLNFAAELIRECLSTDASQSQFVYTL
EWFQQWVSIFQRSHSPEKAFVPFITQLTKQNILKAEDSSFFFRVCAESSVNSYIKCIQAGEFTYAFQALDAMSR
LIVYIIKYHGDANNDQAKVHYLTKILSIFVLVLADMHEEQGFEQKPFFRFFCSLLNDLHSIEGSLGTAYFQLLV
AISDTFSSLQPTYFPGFSFSWMSLISHRLFMPKLLLSENRAGWSAFYKLLVSLFKFLSPFLKAADLQPASRDLY
RGSLRLLLVLLHDFPDFLSEYYFSLCDVIPPRCIQLRNIILSAFPPTIMLPDPHLRSFEMGPIPPILSDFASVL
KAGDLRLYLDQYLLNRGVQSFLPSLKDRLETYNLSLINALVMYIGVSSVAQAKARSGSSLFVASDPGVVALQYL
ATELDAEGQHHLLGAMVLHLRYPNAHTHWFSSLMLHLFVEVKSNQFREILTKVLLERFLVHRPHPWGALVTFIE
LLRTSKYDFWNQEFIRVAPEVTLLLENVGHRRLYLQLHFRNVSDLLTVRRDLMPLALANSAKLDAVDAYAEVVD
PREGIIDIREYDVPYYLRAAMDNEIRVGLWYAVTFTAGQPAFERVKRADPVVMAYDIETTKAPLKFPDQQIDQV
MMISYMIDGQGFLITNREIVSEDIEDFEYTPKEGYEGPFTVFNEADEAATIQRFFSHIQDVKPTVMATFNGDFF
DFPFLCSRAKIHGIDMFLETGFTKDSEDEFKSRGCVHMDCFRWVKRDSYLPQGSQGLKAVTTAKLGYDPIELDP
ELMTPYAMEQPQTLAQYSVSDAVATYYLYMKYVHPFIFSLCNIIPLCPDEVLRKGTGTLCETLLMVEAFRGKII
MPNRHEDEHGNMYEGHLLASETYVGGHVEALEAGVFRSDIPTHFKIEPSAAQLIDELDAALTFYVTNYDEIKGQ
IQAALEEMRDNPLRMDKPLIYHLDVAAMYPNIMLSNRLQPDSVVDEAVCAVCDFNRPGKACDRRMTWAWRGEFF
PARRDEFNMETFPPKKPGGPQRKFIDLAQAEQTALLHKRLGDYSRKVYKKIKDTRVENREAIICQRENPFYIDT
VRRFRDRRYEYKGLHKTWKKNLDSIAEVDEAKKMILAHKCILNSFYGYVMRKGARWHSMEMAGITCLTGATIIQ
MARQLVEQIGRPLELDTDGIWCMLPGIFPENFKFQLANGKALSISYPCTMLNHLVHAQFTNHQYHDLEAETGDY
KVHSENSIFFELDGPYKAMILPSSKEEDKLLKKRYAVFNDDGSLAELKGFEVKRRGELQLIKIFQSQIFERFLL
GETTEECYSAVAQIADRWLDVLFSKAENLPTQELVELIAENRSMSKTLAEYAGQKSTSISTAKRLAEFLGDQMV
KDKGLACKFIISARPQGAPVTERAVPIAIFSAEDSVKQTYLRRWLKDNSLTTFDIRSILDWEYYIERLGSVIQK
LITIPAAMQKVSNPVPRIKHPDWLHRRVVALDDKFHQHKVTDFFPGHFAMWLSVGGDLFSVPLRIPREFYIHVR
SPELYTYEKVMRGLPRDRPCVNLFIDLTNDPSVDGVFEMQTLNRAEAIGFELAELDRKYIFLYHACSSSAPVHV
FAIFSPGTVRLHIVDPATRRQPSTYHGTDVAALKAISRELGTMENQSYTVVISSSKEQPYFDAHVPKLGKFPVL

SMPKTKVAHTLDFPWQTNVAQKMVNRYLAMGTWLDRTIALASYYPLLLADVDFARRLMEHDFVLWWSPGNRPDL
GGIEEDARPTEELPKTDFMSPGCYSNVCLEITVRNLAVNSVLHSLMVNELEGAGGATAFDSTQRDLTLGDSNVS
PQMFSVLKNMVKTWLLDKISPATVAIDHFWRWISSKAACMYDPSLHRFIHGLMRKTFIQMLAEFKRLGSHVVYA
DLSRVLLVTSKPPGTAHAYATYITTAVTSHELFQHVYLRTERFYDFLVFMDQANLGGVVCEDPLAIDPPEEICI
EMRWNIETFLPPAIQRDFRNVVRFFLVELFRTCQKKTQEVEASREFISRKLTRRLLKAVFPVLPGSHLTMTNPI
LEFVKFSCTVFTLAKDYQIEVDLLKRNLLELVGVREFASEATFHNPCEPLILSNVPCRHCDALRDFDFCRDPEL
LPWLCGNCGGEYDRTMVEFALMDMVWDLERRFAQQDLRCAKCKRIRSDNLSRHCCSGSYQLTVNKADVRRKLRT
IVNVSVAHNLNRLFEVEHLRYATLATSPHLVTQKQIAGVKTVRNVIEYNEQYLEKRLLVSIIWAFSGDARGEWF
PWTSRVPVIDILYSWLSEHKPLMLCGPPGSGKTMTLFSALRKLPDMEVVGLNFSSYRKTPNGVMLAPAQIGRWL
VVFCDEINLPATDKYGTQRVISLVEANGYWRTTDMAWVKLERIQFVGACGRVPLSHRFLRLVMVDYPGELSLKQ
IYGTYNRAALKVLPNLRTYAEPLTDAMVALYLASQKRFTTDSQAHYVYSPRELTRWVRGIYEAIKPLETLTVEG
LVRVWAHEALRLFSDRLVTEEEKSWTDESIDNVAMENFPTINILFSNWTSKNKARLRIFYEEELDVPLVLFNDV
LDHVLRIDRVFRQTTLSRFVAWMNGLNIFQIKVSNKYTGDDFDLLANAEVPGLFEGFTMNPPENGLASRAATSP
ALFNRCVLDWFGDWLPSYDPPAMFPTAYRVVNALVFVHQSLHAINQRLSRRQGRYPRHYLDFINHYVRLHNEKR
EELERHLHVGLDKLVTQVEELRKSLAIKRKLKRMVADQQEAEQKKAASIEIQAALAEQDKHIEQRRAVVMADLA
DAEQMTKHLRELMKKDFVSRPSFNFETVNRASKACMVAELEASIEQYKQEYAALIRVESKVNRSMTLLDSLSRT
FDTEMSTIVGDVLLSAAFLAYGEWSAHLVEANVKFKTELSMPRLSWQSKGLPSDNLCTVTSFLDEAFLKVLESA
LRFGNTLLIQDVEHLDPILNEIRRTGGRVLIRLGSQDIDFSPSFPSVEFSPDICSRVTSQSLDQVLKVETDLMK
MQGEFRLRLRTLEKLLLQALNESSGNILDDDKVINTLETLKREAAETDLVMREVEQVTAEYLPLAQACSAVFFI
LEQLNLVNHFYQFSFLDIFDYVLHQNPNLKGVLLNDLFLVAYKRTSRALDELEFLLESGLESYSKHAIFKPVLE
HVVQHENEWITLVLIIKCLRPDRLLQSVTGYDASYRVENQEGFTLADQAIALAARQGSWVLLKNVHLAPSWLGE
KKLQTLNPHRNFRLFLTMEANPSIPVNILQSRILMNEPPPGIFLLAWFHAVVQEDMASAFTTIDIWINSVAKGR
ANVDPAAIPWDALRTLVKQCVYGGRVDSDFDQKIIDAFVDGLFTPAAYNVDFPDGTKLDHFLTWPAWLSLPPTA
ERRKMRMLSDDDHERCKEWLEQLPSTFHTLEKQTSDNQDPLYRLFYREGTIGKLLSQVRRDLGDVLMSSLTKGT
IPDHWRRYKVHRISDFARRLAQLDQIAGLDNGGLFFPEAYITATRAVAHRKKWSLETLNLKLDIDKVNDPGLVL
EGAKWDTDVLPNTAFTLPPLQGRNVDEHFHAIGASAAQPWLSLAKDFAEDILVFDVETLPNYSPYAVMACAASK
SAWYSWVSPWLLGRAVVGHNVSYDRGRILEEYSVEGTRTRFLDTMALHIAVKGISSNQRPAWTKHRKAKKRWED
LTSANSLADVAKLHCSIEMDKEIRNDFMTSTREDILQGIQEYLNYCAGDVAVTHAVFCKTLPAFLTACPSPVSF
AGVLTMGSSFLTVNEQWEEYLANAERTYKELEAKVQKQLHDLAYQAWKSDVWLSQLEWTPRWPKWYWELTKPRK
GSLDLTVRSRVAPLLLRLSWQGWPLFHSREHGWTFRVRATPLAFKLDADASMFYKLPHKDGEQANVGSPLGKTF
VKFSQDGTLTSPGDEAKEALDMNAQCSYWISARDRVLNQMVVWQKWGIILPQVITMGTVTRRAIEKTWLTASNA
KKNRVGSELKAMVRAPEGYAIVGADVDSEELWISSIMGDAQFGLHGATAIGWMTLEGTKAAGTDLHSKTASILG
ISRDQAKVFNYSRIYGAGMRHAMLLLLQSNAAMLPEQAQQLAQNLYASTKGKNTHRDLFGRKFWYGGTESFVFN
KLEEIALSDHPQTPALGCGVTYALSKEYLPATFGDDYMPSRINWVVQSSGVDYLHLLIVSMEHLIKKYNIQARY
LISVHDELRYLVKDEDRYRAALALQIANLWTRSLFAFKLGMSDLPQGVAFFSSVDVDRVLRKEVDMPCVTPSQS
SAIPPGESLDITTVLEKTNGGSLWADGYRQPDCLVHRADSAAWLRAQASAEIEEIRQLAAELINKLLDTPEDDL
HEVLSQIDTWRWPRSDLNAWIKVLNKFDAVLEDVIRDYDIDKLQTNVFTPATKKTVCEILRFERLLLENSTNRK
TYNSYDRLNSLMFTSDLDVLILALNLLLRPAQQYTAQPAVLHALSISTPRLTSLAKRWPNLREYDISLVDLVTK
GRPQLEALQNEAREVNFTFYRHVDSKTIESKEPMDVLADTIDAYSVPDEKFELLTRIRAARAVNVSREKLVIAR
LLATAIFGHTHSEAQAQSSLLLYEPDLVAHVAELLQLDRGVRKTVADVAQPTSTLPQSFVEALFSFVTFIATHA
SGGNMVVGAGLIPLLVQVIENRLPNRLYVVSKAMQLVDNVLYGFNNAFQLFCNSRGVDVLVDRIEYEVVGKLSV
ARAAVLKHTLRSMHRMMQSSGTSEGLRGLLDSSLLKSVKKIMENRSVFGPTVLPIAINIMSTFVHNEPTCLPVV
QEAGLPEAFYSVVESGLEPVIEVIQAVPNAIGALCLNQTGQNQLAERPNIIPGLFSIFTSERHQRVLQDKENAV
LIGTAVEELIRHHPSLKQSVFASIKATMEKIYSLGKNYTPTDDIKQWYILDNTIVSFVDVLCKFLEGFFQHQEA
LDRFGKLTALPCLPYDFTSSLFVQVIRTMAEGATNETVAFLINIVREALEDTKELWETLQEESNDRLRRFITLH
IRTSLLSDIYATYAHGRAATALLLGALHRACVWENIILKSKLNAQILKHLASQIPLAPFFQAVVRRRTADSAHK
QKVRDTAAIVAQVMRQHLFYTVMLGLTTILVVDELLVAFNVAGGQELVHAFGGLKVALHLLHPIITAKPLLEAS
QTAMLQSSRKEADPDYFEPHNFLVRMRVAIISLLTRLWDASWLVSAPLGVTKSAVQIVMEVLNTEGEPRSAAER
ALTRTRNNVTAATELLLAHPELNDSRDPFKNSMGAHVLKLVDEHPSIKSFSTRAYDVQEQPMAVRCRLLALVLP
TLPKWLAAHLLVTEAILLAGDEPKSITLPLTAGSTYPEAKGISFDFCMRLLAIPMLPKDELISALRLLVLLTRE
RRFADDFVRREGLNFLGLQSHVAIILRHVVEDMPTLQHVMRQEIKRFFSHPRMVDVSGYVSGCNALALRDPSVF
VQITRELCQLSQPEALVHFLISELVRSVKYSCFLMQCLTELLFSYESCKVAFLSYSPKKYRTAAIQFLLSDMMS
FGTISARKRILLCNWAMSVIVALCVDTSKEVPAELVSVRKFVLEAISRALKDLPASEKAETRYSRLLALADLCH
RLLTVRFEVPTHIAKVMLEKNFVATLTNALAEVDLNYPNIRGVVTSILRPLEFLSKIAIKMSREEAPDLYRNSS

LGLFPDVAVHPLLVDPSQGRSLDPLLTVQRWLEEVKMLHGKFEQARLNKLVGHVVLSLLPAAVERVIVTIHGNP
VDITDTGIDPTFLEALPDDMREEVLNQHVRDQRAARVERPADSQISPEFLDALPPELRAEILQQESIEVADLDP
ATFIASLDPQLRQVVLMDSDDMLIQSLPKPPAPRDAIQLLEKPAIAVLIRLLFYPHKNLLYKVLVNLCENSKTR
TDLFNLLLNILQDGSGDLAAIDRSFAQMSFRPELIVQRCLEALTYIVSFNELSTIFFLTDHELPAGLRRSKKGK
GKEKQPQTHYPVVLLLSLLDRPSVLKTPSIVESVVTLLATVTRPLASLKKTLLAHPPVIPHTVMRSIVNILTAG
ECSGRTFSQSLSLIQHLSYVPDAREVIANELRSRAQDFGHSLLTALDELVVALASKFSPASSDQAKLLRVLKTI
DYMYSIYESFRFTPLWKRLGDCLAIIEEKPETEHIATVLLPLIESLMVVCKYVGSKESMEELFVSFTDSHRKVL
NLMVRNNPSLMSGSFSLLVHNPRVLDFDNKRNYFNQQLHRRREHHGTLQLNVRRPRVFEDSFQYLQRKTGEQIK
YGKLSVRFYDEEGVDAGGVTREWFQILARQMFDPNYALFQPCAADRLTYQPNKNSWVNPEHLSFFKFVGRIIGK
AIYDGRLLDAYFARSLYRQLLAKPVDYKDVEWVDPEYYNSLCWILDNDPSALELTFSFGVTKIIDLKENGRSIP
VTQENKREFVQLSAQYRLYSSIKDQIEALLGGFYEIIPKDLVSIFNEQEVELLISGTPDIDVDEWRAATEYNGY
TSSDPVIVWWWRALKSFNREERAKVLSFATGTSRVPLGGFTELQGVQGVQRFSIHRAYGDADRLPQAHTCFNQI
DLPQYSSYEMLRQQLLLAINEGGEGFGFAKSSASSATRKKHARKLRRLAKKDSITKRRALEDFQHMPALFLHPS
RRIRLLSVGLHSSLLGSWLLAAHDVDRQVASLARDCWFVHRTLLDPGGVYLYVNPEENEADRRARLRMGACGSA
EWGWNQPGVRRAAWSLLQTVLSSAILRSAWVEPDPNVRTAMWQPLLTFLREHPIAWAYREFLQFLELGSPLQGY
PTIIVIISTIPPSIFASFWAAVDGRALAFFSALLECLVFLVRRAEVFLFANLLPPSRSSLDDMLERFPSVPGDS
SLAVLDPLDASGMSEYARAVGALLDRHLAKTNGWALRHLLALDTVRESCILHVVLEHTLGNASKEDAQQWVQLG
RRLERQAPHTSIAIVYSVTQHAPEPPLLDRYRNELAAGIYGVPGLWLLRRLVATAPDPESDIVFLPQLRAVNLM
KTCQQWITSDIDEDVESEMTLIFLHLAPILQNVPGAHWDFIFDVMENNLENSSLVEQGSLVVLSRTLRLFIAIQ
DLASTNKALRDWHDRENTSLTFVRDISAPLSACRELALQIEHMVIEAAVESASLKVKSAYIEQLLDLGLVSDRL
LPNVITTLGLQKPFKLDIWSIEEMYLDSYSGLSVGLLAAHLYYRALLIVPSLIRSWLSECRDRQLLNRVSTYTA
THFSPAIIRTELTEVKDPVPEDRWRAWMLGIQQIRSGSITDGLSFFKKNVSSHFEGLAECAICYSIIKPCRTCK
NRFHAGCLYIRVTSFLDLS TVKTPNRRPF GLTDCPVFHP TLEQWKDPLA YVKSISDNAR KYGMCKIVPP
AGWSMPFVTD TEFRFKTRLQ RLNSIEASSR AKVNFLEQLY RYHKQQGNPR VTVPTINHKP
LDLWLLRKEV QKQGGYEAVR NKKWADLGRL LGYSGPGLST QMKNSYSRVI LPYENYRERV
RNSLCDGCDC GFHMFCLDPP LGAIPKGQWF CHTCLMGTGD FGFDEGEEHT LSTFQARDLE
FRRLWFKSHP PVTETDVENE FWRLVQSQQE TVEVEYGADV HSTTHGGMPT LETHPLDPYS
KDPWNLNNVP ILPDSLLRYI KSDISGMTVP WTYVGMVFST FCWHNEDHYT YSINYMHWGE
TKTWYSIPGE DAEKFEAAIR REAPDLFEVQ PDLLFQLVTL MNPNRIKEAG VDVYSCNQRA
GELVITFPKA YHAGFNHGLN FNEAVNFALP DWLPFGLDCV KRYQEHRKLP VFSHDELLIT
ITQQNHSIQT ALWLNDSLQE MMVREMRIRD KARLQMSEVT EGMDAEDQCQ CTKCKVFCYL
SQITCSCAVC IDHIDDLCKC RVLRKRFDDA ELQEIQMKVS ERAAVPGAWK AKLTKLLSEH
SRPPLRSLRA LLAEGERINH PLPELASLRK CVARANEWVD AANAFLVRKP SRKRDRPEHG
LTDLYAVLNE VDRLGFDCQE IGFLKNLAKE AEETREKARL LLCDRLLLHG LSLNVLVEEL
LEVEKIVLRE QLLKELLEDV RNWVARARQC DLPPENSFMK RIERLLRLGD EWERCVKDVL
MKPQRTIEQL PIYPDLLDRL LDARSRAKDY EKQAKLWISP EKPKVQDAVK LVARADKEFN
IPAVSDLKRT VDFAMDLETR CDAVLKHRYQ HSDIFQTMLQ WRKYAKEHLT FTLPNFERLD
KQLSAHFRWL ESLPWFCHAQ NLMEDVIEST RPEDDLPPND EYFTCICMTP VRPPAQGTTS
DAVQCDHCFA RFHGVCAANG GSCPFCDHHH WNGTIHKERN WHFCYLPTIL LHAPEVTKNY
SDQWKQLEII VHRVDRLAGV IGQFLSFASN QRAEYIPQVR HYMRKLYKIQ FAVSPNPEVS
FGLDLAGLHR ILAGQRVKKR RRPKFIFGQD VDPDWRDGTR CICRGRTCDK LYHAGCVFMC
PLCCIRKNRA YPYSEVRVKN FSKEIIYMKL PPPYTQTLFV ELIRFTP
>Gloephyllum trabeum
MRRTGQDQSILLSGETGSGKSENRRLSIKTLLELSVSSPGKKGSKLAHQVPAAEFILETFGNSRTLFNANASRY
GKYTELQFTRGRLCGVKTLDYYLERNRVAQVPSGERNFHIFYYLVAGASPEERQHMHLDERANYRYLGQRDGAR
FEQLKMAMKNVGLSKRHVAQTCQLVAAILHLGNLEFTIDRHRNEDAAVVRNTDTLGIVADFLGVQPAALEAALS
YKTKLVRKELCTVFLDPDGASDNRDDLAKTLYSLLFAWLNEHINQKLCKEDFATFIALFDLPGPQNMSRPNSLD
QFCVNFANERLHNFIHKRIFERDTAQYASEGLSNYVPQVPYFDNSECLRLLQNKPGGLIHIMDDQALRMPKKTD
HSMVEAFGKRWGNHSSFKMGSIDRYPTFTVHHYNGPVTYSSEGFLERNLDAVNPDFVSLLRGGSANPFVKGLFS
AKAIATQAHPRNEDTIVAAQQPQKPMRAPSTRRKNTPCVAGEFCSALDTLFETLEESQSWYVFCITPNDSQLPN
QLEGRSVKGQVRAMGLPEIARRFINVFEVNMTPDEFVERYKESLASLNVHEGDQDIVIGQHKVFLSQAAFHKLE
DHLRSKDVEEQKRNRLRDAEASSQALPLVSHASPFDDFDGRSRLTSNRESYAPSRNMFQNADADVQEGETTEVL
KESSARRRWVALCWMLTWWVPTPCIRWVGRMKRPDVQQAWREKLALNMIIWFVCACAIFVIAVLGNVICPTEHV
FSTSELASHNNNVYTSIRGEYGGTSSDNIFPVQVSALCNGVSGSVSPYVVLDSTNTDPNAQYHDFRAFTNDSRP

DWYFEVMTQMRWKNRVGFMGYTPKEIRNMASNKKAVGIYNGLSVPAVMFTSSSSTFLTFKPKESLHFTFLTIGS
RGDVQPYIALAKGLMADGHGIEFSCVGGDPAELMRICVENGTFTVAFLKEGLLKFRGWLDDLLKTSWEACQGTD
VLVESPSAMAGYHIAEALGIPYYRAFTMTWTRTRAYPHAFAVPEHRSYVLFDQVFWKAISGQINRWRRNVLRLG
GTNLDKMEPHKVPFLYNFSPTVVPPPLDWPEWIHVTGYWFLDDAEVGAKKWSPPPDLLEFIDSAHNAGKKVVYS
NPKAMTRCVIDAIVKSGVCAILSKGWSDRLEVEEPLPKQIYSIASLPHDWLFAGLPTIIHPFFGDQFFWADRVE
ALGIGTGVRHLTVEALTAALLAATTDEKQIARARQVGEKIRSENGDRPREIFEPLRLACETRNEKLMVTSLDCI
SKLISYSFFVELVDLVVHTITSCHTETTPETVSLQIVKALLALVLSPTVLVHQSSLLKTVRTVYNVFLLSTDPV
NQMVAQGGLTQMVHHVFDLFIKDAFLVFRALCKLTMKPLNTESERDLKSHAMRSKLLSLHLVLTVLNSHMALFV
APTSIIYSSSSHEATPFVQAVNQYLCLSLSRNAVSSVPQVFELSVEIFWRVISGMRTKLKKEIEVLLHEIFIPI
LEMKTSTLKQKAAILGMLQRLAQDPQALVEIYLNYDCDSQAADNIYEHLMNIISKIGTPSLSTTALSVPGSTLG
LSDFQLRKQGLECLVTVLRSLVAWGTDDPSKFESARQKKTTLLEGIKKFNSKPKGIEFFIENGFIPSRSPQDIA
RFLLHTDGLNKTMIGEGNEENIAIMHAFVDMLEFRNLGFVDALRTFLQTFRLPGEAQKVERFMEKFAERYFTTN
GAAFVLAFSTIMLNTDAHSPQVKSRMSKADFLKNNRGINDLPEEYLSALYDDIVANEIRMKDEIANVGRDLQRE
AYLMQSSGMANDQFFSASHAVHVRPMFEAAWMPFLAGLSGPLQDTDDPEVIELCLDGFKAFVTTLAKFTFLNNL
GEMKSKNMEAIKTLLDVAVSEGNYLKSSWHEVLTCVSQLERMQLISSGRPRMLPTEELANESRSTHITVAADMI
FSLSHYLNGTAIVDFVQALCDVSWDEIQSSGLSPRPRLFSLQKLVEISYYNMNRMRLEWLSLWDILGQHFNQVC
CHSNPNVGFFALDSLRQLAMRFLEKEELPHFTFQKDFLKPFEYTMVHNANPDVRDMVLQCLSQMIQARVHNLRS
GWRTLFGVLSAASKVLTERIVSSAFEIVTRVNRDHFSAIARHGAFADLTVCITDFCKVSKDDPMIRFWFPVLFG
FYDIIMNAEDLEVRRLALDSLFTTLKTYGPDYPIDFWDTVCQELLFPIFAVLKSSQDLSRFNSQEDMSVWLQST
MIQALRDLIDLYTYLFDILERFMDGLLELLCVENDTLARIGTSCLQQLLEHNVKKMSPARWDRITATFVKLFRT
TTPHQLFDESLRVDRRRIFKQIIVKCVLQLLLIETLNDLLRNDLVYSTIPPEHLLRMMGVLDHSYQFARSFNED
KDLRMALWKESSSASTLVHVLLRMYYDPRPEHQAARPQIAERLLPLELGVLQDYNRLRLDTQAKNIAAWTPVVA
EILQGFCRFDDKAFLRYMPAVYPLATELLSRGDEIREGLKDYYMRVGYAQRIQRQALIMAAQTKYGLDIVMPIV
KRILPNLTLPPGTTVVQALQQLGPEITEDPEIVLALFHRFGISKTNPPRDEQVIEIISTFARLATEGAVLCDVS
SVIRAINSFNLELDWPAVIKSFDRPERAVDTATLKLLITILLSAPRFEPHAVAGFWSAWENPMYQLRLLDALLS
LPADTFNFVSLPGRRIVTVDDVANASPTIKSLAANVQGQTWNSLDLFEVLVTHAGSESLEVHNYVREMLDKAVK
ISAELVHMGLLQVSSWNDIRLEYTQKLLAMFLAGHPNHQLVFMRIWQIDPPYLTNAFRDFYEESPLNITRILDI
LDSLLEVRPFTFALDEYLNLDKWLADNVSAHGAEFLHSVIQFLDIKMESEKATRITDPAVRTMPLNPQTIAIFL
RVLRNSSSMLHESDVDYCLEVRNACLQIHPRLMDAEPGFTVVSYSPEIEAEVDAIYKQMYDEQITIDEVIAMLQ
RNKASSNPRDHEIFSCMLHFLFDEYKFFQYPPRELAMTGYLFGSIIQYELVDYIPLGIAIRYVLDALNCPPETN
LFKFGIQALMRFESRLSEWQPLCQALLKIPHLLEARPELVPIIQRAETSDKILFIVNNLAPSNFDSKVTEMKEY
FSTEPNNHPLYLRFLDMLDGPILPKFILHETYIKSAALLNSEKTAQSSSERVILKNVGVWLDKPIKHKNLSFKD
LLIEGYDNNRLIVVIPFVCRTLEPAAKSTVFKPPNPWLMAIISLLAELYHFAELKLNLKFEIEMLCKSLDIDLD
GIEATSVLRNRPRVVGAHIESLLSQLSQRVMISPQLPFAHNHVFKRAVQLAVDRAVREIIMPVVERSVTIAGIS
TRELVAKDFVTEPNEEKLRKAGHYMAQKLAGSLALVTCKEPLKSNLTTHIRQFLLEQGFSDQSVVMLLVQDNLD
EACHAIEQAAMDRAVADVDEGFATAYELRRRHREQRTGSSFVASLPDPLRIRAAGVQANQMLVYEDFFALLNQE
LEAFIPQMPQSLAALPPNHDLRQIIRQIVLVTEAERTALLVSQKIVQYLYRTQWQLAREIYVALLDQLCQNFAD
VAKEAITWLIYAKDDRKFNVPATLTLLRSGLVQVQTQDEQLAKDPKIQLQNFAARLIRECLSIDGSQTQWSFTI
DWFQQWVSIYQRSPSPEKAFVPFITQLTKQGILKVEDSSFFFRVCAESSVQMYMKCVATGDFTHAFQALDAMAR
LIVYIIKYHGDANNDQAKVHYLTKILSIFVLVLANMHEEQGFQQKPFFRFFSSLINDLHTIEAQLGTAYFQLLI
AISDTFSSLQPTYFPGFAFSWMSLISHRLFMPKLMLHENREGWAAFHKLLLSLFKFLSPFLKTADFRLPSRDFY
RGTLRLLLVLLHDFPEFLSEYYFSLCDVIPPRCIQLRNIILSAFPPSLVLPDPHLHDVKMRTIPPILSDFTASL
KNGDLRDILDQHLLGRGTPSTLPSLKERLEAYNLSLINSVVMYIGVSSVAQSKAKNGSSLFLPSDPGVVLLQYL
VENLDVEGQHHILGAVVLHLRYPNAHTHWFSQLLLHLFSEVKDSRFREVMTKVLLERFIVHRPHPWGALATFIE
LLSNPKYGFWNEDFVRVTPEVTLLLESVGHRRLFLQLHFRNVSDLLSVRRDIVPLALANGAKRDAVDAYAEVVD
PREAIIDVREYDVPYYLRVAIDNEIRVGLWYAVTFTAGQPSFERVKRADPVVMAYDIETTKAPLKFPDQAIDQV
MMISYMVDGQGYLITNREIVSEDIEDFEYTPKEGYEGPFTIFNEPDEAAAITRFFQHIQEVKPTVMATFNGDFF
DFPFLCARSKVHGIDMFLETGFAIDSEDEFKSKTCVHMDCFRWVKRDSYLPQGSQGLKAVTTAKLGYDPIELDP
ELMTPYAMEQPQVLAQYSVSDAVATYYLYMKYVHPFIFSLCNIIPLKPDEVLRKGSGTLCETLLMVEAYRGHII
MPNRHEEAQGNMYEGHLLASETYVGGHVEALEAGVFRSDIATHFKIEPSAAQLIDELDAALMFCVTNYDEVKQQ
IQSALEEMRDNPLRHDKPLIYHLDVAAMYPNIMLSNRLQPDSVVDESVCAVCDYNRPGKQCDRRMTWAWRGEFF
PAHRDEYNMETFLSKRPGGPHRRFTDLSESEQTALIHKRLGDYSRKVYKKMKDTKVENRESIICQRENPFYIDT
VRTFRDRRYEYKGLHKTWKKNLDTIAEVEEAKKMILAHKCILNSFYGYVMRKGARWHSMEMAGITCLTGATIIQ
MARQLVEQLGRPLELDTDGIWCMLPGIFPENFKFKLKNGKAIAFSYPCTMLNHLVHDKFTNHQYHDLDPVTGQY

TIHSENSIFFELDGPYKAMILPSSKEEDKLLKKRYAVFNDDGSLAELKGFEVKRRGELQLIKIFQSQIFEKFLL
GSTTEECYAAVAEVADRWLDVLFSRAENMPDNELFDLIAENRSMSKTLAEYGGQKSTSISTAKRLAEFLGDQMV
KDKGLACKFIISARPMGAPVTERAIPVAIFSAEESVKRTYLRKWLKDNSLANFDIRSILDWEYYIERLGSVIQK
LITIPAAMQKVANPVPRIHHPDWLHRRVVAMDDKFRQHKVTDFFPGRYTLWLSIDSELVAIPLRVPREFYIHFR
SPDQYFCEQVVKHLPRGQPCVNLFVDLTIDPNVDSVFELQTLNRAESAGVDLHQLTHKYIFVYHACSNNAPVHV
FAVFHPDGVKLHIVDPATRRQGTTYHSSDVTALKAVSRELGLLEGRSYTVVISSMKEQIYFDSHVPRLAKFPVL
SMSKAKGPHSLDFPWQANVAQKMLSRYLSLGPWLDRTISLADYYPLYVADLEFARRLLKEDMVLWWSPGDRPDL
GGIEEDKRQTQEIASTEFLSPGCYVNVCLEIAVRNLAVNSVLHSVVVNELEGSGGATAFDSAPRDLTLGESNVS
PKTLNVLKSMIKTWLLDKISPATLAIDHFWRWISSNASNMYDGSIHRFVHSLMRKTFIQLLAEFKRLGSHVVYA
DFSRILLVTSKPPGTAHAYATYITTAVTSHELFQHVYLNTERFYDFLVFMDQANLGGVVCEDPLAIEPPEELAI
EMRWNIETFLPKAIQEDFRSIVRFFVINLYRTQQQKVKEMEATREFIARRLTRRCLKTVFPVLPGAHLAMTNPP
LEFVKFVCAVFHLAKDYQVEMGLLRRNLLELVGVREFADDAVFRNPCEPLRLTNVPCRHCDALRDFDFCRDTEL
LPWACGECGGDFDRTAIELALIDIVYTLERTFTQQDLRCSKCKQIQSDNVSRHCCSGAYQLTISKADVRRKLKT
IVNVAMVHNLGRLFEVEHLRYATLATSANLIAQKQIAAVKTVRNVIEYNEQYVTKRLLVNIIWAFSGDAKAEWT
AWASKVPVIDILYSWLSEHKPLLLCGPPGSGKTMTLFSALRKLPDLEVVGLNFSSYRKTPNGVVLAPTQIGRWL
VVFCDEINLPATDKYGTQRVISLVECNGFWRTSDMSWVKLERIQFVGACGRVPLSHRFLRLVMVDYPGELSLKQ
IYGTYTRAALKVVPNLRGHAEPLTDAMVEFYLASQKRFTTDVQAHYVYSPRELTRWVRGIYEAIRPLEMLTVEG
LVRVWAHEALRLFQDRLVTEEEKQWTDDNIDLVAMQHFPTINILFSNWTSKNKARLRVFYEEELDVPLVLFNDV
LDHVLRIDRVFRQTTLSRFVAWMNGLSIFQIKVSNKYTGDDFDLLANAEVPGLFEGFTMNPPQNGLASRAATSP
ALFNRCVLDWFGDWLPAYNPPAHFPIAYRVVNALVFVHMSMYAINQRISRRQGRYPRHYLDFINHYVRLFNEKR
NELERHLHVGLDKLVTQVEELRKSLAIKRKLKRMVTDQQEAEQKKAASIEIQAALVEQEKHIEQRRAVVMADLA
DAEQMTKQIRELMKKDFLSRPSFNFETVNRASKACMIAELEAKIQTYKEEYALLIRVQSKVDRSMKLLDSLSRT
FDVEMSTIVGDVLLSAAFLAYGEWASHLGEAGVKFKQELSFPRLSWQSKSLPSDNLCTVTSFLDEAFLKVLESA
LRFGNPLLIQDVEHLDPILNEIRRTGGRVLIRLGNQDIDFSPSFPSVEFSPDICSRVTSQSLDQVLKVETDLMK
VQGEFRLRLRTLEKLLLQALNESTGNILDDDKVIDTLETLKREAAETDLVMREVEQVTAEYLPLAQACSSVFFI
LEQMNLVNHFYQFSFLDIFDYVLHQNPNLKNVLLNDLFLMVYKRTSRALDELEFLLESGLQSFSKHSIFKPVQS
HISEHENEWIPFLLIIKCLRPDRLLQSVPGYDASYRVDNQEGFSLADQAIAGAARQGSWVLLKNVHLAPSWLGE
KKLQTLNPNRNFRLFLTMEANPSIPVNILQSRIIMNEPPPGIFLLAWFHAIIQEDMISAFNTIDAWLGAAARGR
ANIDPAAIPWDALRTLIKQSVYGGRVDSDFDQRTLDAFVDGLFTPAAYNVDFPDGTKLEHFLSWPSWLSLPPTA
ERRKMRQLADDEHDRCQEWLSYLPSSFNTLSKPSTENQDPLYRLFFREGTIGKLLEQVRRDLEDVLMSSLTKGT
IPDHWRRYKVHKIPNLARRLGQLDKIAGLDSGGLFFPEAYITATRAVAHRKKWSLETLNLRLDIERIDDPGLVL
EGAAWAQDVLPGTAFPLPPLLGANLDQHFHAVGRRAGQPYLALARALAEEMLVFDVETMPRYGPHPVLACAAST
NAWYAWISPWLLGRVVVGHNVAYDRARVREEYALGGTGNRWLDTMALHVAVTGISSHQRPAWMKYRKSQKRWED
LTSANALADVAKLHCGIEMDKEVRNDFMTRTPEEILENVHDYLTYCANDVAVTHAVFSKVLPEFLARCPHPVSF
AGILTMGSSFLTVNESWEEYIENAERKYKELEDKVKSRLVQLAQQAWKDDVWLSQLDWTPKWPQWYWELTRPKK
GTIDLSVRTRIAPLLLRLSWLGWPLFHSREHGWTFRVRASELDFYHPDDETLFYKLPHKDGEKANVGSPLGKTF
MKYAQDGTLKSPGDEAKDALDMNAQCSYWISARDRVMNQMVVWQKWGMILPQVVTMGTVTRRAMEKTWLTASNA
KKNRVGSELKAMVRAPEGYSIVGADVDSEELWISSVMGDAQFGLHGATAIGWMTLEGTKAAGTDLHSKTASILG
ISRDQAKVFNYSRIYGAGMRHAVLLLMQANAGMLPEQAQALAEQLYAQTKGKNTHRDLFGRKFWYGGTESFVFN
KLEEIALSDSPRTPALGCGITHALSKEYLPPGFGTDYMTSRINWVVQSSGVDYLHLLIVSMAHLIDKYKINARY
LISVHDELRYLVADEDKYRAALALQIANLWTRCLFAYRLGMGDLPQGVGFFSSVDVDKVLRKEVDMPCVTPSQP
HPIPPGESLNIQALIEKMDGASLWKDGYQEPDCLVHRASSAAWLRAQATTELSEIKLLSAELINKIHDTPDEDL
PEVLGEIDSWKWPRSDLNAWVKVLNKFDGVLEEVIKDYEIDKVQINMFTPRTKKTTSEILRFERLLLENSTNRK
IFSSYDRLNSLLSTADLDILILTLNLLLRPAQQYSAQPSVAQALHISTPRLQSLAKRWPNLREYDVGLVDLVSN
GKARVEELPSEAREVNFTFYRHLDNETLDAKPAMEILADATETYAMPDDKFELLCRIRSAQVLAAGREKLVIAR
LLAIAIFCHTHSESQASAALFLYELDIITHIAELLQLDRGVRKTVNDVANPDSTLPHAFVDALLSFVTFIASHA
AGGNMVVGAGLIPLLIQIIENKLPQRLPVVSKTMQLVDNVLYGFSNAFQLFCNNRGVEVLVDRIEHEIDGQLPV
ARGAVLKHLLRSLHRMMQSSGTAEGLRGLIDSSILKSIKKVIENRILFGPSVLPIAINIMSTFVHNEPTSLAVI
QEAGLPETFYKTIEAGIEPVIEVIQAIPNAIGALCLNQAGQDQLSARPTIIPGLFSVFTSERHLKVLQDKENSV
LIGSAIDELIRHHPSLKPPVFEAIKSTLGKIEDLGNAYVVPEDLRPWYGLDNIIVHYIDVVGRFLEGLFQHTDG
MERLLRLTALPCLPYDFANSVLVQVIRTMGETAPNEALVYLAKQVKESLADTKGFWESLEEDSNRRFRNLVTLH
TRITLLSEVYATYAHSRATAGFLLGALHRACVWENIIFKTGLNAKALHHLVKQIPLSPLFQAVVKRRNPDPVQR
KHISDAASIVANVMLKHLYYTVMLGLVTILLVDDLLVAFYRAGGRETLPAYGGLKVALHLLLPLVSSKPLFDSG
QTAFATTRKEDTDPDYFEPHNFLVRVRLAALPLMRELWEAPWLVSAPLSLSKSVIQIILQIVSGEHEPRSAAER

ALMRTHNNVNAATEMLLAHPELDEARAPLKAQMGRQALRLVDEHPSIKRFSPQAYDMQEQPLSVRCRLLALVLP
PIPKWLASHLLVVEALLVLAQEPTTITLPLRIGPDYAEARSTLFNFCMRLLALPNLPRDELLSALRLLVLLTCD
HQMAVQFAKQGGVSLVGMKSHITLLLRHIVEDSTVLHNIMRQEIKRFLSHPRIVDVGSYLKQCSSLALRDPAVF
IKVTASICQLSQPDPMVHFLITELHQAVKYPAFLIQCLTELLFSYDTCKSAFLSYSFKKHKAAALHFVLSDLIS
FGTIQSRRRIMLCNWAMSIIVALCVDTFRDIPADVVAARKIVIEAIGRAIRDLPSSEPVDSRYGRLLALADLCN
RLLTVRFESPTHIGKIMLEKNFVATLTNALGEVDLNYPNVRGVVTALLRPLEHLTKIAIRMSREETPDLYRNSS
LGMYPEPTTHPLLLDRSRTREFEPFLTLQRWAEQARIFHGKFVSDRLNKLSNHVVIALLPAAIERVTVMIHGSP
VDITDTGIDPTFLEALPDDMREEVLNQHVREQRTSRVERPSDSQISPEFLDALPPDIRAEIIQQENLEPSEMDA
ASIIASLDPHLRQAVLLDQEDGFIQSLPKPPTPRDAIQLLDKAGIVILARLLFFPHKNLLFQVLVNLCENAKTR
TELFNLLLNILQDGSGDLSAVDKSFSQMSFRPDVIAQRCLDALTFIVNSSDHFPLFFLTEHELPVGLRRSKKGK
GKEKQAQTYYPIVLLLGLLDRQSLLKAPTIMESVVSLLATITRPLSSLPKMLLANPPQIPHAVLRFVVNILTAG
ECSGRTFQQSLALIQHLSAIPDAREVIAQELKSRAQELGQSLYADLDALAEVLASKFASASSDQAKLLRVLKTI
DYMYSIYESFRFTPLWRRLGDCLSVIEERPELEHIATVLLPLIESLMVVCKYVGPKESMEDLFISFTDAHRKVL
NLMVRNNPSLMSGSFSLLVNNPRVLDFDNKRNYFNQQLHRRRDAHTTLQLNVRRSRVFEDSVQHLQRKTGDQIK
YGKLSVRFYDEEGVDAGGVTREWFQILARQMFDPNNALFQPCAADKLTYQPNSRSWINPEHLIFFKFVGRVIGK
AIYDGRLLDAYFARSLYRQILGKPVDYRDVEWVDPEYYNSLCWILENDPSPLDLTFSFGTTKVIPLKEGGTSIP
VTQENKKEFVQLSAQYRLYLSTKDQIEALLAGFYEIIPKDLIAIFNEQELELLISGTPDIDVDEWRAATEYHGY
SSSDPVIVWWWRALKSFNRDERAKVLSFATGTARVPLGGFTELQGVQGTQRFSIHKAYGDPDRLPQAHTCFNQI
DLPQYTSYEMLRQQLLLAINEGGEGFGFAKSSASSGTRKKHARKLRSLRKKDAVTKAKALEELLHMPALFLHPS
RRIRLLAASVHSSLLGAWCMAGSDADRLVARHARASWMLQRTALDPGAVYLSLNPEESEDDRNARLRVGALGGL
KWGCGQPVVRRAAWALVQALLSTVVLRSAWVEPDPTVRTVMWQPLMMFLKEFPQAWAYDEFLQFLQLGSPLQGY
PTVVIVLSTLPASFFTSFWAAVDARALAFLSSLLECTAFLVRRPDLCLFAYVLPPDSGTMLRMLEGLPTYPEDP
SAAVVNNLDSEGYSQYGRVTTAFADRQAAKEHIWALQHLLAADNPREARVLYAALQTVMSGATKEEADEWIMLA
RKLEKKAPLTSIALVSCVTAFGPEPPRLDRYRNELAAGIFGVPGLLSLLRLAATAPDPESDVVFLPQPRAINLM
KACQQWVESDISEDVESAMTLIFYHLVPILQDVPGGHWDFIFDVIENNLENGSFNENSTLVTLARTFRLIIAIQ
DIATTNKSLRAWDERRQAVLTLVRDLSTPQLMSLELAMSIENLVIEAGVDTASFKVKSGYIQHLRDLDLISTRF
LPYIFNALGLYSTFKLDVWAVDEYHVSLYDPVSAALQAAHLYYRGLLIIPSLIRAWLQDCKDRTLSTTVTTFTA
QHYSPVIIATEFARVKSPVEENRWRAWVLGVQQIQNGSIVDGLSLFKRNVSLHFEGQVECAICYSIIKPCKTCK
NRFHSTCLYIPVSPTLDMS SVKTSAPRPF GLEDCPAFYP TSEEFQDPMA YIRSISDIAK DYGICKVVPP
EGWKMPFVTD TEFRFKTRLM RLNSIEASSR AKVNYLEQLY RFHKQQGNPR VCVPTINHKP
LDLWLLRKEV KRLGGYEAVR AKKWADLGRI LGYGGPGLST QIKNSYTRVI LPYEHFQERV
RSSLCDGCDC GFHMFCLDPP LTSIPKGQWF CHTCLFGTGD FGFDEGQEHS LSSFQARDAA
FRKMWFKTHP PVSEHDVEEE FWRLVQSPTE TVEVEYGADV HSTTHGAMPT LETHPLDPYS
KDPWNLNNIP IVHDSLLRYI KSDISGMTVP WTYVGMVFST FCWHNEDHYT YSVNFMHWGE
TKTWYGIPGE DAEKFEAAIK REAPDLFEAQ PDLLFQLVTL MSPKRLTEAG VRVYACNQRA
GEFVITYPKA YHAGFNHGLN FNEAVNFALP DWLPYGLSCV QRYREHRKLP VFSHDELLVT
ITQQSQSIQT AIWLNDSLKE MTEREKDVRQ RARLGLTEVL EEEDAEDQYQ CTVCKAFCYL
SQITCPCVAC IDHVAFLCEC RVLRLRFSDD TLLETQAKVE ERAAIPDNWR AKLHKTLAET
ARPQLRTLRA LLAEGERINH HLPDLATLKK CVYRANEWTD AANSFIIRKQ SRKRDRQDGG
LEEVYELLRQ VDNLGFDSQE ITQLRNLAKE AEDTKAKALE LLCERLLLHE SSLNVHLDEL
DQVKNLVLRE QLIKELLDEV RHLLSRARSI NIPEDDKYMM LLRSRERAGS EWEESVKQLL
SKPCKDINKL PVDPTVFDRL MNARAKAKEY EKQARLYLFP EKPRPQDAMK LVTRVEKDYA
IPSVHDLKRT AEFALDLETR CDTVLKNRYS HGDMFDIMHQ WQGYAREHLT FALPNFEKLD
AQLKSHYRWL EGLPWYCHGK QILEDVIEST RPEDDLPPSD EYFTCICTKA VRPPPPGSVS
DAVQCDHCYA RFHGVCAANG GSCPFCDHHH WNGAIHKDRS WHFCYLPTIL LSAPEITKNY
SQDWKDLEII VHRVDRLSMV IGQFLAFASN QRPEYIPQVR HYMRKLYKIQ FAVSPNPEVS
FGLDLAGLHR ILAGQRMKKR RRPRFTFGQD VDKDWQDGTR CICRGRTCNK WYHAGCVFMC
PLCCLRKNRT YPYAEVRVKT FSKDIVYMKL PPPYTNTLFV ELIRFTP
>Postia placenta
MRRTNQDQSILLSGETGSGKSENRRLAIKTLLELSVSQPGKKGSKLAHQVPAAEFVLEAFGNSRTLFNPNASRF
GKYTELQFTRGRLSGMKTLDYYLERNRVSGAPSGERNFHIFYYLVSGASAEERQHLHLQEKTTYRYLGQRDAVR
FDQLKVALKNVGFSKRHVAQTCQLLAAILHLGNLEFTIDRARNEDAAVVRNTDILEIVADFLGVQPAALEAALS
YRTKLVKKELCTVFLDPDGASDNRDELAKSLYALLFAWLNEHINQRLCRDDFNTFIGLFDLPGPQNLSRPNSLD
QFCINFANERLQNFIQKRLFESHASEYANEGISRFVPQIPYFDNSECLRLLQNKPGGLIHIMDDQARRIPRKND

HTMVESFTKRWGNHSSFKSGGMDRFPTFTVNHFGGPVTYSAESFLERNLDALNPDFVSLLRGGSTNPFVRGLYS
AKAIATQAHPRDEETIVAAQQPVKPMRAPSTRRKGTPCVGGEFRAALDTLFETLEETQSWYVFCINPNDSQLPN
QLEGRSVKGQVRSIGLAEIARRNINVFEANMTQDEFVDRYKAQFTSLNVVEGEKDVVLGTNKVFLSQAAFHAFE
DDLRSKDTEEQKRNRLRDAEASNQALPLVNNAAPFDDFDGRSAYTSHRESYAPSRNMFQDADGEIQDGETAEIL
KETPLRRRWVMLCWVLTFWVPTPLLAWAGRIKRPDVQQAWREKLAINMLIWFVCGCVIFIIAIFGPLICPTQHV
FNTNELETHSNNAYVAIHGEYAGTTADALFPLQVSALCNGVTGSVNPYVVFDSGNTDVNAQYHDFRAWTNDPRP
DWYFESMVQMRWNNRVGWIGYTMKDLSSMASSGKSVGVYDGISLPAVMFNSTSSTFLTFRPTQSLRFTFLTIGS
RGDVQPYISLAKGLMKDGHGIEFGYVGGDPAELMRICVENGTFTVAFLREGMLKFRGWVDDLLQTSWEACQDTD
VLIESPSAMSGFHIAEALKIPYFRAFTMTWSRTRAYPHAFAVPDHKSYVLFDQVFWRATAGQINRWRRNTLGLP
STSLDKMEPHKIPFLYNFSTKVVPRPIDWPEWIHVTGYWFLDDAEVSSKKWAAPPDLIEFLENARKESKKIVYS
DPKAMTRCVIDAVVRSGVYAILSKGWSDRLEPEEPLPKQIYPVASIPHDWLFAGIPTIIRPFFGDQFFWGDRVE
ALGVGTALRKLTVESLTQALIAATTDQKQIERARELGEKIRAEDGDRPREIFEPLRLACETRNEKLMIASLDCI
SKLISYSFFVELVDVVVHTITSCHSENAPETVSLQIVKALLALVLSSTILVHQSSLLKAVRTVYNVFLLSTDPV
NQTVAQGGLTQMVHHVFDLFIKDAFLVFRALCKLTMKPLNSESERDLKSHGMRSKLLSLHLVLTILNSHMALFV
SPSAVIYSSSTHEATSFVQAVNQYLCLCLSRNAVSPVLQVFEISVEVFWRVISGLRTKLKKEVEVLMHEIFIPI
LEMKTSTLKQKAIILGMLQRLCQDPQVLVEIYLNYDCDSEAVDNIYEHLMNIISKIGTPSLSTTALTGPGSQPV
LSEQQLRRQGLECLVAVLKSLVAWGTDDPSKFESAKQKKNTLLEGVKRFNTKPKGIQFFIETGFIPSNSSQDIA
RFLHETDGLNKAMIGEGDEENIVIMHAFVDQMDFRNLPFVDALRTFLQGFRLPGEAQKIDRFMLKFAERYIAGN
DAAYILAYSTILLNTDAHNPQVKRRMSLQDFIKNNRGINDLPEDFLTSIYQSIVTNEIRMKDEVANVGRDLQKE
AYVMQSNNMTNEHFFSASHFVHVRPMFEVAWIPFLAGLSGPLQDTDDLDIVEICLDGFKAFVTTLAKFTFLNNL
GEMKTKNMDAIKTLLDVAVTEGNNLKGSWREVLTCVSQLEHMQLISSSRVRKPPTEELANESRSTHITVAADMV
FSLSHYLSGTAIVEFVRALCDVSWEEIQSSGLSQHPRLFSLQKLVEIAYYNMNRIRLEWSNIWEILGEHFNQVC
CHNNPHVGFFALDALRQLAMRFLEKEELPHFKFQKDFLRPFEYTMIHNSNPDIRDMVLQCLQQMIQARVGNMRS
GWRTMFGVFSAASKVPTERIVSSAFELVTRLNKEHFTAIVRHGAFADLTVCITDFCKVNKDDPMIKFWFPVLFG
FYDVIMNGEDLEVRRLALDSLFTTLKSYGKTFPVDFWDTVCQELLFPIFAVLKSSQDLSRFSTQEDMSVWLSTT
MIQALRNLIDLYTFYFETLERFLDGLLDLLCVENDTLARIGTSCLQQLLESNVKKLSPARWERVATTFVKLFRT
TTPHQLFDESLRVERRRIFRQIIVKCVLQLLLIETTNDLLRNDEVYNTIPPEHLLRLMGVLDHSYQFARMFNED
KELRTGLWKESSSASTLVHVLLRMYYDLRPEHQAARPQVAERLLPLGLGVLGDFNKLRIDTQLKNIAAWTPVVA
EILQGFVRFDDKAFARYLPAIYPLATDLLSRDPEIREGLRDYFMRVGYIQGIQRQALIAAAQNKYGSEIVAPIV
QRLVPSLSLLPDATIGQSLADLGLDMSNDPEVVLAMLARFGVSATSPPTNEQVVDVVQGLALMAAEGRMLPDVG
TLIRVLASFDSNIQWASIIKAFDMPDRGVDTATLKLLIAILVNAPRDEDHAVTGFWQLWNNPLYQLRLLDALLS
LPADTFNFVSLPGHRIVTVDDVANASPTIKSLAANVQGHTWNSLDLFEVLVRLSESDSIDVRNFVREMLDKAVK
ISAELVHMGLLEVPPWSEIRLDYTQRLLSMFLAGHPNHQLVFMRIWQIEPSYLTNAFRGFYEESPLNITRILDI
LDALLEVRPFTFALDEYLNLDKWLADNVTAHGADFLHSVIAFLDLKMESEKATRISDPVVRTMPLNPQTITIFL
RVLRNSSSMMHESDVDYCLEVRNACLQIHPRLMDVEPGFTVVSYSAEIEAEVDGIYKQMYDEHISIEDVIALLQ
RHKTSTNPKDHEVFSCMLHFLFDEYKFFQYPPRELAMTGFLFGSLIQHQLVDYIPLGIAIRYVLDALNCAPETN
LFKFGIQALSRFESRLSEWQPLCQALLKIPHLLEARHDLAASIQRAEVTDKILFIVNNLAPSNFEAKLADMREQ
FSTEPNNHQLYLRFLDALDVQPLNRYVLHETLVKAAALLNADKTMQLTSERVILKNMASWLDRPVKHKNLSFKD
LLLEGYENGRLLVAVPFVCKTLEPCAQSKVFRPPNPWLMAVISLLAEMYHFADMKSILKFEVELLCKALDIDLD
AVQLTATLRNRPRVLGAHIENILSSILPRVTINPQLSLHTNQTFKRAVQMAVDRAVREIIVPVVERSVTIAGIS
TRELVAKDFATEPNEEKMRQAGHLMAQKLAGSLALVTCKDPLKSNLGGHIRALLVDCGFSEVGGIMVLANDNLD
LACQAIEKAAMDRAVIDVDEGFAAAYETRRRHREQRPTTAITSTFPDPLRIKASGVQPIQAAVYEDFFNIMVKD
LEALLLQLPQSLAALPPNHDVRQLVRQVLFLADRHRTPLLMSQKIVQLLYKTPSQLGREIYVALLDQLCHSFED
VAKEAITWLIYAEDDRKLNVPVTVTLLRSGLINVVQEDQQLAKDPRPSLLNFAAELIRECLTADASQNQFTHTL
AWFQQWVTIFQRSHSPEKAFVPFITQLTKQGILKVEDSSFFFRVCAEASVESYITCINAGEYGYAFQALDAMSR
LIVYIIKYHGDANNDQAKVHYLTKILSIFVLVLANLHEEQGFQQKPFFRFFCSLLNDLHTIEANLGTAYFQLLL
AIGDTFSSLQPTYFPGFAFSWMSLISHRLFMPKLLLSENREGWSAFYKLLVSLFKFLSPFLKAADLEPASRDLY
RGSLRLLLVLLHDFPDFLSEYYFTLCDVIPPGCIQLRNIILSAFPPTTLLPDPHLRNVDMGPIPPILSDFTSGL
KAGDLRAYLDQYLLNRGSPSFLPSLKERLEAYNLPLVNSLVMYVGVSSVAQAKARSGSSLFTPTDPGVIVLQYL
ATNLDMEGQHHLLGSMVLHLRYPNAHTHWFSSLMLYLFQEVKNDQFREVMTKVLLERFLVHRPHPWGALVTFIE
LLRNPKYDFWSQEFIRVAPEVTLLLETVGHRRLYLQLHFRNVSDLLAVRRDIMPLALANSAKLDAVDAYAEVVD
PREGIIDIREFDVPYYLRVAMDNDIRVGLWYAVTFTAGQPSFERVKRADPVVMAYDIETTKAPLKFPDQAIDQV
MMISYMVDGQGFLITNRDIVSEDIEDFEYTPKEGYEGPFTIFNEPDEAATIRRFFSHVQDVKPTVMATFNGDFF
DFPFLCARAKVHGIDMFLEIGFAKDSEDEFKSRTCVHMDCFRWVKRDSYLPQGSQGLKAVTTAKLGYNPIELDP

ELMTPYAMEQPQTLAQYSVSDAVATYYLYMKYVHPFIFSLCNIIPLNPDEVLRKGSGTLCETLLMVEAYRGKII
MPNRHEDEYGNMYEGHLLASETYVGGHVEALEAGVFRSDIPTHFKIEPAAVQLIDELDAALTFCVTNYDEVKAQ
IQVALEEMRDNPLRMDEPLIYHLDVAAMYPNIMLSNRLQPDSVVDESVCAVCDYNRPGKACDRRMTWAWRGEFF
PARRDEFNMETFPPKRPGQPQRKFVDLSDAEQTALLHKRLGDYSRKVYKKIKDTKVENREAIICQRENPFYIDT
VRRFRDRRYEYKGLHKTWKKNLDSIAEVDEAKKMILAHKCILNSFYGYVMRKGARWHSMEMAGITCLTGATIIQ
MARQLVEQIGRPLELDTDGIWCMLPGIFPENFKFKLANGKAISMSYPCTMLNHLVHAQFTNHQYHDLDPETGEY
KVHSENSIFFELDGPYKAMILPSSKEEDKLLKKRYAVFNDDGSLAELKGFEVKRRGELQLIKIFQSQIFERFLL
GNTTEECYSAVAQVADRWLDVLFSKAENLGDDELVELIAENRSMSKTLAEYAGQKSTSISTAKRLSEFLGAQMV
KDKGLACKFIISERPMGAPVTERAVPVAIFSAEENVKRTYLRKWLKDNSLTAFDIRSILDWNYYIERLGSVIQK
LITIPAAMQKVANPVPRIRHPDWLHRRVAALEDKFRQHKVTDFFAGRFVLWLAVGGDLISIPLRIPREFYLHLR
TPDMYSSEKVTRGLPRERPCVNLFIDLTNDPNVDGVYELQSLNRAETIGFDLDQLGRKYIFLYHACASNAPVHV
FALFIPGNVKLHIVDPATRRQPSNYHGNDVAALKAISRELGLVETQSYMIVISSMKEQTYFDAYVPRLSKFPVI
SMSKAKAPHTLDFPWHTSVAQKMVNRYLSMGTWLDRTIALADYYPLLLSDIEFARRLTEHDLVLWWSPGDKPDL
GGAEDDCRPSEELPKTEFMSSGCYSNVCLEITVRNLAVNAVLHSVIVNELEGSGGATAFDSSQRDLTLGDSSVS
PQTFSILKSMVKTWLLDKISPATIAIDHFWRWISSNASHMYDPSIHRFVHGLMRKTFIQMLAEFKRLGSHVVYA
DLTRVLLVTSKPPGTAHAYATYITTAVTSHELFQHVYLHTERFYDFLLFMDQANLSGVVCEDPLAVNPPEELTI
EMRWNIETFLPPAIQRDFRTVIRFFLVELFRTLEKKMRELEASREFITRRLTRKMLKAVFPLLPGSYLTLTNPI
LEFVKFTSAVFSLAKDYQIEVGLLKRNLLELIGVREFASEAAFRNPCEPLRLTNVPCRHCDGLRDFDFCRDPEL
LPWLCVACGGEYDRTMVEFALLGMVWDLERRWAQQDLRCGKCKQLQSDNVSRHCCSGSYQLSMNKADVRRKLRT
IVNVSIAHNLTRLFEVEHLRYATLATSANLITQKQVATIKTVRNVIEYNEQYVTKRLLVNIIWAFSGDARGEWF
GWQSRVPVIEILYSWLSEHKPLMLCGPPGSGKTMTLFSALRKLPDMEVVGLNFSSYRKTPNGVILAPVQIGRWL
VVFCDEINLPATDKYGTQRVISLVEAGGYWRASDMAWVKLERIQFVGACGRVPLSHRFLRLVMVDYPGELSLKQ
IYGTYNRALLKVVPNLRTYAEPLTDAMVTLYLASQKRFTTDIQAHYVYSPRELTRWVRGIYEAIRPLEVLSVEG
LVRVWAHEALRLFQDRLVTEEEKQWTDENIDTAAMEHFPTINILFSNWTSKNKARLRVFYEEELDVPLVLFNDV
LDHVLRIDRVFRQTTLSRFVAWMNGLNIFQIKVSNKYTGEDFDLLANAEVPGLFEGFTMNPPANGLASRAATSP
ALFNRCVLDWFGDWLPSYNPPTMFPIAYRVVNALVFVHQSLHQINQRLSRRQGRYPRHYLDFINHYVRLHNEKR
DELERHLHVGLDKLVTQVEELRKSLAIKRKLKRMVADQQEAEQKKAASIEIQAALVEQDKHIAQRRAVVMTDLA
DAEQMTKQLRDVMKRDFVSRPSYTFETVQRASKACMISELEASIEQYKEEYAALIRVESKVNRSMKLLESLSRT
FDTEMSTIVGDVLLSAAFLAYGDWSTHLAEANIKFKTELSLPRLSWQSKGLPSDNLCTVTSFLDEAFLKVLESA
LRFGNTLLIQDVEHLDPILNEIRRTGGRVLIRLGSQDIDFSPSFPSVEFSPDICSRVTSQSLDQVLKVETDLMK
MQGEFRLRLRTLEKLLLQALNESTGNILDDDKVIDTLETLKREAAETDLVMKEVEQVTAEYLPLAQACSAVFFI
LEQLNLVDHFYQFSFLDIFDYVLLHNPNLKNVLTNDLFLMVYKRTSRALDEMEFLMESGLESYGKQPLFQPVVT
HVLQHEDEWLQFLIIVKCLRPDRLLQSVPGYDASYRVENQEGFTLADQAIAFAARQGSWVLLKNVHLAPSWLGE
KKLQTLNPHRNFRLFLTMEANPSIPVNILQSRILMNEPPPGIFLLAWFHAVVQEDMASAFNTIDAWINAVAKGR
ANVDPAIIPWDAIRTLVKQCVYGGRVDSDFDQKILDAFVDGLFTPSAYNVDFPDGTKLDHFLSWPAWLSLPPTA
ERRKMRMLSDDDLERCREWLEQLPSNFNTLQKQGGDNQDPLHRLFSREGSIGRLLTQVRRDLADVLMSALTKGT
IPDHWRRYKVHKISDFSRRLAQLNNIAGLDNGGLFFPEAYVTATRAVAHRKKWSLETLHLKLDIDKVNDPGLVL
EGAAWESDVLPDISFTLPQLQGHTLDEHFYAIGSAAAQPWLGLAKDLAENMLVFDVETMPAYSPYPVMAVAASK
NCWYSWISPWLLGRVVVGHNVSYDRGRILEEYNIQPTQTRFLDTMALHIAVKGISSHQRPAWIKYRKSKKRWEE
LTSANSLADVAKLHCDITMDKEVRNDFMKCTPAEILDGVQDYLNYCSTDVGVTHAVFSKTLPGFLSACPSPVSF
AGILTMGSSLLTVNEEWDAYLENAERTYKELEDKVKTRLHELAHKAWKSDVWLSQLDWTPKWPKWYWDLTRPKK
GSMDLTVRNRFSPLLLRLSWQGWPLFHSREHGWTFRVRAKPLAFNDSADATLFYKLPHKDGDKANVGSPLGKTF
IKYAQDGTLTSPGDEAKEALDMNAQCSYWISARDRVLKQMVVWQRWGIILPQVITMGTVTRRAIEKTWLTASNA
KANRVGSELKAMVRAPKGYAIVGADVDSEELWISSVMGDAQFGLHGATAIGWMTLEGTKAAGTDLHSKTASILG
ISRDQAKVFNYSRIYGAGMRHAVLLLLQSNAGMLPEQAQKLAQDLYASTKGKNTHRDLFGRKFWYGGTESFVFN
KLEEIALSDRPQTPALGCGVTYALSKEYLPATFGADYMPSRINWVVQSSGVDYLHLLIVAMDHLIKKYNIHARY
LISVHDELRYLVKEEDRYRAALALQIANLWTRSLFAFKLGMDDLPQGVAFFSAVDIDKVLRKEVDMTCVTPSQP
VPIPPGESVDIGTVLAKTNGGSLWPDSYRQPHCLTHRASSTAWLRAQATSSIAEINGLATELIEKLLETPDEDL
AEVLGQIDSWKWPRSDLNAWIKMLNKFDAILEEVIRDYDIDKLQVNALTPTTKKTVCEILRFERLLLENSTNRK
TFNSYDRLNSLMFTSDLDVLILALNLLLRPAQQYSSQPAVLHALSISTPRLTSLAKRWPNLRDYDVNLADLVSK
GKAQVEALPNEAREVNFIFYRHIDSKIIESREPMDILADTIEAYSVPDEKFELLTRIRAARALTSTREKLVTVR
LLATAIFGHTHQESQAQSLLLLYEPDLITHVAELLQLDRGVRKTVAEVAQPSSTLPQSFVEALLSFVTFIATHA
SGGNMVVGAGLLPLLIQVTENRLSNRLYVVSKTMQLLDNVLYGFNNAFQLFCNARGVEILVERIKYEVDGKLPV
ARTAVLKHTLRSIHRMMQSSGTAEGLRGLLDSSLLKSVKKIMENRSVFGPSILPIAINIMATFVHNEPTCLPVL

QEAGLSEVFYTLVESGLEPVIEVIQAVPNAIGALCLNQAGQDQLTARPNIIPGLFSIFTSERHQRVLQEKENSV
LIGTAIEELIRHHPALKQSVFDAIKSTMDKIIELGNNYIPPSDIKHWYMLDNVIVSYIDVLGKFLEGFFQCQEA
LYRLGRLTALPCLPYDFANSVLVQVIRTMAEASTLETIAFLVSLVHQSLEDTKQFWAEMEERSNAQVRQLVILH
IRTSLLSDIYATYSHGRATTSLLLGALHRACVWENIVLKAGLNVKILKHLASQIPLAPFFQAVVRRRNADPGHK
QKIRETSSIVADVMVKHLYYTVMLGLTTILLVDELLVAFNQAKGQELLHAYGGLKVALHLLHPLISSKPLFESA
QTALITTSKKETEPDYFEPHNFFVKMRVAVIPLLRDLWEASWLVTAPLGLIKSIVLAAMEILNVEGEPRSAAER
ALARTRNNINAATELLLAHPELNTSREGLKVGLGKQALRLVDEHPSIKAFSPSAYDVQEQPMGVRCRLLALVLP
NIPKWLAAHLLVAEALLVLGNDPRSISLPLTTGRRYPEGRAILFDFCLRLLALPSLPKDELLSSLRLFVLLTED
HQMAQDFLRREGVTLLGIQSHIAIILRHVVEDGPTIRHIMHQEIRRFFAHPRAVDVGSYVAGCNALALRDPAAF
VETTREICQLSQPESLIHFLIGELVKSVKYACFLMQCLTELLFSYDSCKLAFLSYSPKKHRTAAINFFLSDVMS
FGTINAKRRISLCNWAMSIIVALCVDTAKDVAPELVSVRKFVLEAVSRALKDLPTSEDPEARYSRLLALADLCH
RLLTVRFDMPTHIAKVMLEKNFVATLTNALADVDLNYPHIRGVVSAILRPLEYLSKIAIKMSRDDTPDLYRNSS
LGLCGEAAIHPLLIDRTDVRGLDPLLTVQRWLEEVKMLHGRFEQGRLTKLSNHVILALLPAAVERVTVMIHGSQ
VDITDTGIDPTFLEALPDDMREEVLNQHVRDQRAARVERPADSHISPEFLDALPPEIRAEIIQQESLEPAEIDP
ASFIASLDPQLRQVVLMDSDDVFIQSLPKPPTQRDAIQLLDKSSMAILVRLLFFPQKTLLYKVLVNLCENSKTR
TDLFNLLLNILQDGSGDLASIDKSFAQMSVRPELIVQRCLEALTYIVSSNELSSLFFLTEHELPAGLRRSKKGK
GKEKQPQTHYPVVLLLSLLDRPSILKTPSIVESVVTLLATVTRPLASLKKVLLTRPPVVPHTVMRFIVNVLTAG
ECSGRTFSQSLSLIQHLSFVPDARDIIANELRSRAQDFGHALYTALDELAAALAAKFSSPSSDQAKLLRVLKTI
DYMYSIYESFKFAPLWQRLGDCLSIIEEKPELEHVATVLLPMIESLMVVCKYVGAKETMEDLFVTFTDAHRKVL
NLMVRNNPSLMSGSFALLVHNARVLDFDNKRNYFSQQLHRRREHHTTLQLNVRRPRVFEDSFQYLQRKTGEQIK
YGKLSVRFYDEEGVDAGGVTREWFQILARQMFDPNYALFQPCAADRLTYQPNKASWVNPEHLSFFKFVGRIIGK
AIYDGRLLDAYFARSLYRLILGKPVDYRDVEWVDPEYYNSLCWILDNDPSALELTFNFGVTKIVDLKENGRSIP
VTQESKREFVQLSAQYRLYSSIKDQIEALLAGFYEIIPKDLISIFNEQELELLISGTPDIDVDEWRAATEYNGY
TSSDPVIVWFWRALKSFNREERAKVLSFATGTSRVPLGGFTELQGVQGVQRFSIHRAYGDTDRLPQAHTCFNQI
DLPQYSSYEMLRQQLLLAINEGGEGFGFAKSSASSGTRKKHARKLKRLAKKDSITKRRALEDLQHVPALFMHPS
RRIRLLAVGLHSSLLGAWCTAAHDVDRQVSLLARQSWYTHRTLLDPSGVYAYVNPEENELDRNARLRVGACGAA
EWGFAQPGVRRAAWSLLQTMLSGAVLRSAWVEPDVNVRATMWQPLLTFLKDHPSAWAYREFLQFLELGVPLQGY
PTVLIILATIPPTVFTSFWAALDGRALAFLSSLLECLVLMVRRPDVFLFSHLLPPSRADLDDMLIRLPYTPADP
SLAVVDPLDQAGFSVYARAVYALLDRHAAKINVWALRHLLALDTPRESRIIHVILQHVLSFVTKPEAEQWLQLA
RRLEKQAPHMSLAIIYSITQYAPEPLMLDRYRNELAAGMLGVPGLWLLRRLAAVAPDPESDIIFLPQLRAVNLM
KVCQQWIASDLDEEVESEMTLVFFHLAPILQNVQGVHWDLIFDVMENNLENASLKEASTLVNLSRTLRLLIAVE
DLTSTNRSLRAWQERETTCLSLVRDLFTPVSICRELALQIEHIVIEAAVESASLKVKSGYIDQLRELGLVYDNL
LPSIFTILGLYKAFKLDIWAIDEYYLDFCSPLSLRLLAAHLYYRALLVVPSLIRGWLSDCRDRQLLSAITAYTA
AHFSPAIVRSVLATLRDPVAEERWRAWVLGVQQIRSGSIVDGLAFFKKNITSYFEGLAECAICYSFIKPCKTCK
NRFHAGCLYIPVKPALDMS TVKTPNRRPF GLTDCPVFRP TLEQFKDPLA YIKSISEKAK AYGMCKIVPP
LGWEMPFVTD TEFRFKTRLQ RLNSIEASSR AKVNYLEQLY RFHKQQGNPR VVVPTINHKP
LDLWLLRKEV QKQGGYEMVR NKKWADLGRL LGYSGPGLST QMKNSYTRVI LPYEHYRERV
SKSSPHDQSA CFHMFCLDPP LTTIPKGQWF CHTCLCGTAD FGFDEGEEHS LSSFQARDLE
FRRQWFKRHP PVTETDVENE FWRLVQSQHE TVEVEYGADV HSTTHGGMPT LETHPLESSS
KDPWNLNNIP ILPDSLLRYI KSDISGMTVP WTYVGMVFST FCWHNEDHYT YSINYMHWGE
TKTWYSIPGA DADKFEAAIR REAPDLFEVQ PDLLFQLVTL MNPNRIRDAG VDVYACNQRA
GEFVITFPKA YHAGFNHGLN FNEAVNFALP DWLPFDLDCV RRYQEHRKLP VFSHDELLIT
ITQQNQSIQT ALWLNDNLQE MMVRERRIRD KARLGLKDRP EKTDPEDQYQ CSFCKVFCYL
SQITCDCVVC VDHIDELCKC RYLRLRFDDT EIQDIQMKVS DRAAIPSTWR AKLDRLLMES
ARPSLRSLRA LMAEGERINY PLSELHALRK CVVRANEWVD TANSFLIRKV SRKRDKPERT
LDELYTALSE VEDLGFDCQE INALKALANE AQKTREKARA LLCERLLIEG STLNVLVDEL
VEIEKIVSRE QLLKELLEDI RQFAARASQC ELSTDNPQMV VLSRMLHAGE EWEARVKDVL
GRPLKTVEEL PVDVELVDRL VATLVRAKDY EKQAKAWLMP EKPKVQEAAK LVTRAEKDFN
IPAVQDLKRT VDFALDLEAR CDAVLKNRYE HGDIFQTMLQ WRKYAKEHLT FTLLNFDKLD
KQLVQHARWL EGLPWYCHGQ AILEDVIEST RPEDDLPPND EYFTCICTTP VRPPAAGTVS
DAVQCDHCFA RFHGVCAANG GSCPFCDHHH WNGTIHKERS WHWCYLPTIL MHAPDVTKNY
SEDWKQLEII VHRVDRLSQV IGQFLSFASN QRADYIPQVR HYMRKLYRIQ FAVSPNPEVS
FGLDLAGLHR ILAGQRVKKR RRPKFTFGQD VDKDWTDGTR CICRGRTCSK LYHAGCVFMC
PLCCIRKNRA YPYSEVRVRT FSKDMIYMRL PPPYTQTLFI ELIRFVP

>Wolfiporia cocos
MRRTTQDQSILLSGETGSGKSENRRLAIKTLLELSVSQPGKKGAKLAYQVPAAEFVLETFGNARTLFNPNASRY
GKYTELQFTRGRLCGMKTLDYYLERNRVAGAPSGERNFHIFYYLVAGASVEERQHLHLQEKTTYRYLGQRDAVR
FDQLKMALKNVGFSKRHVAQTCQLIAAILHLGNLEFTIDRSRNEDAAVVRNTDVLELVSEFLGVQPAALEATLS
YRTKLVKRELCTVFLDPDGASDNRDELAKSLYALLFAWLNEHINQRLCRDDFNTFIGLFDLPGSQNLSRPNSLD
QFCVNFANERLQNFVQKRLFESHVSEYNSEGIARYVPQVPFFDNSECIRLLQHKPGGLIHIMDDQARRMPRKTN
QTMVEAFSKRWGNHSSFKAGAIDRCPTFTVNHFSGPVTYSSENFLERNLDALNPDFVSLLRGGSTNPFIRELYS
AKAIATQAHPRDEETIVSAQQPVKPMRAPSTRRKNTPCIAGEFRAALDTLFETLEETQSWYVFCINPNDSQLPN
QLEGRSVKGQVRSLGLSEVSRRCVNVFEVNMTSDEFVQRYRAHLTALNVTEGEKDIVLGVRQVFLSQAAFHAFE
DDLRSKDTDEQKRNRLRDAEASSQALPLVSNASPFDDYDGRSAYTSHVESYAPSRNMFQDADGEIQEGETAEVI
KETPLRRRWVILCWILTWWVPNPLLAWLGRMKRPDIQQAWREKLAINMLIWFVCGCAVFVIVFFGPIICPTEHV
FTTSELQSHSNNVYTSIRGEYGGTSSDAIFPVQVSALCNGVTGSVSPYVVLSTSNTDANAQYHDFRAWTNDPRP
DWYFEVMVQMRWNNRVGWMGYTSKDLQNMASEGKSVGVIDSISLPAVMFNSASSTFLTFKPKQSLHFTFLTIGS
RGDVQPYIALAKGLMKDGHGIEFGYVGGDPAELMRICVENGTFTVSFLKEGMMKFRGWIDDLLKTSWEACQGTD
VLVESPSAMAGYHIAEALKVPYFKAFTMTWSRTRAYPHAFAVPEHKSYVLFDQVFWRATAGQINRWRRETLGLP
STSLDKMEPHKAPFLYNFSPTIVPPPLDWPEWIHVTGYWFLDDADVSSKKWTPPPDLVEFIDSAHKAKKKIVYS
DPKAMTRCVIDAIVQSGVHAILSKGWSDRLDPEEPLPKQIYPITSIPHDWLFAGIPTIIKPFFGDQFFWADRVE
ALGIGTAVRKLTVESLTQALVNAATDQKQIDRARVVGERIRSEDGDRPREIFEPLRLACETRNEKLMIASLDCI
SKLISYSFFVELVDVVVHTITSCHSETTPEAVSLQIVKALLALVLSPTILVHQSSLLKAVRTVYNIFLLSTDPV
NQTVAQGGLTQMVNHVFDLFIKDAFLVFRALCKLTMKSLNTESERDMKSHAMRSKLLSLHLVLTILSSHMPIFV
SPSAIIYSSSSHEATSFVQAVNQYLCLCLSRNAVSPVPQVFEISVEIFWRVISGLRTKLKKEIEVLLHEIFIPI
LEMKTSTLKQKAVILNMLQRLCQDPQVLVEIYLNYDCDSEAVDNIYEHLMNIITKIGTPSLSTTALVGPGAPVA
LSEQQLRRQGLECLVAVLKSLVAWGTDDPSKFESAKQKKTTLLEGIKKFNFKPKGIQFFIETGFISSNSPKDIA
KFLLESDGLSKAMIGEGDEENIAIMHAFVDMMDFRNLAFVNALRTFLQAFRLPGEAQKIDRFMLKFAERYIAGN
DTAYVLAYSIILLNTDAHNPQVKRRMTLADFVKNNRGINDLPGEFLSSIYDDIVSNEIRMKDEVANVGRDLQKE
AYMMQSNNMTNDQFFSASHFVHVRPMFEVAWIPFLAGISGPLQETDDLEIVEICLDGFKAFVTTLAKFTFLNNL
GEMKTKNMEAIKALLDVAVTEGNHLRTSWREVLTCVSQLEHMQLISSNRVRKPPTEELANESRSTHITVAADMV
FSLSHYLSGTAIVEFVRALCDVSWEEIQSSGLSQHPRLFSLQKLVEISYYNMSRIRLEWSNLWEILGDHFNQVC
CHNNPHVGFFALDALRQLAMRFLEKEELPHFKFQKDFLRPFEYTMIHNSNPDIRDMVLQCLQQMVQARVGNMRS
GWRTMFGVFSAASKVPTERIVNSAFEIVTRLNKEHFSAIVRHGAFADLTVCITDFCKVSKDDPMIRFWFPVLFG
FYDVIMNGEDLEVRRLALDSLFTTLKTYGSTFPVDFWDTVCQELLFPIFAVLKSSQDLSRFSTQEDMSVWLSTT
MIQALRNLIDLYTFYFETLERFLDGLLDLLCVENDTLARIGTSCLQQLLESNVKKLSSPRWERVATTFVRLFRT
TTPHQLFDESLRVERRRIFRQIIVKCVLQLLLIETMNDLLRNKEVYDTIPPEHLLRLMGVLDHSYQFARMFNED
KELRTGLWKESSSAATLVHVLLHMYYDPRPAHQAARPQVAERLMPLGLGVLGDFIKLRVDTQLKNIAAWTPVVA
EIMQGFAQLDDKAFARYLPAIYPLATELLSREAELREALRDYYRQVGYIQGIQRQALIAAAHAKYGSEIVVPIL
QRILPHMSLPPETTLVQALVQMGADVTSDPEVIRALLVRYGISDKDPPTDKQVVEIIESLARLASEGAALPDVG
SLVRALSSFGGKIQWANAIKVFDMPERGVDTATLKLLIAILLNAPRADPHAVTGFWQIWNNSMYQLRLLDALLS
LPADTFNFVSLPGHRIVTVDDVNTASPTVKSLAANVQGHTWNSLDLFEVLVRLADADSPGLRNFVRDMLDKAVK
ISAELVHLGLLQAPNWGEIRLAYTQRLLSMFLAGHPNHQLVFTRIWQIEPTYLTNALREYYDESPQNITRILDI
LDTLLEVRPFTFALDEYLNLDKWLADNVATHGAEFLHAVIEFLEIKMEDEKATRITDPAVRTLQLSPQTITIFL
RVLRNNSNIMHESDIDYCLQIRNACLQIHPRLMDAEPGFTVITYPPDIEAEVDAIYRQMYEDQISIDDVIVLLQ
RNKASNNPRDHEIFSCMLHFLFDEYKFFQYPPRELAMTAYLLGSIIQCELVDYIPLGIAIRYVLDALKCPPETN
LFKFGLQALSRFEGRLHQWRPLCNALLDIPALLEVRPELGIIIRRAEVSDKILFIVNNLAPSNFDSKLTEMREH
FSTEPNNHQLYLNFLDALDVQALFRFVLHETYIKSAALLNSEKTMQSTSERTVLKNVASWLDRPIRHKNLSLKD
LLIEGYDNGRLIVAVPFVCKTLEPCAKSKVFKPPNPWLMAVISLLAELYRYAEMKSLLKFEIELLCKALDINLD
AIQPTTILRNRPRVLGTHIENILQSLLPHVIIHPQLALHTNQTFKRAVQMAIDRSVREIIVPVVERSVTIAGIS
TRELVAKDFAMEPSEEKMRKAGHLMAQKLAGSLALVTCRDPLKTNLVGHIRTFLNECGFNEQPVVQLLVQDNLE
LACQVIEKAAMDRAIVDVDDGFAAAYEVRRRHRETHPGHPLATQFPEPLRIKPAGIQQIQAVVYEDFFNAMVKD
LEALLLQLPQSLAALPPTHEIRLLVRQILYLADHQRTPLLMSQKIVQLLYKTSSPLGREIYVALLDQLCHIFED
VAKEAITWLIYAEDERKFNVPVTVTLLRSRLISIEQEDQQLAKNPRPTLLTFAAELIRECLSIDATQSQFAYTL
EWFQQWVSIFRGSHTPEKSFIPFITQLTKQGILKVEDSSFFFRVCAESSVNSYIKCVNAGEYGLAFQALDAMSR
LIVYIIKYHGDANNDQAKVHYLTKILSIFVLVLANMHEEQGFQQKPFFRFFCSLLHDLHANASSLETAYFPLLL
ALSDTFSSLQPTYFPGFAFSWMSLISHRLFMPKLLLSENRERWSAFYKLLISLFKFLSPFLKAADLQQASRDLY
RGSLRILLVLLHDFPEFLSEYYFSLCDVIPARCIQLRNVILSAFPPGVMLPDPHLRSIDTGPIPAILSDITSGF

KPGDLQGYLDQYLLNRGSPSVLSSLKDRLESYNLSLINSLVMYIGVSSVAQAKTRSGSALFVSTDPGVVALQYL
ATNVDAEGQHHLLSAMLLHLRYPNAHTHWFSSVMLHLFLEIKSEQFREVMTKVLLERFLVHRPHPWGALVTFIE
LLRNPKYDFWSQECIRSVPEVTILFEQVGHRRLYLQLHFRNVSDLLAVRRELMPLALANSAKLDAVDAYAEVVD
PREGIIDIREFDVPYYLRVAMDNEIRVGLWYAVTHVAGQPQFERVKRADPVVMAYDIETTKAPLKFPDQAIDQV
MMISYMVDGQGYLITNRDIVSEDIEDFEYTPKEGYEGPFTVFNEPDEASVIRRFFSHVQDVKPTVMATYNGDFF
DFPFLCARAKVHGIDMFVEIGFAKDSEDEFKSRTCVHMDCFRWVKRDSYLPQGSQGLKAVTTAKLGYNPIELDP
ELMTPYAVEQPQTLAQYSVSDAVATYYLYMKYVHPFVFSLCNIIPLCPDEVLRKGSGTLCETLLMVEAYRGKII
MPNRHEEEHGKMYEGHLLASETYVGGHVEALEAGVFRSDISTHFKIEPSAVQLIDELDAALTFCVTNYDEVKQQ
IQSALEEMRDNPHRMDKPLIYHLDVAAMYPNIMLSNRLQPDSVVDEAVCAVCDYNRPGKTCDRRMTWAWRGEFF
PARRDEFNMETFPPKRPGGEQRKFVDLTDAEQTALLHKRLGDYSRKVYKKIKDTKVENREAVICQRENPFYIDT
VRRFRDRRYEYKGLHKTWKKNLDPITEVDEAKKMILAHKCILNSFYGYVMRKGARWHSMEMAGITCLTGATIIQ
MARQLVEQIGRPLELDTDGIWCMLPGIFPENFKFKLSNGKAIGFSYPCTMLNHLVHAKFTNHQYHDLNPETGEY
GVHSENSIFFELDGPYKAMILPSSKEEDKLLKKRYAVFNDDGSLAELKGFEVKRRGELQLIKIFQSQIFEKFLL
GTTTEECYAAVAQIADRWLDVLFSKADSLEDEELIELIAENRSMSKTLAEYGGQKSTSISTAKRLAEFLGDQMV
KDKGLACKFIISASPQGAPVTERAVPVAIFSAEESVKQAYLRKWLKNNSLADFNIRSILDWGYYIERLGSVIQK
LITIPAAMQKVANPVSRIQHPDWLHRRVVALDDKFHQHKMTDFFPGRFAMWLNVGGDLVSIPLRIPREFYVHLR
APDMYLCEKVVRSLPRDRPCANLFIDLTNDPNIDGVFELQTLNRAETVGFELDQLGRKYIFLYHACSNSAPVHV
FALFIPGSVRLHIVDPATRRQPSTYHGNDATALKAISRELGLIENQSFMVVISSSKEQTYFDAYVPRLAKFPVL
SMSKTKIPHTLDFPWQTNVAQKMISRYLCLGTWLDRTIALADYYPLLLADIDFARRLSEQDHVLWWSPGSRPDL
GGIEDDARPTEELPKTEFMSPGCYSNVCLEITVRNLAVNAVLHSVIVNELEGSGGSTAFDASQRDLTLGDSNVS
PQMFSILKGMVKTWLLDKISPASIAIDHFWRWISSNASCMYNPSLHRFIHGLMRKTFIQMLAEFKRLGSHVVYA
DLSRVLLVTSKPPGTAHAYATYITTAVTSHELFQHVYLRTERFYDFLLFMDQANLGGVVCEDPLAVEPPEELSI
EMRWNIETFLPLAIQRDFRNTIRFFLVEFFRTCQKKTQEMGACREFIARRLTRKTLKAVFPLLPGSHLHLSNPI
LEFVKFCCAVFGLAKEYQIDVGLLKRNLLELVGVREFASEATFHNPCEPLRLANVPCRHCDNLRDFDFCRDPEL
MPWLCSNCGGEYDRTMIEFALMDMVFDLERRFAQQDLRCSKCQRLQSDNVSRHCCSGNYQLSIIKADVRRRLRT
IVNVSIAHSLSRLFEVEHLRYATLATSANLATQKQIAAIKTVRNVVEYNEQYVTKRLLVNIIWAFSGDARGEWF
AWQSRVPVIEILYSWLSEHKPLMLCGPPGSGKTMTLFSALRKLPDMEVVGLNFSSYRKTPNGVILAPVQIGRWL
VVFCDEINLPATDKYGTQRVISLVESGGYWRTSDMAWVKLERIQFVGACGRVPLSHRFLRLVMVDYPGELSLKQ
IYGTYNRALLKVVPSLRTYAEPLTDAMVTLYLASQKRFTTDIQAHYVYSPRELTRWVRGIYEAIRPLETLSVEG
LVRVWAHEALRLFQDRLVTEEEKRWTDESIDSAAMEHFPTINILFSNWTSKHKARLRVFYEEELDVPLVLFNDV
LDHVLRIDRVFRQTTLSRFVAWMNGLNIFQIKVSNKYTGDDFDLLANAEVPGLFEGFTMNPPENGLASRAATSP
ALFNRCVLDWFGDWLPSYNPPAMFPIAYRVVNALVYVHQSLHQINQRLSRRQGRYPRHYLDFINHYVRLHNEKR
EELERHLHVGLDKLVTQVEELRKSLAIKRKLKRMVADQQEAEQKKSASIEIQAALVEQDKHIEQRRAIVMNDLA
DAEQMTKHLRDTMKREFLSRPSFNFETVQRASKACMIAELEASIERYKEEYAALIRVESKVNRSMKLLESLSRT
FDTEMGTIVGDVLLSAAFLAYGEWSSHLNDANIKLKAELSLPRLSWQSKGLPSDNLCTVTSFLDEAFLKVLESA
LRFGNTLLIQDVEHLDPILNEIRKTGGRVLIRLGSQDIDFSPSFPSVEFSPDICSRVTSQSLDQVLKVETDLMK
MQGEFRLRLRTLEKLLLQALNESTGNILDDDKVIDTLETLKREAAETDVVMREVEQVTAEYLSLAQACSAVFFI
LEQLNLVNHFYQFSFLDIFDYVLHHNPRLANVLLNDLFLMVYKRTSRALEELEFLLESGLDSYGKHPLFSSVVT
HVLQNEDQWIPFLLIIKCLRPDRLLQSVTGYDASYRVENQEGFTLADQAIAFAARQGSWVLLKNVHLAPSWLGE
KKLQTLSPHRNFRLFLTMEANPSIPINILQSRILMNEPPPGIFLLAWFHAVVQEDMASAFTTIDTWVNTVAKGR
ANVDPAIIPWDAVRTLVKQCVYGGRVDSDFDQKILDAFVDGLFTSAAYNVDFPDGTKLEHFLSWPSWLSLPPTA
ERRKMRMLADDDHERCKEWLGQLPSSFNTLQRASGDNQDPLYRLFSREGSIGKLLSQVRRDLADVLMSSLTKGT
IPNHWRRYKVHKISDFARRLAQLDQIARLDNGGLFFPEAYITATRAVAHRKKWSLETLQLQLDIDRVNDPGLVL
EGATWETDVLPDISFALPPLQGRTIDQHFYAIGMDAAQPWLGLAKDLAEEMFVFDVETLPKCSPYPIMACAVSK
NAWYSWVSPWLIGRVVVGHNVSYDRARIKEEYNINTTGTRFLDTMALHVAVKGISSHQRPAWTKYRKEKKRWEE
FTSANSLADVASLHCGITIDKEIRNDFLICTREEIHEKLQDYLNYCATDTAVTHAVFTKTLPAFLKTCPSPVSF
AGVLTMGSSLLTVNEQWEKYLKSAERTYNDLSNAVKQRLIKLAEQAWEDDPWLSQLDWTPKWPKWYWDLTAPNK
GTPDITVRSRIAPILLRLEWREWPLFHSREHGWTFRVRATALEFKHEADASLFYKIPHKDGEKANVGNPLAKSF
MQHARDGILTSPEPVANAALDMNAQCSYWISARDRVMNQMVVWQKWGIILPQVITMGAVTRRAIEKTWLTASNA
KKNRVGSELKAMVRSPPGYSIVGADVDSEELWISSVMGDAQFGLHGATALGWMTLEGTKAAGTDLHSKTAKILG
ISRDQAKVFNYSRIYGAGMRHAVLLLLQANASLSPDEAQKAAERLYASTKGKSIRHHCFGRKFWHGGMESLVFN
KLEEIAMSSKPQTPALGCGVTDALSKEYLPETFGGDYMTSRINWVVQSSGVDYLHLLIVAMDHLIRKYKIHARY
LISVHDELRYLVKEEDQYRAALALQIANLWTRSLFAFKLGMSDLPQGVAFFSAVDIDKYLRKEVNMDCVTPSQP
TPLPPGESLDIKEILEKTGGGSLSPDGYKEPECLAHRAQSVHWLKAQVTDDFNEVKTLDVEFVDKLYNTPDDEL

HQALGQIDTWRWPRSDLNAWTKVLNKFDAILEEVIRDYDIDKLQVNVLTPVTKKTVCEILRFERLLLENSTNRK
IFNSYDRLNSLMFTSDLDVLLLTLNLLLRPAQQYSAQPAVIHALSLSTPRLTSLAKRWPNLREYDLNLAELVSK
GKAQVEALPNEAREVHFTFYKRIDSKTIESKEPMDILADTIEAYNVPEEKFELLTRIRTARALVPARERLVTIR
LLATAVYGHTHSESQAQSSIFLYEPDVIVHIAELLQLDRGVRKTVADVAQPSSTLPQPFVEALLSFVTYIATHA
SGGNMVVGAGLVPLLIQVIENRLPNRLYVVSKTMQLLDNVLYGFNNAFQLFCNGRGVDILVDRIEYEVDGKLSV
ARAAVLKHTLRSMLRMMQSSGATEGLRGLLDSSLLKSVKKIMETRSIFGPSVLPIAINIMATFVHNEPTCLAVI
QEAGLPEAFYRVVEAGLEPVIEVIQAVPNAIGALCLNQAGQDQLTARPNIIPGLFSIFTSERHQRVLQDKENAV
LIGTAVEELIRHHPSLKQSVLDSIKSTMDTICQIASEYVPAESDRHWYLLDNVIVSYIDVVGKFLEGFFQHQGA
LERLGKLTALPCLPYDFATSVLVQVFRTMSDTSPGETVTFMTNLIHEALADTREFWASMQEQSNAHLRKLITLH
IRTSLLSDIFSAYSHGRSSVSLLLGALHRACVWENIVLKAGLNARIFKHLASQIPLAPFFQSVVRRRSTDIVQR
QKVREVASVFADVMGKHLYYTVMLGLSTILLVDELLTTFNRVGGQELVHAYGGLKVALHLLHPIVSVKPLFESP
QTALMMTSRRDSDSDFFDPHNFMVRMRVAVVPLLRDIWQAPWLVSAPLGVCKSVVQIAMEILNVEGEPRSAAER
ALVRTRNNVSAATELLLAHPELNAARDPIKDGLGAQALRIVDEHPSVKAFSPAAYDVQEQPLAVRCRLLALVLP
TIPKWLAAHLLVTEALLVADQDCPTIELPFTITRHYQEARTIVFDFCLRLLAVPSLPKDELLSALRLFVFLTED
HEMAKEFVRRDGVGLVGIQSHVAIILRHIAEDRHTLQHVMRQEIRRYLAHPRLVDAGSFVVGCNALALRHPESF
IQVTQDICQLSNPETLVHFILTELIQSIRYPCFLMQCLTELLFSYESCKVALLSFSPKRHRTAALQLLLSDVMS
FGTINARKRIMLCNWAMSVVVALCVDTSKDVSTELVSVRKFVLEAISRQLKDLPSSENPETRYSRLLALADLTY
RLLTVRFEAPTHIAKVMLEKNFVATLTNALAEVDLNYPNIRGVVTAVLRPLDFLSKIAIKMSRDESTDLYRNSS
LGLFPEAVTHPLLVDRSDARILEPLLTVQRWLEEVKMLHGRHEQGRLSKLNNHVILALMPAAVERVTVMIHGSP
VDITDTGIDPTFLEALPDDMREEVLNQHVRDQRAARVERPPDSQISAEFLDALPPELRAEILQQESIEPAELDP
ASFLASLDPGLRQVVLMDSDDVFIQSLPKPSTPRDAIQLLDKPSITILVRLLFFPHKNLLFKVLVNLCENSKTR
SDLFNLLLNILQDGSGDLASIDRSFAQMSFRPELIVQRCLEALTYIVSSNELSSLFFLTEHELPLGLRRSKKGK
GKEKHPQTHYPVVLLLSLLDRPSILKTPSIVESVVTLLATVTRPLASLKKVLLTKPPVIPHTVMRLIVNILTAG
ECSGRTFSQSLALIQHLSFVPEARDVIANELRSRAQDFGHGLHAALDELATALASKFSPASSDQAKLLRVLKTI
DYMFSIYESFRFAPLWRRLGDCLAIIEERQEMEHIATVLLPLIESLMVVCKYVGTKESMEDLFVTFTDAHRKVL
NLMVRNNPSLMSGSFSLLVHNPRVLDFDNKRNYFNQQLHRRREHHGTLQLNVRRQRVFEDSFQYLQRKTGDQIK
YGKLSIRFYDEEGVDAGGVTREWFQILARQMFDPNYALFQPCAADRLTYQPNKASWVNPEHLSFFKFVGRVIGK
AIYDGRLLDAYFARSLYRQLLGKPVDYKDVEWVDPEYYNSLCWILENDPSALELTFSFGVTKIVDLKENGRSTP
VTQENKREFVQLSAQYRLYSSIKDQIEALLNGFYEIIPKDLITIFNEQELELLISGTPDIDVDEWRAATDYNGY
SSSDPVIVWFWRALKSFNREERAKVLSFATGTSRVPLGGFTELQGVQGVQKFSIHRAYGDTDRLPQAHTCFNQI
DLPQYSSYEMLRQQLLLAINEGGEGFGFAKSSASSATRKKHARKLRRLAKKDGVTKRRALEELVHLPFLLLHPT
RGIRQLATSLHSSLLGTWLLATYDVDRQTAARARETWFLLRALRDPGGLYAALNPEESEGDRRARLRVGACGAV
GWGHDQPGVRSAAWSLLHALLSRVVLRSAWVELDVGVRGVLWVPVLIFLRDFPQCWAWREFMDFLALGAPVQGY
PAVLVVLSTVPDSILVNFWAALDGRALAFLEALCECVVLIVRRPEIFLYAHLLPPTAQEMASMLETLPSCPADA
SIAVLDPLSSDGFSSYARISYGLLDRHTAKTNAWTLRHFLALDTVRESRILYSILQHALDEATTAEAEQWILLA
RKTERTARQMSLAILYAVTQYAPEPPILDRYRNELAAGMMGVPGLWLLRRLAASAPDPESDVIFLPQQRAVNVL
KACQQWITADLEEEVDSAMTLVFLPLAPILQNVPGGHWDLMFDVLENNLEDVSLTEPDTLVTLSRTLRLFMATE
DLASTNKALREWQQRRLTCLTLIRDLSKPLSVCRELALQAEHMVIEAAVDTASFGVKSQFISQLRDLALVGTHL
LPCLFSILGLYTPFKLGIWEIDQYDLDLYTALCLSLLSAHIYYRALLTVPTLVRSWLSDCRDRQLALSVSSYTA
RNFSPALIHAELVHVRDPVPEEKWRAWILGLQQIRSGSIVDGFGFFKRNIISLFEGLTECAICYSIVKPCKTCK
NRFHAGCLYIPVSRTLDPL SVKTPNPRPF GLTDCPIYAP TMEQWKDPLA FIRSISDEAR TYGMCKIVPP
MGWEMPFVTD TEFRFKTRLQ RLNSIEASSR AKVNFLEQLY KFHKQQGNPR VVVPTINHKP
LDLWLLRKEV QKLGGYEAVR GKKWADLGRM LGYGGPGLST QMKSSYARVI LPYEEYRDIV
RNSLCDGCDC GFHIFCLDPP LNSIPKGQWF CHTCLFGTGD FGFDEGEEHS LSSFQARDLE
FRRLWFKSHP PVSETDVENE FWRLVQSQQE TVEVEYGADV HSTTHGGMPT LETHPLNPYS
KDPWNLNNIP ILPDSLLRYI KSDISGMTVP WTYVGMVFST FCWHNEDHYT YSINYMHWGE
TKTWYSIPGS DAEKFEAAIR REAPDLFEVQ PDLLFQLVTL MNPNHLREAG VDVYSCNQRA
GEFVITFPKA YHAGFNHGFN FNEAVNFALP DWLPFGLDCV KRYQEHRKLP VFSHDELLIT
ITQQNQSIQT AIWLNDNLQE MMAREMRLRD QARFQMREAL EELDPEDQYQ CTICKVFCYL
SQITCHCVVC IDHVDQLCKC RILRKRFDDM ELKEILTKVS ERAAIPSTWR NKLNKLLTES
ARPQLRNLRG VLAEGERINY PLPELSSLRK CVLRANEWVE TANALLVRKP GRTRDKPERS
LDELYAILRD VDNLGFDCQE IGHLKTLAKD AEETKEKALT LLCERLLLHG FSHNVLVDEL
VEVEKIVLRE QLLKDLLEDI RQFVARATQC DLPADNQQMK HLKSLLRVGE DWESRALEVL
NKPQKTIDEL PVDPEVMDRL VTACVRAKEY EKQAKAWLIP GKPKVQEAMK LVSRAEKDLN

IKAIHDLKRT VDFALDLESR CEAVLRHRYQ HGDIFHTMLQ WRKYAKEHLT FILPNFEKLD
KQLTLHYRWL EGLPWFCHGQ SILEDVVEST RPEDDLPPND EYFTCICTTP VRPPAAGTVS
DAVQCDHCFA RFHGVCAANG GSCPFCDHHH WNGTIHKERS WHFCYLPTIL LHAPDVTKNY
SEDWKQLEII VHRVDRLSAV IGQFLSFASN QRPEYIPQVR HYMRKLYRIQ FAVSPNPEVS
FGLDLAGLHR ILAGQRIKKR RRPKFVFGQD IDKDWLDGTR CICRGRTCNK RYHAGCVFMC
PLCCIRKNRG YPYSEVRVKT FSKDLIYMKL PPPYTQTLFV ELIRFTP
>Phanerochaete chrysosporium
MRRTAQDQCILLSGETGSGKSENRRLAIKTLLDLSVSNPGKKGSKLAHQLPAAEFVLESFGSARTLFNANASRF
GKYTELQFTRGRLCGVKTIDYYLERARVASVPSGERNFHIFYYLMAGASPEERQHMHLNEKATYRYLAQRDAIR
FDQLKMALKNVGFSKRHVAQTCQLIAAILHLGNLEFTIDRHRNEDAAVVRNMDVLEIVAEFLGVQPAALETALT
YRTRLMKKELCTVFLDPDGAADNRDELAVTLYSLLFTWLNEHINQRLHKDDFTSFIALLDFPGPQNLSRPNGLD
QFCVNFANERLQNFIQKRLFESHVSEYNAEGIARFVPQVPYFDNSECIRLLQHRPGGLIHIMDDQARRMPRKTN
HTMVEAFSKRWGNHSSFKVGSADRFPTFTVNHFTGPVTYSAEGFLEKNQDTLSPDFVQLLRGGSINPFVRGLFT
SKAVATQVHPKNEETIIAAQQPVKPMRAPSTRRKNTPCIAGQFRAALDMLFETLEEAQAWYVFCINPNDSQLPN
QLEGRSVKGQVRSLGLAEVARRCVNVFEVAMTPQEFVERYRDTLHQVGIQEGEKDIVLGMTMVFLSHAAFHRLE
DDLRAKDTEEQKRNRIREAEASSQQLPLVSNASPFDEYDVRSALTSHRESYAPSRNMFQNADGEIQEGETVEIL
KESSARRRWVMLCWILTWWIPTPVLVHIGRMKRPDIQQAWREKLALNMLIWFMCACAVFVIAVIGPLICPTEHV
FSTSELQSHSNNVYTSIRGEYGGVAADNLFPVQVSALCNGVTGSVSPYVVLDSSNTDPNAQYHDFRAWTSDPRP
DWYFESMTLMRWNNRVGFVGIDSKGLKNMANQRRIVAVYRGLSLPAVMFASTSSTFLTFKPKKPLHFTFLTIGS
RGDVQPYIALAKGLMADGHGIEFGYVGGDPAELMRICVENGTFTVAFLKEGVQKFRGWIDDLLKTSWEACQGTD
VLIESPSAMSGIHIAEALRIPYFRAFTMTWSRTRAYPHAFAVPERKTYVLFDQVFWRGTAGQINRWRRNTLGLP
GTSLDKMEPHKVPFLYNFSPVVVPPPLDWPEWIRITGYWFLDDANVSSQKWLPPPDLLEFIAAAHKENKKIVYS
DPKAMTRCVVDAVVQSGVYAILSKGWSDRLDAPEPLPKQIYPVQSIPHDWLFAGIPTIIKPFFGDQFFWADRVE
ALGVGSAVRKLSVQSLTDALIAATTDQKQIQRAKEIGEQIRAENGDRPREIFEPLRLACETRNEKLMIASLDCI
SKLISYSFFVELVDLVVHTITSCHSESTPETVSLQIVKALLALVLSPTILVHQSSLLKAVRTVYNVFLLSVDPI
NQTVAQGGLTQMVNHVFDLFIKDAFLVFRALCKLTMKPLNAESERDLKSHAMRSKLLSLHLVLTILNNHMALIV
SPNSIIYSSSSNDSTTFVQAITQYLCLCLSRNAVSPVPQVFEASVEIFWRVISGMRTKLKKEIEVLLHEIFIPI
LEMKTSTLKQKAVILNMLQRLSQDPQALVEIYLNYDCDSEAVDNIYEHLINIVSKLATPALSTNALSGPGSTLG
TSESQLRRQSLECLVSTLKSLVTWGTDDPTKFESAKQKKTTLLEGIKKFNFKPKGIQFFLETGFIPSPAPQDVA
RFLLETDGLSKAMIGEADEENVATMHAFVDLMDFRGLEFVDALRVFLQAFRLPGESQKIDRYMLKFAERYIAGN
DTAYVLAYSTIMLNTDAHNPQVKSRMTKADFIKNNRGINDLPEELLSSIYDDIVNNEIRMKDEVANVGRDLQKE
AYMMQSNNMANEQYFNASHFVHVKPMFEVAWIPFLAGLSGPLQGTDDLEIVELCLDGFKAFVTTLAKFTFLNNL
GEMKTKNMEAIKALLDVAVTEGNNLKSSWREVLTCVSQLEHMQLITSGRPRKLPTEELANESRSTHITVAADMV
FSLSHYLSGTAIVDFVRALCDVSWEEIQSSGMSQHPRLFSLQKLVEISYYNMNRIRLEWSNLWDILGEHFNQVC
CHNNPHVAFFALDALRQLAMRFLEKEELPHFKFQKDFLRPFEYTMVHNSNPEVRDMVLQCLQQMIQARVANLRS
GWRTMFGVFSSAAKVPTERIVSSAFEIVTRLNKDHFQAIVRHGAFADLTVCITDFCKVTKDDPMIKYWFPTLFG
FYDVIMNGEDLEVRRLALDSLFSTLKTYGSTFPVEFWDTVCQELLFPIFAVLKSSQDLSRFSTQEDMSVWLSST
MIQALRNLIDLYTFHFEILERFLDGLLDLLCVENDTLARIGTSCLQQLLESNVKKLSPARWERVATTFVKLFRT
TTPHQLFDESLRVERRRIFKQIIVKCVLQLLLIETTNDLLRNEEVYNTIPPEHLLRLMGVLDHSYQFARAFNED
KELRTGLWKESSSASTLVHILLRMYYDPRPEHQASRPQVADRLLPLSLGVLQDFTKLRLDSQAKNIAAWTPVIA
EILHGFAKFDDKAFATYLPAIYPLATELLAREPEIRQNLRDYFLRVGYIQGIQRQALIMAAQTKYGPEIIMPIL
QRILPNIHLQQNTTLVQLMIQLGPEMTCDPDITSAVLARFGMTENNPPQDEQVVEIVSQLARLAAEGPVSVDVG
TLVHVLARLNPSLNWAMAIQAFDRPDRGVETSTLKLLIAILLSCPLSEHHAVSGFWQMWKNSLYQLRLLDALLS
LPSDTFNFVTLPGRRIVSTEDIPATSPSIKALAANVQVHTWNSLDLFEVLVRLADSESLEVRNFVREMLDKAVK
ISAEIVQMGLLQVTPWGEIRLEYSQRLLAMFLAGHPNHQLVFMRIWQIEPKYLLNSLREFYEENPLNITRILDI
LDHLLDCKPFAFALDEYLNLDKWLADNVTAHGADFLHGVIGFLDSKMESEKLTRISDPAVRTMPLSPQTITIFL
RVLRANSNLMHESDVDYCLEVRNACLQIHPRLMDVEPGFNVITYSPEIEAEVDSIYKQMYDEHITIDDVIALLQ
RNKTSNNPRDHEIFSCMLHFLFDEYKFFQYPPRELAMTGYLFGSLIQYQLVDYIPLGVAIRYVLDALNCPPETN
LFKFGLQALVRFESRLPEWQPLCQALLKIPHLLDARPELVNILSRAEVSDKILFIVNNLAPSNFDAKLTEMKEQ
FSTEPNNHQLYLRFLDALNIKTLFNFVLHETFVKSAQLLNSEKTLQVTSERTILKNVGAWLDRPIKHKNLSFKE
LLIEGFDNGRLIVAIPFVCKTLEPCARSKVFRPPNPWLMAVISLLAELYHFAELKLNLKFEIEVLCKSLEIDLE
TVQATTILRNRPRALDAHIENILASVLPLVVINPALALHTNQTFKRAMQMAVERSIREIIMPVVERSVTIAGIS
TRELVLKDYVTEQSEEKLRHAGRLMSQKLAGSLAQVTCKEPLRSNLASHLRLVLTEFGFAEVEVIALLVNDNVD
IACQAIEKAAMDKAALEVDEAFVTHFEARRRHREQRPGSQFANSLPDPLRIRPIGVQPIQAAVYEDFFTALIKD

LESVLPQLPQSLAALPPNHEIRQLLREILFLTDRQRTPLLISQKIVQLLYKTTLQLAREIYVALLDQLCHSFEE
VAKEAINWLIYAEDERKFNVPVTVTLLRSGLVNIGQEDQQLAKDPRPSLLNFAAGLIRECLSSDASQAQFAYTL
EWFQQWVHIYQRSPSPEKSFVPYISQLTKQGILKAEDSSFFFRVCMESSVNSYVKCVNSGEFDYAFQALDAFSR
LIVYMIKYHGDANNEQAKVHYLTKILSIVVLVLANMHEEQGFPQKPFFRFFSSLLNDLHAIEASLGAVYFPLLL
AVSDTFSSLQPTFFPGFAFSWMSLISHRLFMPKLLLSESREGWPTFYRLLLALFKFLAPFLKAADLQPATRDLY
RGSLRLLLVLLHDFPEFLSEYYFTLCDIIPPHCIQLRNVILSAFPPTIILPDPHLRNVKMGPIPPVLSDFSSVL
KAGDLRTYLDQYLLNRGSPSFLPSLKERVDKYNLSLMNALVMYIGVSSVAQAKARSGSSLFVASDPGVVALQYL
ANNLDIEGQHHLLSAMVLHLRYPNAHTHWFSSLMLHLFNEIKDDKFREIMTKVLLERFLVHRPHPWGALVTFIE
LLRNPKYDFWNHDFIRIAPEVTLLLETVGHRRLYLQLHFRNVSDLLTVRRDVMPLALANSAKLDAVDAYAEVVD
PREGIIDIREFDVPYYLRVAMDNEIRVGLWYAITFETGQPVFERVKRADPVVMAYDIETTKAPLKFPDQAIDQV
MMISYMVDGQGFLITNREIVSEDIEDFEYTPKEGYEGPFTVFNEADEPSAIRRFFSHIQEVKPTVMATFNGDFF
DFPFLCARAKIHGIDMFLETGFAIDPEEEFKSRTCVHMDCFRWVKRDSYLPQGSQGLKAVTTAKLGYNPIELDP
ELMTPYAMEQPQVLAQYSVSDAVATYYLYMKYVHPFIFSLCNIIPLNADEVLRKGSGTLCETLLMVEAFRGNII
MPNKHEEAHGKMYEGHLVASETYVGGHVEALEAGVFRSDIATQFKIEPTAVQLIDQLDAALTFCVTNYDEVKAQ
IQAKLEEMRDNPLRMDNPLIYHLDVAAMYPNIMLSNRLQPDSVVDESVCAVCDFNRPGKCCDRRMTWAWRGEFF
PARRDEFNMETFPPKKPGQPQRKFADLSPAEQTALLHKRLGDYSRKVYKKVKDTRVENREAIICQRENPFYIDT
VRRFRDRRYEYKGLHKTWKKNLDSIAEVDEAKKMILAHKCILNSFYGYVMRKGARWHSMEMAGITCLTGATIIQ
MARQLVEQIGRPLELDTDGIWCMLPGIFPENFKFKLANGKAIAFSYPCTMLNHLVHAQFTNHQYHDLDPETGEY
KIHSENSIFFELDGPYKAMILPSSKEEDKLLKKRYAVFNDDGSLAELKGFEVKRRGELQLIKIFQSQIFEKFLL
GTTTEECYAAVAQVADRWLDVLFSKADSLSDEELVELIAENRSMSKTLAEYAGQKSTSISTAKRLAEFLGDQMV
KDKGLACKFIISAKPLGAPVTERAVPVAIFSAEESVKRTYLRKWLKDNSLTNFDLRSILDWDYYIERLGSVIQK
LITIPAAMQKVANPVPRIRHPDWLHRRVVAQGDKFRQHKMTEYFPGKFTLWLSTDTGLVSVPLRIPREFYVHLR
LTDMYSVEKVVRTLPRDQPCVNLFIHLTNDPSVDGVYEQQTLNRAEVTGFDLNQLDRKYALLFHAMSSSANVHV
FALFLPSGVRLHIVDPATRRQATTYHANDLTALKALSRELGLLENQGLTMVISSLKEQTYFDSYVPKLAKFPVL
SMSKAKALHSLDLPWQPAAAQKMIRRYLTLGAWLDRTIALADYYPLLLADIEFARRLTAQDILLWWSPTDRPDL
GGIEDDRRPTEELPKTEFMSPGCYSNVCLEVTVRNLAVDAVLHSMIVNELEGSGGATAFDATQKDLTLGESSVS
PVTFSILRSMVKTWLLDKISPASLAIDHFWRWISSNASNLYEPSLHRFVHGLMRKTFIQMLAEFKRLGSHVVYA
DLSRILLVTSKPPGTAHAYATYITTAVTSHELFQHVYLHTERFYDFLIFMDPANLAGIVCEDPLAVDPPEELCL
EMRWNIQSFLPPAIQEDFKKAIRNFLVEFFKVKQKKTKEQEDSKEFIQRKLLRRMLKMVFPVLPGSHLHMTNPV
LEFVKFTCAVFALSKEYQIEVGLLKRNLLELVGVREFSPEAAFQNPCEPLKLSNVPCRHCDSLRDFDFCRDPEL
LEWTCANCYGEYDRTMIEFALIEMVWELERRFAQQDLRCTKCKQIQSDNVSRFCCSGNYQLTISKADVRRRLRT
MVNVALVHKFPRLFEVEHLKYATLATSANLATQKQIAAIKTVRNIIEYNENYVTKRLLINIIWAFSGDAKGEWV
AWQTRVPQIEILYSWLSEHKPLMLCGPPGSGKTMTLFSALRKLPDMEVVGLNFSSYRKTPNGVILAPAQIGRWL
VVFCDEINLPATDKYGTQRVISLVESGGYWRASDMAWVKLERIQFVGACGRVPLSHRFLRLVMVDYPGEVSLKQ
IYGTYNRALLKVVPNLRAYASPLTDAMVDLYLASQKRFTTDIQAHYVYSPRELTRWVRGIYEAIRPLEMLSVEG
LVRVWAHEALRLFQDRLVTEEEKQWTDEHIDSAAMEHFPTINILFSNWTSKNKARLKVFYEEELDVPLVLFNDV
LDHVLRIDRVFRQTTLSRFVAWMNGLSIFQIKVSNKYTGDDFDLLANAEVPGLFEGFTMNPPENGLASRAATSP
ALFNRCVLDWFGDWLPSYNPPVHFPIAYRVVNALVYVHQSLHQINQRLSRRQGRYPRHYLDFINHYVRLYNEKR
DELERHLHVGLDKLVTQVEELRKSLAIKRKLKRMVADQQEAEQKKAASIEIQAALVEQDRHIEQRRAVVMADLA
DAEQMTRQLRELMKKEFLSRPSYNFETVNRASKACMISELEASIERYKEEYAALIRVQSKVDRSMKLLESLSRT
FETEMSTIVGDVLLSAAFLAYAEWSTHLADANIKFKTELSLPRLSWQSKSLPSDNLCIVTSFLDEAFLKVLESA
LRFGNTLLIQDVEHLDPILNEIRRTGGRVLIRLGNQDIDFSPSFPSVEFSPDICSRVTSQSLDQVLKVETDLMK
MQGEFRLRLRTLEKLLLQALNESTGNILDDDKVIDTLETLKREAAETDVVMKEVEQVTAEYLPIAQACSSVFFI
LEQLNLVNHFYQFSFLDIFDYVLHHNPNLKSVLLKDLFLTVYKRTSRALEDLELLLESGLENFAKHPMFKPVIQ
HIVENEDQWAAFLAIVKCLRPDRLLQSVTGYDASYRVENQEGFTLADQAIATAARQGSWVLLKNVHLAPSWLGE
KKLQTLNPNRNFRLFLTMETNPSIPVNILQSRIFMNEPPPGIFLLAWFHAVVQEDLASAFNTIDAWLNAAAKGR
ANVDPASIPWDAVRTLIKQSVYGGRVDSDFDQKILDAFVDGLFTPYAYNVDFPDGTKLEHFLSWPAWLSLPPTA
ERRKMRMIADDDFDRCKEWLGQLPTEFHGLDKQSGDNQDPLYRLFYREGSIGKLLGQVRRDLADVLMSSLTKGT
IPNHWRRYKVHKVSDFARRLAQLTNIAGLGNGGLFFPEAYITATRAVAHRKKWSLETLHLQLDIERVNDPGLVL
EGASWTTDVLPDIGFTLPSLQGRTLDEHFYSIGSVAAQPWLTLAKDLGEQMLVFDVETMPEYSPYPVMACAATK
NAWYAWISPWLLGRVVVGHNVSYDRARILEEYSTDGTQTRFLDTMALHVAVKGISSHQRPAWMKYRKDKKRWED
ITSANSLADVAKLHCNIDVDKSVRNDFCTHSREQISSDITSYLDYCSSDVYVTHAVYRQVLPAFLTSCPSPVSF
AGILTMGSALLTVNHEWERYIENAERTYKELEDKVKNRLIDLARQAWKEDVWLSQLDWTPKWPKWYWDLAKPKK
GSLDITVRNRIAPILLRLSWQGWPLFYSREYGWTFRVRATPLTFYDAADEALFYKLPHKDGEKANVGSPLSKTF

IKYAQDGTLTSPGDEAKDALDMNAQCSYWISARDRILKQMVVWQKWGIIIPQVITMGTVTRRAIEKTWLTASNA
KKNRVGSELKAMVRAPNGYAIVGADVDSEELWISSVMGDAQFGLHGATAIGWMTLEGTKAAGTDLHSKTASILG
ISRDQAKVFNYSRIYGAGMRHAVLLLLQANPGMLPEEAQKLAENLYASTKGKNTHRDFFGRKFWFGGTESFVFN
KLEEIALSDKPQTPALGCGITYALSKEYLPAEFGSDYMTSRINWVVQSSGVDYLHLLIVSMEHLIKKYDIKARY
LISVHDELRYLVKEEDKYRAALALQIANLWTRCMFALKLGLDDLPQGVGFFSAVDIDEVLRKEVDMPCVTPSQP
EPIPPGESLDIYKVLEKTNGGSLHPDGYQEPDYLAHRANDAAWLRAQATSELAEIKILAAELIDKLADTPHEQL
PDVLAEIDHWRWPRSDLNAWIKVLNKFDAIMEDIIRDYEIEKVQLKPFSAFDKRLLCEILRFERLLLENSTNRK
TYNSYDRLNSFMTTSDLDVLIYSLNLLLRPAQQYSAQPAVSHALSLNTNRLTSLSKRWPSLHDFDINLVALAGQ
GRAQVDALPNEAREVSFTFYRHIDSQTLETKPTMEIWAEAIETYSVPDERFELLCRIRSARALTRAREKLVIVR
LLAIGLFCHTHPEQTTFNNLFLYEPDLVHHIAELLQLDRGIRKTITELANAESNIPQAFVEAILAFVTYIAAHA
AGGNMVVSAGLIPLLVQVIENRLPNRLYALSKTMQLLDSILYGYTNAFQLFCNARGIEVLVGRIEYEVDGKISV
GRATVLKHIMRSMHRMMQSSGTSEGLRGLLDSSLVQSVKKVMANRDVFGANVLAIAINIMATFIHNEPTCLPVI
QEAGLPEAFYGVVESGLEPVIEVVQSIPNAMGALCLNQVGQDQLASRPGIIPGFFSIFTSEKHQRMLQEKENAV
IIGTAVEELVRHHPSLKNQVFEAIKQTMARIEELGNAYQVSDDNKHWYVLDNVIVSFIDVLGKFLEGYFQHTDG
LDRLARLTALNCLPYDFANSVLVQVVRTMVEASTTESLAFIVKIVNESLQATTNLRDDNAGDSNVAFRNLVTLH
VRIMLLSDIFATFSQGRALTTLLLGSLHRSSIWENVMLKEKMNMKALKHLASQLPLAPFFQSVVRRRNPDPAQR
QRILDAADVVADVAVKHLYYTMMLGLVTILLIDELLQAFLRKGGQTLIHAHGGLKVALHLLHPLISFKPIVDSP
QSSFLLTTKKDTEPGWFDPRNLVVQLRIAILPLLKDIWQSSWLISAPLGVSKSVIHAVLELLNTDQEPRSAAER
ALIRARNNVSHATELLLAQPELNELREPLKEGLGSRILGLVDEHPAVKKFSGAALDHHEQPLSVRFRILALVLV
TIPKWLASHLLVTEALLMMGEEPRSVGLPLETGPKFNEKRPVILDFCLRLLHVPQLPRDELLSSLRLFMLLTRD
HTLALEFVKRDGISALGTHPYIASILRHIVEDPATLKQIMQQEVKAFLSNPRLLEVTGFVRSCGAMALRDPQAF
VQATAEVCRLSSPDSLVHFLISELIKSVKYSCFVMQCLTELLFSYDSCKVAFLSYSPKKHRTHALQFLINDLLS
FGTINAKQQIMLCNWAMSVIVALCVDTTKDVPPERASVRKFVLDAINRAIKDLPGHETGEARYSRLLALADLCY
RLLTVRFDAPTHIAKVMLEKNFVATLTNALAEVDPNFPDIRGVVTGILRPLEYLTKIAIKMSREEAPDLYRNSS
LGMYPEGTTHPLLLDRSDGRGFEPLLTRDRWAEEVKSLHGRFEQSRITKIAGHVVLALLPEAIERVTVLIHGNE
VDITDTGIDPTFLEALPDDMREEVLNQHIRDQRAARVERPVDSQISPEFLDALPPELRAEIIQQENLEPAEMDP
ADFLASLDPALRQTVLMDSDDMFIQALPKPSTPREAIQLLDKHAIAVLIRLLFFPQKNLLSKVLVNLSENGKTR
TDIFNLLLGILQDGTGDLSSIDRSFAQMSFRPELVAQRCLDALTYITATNEASSVFFLTEQELPAGLRRSKKGK
GKEKQSQTYYPVVLLLGQLDRQTLLRTPSLMESVAGLLSLVTKPLTSLKKVLLSHPPNIPQQVLRHVVNILTAG
ECSSRAFSHTLALIQHLSFVPGARDVIAQELCTRAQEFGQRLYSSLDELATALASKFSPASSDQAKLLRLLKTI
DYMYSIYEGFRFTALWRRLGDCLSIIEERTDLEHIATVLLPLIESLMVVCKYVGPKESMEDLFVSFTDAHRKVL
NLMVRNNPSLMSGSFSLLVNNPRVLDFDNKRNYFNQQLHRRREHHGTLQLNVRRQRVFEDSFQYLQRKSGEQIK
YGKLSVRFYDEEGVDAGGVTREWFQILARQMFDPNYCLFQPCAADRLTYQPNKASSINPEHLSFFKFVGRIIGK
AIYDGRLLDAYFARSLYRQILGKPVDYRDVEWVDPEYYKSLCWILENDPTPLDLTFSFGVTKLIELKENGAQIS
VTNENKREFVQLSANYRLYSSIKEQIEALLSGFYEIIPKDLIQIFDEKELELLISGTPDIDVDEWRAATEYNGY
TSSDPVIVWFWRALKSFNREERAKVLSFATGTSRVPLGGFVELQGVQGVQRFSIHKAYGEVDRLPQAHTCFNQI
DLPQYSSYEMLRQQLLLAIHEGGEGFGFAKSSASSGTRKKHARKLRKLAKKDSVTKRRALEDFQHVPALFLHPS
RRVRQLSVNLHVSLLGSWLLASYDIDRQVSVAARGAWFIQRVLLDPSGIYLQINPEESEADRKARMRMSGFGAL
EWGWNQPGVRRACWAAIQALLSVAILRSAWAEPDAAVRNFMWQPLLTFLREYPNAWAYEDFRQFLELGSPVQAY
PAVIIVLSTIPPSIFTSFWAAIDGHALAFLSALLECVVFMVRRPELFLFSTLVPPPQEELDAMLEDLPTYASHV
SLAVVDPLDAFGYSVYARVVLSLLDRDAAKQSLWALRHFQALDNTRESRILHSLLRHILPNVSKEEADLFIGLA
RTIEKKAPRASLAIIYAITQYAPEPPRLDRLRNELAAGLLGVPGLWLLRRLAATAPDPESDIVYLPTNRAVNLM
KPLQQWITSDLDEEVDYQMTSIFIHLAPILQSVPGAHWDLVFDVMENNLENSSLSEASSLPLLHRTLQLVIAIE
DLTSTNKALRAWLERRNQILTLVRDLDVPLSLCRELALTVEHLVLEASVESASLKVKVEYVNHLRQEGLITDYF
LPLIFNLLGLYHAFKLDIWSIDEYYLDLTSSLRLPLLAAHLYYRALRVVPGLVRSWLADCRDRQLNGTVTAYTS
THFSPAIIRAELAQAKDPVTEDRWRSWVLGVQQIRGGSIVDGLAFFKKNVTSHFEGQSECAICYSMIKPCRTCK
NKFHSGCLYIPVSPVLDLS TVKTPNPRPF GLTDCPTFRP TPEQFKDPMA YIKSIAENAK SYGMCKIVPP
LGWSMPFVTD TEFRFKTRLQ RLNSIEASSR AKVNFLEQLY RFHKQQGNPR VSVPTINHKP
LDLWLLRKEV HRMGGYEQVR NKKWADLGRL LGYGGPGLAT QLKNSYTRVI LPYEHFCDHV
RNSLCDGCDC GFHTFCLDPP LQTIPRGQWF CHNCLFGTGD FGFDEGEEHS LSSFQARDLE
FRKLWFTSHP PVSETDVERE FWRLVQSPNE TVEVEYGADV HSTTHGAMPT SETHPLNTYS
KDPWNLNNIP ILPESLLRYI KSDISGMTVP WTYVGMVFST FCWHNEDHYT YSINYMHWGE
TKTWYSIPGD DAEKFEAAIR KEAPDLFEAQ PDLLFQLVTL MNPQRLREAG VEVYACNQRA
GEFVVTFPKA YHAGFNHGFN FNEAVNFALP DWLSLGLDCV KRYQEHRKHP VFSHDELIIS

ITQRSQAIKT AIWLNDSLQE MVERELSARQ RARLDMGEVL EEVDTEDQYQ CKICNCFCYL
SQITCSCVTC IDHADELCKC QVLRKRFSDS YLQDTQYAVA ERAGIPGAWE SKFEKLLNES
ARPPLRAMRA LLAEGDRISY PLKQLHHLRK CVTRANEWVD AANSFLVRKP NRKHDKPDRS
LSDLYALLRE VQDLGFDSPE IGQLQQLAKD AEDTKAKARE LLCEELMNKG GALNVQVDEL
TEIEKIVLRE QLLKELLEDV RRYINRARAC ALPADNRHMK SLENKLRLGT AWEDRVKAVL
DKPHRALQEL PIDPNLLDML KQTSLRGRDI ERQITAWLSG DKPRVQEVVK MVTRAEKEFD
IPVVRDMRRT VDFAVDLETR CDAVLKNRYQ HNDLFQTMRQ WKSYAKEYLS FTLPNFERLD
KQLTLHFRWL EGLPWFCHGK AILDDVVEST RPEDDQPPQD EYFTCICTNP VRPPAPGQTS
DAVQCDHCFA RFHGVCAANG GSCPFCDHQH WNGTIRKERS WHFCYLPTMM MHAPEITKNY
SEDWKQLEIV VHRVERLVGV IGQFLAYVGN QRAEYIPQVR HFMRKLYKIQ FAVSPNPETS
YGLDLAGLHR VLAGQRMKKR RRPKFTFGQD IDKDWVDGTR CICRGRTCSK TYHGGCVFMC
PLCCLRKNRT YPYSEVRVKT FSKEIIYMKL PPPYTQTLFV ELVRFTP
>Dichomitus squalens
MRRTQQDQSVIFTGETGSGKSENRRLAIKTLLELSVSQPGKKGSKLGNQVPAAEFVLETFGNSRTLFNPNASHF
GKYTELQFTRGRICGVKTLDYYLERSRVSGAPSGERNFHIFYYLIAGASAEERTHMHLSDRTQYRYLGARDAVR
FEQLKVALKNVGFSKRHVAQTCQLIAAILHLGNLEFTIDRSRNEDAAVVRNLDVLEIVSEFLGVQPSALEAALS
YKTKLVKKELCTVFLDPDGASDNRDELARMLYSLLFAWLNEHINQRLCRDDFTTFIGLFDLPGPQNMSRPNSLD
QFCINFANERLINFMQKRLFESHVQEYNNEGISRFVPHVPYFDNSECIRLLQNRPGGLIHIMDDQARRMPRKTD
HTMVEAITKRWANHSSFKSGGMDRFPTFTINHYSGPVTYSAQNFLEKNLDAINPDFVSLLRGGSMNPFVRGLFS
GKAIATQAHPRNEDTIVSAQQPVKPMRAPSTRRKNTPCVAGEFRQALDVLFETLEDTQSWYIFCINPNDSQLPN
QLEGRSVKAQVRSVGLSEIARRNVNVFEAMMTPDEYVQRYQSLLTSLGVTEGERDVVLGMSMVFLSQAAFRRLE
DDLRSKDTEEQKRNRIRDAEASNQQLPLVANASPFDDFDGRSAYTSHRESYAPSRNMFQNADGEVQEGETAEVL
KESSARRRWVALCWMLTFWCPTFLLRYVGRMKRLDVQQAWREKLALNMIIWFVCACAVFVIVFISPIICPTEHV
FNTAELNGHSNNVYYVAIRGEYGGTQADDLFPVQVSALCNGISGSVNPYVVLTTKNTDQNAQYHDFRSWTNDPRP
DWYFEQMTMLRWNYRVGFVGYSPKQIKSMASTGSSVAIYKGLSAPAYMFTSQSSTFLSFKPEKSLHFTFLTIGS
RGDVQPYIALAKGLKADGHGIEFGYVGGDPAELMRICVENGTFTVAFLKEGVQSFRGWIDDLLKTSWEACQGTD
VLIESPSAMAGYHIAEALKIPYFRAFTMTWSRTRAYPHAFAVPEHKTYVLFDQVFWRGTAGQINRWRKHTLGLP
GTSLDKMEPHRIPFLYNFSPTIVPPPLDWPEWIRITGYWFLDAADVGSKKWEPPQDLLDFIDAARKANKKIVYP
DPKAMTRCVIDAIVQSGVHAILSKGWSDRLEPEEPLPKQIYPISSIPHDWLFAGIPTIIKPFFGDQFFWGDRVE
ALGIGAAVRKLTVESLSQALREATTNQKVIDRAKLVGEQIRAENGDKPREIFEPLRLACETRNEKLMIASLDCI
SKLISYSFFVELVDLVVHTITSCHTESTPETVSLQIVKALLSLVLSSTVLVHQSSLLKAVRTVYNVFLLSADPV
NQMVAQGGLTQMVNHVFDLFIKDAFLVFRALCKLTMKPLNSESERDLKSHSMRSKLLSLHLVLMILNSHMHIFA
SPSAIIYSSSSNEATPFIQAASQYLCLCLSRNAVSPVPQVFEISVEIFWRVVSGLRTKLKKEIEVLLHEIFIPI
LEMKTSTLKQKAMIVSMLQRLCQDPEALVEIYLNYDCDSEAADNIYEHFMNIISKIGTPSFSTAALSVPGSTIG
NSEAQLRRQGLECLVAALKSLVAWGTDDPSKFESAKQKKTTLLEGIKKFNFKPKGVDFFLETGFIPSREPKDIA
RFLLETDGLSKVAIGEGDAENIAIMHAFVDMLDLSNMPFVDALRQFLQAFRLPGEAQKIDRFMLKFAERYMSGN
DTAYVLAFSTIMLNTDAHSRQVKNRMTKQGFIANNRGINDLPEEFLSAIYDDITTNEIRMKDEIATVGRDLQKE
QYMMQSNNMANEQFFSASHFIHVRPMFEVAWIPFLAGLSGPLQDTDELEIVELCLEGFKAFVSTLTKFTFLNNL
GEMKTKNMEAIKTLLDVAVTEGNQLKASWRDVLTCVSQLEHMQLISSGRSRKPPTEELANESRSTHITVAADMV
FSLSHYLSGTAIVDFVRALCDVSWEEIQSSGMSQHPRLFSLQKLVEISYYNMNRIRLEWSNMWEILGEHFNQVC
CHKNPHVGFFALDALRQLAMRFLEKEELPHFKFQKDFLRPFEYTMIHNSNPDIRDMVLQCLQQMIQARVHNLRS
GWRTMFAVFSAASKAATERIASSAFEIVTRLNKEHFPSIVRHGSFADLTVCITDFCKVSKDDPMIKYWFPVLFG
FYDVIMNGEDLEVRRLALDSLFNTLKTYGSTYPVEFWDTVCQELLFPIFAVLKSSQDLSRFSTQEDMSVWLSTT
MIQALRNLIDLYTFYFETLERFLDGLLDLLCVENDTLARIGTSCLQQLLENNVKKLSAARWERIALTFVKLFRT
TTPHQLFDESLRVERRRIFRQIIVKCVLQLLLIETTNDLLRNDEVYNTIPPEHLLRLMGVLDHSYQFARMFNDD
KELRTGLWKESSSASTLVHVLLRMYYDPRPEHQSARPQVADRLLPLGLGVLQDFNKLRLDSQAKNIAAWTPVVA
EILQGFVKFDDKAFTRYLPAIYPLATDLLSREPEIREGLREYFLRVGYIQGIQRQALILAAQAKYGSEIVSPIL
QHIMPNLSLPPGTSLVQALVQLGPDITSDADVVRSLMARFGISEVNPPTDAQVVDLVTSLARLASEGTSLPDVG
AVVRALSSFNSSLNWAAAIQAFDIPDRGVDTATLKLLIAILMNTPREQHHAVTGFWSLWSNTQYQLRLLDALLS
LPADTFNFVNLPGRKIVTVEDVAGASPTIKSLAANVQGHTWNSLDLFEVLVQAADFNSTEITNLVREMLDKAVK
ISAELVHMGLLQVPSWNDIRLEYTQRLLAMFLAGHPNHQLVFMRIWQIEPAYLTNAFRDFYDESNLNITRILDI
LDALLEVRPFKFALDEYLNLDKWLADNVTTHGADFLHAVIAFLELKMDSEKTVRVSDPPVRTMQLSPQTIAIFL
RVLRNSSSIMHENDVDYCLEVRNACLQIHPRLMDIEPGFSVVTYSTEIETEVDGIYKQMYDEQITIDDVIKLLQ
RNKASSNPRDHEIFSCMLHFLFDEYKFFQYPHRELAMTGYLFGSLIQYQLVDFIPLGIAIRYVLDALNCPPETN

LFKFGIQALSRFESRLSEWQPLCQALLKIPHLLEARPDLAVSIQRAEVSDKILFIVNNLAPSNFESKLAEMKGH
FSIEPNNHQLYLRFLDALDVQSLFRFVLHETLVKSAVLLNSEKTQQLSSERAVLKNVGSWLDRPIKHKNLSFKD
LLIEGYDNNRLVVAIPFVCKTLEPAARSKVFRPPNPWLMAVISLLTELYHFAELKLNLKFEIEMLCKALDIDLD
VVQATTILRNRPRVLGAHIENILSSILPHVTINPQLALNTNPSFKRAIQMAIDRAVREIILPVVERSVTIAGIS
TRELVAKDFVTEPNEDKLRKAGHLMAQKLAGSLALVTCKEPLKGNLGSHIRQFLSEFGFTDQQVIFLLVQENIE
LACQAIEKAAMDRAVIDVDDGFAAAYELRRRHRESRPGSNVFGSLPDPLRIKPTGVQQIQAAVYEDFFNALVRD
LEAVLIQLPASLAALPPNHEVRHLVRQILFIADRSRTPLLMSQKIVQLLYKTPSQLGREIYVALLDQLCHSFEE
VAREAITWLIYAEDERKFNVPVTVTLLRSGLVTISQEDQQLAKDPRPSLQNFAAGLIRECLAADATQAQFSYSL
EWFQQWISIYQRSHSPEKSFVPYITQLTRQGILKAEDSSFFFRVCAESSVNSYIKHVNAGEFGFAFQALDAMSR
LIVYIIKYHGDANNDQAKVHYLTKILSIFVLVLANMHEEQGFQQKPFFRFFSSLLNDLHSVESSLGSAYFQLLI
AISDTFSSLQPTYFPGFAFSWMSLISHRLFMPKLLLSDNREGWSAFYKLLLSLFKFLAPFLKTADLQPAGRDLY
RGTLRLLLVLLHDFPEFLSEYYFSLCDVIPPRCIQFRNIVLSAYPPNVVLPDPHLRDIDMGPIPPILSDFAASL
RAGDLRMYLDQFLLNRGPQTFLSSLKDRLETYNLPLINSLVMYIGVSSVAQARARSGGPLFVPTDPGVVALHYL
ATNLDVEGQHHLLSAMVLHLRYPNAHTHWFSSLMLYLFHDIQVDQFREIVTRVLLERFLVHRPHPWGALVTFIE
LLRNPKYNFWNQEFIHIAPEVTLLLENVGHRRLFLQLHFRNVSDLLTVRCDIMPLALANSAKLDAVDAYAEVVD
PREGIVDIREYDVPYYLRVAMDNEIRVGLWYAISFVQGQPAFARVKRADPVVMAFDIETTKAPLKFPDQAIDQV
MMISYMVDGQGYLITNREIVSEDIEDFEYTPKEGYEGPFIVFNEPDEAATIRRWFSHIQEVKPTVMATFNGDFF
DFPFLDARSKVHGIDMFLESGFAKDSEDEYKSRGCVHMDCFRWVKRDSYLPQGSQGLKAVTTAKLGYDPIELDP
ELMTPYAMEQPQVLAQYSVSDAVATYYLYMKYVHPFIFSLCNIIPLCPDEVLRKGTGTLCETLLMVEAIRGQII
MPNRHEESHGTMYEGHLVASETYVGGHVEALEAGVFRSDIVTHFKIEPSAVQLIDQLDAALTFCVTNYDEVKAE
IQAKLEEMRDNPQRMDSPLIYHLDVAAMYPNIMLSNRLQPDSVVDEATCAVCDFNRPGKTCDRRMTWAWRGEYF
PARRDEYNMETFPPKKPGGPQRKFVDLSEAEQTALLHKRLGDYSRRVYKKIKDTKIENREAIICQRENPFYIDT
VRRFRDRRYEYKGLHKTWKKNLDSIAEVDEAKKMILAHKCILNSFYGYVMRKGARWHSMEMAGITCLTGATIIQ
MARQLVEQIGRPLELDTDGIWCMLPGVFPENFKFKLANGKAIAFSYPCTMLNHLVHAKFTNHQYHELDPETGEY
KVHSENSIFFELDGPYKAMILPSSKEEDKLLKKRYAVFNDDGSLAELKGFEVKRRGELQLIKIFQSQIFEKFLL
GTTLEECYAAVAEVADRWLNVLVSHAEDLSDDELVELIAENRSMSKTLSEYGGQKSTSISTARRLAEFLGDQMV
KDKGLACKFIISAKPMGAPVTERAVPVAIFSAEESVKRTYLRKWLKDNSLTSFDLRSILDWDYYIERLGSVIQK
LITIPAAMQKVANPVPRIHHPDWLHRRVVAMEDKFRQHKVTDFFPGRFTLWLSINGSLTAVPLRIPREFYIHLR
TPELYQCQKVARSMPRDRPCVNLFIDITNDPNVDGVYELQNLTRAEAIGFDLQQLGRKYIFLYHACAANAPVHV
FALFIPGEAKLHIVDPATRRQPTTYHGNDATALKAISRELGLVENRSYIVVISSSKEQSYFDLHTPKLAKFPVL
SMSKAKSPHSLDFPWQTSVAQKLINRYLSLGPWLDRTIAMADYYPLLLADIEFARRLTSQDVILWWSPGDRPDL
GGIEEDNRPQEELAKTEFKSPGCYSNVCLEIAVRNLAVDAVLHSVIVNELEGSGGATAFDASMQDLTLGESNVS
PHIFSILKGLVKAWMVDKISPATIAIDHFWRWISSTASNMYDTGIHRFVHGLMRKTFIQMLAEFKRLGSHVVYA
DLSRILLVTSKPPGTAHAYATYITTAVTSHELFQHVYLHTERFYDFLIFMDEANYGAVVCENPLAIEPPEELAI
EMRWNIQSFLPPAIQADFRAVVRYFLVEFFRTAQKKVEELSACREFVSRKLTRRMLRVLFPILPGSHLPLSNPG
LELVKFTCAVFALAKEYTLEVGLLKRNLLDLVGVREFASEAAFVNPCEPLKLSAVPCRHCDSMKDFDFCRDPVL
LPWLCTKCGGEYDRAGIEFALVRLVHELERRFAQQDLRCARCSQVRADNVSRHCCSGAFVLTMNKADVRRRLRT
VVNVALAHGMGRLFEVEHLKYATLATSPHLATQKQIATIKTARNVIEYNEQYVTKRLLVNIIWAFSGDARGEWS
GWQSRVPVIEILYSWLSEHKPLMLCGPPGSGKTMTLFSALRKLPDMEVVGLNFSSYRKTPNGVILAPTQIGRWL
VVFCDEINLPATDKYGTQRVISLVECGGYWRTSDMAWVKLERIQFVGACGRVPLSHRFLRLVMVDYPGELSLKQ
IYGTYNRALLKVVPNLRAYAEPLTDAMVTLYLASQKRFTTDIQAHYVYSPRELTRWVRGIYEAIRPLEILSVEG
LVRVWAHEALRLFQDRLVTEEEKRWTDEAIDNAAMEHFPTINILFSNWTSKNKARLRVFYEEELDVPLVLFNDV
LDHVLRIDRVFRQTTLSRFVAWMNGLSIFQIKVSNKYTGEDFDLLANAEVPGLFEGFTMNPPENGLASRAATSP
ALFNRCVLDWFGDWLQTYNPPSMFPIAYRVVNALVHVHESMHHINQRLSRRQGRYPRHYLDFINHYVRLHNEKR
EELERHLHVGLDKLVEQVEELRKSLAIKRKLKQMVADQQEAEQKKAASIEIQAALKEQDKNIKQRREVVMADLA
DAEQMTKQLRDLMKRDFLSRPSYNFETVQRASKACMIAELEKSIERYKEEYAGLIRVESKVDRSMRLLESLSRT
FDTEMGTIVGDVLLSAAFLAYAEWSSHLAEAGIKFKAELSLPRLSWQSKSLPSDNLCTVTSFLDEAFLKVLESA
LRFGNTLLIQDVEHLDPILNEIRRTGGRVLIRLGNQDIDFSPSFPSVEFSPDICSRVTSQSLDQVLKVETDLMK
MQGEFRLRLRTLEKLLLQALNESSGNILDDDKVINTLETLKREAAETDVVMKEVEEVTAEYLPLAQACSAVFFI
LEQLNLVNHFYQFSFLDIFDYILHHNPNLQGVLMNDLFVIVYKRTSRALDELEFFLESGVVTFAKQPMFKPVLT
HLTEHEDEWIPFLIIIKCLRPDRLLQSVPGYDASYRVENQEGFTLADQAVALAARQGSWVLLKNVHLAPSWLGE
KKLQTLNPHRNFRLFLTMEANPSIPVNILQSRILMNEPPPGIFLLAWFHAVVQEDMAAAFTTIDTWLNSVAKGR
ANVDPAAIPWDAIRTLVKQSVYGGRVDSDFDQRIVDAFVDGLFTPHAYNVDFPDGTKLEHFMSWPSWLSLPPTA
ERRKMRMLADDDHERCREWLQQLPANFQTLQKQSAEHSDPLYRLFFREGSIGRLLSQVRKDLADVLMSSLTKGT

IPTHWRRYKVHKISNFARRLAQLDQIAQLDNGGLFFPEAYITATRAVAHRKKWSLETLRLELDIEKVNDPGLVL
EGAAWATDVLPNVGFTLPPLQGRTLDEHFYRIGTSAAQPWLTLVQDLAEEMLVFDIETMPRYHDYAVMACAATP
NAWYAWISPWLLGRVVAGHNVSFDRKRVLEEYNIQPTQTRFLDTMSLHVAVKGISSHQRPAWQKYRKSKERWED
KTSLSSLADVAKLHCGIEVDKGARDDFLSETREAILEDIHTYLDYNATDVEVTHAVFAKTLTAFLAACPSPVSF
AGILTMGSSLLTVNEEWEAYLANAERAYKELEDRIKKRLIELAQQAWKEDVWLAQLDWSPKWPRWYWELAKPKK
GTLDLTVRNRYAPLLLRLSWQGWPLFYSRGHGWTFRVRASPLAFDDNVDPQLFYKLPHKDGEDANVGSPLGKTF
MKYAQDGTLASPFEEAKDALDMNAQCSYWISSRDRVTKQMVVWQKWGIILPQVITMGTVTRRAIEKTWLTASNA
KQNRIGSELKAMVRAPPGYAIVGADVDSEELWISSVMGDAQFGLHGATALGWMTLEGTKAAGTDLHSKTASILG
ISRDQAKVFNYSRIYGAGMRHAVLLLLQGNAGMLPEKAEELARNLYASTKGQKSYGRYFDRKFWYGGTESFVFN
KLEEIALSDRPRTPALGCGITHALSKSLLPDEFGSDFMTSRINWVVQSSGVDYLHLLIVSMEHLIAKYNIQARY
LISVHDELRYLVKEEDRYRAALALQVANLWTRSLFAFKLGLDDLPQGVAFFSAVDIDHVLRKEVDMPCVTPSQP
NPIPPGESVDILKTLEKTRGGSLWQGGYSSPDCLAHRANSASWLRAQSTSNFDEVKSLAAELIDRLINTPNDDL
HEVLSQIDSWKWPRSDLNAWMKVLNKFDAILEEAIRDYDIDNLQVNVFTPLTKKTVCEILRFERLLLENSTNRK
TFNSYDRLNSLMFSSDLDVLILALNLLLRPAQQYSSQPAVSHALSISTPRLTSLAKRWPNLRDYDLNLVDLVTK
GRAQVEALPTEAREVNFVFYRHIDSKTIESKPAMDILADAIEAYHVPDEKFELLMRIRGAQAMTHAREKLVVVR
LLATAIFGHTHSDSQAQSSLFLYEPDLITHIAELLQLDRGVRKTVADVAQSTSTLPQAFVEALFSFITFLATHA
SGGSMIVGAGLIPVLIQAIENRLPNRLYVVSKTMQLLDNVLYGYTNAFTLFCNARGVDILVDRIEYEVDGKLSV
ARTAVLKHTLRSIHRMMQSSGTAEGLRGLLDSSLLKSVKKIMQHRAIFGPSALALAINIMAIFVHNEPTCLPVI
QEAGLPEVFYSVIEKGLEPVIEVIQSVPNALGALCLNQAGQDQLTARPNTIPSLFSIFTSEDHQRVLQEKENAV
LIGTSVEELIRHHPTLKEKVFVAIKSTMARIEELGTSYSVPDDIKHWYRLDNLIISYIDVFGKFLEGFFQHTDG
LNGLAKLTTLPCLPYDFANSVFVQVVRTMAEAATNETLAFLVRLVQESLAECKDFWGSLDEQPNTQFRNLITLH
TRVSLLSDIYATYSHGRATQTLLLGALHRACVWENIILKSILNAKGVKHLVGQIPLAPFFQSIVRRRGSDLAQK
QKIKEAAGIIADVLVKHLYYTSMLNAATILLVDELLVAFVRVGGQELSHAFSGLKVALQLIQPTISAKPLFDAT
QTALALTVKKDTDADYFEPHNFLVRMRAAALPVVRSIWEAEWLPSAPISVSKLVVHIVMELLNAENEPRSAAER
ALTRTRNNVNAATELLLSHPELTTAREPLKEGIGRRVLSLVDEHPSIQAFLPSAYDVQEQPLSVRCHLLALVLP
TLPKWLSAQLLVIESLLVLGDSPRSVTLPIAQGPRYAEARNTLFDFSMRLLGVPTLPKDELISVLRLIVLLTRD
HNIAEAFMERNGVAALGIQSYIALIVRHIVENQITLQHVMRQEVKRYFSHPRNVDIGSFVSGCSSTALRDPEAF
VKVAEEMCTLSQPETLIHYLIGELMKTVKYPCFIMQVLTELLFSYESCKIAFLSYTPKKHRTAAIQFLLSDLMT
FGTINARKQITLCNWAMSVIVALCVDTVKDVPTDLVSVRKFVLEAISRALKDLPSSDSPELRYSRLLALSDLCH
RLLTVRFEVPTHIAKVMLEKNFVATLTNVLSEVDLNYPNIRNVVSSVLRPLEYLTKIAMKMSREEASQLYRNSS
LGLFAENVTHPLLLDRTEGRSLEPLLTIQRWSEEVKMLHGKFEQARLSRLHNHVVLALLPAAAERVTVMIHGAP
VDITDTGIDPTFLEALPDEMREEVLNQHVRDQRAAQLERPADSAISAEFLDALPPDIRAEIIQQEAAEPLDIDP
ADFIASLDPQLRQVVLMDSDDVFIQSLPKAPQSREAIQLLDKSALAALVRLLFYPHKNLLYKVLVNLCENAKSR
TDLFNLLLSILQDGSGDLASIDKSFAQMSVRPELIVQRCLEALTYIVSSNELSSLFFLTEHELPIGLRRSKKGK
GKEKQPQTHYPIVLLLGLLDRPSILRTPSIVESVVGLLATVTRPLASLKKVLLANSPHIPHHVLRLIVNVLTAG
ECSGRTFMQSLALIQHLSYIPDAREVIASELRSRAQEFGQSLFAALDELAVALAAMFSSASSDQAKLLRVLKTI
DYMYSIYETFRFSPLWKRLGDCLSIIEEKPEMEHIATVLLPLIESLMVVCKYVGPKESMEDLFVTFTDAHRKVL
NLMVRNNPSLMSGSFSLLVHNPRVLDFDNKRNYFNQQLHRRREHHSTLQLNVRRQRVFEDSFQYLQRKTGEQIK
YGKLSVRFYDEEGVDAGGVTREWFQILARQMFDPNYALFQPCAADKLTYQPNRASWVNPEHLSFFKFVGRVIGK
AIYDGRLLDAYFARSLYRQLLGKQVDYKDVEWVDPEYYNSLCWILENDPSPLDLTFSFGVTKLVELKEGGASIP
VTQENRKEFVQLSANYRLYSSIKDQIESLLAGFYEIIPKDLVSIFNEQELELLISGTPDIDVDEWRSATEYNGY
TSSDPVIVWFWRALKSFNREERAKVLSFATGTSRVPLGGFVELQGVQGTQRFSIHKAYGDTDRLPQAHTCFNQI
DLPQYSSYEMLRQQLLLAINEGGEGFGFAKSSATSATRKKHARKLRLLAKKDATTKRRALEDMQHIPALFLHPS
RRIRLQAIALHSSFLGSWCLAVHDVDRQVSSFARESWFVQRTLLDPSGVYLYINPEENEQDRKARLRVGAFGAT
EWGFEQPVVRRSAWSLLQTLLSSAILRSAWVEPDPNVRAAMWQPLLVFLKEHPNAWAYTEFLQFLATGSPAQGY
PTVLIILSTIPSSIFVSFWAAVDARVLAFFASVLESLVFVVRRPYAFLLAFLLPPSRAELDSMLEALPSTPLDP
SLAVVDPLDTQGLAKYARTVHGLLERQTAKESMWALHHILALDTVRESRVLHMILRHALSTATKADSEQWMLVA
RKIEKLAPHTSLAIIFSITRYAPEPTRLERFRNELAAGTLGIPGLWLLRNLAASAPDPDSDVVFLPQQRAINLI
KACQQWITSDLEEDVQSEMTLVFASLVPILSNVPGGHWDLVFDVVENNLENSTLDDPSTYVTLSRTLQLFMIIE
DFASSNKALKAWDERRSAILTLIRDLSTPLSMCRELALQIEELVIEAAVDTASMKVKSGYIDQLKDLDLVGSRL
LPVVFGLLDLYKAFKLEIWDVDEFYLDYYTTISLRLLTAHLYYRALLLVPSLIRAWLNDCRDRQLHTTVTAYTS
RHFSPAIIRTELERVKDPVTEDRWRAWVLGVRQIRSGSIVDGLSFFLKNVTSHFAGIAECAICYSIVKPCKTCK
NRFHAACLYLPVAPTLDLS SVKTPNTRPF GLTDCPTFHP TPEQFKDPMA YIASISDTGK KYGMCKIVPP
MGWNMPFVTD TEFRFKTRLQ RLNSIEASSR AKVNFLEQLY RFHKQQGNPR VSVPTINHKP

LDLWLLRKEV HKLGGFDAVR DKKWADLGRL LGYTGPGLAT QIRNSYNRVI LPYEQFCERV
RNSICDGCDC GFHMFCLDPP LANIPRGQWF CHSCLFGTGD FGFDEGQEHS LSSFQARDRE
FRRLWFLSHG PVTEDDVELE FWRLVQTPTE TVEVEYGADV HSTTHGGMPT LETHPLDPYS
KDPWNLNNIP ILPQSLLRYI KSDISGMTVP WTYVGMIFST FCWHNEDHYT YSINYMHWGE
TKTWYSIPGS SAEKFEAAIK KEAPDLFEAQ PDLLFQLVTL MNPQRLKEAG VEVHACNQRA
GEFVVTFPKA YHAGFNHGLN FNEAVNFALP EWLPLGLDCV KRYQEHRKMP VFSHDELLIT
ITQQSHSIQT AMWLNDSLQE MTDREMDART RARLQMGEVL EETDGDDQYQ CATCKVFCYL
SQITCPCIVC IDHVDQLCKC HVLRKRFSDT ELQDIQAKVS ERAAIPGMWR NKLKKLLDES
PCPPLKSLKA IFTEGERIQH PLAELNSLRK CVNKANEWLE AANAIIIRKP TRKRDKPERS
LDDLYALLSE VANLGFDAPE IAQLRNIAQE AEETRKKARL LLCRRLILQS ATLNVTVDEI
VEVDKIVLRE QLLKDLLEDV RQLVGRADAC SLPMEHSQMQ RLQALYQAGT TWEDRAQRLL
EKSDRTLEEL PVDPELLERI IDLRKKGREY EKQAKIWLLA DKPKVQDVVK LVADAQRDFA
IPAVQDLQRT VAWAQDLETR SEAVLNATYS GKDIFETMQT WRDYAKKHLT FSLTNFSKLE
SQLESHNRWI QSLPWYCHGQ PILDDVVEAT RPEDDLPPSD EYFTCICTTP VRPPALGQVS
DAVQCDHCFA RFHGVCAANG GSCPFCDHHH WNGTIHKERN WHFCYLPTIL MHAPDITKHY
SEEWKQLEII VHRVDRLCGV IGQFVSFLGN QRAEYIPQVR HYMRKLYKIQ FAVSPNPEVS
FGLDLASLHR VLAGQRMKKR RRPKFVFGQD QDKDWLDGTR CICRGRTCGK LYHGGCVFMC
PLCCVRKNRV YPYSEVRVKT FSKDIIYMRL PPPYTQTLFV DLLRFTP
>Trametis versicolor
MRRTAQDQSMLFTGETGSGKSENRRLAIKTLLELSVSQPGKKGSKLGNQVPAAEFVLEAFGNSRTLFNPNASHF
GKYTELQFTRGRISGVKTLDYYLERNRVAGAPSGERNFHIFYYLVAGASPEERQHMHLSDKTQYRYLGARDAVR
FDQLKVALKNVGFSKRHVAQTCQLIAAILHLGNLEFTVDRHRNEDAAVVRNLDVLEIVSEFLGVQAAALEATLS
YKTKLVKKELCTVFLDPDGASDNRDELAKTLYSLLFAWLNEHINQRLCRDDFATFIGLFDLPGPQNMSRPNSLD
QFCVNFANERLLNFIQKRLFEAHVQEYNTEGISRFVPHVPYFDNSECLRLLQHRPGGLIHIMDDQGRRMPRKTD
HTMVEAFTKRWGNHSSFKAGGMDRFPTFTVNHYSGPVTYSASGFLEKNLDAINPDFVSLLRGGSMNPFVRGLFS
AKAIATQAHPRDEDTIVAAQQPVKPMRAPSTRRKNTPCVAGEFRQALDVLFETLEDTQSWYVFCINPNDSQLPN
QMEGRSVKAQVRSVGLSEIARRSVNVFEAMMTPDEYTQRYGGLLQAVGVSEGERDVVLGLSMVFLSQAAFHRLE
DDLRSKDTEEQKRNRMRDAEASNQALPLVQHASPFDDFDGRSAYTSQRESYAPSRNMFQNTDGEIQEGETTEVV
KESSARRRWVLLCWMLTWFIPNFALTKIGRMKRDDVRQAWREKLAINMIIWFVCGCAIFVIAIISPLICPREHI
FNSQELAEHSNNVYTSIRGEYGGASADNVFPVQVSALCNGISGTVSPYVVLSSSNTDVNAQYHDFRSWTTDPRP
DWYFESMTQMRWQARVGYVAYSSKQLKSMASSGNSVGIYRGFAAPAHMFTSQSSTFLSFKPDKSLHFTMLTIGS
RGDVQPYIALAKGLMADGHGIEFGYVGGDPAELMRICVENGTFTVAFLKEGVAKFRGWIDDLLKTSWEACQGTD
VLIESPSAMGGYHIAEALKIPYFRAFTMTWTRTRAYPHAFAVPEHKTYVLFDQVFWRGTAGQINRWRRNTLGLP
GTSLDKMDPHKIPFLYNFSPIIVPQPLDWPEWIRVTGYWFLDDADVGSKKWEPPQSLLDFMAEARKAKKKIVYP
DPKTMTRCVIDAIVESGVYAIMSKGWSDRLEPEEPLPKQIYPISSIPHDWLFAGIPTIIKPFFGDQFFWADRVE
ALGVGAAVRKLTVDVLAQALRDATTNQKQIDRAKAVGEQIRAENGDRPREIFEPLRLACETRNEKLMIASLDCI
SKLISYSFFAELVDLVVHTITSCHTENTPETVSLQIVKALLALVLSPTILVHQSSLLKAVRTVYNIFLLSLDAV
NQMVAQGGLTQMVNHVFDMFIKDAFLVFRALCKLTMKPLNSESERDLKSHAMRSKLLSLHLVLMILNSHMPIFV
SPSAIIYSSSSHEATPFIQAASQYLCLSLSRNAVSPVPQVFEISVEIFWRVVAGLRTKLKKEIEVLLHEIFIPI
LEMKTSTLKQKAVILSMLQRLCQEPQALVEIYLNYDCDGEAVDNIYEHLMNIISKIGTPSFSTASLSVPGSTIG
NSEAQLRRQGLECLVAVLKSLVSWGTDDPTKFESAKQKKTTLLEGIKKFNFKPKGIEFLIETGFIASREPKDIA
RFLLETDGLNKAAIGEGDEENITIMHAFVDTMDLGNMPFVTALRTFLQAFRLPGEAQKIDRYMLKFAERYIATN
DTAYVLAYSTILLNTDAHNPQVKNRMTKQGFIANNRGINDLPEDLLNAIYDEIVSNEIRMKDEVANVGRDFQKE
AYVMQSNNMASEQFFSASHFVHVRPMFEVAWIPFLAGISGPLQDTDDIEVVELCLEGFKAFVSTLAKFTFLNNL
GEMKTKNMEAIKTLLDVAVTEGNHLKASWREVLTCVSQLEHMQLLSSGRVRKPPTEELANESRSTHITVAADMV
FSLSHYLSGTAIVDFVRALCDVSWEEIQSSGLSQHPRLFSLQKLVEISYYNMNRIRLEWSNMWDILGEHFNQVC
CHKNPHVGFFALDALRQLAMRFLEKEELAHFKFQKDFLRPFEYTMIHNSNPDVRDMVLQCLQQMIQARVHNLRS
GWRTMFAVFSAASKAATERIANSAFEIVVRLNKEHFSSIVRHGSFADLTVCITDFCKVSKDDPMIKYWFPVLFG
FYDVIMNGEDLEVRRLALDSLFSTLKKYGSTYPLEFWDTVCQELLFPMFAVLKSSQDLSRFSTQEDMSVWLSTT
MIQALRNLIDLYTFYFETLERFLDGLLDLLCVENDTLARIGTSCLQQLLESNVKKLSPARWERVATTFVKLFRT
TTPHQLFDESLRVERRRIFRQIIVKCVLQLLLIETTNDLLRNDEVYNTIPPEHLLRLMGILDHSYQFARMFNED
KELRTGLWKESSSASTLVHVLLRMYYDPRPDHQAARPQVADRLMPLGLGVLQDFNKLRLDTQAKNIAAWTPVVA
EILQGFVRFDDKAFTRYLPAVYPLATDLLSREPEIREGLREYFLRVGYIQGIQRQALILAAQAKYGSEIVSPIL
QRILPNLSLPNGTSLVQALVQLGPEATADPDVVRALFVRFGLTENNPPTDAQLVEVITSLARLASEGTHLPDVG

SVVRALNSLNGELNWAAAIKAFDMPDRGVDTATLKLLIAILMNAPRDNQHAVTGFWQLWSNTLYQLRLLDALLS
LPADTFNFVNLPGRKIVTVDDVAGASPTIKSLAANVQGHTWNSLDLFEVLVQAADLNNPDVENTVREMLDKAVK
ISAELVHMGLLQVPPWNDIRLDYTQRLLAMFLAGHPNHQLVFMRIWQIEPVYLTNAFRDFYEESPLNITRILDI
LDSLLEVRPFTFALDEYLNLDKWLADNVSAHGADFLHAVIAFLDLKMESEKAQRVSDPPVRTMQLSPQTITIFL
RVLRNSSAIMHESDIDYCLEVRNACLQIHPRLMDTEPGFAVVSYSAEIETEVDGIYKQMYDEQITIDDVIKLLQ
RNKTSSSPRDHEIFSCMLHFLFDEYKFFQYPPRELAMTGYLFGSLIQFQLVDFIPLGIAIRYVLDALGCPPETN
LFKFGLQALSRFESRLAEWQPLCQALLKIPHLLEARPELAVIIQRAEVSDKILFIVNNLAPSNFDAKLLEMKGQ
FSTEPNNHQLYLRFLEALNLQPLSHFILHETLVKSAILLNSEKTMQLGSERAILKNVASWLDRPIKHKQLSFKD
LLIEGYDNGRLIVAIPFVCKTLEPAARSKVFRPPNPWLMAVVSLLTELYHFAELKLNLKFEIEMLCKALDVDLD
IMQATTILRNRPRVLGAHIENILSSVLPQVTFSPQLALNTNPSFKRAVHMAIDRAVREIILPVVERSVTIAGIS
TRELVAKDFVTESSEDKMRRAGHLMAQKLAGSLALVTCKEPLKGNLGSHMRHFLTEFGFTEQQVIFLLVQDNIE
LACSAIEKAAMDRAVIDVDEGFAAAYELRRRHREQRPGSPIVTSLPDPLRIKASGVQPSQMRVYEEFFNALVRD
LEAVLLQLPTSLAALPPNHEVRHLVRQILFLADRTRTPLLMSQKIVQLLYKTPSQLGREIYVALLDQLCHSFED
VAKEAITWLVYAEDERKFNVPVTVTLLRSGLVTIVQEDQQLAKDPRPSLQTFAAGLIREYLAADSTQGQFSYSM
EWFQQWVSIYQRSHSPEKSFVPYITQLTRQGILKAEDSSFFFRVCAESSVNSYIKHVNAGEFGFAFQALDAMSR
LIVYIIKYHGDANNDQAKVHYLTKILSIFVLVLANMHEEQGFQQKPFFRFFSSLLNDLHSIESSLGTAYFQLLL
AIGDTFSSLQPTYFPGFAFSWMSLISHRLFMPKLLLSDGREGWSAFYKLLLSLFKFLGPFLKSADLQAAGRDLY
RGTLRLLLVLLHDFPEFLAEYYFSLCDVIPPRCIQLRNIILSAYPPNIILPDPHLPDIDMGPIPPILSDFAAGL
RAGDLRLYLDQFLLNRNSQTFLSSLKDRLEPYNLSLINSLVMYIGVSSVAQARARSAGPLFVPTDPGAMALHYL
ATNLDVEGQYHLLNAMVLHLRYPNAHTHWFTLLMLHLFHEIEADSFREVMTRVLLERFLVHRPHPWGALVTFIE
LLRNPKYQFRQQDFVNVAPEVTLLLENVGHTRLYLQLHFRNVSDLLTVRRDIMPLALANSAKLDAVDAYAEVVD
PREGIIDIREYDVPYYLRVAMDNDIRVGLWYAVSFTAGQPGFERVKRADPVVMAYDIETTKAPLKFPDQAIDQV
MMISYMIDGQGFLITNREIVSEDIDDFEYTPKEGYEGPFTVFNEPDEAAVIRRWFSHIQDVKPTVMATFNGDFF
DFPFVAARAKVHGIDMFLEIGFAIDSEDEYKCRSCVHMDCFRWVKRDSYLPQGSQGLKAVTTAKLGYNPIELDP
ELMTPYAIEHPQILAQYSVSDAVATYYLYMKYLHPFIFSLCNIIPLCPDEVLRKGTGTLCETLLMVEAFRGQII
MPNRHEESHGNMYDGHLLASETYVGGHVEALEAGVFRSDIETHFKIEPSAVQLIDDLDAALTFCVTNYDEVKAA
VQAKLEVMRDNPQRMDQPLIYHLDVAAMYPNIMLSNRLQPDSVVNEATCAVCDFNRPGKECDRKLQWAWRGEFF
PARRDEFNMESFPPKKPGGPQRKFNDLPQAEQTALLHKRLGDYSRRVYKKIKDTKIETREAIICQRENPFYIDT
VRRFRDRRYEYKGLHKTWKKNLDSIAEVDEAKKMILAHKCILNSFYGYVMRKGARWHSMEMAGITCLTGAKIIQ
MARQLVEQIGRPLELDTDGIWCMLPGVFPENFKFKLANGKALAFSYPCTMLNHLVHAEFTNHQYHELDPEAGEY
MVHSENSIFFELDGPYKAMILPSSKEEDKLLKKRYAVFNDDGSLAELKGFEVKRRGELQLIKIFQSQIFEKFLL
GSTLDECYAAVAQVADRWLDVLFSHADDLSDEELVELIAENRSMSKTLAEYGGQKSTSISTARRLAEFLGDQMV
KDKGLACKFIISSKPMGAPVTERAVPVAIFSAEESVKRTYLRKWLKDNSLTNFELRSILDWDYYIERLGSVIQK
LITIPAAMQKVTNPVPRIRHPDWLFRRVAALDDKFHQHKVTDFFPGRFTLWLSVDGTLSAVPLRIPREFYMHLR
TPEMYQCEKVVRSLPRDRQCVNLFIDITNDPNVDGVYEFQTLNRAETTGFDLQQLSRKYIFLYHACSNNAPVHV
FALFIPGEVKLHIVDPATRRQPSTYHGNDVTALKAISRELGLVENRSFIVVVSSSKEQSYFDLHTPKLSKFPVL
SMNKTKSAHSLDFPWQTSVAQKLVSRYLSLGKWLDTAMAQADYYPLLLADVEFARRLMSQDVVLWWSPGDRPDL
GGIEEDNRPQEELAKTEFTSPGCYSNVCLEITVRNLAVDAVLHSVIVNELEGSGGATAFDAAMQDLTLGESNVS
PLAFSILKSLVKTWMLDKISPATTVLDHFWRWISSTASHMYDTSIHRFIHGLMRKTFIQMLAEFKRLGSHVVYA
DLSRILLVTSKPPGTAHAYATYITTAVTSHELFQHVYLHTERFYDFLIFMDEANYGAVVCENPLAIEPPEELAI
EMRWNIQSFLPPAIQSDFRALVRYFLVEFFRTSQKKANEISACREFVQRKLIRRTLRMLFPVLPGSHLHLTNPG
LEFAKFACAVFALAKEHTLEVGLLKRSLLELVGVREFAPEASFQNPCEPLKLASVPCQHCDAMKDFDFCRDPAL
LPWLCARCDCEYDRTAIEFALVRMVGDAERRFAQQDLRCSRCAQLRSDNVAKHCCSGNYQLTMNKAEVKRRLRT
IVNVALAHNMGRLFEVEHLKYATLATSPHLVTQKEIALIKTVRNVIEYNEQYATKRLLVSLIWAFSGDARGEWT
GWQGRVPVIDILYSWLSEHKPLMLCGPPGSGKTMTLFSALRKLPDMEVVGLNFSSYRKTPNGVILAPVQIGRWL
VVFCDEINLPATDKYGTQRVISLVECGGYWRTTDMAWVKLERIQFVGACGRVPLSHRFLRLVMVDYPGELSLKQ
IYGTYNRALLKVVPNLRAYSEPLTDAMVALYLSSQKRFTTDVQAHYVYSPRELTRWVRGIYEAIRPLEILSVEG
LVRVWAHEALRLFQDRLVTEEEKRWTDEAIDNAAMEHFPTINILFSNWTSKDKARLRVFYEEELDVPLVLFNDV
LDHVLRIDRVFRQTTLSRFVAWMNGLSIFQIKVHNKYTGDDFDLLANAEVPGLFEGFTMNPPENGLASRAATSP
ALFNRCVLDWFGDWLPSYNPPALFPISYRVVNALVHVHESMHQINQRLSRRQGRYPRHYLDFINHYVRLHNEKR
EELERHLHVGLDKLVEQVEELRKSLAIKRKLKRMVADQQEAEQKKAASIEIQAALVEQDKHIKERREVVMADLA
DAEQMTKQLRDVMKRDFLSRPSFNFETVQRASKACMIAELEASIERYKEEYAGLIRVESKVDRSMKLLESLSRT
FDTEMGTIVGDVLLSAAFLAYAEWSSHLAEAGVQFKAELSLPRLSWQSKSLPSDNLCTVTSFLDEAFLKVLESA
LRFGNTLLIQDVEHLDPILNEIRRTGGRVLIRLGSQDIDFSPSFPSVEFSPDICSRVTSQSLDQVLKVETDLMK

MQGEFRLRLRTLEKLLLQALNESSGNILDDDKVIDTLETLKREAAETDLVMKEVEEVTAEYLPIAQACSAVFFI
LEQLNLVNHFYQFSFLDIFDYILHHNPNLRSVLMNDLFLVVYKRTSRALDELEFFLDSGLVTFAKHPLFKPVLT
HLTEHEDEWVGFLTVVKCLRPDRVLQSVPGYDASYRVENQEGFTLADQAVAMAARQGSWVLLKNVHLAPSWLGE
KKLQTLNPHRNFRLFLTMEANPSIPVNILQSRILMNEPPPGIFLLAWFHAVVQEDMAAAFNTIDTWLNSVAKGR
ANVDPASIPWDAIRTLIKQSVYGGRVDSDFDQKIIDAFVDGLFTSAAYNVDFPEGTKLDHFLSWPSWLSLPPTA
ERRKMRMLADDDNERCREWLEQLPANFQTLQKQSAEHSDPLYRLFFREGSIGKLLTQVRRDLADVLMSSLTKGT
IPDHWRRYKVHKISNFARRLAQLDQIAGLDNGGLFFPEAYITATRAVAHRKRWSLETLRLQLDIEKVNDPGLVL
EGAAWASDVLPNIEFTLPPLQGRTLDEHFYRIGSSAAQPWLTLAQDLAEEMLTFDVETLPAYTPFAVMACAASK
HAWYSWISPWLLGRVVVGHNVSYDRGRVLEEYNINGTQTRFIDTMALHIAVKGISSHQRPAWIKHRKSKKRWED
LTSANSLADVAQLHCDITMDKEVRNDFMTSTPEEIRDGIHTYLDYCSTDVAVTHAVFAKTLPAFLTACPNPVSF
AGVLTMGSSLLTVNEEWDKYIANAERTYRELEEKVKMRLVDLAHEAWKDDAWLSQLDWTPKWPKWYWDLTKPKK
GTLDLTVRNRISPILLRLSWLGWPLFHSREHGWTFRVRATPLVLADDADADLFYKLPHKDGEKANVGSPLAKTF
MKYSQDGTLASPFEEAKGALDMNAQCSYWISARDRVMNQVVVWQKWGVILPQVITMGTVTRRAIEKTWLTASNA
KKNRVGSELKAMVRAPPGYALVGADVDSEELWISSVMGDAQFGLHGATAIGWMTLEGTKAAGTDLHSKTASILG
ISRDQAKVFNYSRIYGAGMRHAVLLLLQSNAGMLPEQAQKLAENLYASTKGKNTHGRLFGRKFWYGGTESFVFN
KLEEIALSDRPQTPALGCGITYALSKEYLPVGFGSDYMPSRINWVVQSSGVDYLHLLIVSMEHLIAKYNIQARY
LISVHDELRYLVKDEDRYRAALALQIANLWTRSYFAYKLGMDDLPQGVAFFSSVDVDRVLRKEVDMPCVTPSQP
VPIPAGESLDILQTLEKTHGGSLWADGYSPPDCLAHRAHSAPWLRAQATSDLDEIKRLAVEFINKLADTPNEDL
PEVLSQIDSWKWPRSDLNAWIKVLNKFDEILEEAIRDYDIDNLQVNVFTPLTKKTICEILRFERLLLENSTNRK
TFNSYDRLNNLMFSSDLDVLILALNLLLRPAQQYSSQPAVSHALSISTPRLTSLAKRWPNLRDYDINLVDLVSK
GKAQIEALPTEAREVNFVFYRHIDSTTIQSKPAMDILADAIEAHNVPDEKFELLMRIRGAQVMTNEREKLVIVR
LLATAIFGHTHSDSQAQSSLFLYEPDLIAHIAELLQLDRGVRKTVSDVAQPTSTLPQAFVEALFSFITFLATHS
SGGNMIVGAGLVPVLIQAIENRLPNRLYLVSKTMQLLDNVLYGYNNAFQLFCNAHGVDILVDRIQYEVDGKLSV
ARTAVLKHTMRSMHRMMQSSGTSEGLRGLLDSSLLKSVKKVLQNRAIFGPSVLGLAINIMSIFVHNEPTCLPVI
QETGLPEAFYGIVEKGLEPVIEIIQSVPNAIGALCLNQAGQDQLAARPNIIPSIFYIFTSEEHQRVLQEKENAV
LIGTSLEELIRHHPSLKDKVFIALKATMAKIEELGNSYTPPNEIKHLYRLDNIIISYIDVFGKFLEGFFQHAEG
LDHLARLTALPCLPYDFANSVLVQVIRTMAEAATTETLAFLLKLVQDSLEECKDFWESMEERPNERFRKLVTLH
VRTSLLSDIYATYSHGRASATLLLGSLHRSCIWENIVLKATLNAAGLRHLVSQMPLSPFFQSVVRRRSSDVAQK
QKIREAAGILADVLVQHLYYTTMLSTTTVLLVDELLAAFARVGGQELTHAYGGLKVSLVLLQPLVSSRPLFDST
QTPMAITTKKDTDAEYFEPHNFLVRMRLAALPLIRTIWESSWLTAAPISVSKTVVQVVMEILNAENEPRSAAER
ALVRTRNNVAAATELLLAHPELNAAREPFKEELGRRVLSLVDEHPSIHAFSPKAYDVQEQPLFVRCHLLALVLP
VIPKWLPAQLLVIECLLVIGEAPRSITLPIAQGTRHAEARNALFEFCMRLLAVPTLPKDELISALRILVLLTRD
SSVAEGFVKRGGVAMLGIQSYIAIIMRHIVENPTILQHVMRQQLRQFFSHPRVVDVGTFVSGCNSAALRDPEAF
VKVTEDLCQISNPETLIHYLIGELTRTVKYPCFIMQVITELLFSYDACKIAFLSYSPKKHRTAAIQFLLSDLLT
FGTINARKQITLCNWAMSVIVALCVDTTKDVPTELVSIRKFVLEAISRALKDLPTSENTESRYSRLLALSDLCH
RLLTVRFEVPTHIAKVMLEKNFVSTLTNVLAEVDLNYPNIRNVVSSVLRPLEYLTKVAMKMSRDAATDLYRNSS
LGLFAENVTHPLLLDRTEGRSLEPLLTTQRWGEEAKMLHGRFEQTRLSKLQNHVIIALLPAAIERIMVTIHGNP
VDITDTGIDPTFLEALPDDMREEVLNQHVRDRRAAQVERPADSQISAEFLEALPPDIRAEIIQQEAVEGVDIDP
ATFIASLDPQLRQVVLMDSDDVFIQSLPKALPSRDAIQLLDKSALAALLRLLFYPHKNLLYKVLVNLCENAKSR
TDLFNLLLSILQDGSGDLASIDKSFAQMSVRPELIVQRCLEALTYVVSSNELSSLFFLTEHELPFGLRRSKKGK
GKEKPPQTHFPIVLLLSLLDRPSILRTPSIVESVVALLATVTRPLAGLKKVLLANPPHIPHHVLRLIVNVLTAG
ECSGRTFAQSLSLIQHLSFIPDAREVIASELRARAQEFGQSLHSALAELSVALASKFSTASSDQAKLLRVLKTI
DYMYSIYESFRFSSLWQRLGDCLSIIEEKPEMEHIATVLLPLIESLMVVCKYVGPKESMEDLFVSFTDAHRKVL
NLMVRNNPSLMSGSFSLLVHNPRVLDFDNKRNYFNQQLHRRREHHGTLQLNVRRQRVFEDSFQYLQRKTGDQIK
YGKLSVRFYDEEGVDAGGVTREWFQILARQMFDPNYALFQPCAVDKLTYQPNRASWVNPEHLSFFKFVGRVIGK
AIYDGRLLDAYFARSIYKQLLGKPVDYKDVEWVDPEYYNSLVWILENDPSPLDLTFSFGVTKLVELKEGGATIP
VTQENKKEFVQLSANYRLYSSIKEQIEALLTGFYEIIPKDLGSIFNERELELLISGTPDIDVDEWRSATEYNGY
TGSDPVIVWWWRALKSFTREERAKVLSFATGTSRVPLGGFVDLQGVQGTQRFSIHKAYGDTDRLPQAHTCFNQI
DLPQYSSYEMLRQQVLLAINEGGEGFGFAKSSASSGTRKKHARRLRLLSKKDATTKRRALEELQHLPTLFLHPS
RRLRLLAIGLHTSILGSWCLAAHDVDRQVSSHARESWFVQRTLLDPGGVYLYLNPEEDELDRKARLRVGAFGAT
KWGFGQPVVRRSAWSLLQTLLSSAVLRSAWVEPDTIVRAAMWQPLLMFLKDYPNAWAYTEFLQFLATGSPAQGY
PTVLVILSTIPPSIFTSFWAAVDARVLAFLSSVSESLTFFVRRPYLFLYAFLLPPSQEELLVMLDDLPSTPLDP
SLGAVDPLDSAGFNKYARVVNGLLDRQMSKENVWALRHLFALDNVRTSRILHMILQHAFRNATKADAELWMGAV
RQTEKLAPHTALAIVFSVTRYAPEPPRLDRYRNERAAGALGIPGLWLLRNLAAAAPDPESDVIFLPQLRAVNLI

KACQQWITSDLDEEVQSEMTLLFAHLAPILQGVPGAHWDLIFDVVENNLEDASLDDPSTLVTLSRTLRLFIIIE
DLVSTNKALRAWEERRAANLTLIRDLSTPLSVCRELALQIETLVIEAAVDTASLKVKSGYIDQIRDLDLVAAKL
LPTLFSLLDLYKAFKLDIWDIDEFYLDYYTSISLRLLAAHVYYRTLLLLPSLIRTWLTDCRDRQLSTAVASYTS
KHFSPAIIRTELARVKDPVTDDRWRAWELGVQQIRSGSIVDGLSFFLKNVTSHFEGVAECAICYSVVKPCKTCK
NRFHAACLYLSVPQMLDLS SVKTADARPF GLTDCPTFHP TPEQWKDPMA YIASIADAGK RYGMCKIVPP
AGWNMPFVTD TEFRFKTRLQ RLNSIEASSR AKVNFLEQLY RFHKQQGNPR VSVPTINHKP
LDLWLLRKEV HKLGGYDAVK DKKWADLGRL LGYTGPGLAT QLRNSFSRVI LPYEQFCDRV
RSSICDGCDC GFHMFCLDPP LVNIPRGQWF CHTCLFGTGD FGFDEGEEHS LSSFQARDLE
FRRLWFLSHP PVTEDDVEKE FWRLVQSQNE TVEVEYGADV HSTTHGGMPT LETHPLDPYS
KDPWNLNNIP ILPQSLLRYI KSDISGMTVP WTYVGMIFST FCWHNEDHYT YSINYMHWGE
TKTWYSIPGC DAGKFEAAIR KEAPDLFEAQ PDLLFQLVTL MNPQRLKEAG VDVYACNQRA
GEFTVTFPKA YHAGFNHGLN FNEAVNFALP DWLPLGLDCV KRYQEHRKMP VFSHDELLIT
VTQQSQSIQT ALWLNDPLQE MTDREMDART RARRQMNEVL EETDGDDQYQ CSVCKVFCYL
SQITCTCIAC IDHVDQLCKC HVLRKRFSDT ELQDIQARVS ERAAVPTVWR GKLKRLLDDS
PRPPLKGLRN LFTEGERIQF PLAELNSLRK CVNKANEWLE AANGILIRKP TRKRDKPDRT
LEDLYALLSE VENLGFDAPE IAQLRTIAAE AEDTRRKARA LLCEHLILHA QTLNVLVDEI
VEVEKIVLRE QLVKELLEDV RQLMARAHAC NLPAENMYMQ RLETLLRAGS NWEEKAKALL
DKQQRTLEDL PVDPDLLERI LDLRKKGKDF EKQAKIWLLS EKPKVQEVVR LVTEHDKDFE
IPAVEDLKRT VDFAQDLEAR CEAVMRGIYN PPDVFHTMLQ WRKYAKEHLT FSLPNFETLD
KQLTTHFRWL EGLPWWCHGQ PILDDVVEST RPEDDLPPND EYFTCICTTP VRPPAHGTVS
DAVQCDHCFA RFHGICAANG GSCPFCDHHH WNGTIHKERN WHFFFLPQLL LQAPDVTKNY
SEEWKQLEII VHRVDRLCAV IGQFLSFAAN HRVDYIPQVR HYMRKLYKIQ FNVASNPDAN
FGLDLAGLHR ILAGQRMKKR RRPKFVFGQD VDKDWNDGTR CICRGRTCNK FYHGGCVFMC
PLCCVRKNRV YPYSEVRVKT FSKDIVYMRL PPPYTQTLFV ELIRFTP
>Schizophyllum commune
MRRTNQDQSILLSGETASGKSENRRLAIKSIIELSVSSPGKKGSKLASQIPAAEFVLETFGNARTLFNPNASRF
GKYTELQFSRGRLCGIKTLDYYLERSRVSAVPNGERNFHIFYYLVAGAAPEERQHMHLTDKATYRYLGASDANR
FDQLKVALKTIGLSKRHVAQTCQLIAAILHLGNLEFTIDRHRNEDAAVVRNVDELEIVAEFLGVTPATLESALS
YKTKLVKKELCTVFLDPDGASDNRDDLAKTLYSLLFAWLNEHINQRLCKDDFATFIGLFDLPGPQNMSRSNSLD
QFVVNFANERLHNFIQKRLFENHVNEYAAEGIAHYVPQVPYFDNSECVRLLQNKPGGLIHIMDDQSRRLPKKTD
QTMVEAFQKRWGNHTSFRAGGLDRFPTFTVSHFNGPVTYSAESFLERNLDALNPDFVSLLRGGSDNPFVKGLFS
AKAIATQAHPRNEETIVAAQQVVKPMRAPSTRRKNTPCVVGEFRSALDTLFETMEDTQSWYVFCINPNDSQLPN
QLEGRSVKGQVRSAGLTEVARRNTVVFEVGMTPEEFCERYKDDLESLGVTEGDTDIVLGKHKVFLSHGAFHRFE
DQLRSRDTEEQKRNRMRDAEASNQRLPLVANAAGFDDFDGRSRFTGRDESYAPSRNMFGNADGEIQDGEVTEII
KESSARRRWVMLCWILTFWVPTPCLAWVGRMKRPDVQQAWREKLALNMLIWFICGCAVFVIAILGNIICPTQHV
YSTGDVAEHNDSVYTYIRGEYGGGSSDDIFPVQVSAVCNGKDGSVSPFVSMEVSNTDEYAKYHDFRAWTNDSRP
DWYYESMTEMRWTSRVGFIGWTKKELKNKASSGSSVAIIDNMSLPAVMFTSASSTFLTFKPNKSMHFTCLTIGS
RGDVQPYIALAKGLMADGHGIEYGYVGGDPAELMRICVENGMFTVSFLKEGLQKFRGWLDDLLKTSWDACQGTD
VLIESPSAMGGLHIAEALRIPYFRAFTMTWTRTRAYPHAFAVPERKSYVMFDQVFWRAIAGQVNRWRRKSLNLD
STNLDRMEPHKIPFLYNFSPTVVPPPLDWPEWIRITGYWFLDDADASGKKWSPPDSLNKFIHKARKEHKKIVYS
DPKAMTRCVIEAVVNSGVRAILSKGWSDRLDIEEPLPSSIYPIASIPHDWLFAGIPTIIHPFFGDQFFWADRVE
ALGVGSGVRRLTVESLTDALRAATTDVKQIEKAKAVGEKIRSEDGDRPREIFEPLRLACETRSEKLMIASLDCI
SKLISYSFFEELVDLVVNTITQCHNESTPESVSLQIVKALLALVLSQTILVHHSSLLKAVRTVYNIFLLSNDPV
NQTVAQGGLTQMVNHVFDLFIKDAFLVFRALCKLTMKPLNTESERDLKSHAMRSKLLSLHLVLSILNSHMPLLV
DPSAIIYSSSSHDATTFVQAINQYLCLSLSRNAVSPVLQVFELSVEIFWRVLSGMRTKLKKEIEVLFREIFMPI
LEMKTSTLKQKAIILGMFSRLCQDPQALVEIYLNYDCDRESADNIYEHLMNIISKIGSPALSTQAMSIAGSNMG
HSEAQLKRQGLECLVTVLRSLVAWGTDDPSKFETAKQRKTTLLEGIKKFNFKPKGIQFLIENGFIPSNSPQDIA
AFLLHTDGLSKAMIGEGDEANVTIMHAFVDMMDFRGLGFVDALRTFLQSFRLPGEAQKIDRYMLKFADKYMANN
NAAYVFAYSVILLNTDAHNPQVKKRMTKADFIKNNRGINALPEDFLTTVYDEIVSNEIRMKDEVANVGRDLQKE
AYVMQSSGMASEQFFSATQFVHVRPMFEVAWIPFLAGLSGPLQETDDIEIVELCLDGFKAFVTTLAKFTFLNNL
GEMKAKNMEAIKTLLDIALHEGNHLKGSWHEVLTCVSQLEHMQLLSSGRSKKLPAEALANESRSTHITVASDMV
FSLSHYLSGTAIVDFVQALCDVSWEEIQGSGLSQNPRLFSLQKLVEISYYNMNRIRVEWTNIWAILGEHFNQVC
CHNNPHVGFFALDALRQLAMRFLEKEELPQFKFQKDFLKPFEYTMVHNQNPEIRDLVLVCLQQMIQARVENMRS
GWRTMFHVFSAASRVLTERIVNSAFEIVTRLNKEHFGAIVRHGAFADLTNCITQFCKVSKSQPSDHFWHPVLFG

FYDIIMTGEDLEVRRLALDSMFSTLKTYGAGFPLEYWDAICSELLFPIFSVLKSSQDLSRFSTQEDMSVWLSTT
MIQALRNLIDLYTYYFEILERSLDGLLDLLCVENDTLSRIGTSCLQQLLENNVKKLSPARWERVVTTFIKLFKT
TTPHQLFDESLRIERRRIFKQIIVKCVLQLLLIETTSDLLRNDEVYSTIPPEHLLRLMGVLDQSYRFARDFNED
KELRTGLWKESSSAATLIHILTQMYFDPRPEHRKARPQISERLLPLGLGVIEDFNKLRQESQAKNILAWTPVVS
EILDCFSRLDDKSFKMYLPAIYPLATHLLDRDPEIRAGLKAYYLRVGFAQGIQRQALIVASQSKYGREVVAPIL
YNILPQLSLPPGATLVQTLMQLGPDVTNDPDVVRAIFARFGITDAAPPSDNQVVELIQTLGRLAAEGSTMCDVG
AVVRALSSYPVQLDWPAVIRSFDWPDRGVDTATLKLLIAVLLNSPRAHPHAVTGFWTVWNNSLYQLRLLDALLS
LPADTFNFVSLPGHRIVTVDDVSIASPTIKSLAANVQGHTWNSLDLFQVLVRLADSDSPDIKGCIRDMLDKAIK
ISAELVHMGLLQVPNWNEIRLEYSRKLLAMFLGGHPNHQLVFMRIWQIQPSYLTDAFRDFYEENPQNITRILDI
LEALLEVRPFTFALDEYLNLDKWLGDKVAAHGTEFLRSIADFLKEKMESEKLTRITDPPVRTMPINPSTVAIIL
RVIRGSSHLMEQADVDYALDVQNMCFQVYPRLMDVESGLAVIQYPQDIENEVDLIYKRMYDENISIDEVITMLQ
QLRTSSNPRDQEVFSCMIHFLFDEYRFFQYPDRELAMTGYLFGSIIQHDLLDYMPLGIAVRYVIDALNCPPDKN
LFRFGVQALSRFESRLPEWQPLCAELLKNQHLLEARPDLAITLQRAELSDKILFIVNNLAPSNFDTKLVDMKQS
FSTEPNNHSLYLRFLDALNRSSLNKYILHETFVKAAALLNSEKTVQSSTERSILKNVGSWLDRPIKHKNLSFKD
LLMEGYDSGRLIVAIPFVCKTLEPAAQSKVFRPPNPWLMAVISLLAELYHFAELKLNLKFEIEVLCKSLDIDLD
TVEAAVIMRNRPRQVGTQIEQILTTLAQHVQISSTLSMLGNPAFKRAVQLAVDRAVREIILPVVDRSVTIAGIS
TRELVAKDFATEANEEKMRKAAHSMAAKLAGSLAMVTCKEPLRTNLAQHLRQYLAEHGFSDINHIMDLALDNLD
NACSAIERAAMERAISDVDEGFAPAYEMRIHHRETRSGHSLTTNLPDILRLHKNGVLPVQLNVYEDFFGVLIRD
LDNVLVQLPQSLALLPPNHDVRHLVRQIMLNADRRQAPLQMSQKIVQLLYKAPTQLGREVYVALLDQLCRHFEE
TGKEAINWLIYAEDERKYNIPVTVTLLRSRLFDLAVYEQQLAKDPRPSLINFVIGLIRECLTADATQSQFPYCI
EWFQSWVQIYHRSPNPEKSFVGFITQVLKSGVLNIDESQLFFRVCAETSVNHYAKAVAVGNYASAYSYVDAMSK
LVVFIIKYHGDPNADQAKVHYFKKVLSILILVLAYFHEEQEFQQKPFFRLFSSLLNDLNSMEASLGTVYFPLLV
VFCETLSGLQPIYFPGFAFSWMGLISHRLLMPKLLLSENREGWAIMQKLLIALFKFLAPFLKDSELSVPARDMY
RGALRILLVLLHDFPDFLSEYYFSLCDVIPYRCIQLRNIVLSGFPQTMVLPDPHLRGMKMGPIPPILSDFAAGL
KGGDLKAYLDQYLLGRGSTSFLPSLKERLETYNLSLLNALVMYIGVSSVAQAKARNGTALFAPSDPGVVALQYL
ANSLDAEGQFHLITSMTLHLRYPNAHTYWFCSLLLHLFTEVEDEKFQEIMTRVLLERFMVHRPHPWGAVMTFVE
LLRNPKYNFWSKEFINVAPEVSMLLESVGHQRLYIQLCFRNVTDLLTVRRDIMPIALANGAKRDAVDAYAEVVD
PQDSIIDIREYDVPYYLRVAIDIDIRVGLWYGVTFEAGQPHFERVKRADPVVMAYDIETTKAPLKFPDQALDQV
MMISYMVDGQGYLITNREIVSEDIGDFEYTPKEGYEGPFIVFNEADEAATISRFFQHIQEIKPTVMATFNGDFF
DFPFLDARAKANGIDMFLEIGFTKDNEDEYKSRCCVHMDCFRWVKRDSYLPQGSQGLKAVTTAKLGYNPIELDP
ELMTPYAQEQPQVLAQYSVSDAVATYYLYMKYVHPFIFSLCNIIPLCPDEVLRKGTGTLCETLLMVEAYRGHII
MPNRHEEAHGNMYEGHLLASETYVGGHVEALEAGVFRSDIPTDFKIDPTAIQLIDQLDAALTFCVTNYEEVKGE
IQRMLEVMRDDPKRTDKPLIYHLDVAAMYPNIMLSNRLQPDSMVDESVCAVCDYNRPGKTCDRRLEWAWRGEFF
PAHRDEYNMEHFPPKRPGNPERRFVDLSPAEQTALLHKRLGDYSRKVYKKVKDTKVETRTSIVCQRENPFYVDT
VRRFRDRRYEYKGLHKTWKKNLDSMAEVDEAKKMILAHKCILNSFYGYVMRKGARWHSMEMAGITCLTGAKIIQ
MARALVERIGRPLELDTDGIWCMLPGVFPENFKFKLDNGKNIGFSYPCTMLNHLVHDQYTNHQYHDLDPETGEY
KIQSENSIFFELDGPYKAMILPASKEEDKLLKKRYAVFNDDGSLAELKGFEVKRRGELQLIKIFQSQIFEKFLL
GTTTQECYAAVAEVADQWLDVLFSHADSLPDEELVELIAENRSMSKTLAEYAGQKSTSISTARRLAEFLGDQMV
KDKGLACKFIISQRPIGAPVTERAVPIAIFSAEEDVKRTYLRKWLKDNSLVNFDLRSILDWDYYIERLGSVIQK
LITIPAGMQKIPNPVPRIRHPDWLHRRVANAVDKFKQNKVTDFFPGRYMLWLSVEADLVSVAVRVPRQFYINLR
TPDLYQWEKVTRTLPRGLPATNLFIDLMNDPNVDGVFEQQTLNKAETVGFNLQQLETKFILLYHACSATAALHV
FALFYPGGVKMHVVDPATRRQPTTFHGNDATALKAVSRELGLIENRGYTVVISSSKDQSYFDRSVQKLTKFPIL
SMTKIRQAHTLDFPWQLHVAHKMNARYLAMGPWLDRLIATAQYFPLVVSDISFARRLVQQDMVLWWSPGENPDL
GGIECDRRPTDDFPRTEFMSPGAYPNVCLEITIRNLAVNSVLHSVLVNELEGSGGATAFDANQRDVTFGESNVS
PHMFSILRNMLKGWLLDRIGPASMGIDHFWRWISSSNATMYDPSLHRFVHGLMRKTFIQMLAEFRRLGSQIVCA
DFGRVLLATSKPPGTAYAYATYLTSAVTSHELFEHVKLTTDRFYDFLLFMDPANVGGVVCEDPLAVEPPTELAL
EMRWNIAQFLPKAIRPDFEAVVQYFIVELFKARQKKVQEADLIQEFISRKLTRKMLRAVFPVLPGSHLTLESPP
LEFVKFACAVFTLAKDYTVEVGLLKRLLLELIGVREFASEAAFRNPCEPLVLHGVPCRHCDALRDFDLCRDPDL
LPWACATCGGEYDRLAIEFRLVGMVQGMERAFAQQDLRCSRCQQVQSDNVSRTCCSGAYQHTMNKADIRRRLRT
AVNVAIVHNLPRLFEVEHLKYATLATTANLATQQQMADMKTARNVIEYNEQYVTKRLLLSIIWAFSGDAKGEWI
QWQSKVPVIEILYSWLSEHKPLMLCGPPGSGKTMTLFSALRKLPDMEVVGLNFSSYRKTPKGIILAPVQIGRWL
VVFCDEINLPAADKYGTQRVISLVEHGGYWRTSDMAWVKLERIQFVGACGRVPLSHRFLRLVMVDYPGEVSLKQ
IYGTYNRAALKVVPNLRTYAEPLTDAMVAFYLASQKRFTTDIQAHYVYSPRELTRWVRGIYEAIRPMEVLSVEG
LVRVWAHEALRLFQDRLVSEDERQWTDEHIDNAAMEYFPTINILFSNWTSKNKARLRVFYEEELDVPLVLFNDV

LDHVLRIDRVFRQTTLSRFVAWMNGLSIFQIKVSNKYTGDDFDLLANAEVPGLFEGFTMNPPENGLASRAATSP
ALFNRCVLDWFGDWLPSYSPPAIFPIAYRVVNALVHVHLSLHAINQRLSRRQGRYPRHYLDFINHYVRLYSEKR
DELERHLHVGLDKLVTQVEELRKSLAIKRKLKRMVSDQQEAEQKKAASIELQAALVEQDKHIEQRRAVVMADLA
DAEQMTKHLRDIMKRDFLSRPSYNFETVQRASRACLIAELEGKIAKYKEEYALLIRVQSKVDRSMKLLESLSRT
FDAEMSTIVGDVLLSAAFLAYGGWSNHLSEANIKFKPELSFTRLSWQEKSLPSDTLTTVTSFLDEAFLKVLESA
LRFGNPLLIQDVEHLDPILNELRRTGGRVLIRLGSQDIDFSPAFPSVEFSPDICSRVTSQSLDQVLKVETDLMK
VQGEFRLRLRTLEKLLLQALNESTGNILDDDKVIDTLETLKREAAETDVVMKEVEEVTVEYLPLAQACSAVFFV
LEQLNLVNHFYQFSFLDIFDYILHHNPHLKGVLMNDLFLIVYKRTSRALDEAEFLLESGLEAYAKSSIFKPVQN
HFIENENQWAAFLLIIKCFRPDRLLQSVPGYDASYRVENQEGFSLADQAIAAASRQGTWVLLKNVHLAPSWLGE
KKLQTLNPHRNFRLFLTMEANPSIPTNILQSRLIMNEPPPGIFLLAWFHAIVQEDMAAAFGTIDAWLVAASKGK
ANVDPATIPWDALRTLVKESVYGGRVDSDFDQRIVNSFVDGLFTPAAYNIDFPEGTKMEHFLTWPSWLSLPPTA
ERRKMRMLADDDLDRCREWLGHLPEKFNVLERPTGDNQDPLHRLFSREGNIGNLLTQVRRDLSDVLMSALTKGT
IPDHWRRYKVHHIADFSRRLAQLDHIAGLDNGGLFFPEAYITATRAVAHRKKWSLETLNLRLDIERVNDPGLVL
EGASWSTDVLPNTSFTLPPLLGSTLDQHFHAMGVEASEPYLSFAKRFAEDMLAFDVETLPKVHQYPIMACAASP
TAFYAWLSPWLLGRVVVGHNVSYDRARIKEEYELAQSRTRFLDTMSLHVAVKGMSSHQRPAWMKYKKEKKSWQD
VTSANSLRDVAKLHCGIEVSKEIRDDFMTSTRESILADVTDYLSYCAKDVVTTHAVYCKVLPGFLEACPHPVSF
AGILTMGSAFLTVDDGWKRYIQSAEGIFRKMEDGVKRKLHDMAENAWKDDVWLSQLDWEPKWPKWYWEITKPKK
GSMDLTVRNRLAPLLFRLSWLGHPLFHSREHGWVYRVRRKMVSFYDPADDTLFFKLPHKDGDSANVGSPLSKTF
MSHASNGTLSSPFNEAKEALELNAMCSYWISSRDRIMNQMVVWQQWGVIVPQVITMGTVTRRAIEKTWLTASNA
KKNRVGSELKAMVRAPPGYAIVGADVDSEELWISSCMGDAQFGIHGATALGWMTLEGTKSAGTDLHSKTASILG
ISRDQAKVFNYSRIYGAGMRHAVLLLMQSNASMKHEEAQKLAENLYASTKGKNTHRDLFERKFWFGGSESYVFN
KLEAIALSDKPETPALGCGVTHALVKEYLPTGFGSDYMPSRINWVVQSSGVDYLHMLIVSMDHLIQKYDIKARY
LISVHDELRYLVEEKDRYRAALALQIANCWTRCMFAYKLGMDDLPQGVAFFSAVDVDHVLRKEVDMPCVTPSQP
HPIPPGESLNITKVLEKTHGGSLWPDGYKEPNCLTHRAQNAAFLQAQATTEFNEIKRLAAELINKLLATPNDDL
ADVLSQIESWKWPRSDLNAWIKVLNKFDAVLEEVIRDYDVDKLQINPFTPATKRTVSEILRFERMLLENSTNRK
MFSSYDRLNSLLFTSDLDILILAENLLLRPSQQYSAQPSVSHALSIQTPRLLSLAQRWPRAREYGLSLYDLATK
GKAKVEALPPDAREVDFTFYRHIDEQTLQSKPAIEVLADAVETYSVPEEKLELLCRIRTAQCLAPGRQKLVISR
LLAIAIFAHTHPESQATSSIFLYEPDIITSIAELLQVDRGVRAIVADIARPESTITNSYVEAILGFLSYIASHA
SGGNMIVGAGIVPLLIQLIDNRLPKRLTMVSKTMQLVDNVLYSFTNAFNLFCSSRGVTTLVDRIGYEVDTEMPV
ARSAVLKHLLRSLHRMMQASGTSEGLRGLIDTSILKSIKKIIEHRSTFGSNVYPIAINVMATFVHNEPTALPII
QETGLPEAFYKAVESGVEPAIETLQAIPNAIGALCLNEAGQAQLAARPSIIPSIFSIFTSESHIKVLLDKENAV
LLGSAIDELIRHHPTLKTSVFQAITATLGKIETLGNEFVVPNNIRHWYQLENIIVSFVDIFNRFLEGLFQHTDG
LDRLAKLTALPCLPYDFASSVIVQVIRTMAEVSTTETMALLTKSIKESLEAAGFFWMLLDKQSNRKFRLLTTLH
VRLTLLSDVFATYTPTRVSNSLLLGNLHRATVWENILLKAGLNAAALKHLTHVFPLAPFFQALVKRRNTDQAFK
KQISDTASIISKIAIDHFYYTAMLGFTAVLLVDEQLWNWYRAGGQELAHALGVLKVALHLLLPLISSKPLFESP
QTALVMTRKPETDPDYFAPHDFLVRLRLAILPLLQSMWQAPWIKNVPPTIRQPVIHAVLELIGAENEPRSAVER
ALRRTHNNISAATELLLAHPELNAIREPLRASVSRIALTIVDEDPSVKTFSPYAYDTQEQPLANRCRLLALVLP
PVPKWLAAHLLVVEGLLTLAASPKAITLPVSVGPALVEARKVVFDFCLRLMAIPDLPDDPVLSSLRIFVICTRD
HSMACEFVKKDGLSRMGSSSYVAIILRHVAEDLPTVESIMKQNIKRFLNQPRNVDVLTYTRNCSAMALRDPKAF
LRATEALCQLASPDAMVHYLIAELMRVVKYACFLMQCLAELLFSYDSCKMAFLTYSPKKYKSSTLHFLLSELVT
YGAINSKSRLALCNWAMSVLVALCVDSSKEVPAELASVRKFVLEALSRSIKDAQNVEGQEARYGRLLALSDACY
RLLTVKVDTPTHIARVMLEKGFVATLTNALSDVDLNYPHVRQLVASMLRPLEHLTKVAIRMSKEETPDLYRNSA
LGMFPEATTHPLLLDGSSSRELEPLLTIQRWAEELKIVNGDFANERATKLANHVTLTLLPSAIERVTVMINGNP
VDITDTGIDPTFLEALPDDMREEVLNQHIRDQRAARIERPADSQISDEFLDALPPEIRAEIIQQEAIEPAEIDN
ASFIASLDPTLRQAVLLDQDDGFIQSLPKPPAHHDAIQLLDKTGIAVLVRLLFFPQKSLLHKVLVNLCENSKTR
TELFTLLLNILNDGTVDVATVDTRFSQLSMKPDLVAQRCLEALSYIVNENEASSLFFLTEHELSAGLRRSKKGK
GKERQPQLQYPVVLLLSLLDRPTLLKTPSIMEQVATLLATVTRPLTSLKRVTLSHPPTILHNVLRLIVNFLTIG
ECTGRTFQQSLALIQHLSYIPDARDVIAEELKTKAQEFGQALYQDLDELANAMVSKFSASTSVQAKFLRVLKTI
DYMFTIYESFRFTPLWKRLGDCLAIIEQKPETEIVATVLLPLIESLMVVCKYVGTKDSMEELFVSFTDAHRKIL
NLMVRNNPSLMSGSFSLLVNNPRVLDFDNKRNYFTQQLHRRREHYGTIQLNVRRARVFEDSFQHLQRKTGDQIK
YGKLSVRFYDEEGVDAGGVTREWFQILARQMFDPNNALFQPCAADRQTYQPNKNSWVNPEHLSFFKFVGRVIGK
AIYDGRLLDAYFAKSLYRQLLGKPVDYRDVEWVDPEYYNSLCWILENDPTPLDLTFSFGVQRIVPLKEGGETLP
VTNENKREFVQLSAQYRLYSSIKSQIEALSEGFYEIIPKDMITIFNEQELELLISGTPDIDVDEWRAATDYVGY
TSSDPNIVWWWRALKSFDRDERAKVLSFATGTSRVPLGGFTELQGVQGTQKFSIHRAYGDEDRLPSAHTCFNQI

DLPQYSSYEKLRQQLLLAISEGATGFAFAKSSASSGTRKKNARKLRNLNKKAWPTKIKALEDLRHFPGLLLHSS
RRIRMLAVSLHAALLGAWCMAAHDVDRSVAIAGSRTWLPRRAIFDPDGAFSAEQASEAVEDMRARLRVGALGCL
RWGQDQPAVRRAGWTLLQATVLRLILRSSLTEVDNNVQNVLWGPLISFLQEYPQTWPFQLLLTFLGLGSPVQGY
PTVMLMLSTIPSTVFTAFWSALPSLSSNLLSSVVECLVFCIRRRNLWLAAHLIPPPSDTLDRRLDALPPQPIDP
SLAVLVDTDAHGYSSYARIVALLPLRALARQQPWVLRHLIAWDTIRQARIVTALLDHVFEEVPKEEADGWVALV
RTSQKTAPETSLAILAAIARTAPDAPRLDRYRNEVAANLQGTSALPMVRQLVAMAPNADSGVPILPGPRAVNVM
KACQAWIAGDAAEDLEALLPLLFTHLAPVLQTVPGAHWGLIFDVMENNMETLSLEDDAALPALARTLRLIIAVR
DLATTTKSLRAWQEREKGILALVRDMSAPRSACRELLLTVEHLVIESAVDTASMKVRMGYIDQLRSLSVVDTYL
MPNIIHMLRLDRAFKLDQWAIDEFYVSLYESFALPAFAAHIYFRALSTIPSLIASWIQDCKDRSLSATVGTLTA
THFSPVIIARELAHVRTAVDDRRWKSWMLAVQQVQSGHILDGLALFKKNVTLHFEGQVECAICYSIIKPCKTCK
NRFHAGCLYIPISPTLDLN SVKTAGQRLF GLQDCPEYHP TAEQFQDPMA YIQSIAEEAK QFGICKVVPP
PDWKMPFVTD TEFRFKTRLQ RLNSIEASAR AKINFLEKLY RFHKQQGHPR VSVPTINNKA
LDLWTLRKEV DKLGGYEAVK AKQWADLGRV LGYRGPGLST QIKNSYARII LPYEHYMARV
KNSLCDGCDC GFHTFCLDPP LEAIPKEQWF CFACLSGTGD YGFDEGEEHC LSSFQTRDNE
FRRMWFEGHP PVPEYYLEEE FWRLVQSTQE TVEVEYGADV HSATHGAMPT LETHPLDPYS
KDQWNLNNIP IVADSLLRYI KSDISGMTVP WTYVGMTFST FCWHNEDHYT YSINFMHWGE
TKTWYGIPGD DAERFEAAMK REAPDLFEAQ PDLLFQLVTL MNPKHVRDAG VRVYACNQRA
GEFVLTFPKS YHAGFNHGLN FNEAVNFALP DWLSYDRDCV ERYRRHRKMP VFSHDELLVT
ITQQAQTVKA ATWLLDSLKE MTDREMADRQ SVRRGIKERV EAEDPEEQYQ CAVCKVFCYL
SQVVCPCVVC AEHVDALCQK LTLRLRFSDH DLYSTLATVQ ERSSVPAQWR QKYRSLIAET
ARPPLKTLKS ILAEGDKMGC AVPELLTLRK CVIRAGEWLD EATHFLQRKQ NRKREKLEKG
LDDLLQLLKE VEDMGFDTPE IAALRVLAER AATLQQRALD ILAKALSADA SSVNVALEEV
QAVERIVERE ELEKELMEQV RGLLSRARAC GLTNENKYVE ILEAKDKEGG DWEEKAQAIL
HAEVKTVADL PVVPNVLARL RTLYDRAKDY EKQVGIWLGA DKPTLADVHR FIDKVEKEFR
IDMVEDLKRA VKIADDLESR CKQVINHKYQ PDDMFETISQ WTTYARNHLS FQLPFFQKLD
SQLEQHRAWL REIPWYCHGS DLLKDVMDAT RPEDDFAPAD EYFTCICDLP VRPPAEGEVN
TAVQCDHCYA RFHAECANNG GSCPFCDHHH WNGQIPRART WHFCYLPSIL LKAPALTKQY
SREWRELEVI VHRVDRLSAL IGQFLAYCSN QRPEHIPQVR HYMRKLWRIQ FVVSPNPDVS
FGLDLAGLHR LLAGRKTKRK RRPKFLFGQD QDRDWVDGTR CICRALACRR KYHGPCVFLC
PLCCVRKNRR YAWGDVRVKT HAKEQIYVKL AEPRMSTLIL ELIKYIP
>Punctularia strigosozonata
MRRTNQDQSILLTGETGSGKSENRRLAIKSLLELSVSNPGKKGSKLAAQVPAAEFVLETFGNARTLFNPNASRF
GKYTELQFNRGRLCGVKTLDYYLEKGRVAGAPSGERNFHIFYYLVAGASPEERQHMHLIDKATYRYLGHHDGIR
FDQLKMALKNVGFSKRHVAQTCQLIAAILHLGNLEFTIDRGRNEDAAVVRNLDILNVVAEFLGVQPAALEAALS
YRTKMVRKELCTVFLDPDGASDNRDDLAKTLYSLLFAWLNEHINQKLCKDDYATFVALFDLPGSQNMSRPNNLD
QFCINYANERLQNFILNQIFENHVSEYESEGIAAWVPRVPYFENSECLRMIQNQPGGLIHIMDDQARRAPKKTD
HTMVEAFSKRWGNHSSFKAGGVDRYPTFTVNHFNGPVTYSSENFLERNLDAVNPDFVSLLRGGSNNPFVKGLFS
SKAIATQAHPRNEETIVAAQQPVKPMRAPSTRRKNTPCVAGEFRSALDTLFETLQDTQSWYVFCINPNDSQLPN
QLEGRSVKGQVRSAGLSAIAQRNRCVFEVGMTPQEFVDRYQEPLTAIGVTEGEGDIVLGNHKAFLSQAAFHKLE
DQLRSQDAEEQKRNRMRDAEASSQQLPLVQNASSLDDYDQQSRFTSARESYAPSRNMFQGADGEIQEGETTEVV
KETSARRRWVALCWILTFWMPNPFLRWFGRMKRMDVRQAWREKLALNLIIWFVCACAVFVIAVLGNLICPTEHV
FNTNELASHSNNVYTSIRGEYGGTSADNIFPVQVSALCNGVDGTVSPYVILDSSNTDVNAQYHDFRVFTNDSRP
DWYFEQMTVMRWNNRVGFMGYTPRALKNLANNKKNVAIYDGMSLPAHMFTSASSTFLTFKPKESLHFTFLTIGS
RGDVQPYIALAKGLMADGHGIEFGYVGGDPAELMRICVENGTFTVSFLKEGLLKFRGWLDDLLKTSWEACQGTD
VLIESPSAMAGFHIAEALRIPYFRAFTMTWTRTRAYPHAFAVPERKTFVMFDQVFWRAISGQINRWRRKTLGLP
STSLDKMEPHKVPFLYNFSPTIVPPPLDWPEWIRVTGYWFLDSAEVSAQKWTPPAELVQFIDSAHQAGKKVVYS
DPKGMTRSVIEAIVRSGVYAILSKGWSDRLEPEEPLPKQIYQINSIPHDWLFAGIPTIIRPFFGDQFFWADRVE
ALGVGAGVRHLTSESLAQALIAATTDEKQIQRAKVVGERIRAENGDKPREIFEPLRLACETRNEKLMIASLDCI
SKLISYSFFVDLVDLVVHTITACHTETTPDPVSLQIVKALLAIVLSSTTLVHQSSLLKAVRTVYNVFLLSTDPI
TQTVAQGGLTQMVHHVFDLFVKDAFLVFRALCKLTMKPLNSESERDLKSHSMRSKLLSLHLVLTVLNSYMPLFV
SPSALIYSSSSHEATPFVQAVNQYLCLSLSRNAVSPVPQVFELSVEIFWRVLSGMRTKLKKEIEVLLHEIFIPI
IEMKSSTLKQKGVILGMFYRLCQDPQALVEIYLNYDCDSEASDNIYEHIANLISKIATPSYTTTSLAVSGSTVG
LSERQLKRQGLECLVAILKSLVVWGTDDPNRFESARQKKTTLLEGVKKFNMKPKGVEFFLETGFIPNRNPQDIA
KFLLYTDGLSKAMIGEGDEQNIAIMHAFVDLLDFKDLSFVDALRLFLQSFRLPGEAQKIDRYMLKFAERYIAGN

DAAYVLAYSTVMLNTDQHNPQVKKRMTKADFIKNNRGINDLPEEYLSLVFDEIASNEIRMKDEVANVGRDLQRE
AYIMKSHGMANDQFFSASHFVHVRPMFEVAWIPFLAGLSNPLQDTDDLEIVELCLDGFKAFVTTLAKFTFLNNL
GEMKAKNMEAIKALLDIAVTDGNNLKGSWREVLQCVSQLEHMQLITSGRLRKLPAEELANESRSTHITVAADMV
FSLSHYLSGTAIVDFVRALCDVSWEEIQSSGLSQHPRLFSLQKLVEISYYNMNRIRLEWSNLWDILGEHFNQVC
CHNNPHVGFFALDSLRQLAMRFLEKEELPHFKFQKDFLKPFEYTMVHNANPEIRDMVLQCLQQMIQARVQNLRS
GWRTMFGVFTAAAKVLTERIVNSAFEIVTRLNKEHFSAIVRHGSFADLTVCITDFCKASKDDPMIKFWFPVLFG
FYDIIMNGEDLEVRRLALDSLFTTLKTYGSSYPVAFWDTVCQELLFPIFAVLKSSQDLSRFSTQEDMSVWLSTT
MIQALRDLVDLYTFHFDILERFLDGLLDLLCVENDTLARIGTSCLQQLLENNVRKLSPARWERVVTTFIRLYKT
TTPHQLFDESLRTERRRVFKQIIVKCVLQLLLIETTNDLLRSKQVYDTIPPEQLLRLMAVLDHSYQFARMFNED
KELRTGLWKESSSASTLVHILLQMYYDPRADHRSARPQIADKLLPLGMGVLQDYSKLRPDTQAKNIAAWTPVVA
EILHGFSRFDEKAFSRYLPAVYPIAVDILARDPEIREGLRSYFSRVGYVQGIQRQELLRAARTKYGDEIIEPIL
QRILPNLSLAPGTDLVDTFIDLGPENTGDVQSMRALLFRFGITDANPPTDSQLLEVVQALARFAVEGVGVGDVS
ALVAAFGSFHTKLDWPAVIRSFDWPDRSVDTATLKLLIAILLNSPEAEPHAVTGFWDIWDNALAQLRLLDALLS
LPGDTFNFVTLPGRRIVTVDDVSVASPTIKSLAANVQGHTWNSLDLFEVLVRFSEIGTPEIVSCVHEMLDKAVK
ISAELVHMGLLQVEDWSGIRREYGDKLLHMFLNGHPNHQLVFMRIWQIRPTYLTDAFRDFYNESEVNITRILDI
LDSLLDVQPFTFALDEYLNLDKWLQDNINTHGKEFLMAILDFLQAKMESEKAARTSEVAVRTMPLNPQTITIFL
RTLRNSSSMMEKKDVDYCLEVRNSCLQVHPRLMEVEPGFTVVSYSPEIEAEVDSIYKQMYDESITIDEVIVMLQ
RFKNSTNPRENEIFSCMLHFLFDEYKFFQYPARELAMTGYLFGSLIQYELVDYIPLGIAIRCVMDALKCPPQTN
LFKFGLEALARFEGRLAEWRPLCELLLEIPNLLQQRPELGPIIHRAESSDKMLFIVNNLAPSNFESKIDEMREQ
FSTEPNNHQLYLRFLDAIGSKTLFRFILHETFVKSAALLNSDKTMQSSSERAILKNVGSWLDQPIKHKNLAYKE
LLLEAFDNTRLIVAIPFVCKSLEPCASSKVFRPPNPWLMAVIGLLAELYHFAELKLNLKFEIEVLCKALNINLD
TVEATSLIRNRPRAVGSHIEGILSSLLSLVTISPQLALHTNIAFKRAIQLGVDRAVREIILPVVERSVTIAGIS
TRELVVKDFATDTNVDRLQKSAHMMAQKLAGSLALVTCKEPLRSNLATHMRQSLAEHGFGEIQIIMLLVQDNLE
FACQAIEQAAKDRAVDEIDDALMPSYEGRRRHNQQRPNNIYPVPLPDPLRIKASGVQPVQLRVYEDFFLIIVKE
LDAILEQLPDSLTALPANHEVRQLVRQVLFLTEPSHTALALSQKIVQCLYRTQSQLGREIYVALLQQLCDMFRD
VRQEALPWLAEAEDDRKFNVPVTVLLFKSGLLKVSQQDVHLAKNPRPILQTYVAGLIRECVTANASHHQFVTCV
PHFQRWVGIYQRSPKLDVHFEYFVRELEKTRVLSTDDSLLFFRVCGEASISHYVRSVATGQFDYAFQAVDAFAR
LVTMLVRFQGDKGFDQAKVYYLKKILSTVTLILAHLHEEQGFQQKPFFRFFSSLVNDFHAIKSSLGSVYFRFLT
TISETFSSLQPRYFPGFAFSWTCLVSHRHFMPNLLMSEHREGWSSFHELLLSLFKFLAPFLKNADLQPAMKDLY
RGTLRLLLVLLHDFPEFLSEYYFTLCDAIPPRCVQMRNIILSAYPAGLILPDPNLRDLKMGPIPAVLSDFAAGL
RDPELRSQLDQYLVNRGTPLFLPSLKSRLEGYDLSFINSLVMYIGMSSVAQAKVRSGATLFVPTDPGVVALQYL
ATNLDMEGQYHLLSSMVLHLRYPNAHTQWFSSLVLHLFLEIQEGRFGEVVTRVLLERFVVHRPHPWGAMVTFIE
LMRNPKYEFASKEFVRIASEVTLLLESVNPGRLFLQLCFRNVSDLLTVRREIVPLALANGAKRDAVDAYAEVVD
PREGIIDAREYDVPYYLRVAIDNDLRVGLWYAVTLTAGQPSFERVKRADPVVMAYDIETTKAPLKFPDQATDQV
MMISYMIDGQGYLITNREIVSEDIEDFEYTPREGYEGPFTVFNEPDEPSTIMRWFRHVQEVKPTVMATFNGDFF
DFPFLCARAKVHGIDMFQEIGFAIDNEEEFKSRSCVHMDCFRWVKRDSYLPQGSQGLKAVTTAKLGYNPIELDP
ELMTPYALEQPQVLAQYSVSDAVATYYLYMKYVHPFIFSLCNIIPLNPDEVLRKGSGTLCETLLMVEAYRGHII
MPNRHEEEHGNMYEGHLLASETYVGGHVEALEAGVFRSDIPTHFKIDPTAAQLIDQLDAALEFCVTNYDEVKGQ
IQAKLEEMRDNPLRMDKPLIYHLDVAAMYPNIMLSNRLQPDSIVDEATCAVCDYNRPGKTCDRKLTWAWRGEFF
PAHRDEFNMESFPPKKPGWPQRRFTDLSEAEQTALMHKRLGDYSRKVYKKTKDTKVEERESIICQRENPFYVDT
VRTFRDRRYEYKVLHKTWKKNLDGIAEVDEAKKMILAHKCILNSFYGYVMRKGARWHSMEMAGITCLTGAKIIQ
MARQLVERIGRPLELDTDGIWCMLPGTFPEDFKFKLKSGKPIGFSYPCTMLNHLVHAQFTNHQYHDLDPETGEY
KVHSENSIFFELDGPYKAMILPSSKEEDKLLKKRYAVFNDDGSLAELKGFEVKRRGELQLIKIFQSQIFEKFLL
GTTTQECYAAVAQIADRWLDVLYSRADDLSDEELVELIAENRSMSKTLAEYAGQKSTSISTAKRLAEFLGDQMV
KDKGLACKFIISQKPMGAPVTERAVPVAIFSAEESVKRTYLRKWLKDNSLANFELRTILDWDYYIERLGSVIQK
LITIPAAMQKVPNPVPRIRHPDWLHRRVMAIDDKFHQRKVTDFFPGRFMLWLNVDERLVPIPLRIPREFYIHTR
TPELWTAEKVVRGLPRGAPCVNLFIDLTNDPNVDGVYELQTLNRAQGTGLDLYQLERKYVFLYHAMAATAPVHV
FALFFPDGVRLHVVDPATRRQPTTYHGNDVTALKAISRELGLMENRSFTVVISSLKDQAYFDANVPRLANFPVW
TMPQAKSAHSLDFPWQSSVAQKMVIRYLTVASWIDASVALATYYPLLLADIDFARRLTKEDLVLWWSPGDLPDL
GGIQDDKRPSEPLPGTEFLSPGCYFNVCLEIEVRNLAVNAILHSVIVNELEGSGGATAFDSAQRDLTLGESNVS
PKTFGILKAMVKTWLLDKISPSSLTIDHFWRWISSSVSHLYDPSIHRFVHGLMRKTFIQLLAEYKRLGSHVVYA
DLSRILLVTSKPPGTADAYATYINTAVQSNELFQHIWLKTERFYDFLLFMDPANLGGVVCENPKAVEPPEEIVI
EMQWNIQNFLPPAIQKDFASIVRYFMIELYRSRQKKVKEMELTREFIARRLTRKLLRMVFPVLPGSHLRLYDPV
LEFVKFVCAVLALAKEYQIEVGLLRRSVLELTGVREFSNDAVFRQPCEPLKLSNVPCKHCDSLRDFDFCRDPEL

LPWLCGNCGGEYDRTAIEFALIDLLFGLERTFAQQDLRCTKCKQIQSDNVSRHCCSGSYQLTINKADVRRKLRT
MVNVAILHNLHRLFEVEHLRYATLATSANLTTQQQVAALKTVRNIVEYNEQYATKRLLLNIIWAFSGDAKGEWT
PWQGKVPVIEILYSWLSEHKPLLLCGPPGSGKTMTLFSALRKLPDMEVVGLNFSSYRKTPNGVVLAPVQIGRWL
VVFCDEINLPAADKYGTQRVISLVECNGYWRASDKAWVKLERIQFVGACGRVPLSHRFLRLVMVDYPGELSLKQ
IYGTYIRGALKVVPNLRAYSEPLTNAMVDFYLASQSRFTTDAQAHYVYSPRELTRWVRGVYEAIRPLEILSVEG
LVRVWAHEALRLFQDRLVTEEERRWTDEQIDNIAMEHFPTINILFSNWTSKYKARLRVFYEEELDVPLVLFNDV
LDHVLRIDRVFRQTTLSRFVAWMNGLSIFQIKVSNKYTGDDFDLLANAEVPGLFEGFTMNPPENGLASRAATSP
ALFNRCVLDWFGDWLASYTPPIHFPIAYRVVNALVFVHQSMHQINQRLSRRQGRYPRHYLDFIHHYVRLYNEKR
NELERHLHVGLDKLVEQVEELRKSLAIKRKLKQMVTDQQEAESKKKASIEIQAALVEQDKHIAQRREIVMADLA
DAEQMTKAHRDLMKKDFLSRPSFNFETVNRASKACMISELEASIQKYKEEYALLIRVQGKVDRSMKLLESLSRT
FDAEMSTIVGDVLLSAAFLAYGEWSNHLTEANIKFKPELSFARLSWQSKSLPSDSLCTVTSFLDEAFLKVLESA
LRFGNPLLIQDVEHLDPILNEIRRTGGRVLIRLGNQDIDFSPAFPSVEFSPDICSRVTSQSLDQVLKVETDLMK
IQGEFRLRLRTLEKLLLQALNESSGNILDDDKVIDTLETLKREAAETDVVMKEVEQVTAEYLPLAQACSSVYFI
LEQLNLVNHLYQFSFLDIFDYVLHQNPHLKGILLNDLFLVVYQRTSRALDELEFLLESGLQTFAKDEIFKPVKK
HIQENESDWIPFLLVIKCLRPDRLLQSVPGYDASYRVENQEGFTLADQAISLAARQGSWVLLKNVHLAPAWLGE
KKLQTLNPHRNFRLFLTMEANPSIPVNILQSRLIMNEPPPGIFLLAWFHAIVQEDMRSACNTIDIWLNAAARGR
ANIDPAAIPWDAVRALIKQAVYGGRVDSDFDQRVLDAFVDGLFTPSAYNVDFPDGTKIEHFLSWPSWLSLPPTA
ERRKMRTLEDDDHERCKEWLALLPANFNTLSKPTTENQDPLYRLFHREGMIGKLLSQVRRDLTDVLMSELTKGA
IPTHWKRYKVQKIPNLARRLGQLDHIAGLENGGLFFPEAYITATRAVAHRKKWSLETLRLYLDIEQINDPGLVL
EGANWSTDVLPDVSFTLPPLWGDNIDEHFHFIGSTIYQPWITIAEDFAERMLVFDVETMPEYSPYPVMACAATQ
NGWYAWISPWLLGRIIVGHNVSYDRARIAEEYRLEGTKTRFVDTMSLHVAVKGISSHQRPAWNKHRKAKKRWED
LTSANSLADVASLYCGIKVEKEIRSDFMTHSREEILEGVQDYLNYCANDVDVTHAVYSKVLPAFRERCPHPVSF
AGILTMGSSFLPVNQEWERYLENAERTYKELDDGVKSRLVELAEQAWKESPWLSQLDWTPKWPKWYWDLTRPRK
GSLDLTVSKRISPLLLQLSWQGWPLFHSREHGWAFRVTSTPLYFAHQADAKLFYKMPHKDGEEANVGNPLGKTF
MKYAQDGTMTSPSDAAKDALDMNGQCSYWISARDRILNQIVVWQKWGVILPQMVTMGTVTRRAMERTWLTASNA
KKNRVGSELKAMVRAPPGYAIVGADVDSEELWIASVLGDAQFGMHGATALGWMTLEGTKAAGTDLHSKTASILG
ISRDQAKVFNYSRIYGAGMRHAVQLLLQNSAGMSVEKAQELATELYARTKGKNTHRDLFGRKFWYGGTESVVFN
KLEAVALADVPQTPALGCGVTDALTKAYLTEGFGTDYMTSRINWVVQSSGVDYLHMLIVAMEHLMRDFNIRARY
LISVHDELRYLVKEEDKYRAALALQIAHLWTRTMFAYRLGLDDLPQGVAFFSAVDVDKVLRKEVDMPCVTPSQP
NPIPPGESLSIVDVLKKTDGGSLYPGGYVPPNTMQHRAESAYFLRAQTTSELAEVRMLAADLIAKISDTPNELL
GTALSQIETWAWPRSDLNAWIKVLNRFDEILEDCIRDYEIDKIQTRPFDNDIKYLVSGILKFERLLLENTTNRK
MFASYDRIKSLMSTSDLDILLLALNLLLRPAQQYSAQPHVSQALSISTQQLLCLAKRWPHARDNNLSLVDLASQ
GVEQVNGLPPEAREVNFTFYKHIDHQTIESQSAMDVLKEAVNKYEIPEEKYELLCRIRAAGVLVPSRQKYLTVR
LLAISIFCHTHGETQAQSSLFLYEPDLVAHIAELLQSDKGVRRTVASVSDPLSSMPQSYVEGVLSFVTYLASHT
TGGNMLVGAGLVPLLIQLLDNRLPNRLAIVSKTVQLTDNVLYGFPNAFQLFVNNRGVDTLVGRIQSEVTGQLSV
SRAAVLKHTLRSMHRMMQSSGTAEGLRGLIDSSLLKSIHQILENRTLFGPSIVSLAINIMATFIHNEPTCLTVI
QEAKLPEVFYKSLDAGIEPSIEVIQAIPNALGALCLNQTGQDQLAARPTVIPGLLSIFTSEKHLKVLQDKENAV
LVGTAVDELVRHHPSLKAPVFEAIKSSLSKIEDMGSEFEVPAKLKPWYTLDNIVVSFIDVFGRFLEGLFQHVDG
LTRLGRITALPCLPYEFANSVLVQVIRTLAEVATNETLAFLSGIVKESLTETKDFWFKVGGDSNHEFHKLTVLH
TRITLLSEVYATFGHPRTSVALLLGALHRTCVWENIVFKVDPNGKALRHLVTQIPLGPFFQAIVKRRQLERTQR
QQFLHSGSVIADVLVQHLYHTVMLGHAAILLVEELVFAFYRAGGQELVHSYGGLKVALRLLEPLVTAKSLLEAT
QTQLVATTKPETDSAYFEARNFLVKLRTAILPVVRTMWESSWLPQVPVGVSRSIASVVLELAKGEGEPRSAAER
ALIRMHNNVAAAAELLLAQPELAKAREPLRNNMGKLALRLVDEQPSIKEFQTTAYDVHEQPMALRFRLLALVMP
VLPKWLATHMLVAESLLTAGEDIRSITLPLMTGPTHPEARSFMFDLCLRLLAVPQLPRDEYLSSLRLLVFLTRD
HSTASEFIARGGLPLLGAKSYISLIVRHIVEEPRVLQHTMQREIKRLLAHSNFPDPSHYVRTCSAAALRDPAAF
IQATEAVCQLHRPDEVVYCLISELMQTVKYPCFLMQCLTELLFSYDACKLAFLSYSPRKHRTAALNFFLYDLIS
FGSIHARKRVTLCNWAMSVLVALCVNGSKDVSDDLVSVRKFVVDAISRAIKEVIPSDTIDARYGRLLALAELSH
RLLTVRFDSPTHIAKVMLEKNFVSHLTNALGEIDLNYPNVRGLVTALLKPEQHLTKIAIKMSREETPDLYRNSS
LGMFPEATTHPLLLDRPENRGFDPLHTGQRWAEETKMLYRTSESERLAKLADHVVLALLPAAIERITVMIHGSP
VDITDLGIDPTFLEALPDDMREEVLNQHVRDQRAARVERPADSQLDAEFLDALPPELRAEIIQQETLEPADIDP
ASFIASLDPQLRQVVLMDQDDGFIQTLPKPNAARESIQLLDKGQITVLVRLLFFPEKNLLFKVLVNVCENGKTR
AELLNILINILQDGTGDLAAIDKSFSQMSVRPELIAQRCLDALTYIVNNNETSSLFFLSEHEIPAGLRRSKKGK
GKEKQSSTHYPVVLLLGLLDRPTLMRTPSILESVVGLLATVTRPLATLKRPLLTDPPQIPQHALQLIVNILTMG
ECSARTFQQCLALIQHLSYMADAREVIAQELRSRAQELGQNIQADLDVLAKALPSKFSSPSSDQAKLLRVLKTI

DYMYSIYESFRFTPLWKRLGDCLAIIEQRPEVEQVATVLLPLIEALMVVCKYVVLDESMEDLFVTFTDTHRKVL
NLMVRNNPSLMSGSFSLLVHNPRVLDFDNKRNYFTQQLHRRREQHSTLQLNVRRARVFEDSFQYLQRKTGDQIK
YGKLSIRFYDEEGVDAGGLTREWFQILARQMFNPDYALFQPCVADKLTYQPNRASWVNPEHLSFFKFVGRIIGK
AIYDGRLLDAYFARSFYRQLLGKPVDYRDVEWVDPEYYNSLCWILENDPTGLDLTFDFGVTKIVPLKENGTQIH
VTNENKKEFVQLSAQYRLYTSIKDQIDAILAGFYDIIPKDLISIFNEQEVELLISGTPDIDVDEWRAATEYHGY
TSSDPTIVWWWRALKSFNREERAKVLSFATGTARVPLGGFGDLQGVQGVQKFSIHKAYGEQDRLPQAHTCFNQI
DLPEFSSYEMLRQQLLLAINEGGEGFGFAKSSATSGTRKKHARKLRNLRKKDAVTKARALEDLQHLPSLLTSAS
RRVRLLAAGLHDSLLGTWCAATHDVDRQVALQASRSWWTTKVVLDPGGAHAYLNPEEKGQDRDARLRIGGLGAL
GWGFEQPGVRRAAWGLLSVLLSPALLRAAWTEPDALVRAAMWEPLLVFLQGFPQAWTWIEFLQFLALGSPLQGY
PVVVLVLSTIPPSLFTAFWAALDGGALAFIGALLECILFLSRRRDVFVVGHVLSPSRAELDAMLGRLPATPLHV
SLGVVDPLDVDGFSSYARVVSALLDRQLARTNIWALRHLLALDNPRTSRIVYPVVHHVLSGSDKPHAEQWVQIL
RKMEKQAPESSLAILFAVAQSGLEPTRLDRYRNELAAELTGVPGLKYLRRLHMTIPNPDSDVAFLPQPRAVNLV
KSFQKWVADAVNEEVESVMTDIFVALAPILQNVSGSHWDFMFDVVENNLENCSLAEEETLTLLARSLRFVSTIL
ELVTSNKQLKAWNERQSAVLVLIRNISVPLSTCRELALSIEFLVVEAGVDTASLRVKAGYIEQLRDLDIVASRL
LPAIFELLQVYKAFKLGMWAVENYYVDLYDPISLQVLAAHVYYRALLVVPSLIRTWVNDCKDRTLSRTITTYTS
THFSPVLLEAELTHIKESVLEDRWRSWVLGVFALQNGSIIEGLRLFMKNVAGHFEGQVECAICYSVIKPCRTCK
NKFHAGCLYATVSPPMDMS TVKTASPRPF GLEECPTFYP TMEEFKEPMA YVQSISEEGM KYGLCKIVPP
SGWKMPFVTD TEFRFKTRVM TLNSIEASSR AKLNFLEQLY RYHKQQGNPR VSVPTINHKA
LDLWRLRREV HDRGGYEEVR HKLWAEIGRA LGYTGPGLSA QLKNSYTRVI LPYEHFQHHV
RNSLCDGCDC GFHIFCLDPP LASIPRGQWF CHTCLFGTGD YGFDEGEEHC LSSFQKRDRI
FRQMWWTRHP PVSEDDVERE FWRLVQSPRD TVEIEYGADV HSTTHGGMPT LETHPLDPYA
KDPWNLSNIP ILADSLLRYI KSDISGMTVP WTYVGMVFST FCWHNEDHYT YSINYMHWGE
TKTWYGIPGK DAELFEAAIK KEAPELFEAQ PDLLFQLVTL MNPQTLRDAG VPVYACNQRP
GEFVITFPKA YHAGFNHGFN FNEAVNFALP HWLPFGLDCV QRYREHKKPP VFSHDELLIT
ITQHSQTIKT ALWLQDSLRE MTNREMRMRE QARDGLGEVL EEEDPEDQYQ CTTCKTFCYL
SQITCQCVSC IEHRQQLCAC RILRKRFTDA TLRETLAKVE ERAAIPKQWR SKLHKLLMDS
ARPQLRSLRA LLAEADRINY PLPEVSALRK CVMRANEWVE SANAFLVRKQ TRKREAKERT
LDDLRKVLDE VEDLGFDAPE IASLQALEKQ ATDAKAQAQA LLCERLILDC STLNVQVEEL
AELDKIVASE QLVQDLSGEV QQLLGRIRTC GLPPDNKYVM MLHARRHAAS SFEEKVRELL
ADPHKTIEQM PVDPEVMGDL QLVIVRAKEY QAKAKAWLVP DKPRVEEAME LVTLAENEFN
ILAVQDLKRT TEFAQDLEAR CEAVHEHRYR HGDLFEMFDK WKAYGRDHLT FYMPNFEKLD
VQLKLHYRWV ESLPWYCHGK QILEDVIEST RPEDDLPPND EYFTCICTQA VRPPPAGTVS
DAVQCDHCYA RFHGVCAANG GSCPFCDHHH WNGTIHKDRS WHFCYLPTIL LSAPDITRNY
SEDWKQLEII VHRVDRLSTV IGQFLSFSSN QRPEYIPQVR HFMRKLYKIQ FAVSPSPEVS
FGLDLAGLHR ILAGQRTKKR RRPKFTFGQD IDKNWVDHTR CICRGRTCNR LYHAGCVFMC
PLCCLRKNRP YPYAEVRVKN FAKEIIYRKM PPPYTKTLFV ELTANAA
>Laccaria bicolor
MRRTTQDQCILFSGETGSGKSENRRLAIKSLLELSVSNPGKKGSKLATQLPAAEFVLETFGNARTLFNPNASRF
GKYTELQFSRGRLSGVKTLDYYLERNRVAAVPSGERNFHIFYYLVAGASPEERQHLHLLDKSTYRYLGQRDAQR
FDQLKIALKTIGFSKRHVAQTCQLIAAILHLGNLEFTVDRHRNEDAAVVRNTDILEIVADFLGIQPAALEAALS
YKTKLVKKELCTVFLDPDGASDNRDDLAKTLYSLLFAWLNEHINQRLCKDDFTSFIGLFDLPGPQNMSRPNSLD
QFCINFANERLQNWVQKRLFESHVNEYNLEGIARLVPQVPYFDNSECIRVLQNSPGGLVHIMDDQARRQPKKTD
HTMVEAFQKRWGNHSSFKTGAVERFPSFTVNHFNGPVTYSSEGFLDRNLDAVNPDYVSLLRGGSINPFVKGLFS
TKAIATQAHPRNEDTIVAAQQAVKPMRAPSTRRKNTPCVAGEFKAALDTLFETLDETQPWYIFCVNPNDSQLPN
QLEGRSVKGQVKSTGLVEIAKRCTSMFEVNMTPEEFCERYSEGLEGGGISEGDRDLVLGQHKVFLSQVAFHKFE
DQLRSHDVEEQKRNRIRDQEASNQALPLVSNASPFEDFDGRSRFTSNRESYAPSRNMFQNTDGEIQEGETSEVL
KESSARRRWVALCWMLTFWVPTPLLTYVGRMKRMDVRQAWREKLALNLLIWFICACAVFVIAVLGVVICPTEHV
FSTSELASHSNNVYTSVRGEYGGQSADNIFPVQVSALCDGTSGSVSPYVTLDSSNTDPNSVYHDFRAFTNDSRP
DWYFESMVVMRYTARVGFLGYTPKEIRNMASAGRSAAIYNGLSLPALMFTSASSTFLNFKPHKSLRFTFLTIGS
RGDVQPYIALAKGLIADGHGIEYGYVGGDPAELMRICVENGTFTVSFLKEGLQKFRGWLDDLLKTSWEACQGAD
VLVESPSAMGGYHIAEALAIPYFRAFTMTWTRTRAYPHAFAVPERKTYVMFDQVFWRATAGQINRWRRDLLHLG
PTSLDKMEPHKIPFLYNFSPHVVPPPLDWPEWIRVTGYWFLDDADVSSKKWTPPQDLIDFIDNAHQSRQKVVYS
DPKTMTRCVVEAVLQSGVRAILSKGWSDRLESEEALPAEIYPISSVPHDWLFAGIPTIIRPFFGDQMFWADRVE
ALGVGTGVRKLTVQSLTEALVLATTDQKQIDRAKAIGEQIRGEKGDRPREIFEPLRLACETKNEKLTVASLDCI

SKLISYSFFAELVDLVAHTITSCHTETTPDPVSLQIVKALLSLVLSPTIIVHHSSLLKAVRTVYNVFLLSSDPV
NQMVAQGGLTQMVHHVFDLFIKDAFLVFRALCKLTMKPLNTESERDPKSHPMRSKLLSLHLVLTVLNSHMSLFV
DPTAIIYSSSTNEATNFVQAINQYLCLSLSRNAVSPVPQVFEISVEIFWRVLAGMRTKLKKEIEVLLHEIFIPI
LEMRTSTLKQKAVIIGMLSRLCQDPQALVEIYLNYDCDSEAADNIYEHLMNIISKFGTISWSNSGLAISGSTMG
LSDAQLRRQGLECLVAVLRSLVVWGTDDPSKFESAKQKKTTLMEGIKKFNFKPKGIQFLLEAGFIASKDPRDIA
TFLLTTDGLSKSMIGEGDEENISIMHAFVDQLDFKDHPFIDALRIFLQSFRLPGEAQKIDRYMLKFADRYIAGN
DTAYVLAYSVIMLNTDAYNPQVKKRMTKTDFIKNNRGINDLPEELLSEIFEDIANNEIRMKDEVASVGRDLQKE
AYVMQSNGMANDQFFSASHFVHVRPMFEVAWIPFLAGLSGPLQETDDLEVVELCLDGFKAFVTTLAKFTFLNNL
GEMKTKNMEAIKALLDVAVTEGNNLKGSWHEVLTCVSQLEHMQLISSGRTHKLPTEELANESRSTHITVAADMV
FSLSHFLSGTAIVDFVQALCDVSWEEIQSSGLSQHPRLFSLQKLVEISYYNMTRIRLEWSNLWDILGEHFNQVC
CHNNPHVGFFALDALRQLAMRFLEKEELAHFKFQKDFLKPFEYTMVHNQNPEIRDMVLQCLQQMIQARAQNMRS
GWRTMFGVFSAASRVLTEHIASSAFEIVTRLNKDHFPAIVRYGAFADLTVCITEFSKVSKDDEMIRYWFPVLFG
FYDIIMNGEDLEVRRLALDSLFTTLKTYGSTYPLEFWDTVCQELLFPMFAVLKSSQDLSRFNTQEDMSVWLSTT
MIQALRDLIDLYTFYFDILERFLDGLLDLLCVENDTLARIGTSCLQQLLENNMTKLSSTRWERVTTTFVRLFRT
TTPHQLFDDNLRVERRRTFKQIIVKCVLQLLLIETTNDLLRNENVYNTIPPEQLLRLMGVLDHSYQFARMFNDD
KELRTGLWKESSSAATLVHVLLRMYYDSRPEHQAARPQIAERLLPLGLGVLQDYNKLRPDTQSKNIIAWTPVVA
EILDGFCRFDDKAFSRYLPAIYPLAIDLLSREAEVRLALKTYFVRVGYAHGIQRQALILAAQTKYGRETMGPII
RRIFANLSVTPGTTSVQALVQFGPDITSDVDAIRALLERFGISDANPPRDAEVMEMMTTLGRLAAEGSVLCDVG
ALVRALGTYHVKISWPSVIKSFDWPDRGVDTATLKLLIAILLNSPRVEPHAVTGFWEAWSNSLYQLRLLDALLS
LPADTFNFVQLPGRRIVTVDDVAVASPTIKSLAANVQGHTWNSLELFQVLVRLAGSDSSEVRNCVREMLDKAIK
ISAELVHMGLLQVPPWNDIRLEYSRKLLAMFLAGHPNHQLVFMRIWQIEPSYLTDAFRDFYDENPLNITRILDI
LESLLDVRPFTFALDEYLNLDKWLADNVANHGGEFLHSIIRFLEQKMESEKTCRLSDPAVRTMSLNPNTITIIL
RVLRNNSAAMLEGDIEACREVRNACLQVHPRLMDIEPGLTVVTYSAEIEAEVDGIYQQMYDENTSIDDVIAMLQ
QHKESANPRDHEVFSCMIHFLFDEYKFFQYPARELAMTGYLFGSLIRHQLIDYIPLGIAIRYILDALNCPPETN
LFKFGIQALGRFEFRLTEWRPLCEALLQIPHLAEVRPDLMAIIQRGELSDRILFIVNNLAPSNFDVKLDEMREQ
FSSEPNNHSLYLRFLDALNRQKLSKFVLQETFIKAAALLNSERSMQSGSDRNTLKNVGAWLDQPIKFKNLSFKE
LLIEGYESSRLIVAIPFVCKTLEPCVKSKVFKPPNPWLMAVMSLLAELYHFAELKLNLKFEIEVLCKSLDINLD
AVEATTILRNRPRAVGAHIEAILLNLSHQVQINAQLSLNVNHAFKRAVQLAVDRAVREIIIPVVERSVTIAGIS
TRELVTKDLATDPNEDKVRRAGHLMAQKLAGSLALVTCKEPLKSNLATHLRQYLNEHGFNEQQVVLLLVSDNLD
FACTAIEKAAMDRAVSDVDEGFATSYESRRRHREQRNGSNFSSTLPDPLRIKSTGLQQHQFRLYEDFFGVLTRD
LEAVMVQIPQSLAALPPNHDIRHLVQQVVILADRERTPLMMSQKIVQLLYKTPSQLGREVYVALLDQLCRTFED
VAKEAITWLLYAEDERKYNVPVTLALLKSGLINTTLQDQQLAKDPRPTLLAYTAALIRECLSSDASQNQFQYSI
EWFQQWVNIFQRSHSPEKNFVPFITQLTKQGILKVEDSSFFFRVCAESSVNSYIKSISVGDYDYAFQALDAVSR
LIVYIIKYHGDANNDQAKVHYMTKILSIFVLVLANLHEEQGFQQKPFFRFFSSLVNDLQSVESHLGPAYFQLLI
AISDTYSSLQPTYFPGFAFSWMCLISHRLFMPKLLLSENREGWSAFHKLLLSLFKFLSPFLKDADLQPAARDLY
RGALRLLLVLLHDFPEFLSEYYFTLCDAIPPRCIQLRNIILSAFPPAVILPDPHLRNVNMGPIPPILSDFTSGL
KSGDLRGYLDQYLLNRGTPSFLHSLKDRLESYNLSLINSLVMYIGVSSVAQAKARSGSSLFIASDPGAVALQYL
TTNLDVEGQHHLMSSIVLHLRYPNAHTHWFSSLLLHLFVEVKDDRLREVMTKVLLERFIVHRPHPWGALVTFIE
LLRNPKYDFWSKDFIRIAPEVTLLLESVGHRRLFLQLCFHNISDLLTVRRDILPLAQANSAKRDAVDAYAEVID
PREGIIDIREYDIPYYLRVAIDNDIRVGLWYAVTFTAGHPSFERVKRADPVVMAYDIETTKAPLKFPDQAIDQV
MMISYMVDGQGYLITNREIVSEDIEDFEYTPREGYEGPFIIFNEKDEAATIKRFFSHIQDVKPTVMATFNGDFF
DFPFLDARSKVNGIDMFLETGFAKDAEDEYKSRTCVHMDCFRWVKRDSYLPQGSQGLKAVTTYKLGYNPIELDP
ELMTPYALEQPQALAQYSVSDAVATYYLYMKYVHPFIFSLCNIIPLNPDEVLRKGSGTLCETLLMVEAYRGHII
MPNRHEDAHGSTYEGHLLASETYVGGHVEALEAGVFRSDIATDFKIVPAAVQLMDDLDAALTFCVTNYEEVKAE
IKAALEVMRDNPKRVDNPLIYHLDVAAMYPNIMLSNRLQPDSMVDESVCAVCDYNRPGKTCDRRLEWAWRGEFF
PAHRDEFNMESFPPKRPGGPQRRFPDLNPTEQTALLHKRLGDYSRKVYKKTKDTKVENREAIVCQRENPFYVDT
VRRFRDRRYEYKGLHKTWKKNLDSITEVDEAKKMILAHKCILNSFYGYVMRKGARWHSMEMAGITCLTGATIIQ
MARALVEQIGRPLELDTDGIWCMLPGVFPENFKFKLTNGKNIGFSYPCTMLNHLVHAKFTNHQYHDLDPDTGEY
VIHSENSIFFELDGPYKAMILPSSKEEDKLLKKRYAVFNDDGSLAELKGFEVKRRGELQLIKIFQSQIFEKFLL
GTTTQECYAAVAQVADQWLDVLYSQAESLGDDELVELIAENRSMSKTLAEYGSQKSTSISTAKRLAEFLGDQMV
KDKGLACKFIISAKPMGAPVTERAIPVAIFSAEESVKRTYLRRWLKDNGLVTFDLRSILDWNYYIERLGSVIQK
LITIPAAMQKVSNPVPRVRHPDWLFRRVAGAVDKFKQNKLTDFFPGRFMLWLQVDSELLAVPLRVPREFYIHLK
KPEYYSCEKVTRNLPHDLPCINLFVDLTNDPNVDGVFEQQTLNRARQVGFDLNQLDRKYLFLYHACSANAPLHV
FAVFMPGGVRLHIVDPATNRQPTAYHSNDTTALKAVSRELGLFEDKSFIVVISSSKDQSYFDRLVPKLAKFPVL

CMSQAKGPHTLDFPWHSHLAQKMLNRYLSLGLWLDRMIDLAEYYPLMLSDISFARRLVQQDIVLWWSPGDQPDL
GGIEHDRRPTEDLPKTDFLSAGVYSNVCLEITVRNLAVNSILQSVMVNELEGSGGATAFDSTQRDLTLGESQVS
AQTFGIMKNMLKSWLVDKINPANLALDHFWRWITSSASHLYDQSIHRFVHGLMRKTFIQLLAEFKRLGSVVVYA
DFSTILLATSKPPGTAHAYATYITTAVTSHELFQHIYLNTERFYDFLVCMDRANLGGIVCEDPLALEPPEELAM
EMRWNIEQFLPPAIQGDFSMIIQYFIIELYRIKQKKANEIELISEFIARRLTRKMLKVVFPVLPGSYLHMSNPT
LEFTKFACAVFALAKEYRNEVGILKRNLLELVGVREFAGEATFRNPCEPLKLANVPCRHCDVLRDFDFCRDPEL
LPWLCNHCGGEYDRVAIEFMLIEMTRALERNFAQQDLRCGKCQQIQSDNVSRYCCSGSYQFTISKADMRRKLRT
IVNVAREYNLPRLFEVEHLKYATLATSANLETQKQIASIKTVRNVIEYNEQYVTKRLLISIIWAFSGDAKGDWI
AWQSRVPVIEILYSWLSEHKPLMLCGPPGSGKTMTLFSALRKLPDLEVVGLNFSSYRKTPNGVILAPIQIGRWL
VVFCDEINLPAADKYGTQRVISLVECGGYWRASDMAWVKLERIQFVGACGRVPLSHRFLRLVMVDYPGEISLKQ
IYGTYNRALLKVVPNLRAYAEPLTDAMVSFYLASQKRFTTDVQAHYVYSPRELTRWVRGIYEAIRPLEILSVEG
LVRIWAHEALRLFQDRLVSEEERLWTDENIDAAALEHFPTINILFSNWTSKNKARLRVFYEEELDVPLVLFNDV
LDHVLRIDRVFRQTTLSRFVAWMNGLSIFQIKVSNKYTGDDFDLLANAEVPGLFEGFTMNPPENGLASRAATSP
ALFNRCVLDWFGDWLPSYGPPAHFPVAYRVVNALVHVHSSLHPINQRLSRRQGRYPRHYLDFINHYVRLYTEKR
DELERHLHVGLDKLVTQVEELRKSLAIKRKLKRMVADQQEAEQKKAASIEIQAALVEQDRNIEQRRAVVMADLA
DAEQMTRQLRDTMKKDFLSRPSYNFETVQRASKACMIAELESKIARYKDEYALLIRVQSKVDRSMKLLESLSRT
FEAEMSTIVGDVLLSAAFLAYGEWSNHLAEANIKFKPELSFTRLSWQSKSLPSDNLTTVTSFLDEAFLKVLESA
LRFGNPLLIQDVEHLDPILNEIRRTGGRVLIRLGSQDIDFSPSFPSVEFSPDICSRVTSQSLDQVLKVETDLMK
VQGEFRLRLRTLEKLLLQALNESSGNILDDDKVIDTLETLKREAAETDVVMREVEQVTAEYLPLAQACSSVFFV
LEQLNLANHFYQFSFLDIFDYILHHNPNLKNVLLKDLFLVVYKRTSRALDELEFLLESGLNNFAKQTLFKPVQA
HITEHEDHWIPFLLIMKCFRPDRLLQSVPGYDASYRVENQEGFTLADQAIAAAARQGTWVLLKNVHLAPSWLGE
KKLQTLNPHRNFRLFLTMEANLSIPVNILQSRLIMNEPPPGIFLLAWFHAVVQEDMASAFSTIDAWLNSVSKGR
ANIDPASIPWDALRTLVKQSVYGGRVDSDFDQRILNSFVDGLFTAQAYNVDFPDGTKMEHFLTWPSWLSLPPTA
ERRKMRMLADDDLDRCREWLSQLPSEFNTLTPQSAEHQDPLYRLFSREGSIGKLLDQVRKDLTDVLMSALTKGQ
IPEHWRRYKVNKIANFSRRLSQLDSIAKLDKGGLFFPEAYVTATRSVAHKKKWSLETLTLRLDLERVNDPGLAL
EGASWSTNVLPDTGFQLPPLQGSNLLEHFHRIGSHAAQPWLNLGKSFAEDMLTFDVETMPNYHPYPVLACAASL
NSWYVWISPWLLGRVIVGHNVSYDRGRILEEYNVVSTKNRFIDTMALHVAVKGISSHQRPAWMKYRKSKKRWED
LTSANSLADVAKLHCGIDMDKQTRSDFMTSTPKEILNNIADYLDYCSTDVSVTHQVYATALPDFLTACPHPVSF
GGILTMGSSFLTVNEEWDAYLARAEKVYRDLEEGVQNKLKKLAEDAWKDDVWLSQLDWTPKWPKWYWDATKPKK
GTLEITVRNRIAPILLRLSWFGWPLFHSREHGWTFRVRLSPLSFYDPTDDKLFYKLPHKDGEKANVGSPLGKSF
IKFAQDGTMKSPGDEAKEALDMNALCSYWISARDRILNQMVVWQKWGIILPQVITMGTVTRRAIEKTWLTASNA
KKNRVGSELKAMVRAPEGYAIVGADVDSEELWISSCMGDAQFGLHGATAIGWMTLEGTKAAGTDLHSKTASILG
ISRDQAKVFNYSRIYGAGMRHAILLLLQSNASMLPEIAQKLAENLYASTKGKNTHRDIFERKFWFGGTESYLFN
KLEEIALSDKPQTPALGCGVTYALSKEYLPAEFGSDYMPSRVNWVVQSSGVDYLHLLIVSMDHLISKYNINARY
LISVHDELRYLVAEEDKYRTALALQIANLWTRSLFAYKLGMDDLPQGVAFFSAVDIDKVLRKEVDMPCVTPSQP
IPIPPGESLDIFGVLAKTNGGSLWKDGYAEPDCLKHRSASAAFLSAQATTDFGEVKGLAAELITKLINTSTSEL
LDVLSQIDSWKWPRSDLNAWIKVLNKFDGILEEIINEYDLDKLQLNPFTPTSKRLIAEILRFERLLLENSTNRK
TFSSYDRLNSLLFTSDLDILVLALNLLLRPSQQYSAQPSVSHALSISTTRLQSLAKKWPHLREYGIGLVDLSCE
GNAELEALPAEAREVNFTFYRHIGEATVLAQPVMNVLADAIDAYSIPDEKFEVLCRIRTAASLVKDREKLVIIR
LLAIAIYGHTHPESQATSNLFLYEPDLIVHIAELLQIDHGIRKTVGDVANEESRLPHTFIEALLSFVTFIASHA
SGGNMVIGAGLIPLLIQMIDNKSPLRLQAVSKTMQLVDNVLYSFTNAFHLFCGARGVDVLVERIEHEVDGELPI
ARVAVLKHILRSMHRMMQSSGTTEGLRGLIDMSLLRSVKKIIEYRGLFGSSILPFAINIMATFVHNEPTSLTII
QEAGLPKTFYEAIEAGIEPSIEVIQAIPNAIGALCLNESGQAQLANRPSIIPAIFTIFTSQRHLKVLIEKENAV
LIGTAIDELIRHHPTLKSAVFEALKSTMSNIENLGLSYRIPKDILQWYQLDNMVVSFIDILGRFLEGLFQHTDG
LSHLGRFTALPCLPFDFANSVMVQVMRTMTEVATSETLLHLATLVKDSLAETHGFWENVTEPSNERFRCLVTLH
IRITLLSDVFATYAHGRGAIGLLLGSLHRASIWENIALNAGLNASALKHITHGLPLAPFFQAMVKRRNPDAAQK
KQMAESSKIVADIMLKHLYYSVILGLLALLLYDDQLAAFYRADGKDLLHAYGGLKVALSLLHPIISSRPLLDSG
QTFLLVSRKKDTDPDYFEPHNFLVRLRTAALPTVRRLWESSWLVQAPLGVIRAVVRTVLELANGENEPRSAAER
ALTRTHNNVNAATEYLLSHPSLNEAREPLVASISRQSLLLIDEHTQVKAFSLSPDNAHEQPLANRCRLLALVLK
HPPRWLASHLLVTEALFTLAEEPRTITLPIPVGPKHLEARGVIFDFCLRLLEVDDLASDELLSALRLLVLFTRD
KDMASQFVNREGLDLLGGSSYIATILRHVVEDSTTVQHIMKQAIRRYFAQPRVVEIATYVRNCSAMALRDTALF
IETTSSLCQLGQPEALIHLLINELMATLKYLCFLMQCLTELLFSYDTCKLAFLSFSPKRFRTVTLHFLLNELIT
FGTINARNRITLCNWAMSVVVALCVDTSKDVSSDLVSVRKFVLETLSRAIKELSPSESMEARYGRLLALADLCH
RLLTVRFEVPTHLAKVMLEKNFVATLTTALSEVDLNYPNVRNLVTSILRPLEHLTKIAIKMSREETPDLYRNSA

LGMYPESTTHPLLLDQRQGREFDPLLTLQRWAEEMKILHGDFVTERIGKLINHVTLALLPAAVERVTVIIHGSA
VDITDTGIDPTFLEALPDDMREEVLNQHVRDQRAARVERPPDSQISSEFLEALPPEIRAEIIQQEAIEPADIDP
ASFIASLDPTLRQAVLMDQDDGFIQTLPKFSPLHDAIQLLDKAGIAVLVRLLFFPQKTLLFKALVHLCENAKTR
TELFNLLLNILQDGTGDLAAVDKSFSQLSVKPDLIAQRCLEALSYIVSANDLSSLFFLTEHELPFGLRKTKKGK
GREKQPQTHYPIVLLLGLLDRQSLLRTPAIMEAVVSLLATVTRPLTSLKKILLANPPQVPHAVLRLIVNILTIG
ECSGRTFQQSLSLIQHLSYIPDARDVIAHELKSKAQEFGQTLDTDLEELAGALAAKFSPASSTQAKLLRVLKTI
DYMYTIYESFRFTPLWRRLGDCLSVIEKKPDTENIATVLLPLIEALMVVCKYVGSKESMEDLFISFTDSHRKVL
NLMVRNNPSLMSGSFSLLVNNPRVLDFDNKRNYFNQQLHRRREHHGTLQLNVRRARVFEDSFQYLQRKTGDQIK
HGKLSVRFYDEEGVDAGGVTREWFQILARQMFDPNNALFQPCAADKLTYQPNKNSWVNPEHLSFFKFVGRVIGK
AIYDGRLLDAYFARSLYRQLLGKPVDYKDVEWVDPEYYKSLCWILENDPTVLDLNFSFGVNQIIPLKEGGESIS
VTQENKREFVQHSAQYRLYSSIKDQIESLSTGFYEIIPKDLITIFNEQELELLISGTPDIDVDEWRAATEYNGY
TSSDPNIVWWWRALKSFNRDERAKVLSFATGTSRVPLSGFVDLQGVQGVQRFSIHRAYGESDRLPQAHTCFNQI
DLPQYSSYEMLRQQLLMAINEGGEGFAFSKSSATSATKKKHAKKLRSFNKKAQPTKQRALEELQHISALFIHPS
RRIRALTASLHTSFLGTWCIAAHDVDRVVSVSALKAWFVQKTALYPSEMYVHLNPEEDEQDRKSRLRVGALGVA
RWGWGQPNVRKAAWALVQTLLGPAILRSAWVEPDLAVQTTMWQPLLTFLKEFPNSWAYREFLQFLELGSPMQGY
PTVIIILSSIPSSLFKSFWAAIDSRALAFLASLLECMVFLLKRPSIFFFAYLLPPSAAELNEMLAAIPADAIDP
SMGVIEPLDKRGFSSYARIVDALLDRIHAKQNLWALRHFIVLDGVRDTRVLSVVLDRILGDIDVEEADMWIQLA
RKLERLAPQTAMTIAAAIAATGTEPQKLDRYRNELAASLLGIRGLLTLRKLAATAPDPDSEVVFLPQVRAVNVV
KVFQQWVASDVGEDVESAMIHVYIHLTPILQNLAGNHWEFIFDVLESVLENSQITDDEALVPLANALRLIIVLQ
DLTVTNKLLKAWDERSSVLLTMVRDISLPRSTCRELTLSIEYLVIEAAAAAASFKIKSGYVEQLRNSNLIVTQF
IPTLLGLLRLDKVFKCDSWFVDEFYIELYETITLRVLAAHLFYRALLCVPSLIHTWVLDCKDRQLTYCVTTYTS
TYFSPVLIRAELAHVRSPVDEDRWRAWVLAVQQTHNGRITDGLGLFKKNVTLHFEGQVECAICYSIIKPCKTCK
NRFHAGCLYIQAPSIFDLS SVKTSSPRPF GLQDCPEFFP TAEEFKDPMS YIRSISERAE PYGICKIIPP
ENWKMPFVTD TKFRFKTRLQ RLNSIEASSR AKLNFLEQLY RFHKQQGNPR VLVPTINHKP
LDLWLLRKEV QKMGGYDAVK GKKWSDLGRI LGYRGPGLST QIKNSYTRVI LPFEHFCDRA
RNSLCDGCDC GFHTFCLDPP LSSIPKEQWF CFTCLSGTGD FGFDEGEEHS LSTFQARDLE
FRRMWFESHP PVSEYDVEEE FWRLVQSPNE TVEIEYGADV HSTTHGAMPT METHPLNPYS
KDPWNLNNIP ILPESLLRFI KSDISGMTVP WTYVGMAFST FCWHNEDHYT YSINFMHWGE
TKTWYGIPGD DAEKFEAAIK CEAPDLFEAQ PDLLFQLVTL MNPQRVTEAG VRVFACNQRA
GEFVVTFPKA YHAGFNHGLN FNEAVNFALP DWLPYARACV QRYREHRKLP VFSHDELLIT
ITQQSQSIKT AMWLIGSLEE MTQREMNDRR KARLGLAEIL EEEDPEDQYQ CNICKAFCYL
SQVTCQCVVC VDHVSLLCEN QTLRKRFSDE ELLDIQAKVA ERAAVPSTWR GKLSKLLLEN
ATPQFRLLRA LLTEGDRINY PLPELASLRK CVTRATEWMD SANAFLIRKQ SRKRDRPDQG
LDDLYALIRE VKNLGFQCNE IGSLDKLVQD CEKMKSEASA LLCRRLLIQG SSLNVLLDEL
IEIEKIVDRE QLVTELLEEV RHLLTRARLC NLPQDNKHVQ LLEVRQREGD DWEGRARNVL
EQPIKTIAEM PIDPTVLDRL MTARTKALDF DKQARAWLSC EKPRLTDVLR LASRAEKDFS
ISSIQLLKQT ADIAADLETR CEQVLKNHYH SEDIFETIGQ WKDYAKDHLK FSLPCFEKLD
AQLKLHEQWR RELPWYCHIQ GLLEDVLECT RPDDDLPPTD EYFTCICNAP VRPPPPGIVS
DAVQCDHCYA RFHGECAKNG GSCPFCDHHH WNGTIHKQRS WHFCYLPNIL NNAPEITRHY
SEDYRQLEII VHGVDRLSAV IGQFLSYTSN QRPEYIPQVR HYMRKLYKIQ FAVSPNPDVS
FGLDLAGLHR ILAGRKPKKR RRPRFTFGQD IDQDWADGTR CICRGRTCGK RYHAGCVFSC
PLCCVRKNKT YEYSDVRVKT FSKDLMFKRL LLPYKPTLFV ELVSFQP
>Heterobasidion irregulare
MRRTTQDQCIILNGETGSGKSENRRLAIKTLLELSVSNPGKKGSKLAHQVPAAEFVLESFGNARTLFNANASRF
GKYTELQFSRGRLSGVKTLDYYLERNRVAGAPSGERNFHIFYYLVAGASPEERQHMHLTDKMTYRYLGQRDAQR
FDHLKMALKNVGFSKRHVAQTCQLIAAILHLGNLEFTTDRSRNEDAAVVRNTDILALVAEFLGVTASALETALS
YKTKMVKKELCTVFLDPDGASDNRDDLAKTLYSLLFAWLNEHINQRLCRDDFVTFIGLFELPGPQNMSRPNSLD
QFCINFANERLQNFIQKRLFESHVAEYNSEGISRFAPQVPYFDNTECIRLLQNKPGGLIHIMDDQARRLGKKTD
HTMVEAFGKRWGNHSSFKVGSMDRFPTFTVNHFNGPVTYSCEGFLERNLDALNPDFVSLLRGGSINPFVRGLFS
GKAIATQMHPRNEETIVAAQQPIKPMRAPSTRRKNTPCVAGEFRAALDTLFETLEETQAWYVFCVNPNDAQLPN
QLEGRSVKGQIRSMGLPEIARRCETMFEVGMTPREFCDRYREQIAGAGVSEGEKDLVLGQYKVFLSQVAFHKLE
NYLRSKDGEEIKRNRMREAEASNQALPLVSNASPFEDFDARSRLTSNHESYAPSRNMFQNADGEIQEGETTEVV
KETSARRRWVALCWLLTWWVPTPFLKWFGRMKRFDVQQAWREKLALNLIIWFICACAVFVIAVLGNLICPTEHV
FSTNELSAHSNNVYTSIRGEYGGKSADDLFPVQVSALCNGVTGSVSPYVTLKFSNTDVNSKYHDFRVFTNDSRP

DWYFESMTVMRWNNRVGFVGYTPKEIKNMASQKRSLAVYNGLSLPAVMFTSTSSTFLTFKPKNPLRFTFLTIGS
RGDVQPYIALAKGLMADGHGIEFGYVGGDPTELMRICVENGTFTVSFLREGMQKFRGWIDDLLKTAWDACQDTD
VLVESPSAMAGYHIAEALRIPYFKAFTMTWSRTRAYPHAFAVPERKTYVMFDQVFWRGTAGQINRWRRNTLGLP
STSLDKMEPHKIPFLYNFSPIVVPPPLDWPEWIRVTGYWFLDDASVGAKKWTPPPDLVEFIDSAHSQGKKVVYS
DPKAMTRCIVDSIVQGGVHAILSKGWSDRLEAEEPLPKSIYPLASVPHDWLFAGIPTIIRPFFGDQFFWADRVE
ALGVGTGVRKLTVDSMAEALIAATTDHKQIDRAKLVGEQIRSENGDRPREIFEPLRLACETRNEKLMVASLDCI
SKLISYSFFVELVDLVVHTITTCHTETTADSVSLQVVKALLALVLSSTILVHQSSLLKAVRTVYNVFLMSTDPV
NQTVAQGGLTQMVHHIFDLFLKDAFLVFRSMCKLTMKPLNTESERDLKSHAMRSKLLSLHMVLVILNAHMDVFV
SPSSLIHSSSSHEATPFVQMANQYLCLSLSRNAVSPVPQVFEISVEIFWRVITGLRTKLKKEIEVLFHEIFFPI
LEMKTSTLKQKAVILGMLSRLCHDPQALVEIYLNYDCDSQAADNIYEHLMNIITKIGTPSLATSAVSVSGSALG
HSEQQLRRQSLECLAAVLRSLVAWGTDDPGRFESAKQKKTTLLEGVKKFNFKPKGIQFLLETGFIPSKAPQDVA
RFLLQTDGLSKSMIGEGDEENIATMHAFVDMLDLRNMPFVDALRVYLQAFRLPGEAQKIDRFMLKFAERYIEGN
DTAYVLSYSVILLNTDAHNPQVKKRMTRADFVKNNRGINELPEELLSVIFDDIVNNEIRMKDEVATVGRDLQRE
AYMLQSNGMANEQFFSASHFVHVRPMFEVAWIPFLAGISGPLQETDDLEVVELCLDGFRAFVTTLGKFTFLNNL
GEMKTKNMEAIKTLLDVAVTEGNNLKGSWREVLSCVSQLEHMQLISSGRTRKLPNEELANESRSTHITVAADMV
FSLSHYLSGTAIVDFVQALCDVSWEEIQSSGLSQHPRLFSLQKLVEISYYNMSRIRLEWSNLWDILGEHFNQVC
CHSNPHVGFFALDALRQLAMRFLEKEELPHFKFQKDFLKPFEYTMIHNANPDIRDMVLQCLQQMIQARVQNMRS
GWRTMFGVFSAASKVLTERITSSAFEIVTRLNKEHFPAIVRYGSFADLTVCITDFCKVSKDDPMIKYWFPVLFS
FYDVIMNGEDLEVRRLALDSLFSTLKKYGATFPVDFWDTVCQELLFPIFAVLKSSQDLSRFSTQEDMSVWLSTT
MIQALRDLIDLYTFYFEILERFLDGLLDLLCVENDTLARIGTSCLQQLLESNVRKLSPARWERVATTFVKLFRT
TTPHQLFDESLRVERRRIFKQIIVKCVLQLLLIETTNDLLRNDEVYNTIPPEHLLRLMGVLDHSYQFARLFNED
KELRTGLWKESSSASTLVHVLLRMYYDPRAEHQAARPQVAERLLPLGLGVLQDYSKLRSDTQAKNIAAWTPVIG
EILRGFCRFDDKAFTRYLPAIYPLAADLIARDPEIREGLRDYFVRVGYAQGIQRQALIAAAQAKYGSETVAPML
HQIFPRLSLPPGTTLVQAFNQFGPEITNDVDTVRALLLRFGVSDSNPPRDAQVIEYMSTLARQAAEGSTLGDVN
AFVRALSSYSVPLNWANAIKAFDIPDRGVDTATLKLLIAILLNSPRAEVHAVTGFWSMWSNSLYQLRLLDALLS
LPADTFNFVTLPGRRIVTMDDVTNASPTIKALAANVQGHTWNSLDLFEVLVRLADSDSADIRNFVREMLDKAVR
ISAELVHMGLLEAPSWNEIRLEYSQKLLNMFLAGHPNHQLVFMRIWQIQPTYLTNAFRDFYEDSPLNITRILDI
LDALLDVRPFAFALDEYLNLDKWLADNVTAHGADFLHDVIAFLDTKMESEKATRISEAAVRTMTLNPLTITIFL
RVLRNSASIMHPNDVDYCLEVRNACLQIHPRLMDAEPGFSVVTYASDIEVEVDGIFKQMYDEHITIDEVIAMLQ
RNKNSSNSRDHEIFSCMLHFLFDEYKFFQYPPRELAMTGYLFGSIIQYELVDYIPLGIAIRYVIDALNCSPETN
LFKFGIQALSRFESRLSEWQPLCQALLSIPHLLEARPDLAASIQRAEVSDKILFIVNNLAPTNFDSKLEDMKGS
FSTEPNNHQLYLRFLDALDKGTLLKFVLQETIAKSAHLLNAEKTMQSSSERAILKNMGSWLDKPIKHKNLGFKE
LLVEGYDNGRLIVAIPFVCKTLEPAAKSKVFRPPNPWLMAVVSLLAELYHYAELKLNMKFEIEVLCKSLDIDLD
TVEATAVLRSRPRVIGSQIEVILSDLIQHVTVNAQLAFSTNHTFKRAVQLAVDRSVREIILPVVERSVTIAGIS
TRELVAKDFATEGNEETLRQAAHSMAQKLAGSLALVTCKEPLRSNLSSHMRQFLIEHGFSEQPVVVMLVQENLD
LACQAIEKAAMERAIADVDESFAPAYEARRRHRQTNRGSNFSQTLPDPLRIKPSGVQPNQIGVYEDFFGALVKE
LEAVLIQLPQSLAALASNHEVRHLVRQILYLADRHRTPLLISQKIVQLLYKTPSQLARDIYVALLDQLCHSFED
VAKEAITWLIYADDERKLNIPVTVTLLRSGLITIAQQDQQLAKDQRPSLQNFAAGLIRECLTCDASQSQFSFTI
EWFQQWVTVFQRSHSPEKSFVAYITQLTKQGILKAEDSSFFFRVCAESSVNSYLKCMASGDYAYAFHALDAMSR
LIVYIIKYHGDANNDQAKVHYLTKILSIFVLVLANMHEEQGFQQKPFLRFFSSLINDLHAIEGHLGSVYFQLLL
AISDTFSSLQPTYFPGFAFSWMSLISHRLFMPKLLLSENREGWSAFYKLLLSLFKFLSPFLKSAELQQSSRDLY
RGSLRLLLVLLHDFPEFLSEYYFSLCDMIPPRCIQLRNIVLSAFPPTLTLPDPHLRNVKMGPIPPILSDFTSGL
KTGDLRTHLDQYLLNRGSPSFLPSLKDRLETYNLSLINSLVMYIGVSSVAQAKARSGSSLFNAGDPGVVALRYL
ATSMDTEGQHHLLSSIVLHLRYPNAHTHWFSSLVLYLFVEVKDDMFSEVVTKVLLERFIVHRPHPWGALVTFIE
LLRNPKYDFWSKDFIRAAPEVTLLLESVGHRRLYLQLCFRNVSDLLAVRRDIVPLALANSAKRSAVDAYAEVVD
PREGIIDVREYDVPYYLRVAMDNEIRVGMWYAITFNAGHPALDRVKRPDPVVMAYDIETTKAPLKFPDQAIDQV
MMISYMVDGQGYLITNREIVSQDIEDFEYTPKEGYEGPFIVFNEADEAATITRFFEHIQEVKPTVMATFNGDFF
DFPFLNARAQVHGIDMFLETGFAKDTEDEYKSRTCVHMDCFRWVKRDSYLPQGSQGLKAVTVAKLGYNPIELDP
ELMTPYALEQPQTLAQYSVSDAVATYYLYMKYVHPFIFSLCNIIPLCPDEVLRKGTGTLCETLLMVEAFRGHII
MPNRHEEEHGNMYEGHLLASETYVGGHVEALEAGVFRSDISTHFKIEPSAAQLIDELDAALSFCVANYDEVKGQ
IQAALEEMRDNPLRMDKPLIYHLDVAAMYPNIMLSNRLQPDSVVDESVCAVCDYNRPGKTCDRRLTWAWRGEFF
PARRDEYNMETFPPKKNGGPPRKFVDLSETEQTALIHKRLGDYSRKVYKKIKDTKVENREAILCQRENPFYVDT
VRRFRDRRYEYKGLHKTWKKNLDSLAEVDEAKKLILAHKCILNSFYGYVMRKGARWHSMEMAGVTCLTGATIIQ
MARQLVEQVGRPLELDTDGIWCMLPGVFPENFKFKLRNGKAIGFSYPCTMLNHLVHDQFTNHQYHDLDPETGEH

KVHSENSIFFELDGPYRAMILPSSKEEDKLLKKRYAVFNDDGSLAELKGFEVKRRGELQLIKIFQSQIFEKFLL
GSTTEECYAAVAQVADQWLDVLFTKAEDLSDEELVELIAENRSMSKTLAEYGTQKSTSISTAKRLAEFLGSQMV
KDKGLACKFIISERPFGAPVTERAIPVAIFSADESVKRTYLRKWLKDNGLTQFDLRSILDWEYYIERLGSVIQK
LITIPAAMQKVANPVPRIRHPDWLHRRVVALDDRFQQHKVTDFFPGRYNLWLSVDSKLVSAPLRIPREFYLHLR
NPDLYSCDKVVKNLPRNLPCVNLFVDLTNDPNVDGIFELQTLNRAQITGLDLDALQPKYVFLYHACSINRPTQI
FALFLPNGVRLHLVDPATRRQPADYHANDITALKAVSRDLGMLEDQSLTVVISSVKEQSYFDAFLPKLSKFPVL
SMPKARAPHSLDFPWQTHFAQKMLIRYFSLGPWLDRMVSLAEYYPLFLSDLDFARRLTNQDTLLWWSPSGRPDL
GGMEDDRRFTEELPQTDFVSPGCYSNVSLEITVRNLAVNAVLHSVVVNELEGSGGTTAFDSAKRDLTLGESNIS
PQTIGVMKSMVKTWLLDKISPASLAIDHFWRWISSRASHMYDPSMHRFVHGLMRKTFIQLLAEFKRLGSHVVYA
DFSRILLVTSKPPGTAHAYATYITTAVTSHELFQHIYLFTEKFYDFLIFMDEANLGGIVCEDPLAVEPPDELII
EMNWNIQKHLPVAIQRDFIDLVRYFIIHLYKTKQKRTKEMDSVKDFLARRLMRKLFMFIFPLLPGSYLNMSDPT
LEFIKFVCAVFELAKDYQSEVGLLKRNALELIGVKYFGDEVIFRNPCDPLKLPSVPCRHCDALRDFDFCRDPDL
LPWSCSDCGGEYDRVAIELELMEFVHGLERTFAQQDLKCGKCKQLRSNNVAKHCCSGSFQLTINKADVRRKLKT
VFNVAIVHKLVRLFEVEHLRYATLATSANIITQKQVAGIKTARNILEYNEQYVSKRLLISIIWAFSGDAKGEWS
AWAARVPQIEILYSWLSEHKPLMLCGPPGSGKTMTLFSALRKLPDMEVVGLNFSSYRKTPNGVILAPVQLGRWL
VVFCDEINLPAADKYGTQRVISLVESGGYWRTSDMSWVKMERIQFVGACGRVPLSHRFLRLVMVDYPGEVSLKQ
IYGTYNRGILKVVPNLRTYAEPLTDAMVAFYLASQKRFTTDIQAHYVYSPRELTRWVRGIYEAIRPLEVLSVEG
LVRVWAHEALRLFQDRLVAEDERQWTGDNIDNTALEHFPTINILFSNWTSKNKARLRVFYEEELDVPLVLFNDV
LDHVLRIDRVFRQTTLSRFVAWMNGLSIFQIKVSNKYTGDDFDLLANAEVPGLFEGFTMNPPENGLASRAATSP
ALFNRCVLDWFGDWLSSYQPPAMFPIAYRVVNALVHVHMSLYQINLRLSRRQGRYPRHYLDFINHYVRLYNEKR
DELERHLHVGLDKLVTQVEELRKSLAIKRKLKQMVSDQQEAEQKKAASIEIQAALVKQDKHIEQRRSIVMADLA
DAEQMTKQLREVMKKDFLSRPSFNFETVQHASKACMISELEASITKYKEEYAFLIRVQGKVDRSMKLLESLSRT
FDAEMSTIVGDVLLSAAFLAYGEWMNHLVEANIKFKPELSFTRLSWQSKGLPSDNLCTVTSFLDEAFLKVLESA
LRFGNPLLIQDVEHLDPILNEIRRTGGRVLIRLGSQDIDFSPSFPSVEFSPDICSRVTSQSLDQVLKVETDLMK
VQGEFRVRLRTLEKLLLQALNESSGNILDDDKVIDTLETLKREAAETDLVMREVEQVTAEYLPLAQACSSVFFI
LEQLNLVNHFYQFSFLDIFDYVLHHNPNLHGILLKDLFLVVYRRTSRALDELEFLLESGLDTYAKQSLFKPVQA
HILEHESDWIPFLLIIKCLRPDRLLQSVAGYDASYRIDNQEGFALADQAIGVASRQGTWVLLKNVHLAPQWLGE
KKLQTLNAHRNFRLFLTMEANPSIPVNILQSRVIMNEPPPGIFLLAWFHAVVQEDMAAAFTTIDAWLHSIAKGR
ANVDPEQIPWDALRTLIKQSVYGGRVDSDFDQKIIDAFVDGLFTSAAYNVDFPEGTKLDHFLSWPSWLSLPPTA
ERRKMRTLTDDDLERCREWLTQLPSTFNVLQKQSGDNSDPLYRLFSREGSIGKLLNQVRKDLIDVLMSSLTKGT
IPDHWRRYKVKKIPDLGLRLAQLDNLATLDNGGLFFPEAYITATRAVAHRKKWSLETLHMRLDIERVNDPGLAL
EGASWIEEVLPDTNFDLPPLQGSTIDEHFHRIGDRAAHPWLELAVTLAEDMIVFDVETMPNYHPYAIMACAASR
NGWYAWISPWLLGRVIVGHNVSYDRARIKDEYHIQGSQNRFLDTMALHVAVKGISSHQRPAWMKYRKSKKRWED
ITSANSLADVAKLHCDIEMDKEIRNDFMTQNPETIRQGIQDYLHYCAEDVFVTHSVYSHVLPEFLTVCPSPVSF
AGILTMGSSFLTVNESWEKYLRDAERTYRDLEDKVKTRLIDLATEAWKDDVWLSQLDWTPKWPKWYWDLTKPKK
GTIDITSRSRISPILLKLSWQGWPLFHSREHGWTFRVRSASVDFHDAADDVLFYKLPHKDGESANVGNPLAKAF
IKFSQDGTLTSPGTEAKDALDMNAQCSYWISARDRVLNQMVVWHKWGIIVPQVITMGAVTRRAIEKTWLTASNA
KKNRVGSELKAMVRAPDGYAIVGADVDSEELWISSVMGDAQFGLHGATAIGWMTLEGTKAAGTDLHSKTAGILG
ISRDQAKVFNYSRIYGAGMRHAVLLLLQANASMLPDEAQKLAEQLYASTKGKNTHRDIFGRKFWFGGTESFVFN
KLEEIALSDRPQTPALGCGVTYALSKEYLPAEFGSDYMPSRINWVVQSSGVDYLHLLIVAMEHLIATYDIEARY
LISVHDELRYLVKEEDRYRAALALQIANLWTRSQFAYKLGMDDLPQGVAFFSAVDVDHVLRKETDMPCVTPSQP
DPIPPGECLDIQKTLDLTNGGSLLRGGYPQPDCLQYRAESAAFLQAQATNEFAEVKHLAAELINKLSNTPDEDL
HEVLGKVDAWTWPRSDLNAWIKVLNKFDAILEDVIRDYDVDKLQVNVFTPSTKKTVCEILRFERLLLENSTNRK
TFSSYDRINGLLSSSDLDVVVLALNLLLRPAQQYSAQPAVSRALSISTPRLQSLAKRWPNLREYGVSLVDLVAK
GKESVEALPAEAREVHFTFYKHVDVHTLESKPTMDVLADLTDAFNVPEERFELMCRIRSAQVLLPGREKLVIIR
LLANAIFVHTHSEHQALSSLFLYEPDLISHISELLALDSGVRKTVADVASSESTLPQSFVDALLSFVTFLASHA
AGGNMVVGAGLVPLLIQILENRLANRLPVVSKAMQLVDNVLYSFANAFQLFCNNRGVDVLVDRIEYEVDGQLPV
VRAAALKHILRSTHRMMQSSGTSEGLRGLIDSSLLKSIKKIIEHRSLFGPSVLPLAINIMACFVHNEPTSLSII
QEANLPEAFYKAVEAGLEPVIEVIQSIPNALGALCLNQAGQDQLEARPTIIPGLFSIFTSERHQRVLQDKENAV
LIGTAVDELIRHHPFLKAAVFEAIKSTLSKIEELGNAYLVPDGIKSWYSLENIIISYIDVHCRFLEGLFQHSDG
LERLGRLTELPCVPYDFANSVLVQVIRTMVDADRHGTLTFLAKLVKVSLGETKEFWNGVKQESNSTFRKLVTLH
IRVTLLSDIYATYSHARSSTGLLLGSLHRVCIWENVLLKSAFNGKALRHIANQIPLSPFFQAVVKRRTPDPPQR
HQIVESAAVVAEVMLKHVYYTFMLGLVTTLLVDGLLDAFFRKGGQELVHAFGGIKVALHLLLPLVSPKPIFDSS
QTIILVTRKKDGDPDYFEVHNFLVQLRLVVFPVARDLWEASWLVSAPQGLIRSVVQVTLEIVNGESEPRASVER

ALMRTHNNVNAATELLLANPELDVLREEVKPNLGALALRLVDEHHAIKRFAPDAAGSREQHLAVRCRLLALVLS
TVHKWLAALLLVAEALLVLGEEPRSITLPLLEGPPYTEARPVLFDVALGILRLTNLPRDELLASLRLLVLLTRD
HEISLEFIKRDGVPLLGSQSYITIILRHIVEDPAVVRFIMRQELQRLFSLPRVTDVQNYVRHCSSMALRDPETF
VEVTQSLCELDRPETVVHFLLAELMKAVKYCGFLMQCLTELLFSYDICKVAFLSYSPKRHRMVALHFLLYDLIT
YGTISAHKRVILCNWAMSLIVALCVETSKDVSPELISVRKFVLEAVNRAIKDLAPSDTMDARYGRLLALSDLCN
RLLTVRFEGPTHIAKIMLEKNFVSTLTNALADVDLNFPNVRGVVAAILKPLEVLSKIAIKMSREETPDLYRNSS
LGMYADIVTHPLLLERPESRQFDPQITLQRWLEEGKILHGKFSADRFLKVPNHIILTMLPAAIERVTVMIHGSP
VDITDTGIDPTFLEALPDDMREEVLNQHVRDQRAARVERPADSQISSEFLDALPPEIRAEILQQERIEAAEIDP
ASFIASLDPQLRQDVLMEQDEGFIQTLPKPPPPRDAIQLLDKGGIAVLIRLLFFPQKSSLFKVLVNLCENSKTR
TELFNLLLNILQDGTGDLASVDRSFAQMTFRPDLIAQRCLEALTFIVSSNELSSLFFLTEHEVPASFRKSRKGK
GKEKQPQTQYPIVLLLSLLDRESLLKTPSILESVVSLLDTVTRPLTSLKKILLAHPPQISHSVLRLIVNILTVG
ECGAKPFHHSLSLIQHLSYIPDAREVIAQELRAKTQDFGHSLYQDLDELANALASKFSSPSSDQAKLLRVLKTI
DYMYSIYESFRFTSLWKRLGDCLSIIEQRPEIEHIATVLLPLIESLMVVCKYVGSSESMEDLFVTFTDAHRKVL
NVMVRNNPSLMSGSFSLLVHNPRVLDFDNKRNYFNQQLRRRREHHGTLQLNVRRQRLFEDSFQYFHRRTGEQIK
YGKLSVRFYDEEGVDAGGVTREWFQILARQMFDPNNALFEPCAADRLTYQPNKASWVNPEHLSFFKFVGRVIGK
AIYDGRLLDAYFARSLYRQILGKPVDYKDVEWIDPEYYKSLCWILENDPSLLDLTFSFGVTKIVDLKENGASIP
VTMANRREFVQLSAQYRLYSSIKEQLEHLLSGFYEIIPKDLVAIFNEQELELLISGTPDIDVDEWRAATDYNGY
TSSDPVIVWWWRALKSFNRDERAKVLSFATGTSRVPLSGFGDLQGVQGVQRFSIHRAYGDSDRLPQAHTCFNQI
DLPQYSSYEMLRQQLLLAINEGGEGFGFAQSSASSATRKKHARKLRKFSKKDAVTKTKALEELQYIPSLFTHPS
RRIRLLTAGLHSTLLGSWCMASRDVDRQVSIYARRSWFVQRAILNPTELYLYLNPEENDSDRRARLRVGGLGVV
QWGLAQPVVRTSAWSLLQVLLSVAVLRSAFVEPDTQVRSVMWRPLLTFLKEFPRAWAYHEFLQFLELGSPLQGY
PTVLIILSTIPSSIFTSFWAAIDGRALEFLSALLECMTFIIRRPSLFLFAYLLPPSQARLYDELKASRSDPAHP
SLAINHPLDIYGFTSYARAVSTLLDRHAAKTSIWALRHLLVLDSYRDSAVLLAALQYIVGDADKGEADLWMGLA
RKIEKSAPQTALAIITAVVQFGSEPPRLERYRNELAAGILGIPGVLLLRRLAATAPDFDSDVVFLPQPRAVNFM
KQCQQWIASDIDEDVESEMTAVFYHLVPILQNVSGSHWDFIFDVIENNLENSSFDDDMTLTILWRTIRLIQVIE
DQVTYNKALRAWQRRQIAILSLLRDLSTPRSTCREAALSIEYLVIESGVNTASMKVRSGYTEHLRSLDLIAKHF
IPLVLDALGLYQFFKLGIWAVDEFYLESFDPLSLPLFAAHLYYRGLVTAPSLFRMWLLDCKDYTLSTSVGNYTS
QHFSPVIIGHELGHVKSPVTENRWRGWLLGTQQIQNGHIADALSLFKKNVSLHFEGLIECAICYSIIKPCKTCK
NLFHSSCLFIPVAPALDFA GVRTSSPRPF GLEDCPTYYP TQEDFRDPMA YVRKISEQAK EYGMCKVVPP
PGWKMPFVTD TEFRFTTRLQ RLNNIEASSR AKMNYLEQLY RFHQQQGNTR VAVPTINNKP
LDVWRLRKEV HALGGFNEVK ARKWADLGRL LGYGGPGLST QMRWSYIRVI HPYEEFCERV
RASLCDGCDC GFHMFCLDPP LASVPKGQWF CHTCLFGTGD FGFDEGEEHS LSSFQARDRE
FRKRWFEDHP PVSEHDVENE FWRLVESPTD TVEIEYGADV HSTTHGAMPT LETHPLDPYS
KDPWNLNNIS ILSDSLLRYI KSDISGMTVP WTYVGMVFST FCWHNEDHYT YSINYMHWGE
TKTWYGIPGE DAEKFEAAIR REAPDLFEAQ PDLLFQLVTL MNPKRLTDAG VRVYACNQRA
GEFVITYPKA YHAGFNHGLN FNEAVNFALP DWLPYGRQCV QRYREHRKLP VFSHDELLVT
ITQQSQSIAT AIWLNDSLKE MTDREMEDRR KARLNMGESL IEADIEEQYQ CKVCNVFCYL
SQVTCACIVC VEHAGMLCSC RVLRTRFSDT QLLETQAAVA ERAAIPGNWQ AKFDKVLKES
ARPPLRALRA LLAEGDRIGY SLSELHDMRK CVNRANEWLD AANGFIVRKQ SRKRERPDRG
LDELYEMLKE VEDLGFDCPE IGVLKERAAK AEEMKERART LLCQRLLMEG SSNNVYLDEL
TDLDKLVARE QLLDELLEEA SQYLVRARAC DLPLDNKHLK KLEARQRAGQ TWDERARHVL
SQPYKTIEEL PIDPTILDRI GTARLKAKEF EKQAQTWLVP EKPRVQDVLR LVTRAEKDFS
IPAVRELKRM TDFALDLESR CDQVLKNTYE HEDIFVIMLK WVDYAKQHLD FSLPKFEKLD
QQLTQHYRWI ESLPWYCHGK QLFDDVMEST KPEDDHPPDD EFFTCICNDA VRPPAPGTVS
DAVQCDHCYA RFHGQCARSG GSCPFCDHHH WNGSIHKERS WHFYYMPTIL MYAPEITKNY
SEDWKQLEVI VHRVDRLAAV IGQFLSFASN QRREYISQVR HFMRKLFKIQ FAVSPNPEVS
FGLDLAGLHR IVAGQRTKKR RRPKFQFGQD IDQDWLDGTR CICRGRTCSK VYHGGCVFIC
PLCCLRKNRR YEYAELRVKM FSKDIIYKTL RPPYTQTLFV ELIRFTA
>Stereum hirsutum
MRRTTQDQCIVLSGETGSGKSENRRLAIKTLLELSVSNPGKKGSKLASQVPAAEFVLESFGNARTLFNANASRF
GKYTELQFTRGRLCGIKTLDYYLERSRVAGAPSGERNFHIFYYLVAGASPEERQHMHLLDKTTYRYLGQRDALR
FDQLKLALKNVGFSKRHVAQTCQLIAAILHLGNLEFTIDRARNEDAAVVRNTDILALVAEFLGVTTSALETTLS
YKTKMVKKELCTVFLDPDGASDNRDDLAKNLYSLLFQWLNEHMNQRLFKDDYVTFIGLFDLPGPQNMSRPNSLD
QFCINFANERLHNFIQKRMFEAHRAEYQSEGIAQYVPEVPYFDNSECLRLLQNKPGGLIHIMDDQARRSAKKTD

HTMVEAFSKRWGQHSSFKVGSVDRFPTFTVNHFNGPVTYSSDGFLEKNAHSLNPDFVSLLRGGSVNPFIRGLYN
SSAIATQNHPRNEDTIVAAAQPVKPMRAPSTRRKGGTCVAGTFRSALDTLFETLEETQTWYVFCINPNDSQLPN
QIEGRAVKGQTRSMGLTEISRRCVNVFEVGMTPREFCDRYREQIAAVGISEGGNDLVLGQYKVFLSQRAFHKLE
DYLRAKDVDEQKRNRMREAEISTGALPLVSNASPFDDFDANSRLTSHRESYAPSRNMFQNADGEVQEGETTEVI
KETSARRRWVALCWMLTFWVPTPFLRWFGRMKRLDVQQAWREKLALNILIWFICGCAVFVIAVLGNLICPTEHV
FNTSELSSHSDSVYTSIRGEYGGTSSDDIFPVQVSALCNGIDGSVNPYVALQFSNTDTNAQYHDFRVSSNDSRP
DWYFESMTVMRWNNRVGFVGYTPKELKSKAAAGSSLGIYNGLSLPAVMFTSTSSTFLTFKPKESLRFTFLTIGS
RGDVQPYISLAKGLMRDGHGIEFGYVGGDPAELMRICVENGTFTVSFLREGVAKFRGWIDDLLKTAWDACQDSD
VLIESPSAMAGYHIAEALRIPYFRAFTMTWSRTRAYPHAFAVPERKTYVMFDQVFWRGTASQINRWRRNILGLP
STSLDKMEPHKIPFLYNFSPTVVPPPLDWPEWIRVTGYWFLEDASASASKWTPPPDLVEFIDNAHALGKKVVYS
DPKAMTRTVIEAIVQSGVHAILSKGWSDRLEPEEPLPKQIYPLASVPHDWLFAGIPTIIKPFFGDQFFWADRVE
ALGVGSGVRKLTVESLAEALGTATTDERQITRAKVIGEAIRSENGDRPREIFEPLRLACETRNEKLIIASLDCI
SKLISYSFFVELIDLVVHTITQCHTETTADAISLQVVKALLALVLSSTILVHQSSLLKAVRTVYNVFLMSTDPV
NQTVAQGGLTQMVHHIFELFLKDAFLIFRSMCKLTMKSLVTESEREMKSHGMRSKLLSLHMVLIILNSHMDIFV
NPYSMVYSSSSRDPVPFIQMANQYLCLTLSRNAVSPVPQVFEVSVEIFWRVLSGLRTKLKKEIGVLFHEIFIPV
LELKTSTLKQKSVILGMLSRLCQDPQALVEIFLNYDCDSKAIDNIYEHLMNILTKIAAPSLSTSALSVPGTTLG
QSEQQLRRQGLESLVAVLRSLVAWGTDDPSRFESAKQKKTTLLEGIKKFNFKPKGISFLIETGFIPSKEPQDIA
RFLLNTDGLSKTMIGEGDDEHIATMHAFVDLIDLRNMPFVDALRAFLQAFRLPGEAQKIDRFLLKFAERYIEGN
DTAYILSFSVIMLNTDAHNPQVKNRMTKADFVKNNRGINDLPEELLHAIYDDIVNNEIRMKDEVATVGRDLQRE
QYMLQSSGMINDQFYSASHFVHVRPMFEVAWIPFLAGISGPLQETDDMETVELCLDGFKAFVTTLAKFTFLNNL
GEMKAKNMEAIKTLLDIAVTEGNNLKGSWHEVLSCVSQLEHMQLISSGKPRKLPNEELANESRSTHITVAADMV
FSLSGYLSGTAIIDFVQALCDISWEEIQSSGLSQHPRLFSLQKLVEISYYNMSRIRLEWSNIWDILGEHFNQCC
CHSNPHVGFFALDSLRQLAMRFLEKEELTHFKFQKDFLKPFEYTMTHNQNPDIRELVLQCLQQMIQARVQNMRS
GWRTMFGVFSAASKVLTERITSSAFEIVTRLNKEHFASIVRYGSFADLTVCITDFCKVSKDDPMIKYWFPVLFS
FYDVIMNGEDLEVRRLALDSLFSTLKTYGAAFPVDFWDTVCQELLFPIFAVLKSSQDLSRFSTQEDMSVWLSTT
MIQALRDLIDLYTYYFEILERFLDGLLDLLCVENDTLARIGTSCLQQLLENNVKKLSAARWERVATTFVKLFKT
TTPHQLFDESLRVERRRIFKQIIVKCVLQLLLIETTNDLLRNSEVYSTIPPEHLLRLMGVLDHSYQFARMFNED
KELRTGLWRESSSASTLVHVLLRMYYDPRPEHQAARPQVAERLLPLGLGVLQDYTKLRSDTQAKNIAAWTPVVG
EILHGFCRFDDKAFMRYLSAIYPLAAELIAREPQIREGLRDYFVRAGRLQGIQRQALIAAAQAKYGSETVAPML
HQILPRLSLPPGTTLVQTFNQLGPDITNDVDTIRALMQRFGMTEANPPTDIQIVEYMSTLARQAAEGTTLGDVN
AFVRALSNSSTTLNWANVIKAFDIPDRGVDTATLKLLIAILLNSPRTEPHAVTGFWMPWTNSIYQLRLLDALLS
LPGDTFNFVSLPGKRIVTMDDVTNASPTIKALAANVQGHTWNSLDLFEVLVRAADSDSTDLRNLVREMLDKAVR
ISAELVHMGLLEAPTWNEIRLEYSNKLLGMFLGGHPNHQLVFMRIWQIQPTYLTNAFRDFYEESPLNITRILDI
LDSLLDVRPFGFALDEYLNLDKWLSDQVSKHGADFLHDVIAFLDAKMESEKTTRVSDPQVRTMTLNPLTITIFL
RFLRNNATSMRPNDVDYCLEIRNACLQIHPRLMDAEPGFTVINYSPEIEAEVDGIFKQMYDEQITIDEVIAMLE
RNKSSTNPRENEIFSCMLHFLFDEYKFFQYPARELAMTGYLFGSIIQFQLVDYIPLGIAIRYVIDALNCPPETN
LFKFGIQALSRFESRLSEWQPLCQALLNIPHLLEARPDLGATIQRAEVSDKILFIVNNLAPTNFEAKLEDMKGS
FSTEPNNHNLYLRFLDALDRKVLSKFVLQETIVKSASMLNSEKTMQSSSERSVLKNVGSWLDKPIKHRNLSFKD
LLIEGYESGRLLVAIPFICKTLEPAAKSTVFRPPNPWLMAVMALLAELYHFAELKLNQKFEIEVLCTSLSVALD
SIEPTAILRHRPRAVGAQIEVLLGELVGRVTISGQLALPSNPAFKRAVQLAVDRSVREIILPVVERSVTIAGIS
TRELVAKDFATEPNEETLRGAAHSMAQKLAGSLALVTCKEPLRSNLSNHLRQFLNDHGFSDQAVIMLLVQDNID
LASGTIEKAAMDRAVAEVDEGFAGAYDARRRHRQTAPRSAFSASLPDPLRIKVNGVQPNQIGVYEDFFNAIIKE
LDAVLPQLPSALSTPSSTPDLRIPVRQILFIADRVRTPLLISQKIVQLLYKTNVQLARDIYVMLLDQLCHAFDE
VAKEAITWLIYADDERKLNVPVTVTLLRSGLITIAQQDQQLAKDQRPSLQNFAAGLIRACLTSDASQSQFTYTI
EWFQQWIVIFQRSPSPEKNFVPFITQLAKQNILKADDSSFFFRVCAESSVESYMKSMARGDFTYAFQSLDAVAR
LIVYIIKYHGDANNDQAKVHYLTKILSIFVLVLANMHEEQGFQQKPFLRFFSSLINDLHSIEKDLGAVYFQLLI
AISDTFSSLQPTYFPGFAFSWMSLISHRLFMPKLLLSQNREGWSAFYKLLLSLFKFMSPFLKSAEFQTSSRDLY
RGSLRLLLVLLHDFPEFLSEYYFTLCDVIPSHCIQLRNIILSAFPPTLVLPDPHLRNVKMGPIPPILSDFTSGL
KTGELRNYLDQYLLSRGSPAFLSSLKDRLEEYNLSLINSLVMYIGVSSVAQAKARSGSSLFNPADPGVVALQYL
ATNLDTEGQHHLLSAMVLHLRYPNAHTHWFSSLLLHLFVEVKDDMFCEVTAKVLLERFIVHRPHPWGALVTFIE
LLRNSKYDFWTKDFIRAAPEVTLLLESVGHRRLYLQLCFRNVSDLLAVRRDIVPLALANSAKRSAVDAYAEVVD
PREGIIDVREYDVPYYLRTAMDNELRVGMWYGVTFNAGQPEFERVKRPDPVVMAYDIETTKAPLKFPDQAIDQV
MMISYMVDGQGYLITNREIVSEDIDDFEYTPKEGYEGPFIIFNEADEAATIRRFFQHIQEVKPTVMATFNGDFF
DFPFLCARAQTHDIDMFLEIGFAKDSEDEFKSRTCVHMDCFRWVKRDSYLPQGSQGLKAVTVAKLGYNPIELDP

ELMTPYAMEQPQVLAQYSVSDAVATYYLYMKYVHPFIFSLCNVIPLCPDEVLRKGTGTLCETLLMVEAFRGHII
MPNRHEEEHGNMYQGHLLASETYVGGHVEALEAGVFRSDIETDFKIVPSAAQLIDELDAALTFCVTNYDEVKQQ
IQTALEFMRDNTSLQAKPLIYHLDVAAMYPNIMLSNRLQPDSVVDEAACAVCDYNRPGKTCDRRLTWAWRGEFF
PARRDEFNMETFPPKRVGLPPRRFPDLSPSEQTALLHKRLGDYSRKVYKKIKDTKVENRETIICQRENPFYVDT
VRRFRDRRYEYKGLHKTWKKNLDSLAEVDEAKKLILAHKCILNSFYGYVMRKGARWHSMEMAGVTCLTGATIIQ
MARQLVEQVGRPLELDTDGIWCMLPGCFPENFKFKLTNGKSIGFSYPCTMLNHLVHDQFTNHQYHDLDLESGEY
KVHSENSIFFELDGPYRAMILPSSKEEDKLLKKRYAVFNDDGTLAELKGFEVKRRGELQLIKIFQSQIFEKFLL
GTTTQQCYAAVAEVADQWLDVLFSKAADLTDEELVELIAENRSMSKTLAEYGGQKSTSISTAKRLAEFLGDQMV
KDKGLACKFIISARPLGMPVTDRAVPVAIFSAEESVKRMYLRKWLKDNGLTNFDLRSILDWDYYIERLGSVIQK
LITIPAAMQKVPNPVPRVRHPDWLHRRVAALGDTFRQHKMTDFFPGRFNLWLQVNTELVALPVRIPREFYVHMR
SAELYSCDKVVKNLPRNTPCLNLFVDLTNNPNVDGVFELQTLNRAQTTGLDLEQLERKYIFLFHACSLNAPVHV
FAIFMPTGVRLHIVDPATRRQPSTYHSSDLTALQAVSRELRLYESQSYTVIISSMKEQSYFDEHCPKLSRFPVL
SMSKSKGPHSLDFPWQSNVAHKMLSRYISLGSWMDRMVALAEYYPLFLSDLEFARRLISQDCVLWWSPSGSPDL
GGMEGDQRYTEELPHTEFKSPGCYSNVSLEITVRNLAVNSVLHSVVVNELEGSGGTTAFDSAQRDLTLGEANIS
PQTFGIMKSIVKGWLLDKISPASLTIDHFWRWVTSKSSHLYDPSIHRFIYGLMRKTFIQLLAEFKRLGSHVVYA
DFSRILLVTSKPPGTAHAYATYLTTAVSSNELFQHIFLQTERFYDFLLFMDQANLGGVVCEDPLAVVPSRDVSI
NMQWNIQKYLPPAIQRDFADFVRYFLVELYKARQKRSKEVDSVKEFIARKLTRKLFKYIFPLLPGSYLRLVDPT
LEFIKFACAVLELAKDFQSEIGLLKRNALELIGVKSFNERAVFRNPCEPLKLSNVPCRHCDALRDFDFCRDMDL
LPWLCAVCGGEYDRTAIELELMEMVHALEQTFAQQDLKCGKCKQVRSDNVSRICCSGTYQPTLSKADVRRKLRT
IVNVAIVHNLGRLFEVEHLRYATLATSANLMTQRQVAGLKTVRNILEYNEQYVSKRLLVSIIWAFSGDSKGEWS
AWAAKVPTIEILYSWLSEHKPLMLCGPPGSGKTMTLFSALRKLPDMEVVGLNFSSYRKTPNGVILAPIQLGRWL
VVFCDEINLPAADKYGTQRVISLVECGGYWRTTDMAWVKLERIQFVGACGRVPLSHRFLRLVMVDYPGEVSLKQ
IYGTYTRALLKVVPNLRGHAEPLTDAMVSFYLASQKRFTTDVQAHYVYSPRELTRWVRGIYEAIRPLELLSVEG
LVRVWAHEALRLFQDRLVSEDEKQWTDDNIDMTAMEHFPTIDILFSNWTSKHKARLRVFYEEELDVPLVLFNDV
LDHVLRIDRVFRQTTLSRFVAWMNGLSIFQIKVSNKYTGDDFDLLANAEVPGLFEGFTMNPPENGLASRAATSP
ALFNRCVLDWFGDWLSSYSPPAIFPIAYRVVNALVHVHMSLYNINQRLSRRQGRYPRHYLDFINHYVRLYSEKR
DELERHLHVGLDKLVTQVEDLRKSLAIKRKLKQMVSDQQEAEQKKAASIEIQAALVEQDKHIAQRRNVVMADLA
DAEQMTKQLRDLMKKDFLSRPSFNFETVNRASKACMIAELEASITRYKDEYALLIRVEGKVDRSMKLLGSLSRT
FDAEMSTIVGDVLLSAAFLAYGEWMSHLVDANIKFKPELSFTRLSWQSKGLPSDNLQTVTSFLDEAFLKVLESA
LRFGNPLLIQDVEHLDPILNEIRRTGGRVLIRLGSQDIDFSPSFPSVEFSPDICSRVTSQSLDQVLKVETDLMK
VQGEFRVRLRTLEKLLLQALNESTGNILDDDKVIDTLETLKREAAETDIIMREVEQVTAEYLPLAQACSAVFFI
LEQLNLVNHFYQFSFLDIFEFILHHNPNLQGVLMKDLFLTVFKRTSRALDELEFLLESGLEVYAKQPLFRPVQN
HIAEHEEEWATFLLVIKCLRPDRLLQSVTGYDASYRVESQEGFSLADQAIATASRQGTWVLLKNVHLAPSWLGE
KKLQTLNAHRNFRLFLTMEANPSIPVNILQSRIIMNEPPPGIFLLAWFHAVTQEDMASAFTTIDAWCRSVAKGK
ANIDPALIPWDALRTLIKQSVYGGRVDSDFDQRILDAFVDNLFTPAAYNVDFPEGTKIDQFLAWPSWLSLPPTA
ERRKMRTLADDDLDKCREWLAQLPSSFNVLTKQTGDNQDPLYRLFAREGSIGNLLGQVRKDLADVLMSSLTKAT
IPVHWRRYKVKRIPDLARRLAQLDNLASLDTGGLFFPEAYITATRAVAHRKKWSLETLHLRLDIERMNDPGLVL
EGASWAEDVLPDTSFTLPPLTGPDLSSHFHRIGSACSHPYLSLARSLAEEALVFDVETLPEYGPYAVMACAASK
NGWYAWISPWLMGRVVVGHNVSYDRARVKEEYHVEGSKNRFLDTMALHVAVKGISSHQRPAWMQYRKSKKRWED
ITSANSLADVAKLHCGIEVDKEIRNDFMTHTPEEIMANVQDYLTYCASDVNVTHSVYAKVLPDFLDACPNPVSF
AGILTMGSSFLTVNQEWERYIENAERTYRELDTKVKERLLELAVEAWRDDVWLSQLDWTPKWPKWYWELTKPKK
GTIDITTRSRVTPILLKLSWKDCPLFHSREHGWTYRIRQTPLSFHDPSDETLFHKVPHKDGESANVGSPLGKTF
IKFSQDGTLKSPGDLANSALDMNAQCSYWISARDRVLKQMVVWEKWGIIIPQVITMGTVTRRAIEKTWLTASNS
KKNRVGSELKAMVRAPDGYAIVGADVDSEELWISSVMGDAQFGLHGATAIGWMTLEGTKAAGTDLHSKTASILG
ISRDQAKVFNYSRIYGAGMRHAVLLLLQANSGMLPEEAQKLAENLYASTKGKNTHRDIFGRKFWFGGTESFVFN
KLEEIALSDRPQTPALGCGVTYALSKEYLPAEFGSDYMPSRINWVVQSSGVDYLHLLIVSMDHLIQKYDIQARY
LISVHDELRYLVKKEDKYRLALALQVANLWTRSMFAYRLGMDDLPQGVAFFSAVDVDFVLRKETDMTCVTPSQP
VPIPPGESLNVEQVLAKTKGGSLWADGYVEQDCLQHRAHSAEFLRAQATQEFEEIKHLAAELINKLHNTPNDEL
HEVLSEIDVWKWPRSDLNAWIKVLNKFDAILEEYIRDYEVDKVQVNVFTPITKKTICEILRFERLLLENSTNRK
TFSSYDRINSFLSSSDLDVVVLALNLLLRPAQQYSAQPAVTRALSISTPRLQSLAKRWSNLREYGVSLVDLVTS
GKHIVEELPSEAREVHFSFYKHIDAPTLESKPTMDILADVIEKYDVPEERFELMCRIRTAQVLFPGREKVVIVR
LLANAIFVHTHPESQAMSSLFLYEPDLIPHIAELLGLDRGVRKTVNDVANPDSTLPQSFVDSLLSFVTFLASHA
AGGNMVVGAGLVPLLIQIIENRLPQRLSVVSRAMQLVDNVLYSFANAFQLFCNNKGVDVLVDRIQYEIDGQLPV
VRAAVLKHILRSMHRMMQSSGTAEGLRGLIDSSLLKSIQKVMEHRSLFGPSVLPIAINIMATFVHNEPTSLAII

QEAGLPETFYKTVDAGLEPVIEVIQSIPNAIGALCLNQAGQDQLTARPTIIPGLFSIFTSERHQRVLQDKENAV
LIGTTVDELIRHHPMLKTSVFEAIKSTLSKIEDLGNAYVVQDNVKQWYKLENTIISYIDVLCRFLEGLFQHSDA
LERLGRLTALPCLPYDFGNSVLVQVIRTMVEAAPAETMTYLAKLVRTSLDETKEFWASQNEDSNQSFRKLVTLH
IRVTLLSDIYSTYAPSRGANSLLLGTLHRVCVWENVVLKSKLNGKALKHLANQIPLSPFFQAVVKRRNPEPSQK
QQILSSAGVVADILIKHLYHTFMLGLVTTLIADELLDAFYRKSGQELIHAYGGIKVALHLLHPLVSPKPIFESS
QTIILLSRKKETDPDYFEPHNFLVKLRIAVLPLTRELWEAEWLAMAPHSLIRSVVQVILEIVNGDGEPRPAAES
ALTRTHNNVNAATEFLLANPELNTARESLKADIGRLSLRLVDEHPAIKEFSPQARGEKEQALAVRFRLLALVLA
GVAKWLAAMLLVSEAMLTLGEEPRTISLPLATGPLYLDARPALFDLCIKLLSVPKLTRDDLLASLRLLVLFTRD
HYQALAFVERGGIPLLGSQSYITIILRHIVEDDDELAVIMRNEIARFLLHPRVIDVPNYIRHLNSMALRFPIAF
IKITSEMCKLDHPETLIHFLLGELIRVVKYSGFLMQCLTELLFSYDFCKTAFLSYSPKKHRTAALHFLLNDLVS
YGTVGEYKRVTLCNWAMSVIVALCVDANRDLTPEIVSTRKFVLEAVSRAIKDPAPSDSVDARYGRMLALADLCQ
RLLTVRFENPTHIAKIMLEKNFVATLTTALAEVDLNFPGAKSLVACILKPLELLSKIAIKMSREETPDLYRNSS
LGMFPEVVTHPLLLEPPHAREFDPFSTLQRWGEEAKVLHGKFSAERFMKLANHLILSMLPDAIERVTVMIHGSA
VDITDTGIDPTFLEALPDDMREEVVNQHIRDQRAARVERPPDSQISSEFLDALPPEIRAEILQQERVEAAEIDP
ASFIASLDPQLRQDVLMDQDDGFISSLPKPPAQRDAIQLLDKSGIAVLVRLLFFPQKNTLFKVLVNLCENSKTR
TELFNLLLNILQDGATDLAAIDRSFSQMTVRPDLIAQRCLEALTYIVTSNELSSLFFLTEHELSIGLKRSRKGK
GKEKQPQTQYPIVLLLGLLDRQSLLKTPSMLESVVSLLDTVTRPLTSLKKILLANPPQIPHSVLRLIVNILTVG
ECGAKPFHHSLSLIQHLSYIPDAREVIAQELRAKTQDFGTSLHQDLEELSNALASKFSSPSSDQAKLLRVLKTI
DYMYSIYESFSFTPLWRRLGDCLSVIEQRPEIEHIATVLLPLIESLMVVCKYVGATESMEDLFVTFTDDHRKVL
NVMVRNNPSLMSGSFSLLVHNPRVLDFDNKRNYFNQQLRRRREHHGTLQLNVRRQRLFEDSFQYLQRKTGDQIK
YGKLSVRFYDEEGVDAGGVTREWFQILARQMFDPNNALFEPCAADKLTYQPNKASWVNPEHLSFFKFVGRVIGK
AIYDGRLLDAYFARSLYRQILGKPVDYRDVEWIDPDYYKSLCWILENDPTALDMTFSFGVMKIVPLKEGGETLP
VTLENRREFVQLAAQYRLYSSIKDQIENLLSGFYDIIPKDLVSIFNEQELELLISGTPEIDVDEWRAATDYNGY
NSSDPVIVWWWRALKSFNRDERAKVLSFATGTSRVPLSGFGDLQGVQGTQRFSIHRAYGDPDRLPQAHTCFNQI
DLPQYSSYEKLRQQLLLAINEGGEGFGFAKSSASSGTRKKHARKLRRLSKKDAVTKGKALEELMHLPSLFLHPS
RRIRLLATSLHVSFLGAWSMASRDVDRQVASYARRSWFVQRAVMDPAGLYLYLNPEESEVDRRARLRIGGLGVL
QYGHGQPVVRTATWNVLAALLSVAVLRSAFVEPDPLVRTAMWKPLLLFLKDHPTAWAYSEFLQFLELGSPTQGY
PTIVIILSTIPSSIFTSFWAAIDGRALAFLTSLLECLSFIIRRVEAFIVGYLLPPTVHQLDIKLSSLRSTPAHP
ILAITQSLDSIGFSSYARLVSALLDLYKARSNIWALKHFIALDTYRTSRVLLEVLKHVLVDAGKADTEQWMMLA
RRLERGAPQTSLAIVHAIARQGSEPPLLARYRNELAAGILGISGLTLLRRLTASAPDPESDVVFLPQPRAVNFM
KTCQKWITGNIDDDVQSEMTGVFMDMVPLLQNVPGSHWDLIFDIIENNLENASFEDDETLVVLWRTVRLIQMIQ
DLVVYNKALKAWQERQMTILGLLRDLSTPRSLCREAALSIEYLVIEVGVDTTSMKVRSGYTEQIRNLDLVASNF
IPLVLNILNLWKAFKLDLWSINEHYLSSYDPLSLKLLTAHLYYRALLTIPSLIRSWLHDCKDRNLSASVTNYTS
QHFSPVIINTELVQLRIRVTESRWRGWLLGVQQIQNGHIIDALSLFKKNVSLHFEGQGECAICYSIIKPCKTCK
NRFHSSCLYIPVNSRLDFS KIRTQSPRPF GLEDCPVFEP TEEEFRDPMG YVKKIENQGR RYGMVKIIPP
KGWKMPFVTD TEFRFTTRLQ RLNSIEASSR AKINFLEQLY RFHQQQGNTR VSVPTINNKP
MDLWVLRKEV QDMGGYEVVK QKKWADIGRL LGYTGPGLST QLKNSYTRVI LPYEHFCERV
KNSLCDGCDC GFHMFCLDPP LLSIPKGQWF CHTCLFGTGD FGFDEGEEHS LSSFQARDLA
FRKLWFLGHP PVTEDDVERE FWRLVKSSNE TVEIEYGADV HSTTHGAMPT LETHPLDPYS
KDPWNLNNMP ILQESLLRYI KSDISGMTVP WTYVGMIFST FCWHNEDHYT YSINFMHWGE
TKTWYGIPGE DAEKFEAAIR REAPDLFETQ PDLLFQLVTL MNPTSLREAG VRVYACNQRA
GEFVITYPKA YHAGFNHGLN FNEAVNFALP DWLPYGRQCV QRYREHRKLP VFSHDELLIT
ITQQSQSIAT AIWLNPSLME MTDRELANRR KARLRLRESL EREDTEEQYQ CNICKSFCYL
SAVTCTCVVC VDHVSSLCKC RSLRLRFSDN ELMETQEKIQ ERAQIPDQWK AKLSRLLLEN
ARPHLKALKA LLAEGDKIQY DMKGLEDLRR CVVKAGEWVQ MANEILVRKP NRKRDRPDKS
LDDLYELLRQ VDDLGFDCPE IGMLRTLAGQ AEEVRGKAKA LLCEKLLLDA SSLNVSLDEL
IDVEKMVARE QLIKELLEEV SGYLTRARIC GLSSENRHIK DLEARQRAGQ TWDERARHVL
SQPFKTIEEL PIDPAVLSKI ANAREKAKDY ERQATTWLNP EKPKVQDVMR LVTRAEKDFN
IPAVKELKRM ADLAGDCENK CDELLRDRFE HDDVFEIIDK FVNYAKQHLT FAMPKFETID
EQLTQHYRWL ETMPWKKDVD LLMKDIMDNT RPEEDLPPED EFFTCICLDA VRPPEQGQVS
DAVQCDHCSA RFHAACAKSG GSCPFCDHSH WNGTIRKERS FQFAVLPVIL NEAPVITRFY
SPEWKQLEIM VHRISRLSTV IAQFLSFSSN QKRDYIGQVR HYMRKLYKLQ FNISPREDLT
FGLDLAGLHR IIASQRTKKR RRPKFMFGQD VDADWLDKTR CICRGRTCSK MYHGGCVFIC
PLCCLRKNRR YEYAELRVKL FSKEMIYKKL PPPYTQTLFV ELIRFTP

>Coniophora puteana
MRRTTQDQSLLISGETGSGKSENRRLAIKTLLELSVSNPGKKGSKLASQVPAAEFVIESFGNARTLFNPNASRY
GKYTELQFTRGRLCGVKTLDYYLERNRVAAVPSGERNFHIFYYLVAGATTEERQHLRLVDKAAYRYLGQRDANR
FEQLKGALKSIGFSKRSVAQTCQLLAAILHLGNLEFTIDRGRDVDAAVVRNTDTLAIVAEFLGVQPAALEATLS
YKTKMVKKELCTVFLDTDGASDNRDDLAKTLYSLLFAWLNEHINSRLCRDDFDTFIGLVDLPGPQNMSRPNSLD
QFCINFANERLQNFIQKQIFEYHVTEYTSEGVAEYVPTVPYFDNSECLRLLQNQPGGLIHIMDDQARRSQKKTD
QSMVEAFGRRWGNHSSFKVGSMDRFPTFTVNHFNGPVTYSAEGFLERNLDALNPDFVSLLRGGSVNPFVKGLFT
GKAIATQAHPKDEDTIVAAQQTIKPMRKPSTRRKGTPCVAGEFRSALDTLFETLGDTQNWFVFCVNPNDSQLPN
QIEGRSVKGQVRSACIAEVARRNVTVFEAGMTPEEFVDRYREALTAIGIGAAERDLVTGTYKVFLTHAAFHALE
NHLRSLDTEEQKRNRMRDAEASQQNLPLVQHAAGGDDYDARSRLTSNRESYAPSRNMFQNADGEVAEGETTEVV
KETSARRRWVMLTWMLTFWVPTPFLTWFGRMKRPDVRQAWREKLAINMLIWFICGCAVFVIAIMGLIICPTEHV
YSTSELQSHSNNVYTSIRGEYGGTDASDIFPVQVSALCSGEGQGVSPWVQLNSGNSDPNAQYHDFRAATNDSRP
DWYFESMTVMRWNNRVGYMGYTPKEISSLANSGSSVGIIDGLSVPAVMFASTSSTFLTFKPKESLHFTFLTIGS
RGDVQPYISLAKGLMQDGHGIEFGFVGGDPAELMRICVENGTFTVAFLKEGLLKFRGWLDDLLRTSWEACQGTD
VLVESPSAMGGIHIAEALQIPYFRAFTMTWTRTRAYPHAFAVPDRKTYVMFDQVFWRAISGQVNRWRRNVLHLP
NTSLDRLEPHKVPFLYNFSPTLVPPPLDWPEWIHVTGNWFLDDADVSATKWTPPPDLLPFIDSAHAQGKKVVYS
DPQAMTRCVIEAVVRSGVYAILSKGWSDRLEPKEPLPPQIYSISSIPHDWLFAGKPTIIRPFFGDQFFWADRVE
ALGIGTGVRKLTVEALTDALTSATTDIKQIDRARIIGEQIRSENGDRPRAIFEPLRLACETRTEKLMIASLDCI
SKLISYSFFAELVDLVVHTITSCHTEATPETVSLQVVKALLALVLSPTILVHHSSLLKAVRTVYNVFLLSSDPV
NQMVAQGGLTQMVHHVFDFFVKDAFLVFRALCKLTMKPLNTESERDLKSHAMRSKLLSLHLVLTILHSHMIMFT
HPQAIIYSTSSNEATSLVQAINQYLCLSLSRNAVSPVPQVFEVSVEIFWRVLLGMRTKLKKEIEVLLHEIFIPV
IEMRTSTLKQKAVILAMFARLCQEPQALVEIYLNYDCDSGATDNIYEHLMNILSKIATTPHSSAMLQVPGSLIG
LSEGQLRRQGLECLVSVLRSLVTWGGDDPSKFESAKQKKTTLLEGIKRFNYKPKGIQMFIETGWIPSNAPKDIA
KFLLTTDGLSKAMIGEADEENVAVMHALVDYLDFRNLPFLDALRMFLQSFRLPGEAQKIDRFMLKFADRYIAGN
DAAYILAYSVILLNTDAHSPQVKNRMTKLDFRKNNRGINDLPEEFLDTIYDQIQSNEIRMKDEVANVGRDLQKE
AYLTQSNGMANAEFFSASHFVHVRPMLEVTWIAFLAGLSGPLQNTDDLETVELCLEGFRAFVTTLGKFTFLNNL
GEMKTKNMEAIKTLLDIAVNEGNYLKGSWHEVLSCVSQLEQMQLISSGKGRKLPAEELANESRSTHITVAADMV
FSLSHYLTGTAIVDFVRALCDVSYEEIKSSGLSQHPRMFSLQKLVEISYYNMNRIRLEWSNLWEILGEHFNQVC
THDNPSVSFFALDSLRQLSMRFLEKEELAHFKFQKDFLKPFEYTMTKNPNPDVRDMVLQCIQQMIQARVQNMRS
GWRTMFGVFSAASKVLTERIAASAFEMVNSLNNEHFASIVRHGAFADLTVCITEFCKVSKDDVMIRFWFPVLFS
FYDIIMNGEDIEVRRLALDSLFSTLKTYGATFPVDFWDTVCQELLFPIFAVLKSSQDVSRFSTQEDMSVWLSTT
MIQALRDLIDLYTHYYEILERFLDGLLDLLCVENDTLARIGTSCLQQFLENNVSKLSSARWERVASTFVKLFKT
TTPHQLFDDSLRVERRRVFKQIIVKCVLQLLLIETTSDLLRNDTIYTTIPPEQLLRLMGVLDHSYQFARMFNDD
KELRTGLWKESSSAATLVHVLLRMYFDERPEHQAARPQIAERLLPLGLSVLQDYIKLRADTQARNIAAWTPVVA
EILHGFCRFDNKAFLRYLPAIYPLTTGLLARDPEIRLGLKMYFERVGYSQGIQRQSLIAAAQAKYGPDIVAPIL
QKIFPTMSLPPNASLVQTLVQLGPEITSDADVVKALLMRFNITESSPPREAQVTEIVTSLARFVTEGVPLCDVG
ALIRALSSLQPTMNWVNVIKSFDRPDRGVDTATLKLIISVLMNSPLVTPHAVSGFWEPWSNSLYQLKLLDALLS
LPGDTFNFVSLPGRRVVTVDDVASAGPTIKALASNVQVHTWNSLELFEVLVSLADSESADTRAFIREMLDKAIK
ISAELVHMGLLQVQNWNEIRTEYSRKLLGLFLAGHPNHQLVFMRLWQIDPTYLLDAFRDFYNENPLNITRILDI
LENLLEAQPFTFALDEYLNLDKWLADHVAEHGASFLHAMVSFLEIKMESEKTARLSDPAVRTIPLSPQSVTVFL
RTLRSNTSVMSREDIDYCLEVRSACLQVYPRLMEVEPGFTVVNYSPDIEAEVDAIYKQMYDEQTTIDEVIAMLV
RCKGSSDQRDSEIFSCMLHFLFDEYRFFQYPARELAMTGYLFGSIIQHQLVDYVPLGVAIRYVVDALSCPADTN
LFKFGLQALGRFESRLPEWQPLCQALLRIPTLMEARPDLSSVIHRAELSDKILFIVNNLSPNNLEAKLAEMKEY
FSTEPNNHQLYLRFLDGLDRKVLSKFILHETFVKSASVLNHEKTMQQTSERHILKNIGAWLDRPIKHKNLSFKD
LLIEGYDNGRLMVAIPFVCKTLEPCAKSKVFKPPNPWLMAVISLLAELYHFADLKLNLKFEIEVLCKGLDIDLD
TVEATTMLRTRPLDVGGHIESILQGLSQRVVINPQLALHINQAFKRAVQLAVDRSVREIIVPVVERSVTIAGIS
TRELATKDFATEPSEDKLRKAGHLMAQKLAGSLALVTCKEPLKSNLATHLRSFLVDHGFNEQQVFAILVQDNLD
VACSAIEKAAMERVISDVDEGFAASYDLRRRHREVRGGSNFTINLPDPLRIKANGLQANQFAVYEDFFTAILRD
LEALMTQLPQSLASLPPNHDVRHLVRQILYLADRHRTPLMMSQKIVQLLYKTSSQLGREIYVALLDQLCRSFED
VAKEAITWLVYAEDERKLNIPVTVTLLRSGLISFSLQDQQLAKDPRPSLLNFAAGLIRECLSGDASVSQFTFSL
EWFQQWVQIYQRSHTPEKAFVPYITQLTKQGVLKAEDSSFFFRVCAESGVNSYLKCVAAGDYEHAFQALDALSR
LIVYIIKYHGDANNDQAKVHYFTKILSIFVLVLANMHETQGFQQKPFFRFFSSLINDLHAVESHLRTAYFQLLL
SISDTLSSLQPTYFPGFAFSWLCLISHRLFMPKLLLSENREGWSAFHRLLLSLFKFLAPFLKEADLQIASRDLY
RGSLRLLLVLLHDFPEFLSEYYFTLCDSIPSRCIQLRNIILSAFPSTITLPDPHLLNYKMGPIPPILSDFTSNL

KNGDLRTHLDQYLLNRGSPTFLPSLKDCLDSYNLPLINSLVMYVGVSSVAQAKARSGSSIFVSGDPGVVALHYL
ATNLDVEGQHHLLSSMVMHLRYPNAHTHWFSSLLLYLFVEVQDDHFREVMTRVLLERFIVHRPHPWGALVTFIE
LLRNQKYEFWSKEFTRVAPEVHMLLDSVGHRRLYLQLCFRNVSDLLAVRRDVAPLALANGAKRDAVDAYAEVVD
PGECIIDVREYDVPYYLRVAMDNAMRVGLWYAVTFTAGQPGFERVKRADPVVMAYDIETTKAPLKFPDHALDQV
MMISYMIDGEGYLITNREIVGEDIEDFEYTPKEGYEGPFTVFNEPDEAATIMRFFHHIQSAKPTVMATFNGDFF
DFPFLCARSKANGIDMFLETGFAIDQEDEFKSRCCVHMDCFRWVKRDSYLPQGSQGLKAVTTAKLGYNPIELDP
ELMTPYAMEQPQVLAQYSVSDAVATYYLYMKYVHPFIFSLCNIIPLCPDEVLRKGSGTLCETLLMVEAYENHII
MPNRHEDAHFNMHEGHLLASETYVGGHVEALEAGVFRSDIPTDFKIVPEAAQLIDQLDDALKFCVTNYDEVKSE
IQAALEEMRDNPKRLDKPLIYHLDVAAMYPNIMLSNRLQPDSIVDESVCAVCDYNRPGKTCDRRLEWAWRGEFF
PAHRDEYNMETFPSKRPGGPQRRFADLTDAEQTALLHKRLGDYSRKVYKKIKDTKVEMRTSIVCQRENPFYVDT
VRRFRDRRYEYKGLHKTWKKNLDSIAEVDEAKKMILAHKCILNSFYGYVMRKGARWHSMEMAGITCLTGATIIQ
MARALVEQLGRPLELDTDGIWCMLPGVFPENFKFKLNNGKALAFSYPCTMLNHLVHAQFTNHAYHDLNPETGDY
DIHSENSIFFELDGPYRAMILPSSKEEDKLLKKRYAVFNDDGSMAELKGFEVKRRGELQLIKIFQSQLFERFLL
GTTTEECYRAVAHAADQWLDILFSKAETLSDEELVDLIAENRSMSKTLAEYAGQKSTSISTAKRLAEFLGDQMV
KDKGLACKFIISAQPHGAPVTERAVPVAIFSAEESVKRTYLRKWLKNNGLTSFELRAILDWDYYIERLGSVIQK
LITIPAAMQKVANPVPRIRHPDWLHRRVAGQVDKFKQNKMTDFFLGRFNLWLSVDNSVVSIPLRIPREFYLHFK
TPEYYSCAKVVRHLPRNFQCNNLFTDLTNDPNVDGVFEMQTLNRAQSVGVDLTQLDRKYVFLYHACSVSAPIHV
FALFLPAGVKLHIVDPATRRQPTTYHNKDTTALKAISRELGTLEDRSYIVVLSSMKDQSYFDMWTPKLAKFPVL
SMPKTRKPHSLDFPWQTHVAKNMLSRYLNVGSWLDRSIALADYYPILLSDISFARRLAEQDIVLWWSPSDRPDL
GGVEQDNRPTEELQTTEFNSPGCYSNVCLEITVRNLAVNSVLHSLVVNELEGAGGATSFDSQQRDLTLGESQVS
SITFGILKSLIKGWLIDKISPATLTIDHFWRWVSSSASHMYDPNIHRFIHGLMRKTFIQMMAEFKRLGSHVVYA
DFSRILLATSKPPGTAHAYATYITTAVNSNELFQHIFLRTERFYDYLLFMDQANLGGVVCEDPLALEPPEDISI
ELRWNIQTFLPAAIQQDFANIMQYCIVKLFKIRQKKENEIELTRDFIARQLQRKLLSTVFPVRPGSYLALSNPV
LEFVKFACAVFGLARDYSVEVGLLKRSLLDLAGVREFAAEAVFRNPCEPLKLSNVPCRHCDQLRDFDFCRDEEL
APWTCGTCGGEFDRMAIELDLMKMVANLERAYTQQDMVCVKCKQLRSDNVSRWCCSGAYQLTIGKAEMRRRLRT
IVNVALVHGLPRLFEVEHLRYATLATSAHLETQKQIASNKTARNIIEYNEQYVTKRLLVSIIWAFSGDAKGEWV
AWQSKVPVIEILYSWLSEHKPLMLCGPPGSGKTMTLFSALRKLPDMEVVGLNFSSYRKTPNGVILAPVQIGRWL
VVFCDEINLPAQDKYGTQRVISLVECGGYWRSSDMAWVTLERIQFVGACGRVPLSHRFLRLVMVDYPGELSLKQ
IYGTYSRALLKVVPTLRAYAEPLTDAMVAFYLDSQKRFTADIQAHYVYSPRELTRWVRGIYEAIRPLEILSVEG
LVRVWAHEALRLFQDRLVTEEERQWTDEHINNAAMENFPTINILFSNWTSKNKARLKVFYEEELDVPLVLFNDV
LDHVLRIDRVFRQTTLSRFVAWLNGLSIFQIKVSNKYTGDDFDLLANAEVPGLFEGFTMNPPENGLASRAATSP
ALFNRCVLDWFGDWLPSYSPPDHFPIAYRVVNALVYVHSSLHAINQRLSRRQGRYPRHYLDFINHYVKLYNEKR
EELERHLHVGLDKLVTQVEELRKSLAIKRKLKRMVSDQQEAEQKKAASIEIQAALVEQDKHIAQRKEVVMADLA
DAEQMTKPLRDLMKREFLSRPSYNFEMVQRASKACMISELESKIATYKDEYALLIRVQNKVDRSMKLLESLSRT
FDAEMSTIVGDVLLSAAFLAYGDWSGHLSEANVKFKSELSLTRLSWQSKSLPADNLTTVTSFLDEAFLKVLESA
LRFGNPLLIQDVENLDPILNEIRRTGGRVLIRLGNQDIDFSPSFPSVEFSPDVCSRVTSQSLDQVLKVETDLMK
AQGEFRLRLRTLEKLLLQALNESTGNILDDDKVISTLETLKREAAETDVVMREVEEVTAEYLPLAQACSAVFFI
LEQLNLVNHFYQFSFLDIFDYVLHHNPNLAGVLLSDLFVVVFKRTSRALDELEYLLEAGLEHYAKNSIFKPVLN
HVMERPEEWAPFILIIKCLRPDRLLQSVTGYDASYRVENQEGFALADQAIAAAARQGTWVLLKNVHLAPTWLGE
KKLQTLNPHRNFRLFLTMEANPSIPVNILQSRLIMNEPPPGIFLLAWFHAVVQEDMASAFGTIDTWLNSVAKGR
ANVDPATIPWDALRTLIKQSVYGGRVDSEFDQRILDSFVDKLFTPMAYNVDFPDGTKVEHFLDWPSWLSLPPTA
ERRKMRMLSDDDFERSREWLAQLPSKLNVLPKQASEKSDPLYRFFSREGTIGKLLGQVRKDLESVLMSSLTKGT
IPNHWRKYKVPKIPDLSRRLAQLEEIVTMQQGGLFFPEAYITATRAVAHRKRWSLETLHLRLEIGEVKNPGLSL
EGASWNVEVLPDTGFQLPPLQGTSIDEHFFAIGARAAQPWMDMAHDLAETALVFDVETMPKYHPYAVMACAASP
HAWYAWISPWLLARVIVGHNVSYDRARMREEYSLDGTETRFLDTMSLHVATSGISSHQRPAWMKYRKDKKRWED
ITSANSLADVAKLHCGIAVDKELRSDFMTHAPAQIRADVRRYLGYCANDVGVTHAVYARVLPTFRQRCPSPVSF
AGVLTMGSSFLTVNESWEAYIADAERAYRELERGIKKRLVELAEEAWREDPWLAQLDWTPKWPKWYWDLAKPKK
GTLDITVRNRLAPLLLRLSWLGFPLFHSREHGWMFRVRLTPLEFFDPADEHLFYKLPHKDGEKANVGSPLAKTF
GKYARDGTLASPWEAAKDALDMNARCSYWISARDRIINQMVVWDKWGMILPQVVAMGTVTRRAIEKTWLTASNA
KKDRVGSELKAMVRAPPGYSIVGADVDSEELWISSCMGDAQFGLHGATALGWMTLEGTKAAGTDLHSKTASILG
ISRDQAKVFNYSRIYGAGMRHAVLSLLQANAGMSQEEAQRLAENLYASTKGKNTHRDLFGRKFWFGGTESFVFN
KLEEIALSERPTTPALGCGITHALSREYLPETFGGDYMTSRINWVVQSSGVDYLHLLIVAMDYLVRAYGIKARY
LLSVHDELRYLVADRDRYRAALALQIANVWTRSLFAYKLGMDDLPQGVAFFSSVDVDRVLRKEVDMPCVTPSQL
SPIPPGESLNIIQTLEKTHGGSLWPVDYVPPDCMKHRAESAGFLRAQATNDFSELKHHAAELISKLLNTPNDDL

ADALAEIDVWKWPRSDLNAWTKVLDKFDEILEEVIRDWDLQKLQVNVFTPLTKSLICEILKFERLLLENSTNRK
TFNSYDRLNDLLFTSDLDVLILALNLLLRPSQQYSAQPAVSHALNISTPRLQSLAKRWSGLREYGVTLFDLARN
NTAQLDVLPADAREVNFVYYKHVDERIIREKEVTEIVADVLEAHTLSSDKFELICRTRCAQALFPEREKLIIVR
LLAIAIYGHTHNESQASSALFLYEPDLPMHVAELLQLDQNIRNTIVETSNPESKLPHSFVDALLSFVTFLASHG
AGGNMVVGAGLIPLLVQVIENRLVQRLPMVSKAMQLVDNVLYGFANAFQLFANARGVDVLVGRIQHEVDGELPV
ARAAVLKHTLRSLHRMMQSSGTSEGLRGLIDSSVLASVKKVVQYRGLFGPSVLPIAINIMATFVHNEPTCLPTI
QEAGLPLVFYKAIEDGIEPVIEVVQSIPNAIGALCLNQAGQDQLAARPSIIPGILSIYTSERHLKMLKDKENAA
MVGTAIDELIRHHPSLKTAVFESIKATMTKIETLGEAYEVPENMKEWYGLDNIIISFFDVFCRFLEGFFQHADG
LERIGRMTALPCLPYDFANSILIQVLRLMVEVGPNEALFYFSKLLATSLAETQPFWGSLGPDSNYQFRSLVILH
IRTTLVADIFLAYTHGRGAMGLLLGALHRACVWENILLKDTLNAKALKHLTHGLPLSPFFQSIAKRRNPDPVQK
KQILNASSVVANIMVEHLYYSVMLGLVTILLVDELLFSFHKIGGQELTRAQGGLKVALHLIHPLISAQPLFESG
QTLLVMTRKKETDADYFESHHFLVQLRLACAPFLQRVWEAPWILSCPLPLVKTTVQAILELTSAENEPRSAAER
ALVRTHNNVNAATELLLAHPELQAARAPILDGLCQRSLQLVDNFPSDIGFHTTSHDGKEEALAVRCRVLALVLP
NVPKWLAAHLLVTEMLLSVGQQPSNVAVPILAGPLYPEARPTIFSYCLRLLAVPDLPRDELLSTLRILVVVTQD
HDTASQLVEQDVISSLGSQSYVAIILRHIAEDPGTVRSIMTQEIKRFFSQPHVIDVHGFLRHCSSLALRDPTIF
VQATRSLCQLQQPESLVHFVINELIKSLKYSCFLMQCLTELLFSYDACKAAFLSFSPKKPRTAALQFLLSELIT
FGAVDNKGKATLCGWAMSVIVALCIDTSKELSNELVSIRKFVLDSINRAIKELPSSESLDARYGRLLALSDLCS
RLLTVRFENSTHVAKIMLEKNFVATLTNALAEVDLNYPHVRSLVAAILKPLENLTRIAIKMSREEMPDLYRNSA
LGMYSENTVHPLLMDKSESGGFDPLLTVQRWAEESKIIHGNHVAERAGKLANHIIIALLPAALQRVTVMVHGSS
VDITDMGIDPTFLEALPDDIREEVINQHVRDQRAAAVERPSESQISPEFLDALPPDIRAELIQQERMEGEEMDP
ASFIASLEPPLRQVVLLDSDDGFIQTLPKPPPPRDAIQLLDKSGVATLVRLLFFPQKNILFKVLVNICENSKTR
AELFNLLLSVLQSGPGDLAAVDRSFAQMTTRPDLVAQRSVEALTYIVNANELSSLFFLTEHELPYALRKTKKGK
GKEKQPQTHYPIVLLLGLLDRQSLLKTTTTMDSVVTLLSTVTKPLVGLKKVVLQNPPQIPHSVLRLIVNILTIG
ECSSRTFQQSLTLISNLSYIPDGRDVIAQELKVKAQECGHNIFLDLNELMASLTSKFSSPSSDQAKLLRVLKTI
DYMYTIYESFNFTTLWRRLGDCLAAIEERPEIEHIATVLLPLIESLMVVCKHVGSKETMEDLFVSFTDAHRKIL
NSMVRTTPSLMSGSFSLLVQNPRILDFDNKRNYFTQQVHRRREHHSTLQLNVRRARVFEDSFQQFHSKDGERIK
HAKLNVRFYDEEGVDAGGVTREWFQILARQMFDPNNALFQPCAADRLTYQPNKNSWVNPEHLSFFKFVGRVIGK
AIYDGRLLDAYFAKSIYRQLLGKPVDYRDVEWVDPEYYNSLCWILENDPTPLELTFSFGRNRIFPLKEGGEQIS
VTNENKREFVQLSASFRLYSSIKEQIEHLVSGFHDIIPKDLVNIFNEKELELLISGTPDIDVDEWRAATEYNGY
TSSDPVIVWWWRALKSFNREERAKVLSFATGTSRVPLGGFVDLQGVQGVQRFSIHRAYGDPDRLPQAHTCFNQI
DLPQYSSYEMLRQQLMLAISEGGEGFGFAKSSASSGTRKKVARKLRALGKKDPVTKAKALDELNRLPVLFTHPA
RRIRLLAASVHAALLGTWLLLAHDLDRVVVTQAERSWFTERVLLDPLSVHAALNPEEQDADKSARLRYAALGAL
RWGLGQPQVRVAAWTLVLCLLGPAVLRSAWVETDPGVRSAITRPVLIFLKEYPNAWAYTDFLSYLALGSPIEGY
PSVLIVLSTIPSSIFAAFWAAIDGRALAFLGAVLECLVFMVQRPAVFICAYLLPPSRSELEGMLASLPAHAADP
SIALLEPADNRGYGPYARVVSALLRRRIANEETWVLPHALRLDEVRESRVLYALLQHLFADADTSDAEHWLLLA
RRLEKTAPQTALTIVAALTTHAPEPARLDRYRNELAADALGIKGLLLLRRLVATAPDPESDVVFLPQQRAVNLM
RVCQAWIAGAGLEGVESVMTLLFYHLAPIVQNVAGAHWDLVWDVVENNLENCSFDDDATLTTLGRSLRLIILIE
DLVKTNKSLKAWAERRSAILSLVKDLSAPRSLCRELAVSIEHLVVEAAVDTASLKVRTGYVQHLRSLGVVGKNF
IPHIFEVLNLFKPFKLSIWEVDGYHLDYYDPLSIRLFAAHLYHRALLTIPVLVRSWISDCTDKQLLSRVLDFTS
SFFSPGIIRAELALVRQAVSEERWRAWVLGVQQTQNGHIVDGLSMFTKNVKLHFAGLVECAICYSIIKPCRTCK
NRFHAACLYIEVAPVLNLA SVKTISPRPF GLEDCPAFYP TPEEFKDPMA YIRKISDKAT EYGICKVVPP
VGWKMPFVTD TEFRFKTRLQ RLNSIEASSR AKVNFLEQLY RFHKQQGNPR VSVPTINHRP
LDLWLLRKEV HKLGGYEAVK MKKWSDLGAL LGYRGPGLST QIRNSYTRVI LPYEHYCERV
RNSLCDGCDC GFHMFCLDPP LSSIPKGQWF CHTCLLETGD FGFDEGEEHS LSTLQVRDAE
FRRMWWSKHA PVSEEEVEQE FWRLVQSPDE TVEVEYGADV HSTTHGAMPT METHPLDPMA
KDPWNLNNMP IVSDSLLRYI KSDISGMTVP WTYVGMVFST FCWHNEDHYT YSINFMHWGE
TKTWYGIPGD DAEKFEAAIK SEAPDLFEAQ PDLLFQLVTL MNPARLTEAG VRVYACNQRA
GEFVITFPKA YHAGFNHGFN FNEAVNFALP DWLRLGRDCV ERYREHRKLP VFSHDELLIT
ITQQSQSIKT AIWLADSLRE MVVRELGERA RVRLGMKEVL EEADPEDQYQ CAICKMFCYL
SQVTCQCVVC ADHVDLLCEH LTLRLRFSDG ELQDTLSKVV ERSEVPGAWK KKLQKVLEES
ALPPLRSLRA LLAEGERINY PLPELPTLRK CVQRANEWVD SANGFIIRKQ SRKRDRPDRT
LSDLYALLRE AENLGFDAPE LGVLEALARQ AEDISANAQV LCSERLLLDA SSLNVYLDPV
LEIEKVVSRE RLLDELLDEV RRLLTRARAC DLAPDNKYVQ RLEARHRAGA DWEERARMML
EQPGKTIEEL PTDPTIHDRL KSALAKAMDF DKQAKGWATA DKPTPSQLSG FIARCEKDFH

IPSVKNLSHI AQYGREVEDK CDAVLRNRYP HKDVFKEIEE WGRYARQHLS LSLPFFQQLD
KQLNLHYRWL EDLPWYCHGS EILDDVIEAT RPEDDLPPTD EYFTCICQDP VRPPPPGGQS
DAVQCDHCFA RFHGKCAANG GSCPFCDHNH WNGKIHKERN WHFCFLPLIL TRAPDLTHNY
SDTWKQLEII VHRVDRLSSV IGHFLTFASN QKTSLLPQVR HYMRKLYKIQ FAVSPSREIS
FGLDLAGLHR ILAGLPHKKR RKPKLVFGPD ADEDWYDGTR CVCRRNACKR RYHAPCVWTC
PLCCVRKNKS YPFAEVRVKM HGQELIVERM PLPRPDAIVL DLVSFTP
>Fibulorhizoctonia sp
MRRTTQDQSILLSGETGSGKSENRRLAIKAFLELSVSNPGKKGSKLAAQVPASEFVLESFGSARTLFNPNASRF
GKYTELQFSRGRLTGIKTLDYYLERNRVCGAPNGERNFHIFYYLVAGASPEERQHLHLGDKTTYRYLGARDANR
FDQLKVALKAIGLSKRHVAQTCQLVAAILHLGNLEFMIDRGKNEDAAVVRNTDILEIVAEFLGIQAHDLESALS
YKTKLVKKELCTVFLDPDGASDNRDDLAKTLYSLLFAWLNEHINQRLCRDDFATFIGLFDLPGPQNMSRPNSLD
NFCMNFANERLQNWIQQRVFDSQTEEYAAEGISRFVPTVPYFDNAECIRLLQNKPGGLIHIMDDQTRRAPKKTD
QTMVEAFGKRWGNHSSFKVGSADRFPTFTVSHFNGPVTYASDGFLARNADALNPDFVSLLRGGSMNPFVKGLFS
GKAIATQAHPRNEDTIVSAQQPVKPMRAPSTRRKGTPCVAGEFRSALDTLFETLNETQAWLVFCLNPNDSQMPN
QIEGRSIKGQVRSAGLPEVAKRSVCVFEVGMEPEEFCDRYRLALNDLGIIEGDEDVVMGAHKVFLSQMAFHALE
DRLRGTDVEEQKRNRMRDAEASSQALPLVANASPFDDFDNRSRLTSQHESYAPSRNMFQNTDGEIHEGEVTEIV
KETANRRKWVALCWALTWYVPNFALTHIGRMKRLDVRQAWREKLAINIIIWFVCGCAIFVIAFLGDVICPTEHV
FSAAEFASHNTAPYTSIRGEYAGVPSDNIFPVQVSALCNGVTGTVSPYVTLSSKNTDVNQVYHDFRAYTLDPRP
DWYFEQMTQMRWNNRVGFLGITTKLMKSMASAGSSIGIYNGLSMPAIMFNSTSSTFLTFKPKESLQFTFLTIGS
RGDVQPYIALAKPLMADGHGIEYGYVGGDPAELMRICIENGTFTLSFVKEGLLKFRGWVDDLLATSWEACKGSD
VLIESPSAMGGYHIAEALGIPYFRAFTMTWSRTRAYPHAFAVPEHKSYVMFDQVFWRATSGQINRWRRNVLHIG
GTSLDKMEPHKIPFLYNFSPMIVPPPLDWPEWIRVTGYWFLDDAEVGSKKWSPPPELEAFIDNAHALGKKVVYS
DPKAMTRSVIEAVVRSGVHAILAKGWSDRLEAEDPLPKQIFPVNSVPHDWLFAGIPTIIRPFFGDQFFWADRVE
ALGIGCGVRKLTVETLTEALRAATTDIKQIDRARSVGEHIRAENGDRPREVFEPLRLACETRSEKLMIASLDCI
SKLISYSFFAELVDLVAHTITACHTEVTPETVSLQIVKALLSLVLSPVVLVHQSSLLKAVRTVYNVFLLSTDPV
NQMVAQGGLTQMVHHIFDLFVKDAFLVFRALCKLTMKALNTESERDLKSHAMRSKLLSLHLVLTVLNSHMALFV
DPTAIIYSSSSNEATTFVQAINQYLCLSLSRNAVSPVPQVFEISVEIFWRVLAGMRTKLKKEIEVLLHEIFIPI
LEMRTSTLKQKAVILGMLSRLCQDPQALVEIYINYDCDSEAVDNIYEHLMNIISKIGTPALSTYTLSVSGSTMG
LSESQLKRQGLECLVGVLRSLVAWGIDAASRFESAKQKKTILVEGVKKFNFNPKGMQFFIESGFIPSNDPPVIA
EFLLTTDGLSKAMIGESDEHNVAVMHAFIDQLDFKDMTFLQALRALLAAFRLPGESQKIDRIVLKFSERYIANN
DCAYVLSYSTIMLNTDQHSPQVKKRMDKDEFVRNNRGINDLPEELLHEIYDSIRTNEIRMKDEVANVGRDLQKE
AYVTQSNGMANDQFFSASDFVHVKTMFEVAWMSFLAGLSGPLQETDDLEVVELCLDGFKAFVTTLAKFTFLNNL
GEMKTKNMEAIKALLDIAVTEGNNLKGSWQEVLMCVSQLEHMQIITSTKGRKLPTEELANESRSTHITVAADMV
FSLSHYLSGTAIVDFVQALTDVSWKEIQSSGMSQHPRLFSLQKLVEISYYNMSRIRLEWSNLWDILGEHFNRVC
CHNNPHVGFFALDSLRQLAMRFLEKEELSHFKFQKDFLKPFEYTMTHNQNPDVRDMVLQCLQQMIQARVQNMRS
GWRTMFGVFSAASKVLTERIANSAFEIVTRLNKDHFATIVRYGSFADLTVCITEFCKCSKDDPMIRYWFPVLFG
FYDIIMNGEDLEVRRLALDSLFSTLKTYGKTYTVEFWDTVCQELLFPIFAVLKSSQDLSRFSTQEDMSVWLSTT
MIQALRDLIDLYTFHFDILERFLDGLLDLLLAENDTLARIGTSCLQQLLEKNVTKLSPARWERVATTFVKLFRT
TTPHQLFDETLRVERRRTFKQIIVKCVLQLLLIETTNDLLRNDEVYNTIPPDHLLRLMSVLDHSYQFAREFNED
KDLRTGLWKESSSAATLVHVLLRMYYDERPEHEAVRPQIATQLMPLGLGVLQDYTKLKADTQAKNITAWTPVVA
EILEGFCKFDEKSFARYLPAVYPLVTELLSRDPEIRQGVRLYFVRVGHAQGIQRQAIVAAAQAKYGNEIVAPIL
QRIFPTLSLPPNTSLVQTLIQLGPDITSDPDTVRALLLRFGISEVNPPRDVQVVEIISALARLAADGTAMCDVG
ALVRALSSFPVNLDWAAVIKSFDWPDRGVDTATLKLLIAILVNCPRAEPHAVTGFWLTWTNPMYQLRLLDALLS
LPADTFNFVTLPGRRVVTVDDVAGASPTIKSLAANVQGHTWNSLDLFEVLVKLGDSDVMEIRNTVREMLDKAVK
ISAELVHMGILQVPHWGEVRLEYSRKLLAMFLGGHPNHQLVFMRIWQIEPTYLTDAFRDFYEESPLNITRILDI
LESLLQVQPFIFALDEYLNLDKWLADNVNNHSGDFLHAVIQFLDIKMESEKVARISDPAVRTMPLSPQTITIFL
RMLRSNSAKMHNDDVDYCLEVRNACLQIHPRLMDVEPGFTEVRYSADIENEVDSIYKQMYEEQITIDEVIALLQ
RTKNSHDPHDHEIFSCMLHFLFDEYKFFQYPPAELALTGYLFGSLIQYQLVDYIPLGITIRYIIDALTCPPETS
LFKFGIQALKRFKDRLSEWQPLCQALLNIPHLMEAHPELASTIHRADVSDRILFIVNNLAPSNFDSKLKEMKEQ
FSTEPNNHSLYLRFLDALDRQALSKYVTQETLVKSATMLNSERTLQNSSERATLKNVGAWLDVPIKHKNLSFKD
LLIEGYDNGRLIVAIPFICKTLEPCAKSKVFQSPNPWLMAVVSLLAELYHYADLKLNLKFEIEVLCKGLDIDLD
TVDATTVLRNRPRAVGAHIESILASLAHHVIVSQQLALHGNHSFKRAVQLAVDRAVREIILPVVERSVTIAGIS
TRELVAKDFATEPSDDKLRKAGHLMAQKLAGSLALVTCKEPLRSNLGTHLRQHLAEHGFAEVQVLAILAADNLD
VACAAIEKAAMERAVSDVDEGFAASYEARRRHRELRNGSNFALNLPDPLQIKANGLQPHQAGVYEDFFSATIRD

LEAVMIQLPQSLAALPPNHDIRHLVRQILFLADRHRTPLLMSQKIVQLLYKTSSQLGREVYVALLDQLCHSFED
VAKEAITWLLYAEDERKFNVPVTVTLLQSGLVNISLQDQQLAKEPRPSLLNFAAALIRECLSTDASQSQFAYSI
EWFQQWVAIFQRSHSPEKSFVPFINQLTKQGILKVEDSSFFFRVCAESSVNSYVKHIAAGDFEYAFQALDAMSR
LIVYIIKYHGDANNDQAKVHYLTKILSIFVLVLANLHEEQGFQQKPFFRFFSSLINDLHSIEAQLGSAYFQLLI
AISDTFSSLQPTYFPGFAFSWMCLISHRLFMPKLLLSENREGWSAFHKLLLSLFKFLSPFLKEADLQHASRDLY
RGGLRLLLVLLHDFPEFLSEYYFTLCDVIPPRCIQLRNIVLSAFPPSITLPDPHLRSVKMGPIPPVLSDFTSGL
KSGDLRSYLDQYLLNRGTPSFLPSLKDRLESYNLPLINSLVMYIGVSSVAQAKARSGSALFVPSDDGVVALQYL
ATNLDVEGQHHLLSSVVLHLRYPNAHTHWFSSLLLHLFLEVKDDRFREVMTRVLLERFIVHRPHPWGALVTFIE
LLRNPKYDFWSKEFIRVAPEVTILLESVGHRRIYLQLCFRNVADLLNVRRDIVPLALANSAKRDAVDAYAEVVD
PREGIIDVREYDVPYYLRVAIDNEIRVGVWYSVTFNAGQPSFERVKRADPVVMAYDIETTKAPLKFPDQAIDQV
MMISYMVDGQGYLITNRDIVSEDIEDFEYTPKEGYEGPFIVFNEADEAATIMRFFEHIREVKPTVMASFNGDFF
DFPFLDARSKANGIDMFLETGFAKDSEDEYKSRACAHMDCFRWVKRDSYLPQGSQGLKAVTTSKLGYNPLELDP
ELMTPYAVEQPQILAQYSVSDAVATYYLYMKYVHPFTFSLCNIIPLNPDEVLRKGSGTLCETLLMVEAYRGQII
MPNRHEEAHGSMYDGHLLASETYVGGHVEALEAGVFRSDIPTDFNVVPSAVQLIDDLDAALKFCVTNYDEVKAE
ITAALELMRDNPKRTDKPLIYHLDVAAMYPNIMLSNRLQPDSVVDEAVCAVCDYNRPGKTCDRRLDWAWRGEFF
PAHRDEFNMETFPPKFPGKPQRRFPELTEAEQTALLHKRLGDYSRKVYKKTKDTKVETREAIVCQRENPFYVDT
VRAFRDRRYEYKGLLKTWKKNLDSITEIDEAKKLVLAHKCILNSFYGYVMRKGARWHSMEMAGITCLTGATIIQ
MARALVEQLGRPLELDTDGIWCMLPGVFPENFKFKLKNGKSIGFSYPCTMLNHLVHAQFTNHVYHDLDPETGEY
IVHSENSIFFELDGPYRAMILPSSKEEDKLLKKRYAVFNDDGSLAELKGFEVKRRGELQLIKIFQTSIFEKFLL
GSTTEECYAAVAQVADQWLDVLFSKADTLGDDELVDLIAENRSMSKTLAEYGGQKSTSISTAKRLAEFLGDQMV
KDKGLACKFIISAKPMGAPVTERAVPVAIFSAEESVKRVYLRKWLKDNSLANFELRSILDWNYYIERLGSVIQK
IITIPAAMQKVSNPVPRIRHPDWLHRRVAGAVDKFKQNKVTDFFAGRFTLWLSVDAKLVSVSLRIPRTFYLNLK
SPEFYSYEKVVRNLPHDIRGANLFMDLTNDPNVDGVFEQQTLNRAENIGFDLDQLDRKFIFIYHASSSTGNLHV
FAVFVPTGVKLHVVDPAVHRQNTTYHSKDITALKAINRELSFMEDMSYTLVISSTKEQAYFEAHIPRLTKFPVL
SMPKTKMAHALDFNWQTHVGPKMLNRYLRLGQWLGRQISWADYYPLFLADINFARRLNEQDMIIWWSTSDRPDL
GGIENDRRPIEDLPNTEFVSPGCYPNVCLEVTVRNLAINSVLHSVMVNELEGSGGATAFDSAQRDLTLGESSVS
VQTFGILKTMIKTWLLDKISPAAVAVDHFWRWISSSASHMFDPSLHRFIHGLMRKTFIQMLAEFKRLGSHIVYA
DFSHILLATTKPPGTAHAYATYIMTAVTSNELFQHIYLNTERFYDFLLFMDQSNMGGMVCEDPLAITPPEELSI
EMRWNIASFLPLVIQSEFNSIIQFFIVELFRIRQKKTKEMDAMREFIARRLTRKVLRAVFPTLAGSHLRLENPV
LEFCKFACAVFGLAKEYQVEIGLLKRSLLELINVREFSSEATFRNPCEPLKLSNVPCRHCDALRDFDFCRDPDL
SAWLCDTCGGDYDRTTIELMLMEVVYSLERSFAQQDLKCAKCKQIQSDNVSRYCCSGSYQHTVSKVDVRRKLKT
VVNVAIMHNLGRLFEVEHLRYATLATSANLTTQKEIVTIKTVRNVTEYNEQYVEKRLLVSIIWAFSGDAKGEWI
AWQTRVPVIEILYSWLSEHKPLLLCGPPGSGKTMTLFSALRKLPDMEVVGLNFSSYRKTPNGVILAPVQIGRWL
VVFCDEINLPAADKYGTQKVISLVECGGFWRASDMSWVKLERIQFVGACGRVPLTHRFLRLVMVDYPGEVSLKQ
IYGTYNRAVLKVVPSLRAYAEPLTDAMVSFYLASQKRFTTDIQAHYVYSPRELTRWIRGIYEAIRPLEVLSVEG
LVRVWAHEALRLFQDRLVNDEERLWTDEHIDAAALEHFPTINILFSNWTSRNKARLRVFYEEELDVPLVLFNDV
LDHVLRIDRVFRQTTLSRFVAWMNGLSIFQIKVSNKYTGEDFDLLANAEVPGLFEGFTMNPPQNGLASRAATSP
ALFNRCVLDWFGDWLASYNPPTNFPIAYRVVNALVYVHSSLHQINQRLSRRQGRYPRHYLDFINQYVRLYNEKR
DELERHLHVGLDKLVTQVEELRKSLAIKRKLQRMVADQQEAEQKKAASIEIKAALREQDMHIEQRRAVVMADLA
DAEQMTKQLRETMKKDFLSRPSYNFEAVQRASKACMIAELEAKITQYKDEYALLIRVQGKVERSMKLLESLSRT
FDAEMSTIVGDVLLSAAFLAYGEWSNHLAEASITFKPELSLTRLSWQSKSLPADNLTTVTSFLDEAFLKVLESA
LRFGNPLLIQDVEHLDPILNEIRRTGGRVLIRLGNQDIDFSPSFPSVEFSPDICSRVTSQSLDQVLKVETDLMK
VQGEFMLRLRTLEKLLLQALNESTGNILDDDKVIDTLETLKREAAETDVVMKEVEEVTAEYLPLAQACSSVFFI
LEQLNLVNHFYQFSFLDIFDYILLHNPNLKNILLKDLFLVVYKRTSRALDELEFLLESGLEGYAKQSLFKPVQN
HITEHESEWVPFLLIVKCLRPDRLLQSVPGYDASYRVENQEGFALADQAIAAASRQGSWVLLKNVHLAPSWLGE
KKLQTLNPHRNFRLFLTMEANPSIPVNILQSRIIMNEPPPGIFLLAWFHAIVQEDMAAAFGTIDTWLNAVAKGR
ANIDPAAIPWDALRTLVKQSVYGGRVDSDFDQRILDAFVDTLFTPSAYNVDFPDGTKIEHFLSWPAWLSLPPTA
ERRKMRMLQDDDLERCREWLSQLPSVFNILSKQSGDNQDPMYRLFSREGSVGKLLGQVQKDLADVLMSSLTKGT
IPTHWRRYKVNKIPNLARRLVQLNEIAGLDNGGLFFPEAYITATRAVAHRKRWSLETLTMRLDIEKVNDPGLAL
EGAAWSTDVLPDTAFTLPPLHGRTLDEHFHAIGAAAAEPYRALAAAFAEGAVCFDVETMPNEHAYAVLATAATA
DAWYAWISPWLLGRVVVGHNVSYDRARIRDEYALAGSATRFVDTMALHIAVKGISSHQRPAWMKHRKGKSRWED
ITSVNSLAAVAKLHCGIALDKTARSDFMSATPAQVRGDVRAYLDYCAGDVHATHRVLAAVLPQFLARCPSPVSF
AGMLSMGSSFLCVDEGWEGYLADAERTYRDLDRGVKARLLELAEAASRGDPWLSQLDWTPKVPKWYWDLSKPKK
GAVDITVRTRVAPLLLRLSWLGWPLVHSREHGWAFRVRLAPLAFYDPADDALFYKLPHKDGAAANVGNPLAKPF

MKFAQDGTLTSPEAAAAEALDMNAQCSYWISSRDRIMNQNVVWQKRGVIIPQVIAMGTVTRRAIERTWLTASNA
KANRVGSELKAMVRAPAGYAIVGADVDSEELWIASCMGDAQFGLHGATAIGWMTLEGTKAAGTDLHSKTAGILG
ISRDQAKVFNYSRIYGAGMKHAMQLLVQSNAGMLPEVAQQLAQNLYASTKGKTTHRDVFGRKFWFGGTESYLFN
KLEEIALSDKPETPALGCGVTDALSKKFLPEEFGSNYMTSRINWVVQSSGVDYLHLLIVSMDHLIAKYNINARY
LISVHDELRYLVTESDKYRLALALQIANIWTRSLFAYKLGMDDLPQGVAFFSAVDVDTVLRKEVDMPCVTPSQP
HPIPAGESINIAQTLQRTHGGSLHADGYQKPDCMKHRSQGAAFLQAQATTDFAEIKHLATEIIIKLLKTSSEDL
PDVLAQINSWKWPRSDLNAWIKVLNKFDAIFEHVIQKHDIDKLQVNAFDAVTKKTVCEILRFERLLLENSTNRK
LFSSYDRINSLLLTSDLDVLVLSLHLLLRPSQQYSAQPAVTSALNISTPRLKSLAKRWPNLREYDVSLVDLVSN
GRSVVDALPSEAREVNFTFYRYIDQPAIESQNEMKILADVIEKHSVPDEKFELLCRIRAAAVLANGREKLVIVR
LLAIAIYGHTHSESSAMSSLFLYEPDLIPHLAELLQLDRGVRKTVADIADPDCKLPHSFVEALLTFVTYTASHQ
AGGNMVVGAGLVPLLVQVIGNTIPERLAVVSKTMQLVDNVLFSFANAFQLFCNGRGVEALVDRIEFEVDGELPV
ARAAVLKHTLRSMHRMMQSSGTAEGLRGLIDSSILKSLKKIIENRGLFGPSVVPIAINIMATFVHNEPTSLPVI
QEAGLPEAFYKAIEAGLEPVIEVIQAIPNAIGALCLNQAGQDQLAGRPSIIPGIFAIFTSERHLKVLQEKENAV
LIGTAIDELIRHHPSLKVAVFESIKSTMGKIEDLANTQVVGKDIEHWYKLDNIVVSFIDIVGRFLEGLFQTTDG
LERIGRFTALPCIPYDYANSLLVQIMRNMTDVAPNETLAFLQNLVKSSLAETQEFWGTMDDHSNRQFRNLITLH
IRVALLSDVFSTYAHGRAAISILLGTLHRACIWESILLKAGLNAKALQHLTHGLPLAPFFQAIVKRRNPDPVQR
RQIMGSASNIAQVMLKHIYFSIMLGTITVLLVDELLSEFYRAGGQELAHAYGGLKVALRLIHTLISPKSLQESP
QTLLMASRKKDTDPGYFEAHNFLVRVRVAFLPLLKEMWEAPWLAPAPLGVVKSVVQAVMEMMAGESEPRSAAER
ALIRMHNNVNAATELLLSQPELNDAREPLIGSIPKQALQLVDAHPTIKSFSPHAHGVKEEPLSMRCRLLALVLP
GVPKWLAAHLLVTEALLAMGEQARTITLPLSAGPSYPEARNIVFDYCLRVLAIADLPRDEFLAVLRLLVILTQD
HRVACQFVQRDGVALIGSQSYVAIVLRHVIEDSSVLQQIMKQEIKHFFAQPRVLDVGNYVRHCSAMALRNPLIF
VQLTKSLCKLQSAEILVHYLIGELMRVSKYACFIMQCLTELLFSYDSCKVALLSYAPKKYRMTVLQFLLSDLVS
FGGINSQHRHLLCNWAMSIVVALCVDTSKDVSHDLISVRKFVLEAVSRAIKDLSQSGTIDSRYGRLLALSDLCH
RLLTVRFETPTHIAKVMLEKNFVATLTNALAEVDLNYPNVRSLVAGILRPLEHLTKIAIKMSREETPDLYRNSA
LGMYAEATTHPLLLDKPDGPEFDPQLTLQRWGDEVKTLHGKFVSERVGKLANHVVLALLPAAIERVTVMIHGSA
VDITDTGIDPTFLEALPDEMREEVLNQHVRDQRAARVERPADSTISDEFLDALPPEIRAEIIQQERLEPTDMDP
ASFIASLDPQLRQTVLLDSDDGFIQSLPKLPVPRDAIQLMDKTGVAVLVRLLFFPQKNHLFKVLLNLCENTKTR
MELFNLLLNILQDGTGDLAAVDKSFAQMSVRPELVAQRCLEALTYIVGANEMSSLFFLTEHEIPPGLRKSKKGK
GKEKQPQIHYPVVLLLGLLERQSLLKTPSTMESVVGLLAIVTRPLTSLKRTLLTHPPQIPHAALRLIVNILTVG
ECSGRTFQQSLALIQNLSHIPDARDVIAQELKAKAQEFGQSLYVDLDELATALASKFSPASSIQAKLLRVLKTI
DYMYSIYESFRFTPLWRRLGDCLAVIEEKPDTEHIATVLLPLIEALMVVCKYVGSKESMEDLFVSFTDAHRKVL
NLMVRNNPSLMSGSFSLLVNNPRVLDFDNKRNYFTQQLHRRREHHNTLQLNVRRARVFEDSFQYLQRKTGDQIK
HGKLSIRFYDEEGVDAGGVTREWFQILARQMFDPNNALFQPCAADRLTYQPNKNSWVNPEHLSFFKFVGRVIGK
AIYDGRLLDAYFARSLYRQLLGKQVDYKDVEWVDPEYYNSLCWILENDPTALDLTFSFGVSRIAPLKEGGDTLP
VTQENKREFVQLSAQYRLYASIKEQIESLLTGFYEIIPKDLVTIFNEQELELLISGTPDIDVDEWRAATEYNGY
NSSDPVIVWWWRALKSFNRDERAKVLSFATGTSRVPLSGFVDLQGVQGVQRFSIHRAYGDSDRLPQAHTCFNQI
DLPQYSSYEMLRQQLLLAINEGGEGFGFAKSSATSTTRKKHARKLRRLGKKDAVTKRRALEELKHAPLLLLHPS
KRIRLLAATLHAAILGAWRMSAHDPDKHVASVASTSSFLCRALLDSNGLWAALNPGEGETDRNARLRIGALGAL
ARGFTQPPVRRAAWAVVATLLSRVALRSAWIESDVGVWRALRDPLLVFLRAYPEAWAYASFLAFLPHPAPGATY
PALVVLLSTLPPSLFDALWAGLGGGFGMLVKAILECLVFVVRGPSVFGLAFLLQPTRFELDNMLVALPSEPIDS
SLGVLDPLDARGFSAYARVVYAVLDRQLARKNLWALRHLLAFEGARGCRVLFRVLHHFFANASKEDADAWFTLA
RKVEKTAPRTSLTIIAAITQFAPEPSRLDRYRNELAADLFGVPGLHTLRKLAAVAPDPESDVVFLPQQRTINVM
KTCQQWITSDIDEEVESEMTLIFLHFAPLLQNVPGAHWDLVFDVIENNLESCSLSDNTALVTLARTLRLIIAIE
DLAATNKALRAWHERRISALTLVRNLSTPRSTCRELVLYIEYLVIESGVDTASMKVRSDYVNQLRNLDLIAMRF
IPSIFTLLNLYKAFKLDPWAVDEYYIEHYEPLSLQLLASHLFYRALLTVPSLVRKWLHDCTDRQLSSAVVAYTS
LNFSPVIIRTELLEVKSPVLEDRWRAWVLGVQQIQNGRIVDGLRHFKKNVALHFEGQVECAICYSIIKPCRTCK
NRFHAACLYIPVSPVLDMS SVKTDAPRPF GLEDCPSFYP TEEEFKDPMA YVRSISDTAQ NYGICKVVPP
VGWKMPFVTD TEFRFKTRLQ RLNSIEASSR AKLNFLEALY RFHQQQGNPR VSLPTINHKP
LDLWLLRKEV HKMGGYDEVK GKKWSDLGRI LGYGGPGLST QIKTSYTRVI LPFEQFSERV
KNLLCDGCDC GFHMFCLDPP LTAIPKGQWF CHTCLFGTDD FGFDEGEEHS LSSFQARDLE
FRKLWFESHP PVSEYDVESE FWRLVQSPAE TVEIEYGADV HSTTHGGMPT LESHPLDQYA
KDPWNLNNIP IVSDSLLRFI KSDISGMTVP WTYVGMIFST FCWHNEDHYT FSVNFMHWGE
TKTWYGVPGD DAEKFEAAIK KEAPDLFEAQ PDLLFQLVTL MNPKRLVDAG VRVYGCNQRA
GEFVVTFPKA YHAGFNHGFN FNEAVNFALP EWLSYGRDCV QRYRDHKKLP VFSHDELLIT

ITQQSQTIKT ASWLLGSLKE MTDREMARRV YARLGLAEIL EEEDPEDHYQ CTVCKMFCYL
SQITCQCVVC IDHAEHLCDH LIMRKRFSDA HLLDTLNKVA ERAAAPSTWR GKLGKLLTES
ARPQFKSMRA LLAEGDRIGY HIPELAPLRK CVARGNEWMD ASNTYLIRKQ SRKRDRPDGG
LEELYALLRE VENLGFDCPE IASLQALAQH AEEAKAQARR LLCERLLLQG TSINVHLDEL
LEVQKIVTRE QLIKELLEDI RHLLTRARAC NLPPDNEHMK LLADRQRAGD NWEERAKSIL
AQPFKTIDEL PIDHAVLDRL LSALSKAQEL DKQAKAWLVP EKPKVADVMR LVIKAEKEYS
IPSISDLKRT AEIAADLESR CDDVLKNRYQ HGDLFDSMRK WKAYAVDHLT FALPMFDKLD
KQLIVHYKWL ESLPWYCEAQ KLIADVLDAT RPEDDNPPND EFYTCICTAA VRPPPPGILS
DAVQCDHCFA RFHGECAKSG GSCPFCDHNH WNGNIHKERS WHFHLLPEVL HAAPEITKNY
SEHWKQLEII VHRVDRLTAH IGQFCMFAQN HLAHFIPHVR HYMRKLYKLQ FQIGATREES
YGLDLASLHR VLAGQKMKKR RRPKFTFGQD LDKDWQDGTR CICRGRTCNK TYHTGCVFIC
PLCCLRKNIT YPYSDVRVKN FSKDIVYVKM LPPRTQTLFV ELVRFSP
>Piloderma croceum
MRRTSQDQSVLLSGETGSGKSESRRLAIKALLELSVSSPGKKGSKLAAQVPASEFVLESFGNARTLFNPNASRF
GKYTELQFSRGRLSGIKTLDYYLERNRVAGAPSGERNFHIFYYLVAGASPEERQHLHLLDKSVYRYLGSQDANK
FDQLKVALKTIGLSKRHVAQTCQLVAAILHLGNLEFMVDRSRNEDAAVVRNIDILEIVADFLGVQPYALEAALS
YKTKLLKKELCTVFLDPDGASDNRDDLAKTLYSLLFAWLNEHINQRLCRDDFATFIGLFDLPGPQNMSRPNSLD
HFCINFANERLHNWIQRRLFESHVDEYAAEGISRFVPTVPYFDNAECIRLLQNKPGGLIHIMDDQARRAPKKTD
QTMVEAFAKRWGNHSSFKVGTLDRSSTFTINHFNGPVTYSSEGFLERNLDALNPDFVSLLRGGSINPFVKSLFS
GKAIATQAHPRNEDTIVAAQQPVKPMRAPSTRRKGTPCVTGEFRSALDTLFETLNETQAWFVFCINPNDSQLPN
QLEGRSVKGQVRSAGLPEIARRCVNTFEVNMTPEEFCERYRAPLVELGVIEGARDVVLGQHKVFLSQAAFHGLE
DHLRSSDTEEQKRNRMRDAEASNQVLPLVANASPFEDYDGRSRYTTHRESYAPSRNMFQNADGEIQEGETTEVM
KETSAHRKWVALCWMLTFWVPSFLLKWWGRMKRPDVRQAWREKLALNMLIWFICACAVFVIAVLGDVICPTQHV
FSTSELASHSNNVYTSIRGEYGGTSADNIFPVQVSALCNGVTGSVSPYVTLDSSNTDVNAQYHDFRAFTADSRP
DWYFESMTEMRWNARVGYVGYTPKELKNMANTGSSVGIIDSLSLPAIMFTSASSTFLTFKPKESLHFTFLTIGS
RGDVQPYIALAKALLADGHGIEYGYVGGDPAELMRICVENGTFTLAFMKEGLLKFRGWLDDLLATSWEACQGTD
VLIESPSAMGGYHIAEALGIPYFRAFTMTWSRTRAYPHAFAVPERKSYVMFDQVFWRATSGQINRWRRNVLHLA
STSLDKMEPHKIPFLYNFSPIVVPPPLDWPEWIRITGYWFLDDADVGSKKWTPPSDLEIFIDSAHKVGKKVVYS
DPKAMTRTIVEAIVHSGVHAVLSKGWSDRLEPEEPLPPQIFRISSVPHDWLFAGIPTIIKPFFGDQFFWADRVE
ALGVGSGVRKLTVESLTDALRAATTDIKQIDRAKLIGEQIRAENGDRPREIFEPLRLACETRNEKLMIASLDCI
SKLISYSFFAELVDLVAHTITACHTETTPETVSLQIVKALLSLVLSPVILIHQSSLLKAVRTVYNVFLLSTDPV
NQMVAQGGLTQMVHHVFDLFIKDAFLVFRALCKLTMKALNNESERDLKSHAMRSKLLSLHLVLTVLNSHMPLFV
DPSAIIYSSSSNEATTFVQAINQYLCLSLSRNAVSPVPQVFEISVEIFWRVLSGMRTKLKKEIEVLLHEIFIPI
LEMRTSTLKQKAVILGMLSRLCQDPQALVEIYLNYDCDSEAVDNIYEHLMNIISKIGTPSLSTYSLSVSGSTMG
LSEHQLKRQGLECLVAVLRSLVAWGTDDPSRFESAKQKKTTLLEGIKKFNYKPKGVQFLIETGFIPSKDPSTIA
YFLLTTDGLSKAMIGEAEDENIATMHAFVDQLDFKDLSFLDALRIFLQAFRLPGEAQKIDRFMLKFAERYIAGN
DTAYVLAYSTIMLNTDAHSPQVKKRMTKADFVKNNRSINDLPEELLHSIFDDIVSNEIRMKDEVANVGRDLQKE
AYVMQSNGMANDQFFSASHFVHVRPMFEVAWMSFLAGLSGPLQETDDLEVVDLCLEGFKAFVTTLGKFTFLNNL
GEMKTKNMEAIKTLLDIAVTEGNSLKGSWQEVLTCVSQLEHMQIITSGKTRRLPTEELANESRSTHITVAADMV
FSLSHYLSGTAIVDFVQALTDVSWKEIQSSGLSQNPRLFSLQKLVEISYYNMNRIRLEWSNLWDILGEHFHKVC
CHNNPHVGIFALDSLRQLAMRFLEKEELPHFKFQKDFLKPFEYTMIHNPNPDIRDMVLQCLQQMIQTRVQNMRS
GWRTMFGVFSAASKVLTERIANSAFEIVTRLNKDHFAAIVHYGSFADLTVCITEFCKVSKEDNMIRYWFPVLFG
FYDIIMNGEDLEVRRLALDSLFSTLKTYGSTYTVEFWDSVCQELLFPIFAVLKSSQDLSRFSTQEDMSVWLSTT
MIQALRDLIDLYTFHFAILERFLDGLLDLLCVENDTLARIGTSCLQQLLENNVAKLSPARWDRVATTFVKLFRT
TTPHQLFDETLRVERRRIFKQIIVKCVLQLLLIETTNDLLRNDEVYNTIPPEHLLRLMSVLDHSYQFAREFNED
KDLRTGLWKESSSAATLVHVLLRMYYDNRPEHEAARPQIAKQLMPLGLGVLRDYSKLRADTQVKNIAAWTPVVA
EILEGFCRFDDKAFARYLPAIYPLATDLLSREPEIRQGLKMYFVRVGYVHSIQRQALIAAAQAKYGNEIVAPIL
QRIFPTLSLPPNTSLVQTLIQLGQDITSDPDTVRALLLRFGISESNPPRDAQVVDIMSTLARLAAEGTTMCDVG
ALVRALSSFHVSLDWASVIKAFDWPDRGVDTATLKLLIAILVNCPRPEPHAVTGFWSTWANPMYQLRLLDALLS
LPADTFNFVSLPGRRVVTVDDVAGASPTIKSLAANVQGHTWNSLDLFEVLVRLADSESVDIRNCVREMLDKAVK
ISAELVHMGLLQVPNWNEIRLEYSRKLLAMFLGGHPNHQLVFMRIWQIEPSYLTDAFRDFYEESPLNITRILDI
LESLLQVQPFVFALDEYLNLDKWLADNVTNHGGDFLHAVIQFLDVKMESEKATRVSDPAVRTMQLSPQTITIFL
RMLRNNSVKMNPDDVDYCLEVRNACLQIHPRLMDVEPGFTEVRYAQDIESEVDSIYKQMYDEQITIDEVIALLQ
RTKSSHDPRDHEIFSCMLHFLFDEYKFFQYPARELAMTGYLFGSLIQHQLVDYIPLGIAIRYILDALTCAPDTN

LFKFGLQALSRFENRLSEWQPLCQALLRLPHLMEARPELAGSIHRADVTDRILFIVNNLAPSNFDSKLTEMREQ
FSTEPNNHSLYLRFLDALDRQVLFKLITEETLVKSASMLNSEKTLQNSSERATLKNVGAWLDIPIRHKHLSFKD
LLIEGYDSGRLIVAIPFVCKTLEPCGKSTVFKPPNPWLMAVVSLLAELYHYADLKLNLKFEIEVLCKSLDIDLD
SVEATTVLRNRPRVVGAHIESILSGLAHHVTVSQQLTLHVNPSFKRAVQLAVDRAVREIILPVVDRSVTIGGIS
TRELVAKDFATEPSDEKLRKAGHAMAQKLAGSLALVTCKEPLRSNLGTHLRQHLAEHGFAEVQVLAILAADNLD
VACSAIEKAAMERAVSDVDEGFAASYEARLRHRELRNGSNNTISLPDPLQIKANGLQPHQASVYEDFFTVLIQD
LEAVMVQLPQSLAALPPNHDIRHLVRQILFLADRHRTPLLMSQKIVQLLYKTPSQLGREVYVALLDQLCRSFED
VAKEAITWLLYAEDERKFNVPVTVTLLQSGLVDIPLQDQQLAKDPRPSLLNFASALIRECLSTDASQSQFAYSI
EWFQQWVNIFQRSHSPEKSFVPFINQLTKQGILKVEDSSFFFRVCAESSVNSYVKHVATGDYDYAFQALDAMSR
LIVYIIKYHGDANNDQAKVHYLTKILSIFVLVLANLHEEQGFQQKPFFRFFSSLINDLHAIETQLGTAYFQLLI
AISDTFSSLQPTYFPGFAFSWMCLISHRLFMPKLLLSENREGWSAFHKLLLSLFKFLSPFLKEADLQHASRDLY
RGALRLLLVLLHDFPEFLSEYYFTLCDAIPPRCIQLRNIILSAFPPTIALPDPHLRNVKMGPIPPILSDFTSGL
KSGDLRSYLDQYLLNRGTPSFLPSLKDRLENYNLPLINSLVMYIGVSSVAQAKARSGSSLFVASDDGVVALQYL
ATNLDVEGQHHLLSSVVLHLRYPNAHTHWFSSLLLHLFLEVKDDRFREVMTKVLLERFIVHRPHPWGALVTFIE
LLHNTKYDFWSKDFIRVAPEVTLLLDSVGHRRIYLQLCFRNVSDLLTVRRDIMPLALANSAKRDAVDAYAEVVD
PREGIIDIREFDVPYYLRVAIDNDIRVGLWYAVTFTAGQPSFQRVKRADPVVMAYDIETTKAPLKFPDQAIDQV
MMISYMVDGQGYLITNRDIVSEDIEDFEYTPKEGYEGPFIIFNEADEAATITRFFEHIRDVKPTVIASFNGDFF
DFPFLDARARINGIDMFLETGFAKDAEDEYKSRTCVHMDCFRWVKRDSYLPQGSQGLKAVTTAKLGYNPIELDP
ELMTPYAMEQPQILAQYSVSDAVATYYLYMKYVHPFIFSLCNIIPLNPDEVLRKGSGTLCETLLMVEAYRGQII
MPNRHEEAHGSMYDGHLLASETYVGGHVEALEAGVFRSDIPIDFKIEPSAVHLIDDLDAALTFCVTNYDEVKRE
ITAALELMRDNPKRTDKPLIYHLDVAAMYPNIMLSNRLQPDSMVDESICAVCDYNRPGKTCDRRLDWAWRGEYF
PAHRDEFNMESFPPKRPGGGQRRFPDLTETEQTALLHKRLGDYSRKVYKKTKETKIETRQAIVCQRENPFYVDT
VRRFRDRRYEYKGLLKTWKKNLDSIAEIDEAKKLILAHKCILNSFYGYVMRKGARWHSMEMAGITCLTGATIIQ
MARALVEQIGRPLELDTDGIWCMLPGVFPENFKFKLDNSKSIAFSYPCTMLNHLVHAQFTNHEYHELNPETGEY
VVHSENSIFFELDGPYRAMILPSSKEEDKLLKKRYAVFNDDGSLAELKGFEVKRRGELQLIKIFQTSIFEKFLL
GATTEECYAAVAEVADQWLDVLFSKADTLGDDELVELIAENRSMSRTLAEYGGQKSTSISTAKRLAEFLGDQMV
KDKGLACKFIISARPMGAPVTERAVPVAIFSAEESVKRVYLRKWLKDNSLTNFDLRSILDWDYYIERLGSVIQK
LITIPAAMQKVSNPVPRIRHPDWLHRRVAGAVDKFKQNKVTDFFSGRFTLWLSVDSELVSVTLRVPRIFYLNLK
TPDFYSCEKVIRNLPRDLNGANLFVDLINDPNVDGVFEQQTLNRAERTGFDLSQLDRKFVFFYHACSGNGAVHV
FALFLPAGVTLHIVDPATRRQPTTYHSKDATALRAISRELGLLENMSHTVVISSSKDQTYFDSSMPKLSNFPVL
AMSKAKGHHSLDFPWHSHVAQKMLNRYLNFGPWLDRMIQLADYYPLLLADISFARRLSQQDMVLWWSPGDRPDL
GGVENDKRPTEELPNTEFVSPGCYSNVSLEVTVRNLAVNSVLHSVMVNELEGSGGATAFDSTQRDLTLGESSVS
AQTFAILKSMIKTWLLDKISPATLAVDHFWRWISSSASHMFDPSIHRFIHSLMRKTFIQMLAEFKRLGSHIVYA
DFSRILLATSKPPGTAHAYANYITSAVTSNELFQHIYLNTERFYDFLLFMDQANMGGVVCENPLAVAPPEELAI
EMRWNIESFLPPAIQGDFSAVVQYFLVELFRIRQKKANEMDDSREFIARRLTRKLLKVVFPTHPGAHLQLVNPV
MELVKFMCAIFGLVKDYSVEIGLLKRNLLELIGIKEFATEAIFRNPCEPLKLSNVPCRHCDALRDFDFCRDIEL
IPWFCQHCGGEYDRIAIELMLMEVVQTLERTFAQQDLKCSKCKQIQSDNVSRYCCSGTYQFTISKADVRRKLRT
IVNVALVHNLSRLFEVEHLRYATLATSANLLTQKEIAAIKTVRNIIEYNEQYVTKRLLVSIIWAFSGDAKGEWV
AWQTRVPVIEILYSWLSEHKPLMLCGPPGSGKTMTLFSALRKLPDMEVVGLNFSSYRKTPNGVILAPVQIGRWL
VVFCDEINLPAADKYGTQKVISLVECGGYWRASDMSWVKLERIQFVGACGRVPLSHRFLRLVMVDYPGEVSLKQ
IYGTYNRAVLKVVPSLRAYAEPLTDAMVAFYLASQKRFTTDVQAHYVYSPRELTRWARGIYEAIRPLEVLPVEG
LVRVWAHEALRLFQDRLVNEEERLWTDENIDAAALEHFPTINILFSNWTSKNKARLRVFYEEELDVPLVLFNDV
LDHVLRIDRVFRQTTLSRFVAWMNGLSIFQIKVSNKYTGEDFDLLANAEVPGLFEGFTMNPPENGLASRAATSP
ALFNRCVLDWFGDWLPSYNPPAHFPIAYRVVNALVYVHSSLHQINQRLSRRQGRYPRHYLDFINHYVRLYNEKR
DELERHLHVGLDKLVTQVEELRKSLAIKRKLKRMVADQQEAEQKKAASIEIQAALVKQDKHIEQRRAIVMADLA
DAEQMTKLIRETMKKDFLSRPSYNFETVQRASKACMISELEAKIDKYKEEYALLIRVQSKVDRSMKLLESLSRT
FDAEMSTIVGDVLLSAGFLAYGEWSNHLAEASIKFKPELSLTRLSWQSKSLPADNLTTVTSFLDEAFLKVLESA
LRFGNPLLIQDVEHLDPILNEIRRTGGRVLIRLGSQDIDFSPSFPSVEFSPDICSRVTSQSLDQVLKVETDLMK
VQGEFRLRLRTLEKLLLQALNESSGNILDDDKVIDTLETLKREAAETDVVMKEVEQVTAEYLPLAQACSSVFFI
LEQLNLVNHFYQFSFLDIFDYVLHHNPNLKNVLVNDLFLVVYKRTSRALDELEFLLESGLESYSKQPIFKPVQN
HIMEHESDWIPFLLIIKCLRPDRLLQSVPGYDASYRVENQEGFTLADQAIATASRQGSWVLLKNVHLAPSWLGE
KKLQTLNPHRNFRLFLTMEAQPSIPVNILQSRILMNEPPPGIFLLAWFHAVVQEDMASAFGTIDTWLNSVAKGR
ANVNPATIPWDALRTLIKQSVYGGRIDSDFDQRILDAFVDNLFTPSAYNVDFPDGTKMEHFLSWPSWLSLPPTA
ERRKMRMLADDDLDRCREWLSQLPSTFNTLVKQSGENQDPMYRLFFREGSVGKLLGQVRKDLADVLMSSLTKGT

IPTHWRRYKVTKIPNLARRLAQLNDIADLENGGLFFPEAYITATRAVAHRKRWSLETLNMRLDIEKVNDPGLAL
EGAAWSTDVLPDISFTLPHLQGDNLDEHFYRIGTSAAEPWLSFSKEFAERMLCFDVETMPEYHPFAVMATAASK
NAWYAWISPWLLGRIIVGHNVSYDRSRILEEYNLNGTQNRFIDTMALHIAAKGISSHQRPAWMKHHKNKKRWED
LTSINSLADVAKLHCNIDMDKEIRNDFMTSIPSAIRDSIHTYLDYCANDVFVTHSVFAKVLPSFLDRCPNPVTF
AGVLTMGSSFLTVNESWDAYLRDAERTYKNLELSIKKRLVELAEQAWKDDPWLSQLDWTPKWPKWYWNLAKPKK
GTIDITARNRVAPLLLRLSWLGWPLFHSREHGWTFRVRLAPLEFHDPADDKLFYKVPHKDGEHANVGNPLAKPF
MKFAQDGTMTSPGDEAKEALDMNAQCSYWISARDRILNQNVVWQKWGVIIPQVITMGTVTRRAIEKTWLTASNA
KKNRVGSELKAMVRAPDGYAIVGADVDSEELWISSCMGDAQFGLHGATAIGWMTLEGTKAAGTDLHSKTAGILG
ISRDQAKVFNYSRIYGAGMRHAVLLLLQSNAGMLPDEAQKLAKNLYASTKGQSTQRDMFGRKFWFGGTESYLFN
KLEEIALSDRPQTPALGCGVTDALSKEFLPPGFGTDYMTSRINWVVQSSGVDYLHLLIVAMNYLVAKYDIKARY
LISVHDELRYLVKEEDKYRAALALQIANLWTRSLFAYKLGMDDLPQGVAFFSAVDVDKVLRKEVDMPCITPSQP
EPIPSGESLNITQILEQTNGGSLWVDGYQNPDCLAHRAKGAAFLQAQATTEFAEVKHLAADIITKLLTTPNDNL
PDVLGQINSWKWPRSDLNAWIKVLNKFDTILEDVIRDYDIDKLQSKAFTPPTKKTLCEMLRFERLLLENSTNRK
MFSSYDRLNSLLFTSDLDVLVLTLHLLLRPSQQYSAQPAVTSALNISTPRLRSLAKRWPNLREYGVGLVDLASK
GRPEVEALPSEAREVNYSFYRHIDQQNLESKEAMSLLADTVERYSVPEEKFELLCRIRSAAVLTKGREKLVIIR
LLSIAIFGHTHIEAQAMSSLFLYEPDLIVHIAELLQLDRGVRKTIADVGRLDCKLPHSFVEALLSFVTYIASHS
AGGNMVVGAGLVPLLVQIIGNKIPERLAVVSKTMQLVDNVLYSFTNAFQLFCGAHGVEALVDRIEHEVDGELPV
ARAAVLKHTLRSMHRMMQSSGTAEGLRGLIDSSILQSLKKIIEHRALFGPSVVPIAINIMATFVHNEPTSLPII
QEAGLPEAFYKAIEAGLEPVIEVIQAIPNAIGALCLNQAGQDQLSGRPSIIPGIFAIFTSERHLKVLQDKENAV
LIGTAIDELIRHHPSLKTPVFEAIKSTLSKIEDLGNAYVVPEDIQQWYKLDNMVVSFIDIVGRFLEGLFQTTDG
LDRIGRLTALHCIPYDYANSLLVQVMRTMTDVASNETLLYLQKLVIASLEETKEFWGSVDDESNRRFRSLITLH
IRIALLSDVFATYTHGRAAIGILLGTLHRACILENILLKAGLNAKALRHLMHGLPLTPFFQAIVKRRNPDTAQR
KQIMSSSIAIADIMLKHIYLSVMLGTVTILLLDELLAAFYRAGGQELVHAYGGLKVALHLIYPLISSKPLHESP
QTLLIASRKKDTDPDYFEGHNFLVRLRLAALPLLKDLWEATWLVPAPLPVVKSVVQAVMELTGAENEPRSAAER
ALARTHNNVSAATELLLSQPDLDEARRQLKAGMSKKALRLVDQHPSIKAFSPFAHDVREEPLAMRCRLLALVLP
SVPKWLASHLLVTESLLTMGEQPRTIILPIAAGPPFLEARAIMFDFCLRLLAIPDLPRDDLLAVLRLFVLLTKD
HNVACQFVQRDGISLLGSQSYIALILRHVIESPSVLQNIMKQEIKRFFAQPRIIDVGSYVRHCNPMALRDPEIF
IHVTESLCQLQHPETVVHFLVSELMRTSKYSCFLMQCLTELLFSYDSCKIAFLSYSPKKHRTATLQFLLSELVS
YGTLNSRNRHSLCNWAMSVIVALCVDSSKDVTPDLVSVRKFVLEAVSRATKELSPSESLDSRYGRLFALSDLCH
RLLIVRFETPTHIAKVMLEKNFVSTLTNALAEVDLNYPNVRSLVAAILRPLEHLTKIAIKMSREETPDLYRNSS
LGMYAEATTHPLLLDRFEGREFEPMSTLQRWTEEVKILHGKFVSERVGKLGNHVILSLLPAAVERVTVMIHGSP
VDITDTGIDPTFLEALPDEMREEVLNQHVRDQRAARVERPPDSQISDEFLNALPPEIRAEIIQQERQEPAEIDP
ASFIASLDPQLRQTVLLDSDDGFIQTLPKLPPARDAIQLLDKGGVAVLVRLLFFPQKNHLFKVLVNLCENAKTR
TELFNLLLNILQDGTGDLAAVDKSFAQMSFRPELVAQRCLEALTFIVSSNELSSLFFLTEHEVPPGLRRSKKGK
GKEKQPQIHYPVVLLLGLLDRQSLLKTPSTMESVVGLLATVTRPLTSIKKILLTNPPQIPHAVLRLIVNILTVG
ECSGRTFQQSLALIQHLSYIPDARDVIAQELKSKAQEFGQSLYADLDELATALAAKFSPASSIQAKLLRVLKTI
DYMYSIYESFRFTPLWRRLGDCLGVIETKPDTEHIATVLLPLIEALMVVCKYVGSKESMEDLFVSFTDAHRKVL
NLMVRNNPSLMSGSFSLLVNNPRVLDFDNKRNYFTQQLHRRREHHGTLQLNVRRARVFEDSFQYLQRKTGDQIK
HGKLSIRFYDEEGVDAGGVTREWFQILARQMFDPNNALFQPCAADRLTYQPNKHSGVNPEHLSFFKFVGRVIGK
AIYDGRLLDAYFSRSLYRQLLGKQVDYKDVEWVDPEYYNSLCWILENDPTALDLTFSFGVRRIAALKEGGETLP
VTQDNKREFVQLSAQYRLFSSIKDQIENLLAGFYEIIPKDLVTIFNEQELELLISGTPDIDVDEWRSATEYNGY
TSSDPVIVWWWRALKSFDRDERAKVLSFATGTSRVPLSGFVDLQGVQGVQRFSIHRAYGESDRLPQAHTCFNQI
DLPQYSSYEMLRQQLLLAINEGGEGFGFAKSSASAGTRKKHARKLRLFGKKDAVTKRRALEELQHVPALFLHPS
KRIRLLTSSLHMSLLGTWCMASHDIDRQVSTHAHRSWFVQRALLDPGGVYLYLNPDENEQDRKARLRVSAFGAI
TWGWNQPVVRKSAWTTLQALLSTAVLRSAWVEPDAAVQGVMWQPLLTFLKEFSSAWAYREFLQFLELGSPSQGY
PTIVIILSTIPSPIFTSFWAAIDGRALAFLSSLLECTIFLIRRPCVFIFAYLFPPSPGEVDQMLRAMPSDPIHS
SLAVLDPLDNRGYSAYARLVCALLDRQAARRNFWALQHFLALDNVMDSRILRRVLQPVLSDVSMDEADQWMILA
RRLEKTAPQASMAIVSSITQFAPEPPRLDRYRNELAAGLLGIPGLLSLRKLSATAPDPASDVAFLSQPRAVNVV
KAFQQWITSDVDEEVESEMTLIFIHLAPLLQNVSGAHWEFMFDIIENNLENCSFADDITLVALARTLRLIVAIQ
DLALTTKTLRAWEKRHISILTLVRDLSAPRSVCRELVLSIEYLVIELGVDTASLRVRSGYINHIRNLDIIATHF
VPSILGLLELYKAFKLDFWAVDEYYLDFYESISLQLLASHLFYRALLTVPSLIRAWLLDCTDRQLSSSVVSYTS
QYFSPVIIRTELAHVKSPVLEDRWRAWVFGIQQIQNGRIVDGLSLFKKNVALHFEGQVECAICYSIIKPCRTCK
NRFHAGCLYIPVSPILDLS SVKTRGPRPF GLEDCPAFYP TVDEFKDPMA YVRSISTMAK NYGICKIVPP
AGWKMPFVTD TEFRFKTRLQ RLNSIEASSR AKLNFLEALY RFHQQQGNPR VSLPTINHKP

LDLWLLRKEV HKMGGYDAVK GKKWSDLGRT LGYGGPGLST QLKNSYTRVV LPFEQFSERV
KNLLCDGCDC GFHMFCLDPP LTVIPKGQWF CHTCLFGTDD FGFDEGEEHC LSSFQARDLE
FRKLWFESHP PVSEYDLENE FWRLVQSPHE TVEIEYGADV HSTTHGAMPT METHPLNQYS
KDPWNLNNIP IVSESLLRFI KSDISGMTVP WTYVGMVFST FCWHNEDHYT FSINFMHWGE
TKTWYGIPGD DAEKFEAAIK SEAPDLFEAQ PDLLFQLVTL MNPQRLTDAG VRVYGCNQRA
GEFVITFPKA YHAGFNHGFN FNEAVNFALP EWLPYGRDCV QRYREHRKLP VFSHDELLIT
ITQQSQSIKT ATWLIDSLKE MVDREMGGRA YARLGLGELL EEEDPEDQYQ CTICKMFCYL
SQITCQCVVC IDHAEFLCDH LIMRKRFSDA DLLETLNKVS ERASLPAVWR GKLNKLLMES
ARPQFRSLRA LLAEGERINF FIPELAPLRK CVTRGNEWMD AANTFLIRKQ SRKRDRPDGG
LDELYALLRE VENLGFDCPE IGNLQTLGQH AEEAKHKARH LLCERLLLQG TSINVHLDEI
TEVQKIVTRE QLIRELLEDI RHLLTRARAC NLPADHRLMK LLEDKQRAGD NWEERAQSIL
AQPFKTIDEL PIDPAVLDRL LAALAKAQDL DKQAKVWLVP ERPKVQDVMR LVQKAEKEYS
IPSISDLKRT AEIAVDLENR CEDVIKNRYQ HGDLFDMMRK WKIYAADHLA FALPMFDKLD
KQLVIHYKWL ESLPWYCEAQ KLISDVLEAT RPEEDNPPND EYYTCICTLA VRPPPPGILS
DAVQCDHCFA RFHGDCAKSG GSCPFCDHNH WNGTIHKERA WHFHLLPEIL HAAPDITKNY
SEHWKQLEII VHRVDRLTAH IGQFCMFAQN HLAHFIPHVR HYMRKLFKLQ FQIGATLEES
YGLDLAGLHR ILAGQKVKKR RRPRFTFGQD IDKDWTDGTR CICRGRTCNK VYHTGCVFVC
PLCCLRKNRT YPYSEVRVKN FSKEIVYMKM LPPYTQTLFV ELIRFTP
>Serpula lacrymans
MRRTSQDQSILLSGETGSGKSENRRLAIKTLLELSVSNPGKKGSKLAGQVPAAEFVIESFGNARTLFNPNASRF
GKYTELQFTKGRLCGIKTLDYYLERNRVAGAPSGERNFHIFYYLVAGATAEERQHLHLLDKTNYRYLGSRDANR
FDQLKVALKSIGLSKRHVAQTCQLVAAILHLGNLEFTVDRSRNEDAAVVRNVDILDVVSEFLGVQPSALEAALS
YKTKMVKKELCTVFLDPDGASDNRDDLAKTLYSLLFAWLNEHINQRLCRDDFDTFIGLFDLPGPQNMSRPNSLD
QFCINFANERLQSFTQKRLFESHVDEYTNEGISRFVPTVPYFDNSECVRLLQNKPGGLIHIMDDQARRAHKKTD
HSMVEAFGKRWGNHSSFKMGSLDRFPTFTVNHFNGPITYSAEGFLERNLDALNPDFVSLLRGGSINPFVKGIFS
GKAIATQAHPKNEDTIVSAQQPIKPMRAPSMRRKGTPCVAGEFRAALDTLFDTVAETQAWYVFCVNPNDSQLPN
QLEGRSVKGQVRSAGLSEIARRNVNVFEVGMTPEEFCDRYKEPMGAMGIMSSERDVVLGNFKVFLSGVAFHKLE
DQLRSRDVEEQKRNRLRDAEASKEGLPLVANASPFEDFDGRSRLTSNRESYAPSRNMFQNLDGEIQEGETAEVV
KESSARRKWVMLCWMLTWWVPSPFLKWFGRMKREDVRQAWREKLALNIIIWFVCGCAVFVIAVLGLIICPREYV
FSASELESHSNNVYTSIRGEYGGVAADNIFPVQVSALCNGVSGTVSPWVILDSSNTDPNSQYHDFRAWTNDSRP
DWYFESMTMMRWKNRVGYSGYTPQEIGNLASSGKSIGIYNNLSLPAVMFTSASSTFLTFKPQKPLHFTFLTIGS
RGDVQPYISLARGLMADGHGIEFGYVGGDPAELMRICVDNGMFTVSFLKEGVQKFRGWLDDLLKTSWDACQGTD
VLIESPSAMGGIHIAEALQIPYFRAFTMTWTRTRAYPHAFAVPEHKSYVMFDQVFWRATSGQINRWRRNVLHLG
STSLDKMEPHKIPFLYNFSPHVVPPPLDWPEWIRVTGYWFLDDAEVGAKKWVPPPDLIPFIDSAHQAGKKVVYS
NPQAMTRCIIEAIVQSGVYAILSKGWSDRLEPEEPLPKQIYAISSIPHDWLFAGIPTIIRPFFGDQFFWADRVE
ALGIGSGVRKLTVESLTEALRSATTDVKQIDRAKLVGEHIRAPLLDRPREIFEPLRLACETRNEKLMIASLDCI
SKLISYSFFAELVDLVVHTITACHSENTPETVSLQVVKALLSLVLSPTIFVHHSSLLKAVRTVYNVFLLSTDPV
NQMVAQGGLTQMVHHLTDLFVKDAFLVFRALCKLTMKPLNSESERDIKSHAMRSKLLSLHLVLTVLNSHMPLFN
DPSAIIYSSSSNDDTMFIQAINQYLCLSLSRNAVSPVPQVFEVSVEIFWRVLSGMRTKLKKEIEVLLHEIFIPI
LEMKTSTLKQKAVILGMLSRLCQDPQALVEIYLNYDCDSEAVDNIYEHLMNIISKICTPSLNSTSLSVSGSMMG
LSEAQLRRQGLECMVAVLRSLVSWGTDDPTKFESAKQKKTTLLEGIKKFNFKPKGVQFLIETGFIPSRAPRDVA
QFLLTTDGLAKAMIGEGEEENIATMHAFVDLLDFRNLPFIDALRIFLQAFRLPGEAQKIDRFMLKFAERYIAGN
DTAYVLAYSTILLNTDAHSPQVKNRMTKSDFYKNNRGINDLPEEFLSTIYDDIVKNEIRMKDEIANVGRDLQKE
AYMLQSNGMANDQFFSASHFVHGRPMFEVAWIPFLAGLSGPLQNTDDLEIVELCLDGFKAFVTTLGKFTFLNNL
GEMKAKNMEAIKTLLDVAVTEGNSLKGSWREVLTCVSQLEHMQLISSGRSRKLPAEELANESRSTHITVAADMV
FSLSHYLSGTAIVDFVQALCDVSWEEIQSSGLSQHPRLFSLQKLVEISYYNMNRIRLEWSNLWDILGEHFNQVC
CHNNPHVGFFALDSLRQLSTRFLEKEELPHFKFQKDFLKPFEYTMTHNANPDIRDMVLQCLQQMVQARVQNMRS
GWRTMFGVFSAASRVLTERIASSAFEIVTRLNEDHFAAIVRHGAFADLTVCITDFCKVSKDDTMIRFWFPVLFG
FYDIIMNGEDLEVRRLALDSLFTTLKTYGSTYPVEFWDTVCQELLFPIFAVLKSSQDVSRFSTQEDMSVWLSTT
MIQALRDLIDLYTFYFDILERFLDGLLDLLCVENDTLARIGTSCLQQFLENNVTKLNPSRWERVATTFVRLFRT
TTPHQLFDDNLRVERRRIFKQIIVKCVLQLLLIETTNDLLRNDAVYNNIPPEQLLRLMGVLDHSYQFARMFNDD
KELRTGLWKESSSAATLVHVLLRMYFDERPEHQAARPQIAERLLPLGLSVLQDYTKLRSDTQAKNITAWTPVVA
EILEGFCRFDNKAFVRYLPAIFPLTTGLLARDPEIRLGLKMYFERVGYSQGIQRQALIAAAQAKYGPEIVAPIL
QRIFPTLSLPPGASLVQTLIQLGPDITSDADTIRALLLRFGISDATPPRDSQVIELITSLARLAAEGTTLCDVG

ALVRALSSFPVNLNWANVIKAFDWPDRGVDTATLKLLIAILVNCPRADPHAVTGFWGTWSNSLYQLKLLDALLS
LPADTFSFVSLPGRRIVTVDDMANASPTIKSLAANVQGHTWNSVELFEVLVRQSCSESIDIKNCVQEMLDKALK
ISAELVHMGLLEVKRWSEIRIECSQKLLNMFLAGHPNHQLVFMRLWQIEPSYLTDAFRDFYEESPLNITRILDI
LENLLEVRPFTFSLDEYLNLDKWLLDNVNNHGAEFLHAAILFLEIKMDAEKAARVSDPATRTMSLNPQIIAVFL
RLLRHNSAKMSREDIDYCLDVRNTCLQVYPRLMDAEPGLTVVNYSPEIEAEVDAIYKQMYDEQTTIDEVVSLLQ
RSKASTDSRDHEIFSCMLHFLFDEYKFFQYPPRELAMTGYLFGSLIQHQLVDYLPLGIAIRYVVDALNCPPETN
LFKFGLQALSRFESRLSEWQPLCQALLRIPHLMEARPDLTAVIHRAELSDKILFIVNNLAPSNFDSKLAEMQEH
FSTEPNNHQLYLRFLDALDKQPLAKLILQETFIKSAALLNSEKTAQNSSERATLKNVGAWLDKPIMHKNLSFKD
LLVEGYDNGRLIVSIPFVCKTLEPCARSKVFKPPNPWLMAVISLLAELYHYADLKLNLKFEIEVLCKGLDIDLD
AVEATTVLRNRPRAISAHIEAILATLVHHVVINPQFPLQSNHSFKRAIQLAVHHAVREIIMPVVERSVTIAGIS
TRELVAKDFATEASEEKLRKAAHLMAQKLAGSLALVTCKEPLKSNLGTHIRSFLAEHGFNEQHVIVILVQDNLD
IACSAIEKAAMERAISDVDEGFAASYDVRRRHHETRNPSSFSGNLPEPLRIKSTGLQPHQAAVYEDFFSVLLRD
MEAVVMQVPQSLASLPPNHDIRHMVRQILYLADRQRTPLLMSQKIVQLLYKSSSQLGREIYVTLLDQLCHSFED
VAKEAITWLLYAEDDRKLNVPVTVALLRSGLVNMSLQDQQLATEPRPTLLTFAANLIRECLSSEASQSQFAYSL
EWFQQWVAIFQRSHSPEKAFVPFITQLTKQGILKVEDSSFFFRVCAESSVNSYIKCASTGEYEYAFQALDAMSR
LIVYIIKYHGDANNDQAKVHYLTKILSIFVLVLANMHEEQGFQQKPFFRFFSSLINDLHSIEAHLGTAYFQLLI
AISDTFSSLQPTYFPGFSFSWMCLISHRLFMPKLLLSENREGWSAFHKLLLSLFKFLSPFLKEADLQVPSRDLY
RGSLRLLLVLLHDFPEFLSEYYFTLCDVIPPRCIQLRNIILSAFPPAIILPDPHLRNIKMGPIPPILSDFASGL
KNGDLRNYLDQYLLNRGTPSFLPSLKERLESYNLSLINSLVMYIGVSSVAQAKARSGSSLFVASDPGVIALQYL
VTNLDVEGQHHILSSMVLHLRYPNAHTHWFSSLLLHLFVEVKDERFKEVMTRVLLERFIVHRPHPWGALVTFIE
LLRNPKYEFWHKEFIRVAPEVTLLLESVGHRRLYLQLCFRNVSDLLTVRRDIAPLALANGAKRDAVDAYAEVVD
PGECIIDIREYDVPYYLRVAIDNELRVGLWYAVTFTAGQPSFERVKRADPVVMAYDIETTKAPLKFPDQAIDQV
MMISYMVDGQGYLITNREIVSEDIEDFEYTPKEGYEGPFIVFNEPDEAATMMRFFQHIQEVKPTVMATFNGDFF
DFPFLCARAKANGIDMFLETGFAIDSEDEFKCRTCVHMDCFRWVKRDSYLPQGSQGLKAVTTAKLGYNPIELDP
ELMTPYAMEQPQVLAQYSVSDAVATYYLYMKYVHPFIFSLCNIIPLCPDEVLRKGSGTLCETLLMVEAYRGRII
MPNRHEDAHGNMHQGHLLASETYVGGHVEALEAGVFRSDIPSDFKVVPSAVQLIDDLDAALMFCVTNYDEVKNE
IQAALEVMRDNPKRTDKPLIYHLDVAAMYPNIMLSNRLQPDSMVDESVCAVCDYNRPGKTCDRRLEWAWRGEFF
PAHRDEFNMESFPSKRPGGPQRKFADLTETEQTALLHKRLGDYSRKVYKKIKDTKIETRESIVCQRENPFYVDT
VRRFRDRRYEYKGLHKTWKKNLDSIAEVDEAKKMILAHKCILNSFYGYVMRKGARWHSMEMAGITCLTGATIIQ
MARALVEQIGRPLELDTDGIWCMLPGVFPENFKFKLNNGKSIAFSYPCTMLNHLVHAQFTNHQYHDLDPETGEY
VVHSENSIFFELDGPYRAMILPSSKEEDKLLKKRYAVFNDDGSLAELKGFEVKRRGELQLIKIFQSQIFEKFLL
GSTTEECYAAVAEVADQWLDILFSKAETLGDDELVDLIAENRSMSKTLAEYGGQKSTSISTAKRLAEFLGDQMV
KDKGLACKFIISARPMGAPVTERAIPIAIFSAEESVKRTYLRKWLKDNSLTNFDLRSILDWDYYIERLGSVIQK
LITIPAAMQKVTNPVPRVRHPDWLHRRVAGAIDKFKQNKVTDFFQGRFTLWLSIDGELTPVALRIPRVFYLHLK
SLGHYSCDKVIRNLPRGLHCSYLFTDLINDPNVDGVFEQQTLNRAQSSGVDLVQLDRKYIFLYHACSNSAPIHV
FVLFLPSGVKLHVVDPATRRQPTTYHNNDITALKAISRELGLLENMSYSIVVSSTKEQTYFDTWVPKMSKFPVL
SMSKTKAAHTLDFPWQTHVAQKLLYRYLSVGGWLDRLVVLADYYPLLLSDITFARRLIQQDTVLWWSPSERPDL
GGLEDDFRPVEELQNTEFMSPGCYSNVCLDVAVRNLAVNSVLHSVVVNELEGSGGTTAFDSAQRDITLGESSLS
PQTFGILRTMVKGWLLDRISPATLAIDHFWRWVSTSASNMHDPSIHRFVHGLMRKTFIQMLAEFKRLGAHVVYA
DFSHILLATSKPPGTAHAYATYVATAVTSHELFQHIYLKAERFYDFLLFMDPANMGGVVCEDPLAVDPPEELSI
EMRWNIQTFLPPAIQDDFGKVLQYFIVELFQIRQKKVNEMDATREFIARRLTRRLLKVVFPLLPGSHLHLINPI
LEFIKFTCAVLGLAQEYRIEVGLLKRNLLDLAGVREFATEAIFRNPCEPLRLANVPCRHCDTLREFDFCRDPEL
MPWLCGGCGVEYDRIAIELELMRMVCSLERSFAQQDLRCAKCKQVRSDNVSRYCCSGAYQLTMNKAEVRRRLRT
VVNVAIVHGMAKLFEVEHLRYATLATSANLITQTQVASIKTVRNIIEYNEQYVTKRLLVSIIWAFSGDAKGEWT
AWQSKVPVIEILYSWLSEHKPLMLCGPPGSGKTMTLFSALRKLPDMEVVGLNFSSYRKTPNGVILAPVQIGRWL
VVFCDEINLPAQDKYGTQRVISLVECGGYWRASDMAWVKLERIQFVGACGRVPLSHRFLRLVMVDYPGEISLKQ
IYGTYNRALLKVVPTLRAYSEPLTDAMVAFYLASQKRFTTDIQAHYVYSPRELTRWVRGIYEAIRPLEMLSVEG
LVRVWAHEALRLFQDRLVTEEEKHWTDEHIDNAALEHFPTINILFSNWTSKNKARLKIFYEEELDVPLVLFNDV
LDHVLRIDRVFRQTTLSRFVAWLNGLSIFQIKVSNKYTGDDFDLLANAEVPGLFEGFTMNPPENGLASRAATSP
ALFNRCVLDWFGDWLPSYSPPAHFPIAYRVVNGLVYVHSSLHQINQRLSRRQGRYPRHYLDFINHYVRLYNEKR
DELERHLHVGLDKLVTQVEELRKSLAIKRKLKRMVADQQEAEQKKAASIEIQAALVEQDNHIAQRRAVVMADLA
DAEQMTKPLRDLMKRDFVSRPSYNFETVQRASKACMISELEAKIGKYKEEYALLIRVQSKVDRSMKLLESLSRT
FDAEMSTIVGDVLLSAAFLAYGEWSNHLSEANITFKPELSLVRLSWQSKSLPSDNLTTVTSFLDEAFLKVLESA
LRFGNPLLIQDVEHLDPILNEIRRTGGRVLIRLGSQDIDFSPSFPSVEFSPDICSRVTSQSLDQVLKVETDLMK

VQGEFRLRLRTLEKLLLQALNESTGNILDDDKVIGTLETLKREAAETDVIMKEVEQVTSEYLPLAQACSSIFFI
LEQLNLVNHFYQFSFLDIFDYVLHHNPNLKNVLLSDLFLIVYKRTSRALDELEFLLESGLENYAKQALFRPVLT
HITERENDWVPFLLIIKCLRPDRLLQSVPGYDASYRVELQEGFTLADQAIAAASRQGSWVLLKNVHLAPTWLGE
KKLQTLNPHRNFRLFLTMEANPSIPVNILQSRIIMNEPPPGIFLLAWFHAVVQEDMASAFGTIDTWLNAIAKGR
ANVDPAIIPWDALRTLIKQSVYGGRVDSDFDQRILDTFVDGLFTPTAYNVDFPDGTKLEHFMTWPSWLSLPPTA
ERRKMRMLADDDHDRCREWSSQLPATFNTLPKQSNDNPDPLYRLFFREGSIGKLLDQVRKDLADVLMSSLTKGT
IPSHWRRYKVPKIPDLARRLAQLDGITSMDNGGLFFPEAYVTATRAVAHRKRWSLETLNLRLDIGRVNDPGLVL
EGAAWATDILPDTGFTLPPMQGRNIDEHFHRIGSNSAQPWLSLAKGFSEQMLVFDVETMPPYHPYAVMACAASG
NAWYSWISPWLLGRIVVGHNVSYDRGRILEEYDLRGTQTRFLDTMSLHVAVKGISSHQRPAWMKHRKSKKRWED
LTSANSLADVAKLHCDITVDKGIRNDFMSHTPSEILANIHDYLNYCSQDVFVTHAVFSQVLPAFLARCPSPVSF
AGILTMGSSFLTVNESWEAYLENAERTYRELDEKVKTRLVDLAEEAWKDSSWLAQLDWTPKWPKWYWDLAKPKK
GSLDITVRNRIAPILLRLSWLGWPLFHSREHGWMFRVRLTPLQFYDPADGVLFYKLPHKDGEKANVGSPLGKSF
MKYAQDGTMTSPGGEANEALDMNAQCSYWISARDRIIKQMVVWQKWGIILPQVITMGTVTRRAIEKTWLTASNA
KKNRVGSELKAMVRAPEGYAIVGADVDSEELWISSCMGDAQFGLHGSTAIGWMTLEGTKAAGTDLHSKTASILG
ISRDQAKVFNYSRIYGAGMRHAVLLLLQSNASMLPDQAQKLAENLYASTKGKNTHRDIFGRKFWFGGTESFLFN
KLEEIALSDCPRTPALGCGITYALSKEYLPAEFGSDYMTSRINWVVQSSGVDYLHLLIVSMDYLIKTYDIKARY
LISVHDELRYLVADDDRYRAALALQIANLWTRSLFAYKLGMDDLPQGVAFFSAVDVDTVLRKEVDMPCVTPSHP
TPIPPGESLNIVRTLEKTNGGSLWADGYRSPDCLTHRAKSAAFLRAQATSEFSEVKHLAVEVIEKLANTSNDDL
AEVLSEVDSWKWPRSDLNAWIKVLNKFDVVLEEAIRDYDVDKIQVNIFTPATKKVVCEILRFERLLLDNSTNRK
MFSSYDRLNSLLFTSDLDILILALNLLLRPSQQYSAQPAVSHALNISTPRLQSLCKRWPSLQESGISLLDLASN
TASHVGAIPTDVREVNFTYYRHIDEEILSKKEPMDVLADIVEVHALSDDKFELLCRIRSAMALAPGREKLIIVR
LLAIAIFGHTHSESQATSSLFLHEPDLMTHIAELLQLDHGIRKTVADVANPECQLPHSFVDALLSFVTYLASHA
AGGNMVVGAGLVPLLVQVIENRLTQRLAMVSKIMQLVDNVLYGFTNAFQLFCNGRGVETLVGRIQHEIDGELPV
ARAAVLKHTLRSMHRMMQSSGTAEGLRGLIDSSILQSIKKIIQYRGLFGPSILPMAMNIMATFVHNEPTSLPTI
QEAGLPEMFYQAIEAGLEPVIEVIQAIPNAVGALCLNQVGQDQLSARPSIIPGILSIFTSERHLKVLQEKENAV
LIGTGIDELIRHHPSLKAPVFDAIKSTLGKIEELGQSFVVPEELEQWYGLDNILVSYIDVVCRFLEGLFQHEDG
LDRLGRLTALPCLPYDFANSVLVQVMRTMAEVGPNEALFYLMTLVGSSLAETQTFWQSLDSQSNQHFRSLITLH
VRITIMADIFSTYAHGRAAISLLLGALHRACIWENILLKAGLNAKALKHLTHGLPLAPFFQAVVKRRNPDPTQK
KQILTSSAVIAEIMLGHLYHSIMLGLITILLVDELLLAFHRIGGQELIHAHGGLKVALHLLHPLVSSKPLFESG
QTLLVMSRKKDDDPDYFEPHNFLVRLRLAITPLLHNIWDAFWLVSAPLPLLKSVIQTVLELTGGENEPRSAAER
ALIRSHNNVNAATELLISHPLLNIAREPLKSNLVRQTLRFVDEHPSIIAFSPAAYDLQEQPLAVRCRLLALVLL
SIPKWLAPHLLVIESLFTIADQPPVATIPMECGTSYHDARPIVFEFCLKLFANPNLPRDELLSALRLLVLLTRD
YDVARELMKRNGVALIASLSYVAIILRHVAEDKSALRGIVLQEVNKLFSQPRVLDVASYMRSCGTMALRDPHVF
LQVTQSVCQLQEPESLVHYLIGELIKSIKFSCFLMQCLTELLFSYDACKTAFLSYVPKKPKTAALHFLLSELVS
FGTINARARITLCSWAMSVIVALCVDCSKEVSPELVSVRKFVLEAVSRAIKDLSPAENPDARYGRLLALADLCN
RLLTVRFENSTHIAKIMLEKNFVSTLTTALAEVDLNYPNVRGLVASILRPLENLTRVAIKMSREETPDLYRNSS
LGMYPEVTVHPLLLDPPAGHAFDPLLTMQRWTEEAKILNGKFVSERGSTLANHVTLALLPAAIERVTIMIHGNS
VDITDMGIDPTFLEALPDDIREEVINQHVRDQRAARIERPPDSQISPEFLNALPPEIRAELIQQERIEPGDIDP
ASFIASLDPQLRQVVLLDSDEGFIQTLPKPPSPRDAIQLLDKSGVATLVRLLFFPQKNLLFKVLVNICENAKTR
AELFNLLLSILQSGPGDLSAVDKSFAQMTTRPDLVAQRCLEALTYIVSANELSSLFFLTEHELPVGLRKSKKGK
GKEKQPQTHYPIVLLLSLLDRHSLLKTPSNMESIVALLSTVTKPLTSLKRVILGNPPQIPHSVLRLIVNILTIG
ECSGRTFQQSLMLIQNLSHISDARDVIAQELKVKAQEFGQNIYIDLNELAMALASRFSLPSSDQAKLLRVLKTI
DYMYSIYESFRFTPLWRRLGDCLATIEEKPDTEHIATVLLPLIEALMVVCKYVGSKEAMEDLFVNFTDTHRKVL
NLMVRNNPSLMSGSFSLLVHNARVLDFDNKRNYFGQQLHRRREHHVTLQLNVRRARVFEDSFQYLQRKTGDQIK
YGKLSVRFYDEEGVDAGGVTREWFQILARQMFDPNNALFQPCAADRLTYQPNKNSWVNPEHLSFFKFVGRVIGK
AIYDGRLLDAYFAKSLYRQILGKQVDYKDVEWVDPEYYNSLCWILENDPTPLDLTFSFGRSRIFPLKEGGESIP
VTQENKREFVQLSANFRLYSSISEQIENLLAGFYEIIPKDLITIFNEQELELLISGTPDIDVDEWRAATEYNGY
TSSDPVIVWWWRALKSFNREERAKVLSFATGTSRVPLGGFVDLQGVQGVQRFSIHRAYGDSDRLPQAHTCFNQI
DLPQYSSYEMLRQQLLLAINEGGEGFGFAKSSASSGTRKKNARKLRALGKKDPVTKAKAIEELQRVPVLFTHPA
RRIRLLAASLHASLLGTWCMSSYDIDRQVASFGQKSWFIQKALLDPNGIYLYLNPEESEMDRNARLRVSAFGAF
RWGFGQPLVRQNAWVLLSLLLSTAVLRSAWIDTDVTVRNTMSRPLLLFLKEFPKSWAYNEFLQFLELGSPTEGY
PIVIIILSTIPSSIFSSFWAALDGRALAFLSSLLESMVFLLRRPSVFLAAYLFPPSSEELDSMLSDLPSEPAEP
SLALFESLDRKGYSPYVRVVSTLLSRQLSRDNLWALRHILALDTIRDSRILSSVLQHIFANATKGDADSWMVLA
RKFEKTAPQTSLSIMSSITRFAPEPSRLDRYRNELAANMLGISGLLTLRKLVAVAPDPDSEVVFLPQQRAVNVM

KACQEWITSDVNEEVESMMTLLFYHLAPILQNVPGSHWELVFDVIENNLEHCSISDNTTLVTLGRSLQLIIIIQ
DLVTTNKALRAWEERQLSILTLVKNISAARSLCRELALSVEYYVIEAAVDTASMKVRSSYFNHMRSLDIISTHF
IPNIFDILDIFKAFKLDVWTTDEYYLDMYEPLSARLLAAHLYYRALVTVPALIRSWVSDCTDKQLLARVIAYTS
SYFSPVIIKAELAQVRDSVSEDRWRGWVLGVQQIQNGRVVDGLTLFTKNVTLHFAGQVECAICYSIIKPCKTCK
NRFHAGCLYIPVSPLLDLS SVKTSGPRPF GLEDCPVFYP TLDEFNDPMT YVRSISDSAK DYGICKIVPP
NGWKMPFVTD TEFRFKTRLQ RLNSIEASSR AKINFLEQLY RFHKQQGNSR VSVPTINHKP
LDLWLLRKEV HKLGGYDAVK NKKWSDLGRL LGYGGPGLST QIKNSYTRVI LPYEDFCERV
RTSLCDGCDC GFHMFCLDPP LATIPKGQWF CHTCLFGTGD FGFDEGEEHS LSSFQARDAE
FRRLWWSAHP PVSEYDVENE FWRLVQTPQE TVEIEYGADV HSTTHGAMPT METHPLDPYA
KDPWNLNNMP IVSDSLLRFI KSDISGMTVP WTYVGMVFST FCWHNEDHYT YSINYMHWGE
TKTWYGIPGD DAEKFETAIK SEAPDLFEAQ PDLLFQLVTL MNPKRLIDAG VRVHACNQRA
GEFVITFPKA YHAGFNHGFN FNEAVNFALP DWLPYGRDCV QRYREHRKLP VFSHDELLIT
ITQQSQSIKT ALWLADSLKE MTEREIAARE KARFGMTEVI DEVDPEEQYQ CIICKVFCYL
SQVNCQCVVC VEHADLLCDH LTLRLRFTDA DLLDTQTKVV ERAGIPSSWK GKLSKVLIDS
ARPQLRSLRA LLAEGERVNY HLPELPALRK CVNRAGEWVD SANTFLIRKQ SRKRDRPDRG
LEDLYSLLRE VENLGFDTPE ISVLQGLARQ AEEIKLKALD LLCERLLLQG SSLNVHLEEI
VEIEKIVMRE HLLRELLEDI RGLLTRARSC NLPQDNKYMK TLESRQRAGD NWEERARHVL
EQPYKTIDEL PIEVTVYDRL MSARNKAKDF EKQAKAWLSP DKPKVQEVMR FVARAEKDFS
IPAIQDLKRT ADIAFDLETR CEAVIKNRYQ HGDIFDMMRR WKTYTREHLP FSLPIFEKLD
VQLSQHQRWL EDLPWYCHGQ AILDDVVEST RPEDDLPPSD EYFTCICTTP VRPPPPGVVS
DAVQCDHCFA RFHGVCAANG GSCPFCDHHH WNGAIHKERS WHFCFLPTIL LSAPDITRNY
SDAWKQLEII VHRIDRLSSV IGHFLSYASN RRAEYIPQVR HYMRKLYKIQ FAVSPSREVS
FGLDLAGLHR ILAGQRIKKR RRPRFTFGQD IDKDWTDGTR CICRGRTCNK LYHAACVFMC
PLCCLRKNRS YPYSEVRVKT FSKDIIYVKM LPPYTQTLFV ELTRFVP
>Rigidoporus microporus
MRRTGQDQSILFSGETGSGKSENRRLAIKSILELSVSNPGKKGAKLSTQVPSAEFVLECFGNARTLFNSNASRF
GKYTELQFTRGRLCGIKTLDYYLERSRVAGAPSGERNFHVFYYLVAGASAEEKQHLGLSDKANFRYLGARDAVR
FDQLKVALKNIGMSKRHVAQTCQLLAAILHLGNLEFVIDRRRNEDSAVVKNIDALELVADFLGVQPTALETALS
CKVKLVKKELCTVFLDPDGASDNRDDLAKMLYSLLFAWLNETINQKFCRDDFTTFIGLFDLPGPQNMSRSNSLD
QFCVNFANERLHNWIQRRMFEMHVAEYNSEGIARFVPEVPYFDNSECVRLLSNMPGGLIHIMDDQARRAPKKTD
HSMVEAFGKRWGNHSSFKQGGMDRFPTFTVNHFNGPVTYSSEGFLERNLDALNPDFVSLLRGGSVNPFVRSLFS
AKAIATQAHPRNEDTIVAAQQPQKPMRQPSMRRKGTPCIAGEFRAALDTLFQTLDETQAWLVFCVNPNDAQMPN
QLEGRAVKGQVRSSGLSEVARRCVNVFEVNMSPAEFCDRYRDFLAVLNVHEGERDVVVGQHKVFLSQLAFHKLE
DYLRSTDTDEQKRNRMRDAEGSSQQLPLVANASPFDDIDGKSRLTSHRESYAPSRNMFQNADGEVMEGETTEDI
KETSTRRRWVAFVWILTWWVPNPFLRWFGRMKRLDVRQAWREKLAINIIIWFLCLSVAFVIAVLGNLICPTEHV
FTVAELNSHSNNVYTAIRGEYGGTKADNIFPVQVSALCNGIDGQVNPYVVLDSVNTDINAQYHDFRVFTNDSRP
DWYFQAMTIMRWGYRVGFMGYTPKELRNMANSGSTVGIYNGLSLPAVMFTSQSSTFLSFKPDRSMHFTCLTIGS
RGDVQPYIALAKGLIADGHGIEFGYVGGDPAELMRICVENGMFTVSFFKEGIQKFRGWIDDLLKTSWEACQGTD
VLIESPSAMGGIHIAEALAIPYFRAFTMTWTRTRAYPHAFAVPEHKSYVMFDQVFWRAISGQINRFRRNALRLP
STNLDRMEPHKVPFLYNFSPSVVPTPLDWPEWIRITGYWFLDDAEVSAQRWHPPKPLEDFLTSARNAGKKHCRF
RPRSMTRCVIDAIKASGVYAVLSKGWSDRLEPEAPLPPQIFPINSLPHDWLFAGIPTIIKPFFGDQFFWGDRVE
ALGVGSCVRKLTVESLAEALIQATTNEKQIDRARILGEQIRSENGDKPREIFEPLRLACESGNEKLMIASLDCI
SKLISYSFFVDLVDLVTHTITACHTETTSDAVSLQIVKALLALVLSPTILVHQSSLLKAVRTVYNIFLLSPDPI
NQTVAQGGLTQMVHHVFDMFTKDAFLVFRALCKLTMKTLNTESERDLKSHAMRSKLLSLHLVLTILNSHMQVFV
DHNSIIYSSSTNESTSFIQATKQYLCLAISRNALSPVPQVFEISVEIFWRVLSGMRTKLKKEIEVFFHEIFTPI
LEMKTSTLKQKSVILGMLARLCQEPQALVEIYLNYDCDREAVDNIYEHLMNTLSKIASPALSTSALAVPGANLG
LSEQQLKRQGLECLISVLRSLLAWGTDDPGRFESEKQRKNALQEGIRRFNYKPKGIEYLIENRFIPSKAPVDIA
KFLLSTDGLNKATIGEGDEANIATMHAFVDLMDFSDQPFVNALRMFLQSFRLPGEAQKIDRFMLKFAERYIAGN
DAAYVLAYSTILLNTDAHNPQIKNRMTKTDFIKNNKGINDLPEELLLAIFDEITNNEIRMKDEVANVGRDLQKE
AFVIQSLGTLQDQFFSASHFVHVKPMFEVAWIPFLAGISGPLTDTDNLEIVELCLEGFKAFVTTLAKFTFLNNL
GEMKTKNMEAIKTLLDIAVTEGNHLRGSWQEVLTCVSQLERMQLISSGKSRKIPNEELANESRSTHITVAADMV
FSLSHYLSGTAIVEFVRALSHVSWEEIQSSGLSEHPRLFSLQKLVEISYYNMNRIRLEWSNLWEILGEHFNKVC
CHHNPHVGFFALDALRQLAMRFLEKAELPHFKFQKDFLKPFEYTMIHNSNPEIRDLVLQCLQQMVQAKAHNLVS
GWRAMFGVFSAASKVPTERIVNSSFEIVSRINKDHFSDVVKHGAFADLTVCITDFCKVSKDEAMIKFWFPVLFS

FYDIIMNGEDLEVRRLALDSLFNTLKEYGTDFTIDFWDSICQELLFPIFAVLKSSQDITRWSTQEDMSVWLSTT
MIQALRDLIDLYTYFFETLERFLDGLLELLCVENDTLARIGTSCLQQLLEKNVKKLSPARWERVVTAFVRLFKT
TTPHQLFDESLRMDRRRIFKQIIVKCVLQLLLIETTNELLRNSDVYNTIPPEHLLRLMGVLDHSYQFARVFNED
RELRTALWKETSSASTLVTVLVQMYNDPRPEHQAIRVQVAERLMPLSLGVIQDFNKLRPESQSKNITAWIPVIG
QIAQTFCRLDEKTFARYLPAIYPLTTDLLSKEQEVRGYLREYYIRVGQSQGIQRLALITAAQARFGTEIMSPML
QQLFTNLSLPPGTTLVSALVQLGGEITSDAEVVRGLLTRFGIAESNPPSDVQVTEVFSTLSRLATEGASLFDVG
ALVRALSGFRVQPDWSKVIESFDRPERGVDAPTLKLLIAVLMNSPRAVPHAVTGFWSNWSNQIYQLKLLDALLS
LPNDTFNFVALPGRRVVTVDDVAGASPTIKSLAANVQSHTWNSLDLFEVLVRLGVSENTDVRGIVREMLDKAVR
ISADIVHMGLLQVPPWNAIQLEYSSQLLGMFLAGHPNHQLVFMRIWQIEPTYLTTALREHYEENPVNITRILDI
LDALLEVRPFVFALDEYLNLDKWLADNVNAHGAEFLHAVINFLELKAQNEKIARTSDPSARTMSLSAQTIAIFL
RVLRNSSTLMDEKDVDYCLEVRNACLQIYPRLMDQEPGFSVISYSTEVEAEVDGIYKQMYDEQITIDGVIALLQ
RTKVSTAPHDHEIFSCMLHFLFEEYKFFQYPPRELAMTGYLFGSLIQHQLVDYIPLGIAIRYVLDALHCPPETN
LFKFGLQALSRFESRLQEWRPLCQALIEIPHLIEARPDLADNIRRIGISDKILFIVNNLAPNNFDSKLDEMKEW
FGTEPNNHQLYLRLLDGMDCTVLVKFILHETITKSATMLNSEKTKQSGIDRTVLKNLGSWLDKPIKQRNISFKE
LLLEGADSDSLLLAIPFVCKVLEAASRSRVFKPPNPWLIGIMGLLAELYNHADLKLHLKFEIEVLCGALGIQLD
QVEVSSIYRGRPRVVGSHIENILSTLTAVVKINPQLALHTNQSFKRAVQLAIDRSVREIILPVVERSVTIATIT
ARELGVKDFASEVNEDRLRKAGQIISQQLALSLALVTCKEPLKSNMPTHIRHYLNEQGFNEQPVVQMIVQDNLD
VACATIERAATDRAVVELDEALASACEVRRRHLRERPDTQFIANLPDILRIKPGGLIARQLRVYEDFFNGIVAQ
LESVLAQTPISLSALPPNHDIRNLLRRILQIADRSQTPLLLSQKLVQHLYKTTSQLGRDVYAALLEQLCRAFDE
VAKQAIPWLLFADDDRKFNIPVMITLLRCRLINVAEQDQHLAKDPRHALQNFSAGLIRECLTSSASQADYVYTI
PCFQQWVSVFQRSSTPEKAFIPYITQLTKQGILKAEDSSLFFRVCAESSVNAYTSAVLAGEYENAFLALDAMSR
LIVYIIKYHGDANNDQAKTHYFSKIMSILVLVIANFHEELGFQQKPFFRFFSTLLNDLHDMENYLGNVYLHLLV
TLSDTFSSLQPVYFPGFAFSWTTLISHRLFMPKLLLTDNREGWAAFHKLVLALLRFMAPFLRTADMQVTSRNLY
RGLLRLLLVLLHDFPEFLSEYYFTLCDAIPSRCIQLRNIVLSAYPPSCILPDPHLVHTRMGPIPPILSDFTLVL
KNGDLRTLLDQCLLNRVTTSPLSVLADRLEMYSLTLVNATVMYIGVSSVAQAKARSGSALFAPGDPGVVAMTYL
AVNLDTEGQHHLVSAIVMHLRFPNAHTHWFGSLLLHLFAEIRDECFREVVTKVLLERFVVHRPHPWGALMTFIE
LLRNGKYDFWSGSYIRAVPEVALLLDSVGQRKLYLQICFRNVSDLLAVRKELVPLALENGAKLSAVDAYAEVVD
ARDSIIDAREFDVPYYLRVAIDNEIRVGLWYSISFVNGQPQIDRLDRADPVVMAYDIETTKAPLKFPDQAIDQV
MMISYMIDGQGYLITNREIVSEDIEDFEYTPKEGIEGPFTIFNEADEAATITRFFQHIQAVKPTVMATFNGDFF
DFPFLCARAKVHGIDMFLETGFAKDSEDEFKSRSCVHMDCFRWVKRDSYLPQGSQGLKAVTTAKLGYNPIELDP
ELMTPYAMEQPQVLAQYSVSDAVATYYLYMKYVHPFIFSLCNIIPLCPDEVLRKGSGTLCETLLMVEAYRAQVI
MPNRHEDPLGNMFEGHLLASETYVGGHVEALEAGVFRSDINTNFKIVPAAAQLIDQLDAALTFYITNYDEVKAE
IQAKLETMRDNPIRFDKPLIYHLDVAAMYPNIMLSNRLQPDSVVEEAMCAVCDFNRPGKTCDRRMTWAWRGEYF
PAQRDEFNMEWFPPKKPSGPKRRYVDLSPAEQTALLHKRLGDYSRKVYKKTKETKVVNRESIICQRENPFYVDT
VRTFRDRRYEYKGLHKTWKKKLDGIAEVDEAKKMILAHKCILNSFYGYVMRKGARWHSMEMAGITCLTGATIIQ
MARQLVEQIGRPLELDTDGIWCMLPGIFPENFKFKMKNGKTLALSYPCTMLNQMVHARFTNHQYHDLDKETGHF
QVHSENSIFFELDGPYKAMILPSSKEEDKLLKKRYAVFNDDGSLAELKGFEVKRRGELQLIKIFQSQIFEKFLL
GTTTEECYAAVAEIADRWLDVLFSKAESLSDEELVDLIAENRSMSRTLAEYGGQKSTSISTAKRLAEFLGDQMV
KDKGLACKFIISQKPIGAPVTERAVPVAIFSAEESVKRTYLRRWLKDNGLANFDLRSILDWDYYIERLGSVIQK
LITIPAAMQKVANPVSRIRHPDWLHRRVRAMDDKFKQHKMTDFFRGRFTMWLAVNNDIVPVTIRIPREFYLNYK
TPEHYTRERVVRMLPHNRACGDILMNKINQPNVDGAYELQTLNRARDNGVDLGQMSRKIVFLHHAYTPSAPVHC
FSIVLPDGIKLHVVDPATRRQPTDYHSNEATALRAISRELGLLENKAYTLVLSSTRDTHYFTSMVPKLAKFPIL
RMPSSKPGHILDFPWQSALAKKLLSRYLSLSAWLHRTIAQASHYPLFFSDIDFARRLVAQDMVLWWSPGEHPDL
GGYEDDALPSDELVNPEFTSPGVYSRVCLSIQVRNLAVNSVLQSSIVNELEGCGGTTGFDTAQATATLSDSNLP
PQTFMVLKHMVRSWLTKNAGPAGLTIDHFWRWISSSSSHMFDSCIQRFVHGLMRKTFIQLLAEFKRLGSNLVYA
DFTRLVLVTSKPPGTANAYATYLLTAVTSHELFKHVYLRTDRFYDFLSFMDPANEGGVLCEDPLAIEPPKQLSV
FLTWNIKKFLPPAVQDHFKAVIKYFIVEMHKISRARVKEIDAIKGFISQRLTRKMLQTVFPILPGSYLNLTDPA
LEFVKFTCAIFGLAHDYQIEIGLLKRNLLELVGVKEFSEVASFHNPCDPLKLSMVTCQYCDYLRDFDFCRDDDL
FPWTCPECLCEYDRTAIEFSLIQMLHRLERGFSQQDLKCSKCKQIRSDNVSKHCCSGSYELTIGKVEVRRKLRT
IVNVAIVHKLNRLFEVEHLRYATLATSPNLSTQGQIVSIKTVRNVVEYNEQYVTKRLLLNIVWAFSGDAKGDWI
AWSSKVPVIEILYSWLSEHKPLMLCGPPGSGKTMTLFSALRKLPDMEVVGLNFSSFRKTPSGVILAPMQIGRWL
VVFCDEINLPATDKYGTQRVISLVEYGGYYRTSDMAWVKLERIQFVGACGRVPLSHRFLRLVMVDYPGEASLKQ
IYGTYNRAMLKVVPNLRAYAEPLTDAMVSFYLASQKHFTTDMQAHYIYSPRELTRWVRGIYEAIKPLELLSVEG
LVRVWAHEALRLFQDRLVSEDERVWTDDNIDATAMEHFPTINILFSNWTSKNKARLKVFHEEELDVQLVLFNDV

LDHVLRIDRVFRQTTLSRFVAWMNGLSIFQIKVSNKYTGEDFDLLANAEVPGLFEGFTMNPPENGLASRAATSP
ALFNRCVLDWFGDWLPSYAPPTYFPIAYRVVNALVYVHQSLYQINRRLSRRQGRYPRHYLDFIQQYVRLYNEKR
DELERHLHVGLDKLVTQVEELRASLAIKRKLKRMVTDQQEAEQKKAASIEIQAALVEQDRHIEQRRAIVMADLA
DAEQMTKPLREVMKKDYLSRPSFNFETVNHASKACMIAELEASILKYKEEYALLIRVQSKVDRSMKLLDSLSRT
FDTEMSTIVGDVLLSAAFLAYGEWSHHLSDAGIKFKAELSFPRLSWQSKTLPSDNLCTVTSFLDDAFLKVLESA
LRFGNPLLIQDVEHLDPILNEIRRTGGRVLIRLGSQDIDFSPSFPSVEFSPDICSRVTSQSLDQVLKVETDLMK
VQGEFKLRLRTLEKLLLQALNESTGNILDDDKVIDTLETLKKEAAETDVVMREVEQVTAEYLPIAQACSSVFFI
LEQLSLINHFYQFSFLDIFDYVLHHNPNLHGILFDDLFLVVFKRTSRALEEVEFLLESQLNSYSKHTLFKDVKT
HMTDHEENWKSFLLLIRCFRPDRLVPSVTGYDASYRVDNQEGFALADQAIALAARQGTWVLLKNVHLAPSWLGE
KKLQTLNPHRNFRLFLTMEANPVIPVNILQSRVVMNEPPPGIFLLAWFHAVVQEDMSAAFGTIDAWLGAVAKGR
ANIDPAAIPWDAIKTLIKQSVYGGRIDSDFDQKILDTFVDSLFTPSAYNLDFPEGTKLEHFLSWTSWLYLPPTA
EQRKMRTLADDELEKCKEWLAILPTTFATFAKQSVENQDPLYRLFFREGQVGRLLTQVRRDLADVLMSHLTKGT
IPAHWLRYKVKKVPGLARRLQQLDRIASLDGGGLFYPEAYITATRAVAHRKKWSLETLDLRLDIEDVSDPGLVL
EGAAWSSSRASGGKIHFTPLQGQTIDEHFYNIGAKAAEPWLSLAKDFAEDLLVFDVETMPGIHPFAVIACAMSP
TNWYSWISPWLLGRVVVGHNVSYDRARILEEYHVSGTQTRFLDTMALHVAVKGISSHQRPAWMKYRKVKKRWED
ITSANSLEDVAKLHCGIRLRKAARNDFLTHTREEILSGIQEYLDYCWRDVDVTHAVYAKVFPEYLKACPNPVSF
AGILTMGSSFLTVNHSWRDYLENSERVYNELNERVKQRLLELAEEAWKNDVWLSQLDWTPKWPKWYWELAKPRK
GSLNLTVRTRFAPLLLRLSWLGWPLFYSREHGWTFRVRLKPLKFYHHHDEPLFYKLPHKDGEVANVGSPLGKTF
IKYAQDGTLTSPGNGAESALDMNAQCSYWISSRDRILNQMVIWDSWGMILPQVITMGTVTRRAIEKTWLTASNA
KANRVGSELKAMVRAPPGYSIVGADVDSEELWISSAMGDAQFGLHGATALGWMTLEGTKAAGTDLHSRTASILG
ISRDQAKVFNYSRIYGAGVRHAVLLLLQGNPSLSPDVAQKQAEKLYASTKGKNTHRDLFNRKFWFGGSESYVFN
KLEEIALSDKPKTPALECGITHALSKEFLPPGFGTDYMTSRINWVVQSSGVDYLHLLIVSMDHLISKYDIKARY
LISVHDEVRYLVKQEDRYRAALALQIANLWTRCQFAYRLGMDDLPQGVAFFSAVDVDKVLRKEVDLPCVTPSQT
VPIPPGESLDIFKALEKTNGGSLHCDGYTTPNCIVHRSMSPAFIRAQATTEPGEVKRLATALIADILAKDKEEL
PDFLASIDSWKWPRSDLNIWIKVLNKFDQLLEDCIRDYEVDKLQTREFDQHTKQLLCEILKFERFLLENSTNRK
MFNSYDRLNGLLFTSDLDVLIATLFLLLRPSQQYSSQPAVSHSLHISTTRLECLARFWPTIRDHGVDMLDFVSK
TQEKVENIPPEASEVNLTFYRHLGPLSLSSRSAMEILNDAVKSYQVPEERYELMCRIRMARSLGVGRQKLMIAR
LLAIAIYAHTHNETQAQSSLFLYETDLLARLSDLLQLDRGVRKTVAEISNPESTLPHMFVDALLTFVIYLASHT
SGGNMVVSAGLVPLLIQIIGITLPQRLAVVSKTMQLVDNVLYGFVNAFQMFCNSHGVEALTERIRHEVQGLLPV
SRASVLKHILRSMLRMMQSSGTAEGMRTLIDSSLLKSVETIIEYRGVFGPTILPIAINIIATFVHNEPTSLATI
QEAKVPETIYNALEAGLEPSIEVIQSIPNAIGAMCLNQAGQDQLASRPTVIPALFSIFTSERHLRVLQDKESAA
IIGAAIDELIRHHPSLKTLVFNAVVSVIGKIETLGNAYVPPKDIEHWYKIENVIVDFIYILGRFLEGLFQHKDG
LNTFTRLLSLPCLPYDLSSSQLVQVIHTMAEVAPTETHNHLVQQIKQSVRATEGFWQKSGATSNVTFRKLITLH
NCLTLLADVCTSYTHGQSPISLLLGSVHRNCLFENITLKNELNAHALRYIAIQIPLVPLFQALGRRRGHDTSMR
QQAISMAETIATVMCGHLYNTFMLRLVAVLLSDELLYQFRQRGGQELVHVYGGLKVALPLLQALVSYKPTSESP
SMVTGSSARSETHRDYFEPHDFLVKLRLAIAPLIADIWQSDWLVSSPPGVSKHVIQSMFAILAGENEPRSAAVR
ALTRLHNNISAATEYLLSQTELSTLRTEWRSNVGALALRLLDVHSTIEKFMPAAYDINEEPLSLRFRLLALILQ
PLPKWLASLLLVMESLLVLSEEPQAISVPLFTGPLYTEVRAHLLELSLRLLGLPVLQRDDYLATLRIVVFLTRP
EEGARQFKERNGLSLLACQSYTAIILRHLTEDTVTLDHFMRQEVKRVLTSRDGDNTSNYIRTCVPIASRDPRLF
VSVTKSLCQLSSPEMVVHFLLNELLRFGKYACFLMQCLTELLFSYDQCKSAFLSFGKKRSKPVALHFLLSDLVS
VGAFNTKKRVMLCNWAMSVIVALCVDSTNNMSVDLSAVRKAVLDAIAKAIKEAPPGETIDSRYGRLLALADLCH
RLLTVRFETPLHLAKMMLEKNFVAILTNALSSVDLNYPNVRSLIAAKLRPLEHLTKVAIKMGREETPDLYRNSS
LGMYPDTTTHPLLVERTDSTDFGPLPTIQRLTEEAKIMHGKHLYDRITRLSNHIILSLLPDARERVTVLIHGNP
VDITNTGIDPTFLEALPDDMREEVLNQHFREQRSARVEQPAESQISPEFLEALPPELRAEILQQERLEPVDMDA
ATFIASLDPNLRQVVLLEQDDGFLQTLPKLAPPRDAIQLLDRSGVASLVRLLFYPKRNVLHKIMLNLCENTKTR
TELFNLLLSVLHDGTGDVAMVDRSFSQLSFKPDLVAQRCLDALASIVVNNDVSSLFFLTEHELPAGLKRSKKGK
GKEKQPQSYYPIVLLLSLLDRQALLKTPSIMDSVAGLLDAITRPLANLRKLFLANPPQIPHPSLRLIVNILTVG
ECSGRTFQHTLALIQHLSYLPDARDVIAQELKSKAQEFGQNLYADLEALILALASKFSPASSEQAKLLRVLKTI
DYMYSIYESFNFAPLWQKLGDCLTVVEQKPNVEHVATILLPLIESLMVVCKHVGLKESMEDLFVTFTDNHRKVL
NLMVRNNPSLMSGSFSLLVQNPRVLDFDNKRNYFNQQLRRRRDHYGALQLNVRRAQVFQDSYHIFIHKSGDQIK
YGKLSVRFYNEEGVDAGGVTREWFQILARQMFDVNYALFEPCAADTQTYQPNRASAVNPDHLSYFKFVGRVIGK
AIFDGRLMDAHFARSLYRQLLGKKVDYRDVEWVDPEYYKSLCWILDNDPTVLDLTFIFGRRDIIPLKENGTSIP
VTLENRKEYVQLSAQYRLTDSIKDQIEKLLEGFYEIIPKDLISIFNEQEVELLIAGTPDIDVDEWRAATEYNGY
SSSDPVIVWWWRALKSFSRDERAKVLSFATGTSRVPLGGFTDLQGVQGVQRFSIHKAYGQPDRLPQAHTCFNQI

DLPQYTSYEMLRQQLLLAINEGGEGFGFAKSSASSGTRKKHARKKERLGKKDSVTKRKALEDWFSLPTLFLHTS
RHIRQLSASIHASLLGSWCMAMHDPDRQAAMYARRSWFVRRSLLDPQRVYLDLNPEESENDRKARLRIGGLGAL
KWGDGQPLVRKSAWGLLSTLLSSAVLRSAWVEPDGQVRSIMWEPLLLFLLHFPESWAYQEFLQFLQLGSPTQGY
PAIILVLSSIPSTILTSFWSALDGRALAFLSSLLECLILLIRRPDFFTFGYLYPPSREVLDEMLEELPSNPIDP
TLSAIEPLDTSGLCEYARAVDALLERHLAKENNWLLRHCMCLESIRDARILYATIQRILNGATTGDAEHWMLLA
RKFETKARLASEAIILAVSESGLEPPRLDRLRNELAAESLGVKGLRLLHRLALTVPDPDSDVVFLPQNRAVNFM
KACQSWITSDIEEDVESEMLPVFLHLLPILQSIPGAHWDLVFDVIENNLENSSFDDNSSLVTLTRTLQLLTAVQ
ELTRTNKALRAWQEPGKYPLQVCFMVSTPRSMCWEMAFSIAIPVIEAGIDTDSLRVKMGYGNQLRDLGLIENNL
LPTVLDMLDLYKPFPLEPWAVSEFYIDYYDPLSPKLLSAHLFYRALLSVPSLVSTWWSALKDKQLQGTISTFTT
RHFSPVLISAELAHVRNPAEENRWRGWLLAVQQVQNGRIVDGLSMFKKNVTHHFENQTECAICYSIIKRCKTCK
NRFHASCLYIHVSPKLDMS SVKTASPRPF GLEDCPVYYP TADEFKNPMV YIRSISEHAR KYGICKIVPP
EGWKMPFATD TEFRFKTRLQ RLNSIEATSR AKINFLEALY QFHKQQGNPR VTVPTINHTP
LDLWLLRKEV QKLGGFETVK EKKWVDIGRQ LGYTGPGLST QIKNSYTRVI LPFEHYQSEG
ARNLCDGCDC GFHTFCLTPA LSTIPKGQWF CHTCLFGTGD FGFDEGEDHS LSTFQARDLA
FRKMWFQSHP PVTEDDVEEE FWRLVEAQDD TVEIEYGADV HSTTHGGMPT LETYPLDPYS
KDPWNLNNLP ILSDSLLRYI KSEISGMTVP WTYVGMVFST FCWHNEDHYT YSANYMHWGE
TKTWYGIPGE DAEKFETAIR QEAPDLFELQ PDLLFQLVTL MKPDRLTSAG VRVYACNQRA
GEFVLTFPKA YHAGFNHGLN FNEAVNFALP DWLPFGRDCV RRYQEHKKLP VFSHDELLIT
ISQQSNTIHT ATWLNESFKE MTDRELSGRR RVRLGVKEVL EERDPEEQYQ CNYCKAFCYL
SQIYCTCVVC LEHIDYLCEC RTLRLRFSDE ELSNTQQTIE SSAAIPDIWG DKFARLLEES
PRPSLRALRA LVAEADRVNY PFQHLGSLRK CVEAANEWVR ETNVYTTRRI SRRGDGTRPT
LEGVRDLLRR VEDLGFDTPE IALLQNIATQ AEAAKAKAKL LLDIRELLSG YTLSLQLDEL
VELEEFCNKM DITIEILMAY LDLPVRARAC NYDMTSPVFE KMQKLSKVVD RNNDAVREVL
SRPIKLLEEL LMDPQLEMKL DQVYNQAVAY QKQAEAWLNP RMPEAQEAMK LVQKAEKEFQ
IDNIAELKQL AEHAYDLEER CERVLRNRYL PQPMLQVVTK WREIADKQLR FSLPNCDALL
KQIQLHQQWL TKLPWWCHGD EILEDVVDYT KPEDDSPPHD EFFTCICFEP VRKPPPNVSS
DAVQCDHCFA RFHGRCAKNG GSCPFCDPNH WNGNIHRERS WHSCYLPTVL NNAPDVTRYH
SSEWKQLKVI VEHIDRFCTV TGHFLKQAQN QRPDLIPQVR HFMRKLYKIG FAVSPNPEVS
FGLDLAGLHR ILASRRIKKR RRPRFVFGQD LDKDWIDGTR CICRGRSCNR RYHTGCVFLC
PLCCLRKGKA YRYADVRVKS FSDKPIYVRL NPPTTSTIFV DMIHFMP
>Schizopora paradoxa
MRRTGQDQAIVLSGETGSGKSENRRLAIRSLIELSVSNPGKKGSKLSSQIPSAEFVLESFGNARTLFNPNASRF
GKYTELQYTRGRLCGVKSLDYYLERSRVAGAPSGERNFHIFYYLTAGATAEERQHMRLTDKTHFRYLGPRDSVR
FEQLKVALKSVGLSKRQVAQSCQLVAAILHLGNLEFITDRQRNEDAAVVRNTDTLDTVAEFLGVQPAALETALS
CKMKLVKKELCTVFLDPDGASDNRDDLAKILYSLLFSWLNESINQKLCRDDFTTFIGLFDLPGPQNMSRANSLD
QLCVNYANERMHHWIQKRLFESHIEEYKVEGISRYVPSVPYFDNAECVRLMSNMPGGLIHIMDDQARRAPKKTD
QTMVEAFGKRWGNHSSFKVGGMDRFPTFTVNHFNGPVTYSSEGFLEKNLDSLNPDFVSLLRGGSANPFIRSLFS
NKAIATQAHPRNEETIVSAQQSMKPMRAPSTRRKGTGCIAGEFRSALDTLFSTLDEAQAWFVFCINPNDSQLPG
QLEGRAVKGQVRSAGLPEIARRCVNVFEVNMLPTEFCDRYKDQLSALNIHEGERDVVQGQRKVFLSQRAFHKLE
NHLRVNDADEQKRNRTKDTEGSSQNLPLVNHASPFDDIEGKSRLTSRPESYAPSRNMFQNEDGEVLEGETAEDI
KESSNRRRWVAFVWMLTWWIPNIALVWCGRMKRLDVRQAWREKLALNMLIWFFCGCAVFVIAVLGNLICPTEHV
FSVTELASHSNNIYTAVRGEYGGLDATNIFPVQVSALCNGVTGSVNPYVTLDFTNTDPNAQYHDFRVSTSDSRP
DWYFESMTLMRYNYRVGFVGYTPKELKNMANSGRSVGVYNGLSLPAVMFTSTSSTFLTFKPQQPLHFTCLTIGT
RGDVQPYIALAKGLMADGHGIEFGYVGGDPSELMRICVENGMFTVSFLREGVQKFRGWIDDLLKTSWEACQGTD
VLIESPSAMGGIHIAEAMRIPYYRAFTMTWTRTRAYPHAFAVPEHKSYVMFDQVFWRATAGQINRWRRNTLKLQ
STNLDKMEPHKVPFLYNFSPTVVPPPLDWPEWIRVTGYWFLDDADVTAKKWEPPQDLIEFIDKAHSSGKKIVYS
DPDAMTRCVVEAIEKSGVHAILSKGWSDRLQPEIPLPPSIYPLKSVPHDWLFAGIPTIIKPFFGDQYFWGDRVE
ALGIGSCVRKLTVDGLADAFIAATTDEKQIEKARHIGEQIRSENGDKPREIFEPLRLACETRSEKLMVASLDCI
QKLVSYSFFVELVDIVAHTITSCHTESTPDAVSLQIVKALLSLVLSSTMLIHQSSLLKAVRTVYNIFLMSPDPV
NQTIAQGGLTQMVHHVFDLFVKDAFLVFRALCKLTMKNLNTESERDLRSHAMRSKLVSLHLVLTILNSHMPVFV
DPSSIIYSSSSNEATTFINATKQYLCLSLSRNALSPVPQVFEISVEIFWRVLAGMRTKLKKEIEVFLHEIFIPI
LEMKTATLKQKSVILGMLQRLCEDPRALVEIYLNYDCDREAVDNIYEHLMNTISKIATPALSTAALAVPGANLG
LSEQQLKRQGLDSLVAVLRSLVAWGTDDPGRFENAKQRKTVLQEGLRKFAQKPKGIDFMLEQGFLSSKSPHDIA
AFLLSTEGLNKAAIGEADTENVEIMHAFVDQLNFAGLSFIEAMRTFLQAFRLPGEAQKIDRFMLKFAERYIAGN

DAAYVLSYSTILLNTDAHNPQVKNRMTKAEFIKNNRGINDLPEETLNAIFDDITSNEIRMKDEIANVGRDLQRE
AYVMQSLGMANDQFFSASHFVHVRPMFEVAWMAFLAGISGPLTQADDMEIVELCLEGFKAFVTTLAKFTFLNNL
GEMKPKNMEAIKALLDIAVSDGNNLRGSWQEVLSCVSQLERMQLISSGKTRKLPAEELANESRSTHITVAADMV
FSLSHYLNGTAIVEFVRALSSVSWEEIQSSGLSEHPRLFSLQKLVEISYYNMNRIRLEWSNLWEIIGEHFNQVC
CHHNPHVGFFALDALRQLAMRFLEKEELSHFKFQKDFLRPFEYTMIHNANPEIRDMVLTCLQQMIQARAHNLRS
GWRTMFGVFSAASKVLTERIVNSSFEIVTRLNKEHFTEIVKYGSFADLTVCITDFCKVNKDDANIKYWFPVLFS
FYDIIMNGEDLEVRRLALDSMFSTLKTHGSTFTTEFWDSICQELLFPIFAVLKSSSDVSRWTTQEDMSVWLSTT
MIQALRDLIDLFTYFFETLEHLLDGLLDLLCVENDTLARIGTSCLQQLLENNVKKLSADRWERVVTTFVKLFRT
TTPHQLFDENLRVDRRRIFKQIIVKCVLQLLLIETTNELLQNKDVYDTIPPEHLLRLMGVLDHSYQFARMFNDD
KDLRTALWKETSSAETLVNVLLQMYYDSRPKHQELRSQVAERFLPLGLGVIQDFNKLRPDTQAKNIAAWMPVIA
KILDGFNRLDEKAFVRYLPGIYPLATDLLARDTASRTSLREYFVRVGKVQGIQRQALLSATQAKFGVETIAPVF
QQILSHISLPSGTTLVQLMVQLGPDLTSNANVLRGILGRFGISEHHAPTDALNVELFSTLGRLAAEGTPMGDVG
LLVQMMGAFAPNLDWSKVIESFDRPERGVDMATLKLLIAILGNSSHTNKPSISGFWSTWKNSLYQLKLLDALLS
LPSDTFNFVNLPGRRVVMVDDVSSASPTIKALAINVQGHTWNSLDLFEVLVRLGGSEIIEVRNCVRDILDKAKR
ISAEIVHMGLLQVPPWNAIQEEYLQQLLEMFLNGHPNHQLVFMRIWQIEPPYLTNALRDFYEKNPMNITRILDI
LEPLLEVRPFIFALDEYLNLDKWLADNINQHGAEFLHAVIEFLELKAQNEKITRTSDPSARTMALSPQTIAIFL
RVLRNNSSDMDERDVDYCIEVRSVCLQIHPRLMDQDPGFSVVSYSADIEAEVDSIYKQMYDEQITIDGVIALLQ
KTKESTNPRDHEIFSCMLHFLFDEYKFFQYPARELAMTGYLFGSLIQHQLVDYIPLGIAIRYVLDALQCPPDSN
LFKFGLQALSRFEARLPEWRLVCQALMDIPHLAEARPDIAETVRRAEVSDKILFIINNLAPSNFDAKVEEMNER
FSIEPNNHQLYLRFLDGLKSSALMKLILHETFVKSAALLNSEKTKASSSERSVLKNLGSWLDKPIKFKNMSFKD
FLIEGSESDRLIVAIPFVCKILEACSKSKAFKPPNPWLMGIISLLAELYHFAELKLNLKFEIEVLCKALGLDMD
DIEVANILRSRPRNVGAHIEGILANLANSVIVNSQLAYMRDPNFKRSVQLGIDRAVREIILPVVERSVTIASLT
TRELVVKDFATESSEDRVRKAGHLAGQKLAGSLALVTCKEPLKTTLPGHIRAYLTENGFSEAQIVLLLAQENLD
IACEAIEKAAMDRAIGEIDEAFVPHLEARRRHHEQRPGSSFASGLPDPLRVKANGPQEAQLRVYEEFFSLCMSK
LETILQETSASLAVLPQTHDIRTIVHRILTIADRFKLPLLISQKIVQYLYKTSTQLGRELYATLLEQLCQSYDE
VAKEAITWLIYAEDDRKFNIPVTVTLLRCRLVSVAEEDQQLARSPRPVLQDFAVGLMRECLTCDATRQQFTLTL
DCFQQWVAVFQRSSSPEKSFVPYITNLTKQNILKAEDSSFFFRVCAETSVGHYSNCLASGDFDNAFLALDAMSK
LIVFIIKYHGDANNLNAKVHYMTKILSIVVLVVANRHEEQGFEQKPFFRFFSSLLSDLHGIESHLGDAYLPLLV
ALSDTFSSLQPVYFPGFAFSWMTLVSHRLFMPKLLSSSNRDGWQAFHKLLLALFKFMAPFLRNAKLQLASRNLY
RGTLRLLLVLLHDFPDFLSEYYFSLCDVVPPRCIQLRNIIMSAFPASATLPDPHLPGMKMSPIPSIFSDFTATL
KTGDLRTYLDQYLFNRIAPNSLAPLKERLESYNISVVNAVVMYIGVSSVAQAKARTGSPLFVSTDPGAVTLSYL
AANLDPEGQHHLLSAMVMHLRYPNPHTYWFSSLLLYLFSEVNDDRFREIMTKVLLERFIVHRPHPWGALMTFIE
LLRNPKYEFWARDFVRMVPEVALLLESVGHRRLYLQICFKNVSDLLAVRRDIVPLAVENGAKLNAVDAYAEVVD
PRECILDAREFDVPYYLRVAIDNEMRVGLWYTVTFVAGQPQFDRVKRADPVVMAYDIETTKAPLKFPDSAIDQV
MMISYMIDGQGYLITNREIVSEDISDFEYTPKEGYEGPFTIFNEPDEAATIMKFFHHIQTAKPTVMATFNGDFF
DFPFLCARAKAHGIDMFLETGFAIDPEDEFKSRTCVHMDCFRWVKRDSYLPQGSQGLKAVTTAKLGYDPIELDP
ELMTPYALEQPQTLAQYSVSDAVATYYLYMKYVHPFIFSLCNIIPLNPDEVLRKGSGTLCETLLMVEAYRGNII
MPNRHEDAHGNMFEGHLLASETYVGGHVEALEAGVFRSDIPTHFKIVPEAAQLIDQLDDALTFCVLNYDEVKAE
IQAALEQMRDNPMLFEKPLIYHLDVAAMYPNIMLSNRLQPDSVVDESVCAVCDYNRPGKQCDRRMTWAWRGEFF
PAQRDEYNMEMFPPKKPGLPQRRYPDLSASEQTAILHKRLGDYSRKVYKKTKETKVVNRESIICQRENPFYVDT
VRRFRDRRYEYKGLLKTWKKKLDSIAEVDEAKKLILAHKCILNSFYGYVMRKGARWHSMEMAGITCLTGATIIQ
MARQLVEQIGRPLELDTDGIWCMLPGTFPENFKFKLSNGKPLQVSYPCTMLNHLVYAQFTNHQYHDLDPETGSY
KVHSENSIFFELDGPYRAMILPSSKEEDKLLKKRYAVFNDDGSLAELKGFEVKRRGELQLIKIFQSQIFEKFLL
GNTTQECYGAVAEVADRWLDVLFTHADTLSDEELVDLIAENRSMSKTLAEYAGQKSTSISTAKRLAEFLGDQMV
KDKGLACKFIISAKPIGAPVTDRAVPVAIFSAEESVKRTYLRKWLKDSSLANFELRSILDWEYYIERLGSVIQK
LITIPAAMQKVANPVPRIRHPDWLHRRVVASDDKFKQHKMTDFFRGKFTMWLSVNSELIPITLRIPREFYLNFK
TPVPYLRERVIRTLPRERPCLHLFMAEINNPNVDGVYELQTLNKARDNGIDLTQVDHKYLFLYHAYSPSAPLHV
YALFLPNGVKLHIVDPATRRQPTDYHGSEAAALKAISRELGTLENRSFVLLLSSAKDFTYFESMVPKLVKFPVL
RLPGGKAAHALDFPWQSGIAKKMFARYFSISPWLRRSIQQASYFPLFLCDIDLARRFAAQDMLLWWSAVDRPDL
GGLEDDLQLSEETTNPEFLTPGMYSNVCLNIQVRHLAIDAVLQSAVVNELEGSGGTTAFDSAQPNVTLGEANLS
PQTFATLKQMVRAWLLDKACPSSLTIDHFWRWISTTSSRMYEPSVQKFVHGLMRKTFIQLLAEFRRLGSNIVCA
DFSRIVVVTSKPPGTAYAYATYITTAVTSQELFKHIHLRTDQFYDFLLFMDPANQGGILCEDPLAVEPTDQLAV
FSSWNIKKFLPAAVQDKFRSVIKYFIVEMFKIRSSKQKELDASKNFIMQRLTRKMLQTVFPTLPGSHLNLTDVT
LEFVKFTCAVFGLAKEFQIEVGLLKRNLLELVGVREFADAAAFKSPCDPLKLSMVTCKYCDHIRDFDFCRDEDL

FPWYCSECGCEYDKGVIEYSLLQVLWRLERSFTEQDLRCGKCKQIQSDNVSKYCCSGSYQLTVTKADARRKLRT
MINVAIVHNLSRVFEVEHLKYATLATSSNLLTQKQITAIKTVRNVIEYNEQYVTKRLLVNIIWAFSGDSRGDWI
AWSSRVPVIEILYSWLSEHKPLMLCGPPGSGKTMTLFSALRKLPDMEVVGLNFSSYRKTPNGVILAPVQIGRWL
VVFCDEINLPATDKYGTQKVISLVESGGYYRTSDMAWVKLEHIQFVGACGRVPLSHRFLRLVMVDYPGEISLNQ
IYGTYNRAMLKVVPNLRAYAEPLTESMVSFYLESQRRFTADIQAHYIYSPRELTRWVRGIYEAIKPLEVLSVEG
LVRVWAHEGLRLFQDRLVNEEEKTWTDETIDATAMQHFPTINILFSNWTSKNKARLKVFHEEELDVQLVLFNDV
LDHVLRIDRVFRQTTLSRFVAWMNGLSIFQIKVSNKYTGEDFDLLANAEVPGLFEGFTMNPPENGLASRAATSP
ALFNRCVLDWFGDWLSTYKVPDYFPIAYRVVNALVHVHQTLYQINRRLSRRQGRYPRHYLDFIQQYVRLYNEKR
DELERHLHVGLDKLVDQVEDLRKSLAIKRKLKRMVADQQEAEQKKATSIEIQAALIEQDKNIAQRKEAVMEELA
DAETISKQLREFMKKEYLSRPNFNFETVNRASKACMISELEASIGRYKDEYAVLIRVQGKVDRSMKLLESLSKT
FDTEMSTIVGDVLLSAAFLAYGEWSSHLTEANIKFKTELSFPRLSWQSKSLPTDSLCTVTSFLDDAFLKVLESA
LRFGNPLLIQDVEHLDPILNEIRRTGGRVLIRLGNQDIDFSPSFPSVEFSPDICSRVTSQTLDQVLKVETDLMK
VQGEFRLRLRTLEKLLLQALNESTGNILDDDKVIDTLETLKKEAAETDVVMKEVEQVTAEYLPIAQACSSVFFI
LEQLNLINHFYQFSFLDMFDYVLNHNPKLERILFDDLFLIVFRRTSRALDVLEFLLESGLNSYAKHSLFQPVQS
HMQSNEQEWISFLLLIRCFRPDRLVQSVIGYDASYRVDNQEGFSLADQAIAVAARQGSWVLLKNVHLAPSWLGE
KKLQTLNPNRNFRLFLTMEANPVIPVNILQSRVLMNEPPPGVFLLAWFHAVVQEDMASAFGTIDIWLNSVAKGR
TNIDPMSIPWDALRTLVKQSVYGGRVDSDFDQKLLDSFVDSLFTPAAYNLDFPEGTKLEHFLTWPSWLSLPPSA
ERRKMRSLADDDLERSKEWLAGVPETFNTITKQTLENQDPLYRLFFREGEVGKLLAQVRRDLADVLMSHLTKGT
IPTHWLRYKVKKIPNFARRLQQLDRIAGLDSGGLFFPEAYITATRAVAHRKRWSLETLDLCLDIEKMNDPGLVL
EGASWSSEILPDTGFNIPDLQGKNIDEHFFRIGADAAQPWLDLAKTFAEEMLTFDVETMPNYHNYAVMACAMSK
SNWYSWVSPWLLDRIVVGHNVSYDRARILEEYHIDGTKTRFLDTMSLHVAVKGISSPQRPAWMKYKKLKKRWED
ITCANSLADVARLHCGIDMSKEIRNDFMTHTRSQIFDGIQDYFEYCSKDVAVTHAVYSKVFPDFLKACPSPVSF
AGVMAMGSSFLTVNEEWEKYLAKAEGFYLEAEEKIKRRLIEIAEETWKEDVWLSQMDWTPKWPKWYWDLTKPKK
GTVDITIRSRLAPLLLRLSWLNWPLFHSREHGWIFRVRQKKLTFYDPADAHLFYKLPHKDGDSANVGNPFGKTF
VKYAQDGTLTSPGDEAKSALDMNAQCSYWISSRDRIMKQMVVWQKWGMIIPQVVTMGTVTRRAIEKTWLTASNA
KKNRVGSELKAMVRAPPGYAIVGADVDSEELWISSAMGDAQFGLHGATALGWMTLEGTKAAGTDLHSKTASILG
ITRDQAKVFNYSRIYGAGMRHAILLLLLQSNPNMLPEIAQKQAEKLYASTKGKNTHRDIFGRKFWFGGTESFVFN
KLEEIALSDRPQTPALGCGITHALSKEYLPPGFGTDYMTSRINWVVQSSGVDYLHLLIVSMEYLIKRYNIKARY
LISVHDELRYLVKEEDKYRAALALQIANLWTRCLFSYRLGMDDLPQGVAFFSAVDVDWVLRKEVDLPCVTPSQP
DPIDPGESLNITQILEKTNGGSLWSDGYVSPNCLAHRASSAEFLRAQATSEFGEVKRLAATLIAEILAKPTEDL
TDFLASIDSWKWPRSDLNAWIKVLDKFDTVLEDVIRDYDIDSLQVKDFTPEVKNLVIGILKFERLLLENSTNRK
LYQSYDRLNSLLSSSDLDILVASLLLLLRPSQQYTGQHGLSQTLQVSSDRLESLAKSWPSLREHGIEMLSLIDN
SDERLERLPQETSEVHFTFYRHLGSLAQSSRSAQEVYVDAVKHHDVPEHKFDLLCRIRTAHALGSGRQNLTMAR
LLSIAIYAHTHTETQTQSNMYLFEPDLSNRIAEVLQLERGVRKSVAEISNPTTALPNLFVDALLSFVGLIAQYT
SGGSLIVGAGLIPQLIQIISITAPERVSFVSKSMSIVDHVLYAYPTAFTMFCNGRGVDVLTERIKQEIDGLLQH
GRSGLLKHILRSMHKMMQNSGTAEGMRGLIDSSLLKSVEKVIIYRGVFGPSIFPIAINVVATFLHNEPTSFSAI
QEAKMPDRVYDAIESGVELSMEVIQAFTNVIGALMLNQAGQEQLSSRPSIIPSLFTIFTSERHLKVLQEKDNAV
LIGTYFDELVRHHPTLKEAVFSSIMSTLGKIHALGMVYTPPESEKFWYGLDNPVVDFINAMCQFLNGLFHQLGG
LSRLTDLLELPSLPYNFGGAQLIQLTRIISDVSPRKILDHLSERVKRTLDITKPFWETLGENSNYMFRNLVSLH
VQVALLSDIYAAFAHNRSAIGLLLGALHRAFIWESILFKNALNAKALAFLASQIPLSPVLLAIAKRRGVDVTQR
KDFISVAETLATVMHSHLYNTLMLTFISMMLFDELLIQFRKQGGQELVHATGGFKVALHLLHSLISYKPMTDVH
QIGNLGAVRSESDPDFYEPFDFLVKMRLSIAPIILETWQSSWLVTAPPALSRYVLLAVKDILAAENERRGAALH
ALERARNNVTLATEYLLSVPELDELREQIRVSFGTHALRLADAHPTIEKFSPGALDVQEIPLSVRCRLLALLLQ
PLPKWLAPLFLAAESLLVAAEDIKAISLPLLCGPSYKDVQLVLLDICIRLLHLPSLPRDEGLAVTSMLVQLTRN
RASAKAFVERGGLVRLSFHSHAIIIFRHLMEDPATIEAIMRFEIKRYFSSSRAFDVSNFARSNGDMLVRDPQTF
VSVTKSLCQLEHPEAIIHGILDELTKIGKYACLLMTYLAELLISYDQCKASFLSYPKKRSKSAALSFILSEFVT
YGNFIRKQRMVMSDCAIQLIVAICVDNSSTTTTDFSNVRKLVLECIGKALRESSSVESIGDRYGRLQALGELCY
RLLTVRFEACMQIAKIMLEKSFVAILTSVVSDIDLKFPNVKNLVSGLLRPLEHLTKVAIKMGRRETPDLYRHSA
LGMYADSMMHPLLVDPQNIVPFVPLLTLQRWSEEASATLRKQVQERAGRTSNHITCALLPEARERITVLIHGNP
VDITDTGIDPTFLEALPDDMREEVINQHFREQRTVQAEQAVDSSISADFLDALPPEIRAELLQQERLEPADIDP
ASFIASLDPQLRQSVLMESDEGFLQTLPKPPQIRDAVQLLDKTGVASIVRLLFFPQRNLLFKVFVNLCENSKSR
TDLFNLLLSILQDGTGDLAIVDKNFAQLSFRPDLVAQRCLDALSYIVETNDSSSLFFLTEHELPAGLRRSRKGK
GKEKQPQSHYPVVLLLGLLDRQTLLKTPSIVDAVAGLLNTITRPLSSLKKILLANPPQIPHHALRLIVNILTVG
DCSGKTFQHTLALINHLSFLPDAREVIAQELKSRAQDFGHNISSDLDRLITALASKFSPASSDQAKLLRILKTI

DYMYTIYDSFRFTPLWNKLGDCLAVVENKPDIEHISTILLPLIESLMVVCKNVGLKESIDNLFVSFTDDHRKVL
NLMVRNNPSLMSGSFSLLVQNSRVLDFDNKRNYFTQQLHRRRDHYGTIQLNIRRSRMFEDSYHAFLNKSGDQIK
YGKLSVRFYQEEGVDAGGVTREWFQVLARQMFNPNYALFEPCAADRQTYQPNKASKINPDHLFYFKFVGRVIGK
AIYDGRLMDAHFARSLYRQLLGKPVDYRDVEWVDPEYYNSLCWILENDPTPLELTFIFGQRRTISLKDNGASIP
VTIENRKEYVQLSARYRLYSSIQEQIEHLLSGFYEIIPKELISIFNEQEVELLISGTPDIDVDEWRAATEYTGY
SSADPVIVWWWRALKSFNRDEKAKVLSFATGTSRVPLGGFVDLQGVQGVQRFSIHRAYGDPDRLPQAHTCFNQI
DLPQYSSYEKLRQQLLLAINEGGEGFGFAKSSATSGTRKKHARKLRRLGKKDTVTKRKALEDLQHLPPLLLHYS
RQIRLHAAELLIAYLGSWCLATQDMDRQVSLTARKSWFVKRSVYDPQGVYLELNPEESEDDRNARIRTGGLGVI
RWGSDQPVVRKAAWGIVNSLLSSSILRSAWVEPQTSVRYVMWEPLLLFLTQFPNAWAYREFLQFLELGSPIQGY
PVIVVILSTIPETIFASFWAALDGRALAFLNSLLECLVFLVKRPDIFAFAYLSEPSQVELDGMLQALPSDPSDY
SLATVDRLDKTGLSEYARIVNTLLDRKLARDNMWLLTHFVQLDSVRDSRILHFVVRHLLSGSSASDADQWMLFA
RQLEKKAPLSAVGIVLAITESGLEPPRLDRYRNELASGILSIPGLLLLHRLSATTPSLESDVIFLPQPRAVNFM
KACQQWVTSDIDEDVECEMLVIFQYLVPILQNMPGSHWDLMFDMIENNLETSSFSDQSSLTLLTRTLRLLQDIQ
VLTQSNKSLKAWKEREKAILGLVRDLSVPRAICWELALTIEYVVIEAGVDTASLRVKMGYASQLRDMNIISSKF
LPTIFDLLGLYKPVQLEMWTLQEYFVQLYDEISANLLAAHLYYRALLHIPSLVASWWGDCKDRQLSMAVANMTA
RHFSPVLISAELKHVKDPVEEKRWRGWLLAVQQIQNGRIVDGLGIFKRNVTNHFENQTECAICYSIVKRCRTCK
NRFHAGCLYITVTSALDLN TVKTSSPRPF GLSDCPTFYP TEEQFKDPMA FIRSIGDQGR SSGICKIVPP
EGWKMPFVTD TEFRFKTRLQ RLNSIEASSR AKINFLEALY RYHKQQGNPR VTVPTINHKP
LDLWLLRKEV NKLGGFEVVK GKKWVDIGRL LGYGGPGLST QLRNSYIRVI LPFEHFSDGI
RKSLCDGCDS GFHMFCLVPP LSAIPKGQWF CHTCLFGTGD FGFDEGEEHR LSSFQARDLA
FRKMWFQAHP PVSETDVENE FWRLVQSPYE TVEIEYGADV HSTTHGAMPT LETHPLDPYS
KDPWNLNNIP ILSESLLRYI KSEISGMTVP WTYVGMIFST FCWHNEDHYT YSINYMHWGE
TKTWYGIPGD DAGKFEAAIR KEAPDLFETQ PDLLFQLVTL MNPARLTSAG VRVFGCNQRA
GEFVVTFPKA YHAGFNHGFN FNEAVNFALP DWLPYGLDCV RRYQEHKKHP VFSHDELLIT
ITQQSNTIQT ALWIVDSLNE MVNRELKGRD AARRGVKEEA DNASRDEQFQ CSFCKAYCYL
SRVVCTCAVC LEHINYLCEC AVIRTRFTDP ELHNIQSTIA TRAAMPENWR NKLGKLLMES
ATPQLRTMRA LVAEADRINY PLKELPVLKR CVAKANEWVD SANTFITRKQ SRKRERPEKS
LEDLYALLKE VERLGFESPE IGQLRVLGSQ VEECKRNAAE LLCETLLAHG SSLNVYVKEL
VDVENIVLQE QLLKEMLDEV RQFLMRARAL NLPGDNKHLK VLEAKLQAGN DWDDRAARVL
SQPVKTIEDL PVDPGVLNRL SAARTKALEY ERQAKAWLNP ELPKVSEALR FAQKAEHEFK
IPAIEDLQRT ADFALDLEER CEQVLKGRYQ HGPVFDAIEK WQKYAQEHLS FALPNFDKLN
KQLESHAAWM TKLPWYNQGS EVLTDVLEYT KPEDDGPPDD EFFTCICFLP VRPPPPGQTS
DAVMCDHCFA RFHGRCAANG GSCPFCDPNH WNGNIHKERS WHFYYLPLIM EAAPDISKHY
SEGYRDLQVI THHVERLSAV IKHFLAFASN HRSEYVPQIR HYMRKLYKLQ FAIGSNPENS
FGLDLAGLHR MLSVMRVRKR RRPKFVFGQD VDKDWRDGTR CICRGQTCSR RYHAACVFMC
PLCCLRKCKP FPTADMRVKT FSRELIRVRL DAPKTSTIFI DLVQFTP
>Rickenella fibula
MRRTSQDQSILLSGESGSGKSENRRLAIKSLLELSVSNPGKKGSKLSSQIPSAEFVLESFGNARTLFNPNASRF
GKYTELQFTRGRLCGIKTLDYYLERSRVAGAPSGERNFHIFYYLVAGATLEERQHLHLTDKMTYRYLGQRDALR
FDQLKLALKSIGLSKRHVAQSMQLLAAILHLGNLEFTIDRHRNEDAAVVRNTDILDIVSEFLGVQPAALETALS
CKMKLVKKELCTVFLDAEGASDNRDDLAKMLYSLLFAWLNESINQRLCRDDFATFIGLFDLPGPQNMSRSNSLD
QFCINFANERLQNWIQKRMFEFHVEEYASEGISRFVPQVPYFDNSECVRLLSNMPGGLIHIMDDQARRMPKKTD
HTMIEAFGKRWGNHSSFKVGAMDRFPTFTVNHFNGPVTYSAEGFLEKNLDALNPDFVSLLRGGSINPFVRSLFS
AKAIATQAHPRDEDTIVAAQQPVKPMRAPSTRRKNTPCVAGEFRKALSTLFTTLDETQPWFVFCVNPNDSQLPN
QLEGRAVKGQVRSSGLSEIARRSVNVFEANMMFAEFCERYGDQLAALNIVEGERDVVIGQHKVFLSQAAFHKFE
DHLRAGDVEEQKRNRLRDAEASSYTLPLVQHAASFDFDDGKSRLTSHRESYAPSRNMFQTTDGEVLEGETTEVI
KESSGRRRWVALCWMLTWWIPSPLLTWVGRMKRLDVRQAWREKLALNMIIWFVCLSTAFVIAVLGDLICPTEHI
FNTSELAGHSNNVFTSIRGEYGGTSADNIFPVQVSALCNGVSGSVNPYVILDSSNTDPNSQYHDFRVSSTDPRP
DWYFESMVLMRWNYRVGFVGYTSKELKSMANTGRSVAVYNGLSLPAVMFTSKSSTFLTFKPQKSLRFTFLTIGT
RGDVQPYIALAKGLIADGHGIEFGYVGGDPAELMRLCVENGMFTVSFLKEGVQKFRSWIDDLLKTAWEACQGTD
VLIESPVAMGGIHIAEALKIPYFRAFTMTWTRTRAYPHAFAVPERKTYVMFDQVFWRGTAGQINRWRKHMLGLS
STSLDKLEPHKVPFLYNFSPTVVPPPLDWPEWIRVTGYWFLDDADGGSKTWSPPDGIVEFIDKAHNAGKKVVYS
DPDAMTRCVVEAIVKSGVHAILSKGWSDRLEPEIPLPSQIYPIQSIPHDWLFAGIPTIIKPFFGDQFFWADRVE
ALGIGSGVRKLTVEHLTDALVYATSDARQIARAKLVGEQIRSENGDRPREIFEPLRLACETRNEKLMIASLDCI

SKLISHSFFIDLVDLVTSTITACHMETTPDTVSLQIVKALLSLVLSSTLLVHQSSLLKAVRTVYNIFIMSHDPV
NQTVAQGGLTQMVNHVFDMFIKDAFLVFRALCKLTMKPLNTESERDFTSEGMRSKLLTLHLVLTVLNSHMAVFA
EPWSIIYSTSSNETTTFINATKQYLCLSLSRNALSPVPQVFEISVELFWRVLSGLRTKLKKEIEVFFHEIFVPI
LEMKTSTLKQKGVILGMLLRLCQEPQAVVEIYLNYDCDREAADNIYEHLMNTISKIATPSLSTAALAVPGAGLG
FSESQLKRQGLECLVAVLRSLVAWGTDDPGKFVSAKQRKTTLLEGIKKFNFKPKGIEFLVDTGFIPTRSPYEIA
KFLLQTDGMNKTMIGEGDDENIAIMHAFVDQLDFTNLPFVDALRVFLQSFRLPGEAQKIDRFMLKFAERYIAGN
DCAYVLSYSTIMLNTDAHNPQVKNRMTKLDFIRNNRGINDLPEDFLSAIFDDILSNEIRMKDEVANVGRDLQKE
AYVMQSHGMANDQFFSASHFIHVRPMFEVAWIPFLAGISGPLQDTDDMEVVELCLDGAECFVTTLAKFTFLNNL
GEMKTKNMEAIKTLLDIAVTEGNYLKGSWHEVLTCVSQLERMQLISSGRSRKLPAEELANESRSTHITVAADMV
FSLSHYLSGDAIVDFVKALSSVSWEEIQSSVASEHPRLFSLQKLVEISYYNMNRIRLEWSHLWDILGEHFNQVC
CHHNPHVGFFALDALRQLAMRFLEKEELPHFKFQKDFLKPFEYTMTHNANPDIRDMVLQCIQQMIQARVQNMRS
GWRTMFGVFSAASKVLTERVVNSAFEIVSRLNEDHFSAVVRYGAFADLTVCITDFCKVSKDDPMIKFWFPVLFS
FYDIIMNGEDLEVRRLALDSLFSTLKTYGSSFPVEFWDTVCQELLFPIFAVLKSTSDLTRWTTQEDMSVWLSTT
LIQALRDLIDLYTYFFDTLERFLDGLLELLCVENDTLARIGTSCLQQLLESNVKKLSPTRWERVTSTFVKLFKT
TTPHQLFDDSLRAERRRIFKQIIVKCVLQLLLIETTNELLQNDEVYNTIPPEHLLRLMGVLDHSYQFARVFNED
KDLRTALWKETSSAATLVNILARMYYDTRPEYQVLRPQVVDKLLPLGLGVMQDFNKLRPETHAKNIASWTPVIA
EILNGFCRFDDKAFSRYMPVIYPPAAELLTKDAEVRLGLRDFFTRVGRAQGIQRQALLAAIQAKYGFEALGSRV
HQILTMMSLSSGTSLVQTLIELGPDITSDSDIVRALLNRFGITDQKPPRDAQIVEIFSNLGRLAAEGATLCDVG
ALVRALNSFNVQLNWAKVIESFDWSDRGVDTATLKLLIAVLHNAPRAEPHAVAGFWTKWSNSLYQLRVLDALLS
LPSDTFNFVSLPGRRVVTVDEVASASPTIKSLAANVQGHTWNSLDLFEVLSRLGDAESPEVRGCIHDMLDKAVK
ISADLVHMGLLQVPPWNAIHMEYTQKLLSMFLGGHPNHQLVFMRIWQIEPSYLTTALREFYEENPMNITRILDI
LESLLEVRPFIFALDEYLNLDKWLADNVNQHGAEFLHAVIAFLELKAQNEKTSWLSDPSVRTMALNPQTIAIFL
RVLRNSSSVMDEADVDYCLEVRNACLQIHPRLMDQEPGFSVVSYMPEVEAECDQIYKKMYDEQISIDDVIALLQ
RSKESTNPRDHEVFSCMLHFLFDEYKFFQYRARELHMTGYLFGSLIQYQLVDYIPLGIAIRFVLDALQCPPDTN
LFKFGVQALSRFESRLPEWKPLCQALAEIPHLAEARPDVAEAVERAEVSDKILFLVNNLAPSNFDAKTTEMKER
FSHEPNNHSLYLRFLDALDRPPLGKFILNETFVKSASVLNSEKTLQSASERALLKNLASWLDKPIKHKNVSFKD
LLIEGADSNRLIVAIPFVCKVLESCAKSKAFKPPNPWLMAVISLLAELYTFADLKLNLKFEIEVLCKALDLDLD
KIEPTSILRTRPRAVGAHIESILMNLANLVVINPQLAYHTNQSFKRAVQSAVDRSVREIIMPVVERSVTIAGIS
TRELVAKDFCTEQNEEKMRKAGHLMAQKLAGSLALVTCKEPLRSNMASHVRHYLSDHGFNEQQVILLIVQDNID
VACAAIEKAAMDRAVADVDDGFGQAYETRRRHREQRPGAPFIATLPEPLRIKASGLQMQQTRVYEDFFNLLVSE
LEGMLPHVPNSLTVLPAGHEVRGLVRRILFLADRNRTPLAMSQKIVQLLYKTSSQLGRELYVALLEQLCQSFED
VAKEAITWLIYAEDERKFNIQVTVTLLRSRLINVAQQDQQLAKDPRTTLQDFTAGLIRECLTSDAAQSQFSFAI
ECFQQWVNVFQRSSVPEKSFVPYITQLTKQGILKAEDSSFFFRVCTESSIDQYMKAVATGDYEHAFQALDAMSR
LIVYIIKYHGDANNDQAKVHYLTKILSIVVLVLASRHEEEGFQQKPFFRFFSSLLSDLHSVEAQLGTAYFQLLL
AIGDTLSSLQPTYFPGFAFSWMTLISHRLFMPNLLLSDNREGWSAFHKLLLSLFKFLGPFLRTAHLELPSRDLY
RGTLRLLLVLLHDFPEFLSEYYFTLCDIIPPRCIQLRNIILSAFPPSITLPDPHLRNIKMGPIPPILSDFTFIL
KTGDLRGYLDQFLLNRGTQASLSALKDRLEVYNLSVINAIVMYIGVSSVAQAKARSGSSVFVASDPGVVALTYL
AVNLDPEGQHHLLSSIVLHLRYPNAHTHWFSSLLLYLFSEVKDERFKEIMTKVLLERFIVHRPHPWGALVTFIE
LLRNPKYEFWSRDFIRVAPEVTLLLESVGHRRLYLQLCFRNVSDLLTVRREVVPLALANGAKLDAVDAYAEVVD
PRDAIVDVREFDVPYYLRVAIDNDIRVGLWYTVSFVAGQPQFERVKRADPVVMAYDIETTKAPLKFPDQAIDQV
MMISYMVDGQGFLITNREIVSEDIDDFEYTPKEGYEGPFTVFNEADEAATITRFFQHIQTAKPTVMATFNGDFF
DFPFLCARAKVHGIDMFVETGFAIDSEDEFKSRTCAHMDCFRWVKRDSYLPQGSQGLKAVTTAKLGYHPIELDP
ELMTPYAMEQPQILAQYSVSDAVATYYLYMKYVHPFIFSLCNIIPLNPDDVLRKGSGTLCETLLMVEAYRGEII
MPNRHEEEHGNMFEGHLLASETYVGGHVEALEAGVFRSDISTHFKIEPSAAQLIDELDAALSFCVTNYDDVKSQ
IQSALEQMRDSPLRHDKPLIYHLDVAAMYPNIMLSNRLQPDSVVDESVCAVCDYNRPGKTCDRRMTWAWRGEYF
PAQRDEYNMEWFPPKKPDGPKRRYTDLAPAEQTALLHKRLGDYSRKVYKKTKDTKVVNRESIICQRENPFYIDT
VRTFRDRRYEYKGLLKTWKKNLDAVAEVDEAKKMVLAHKCILNSFYGYVMRKGARWHSMEMAGITCLTGATIIQ
MARQLVEQIGRPLELDTDGIWCMLPGIFPENFKFQLKNGKSVAFSYPCTMLNHLVHAKFTNHQYHDLDRETGTY
AVHSENSIFFELDGPYRAMILPSSKEEDKLLKKRYAVFNDDGSLAELKGFEVKRRGELQLIKIFQSQIFEKFLL
GTTTEECYAAVAEVADRWLDVLFSKADTLGDDELVELIAENRSMSKTLAEYGGQKSTSISTAKRLAEFLGDQMV
KDKGLACKFIISAKPIGATVTERAVPVAIFSADESVKRTYLRKWLKDNSLANFDLRSILDWDYYIERLGSVIQK
LITIPAAMQKVSNPVPRIRHPDWLHRRVAAIDDKFTQHKLTDFFRGRFTLWLSVNNDVVPVKLRIPREFYLNFK
SEQYYSRERVIRTLPRDRPCLHLFVNEMNNPNVDGVYELQTLNRARDHGVELEQLIRKYVFLYHAFSPSSPINV
FALFMPNGVKLHIVDPATRRQTTDYHGNEATALKAVSRDLGVMENKGYTLVLSSVKELGHFERSVPKLSKFPVL

CMPSNRAGHALDFPWQSNVGKKVLTRYFHLAPWLQRVVAQAAYYPLFLCDMEFSRRLLKQDMVLWWSAGEKPDL
GGLEDDMHATEELVNPEFVSPGCYSNVCLSVQVRNLAINSILQAAIVNELEGSGGTTAFDSTQAAVTLGDSSMS
PQTFATLKAMVKTWLLDKASPSHLTIEHFWRWISSSGAHMFDPSIQRFVHGLMRKTFIQLLAEFKRLGSNVVYA
DFSRIVLVTSKPPGTAHAYATYITSAVTSHELFKHVLLRTDQFYDFLLFMDPANQGGVVCEDPLALEPPEQLAV
FMSWNINKFLPPAVQDRFRRIVRYFLVEMYKIRRSKVKEMDATRSFIAQRLTRKMLQTVFPLLPGSHLHLTDPA
LEFVKFTCAAFALARDYQIEVGLLKRNVLDLVGVKEFSDHAVYHNPCDALKLSMVTCKYCDNLRDFDFCRDEDL
FPWFCSECDCEYDRVAIEFALIQIVHRLERSFAQQDLRCSRCKQIQSDNISKHCCSGSYQLTIPKADVRRKLRT
IVNVAIAHNLSRLFEVEHLKYATLATSPNLVTQKQVASIKTVRNVIEYNEQYVTKRLLVNIIWAFSGDAKGEWI
AWQSRVPAIEILYSWLSEHKPLMLCGPPGSGKTMTLFSALRKLPDMEVVGLNFSSYRKTPNGVILAPVQIGRWL
VVFCDEINLPATDKYGTQRVISLVECGGYYRPSDMAWVKLERIQFVGACGRVPLSHRFLRLVMVDYPGELSLKQ
IYGTYNRAMLKVVPSLRAYSEPLTDAMVEFYLSSQKRFTTDVQAHYIYSPRELTRWVRGIYEAIKPLEVLSVEG
LVRVWAHEALRLFQDRLVTEEEKTWTDENIDSTAMQHFPTISILFSNWTSKYKARLRVFHEEELDVQLVLFNDV
LDHVLRIDRVFRQTTLSRFVAWMNGLSIFQIKVSNKYTGDDFDLLANAEVPGLFEGFTMNPPENGLASRAATSP
ALFNRCVLDWFGDWLPSYNPPQYFPLAYRVVNALVHVHISLHQINLRLSRRQGRYPRHYLDFIHHYVHLYNEKR
DELERHLHVGLDKLVTQVEELRKSLAIKRKLKRMVADQQEAEHKKAASIKIQAALVEQDRNIEERRAVVMADLA
DAEQMTKPLRDLMKRDFLSRPSYNFETVNRASKACMISELESSIRTYKDEYALLIRVQSKVDRSMTLLGSLSRT
FDTEMSTIVGDVLLSAAFLAYGEWSHHLTEANVKFKLELSFPRLSWQSKSLPSDSLCTVTSFLDEAFLKVLESA
LRFGNPLLIQDVEHLDPILNEIRRTGGRVLIRLGNQDIDFSPSFPSVDFSPDICSRVTSQSLDQVLKVETDLMK
VQGEFRLRLRTLEKLLLQALNESTGNILDDDKVIDTLETLKREAAETDVVMREVEQVTAEYLPLAQACSSVFFI
LEQLNLVNHFYQFSFLDIFDYVLHHNPNLKGILFNDLFLVVYKRTSRALDELEFLLESGLMSYSKNALFKPVRE
HIQGHESEWVSFLLLIKCFRPDRLLQSVTGYDASYRVENQEGFTLADQAIATAARQGTWVLLKNVHLAPSWLGE
KKLQTLTPNRNFRLFLTMEANPVIPVNILQSRIIMNEPPPGIFLLAWFHAVVQEDMAAAFGTIDTWLNTVAKGR
TNVDPAVIPWEAVRTLVKQSVYGGRIDSDFDQKLLDTFVDALFTPAAYNLDFPEGTKVEHFKTWPSWLSLPPSA
ERRKMRTLEDDDLDRCNEWVSALPATFNSIQKQSTDNQDPLYRLFSREGHIGKLLSQVRKDLGDVLMSQLIKGT
IPTHWRRYKVTKVVDLARRLEQLDKIAGLDNGGLFFPEAYVTATRAVAHRKRWSLETLDLRLDIGNINDPGLVL
EGASWASDVLPDTGFALPPMQGRTIDEHFYRIGNAAAQPWLAFAKDFAEDMISFDVETMPNYHNYAVMACAMSS
THWYSWISPWLLGRIVVGHNVSYDRARVLEEYSIKGTKTRFLDTMALHVAVKGISSHQRPAWMKYRKTKKRWED
ITSANSLADVAKLYCKVEMDKEIRNDFMTHTPAEILANVHEYFDYCANDVHVTHSVFAAVLPQFLTACPNPVSF
AGILTMGSSFLTVDQEWEAYLENAERKYRELEEDIKKRLLALAERAWRTDEWLSQLDWTPKWPKWYWELAKPKK
GTVDITVRNRISPLLLRLSWQGWPLFHSREYGWTFRVRQKPLTFFDKADISLFYKLPHKDGESANVGSPLSKTF
IKYAQDGTLTSPGDEAKDALDMNAQCSYWISSRDRILKQMVVWQKWGMILPMLITMGTVTRRAIEKTWLTASNA
KKNRIGSELKALVRAPPGYALVGADVDSEELWISSVMGDAQFGLHGATAIGWMTLEGTKAAGTDLHSKTANILG
ITRDQAKIFNYSRIYGAGMRHAVLLLLQSNASMKPEDAQKQSTKLYSSTKGKNTHRDLFGRKFWFGGTESFVFN
KLEEIALSDRPQTPALGCGITYALSKEYLSPGFGTDYMTSRINWVVQSSGVDYLHLLIVAMDHLIAKYNIDARY
LISVHDELRYLVAEHDKYRAALALQVANLWTRSLFAYRLGMDDLPQGVAFFSAVDVDTVLRKEVDLPCVTPSQP
IPMPPGESLNIIQVLEKTNHGSLHEDGYIMPDRLAHRSPSAAFLRAQATTELGELRRLAASLIADILAVSNEDL
SQFLSSIDNWKWPRSDLNSWIKVLNRFDTILEEVIRDYDVDRLQTTIFTPRTKATVCEILKFERLLLENSTNRK
MFNSYDRLNSLLFSSDLDVLVASLLLLLRPSQQYSAQPALSHALHISTSRLASLAGRPPILREYGVDMLDLVSA
GKEKIRNLPQEASEVSLSFYRHLGPMAQSSRESMDVLADAIQTYDVPDEKYELLCRIRNAQALGETREKLVVIR
LLSIAVYAHTHTEAVAQSSLFLYEPDLVTRIAELLQLDRAVRKTVAEIANPHSTLSHLFVDALLSFVIYIASHA
AGGNMVVGAGLVPLLIQTIENKLPERLQVVSKTMQLVDNVLYGFTNAFQIFCNGRGVEVLAERIQYEVDGLLPF
ARTGVIKHTLRSMHRMMQSSGTTEGLRGLIDSSLLASVKKIIGHRGLFGPTVLPIAINIMATFVHNEPTSLVVI
QEAGLPEAFYEVIENGLEASIEVIQSIPNAIGALCLNQAGQDQLASRPSIIPGLLTIFTSERHLKVMQDKENSA
LIGSSIDELIRHHPSLKAVVFDALKSTLSKIEDMGSTYTPPSDIEQWYKLDNLIVSFIDILSKFLEGLFQHTDC
LTRLTRMLALPCMPYDFANSVLVQVIRTMTEVAPTETLGHLAKEVKISLEETRDFWQSLDGKSNNIFRRLLSLH
NQTTMLSDVYTSYTHGRGAVGLLLGALHRACLWENILLKTGLNARALKHVASQIPLAPLFQSVARRRHPDPTQK
KQAAATCTAVATVMLQHLYYTVMLGLITVLLFDELLVKFRQIGGQELVHLYGGLKVALHLLHSLVSFKTSSDSN
QNTLFTSQVPETDPEYYESHDLLVKMRLAALPFIRDLWRCDWLVSAPPGVSKYVIQSVLDIVGGERDPRTAAVR
ALTRSHNNVSVATEYLLAHPELNLARETLTADLGTLALRLIDSHPSIRKFSPSAFDVHEEPLAVRCRLLALILP
TIPKWLACHLLVTESLLVLADEPRSITLPILTGPSYSEARPILFDFAFRLLGVLSLPRDELLATLRLLVQLTRD
HALASEFVRRDGVALLGCQSYIAIIFRHVVENKSTLESIMRQEVRRWFTQPRVVDVTSFVRTCAPMAARDPQTF
VKVTQSLCQLLHSESVVHFMIGELLRVGKYACFLMQCLTELLFSYDTCKHAFLSFSTKRHRTHALTFLLTDLVS
FGAFNAQKRMMLCNWAMSVIVALCIDCSKDVSTDLSSIRKTVLEAVSRSIKDAPSLESDDTRYGRLLALAELCH
RLLTVRFETPIQIAKIMLEKNFVATLTGILSDIDLNYPNMRSLVVAILRPLEHLTKIAIKMGREETPDLYRNSS

LGMYPEPMPHPLLIDRSESHDFSPLPTLQRWTEEAKISHGKFLNERFGKLCNHITLALLPDARERVTVMIHGNA
VDITDTGIDPTFLEALPDDMREEVLNQHFRERRSARVEQPPESTINPEFLEALPPEIRAEILQQERLEPAEIDP
ASFIASLDPQLRQVVLLDQDDVFLQSLPKPTSSRDAIQLLDRSSIATLVRLLFFPQKNILHKVLLNLCENSKSR
TDLFNLLLSILQDGSGDLVMVDRSFAQMSVRPELIAQRCLDALSFIVSSNELSSLFFLTEHELPVGLRRSKKGK
GKEKQAQSHYPIVLLLGLLDRQTLLTAPSIMDSVAGLLASVTRPLMSLKKMLLANPPQIPHPALRLIVNILTVG
ECSGRTFQQTLALIQHLTCIPDARDVIAQELKAKAQEFGQILYTNLDDLVKELISKFSPASSDQAKLLRVLKTI
DYMYSIYESFRFAPLWRRLGDCLAIVEQKPDVEHIATILLPLIESLMVVCKYVGSKESMEDLFVSFTDAHRKVL
NLMVRNNPSLMSGSFSLLIHNPRVLDFDNKRNYFNQQVHRRREHYGTLQLNVRRARVFEDSFQYLQRKTGDQIK
YGKLSVRFYDEEGVDAGGVTREWFQILARQMFNPNYALFEPCAADKQTYQPNRASAVNSEHLSFFKFVGRVIGK
AIYDGRLLDAYFARSLYRQLLGKPVDYRDVEWVDPEYYKSLCWILENDPTALDLTFIFGKRDIIPLKEGGASVP
VTQDNKREYVQLSAQYRLYSSIKEQIESLLGGFYEIVPKELISIFNEQEVELLISGTPDIDVDEWRAATEYNGY
SSSDPVIVWWWRALKSFNRDERAKVLSFATGTARVPLGGFVELQGVQGVQRFSIHRAYGDSDRLPQAHTCFNQI
DLPQYSSYEMLRQQLLLAINEGGEGFGFAKSSASSATRKKHARKLRKLAKKDTITKGKALEDLKHLPSLFLSSS
HRIRLLTAALHFSILGSWFLATHDIDRQVSHQSRKSWFLQRALLDPFGLYTDLNPDEGEEDRRGRLRCGALGGV
KWGHGQVAVRKSTWSVLYSLLSSAVLRSAWVEPEQTVRAGMWEPLLTFLTVMGIRYSYREFLQFLQLGSPIQGY
PTIVVILSTIPHTIFDSFWASLDGRALAFLGSLLECLVFLVKRPDIFIFGYLFLPSKDELDDMLDCSSSDPVDP
SLAIIQPLDASGYSTYARILNALLDRQLVRTNVWALRHFMVLDSYRESFVLYSVLQHVLSGATKDDADQWMILA
KSLEKHAPQTSIAITLAVTEYAPEPPRLDRYRNELAAGILGIPGLLLLRKLAATAPDPDSDVVYLPQLRAVNFV
KAYQQWVTSDIDEAVESEMTVILTHLAPILQNVAGSHWDLIFDMVENNLENCSFNDGTTLVMLARTLKLLLSIR
DLAETNKTLRTWQEREIPILTLVRDLSVPRSLCWESAISIEYLVVEAGVDTASLKVKMGYASQIRDLGIIATNF
LPTIFASLDLYRAFQLDIWAIEEYYIQLHEPLSLKLLAAHLFYRALLSVPSLIASWWNDCKDRTLSTAISSMTT
RSFSPVLIAAELAHVKDPVDEKRWRAWLLAVQQVQNGRIVDGLSIFKKNVTSHFEDQTECAICYSIIKRCKTCK
NRFHASCLYIPVSSKLDMS SVKTSSPRPF DLEDCPTFYP THEEFRDPMA YIKSISDRAR NSGICKVVPP
MGWKMPFVTD TEFRFKTRVQ RLNSIEASSR AKLNFLEALY RFHKQQGNPR VTVPTINHKP
LDLWLLRKEV HKLGGYEAVK GKKWADLGRL LGYGGPGLSM QLRNSYVRVI LPYEHFSDRV
RNSLCDGCDC GFHTFCLDPP LSVIPKGQWF CDTCLSVTVD FGFDEGEEHS LSRFQARDLA
FRKMWFESHP PVSEFDVENE FWRLVQSPHE TVEIEYGADV HSTTHGAMPT LETHPLDPYS
KDGWNLNNIP IVSDSLLRFI KSDISGMTVP WTYVGMVFST FCWHNEDHYT YSINFMHWGE
TKTWYGIPGE DAEKFEAAIR REAPDLFESQ PDLLFQLVTL MNPKRLTSAG VRVFACNQRA
GEFVVTFPKA YHAGFNHGLN FNEAVNFALP DWLPFGRDCV QRYQEHRKHP VFSHDELLIT
ITQQSQSIKT AIWLNDSLQE MTDREMAGRR LLRFKIKEVV EARDREDQYQ CAICKAFCYL
SQVICVCVAC LDHAQLLCDC RTLRMRIPDD ELLNTQCMIT SRAAVPGNWQ AKLRRVLSES
ARPPLRSLRA LLAEGDRINY PLPELPTLRK CVTRANEWVD TANSFTTRKQ SRKRDRPERG
LADLYALFTE VGRLGFDTPE IGLLKNLAAQ AEEVKARAAV LLCEALIAHG SSLNVHLDEL
YQVEDIVLQE QLIKDLLDEV RHLLARARAC HLSGDNKYMK ILEARLQAGN DWDQRAADVL
AQPIKTIQDL PVDPAVMIRI MTARAKALEF EKMAKAWLTP ELPRVSDALR LVHRAEREFS
IPSVHDLKRT AEFANDLEER CDRVLKNRYE HGPMFETMQK WRSYAREHLT FILPNFDKLN
KQLDLHQQWI QKLPWFCHGK DVLDDVIETT DPKDDIPPND EFFTCICFVP VRPPSNGEQS
DAVQCDHCSA RFHGLCAKNG GSCPFCDPHH WNGSIHKNRS WHFCYLPTVL HSAPEVTKSY
SQDWKDLETI VRRVDRLSGV IGQFLSSVSS RRLEMIAQVR HYMRKLYQMQ FAVSPNPEVS
FGLDLAGLHR ILAGPARKKK RRSKFIFGQD VDKDWIDGTR CICRGRTCKR QYHKACVYVC
PLCSLRKGRS YRYADVRVKT HSRELIKIRL PKPVTQTLFV ELVEYVP
>Rickenella mellea
MRRTSQDQSILLSGESGSGKSENRRLAIKSLLELSVSNPGKKGSKLSSQIPSAEFVLESFGNARTLFNPNASRF
GKYTELQFTRGRLCGIKTLDYYLERSRVAGAPSGERNFHIFYYLVAGATLEERQHLHLTDKMTYRYLGQRDALR
FDQLKLALKSIGLSKRHVAQSMQLLAAILHLGNLEFTIDRHRNEDAAVVRNTDILDIVSEFLGVQPAALETALS
CKMKLVKKELCTVFLDAEGASDNRDDLAKMLYSLLFAWLNESINQRLCRDDFATFIGLFDLPGPQNMSRSNSLD
QFCINFANERLQNWIQKRMFEFHVEEYASEGISRFVPQVPYFDNSECVRLLSNMPGGLIHIMDDQARRMPKKTD
HTMIEAFGKRWGNHSSFKVGAMDRFPTFTVNHFNGPVTYSAEGFLEKNLDALNPDFVSLLRGGSINPFVRSLFS
AKAIATQAHPRDEDTIVAAQQPVKPMRAPSTRRKNTPCVAGEFRKALSTLFTTLDETQPWFVFCVNPNDSQLPN
QLEGRAVKGQVRSSGLSEIARRSVNVFEANMMFEEFCERYGDQLAALNISEGERDVVIGQHKVFLSQAAFHKFE
DHLRAGDVEEQKRNRLRDAEASSYTLPLVQHAASFDFDDGKSRLTSHRESYAPSRNMFQTTDGEVMEGETTEVI
KESSGRRRWVALCWLLTWWIPNPILRWVGRMKRLDVRQAWREKLALNMIIWFVCLSTAFVIAVLGDLICPTEHI
FNTSELAGHSNNVFTSIRGEYGGTSADNIFPVQVSALCNGVSGSVNPYVILDSSNTDPNTQYHDFRISSTDPRP

DWYFESMVLMRWNYRVGFVGYTSKELKTMANAGRSVAVYNGLSLPAVMFTSKSSTFLTFKPQKSLRFTFLTIGT
RGDVQPYIALAKGLIADGHGIEFGYVGGDPAELMRLCVENGMFTVSFLKEGVQKFRSWIDDLLKTAWEACQGTD
VLIESPVAMGGIHIAEALKIPYFRAFTMTWTRTRAYPHAFAVPERKTYVMFDQVFWRGTAGQINRWRKHMLGLS
STSLDKLEPHKVPFLYNFSPTVVPPPLDWPEWIRVTGYWFLDDADGGSKTWSPPDGIVEFIDKAHNAGRKVVYS
DPDAMTRCVVEAIVKSGVHAILSKGWSDRLEPEIPLPPQIFPIQSIPHDWLFAGIPTIIKPFFGDQFFWADRVE
ALGIGSGVRKLTVDNLTEALAYATTDAKQIARAKLVGEQIRSENGDRPREIFEPLRLACETRNEKLMIASLDCI
SKLISHSFFIDLVDLVTSTITACHTETTPDTVSLQIVKALLSLVLSSTLLVHQSSLLKAVRTVYNIFIMSHDPV
NQTVAQGGLTQMVNHVFDMFIKDAFLVFRALCKLTMKPLNTESERDFTSEGMRSKLLTLHLVLTVLNAHMAVFA
EPWSIIYSTSSNETTTFINATKQYLCLSLSRNALSPVPQVFEISVELFWRVLSGLRTKLKKEIEVFFHEIFVPI
LEMKTSTLKQKGVILGMLLRLCQEPQALVEIYLNYDCDREAADNIYEHLMNTISKIATPSLSTAALAVPGAGLG
FSESQLKRQGLECLVAVLRSLVAWGTDDPGKFVSAKQRKTTLLEGIKKFNFKPKGIEFLVDSGFIPTRSPYEIA
KFLLQTDGMNKTMIGEGDDENIAIMHAFVDQLDFTNLPFVDALRVFLQSFRLPGEAQKIDRFMLKFAERYIAGN
DCAYVLSYSTIMLNTDAHNPQVKNRMTKLDFIRNNRGINDLPEDFLSAIFDDILSNEIRMKDEVANVGRDLQKE
AYVMQSHGMANDQFFSASHFIHVRPMFEVAWIPFLAGISGPLQDTDDMEVVELCLDGFKAFVTTLAKFTFLNNL
GEMKTKNMEAIKALLDIAVTEGNYLKGSWHEVLTCVSQLERMQLISSGRSRKLPAEELANESRSTHITVAADMV
FSLSHYLSGDAIVDFVKALSSVSWEEIQSSVASEHPRLFSLQKLVEISYYNMNRIRLEWSHLWDILGEHFNQVC
CHHNPHVGFFALDALRQLAMRFLEKEELPHFKFQKDFLKPFEYTMTHNANPDIRDMVLQCIQQMIQARVQNMRS
GWRTMFGVFSAASKVLTERVVNSAFEIVSRLNEDHFSAVVRYGAFADLTVCITDFCKVSKDDPMIKFWFPVLFS
FYDIIMNGEDLEVRRLALDSLFSTLKTYGSSFPVEFWDTVCQELLFPIFAVLKSTSDLTRWTTQEDMSVWLSTT
LIQALRDLIDLYTYFFDTLERFLDGLLELLCVENDTLARIGTSCLQQLLESNVKKLSPTRWERVTTTFVKLFKT
TTPHQLFDDSLRAERRRIFKQIIVKCVLQLLLIETTNELLQNDEVYNTIPPEHLLRLMGVLDHSYQFARVFNED
KDLRTALWKETSSAATLVNILARMYYDTRPEYQALRPQIVDKLLPLGLGVMQDFNKLRPETHAKNIASWTPVIA
EILNGFCRFDDKAFSRYMPVIYPPAAELLTKDVEVRLGLRDFFTRVGRAQGIQRQALLAAMQSKYGFEALGSRV
HQILTMMSLSSGTSLVQTLIELGPDITSDSDIVRALLNRFGITDQKPPRDAQIVEIFSNLGRLAAEGATLCDVG
ALVRALNSFNVQLNWAKVIESFDWPDRGVDTATLKLLISVLHNAPRAEPHAVAGFWTKWSNSLYQLRVLDALLS
LPSDTFNFVSLPGRRVVTVDEVASASPTIKSLAANVQSHTWNSLDLFEVLSRLGDAESPEVRGCIHDMLDKAVK
ISADLVHMGLLQVPPWNAIHMEYTQKLLSMFLGGHPNHQLVFMRIWQIEPSYLTTALREFYEESPMNITRILDI
LESLLEVRPFIFALDEYLNLDKWLADNVNQHGAEFLHAVIAFLELKAQNEKTSWLSDPSARTMALNPQTIAIFL
RVLRNSSSVMDDADVDYCLEVRNACLQIHPRLMDQEPGFSVVSYMPEVEAECDQIYKKMYDEQISIDDVIALLQ
RSKESTNPRDHEVFSCMLHFLFDEYKFFQYRARELHMTGYLFGSLIQYQLVDYIPLGIAIRFVLDALQCPPDTN
LFKFGVQALSRFESRLPEWKPLCQALAEIPHLAEARPDVAEAVERAEVSDKILFLVNNLAPSNFDAKTTEMKER
FSHEPNNHSLYLRFLDALDRPPLGRFILNETFVKSASVLNSEKTLQSASERALLKNLASWLDKPIKHKNVSFKD
LLIEGADSNRLIVAIPFVCKVLESCAKSKAFKPPNPWLMAVISLLAELYTFADLKLNLKFEIEVLCKALDLDLD
KIEPTSILRTRPRAVGAHIESILMNLANLVVINPQLAYHTNQSFKRAVQSAVDRSVREIIMPVVERSVTIAGIS
TRELVAKDFCTEQNEEKMRKAGHLMAQKLAGSLALVTCKEPLRSNMASHVRHYLSDHGFNEQQVILLIVQDNID
VACAAIEKAAMDRAVADVDDGFGQAYETRRRHREQRPGAPFIATLPEPLRIKASGLQMQQTRVYEDFFNLLVSE
LEGMLPHVPNSLTVLPTGHEVRGLVRRILFLADRNRTPLAMSQKIVQLLYKTSSQLGRELYVALLEQLCQSFED
VAKEAITWLIYAEDERKFNIQVTVTLLRSRLISVAQQDQQLAKDPRTTLQDFTAGLIRECLTSDAAQSQFSFAI
ECFQQWVNVFQRSSVPEKSFVPYITQLTKQGILKAEDSSFFFRVCTESSVDQYMKSVATGDYEHAFQALDAMSR
LIVYIIKYHGDANNDQAKVHYLTKILSIVVLVLASRHEEQGFQQKPFFRFFSSLLSDLHSVEAQLGTAYFQLLL
AIGDVLSSLQPTYFPGFAFSWMTLISHRLFMPKLLLSENREGWSAFHKLLLSLFKFLGPFLRTAHLELPSRDLY
RGTLRLLLVLLHDFPEFLSEYYFTLCDIIPPRCIQLRNIILSAFPPSITLPDPHLRNIKMGPIPPILSDFTFIL
KTGDLRGYLDQFLLNRGTQASLSALKDRLEVYNLSVINAVVMYIGVSSVAQAKARSGSSVFVSSDPGVVALTYL
AVNLDPEGQHHLLSSIVLHLRYPNAHTHWFSSLLLYLFSEVKDERFKEIMTKVLLERFIVHRPHPWGALVTFIE
LLRNPKYEFWSRDFIRVAPEVTLLLESVGHRRLYLQLCFRNVSDLLTVRREVVPLALANGAKLDAVDAYAEVVD
PRDAIVDVREFDVPYYLRVAIDNDIRVGLWYTVSFVAGHPQFERVKRADPVVMAYDIETTKAPLKFPDQAIDQV
MMISYMVDGQGFLITNREIVSEDIDDFEYTPKEGYEGPFTVFNEADEAATIMRFFQHIQTAKPTVMATFNGDFF
DFPFLCARAKVHGIDMFVETGFAIDSEDEFKSRTCAHMDCFRWVKRDSYLPQGSQGLKAVTTAKLGYHPIELDP
ELMTPYAMEQPQILAQYSVSDAVATYYLYMKYVHPFIFSLCNIIPLNPDDVLRKGSGTLCETLLMVEAYRGEII
MPNRHEEEHGNMFEGHLLASETYVGGHVEALEAGVFRSDISTHFKIEPSAAQLIDELDAALSFCVTNYDDVKSQ
IQAALEEMRDSPLRHDKPLIYHLDVAAMYPNIMLSNRLQPDSVVDEAVCAVCDYNRPGKTCDRRMTWAWRGEYF
PAQRDEYNMEWFPPKKPDGPKRRYTELAPAEQTALLHKRLGDYSRKVYKKTKDTKVVNRESIICQRENPFYIDT
VRTFRDRRYEYKGLLKTWKKNLDAVAEVDEAKKMVLAHKCILNSFYGYVMRKGARWHSMEMAGITCLTGATIIQ
MARQLVEQIGRPLELDTDGIWCMLPGIFPENFKFQLTNGKSVAFSYPCTMLNHLVHAKFTNHQYHDLDKETGTY

AVHSENSIFFELDGPYRAMILPSSKEEDKLLKKRYAVFNDDGSLAELKGFEVKRRGELQLIKIFQSQIFEKFLL
GTTTEECYAAVAEVADRWLDVLFSRADTLGDDELVELIAENRSMSKTLAEYGGQKSTSISTAKRLAEFLGDQMV
KDKGLACKFIISAKPIGATVTERAVPVAIFSADESVKRTYLRKWLKDNSLANFDLRSILDWDYYIERLGSVIQK
LITIPAAMQKVSNPVPRIRHPDWLHRRVAAIDDKFTQHKLTDFFRGRFTLWLSVNNDVVPVKLRIPREFYLNFK
SEHLYTRERVIRTLPRDRPCLHLFVNEMNNPNVDGVYELQTLNRARDHGVELDQLIRKYVFLYHAFSPSSPINV
FALFMPNGVKLHIVDPATRRQSTDYHGNEATALKAVSRDLGVMENKGYTLVLSSVKELSHFERSVSKLSKFPVL
CMPSNRAGHALDFPWQSNVGKKVLTRYFHLAPWLQRVVSQAAYYPLFLCDVEFSRRLLKQDMVLWWSAGEKPDL
GGLEDDMPATEELINPEFVSPGCYSNVCLSVQVRNLAINSILQAAIVNELEGSGGTTAFDSTQAAVTLGDSSMS
PQTFATLKAMVKTWLLDKASPSHSTIEHFWRWISSSGAHMFDPSIQRFIHGLMRKTFIQLLAEFKRLGSNVVYA
DFSRIVLVTSKPPGTAHAYATYITSAVTSHELFKHVLLRTDQFYDFLLFMDPANQGGVVCEDPLALEPPEQLAV
FMSWNINKFLPPAVQDRFRRIVRYFVVEMYKIRRSKMKEMDATRSFIAQRLTRRMLQTVFPLLPGSHLHLTDPA
LEFVKFTCAAFALAKDYQIEVGLLKRNVLDLVGVKEFSDQAVYHNPCDALKLSMVTCKYCDNLRDFDFCRDEDL
FPWFCSECDCEYDRVAIEFALIQIVHRLERSFAQQDLRCSRCKQIQSDNISKHCCSGSYQLTIPKADVRRKLRT
IVNVAIAHNLSRLFEVEHLKYATLATFPNLLTQKQVATIKTVRNVIEYNEQYVTKRLLVNIIWAFSGDAKGEWI
AWQSRVPAIEILYSWLSEHKPLMLCGPPGSGKTMTLFSALRKLPDMEVVGLNFSSYRKTPNGVILAPVQIGRWL
VVFCDEINLPATDKYGTQRVISLVECGGYYRPSDMAWVKLERIQFVGACGRVPLSHRFLRLVMVDYPGELSLKQ
IYGTYNRAMLKVVPSLRAYSEPLTDAMVEFYLSSQKRFTTDIQAHYIYSPRELTRWVRGIYEAIKPLEVLSVEG
LVRVWAHEALRLFQDRLVTEEEKTWTDDNIDSTAMQHFPTISILFSNWTSKYKARLRVFHEEELDVQLVLFNDV
LDHVLRIDRVFRQTTLSRFVAWMNGLSIFQIKVSNKYTGDDFDLLANAEVPGLFEGFTMNPPENGLASRAATSP
ALFNRCVLDWFGDWLPSYNPPQYFPLAYRVVNALVHVHISLHQINLRLSRRQGRYPRHYLDFIHHYVHLYNEKR
DELERHLHVGLDKLVTQVEELRKSLAIKRKLKRMVADQQEAEHKKAASIKIQAALVEQDRNIEERRAVVMADLA
DAEQMTKPLRDLMKRDFLSRPSYNFETVNRASKACMISELESSIRTYKDEYALLIRVQSKVDRSMTLLGSLSRT
FDTEMSTIVGDVLLSAAFLAYGEWSHHLTEANVKFKPELSFPRLSWQSKSLPSDSLCTVTSFLDEAFLKVLESA
LRFGNPLLIQDVEHLDPILNEIRRTGGRVLIRLGSQDIDFSPSFPSVDFSPDICSRVTSQSLDQVLKVETDLMK
VQGEFRLRLRTLEKLLLQALNESTGNILDDDKVIDTLETLKREAAETDVVMREVEQVTAEYLPLAQACSSVFFI
LEQLNLVNHFYQFSFLDIFDYVLHHNPNLKGVLFNDLFLVVYKRTSRALDELEFLLESGLMSYSKNALFKPVRE
HIQEHETDWVSFLLLIKCFRPDRLIQSVTGYDASYRVENQEGFTLADQAIATAARQGTWVLLKNVHLAPSWLGE
KKLQTLTPNRNFRLFLTMEANPVIPVNILQSRIIMNEPPPGIFLLAWFHAVVQEDMAAAFGTIDTWLNTVAKGR
TNVDPAVIPWEAVRTLVKQSVYGGRIDSDFDQKLLDTFVDALFTPAAYNLDFPEGTKVEHFKTWPSWLSLPPSA
ERRKMRTLEDDDLDRCKEWLSALPATFNSIQKQSTDNQDPLYRLFSREGHIGKLLGQVRKDLGDVLMSQLIKGT
IPTHWRRYKVTKVVDLARRLEQLDKIASLDNGGLFFPEAYVTATRAVAHRKRWSLETLDLRLDIENINDPGLVL
EGASWASDILPDTGFTLPPMQGRTIDEHFYRIGNAAAQPWLAFAKDFAEDMISFDVETMPNYHNYAVMACAMSS
THWYSWISPWLLGRIVVGHNVSYDRARVLEEYSIKGTKTRFLDTMALHVAVKGISSHQRPAWMKYRKAQKRWED
ITSANSLADVAKLHCNIEMDKEIRNDFMTHTPAEILANVHDYLDYCANDVHVTHSVFAAVLPQFLAACPNPVSF
AGILTMGSSFLTVDQEWEAYLEKAEGKYRELEEDIKKRLLALAEKAWRNDEWLSQLDWTPKWPKWYWELAKPKK
GTVDITVRNRISPLLLRLSWQGWPLFHSREYGWTFRVRQKPVTFFDKADVSLFYKLPHKDGESANVGSPLSKTF
IKYAQDGTLTSPGDEAKDALDMNAQCSYWISSRDRILKQMVLWQKWGMILPALITMGTVTRRAIEKTWLTASNA
KKNRVGSELKALVHAPPGYALVGADVDSEELWISSVMGDAQLGLHGATAIGWMTLEGTKAAGTDLHSKTANILG
ITRDQAKIFNYSRIYGAGMRHAVLLLLQSNASMKPEDAQKQAQKLYSSTKGKNTHRDLFGRKFWFGGTESFVFN
KLEEIALSDRPQTPALGCGITYALSKEYLSPGFGTDYMTSRINWVVQSSGVDYLHLLIVAMDHLITKYNIDARY
LISVHDELRYLVAEHDKYRAALALQIANLWTRSLFAYRLGMDDLPQGVAFFSAVDIDTVLRKEVDLPCVTPSQP
IPIPSGESLNIVQVLQKTNQGSLREDGYIKPDRLAHRSPSAAFLRAQATTELGELRRLAASLIADILAVSNEDL
SQYLASIDNWKWPRSDLNSWIKVLNRFDTILEEVIRDYDVDRLQTTIFTPRTKATVCEILKFERLLLENSTNRK
MFNSYDRLNSLLFSSDLDVLVASLLLLLRPSQQYSAQPALSHALHISTSRLASLAGRPPILREYGVDMLDLVSA
GKEKIRNLPQEASEVSLSFYRHLGPMAQSSRESMDVLADAIQTYDVPDEKYELLCRIRNAQALGETREKLVVIR
LLSIAVYAHTHTEAVAQSSLFLYEPDLVTRIAELLQLDRAVRKTVAEIASPHSTLSHLFVDALLSFVIYIASHA
AGGNMVVGAGLVPLLIQIIENKLPERLQVVSKTMQLVDNVLYGFTNAFQIFCNGRGVEVLAERIQYEVDGLLPF
ARTGVIKHTLRSMHRMMQSSGTTEGLRGLIDSSLLASVKKIIGHRGLFGPTVLPIAINIMATFVHNEPTSLVVI
QEAGLPEAFYEVIESGLEASIEVIQSIPNAIGALCLNQAGQDQLASRPSIIPGLLTIFTSERHLKVMQDKENSA
LIGSAIDELIRHHPSLKAIVFDALKSTLSKIEDMGSTYTPPSDIEQWYKLDNLVVSFIDILSKFLEGLFQHTDC
LARLTRMLALPCMPYDFANSVLVQVIRTMTEVAPTETLGHLAKEVKVSLEETRDFWQSLDGKSNNIFRRLLSLH
NQTTMLSDVYTSYTHGRGAVGLLLGALHRACLWENILLKTGLNARALKHVASQIPLAPLFQSVARRRHPDPTQK
KQAAATCTAVATVMLKHLYYTVMLGLITVLLFDELLVKFRQIGGQELVHLYGGLKVALHLLHSLVSFKTSSDSN
QNTLFTSQIPETDPEYYESHDLLVKMRLAALPFIRDLWRCDWLVSAPPGVSKYVIQSVLDIVGGEREPRTAAVR

ALTRSHNNVSVATEFLLAHPELNLARETLTADLGTLALRLIDSHPSIRKFSPSAFDVHEEPLAVRCRLLALILP
TIPKWLACHLLVTESLLVLADEPRSITLPILAGPSYSEARPILFDFAFRLLGVLSLPRDELLATLRLLVQLTRD
HALAGEFVRRDGVALLGCQSYIAIIFRHVVENQSTLESIMRQEVRRWFTQPRVVDVTSFVRTCAPMAARDPQTF
VKVTQSLCQLLHPESVVHFMVGELLRVGKYACFLMQCLTELLFSYDTCKHAFLSFSTKRHKTQALTFLLTDLVS
FGAFNAQKRMMLCNWAMSVIVALCIDCSKDVSTDLLSIRKTVLEAVSRSIKDAPSLESDDTRYGRLLALAELCH
RLLTVRFETPIQIAKVMLEKNFVATLTSILSDIDLNYPNMRSLVVAILRPLEHLTKIAIKMGREETPDLYRNSS
LGMYPEPMPHPLLIDRSESHDFSPLPTLQRWTEEAKISHGKFLNERFGKLCNHITLALLPDARERVTVMIHGNA
VDITDTGIDPTFLEALPDDMREEVLNQHFRERRSARVEQPPESTINPEFLEALPPEIRAEILQQERLEPAEIDP
ASFIASLDPQLRQVVLLDQDDVFLQSLPKPTSSRDAIQLLDRSSIATLVRLLFFPQKNILHKVLLNLCENSKSR
TDLFNLLLSILQDGSGDLVMVDRSFAQMSVRPELIAQRCLDALSFIVSSNELSSLFFLTEHELPAGLRRSKKGK
GKEKQAQSHYPIVLLLGLLDRQTLLTAPSIMDSVAGLLASVTRPLMTLKKMLLANPPQIPHPALRLIVNILTVG
ECSGRTFQQTLALIQHLTCIPDARDVIAQELKAKAQEFGQILYTNLDELVKDLISKFSPASSDQAKLLRVLKTI
DYMYSIYESFRFAPLWRRLGDCLAIVEQKPDVEHIATILLPLIESLMVVCKYVGSKESMEDLFVSFTDAHRKVL
NLMVRNNPSLMSGSFSLLIHNPRVLDFDNKRNYFNQQVHRRREHYGTLQLNVRRARVFEDSFQYLQRKTGDQIK
YGKLSVRFYDEEGVDAGGVTREWFQILARQMFNPNYALFEPCAADKQTYQPNRASAVNSEHLSFFKFVGRVIGK
AIYDGRLLDAYFARSLYRQLLGKPVDYRDVEWVDPEYYKSLCWILENDPTALDLTFIFGKRDIIPLKEGGASVP
VTQDNKREYVQLSAQYRLYSSIKDQIESLLGGFYEVVPKELISIFNEQEVELLISGTPDIDVDEWRAATEYNGY
TSSDPVIVWWWRALKSFNRDERAKVLSFATGTARVPLGGFVELQGVQGVQRFSIHRAYGDSDRLPQAHTCFNQI
DLPQYSSYEMLRQQLLLAINEGGEGFGFAKSSASSATRKKHARKLRKLAKKDTITKGKALEDLKHLPSLFLSSS
HRIRLLTAGLHFSILGSWFLATHDVDRQVSHQSKKSWFLQRALLDPSSLYTNLNPDESEEDRRGRLRCGALGGM
KWGHGQVAVRKSTWAVLYSLLSSAILRSAWVEPDQTVRAGMWEPLLTFLTGNRQAWSYREFLQFLQLGSPIQGY
PTILVILSTIPYTIFDSFWASLDGRALAFLGSLLECLVFLVKRPDIFIFVYLFPPSKDELDDMLDYSSSDPVDP
SLAVIQPLDASGYSTYARILNALLDRHLVRTNIWALRHFLVLDSYRESFVLYSVLQHVLSGTTKADADQWMILA
RSLEKHAPQTSMAITLAVTEYAPEPPRLDRYRNELAAEILGIPGLLLLRKLAATAPDPDSDVVYLPQLRAVNFV
KAYQQWVTSDIDEAVESEMTVILTHLAPILQNVAGSHWDLIFDMVENNLENCSFNDGTTLVMLARTLKLLLSIR
ELAETNKALRTWQEREMPILILIRDLSVPRSSCWGSALSIEHLVVEAGVDTASLKVKMGYASQIRDLGIIATNF
LPTIFASLDLYRAFQLDIWAIDEYYIQLHEPLSLKLLAAHLFYRALLNIPSLIASWWNDCKDRTLSTAISSVTT
RSFSPVLIAAELAHVKDPVDEKRWRGWLLAVQQVQNGRIVDGLSIFKKNVTSHFEDQTECAICYSIIKRCKTCK
NRFHASCLYIPVSSKLDMS SVKTSSPRPF DLEDCPTFYP THEEFRDPMA YIKSISDRAR NSGICKVVPP
MGWKMPFVTD TEFRFKTRVQ RLNSIEASSR AKLNFLEALY RFHKQQGNPR VTVPTINHKP
LDLWLLRKEV HKLGGYEAVK GKKWADLGRL LGYGGPGLSM QLRNSYVRVI LPYEHFSDRV
RNSLCDGCDC GFHTFCLDPP LSVIPKGQWF CDTCLSVTVD FGFDEGEEHS LSRFQARDLA
FRKMWFESHP PVSEFDVENE FWRLVQSPHE TVEIEYGADV HSTTHGAMPT LETHPLDPYS
NDGWNLNNIP IVSDSLLRFI KSDISGMTVP WTYVGMVFST FCWHNEDHYT YSINFMHWGE
TKTWYGIPGE DAEKFEAAIR REAPDLFESQ PDLLFQLVTL MNPKRLTSAG VRVFACNQRA
GEFVVTFPKA YHAGFNHGLN FNEAVNFALP DWLPFGRDCV QRYQEHRKHP VFSHDELLIT
ITQQSQSIKT AIWLNDSLQE MTDREMAGRR LLRFKIKEIV EARDREDQYQ CAICKSFCYL
SQVVCVCVAC LDHAQLLCDC RTLRLRISDD ELLNTQCMIT SRAAVPGNWQ AKLRRVLSES
ARPPLRSLRA LLAEGDRINY PLPELPTLRK CVTRANEWVD TANSFTTRKQ SRKRDRPERG
LADLYALFTE VGRLGFDTPE IGLLKNLAAQ AEEVKARAAV LLCEALIAHG SSLNVHLDEL
YQVEDIVLQE QLIKDLLDEV RHLLTRARAC HLSGDNKYMK ILEARLQAGN DWDQRAADVL
AQPIKTIQDL PVDPAVMIRI MTARAKALEF EKMAKSWLTP ELPRVSDALR LVHRAEREFS
IPSVHDLKRT AEFANDLEER CDRVLKNRYE HGPMFETMQK WRSYAREHLT FILPNFDRLN
KQLDLHQQWI QKLPWYCHGK DVLDDVIETT DPKDDIPPSD EFFTCICFVP VRPPSNGEQS
DAVQCDHCSA RFHGLCAKNG GSCPFCDPHH WNGSIHKNRS WHFCYLPTVL HSAPEVTKSY
SQDWKDLETI VRRVDRLSGV IGQFLSSVSS RRLEMIAQVR HYMRKLYQMQ FAVSPNPEVS
FGLDLAGLHR ILAGPARKKK RRSKFIFGQD VDKDWIDGTR CICRGRTCKR QYHKACVYVC
PLCALRKGRS YRYADVRVKT HSKELIKIRL PKPVTQTLFV ELVEFVP
>Fomitiporia mediterranea
MRRTQQDQCILLSGETCSGKSENRRLAIKSIIELSVSNPGKKGSKLSTQIPSAEFVLESFGNARTLFNPNASRF
GKYTELQFTRGRLSGVKTLDYYLERGRVAGAASGERNFHIFYYLVAGASAEERQHLKLTDKSTFRYLGPRDAMR
FDQLKVALKNVGLSKRVVAQTCQLLAAILHLGNLDFIIDRQRNEDAAVVKNTDILETVAEFLGVQPQALENALS
CKMKLMKKELCTVFLDPDGASDNRDDLAKILYSLLFSWLNEQINQKLCRDDFSTFIGLFDLPGPQNMSRSNSLD
QFIVNFANEKLHSWIQKRMFESHVDEYNQEGIAHYVPPIPYFDNAECVRLLSHMPGGLIHIMDDQARRMPKKSD

HTMVEAFGKRWGNHSSFKVGGIDRFPTFTVNHYNGPVTYSSEGLLEKNLDALNPDFVSLLRGGSVNSFIRNLFC
GKAIATQAHPRNEETIVAAQQSVKPMRAPSTRRKGTPCTAGEFRQALVTLFSTLDETQAWYVFCINPNDSQIPL
QLEGRAVKGQIRSAGLTEIAKRNANVFEVSMTPSEFCDRYGAHLLALNIHEGERDLVIGSFKVFLSHRAFHKLE
DRLRAEDVEEQKRNRLRDAEASSQQLPLVSHASPFDDLDAKSRLTSNRESYAPSRNMFQNDDGEVMEGETTEDI
KESSSRRRWVAFVWLLTWWCPNIFLIWCGRMKRLDVRQAWREKLALNMLIWFCCLVAAFIIAVLGNLICPTEHV
FSSSELQAHNNSMLTAIRGEYAGTTADDLFPVQVSAVCNGVSGSVNPFVTLNSKNTDPNAQYHDFRAWKNDSRP
DWYFESMTLMRWNYRVGFVGLTGKQVKNLATSGRAVAIYNGLDLPAVMFTSTSSTFLTFKPRHPLHFTCLTIGS
RGDVQPYIALAKGLMADGHGIEFGYVGGDPAELMRICVENGMFTVSFLKEGIQKFRGWIDDLLKTSWEACKGTD
VLIESPSAMAGIHIAEALRIPYYRAFTMTWTRTRAYPHAFAVPEHKSYVMFDQVFWRATAGQINRWRRETLGMS
STNLDKLEPHKVPFLYNFSPTVVPQPLDWPEWIRVTGYWFLDDADVSAEKWSAPKDLVDFIDSAHQAGKKVVYS
DPEAMTRCVVEAIIRSGVYAILSKGWSDRLEPEVPLPSQIYTIKSIPHDWLFAGIPTVIKPFFGDQYFWGDRVE
ALGVGSCVRKLSVEALSEALTLATTDEKQIAKARLVGERIRSENGDKPREIFEPLRLACETGNEKLQIASLDCI
SKLISYSFFLELVDIVTHTITACHTETAPDAVSLQIVKALLSLVLSPTLLVHQSSLLKAVRTVYNIFLLSSDPV
NQTVAQGGLTQMVHHVFDLFFKDAFLVFRALCKLTMKNLNTESERDLRSHAMRSKLVSLHLVLTILNSHMQVFV
DPSSIIYSASTNEATSFINATKQYLCLSLSRNAVSPVPQVFEISVEIFWRLLTGMRTKLKKEIEVFFHEIFVPI
LEMKTATLKQKSVILGMLQRLCQEPQALVEIYLNYDCDREAADNIYEHLMNTLSKISSPALSTSALAVPGQNLG
LSEQQLKRQGLESLVAVLRSLVTWGTDDPGKFESAKQRKTILQDGIRRFNYKPKGVEFLIQNGFIPSREPVEVA
KFLLNTDGLSKAVIGEGDDENIATMHAFVDQLDFSGMAFVDALRTFLQTFRLPGEAQKIDRFMLKFSERYIAGN
DTAYVLSYSTIMLNTDAHNPVVKQRMTKADFIKNNRGINDLPEEFLSEIFDDIQTNEIRMKDEMANVGRDLQKE
AYVMQSLGMANDQFFSASHFVHVRPMFEVAWIPFLAGISGPLTDTDDLEVVELCLEGFKAFVTTLAKFTFLNNL
GEMKAKNMEAIKALLDIAVSDGNHLRSSWHEVLSCVSQLERMQLVSNGRARKMPAEELANESRSTHITVAADMV
FSLSHYLSGTAIVEFVRALSAVSWEEIQSSGLSEHPRLFSLQKLVEISYYNMNRIRLEWSNLWEIIGEHFNQVC
CHHNPHVGFFALDALRQLAMRFLEKEELPHFKFQKDFLRPFEYTMIHNNNPDVRDMVLQCLHQMIQARVHNFVS
GWRTLFSVFSAASKVLTERVVNSAFELVTRLNKEHFAEIIRHGAFADLTVCITDFCKVSKDDAMIKFWYPVLFS
FYDIIMNGEDLEVRRLALNSLFTTLKTHGSTFSVEFWDTVCQELLFPIFAVLKSSSDLSRWSTQEDMSVWLSTT
MIQALRDLIDLYTFYFETLERFLDGLLDLLCVENDTLARIGTACLQQLLENNVKKLSAGRWERVVTTFIKLFRT
TTPHQLFDENLRGERKRIFKQIIVKCVLQLLLIETTSELLQNNEVYDTIPPEHLLRLMGVLDHSYQFARMFNED
KELRTALWKETSSADTLVTVLSRMYYDPRPQHLALRAQIADKFLPLGLGVITDFNKLRMESSAKNISAWMPVVA
RIVQGFCGLSDKAFGRFLPATYPAVSELVARDSEVRSHLRDYFVRVGQFQGIQRQALVFSAQKKFGAELVSPIL
AQLFQKLSLPPQTTLVQALNQLGPDISNDVEVVRGLLVRFGISDQNLPTNEQITDTFMTLSRLTAEGAQLCDVG
TLVRVLSSFRAQLDWSKAIEVFDWPERGVDTNTLKLLIAILVNSPRADKPAVAGFWSNWKNSLYQLRLLDALLS
LPSDTFNFVSLPGRRVVTVDDVAAASPTIKTLAANVQSHTWNSLDLFEILVRLGDSEIENVRSYVREMLDKAVR
ISADIVHMGLVQVPPWNALQVEYSRQLLGMFLGGHPNHQLVFMRIWQIEPTYLLTALREFYDENPMNITRILDI
LDSLLEVRPFVFALDEYLNLDKWLSDNIEKYGADFLHSIILFLDHKMQNEKIARTVDPQARTMALSPTTIAIFL
RALRTFSNVMDERDAEYCVETRNACLQIHPRLMNQEPGFNVVSYSTDIEAEVDGIFKQMYDEQITIDQVIIMLQ
RTKESTNTRDHEIFSCMLHFLFDEYKFFQYPPRELAMTGYLFGSIIQQQLVDYIPLGIAIRYVIDSLQCPPDTN
LFKFGVQALTRFESRLAEWKLLCEALLNIPHLAEQRPDIIEAVRRAETSDKILFIINNLAPSNFEAKLTEMKER
FSTEPNNHQLYLRFLDGLDSKPIAKFILHETFVKSASMLNSDKTKSSTSERTILKNLASWLDKPIKHKNLSFKE
FLIEGADSDRLVVAIPFVCKVLEGAAKSKAFRPPNPWLMAVISLLAELYHFAELKLNLKFEIEVLCKALSIDLD
TVEVANILRSRPRTVGAHIEEILANLATSVIINHQLALHTNHAFKQAVQEGIDRAVREIILPVVERSVTIASIT
SRELCVKDFASEPNEEKLRKAGHMACQKLAGSLALVTCKDPLRTNMAAHIRSYLLDHGFTEQQVIMLIVQDNID
VACEAIEKAAIDRAIKEVDAALAQSYDARRRYRDVRSGSAFIAGLPDPLRIRPNGLQPLQQRVYEEFFNILVQK
LESILSEIPTSLALVPSNHELRNVVNRVLSLADRVRTPLFMSQKIVQHLYKTPSQIGRDIYVALLDQLCQSFEE
VAKEAINWLICAEDERKFNIPATVTLLRSHLITPTDEDMQLAKNSRPSLQDFAAGLIRECLTSETTQHQFQYTL
DRFQQWVGIFQSSSNPEKMFVQYVTALSKQGILKVEDSSFFFRVCAESSISHYTKSVAAGDFGHSFLALDAMSR
LIVYIIKYHGDANNLQAKVHYFTKILSILVLVVANRHEEQGFQQKPFFRFFSSLLSDLSSLGDQLGNVYFHLLV
ALSDTFSSLQPVYFPGFAFSWMTLISHRLFMPKLLLSENRDGWSAFHKLLLALFKFLAPMLRSANFTLASRNLY
RGGLRLLLVLLHDFPDFLSAYYFSLCDVIPPRCTQLRNIVLSAFPASITLPDPSLRNTKMGPIPLVLSDFSAIL
KNGDLKGYLDQCLLSRMPQTSLATLKERMETYNVPLINAMVMYIGVSSVAQVKARSGSSVFIPTDPGVVALTYL
AYNLDPEGQYHLANAMILQLRYPNAHTHWFCCLMLYLFEHAKDDRFREIMTRVLMERFFVHRPHPWGALLTFIE
LVRNPKYDFWNKDFLRVAPEVTAILDNVGHRRLYLQLCFRNVSDLLAVRREIVPLAMENGAKLSAVDAYAEVVD
PRDAIIDVREFDVPYYLRVAIDNELRVGLWYNVSFTAGQPQFDRVKRPDPVVLAYDIETTKAPLKFPDSATDQV
MMISYMIDGQGFLITNREIVSEDIDDFEYTPKEGLEGPFTIFNEPDEAATITRFFQHFQEVKPTVVATFNGDFF
DFPFLCARAMVHGIDMFLEIGFSKDSEDEYKSRTCVHMDCFRWVKRDSYLPQGSQGLKAVTTAKLGYDPIELDP

ELMTPYAMEQPQTLAQYSVSDAVATYYLYMKYVHPFIFSLCNIIPLNPDEVLRKGTGTLCETLLMVEAYRGHII
MPNRHEDPHGNMFEGHLLSSETYVGGHVEALEAGVFRSDIPTNFKIVPEAAQLIDELDAALTFCVVNYEQVKGQ
IQAALELMRDKPLRFDKPLIYHLDVAAMYPNIMLSNRLQPDSVVDESVCAVCDYNRPGKTCDRRMTWAWRGEYF
PAQRDEYNMEQFPAKRPGGPQRRFNELSPAEQSALLHKRLGDYSRKVYKKTKETRIVQRETIICQRENPFYVDT
VRRFRDRRYEYKGLHKTWKKNLDAVAEVEEAKKMILAHKCILNSFYGYVMRKGARWHSMEMAGITCLTGATIIQ
MARQLVEQIGRPLELDTDGIWCMLPEVFPENFKFQLANGKTIGFSYPCTMLNHLVHAKFTNHQYHDLDPETSQY
KVHSENSIFFELDGPYKAMILPSSKEEDKLLKKRYAVFNDDGSLAELKGFEVKRRGELQLIKIFQSQIFEKFLL
GSTTQECYAAVAEIADRWLDVLYSRAESLSDEELVELIAENRSMSRTLAEYAGQKSTSISTARRLAEFLGDQMV
KDKGLACKFVISAKPIGAPVTERAVPIAIFSAEESVKRTYLRKWLKDNSLASFDLRAILDWDYYIERLGSVIQK
LITIPAAMQKVPNPVPRIRHPDWLHRRVVALDDKFKQHKVTDFFRGRFTMWLCVNAELIPVTLRIPREFYLNFK
APEHYIRDRVIRTLPRDRPCFHLFTNEINNPNVDGAYELQTLNRARDNGVDLWQMDRKYLFLYHAYTASAPVHV
YALFLPNGVKLHVVDSATRRQLTDYHSNETTALKAISRELGALEKKSFTIVLSSAKDFTYFSSSVPKLNRFPVL
RMPSTKASHILDFPWQSAIAKKMFLRYFSLAPWLQRTIAQAAYYPLFFCDIELARRLSAQDMVLWWSAGEKPDL
GGFENDMPPAEELTNPEFLSPGLYSKVCLSIQVGNLAIDSVLQSALVNELEGSGGTTAFDSAQPNVTLGDSNLS
PQMFAIIKQMVRAWLLDKASPVSVTLDHFWRWVSSSSAMMYEPSVQRFIHGLMRKTFIQLLAEFKRLGSNVVYA
DFSRILLVTSKPPGTAHAYATYITTAVTSHDLFKHIYLRTERFYDFLLYMDPANYGAVVCEDPLAIEPPKQIAI
SSTWNINKFLPPAVQGHFRTVVRYFIAQMAKIRREKIKEIDESKVFIAQKLTRRMLQVVFPVLPGSYLHMTEPT
LEFIKFSCAVFSLAQDYQIEIGLMKRNLLELVGVREFSDQALFRNPCDPLKLSMVTCCYCDHIRDFDFCRDDDL
LPWYCPECDGEYDRTAIEFALIQHLHRLERNFVQQDLRCARCKQIQSDNVSRHCCSGNYQLTTSKADVRRKLRT
IINVAITHNLTRLFEVEHLKYATLATSPNLRTQREIAGIKTARNVLEYNEQYVTKRLLVNIIWAFSGDARGEWI
AWSSRVPVIEILYSWLSEHKPLMLCGPPGSGKTMTLFSALRKLPDMEVVGLNFSSYRKTPNGVILSPVQIGRWL
VVFCDEINLPATDKYGTQRVISLVECGGYYRTADMAWVRLERIQFVGACGRVPLSHRFLRLVMVDYPGEISLKQ
IYGTYNRAMLKVVPTLRAYAEPLTDAMATFYLESQKRFTTDMQAHYIYSPRELTRWVRGIYEAIKPLEMLSVEG
LVRVWAHEALRLFQDRLVSEDEKVWTDENINSTAMQYFPTINILFSNWTSKHKARLRVFHEEELDVQLVLFNDV
LDHVLRIDRVFRQTTLSRFVAWMNGLSIFQIKVSNKYTGEDFDLLANAEVPGLFEGFTMNPPENGLASRAATSP
ALFNRCVLDWFGDWLPAYKPPQFFPIAYRVVNALVYIHQSLYQINKKLSRRQGRYPRHYLDFISQYVRLYNEKR
DELERHLHVGLDKLVTQVEELRKSLAIKRKLQRMVSDQQEAEQKKAASIEIQAALVEQDKHIEQRRSIVMADLA
DAEQMTKPMRELMKREYLARPTFNFETVNRASRACMIAELEASIRTYKEEYALLIRVENKVERSMRMLDSLSQT
FDEEMSTIVGDVLLSAAFLAYGEWSHHLHEAHIKYKAELAFPRLSWQSKSLPSDSLCIVTSFLDDAFLKVLESA
LRFGNPLLIQDVEHLDPILNEIRRTGGRVLIRLGSQDIDFSPSFPSVEFSPDICSRVTSQTLDQVLKVETDLMK
LQGEFRLRLRTLEKLLLQALNESTGNILDDDKVINTLETLKKEAAETDVVMREVEQVTSEYLPIAQACSSVFFI
LEQLNLINHFYQFSFLDIFDFILHHNPNLQNVLFDDLFLVVFKRTSRALDEYEFLLESGLDNYAKHPMFKPVLD
SIRSNESDWVPFLLLIKCFRPDRLVSSVTGYDASYRVEGQEGFALADQAIAVAARQGTWVLLKNVHLAPSWLGE
KKLQTLNPNRSFRLFLTMEANPVIPVNILQSRVIMNEPPPGIFLLAWFHAVVQEDMSAAFGTIDVWLNSIAKGR
ANVDPVSVPWDAVRTLIKQSVYGGRIDSDFDQKLLDTFVDSLFTPAAYNLDFPEGTKIDHFLSWPSWLSLPPTA
ERRKMKSLADDDSEHCREWLALLPENFNTLAKQAVENQDPLNRLFLREGEIGKLLNQVQRDLGDVLLSHLTKGT
IPTHWLRYKVKKIPNLARRLAQLDRIAGLDTGDLFFPEAYVTATRAVAHRKKWSLETLDLRLDIERVNDPGLVL
EGASWSSDILPNTGFTLPPLQGSSIDEHFHRIGHAASEPWLGLAKQFAEEMLSFDVETMPNHHQYAIMACAMSP
THWYSWISPWLLGRIIVGHNVSYDRARVLEEYHVKGTNSRFIDTMSLHVAVKGISSHQRPAWMKYRKNKKRWED
ITSANSLADVAKLHCGIDIGKEIRNDFMTRSREEIFEGINDYLDYCATDVEITHAVYTKVFPDFLQACPSPVSF
AGVMTMGSSFLTVNEQWENYLRNAEGIYRELNEKIKKRLISIAEDVWKDDVWLSQLDWTPKWPKWYWDLAKPKK
GTLDLTIRSRLAPLLLRLSWSGWPLFHSREHGWIFRVRSKKLDFYDPADAHLFYKLPHKDGESANVGSPFGKTF
MKYSQDGTLTSREGDTKDALDMNAQCSYWISSRDRILNQMVVWNKVGMIIPQVITMGAVTRRAIEKTWLTASNA
KKNRVGSELKAMVRAPKGYAIVGADVDSEELWISSAMGDAQFGLHGATALGWMTLEGTKAAGTDLHSKTASILG
ISRDQAKVFNYSRIYGAGMRHAILLLLQSNPNMLPDIAQQQAEKLYASTKGKNTHRDIFGRKFWFGGSESYVFN
KLEEIAMSDKPRTPALGCGVTYALSKEYLPAGFGTDYMTSRINWVVQSSGVDYLHLLIVSMEHLIAKYDIKARY
LMSVHDELRYLVREEDKYRAALALQIANLWTRCLFAYKLGMDDLPQGVAFFSAVDVDHVLRKEVDLPCVTPSQP
TPIPPGESLNIIKALEKTNGGSLWRDGYVKPDCLIHRASSANFLRAQATTEFGEIKQLAAALIADILATPTNGL
SELLAPIDAWKWPRSDLNAWIKVLNKFDAVLEDVIREYDIDGLQVKPFTRETKEIICEVLKFERLLLENSTNRK
MFNSYDRLNSLLFSSDLDVLVATLLLLLRPSQQYSSQPALSHSLHISTSRLESLAKTSPMLREHAIEMIDLVSK
GGKRLRDLPQEASEVNFTFYSHLGPLAQSSRSAIEIFADAVKSHQVPDERYELLCRVRFAQALGAGRQKLVIAR
LLAIAVYAHTHSETQAQSSLFLYDTDLVNRIAELLQQDHEVRKTVADISNIESTLPNLFVDALVSFITFIASHA
GGGNLVVGAGLVPILIQIIGITHEQRLPIVSKSMQLVDNVLYGVMNAFTLFCNSRGVEVLTERIKYEVDGLMHT
TRAGVLKHLLRSIHRMMQASGTTEGMRGLIDSSLLKSVQNIIEYRGVFGPTVLPIAINVVATFVHNEPTSLTAI

QEAKLPETILKAFEAGIEPSFEVVQSIPNALGALCLNQAGQDHLALHPSIIPALFSIFTSEAHLKVLLEKENAV
SIGSSIDELVRHHPSLRQIVFDSLISTLKKIEVMGTNYVPPCDIQQFYSLDNVIINYIDVIGRFLEGLFQHTEG
LACIARLLALPCLPYDYANAVLVQVIRTMVEVAPADTLTHLAQQVSESLNETKGFWQSTNGESNAFFHKLITLH
VRITLLSDVYSTYTHGRHAVGLLLGALHRSCIWENIVLKNGLNAVALKHIASQIPLGPFFQAVAKRRNPDEAQK
KQAASISATLADVMYKHLYHTVMIGLITFLLFDELLLQFRKVGGQELVHVFGGLKMALHLLYLMISFKPAIDPG
QIAQYISKKPENHPDFYEPHDFLVKMRLQIAPLIRDIWQSTWLVSAPPAVSKYVIQCVQEITSGENEPRAAAVR
ALSRTNNNVNFATEYLLTHPDLDEIRKSLISDIGPQALRLADAHPSIEKFSPAAYDVQEEPLAVRCRLLALILQ
ALPKWLPALLLAMESLLVTAEEPRAVPMVLVVGPPYVEARSMLFELCIRLLHIPSLPRDELLATLRMLVQLTRD
RNMADQFVRRNGVSLLGFQSHIAIILRHLVEDRLVLETVMKQEIKRWLSTSKTVEVLTYVRNSTSMAARDPQVF
LDITKELCTLVRPESVVHFMLGELMRVGKYACFLMQSLSELLFSYEQCKFAFLTYPKKRSKSSALSFLLTELLS
FGPFNSKKRVILCNWAMSVIVALCVDSSQENAPDVTSIRKLVLESISRSFKDILATEPMDVRYGRILAMADLCH
RLLSVRFETPMHLAKIMLEKNFVSTLTTVLAEVDLNYPNMRSLVAAILRPLEYLTKVAIKMGREETPDLYRNSS
LGMYAEAMSHPLLVDRTDTPEFGPLQTIQRWSEEAKITHGKHLQERVQRLCNHIVLALLPDARERVTITINGNE
VDITDTGIDPTFLEALPDDMREEVLNQHLREQRPTQVAPPVESQISADFLDALPPEIRAEILREERLEPTDMGA
ADFIASLDPQLRQVVLLDSDDGILQTLPKVPTPRDAIQLLERPGIAALVRLLFFPQKSTLHKVLLNLCENSKSR
TELFNVLLSILQDGTGDLALVDKSFSQLTFRPDLIAQRCLDALNYIVGTNELSSLFFLTEHELSAGLKRSKKGK
GKEKQAQSHYPIVLLLGLLDRQTLLKTPSIMDSVAGLLDSVTRPLTSLKKILLANPPQIPHPALRSIVNILTVG
ECSGRTFQHTLALIQHLSFLPDARDIIAQELRLKANDFGANLSKDLDELITALVAKFSPASSDQAKFLRVLKTI
DYMYSIYETFRFTLLWEKLSECLSVVESRDNVEHIATILLPLIESLMVVCKHVGVKQSVDDLFVSFTDDHRKIL
NMMVRNNPSLMSGSFSLLVQNPRVLDFDNKRNYFNQQLHKRREHYPSLQVNVRRSRVFEDSFHAFQHKTGDQIK
YGKLSVRFYAEEGVDAGGVTREWFQILARQMFNPNYALFEPCAADRQTYQPNRASEINPDHLSYFKFVGRVIGK
AIYDGRLMDAHFARSLYRMLLGKRVDYRDVEWVDPDYYKSLCWILENDPSMLDLNFIFGRHAVIPLKENGASIP
VTMENRKEYVQLAAQYRLHSSIAKQIENLLAGFYEIVPKELISIFNEQEVELLISGTPDIDVDEWRAATEYHGY
SSSDPVIVWWWRALKSFNRDERAKVLSFATGTTRVPLGGFGELQGVQGVQRFSIHRAYGEPDRLPQAHTCFNQI
DLPEYSSYERLRHQLLLAINEGGEGFGFAKSSATSATRKKHALKLRRLGKKDSITKRKALEELQHLPSLLLNVS
RRIRLLAAAIHTSLLGSWCMAAEDVDRMVSIQAKQAWFIRRALFDPMAVYSDLNPEESDDDRKGRFRVGALGVL
RWGDEQPAARKAGWSVLRILLSSAVLRSAWVEPDSGVQSSMWEPLLKFLTSFPQAWAFREFLLFLELGSPAQGY
PTIVIILSTIPPPILTSFWAALDGRALAFLSALLECLILLIKRPDVFVFGYILPPSQDQLEALLYDLPSNPVDP
ILAIVDPLDRSGLCEFARVVNSLLSRDFARQNIWLLRHFLILDDLQESRAFCTVVQYVLNGAVRKDAEQWMTLA
KRLENRSPNTALAIAHAVNKSGLEPPSLDRYRNELAASALGVGGVALLHRLNLVAPDPNSDVVFLPQNRAINFM
KACQSWIASDISEDVESEMTLVFQHLAPILQTVSGAHWDFVFDVVENNLENCSFVDTSSLVALTRTLKLILIIQ
DLCGSNKSLRAWQEREMQILSLVRNISIPRSICWEMALKIEYLVVEAAVETASLRVKIGYATQLRNLDLIGSNF
LPFIFDLLEVYKPFPLEQWSVEEYFVQFYDTLSPRLLAAHLFYRALLNVPSLIASWWTGCKDRQLVIAVSNLTT
KYYSPVLIAAELQHVKDPVEENRWRGWLLAVQQIQNGRIVDGLSIFKKNVTHHFENQTECAICYSIIKRCKTCK
NRFHAGCLFISVSSTLDMS TVKTTSPRLF ELEDCPTFYP SPEEFKDPMS YIRSISPRGQ EYGIIKIVPP
IGWKMPFVTD TEYRFKTRAM RLNSIEASSR AKINFLEALY RFHRQQGNPR VTVPTINYKP
LDLWLLRKEV QKLGGFEKVK EKKWSEVGRL LGYTGPGLST QLRNSYIRVI LPYEVYSDGI
RSSLCDGCDC GFHMFCLVPP LTSVPKGQWF CHTCLFGTGD YGFDEGEEHS LSSFQARDQA
FRKMWFETHP PISEPDVENE FWRLVQSPYE TVEIEYGADV HSTTHGAMPT LETHPLDPYS
KDPWNLNNIP ILQDSLLRYI KSEISGMTVP WTYVGMVFST FCWHNEDHYT HSINYMHWGE
TKTWYGIPAE DAEKFEAAIK KEAPDLFETQ PDLLFQLVTL MSPARLKESG VHVYACDQRA
GEFVITFPKA YHAGFNHGLN FNEAVNFALP DWLPLGRECV KRYQSHKKLP VFSQDELLIT
VTQQSHSIRT AIWLNDSFKE MTETEIKNRK AVRLGVPETL IEHDPEDQYQ CAYCKAFCYL
SQVMCPCVVC LEDVKYLCDC QLLRLRFSDE ELLNIQSTVS SRAAIPENWH KKLMKLLNDS
PKPQLRALRA LVAEADRINY PMKEVASLRR CVTRANEWVE AANSFITRKQ SRKRDRPEKT
LEELYSVLRD VENLGFDCPE IGLLRNLASQ AEEFKTKAKA LLCETLLAHG TSLNVYLEEF
YKIENYVLQD RLVKELLDEI REFLNRAKAC ELPSGNKYMI LLEERLKAGT DWDERAAGVL
NQPIKTIEEL PVDPSVLKRI QTTRSRALEY EKQAKEWLSP QLPTVQEALR LVQKAEKEFN
IQAIQDLKRT VDFAYDLEER CEAVLKNRYE HGSCFDAMNR WRTYAREHLT FRLPSFDKLN
VEIEKHEQWQ KKLPWYCHAD EILRDVVDYT KPEDDEPPHD EFFTCICFEP VRPPPPGVVS
DAVQCDHCYA RFHGRCAVNG GSCPFCDPNH WNGTIHSDRS YHFCYLPTVL HNAPEISKNY
SEHWQELKTI VEHIERLCNV VGNFLSIASY QRAEYIPQVR HYLRKLYKIK FAVSPNPEVS
FGLDLAGLHR ILANRRMKKR RRPKFVFGQD VDRDWVDGTR CICRGQTCLR KYHNACVFYC
PLCCLRKGKM YRWADIRVRT FSRNLIKIKL APPVTPTIFI DLVQFYP

>Phellinus noxius
MRRTQQDQCILLSGETCSGKSENRRLAIKSIIELSVSNPGKKGSKLSTQIPAAEFVLETFGNARTLFNPNASRF
GKYTELQFTRGRLSGVKTLDYYLERSRVAGAPSGERNFHIFYYLVAGATAEERQHLRLTDKASFRYLGPRDGVR
FDQLKVALKSVGLSKRSVAQTCQLLAAILHLGNLEFIIDRQRNEDAAVVKNTDVLETVAEFLGVQPQALENALS
CKMKLVKKELCTVFLDPDGASDNRDDLAKILYSLLFSWLNENINQKLCRDDFTTFIGLFDLPGPQNMSRSNSLD
QFCVNFANERMHNWIQRRMFEIHVDEYNQEGITRYVPSVPYFDNSECVRLLTNMPGGLIHIMDDQARRMPKKTD
HTMVEAFSKRWGNHSSFKVGGMDRFPTFTVNHFNGPVTYSSEGLLEKNLDAVNPDFVSLLRGGSVNPFIRGLFT
GKAIATQAHPRNEDTIVAAQQTVKPMRAPSTRRKGTPCTAGIFRTALETLFSTLDEAQAWFVFCINPNDSQLPL
QLEGRAVKGQIRSAGLTEIAKRCANVYEVNMTTSEFCDRYKAQIATLNVFEGESDIVLGNYKVFLSQRAFHKLE
DRLRAEDVEEQKRNRIRDAEASSQQLPLVANASPFDDMDAKSRLTSNRESYAPSRNMFQNDDGEVLEGETTEDI
KESSARRRWVAFVWLLTWWCPNIFLIWCGRMKRLDVRQAWREKLALNLIIWFICLAAAFVIAVLGNLICPTEHV
FSQSELASHDGNMYTSIRGEYAGTSSDDLFPIQVSAVCNGIDGSVDPLVTLNSKNTDTYTQYHDFRAWQNDSRP
DWYFESMTYMRWNYRVGFLGLTSKEVSSQAKNGKAVAIYNGLDLPAVMFTSTSSTFLTFKPRQPMHFTCLTIGS
RGDVQPYIALAKGLIADGHGIEFGYVGGDPAELMRICVENGMFTVSFLKEGIQKFRGWIDDLLKTSWEACQGTD
VLIESPSAMAGIHIAEALGIPYYRAFTMTWTRTRAYPHAFAVPDHKSYVMFDQVFWRATAGQINRWRRNTLNLP
STTLDKLEPHKVPFLYNFSPTVVPPPLDWPEWIRITGYWFLDDAEVSASKWTPPQDLADFIDNAHKAGKKVVYS
DPDVMTRCVIEAIMKAGVCAILSKGWSDRLEPEIPLPPEIYPVKSVPHDWLFAGIPTIIKPFFGDQFFWGDRVE
ALGVGSCVRKLTVDGLAEALIAATTDERQIAKAKLVGERIRSENGDKPREIFEPLRLACETGNEKLQIASLDCT
SKLISYSFFLELVDLVTHTITACHTETTPDTVSLQIVKALLALVLSPTLLVHQSSLLKAVRTVYNIFLLSPDPI
NQTVAQGGLTQMVHHVFDLFFKDAFLVFRALCKLTMKNLNTESERDLKSHAMRSKLVSLHLVLTILNSHMQVFV
DPSSIIYSSSSNEATSFINATKQYLCLSLSRNALSPVPQVFEISVEIFWRVLTGMRTKLKKEIEVFFHEIFIPI
LEMKTSTLKQKSVILGMLQRLCQEPQALVEIYLNYDCDREATDNIYEHLMNTISKISTPNLTTAALAVPGHNLG
LSEQQLKRQGLESLVAVLKSLVTWGTDDPGRFESAKQRKTILQEGIKRFNYKPKGIEFLLDNGFIPSREPVEIA
KFLLSTDGLSKATIGEGDEENIAIMHAFVDLLDFSNLPFVDALRLFLQSFRLPGESQKIDRYMLKFAERYIAGN
DTAYVLSYSTIMLNTDAHNPQVKNRMTKEEFIKNNRGINDLPDEFLLSIFDEIQTNEIRMKDEIASVGRDFQKE
AYVMQSLGMANDQFFSASHFVHVRPMFEVAWIPFLAGISGPLTDTDDLEVVELCLEGFKAFVTTLAKFTFLNNL
GEMKTKNMEAIKALLDIAVSDGNHLRGSWHEVLTCVSQLERMQLISSGRARKMPAEELANESRSTHITVATDMV
FSLSHYLSGTAIVEFVRALSAVSWEEIQSSGLSEHPRLFSLQKLVEISYYNMGRIRLEWSNLWEIIGEHFNQVC
CHHNPHVGFFALDALRQLAMRFLEKEELPHFKFQKDFLKPFEYTMIHNNNPDIRDMVLQCLQQMIQARVHNFVS
GWRTLFSVFSAASKVLTERVVSSAFEIVTRINKDHFSEIARNGAFADLTVCITDFCKQCLDDAMIKFWFPVLFS
FYDIIMNGEDLEVRRLALDSLFTTLKEYGSTFTVEFWDTVCQELLFPIFAVLKNSSDLSRWSTQEDMSVWLSTT
MIQALRDLIDLYTYYFETLERFLDGLLDLLCVENDTLARIGTACLQQLLEKNVKKLSAARWERVVTTFVKLFKT
TTPHQLFDENLRTDRRRIFKQIIVKCVLQLLLIETTNELLQNDEVYDTIPPEHLLRLMGVLDHSYQFARMFNED
KELRYALWKETSSADTLVTVLCRMYHDTRPQHLELRPQIADKFLPLGLGVVNDFNKLRMETSQKNIAAFMPVVS
KIIKGFCSLSDKAFGRFLPAIYPSTAELCARDPEVRSHLRDYFVRVGQFQGIQRQALVAAAQTKFGVEMMSPIL
SQLFQNLSLPPQTTVVQVLNQFGPEITGDVDVVRGLLTRFGMNDQTPPSDEQIVDIFSTLSRLTVENAQLCDVA
ALVRALSSFRVKHDWSKVIHVFDWPDRGVDTNTLKLLIAILVNSPRVEKPAVSGFWSQWNNSLYQLRLLDALLS
LPSDTFNFVSLPGRRVVTVEDVAAASPTIKTLAANVQSHTWNSLDLFEVLVRLGDSENENVRNCVREMLDKAVR
ISADIVHMGLLQAPPWNALQVEYSRQLLGMFLSGHPNHQLVFMRIWQIEPSYLTSALREFYEENPMNITRILDI
LDSLLEVRPFVFALDEYLNLDKWLEDNIIKHGAEFLHGIIVFLETKMQNEKIARTIDPQARTMALSPTTIAIFL
RALRNFSSVMDEADMDFCIETRNACLQIHPRLMNQEPGFSVVNYPPNIEAEVDGIFKQMYDEQITIDQVIVMLQ
RTKESTNPRDHEIFSCMLHFLFDEYKFFQYPPRELAMTGYLFGSIIQQQLVDYIPLGIAIRYVIDSLQCPPETN
LFKFGVQALSRFESRLPEWKVLCEALLSIPHLAEQRPDIIEVVRRAETSDKILFIINNLAPSNFDAKLAEMKSS
FSSEPNNHQLYLRFLDGLDCKKLAKFILHETYIKSTAMLNSDKTKASASERTLLKNLASWLDKPIKHKNIAFKE
LLIEGAESDRLLVAIPFVCKVLEGASKSKAFRPPNPWLMAVISVLAELYHFAELKLQIKFEIEFLCKALGIDLE
TVEVSNIFRSRPRNVGAHIEELLAQLASAVTINSQLGLSNNQLFKQAIQDAIDRSVREIILPVVERSVTIAGIT
SRELCVKDFASEASEEKLRKAGHLTCQKLASSLALITCKEPLRTNIPGHIRTYLAEHGFTEQQVIMLITQDNLD
IACETIEKAAMDRATREVDTSLALSYDARRRHREMRSNIPFIANLPDSLRIKPMGLQPHQLRVYEDFFNVLIQK
LEAILPDVTPSFSSVPLSHEIRNILHRLVSLSDRVRTPLLMSQKIVQHLYKTPLQLGREVYVTALEQLCRTYDD
VAKEAINWLICAEDDRKFNIPVTVTLIRSRLITVTDEDLQLAKNIRPNLQDFASGLIRECLSTEATQISFQYTL
DCFQQWVSVFQRSSAPEKMFVPYVTQLSKQGILKAEDSSFFFRVCTESSIAHYSKCMASGEYDHSFLALDAMSR
LIVYIIKYHGDANNLQAKVHYFSKILSIVVLVIANRHEEQGFQQKPFFRFFSSLLSDLHSLETQLGSVYFALLL
ALSDTLSSLQPTYFPGFSFSWMTLISHRLYMPKLLSSENREGWSAFHKLLTALFRFLAPFLSSANFTPASRSLY
RGTLRLLLVLLHDFPDFLSEYYFSLCDIIPHRCIQLSNIILSAFPASVNLPDPHLRNINMGPIPPIVPDFAILQ

KSGDLKAHLDQCLANRVSQVSLSTLKERLENYNISLMNTMVMYIGVTTVAQAKARSGNPIFNPSDPAVVVLSYL
AYNLDPEGQHHLVSAMVLHLRYPNAHTHWFSSLVLHLFEQANDETFREILTRVLLERFIVHRPHPWGALLTFIE
LLRNPKYDFWNKDFVRAVPEVTLMLDNVGHRRLYLQLRFRNVSDLLAVRRDIVPLAQANGAKLNAVDAFAEVVD
PRDAIIDVREYDVPYYLRVAIDNEIRVGLWYSVTFTAGQPQMDRVKRADPVVLAYDIETTKAPLKFPDSATDQV
MMISYMIDGQGFLITNREIVSEDIEDFEYTPKEGLDGPFTVFNEADEAATIMRFFRHIQEVRPTVLATFNGDFF
DFPFLCARAKTHGIDMFLEIGFAKDSEDEFKSRTCVHMDCFRWVKRDSYLPQGSQGLKAVTTAKLGYDPIELDP
ELMTPYAIEQPQTLAQYSVSDAVATYYLYMKYVHPFIFSLCNIIPLCPDEVLRKGTGTLCETLLMVEAYRGHII
MPNRHEDPHGNMFEGHLLSSETYVGGHVEALEAGVFRSDIATHFKIVPAAAQLIDELDAALSFCVTNYNEVKSQ
IQSALELMRDNPLRFDKPLIYHLDVAAMYPNIMLSNRLQPDSVVDESVCAVCDYNRPGKTCDRRMTWAWRGEYF
PAQRDEYNMEGFPPKRTGDPQRRFTDLSPSEQSALLHKRLGDYSRKVYKKTKETRVVQRETIICQRENPFYVDT
VRRFRDRRYEYKGLHKTWKKKLDAVAEVEEAKKMILAHKCILNSFYGYVMRKGARWHSMEMAGITCLTGATIIQ
LARQLVEQIGRPLELDTDGIWCMLPGVFPENFKFELKNGKTIGFSYPCTMLNHLVHAKFTNHQYHDLDPETGEY
KVHSENSIFFELDGPYKAMILPSSKEEDKLLKKRYAVFNDDGSLAELKGFEVKRRGELQLIKIFQSQIFEKFLL
GSTTQECYSAVAEIADRWLDVLFSKAECLSDEELVELIAENRSMSKTLAEYAGQKSTSISTARRLAEFLGDQMV
KDKGLACKFIISAKPMGAPVTERAVPIAIFSAEESVKRTYLRKWLKDNSLANFDLRSILDWEYYIERLGSVIQK
LITIPAAMQKVPNPVPRIRHPDWLHRRVIALDDKFKQHKVTDFFRGRFTMWLCVNAELIPVTLRIPREFYLNFK
TPEYYTRERVIRTLPRDRQCLHLFTNEINHPNVDGAYELQTLNRLRDDGINLVQLERKYLFLYHAYAPTSPVHV
YALFLPNGLKLHIVDPAPRRQPTDYHSNEIAALKALSRELGTLESKSYTIVLSSAKDFIYFSGMVPKLSKFPVL
RMPATKASHALDFPWQSVVAKKMFVRYLAFGSWLHRAISQAAYYPLFICDIEMARRLVAQDIVLWWSANEKPDL
GGSEDDSPPTEELNNPEFLAPGLYSNVCLSVQVRNLAIDAVLQSALVNELEGSSGATAFDLAQPNVTLGDSNLS
PHTFAVLKQMVRTWLLDKACAASITLDHFWRWVSSSSSRMYEPSIQRFVHGLMRKTFIQLLAEFKRLGSNVVYA
DFSRIILVTSKPPGTAHAYATYINSAVTSNELFKHIYLRTDRFYDFLLYMDPANNGAVVCEDPLALVPPKQLSI
SSNWNIKKFLPPAVQDYFKNVVRYFVVQMAKIKREKVKEMDQTRAFVAQKLTRKMLHYVFPVLPGSYLHMTDPA
LEFIKFSCAVFGLAQEYQIELGILKRNLLELVGVKEFSDLAIFRNPCDPLKLSMVTCRFCDHIRDFDFCRDEEL
LLWYCPECDGEYDRTAIEFSLIQVLYRLERNFTQQDLKCSRCKQIQSDNMSKHCCSGNYQLMVSKAEMKRKLRT
IINVAITHGLSRLFEVEHLKYATLATSPNLSSQKQIASIKTARNVIEYNEQYVTKRLLVNIIWAFSGDARGEWI
AWSSRVPIIEILYSWLSEHKPLMLCGPPGSGKTMTLFSALRKLPDMEVVGLNFSSYRKTPNGVILAPVQIGRWL
VVFCDEINLPATDKYGTQRVISLVELGGYYRTSDMAWVRLERIQFVGACGRVPLSHRFLRLVMVDYPGEISLTQ
IYGTYNRAMLKVVPNLRAYAEPLTDSMVSFYLASQKRFTSDIQAHYIYSPRELTRWVRGIYEAIKPLESLSVEG
LVRVWAHEGLRLFQDRLVTEDEKNWTDENINSTAMTHFPTLNILFSNWTSKNKARLKVFHEEELDVQLVLFNDV
LDHVLRIDRVFRQTTLSRFVAWMNGLSIFQIKVSNKYTGEDFDLLANAEVPGLFEGIYHESPENGLASRAATSP
ALFNRCVLDWFGDWRPSYKPPEFFPTAYRVVNALVYIHQSLYQINKKLSRRQGRYPRHYLDFIQQYVRLYNEKR
DELERHLHVGLDKLVTQVEELRKSLAIKRKLQRMVSDQQEAEQKKAASIEIQAALVEQDKHIEQRRNIVMADLA
DAEQMTKQLRDLMKREFLSRPTFKFETVNHASKACMIAELESSIHKYKEEYALLIRVENKVERSMRMLESLSQT
FDEEMSTIVGDVLLSAAFLAYGEWSTHLGDASIKYKAELSFPRLSWQSKSLPSDSLCIVTSFLDDAFLKVLESA
LRFGNPLLIQDVEHLDPILNEIRRTGGRVLIRLGNQDIDFSPSFPSVEFSPDICSRVTSQTLDQVLKVETDLMK
IQGEFRLRLRTLEKLLLQALNESTGNILDDDKVINTLETLKREAAETDVVIREVEQVTAEYLPIAQACSSVFFI
LEQLNLINHFYQFSFLDIFDYVLHHNPNLKNVLFDDLFLLVFKRTSRALEEYEFLLESGLESHAKHELFKPVRE
HMKNNGEEWITFLLLTKCFRPDRLAQSVTGYDASYRVENQEGFALADQAIALAARQGTWVLLKNVHLAPSWLGE
KKLQTLNPNRNFRLFLTMEANPVIPVNILQSRILMNEPPPGIFLLAWFHAVVQEDMSAAFGTIDAWINSVAKGR
ANIDPVTIPWDAVRTLVKQSVYGGRIDSDFDQKLLDTFVDSLFTPSAYNLNFPEGTKIDHFLSWPSWLSLPPTA
ERRKMRSLADDDLERCKEWLGALPESFSTLARQSSDSQDPLSRLFQREEEVGKLLHQVRRDLADVLLSHLTKGT
IPGHWMRYKVKKIPNLSLRLSQLDRIAKLDDGGLFFPEAYITATRAVAHRKRWSLETLDLRLDIERVNDPGLVL
EGAAWSVDVLPETEFVLPLLQGENIDEHFHCIGIDAAEPYLSVAKKFAEDMLVFDVETLPNYHPYAVMACAASD
KYWYSWISPWLLGKLIVGHNVSYDRARVLEEYNVEDSSIRYLDTMSLHVAVKGISSHQRPAWLKYRKNKKRWED
VTSANSLADVAKLYCNIRIEKATRNDFMTLSREEILGNIQKYLNYCSSDVEATHAVFTKVFPDFLLTCPSPVSF
AGVLRMGSSFLTVDENWEEYLKSAEGIYLGLKEKIKKRLEALADEAWKDDMWLSQLDWTPKWPKWYWDLTKPKK
GTLDVTIRSRVAPLLLQLSWKGWPLFHSREHGWTFRVRQNKLEFHDAADAILFYKLPHKDGDKANVGNPLGKTF
IKYAQDGTLTSPGEDTKAALEMNAQCSYWISSRDRILNQMVVWKKVGMIIPQVITMGAVTRRAIERTWLTASNA
KKDRVGSELKAMVRAPSGYKIVGADVDSEELWIASIMGDAQFGLHGATALGWMTLEGTKSAGTDLHSKTASILG
INRNQAKVFNYSRIYGAGMKHAILLLLQSNPSMPQETAQGQAEKLYRSTKGQKTHRNFFERKFWFGGTESYVFN
KLEEIAMSDKPLTPALGCGVTYALSKEYLQTGFGNDYMTSRINWVVQSSGVDYLHLLIVSMEYLCKKYEIRARY
LISVHDELRYLVKDEDRYRAALALQIANLWTRCLFAYKLGMEDLPQGVAFFSAVDIDHVLRKEVDLPCVTPSQP
SEIPPGESLNIQQVLEKTDEGSSLLRSYTKPDCLKHRTSSTYFLKAQAMSKRRDVEGLESALIQEILATPVEGL

AKLLRSIETWKWPRTDLNAFLKVLNKFDAILEEVIQEYEVDTLQVKGYAPDKKELLLEILRFERLLLEHSMNRK
VFNSYDRLNSLMFTSDLDVLIATLLLLLRPSQQYSSQPALSHSLHISTSRLESLAKSPPTLREHGVEMLDLVSK
GEKSIEKLPMEASEVHFQFYRHLGPLSQSSRSAMDIFADTVKSHQVPDEKFELLCRIRFARALGTGRKKLVIAR
LLAIAIYVHTHAENQAQSSLFLYDTDLVSRIGELLQQDREIRENFGDISNSSSTVPNLFVDALISFVTFISAHQ
TGGNVLVSAGLIPLLIQAISIENVERLSVIPKILQLLDNVLYGYSNAFTLFCNSRGVEALVTRIKNEVDGLIQI
TRSSLLKQLLKSLHRMMQSSGTSEGMRGLIDTSVLKSVQTIIEYRGIFGPPVLPIAINIVATFVHNEPTSLTAI
QEAKLPETIFKAFETGIEPSFEVIQSIPNVLGALCLNQAGQEQLAAHPSIIPALFSILTSETHLKVLLEKENAA
NMGSSIDELVRHHPALKNIVFNSIISTLKKIEELGKDYVPSDDVQQFYRLDNIIINYIDAVGRFLEGLFQHTEG
LDCIARLLALPCLPYDYANHIVVQVIRIIVEVCPVDALVHLIKQVKESLRETQEFWETPREESNDFFHRIITLH
VRVSLLADAYSTYIQGRQTVGPLLGSLHRSCMWENIVLKNAFNAHALRHIVSQIPLGSFFQSVAKRRAPDEQQK
QQAEIMSATLADVLLKHLYHTVMISLVTVLMFDELLVQFKKVGGQELVHVYGGLRMVLNLLILLISHTPNTDMG
QIAQYICRKPETHPDFYQQNDFLVRMRLAVAPVIKELWEAEWLLNAPPAVSKNVIKCIQAIISGDNEPRESAIR
ALARTQNNVSYATDFLLNNPELDIIRQSLVSNIGPLALRLADVQPVIQKFSWAAYDVQEEPLAVRCRLLALILQ
PLPKWLPALLLAMESLLVTSEEPRPIPLVLVTGPRYAEARSTLFELCIRLLLIPSLPRDELLATLRMLVQLTRD
HTMAAQFVHRDGVSLLGFQSHIAIILRHLVEDPKVLSSVMTQEVKKLFNQFKTTEVLTYVRNSMAVAARDYQMF
LDVTKELCSLVRPESVVHFMIGELIIVGKYACFLMQCLSELLFSYEQCKIAFLAYPKKRPKSVALSFLLKELIT
FGAFHARKRIILCNWAMSVLVALCVDNSHTHQHEIGSIRKLVLESISKAIKDAPSHESIDARYGRTLALVDLCY
RLLTVRFEAPMHLAKIMLEKNFVATLTGVLNDIDLNYPNMRSLVNTILRPLEYLTKVAIKIGREETPDLYRNSS
LGMIPDVTSHPLLVDRGDGREFGPLQTVTRWSEEAKITHGKHLQDRTQKLCNHIILALLPVARERVTVMIHGNS
VDITDTGIDPTFLEALPDDMREEVLNQHFREQRSARAEVTVESQISTEFLDALPPEIRAEILREERLEPTEMEA
ADFIASLDPQLRQVVLLDSDDGSHRFPRKTPQARDAIQLLDKSGIAALVKLLFFPQKSVLNKVLLNLCENSKSR
TELFNLLLSILQDGTGDLAVVDKSFSQLSVKPDLVAQRCLDALSYIVETNELSSLFFLTEHELPVGLKKSKKGK
GKDRQPQSHYPIVLLLGLLDRHALIKTPSIMDAVAQLLDAVTRPLTSLKKILFSNPPQIPHPALRSIVNILTVG
ECSGRTFQHTLALIQHLSFLPDARDVIAQELRTRAHEFGQSLSLDLDSLISALVAKFSPASSDQAKLLRVLKTI
DYMYSIYETFRFTSLWKKLSECLTVVESKSNVEHIATILLPLIESLMVVCKNVGVKQSVDDLFVEFTDEHRKIL
NLMVRNNPSLMSGSFSLLVQNPRVLDFDNKRNYFNQQLRKRRESYPSLHVPVRRARVFEDSFQVFQNKTGEQIK
YGKLSVRFHHEEGVDAGGVTREWFQILARQMFNPDYALFEPCAADKQTYQPNRASDVNPEHLSYFKFVGRVIGK
AIYDGRLMDAHFARSLYRQLLGKRVDYRDVEWVDPEYYKSLCWILENDPTVLDLTFIFGKHEVIPLKENGTTLP
VTMENRKEYVQLSAQYRLHTSIAKQIDSLLSGFYEIIPKDLISIFNEQEVELLISGTPDIDVDEWRAATEYNGL
TSSDPVIVWWWRALKSFTRDERAKVLSFATGTSRVPLGGFTELQGVQGVQRFSIHRAYGEPDRLPQAHTCFNQI
DLPEYSSYEKLRQQLLLAINEGGEGFGFAKSSASSATRKKHARKLKRLGKKDSVTKRKALESLQHLPSLLLNAS
RRIRLLTAVLQASFLGSWCMATEDIDRQVAIQAKRAWFIRRALFDPASIYAELNPEESESDRKGRIRTNALGVL
RWGDGQPVVRRTAWDVLKIFLSSAILRSAWVEPDAIVRASMWEPLLTFLKQFPKSWAYREFLQFLALGSPTQGY
PAIVVIISTIPPSIFTSFWAAFDGQALAFLRALLESLVFLIKRPNLFVFGFLLPPPKDTIESMLQSFPSNPLDP
TLAIVDPLDCGGLSKYSRLIYALLNRDLMRENIWVLRHFLVLDKIGDSRVLYEVMRHILKGATRTDAEEWVTLS
RHIEKRAPNTFSAIMLAVNESGLEPPKLDRYRNEFAAGMMGVGGLWLLHKLALIAPDPESETVFLPQNRAVNFM
KTCQNWISSDISEDVESEMTLIFQYLSPILQNVPGAHWEFIFDVIENNLENSSFEGDSSLVVLSRTIKLIIIIQ
DLSSTNKTLRSWKEREISILTLLRDLSTPRFNCWELAFSVEYLVVEAGVDTTSLRVKMGYAAQLQGQDLIGSSF
LPSIFDLLEVHRPFQLEPWLVEEYYVQYYDPQSPKLFSAHLLYRALLNIPSLISSWWGNIKDRQLLGAISSFIT
KHYSPVLISEELRIIRDPVEEKRWRGWLLAVQQTQNGRIVDGLSMFKKNVMHHFENQTECAICYSIIKRCKTCR
NRFHASCLYDLPPNTLDMS NLKKMKTRPF GLEECPTFFP TTSEFKDPLT YIRSISAKGQ EHGIIKIVPP
EGWEMPFVID SEYRFKTRAM RLNSIEAASR AKINFLEALY RFHRQEGNPR VTVPTINHRP
VDLWLLRKEV QKLGGFEAVK GKKWSELGRL LGYSGPGLST QLRNSYIRVI LPYEHYSKNV
RGSLCDGCDC GFHIFCLTPP LSSIPKGQWF CHTCLTGTGD YGFDEGDEHS LASFQARDLA
FRKMWFEKHP PVTEMDVENE FWRLVQSSSE TVEIEYGADV HSTTHGGMPT LETHPLDPYS
KDPWNLNNIP ILQDSLLRYI KSEISGMTVP WTYVGMIFST FCWHNEDHYT HSINYMHWGE
TKTWYGIPGE DAEKFEAAIK KEAPDLFETQ PDLLFQLVTL MSPAKLKESG VRVYACNQRA
GEFVITFPKA YHAGFNHGFN FNEAVNFALP DWLPLGRECV KRYREHKKLP VFSHDELLMT
ISQQSQAIKT AIWLNESFKE MTDDELKGRQ DLRLGVREVL DQQDPEDTSQ CIFCKCFCYL
SEVVCSCVSC WDHSKYLCNC KELRLRYSDE EISNTQTAIA SRAMVPVNWR NKFNKLLADS
PKPQLRALRA LMAEGERINF PLEELSVLKA CVNRANKWVT DANSFTTRNN SRKRDRQEKS
LSDLYKVLDE VDTLGFDCNE VEALRSLTVQ AEDLRVKAKA LLCEALLAHG GSTNVYLEEL
VQIDNLITQH KLTAEILQEI RQFLSRAKAC DLPSDNKFTV LLENQLKNGT DLDGKMAAIL
SREKKTIAEL PFDPAILAQI RAFKERAERL EKQANEWLAP ELPNVEDVLK VVEAAEKEFD

LPSISNLKRT AEFASDLEKR CSAVLSKRYM HEPCFDAMKK WRTYAREHLT FRLPNFDKLC
VEIDREDDWA RGLPWFSEEK KIFEDVIRYT LPMDDQPPKD EFFTCICFSP VRPPPPDQVS
DAVQCDHCFA RFHGKCASNG GSCPFCDPNH WNGTLNKTRN FHFCYLPTIL KNAPEISRKY
SVHYDRLKFI VENVERLCSV IGTYLSHLSN QRPHELPQIR HYLRKLFVLK FAVSPNPEVS
FGLDLSGLHR ILGGKKLKKR RRPRFVFGQD IDQDWVDGTR CICRGQTCQR KYHAACVFTC
PLCCLRKLKS YRWADVRVRT YSRDIIRIKL PAPTQATIFV DLVNFTP
>Onnia scaura
MRRTQQDQCMLLSGETCSGKSESRRLAIKSIIELSVSNPGKKGSKLSTQIPSAEFVLESFGNARTLFNPNASRF
GKYTELQFTRGRLSGIKTLDYYLERSRVAGAPSGERNFHIFYYLVAGATAEERQHLKLTDKSTFRYLGPRDAVR
FNQLKVALKSIGLSKRVVAQTCQLLAAILHLGNLEFIVDRQRNEDAAVVRNIDILETVAEFLGVQPQALENALS
CKMKLVKKELCTVFLDPDGASDNRDDLAKILYSLLFAWLNESINEKLCRDDFSTFIGLFDLPGPQNMSRSNSLD
QFCVNFANERLHHWVQKRMFDSHVDEYNQEGLSRFVPSVPYFDNAECVRLLSNMPGGLMHIMDDQARRMPKKTE
HTMVEAFGKRWGNHSSFKVGGIDRFPTFTVNHYNGPVTYSSEDILEKNLDALNPDFVSLLRGGSVNPFIRSLFS
GKAIATQAHPRNEDTIVAAQQPVKPMRAPSTRRKGTPCVAGEFRAALDTLFSTLDESQAWFVFCINPNDAQMPN
QLEGRAAKGQIRSAGLTEVARRNVNVFEANMAPSEFCNRYHAQLSALNIHEGERDIVLGSTKVFLSQRAFHKLE
DRLRAEDVEEQKRNRLRDAEASNQQLPLVANASPFDDMDGKSRLTSNRESYAPSRNMFQNGDGEILDGETTEDI
KESSTRRRWVAFVWLLTWWCPNIFLIWCGRMKRLDVRQAWREKLAINIIIWFICLCAAFVIAVLGNLICPTEHV
FSTSELASHNNSAYTSVRGEYAGTTADDIFPVQVSALCNGVSGSVNPYVSLNSKNTDPNSVYHDFRAWKNDSRP
DWYFESMTLMRWNYRVGFLGLTGKEVKNKANDGNSIAIYDGLSLPAVMFTSTSSTFLTFKPRQPLHFTFLTIGT
RGDVQPYIALAKGLLADGHGIEFGYVGGDPAELMRICVENGMFTVSFLKEGIQKFRGWIDDLLKTSWEACQGTD
VLVESPSAMAGIHIAEALRIPYYRAFTMTWTRTRAYPHAFAVPEHKSYVMFDQVFWRATAGQINRWRRNTLKLS
GTNLDKLEPHKVPFLYNFSPTVVPPPLDWPEWIRVTGYWFLDDADVSAMTWSPPKDLVDFIEAAHQAGTKVVYS
DPDVMTRCVVEAIVRSGVHAILSKGWSDRLEPEIPLPPQIYSIKSIPHDWLFAGIPTIIKPFFGDQFFWGDRIE
ALGIGTCVRKLTVESLANALTIATTDEKQIAKAKLVGDKIRSENGDKPREIFEPLRLACETGNEKLQIASLDCI
SKLISYSFFLELVDIVTHTITACHTEMTPDAVSLQIVKALLSLVLSPVILVHQSSLLKAVRTMYNIFLLSPDPV
NQTVAQGGLTQMVNHVFDLFFKDAFLVFRALCKLTMKNLNTESERDLKSHAMRSKLVSLHLVLTLLNSHMQVFV
DPSSIIYSASSNEATSFINATKQYLCLSLSRNALSPVPQVFEISVEIFWRVLSGTRTKLKKEIEVFFHEIFIPI
LEMKTSTLKQKSVILGMLQRLCQEPQALVEIFLNYDCDREAADNIYEHLMNTISKIASPALSTTALAVPGQNLG
LSEQQLKRQGLESLVAVLRSLLAWGTDDPGRFESAKQRKTILQDGIKRFNYRPKGIEFLLENGFIASREPAEIA
KFLLNTDGLSKATIGEGDDKNIAIMHAFVDQLDFSNLPFVNALRLFLQSFRLPGESQKIDRYMLKFAERYIAGN
DTAYVLSYSTIMLNTDAHNPQVKNRMTRDDFIKNNRGINDLPEELLSAIYDEIVSNEIRMKDEVANVGRDLQKE
AYVLQSLGMANDQFFSASHFVHVRPMFEVAWIPFLAGISGPLTDTNDLEVVELCLEGFKAFVTTLAKFTFLNNL
GEMKAKNMEAIKALLDIAITEGNHLRGSWHEVLTCVSQLERMQLISSGKPRKMPTEELANESRSTHITVATDMV
FSLSHYLSGTAIVEFVRALSAVSWEEIQSSGLSEHPRLFSLQKLVEISYYNMNRIRLEWSNLWEIIGEHFNQVC
CHHNPHVGIFALDALRQLAMRFLEKEELPHFKFQKDFLKPFEYTMTHNNNPDIRDMVLQCLQQMIQARVHNFVS
GWRTLFSVFSAASKVLTERIVNSAFEIVTRLNRDHFPEVIRHGAFADLTICITDFCKVSKDDAMIKYWFPVLFS
FYDIIMNGEDLEVRRLALDSLFTTLKSYGSTFTVDFWDTVCQELLFPIFAVLKSSSDLSRWTTQEDMSVWLSTT
MIQALRDLIDLYTFYFETLERFLDGLLDLLCVENDTLARIGTSCLQQLLEKNVKKLSAARWERVVTTFVKLFKT
TTPHQLFDENLRTERKRIFKQIIVKCVLQLLLIETTNELLQNDEVYDTIPPEHLLRLMGVLDHSYQFARMFNED
KELRTALWKETSSADTLVTVLRRMYYDPRPHHQALRPKIADKFLPLSLGVITDFNKLRMESSAKNISAFMPVVA
RIIEGFCHLDDKAFGRYLPAIYPLTAELLARDSEVRSHLRDYFIRVGQFQGIQRQALVAASQTKFGAEMMSPIL
QQLFQSLSLPPQTTLVQVLNQFGPDITSDADVVRGLLSRFGMTDQCPPTDEQIVEIFSMLSRLAADGIALCDVG
ALVRALSSFRVKLDWSKVIEAFDWPDRGVDTATLKLLIAILVNSPRADKPAVAGFWTNWNNSLYQLRLLDALLS
LPSDTFNFVSLPGRRVVTVDDVSAASPTIKTLAANVQSHTWNSLDLFEVLVRLGNSEDGDVRIFVRDMLDKAVR
ISADIVHMGLLQVPPWNALQVEYTRQLLGMFLGGHPNHQLVFMRIWQIEPKYLATALREFYEENPMNITRILDI
LDSLLEVRPFIFALDEYLNLDKWLQDNVDAHGAEFLHGVISFLELKMQNEKIARTIDPQARTMALSPSTIAIFL
RCLRNYSSVMDERDVDYCIETRNACLQIHPRLMDQDTGFAVVTYPADIESEVDGIFKQMYDEQITIDQVIAMLQ
RTKESSNPRDHQIFSCMLHFLFDEYKFFQYPPRELAMTGYLFGSLIQQHLVDYIPLGIAIRYVLDSLQCPPDTN
LFKFGVQALTRFESRLPEWKLLCEALLNIPHLAEQRPEIIEMVRRAETSDKILFIINNLAPSNFDAKLAEMKER
FSTEPNNHQLYLRFLDGLDNKTLAKFILHETFFKSATMLNSEKTKSSSSERTVLKNLGSWLDKPIKHKNLAFKE
FLVEGADSDRLIVAIPFVCKILEAAAKSKAFRPPNPWLMAVISLLAELYHFAELKLNMKFEIEVLCKTLGVDLD
NVEVANILRTRPRNVGTHIEEILANLANSVTINHQLALHTNQSFKQAVQEAVDRSVREIILPVVERSVTIASIT
TRELCIKDFASDPNEEKMRKAGHMACQKLAGSLALVTCKDPLRTNMPAHVRSYLADHGFTEQQVILLIVQDNVD
VACEAIEKAAMDRAIREVDNALAPSYEARRRHREVRPGTAFIAGLPDPLRIKPNGLQPYQLRVYEDFFGLWVAK

LEAILAEVPTSLTLVPPNHEIRTCVHRILALCDPVRTPLITSQKVVQYLFKTPSQLGREIYVTLLDQLCRSFDD
VAKEAINWLISAEDERKFNIPVTVTLLRGHLINVTDEDLQLAKNARSNLQDFSAGLIRECLTSDATQQQYQYTL
DCFQQWVGIFQRSSAPEKMFVPYVTQLSKQGILKVEDSSFFFRVCAESSITHYTKSVTAGDFEHAFLALDAMSR
LIVYIIKYHGDANNLQAKVHYFTKILSIVVLVVANRHEEQGFQQKPFFRFFSSLLSDLHALETQLGSVYFHLLI
ALSDTFSSLQPVYFPGFAFSWMTLISHRLFMPKLLLSENREGWSAFHKLLLSLFKFLAPFLRTASLTVASRNLY
RGSLRLLLVLLHDFPDFLSEYYFTLCDIIPSRCIQLRNIILSAFPASLNLPDPHMRNVKMGPIPPILSDFTLTL
KNGDLRGYLDQCLLNRVTQTSLAALKDRLETYDLSLMNAVVMYIGVSSVAQAKARSGSSLFVPSDPGVVALTYL
AFNLDPEGQHHLIGSMILHLRYPNAHTNWFSSLLLYLFAESKDDHFKEILTRVLLERFIVHRPHPWGAIITFIE
LLRNQKYDFWGKDFIRVAPEVTLMLENVGHRRLYLQLCFRNVSDLLSVRREIVPLAVENGAKLNAVDAYAEVVD
PREAIIDAREFDVPYYLRVAIDNEIRVGLWYTVSFVAGQPQFDRVKRADPVVMAYDIETTKAPLKFPDQAIDQV
MMISYMIDGQGFLITNREIVSEDIDDFEYTPKEGLEGPFTIFNESDEAATIMRFFQHIQEVKPTVIATFNGDFF
DFPFLCARAKVHGIDMFLETGFAKDSEDEFKSRTCVHMDCFRWVKRDSYLPQGSQGLKAVTTAKLGYDPIELDP
ELMTPYAMEQPQVLAQYSVSDAVATYYLYMKYVHPFIFSLCNIIPLNPDEVLRKGTGTLCETLLMVEAFRGHII
MPNRHEDPHGNMFEGHLLSSETYVGGHVEALEAGVFRSDIATHFKIVPEAAQLIKELDAALNFCVVNYEQVKNQ
IQSALELMRDNPMRFDKPLIYHLDVAAMYPNIMLSNRLQPDSVVDESVCAVCDYNRPGKKCDRRMTWAWRGEYF
PAQRDEYNMEWFPPKRPDGPQRRFMDLSQAEQTALLHKRIGDYSRKVYKKTRETKVVNRETIICQRENPFYVDT
VRRFRDRRYEYKGLHKMWKKKLDVVTEVEEAKKMILAHKCILNSFYGYVMRKGARWHSMEMAGITCLTGATIIQ
MARQLVEQIGRPLELDTDGIWCMLPGIFPENFKFQLANGKSVGFSYPCTMLNHLVHAQFTNNQYHDLNPETGDY
KIHSENSIFFELDGPYKAMILPSSKEEDKLLKKRYAVFNDDGSLAELKGFEVKRRGELQLIKIFQSQIFEKFLL
GTTTQECYAAVAEVADRWLDVLFSKADSLSDEELVELIAEKRSMSKTLAEYAGQKSTSISTARRLAEFLGDQMV
KDKGLACKFIISAKPIGAPVTERAVPVAIFSAEESIKRTYLRKWLKDNSLANFELRSILDWEYYIERLGSVIQK
LITIPAAMQKVANPVPRIRHPDWLHRRVVALDDKFKQHKVTDFFRGRFTMWLCVNSDLIPVTLRIPREFYLNFK
VPEHYTRDRVIRTLPRDRQCLYLFTNEINNPNVDGAYELQTLNRARDDGVDLWQIDRNFIFLYHAFTPSAHVHV
YALFLPSGVKLHIVDPATRRQPSDYHSNEATALKAISRELGMLENKSFTILLSSAKDFVYFSSMVPKINKFPVL
RMPSTKVSHALDFPWQDVVAKKMLLRYFSLAPWLRRTITQAAYYSLFFCDIELARRLVAQDMVLWWSAGERPDL
GGFEDDMPPTEELVNPEFLTTGLYSNVCLSVQVRNLAVDAVLQSALVNELEGSGGTTAFDSAQPNVTLGDSSIS
PQTFTTLKQMVRTWLLDKASSSSLTLDHFWRWVSSSAAQMYEPSIQRFVHGLMRKTFIQLLAEFKRLGSNVVYA
DFSRIVLVTSKPPGTAHAYATYIITAVTSHELFKHVYLRTDQFYDFLIYMDPANHGAVVCEDPLALEPPKQLAV
LSNWNIKKFLPPAVQDHFQNMMRYFVTHMVKIRRQKLQEMDESRTFIAQKLTRKMLHVVFPVLPGSYLHMSEPA
LEFIKFTCAVFELAQDYQIEIGLLRRNLLELVGVREFSDQAIFRNPCDPLKLSMVTCRYCDYIRDFDFCRDNEL
LPWYCPECQGEYDKTAIEFALIQDLHRLERNFAQQDIRCSRCKQIQSDNVSRHCCSGRYQLVIGKADVRRKLRT
MINVAITHNLSRLFEVEHLKYATLATAPNLLTQKEIASIKTVRNVIEYNEQYVTKRLLVNIIWAFSGDSRGEWV
DWSSRVPVIEILYSWLSEHKPLMLCGPPGSGKTMTLFSALRKLPDMEVVGLNFSSYRKTPNGVILAPVQIGRWL
VVFCDEINLPATDKYGTQRVISLVELGGYYRTSDMAWVRLERIQFVGACGRVPLSHRFLRLVMVDYPGEISLKQ
IYGTYNRAMLKVVPNLRAYAEPLTDSMVAFYLASQKRFTSDVQAHYIYSPRELTRWVRGVYEAIKPLEMLSVEG
LVRVWAHEALRLFQDRLVNEDEKVWTDDNIDTTAMVHFPTINILFSNWTSKNKARLKVFHEEELDVQLVLFNDV
LDHVLRIDRVFRQTTLSRFVAWMNGLSIFQIKVSNKYTGEDFDLLANAEVPGLFEGFTMNPPENGLASRAATSP
ALFNRCVLDWFGDWVSSYKPPQYFPVAYRVVNGVVYVHQSLYQINKKLSRRQGRYPRHYLDFIQQYVRLYNEKR
DELERHLHVGLDKLVTQVEELRKSLAIKRKLQRMVSDQQEAEQKKAASIKIQAALVEQDRHIEQRRNVVMADLA
DAEQMTKQLRDLMRRDFLSRPSFTFEMVNRASKACMIAELEASIRTYKEEYAFLIRVENKVDRSMRMLESLSQT
FDDEMSTIVGDVLLSAAFLAYGEWSSHLSEANIKFKSEISFPRLSWQSKSLPSDSLCIVTSFLDDAFLKVLESA
LRFGNPLLIQDVEHLDPILNEIRRTGGRVLIRLGSQDIDFSPSFPSVEFSPDICSRVTSQTLDQVLKVETDLMK
VQGEFRLRLRTLEKLLLQALNDSTGNILDDDNVINTLETLKKEAAETDVVMREVEQVTAEYLPIAQACSSVFFI
LEQLNLVNHFYQFSFLNIFDYILHHNPNLQNVLFDDLFIVVFKRTSRALDEYEFLLESGLNSYAKHAIFKPVQL
HVRDNEDDWVPFLLLIKCFRPDRLVQSVTGYDASYRVENQEGFSLADQAIALAARQGTWVLLKNVHLAPSWLGE
KKLQTLNPNLNFRLFLTMEANPVIPVNILQSRIIMNEPPPGVFLLAWFHAVVQEDMNAAFGTIDIWLNSVAKGR
ANVDPVSIPWDAVRTLVKQSVYGGRVDSDFDQKLLDTFVDSLFTPAAYNLDFPEGTKLDHFLSWPSWLSLPPTA
ERRKMRSLADDDLERCREWLAALPESFNTLSKQSSVSQDPLGRLFFREGEVGKLLSQVRRDLADVLLSHLTKGT
IPFHWLRYKVKKIPNLARRLAQLDHIAGLDNGGLFFPEAYITATRAVAHRKRWSLETLDLRLDIERVNDPGLVL
EGATWASDVLPDTGFTLPPLQGGSIDEHFYRIGVEAAQPWLNLAKQFAEDMLSFDVETMPNYHPYAVVACAMSP
NHWYSWISPWLLGRLVVGHNVSYDRARILEEYRVEGSNTRYIDTMSLHVAVKGISSHQRPAWMKYRKSKKRWED
ITSANSLADVAKLHCDIDIGKETRNDFMTHAREDILENLQDYLDYCSTDVEVTHAVYAKVFPDFLRACPNPVSF
AGVMTMGSSFLTVNEQWEEYLKNAEGIYRELDDKIKKRLVALAEQVWKEDVWLSQLDWTPKWPKWYWELAKPKK
GTLDVTVRSRVAPLLLQLSWAGWPLFHSREHGWVFRVRLKKLNFYDSGDAHLFFKLPHKDGESANVGSPFGKTF

VKYAQDGTLTSPGDEAKDALDMNAQCSYWISSRDRISNQMVVWDKVGMILPQVITMGAVTRRAIEKTWLTASNA
KKNRVGSELKAMVRAPAGYAIVGADVDSEELWISSAMGDAQFGLHGATALGWMTLEGTKAAGTDLHSKTASILG
ISRDQAKVFNYSRIYGAGMRHAILLLLQSNPNMLPDVAQKQAEKLYASTKGKNTHRDTFRRKFWFGGSESYIFN
KLEEIAMSDKPLTPALGCGVTYALSKEYLSPGFGTDYMTSRINWVVQSSGVDYLHLLIVSMEHLLAKYDIKARY
LLSVHDELRYLVKEEDKYRAALALQIANLWTRCLFAYKLGIDDLPQGVAFFSAVDVDHVLRKEVDLPCVTPSQP
TPIPPGESLNIVKVLEKTNGGSLWRDGYVKPDCLTHRASSANFLRAQATSEFGEIRRLAAALIADILATSTDDL
SKLLAPIDSWRWPRSDLNAWIKVLNKFDAVLEDAIREYDVDGLQVSDFSPQRKQSVCEILKFERLLLENSTNRK
MFNSYDRLNSLLFTSDLDVLVSTLLLLLRPSQQYSSQPALSHSLHISTSRLESLAKGAPALREHGVEMLDLVSK
GEKPVESLPQEASEVNFTFYRHLGPLAQSSRSAMEIFADAVKSHQVPDDKYELLCRVRTARALGAGRQKIVVAR
LLAIAVYAHTHSETQTHSSLFLYDTDLVNRIAELLQHDRGVRKTVSDISNAESTLPNLFVDALVSFITYIASHA
SGGNMVVGAGLVPLLVQVIGITHPQRLPVVSKTMQLVDNVLYGFMNAFTMFCNGRGVEALTERIRYEVDGLLQF
TRAGVLKHLLRSMHRMMQSSGTTEGMRGLIDSSLLKSVDKIIEYRGVFGPTVLPIAVNIVATFVHNEPTSLTAI
QEAKLPETIYKALEAGIEASFEVVQSIPNALGALCLNQTGQDQLAAHPSIIPALFSIFTSEKHIRVLTDKENAA
SIGSSIDELVRHHPSLRTIVFSSLISTLRKIEELGNAYVPPSDIRQYYLLDNIIINYIDAIGRFLEGLFQHTDG
LDCFARLLALPCLPYDYANAVLVQVIRTMVEVAPTETLTHLAKQVKESLDETTEFWQLTGGASNSIFHKLVTLH
IRITLLSDVYATYTHGRQAVGLLLGALHRVCIWENIVLKEGLNALALKHVASQIPLGPFFQAIAKRRNPDEASK
QQAVAISATLANVMYKHLYYTVMLGLITVLLFDELLVQFRKVGGQEMVHVYGGLKMALHLLYSLISFKPAADPG
QVTQYISRKPETHPDYYEAHDFLVKMRLAVSPLIRDIWQSSWLVSAPPAVSKYVIQSVQEIVTGDNEARAPASR
ALTRTNNNVNFATEYLLSHPELDIMRKSFTADIGPLALRLADAHPSIEKFSPAAYDVQEEPLAVRCRLLALILQ
PLPKWLPALLLAMESLLVSAEEPRAVPLVLLTGPAYSEARTTLFELCIRLLHISTLPRDELLATLRMFVELTRD
RNMASQLVHRDGVSLLGFQSHIAIILRHLVEDRNVIESIMKEEVKRWFSNPRAVEVLTYVRNSTSMAARDPLVF
VDVTKDMCTLLRPEAVVHTLLSELIRVGKYACFLMQSLSELLFSYEQCKVAFLTYPRKRSKSVALNFLLSELVS
FGAFNARKRIILCNWAMSVIVALCVDSSPNHTLDLTSIRKLVLESISRSIKDTPSSEPVDTRYGRTLALADLCH
RLLTVRFETPMHLAKIMLEKNFVATLTNVLSDVDLNYPNMRSLVAAILRPLEYLTKVAIKMGRQETPDLYRNSS
LGMYAEAMTHPLLVDRSDNPEFGPLPTIQRWSEEAKITHGKHLQDRVQRLCNHIVLALLPDARERITVTIHGNT
VDITDTGIDPTFLEALPDDMREEVLNQHFRDQRSSRVEQSVESQISPEFLNALPPEIRAEILREERLEPADIDA
ADFIASLDPQLRQVVLLDSDDGIIQTLPKPSQARDAIQLLDRLGIAALVRLLFFPQKTILHKVLLNLCENSKSR
TELFNVLLSILQDGTGDLAMVDKSFSQLSFRPDLVAQRCLDALTFIVATNELSSLFFLTEHELLAGLKKSKKGK
GKEKQAQSHYPIVLLLGLLDRHNLLKTPSIMDSVAGLLDAVTRPLTSLKKILLANPPQIPHPALRSIVNILTVG
DCSGRTFQHTLALIQHLSFLPDARDVVAQELRSKALDFGQNLSADLDALIAALVAKFSPASSEQAKLLRVLKTI
DYMYSIYETFRFTPLWRKLGDCLAVVESKANVENIATILLPLIESLMVVCKNVGVKESVDDLFVSFTDDHRKIL
NLMVRNNPSLMSGSFSLLVQNPRVLDFDNKRNYFNQQLHKRREHFGTLQLNLRRARVFEDSYHAFQHKTGDQIK
YGRLSVRFYAEEGVDAGGVTREWFQILARQMFDPNYALFEPCAADKQTYQPNRASEINPDHLSYFKFVGRVIGK
AIYDGRLMDAHFARSLYRQLLGKRVDYRDVEWVDPEYYKSLCWILENDPTVLDLTFIFGRHAVIPLKENGASIP
VTMENRKEYVQLSAQYRLHSSIEKQIDSLLSGFYEIIPKDLISIFNEQEVELLISGTPDIDVDEWRAATEYNGY
TSSDPVIVWWWRTLKSFNRDERAKVLSFATGTSRVPLGGFGDLQGVQGVQRFSIHRAYGDPDRLPQAHTCFNQI
DLPQYSSYEKLRQQLLLAINEGGEGFGFAKSSASSATRKKHARKLRRLGKKDSVTKRKALEDLQHLPSLLLSSS
RRIRLLAAGIHASLLGSWCMATEDVDRQVAVQAKKSWFIKRALFDPVAIYTDLNPEESEGDRKGRIRVGALGVL
KWGDSQPTVRKAAWGVLLSLLSEAVLRSAWLEPDAGVRASMWEPLLTFLTQNPKAWAYREFLQFLQLGSPVQGY
PTVIIIVSTIPPSILISFWAALDGRALAFLSSLLECLVLLVKRPDFFVFGFLLPPSQEGLDDILRDFPSNPLDS
VLAIVDPLDHAGFSEYTRVVNALLNRPLARSNIWLLRHFLTLDNVRETRVLYAVMQHVLNGAASLDAEQWMTLA
RRLEKQSPNTALAIILAVTESALEPPSLNRYRNELAAGILGIRGLALLRRLAITAPDPDSDVVFLPQNRAVNFM
KVCQTWVTSDIDENVESEMTLIFLHLAPILQNVPGAHWEFVFDVIENNLENCSFADSSTLPALARTLKLILAVQ
DLAITNKSLRSWHERELPILTLVRDLSVPRSVCWEMAFSIEYLVVEAGVDTASLRVKMGYATQLRDLDMIGSSF
LPIVLDLLDVYKPFQLEVWSVEEYFVQLYDQFSPKLLAAHLFYRALLNVPSLISSWWGSCKDRQLSTAVASLTT
KHYSPVLIAAELGHVKDPVEEKKWRGWLLAVQQIQNGRIVDGLSMFKKNVTHHFENQTECAICYSIIKRCRTCK
NRFHAGCLYISVSSTLDMS TVKTSSPRPF ELEDCPTFYP SPEEFKDPMS YIRSISPRAQ NYGIVKVVPP
IGWKMPFVTD TEYRFKTRAM RLNSIEASSR AKINFLEALY RFHRQQGNPR VTVPTINHKP
LDLWSLRKEV QKLGGFEAVK GKKWADLGRL LGYGGPGLST QLRNSYIRVI LPYEHYSERV
RGSLCDGCDC GFHTFCLVPP LSTIPKGQWF CHTCLFGTGD FGFDEGEEHS LSSFQARDQA
FRKMWFQTHP PVTELDVENE FWRLVQSPFE TVEIEYGADV HSTTHGGMPT LETHPLDPYS
RDAWNLNNIP ILPDSLLRYI KSEISGMTVP WTYVGMIFST FCWHNEDHYT HSINYMHWGE
TKTWYGIPGE DAAKFEAAIK KEAPELFETQ PDLLFQLVTL MSPARLKESG VRIYACDQRA
GEFVVTFPKA YHAGFNHGLN FNEAVNLALP EWLPLGRECV KRYQEHKKLP VFSQDELLIT

ITQQSHSIRT AIWLNDSFKE MTDQEMEGRR AVRLGVREIL EERDAEEQYQ CVYCKAFCYL
SQVICPCVVC LEHVKYLCDC RVLRLRFSDE ELLNIQSTIA ARAAIPENWH SKLTKLLNDS
AKPQLRALRA LVAEADRINY PLKELSALRK CVTRANDWVD VANSFTTRKQ SRKRERPEKT
LDELYAVLKE VDNLGFDSNE IALLRNLATQ AEDFKARASA LLCDALLAHG SSLNVNLEEL
YKVENTVLQD HLIKELLDEI RQYLARAKAC DLPPDNKYMK VLEAKLQAGN DWDERAASVL
TQPVKTIDEL PVDPAVLNRI KATRTRALEF ERQAKGWLSP ELPSVQDALR LVQRAEKEFS
IPAIHDLKRT ADFAYDLEER CEAVLKDRYQ HGSVFDAMDK WRTYAREHLT FRLPSFDKLN
AELELHEQWV KKLPWYCHGR EILNDVLDYT KAEDDTPPDD EFFTCICFDP VRPPPPGKTS
DAVQCDHCFA RFHGKCAVNG GSCPFCDPNH WNGNIHNERN YHFCYLPTVL HNASEITKSY
SEYWEQLKLI VQHVERLCAV IGHFLSFASN QRAEYIPQVR HYMRKLYKIQ FAVSPNPEVS
FGLDLAGLHR ILASRRMKKR RRPRFLFGQD IDRQWVDGTK CICRGQTCQR KYHTACVFTC
PLCCLRKAKP YRWADIRVRQ FSRDIIRIKL TPPVTPTIFI DLIQFTP
>Trichaptum abietinum
MRRTTQDQSIVLSGETGSGKSENRRLAIKSLIELSVSNPGKKGSKLSSQIPSAEFVLESFGNARTLFNPNASRF
GKYTELQFTRGRLCGVKTLDYYLERSRVAGAPSGERNFHIFYYLAAGATAEERQHLRLTDKAHFRYLGPRDAMR
FDQLKVALKSVGLSKRMVAQTCQLVAAILHLGNLEFIVDRHRNEDAAVVKNTDVLDIVAEFLGVQPQALETALS
CKMKLVKKELCTVFLDPDGASDNRDDLAKILYSLLFSWLNESINQKLCRDDFSNFIGLFDLPGPQNMSRSNSLD
QFCVNFANERLHGWIQKQMFEAHVDEYNQEGISRFVPTVPYFDNAECVRLLSNMPGGLIHIMDDQARRAPKKTD
HSMVDAFGKRWGNHSSFKVGGMDRFPTFTINHFNGPVTYSSEGFLEKNIDALNPDFVSLLRGGSVNPFIRSLFS
NKAIATQAHPRNEDTIVAAQQPVKPMRAPSTRRKGTPCVAGEFRSALNTLFTTLDETQAWNVFCINPNDSQLPN
QLEGRAVKGQVRSAGLSEIARRCVNIFEVNMTLEEFCERYRDQLSALNIHEGEKEIVIGQHKVYLSQRAFHKLE
DRLRGEDMEEQKRNRLRDAEASSQQLPLVANASPFDDIDGKSRLTSNRESYAPSRNMFGNTDGEVLEGETTEDI
KESSARRRWVAFVWLLTWWCPNIFLIWCGRMKRLDVRQAWREKLALNMIIWFMCLCTAFVIAVLGALICPTEHV
FSTNELSSHSNNVYTAVRGEYGGTTADSIFPVQVSALCNGVSGSVNPFVTLDSSNTDPNAQYHDFRAFTSDSRP
DWYFESMTLMRWNFRVGFVGYTMKEVKQMANSGRSAAIYNGLSLPAVMFTSTSSTFLTFKPQRPLHFTCLTIGS
RGDVQPYIALAKGLMADGHGIEFGYVGGDPAELMRICVENGMFTFSFMKESLQKFRGWIDDLLKTSWEACQGTD
VLIESPSAMAGIHVAEALRIPYYRAFTMTWTRTRAYPHAFAVPEHKSYVAFDQVFWRFTAGQINRWRRNTLKLG
STNLDKLEPHKVPFLYNFSPSVVPPPLDWPEWIRVTGYWFLEDAEVSAQKWSPPQDLVDFIEGAHKIGKRVVYS
DPEAMTKCVVDAIKRGGVYAILSKGWSDRLEPEVPLPPEIYPIKSIPHDWLFAGIPTIIKPFFGDQFFWGDRVE
ALGVGTSVRKLTVENLADAFIAATTDEKQIAKAKLIGQRIRAVSKDRPREIFEPLRLACETGNEKLQVVSLDCI
SKLISYSFFVELVDVVVHTITACHAETTPDTVSLQIVKALLSIVLSATLLVHQSSLLKAVRTVYNIFLLSPDAV
NQTVAQGGLTQMVHHVFDLFIKDAFLVFRALCKLTMKNLNTESERDLKSHAMRSKLVSLHLVLTILNSHMNVFV
DPSSIIFSTSSNEATSFINATKQYLCLSLSRNALSPVPQVFEISVEIFWRMLSGMRTKLKKEIEVFLHEIFIPI
LEMKTATLKQKSVILGMLQRLCQEPQALVELYLNYDCDREAADNIYEHLMNTISKIATPALTTNALAVPGHNLG
MSEQQLKRQGLESLVAVLRSLVTWGIDDPERFESAKQRKTILQDGIRRFTSSPKGIAFLLENGFIPARTPADIA
RFLLHTDGLNKSAIGEGDEENIAIMHSFVDQMDFANTPFVDALRMFLQAFRLPGEAQKIDRFMEKFAQRYIEGN
EAAYVLSYSIILLNTDAHNPQVKKHMTKEDFFKNNRKINDFPEEFMSIIYDDITMNEIRMKDEIANVGRDLQKE
AYVMQSLGIANNQFFSASHFVHVKPMFEVAWIPFLAGISGPLTDTDDLEVVELCLEGFKAFVTTLAKFTFLNNL
GEMKAKNMEAIKTLLDIAVSEGNHLRGSWYEVLTCVSQLERMQLISSKSSRRMPAEELANESRSTHITVAADMV
FSLSHFLSGTAIVEFVRALSSVSWEEIQSSGLSEHPRLFSLQKLVEISYYNMGRIRLEWTNLWEIIGEHFNQVC
CHHNMHVGFFALDALRQLAMRFLEKEELPHFKFQKDFLKPFEYTMIHNTNPDIRDMVLQCLQQMIQARVHNLVS
GWRTMFSVFSAASKVLTERIVNSAFEIVTRLNKEHFSEIVRHGAFADLTVCITDFCKVSKDDAMIKFWFPVLFG
FYDIIMNGEDLEVRRLALDSLFTTLKTYGSSFTVEFWDTVCQEILFPIFAILKSSSDLSRWSTQEDMSVWLSTT
MIQALRDLIDLYTFYFETLERFLDGLLDLLCVENDTLARIGTSCLQQLLESNVKKLSVAKWERVVTTFVKLFKT
TTPHQLFDENLRMERRRIFKQIIVKCVLQLLLIETTYELLQNKDVYNTIPPEHLLRLMGVLDHSYQFARMFNED
KELRTALWKETNSAETLVNILVQMYYDPRPEHVALRPQVADKLLPLGLGVISDFNKLRIDTQAKNIAAWMPVIS
IILQGFVRLDEKAFGRYLPAIYPLTSELLAKDSEVRAGLRDYFIRVGQVQGIQRQALIAAAHTKYGHEAVSPIL
QQLLPKMSLPPGTTLVQCLIQLGADITSEGDVTRALLARFGITEQNPPKDAQVVEIFSTLGRHAAEGTTLCDVG
ALVRAISSLRAKLDWSKVIEAFDWSDRGVDTATLKLLIAILVNSPRADKPAVAGFWTNWKNSLYQLRLLDALLS
LPSDTFNFVTLPGRRVVAVEDVASASPTIKSLAANVQSHTWNSLDLFEVLVRLGDSENADVRNCVRDMLDKAMR
ISADIVHMGLLQVPPWNSLQIEYSRQLLGMFLAGHPNHQLVFMRIWQIEPTYLTTALREFYDENPLNITRILDI
LDSLLEVRPFTFALDEYLNLDKWLADNVSAHGAEFLRAVITFLDLKAQNEKIARTLDPNAKTMALSAPTITIFL
RTLRMHSSLLDDRDAEYCLEVRNACLQIHPRLMDQEPGFSVVSYTPDIEAEVDGIYKQMYDEQITIDQVIQLLQ
RKKDSTNPRDHEIFSCMLHFLFDEYKFFQYPARELGMTGYLFGSLIQHQLVDYIPLGIAIRYVLDALQCPPDTN

LFKFGVGALLRFESRLPEWKPLCQALLEIPHLAEQRPDIVENVRRAEVSDKVLFIINNLAPSNFDDKLAEMKER
FSTEPNNHQLYLRFLDGLDSKVLARFILHETYVKSAVMLNAEKTKTSSSERAILKNLASWLDKPIKHKNLSFKD
FLIEGADNDRLIIAIPFTCKVLEWAGKSKAFKPPNPWLMAVIGLLAELYYYAELKLNLKFEIEVLCKSLGLDLD
KVEVANILRSRPRNVGAHIEEILTALSNSVVVNPQLALHGNPVFKRAVQEAVDRSVREIILPVVERSVTIATIT
TRELGMKDFASEASEEKMRKATHLAGQKLAGSLALVTCKDPLRQNMPGHMRAYLNDHGFSEQQVVLLLVSDNLD
IACEAIEKAAMERAIREVDTALAGSFDSRRRHRDARSGSPFISNLPDPLRIKLTGLQPHQLRVYDEFFNLLIEK
VEASLANCPPSLSTVPSNHELHSLIPFLRTILDRLRAPLLMSQKIVQLLYKTPTQVGRDVYVTLLKELCKEYAE
VKKEATNWLIFAEDERKFNVAVTVTLLRNGLVTITDEDMQLAKNARPSLQDFAAGFLRECLTTDATRQSIPYAL
DCFQQWVNVFQRSSTPEKSFVPYITQLTKQGILKAEDSSFFFRVCAESSAAHYTACMAQGDYENQFLALDAISR
LIVYIIKYHGDADNVHAKVHYFKKILSIVVLVVANMHEEQGFQQKPFFRFFSSLLSDLHSIETYLGNAYFQLLI
ALCDTFSSLQPVYFPGFAFSWMTLISHRLFMPKLLTSENREGWSAFHKLLLALFKFLAPFLRSAKMSGASRNLY
RGTLRLLLVLLHDFPDFLSEYYFTLCDIIPPRCVQLRNVILSAFPTSITLPDPHLRGLSMGPIPPILSDFTLIL
KNGDLRTYLDQCLLNRATPGSLAALKDRLETYNLSLMNAVVMYIGVSSVAQAKARSGSALFVPADPGVVALMYL
AVNLDPEGQHHLISAMILHLRYPNAHTHWFSSLLLYLFSEVSNERFKEVMTRVLLERFIVHRPHPWGALVTFIE
LLRNPKYDFWKKEFIHVAPEVTLMLEGVGHKRLYLQLCFRNVSDLLSVRREIVPLATENGSKLNAVDAYAEVVD
PRDCIIDAREFDVPYYLRVAIDNEIRVGLWYAITFVAGHPQFDRVKRADPVVMAYDIETTKAPLKFPDQAIDQV
MMISYMIDGQGFLITNREIVSEDIEDFEYTPKEGLEGPFTIFNEADEAATIMRFFQHIQEVKPTVMATFNGDFF
DFPFLYARSKVHGIDMFLETGFTKDSEEEYKSRTCVHMDCFRWVKRDSYLPQGSQGLKAVTTAKLGYNPIELDP
ELMTPYAMEQPQTLAQYSVSDAVATYYLYMKYVHPFIFSLCNIIPLCPDEVLRKGTGTLCETLLMVEAFRGHII
MPNRHEDEHGNMFDGHLLASETYVGGHVEALEAGVFRSDIATHFKIVPEAAQLIDELDAALTFYVTNYDEVKAQ
IQSALEEMRDNPLRFDKPLIYHLDVAAMYPNIMLSNRLQPDSVVDESVCAVCDYNRPGKKCDRRMTWAWRGEYF
PAQRDEYNMEMFPPRKPGGPQRRYVDLTQTEQTALLHKRLGDYSRKVYKKTKETKVVNRESIICQRENPFYVDT
VRRFRDRRYEYKGLHKTWKKTLDAVAEVDDAKKMILAHKCILNSFYGYVMRKGARWHSMEMAGITCLTGATIIQ
LARQLVEQIGRPLELDTDGIWCMLPGVFPENFKFKMSNGKTLPISYPCTMLNHLVHAKFTNHQYHDLDPETGEY
MVHSENSIFFELDGPYKAMILPSSKEEDKLLKKRYAVFNDDGSLAELKGFEVKRRGELQLIKIFQSQIFERFLL
GTTTQECYAAVSEIADRWLDVLFSKADNLSDEELVELIAENRSMSKTLAEYGGQKSTSISTAKRLAEFLGNQMV
KDKGLACKFIISAKPIGAPVTERAVPIAIFSAEESVKRTFLRKWLKDNSLANFELRSILDWDYYIERLGSVIQK
LITIPAAMQKVPNPVPRIRHPDWLHRRVAALDDKFKQHKVTDFFRSRYTMWLSVNSDLVPVTVRIPREFYLNFK
TAEHYSRERVVRTLPRDKPCLHLFMNEINNPNVDGAYELQTLNKARDDGVDLSQVDRKYIFVYHAFSPSAPVNV
FAIFLPDGVRLHVVDPASRRQPTDYHSNEATALKAISRELGTLENKSFTVVISSAKDLTYFTSAVPKLGKFPVL
RMPSSRAGHALDFPWQANVAKKMFSRYLSFAPWLKRAITQAAYYPLFLCDIELARRVVAHDMVLWWSNGERPDL
GGLEYDLPPTEELISPEFLTPGLYSNVCLLIQVRNLAIDAVLQSAVVNELEGTGGTTAFDSSQPNVSLGESNLS
PQTFSVLKQMLKTWLLDKASPSSITLDHFWRWVSSSASRMYEPSIQRFVHGLMRKTFIQLLAEFKRLGSNVVYA
DFSRIILVTSKPPGTAHAYATYITTAVTSHELFKHIYLRTDRYYDLLLYMDPANFGGVVCEDPLALEPPVKLSI
LSCWNIKNFLPPAMQDHFRSIIRLFISQMAKIKRDKLREIDGGKAFIAQKLTRKLLQIVFPVLPGSYLHLTHPA
LEFIKFTCAVFGLAQDLQIEIGILKKNLLELIGVRQFADDAIFRNPCDPLKLSMVTCKYCDHIRDFDFCRDDEL
LPWYCPECEGEYDRAAIEFSLIQVLHRLERNFAQQDLRCAKCKQIQSDNVSRHCCSGSYQLTIAKTDVRRKLRT
IVNVAKVHNLGRLFEVEHLKYATLATSPHLETQKQVASIKTARNIIEYNEQYVTKRLLVNLIWAFSGDARGEWI
SWSSRVPKIEILYSWLSEHKPLMLCGPPGSGKTMTLFSALRKLPDMEVVGLNFSSFRKTPNGVILAPVQIGRWL
VVFCDEINLPATDKYGTQRVISLVESGGYYRTSDMAWVKLERIQFVGACGRVPLSHRFLRLVMVDYPGEISLKQ
IYGTYSRAMLKVVPNLVNYAEPLTDAMVLFYLASQKRFTSDTQAHYVYSPRELTRWVRGIYEAIKPLEILSVEG
LVRVWAHEALRLFQDRLVTEDEKVWTDENIDMTAMQCFPTINILFSNWTSKNKARLRVFHEEELDVQLVLFNDV
LDHVLRIDRVFRQTTLSRFVAWMNGLSIFQIKVSNKYTGEDFDLLANAEVPGLFEGFTMNPPENGLASRAATSP
ALFNRCVLDWFGDWVPKYNPPQYFPIAYRVCNALVYVHQTLYQFNKKLSRRQGRYPRHYLDFLQHYVKLYNEKR
DELERHLHVGLDKLVTQVEELRKSLAIKNKLKRMVSDQQEAEQKKAASIEIQAALVEQDRNIDQRRNVVMADLA
DAQQMTKQLRELMKRDFLSRPSFNFETVNRASKACMITELETSIKRYKEEYALLIRVQSKVDRSMRLLESLSRT
FDAEMSTIVGDVLLSAAFLAYGEWSNHLTEAGIMFKPELSFPRLSWLSKSLPSDNLCTVTSFLDDAFLKVLESA
LRFGNPLLIQDVEHLDPILNEIRRTGGRVLIRLGNQDIDFSPSFPSVEFSPDICSRVTSQTLDQVLKVETDLMK
VQGEFRLRLRTLEKLLLQALNESTGNILDDDKVIDTLETLKTEAAETDIVMSEVEQVTAEYLPIAQACSSVFFI
LEQLNLINHFYQFSFMNIFDYVLHHNPNLQGVLYDDLFLIVFRRTSRALDEYEFLLEGGLQSHSRHIIFKPVQV
HLRDHEDEWSAFLILVKCFRPDRLVQSVKGYDASFRVENQEGFSLADQAIAQASRQGTWVLLKNVHLSPSWLGE
KKLQTLNPHRNFRLFLTMEANPVIPVNILQSRILMNEPPPGIFLLAWFHAVVQEDMNAAFGTIDTWLHSVAKGR
ANIDPMQIPWDAIRTLVKQIVYGGRVDSDFDLKLLDTFVDSLFTPASYNLDFPEGTKLDHFMSWPSWLSLPPSA
ERRKMRSLTDDDLERCKEWLGALPESFNTLQKQSADNQDPLYRLFFREGEVGKLLRQVRRDLADVLMSHLTKAT

IPTHWLRYKVKKIPNLALRLQQLDKIASLDTGGLFFPEAYITATRAVAHRKKWSLETLDLRLDVERVNDPGLIL
EGAAWVGNILPETNITLPPLQGNSIDEHFYHIGAASAQPWLDLAQRFAEEMLSFDVETLPNYHPYAVMACAVSA
THWYSWISPWLVGRVVVGHNVSYDRARILEEYHVGGTNTRFLDTMSLHVAVKGISSHQRPAWMKYRKSKKRWED
ITSANSLADVARLHCGIDISKEIRNDFMTHSREEILENIQDYLDYCSSDVSVTHSVFSKVFPDFLKACPSPVSF
AGILTMGSSFLTVNEQWEEYLKRAEGTYQELEEKVRRRLIELAEKAWKNDVWLSQLDWTPKWPKWFWDLTAPKK
GTVDITVRNRFAPLLLRLSWSGWPLFHSRQHGWIFRVRLKKLNFHDPNDTLLFYKLPHKDGELANVGSPFGKSF
LKYAQDGILTSPGDEAKDALDLQAQCSYWISSRDRILKQMVVWDKWGMIIPQMITMGTVTRRAIEKTWLTASNA
KKNRVGSELKAMVRAPKGYAIVGADVDSEELWISSAMGDAQFGLHGATALGWMTLEGTKAAGTDLHSKTASILG
ISRDQAKVFNYSRIYGAGKKHAILLLLQSNPNMLPEKAEAQAEKLYASTKGKNTHRDLFGRKFWFGGTESFVFN
KLEEIALSDNPQTPALGCGITHALSKEYLSPGFGTDYMTSRINWVVQSSGVDYLHLLIVSMEHLIKKYNIQARY
LISVHDELRYLVKEEDKYRTALALQIANLWTRCLFAYKLGMDDLPQGVAFFSAVDVDTVLRKEVDLPCVTPSQP
VPIPPGESLNITQVLERTNGGSLWSDGYTRPNCLVHRASSAYFLRAQATAEFGEVKRLAAALIADIIGTSTEDI
PKFLGAIISWRWPRSDLNAWIKVLNKFDTVLEDVIREYDVDGLQVRPFSPQTKETICAILNFERLLLENSTNRK
MYNSYDRLNSLLSTSDLDVLVATLMLLLRPSQQYSSQPALSHSLHISTQRLESLAKVPPSFREQGIEMLDLVSK
WDKSIGELPQEISEVNFSFYRHLGPLAQTSRSTMEILADAVKSHQVPEEKFELLCRIRNAWALGQDRKKLVISR
LLSIAVYAHTHPENQAQSSLFVFDSDLVSRIADLLQLDRGVRKTVTDISRPESELSNSFVDALLSFVILLASQP
MGGNMVVGAGLVPLLIQVIGINQPQRLPIVSKTMQLVDNVLYTFVNAFSIFCNCRGVDALTERIRHEVDGLLQL
QRAGVLKHILRSMHRMMQSSGTTEGMRGLIDSSLLKSVDKIIQYRGVFGPTALPTAINIVATFVHNEPTSLTAI
QEAKLPETIYSALEAGIEPSFEVIQAIPNVIGALCLNQTGQDQLASHPSVIPALFSILTSERHLKVLLDKENAV
SIGSAMDELVRHHPSLKTIVFSALVATLKQIEDLGNNYVPPEEIRQWYSLDNLVVNFIDALGRFLEGLFQHTEG
MDCFGRLLALPCLPYDYANSVIVQVVRTMAEIAPTETLGHLSKQVNESLDETNKFWQAPQSRSNDFFRRMVTLH
IRLTLLSDVYATYTHGRNAVALLLGALHRVCIWENIALKSSLNALALKHLASQIPLSPFFQAIAKRRNPDESQK
QQAAAVSAVLAETMFKHIYHTVMLGLITVLLFDDLLVHFVRLGGQELVHVFGGLKMALHLLYSLISHKPSADAA
QIALFISRKPESHPEYYEPHDFLVKMRLAVAPLIKEIWQSSWLTSAPPAVNKYVIQSVQEIIVGANEPRASAIR
ALQRSHNNVSFATEYLLTHPELSTLRETFTTDLGPIMLRLVDAHPSIEKFSPMAFDVQEEPLSVRCRLLALVVQ
PLPKWLAALLLAMEIFLTCAEEPRPVPLVLLTGPPYTEARATLFDLSLRLLRIPSLPRDELLAILRVLVVLTRE
RDYADKLIGQGGVPLLGIQSHVAIIFRHLVEDRILLESVIGQEIKRIMTGSRTIDVLSYVRSSQALAARDPRIF
IDVTQKCFTLVRPDIVIHHLLGELLRVGKYACFLMQCLSELLFSYDQCKLAFLSYPKTRSKSTALNFILSELVS
FGAFNARKRIILCNWAMSVIVALCVDGGSNGSVDLPSVRKLVLESISKSLKESPPHETIDARYGRTLALADLCH
RLLTVRFETPMQLAKIMLEKNFVATLTNVLTDIDLNYPNMRSLVAAILRPLDFLTKIAIKMGREETPDLYRNSS
LGMFTESMAHPLLIDRSETTEFGPQPTSQRWLEEAKILHGKHLQERMQRLCNHLVLALLTEARERITITIHGNE
VDITDTGIDPTFLEALPDEMREEVLNQHFREQRAARADQRADSQISPEFLDALPAEIRAEILQQERVEPAEINA
ADFIASLDPQLRHVVLLDSDDAILQSLPKATQIRDAIQLLDRTGIAALLRLLFFPQKNTLHKVLLNLCENSKSR
TDVFNLLLGILQDGSGDVALVDKNFAQLSFRGDVVVQRCLDALSFIVETNELSSLFFLTEHELPASLKRSKKGK
GKEKAPQSHYPIVLLLGLLDRQTLVKTPSILDSIAALLDAVTRPLTSLKKILLLNPPQIPHPALRLIVNILTVG
ECSSRTFQRTLALIQHLAYLPDAREVIAQELKVRAQEFGQNLLVDLDALISALASKFSPASSDQAKLLRVLKTI
DYMYSIYESFRFTPLWRKLGDCLSVVESKANVEHVSTILLPLIETLMVVCKNVGLKPSVDDLFVSFTNDHRKIL
NLMVRNNPSLMSGSFSLLVQNSRVLDFDNKRNYFNQQLHKRREHYPTLQINVRRARVFEDSYHTLQGRSGDQIK
YGKLSVRFYGEEGVDAGGVTREWFQILARQMFDPNYALFEPCAADKQTYQPNQASSINPDHLLYFKFVGRIIGK
AIYDGRLMDAHFARSLYRQLLGKPVDYRDVEWVDPEYYKSLCWILENDPSILDLTFIFGQHKIIPLKENGASIP
VTLENRKEYVQLSAQYRLHSSIKDQIESLLSGFYEIIPKELISIFNEQEVELLISGTPDIDIDEWRAATEYNGY
TSSDPVIVWWWRAMKSFNRDERAKVLSFATGTSRVPLGGFVELQGVQGVQRFSIHKAYGDPDRLPQAHTCFNQI
DLPQYSSYEKLRQQLLMAINEGGEGFGFAKSSASSATRKKHARKLRRLSKKDAVTKRKAIEDLRHLPTLLLHAS
RQIRATSASLHYSLLGSWALSAHDVDRQASSSARRVWFAHNTVLDPLALYHELNPEESEDDRKARLRIGALGIF
KWGANQPAVRRSAWSMLLSLLSSAVLRSAWVEPDPLVRGVMWEPLLVFLTQKSRAWAFAEFLRFLQLGSPVQGY
PTVVVILSTVPSSILTSFWAALDGRALAFLSALLECTVFLVKRPDIFIFAFVLAPSEDSLADMLQELPSYPLHP
AIAVVDPLDSDGYSEYARVVSALLDRHLARNSIWLLRHFLVLDTAQTARILYTTMKHVLSGADKADAEQWMTLG
RRLERQAPQASLAIVLSITESGLEPPSLDRYRNEQASGILGIPGLALLRRLAVSAPDPESDVIFLPQTRAVNFM
KTCQAWLSSDIAEDVESEMTGVFVHLAPILQNVPGSHWELIFDLIENNLENISSLENSNLPVLSRTLKLLLTIQ
DLSKTNKSLRTWKEHELAILKLVRDLSVPRQVCWEMAFSIEYLVVEAGVDNASLRVKMGYASQLRDMGLISTSF
LPNVLGLLNVYKSFSLEYWKVDEYYVQLYDVLSPKLLAAHLFYRALLNVPSLIASWWGECKDRQLSNAVATFTT
KYFSPVLIAAELGHVKDPVDEKKWRGWLLAVQQVQNGRIVDGLSMFKKNVTHHFENQTECAICYSIIKRCKTCK
NRFHAGCLFIPISPTLNLD SVKTSSPRPF DLEDCPTYYP SPEEFKDPLG YIRSITPQAK NYGIAKIVPP
IGWNMPFVTD TEFRFKTRIQ RLNSIEASSR SKINFLESLY RFHKQRGRPN VAVPTINHQP

IDLWSLRNEV DNLGGLEAVQ TKKWTDVGRL LGYRGPGLST QLRNSYVRVI RPYEEFLAEV
RKTLCDGCDC GFHIFCLNPP LSMIPKGQWF CDTCVIGTGD FGFDEGEEHS LSSFQARDKA
FRQMWFQSHP PMSEYDVEKE FWRLVQSPYE TVEIEYGADV HSTTHGAMPI LEMHPLNEYS
RDPWNLNNIP ILPDSLLRYI KSEINGMTVP WTYVGMVFST FCWHNEDHFT YSINYMHWGE
TKTWYGIPGE DAEKFEAAIK KEAPELFESQ PDLLFQLVTL MSPARLTQSG VRVFACNQRA
GEFVVTFPKA YHAGFNHGFN FNEAVNFALP DWLPYGRECV RRYQEHRKLP VFSHEELLIT
ISQQSHSIKT AIWLTESFGE MVDKELANRK AITLGISEVL EERDPEDQYQ CIYCKAFCYL
SQIICPCPVC LQHNEFLCDC RTLRLRFSNE DLRNMKMTIA NRAQIPTAWR SKLMKLLKES
PKPQLRAMRA LVAEADRINY PLPELVWLRK CVARANQWVD SANSFITRKQ SRKRDRPEKT
LEELFAHLHE VEDLGFDCPE IGQLRTLASS AKDFKVNAGV LLCETLLAHG SSLNVHLEEL
YEVENIVLQE QLMKELLEET RQFLSRAKAC DLPDDNKYMK LLEDKLRAGN DWDERAANIL
KQPIRTIEEL PVDPAVLSRI AATRARALEL DRQAKVWLSP ELPRVQDALR LVQRAEKEFS
IPSIDDLKRT ADFASDLEER CEAVLKCRYQ HGSIFDGIEK WVAYAQEHLT FYLPNFDKLK
KQLELHRLWI KKLPWYCHGH EILNDVLEYT KPEDDNPPED EFFTCICFDP VRPPPQGQIS
DAVQCDHCFA RFHGKCAANG GSCPFCDPNH WNGNIHQERS WHFCYLPTVL QCAPDITKNY
SEAWKELENI VQHIERLCGV IGHFLSFASN QRQEYIPQVR HYMRKLYKIQ FAVSPNPEVS
FGLDLAGLHR ILASRRLKKR RRPRFVFGQD VDKDWIDGTR CICRGQTCNR KYHAACVYAC
PLCSLRKVKI YRWADIRVRN FSRELIRIKL NPPMTPTIFI DLIQFTP
>Sphaerobolus stellatus
MRRTGQDQSIILTGETCSGKSENRRLAIKAILELSVSNPGKKGSKLSQQIPSAEFVLETFGNARTLHNPNASRF
GKYTELQFTRGRLCGVKTLDYYLERNRIAGALSGERNFHIFYYLVAGASAEERTHLKLAEKTAYRYLGHGDSQR
FDQLKQALKSVGLSKRHVAQTCQLLAAIIHLGQLDFTIDRYRNQDAAVVRNLDVLQIVAEFLGVQPSALESVLS
YKTTMVKKELCTVFLDAEGASDNRDNLAKTLYSLLFAWLNESINQKLCREDYNTFIGLFDLPGPQNLSRSNSLD
QFCINYANERLQNWIQRELFENYTEEYNIEGIARFVPKIPYFDNSECIRLITNKPGGLIHIMDDQARRQPKKTD
HTMVEAFSKRWGNHSSFKVGTMDRFPTFTVNHFNGPVTYSAESFLERNCDAVNPDFVSLLRGGSINPFVRGLFS
AQAIATQAHPKDEDTIVAAQQPVKPMRAPSTRRKKSPGVAGEFRAALDTLFETIDETQSWFVFCINPNDAQLPN
QLEGRGVKIQIRNLGLPEIAKRSAVVFEANMTPAEFCDRYREQIAAVGIIEGEEDIVVGQFKVFLSHVAFHKLE
DYLRANDAEETKRNRIREAEASNQQLPLVAHASPFSDYDGRSQLTSARESYAPSRNMFQNAEGEILEGEVTEEF
RESSARRRWLFIVWTLTFWCPNFLLSYVGRMKRLDIRQAWREKLAINMIIWFLCACAAFVIAVLGVVICPTSHV
FSMSELQDHSNNAFTSIRGEYSGLTADNIFPVQVSALCNGVSGSVNPYVVLNSDNTDPNAQYHDFRVSTNDPRP
DWYFEQMVQMRFLYRMGFLGFQPNEVSNMADQGKAVGIYNGLSLPAVMFTSSSSTFLTFKPKESLHFTFLTIGS
RGDVQPYIALGKRLLQEGHNIEYAYVGGDPAELMRICVENGMFTVSFLKEGATKFRGWIDDLLKTAWEACQGTD
VLVESPSAMGGIHIAEALKIPYFRAFTMPWTRTRAYPHAFAVPEHKTYVMFDQVFWRGTSGQINRWRRKVLGLP
STNLDKLEQHKVPFLYNFSPAVVPPSLDWYEWIRVTGYWFLDDADVGARKWTPPDGLVEFIDSAHAAGKKVVYS
DPGAMTKCVVEAILKSGVYAILSKGWSDRLQPKESYPPQIYPISSIPHDWLFPGIPTIIKPFFGDQYFWADRME
ALGIGSCVRKLTIDHLTAALITATTDERQISRARAIGQTIRAEDGDRPRDIFEPLRLACETRNEKLMIASLDCI
SKLISHSFFVELPDLVAHTITSAYTETTPDSVSLQIVKALLALVLSPTVLVHHSSLLKAVRTVYNVFLLSQDPV
NQVVAQGGLQQMINHVFDLFIKDAFLVFRALCKLTMKALNTESERDVKSHAMRSKLLSLHLVLTILDSHMNLFV
DPRSNIYSSSSNEVTPFIQATKQYLCLSISRNAVSLSPQVFELSVEIFWRVLSGLRTKLKPEIEVLLNQILIPI
LEIRSSTLKQKVVLLDMLHRLCQDPQALVEIYLNYDCDEESTENIYERLMNIISKITTPTLSTNALSHTPSHQT
LTETQLKRQSLECLVAVLHSLKAWGTDDPERFESAKQKKTTLLEGIRKFNIKPKGIQFLLETGLIPSRTPQEIA
KFLLYTDGLNKALIGEGDEENIATMHAFVDQLEFTDVPFTQALRLFLQTFRLPGEAQKIDRFMLKFAERYIAGN
DTAYVLSYSVILLNTDAFNPQVKRRMSKQDFIKNNTGIDDLADEYLGVIYDDITSNEIRMKDEVANVGRDLQKE
AYILQSSNMANEQFFSASRAVHVRPMFEIAWMSFLAGLSNPLQDTDDLEIVEICLDGFKAFVTTLAKFTFLNNL
GEMKTKNMEAIKTLLDVAVDEGNYLKGSWHEVLTCVSQLERMQLISSGRHKKLPNEDLANESRSTHITVAADMV
FSFSPNLSGAAIVDFVQALSDVSWEEIQSSGLSDHPRLFSLQKLVEISYYNMKRIRLEWSNLWAILGEHFNQVC
CHSNPNVGFFALDALRQLAMRFLEIEELSHFKFQKDFLKPFEYTMIHNPNPSIREMVLTCLHQMIQAKVHNMRS
GWRTMFGVFSAASKVLTEDVVNSAFELVTRLNRNHFSDIVRYGAFADLTVCITDFSKCSKDDPMIKYWFPILFS
FYDIIMNGEDLEVRRLALDALFSTLKTYGTSYPVPFWDTVFQELLFPIFAVLKSSQDLSRFSTQEDMSVWLSTT
MIQALRDLIDLYTYYFDILERVLDGLLDLLCVENDTLARIGTSCFQQLLENNARKLTPEKWERIVTTFVRLFKT
TTPHQLFDESLRVERRRIFKQIIVKCVLQLLLIETTNELLQNPEVYQTIPPEHLLRLMGELDHSYQFARIFNED
KELRTGLWKESSSAATLVNVLTRMYKDTRLDAQQSKQQVADRLIPLGLGVLEDFNALKADTQAKTIATWTPVVA
EVLQGFNSLDDQTFSRYLPAFYPLTVDILSRDPGLRESLRSFFLRVGFSCGIQRHDIIMTVQSKYPHDIISPVV
QQVFANLSVPSGSNVVEILCDLGTDITSDPSYVRAVLQRFGFTEAVPPTDVQVTEMMSTLSKRTFESTQLCDAR

TLLRVLNSFDVNINWPSLIKAFDWPDRGVDTPTLKLLIVILLNSPHVEPHAVAGFWTIWTNSLYQLRLLDALLS
LPADTFNFVSLPGRRIVTVDDVANASPTIKALAANVQGHTWNSLELFEVLITLGDSDVPEIRTCVREMLDKAVK
ISAELVHMGLLQVSPWNGTQKEYAAKLLSMFLAGHPNHQLVFMRIWQIDPTYLTTSFRDYYNESPLNITRILDI
LDTLLDVRPFTFALDEYLNLDKWLLDNINTHGAEFLHSVIEFLDFKTASEKTARLADPPEKTMALNPQTITIFL
RILRNNASMLSDEDTSYYFEVRNACLQIHPRLMDQEPGFAVVTYSTEVENEVDSIYKQMYDESITIDQVIQRLK
QAKDSTNPRDQEIFSCMLHFLFDEYKFFQYPPRELAMTGYLFGSLIQHRLVDYIPLGIAIRYVLDALQCPPETN
LFKFGIQALSRFESRLSEWQPLCEKLLQIPHLLEARPDLAESARRAEVSDKILFLVNNLAPSNFDAKVREMKER
FCTEPNNHHLYIRFLEALDRQLLFKFVLHETFVKSANLLNSEKTLTSSSERAILKNLGSWLNKPILHKNLSFKD
LLLEGYDSNRLITAIPFVCKILEGCSKSLVFKPPNPWLMGVISLLVELYHFAELKLNLKFEIEFVCKSLDLDLD
QVPATTILRNRPRGITPQIESILVNLSNMIQISSDLALHTNIAFKRAVQLAVDRAVREIIVPVVERSVTIAGIS
TRELVSKDFASEPNEEKLRSAAHLMARKLAGSLALVTCKEPLKTNLSNHIRHFLTEHGFAEHPIITMIANDNID
LCCAAIEKAAMDRAVADVNENFTPAFDLRRRHREHRFNPNYMAHLPDLLRLKQGGVMPLQGRVYEDFFNRMMND
LDRVLTQLGQSLSSFPPNHELRQFGRTVAVLASPDELALLFSQKVVSHLYKTPLTIGREMYVDILDRLCQEFPK
VAKEAVDWLLTSEDERKYNVPVTIALIRGSLVTIWQEDEQLSKDPRPSLLNFTASFIRECLTTDATRQELALSI
EWFHHWSLLYRESASSEKAFLSFIPQFTSEGYLAEDTTTLFFRICMEASVAAYHQRIATGDDGRAFQDIDAFSR
LIVMLIKYNGDVNADTKKLRYLTKILSIVVLVLVHQHEERGFQQKPFFRFFSSLLHDLHSVESSLHTAYLPLLQ
TICDTFNTLKPAHFPGFAFSWMSLISHRLLMPKLLASDAQEGWHNLHQVLLALLHFLAPFLRAGELPRSIRDVY
QGTLRIFVVLLHDFPDFLTEYHFSLCDAIPPRCVQMRNLITSAFPATLTLPDPYLRNVRMGPIPTTRFDIAAAL
KTYELAPTLEALMLKRGPPSFLQSLKDRLSTYNLSAMNAVVFYIGLSSVAQAKSKSGSPTFVPSDPGVGIVTYL
ATNFDPEGQYHLLGAILLHLRYPNAHTHWFSSLMLHLFIDMKDIRFKEIMTRILLEHIAVHRPHPWGVMVTFIE
LLRNPKYEFWNHEFVRSSPEITLIMENIGHRRLYLQLCFRNVSDLLTVRREILPLALANSKKMDAVDAYAEVVD
PRESIIDIREYDVPYYLRVAIDNDIRVGLWYAVTFIAGQPSFERVKRADPVVMAYDIETTKAPLKFPDQAVDQV
MMISYMIDGQGYLITNREIVSEDIEDFEYTPKEGYEGPFTVFNEKDEVATLRRFFEHVKAAKPTVMATFNGDFF
DFPFLKARSEIHGIDMFLEIGFAIDNEEEFKSRTCAHLDCFRWVKRDSYLPQGSQGLKAVTTAKLGYNPIELDP
ELMTPYAIEQPQVLAQYSVSDAVATYYLYMKYVHPFIFSLCNIIPLNPDEVLRKGSGTLCETLLMVEAYRGEII
MPNRHEDPHGNMFEGHLLASETYVGGHVEALEAGVFRSDIPTHFKVVPEAVQLIDQLDAALTFCVTNYDEVKGK
IQSALEEMRDNPMRFDNPLIYHLDVAAMYPNIMLSNRLQPDSVVDESVCAVCDYNRPDKTCDRRMTWAWRGEYF
PAQRDEFNMEAFPPKRPGDPKRKFVDLPAAEQTALLHKRLGDYSRKVYKKTKDTKVVYRESIICQRENPFYVDT
VRRFRDRRYEYKGLLKTAKKNLDSMAQQDEGKKLILAHKCILNSFYGYVMRKGARWHSMEMAGITCLTGATIIQ
MARQLVEQIGRPLELDTDGIWCMLPGIFPENFTFQVNKGKSVAFSYPCTMLNHLVHAQFTNHQYHDYNKDSGRY
DIHSENSIFFELDGPYKAMILPSSKEEDKLLKKRYAVFNEDGSLAELKGFEVKRRGELQLIKIFQTQIFERFLL
GTTTEECYAAVAEVADRWLDVLFTKADSLSDEELVELIAENRSMSKTLAEYGAQKSTSISTARRLAEFLGDQMV
KDKGLACKFIISQKPIGAPVTERAVPVAIFSAEESVKRTYLRKWLKDNSLANFELRSILDWNYYIERLGSVIQK
LITIPAAMQKVSNPVPRIRHPDWLHRRVAALDDKFQQHKMTDFFPGHFTMWLSMDNDLIPVSLRIPREFYLNLK
QMESYSHEKLVRTLPRSQPCLNLFINLVNNPNVDGVYELKNLNRARDIGFDLKQLERKYIFLFHSSAATAPVHV
FGLFYPTGVHLHIVDPATRRQPTAYHGNDTTALKAISRELGLIENRSFALIISSGKELSYYETQISKLSRFPVF
AMPSNRASHSLDFPWQADVAKKMLSRYLVAGGWLLRTWDQAVYFPLFMADIEFARRLQKQDMVLWWSPNEQPDL
GGSEDDRRAGEELVSPEFHAPGCYSNVCLSISVRNLAVDAVLQSSIVNELEGSGGSTAFDSAQANITLGDSSVS
PSVFAILKNMVKTWLLDKASPADVAVGHFWRWICSTAAQMFEPNLQRFIHGLMRKTFIQLLAEFKRLGSNVVFA
DFSRILLVTSKPPGTAYAYATYITSAVMSHELFKHVDLQSEQYYNYLVYMDHVNCGAIVCKDPLATESAKGLVV
SMDWNIERFLPPAVQQHFTEVVKTFIVDMYKIKQMKQKELEACRTYITQRLTRKMFRAVFPILPGSHLNLTNPP
LEFVKSACAVFGLAKEFSIEVGILKRNLLDLVGVKEFADEAIFRNPCEPLKLPMVICQHCNHIRDFDFCRDADL
FPWKCPECDCEYDRLAIEFALIDLIHRLEANFAPQDLKCGKCKQIRADNVSKHCCSGTYQFTVSPAEFRRKLRT
IVNVATVHNMQKLFEVEHLKYATLATSPNLEVQRSIATIKTVRNVIEYNEQYVTKRLLISIIWAFSGDAKGEWL
PWQSKVPVIEILYSWLSEHKPLLLCGPPGSGKTMTLFSALRKLPDMEVVGLNFSSYRKTPNGVILAPIQIGRWL
VVFCDEINLPAPDKYGTQRVISLVESGGYYRTLDMAWVKLERIQFVGACGRVPLSHRFLRLVMVDYPGEVSLKQ
IYGTYNRAMLKVVPNLRAYAEPLTDAMVAFYLASQKRFTTDVQAHYVYSPRELTRWVRGIYEAIKPLDILSVEG
LVRVWAHEALRLFQDRLVTEEEKQWTDENIDAVAMENFPTINILFSNWTSKNKARLRVFYEEELDVPLVLFNDV
LDHVLRIDRVFRQTTLSRFVAWMNGLSIFQIKVSNKYTGDDFDLLANAEVPGLFEGFTMNPPENGLASRAATSP
ALFNRCVLDWFGDWLPSYTPPINFPIAYRVVNALVHVHQSMHHFNTRLSRRQGRYPRHYLDFLHHYVRLFTEKR
DELERHLHVGLDKLVTQVEELRKSLAIKRKLRRMVADQQEAEQKKAASIEIQAALAEQERSIEQRRAVVLADLA
NAEQLTKPLREFMKKEYLSRPSYNYEVVNRASKACMVAELETSIKRYKEEYAFLIRVQSKVDRSMTLLESLSKT
FETEMSTIVGDCLLSAAFLAYGEWCAHLTDANTKFKPELSLTRLSWQSKSLPSDTLCTVTSFLDESFLKVLESA
LRFGNALLIQDVENLDPILNEIRRTGGRVLIRLGNQDIDFSPSFPSVEFSPDICSRVTSQSLDQVLKVETDLMK

VQGEFKLRLRTLEKLLLQALNESTGNILDDDKVIDTLEKLKREAAETDIIMKEVEEVTAEYIPLAQACSSIFFI
LEQMNLVNHFYQFSFLDIFDYVLHQNPNLKNILMDDLFLVVYQRTSRALDEMELLLESGLQAYARSSMFKGIVP
HLAEHQADWMKFLLLIKCFRPDRLLQSVTGYDASYRVENQEGFTQADQAIAGAARQGYWVLLKNVHLAPSWLGE
KNLQTLNPHRNFRVFLTMETNPSIPVNLLQSRIFMNEPPPGIFLLAWFHAIVQEDMAAAFNTIDIWLNSVAKGK
ANVDPASIPWDAVRTLVKQSVYGGRIDSDFDQKVLDAFVDRLFTPFSYNVDFPEGTKMEHFLAWPSWLSLPPTA
ERRKMRTLVDDDLQHCREWLSSLPETLNALQKRSGDNSDPLFRFFSREASVGKLLVQVRRDLSDVLLSHLTKGT
IPDHWHRYKVTKIPNLGRRLAQLETISQLNSGGLFFPEAYVTATRAVAHRKKWSLETLDLRLDLEQIQDPGLVL
EGASYSGGVHPEISFTLPPLQGDTIDAHFHAIGAKIAQPYLSLSKEMAEDMLVFDVETLPNYSPFAVMACAASP
TAWYTWISPWLLGRIVIGHNVAYDRCRTLEEYRLERPGTRWLDTMALHVAVNGISSHQRPAWGKYRKQKKRWEE
LTSANSLADVAYLHCGIEVDKSTRDDFMTSTPEAIRSDINSYLTYCANDVAVTHRVFQMTLPAFLQKCPSPVSF
AGICSMGSSFLPVDDNWERYIQSADSKYQVLESKVVEALRKLAEEAWKGDVWLEQLDWTPKIPAWYFKLGKSRV
ESLEVTLRSRMAPLLFKLKWLGWPLVHSREHGWTFRVRLLELSFYHEEDETLYFKLPHKDGDTANVGNPLAKTF
IKFAADGTLSSPNESAVEALNINAQCSYWISARDRIMKQLVIWDKWGMILPQVITMGTVTRRAIERTWLTASNA
KANRIGSELKAMVRAPEGYAIVGADVDSEELWISSCMGDAQFGFHGATAIGWMTLEGTKKAGTDLHSKTASILG
ISRDQAKVFNYSRIYGAGMKHAMLLLLQGNASMLPEEAQRLAGDLYASTKGKNTHRDLFGNKFWYGGTESYLFN
KLEQIAMSDEPKTPALGCGVTYALSKQYLPEKFGSDYLPSRINWVVQSSGVDYLHLLIVAMEHLLQRYDIDARY
LISVHDELRYLVKEEDKYRAALALQVANLWTRSMFAYRLGMDDLPQGIAFFSAVDVDKVLRKEVDMSCVTPSQP
VPISPGETLDIAKTLEKTNGGSLNKDGYVSPDCLAHRAKSEYWLDAQSTDVFERIKFLAAILIATILRTPTESL
PDVLSPIETWKWPRSDLHWWIKVLNKFDIVLEEIIRDYDVDKLQVNEFTPLTKKILLSVLAFEKLLLENSTNRK
LFNSYDRLSSLLFSADLDVVVADLQLLLRPAQQYSAQASVSHALNISTSRLQALAKRWPNIRENDLEFSALASK
KQAEVEGLPNEASEVNYTFYRHLGPLAHSSKSAINILADTVESYSVPNEKFELLAKIRVARSLGKGRVKLVTIR
LLAIAVYCLTQNENTAQTSLFLYEPDLIAQVADFLQLDRGIRKTVTDIANPQSTLPNSFVEALIAFVTFIASHA
SGGNMVVGAGLIPLLIQIIENKNALRLPIVSKTMQLVDNISYGFPNAFQLFCNARGVDVVVKRIEEEVNGHLSI
PRSSVLKHTLRTLHRMMQTSGTSEGLRGLIDSSLTKSIKKIIEHRKLFGSSVLPIAIHVMSTFVHNEPTSLGVL
QEAKLPETFYSSVEDHIEPSIEVIQAIINAIGALCLNQTGQEQFTARPNIIPSIFSIFTSDRHLKVLSEKENAV
VFGTAVDELIRHHPSLKDKVFAAISAIFEQMEQKGKDLPEDTENESSYKLDNIIVAFVDVVSRFLEGMFQHTDC
VDRLSRIIVLPCIPYNYACSVMVQLLRVMTEVAPTQTVNKILEFVKVALDDTKEFWSTMDGKSNDEFRKLVSLY
TRVTLLADVFSTYTHGRSALSLVLGSLHRACIWENIVLKGEFNAKALKHLVAEIPLTPFFQCVVKRRNPDTTAK
RQVAGIIDQVAKIVLSHLYSTVMMGLITLMIFEELLWSFTKFGGEELVHVSGGLKVALHLLHILVSSKPLLDSP
QTVLITTKKKETDPDYFEPHNFIVMLRAIVLPFMQELWDAPWLVNAPVSVCKSVVQTMLELMAGENEPRGAATR
ALTRMRNNVAAAAEYLLAQPELNDAREALKETLGRRSLQLLDEHHSIKDFSPSAYEVHEQPLAIRCRLLALVLP
VVPKWLAPHLLVTEALLVAGDEPKPATLPVSDEPCYPAAGDKIFHICLKFLNYPDLPQDELIAVMRLLVYLTRE
PSKARELVKLGGVASLGCQVYVAIIFRHIIEDKTTLDNIMRQEIKRFFTNPRITDVTSFIRNSGGMALRDPKTF
VHTVESMCQLDQSEAMLHYLISELMRVGKYACFLMQCLTELLFSYNSCKMAFVNYTKKKHRPTMLNFLISELTS
FGTIDSKRRVMLCNWAMSVIVALCLHIPKDVPADLITVRRLVLDSINRALRDPLPVESIDERYGRLLALSDLCH
RLLTVKPDGALHMAKLMLEKNYVATLTTALAEVDLNYPNVRTVVTSILRPLEQLSKIAIKMGREETPDLYRNSA
LGIYPESAVHPLLVDRSDIQDFGPLPTQQRWADEAKMANGKFIGDRVNRLSNHVILALLPAALERVTVLINGNP
VDITDTGIDPTFLEALPDDMREEVLNQHFRERRMARQEPTADSQISPEFLDALPPELRAEILQQERAEPVDIDP
ASFLASLDPHLRQVVLMDQEDGFLQTLPKVNVPRDAIQLLDKTGLATLVRLLFFPQKSLLLKIFVNLCENSKTR
GELLNLLLNILQDGTGDVAAVDKTFAQLSTRPNLVVQRSLEALTFIVSANEFASRFFLTEHEIATGLKRSRKGK
GKEKQPQVQYPIVPLLALLDRQTLLKTPSMMDSLAALLATITRPLATLKKIPPLQPPTIPQASLRLIVNILTAG
ECSARTFQSTLALIQHLAFLADVRDTIAMELRARAQDLGHNIYKDLDDLVQVLAAKFSPASSDQAKLLRVLKTI
DYMYSIYESFRFTPLWSRLGDCLDIVEEKPDVEHMATVLLPLIESLMVVCKHVGVQEAIEDLFTSFTDAHRKVL
NLMVRNNPSLMSGSFALLVQNPRVLDFDNKRNYFYQQLRRRREHHGTLQLNVRRQRVFEDSFQYLQRKTGEQIK
YGKLSVRFYDEEGVDAGGVTREWFQILARQMFNPDYALFQPCAADKLTYQPNRASWVNPEHLTFFKFVGRIIGK
AIFDNRLLEAYFARSVYRQLLGKLVDYRDVEWVDPEYYNSLVWILENDPTPLDLTFSFGVTSVVELKEGGATIA
VTNENKREFVQLSAQYRLTNSIKEQLNALLGGFYDVIPKDLISIFNEKEVELLISGTPDIDIDEWRAATEYNGY
TSSDPVIVWWWRALKSFNRDERAKVLSFATGTSRVPLGGFGELQGVQGVQRFSIHKAYGDSDRLPQAHTCFNQI
DLPQYSSYEKLRQQLLLAVNEGGEGFGFAKSSASSATRKKHAKKLRKLAKKDTVTKGRALEELERFPALCVHPS
RRLRQLSAALQTLLLGAWCLSAWDVDRSVSHRASRSWFITTTIMDPSAAYTALVPPEPLEDRNARLRMSALGAL
RWGTGQPIVRRAAWALVNALMATAVLRSAFVESDITVRGSMWEALLLFLTKVKNAWAYTEFLAFLALGSPVQGY
PTVLVILSTLPRKIFDALWSAIDGQALAFLAAWSECLVWVAGRPNLFVLGFVLPPTPEEFRALLNATSTNPLDP
TLAVIEPLDSTGYSPYARAATGLLDRQLARANLWALQYVLTLDSVRDSLVLHTVLQHVLPNTSKEEADGWIELA
REIEGSAHQTSLAVALAVAEFAPEPPRLDRYRNELASKISGVSGYRFLRSLNAIAPDPESDVIFMPQQRAVFLV

QALQGWMGSDIDEGVEVEVTKVLADIAPILLGLQGAHWEFIMDLVESNIENCSFKDLDTLVLLSRTLRLVSVIM
DLAKTNKYLREWGDRSKNVLTLVRDLSIPRSICREQALSIEHLVIEAGVDTASLKVKTAYVEQLRNLDLIGTYF
LPNVFDILGVGGPFKLDAWGIDEYWLPMYDDQSLSLLAAHVYYRALLTVPSLVRTWWEGLKDRQLSTAISTFTS
SYFSPVLIASEFSQIKPTVTEKVWRRWLFAVQQVHNGRIVDGLTLFKKTVSLHFEGQVECAICYSILKPCKTCK
NRFHAGCLFVSAPKVLDMD TVKQRRIRQF GLEDCPTFYP TEEEFSDPLS YIRSISDRAQ QYGICKIVPP
EDWNMPFVTD TEFRFKTRVQ RLNSIEAASR AKLNFLEQLY RYHKQQGNSR VSIPTINHKP
LDLWLLRKEV QNLGGYDVVK SKRWGELGRA LGYSGPGLSA QLKNAYTRII LPYENFYNHV
RNSLCDGCDC GFHIYCLDPP LQSIPKGQWY CHTCLFGTQD FGFDEGEEHS LASFQARDLE
FRRRWFATHP PISENNVEEE FWRLVQSPFE TVEIEYGADV HSTTHGAMPT LENQPLDPYS
RDGWNLNNIP ILSDSLLRYI KSDISGMTVP WTYVGMVFST FCWHNEDHYT SSINYMHWGE
TKTWYGIPGD DAEKFEAAIK KEAPDLFEAQ PDLLFQLVTL MNPARVREAG VRVYACNQRA
GEFVITFPKA YHAGFNHGFN FNEAVNFALP EWLPYGRSCV QRYQEHRKLP VFSHDELLIT
ITQQSTSIKT AMWLYPNLQE MHQREMQRRA ILRPNMEEVL VNHDSEEQYQ CAICKVFCYL
SQVTCSCVVC LDHADNLCND RVLRKRFSDE YLTDILQKVA ERAAVPIAWN NKLHKLLEES
PKPQLRSLRA LLAEGERINF GLAELPMLRK CVLKGNEWVD SANVFLARKP NRKRDRPERG
LDDLYTVLEE VTHLGFDTPE ITALRTMAGQ AEDIKAKART LLCDTLLAQG SSLNIYLEEM
AKVERISMRS KLLKELLEEV RALVSRARAC DLQEDNQSMK SLEDKLRVGD EWESKATAIL
ARPQKTIEEL PVDATTHDHL TNVLARAKEF EEQAKAWARP GLAKVTDALR LIARSEKEFS
IPAVDELKET ANTAYELEQR CENALKNNYQ HETPFDAMRK WQSYAQDKLA YSLPNVEKMA
RQLELHDQWL KRLPWYCHGQ QVMEDVLECT KPEDDNPPND EYFTCICTVP VRPPPPGQAS
DAVQCDHCYA RFHGACAANG GSCPFCDHHH WNGTIHKERS YHSCFLPTIL LGAPEITKYY
SLAWKHLNII VDRVERLTKV VGNFLQFAAN QRPEFIPRVR HYMRKLFKIQ FAVSPNPEVS
YGLDLAGLHR ILASQRLKKR RRPKFLLAPD ICKEGKDGTR CVCRGLKCKN TYHVTCVYLC
PLCCIRKGRN YPYADLRVKT FSRDPIKFRL PPSTGLVIPV TLLRFFP
>Sistotremastrum niveocremeum
MRRTGQDQGILISGESGSGKSESRRLIIKSLLELSVSNPGKKGSKLSNQIPSAEFVLEAFGNARTLSNPNASRF
GKYTELQFTRGRVSGVKTLEYYLERSRVTGAPSGERNFHIFYYLIAGASTEERQHLHLHDKQAFRYLGQRDGLR
FEQLKMAMKSVGLSKRSVAQMCQLIAAILHLGNLEFTIDRHRNLDAAVVKNVDVLDIVADFLGVQPSALEAVFS
YKTKLVKKELCTVFLDPEGAAENRDALAQTLYSLLFSWVNENINQRLHRDDFSSMIGLLDLPGPQNISRSNSLD
QFCVNYANEKLQNWIQKSLFESHVEEYSAEGISNYVPQVPYFDNSECVRLLENRPGGLIHIMDDQARKMPKKTE
HTMVEAFGKRWGNHSSFKIGNMDRFPSFTVNHYTGGVTYSAEDFLEKNLAALNPDFVSLLRGGSINPFVRGLFS
SKAIATQAHPRNEDTIVAAQQPVKPMRAPSTRRKGTACKAGEFRAALEILFQTFDETQPWFVFCLNPNDSQLPN
QLEGRGVKAQIRSIGLSEIARRNRTVFEVSMTPSEFCERYRDQLALLGVEDGEWDLVQGQYKVFLSHLAFHKLE
DHLRATDVEEQKRNRLRDAEASSQQLPLVANAAPFEDFDGVSRYTSHRESYAPSRNMFQNADGEIQEGEVTEEI
RDTSTRRRWLVLVWFLTFWLPNPFLSWFGRMKRLDVRQAWREKLAINMVIWFICGCAVFVIAVLGNVICPREFV
FGTDELASHSNHIYTAIRGEYGGLDATDIFPVQVSALCDGTDGNLSPYIVLDATNTDVNAQYHDFRVSTTDPRP
DWYWENMVILRYHYRVGFMGYTPSTLRDMASAGLAVGVYDGLSTPAVMFTSTSSTFITFKPEESLQFTLLTIGS
RGDVQPYIALAKGLIAEGHDIEFTYVGGDPAELMRICVENGTFTVAFLREGLSKFRGWIDDLLQTSWDACQNTD
VLIESPSAMSGIHIAEALQIPYFRAFTMPWTRTRAYPHAFAVPDRKTYVMFDQVFWRGTAGQINRWRKNVLGLA
PTNLDKLEQHKVPFLYNFSPTVVPQPLDWYEWIRVTGYWFLDDADVSAKKWTPPTDLIEFIDEARQKNKKIVYS
DPDAMTRCVVEAIVESGVYAILSKGWSDRLGPEAPLPPQIYKISSVPHDWLFAGIPTIIKPFFGDQFFWADRVE
ALGIGSSVRKLTVASLTAALLSATTDRKQIDRAKIIGQAIRSENGFDPRIVFEALKLACETRSEKLMVASLDCI
SKLISYSFFSDLAELITHTVTSAYTETTPDAVSLQIVKALLALVLSPDILIHHSSLLKAVRSVYNVFLLSQDPV
TQMVAQGGLTQIVNHVFELFVKDAFLVFRALCKLTMKPLATESERDIKSHAMRSKLLSLHLILNVLNSHMSVFV
DPTSIIYSGSSGEATQFLQATKQYLCLSLSRNAVSSVPQIFEISVEMLWTVISGMRTKLKKEIEVLLTEIFIPI
LELRMSTLRQKASILSLLSRLSQDPQALVDIYINYDCDSEALENIYERVINIISKMASPSLSTSALSMPGNGAV
LSESLLKRQALECLVSVLRSLAAWGTDDPDRFQNAKQRKTTLLAGIKKFNFKPKGVAFFIDAGFISAREPEPIA
KFLMSTDGLSKTMIGEGEEANKAIMHAFVDLLDFSDLSFTDALRMFLQAFRLPGEAQKIDRFMLKFAEKYIEGN
DAAYVLAYSTIMLNTDAHNPQVKNRMTKLDFVKNNRGINDLPEEFLGTIFDEIQSNEIRMKDEVANVGRDFQKE
AYIMQSTGMLSHQFFSASHYHHVKPMFEVAWMSYLAGLSGPLQDTNDLEIVELCLDGFKSFVTTLAKFTFLNNL
GEMKTKNMEAIKTLLDVAVNEGNQLKGCWRDILTCVSQLERMQLIGGRRQRKPPAEELAAESRSTHITVAADMV
FSLSHYLSGSAIVDFVQALSDISWEEIQSSGMSETPRLFSLQKLVEISYYNMGRIRIEWSNMWAILGEHFNMIC
CHNNIHVVFFALDALRQLAMRFLEKEELSHFKFQKDFLKPFEYTMIHNTNNDARDMVLQCLQQMIQARMQNMRS
GWRTMFGVFSAAARANNERVTTTGFEIVTRLNEDHFETVVQNGAFADLTVCITDFCKASRDEPTIKFWYPVLFG

FYDIIMNGEDLEVRQMALDSLFSTLKKYGTNFPADFWDTICKELLFPIFAVLKSTADVSRFSTQEDMSVWFSTT
MVEALRNVIDLYTHYFDLLERTLDGLLELLSVENDTIARIGTSCFQKLLESNVKKLSKSHWERVVSTFVKLYKM
TTPHQLFDENLRVERRRVFQQIVVKCVLQLLLIETTHELLQNREIYETIPPEQLLRLMGVLDHSYQFARSFNDD
KELRNGLWKESASASTLVNVLFRMYFDTRPDHQASKQLVADRLLPLGLGVIQDFNKLKPESNMKSIAAWSPVVA
VILHGFCGFEDQGFKRYLPAIYPLVTDILSREPDIRLAARTFFVRVGKLQNLQRQALIVAARTKYGAEIVNPIL
HQIFPNLSLRPGTDLVQVLNELGPEITSDVDTVRALFRRYGLTDSSPPNDAQVAEIISSMSRHASEGGQLCDVN
ALVRAIASFHTSLNWPALVHVFDRPDRGVDTATLKLLISILLNAPREEPHAVTGFWMEWRNSLHQLRLLDALLS
LPGDTFNFVLVPGRRVVTVDDVAGASPTIKALAANVQGQTWNSLSLFETLIHFGNSDQAEIQACVRDMLDKAVK
ISAELVHMGLLQVPPWNALQAEYSGKMLAMFLAGHPNHQLVFMRIWQIDPTYLTTAFTDFYHDNQLNITRILDI
LDTLLAVRPFTFALDEYLNLDKWLADNIADHGAEFLRAVIEFLDAKTASEKETRVTEPAARTMALSPQTIAIFL
RVLRNNSSVLAPGDVDYCLEIRNACLQIHPRLMEQEPGFSVVSYSAEIEKEVDSIYKQMYDENISIDQVIALLQ
RSKTSKNPRDHEIFSCMLHFLFDEFKFFNYPDRELHMTGYLFGSLIQYNLVDYIPLGIAIRYVLDALQCPPDTN
LFKFGIQALSRFETRLPEWQPLCQALLRIPHLLEARPDLATNLRRAEVSDKILFIVNNLAPSNFESKVDEMKER
FSTEPNNHQLYLRFLDSLSKPDLHKLIVHETFVKSAALLNSEMTQKSTTERTILKNLGAWLNKPIKHKNMAFKE
FLLEGYDTNRLIVAIPFVCKILEACAKSTIFKPPNPWLMAVIALLVELYHFAELKLNLKFEIEVLCKTLDIDLT
RIDASTVLRNRPRVVGSHIEGILGNLSNLVVINAQLALHTNHSFRRAVTVAIDRGVREIIVPVVERSVTIAGIS
SRELVSKDFAMEGSEDKMRKAALLMAQKLAGSLALVTCKEPLKSNLGAHIRHALTEQGFNEQQVIGMLVLDNIE
AACAAIERAAMDRAVMEIDDALAPAYEMRRRHREVRPGLEFLAHLPDPLRIKPNGLQLPQWRVYEDFFVVMTHE
LEMLVTQIPQPLSALPVAHEVKQLIRQILFCADRDKVALQMSQRIVQHLYKTASQLGREAYVTILDQLCRSFPE
VQKEATEWLIYAEDERKYNVPVTVTLLRSGLIPVAQQDAQLAREARPSLIDFTANLIRECLAGDATQAQFQDSL
SWFQQWVMIFQRSPSPEKAFIPFVSQLQKEGILKQDDSFLFFRICTEASIESYTGAVTGEDPEAAFEAIDAMSK
LIALIIRYHGEANNDQLKAHYLTKILSIVVVVLAHAHEEQGFQQKPYFRFFSSLFNDLHAIEPHVGSAYFPLMI
ALSDTFSSLQPIYFPGFAFSWMCLISHRLFMPKLLSSESREGWSAFHTLLVSLFKFLGFFLNALDLKDAARDLY
RGAVRILLVLLHDFPEFLAEYYFSICEAIPPKCIQLRNVVLSAFPMHVVLPDPYLRNLQIGPIPPILSDFTSAL
QPGELRTYLDHFLLNRGTTSFLPSLKERLEKYNLSLINSLVMYIGVSSVAQAKARSGSAVFVASDPGVVILQYL
AMNLNVEGQHHLLSSMVLHLRYPNAHTHWFSSLLLHLFVEVKDEVFCELMTKVLLERFIGHRPHPWGALVTFIE
LLRNPKYEFWSREFTRIAPEITMLLEGVGHRRLYLQLCFRNVSDLLTVRRDLLPLAQANAAKIDAVDAYAEVVD
PRDAIIDIREYDVPYYLRVAIDQDIRAGLWYAVTFVDGKPLFDRVKRAEPVVMAYDIETTKAPLKFPDQAIDQV
MMISYMIDGQGFLITNRDIVSEDIEDFEYTPKDGYEGPFTIFNEADEEAVLRRWFEHIRTARPTVMATFNGDFF
DFPFVAARAKVHGLDLYLETGFAIDSEEEFRSRTCVHMDCFRWVKRDSYLPQGSQGLKAVTVAKLGYNPIELDP
ELMTPYAVEQPQVLAQYSVSDAVATYYLYMKYVNPFIFSLCNIIPLNPDEVLRKGSGTLCETLLMVEAYRGNII
MPNRHEEEHGNMFEGHLIASETYVGGHVEALEAGVFRSDIPTHFKIDKTAAQLIDQLDAALKFCVANYDEVKQQ
ITEALEEMRDNNIRSDKPLIYHLDVAAMYPNIMLSNRLQPDSVVDESVCAVCDYNRPGKQCDRRMKWAWRGEYF
PAHRDEFNMEQFPPKRPNDPKRRFHDLSPAEQSALLHKRLGDYSRKVYKKTKETKVIERESIICQKENPFYIDT
VRRFRDRRYEYKGLLKTWKKNLEPIAEIEEAKKLTLAHKCILNSFYGYVMRKGARWHSMEMAGITCLTGATIIQ
MARQLVERIGRPLELDTDGIWCMLPSIFPENFKFKLKSGKSIAFSYPCTMLNHLVYDGFTNHQYHDLDPDTGEY
KIHSENSIFFELDGPYKAMILPSSKEEDKLLKKRYAVFNDDGSLAELKGFEVKRRGELQLIKIFQSQLFERFLL
GTTTEECYAAVAEVADRWLDVLFTKGESLEDDELVELIAENRSMSKTLAEYGAQKSTSISTAKRLAEFLGNQMV
KDKGLACKFIISARPMGAPVTERAIPVAIFSAEESVKRTYLRKWLKDNSLANFELRSILDWNYYIERLGSVVQK
LITIPAAMQKVSNPVPRIHHPDWLQRRVAAMDDKFQQHKMTDFFHGKFIMWLSLANELVAIPLRIQREFYINFK
TSPDYTHEAVTRTLPQNRPCLFLFMHLTSNSDIDGIYERQTLNRARNVGFDLQQLETEPVFLFHASSSDSRIHV
FALFDPVGARIHIIDPSSQRQRTVYHGSLSTAFKGLTRELNNLENQSHVLVISSHKDVDYYEANISNLIKFPVF
MMAGPKKAHSLDLLWQKDVSKKMFSRYIFLGRWLYELTEKAKYFSLSCQDLDFARRLVKQDMLLWWSPLPKPDL
GGVEDLNTVVEEPKNPEYCTPGLYTNVSIAVNMYNLAINSVIQSSLVNEMEGSGGTTAFDAPQTALTLGESATS
VQVFQTLKTMVKTWVIDAHGPAELSVEHFWRWLCSSASAMHDPSLQRFVHGLMKKTFYQLLAEFKRLGSNVIYA
DFNTIWLVTSKPPGAAYAYSTYITTAATSHDLFRHILLRTETYYNFLLFLDNANYGAIVCENPEEDKLPEGLPL
AMSWNIAAFLPPAFQPHFRQAVAFFIVEMAMIKTRKIKELEVSQAFIAERLTRKLLSMVFPILPGSHINMEDPA
FEFVKFVCAAFSPSKDYGNELGILRRNLLEIVGVKEFNDAAIFKNPCEPFKLPMVICQFCDHVRDFDFCRDQEL
MTWLCPMCDGEYDRLAIEFMLVSCVDKIIELFGAQDLRCHRCKQSRAENVSRSCCSGHYELTVNKVELRRRLRT
IVNISIVHGLDKLMSVDHKRVAHFIGTKVLIANNGIAAVKEIRSIRQWSDTFGTDRAIEFTVMATPEDLKAVWA
GWGHASENPRLPESLAASKHKVVFIGPPGSAMRSTIVAQSADVPCMPWSGTGISDTVQSENGFLTVPDAAYAAA
CVTSAEQGMERAAKIGYPIMIKASEGGGGKGIRKVDAPEAFKNAYIAVCGEVPGSPVFVMLLADQYGSAISLFG
RDCSVQRRHQKIIPVTIAKREKFEEERAAVRLAKLVGYVSAGTVEYLYSPAEDVFYFLELNNMVVALKELSIRG
DFRTTVEYLIKLLETQVFEENTTGWLDTLISNRLTAERPEATVICGAVTKAHSEECWTEYKRVLDKGQVPARDT

LKTVFGVDFIYEGDGKSHSVYWREEVGSVRLMVDSKTCLIEQEAYAEIEVMKMYMPFEGQLPKMGLPSVVGSKP
HQQLNFGIDILNNILDGYDNQAIMASTLKVVEVLRNPELPFSEASAVLSALSGRIPGKLEDSIRATIENAKTKT
NPVETMFRASLAPLVDVVDRYKSGLKVHEVIANLLSRYEATEKLFGGSIEAVVALVLSHTKAQSKNKLVLSLLD
MIKQMEQVLRASVTATYYGEPGGSHQLPSADILREVLAAFEVYVRRAYRAYELLSRRQGSVSDLTYLLGQNIAS
FPDFKSLTNGFAKVAASLPLFNQAPNVLNLALRIFDKEDDMPEEEWRQKFLTLLNDSKAGVWQEEEAIRNIEPA
LAFQLELSRLSNYNLTPCFVMDSRFFVRALIRPGRIRGNMRMSEVSAQYRHSDCNHIATVTFDEVVEAISGFIE
RHGKRLWRLQVTGAEIRIALEDSEGNVTPIRCVIDNVSGFIVNFHKGSTILKSIGEKPLHLQPVNQPYPTLQPK
RYQAHLVGTTYVYDFPDLFGKALHNHWLARRVLDPSVKIPQTLLESKELDENEELQEVDNDITYKIGSFGPQED
HFFYAASQYARSRLGVECLKGSGLIATSRAYDDIFTITLQRAVQVEGQPIILTKVLGREVYTSNLQLGGTQIMG
SDLEGVAHIIQWLSYVPASKGTSLPVAISWDRDITYVPPKGPYDPRWFIEGKVDDKGSFQETLSGWAQTVVVGR
ARLGGIPMGLIAVETRTIERVADPANPTSFEQHIMEAGQNSAYKTAQAIFDFREGLPLIIFANWGAWVVLDPSI
NSAQMEMYADVDRDKVLGLMERLDPSYASYKKDSKDAAQASEKLVARETELQKQIALLYADLHDRGRMEAKGCA
KPTVWKDARRRFYWNLRARLAQESVLKQLQSAASERLESLVTSSSSDARAYAEELEQLDLKPTIVDLQADKAAL
HSLVHVFGRLPDISFDLPPLQGRTLDEHFWNVGAESAQPWLGKAKNFADDMLVFDVETLPNYSPFAIMATAAST
SAWYAWISPWLLGRIVVGHNVSYDRARIREEYHIQRTTTRYIDTMALHVAVKGISSGQRPSWIKYNKAKKRWED
LTSANSLADVAKLHCGIEIDKTVRNDLMTHSPSQILDDIRTYLSYCATDVSTTHAVFTRVLPAFLTACPNPVSF
AGVLTMGSSFLSVNGTWKEYLKAAENKFRELEESVKDALKELAEVAWKDDVWLSQLDWSEKWPKWYWELAKPRK
GSIDVTVRNRIAPLLLQLKWRGYPLFHSREYGWIFRVRQRELSFYEDADLHLFYKVPHKDGEDANVGSPLAKPF
MKYLQDGTLTSPSSVAKEALDMNAQCSYWISARDRVLNQMVVWDSWGMIVPQLITMGTVTRRAIEKTWLTASNA
KSNRVGSELKAMVRAPPGYAIVGADVDSEELWISSVMGDAQFGLHGATAIGWMTLEGTKAAKTDLHSKTADILG
ITRDAAKVFNYSRIYGAGMRHAQLLLLQNSASMVPEKAKKLAEDLYAATKGKNTHRDLFGRKFWFGGSESFVFN
KLEEIALSDRPQTPALGCGVTYALSKEYLPNEFGSDYMPSRINWVVQSSGVDYLHLLIVAMEHLILRYGIQARY
LISVHDELRFLVKEEDKYRAALALQIANLWTRSQFAYRLGMDDLPQGVAFFSAVDVDTILRKEVFMTCETPSHQ
HKLEPGESLDIQQILEKTDGGKLGDVVYIEPDCLVHRAPGPEFLQAQATDQFNEIKALAASLISTLLTTPEDGI
ADALDPIATWNWPRSDLHSWIKVLNRFDGILENLVNIHKVDQLQYDTFTAAEKKTLLGILKFEKLLLENSTNRK
LFNSYDRLNCLLYTSDLDILLSVLQLILRPAQQYSAQPTVSAALKISTSRLQALAKRWPGLREQGIELADLVSA
KIAEVEALASQINEVNFTFYRNLRPFLHLSKDASSIAAEISSTANVPEERFELLCRIRTSQALAPARVKLVIIR
LLALSIFAHTHNESEAAATLFLYEPDLVPHISDLLQLESQIRHTVSEIAKADAVVPQSFVEALLSFLSFLATHT
SGGNLVVGAGLVPLLIQVIENRVAGRIGIVSKTLTLLDNVLYGYSNAFQIFVNSRGVETMVQRIEYEVDEHLAT
SRSALLKHLLRSIHRMMQSSGTSEGLRGLIDSSLPKSIKKIMDNRGLFGVAVLPLAINAMATFVHNEPTYLPVI
QEAGLPTSFYDAVEGGLEPSIEVIQATTNAIGALCLNQAGQDQLTARPSIIPSVISVFTSEKHLKVLQDKENAS
LIGSAIDELIRHHPSLKTQVFDAIGAMLDRIESLGNTWSEKEGKEHLYRLENIIVSYLDIVGKFLESLFQHTDG
LERIGRLIALPCLPYDFATSLLVQVIRTMVEVSPSSTLSSLANQVKTSLNETREFWSVPGGQSNLQFRKLVTLH
IRIALLSDIYQGYAHGRSVNSMLLGTLHRSFIWENLVLKDGLNARALKHLATHIPLAPFFQAIVKRRNPDEAHK
KQIHATSSLVAGIMVEHLYGTVVISLVTMLLFDELLLAFNRAGGKKLLLALSGLKVALHLLHSLVSARPLFDSG
QTQFLVTRKKDTDPEYFEPHDFLVKMRLAVLPLVSELWRADWLPSIALGVAKEVVQTLLVIIGGEREPRSAAER
ALIRSHNNINAATEYLLAHPELDLLRKPLKADLGRVALTLVDEQPGIRAFSPTADDLHEHSLTVRFRLLALILP
TLPKWLASHLLVAQSVLMLGEGLRDVSTPLFVGPSFEEPRSILLDVGIRLLKISTLSSDELLADLRLLVQLTRD
HHSALVFVEREGLSLLALQVHLIILLRHIIEDKTTVISLMRHEIKRRFAHPQVLEATNFVRHTSALVLRDPEAF
LEASKATLKLAQIESVTQFLITELYRVGKYACFLMQCLTELLFSYNSCKIAFVAYSKKKHRPTLLNFLLVDMIS
HQPFSTQKRGILCNWAKSVILALCVDVGKDSSGEITSVRKFVLDGLSKAIKESSTSELLEAHYGKLIALSDLCH
RLLTVRTETPLHMAKLMLEKGFVSTLTSALNDVDLNYPNVRTLVTTILKPLEQLTRVAIKMGRDDTPDLYRNSS
LGMYTDPTSHPLLVDSGDRPDFQPLPTVLRWSEESKITHGQYATDRPTRFINHLINALLPAARERVTVMIGGNP
VDITDTGIDPTFLEALPDDMREEVLNQHFRERRTERATQAAESHISPEFLNALPPEIRAELLDQEAEQPTEMDP
ASFLATLDPQLRQVVLLEQDDGFLQTLPAIPSHREAVQLLDKAGVAVLVRLLFYPHKDTLQKILVNICENAKTR
AEVLNLLLGVLHDSSGDVASVDKSFAQLTVRPHLVAQRSISALTYIVSSNDLSSVFFLTEHELPPGMRKSRKGK
TKERQSQTHFPIALLLGLLDKKVLLKTSTMIESVAALLATVTRPLATLKKSLLQHPPQISEQILRLVVNILTVG
ECSSKTFQHCLSLIQNLAYIPNAKDTVASELRAKAQDLGRSILRDLEDLVILLSTKFSPASADQAKLLRVLKTI
DYIFTIYDSFNFAPLWKELSACLSAVDDSGNVETLATVLLPLIESLMVVCKNVDSKDTAHDLFLSFTDAHRKAL
NLMVRNNPSLMSGSFALLVNNPRVLDFDNKRNYFSQQLRRRREHHTTLQLNVRRARVFEDSFHYLQRKTGDQIK
YGKLSIRFYEEEGVDAGGLTREWFTILARQMFNPDYALFQPCAADKLTYQPNRASAINPEHLSFFKFIGRIIGK
AIYDGRLLDAHFARSLYRQILGKPVDYRDVEWVDPEYYNSLIWILENDPSHLELTFSFGENKVVDLKENGSTIP
VSQENKREFVQLSAQYRLISSIKEQIDALLGGIYEIIPKDLISIFNEQEVELLISGTPDIDVDEWRAATDYNGY
TSSDPVIVWWWRALKSFNREERAKVLGFATGTARVPLSGFTDLQGVQGNQRFSIHKAYGDPDRLPQAHTCFNQI

DLPQYTSYEKLRAQLLLAITEGSTGFGFAKSSATSSTRKKHAKKLRRLAKKDNVTKRRALEDLQHLPILLLHQS
RRLRLLASGLHTSLLGAWCMAVHDIDRQVAIQARKSWFLRESFFDPEVVYNRLNPEEEEADRHARLRFGALGSL
RWGIAAVPVRKAAWSLLQSLVGPAVVRSAWIESDAIVRSGMWEPLVVFLSKFPQSWAYREFLEFLELGSPLQGY
PTIMIVISTLPSRIFTSFWAAIDGRALAFLSALLECVLFIVAKPDVYLLGYLMPPAEKDLNSLLNAGSPVVVDS
TASVLDPFDSLGFSEYSRIVMAILDRQLIKENIWSVRHVMILNNARDALVLLQIMQLVLSDVSKASADLWVQLA
RSIEAKSSETSLAITYALTRAGPEPPRLDRLRNELASTLAGVPGRRLLRRLAASAPNPDGDVVFLPQQRAIFLV
QTFQKWIASDLDIGVENLMTLMFFYLAPILQNLPGSHWELIFDIMENNLENASFEDDSTLFALFWTLRLIISLE
DLTASNKALKAWVERRSTMLRFVRDLSEPREKCRELLLNAESLVVEAGVDTTSLKVKMAYLDGLREAEVIGNNL
LPVIFDLLGVSKPLNLSPWAVDEYFIDLYEPLSNQLLASHIYYRVLMTVPSLVRSWWLECTDRQLTSSVSSFTS
GSFSPVIIAQQLSHIRQPVKEDRWRAWLFAVQQVQNGRIFDALSLFKKNVSLHFEGQVECAICYSIIKPCKTCK
NRFHASCLYVTVPPDLDMK SLKMESPRPF GLEDCPTFRP TPEQFKDPMA YINSISATAK DFGICKVVPP
AGWKMPFVTD TKFRFKTRVQ RLNQIEAAAR AKVNFLEQLY RYHKTQGHSR MSIPTINHKP
VDLWLLRKEV TKLGGYEAVR NKKWGDLGRL LGYTGPGLSA QLKNAYTRVI LPFEQFSEQV
RNSLCDGCDC GFHIFCLDPP LTAIPRGQWF CTTCLVGTND FGFDEGEEHT LASFHARDLA
FRKLWFERHP PVSESDVERE FWRLVQTPNE TVEIEYGADV HSTTHGAMPS LETHPTEPYS
RDGWNLNNMP INPDSLLRYI KSDISGMTVP WTYVGMIFST FCWHNEDHYT YSINFMHWGE
TKTWYGIPGD DAEKFEAAIR QEAPDLFEAQ PDLLFQLVTL MNPGRLREAG VRVYGCNQRA
GEFVITLPKA YHCGFNHGFN FNEAVNFALP DWLPHGRACV QRYQEHRKLP VFSHDELLIT
ITQYSQSIKT AVWLYDSLKE MVDREFRRRE EIRPGITEVH DDSDTEEQSQ CSVCNVFCYL
SQISCQCGVC LDHSDELCSC RTLHLRFMDD ELTDILSKVE ERAMLPKAWQ AKLQKTLGES
SRPLLRLLRA LLAEGERINY RLPELPSLRK FVQKANEWVE SVNNMTVRKR KRPREKPERR
LSDVYQLLDE VDGLGFDCPE IALLRGLASQ AEDLKTRSQR LLCDTVISEA SSMNIYLEEV
QDLERIVMRH KLLQDMLDEI RNLLARARAC ELPADNQYMK VLLAKQNAGD DWDQKAARVL
AQPLKTIEEL PVDPAVLDKL RAIRAKAKDF ERQALQWLHP DLPKVQEAIR LVTRAEKEFS
IPAIRELRRT ADFAADLEQR SEAVLKNRYQ NSQIFDTMRK WGEYAREHLQ FQLPNFTKLE
AQLESHELWM ERLPWYCHGQ PVLDDVLAST KPDDDTPPQD DFQTCICFEP VRPPPPGVTS
DAVQCDHCYA RFHGPCVLKG GSCPFCDHHH WNGTLHKERT YHYCFLPTIL LSAPEITKNY
SPAWRQLETI VSRVDRLSQL ISAFLAFAAN HRPEFIPQVR HYMRKLFRIG FAVSPNPEVS
YGLDLAGLHR ILASQRPKKR RRPKFVFGSD VDKDWEDGTR CVCGGKKCHR IYHEPCVLQC
PLCSLKKLKP YPTAEIRVKK FSKELLRVQL PPPTAPTISI ELIRFIP
>Auricularia subglabra
MRRTGSDQSIILSGDTASGKSENRRLAIKSILELSVSNPGKKGSKLAVQLPAAEFVLESFGNARTLFNANASRF
GKYTELQFTRGRLCGVKTLEYYFERNRAAGAPSGERNFHIFYYLLAGASPEERQHLKLSEKTTFRYLGQRDALR
FEQLKGALKNVGMSKRHVAQACQLVAAILHLGNLDFTIDRHRNEDAAVVRNTDVLEIVADFLGVDSAALEAVLS
YKTKLVKKEVCTVFLDPDGASDNRDDLAKMLYSLLFSWLNESINQRFCRDDFSTFIGIFDLPGTQNLSRSNSLD
QFCVNFANEKLHQFIQRSIFEKHTDEYANEGISRFVPSVPFFDNSECVRLLCNKPGGLIHIMDDQARRMQKKTD
HTMVEAFGKRWGNHSSFKVGAMDRFPTFTINHYNGPVTYSSESFLERNLDALNPDFVSLLRGGSINPFVRGLFS
SKAISTVAHPRNEDTVVAAQQPQKPMRAPSTRRKGTPCAAGQFQNAMETLIQTLDDTQPWYVFCINPNDSQLPN
QFEGRSVKAQVRCAGLPEIARRAAIVFEVSMTPAEFCVRYKDPLVALNLSEGEHDVVLGQFKVFLSHAAFHKFE
DRLRATDVEEQKRNRLREMEASSQALPLVAHAQPFSDDDGQSRYTSHRESYAPSRNMFQNFEGEVMEGEVAEEI
HDSSARRRWLALVWLLTWWLPTPFLSWFGRMKRMDVRQAWREKLAINLIIWFLCGCTVFVIAVLGNLICPREYV
FNADELSSHAKHVYTAIRGEYGGQDIARLFPVQVSALCNGINGRVSPWLTLDPVNIATDSQYHDFRAWSSDYRP
DWYFEQMTVMRYKYRVGFVGFTNTVLRDMAGAGRIVAVYNNITVPAVMFTSTSSTFLTFKPQESLRITLLTIGS
RGDVQPYIALGKGLIADGHGMEFGYVGGDPAELMRICVENGTFTVGFIREGVQMFRGWIDDLLKTSYEACKGSD
LLIESPSAMAGIHIAEALKIPYYRAFTMPWTRTRAYPHAFAVPEHKTYVMFDQVLWRGTASQINRWRRRLLGLP
PTNLDKMEQHKVPFLYNFSPAIVPPPLDWYEWIRVTGYWYLDDADVSAKKWQAPQDLLDFIASARDAGKKIVYP
DPTSLTRTVVDAIQQSGVHAILSKGWSDRLEPEIQLPSSIFPIASVPHDWLFAGIPTIIKPFFGDQFFWADRVE
ALGIGSSVRKLTVESLAQALHAATTDEKQIAKAAIVGQQLRAENGDKPREIFEPLRLACETRNEKLMIASLDCI
SKLISYSFLVELVDLVTHTITSCYTESTPDAVSLQIVKALLALVLSPVTLVHHSSLLKAVRTVYNVFLLSQDPV
NQVVAQGGLTQIVNHVFDLFLKDAFLVFRALCKLTMKPLNTESERDLKSHAMRSKLLSLHLVQTILSTHMNIFV
SPSSYIYSSSSRESTPFIQATKQYLCLALSRNAVSPVPQVFEISVEIFWRVLSGMRKQLKREIEVLLNEIFIPI
LEMRNATAKQKGVLLNMLSGLCQDPQALVEIYLNYDCDKDAIDNIYERLMNVISKIGTPSLSTTALGHQPDAAH
QNEVNLRRLSLECLVFVLRSLVAWGTDDPGKFESAKQMKTTLNEGIKKFNFKPKGIEFFLDTGFIPSNTPQDIA
RFLLETEGLSKAAIGEGDDLNVAVMHAFIDMLDFTELNFLDSLRLLLQSFRLPGEAQKIDRYVLKFAARFMECK

DAAYVLSYSVILLNTDAHNPQVKKRMTKTDFLKNNRGINDLPEEFLNEIYDDIVHNEIRMKDEVLSLGRDLQKE
AYALQSSGMANDQYFSASHFVHVKPMFEVAWMPVLAGISGPLQDTDDLEIVELCLDGFKAFVTTLAKFTFLNNL
GEMKSKNMEAIKTLLDIAVTDGNQLKGSWHEVLTCVSQLERMQLISSALSRKALKEELANESRSTHITVAADMV
FSLSHYLSGTAIVDFVQALSDVSWEEIQSSGMSQHPRMFSLQKLVEISYYNMNRIRLEWSNMWVILGEHFNQVC
THSNVHVAFFALDALRQLAMRFLEKEELPHFKFQKDFLKPFEYTMINNANPDVRDMVLQCLHQMIQARVHNMRS
GWRTMFGVFSASARVVTERIPTTAFEIVTRLYHEHFPDVVRHGSFADLTVCMGEFCKVSKDDPMVKFWYPILFA
FYDIIMNGEDIEVRRLALDSLFNTLKEHGPTFPVEFWDTICREILFPIFAVLKSKKDFSRFSTEGDMSVWLSTT
MIQALRDLIDLYTFFFDTMERFLDGLLDLLSTENDTLARIGTSCLQQLLEKNAAKLSNDKWERVIKTLIGLFKL
TTPHQLYDEKLRADRKRIFKQIIVKCVLQLLLIETVRDLLQNHDVYRNIPPQLLLRLLSVLEHSYQFARAFNDD
KELRTGLWKESSSAATLVTIYLRMYNDPRPDYMSLRQPVADRLLPLGQGVIQDFNKLKIDSQGKNIAAWSPVVA
ELLRGFNDFDDQTFTRYLPAIYPLATDLMARDKDIRESLKSVFTRVGVAKGIQRQAIILAAQAKFGPEAVAPIL
QAIFPTLSLQVGTSLVTALIQLGPEITSDSQTVRAVLARFGLTAAIPPTDAQVVEIVQTLARRVADTHPLCDVG
ALIRALSSFGVSINWANVVRAFDWPDRGVDTATLKLVIAVLVHSPRAEHPAVAGFWQTWSNPLSQLRLLDALLS
LPSDTFNFVTLPGRRVVKVDDVAGASPTIKALAANVQGHTWNSLDLFETLVRVGVSDSPEVRACVHDMLDKAVK
ISAELVHMGLLQVPPWNELQVEYSTKLLNMFLAGHPNHQLVFMRIWQIEPTYLTTAFRDFYTENPLNITRILDI
LDALLDVRPFIFALDEYLNLDKWLADNITQHGSAFLRAVIDFLDVKTTSEKQARVTENPDRTMALNAQTIAIFL
RVLRNSSSMLAQADIDYCLEIRDACLQVYPRLMDQEPGFSVVSYSQEIESEVDGIYKQMYEENISIEQVIQMLQ
RMKESTTARDHEIFSCMLHFLFDEYKFFQYPARELNMTANLFGSLVQHKLIDYIPLGIAIRYVLDALQCPSDSN
LFSFGVQALSRFEGRLREWQPLCQALLRIPHFAEDRPDLADAARRAELSDKILFIINNLAPSNFDSKLQEMKER
FSTEPNNHALYLRFLDGMEKQPLMKLIVHETIVKSANLLNSEKTMNSPSERTVLKNLASWLNQPIKHKNIAFKE
LLLEGYDQHRLILAVPFVCKILEGSAKSKIFQPPNPWLMAVIALLAEVYHYADIKLNLRFEIEVLWKKLDIDGA
NIEPTSLIRNRPRNMTMHIETILGSLPSVVVINPQLALQATQAFKQAVTMAVDRSVREIILPVVERSVTIAGIS
TREMITKDFATEGDEGRLRTSAHAMARRLAGSLALVTCREPLRSNLTAHIRSFLLEHGFTEQMLISLLVNDNID
IACNAIERAAMDRAAADVDESFVQAFDARRRHREQRPGSNVTSNLPDLLRIKPGGLTNQQLHVYEEFFTHLMAE
VDNVLGQSPQSLTMLPQNHPLKVLGRQIEGLADNEDTLLNFSQRIVHALFKVSTQLGRDFYTAMLERLCRTSEK
VAHEALSWLLYSEDERKFSVPVIATLMRAGLIPLVEHDAHLAKTNNPTIIDFAVGLIRQLTSAESTLAKFHNSI
DWFMKWVQIFQRSASAEKSFVTFIQQLTKEGVLNGDETFAFFRVSAEACIDNYRKQTSTGNLTNIFQPIDALSR
LIALLVKYHGESSQDSFKIKLLSKILTIIVLVLAHAHETQGFQQKPFFRFFSSFLNDLHSMEANLGSTYFQILL
SLANNFQTLQPTYFPGFAFSWITLISHRLFMPKLLLSENREGWACFHTLVICLFKFLANFLRPVQLSDAVRDLY
RGAMRLLVVLLHDFPEFLSEYYFTICDVIPPRCIQLRNVVLSAYPASLVLPDPHLRNIKMGPIPPILSEFTVPL
KIGDLRSFLDQFLLNRASTASLPFLKEFLEKYNLSAINALVMYVGVSSVAQAKARSGSSIFVPSDPGVVLLQYL
VTNLDAEGQYHVLSAAIMHLRYPNAHTHWFSSLLLFLFAEINDEGFREIMTRALLERFIVHRPHPWGAMVTFIE
LLRNPRYEFWTHDFTRVAPEIQLLLDGVGHRKLYLQLCFKNVTDLLAVRRDIMPLALANSAKMNAVDAYAEVVD
PRESIVDIREFDVPYFLRVAIDNEIRVGLWYTVTFSAGKPTISRVQRAEPVVMAYDIETTKAPLKFPDQQIDQV
MMISYMIDGQGFLITNRDIISEDIDDFEYTPKEGYEGPFTVFNEPDEAATIKRFFQHIQTAKPTVMATFNGDFF
DFPFLCARAKVHDIDMLLETGFAKDSEDEFKSNTCVHMDCFRWVKRDSYLPQGSQGLKAVTTAKLGYNPIELDP
ELMTPYALEQPQTLAQYSVSDAVATYYLYMKYVHPFIFSLCTIIPLRPDEVLRKGSGTLCETLLMVEAYRAGVI
FPNKHEDPHGNTFEGHLLASETYVGGHVEALEAGVFRADIPTHFKIVPSACQLIDGLDAALKFCVTNYEDIKSQ
IQSALEEMRDNPMRMDKPRIYHLDVAAMYPNIMLSNRLQPDSVVDEATCAVCDYNRPGKTCDRRMTWAWRGEYY
PAQRDEYNMEMFPPRRPGLPKRRFVELGEAEQAALLHKRLGDYSRKVYKKTKETRVVEREAIICQRENPFYVET
VRTFRDRRYEYKGLHKTWKKNLDSVADIEEAKKMILAHKCILNSFYGYVNRKGARWQSMEMAGITCLTGAHIIQ
MARQLVEQIGRPLELDTDGIWCMLPDVFPEDFTFQVSKGKPLKFSYPCTMLNHLVHDKFTNHQYHDLEKETGEF
KVHSENSIFFELDGPYLAMILPSSKEEDKLLKKRYAVFNYDKSLAELKGFEVKRRGELQLIKIFQSEIFDKFLL
GSTTEECYAAVAEIANQWLDILFSRANSMSDDELFELIAENRSMSRTLAEYAGQKSTSISTAKRLAEFLGEQMV
KDKGLACKFIISAKPIGAPVTERAVPVAIFSADEAVKRTYLRRWLKDNSLSTFDLRSILDWDYYIERLGSVIQK
LITIPAAMQKVSNPVPRIRHPDWLFKRVAALDDKFHQHKVTDFFPGRFALWLSTGSDLVSITLRIPREFYINLK
RAPGYLSEQVARTLPRNRPSGSLFAELTNNTNVDGVYELETLNKARDDGVDLTGLEHKYLFLYHASSGSSSVNV
FALFTPRGARLHVVDPATRRQPVSYHKTESAAQKAVSRDLGLLEKEQYVLVISSAKGMSWHQAAIPKVERFPVV
MMTSSLRSTHSLLNWHVEVVKRMCTRYLAAGPWLHSLVGQATYYALFFADVDFARRLLKEDMILWWSSGSRPDL
GGIEDDVRTTEEVINPQLVVPGCYDNVCLSVQVRNLAINSVLQSSLVNEMEGAGGSTAFDSAQSSTTLGDAAVS
PLTFNVIKSMVRSWLLDKAGPADLVISHFWRWMSSIAAQMHDPGLMRFVHGLMHKTFMQMLAEFKRLGSNVVAA
DFGSILLVTSKPPGTAYAYGTYLKGAVTSHELFKHMYLEIDRYYDYLVYMDNANQAAVVCQNPQEVEPPKSLAV
TMAWNIENFLPPAVQRIFHESVNFFMISMYRIKRERNKEMEAVRSFIATRLTRKMLRSVFPVLPGSHLTLHNPA
LEFVKAVCAVFALAKDFKTEIGILKRNMLDLVGVREFAEEAVFRNPCEPFTLTMVICPCCADMRDMDFCRDVDL

LPWQCAQCGYDFDKQEIDFALVSIIERLEASFTTQDLRCSKCKQVRSDNVSKHCCSGEWRYILSKAEFRRRLRT
AVNVATVHNLPLLAAVDHAKVAHFIGTKVLIANNGIAAVKEIRSIRQWSETFGTERAVEFTVMATPEDLKAVWA
GWGHASENPRLPESLAASKHKIVFIGPPGTAMRSTIVAQSAGVPTMAWSGTGITDTELSPQGWVTVPDKAYKDA
CVTTVEEGLEKADQIGYPVMIKASEGGGGKGIRKVESPDAFKNAFGAVSGEVPGSPIFIMVLADQYGNAISLFG
RDCSVQRRHQKIIPVTIAKPELFEEERAAVRLSKLVGYVSAGTVEYLYSHQEDSFYFLELNNMVVALKELSIRG
DFRTTVEYLINLLQTQAFEENTTAWLDTLISNKLTPERPDWAVICGAVTKAHSEGCWAEYKRILDKGQVPAKDV
LKTVFAVEFIYEGDGKSHSVYWREEVGATRLMIDSKTCLIEQEAYAEIEVMKMYMPFEGLLPPMGMPHVSGNKP
HQHLEYCVDVLNNILDGYDNAAMMGATLKLIEVLHNPELPFYAVGSILSTLSQRMPAKLEDAIRHAMDMAKAKG
AGQEAMVRAQLAPLVEAVERFRGGLKAHETITALLARYQETEHLFGGSIEAVAALVLSHTKAQSKSKLVLALLD
IVKQMEQVLRASVSSSYYGESGIGHRMPSAEVLREVLAALEVYVRRSYRAYTINYRRQGSVSDLSYVINKHIAA
FTNLAALERGFEKVVSALPAFDQPPNVLTIAVQIFDKEDDLVDSVWQEKFIALVNDRTAGVWQEEAALRNIEPA
LAYQLELGRLSNYNLAPCFAFDTRFFIRALVRPGRLRGGMTTAEVGQQYRNSDCNHIYNVTYDEVLQAMAGFIE
RHGKRLWRLHVTGAEIRIILEDNEGNVTPIRAVIENVSGFIVNYHKGTKILKSIGEMPLHLQPVNQPYPTLQPK
RYQAHLIGTTYVYDFPDLFSKALQNAWLEARAARPGLAIPKKVLESRELDEHDRLQEVDNDITYKIGSFGPLED
QFFNLASQYARSYLGVESLRGSGLIATSRAYDDIFTITLQRAVQVEGQPIILTKVLGREVYTSNLQLGGTQIMA
SDLEGATHIVRWLSYVPERRGAPLPVTIPWDREIGYTPPKGAYDPRWFIEGKEEDDKSFQETLGGWAQTVVTGR
ARLGGIPMGVIAVETRTIERVADPANPLSFEQRIMEAGQNSSYKTAQAIFDFREGLPLMIFANWGAWVVLDPSI
NSEQMQMYADVDREKLLGLMDRLDPAYAEFKRASTDKTEATEKLAAREQELQKSLALLYADLHDRGRMEAKGCA
KRSVWKESRRYFYWALRARLARSSALAEIAKQRIQLLDSLLPSVAATMRETAEMLEALDLTRLLTQVRGDQAVV
EGLERLRDKLPDITFDLPPLQGDSIDEHFHNIGRNVAEPYLSMASEFAEQLLCFDVETMPHISPFPIMATARST
THWYSWLSPWLLGRIVVGHNVSYDRARIREEYNLERTATRFIDTMSLHVAVKGISSHQRPAWMKWKKEKRRWEE
LTSANSLADVAMLHCGITVRKAVRDDFLVASREEIAGNLDKYLGYCGTDVKTTYAVFRAVFPKFVAACPSPVSF
AGVMSMGSAFLTVNQEWENYLQRAEAKYRELERAVKVSLLKLAKDAWAQDVWLSQLDWTPKIPAWYRELLPRLE
DELDLTTRSAVAPLLLKLGWRGHPLFRSREHGWMYRVQETAREFKDAHDVEAFFKLPHKDGEEANVGNPLSKTF
MKYAEDGTMTSPSATTRLAIDLNAQSSYWVSARDRVLQQLVVWDKWGMIIPQVITMGTVTRRAIEKTWLTASNA
KSNRIGSELKAMVRAPPGYAIVGADVDSEELWISSVMGDAQFGLHGATAIGWMTLEGTKSAGTDLHSKTASILG
ISRNDAKVFNYSRIYGAGMKHASLLLLQSNPGMSLEKAQELATELYVKTKGRNTHRDIFDRKFWFGGSESYVFN
KLEEIAMSNRPQTPALGCGVTDALAKEYLPQTFGSDYLPSRINWVVQSSGVDYLHLLIVSMEYLLKTYDIKARY
LISVHDELRYLVVEEDKYRCALALQIANLWTRCQFAWRLGMDDLPQGVAFFSAVDVDWLLRKEVDMPCVTPSQL
TPLAPGESLDMRGILEKTHSGSLRRESYTAPNHMSHRATTSHFLKAQATQEFGEVKLMAAALVQTIASTPQDEL
AGLLASYTAWVWPRSDLHGWINVLNNFDGMFEKIITAYAVDKLQTAPFSQPDKHLLLEILRFERMMFENSTNRK
LFSSYDRLNALLASSDLDVVVSVLQLLLRPAQQYSAQPAVAHVLHIASGRLESLSRRWTGLRDHGLEVADLVSK
KSEEVDELPASASDVRFTYYPQISSVATSNKEPMAILRETIEAHHVPEEQFELMCKIRTAWALGKSREQLVIIR
MLAIAVYAHTQSESHAQAALFLYEPDLVSHLAEILQLDRDARKVVADAANSESTISSAFIETVLSFVSYIASHA
AGGNMIIGAGLVPILIQIMDNTLPSRLSVVSKTMSLLDSVLYGFSNAFSILCNAHGVESLVARIEHEVDPRLSV
ARTNVLKHLLRSVHRMMQSPGTTEGLRGLIDSSLPKTIKKIFQNKALFGPVVLPLAINIMSTFVHNEPTSLAIM
QESGLPETFYDTIEGGLEPSIEVLQAIPNAIGALCLNQAGQDQLAARQNVIPMLFATFTSEKHVKVLHDKENAM
VMGSAIDELIRHHPNLKTAVFDAVITTLTKIEELGNAYVDPKGGETPFKLDNIIVTFVDVIGRFLEGLFQHTDG
LERLGRLFTLPSMPADFGGTEFLQVIRTIAEVEPTQTLQKLAQHVKSSLEETRDFWKDMGATANIKFRQLITLN
TRISMLAEVYSAIAHGRSASASLLGDVHRAFIWENILLKSAVNITALKHLAGQIPVTLFLQALIKRRNVDSSHK
KHATQMAKYVADILVKHLYHTVMIGFASVLLTGDLLITIWQAGAQSLIHAFGGLKVTLHLLHQMTSSKALFEST
QAPLLITRLKDTDKDYFEPHDLLVKLRASVLPLVARMWTPTWVTGPPLSITKSIVQSLLEIMRADKEPRSAAER
ALVRTGNNVNAATEYLLSHPELQALRAKLQDGLAAHAFKLVDEHPNIKTFSPSAYDVHEQPLAVRLRLLGVILP
PPPKWVGALLLVAELLLALSEEAPAVTLPLAVGPDYADARTKLFGFALKLLQSAELIRDDMLAVLRILVLLTRD
ATFASEFVRRDGLARLGLESYVAILMRHAVEDKSILRSIITQEIKRFFNQSRLVDISSFMRGGISLVLRDPKAF
IDAAAANCELTGTVAVVHFLTSELMRVIQYACFLMQCLTEMLFSYEPCKTAFLSYSRTKSRPTTLHFLLHEIIS
YGDYHARRRMLLCNWASSVVVALSVDVSKDVSADLVAVRKTVVDAVNKAIKESSTTEPVAARYGRLLAMAELCH
RLLSVKVDVAIHTAKIMLEKGFVATLTNALADVDLNYPHVKTLISAVLRPLEYLTRIAIKMGREETPDLYRNSA
LGMYPDVVTHPLLMDRHDHADLQPQPTSTRWAEEVQISHGKFAPGRMEKLTNHVVLALLPAARERVIVRVNGAD
IDITDTGIDPEFLEALPDDMREEVLNQHFRERAIAQQQIPADSHISAEFLEALPPDIRAELVQQESAEPSDIDP
ASFLASLHPHLRQAVLLEQDGTFLQILPRPAVQRDAIQLLDKNGIATLVRLLFFPQKTLLHKVLVNLCENSKSR
IELLNLLLSILHEGTGDLLAVDKSFSQLSVRPNLVAQRSLEALAYIVSNNELSSLFFLSEQEVAAGLRRSRKGK
GKERQPAVHYPVVALLNLLDRQLVLKTVSIMDSVASLLAIVTKPLTTLKSSLLSRPPVIQAGALRLIVNILTGG
ECSSRTFQHSLTLIQNLACLPESRDTIASELRSRAQDLGSSIYMDLDELVKQLAVKFSPASSDQAKLLRVLKTI

DYMYSVYETFKFSSLWRRLGDVLSIVQEKSDVEHTATVLLPLIESLMVVCKHVGTKESMEELFVGFTDAHRKVL
NLMVRNNPSLMSGSFSLLVHNPRVLDFDNKRNYFNQQLHRRREHYGTLQLNVRRARVFEDSFQYLQRRTGDQIK
YGKLSVRFYDEEGVDAGGVTREWFQILARQMFNPDYCLFQPCAADKLTYQPNRASAVNPEHLSFFKFVGRVIGK
ALYDGRLLDAYFARSLYRQLLGKPVDYRDVEWVDPSYYSSLCWLLENDPAPLDMTFSFGVTKVVPLKENGASIP
VTIENRREFVQLAAEYRLYSSIKDQIESLLSGFYEIIPKDLISIFNEQEVELLISGTPDIDVDEWRAATEYNGY
TASDPVIVWWWRALKSFSRDERAKVLSFATGTSRVPLGGFVELQGVQGVQRFSIHKAYGGTDRLPQAHTCFNQV
DLPQYSSYEMLRTQLLLAINEGGEGFGFAKSSASSATRKKHARKLRRLGKKDAVTKRRALDELAHFPALALHAE
RRVRALTASTHAVLLGPWCMLAHDVDRAIAAAARPAWFVARAAIDPASVHAELNPEEASPDRNARLRVGALGAL
AWGDAQPAVRRASWALVGSLLSVAALRSAWVEADAGVRLAMWEPLLVFLTKYPQAWAYAEFLQFLTLGSPARGY
PTVLVALSTIPPDIFTALWGAVDGRALAFLAALVECALFFASRPKAFLIARLVPPQPAVLDHMRAEAAGEPFPE
GWAVVEPLDTHGKTAYERVLGALLERALARDNLWVLAHLALLATHREAVGVAWLLRRLLSGAEVADAEAWIKAA
RGMERSAPLAAHAILRTVSSTSLEPPLLARYRTELAATLAGVPGLRLLRTLNAAAPDVSGDAALVPQQRAVFLA
KALEKWLASDLDEDVECELAELFVHLAPVLQTVIGKHWEVILDIIENNLENVDLNEDGDLLLLSRTLRLLAAVI
ELAASTKMLRVWASRERAVMGLVLRLSKPRSICKHLLVNLEFIVIESAASTASAKVKTGFTEQIRGLDLIATYF
APNILGMLGVTKPFKLDCWAVDEFYLALYDPLSARLLAAHLYFRALQCVPSLVRAWFVKSSDRQLHNAVSTFTS
SYFSPPLIAHLLAPLRSLIPENKWRAWVLGAQIVGEAGVLGGLLHFRKNVAGHFEGQVECAICYCVIKPCRTCK
NRFHASCLYAPAAGKLDFT AIKTSSPRPF ALEDCPVFYP SLEEFKDPMK YMQVVGPKAR DYGICKIVPP
VGWKMPFVTD TEFRFTTRLQ RLNSIEASSR AKLTFLEQLY RFHSSQGNTN IAVPTVNYRR
LDLWLLRKEV QKLGGYDAVK NKKWGELAQI MGYNAQGVAA QLKASYSKVI LPFENYSDHV
RSALCDGCDA GYHTFCLDPP LSAIPRGQWF CQKCLFGTGD YGFDEGEEHT LQSFMMRDLT
FRRLWFASHP PYSESDVERE FWRLVQTPFE TVEVEYGADV HSTTHGGMPT PETHPRNPYS
RDPWNVNNVP ILPESLLRYI KSDISGMTVP WTYVGMIFST FCWHNEDHYT YSMNYMHWGE
TKTWYGIPGA DALKFEAAIR KEAPDLFDAQ PDLLYQLVTL MNPARLRDAG VRVYACNQRA
GEFVVTFPRA YHAGFNHGLN FNEAVNFALP DWLPFGLECV KRYQEHRKLP VFSHDELLIT
VTQHSHSIKT AVWVLDSLRE MIDRETAQRR VVRPGLQETL EEYDPENQYQ CHVCKAFCYL
AQITCGCVAC LEHAQLLCGC RVLRKRFSDE QLEDIYSKIM ERASIPTDWQ AKLQRTLQDS
ARPNLRVLRA LLAEGERVSF HLPELLALRK CVQRANEWVE VATSFTTRKQ ANKRDRPERG
LKDVYALLEE VELLGFDCPE IETLRRITQS AEEFKKKARL TILETQITIG AGLNMQLEEL
DEMRRIFMRC KLVRDLLDDI RSLLVRAKAC GLTEESKLMK TLLEKQRIGE DWVQKVTALL
NLEQKPLPEI PIDPTVLDRL KAVRAKARDL ERQARIMLAP ELPRPSDALK LVARAEKDFM
IPVIDELKRS AEFANDLEEK CDAVLSKRFR YGTPFGLFRK WVAYGHTHLS FQLHNFEKLD
RQLIAHSQWI ERLPWYCHSD AILRDVRDCT NPDEDHPPSD EFISCICDRQ VRPPPPGEAS
DAVQCDHCFA RFHGACAANG GSCPFCDHHH WNGSIHKERN WHFCFLPTML VTAPDITKFY
STAWKELEYI ISRVDRLCVS IGSFLSFASN QRPEFLPQVR HYMRKLFRIQ FAISPNPDVS
YGLDLAGLHR ILAGKMHKKR RRVKIVFQQD VGPEPADGTQ CLCKGTVCSR WHHETCVFFC
PACSIKRNRA YPYGEVRVRQ APAEQLRRVQ LPPNKNALVL DLIRYSF
>Exidia glandulosa
MRRTGQDQSILFSGDTASGKSENRRLAIKSILELSVSNPGKKGSKLATQLPAAEFVLESFGNARTLFNSNASRF
GKYTELQFTRGRLCGVKTLEYYFERNRAAGAPSGERNFHIFYYLLAGASPEERQHLKLSDKATFRYLGPRDALR
FDQLKGALKNVGMSKRHVAQTCQLVAAILHLGNLEFTIDRHRNEDAAVVRNTDVLEIVADFLGVDPAALEAVLS
YKTKLVKKEVCTVFLDPDGASDNRDDLAKMLYSLLFSWLNESINQRFCRDDFVTFIGIFDLPGTQNLSRSNSLD
QFCVNFANEKLHHWIQRSIFEKHTDEYAKEGISRFVPTVPFFDNSECIRLLSNKPGGLIHIMDDQARRMPKKTD
HTMVEAFGKRWGNHSSFKVGPMDRFPTFTVNHYNGPVTYSSESFLERNLDALNPDFVSLLRGGSINPFVRDLFS
NKAISTVMHPRNEDTVVAAQQPQKPMRAPSTRRKQTPCTSGQFQNAMETLIQTFDDTQPWYVFCINPNDSQLPN
QFEGRSVKAQVRCSGLPEIARRSGIVFEVSMTPAEFCARYAECMTAWGVTGGDHDLVVGQFKVFLSHAAFQKFE
DRLRATDVEEQKRNRMREMEASSQQLPLVAHAAPFSDDDGHSRYTSNRESYAPSRNMFQNFDGEVMEGEVTEEI
HDTSARRRWLAVVWLLTWWLPTPFLTWFGRMKRLDVRQAWREKLAINIIIWFMCGCTIFVIAVLGNLICPREYV
FNADELSSHSNHVYTAIRGEYGGQDISRLFPVQVSALCNGITGSVSPWLTLDTINIAVDAQYHDFRAWSTDYRP
DWYFEQMTVMRYKYRVGFVGYTTQLLRDMAAAGRTVAIYNNISVPAVMFTSTNSTFLTFKPQESLRFTFLTIGS
RGDVQPYIALGKGLVADGHGLEFGYVGGDPAELMRICVDNGTFTVGFLREGVTMFRGWIDDLLNTSWEACQNTD
VLVESPSAMAGIHIAEALRIPYFRAFTMPWSRTRAYPHAFAVPEHKTYVMFDQVFWRGTASQINRWRRRTLGLP
STTLDKIEQHKVPFLYNFSPAVVPQPLDWYEWIRVTGYWFLDDADVSAKKWEAPPDLVEFIDNAHNAGKKVVYP
DPTALTRTVVEAIEKSGVHAILSKGWSDRLEPEVRLPPQIFPIASVPHDWLFAGIPTIIKPFFGDQFFWADRVE
ALGIGSAVRKMTVDSLATALHAATTDEKQIGRAALVGQQIRAENGDKPREIFEPLRLACETRNEKLMIASLDCI

SKLISYSFLVELVDLVTHTITSCYTESTPDAVSLQIVKALLSLVLSPVTLVHHSSLLKAVRTVYNVFLLSQDPV
NQVVAQGGLTQIVNHVFDLFLKDAFLVFRALCKLTMKPLATESERDLKSHAMRSKLLSLHLVQTILSTHMNIFV
SPSSYIYSSSSRESTPFIQATKQYLCLALSRNAVSPVPQVFEISVEIFWRVLSGMRKQLKREIEVLLNEIFIPI
LEMRNATPKQKAVLLTMLSRLCQEPQALVEIYLNYDCDKDAIDNIYERLMNVISKIGTPSLSTTALAGHGKDAV
PNEINLRRLSLECLVFVLRSLVAWGTDDPTRFESAKQLKTTMMEGIRKFNSKPKGIEFFIDVGFIPTNSAQDIA
KFLLETDGLSKAAIGEGDEFNVSVMHAFTDMLDFGGHDFIGALRLFLQSFRLPGEAQKIDRYMLKFAAAFMASQ
DAAYVLAYSTIMLNTDQHNPQVKKRMTPLDFIKNNRGINDLPDEFLNSIFDDIVKNEIRMKDEILSLGRDLQKE
AYVLQSNGMASDQYFSASHFVHVKPMFEVAWMPVLAGISGPLQDTDDLEIVELCLDGFKAFVTTLGKFTFLNNL
GEMKTKNMEAIKTLLDIAVTDGNQLKGSWHEVLTCVSQLERMQLISSGVSRKQLKDELANESRSTHITVAADMV
FSLSHYLSGTAIVDFVQALSDVSWEEIQSSGMSQQPRMFSLQKLVEISYYNMNRIRLEWSNMWVILGEHFNQVC
THSNPHVAFFALDALRQLAMRFLEKEELPHFKFQKDFLKPFEYTMTHNTNPDVRDMVLQCLHQMVQARVHNMRS
GWRTMFGAFSAASKVVTERIPTTAFDIVTRLYKEHFPSIVRHGSFADLTVCISDFCKASKDDQMIKFWFPILFA
FYDIIMNGEDLEVRRLALDSLFSTLKEYGSTFPVEFWDSICQETLFPIFAVLKSRQDLSRFHTQEDMSVWLSTT
MIQALRDLIDLYTFYFDTLERFLDGLLELLGTENDTLARIGTSCLQQLLENNAAKLSVDKWERVIKSLIGLFKL
TTPHQLYDEKLRVERKRIFKQIIVKCVLQLLLIETVRDLLQNQDVYRNIPPQLLLRLLSVLEHSYQFARAFNDD
KELRMGLWKESSSAATLVTVLLRMYNDPRPDYSPLRQPVADRLLPLAQGVIQDFNKLKVDSQGKNITAWSPVVA
ELLRGFNDFDEQTFTRYLPAIYPLATDLMARDKDIREALRAVFTRVGQAKGIQRQAIILAAHAKFGPEAVAPIL
QTIFPSISLQPGTSLVQALIQLGPEITSDTVTVRALLARFGITPPTPPTDAQVVEIVQTLARRVADTHPLCDVG
ALVRALSSYGVPLNWAAVIRAFDWPDRGVDTATLKLVIAILVHSPRAEHPAVAGFWMTWANPLSQLRLLDALLS
LPSDTFNFVTLPGRRVVKVDDVAGASPTIKALAANVQGHTWNSLDLFETMVRLGVHESPEVRACVHDMLDKAVK
ISAELVHMGLLQVPPWNELQVEYATKLLNMFLAGHPNHQLVFMRIWQIDPTYLTTAFRDFYAENPLNITRILDI
LDALLDVRPFIFALDEYLNLDKWLADNIAQHGATFLRAVIDFLDVKTTSEKNARVTESPERTMALNAQTIAIFL
RVLRNSSSMLAQADIDYCLEIRNACLQVYPRLMDQEPGFSVVSYSQEVETEVDSIYKKMYEDDISIDQVIVKLQ
AYKESTNTREHEIFSCMLHFLFDEYKFFQYPHRELGMTAYLFGSLIQHRLIDYIPLGIAIRYVLDALQCPPESN
LFQFGAQALERFVGRLREWQPLCQALLRIPHFVDSRPDLAEAARRAEVSDRILFIINNLAPSNFESKLQEMKER
FSSEPNNHALYLRFLDGLEKQGLMKLVIHETIMKSAMLLNAEKTMSSPSERSVLKNLASWLNQPIKHKNIAFKE
LLLEGYDNGRLILAIPFVCKILEACAKSKVFQPPNPWLMAVIALLSELYHFADIKLNLRFEIEVLWKKLDIDGA
KIEPTLLVRNRPRAMNLHIEELLASLSNTIVINPQLALQATQAFKGAVTLAVDRSVREIILPVVERSVTIAGIS
TRELVTKDFAMEGDEQRMRIAAQVTATKLAGSLALVTCKEPLRSNLTTHIRNYLLEHGFTEQMLISLLVNDNIE
QACKAIEQAAMDRAKADVDDSFMQAFETRRRHREHRPGSTATSNLPDALRIRPGGLSQQQMHVYEEFFTHLMVE
VDAVLNQAPQTFSMLPQNHPLRTYGRQIESLAKSEETLLNFSQRVVHALFKVQTQLGRDFYAAMLERLCRTSEK
VAQEALLWLLYAEDERKFSVPVIATLLRAGLIPALDQDIQLAKSFNPTIIDFTVGLLRQLFPGDASQKQFQYSF
EWFLKWVQIFQRSATTEKSFVTFIQQLQKEGILSGDEQFSFFRVCAEASVESYRKQAMTGNLTNIFQPIDALSR
LIALLIKYHGDTTQDHVKVKYLSKILTIIVLVLAHAHEKQGFQQKPFFRFFSSFLNDLHSMESNLGTAYFQILI
TLSDNFSTLQPTFFPGFAFSWITLISHRLFMPKLLLSENREGWSAFHKLVICLFKFLASFLRPVHLSVAVRDLY
RGAMRLLVVLLHDFPEFLGEYYFTICDVIPPRCIQLRNVVLSAYPATLVLPDPHLRNVKMGPIPPILSDFTSTL
KTGDLRNYLDQFLLNRANSSTLPFVKECLEKYNLSAINALVMYVGVSSVAQAKARSGSSVFVPSDPGVVILQYL
VTNLDAEGQYHVLSAAIMHLRYPNAHTHWYSSLLLFLFAEINDERFREIMTRALLERFIVHRPHPWGAMVTFIE
LLRNPRYDFWNHEFVRVAPEIQMLLEGVGHRRMYLQILFKNVTDLLAVRRDIMPLALANSAKMNAVDAYAEVVD
PRECIIDIREYDVPYYLRVAMDNEIRVGLWYTITFSAGKPTVSRVARAEPVVMAYDIETTKAPLKFPDQEIDQV
MMISYMIDGQGFLITNRDIVSEDIEDFEYTPKEGYEGPFTVFNEPDEEATIKRFFQHIQTAKPTVMATFNGDFF
DFPFLYARSKVHGIDMLLETGFAKDNEDEFKSNTCAHMDCFRWVKRDSYLPQGSQGLKAVTTAKLGYNPLELDP
ELMTPYAMEQPQTLAQYSVSDAVATYYLYMKYVHPFIFSLCNIIPLNPDEVLRKGTGTLCETLLMVEAFRGGVI
MPNRHEDPYGNLFEGHLLASETYVGGHVEALEAGVFRSDIPTHFKIKPEACQLIDDLDRALKFCVTNYDDVKGQ
IQAALEEMRDNPMRMDKPRIYHLDVAAMYPNIMLSNRLQPDSIIDEATCAVCDYNRPGKTCDRRLPWAWRGEFF
PAQRDEYNMEWFPPKRVGLPKRRFIELSESEQSALLHKRLGDYSRKVYKKTKETRIVEREAIVCQRENPFYVDT
VRRFRDRRYEYKGLHKTWKKNMDALADIEEAKKMILAHKCILNSFYGYVMRKGARWHSMEMAGITCLTGAHIIQ
MARALVDQIGRPLELDTDGIWCMLPDVFPEDFTFEVSKGKPLKFSYPCTMLNHLVHDKFTNDQYHDLDKETGEY
AIHSENSIFFELDGPYRAMILPSSKEEDKLLKKRYAVFNFDGSLAELKGFEVKRRGELQLIKIFQSQIFEKFLL
GSTTEECYAAVAEIANQWLDVLFSHADTLNDEELVDLIAENRSMSRTLAEYAGQKSTSISTAKRLAEFLGEQMV
KDKGLACKFIISAKPIGAPVTERAVPVAIFSADEAVKRTYLRRWLKDNSMTTFDLRSILDWSYYIERLGSVIQK
LITIPAAMQKVSNPVPRIRHPDWLHKRVAALDDKFQQHKVTDFFPGRFQLWLSTGSELVSIALRIPREFYLNLK
TAPGYLSEPAVRTLPRNRPCHSLFAQLTNNPAVDGVYELETLNKARAEGVDLSTLEHKYIFLFHASSSSASVNV
FALFMPTGVRLHVVDPATRRQPVSYHKTEGAAQKAVSRDLGLVEKESYILVISSSKSMSWHQSAIPKIERFPVV

MMTSSLRSTHALLNWQVEVVKRMSTRYLAAGPWLHDSVEHATYYALYFADLDFARRLVKEDMVLWWSSGSRPDL
GGIEDDIRTTEELANSQLTYPGCYDNVCLSVQIRNLAINSVLQSSLVNEMEGAGGSTAFDSAQAATSLGDAAVS
PLTFAVVKSMVKSWLLDKAGPADLVVNHFWRWMTSMSSQMFDPGLMHFVHGLMHKTFMQLLAEFKRLGSNVVSA
DFGSILLVTSKPPGTAYAYATYITSAVTSNELFKHIHLETDRFYDYLVFMDNANQAAVVCQNPREVEPPQSLAV
TMAWNIETFLPTALQRHFHDSVNYFMVSMYRIKREKNKEADAVRTFIAQRLTRRMLRAVFPVLPGSHLTMTNPA
LEFVKFVCAIFALAKDFKTEIGILKRNMLDLVGVREFADDAVFRNPCEPFVLSMVICPYCADVRDMDFCRDADL
LPWQCAQCDYDFDRQEIDFALVSIVERLETAFTTQDLRCAKCKQVRSDNVSKHCCSGEWRYSLSKAELRRRLRI
AVNVATVHNLVLLTTVDHSKVSHFIGTKVLIANNGIAAVKEIRSIRQWSETFGTERAVEFTVMATPEDLKAVWA
GWGHASENPRLPESLAASKHKIVFIGPPGTAMRSTIVAQSANVPCMAWSGTGITDTELTPQGWVTVPEEAYKKA
CVTTVDEGLQKAEEIGYPVMIKASEGGGGKGIRKVESPDAFKNAFGAVLGEVPGSPVFIMVLADQYGNAISLFG
RDCSVQRRHQKIIPVTIARPERFEEERAAVRLAKLVGYVSAGTVEYLYSPSDDLFYFLELNNMVVALKELSIRG
DFRTTVEYLINLLQTQAFEENTTAWLDGLITNKLTPERPDQTVICGAVTKAHSDACWAEYKRILDKGQVPAKDV
LKTVFAIDFIYEGDGKSHSVYWREEVGATRLIIDSKTCLIEQEAYAEIEVMKMYMPFEGQLPSMGLPGVAGNKP
HQRLEFYVDVLNNILDGYDNSALMTSTVKLLDVLRNPELPFFTTSAILAALSGRMPAKLEDQVRNALEVAKGKS
GSPEAMVRAQLAPLTEAVERFRGGLKAHETITALLSRYEQTESLFGGSIEAVAALVLSHTKTQSKSKLVLALLD
IVKQMEQVLRASVSSSYYGESGLGHRMPSAEVLREALAALETYVRRSYRAYTVNYRRQGSVSDLSYVISKHIAS
FPNIAALEHGFKKVVATLPPFDQPPNVLTIALRVFDKEDSLEDSIWQEKFASLVNGNVDGTWTEEAAIRNIEPA
LAYQLELSRLSNYKLTPCFVFDTRFFIRALVRPGRLRGGMTMAEVSQQHRNADCNHISNVTYDEVLQAMAGFID
RHGKRLWRLHVTGSEIRIALEDDEGNVTPIRAVIENVSGFIVNYNRGSKILKSIGEMPLHLQPVNQPYATLQPK
RYQAHLIGTTYVYDFPDLFSKALHNVWLEARNARPDLVLPKKVLESRELDEHDCLQEVDNDITYKIGSFGPQED
QFFYIASQYARRFLGVESLRGSGLIATSRAYDDIFTITLQRSVQVEGQPIILTKVLGREVYTSNLQLGGTQIMA
SDLEGATHILRWLSYVPMRRGGPLPITVPWDRDIGYTPPKGPYDPRWFIEGKEEDDKSFQETLSGWAQTVVVGR
ARLGGIPMGVIAVETRTIERVADPANPTSFEQRIMEAGQNSAYKTAQAIFDFREGLPLIIFANWGAWVVLDPSI
NSEQMQMYADVEREKLLGLMDRLDATYAGHKRASLDKTQAAELLAKREQELQKSLALLYADLHDRGRMEAKGCA
QPTVWKESRRHFYWALRARLARSSALAQFAESRVQLLDSLLPSDRTSIRETADMLEALDLSSTLTQVRGDQAVM
EGLERLRDNLPDLAFTLPQLHGASIDEHFHNIGSDVAKPYLDLATQFAEQLLCFDVETLPHISPYAVMAVAVSP
TNWYSWLSPWLLGRIVVGHNVSYDRQRIREEYSLERSASRFLDTMSLHVAIKGISSHQRPAWNKWKKQKRSWEE
LTSANSLLDVAQLHCGITLKKAVRDDFLTATREEILEDVDRYLTYCAGDVHATHSVYRVVFPKFREACPSPVSF
AGVLSMGSACLPVNQEWEKYLERAETKYRELEDGVKYELYKLAKDAWQDDEWLSQLDWTPVVPAWYRELLPVLD
DVLDLTVRTRAAPLLLKLCWRGYPLFHSRAYGWMYRVQEKERKFVVEHDVEKFFKLPHKDGEEANVGNPLSKTF
LKYALDGTLTSPSAVTRKALDMNAQSSYWISARDRVLNQVVVWQKWGLILPQVITMGTVTRRAIEKTWLTASNA
KANRIGSELKAMVRAPPGYAIVGADVDSEELWISSVMGDAQFGLHGATAIGWMTLEGTKAAGTDLHSKTASILG
ISRNDAKVFNYSRIYGAGMKHASLLLLQSNPGMSIEKAQQLALELYARTKGRNTHRDYFGRKFWFGGSESYVFN
KLEEIAMSDRPQTPALGCGVTDALAKKNLPQTFGSDFLPSRINWVVQSSGVDYLHLLIVSMEHLLKAYDIKARY
LISVHDELRYLVVEEDKYRLALALQIANLWTRCQFAYRLGMDDLPLGVAFFSAVDIDWLLRKEVDMPCITPSQP
EPLAPGESLDMEGVLAKTNGGSLWRDGYVPPDCMTHRAQTSHFLKAQATQELGEVKARAAALVANIAATPQDEL
ADLLASFPAWVWPRSDLHAWIGVLNLFDGILEGIISSYAVDKLQVNAFAPPAKNQLQEILRFERMLLENSTNRK
LFSSYDRLNALLASSDLDVVVSVLQLLLRPAQQYSSQPAVTQVLNISSGRLESLSRRWSNLRDYGLDVSDLVSK
KREEVDQLPPVASDLKFTFYRYLPAVATSTQDPMLILAEIIEAHDIPEERFELMCKIRTAWALGKGREKLVVIR
ILAIALYAHTQSESHAQSALFLYEPDLVTHLAELLQLDRDVRTVVADIAKADSTTPSSFIEAILSFVSYIASHA
SGGNMIVGAGLVPLLIQVIDIELPSRLSVVSKTMSLLDSVLYGFTNSFSIFCNARGVETLVTRIEHEVDSGLSV
ARSNVLKHLLRSVHRMMQSPGTTEGLRGLIDSSLPKSLKKIFVHKALFGPVVLPLATNIMSTFVHNEPTSLAIM
QEQGLPEAFYDTIDDGLEASIEVLQAIPNAIGALCLNQAGQDQLAARPTVIPKLFETFTSEKHIKVLHEKENAM
VMGSAVDELIRHHPNLKTTVFASVIATLAKIEELGNAYVEPTGAETPFKLDNIIVSYVDVIGRFLEGLFQHTDG
LERLGRLFTLPAMPFDFGSTEFLQVIRTIAEVTPTQTLQKLTQHVKMSLEETKDIWQSLGAKANVRFRQLVTLN
TRVTMLAEVYSAIAHGRSASTSLLGDLHRSFIWENILLKSALNIAAIKQLAGQIPVTSFLQALIKRRNVDSTHK
KQASQIAALLADVLVKHLYHTVMLGFFSVLLCGELLVTLWRVGAQALVHSFGGLKVALHLLHTMTSSKLLFEST
QTPVLITRVKDTEPNYFEPHDLLIKLRATTLPLIKRLWTDAWVSGPPLSITRSIVQNMLEIMRADREPRSAAER
ALIRMGNNVSAATEYLLSHPELDAARAEIREYLVQHAFKLVDEHPNVKNFSPSAYDVHEQPLAVRLRLLGLVLS
SPPKWLGALLLVAELLLGLADEPSAVTLPLSSGPDYSDARTKLFDFAFSLLQTPELSREDLLASLRLLVLLTRD
SELASEFVKRDGLPRLGLEVYVAILMRHAVEDSKVLRTVMTQEIRRFFGQTRVVDMTTFMRGASHLVLRDPKAF
MDATSAMCVLTGSETVVHFLVSELMKVSRYACFLMQCLTELLFSYEVCKSAFLSYSRSKSKPTVLHFLLHDMIS
YGELTSRRRMLLCNWAASVVVALSVDVSKEISTDLVAVRKNVIDAVNKAIKESSPTETVAARYGRLMAMAELCY
RLLTVKVDVAIHTSKIMLEKGFVATLTNALAEVDLNYPNVKNLISAILRPLEYLTRIAIKMGKEETPDLYRNSA

LGMYADVTTHPLLVDKVEHIELQPQPTSARWAEEIQITHGKSVPERVERLSNHVALRLLPAARQRVVVTINGAE
VDITDTGIDPEFLEALPDDMREEVISQHFRERGMAQQPIPADSQISAEFLEALPPEIRAELLHQESVEPSDIDP
ASFLASLDPHLRQTVLLEQDDVFLQTLPRPAVQRDAIQLLDRSGIATLVRLLYFPQKSLLHKVLVNICENSKSR
TELLNLLLGILHEGTGELLAVDKSFAQLSVRPNLVAQRSLEALSFIVSNNELASLFFLSEQDQPAGLRRSRKGK
GKEKQSTVHYPVVALLGLLDRQLVLKTVTIMDSVASLLAIVTKPLTGLKSALLSRPPTIPHNVLRLIVNILTGG
ECSSRTFQHSLALIQNLACLPDSRDTIASELRSRAQDLGTSIYMDLDELVKQLAVKFSPASSDQAKLLRVLKTI
DYMYSVYETFKFSSLWRRLGDVLAIVQENPDVEHTSTVLLPLIESLMVVCKNVGATESMDELFVSFTDAHRKVL
NLMVRNNPSLMSGSFSLLVHNPRVLDFDNKRNYFNQQLHRRREHHGTLQLNVRRARVFEDSFQYLQRRTGEQIK
YGKLSVRFYDEEGVDAGGVTREWFQILARQMFNPDYCLFQPCAADKLTYQPNRASAVNPEHLSFFKFVGRVIGK
AIYDGRLLDAYFARSLYRQLLAKPVDYRDVEWVDPSYYNSLCWLLENDPAPLDMTFSFGVTKVIPLKENGASIP
VTIENRREFVQLAAEYRLYSSIKDQIESLLAGFYEIIPKDLVSIFNEQELELLISGTPDIDVDEWRAATEYNGY
SASDPVIVWWWRALKSFSRDERAKVLSFATGTSRVPLGGFVDLQGVQGVQRFSIHKAYGGTDRLPQAHTCFNQV
DLPQYSSYEMLRTQVLLAINEGGEGFGFAKSSASSATRKKHARKLRRLGKKDAVTKRRALEELTHFPSLALHPA
RRVRALTASTHAALLGPWCMLAHDMDRAVAMAARPTWFASRAAMDPAALHAELNPEEAATDRAARLRVGALGAL
GWGQDQPGVRRAAWSLVGLLVASAVLRSAWVEPDAVVRAAMWEPLLVFLTKYPTAWAYAEFLQFLALGSPARGF
PTVLVILSTVPPDIFTALWGAVDGRALAFLSAFVECVLFFASRPKAFVLARLVPPPASELERMRVEAARVPYPP
SYAVVEPLDSHGKTAYTRVLEALVERSLARENPWIIAHVALLATPREAVGLGKLLKRLLNGAEVNDAEAWIRAA
RSLERSTPGASHAILRTISSTALEPPLLARYRTELAATLAGVPGLRLLRALNAAAPDASGDAALVPQQRAVFLM
KTIERWIEDAIDEEVESGMTELLVSLAPVLQSVPGKHWETVLDLIENGLESTDMGEEDELLSLARTLRLLASVI
ELAASTKMLRQWAPREKKIMALVLRLSAARAACKELLISLEYLVIESAASTASAKVKAGFVEQLRDLDLVELYF
APNILGLLGVMKSFKLDVWAIEEFFLSLYDPLSPRLLSAHLFFRALESTPTLIRGWFLKSKDRQLHTAVSAYIS
SYFSPPLIATALQPLRALIPENRWRAWTFGLAQNGDGGVLEGLRNFRKNVAGHFDGQVECAICYWCVVGCGPVA
SPRRRTQFFPAPAAKLDFS SIKTESPRPF GLEDCPAFYP SMEEFKDPMK YLQTVAPKAQ EFGICKIVPP
VGWKMPFVTD TEFRFTTRLQ RLNSIEASSR AKLTFLEQLY RFHSSQGNSS IAVPTVNHKR
LDLWLLRKEV QKLGGYEAVK NKKWAELGQL MGYQSPGLAS QLKNSYMKII LPFENYSDHV
RSALCDGCDA GYHIFCLDPP LSAIPRGQWF CSKCLFGTGD FGFDEGEEHT LQSFMTRDRT
FRRHWFQAHP PYSEGDVESE FWRLVETPFE TVEIEYGADV HSTTHGGMPT LETHPRDPYA
RDPWNVNNIP ILQDSLLRYI KSDISGMTVP WTYVGMIFST FCWHNEDHYT YSINYMHWGE
TKTWYGIPGA DALKFEAAIK KEAPDLFEAQ PDLLYQLVTL MNPSRLREEG VRVYACNQRA
GEFVVTFPRA YHAGFNHGFN FNEAVNFALP NWLPFGLECV KRYQEHSKLP VFSHDELLIT
ITQHSHSIKT AVWVLDSLRE MIDRETAQRR AVLPGIQETL EEYDPENQYQ CHVCKAFCYL
AQITCGCVAC LEHAKLLCNC RVLRKRFSDE QLEDVFSKVV ERAAIPSDWQ AKLQRTLQET
SRPNLRVLRA LLAEGERVSF HLPELLALRK CVQRANEWVE VATSFTTRKQ ANKRERPERG
LKDVYALLDE VELLGFDCPE IEQLRRIAQT AEEFRKKARL TILETQITLG QGLNMHLDEL
DEMKRIFMRC RLVRDLLDDV RQLLNRAKHC GLADDSKVVL GLLEKQRAGD EWCKKVTDLL
ALPKKPLVEI PIEPTLLSNL TASRNSARGY ERQAKFMLAP ELPRPKDALD LVHSAEKAFD
IPIIEDLRRS AEFAQDLEEK CEAILLKRFK HGSPFTVFRK WVAYAHAHLS FRLANFEKLD
RQLIAHSQWI ERLPWYCHSD AILRDVRDCT NPEDETPPQD EFISCICERQ VRPPPPGEVS
DAVQCDHCYA RFHGACATNG GSCPFCDHNH WNGSIHKDRN WHFCFLPTML VTAPDITKFY
SVSWKELEYI VARVDRLCVS IGSFLSFASN QRLELIPQVR QYMRKLFRIQ FAVSPNPEVS
YGLDLAGLHR MLASKPPKKK RRVRLVFQPE VAPDPADGTR CVCNGAGCSQ WYHETCVWFC
PICAVKKTRV YPYAEIRVRQ TPSEQLRRVS LAPNKNAIIL ELHRYSV

References

1. Biasini, M. et al. SWISS-MODEL: modelling protein tertiary and quaternary structure using evolutionary information. *Nucleic Acids Res*. **42**, W252-258 (2014).
2. Huang, C. et al. Manganese-enhanced degradation of lignocellulosic waste by Phanerochaete chrysosporium: evidence of enzyme activity and gene transcription. *Appl. Microbiol. Biotechnol*. **101**, 6541-6549 (2017).
3. Floudas, D. et al. The Paleozoic Origin of Enzymatic Lignin Decomposition Reconstructed from 31 Fungal Genomes. *Science*. **336**, 1715-1719 (2012).
4. Ayuso-Fernandez, I., Martinez, A.T. & Ruiz-Duenas, F.J. Experimental recreation of the evolution of lignin-degrading enzymes from the Jurassic to date. *Biotechnol. Biofuels*. **10**, 67 (2017).
5. Ayuso-Fernandez, I., Ruiz-Duenas, F.J. & Martinez, A.T. Evolutionary convergence in lignin-degrading enzymes. *Proc. Natl. Acad. Sci.* USA. **115**, 6428-6433 (2018).
6. Min, B. et al. Genome sequence of a white rot fungus Schizopora paradoxa KUC8140 for wood decay and mycoremediation. *J. Biotechnol*. **211**, 42-43 (2015).
7. Gold, M.H., Youngs, H.L. & Gelpke, M.D.S. Manganese peroxidase. Met. Ions. Biol. Syst. **37**, 559-586 (2000).
8. Fernandez-Fueyo, E. et al. Structural implications of the C-terminal tail in the catalytic and stability properties of manganese peroxidases from ligninolytic fungi. *Acta Crystallogr D Biol Crystallogr*. **70**, 3253-3265 (2014).
9. Fernandez-Fueyo E. et al. Lignin-degrading peroxidases from genome of selective ligninolytic fungus Ceriporiopsis subvermispora. *J. Biol. Chem*. **287**, 16903-16916 (2012).
10. Ruiz-Duenas, F.J. et al. Substrate oxidation sites in versatile peroxidase and other basidiomycete peroxidases. *J. Exp. Bot*. **60**, 441-452 (2009).
11. Stajich, J.E. et al. Insights into evolution of multicellular fungi from the assembled chromosomes of the mushroom Coprinopsis cinerea (Coprinus cinereus). *Proc. Natl. Acad. Sci. U S A*. **107**, 11889-11894 (2010).
12. Nagy, L.G. et al. Comparative Genomics of Early-Diverging Mushroom-Forming Fungi Provides Insights into the Origins of Lignocellulose Decay Capabilities. Mol. Biol. Evol. **33**, 959-970 (2016).
13. Olson, A. et al. Insight into trade-off between wood decay and parasitism from the genome of a fungal forest pathogen. *New Phytol*. **194**, 1001-1013 (2012).
14. Martin, F. et al. The genome of Laccaria bicolor provides insights into mycorrhizal symbiosis. *Nature*. **452**, 88-92 (2008).
15. Ohm, R.A. et al. Genomics of wood-degrading fungi. Fungal. Genet. Biol. **72**, 82-90 (2014).
16. Chung, C. et al. Comparative and population genomic landscape of Phellinus noxius: A hypervariable fungus causing root rot in trees. *Mol. Ecol*. **26**, 6301-6316 (2017).
17. Kohler, A. et al. Convergent losses of decay mechanisms and rapid turnover of symbiosis genes in mycorrhizal mutualists. *Nat. Genet*. **47**, 410-415 (2015).
18. Gaskell, J. et al. Draft genome sequence of a monokaryotic model brown-rot fungus Postia (Rhodonia) placenta SB12. *Genom. Data*. **14**, 21-23 (2017).
19. Ohm, R.A. et al. Genome sequence of the model mushroom Schizophyllum commune. *Nat. Biotechnol*. **28**, 957-963 (2010).

20. Eastwood, D.C. et al. The plant cell wall-decomposing machinery underlies the functional diversity of forest fungi. *Science*. **333**, 762-765 (2011).
